# Global, regional, and national disability-adjusted life years and prevalence of lymphatic filariasis from 1990 to 2021: A trend and health inequality analysis based on the global burden of disease study 2021

**DOI:** 10.1371/journal.pntd.0013017

**Published:** 2025-04-29

**Authors:** Hang Zhao, Tianshi Xu, Hao Shen

**Affiliations:** 1 Suining Municipal Hospital of Traditional Chinese Medicine, Suining, Sichuan, China; 2 The Quzhou Affiliated Hospital of Wenzhou Medical University, Quzhou People’s Hospital, Quzhou, Zhejiang, China; University of Agricultural Sciences and Veterinary Medicine Cluj-Napoca, Life Science Institute, ROMANIA

## Abstract

Lymphatic filariasis (LF) is a neglected tropical disease predominantly affecting marginalized populations in resource-limited settings. It can lead to long-term deformities, disabilities, and reduced economic productivity. This study examines differences in Disability-Adjusted Life Year (DALY) and prevalence of LF across countries and regions and predicts future trends. Trends were analyzed based on demographic factors and epidemiological changes, and cross-national health inequalities in LF were quantified. Using data from the Global Burden of Disease (GBD) 2021 database, this study evaluated trends in age-standardized LF DALY rates and prevalence from 1990 to 2021, employing estimated annual percentage change. The study also assessed the relative contributions of aging, population growth, and epidemiological changes in LF burden trends. To quantify absolute and relative cross-country inequalities, the Slope Index of Inequality (SII) and Concentration Index (CI) were applied. Additionally, Bayesian age-period-cohort models were used to predict LF case numbers and prevalence from 2022 to 2030. The findings indicated that, in 2021, LF burden varied by age, sex, and region, with the highest prevalence among individuals aged 15–49 years, males, and populations in low SDI regions. The global age-standardized prevalence and DALY rates of LF declined between 1990 and 2021, and predictions suggest this downward trend will continue through 2030. Potential changes adjusted by aging and population growth were the primary drivers of reductions in the number of LF cases and DALYs. Over the past few decades, the LF burden has concentrated in underdeveloped and disadvantaged regions. However, cross-national inequalities in LF are narrowing rapidly. These results emphasize the urgent need for sustained health interventions and public health policies to eliminate LF, particularly in low-income, high-risk regions such as Oceania. Targeted efforts are essential to improving the health and well-being of marginalized populations.

## Author summary

Lymphatic filariasis (LF), a neglected tropical disease, is the second leading cause of disability globally after leprosy. It is endemic in resource-poor regions with inadequate sanitation and weak vector control measures, significantly impairing the physical, mental, and social well-being of affected populations while exacerbating poverty. Since the Global Programme for the Elimination of Lymphatic Filariasis (GPELF) was launched in 2000, several Asian countries have successfully eliminated LF as a public health problem. However, a small number of countries continue to face a high risk of infection.

This study analyzed LF prevalence, disease burden, and demographic variations across different countries and regions, considering age, sex, and geographic disparities. The findings suggest that while substantial progress has been made in eliminating LF globally and nationally, the disease burden remains concentrated in underdeveloped regions. Moreover, the study identified priority endemic areas for intervention, providing a valuable foundation for developing effective health policies and intervention strategies.

## Introduction

The term neglected tropical diseases (NTDs) is derived from the NTDs Strategy and Advisory Group [1], referring to a series of diseases or disease groups caused by a wide range of pathogens (encompassing viruses, bacteria, parasites, fungi, and toxins) [2]. NTDs are usually concentrated among the most marginalized groups in resource-poor settings and can cause long-term deformities, disabilities, and reduced economic productivity [3]. Therefore, sustained control and elimination of NTDs is essential to improve the well-being of vulnerable groups.

According to the latest classification of the World Health Organization (WHO) [4,5], most NTDs are parasitic, including LF. It is estimated that about 0.569 billion people worldwide will suffer from LF in 2021 [6]. Human LF transmission exhibits significant heterogeneity at both individual and spatial levels [7,8] and is strongly associated with filarial parasites such as Wuchereria bancrofti, Brugia malayi, and Brugia timori. These parasites are transmitted by mosquito vectors, developing and maturing in lymphatic vessels. These parasites are transmitted by mosquito vectors, developing and maturing in lymphatic vessels, though they may also occasionally inhabit abnormal sites such as subcutaneous tissues [9].

Owing to the dynamic interactions between live parasites and their hosts, LF usually results in long-term and asymptomatic chronic infections, with a small percentage of infected individuals showing obvious signs of disease [10]. For example, the death of adult worms possibly results in inflammation of lymphatic vessels with inflammation of lymph nodes, leading to local swelling, pain, and fever. In severe cases, the disease manifests as chronic lymphedema (which can progress to elephantiasis) and hydrocele [11]. Due to factors such as persistent infection and delayed treatment [12,13], LF infection acquired early in life may continue to progress, causing latent and irreversible damage to the lymphatic system and leading to permanent disability [14].

In 2000, WHO launched the Global Programme to Eliminate Lymphatic Filariasis (GPELF), focusing on halting transmission through the Mass Drug Administration (MDA) and alleviating disease-related suffering via Morbidity Management and Disability Prevention (MMDP). The program has achieved impressive success in preventing and controlling LF, with a 74% reduction in the number of people living with LF globally in 2018, to approximately 51 million people [15]. To date, countries such as China, South Korea, Brazil, and Timor-Leste have successfully eliminated LF as a public health concern [16–18], while others have made slower progress in disease elimination [12,19].

The Global Burden of Disease Study (GBD) 2021 is a comprehensive epidemiological study that provides robust estimates of disease burden using secondary data sources, including censuses, civil registration, and disease registries, applying different modeling strategies based on causation type [20]. For LF, GBD 2021 provided LF disease and disability-adjusted life years (DALYs) metrics for 72 countries and territories from 1990 to 2021. While previous studies have explored the prevalence of NTDs at the global level or partial subnational level in 2019 and before [15,21,22], this study extends the analysis using the latest GBD 2021 data. Specifically, it aims to: (i) provide trend analyses of LF prevalence and DALYs from 1990 to 2021 for countries where LF remains a public health challenge, examine gender and age-group differences, conduct equity and predictive analyses, and provide policy insights for resource allocation, progress assessment, and targeted interventions; and (ii) investigate geographic variations in LF prevalence and DALYs among countries that have successfully eliminated LF as a public health problem, informing the development of post-validation surveillance programs in the context of transnational population mobility (e.g., migration and emigration) [23].

## Methods

### GBD overview

The data used in this study were derived from the GBD 2021, which is the most extensive collaborative global epidemiological survey to date, led by the Institute for Health Metrics and Evaluation (IHME) [24]. GBD 2021 is based on 328,938 epidemiological data sources, with state-of-the-art modeling estimation of 371 diseases and 88 risk factors in 204 countries and territories around the world.

### Data sources

GBD 2021 integrates a diverse range of epidemiological data sources, including censuses, population registers, civil registries, hospital data, claims data, surveys, disease registries, morbidity notification data, police records, and literature. In regions lacking national vital statistics registries, GBD supplements data with sample registries, demographic surveillance systems, and verbal autopsies. The 565 data sources used for lymphatic filariasis (LF) estimates primarily consist of systematic literature reviews and data from the Global Programme to Eliminate Lymphatic Filariasis.

There were significant differences in the modeling strategies of GBD 2021 for the different causes. After data entry, GBD 2021 carried out LF prevalence estimation by including covariates and age-adjusted geospatial modeling, and the prevalence of activities of daily living (ADLs) based on the estimated prevalence of lymphoma and hydrocele. Further calculations were performed using the Bayesian regression tool DisMod-MR 2.1. For additional details on LF data entry and model estimation in GBD 2021, please refer to pages 697–701 of Appendix 1 of the relevant literature [20].

### Study area and time period

This study included 67 countries and territories (including non-sovereign territories, e.g., U.S. Soma) with LF prevalence from 1990 to 2021 in GBD 2021, which divides the 204 countries and territories into 21 regions based on geographic and epidemiological convergence [25]. The 67 countries and territories, along with their corresponding GBD regions, are listed in S1 Table. Furthermore, the study referenced the prevalence and DALYs of LF since 1990, with a focus on the prevalence and DALYs of LF in the 67 countries and territories in 2021.

## Prevalence definition and metric

All results in GBD 2021 refer to point prevalence. LF prevalence can be defined as the cases confirmed through antigenemia or microfilaremia diagnostic testing in a specified population at a designated time.

GBD 2021 provided estimates of mortality, prevalence, DALYs, and other indicators based on per 100,000 population and their 95 percent uncertainty intervals (UI) [26]. This study extracted age-standardized or crude prevalence and DALY rates stratified by sex, age, Socio-demographic Index (SDI), region, country, and the number of cases and DALYs in 1990 and 2021 as study indicators. Age-standardized rate (ASR) was calculated from the population structure of different age groups. Although ASR is unable to measure the actual prevalence of disease in the presence of significantly different population structures [27], it is more robust to comparisons of population health across time and geographic regions compared to crude rates [28]. The SDI, a composite measure of regional development, serves as a proxy for socioeconomic status. The classification of SDI levels for the 67 countries and territories included in this study is presented in S2 Table.

The data used in this study were obtained from the Global Health Data Exchange (GHDx) platform (https://vizhub.healthdata.org/gbd-results/). The search criteria specified ‘Cause’ set to ‘Lymphatic filariasis,’ ‘Measure’ set to ‘Prevalence and DALY,’ ‘Indicator’ set to ‘Number and rate,’ and ‘Sex’ specified as ‘Male, female, and all,’ providing epidemiological data on LF from 1990 to 2021 for 204 countries and territories worldwide. As this study is a secondary analysis of publicly available data and complies with the Institute for Health Metrics and Evaluation (IHME) data use protocol, no additional ethical review was required.

## Data analysis

### Descriptive analysis

This study described the prevalence of LF by age-standardized LF prevalence and DALY rates, stratified by sex, age, SDI, region, and country, as well as the number of cases and DALYs, in 1990 and 2021, to create spatial visualization maps. Age groups were designed to range from ‘< 5 years’ to ‘95+ years’ in 5-year intervals. Geospatial visualization and subsequent analyses were performed in RStudio 4.2.3 statistical software.

### Trend analysis

Additionally, this study further used estimated annual percentage change (EAPC) to measure the trend in LF age-standardized rate (ASR) stratified by gender, SDI, region, and country from 1990 to 2021. The ASR (per 100,000) can be calculated as the sum of the multiplication of age-specific rates ($a_{i}$) and the number of persons (or weight) ($w_{i}$) in the same age subgroup i of the chosen reference standard population, then divided by the sum of standard population weights:


$$ASR=\frac{\sum_{i=1}^{A}a_{i}w_{i}}{\sum_{i=1}^{A}w_{i}}\times 100,000$$

Assuming that the natural logarithm of ASR is linear with respect to time, it can be expressed by the formula: y=α+βx+εwhere $y=\text{ln}\left(ASR\right)$the age is the logarithmic transformation of the age-standardized prevalence and DALY rates, x=calendar yearand *β* determines the upward or downward trend of the ASR [29]. The EAPC was calculated as $100*\left(e^{\beta }-1\right)$and its 95% confidence intervals were calculated from the linear model to reflect the trend of the ASR. For the interpretation of the EAPC value, a review of the relevant literature suggests that if the lower limit of both the EAPC value and the 95% Confidence Interval (CI) is greater than 0, the response is an upward trend in the relevant indicator; if the upper limit of both the EAPC value and the 95% CI is less than 0, a downward trend is reflected [30,31].

### Decomposition analysis

This study stratified and decomposed trends in LF prevalence and DALY rates by gender, SDI, and region from 1990 to 2021. The Das Gupta decomposition method, which assesses the relative importance of factors by decomposing the difference between two rates as a function of two or three factors [32], has been widely used in the field of communicable and non-communicable diseases [33,34]. The Das Gupta decomposition method starts with the assumption that the rate r is jointly determined by k multiplicative factors $x_{1}$ … $x_{k}$, which can be expressed by the formula:


$$r\left(x_{1}\ldots x_{k}\right)=\;\overset{k}{\underset{i=1}{\sum }}x_{i}$$

In this study, *r* is the prevalence and DALY rates, and $x_{1}$ to $x_{3}$ are aging, population growth, and epidemiological changes (potential changes adjusted by aging and population growth), respectively. For k≥3the Das Gupta method considers decomposing the contribution value of each factor one by one according to all orders and then calculating the average value of the contribution value of each factor under different decomposition orders and using this average value as the contribution value of each factor to the overall change, which can further eliminate the bias caused by decomposition order. The specific methodological content has been described in the related literature [35].

### Cross-country inequalities analysis

The Slope Index of Inequality (SII) and the Concentration Index (CI) were used to assess cross-country inequality in lymphatic filariasis (LF) prevalence and disability-adjusted life years (DALY) rates from 1990 to 2021, with the Socio-Demographic Index (SDI) serving as a proxy for socioeconomic development [36]. The SII is a complex, weighted measure of absolute inequality, specifically: the absolute value of the SII explains the absolute difference in prevalence and DALY rates between the most disadvantaged and the most advantaged. If the value of SII is greater than 0, it indicates that the indicator of interest is concentrated in the advantaged group; if the value is less than 0, it indicates a concentration in the disadvantaged group [37].

The Concentration Index (CI) is a relative inequality measure that responds to the extent to which inequality is concentrated in disadvantaged or advantaged groups, specifically: the value of CI ranges between -1 and 1, with the larger the absolute value, the greater the degree of concentration of inequality; a CI greater than 0 indicates that inequality is concentrated in the advantaged group; if the value is less than 0, it indicates a concentration in the disadvantaged group [38]. In this study, the most disadvantaged group in SII and CI refers to the population in countries with relatively the lowest SDI, while the most advantaged group refers to the population in countries with relatively the highest SDI. The methodological details of SII and CI have been extensively documented in the relevant literature [39,40].

### Predictive analysis

A Bayesian age-period-cohort (BAPC) model was used to predict the age-standardized case numbers and prevalence of LF at the global and national levels from 2022 to 2030, and 95% credible intervals (CrIs) for the posterior distributions were calculated. The BAPC model in this study uses an efficient Integrated Nested Laplace Approximation (INLA), which can effectively overcome the problems related to mixing and convergence and improve the prediction accuracy [38], and has been used for cystic echinococcosis, food-borne trematodes, and other NTD diseases that have been used for predictive analyses [41–43].

The BAPC and INLA packages of RStudio 4.2.3 statistical software were used to construct the BAPC model. Age, period, and cohort effects were modeled using the recommended two-stage random effects (the second-order random walk, RW2) model [44]. Additional random effects (independent and identically distributed, iid) were used to adjust for overdispersion, which may improve short-term prediction. Default parameters were used for the rest of the model parameters. For the implementation of the BAPC model using the INLA method, please refer to the related literature [45].

## Results

### The global burden and temporal trends of LF

In 2021, approximately 56.90 million (95% UI: 48.67 to 67.91 million) of the global population had LF, resulting in a burden of 1.31 million (95% UI: 0.769 to 2.22 million) DALYs. The global age-standardized prevalence and DALY rates for LF are, respectively, 705.97 (95% UI: 603.68, 841.83) per 100,000 population and 16.50 (95% UI: 9.65, 27.96) per 100,000 population. 705.97 (95% UI: 603.68, 841.83) per 100,000 population and 16.50 (95% UI: 9.65, 27.96) per 100,000 population, respectively, as shown in Tables 1 and S5 .The global age-standardized prevalence and DALY rates for LF showed a significant downward trend between 1990 and 2021, with EAPC values of −6.10 (95% CI: −5.49 to −6.70) and -5.53 (95% CI: −4.98 to −6.08), respectively, as shown in Table 1.

**Table 1:** The age-standardized prevalence and DALY rates (per 100,000) of lymphatic filariasis, and estimated annual percentage change in the age-standardized rates, from 1990 to 2021.

Location	Age-standardized prevalence (95% UI)		EAPC (95% CI)	Age-standardized DALY rates (95% UI)		EAPC (95% CI)
	1990	2021	1990-2021	1990	2021	1990-2021
Global	4054.97 (3493.54, 4829.06)	705.97 (603.68, 841.83)	−6.10 −6.70 to −5.49)	79.10 (53.45, 110.49)	16.50 (9.65, 27.96)	−5.53 (−6.08 to −4.98)
**Sex**						
Male	4594.94 (3996.99, 5361.49)	775.00 (647.20, 954.34)	−6.17 (−6.77 to −5.56)	135.27 (90.00, 190.71)	24.63 (12.99, 45.10)	−5.89 (−6.50 to −5.28)
Female	3532.47 (2973.50, 4306.80)	637.13 (552.60, 760.39)	−6.03 (−6.62 to −5.43)	23.16 (15.62, 33.47)	8.32 (5.76, 11.81)	−3.88 (−4.34 to −3.42)
**SDI region**						
Low SDI	14389.38 (12098.02, 17028.63)	1669.80 (1370.97, 2093.63)	−7.96 (−8.97 to −6.94)	279.13 (189.86, 389.94)	37.40 (22.23, 62.02)	−7.52 (−8.48 to −6.54)
Low middle SDI	9744.74 (8575.49, 11330.64)	1370.16 (1150.05, 1664.52)	−6.41 (−7.03 to −5.79)	186.21 (127.24, 261.55)	30.02 (17.67, 50.32)	−5.96 (−6.54 to −5.37)
Middle SDI	3346.18 (2336.31, 5033.76)	579.98 (461.00, 826.49)	−6.04 (−6.44 to −5.64)	67.29 (45.44, 94.84)	13.78 (7.93, 23.76)	−5.51 (−5.93 to −5.08)
High middle SDI	256.94 (121.61, 572.20)	75.31 (37.79, 177.57)	−4.84 (−5.56 to −4.12)	5.31 (3.43, 7.83)	2.16 (1.23, 3.83)	−3.67 (−4.28 to −3.07)
High SDI	0.00 (0.00, 0.00)	0.00 (0.00, 0.00)	–	0.00 (0.00, 0.00)	0.00 (0.00, 0.00)	–
**GBD region**						
Caribbean	6085.03 (2216.30, 13581.98)	851.78 (465.21, 1642.64)	−9.35 (−10.76 to −7.91)	108.22 (73.69, 150.95)	21.17 (13.28, 34.85)	−8.01 (−9.28 to −6.72)
Central Sub-Saharan Africa	14837.02 (8930.28, 23403.91)	1989.86 (1060.32, 3782.61)	−7.94 (−9.09 to −6.78)	286.26 (192.38, 395.92)	39.11 (22.19, 67.62)	−7.98 (−9.18 to −6.77)
Eastern Sub-Saharan Africa	13761.16 (9874.49, 18730.79)	702.62 (469.98, 1088.79)	−11.11 (−12.77 to −9.42)	263.05 (178.89, 366.93)	24.59 (14.43, 43.70)	−9.34 (−10.72 to −7.93)
High-income Asia Pacific	2.33 (0.39, 17.04)	2.77 (0.70, 14.62)	−1.19 (−2.38 to 0.02)	0.06 (0.03, 0.10)	0.09 (0.05, 0.17)	1.21 (0.87 to 1.56)
North Africa and Middle East	853.79 (233.66, 2755.12)	170.36 (48.55, 537.52)	−8.45 (−9.62 to −7.28)	15.62 (9.00, 25.64)	3.20 (1.85, 5.79)	−9.02 (−10.33 to −7.70)
Oceania	33028.44 (17334.72, 52695.86)	7646.47 (2970.42, 18140.42)	−5.54 (−5.96 to −5.12)	607.52 (419.56, 865.23)	163.51 (107.49, 237.26)	−4.73 (−5.06 to −4.39)
South Asia	11084.20 (10335.96, 11905.18)	1884.30 (1646.08, 2191.41)	−5.55 (−6.05 to −5.04)	215.49 (147.28, 302.40)	39.63 (23.14, 66.41)	−5.23 (−5.73 to −4.74)
Southeast Asia	11094.41 (5972.50, 18901.61)	1089.06 (539.69, 2167.24)	−8.77 (−9.34 to −8.20)	225.37 (150.85, 315.75)	27.78 (16.00, 48.62)	−8.05 (−8.65 to −7.45)
Southern Sub-Saharan Africa	300.48 (77.98, 1111.19)	150.93 (52.40, 490.52)	−2.47 (−3.31 to −1.63)	6.40 (3.66, 11.21)	5.05 (2.94, 8.88)	−0.92 (−1.19 to −0.66)
Tropical Latin America	185.25 (132.72, 350.34)	7.94 (6.76, 13.55)	−10.73 (−11.69 to −9.77)	4.59 (3.01, 6.86)	0.68 (0.47, 0.98)	−6.87 (−7.34 to −6.40)
Western Sub-Saharan Africa	17366.89 (11288.48, 25504.07)	1729.07 (1105.91, 2662.41)	−8.08 (−9.14 to −7.00)	322.73 (219.08, 446.79)	41.08 (25.09, 66.57)	−7.40 (−8.33 to −6.45)

Abbreviations: DALYs, Disability adjusted life years; SDI, socio-demographic index; EAPC, estimated annual percentage change; UI, uncertainty interval; CI, conﬁdence interval.

### Regional burden and temporal trends of LF

In 2021, the number of cases and DALYs were predominantly concentrated in South Asia, Southeast Asia, and Western Sub-Saharan Africa, according to Global Burden of Disease (GBD) region classifications. The estimated number of LF cases in these regions was 34.67 million (95% UI: 30.25–40.39 million) in South Asia, 7.88 million (95% UI: 3.89–15.73 million) in Southeast Asia, and 6.93 million (95% UI: 3.89–15.73 million) in Western Sub-Saharan Africa, collectively accounting for 86.95% of the global LF cases. The DALYs were 0.73 million (95% UI: 0.43 to 1.2 million), 0.20 million (95% UI: 0.11 to 0.35 million), and 0.17 million (95% UI: 0.11 to 0.29 million), respectively, as shown in S5 Table.

The highest age-standardized prevalence and DALY rates for LF were found in Oceania, South Asia, Central Sub-Saharan Africa, and South Asia, with age-standardized prevalence rates of 7646.47 (95% UI: 2970.42 to 18140.42), 1989.86 (95% UI: 1060.32 to 3782.61), and 1884.30 (95% UI: 1646.08 to 2191.41) cases of LF per 100,000 population, respectively. The age-standardized DALY rates were 163.51 (95% UI: 107.49 to 237.26) per 100,000 population, 39.11 (95% UI: 22.19 to 67.62) per 100,000 population, and 39.63 (95% UI: 23.14 to 66.41) per 100,000 population, respectively. Eastern Sub-Saharan Africa had the highest decline in age-standardized prevalence and DALY rates of LF from 1990 to 2021, with EAPC values of −11.11 (95% CI: −9.42 to −12.77) and −9.34 (95% CI: −7.93 to −10.72), respectively, as shown in Table 1.

In 2021, based on the SDI quintile, low and medium SDI regions have the highest number of cases and DALYs, 25.51 million (95% UI: 21.40 to 30.99 million) and 0.57 million (95% UI: 0.33 to 0.96 million), respectively. The low SDI region exhibited the highest age-standardized prevalence and DALY rates, at 1,669.80 (95% UI: 1,370.97–2,093.63) per 100,000 population and 37.40 (95% UI: 22.23–62.02) per 100,000 population, respectively. LF prevalence data was unavailable for the high SDI region, which was not among the 67 countries of interest in this study. The prevalence of LF was unavailable in the High SDI region and did not include the 67 countries of interest in the study. Between 1990 and 2021, the low SDI region experienced the fastest decline in the EAPC of age-standardized LF prevalence and DALY rates, with EAPC values of −7.96 (95% CI: −8.97 to −6.94) and −7.52 (95% CI: −8.48 to −6.54) per 100,000 population, respectively, as shown in Table 1.

In 2021, at the national level, the prevalence of LF and DALY was concentrated in India, Indonesia, and Nigeria. The case numbers were 33.38 million (95% UI: 29.00 to 38.82 million), 4.26 million (95% UI: 1.75 to 11.00 million), and 2.85 million (95% UI: 1.23 to 5.65 million), respectively. The three countries accounted for about 71.24% of the global LF cases.

**Fig 1:**
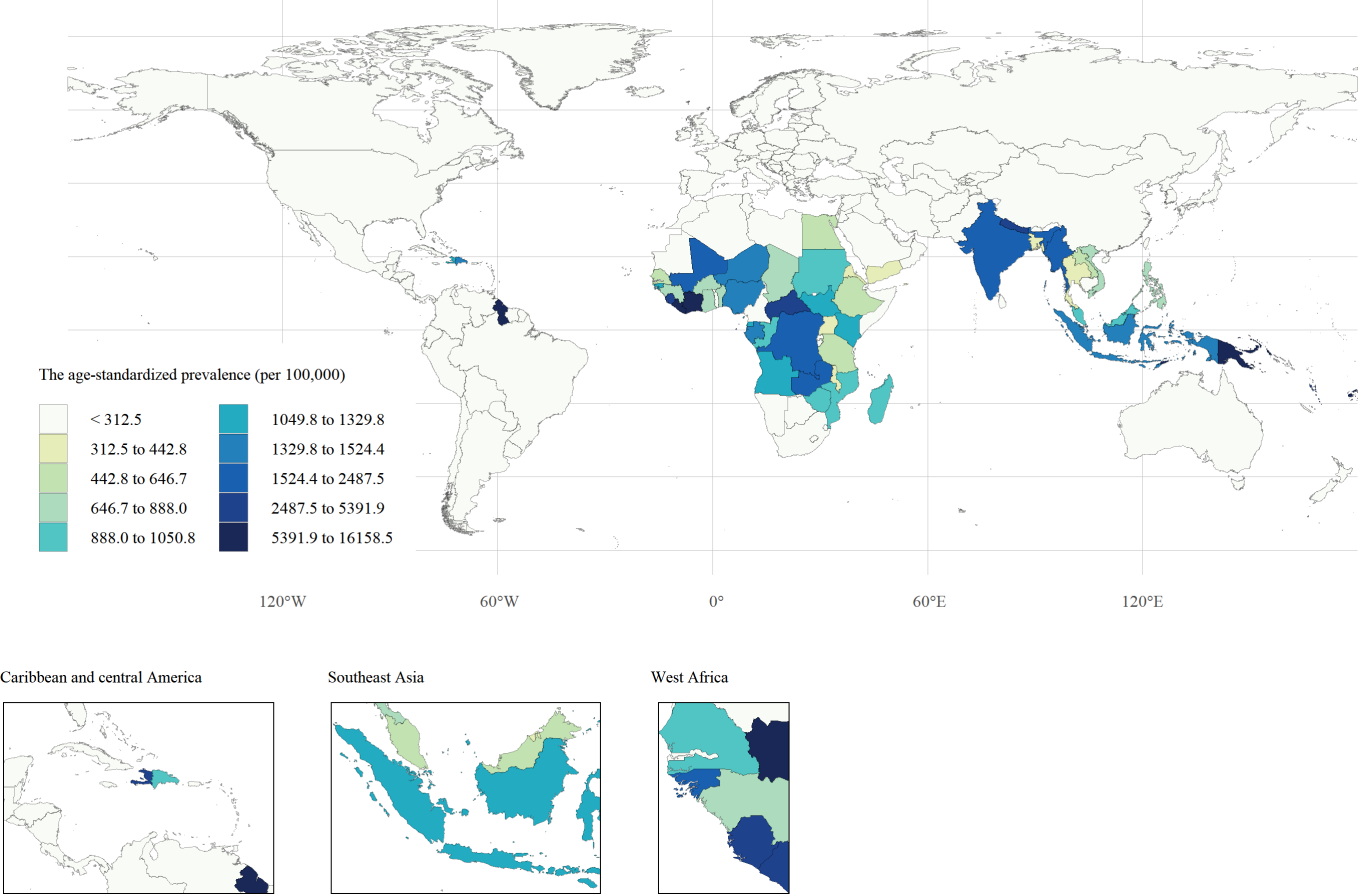
The age-standardized prevalence (per 100,000) of lymphatic filariasis, among 67 countries and territories in 2021. The shapefiles used for spatial visualization are sourced from Natural Earth. The terms of free use for these shapefiles can be found at: https://www.naturalearthdata.com/about/terms-of-use/, the shapefiles available at: https://www.naturalearthdata.com/downloads/10m-cultural-vectors/10m-admin-0-countries/.

The Republic of Guyana had the highest LF age-standardized prevalence and DALY rates of 16158.49 (95% UI: 5921.82, 33023.58) per 100,000 population and 322.77 (95% UI: 219.64, 443.01) per 100,000 population, respectively. The next was the Republic of Liberia, with age-standardized prevalence and DALY rates of 11813.12 (95% UI: 9265.72, 15003.63) per 100,000 population and 259.41 (95% UI: 176.03, 361.54) per 100,000 population, respectively. The age-standardized prevalence rate and DALY rate, along with their age-standardized EAPC, for 67 countries and territories in 1990 and 2021 are shown in Figs 1 and S1-S3 and S3 Table.

### Age- and sex-specific patterns of LF

**Fig 2:**
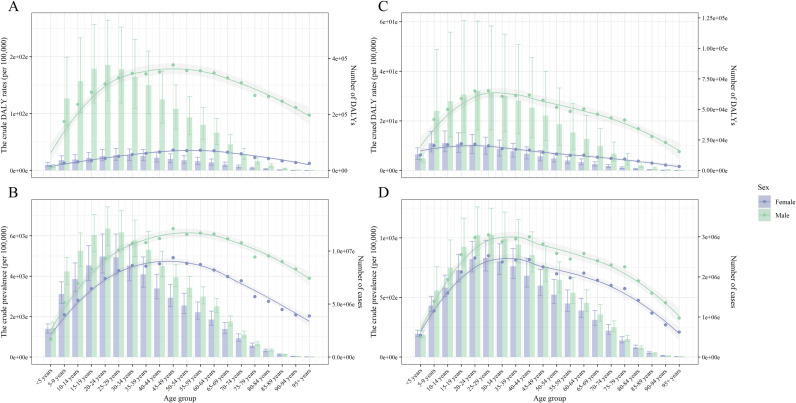
The crude prevalence and DALY rates (per 100,000), number of cases and DALYs of lymphatic filariasis, by age group, sex, at the global level, in 1990 (A, B) and 2021 (C, D). The bar graphs represent the number and the error bars indicate the 95% uncertainty intervals; The smooth line fitted using the loess function represents the trend of age-standardized rates and error envelope indicate the 95% confidence intervals. Abbreviations: DALYs, Disability adjusted life years.

There was significant age and sex heterogeneity in the prevalence and disability-adjusted life years (DALYs) of lymphatic filariasis (LF). In 2021, the highest prevalence and DALY rates were concentrated in males aged 15–44 years, following an overall increasing and then decreasing trend. Among males aged 25–29 years, the crude prevalence was 1,024.11 (95% UI: 856.72–1,248.09) per 100,000 population, while the DALY rate was 32.09 (95% UI: 16.67–59.42) per 100,000 population. Among females, crude prevalence was highest in the 25–29-year-old age group, whereas crude DALY rates peaked in the 15–19-year-old group. Within the same age group, males exhibited higher prevalence and DALY rates than females in those aged >5 years. From 1990 to 2021, the age-standardized prevalence declined at a slower rate in females (EAPC: −6.03, 95% CI: −6.62 to −5.43) than in males (EAPC: −6.17, 95% CI: −6.77 to −5.56). A similar pattern was observed for DALY rates, which declined less in females (EAPC: −3.88, 95% CI: −4.34 to −3.42) than in males (EAPC: −5.89, 95% CI: −6.50 to −5.28), as shown in Fig 2 and Tables 1, S6 and S7.

There was significant age and sex heterogeneity in the prevalence and disability-adjusted life years (DALYs) of lymphatic filariasis (LF). In 2021, the highest prevalence and DALY rates were concentrated in males aged 15–44 years, following an overall increasing and then decreasing trend. Among females, the highest prevalence was observed in the 25–29-year-old group (2.47 million, 95% UI: 2.15–2.91 million), while the highest DALYs occurred in the 10–14-year-old group (33,446.91, 95% UI: 22,932.04–48,347.64). Overall, the burden of LF remained higher in males than females across all age groups over 5 years. However, the gender disparity in disease burden decreased over time. Notably, the age group with the highest prevalence in females shifted from 20–24 years in 1990–25–29 years in 2021, while the age group with the highest DALYs shifted from 20–24 years in 1990–10–14 years in 2021. The GBD estimates by sex, age, SDI level, region, and country, as well as crude prevalence and DALY rates, number of cases, and DALYs, are detailed in Fig 2 and S6–S9 Tables.

### Decomposition analysis

**Fig 3:**
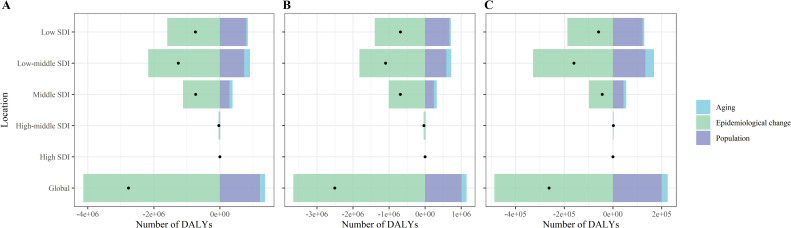
Changes in DALYs number of lymphatic filariasis according to population-level determinants including aging, population growth and epidemiological change, by sex, SDI quintiles, from 1990 to 2021 at the global level and. (A) Both sex (B) Male (C) Female. Abbreviations: SDI, socio-demographic index; DALYs, disability adjusted life years.

**Fig 4:**
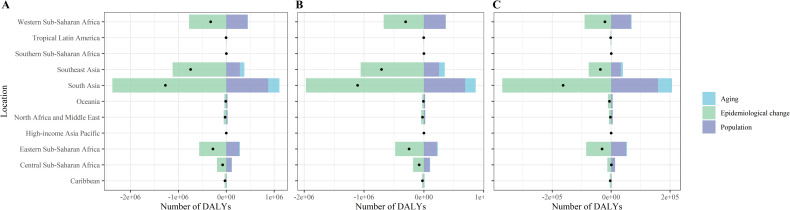
Changes in DALYs number of lymphatic filariasis according to population-level determinants including aging, population growth and epidemiological change, by sex, GBD regions, from 1990 to 2021 at the global level and. (A) Both sex (B) Male (C) Female. Abbreviations: SDI, socio-demographic index; DALYs, disability adjusted life years.

From 1990 to 2021, the global population growth and aging contributed 43.83% and 5.63%, respectively, to increase in the number of lymphatic filariasis (LF) DALYs, and 39.64% and 5.72% to the increase in LF case numbers. Epidemiological changes, after adjusting for population growth and aging, indicated a downward trend in LF prevalence and DALY rates, serving as the primary driver of the global decline in LF burden. By SDI region, population growth was the largest upward contributor to the increase in LF prevalence and DALY rates in low SDI regions, 107.37% and 95.64%, respectively. Population aging made a smaller upward contribution to both the number of LF cases and DALYs in low SDI (6.71% and 5.65%), low-middle SDI (14.10% and 13.46%), and middle SDI (12.96% and 12.93%) regions, and virtually disappeared in middle and high SDI (−1.96% and −0.33%) countries. Furthermore, the differences in the contribution of population size and age structure in different sex groups are shown in Figs 3, 4, S1, and S2, and S10 and S11 Tables.

### Cross-country inequality analysis

**Fig 5:**
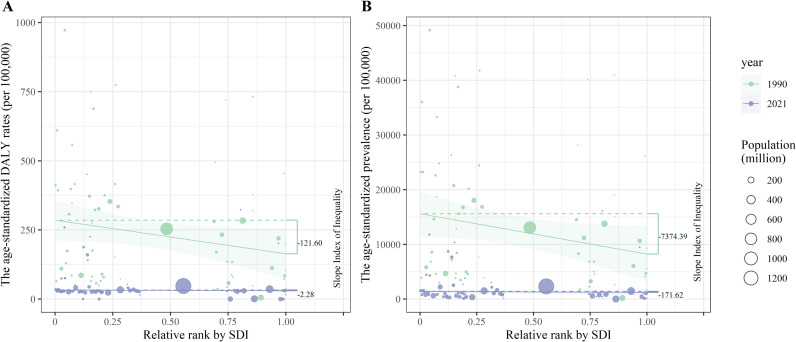
Health inequality regression curves for the age-standardized (A) DALY rates and (B) prevalence of lymphatic filariasis, at the global level, in 1990 and 2021. The smooth line fitted using the generalized linear model represents the trend of crude rates ranked by SDI and error envelope indicate the 95% confidence intervals. Abbreviations: DALYs, Disability adjusted life years. Abbreviations: SDI, socio-demographic index; DALYs, disability adjusted life years.

**Fig 6:**
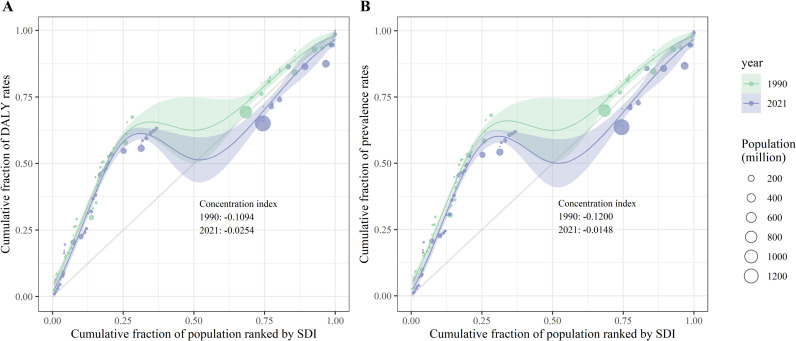
Concentration curves curves for the age-standardized (A) DALY rates and (B) prevalence of lymphatic filariasis, at the global level, in 1990 and 2021. The smooth line fitted using the loess funtion represents the concentration curve and error envelope indicate the 95% confidence intervals. Abbreviations: SDI, socio-demographic index; DALYs, disability adjusted life years.

Using SDI as a proxy for regional socioeconomic status, this study identified significant absolute and relative cross-country inequalities in the age-standardized prevalence and DALY rates of LF. Countries with lower SDI values exhibited higher age-standardized prevalence and DALY rates, with a disproportionate concentration in disadvantaged regions. In 1990, the SII for age-standardized prevalence and DALY rates was −7,374.39 and −121.60, respectively, indicating that countries with the lowest SDI had rates higher than those with the highest SDI by 7,374.39 and 121.60 per 100,000 population. By 2021, this gap had narrowed to 171.62 and 2.28 per 100,000 population, respectively. The concentration indices (CIs) for age-standardized prevalence and DALY rates also declined between 1990 and 2021, reflecting a reduction in inequality over time, as shown in Figs 5 and 6.

### Predicted trends

The present study further predicted the age-standardized prevalence and case numbers of LF at the global and national levels, stratified by sex, and constructed 95% CrIs for the posterior distributions. In 2030, the predicted global number of LF cases was 39.18 million (95% CrI: 0.00 to 174.94 million), and the predicted age-standardized prevalence was 454.54 (95% CrI: 0.00 to 2029.66). The global age-standardized case of LF was higher in men than in women, at 21.43 million (95% CrI: 0.00 to 48.29 million) and 496.01 (95% CrI: 0.00 to 1117.98).

**Fig 7:**
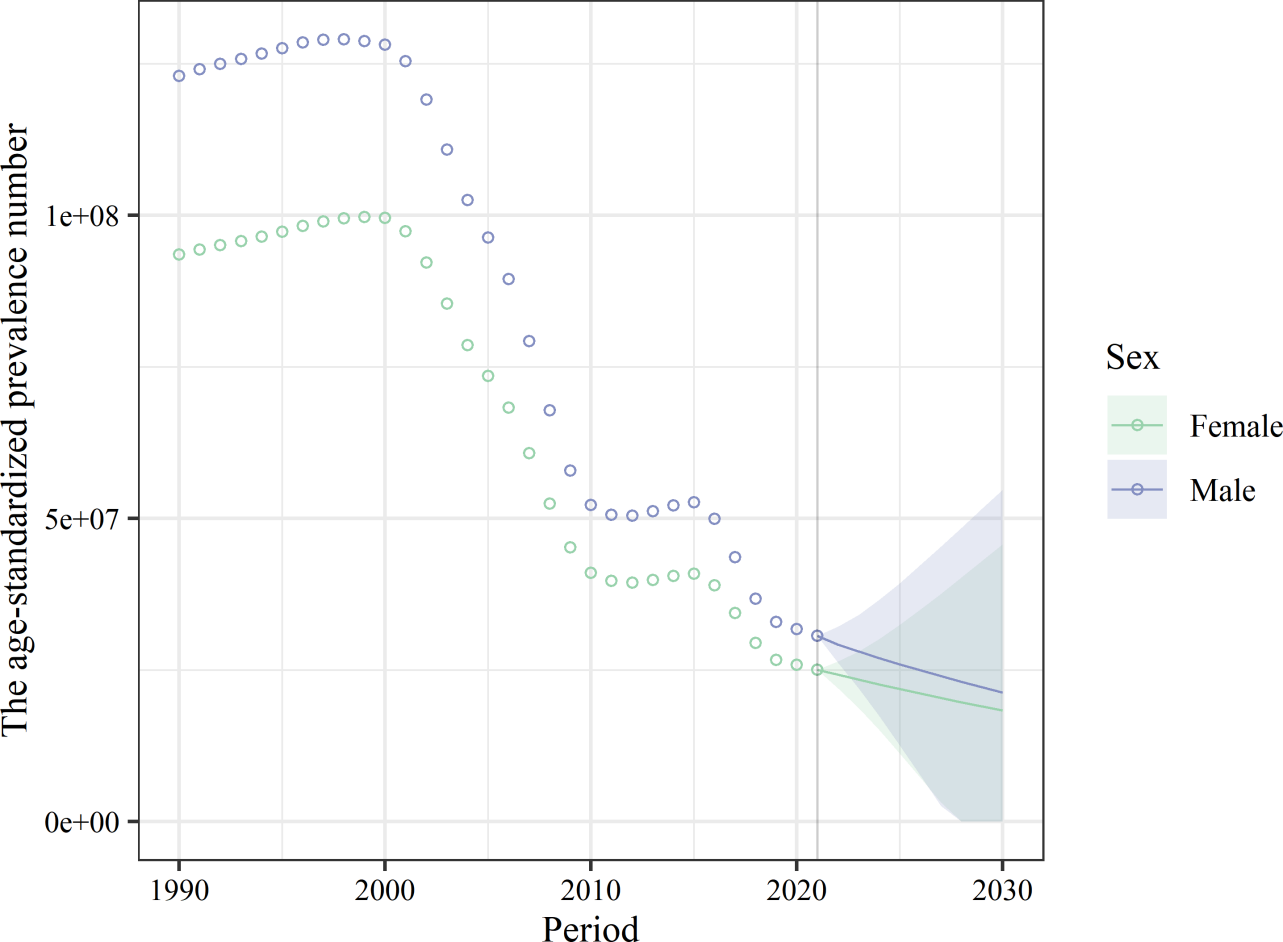
The global change trends of lymphatic filariasis age-standardized cases from 1990 to 2021, and its predicted trends between 2022 and 2030. The posterior distribution 95% credibility intervals for age-standardized prevalence rate are plotted with colors.

At the national level, the decline in age-standardized prevalence and cases of LF varies across countries. The country with the highest predicted age-standardized cases of LF in 2030 is India with 12.43 million (95% CrI: 0.00 to 29.79 million), followed by Indonesia with 1.47 million (95% CrI: 0.38 to 2.56 million). The country with the highest predicted age-standardized prevalence was Guyana, with an age-standardized prevalence of 9976.40 (95% CrI: 4433.26, 15519.55), followed by Liberia with 7050.72 (95% CrI: 0.00 to 32,162.77), as shown in Fig 7 and S12 Table.

## Discussion

Using the comprehensive and representative GBD 2021 dataset, this study examined the age-standardized prevalence and DALY rates, as well as the number of cases and DALYs for LF. It compared gender- and age-group heterogeneity, explored temporal trends across different SDI levels, and analyzed regional and national variations. Additionally, the study assessed cross-country inequalities in age-standardized prevalence and DALY rates and predicted LF prevalence and cases from 2022 to 2030. The findings indicated that, in 2021, the burden of LF varied by age, sex, and region, with a notable concentration among males aged 15–49 years and in the low-SDI areas. From 1990 to 2021, the global age-standardized prevalence and DALY rates declined, and predictions suggested this downward trend will persist until 2030. Adjustments for aging and population growth revealed these factors as primary drivers of the decline in LF cases and DALY numbers. The burden of LF has been heavily concentrated in underdeveloped and disadvantaged regions, but cross-national inequalities have been narrowing rapidly over the past decade. The study’s key findings are as follows:

### Efforts of LF control in the past decades and potential risks

The overall trend in the burden of disease for LF has been markedly downward, reflecting global efforts on LF over the past 30 years. This trend was probably related to the broad support for actions to eliminate LF, such as the development of the GPELF and the Neglected Tropical Diseases Roadmap 2021–2030 by WHO, and certain actions in the areas of coordination of key partners, disease surveillance and prevention, and organization of advocacy for financing. These strategies and actions have reduced the disease burden of LF and provided a significant contribution to the global elimination of LF as a public health problem [5,46]. Despite these efforts, the 2021 age-standardized prevalence and DALY rates remained relatively high in low-SDI regions, underscoring the persistent severity of the LF epidemic in these areas. This challenge is often linked to weaker public health systems and insufficient water sanitation infrastructure. Limited healthcare capacity and human resource shortages may hinder the effectiveness of LF surveillance, epidemiological investigations, and mass drug administration coverage. Additionally, poor sanitation, including inadequate household latrines and drinking water facilities, exacerbates vector-borne transmission risks, particularly in densely populated, low-income communities [47–49]. Further complicating LF control efforts are factors such as migration, political instability, conflict, and climate change, all of which could significantly impact future sanitation and NTD management [50]. Addressing these challenges requires flexible, context-specific interventions that go beyond expanding sanitation infrastructure and water programs. Strengthening targeted, resource-efficient strategies in vulnerable and underserved areas will be critical to sustaining progress toward LF elimination [51,52].

### The vulnerable populations of LF

In 2021, the crude prevalence of LF and DALY rates was concentrated in the male population aged 15–44 years, especially in the male population aged 25–29 years, which is a similar trend to that observed in previous studies [16,53]. Qualitative research from Nepal has suggested that higher prevalence in the male population may be associated with more frequent outdoor activities and gender differences in socio-cultural norms (e.g., patterns of dress), and therefore a greater likelihood of being bitten by parasite-carrying mosquitoes [54]. Apart from the frequency of exposure to vectors, a hypothesis from serology argued that women of childbearing age probably have more immunity to LF infection [55]. Higher rates of LF DALY in men were probably related to the additional disability caused by hydrocele [56], and men with hydrocele also experienced a greater psychosocial burden [57]. Additionally, socioeconomic status was recognized as a central factor in the health of populations and profoundly affects health inequalities in populations, with higher socioeconomic status meaning better accessibility and affordability of health care [63]. It has been widely demonstrated that in low-income households, family members infected with LF often have difficulties in accessing appropriate treatment and maintaining hygiene and adherence to medical prescriptions due to factors such as poorer living conditions, the hazardous nature of their work, and low health awareness [58,59]. Common pathological manifestations such as lower extremity lymphedema could also interfere with occupational activities, limit social participation, and result in reduced work capacity or even unemployment [60,61], all of which push families with already limited income into near abject poverty. Combined with the cross-country inequality analysis in 2021, for low SDI countries with limited capacity, strengthening age-specific monitoring and prevention for both men and women, especially among low-income groups in the most vulnerable poorer areas, may help to prevent LF from creating a new vicious cycle of poverty [62].

### Prevention and control of LF in priority areas

In 2021, at the national level, the number of LF cases and DALYs was concentrated in India, Indonesia, and Nigeria. Projections based on the available dataset showed a declining trend in the age-standardized cases of LF from 2022 to 2030, and the number of LF cases in India, Indonesia, and Nigeria remained worrisome, but the broad CrI suggests caution in interpreting the predictions. Using rate indicators to quantify disease burden and severity, the age-standardized prevalence of LF and DALY rates in 2021 in Oceania were 7646.47 (95% UI: 2970.42, 18140.42) per 100,000 population and 163.51 (95% UI: 107.49, 237.26) per 100,000 population, respectively. This was much higher than in other regions, indicating that the population in this region is at higher risk of developing LF and bears heavier DALYs. The risk of LF and the burden of disease in the region were concentrated in Papua New Guinea (PNG) and Fiji, which is more consistent with earlier findings from Oceania [63–65]. Multiple rounds of national MDA (such as ivermectin, diethylcarbamazine, and albendazole [66]) are still needed in PNG and Fiji to block the spread of LF at the population level [67]. Especially in PNGs with relatively large population bases, complex geography, diverse vector populations, and limited health capacity, MDA coverage for at-risk populations (e.g., Highlands Province) is at a low level [68,69]. Since the Pacific Programme to Eliminate Lymphatic Filariasis (PacELF) was officially launched in 1999, through the planning and funding of PacELF, as of 2020, many Pacific islands such as Kiribati and the Marshall Islands have successfully eliminated LF as a public health problem and have been confirmed by WHO. By 2020, Kiribati, the Marshall Islands, and many other Pacific islands will have successfully eliminated LF as a public health problem, which has been confirmed by the WHO [69]. Because of the high residual age-standardized prevalence rates estimated in the above-mentioned countries even after the elimination of LF, measures such as prevention of recurrence of transmission and morbidity surveillance are necessary [70]. Given the geographic heterogeneity of LF prevalence at the national level, targeted strategies are needed to define and understand areas at high risk of LF prevalence, such as Oceania, to accelerate LF elimination through evidence-based interventions [71].

This study still has some potential limitations. Firstly, the data for GBD 2021 were derived from multiple secondary data sources, and the use of model-estimated disease prevalence to assess district trends in a data-poor setting may mask the true situation in the districts. Second, over-sampling and unreliable screening methods targeting areas with high prevalence may also overestimate/underestimate the true prevalence of LF [72–74], affecting the veracity of secondary data. Finally, the impact of the COVID pandemic on LF has not been estimated due to the lack of available data.

## Conclusion

In summary, based on the comprehensive GBD 2021 dataset, the findings indicate that significant progress has been made globally and nationally in eliminating LF, with cross-national inequalities narrowing by 2021. The disease burden of LF was primarily concentrated among individuals aged 15–44 years, males, and populations with low SDI. Both the prevalence and DALY rates of LF are predicted to decline from 2022 to 2030. These findings underscore the need for more rigorous health interventions and targeted public health policies, particularly in less developed regions. Special attention should be given to low-income populations in highly vulnerable areas, such as Oceania, to promote health and well-being among at-risk groups.

## Supporting information

S1 Table Regional categories of 67 countries and territories by GBD. Abbreviations: GBD, Global Burden of Disease.
(DOCX)

S2 Table The Socio-demographic Index for 67 countries and territories, 1990 and 2021. Abbreviations: GBD, Global Burden of Disease.
(DOCX)

S3 Table The age-standardized prevalence and DALY rates (per 100,000) of lymphatic filariasis, and estimated annual percentage change in the age-standardized rates, among 67 countries and territories, from 1990 to 2021. Abbreviations: GBD, Global Burden of Disease, ASR, age-standardized rate; DALYs, disability adjusted life years; SDI, sociodemographic index; EAPC, estimated annual percentage change; UI, uncertainty interval; CI, conﬁdence interval.
(DOCX)

S4 Table The crude prevalence and DALY rates (per 100,000) of lymphatic filariasis, and estimated annual percentage change in crude rates, by sex, SDI levels, GBD regions, among 67 countries and territories, from 1990 to 2021. Abbreviations: GBD, Global Burden of Disease, DALYs, disability-adjusted life years; SDI, socio-demographic index; EAPC, estimated annual percentage change; UI, uncertainty interval; CI, conﬁdence interval.
(DOCX)

S5 Table The number of cases and DALYs of lymphatic filariasis, by sex, SDI levels, GBD regions, among 67 countries and territories, from 1990 to 2021. Abbreviations: GBD, Global Burden of Disease, DALYs, disability-adjusted life years; SDI, socio-demographic index; EAPC, estimated annual percentage change; UI, uncertainty interval; CI, conﬁdence interval.
(DOCX)

S6 Table The crude prevalence and DALY rates (per 100,000) of lymphatic filariasis, by age group, sex, SDI levels, GBD regions, among 67 countries and territories, in 1990. Abbreviations: GBD, Global Burden of Disease, DALYs, disability-adjusted life years; SDI, socio-demographic index; UI, uncertainty interval.
(DOCX)

S7 Table The crude prevalence and DALY rates (per 100,000) of lymphatic filariasis, by age group, sex, SDI levels, GBD regions, among 67 countries and territories, in 2021. Abbreviations: GBD, Global Burden of Disease, DALYs, disability-adjusted life years; SDI, socio-demographic index; UI, uncertainty interval.
(DOCX)

S8 Table The number of prevalence and DALYs of lymphatic filariasis, by age group, sex, SDI levels, GBD regions, among 67 countries and territories, in 1990. Abbreviations: GBD, Global Burden of Disease, DALYs, disability-adjusted life years; SDI, socio-demographic index; UI, uncertainty interval.
(DOCX)

S9 Table The number of prevalence and DALYs of lymphatic filariasis, by age group, sex, SDI levels, GBD regions, among 67 countries and territories, in 2021. Abbreviations: GBD, Global Burden of Disease, DALYs, disability-adjusted life years; SDI, socio-demographic index; UI, uncertainty interval.
(DOCX)

S10 Table Changes in DALYs number of lymphatic filariasis according to population-level determinants, including aging, population growth, and epidemiological change, by sex, SDI levels, GBD regions, among 67 countries and territories, from 1990 to 2021. Abbreviations: GBD, Global Burden of Disease, SDI, sociodemographic index; DALY, disability-adjusted life years.
(DOCX)

S11 Table Changes in case numbers of lymphatic filariasis according to population-level determinants, including aging, population growth, and epidemiological change, by sex, SDI levels, GBD regions, among 67 countries and territories, from 1990 to 2021. Abbreviations: GBD, Global Burden of Disease, SDI, sociodemographic index; DALYs, disability adjusted life years.
(DOCX)

S12 Table The predicted age-standardized rates (per 100,000) and number of cases of lymphatic filariasis, by sex, at the global level, among 65 countries and territories1, from 2022 to 2030. Abbreviations: ASR, age-standardized rate; CrI, credible interval. ^1^Niue and Palau are excluded from Institute for Health Metrics and Evaluation population forecast data, thus inapplicable to BAPC.
(DOCX)

S1 Fig The age-standardized DALY rates (per 100,000) of lymphatic filariasis, among 67 countries and territories in 1990. The shapefiles used for spatial visualization are sourced from Natural Earth. The terms of free use for these shapefiles can be found at: https://www.naturalearthdata.com/about/terms-of-use/, the shapefiles available at: https://www.naturalearthdata.com/downloads/10m-cultural-vectors/10m-admin-0-countries/.
(TIF)

S2 Fig The age-standardized DALY rates (per 100,000) of lymphatic filariasis, among 67 countries and territories in 2021. The shapefiles used for spatial visualization are sourced from Natural Earth. The terms of free use for these shapefiles can be found at: https://www.naturalearthdata.com/about/terms-of-use/, the shapefiles available at: https://www.naturalearthdata.com/downloads/10m-cultural-vectors/10m-admin-0-countries/.
(TIF)

S3 Fig The age-standardized Prevalence (per 100,000) of lymphatic filariasis, among 67 countries and territories in 1990. The shapefiles used for spatial visualization are sourced from Natural Earth. The terms of free use for these shapefiles can be found at: https://www.naturalearthdata.com/about/terms-of-use/, the shapefiles available at: https://www.naturalearthdata.com/downloads/10m-cultural-vectors/10m-admin-0-countries/.
(TIF)

S4 Fig Changes in lymphatic filariasis case numbers according to population-level determinants including aging, population growth and epidemiological change, by sex, SDI quintiles, from 1990 to 2021. **(A) Both sex (B) Male (C) Female.** Abbreviations: SDI, socio-demographic index; DALYs, disability adjusted life years.
(TIF)

S5 Fig Changes in lymphatic filariasis case numbers according to population-level determinants including aging, population growth and epidemiological change, by sex, GBD regions, from 1990 to 2021. **(A) Both sex (B) Male (C) Female.** Abbreviations: SDI, socio-demographic index; DALYs, disability adjusted life years.
(TIF)
