# Supplementary material for: Global, regional, and national disability-adjusted life years and prevalence of lymphatic filariasis from 1990 to 2021: A trend and health inequality analysis based on the global burden of disease study 2021
Source: PLoS Negl Trop Dis. 2025 Apr 29;19(4):e0013017. doi: 10.1371/journal.pntd.0013017 (PMC12040265; doi:10.1371/journal.pntd.0013017)
Supplement: S1 Table — Abbreviations: GBD, Global Burden of Disease. (DOCX) [file pntd.0013017.s001.docx]

**S1 Table Regional categories of 67 countries and territories by GBD.**

| **GBD region** | **Country and territory** |
| --- | --- |
| Caribbean | Dominican Republic |
| Caribbean | Guyana |
| Caribbean | Haiti |
| Central sub-Saharan Africa | Angola |
| Central sub-Saharan Africa | Central African Republic |
| Central sub-Saharan Africa | Congo |
| Central sub-Saharan Africa | Democratic Republic of the Congo |
| Central sub-Saharan Africa | Equatorial Guinea |
| Central sub-Saharan Africa | Gabon |
| Eastern sub-Saharan Africa | Comoros |
| Eastern sub-Saharan Africa | Eritrea |
| Eastern sub-Saharan Africa | Ethiopia |
| Eastern sub-Saharan Africa | Kenya |
| Eastern sub-Saharan Africa | Madagascar |
| Eastern sub-Saharan Africa | Malawi |
| Eastern sub-Saharan Africa | Mozambique |
| Eastern sub-Saharan Africa | United Republic of Tanzania |
| Eastern sub-Saharan Africa | Uganda |
| Eastern sub-Saharan Africa | Zambia |
| Eastern sub-Saharan Africa | South Sudan |
| High-income Asia Pacific | Brunei Darussalam |
| North Africa and Middle East | Egypt |
| North Africa and Middle East | Yemen |
| North Africa and Middle East | Sudan |
| Oceania | Fiji |
| Oceania | Kiribati |
| Oceania | Marshall Islands |
| Oceania | Micronesia (Federated States of) |
| Oceania | Papua New Guinea |
| Oceania | Samoa |
| Oceania | Tonga |
| Oceania | Vanuatu |
| Oceania | American Samoa |
| Oceania | Niue |
| Oceania | Palau |
| South Asia | Bangladesh |
| South Asia | India |
| South Asia | Nepal |
| Southeast Asia | Cambodia |
| Southeast Asia | Indonesia |
| Southeast Asia | Lao People's Democratic Republic |
| Southeast Asia | Malaysia |
| Southeast Asia | Maldives |
| Southeast Asia | Myanmar |
| Southeast Asia | Philippines |
| Southeast Asia | Sri Lanka |
| Southeast Asia | Thailand |
| Southeast Asia | Timor-Leste |
| Southeast Asia | Viet Nam |
| Southern sub-Saharan Africa | Zimbabwe |
| Tropical Latin America | Brazil |
| Western sub-Saharan Africa | Benin |
| Western sub-Saharan Africa | Burkina Faso |
| Western sub-Saharan Africa | Cameroon |
| Western sub-Saharan Africa | Chad |
| Western sub-Saharan Africa | Côte d'Ivoire |
| Western sub-Saharan Africa | Ghana |
| Western sub-Saharan Africa | Guinea |
| Western sub-Saharan Africa | Guinea-Bissau |
| Western sub-Saharan Africa | Liberia |
| Western sub-Saharan Africa | Mali |
| Western sub-Saharan Africa | Niger |
| Western sub-Saharan Africa | Nigeria |
| Western sub-Saharan Africa | Sao Tome and Principe |
| Western sub-Saharan Africa | Senegal |
| Western sub-Saharan Africa | Sierra Leone |
| Western sub-Saharan Africa | Togo |
| **Abbreviations:** GBD, Global Burden of Disease. | |
