# Supplementary material for: Global, regional, and national disability-adjusted life years and prevalence of lymphatic filariasis from 1990 to 2021: A trend and health inequality analysis based on the global burden of disease study 2021
Source: PLoS Negl Trop Dis. 2025 Apr 29;19(4):e0013017. doi: 10.1371/journal.pntd.0013017 (PMC12040265; doi:10.1371/journal.pntd.0013017)
Supplement: S2 Table — Abbreviations: GBD, Global Burden of Disease. (DOCX) [file pntd.0013017.s002.docx]

**S2 Table** **The Socio-demographic Index for 67 countries and territories, 1990 and 2021.**

| **location** | **1990 SDI Index Value** | **2021 SDI Index Value** | **SDI Quintile in 2021** |
| --- | --- | --- | --- |
| Niger | 0.081 | 0.168 | Low SDI |
| Chad | 0.115 | 0.240 | Low SDI |
| Mali | 0.127 | 0.269 | Low SDI |
| South Sudan | 0.207 | 0.278 | Low SDI |
| Burkina Faso | 0.130 | 0.285 | Low SDI |
| Central African Republic | 0.217 | 0.309 | Low SDI |
| Mozambique | 0.173 | 0.326 | Low SDI |
| Guinea | 0.178 | 0.336 | Low SDI |
| Liberia | 0.235 | 0.352 | Low SDI |
| Guinea-Bissau | 0.208 | 0.353 | Low SDI |
| Ethiopia | 0.148 | 0.359 | Low SDI |
| Sierra Leone | 0.212 | 0.359 | Low SDI |
| Benin | 0.219 | 0.373 | Low SDI |
| Democratic Republic of the Congo | 0.290 | 0.383 | Low SDI |
| Malawi | 0.204 | 0.385 | Low SDI |
| Madagascar | 0.280 | 0.400 | Low SDI |
| Eritrea | 0.216 | 0.404 | Low SDI |
| Senegal | 0.238 | 0.408 | Low SDI |
| Togo | 0.270 | 0.409 | Low SDI |
| Papua New Guinea | 0.311 | 0.418 | Low SDI |
| Uganda | 0.187 | 0.423 | Low SDI |
| Côte d'Ivoire | 0.279 | 0.426 | Low SDI |
| Nepal | 0.200 | 0.433 | Low SDI |
| Timor-Leste | 0.262 | 0.445 | Low SDI |
| United Republic of Tanzania | 0.259 | 0.447 | Low SDI |
| Haiti | 0.310 | 0.448 | Low SDI |
| Yemen | 0.216 | 0.450 | Low SDI |
| Angola | 0.271 | 0.454 | Low SDI |
| Vanuatu | 0.353 | 0.473 | Low-middle SDI |
| Cambodia | 0.289 | 0.474 | Low-middle SDI |
| Zimbabwe | 0.399 | 0.474 | Low-middle SDI |
| Comoros | 0.270 | 0.476 | Low-middle SDI |
| Cameroon | 0.303 | 0.480 | Low-middle SDI |
| Lao People's Democratic Republic | 0.264 | 0.489 | Low-middle SDI |
| Bangladesh | 0.229 | 0.492 | Low-middle SDI |
| Nigeria | 0.306 | 0.503 | Low-middle SDI |
| Sao Tome and Principe | 0.310 | 0.505 | Low-middle SDI |
| Zambia | 0.304 | 0.506 | Low-middle SDI |
| Kenya | 0.334 | 0.524 | Low-middle SDI |
| Kiribati | 0.410 | 0.527 | Low-middle SDI |
| Myanmar | 0.319 | 0.534 | Low-middle SDI |
| Sudan | 0.292 | 0.542 | Low-middle SDI |
| Ghana | 0.373 | 0.565 | Low-middle SDI |
| Marshall Islands | 0.431 | 0.574 | Low-middle SDI |
| India | 0.333 | 0.575 | Low-middle SDI |
| Congo | 0.421 | 0.583 | Low-middle SDI |
| Micronesia (Federated States of) | 0.463 | 0.588 | Low-middle SDI |
| Samoa | 0.487 | 0.593 | Low-middle SDI |
| Egypt | 0.417 | 0.607 | Low-middle SDI |
| Dominican Republic | 0.443 | 0.619 | Middle SDI |
| Tonga | 0.492 | 0.626 | Middle SDI |
| Viet Nam | 0.408 | 0.628 | Middle SDI |
| Gabon | 0.455 | 0.635 | Middle SDI |
| Maldives | 0.332 | 0.651 | Middle SDI |
| Philippines | 0.510 | 0.651 | Middle SDI |
| Guyana | 0.460 | 0.651 | Middle SDI |
| Brazil | 0.500 | 0.653 | Middle SDI |
| Indonesia | 0.457 | 0.657 | Middle SDI |
| Equatorial Guinea | 0.269 | 0.658 | Middle SDI |
| Fiji | 0.535 | 0.675 | Middle SDI |
| Thailand | 0.507 | 0.683 | Middle SDI |
| Sri Lanka | 0.523 | 0.702 | Middle SDI |
| American Samoa | 0.614 | 0.724 | High-middle SDI |
| Niue | 0.588 | 0.726 | High-middle SDI |
| Malaysia | 0.546 | 0.743 | High-middle SDI |
| Palau | 0.663 | 0.754 | High-middle SDI |
| Brunei Darussalam | 0.666 | 0.810 | High-middle SDI |

**Abbreviations:** GBD, Global Burden of Disease.
