# Supplementary material for: Global, regional, and national disability-adjusted life years and prevalence of lymphatic filariasis from 1990 to 2021: A trend and health inequality analysis based on the global burden of disease study 2021
Source: PLoS Negl Trop Dis. 2025 Apr 29;19(4):e0013017. doi: 10.1371/journal.pntd.0013017 (PMC12040265; doi:10.1371/journal.pntd.0013017)
Supplement: S3 Table — Abbreviations: GBD, Global Burden of Disease, ASR, age-standardized rate; DALYs, disability adjusted life years; SDI, sociodemographic index; EAPC, estimated annual percentage change; UI, uncertainty interval; CI, conﬁdence interval. (DOCX) [file pntd.0013017.s003.docx]

**S3 Table** **The age-standardized prevalence and DALY rates (per 100,000) of lymphatic filariasis, and estimated annual percentage change in the age-standardized rates, among 67 countries and territories, from 1990 to 2021.**

| **Location** | **Age-standardized prevalence (95% UI)** | | **EAPC (95% CI)** | **Age-standardized DALY rates (95% UI)** | | **EAPC (95% CI)** |
| --- | --- | --- | --- | --- | --- | --- |
|  | **1990** | **2021** | **1990-2021** | **1990** | **2021** | **1990-2021** |
| American Samoa | 9283.15 (8004.61 to 10780.16) | 4165.65 (1753.50 to 9489.17) | -6.75 (-9.13 to -4.31) | 201.11 (131.76 to 287.66) | 73.12 (41.99 to 121.28) | -7.22 (-9.44 to -4.95) |
| Angola | 8469.42 (2334.60 to 25088.93) | 1146.99 (344.87 to 3591.49) | -8.18 (-9.25 to -7.11) | 175.89 (115.15 to 255.23) | 29.70 (17.07 to 52.84) | -7.52 (-8.48 to -6.54) |
| Bangladesh | 4666.49 (4065.10 to 5335.64) | 322.58 (268.86 to 438.37) | -10.47 (-11.53 to -9.39) | 85.44 (49.46 to 141.07) | 23.32 (14.06 to 40.06) | -5.3 (-6 to -4.59) |
| Benin | 2970.17 (594.56 to 13480.89) | 796.87 (526.51 to 1245.71) | -5.59 (-6.42 to -4.77) | 50.30 (28.60 to 84.51) | 27.37 (15.89 to 48.48) | -2.71 (-3.14 to -2.27) |
| Brazil | 189.98 (136.12 to 359.26) | 8.21 (6.98 to 13.99) | -10.71 (-11.62 to -9.79) | 4.70 (3.09 to 7.04) | 0.70 (0.49 to 1.02) | -6.85 (-7.3 to -6.4) |
| Brunei Darussalam | 1758.80 (265.49 to 13085.78) | 946.30 (223.76 to 5124.79) | -3.74 (-4.92 to -2.55) | 38.13 (21.36 to 67.70) | 29.70 (16.59 to 53.28) | -1.39 (-1.77 to -1.01) |
| Burkina Faso | 23232.26 (7436.99 to 51001.53) | 656.65 (361.66 to 1417.75) | -13.58 (-16.02 to -11.06) | 393.28 (270.37 to 530.14) | 26.28 (15.60 to 46.85) | -11 (-13.13 to -8.81) |
| Cambodia | 3497.82 (500.72 to 21357.13) | 120.04 (2.03 to 779.59) | -13.75 (-15.92 to -11.52) | 57.11 (33.11 to 94.02) | 0.00 (0.00 to 0.00) | - |
| Cameroon | 4583.88 (1459.33 to 12120.63) | 305.78 (221.32 to 463.82) | -10.18 (-11.63 to -8.71) | 79.20 (45.95 to 129.37) | 23.25 (13.73 to 40.17) | -6.33 (-7.28 to -5.36) |
| Central African Republic | 24799.23 (8801.26 to 56277.10) | 4427.90 (1118.06 to 13869.14) | -6.07 (-6.96 to -5.18) | 416.14 (285.50 to 564.52) | 74.29 (44.04 to 121.29) | -5.66 (-6.54 to -4.78) |
| Chad | 3886.59 (1072.36 to 12004.58) | 791.21 (304.72 to 2443.96) | -5.53 (-6.8 to -4.25) | 65.04 (37.50 to 107.49) | 27.57 (15.71 to 49.59) | -3.39 (-4.16 to -2.61) |
| Comoros | 12848.79 (2643.43 to 59277.84) | 3800.81 (585.64 to 18418.36) | -4.52 (-5.17 to -3.86) | 275.42 (185.44 to 382.50) | 65.51 (37.38 to 108.74) | -5.15 (-5.79 to -4.51) |
| Congo | 5027.03 (1215.86 to 17734.28) | 939.79 (321.61 to 2570.71) | -6.91 (-7.95 to -5.85) | 86.92 (51.79 to 139.48) | 28.59 (16.65 to 50.47) | -4.64 (-5.2 to -4.07) |
| Coted'Ivoire | 38787.75 (16376.05 to 71902.10) | 7684.98 (2924.26 to 17988.83) | -5.69 (-6.45 to -4.93) | 688.15 (475.73 to 968.98) | 159.96 (102.79 to 234.79) | -4.88 (-5.54 to -4.21) |
| Democratic Republic of the Congo | 16791.53 (9244.84 to 27537.35) | 2223.67 (1055.09 to 4441.76) | -8.06 (-9.21 to -6.89) | 326.27 (220.87 to 446.60) | 41.12 (23.15 to 71.31) | -8.38 (-9.65 to -7.1) |
| Dominican Republic | 6872.38 (1651.05 to 26484.67) | 1338.60 (361.82 to 4758.35) | -10.23 (-13.07 to -7.3) | 133.80 (85.21 to 200.90) | 32.32 (18.71 to 57.20) | -8.51 (-10.59 to -6.39) |
| Egypt | 3278.99 (669.75 to 13719.41) | 581.81 (62.68 to 2526.12) | -10.11 (-11.79 to -8.4) | 57.97 (33.02 to 98.18) | 0.00 (0.00 to 0.00) | - |
| Equatorial Guinea | 14487.03 (3101.33 to 47674.37) | 1316.66 (321.02 to 5762.15) | -9.57 (-10.88 to -8.23) | 283.47 (189.93 to 391.50) | 32.11 (18.07 to 58.03) | -9.15 (-10.48 to -7.8) |
| Eritrea | 1457.68 (318.90 to 6121.21) | 424.44 (200.19 to 1118.15) | -5.74 (-6.46 to -5.01) | 32.34 (18.40 to 56.70) | 24.84 (14.76 to 44.54) | -1.36 (-1.55 to -1.16) |
| Ethiopia | 5817.28 (1995.68 to 13810.82) | 607.26 (311.74 to 1295.81) | -9.47 (-10.46 to -8.47) | 109.60 (67.93 to 168.73) | 26.34 (15.47 to 47.20) | -6.13 (-6.87 to -5.39) |
| Fiji | 26164.34 (6648.94 to 67591.93) | 9487.12 (2655.77 to 26045.32) | -4.59 (-5.85 to -3.31) | 454.57 (313.37 to 621.65) | 201.55 (133.68 to 288.56) | -3.98 (-5.3 to -2.64) |
| Gabon | 4576.09 (1032.97 to 17939.56) | 1414.34 (335.96 to 5706.92) | -5.82 (-6.98 to -4.65) | 79.30 (46.29 to 128.80) | 32.19 (18.42 to 56.48) | -4.36 (-5.14 to -3.57) |
| Ghana | 8326.96 (2605.93 to 22362.97) | 804.11 (319.30 to 2155.21) | -10.44 (-11.5 to -9.38) | 169.56 (111.08 to 248.19) | 27.27 (15.98 to 47.47) | -9.04 (-10.21 to -7.86) |
| Guinea | 6792.89 (1703.30 to 19386.90) | 743.03 (302.91 to 2038.61) | -7.12 (-7.77 to -6.47) | 129.64 (82.67 to 194.99) | 26.92 (15.50 to 47.95) | -5.81 (-6.39 to -5.23) |
| Guinea-Bissau | 18707.39 (3506.14 to 64628.45) | 1244.14 (294.35 to 5350.61) | -7.23 (-8.85 to -5.59) | 347.60 (237.01 to 471.27) | 30.73 (17.77 to 54.38) | -6.6 (-8.24 to -4.92) |
| Guyana | 40943.33 (22413.07 to 65463.57) | 16158.49 (5921.82 to 33023.58) | -3.39 (-3.85 to -2.93) | 731.86 (500.67 to 1039.54) | 322.77 (219.64 to 443.01) | -2.94 (-3.36 to -2.52) |
| Haiti | 24435.21 (5641.58 to 65264.75) | 1093.10 (568.88 to 2308.35) | -12.95 (-14.59 to -11.29) | 416.06 (284.69 to 563.73) | 29.57 (16.96 to 53.05) | -11.2 (-12.67 to -9.71) |
| India | 13078.58 (12156.99 to 14073.78) | 2326.06 (2022.48 to 2700.89) | -5.33 (-5.81 to -4.85) | 254.17 (174.03 to 355.44) | 47.40 (27.61 to 79.21) | -5.11 (-5.59 to -4.63) |
| Indonesia | 13761.33 (5561.81 to 32163.03) | 1476.16 (605.38 to 3845.58) | -7.91 (-8.28 to -7.53) | 283.78 (188.73 to 395.47) | 35.76 (20.41 to 62.51) | -7.42 (-7.82 to -7.03) |
| Kenya | 14520.01 (5790.54 to 33678.79) | 1054.81 (397.58 to 3064.43) | -10.33 (-12.23 to -8.39) | 279.54 (188.32 to 389.16) | 30.54 (17.63 to 53.95) | -8.98 (-10.62 to -7.31) |
| Kiribati | 40213.03 (7197.53 to 70800.81) | 1455.49 (297.24 to 4976.06) | -12.15 (-12.99 to -11.31) | 720.79 (496.99 to 1023.15) | 32.58 (18.45 to 57.44) | -11.36 (-12.15 to -10.56) |
| Lao People's Democratic Republic | 3807.16 (691.08 to 16679.75) | 510.73 (214.88 to 1773.68) | -7.8 (-8.91 to -6.69) | 64.63 (37.49 to 107.36) | 25.84 (14.99 to 46.33) | -3.5 (-4.13 to -2.87) |
| Liberia | 26347.93 (7148.99 to 64048.69) | 11813.12 (9265.72 to 15003.63) | -5.31 (-6.83 to -3.76) | 450.81 (310.45 to 613.96) | 259.41 (176.03 to 361.54) | -4.54 (-6.13 to -2.91) |
| Madagascar | 15621.82 (6609.50 to 30898.51) | 1049.81 (420.20 to 2510.21) | -11.81 (-13.39 to -10.2) | 322.33 (216.87 to 445.87) | 29.46 (16.82 to 52.40) | -10.46 (-11.9 to -9) |
| Malawi | 22636.59 (6786.50 to 56428.05) | 440.90 (305.62 to 701.20) | -14.53 (-16.53 to -12.47) | 398.07 (274.68 to 541.67) | 24.82 (14.39 to 43.50) | -11.53 (-13.29 to -9.74) |
| Malaysia | 4700.68 (987.23 to 17851.44) | 1033.33 (321.49 to 3771.44) | -5.72 (-6.39 to -5.04) | 83.80 (48.73 to 137.72) | 30.11 (16.72 to 53.43) | -3.75 (-4.29 to -3.22) |
| Maldives | 3949.00 (3498.61 to 4382.91) | 401.63 (180.39 to 955.57) | -11.15 (-14.92 to -7.21) | 71.71 (40.99 to 119.12) | 26.69 (15.01 to 48.89) | -5.58 (-8.53 to -2.54) |
| Mali | 36031.71 (16736.31 to 63743.13) | 1548.32 (465.64 to 4959.48) | -12.66 (-14.74 to -10.54) | 610.22 (421.71 to 861.05) | 33.98 (19.22 to 60.11) | -11.33 (-13.08 to -9.55) |
| Marshall Islands | 1019.41 (269.73 to 1873.46) | 151.63 (141.15 to 164.16) | -8.17 (-9.35 to -6.98) | 29.68 (17.09 to 53.23) | 14.76 (9.95 to 21.11) | -3.1 (-3.59 to -2.6) |
| Micronesia (Federated States of) | 15044.15 (5185.10 to 36119.65) | 2284.47 (387.61 to 15628.36) | -5.12 (-7.72 to -2.45) | 320.22 (214.23 to 442.55) | 42.77 (23.76 to 75.30) | -4.94 (-6.91 to -2.92) |
| Mozambique | 49160.11 (31233.76 to 68500.82) | 998.67 (417.93 to 2361.26) | -12.37 (-14.31 to -10.38) | 972.45 (659.83 to 1384.01) | 28.34 (16.35 to 49.38) | -11.86 (-13.71 to -9.97) |
| Myanmar | 16849.42 (3986.35 to 48123.38) | 1640.53 (369.37 to 6331.40) | -9.65 (-11.1 to -8.17) | 334.16 (227.51 to 455.29) | 34.51 (19.82 to 60.84) | -9.49 (-10.83 to -8.13) |
| Nepal | 14620.15 (12845.44 to 16702.72) | 2527.90 (1959.66 to 3423.10) | -6.63 (-7.29 to -5.97) | 306.92 (207.44 to 425.82) | 43.84 (25.03 to 75.54) | -7.53 (-8.35 to -6.71) |
| Niger | 23198.53 (8236.58 to 46475.83) | 1518.44 (735.49 to 3711.05) | -10.53 (-12.01 to -9.03) | 411.75 (283.68 to 563.99) | 33.73 (18.89 to 60.61) | -9.94 (-11.41 to -8.44) |
| Nigeria | 18057.10 (8019.93 to 32907.81) | 1503.42 (629.43 to 3027.04) | -8.03 (-9.34 to -6.69) | 352.87 (241.01 to 476.46) | 33.50 (19.33 to 57.84) | -7.78 (-8.97 to -6.58) |
| Niue | 5559.04 (1723.85 to 14961.63) | 1462.58 (424.25 to 4717.93) | -4.72 (-5.95 to -3.47) | 101.70 (61.08 to 162.18) | 33.45 (18.79 to 59.50) | -3.71 (-4.57 to -2.85) |
| Palau | 192.23 (167.86 to 227.42) | 166.82 (152.81 to 187.87) | -0.33 (-0.37 to -0.3) | 16.05 (10.71 to 22.88) | 14.64 (9.91 to 21.00) | -0.15 (-0.21 to -0.09) |
| Papua New Guinea | 41763.06 (19123.94 to 70849.06) | 8717.93 (2917.79 to 21797.35) | -5.84 (-6.2 to -5.48) | 773.29 (531.20 to 1096.95) | 187.50 (123.26 to 271.62) | -4.99 (-5.31 to -4.67) |
| Philippines | 10623.59 (2735.11 to 30166.42) | 882.78 (517.37 to 1957.59) | -9.95 (-10.84 to -9.05) | 219.68 (147.07 to 309.24) | 29.51 (17.26 to 52.26) | -8.39 (-9.39 to -7.38) |
| Samoa | 19025.45 (5937.56 to 49506.20) | 6838.01 (1618.22 to 25432.36) | -2.35 (-3.55 to -1.13) | 377.22 (255.84 to 514.91) | 135.65 (85.58 to 205.10) | -2.55 (-3.92 to -1.15) |
| Sao Tome and Principe | 7693.91 (1352.56 to 40760.57) | 3053.31 (474.88 to 16636.29) | -3.57 (-4.27 to -2.87) | 152.86 (97.75 to 224.83) | 53.70 (29.87 to 91.26) | -3.89 (-4.58 to -3.2) |
| Senegal | 7197.37 (1763.60 to 25054.50) | 605.89 (244.13 to 2030.94) | -8.29 (-9.15 to -7.42) | 140.82 (90.15 to 210.21) | 26.14 (15.25 to 47.02) | -6.38 (-6.97 to -5.8) |
| Sierra Leone | 33270.97 (8072.84 to 73528.01) | 4311.47 (967.99 to 13927.42) | -7.93 (-8.95 to -6.91) | 557.42 (387.23 to 784.69) | 75.53 (43.66 to 124.58) | -7.53 (-8.48 to -6.58) |
| South Sudan | 4584.38 (1376.14 to 13235.11) | 1119.74 (418.36 to 2785.32) | -5.86 (-6.89 to -4.82) | 84.97 (48.87 to 139.20) | 29.87 (17.07 to 53.08) | -4.47 (-5.27 to -3.66) |
| Sri Lanka | 1112.82 (862.13 to 1429.80) | 134.46 (98.23 to 182.83) | -8.19 (-8.9 to -7.48) | 30.33 (17.20 to 53.94) | 0.00 (0.00 to 0.00) | - |
| Sudan | 4988.83 (987.69 to 19484.23) | 967.56 (266.24 to 3582.62) | -6.05 (-6.55 to -5.55) | 90.03 (53.62 to 145.23) | 29.19 (16.76 to 52.36) | -4.36 (-4.73 to -3.99) |
| Thailand | 6061.70 (1342.86 to 24069.02) | 437.85 (23.41 to 2185.86) | -10.57 (-11.73 to -9.39) | 112.80 (69.40 to 174.13) | 0.00 (0.00 to 0.00) | - |
| Timor-Leste | 40822.79 (7941.23 to 82246.49) | 10359.28 (1998.52 to 47601.85) | -7.77 (-9.45 to -6.05) | 751.14 (515.17 to 1063.10) | 225.37 (151.40 to 320.26) | -7.42 (-9.32 to -5.48) |
| Togo | 4713.86 (923.59 to 18806.89) | 198.46 (23.67 to 842.59) | -11.62 (-13.01 to -10.21) | 81.04 (47.97 to 132.06) | 0.00 (0.00 to 0.00) | - |
| Tonga | 2670.94 (343.60 to 22363.45) | 450.53 (190.30 to 1792.92) | -7.22 (-9.27 to -5.13) | 47.86 (26.94 to 82.50) | 25.31 (14.92 to 44.78) | -2.4 (-3.38 to -1.41) |
| Uganda | 8546.14 (2300.05 to 23227.77) | 317.00 (206.38 to 627.20) | -12.24 (-14.31 to -10.11) | 173.84 (113.25 to 250.95) | 23.05 (13.80 to 39.72) | -8.4 (-9.66 to -7.12) |
| United Republic of Tanzania | 20734.72 (11247.40 to 33690.00) | 606.25 (383.12 to 1011.40) | -12.77 (-14.82 to -10.68) | 371.74 (255.76 to 502.93) | 25.99 (15.11 to 45.70) | -10.46 (-12.14 to -8.75) |
| Vanuatu | 28185.73 (4258.71 to 84290.96) | 2699.75 (336.15 to 20612.67) | -12.00 (-16.08 to -7.71) | 494.29 (340.14 to 683.20) | 48.58 (27.04 to 82.92) | -10.58 (-13.13 to -7.97) |
| Viet Nam | 11204.87 (2829.86 to 33129.11) | 767.09 (258.93 to 2977.92) | -10.39 (-11.38 to -9.39) | 232.78 (156.01 to 327.90) | 27.75 (15.95 to 49.38) | -8.06 (-9 to -7.12) |
| Yemen | 930.85 (271.03 to 3496.45) | 407.55 (208.61 to 1173.86) | -3.45 (-4.35 to -2.55) | 28.51 (16.40 to 51.55) | 24.83 (14.35 to 44.13) | -0.64 (-0.86 to -0.42) |
| Zambia | 20201.10 (9353.86 to 39938.24) | 2148.68 (727.47 to 6289.39) | -8.24 (-9.38 to -7.08) | 375.08 (257.16 to 509.19) | 40.27 (22.88 to 70.02) | -8.36 (-9.6 to -7.11) |
| Zimbabwe | 1741.78 (442.27 to 6479.36) | 895.91 (296.34 to 2977.76) | -2.29 (-3.2 to -1.38) | 35.75 (20.39 to 62.77) | 27.70 (16.09 to 48.67) | -0.9 (-1.27 to -0.52) |
| **Abbreviations:** GBD, Global Burden of Disease, ASR, age-standardized rate; DALYs, disability adjusted life years; SDI, sociodemographic index; EAPC, estimated annual percentage change; UI, uncertainty interval; CI, conﬁdence interval. | | | | | | |
