# Supplementary material for: Global, regional, and national disability-adjusted life years and prevalence of lymphatic filariasis from 1990 to 2021: A trend and health inequality analysis based on the global burden of disease study 2021
Source: PLoS Negl Trop Dis. 2025 Apr 29;19(4):e0013017. doi: 10.1371/journal.pntd.0013017 (PMC12040265; doi:10.1371/journal.pntd.0013017)
Supplement: S4 Table — Abbreviations: GBD, Global Burden of Disease, DALYs, disability-adjusted life years; SDI, socio-demographic index; EAPC, estimated annual percentage change; UI, uncertainty interval; CI, conﬁdence interval. (DOCX) [file pntd.0013017.s004.docx]

**S4 Table The crude prevalence and DALY rates (per 100,000) of lymphatic filariasis, and estimated annual percentage change in crude rates, by sex, SDI levels, GBD regions, among 67 countries and territories, from 1990 to 2021.**

| **Location** | **Crude prevalence (95% UI)** | | **EAPC (95% CI)** | **Crude DALY rates (95% UI)** | | **EAPC (95% CI)** |
| --- | --- | --- | --- | --- | --- | --- |
|  | **1990** | **2021** | **1990-2021** | **1990** | **2021** | **1990-2021** |
| Global | 3919.70 (3374.39 to 4665.41) | 721.07 (616.71 to 860.62) | -5.94 (-6.54 to -5.35) | 76.54 (51.54 to 107.10) | 16.66 (9.74 to 28.20) | -5.42 (-5.96 to -4.87) |
| **Sex** |  |  |  |  |  |  |
| Male | 4397.28 (3815.22 to 5132.09) | 791.41 (661.00 to 975.63) | -5.98 (-6.58 to -5.38) | 130.23 (86.54 to 185.12) | 25.06 (13.21 to 45.83) | -5.74 (-6.34 to -5.14) |
| Female | 3435.30 (2889.27 to 4189.16) | 650.25 (563.37 to 776.94) | -5.89 (-6.48 to -5.3) | 22.09 (14.92 to 31.94) | 8.20 (5.68 to 11.64) | -3.8 (-4.24 to -3.35) |
| **SDI region** |  |  |  |  |  |  |
| Low SDI | 11418.39 (9570.19 to 13583.49) | 1382.64 (1131.35 to 1747.06) | -7.88 (-8.84 to -6.91) | 222.27 (149.97 to 309.21) | 33.17 (19.67 to 55.52) | -7.27 (-8.15 to -6.37) |
| Low middle SDI | 8208.12 (7211.34 to 9518.93) | 1327.62 (1113.93 to 1613.23) | -5.99 (-6.59 to -5.39) | 157.24 (106.34 to 222.09) | 29.61 (17.40 to 49.89) | -5.5 (-6.07 to -4.91) |
| Middle SDI | 3100.58 (2178.01 to 4626.50) | 607.97 (483.07 to 868.84) | -5.66 (-6.04 to -5.27) | 62.63 (42.26 to 88.70) | 14.17 (8.14 to 24.42) | -5.2 (-5.59 to -4.8) |
| High middle SDI | 263.59 (124.92 to 586.58) | 75.98 (37.27 to 180.20) | -4.95 (-5.66 to -4.23) | 5.46 (3.52 to 8.06) | 2.05 (1.15 to 3.64) | -4.07 (-4.7 to -3.42) |
| High SDI | 0.00 (0.00 to 0.00) | 0.00 (0.00 to 0.00) | - | 0.00 (0.00 to 0.00) | 0.00 (0.00 to 0.00) | - |
| **GBD region** |  |  |  |  |  |  |
| Caribbean | 5840.56 (2131.40 to 13021.58) | 875.43 (476.61 to 1697.14) | -9.15 (-10.51 to -7.76) | 104.76 (71.09 to 146.03) | 21.33 (13.37 to 34.99) | -7.89 (-9.13 to -6.63) |
| Central Sub-Saharan Africa | 11650.98 (7008.88 to 18373.62) | 1657.99 (895.19 to 3149.71) | -7.8 (-8.89 to -6.69) | 230.25 (153.21 to 322.83) | 35.50 (19.97 to 61.95) | -7.69 (-8.81 to -6.55) |
| Eastern Sub-Saharan Africa | 10428.73 (7451.84 to 14277.51) | 606.65 (413.24 to 923.01) | -10.74 (-12.27 to -9.18) | 198.24 (134.77 to 278.86) | 23.36 (13.80 to 41.58) | -8.61 (-9.84 to -7.37) |
| High-income Asia Pacific | 2.35 (0.37 to 17.30) | 2.42 (0.56 to 13.18) | -1.73 (-2.93 to -0.53) | 0.05 (0.03 to 0.10) | 0.07 (0.04 to 0.14) | 0.45 (0.07 to 0.83) |
| North Africa and Middle East | 720.44 (199.66 to 2313.02) | 170.47 (49.02 to 536.65) | -7.89 (-8.99 to -6.77) | 13.50 (7.79 to 22.61) | 3.27 (1.88 to 5.92) | -8.3 (-9.49 to -7.09) |
| Oceania | 27668.33 (14356.99 to 44655.41) | 6807.93 (2589.99 to 16289.14) | -5.34 (-5.73 to -4.94) | 505.44 (349.78 to 716.90) | 143.43 (93.48 to 209.88) | -4.62 (-4.94 to -4.29) |
| South Asia | 9455.84 (8812.55 to 10169.09) | 1877.64 (1638.04 to 2187.25) | -5.07 (-5.55 to -4.58) | 183.47 (123.78 to 258.52) | 39.84 (23.20 to 67.14) | -4.73 (-5.22 to -4.24) |
| Southeast Asia | 9868.72 (5304.63 to 16897.15) | 1128.14 (557.07 to 2253.09) | -8.32 (-8.87 to -7.77) | 202.76 (135.48 to 287.00) | 28.49 (16.37 to 49.87) | -7.63 (-8.19 to -7.07) |
| Southern Sub-Saharan Africa | 274.43 (74.38 to 1002.35) | 152.59 (53.51 to 493.29) | -2.16 (-2.97 to -1.34) | 6.28 (3.60 to 10.96) | 5.20 (3.02 to 9.19) | -0.78 (-1.03 to -0.53) |
| Tropical Latin America | 169.64 (122.28 to 318.24) | 8.03 (6.78 to 14.01) | -10.48 (-11.39 to -9.55) | 4.44 (2.90 to 6.63) | 0.68 (0.47 to 0.97) | -6.85 (-7.29 to -6.41) |
| Western Sub-Saharan Africa | 13824.35 (9011.07 to 20511.20) | 1414.09 (907.09 to 2159.12) | -8.06 (-9.08 to -7.04) | 259.79 (175.41 to 363.05) | 35.80 (21.72 to 58.91) | -7.25 (-8.13 to -6.37) |
| **Country and territory** |  |  |  |  |  |  |
| American Samoa | 7804.78 (6707.80 to 9108.73) | 4225.39 (1779.91 to 9621.37) | -6.22 (-8.63 to -3.75) | 165.26 (106.13 to 240.66) | 74.59 (42.74 to 123.80) | -6.69 (-8.89 to -4.42) |
| Angola | 6565.24 (1781.23 to 19571.74) | 936.70 (303.79 to 2858.13) | -8.02 (-9.06 to -6.97) | 131.74 (84.53 to 195.13) | 27.58 (16.00 to 48.68) | -6.68 (-7.54 to -5.8) |
| Bangladesh | 3704.86 (3221.21 to 4234.27) | 324.03 (269.74 to 441.39) | -9.71 (-10.75 to -8.65) | 67.01 (38.52 to 109.88) | 23.62 (14.23 to 40.60) | -4.36 (-4.98 to -3.74) |
| Benin | 2260.12 (469.84 to 10170.62) | 666.47 (447.82 to 1031.28) | -5.27 (-6.06 to -4.46) | 40.77 (23.28 to 68.86) | 25.69 (15.00 to 45.53) | -2.11 (-2.48 to -1.75) |
| Brazil | 174.26 (125.61 to 326.90) | 8.29 (7.00 to 14.46) | -10.46 (-11.38 to -9.54) | 4.56 (2.98 to 6.81) | 0.70 (0.49 to 1.00) | -6.83 (-7.27 to -6.39) |
| Brunei Darussalam | 1572.04 (249.92 to 11572.70) | 994.70 (230.05 to 5418.38) | -3.21 (-4.38 to -2.02) | 36.10 (20.24 to 64.55) | 30.82 (17.12 to 55.59) | -1.06 (-1.41 to -0.7) |
| Burkina Faso | 17844.00 (5787.25 to 39035.38) | 552.58 (315.92 to 1150.23) | -13.3 (-15.68 to -10.85) | 317.92 (216.57 to 434.66) | 24.58 (14.49 to 43.38) | -10.48 (-12.42 to -8.5) |
| Cambodia | 2801.69 (413.35 to 17016.32) | 115.95 (1.96 to 753.02) | -13.19 (-15.37 to -10.95) | 47.88 (27.87 to 79.36) | 0.00 (0.00 to 0.00) | - |
| Cameroon | 3572.33 (1144.38 to 9447.95) | 278.09 (205.41 to 413.65) | -9.76 (-11.16 to -8.33) | 61.88 (35.67 to 102.19) | 22.11 (13.10 to 37.89) | -5.44 (-6.28 to -4.59) |
| Central African Republic | 19843.61 (7150.68 to 44834.33) | 3674.22 (938.25 to 11490.00) | -5.99 (-6.87 to -5.11) | 351.78 (240.18 to 479.78) | 63.06 (37.11 to 102.84) | -5.84 (-6.74 to -4.93) |
| Chad | 2982.87 (835.08 to 9186.59) | 623.04 (263.31 to 1843.68) | -5.47 (-6.67 to -4.25) | 51.20 (29.62 to 85.41) | 24.93 (14.57 to 44.16) | -2.87 (-3.47 to -2.27) |
| Comoros | 10080.52 (2023.21 to 46512.07) | 3565.58 (551.92 to 17260.44) | -3.96 (-4.62 to -3.3) | 213.28 (144.94 to 303.41) | 62.25 (35.45 to 104.05) | -4.6 (-5.28 to -3.91) |
| Congo | 4044.44 (970.39 to 14286.55) | 864.55 (305.80 to 2330.21) | -6.51 (-7.53 to -5.47) | 70.43 (41.32 to 115.38) | 28.08 (16.30 to 49.36) | -3.96 (-4.47 to -3.44) |
| Coted'Ivoire | 29805.08 (12377.55 to 57088.79) | 6335.94 (2402.46 to 14916.16) | -5.48 (-6.26 to -4.7) | 524.54 (363.90 to 737.88) | 127.98 (79.87 to 192.31) | -4.66 (-5.37 to -3.96) |
| Democratic Republic of the Congo | 13118.15 (7228.27 to 21537.23) | 1860.63 (900.98 to 3700.70) | -7.89 (-9.04 to -6.73) | 262.42 (174.56 to 365.55) | 37.29 (20.82 to 65.30) | -8.11 (-9.35 to -6.85) |
| Dominican Republic | 5885.44 (1391.59 to 22792.86) | 1346.32 (363.54 to 4786.78) | -9.72 (-12.55 to -6.8) | 111.57 (70.09 to 171.90) | 32.54 (18.80 to 57.69) | -7.89 (-9.94 to -5.79) |
| Egypt | 2791.47 (578.65 to 11646.23) | 532.42 (57.36 to 2311.69) | -9.94 (-11.63 to -8.22) | 50.60 (28.73 to 87.07) | 0.00 (0.00 to 0.00) | - |
| Equatorial Guinea | 11352.45 (2384.96 to 37352.42) | 1146.00 (299.56 to 4890.77) | -9.26 (-10.54 to -7.96) | 223.93 (149.53 to 312.09) | 31.39 (17.46 to 57.43) | -8.45 (-9.68 to -7.21) |
| Eritrea | 1165.20 (278.29 to 4766.08) | 392.11 (193.76 to 987.22) | -5.21 (-5.91 to -4.51) | 29.41 (16.68 to 51.83) | 24.53 (14.46 to 43.99) | -1.08 (-1.29 to -0.88) |
| Ethiopia | 4429.50 (1504.87 to 10485.11) | 540.14 (287.70 to 1126.73) | -8.91 (-9.86 to -7.95) | 81.47 (49.68 to 128.72) | 25.44 (14.97 to 45.45) | -5.03 (-5.66 to -4.4) |
| Fiji | 23136.03 (5974.62 to 59999.75) | 9376.85 (2633.62 to 25727.05) | -4.23 (-5.48 to -2.97) | 420.12 (288.00 to 574.79) | 201.10 (133.14 to 288.44) | -3.78 (-5.11 to -2.43) |
| Gabon | 3840.37 (863.27 to 15063.49) | 1296.97 (320.69 to 5180.68) | -5.53 (-6.67 to -4.38) | 67.05 (38.72 to 109.41) | 31.23 (17.96 to 55.37) | -3.8 (-4.51 to -3.09) |
| Ghana | 6580.53 (2012.18 to 17779.56) | 725.66 (297.81 to 1906.74) | -10.06 (-11.12 to -8.99) | 129.39 (83.18 to 192.78) | 26.57 (15.46 to 46.49) | -8.23 (-9.36 to -7.09) |
| Guinea | 5411.79 (1325.52 to 15483.72) | 627.48 (273.85 to 1661.77) | -6.97 (-7.6 to -6.35) | 100.45 (63.49 to 154.23) | 25.37 (14.67 to 44.88) | -5.13 (-5.67 to -4.6) |
| Guinea-Bissau | 14468.21 (2776.55 to 49819.46) | 1036.29 (267.90 to 4350.76) | -7.09 (-8.68 to -5.47) | 279.07 (187.91 to 384.84) | 28.67 (16.51 to 51.06) | -6.5 (-8.07 to -4.91) |
| Guyana | 34892.21 (19024.82 to 56357.43) | 16034.87 (5889.11 to 32738.60) | -2.86 (-3.31 to -2.41) | 612.80 (421.40 to 869.41) | 324.76 (220.41 to 447.26) | -2.29 (-2.66 to -1.92) |
| Haiti | 20351.35 (4790.16 to 54301.59) | 1014.57 (534.33 to 2127.60) | -12.62 (-14.24 to -10.96) | 359.88 (244.65 to 487.61) | 28.87 (16.50 to 51.90) | -10.83 (-12.26 to -9.38) |
| India | 11373.19 (10575.88 to 12258.43) | 2359.92 (2050.24 to 2744.45) | -4.86 (-5.34 to -4.38) | 220.86 (150.22 to 310.76) | 48.32 (28.05 to 81.12) | -4.63 (-5.12 to -4.14) |
| Indonesia | 12271.98 (4991.34 to 28457.41) | 1528.08 (626.19 to 3942.83) | -7.5 (-7.89 to -7.11) | 256.42 (170.91 to 358.98) | 36.81 (20.95 to 64.43) | -7.02 (-7.41 to -6.63) |
| Kenya | 10939.57 (4275.96 to 25782.84) | 959.62 (377.85 to 2717.23) | -9.8 (-11.66 to -7.89) | 210.38 (141.11 to 295.62) | 29.75 (17.13 to 52.98) | -8.19 (-9.77 to -6.58) |
| Kiribati | 34003.71 (5890.76 to 60935.17) | 1348.66 (286.30 to 4566.33) | -11.86 (-12.69 to -11.01) | 601.52 (417.47 to 851.34) | 31.93 (18.03 to 56.48) | -10.9 (-11.72 to -10.08) |
| Lao People's Democratic Republic | 3105.26 (571.22 to 13559.26) | 492.67 (211.77 to 1681.56) | -7.2 (-8.28 to -6.1) | 54.13 (31.41 to 90.51) | 25.73 (14.88 to 45.96) | -2.84 (-3.39 to -2.3) |
| Liberia | 20884.18 (5732.84 to 50751.29) | 9911.38 (7744.11 to 12641.08) | -5.25 (-6.81 to -3.66) | 369.11 (251.83 to 501.14) | 214.34 (144.77 to 304.18) | -4.67 (-6.34 to -2.97) |
| Madagascar | 12275.61 (5163.45 to 24286.38) | 904.61 (378.15 to 2132.25) | -11.56 (-13.09 to -9.99) | 255.24 (170.37 to 357.00) | 28.03 (15.91 to 49.84) | -9.97 (-11.3 to -8.61) |
| Malawi | 17636.95 (5366.10 to 43824.72) | 396.38 (279.85 to 617.09) | -14.19 (-16.13 to -12.2) | 327.02 (222.42 to 445.59) | 24.34 (14.05 to 42.69) | -10.95 (-12.58 to -9.3) |
| Malaysia | 4122.27 (857.72 to 15671.92) | 1064.35 (328.74 to 3894.03) | -5.2 (-5.86 to -4.53) | 73.69 (42.89 to 122.22) | 30.82 (17.06 to 54.79) | -3.24 (-3.73 to -2.75) |
| Maldives | 3052.67 (2701.14 to 3391.24) | 424.00 (184.89 to 1019.02) | -10.13 (-13.88 to -6.21) | 55.43 (32.17 to 91.70) | 28.11 (15.43 to 52.07) | -4.64 (-7.51 to -1.68) |
| Mali | 27962.41 (12909.52 to 49579.67) | 1215.26 (387.83 to 3817.88) | -12.63 (-14.68 to -10.54) | 479.48 (330.88 to 676.39) | 29.90 (17.13 to 52.79) | -11.03 (-12.7 to -9.33) |
| Marshall Islands | 827.13 (245.37 to 1488.04) | 151.55 (141.14 to 163.85) | -7.42 (-8.54 to -6.28) | 27.64 (16.07 to 48.52) | 14.87 (9.97 to 21.39) | -2.82 (-3.29 to -2.34) |
| Micronesia (Federated States of) | 12211.20 (4232.12 to 29317.58) | 2215.24 (382.03 to 15099.15) | -4.54 (-7.13 to -1.88) | 262.41 (176.79 to 366.80) | 42.54 (23.46 to 75.30) | -4.27 (-6.12 to -2.38) |
| Mozambique | 38345.44 (24044.17 to 54555.84) | 821.61 (366.95 to 1888.91) | -12.28 (-14.18 to -10.34) | 722.47 (492.91 to 1032.77) | 26.64 (15.36 to 46.06) | -11.28 (-13.02 to -9.51) |
| Myanmar | 14964.15 (3603.73 to 42598.49) | 1640.78 (370.40 to 6326.98) | -9.31 (-10.77 to -7.82) | 305.76 (205.31 to 422.29) | 34.78 (19.98 to 61.40) | -9.17 (-10.5 to -7.83) |
| Nepal | 11956.91 (10509.67 to 13664.29) | 2433.25 (1886.30 to 3293.93) | -6.09 (-6.72 to -5.46) | 250.93 (168.61 to 349.51) | 42.97 (24.48 to 74.08) | -6.95 (-7.72 to -6.17) |
| Niger | 17248.08 (6132.93 to 34468.38) | 1152.09 (575.84 to 2764.95) | -10.53 (-12 to -9.04) | 319.47 (217.28 to 441.83) | 29.33 (16.61 to 52.33) | -9.71 (-11.12 to -8.27) |
| Nigeria | 14716.93 (6545.40 to 26824.38) | 1230.78 (530.12 to 2443.02) | -8.13 (-9.4 to -6.84) | 291.34 (197.68 to 400.38) | 30.34 (17.61 to 52.49) | -7.71 (-8.84 to -6.57) |
| Niue | 5225.82 (1618.86 to 14071.54) | 1568.46 (447.75 to 5088.50) | -4.35 (-5.56 to -3.13) | 95.06 (56.94 to 152.26) | 34.67 (19.20 to 61.78) | -3.47 (-4.29 to -2.63) |
| Palau | 186.74 (164.85 to 220.40) | 172.87 (157.54 to 196.67) | -0.17 (-0.19 to -0.15) | 15.72 (10.53 to 22.04) | 14.83 (10.06 to 21.18) | -0.09 (-0.13 to -0.05) |
| Papua New Guinea | 34465.40 (15570.32 to 59923.84) | 7568.47 (2490.68 to 18997.45) | -5.67 (-6.03 to -5.31) | 627.47 (433.70 to 888.71) | 159.32 (103.70 to 233.70) | -4.91 (-5.25 to -4.58) |
| Philippines | 9000.50 (2320.04 to 25684.82) | 861.19 (505.20 to 1888.80) | -9.53 (-10.42 to -8.62) | 186.09 (124.40 to 264.13) | 29.53 (17.22 to 52.44) | -7.84 (-8.81 to -6.85) |
| Samoa | 15819.06 (5005.71 to 40973.54) | 6080.76 (1425.76 to 22674.43) | -2.1 (-3.29 to -0.9) | 329.51 (220.87 to 456.26) | 119.42 (74.24 to 184.07) | -2.37 (-3.73 to -1) |
| Sao Tome and Principe | 6064.01 (1026.93 to 32243.38) | 2741.59 (433.66 to 14873.53) | -3.16 (-3.83 to -2.49) | 117.23 (72.98 to 177.94) | 49.78 (27.57 to 85.52) | -3.23 (-3.84 to -2.62) |
| Senegal | 5482.75 (1324.75 to 19201.28) | 541.22 (230.92 to 1744.17) | -7.86 (-8.71 to -7) | 102.76 (63.92 to 157.40) | 25.37 (14.79 to 45.24) | -5.4 (-5.94 to -4.87) |
| Sierra Leone | 26964.57 (6440.27 to 61101.93) | 3594.48 (805.55 to 11625.99) | -7.9 (-8.91 to -6.87) | 456.40 (314.62 to 639.67) | 63.08 (36.46 to 105.21) | -7.64 (-8.63 to -6.63) |
| South Sudan | 3624.37 (1075.21 to 10485.16) | 932.05 (365.50 to 2267.47) | -5.69 (-6.68 to -4.69) | 66.43 (38.00 to 109.49) | 27.78 (16.07 to 49.14) | -3.74 (-4.4 to -3.07) |
| Sri Lanka | 1053.19 (818.05 to 1354.21) | 144.76 (105.76 to 196.83) | -7.83 (-8.55 to -7.11) | 30.02 (17.05 to 53.33) | 0.00 (0.00 to 0.00) | - |
| Sudan | 3987.43 (779.77 to 15615.37) | 859.65 (251.34 to 3116.19) | -5.72 (-6.19 to -5.25) | 71.16 (41.96 to 116.86) | 28.12 (16.10 to 50.57) | -3.63 (-3.94 to -3.31) |
| Thailand | 5765.77 (1270.89 to 22932.19) | 522.44 (27.94 to 2608.14) | -9.9 (-11.07 to -8.72) | 107.17 (64.95 to 168.41) | 0.00 (0.00 to 0.00) | - |
| Timor-Leste | 33695.86 (6266.19 to 72362.20) | 9012.24 (1681.95 to 41575.47) | -7.59 (-9.27 to -5.88) | 608.71 (419.43 to 866.48) | 192.48 (126.69 to 276.55) | -7.15 (-8.98 to -5.28) |
| Togo | 3575.15 (692.99 to 14277.30) | 169.95 (20.27 to 721.52) | -11.28 (-12.65 to -9.9) | 61.77 (36.09 to 101.31) | 0.00 (0.00 to 0.00) | - |
| Tonga | 2248.77 (304.87 to 18597.78) | 423.66 (185.51 to 1633.68) | -6.86 (-8.88 to -4.8) | 42.59 (23.97 to 73.89) | 24.78 (14.57 to 43.89) | -2.18 (-3.11 to -1.23) |
| Uganda | 6327.14 (1628.05 to 17311.51) | 280.55 (191.59 to 526.91) | -11.65 (-13.6 to -9.66) | 122.73 (78.24 to 183.30) | 21.70 (13.13 to 36.91) | -7.25 (-8.31 to -6.17) |
| United Republic of Tanzania | 16184.09 (8784.06 to 26314.02) | 532.81 (344.59 to 877.39) | -12.48 (-14.48 to -10.44) | 303.10 (207.68 to 415.09) | 25.01 (14.42 to 44.20) | -10.05 (-11.65 to -8.42) |
| Vanuatu | 23533.64 (3611.74 to 74432.19) | 2411.55 (309.47 to 18221.93) | -11.71 (-15.74 to -7.48) | 424.80 (292.14 to 586.27) | 44.95 (24.92 to 77.23) | -10.26 (-12.73 to -7.71) |
| Viet Nam | 9645.40 (2398.74 to 28565.59) | 798.19 (265.34 to 3121.66) | -9.8 (-10.8 to -8.79) | 199.70 (132.63 to 284.36) | 28.33 (16.17 to 50.62) | -7.52 (-8.4 to -6.63) |
| Yemen | 728.94 (236.87 to 2616.38) | 372.72 (200.71 to 1016.00) | -2.95 (-3.81 to -2.07) | 25.82 (14.82 to 46.65) | 24.30 (14.04 to 42.91) | -0.43 (-0.7 to -0.16) |
| Zambia | 15449.06 (7167.43 to 30472.41) | 1781.38 (620.25 to 5162.82) | -8.04 (-9.15 to -6.92) | 298.97 (203.71 to 411.23) | 36.18 (20.43 to 63.27) | -8.15 (-9.33 to -6.96) |
| Zimbabwe | 1390.83 (376.95 to 5080.02) | 785.72 (275.54 to 2540.04) | -2.05 (-2.93 to -1.15) | 31.83 (18.23 to 55.54) | 26.80 (15.53 to 47.33) | -0.66 (-0.99 to -0.34) |
| **Abbreviations:** GBD, Global Burden of Disease, DALYs, disability-adjusted life years; SDI, socio-demographic index; EAPC, estimated annual percentage change; UI, uncertainty interval; CI, conﬁdence interval. | | | | | | |
