# Supplementary material for: Global, regional, and national disability-adjusted life years and prevalence of lymphatic filariasis from 1990 to 2021: A trend and health inequality analysis based on the global burden of disease study 2021
Source: PLoS Negl Trop Dis. 2025 Apr 29;19(4):e0013017. doi: 10.1371/journal.pntd.0013017 (PMC12040265; doi:10.1371/journal.pntd.0013017)
Supplement: S5 Table — Abbreviations: GBD, Global Burden of Disease, DALYs, disability-adjusted life years; SDI, socio-demographic index; EAPC, estimated annual percentage change; UI, uncertainty interval; CI, conﬁdence interval. (DOCX) [file pntd.0013017.s005.docx]

**S5 Table The number of cases and DALYs of lymphatic filariasis, by sex, SDI levels, GBD regions, among 67 countries and territories, from 1990 to 2021.**

| **Location** | **Number of cases (95% UI)** | | **Number of DALYs (95% UI)** | |
| --- | --- | --- | --- | --- |
|  | **1990** | **2021** | **1990** | **2021** |
| Global | 209062177.57 (179977279.31 to 248835158.93) | 56902555.80 (48666870.20 to 67914348.40) | 4082608.43 (2749036.51 to 5712220.36) | 1314563.40 (768842.08 to 2224976.28) |
| **Sex** |  |  |  |  |
| Male | 118099634.98 (102466964.16 to 137834694.00) | 31334866.19 (26171553.14 to 38628818.00) | 3497767.07 (2324269.31 to 4971806.09) | 992097.62 (523140.26 to 1814609.47) |
| Female | 90962542.59 (76504400.63 to 110924038.62) | 25567689.60 (22151538.92 to 30548894.35) | 584841.36 (394972.64 to 845634.16) | 322465.78 (223466.21 to 457799.29) |
| **SDI region** |  |  |  |  |
| Low SDI | 57240439.56 (47975437.30 to 68094105.94) | 15449342.10 (12641468.05 to 19521387.37) | 1114257.24 (751803.49 to 1550082.15) | 370596.33 (219787.94 to 620395.47) |
| Low middle SDI | 95329998.83 (83753231.43 to 110553787.17) | 25505048.31 (21399819.03 to 30991947.03) | 1826159.93 (1234997.47 to 2579403.04) | 568754.94 (334301.70 to 958431.67) |
| Middle SDI | 53420026.88 (37525032.70 to 79710350.65) | 14886385.90 (11828205.01 to 21273817.28) | 1079045.69 (728110.07 to 1528288.00) | 346851.97 (199262.03 to 597948.71) |
| High middle SDI | 2803405.76 (1328524.86 to 6238454.43) | 990839.30 (486070.51 to 2349853.23) | 58104.31 (37489.35 to 85685.86) | 26735.44 (15037.23 to 47453.00) |
| High SDI | 0.00 (0.00 to 0.00) | 0.00 (0.00 to 0.00) | 0.00 (0.00 to 0.00) | 0.00 (0.00 to 0.00) |
| **GBD region** |  |  |  |  |
| Caribbean | 2061281.56 (752225.50 to 4595643.41) | 415467.85 (226191.90 to 805439.38) | 36970.97 (25090.49 to 51535.92) | 10123.51 (6343.74 to 16605.73) |
| Central Sub-Saharan Africa | 6403695.86 (3852268.72 to 10098640.12) | 2270255.54 (1225773.20 to 4312847.14) | 126549.41 (84208.85 to 177435.30) | 48605.70 (27345.28 to 84824.71) |
| Eastern Sub-Saharan Africa | 19900610.11 (14219971.03 to 27245037.07) | 2584938.22 (1760794.63 to 3932929.19) | 378285.61 (257183.42 to 532143.60) | 99553.21 (58781.66 to 177185.46) |
| High-income Asia Pacific | 4075.64 (647.94 to 30003.07) | 4487.44 (1037.83 to 24444.26) | 93.58 (52.47 to 167.34) | 139.03 (77.25 to 250.78) |
| North Africa and Middle East | 2443672.45 (677223.76 to 7845587.28) | 1062017.81 (305385.04 to 3343336.06) | 45791.97 (26437.47 to 76677.42) | 20401.76 (11735.10 to 36878.21) |
| Oceania | 1812261.23 (940375.07 to 2924906.23) | 948179.16 (360722.39 to 2268683.24) | 33106.12 (22910.72 to 46956.41) | 19976.45 (13019.26 to 29230.84) |
| South Asia | 103391426.77 (96357563.41 to 111190113.59) | 34671952.04 (30247621.23 to 40389153.81) | 2006046.43 (1353461.22 to 2826707.33) | 735707.66 (428341.87 to 1239749.27) |
| Southeast Asia | 45939562.48 (24693428.25 to 78657418.66) | 7877839.92 (3890013.43 to 15733426.53) | 943883.71 (630656.20 to 1335992.27) | 198952.38 (114300.47 to 348217.11) |
| Southern Sub-Saharan Africa | 143850.46 (38987.22 to 525413.91) | 122536.29 (42972.12 to 396129.13) | 3291.93 (1885.94 to 5744.04) | 4179.06 (2422.47 to 7381.27) |
| Tropical Latin America | 258791.09 (186546.62 to 485474.48) | 18260.04 (15418.28 to 31865.92) | 6775.16 (4429.43 to 10118.69) | 1547.64 (1069.24 to 2214.15) |
| Western Sub-Saharan Africa | 26702949.93 (17405665.61 to 39619176.20) | 6926621.48 (4443188.42 to 10575998.57) | 501813.54 (338827.49 to 701256.46) | 175377.00 (106381.05 to 288534.89) |
| **Country and territory** |  |  |  |  |
| American Samoa | 3785.56 (3253.50 to 4418.02) | 2102.50 (885.66 to 4787.46) | 80.16 (51.48 to 116.73) | 37.12 (21.26 to 61.60) |
| Angola | 674661.11 (183043.53 to 2011244.08) | 306362.83 (99358.82 to 934794.08) | 13537.52 (8686.57 to 20051.63) | 9020.41 (5231.65 to 15922.45) |
| Bangladesh | 4042788.65 (3515024.15 to 4620488.54) | 533469.34 (444089.74 to 726687.88) | 73124.18 (42029.60 to 119904.50) | 38892.80 (23425.00 to 66838.47) |
| Benin | 109630.02 (22790.05 to 493340.05) | 89967.33 (60452.60 to 139214.44) | 1977.46 (1129.43 to 3340.19) | 3468.17 (2024.40 to 6145.84) |
| Brazil | 258791.09 (186546.62 to 485474.48) | 18260.04 (15418.28 to 31865.92) | 6775.16 (4429.43 to 10118.69) | 1547.64 (1069.24 to 2214.15) |
| Brunei Darussalam | 4075.64 (647.94 to 30003.07) | 4487.44 (1037.83 to 24444.26) | 93.58 (52.47 to 167.34) | 139.03 (77.25 to 250.78) |
| Burkina Faso | 1700196.80 (551415.33 to 3719335.18) | 125773.81 (71906.43 to 261805.62) | 30291.40 (20635.02 to 41414.71) | 5594.44 (3298.90 to 9874.84) |
| Cambodia | 287812.74 (42463.15 to 1748059.22) | 19762.73 (333.98 to 128346.00) | 4918.40 (2862.60 to 8152.27) | 0.00 (0.00 to 0.00) |
| Cameroon | 372840.48 (119437.88 to 986071.80) | 88375.56 (65277.70 to 131455.94) | 6458.52 (3723.10 to 10665.04) | 7027.49 (4164.12 to 12041.30) |
| Central African Republic | 541857.65 (195259.36 to 1224264.40) | 201498.99 (51455.02 to 630126.86) | 9605.88 (6558.48 to 13101.07) | 3458.53 (2035.34 to 5639.63) |
| Chad | 179761.79 (50325.82 to 553626.73) | 110584.80 (46735.53 to 327241.18) | 3085.49 (1785.18 to 5146.90) | 4425.26 (2585.97 to 7838.41) |
| Comoros | 46629.30 (9358.74 to 215150.12) | 26541.25 (4108.34 to 128482.25) | 986.58 (670.44 to 1403.48) | 463.40 (263.90 to 774.56) |
| Congo | 97138.52 (23306.69 to 343131.35) | 46613.01 (16487.77 to 125635.86) | 1691.54 (992.33 to 2771.08) | 1514.05 (878.92 to 2661.53) |
| Coted'Ivoire | 3635663.00 (1509829.62 to 6963766.60) | 1765311.47 (669371.68 to 4155921.57) | 63984.09 (44388.42 to 90008.11) | 35657.17 (22253.15 to 53582.05) |
| Democratic Republic of the Congo | 5004268.67 (2757418.76 to 8215952.85) | 1674895.51 (811046.14 to 3331286.04) | 100108.23 (66591.75 to 139448.82) | 33570.70 (18745.50 to 58786.03) |
| Dominican Republic | 420859.65 (99510.48 to 1629886.56) | 148268.35 (40036.05 to 527163.00) | 7977.99 (5011.70 to 12292.24) | 3583.47 (2070.08 to 6352.86) |
| Egypt | 1544611.46 (320188.44 to 6444249.56) | 562382.64 (60586.33 to 2441781.53) | 27999.47 (15895.61 to 48179.94) | 0.00 (0.00 to 0.00) |
| Equatorial Guinea | 47998.51 (10083.70 to 157927.19) | 17331.13 (4530.23 to 73963.74) | 946.78 (632.22 to 1319.55) | 474.76 (264.11 to 868.54) |
| Eritrea | 39686.02 (9478.38 to 162329.56) | 25871.19 (12784.15 to 65136.02) | 1001.80 (568.18 to 1765.19) | 1618.15 (953.76 to 2902.41) |
| Ethiopia | 2239940.93 (760994.11 to 5302181.34) | 588418.06 (313411.26 to 1227441.49) | 41199.26 (25121.19 to 65090.83) | 27710.35 (16306.51 to 49508.90) |
| Fiji | 175458.31 (45310.12 to 455024.13) | 86686.57 (24347.10 to 237839.88) | 3186.09 (2184.16 to 4359.09) | 1859.09 (1230.84 to 2666.57) |
| Gabon | 37771.40 (8490.61 to 148154.90) | 23554.07 (5824.02 to 94085.45) | 659.46 (380.87 to 1076.09) | 567.25 (326.15 to 1005.59) |
| Ghana | 985204.96 (301254.39 to 2661870.72) | 248508.33 (101986.25 to 652980.56) | 19371.58 (12452.88 to 28862.30) | 9100.57 (5293.11 to 15920.33) |
| Guinea | 324384.78 (79452.00 to 928099.87) | 84268.43 (36777.07 to 223172.01) | 6020.98 (3805.51 to 9244.39) | 3406.87 (1970.24 to 6027.51) |
| Guinea-Bissau | 145710.51 (27962.85 to 501735.83) | 21388.11 (5529.23 to 89795.87) | 2810.54 (1892.50 to 3875.75) | 591.79 (340.73 to 1053.86) |
| Guyana | 272041.97 (148329.66 to 439398.58) | 122625.03 (45036.38 to 250365.18) | 4777.75 (3285.49 to 6778.50) | 2483.55 (1685.57 to 3420.34) |
| Haiti | 1298582.44 (305651.22 to 3464885.42) | 130514.58 (68735.92 to 273695.50) | 22963.35 (15610.49 to 31113.56) | 3713.90 (2122.09 to 6676.81) |
| India | 97020728.98 (90219170.28 to 104572425.83) | 33381015.62 (29000588.96 to 38820064.12) | 1884068.31 (1281509.23 to 2650988.05) | 683439.47 (396786.39 to 1147401.75) |
| Indonesia | 22701116.88 (9233152.54 to 52641457.56) | 4262034.78 (1746529.23 to 10997099.11) | 474327.24 (316147.90 to 664059.83) | 102671.46 (58428.00 to 179691.06) |
| Kenya | 2532201.95 (989765.18 to 5968000.80) | 480406.79 (189160.01 to 1360311.63) | 48695.92 (32663.50 to 68428.22) | 14894.56 (8577.88 to 26524.88) |
| Kiribati | 25300.96 (4383.11 to 45339.71) | 1634.05 (346.88 to 5532.61) | 447.57 (310.62 to 633.45) | 38.69 (21.85 to 68.43) |
| Lao People's Democratic Republic | 129473.76 (23816.92 to 565352.55) | 36346.77 (15623.35 to 124057.62) | 2256.86 (1309.77 to 3773.96) | 1898.15 (1097.54 to 3390.57) |
| Liberia | 513879.34 (141063.09 to 1248793.90) | 541109.76 (422788.03 to 690137.41) | 9082.26 (6196.68 to 12331.17) | 11701.90 (7903.84 to 16606.91) |
| Madagascar | 1460867.45 (614479.36 to 2890216.96) | 258332.61 (107989.67 to 608915.29) | 30375.20 (20274.71 to 42484.77) | 8005.61 (4543.17 to 14232.85) |
| Malawi | 1729426.52 (526183.69 to 4297321.84) | 77086.64 (54425.41 to 120010.56) | 32066.68 (21810.22 to 43693.28) | 4734.35 (2731.55 to 8302.95) |
| Malaysia | 728277.24 (151532.58 to 2768744.56) | 338593.19 (104579.72 to 1238778.34) | 13018.01 (7576.50 to 21592.50) | 9803.72 (5426.87 to 17429.54) |
| Maldives | 6784.80 (6003.50 to 7537.31) | 2192.99 (956.28 to 5270.47) | 123.19 (71.50 to 203.82) | 145.38 (79.82 to 269.29) |
| Mali | 2422336.61 (1118330.26 to 4295004.07) | 292928.23 (93484.15 to 920268.39) | 41536.56 (28663.76 to 58594.19) | 7207.19 (4130.08 to 12724.75) |
| Marshall Islands | 375.75 (111.47 to 675.98) | 85.28 (79.42 to 92.20) | 12.56 (7.30 to 22.04) | 8.37 (5.61 to 12.04) |
| Micronesia (Federated States of) | 12636.31 (4379.45 to 30338.24) | 2272.34 (391.88 to 15488.37) | 271.54 (182.94 to 379.57) | 43.64 (24.07 to 77.25) |
| Mozambique | 5122931.65 (3212289.79 to 7288633.50) | 255297.54 (114022.47 to 586933.42) | 96522.24 (65852.71 to 137977.47) | 8277.43 (4773.20 to 14312.62) |
| Myanmar | 6051271.12 (1457291.88 to 17226171.38) | 925720.02 (208976.70 to 3569661.10) | 123644.64 (83022.30 to 170766.45) | 19624.82 (11270.05 to 34640.99) |
| Nepal | 2327909.14 (2046144.01 to 2660321.62) | 757467.08 (587203.12 to 1025395.23) | 48853.94 (32826.72 to 68047.61) | 13375.39 (7620.03 to 23061.16) |
| Niger | 1385406.54 (492611.27 to 2768581.18) | 288462.40 (144180.30 to 692292.41) | 25660.77 (17452.15 to 35488.95) | 7344.69 (4158.91 to 13101.24) |
| Nigeria | 13251382.12 (5893592.33 to 24153152.12) | 2845130.42 (1225447.40 to 5647414.06) | 262331.54 (177995.06 to 360507.35) | 70124.42 (40713.20 to 121337.84) |
| Niue | 120.58 (37.35 to 324.69) | 26.22 (7.48 to 85.06) | 2.19 (1.31 to 3.51) | 0.58 (0.32 to 1.03) |
| Palau | 28.37 (25.04 to 33.48) | 31.28 (28.51 to 35.59) | 2.39 (1.60 to 3.35) | 2.68 (1.82 to 3.83) |
| Papua New Guinea | 1414171.69 (638875.83 to 2458772.89) | 791781.33 (260563.85 to 1987431.94) | 25746.22 (17795.60 to 36465.11) | 16667.24 (10848.96 to 24448.51) |
| Philippines | 5671075.73 (1461824.76 to 16183610.25) | 975293.32 (572137.63 to 2139062.42) | 117251.28 (78383.65 to 166425.16) | 33438.27 (19497.71 to 59389.08) |
| Samoa | 26723.36 (8456.22 to 69217.18) | 12990.93 (3045.99 to 48441.62) | 556.64 (373.12 to 770.76) | 255.12 (158.61 to 393.24) |
| Sao Tome and Principe | 7354.50 (1245.47 to 39105.12) | 5938.77 (939.39 to 32218.75) | 142.18 (88.52 to 215.81) | 107.84 (59.72 to 185.25) |
| Senegal | 418464.44 (101110.19 to 1465514.26) | 85834.92 (36622.44 to 276618.11) | 7842.97 (4878.70 to 12013.18) | 4023.73 (2345.05 to 7174.42) |
| Sierra Leone | 1119478.41 (267378.53 to 2536746.61) | 318732.22 (71430.47 to 1030907.46) | 18948.07 (13062.03 to 26556.82) | 5593.43 (3232.58 to 9329.68) |
| South Sudan | 213019.33 (63194.31 to 616255.64) | 90152.63 (35352.62 to 219321.93) | 3904.40 (2233.60 to 6435.26) | 2687.19 (1554.38 to 4753.50) |
| Sri Lanka | 180417.93 (140136.18 to 231983.57) | 32237.27 (23552.27 to 43834.05) | 5141.81 (2921.48 to 9136.01) | 0.00 (0.00 to 0.00) |
| Sudan | 798337.58 (156120.76 to 3126405.87) | 373250.13 (109130.73 to 1353018.75) | 14247.37 (8400.29 to 23396.15) | 12207.84 (6990.18 to 21955.21) |
| Thailand | 3272919.45 (721414.53 to 13017369.95) | 348379.09 (18629.34 to 1739192.98) | 60835.92 (36869.34 to 95596.39) | 0.00 (0.00 to 0.00) |
| Timor-Leste | 263212.74 (48947.88 to 565252.13) | 125967.10 (23509.21 to 581114.23) | 4754.93 (3276.33 to 6768.41) | 2690.36 (1770.81 to 3865.49) |
| Togo | 130374.35 (25271.08 to 520648.37) | 14226.11 (1696.86 to 60398.23) | 2252.56 (1315.95 to 3694.56) | 26.34 (15.49 to 46.66) |
| Tonga | 2222.94 (301.37 to 18384.14) | 450.35 (197.20 to 1736.60) | 42.10 (23.70 to 73.04) | 0.00 (0.00 to 0.00) |
| Uganda | 1094020.50 (281504.82 to 2993319.46) | 121521.71 (82987.11 to 228229.26) | 21221.93 (13527.89 to 31694.16) | 9398.70 (5689.24 to 15987.14) |
| United Republic of Tanzania | 4181556.30 (2269575.73 to 6798872.21) | 311417.34 (201407.31 to 512814.46) | 78313.39 (53659.79 to 107248.86) | 14616.29 (8426.79 to 25832.86) |
| Vanuatu | 35824.59 (5498.05 to 113306.00) | 7547.72 (968.58 to 57031.34) | 646.66 (444.71 to 892.46) | 140.70 (78.01 to 241.73) |
| Viet Nam | 6580744.26 (1636583.33 to 19489376.64) | 800324.73 (266047.95 to 3130025.29) | 136246.01 (90485.81 to 194008.39) | 28402.73 (16213.32 to 50754.35) |
| Yemen | 99386.62 (32295.71 to 356728.86) | 125394.49 (67526.97 to 341816.72) | 3520.09 (2020.85 to 6359.90) | 8174.90 (4724.26 to 14434.77) |
| Zambia | 1226107.84 (568840.16 to 2418430.14) | 347642.05 (121043.02 to 1007538.05) | 23727.85 (16167.59 to 32636.84) | 7060.52 (3986.00 to 12347.35) |
| Zimbabwe | 143850.46 (38987.22 to 525413.91) | 122536.29 (42972.12 to 396129.13) | 3291.93 (1885.94 to 5744.04) | 4179.06 (2422.47 to 7381.27) |
| **Abbreviations:** GBD, Global Burden of Disease, DALYs, disability-adjusted life years; SDI, socio-demographic index; EAPC, estimated annual percentage change; UI, uncertainty interval; CI, conﬁdence interval. | | | | |
