# Supplementary material for: Global, regional, and national disability-adjusted life years and prevalence of lymphatic filariasis from 1990 to 2021: A trend and health inequality analysis based on the global burden of disease study 2021
Source: PLoS Negl Trop Dis. 2025 Apr 29;19(4):e0013017. doi: 10.1371/journal.pntd.0013017 (PMC12040265; doi:10.1371/journal.pntd.0013017)
Supplement: S6 Table — Abbreviations: GBD, Global Burden of Disease, DALYs, disability-adjusted life years; SDI, socio-demographic index; UI, uncertainty interval. (DOCX) [file pntd.0013017.s006.docx]

**S6 Table The crude prevalence and DALY rates (per 100,000) of lymphatic filariasis, by age group , sex, SDI levels, GBD regions, among 67 countries and territories, in 1990.**

| **Location** | **Sex** | **Metric** | **Age group (95% UI)** | | | | | | | | | | | | | | | | | | | |
| --- | --- | --- | --- | --- | --- | --- | --- | --- | --- | --- | --- | --- | --- | --- | --- | --- | --- | --- | --- | --- | --- | --- |
|  |  |  | **<5 years** | **5-9 years** | **10-14 years** | **15-19 years** | **20-24 years** | **25-29 years** | **30-34 years** | **35-39 years** | **40-44 years** | **45-49 years** | **50-54 years** | **55-59 years** | **60-64 years** | **65-69 years** | **70-74 years** | **75-79 years** | **80-84 years** | **85-89 years** | **90-94 years** | **95+ years** |
| Global | Both | DALY rates | 5.70 (3.92 to 8.05) | 50.40 (32.27 to 76.81) | 66.71 (44.04 to 97.50) | 78.43 (52.20 to 112.46) | 86.96 (58.03 to 123.29) | 94.02 (63.10 to 132.22) | 100.31 (67.35 to 141.09) | 100.85 (68.00 to 141.73) | 104.22 (70.68 to 146.46) | 112.11 (76.25 to 157.13) | 106.16 (72.43 to 146.87) | 105.60 (71.73 to 147.14) | 101.53 (69.75 to 141.23) | 92.37 (63.67 to 127.02) | 84.69 (58.78 to 115.62) | 67.39 (47.12 to 91.56) | 61.90 (43.27 to 84.09) | 51.95 (36.70 to 69.57) | 42.33 (30.00 to 56.50) | 34.09 (24.36 to 45.45) |
| Global | Both | Prevalence | 868.28 (742.97 to 1025.07) | 2401.65 (2053.39 to 2810.39) | 3241.85 (2773.43 to 3833.79) | 3876.66 (3318.03 to 4598.83) | 4386.45 (3755.88 to 5230.87) | 4785.11 (4085.24 to 5737.13) | 5080.91 (4342.58 to 6105.15) | 5086.08 (4382.68 to 6036.82) | 5251.02 (4557.77 to 6231.42) | 5650.64 (4903.78 to 6721.00) | 5361.35 (4630.33 to 6420.43) | 5357.18 (4620.07 to 6395.41) | 5172.25 (4462.70 to 6141.87) | 4844.21 (4164.59 to 5771.24) | 4602.82 (3944.52 to 5482.16) | 3790.44 (3238.03 to 4494.39) | 3601.15 (3079.75 to 4299.73) | 3142.47 (2626.89 to 3904.26) | 2752.24 (2243.95 to 3556.19) | 2508.32 (1959.40 to 3376.34) |
| Global | Female | DALY rates | 6.73 (4.61 to 9.61) | 12.86 (8.83 to 18.39) | 14.94 (10.11 to 21.52) | 17.36 (11.69 to 25.12) | 21.02 (14.19 to 30.64) | 24.76 (16.64 to 35.83) | 28.00 (18.76 to 40.38) | 29.85 (20.10 to 43.41) | 32.38 (21.56 to 47.38) | 35.82 (23.96 to 52.16) | 34.83 (23.14 to 50.25) | 35.53 (23.47 to 51.33) | 34.59 (23.16 to 49.66) | 31.89 (21.61 to 45.53) | 29.29 (19.54 to 41.66) | 22.71 (15.23 to 32.07) | 20.99 (14.27 to 29.46) | 16.83 (11.53 to 23.49) | 13.90 (9.63 to 19.37) | 12.42 (8.63 to 17.09) |
| Global | Female | Prevalence | 881.23 (754.08 to 1040.17) | 2094.66 (1782.64 to 2499.65) | 2812.69 (2371.04 to 3398.42) | 3377.52 (2830.15 to 4113.37) | 3880.22 (3244.71 to 4754.50) | 4276.79 (3562.27 to 5277.40) | 4527.98 (3783.13 to 5566.51) | 4494.02 (3797.91 to 5443.28) | 4609.52 (3924.55 to 5570.61) | 4918.15 (4183.41 to 6028.11) | 4632.32 (3914.79 to 5735.57) | 4577.91 (3874.12 to 5634.77) | 4300.86 (3634.10 to 5284.40) | 3980.73 (3345.89 to 4912.20) | 3771.45 (3155.71 to 4651.37) | 2984.94 (2490.73 to 3669.04) | 2757.55 (2290.04 to 3424.58) | 2349.58 (1895.36 to 3080.64) | 2086.91 (1616.90 to 2829.50) | 2031.01 (1469.03 to 2981.47) |
| Global | Male | DALY rates | 4.74 (3.20 to 6.73) | 85.95 (53.55 to 134.90) | 116.05 (75.47 to 172.81) | 137.56 (91.23 to 197.60) | 151.90 (100.04 to 216.31) | 162.54 (108.30 to 230.12) | 170.70 (113.13 to 239.70) | 169.74 (113.30 to 238.45) | 173.10 (115.61 to 242.47) | 185.44 (124.66 to 257.98) | 175.68 (119.51 to 244.41) | 175.23 (118.79 to 243.14) | 171.46 (117.29 to 236.81) | 162.30 (111.41 to 222.98) | 153.97 (106.01 to 210.04) | 131.70 (91.56 to 178.97) | 129.94 (90.56 to 176.00) | 121.62 (85.38 to 163.27) | 110.68 (78.13 to 148.18) | 97.18 (68.78 to 129.69) |
| Global | Male | Prevalence | 856.10 (732.37 to 1010.66) | 2692.37 (2289.40 to 3137.42) | 3650.96 (3109.87 to 4268.94) | 4360.02 (3761.82 to 5092.48) | 4884.89 (4229.29 to 5713.91) | 5287.90 (4566.05 to 6199.95) | 5619.11 (4849.63 to 6613.70) | 5660.47 (4933.95 to 6633.90) | 5866.06 (5172.71 to 6829.36) | 6354.70 (5605.19 to 7358.12) | 6071.84 (5345.37 to 7071.32) | 6131.61 (5401.02 to 7138.02) | 6082.65 (5350.46 to 7052.28) | 5842.44 (5128.82 to 6778.86) | 5642.46 (4946.26 to 6532.89) | 4949.96 (4341.97 to 5680.41) | 5004.19 (4379.15 to 5755.68) | 4715.54 (4084.50 to 5610.76) | 4351.55 (3733.20 to 5271.50) | 3898.33 (3342.55 to 4679.92) |
| **SDI region** |  |  |  |  |  |  |  |  |  |  |  |  |  |  |  |  |  |  |  |  |  |  |
| Low SDI | Both | DALY rates | 10.87 (7.31 to 15.41) | 117.47 (75.28 to 177.84) | 175.37 (116.80 to 251.21) | 238.25 (159.57 to 336.32) | 284.61 (188.96 to 394.34) | 317.99 (214.74 to 441.14) | 344.93 (232.89 to 479.27) | 363.43 (246.33 to 507.45) | 381.96 (258.41 to 530.91) | 393.32 (269.06 to 545.21) | 406.93 (278.21 to 562.20) | 419.01 (287.63 to 575.04) | 432.87 (300.07 to 596.95) | 425.39 (296.22 to 583.45) | 407.34 (283.25 to 555.55) | 387.80 (273.15 to 527.34) | 371.87 (263.70 to 502.07) | 353.75 (251.94 to 471.39) | 335.96 (240.39 to 450.73) | 294.20 (211.98 to 394.37) |
| Low SDI | Both | Prevalence | 1942.00 (1569.73 to 2375.48) | 5754.32 (4796.06 to 7006.09) | 8343.24 (6959.81 to 10119.25) | 11414.65 (9518.52 to 13734.32) | 14095.15 (11748.46 to 16886.53) | 16165.56 (13467.60 to 19284.91) | 17597.04 (14752.79 to 20883.75) | 18416.12 (15485.69 to 21762.21) | 19423.83 (16345.56 to 22952.09) | 20066.65 (16891.95 to 23772.69) | 20819.84 (17597.15 to 24605.51) | 21542.23 (18280.18 to 25317.76) | 22370.52 (19045.04 to 26179.49) | 22625.73 (19293.84 to 26368.31) | 22283.17 (18987.91 to 26065.17) | 22013.88 (18706.34 to 25830.13) | 21913.07 (18688.14 to 25526.83) | 21870.86 (18639.01 to 25669.61) | 22265.27 (18750.91 to 26449.81) | 21747.57 (18191.70 to 26058.09) |
| Low SDI | Female | DALY rates | 12.78 (8.62 to 18.29) | 26.62 (18.13 to 38.12) | 34.16 (22.97 to 49.11) | 47.13 (31.96 to 67.71) | 63.57 (42.76 to 92.18) | 79.50 (53.36 to 114.78) | 93.72 (63.24 to 134.82) | 103.22 (68.86 to 151.93) | 116.21 (77.59 to 170.23) | 123.79 (83.13 to 179.00) | 133.61 (88.48 to 192.85) | 140.62 (93.82 to 204.38) | 153.20 (102.03 to 221.24) | 157.54 (105.86 to 226.41) | 153.09 (101.46 to 215.85) | 147.26 (99.56 to 208.27) | 143.41 (97.29 to 200.23) | 144.75 (98.72 to 203.27) | 147.37 (101.64 to 205.00) | 146.11 (100.98 to 201.50) |
| Low SDI | Female | Prevalence | 1968.58 (1592.99 to 2407.10) | 5038.04 (4088.09 to 6134.19) | 7263.56 (5869.63 to 8896.14) | 10008.18 (8069.06 to 12294.48) | 12467.80 (10038.31 to 15267.61) | 14311.47 (11582.24 to 17448.18) | 15577.32 (12681.39 to 18846.35) | 16250.56 (13384.69 to 19530.25) | 17243.76 (14255.89 to 20613.93) | 17806.67 (14686.35 to 21538.94) | 18495.18 (15313.66 to 22191.46) | 19137.66 (15907.93 to 22768.03) | 19999.14 (16666.07 to 23726.47) | 20471.58 (17072.52 to 24222.56) | 20351.56 (17014.29 to 24107.99) | 20140.83 (16776.84 to 23973.90) | 20074.76 (16758.80 to 23772.08) | 20310.88 (16858.75 to 24271.52) | 20916.60 (17208.15 to 25359.23) | 21008.07 (17143.54 to 25703.24) |
| Low SDI | Male | DALY rates | 9.04 (6.06 to 12.98) | 205.16 (127.48 to 318.42) | 310.67 (203.50 to 450.44) | 426.34 (284.76 to 602.11) | 514.32 (340.18 to 719.14) | 573.46 (382.63 to 793.83) | 608.67 (411.91 to 838.70) | 629.00 (424.27 to 870.73) | 645.12 (438.40 to 884.42) | 656.44 (445.72 to 900.83) | 669.37 (454.18 to 914.84) | 679.42 (467.79 to 917.19) | 688.17 (476.04 to 933.10) | 676.85 (468.43 to 910.14) | 650.77 (453.27 to 876.17) | 630.47 (443.14 to 838.50) | 620.47 (435.03 to 824.79) | 601.74 (428.14 to 790.23) | 597.66 (425.85 to 783.41) | 556.17 (399.86 to 727.00) |
| Low SDI | Male | Prevalence | 1916.42 (1547.33 to 2339.81) | 6445.65 (5485.06 to 7648.15) | 9377.70 (8005.18 to 11115.52) | 12798.81 (10956.93 to 15148.64) | 15786.36 (13434.82 to 18698.32) | 18151.63 (15419.29 to 21423.13) | 19717.50 (16841.86 to 23144.18) | 20626.29 (17755.83 to 24027.01) | 21582.72 (18518.91 to 25102.23) | 22272.83 (19112.16 to 25954.20) | 23051.94 (19806.11 to 26888.04) | 23791.46 (20519.70 to 27668.99) | 24535.28 (21194.81 to 28408.23) | 24648.13 (21367.69 to 28426.65) | 24132.65 (20875.46 to 27757.57) | 23903.53 (20663.34 to 27583.04) | 23913.48 (20739.34 to 27441.71) | 23721.79 (20703.52 to 27234.53) | 24136.93 (20872.94 to 27735.61) | 23055.77 (19963.68 to 26672.80) |
| Low-middle SDI | Both | DALY rates | 9.52 (6.46 to 13.32) | 86.44 (56.54 to 129.51) | 114.66 (75.79 to 166.94) | 148.17 (99.00 to 211.98) | 175.98 (118.01 to 250.44) | 195.51 (131.76 to 276.72) | 216.66 (147.52 to 304.06) | 239.68 (163.10 to 336.31) | 253.57 (172.90 to 353.98) | 267.00 (182.53 to 371.84) | 280.24 (192.39 to 389.76) | 299.20 (204.62 to 418.89) | 315.08 (215.93 to 437.82) | 304.73 (210.03 to 421.23) | 279.95 (194.28 to 385.04) | 269.86 (187.71 to 368.37) | 273.19 (192.13 to 371.77) | 238.22 (168.81 to 319.10) | 202.91 (144.28 to 270.98) | 165.52 (118.33 to 219.60) |
| Low-middle SDI | Both | Prevalence | 1387.03 (1214.30 to 1633.98) | 3967.10 (3460.86 to 4597.11) | 5589.04 (4876.83 to 6527.36) | 7526.04 (6545.34 to 8816.67) | 9184.51 (8020.22 to 10736.06) | 10310.00 (8997.23 to 12099.95) | 11389.89 (9954.98 to 13370.01) | 12537.04 (11049.49 to 14528.41) | 13183.62 (11681.67 to 15227.07) | 13818.88 (12243.36 to 16057.33) | 14444.33 (12767.91 to 16990.29) | 15386.40 (13630.95 to 18117.05) | 16136.10 (14313.62 to 18816.32) | 15979.41 (14047.62 to 18722.61) | 15208.67 (13321.91 to 17831.98) | 15080.78 (13303.68 to 17465.33) | 15711.29 (13950.87 to 18002.44) | 14153.37 (12349.44 to 16731.98) | 12579.69 (10806.26 to 15277.22) | 10889.80 (9313.78 to 13258.14) |
| Low-middle SDI | Female | DALY rates | 11.17 (7.59 to 15.80) | 21.92 (14.88 to 31.21) | 26.83 (18.22 to 38.58) | 35.17 (23.51 to 51.28) | 46.45 (31.01 to 67.94) | 57.46 (39.14 to 83.20) | 68.79 (45.77 to 99.47) | 82.83 (54.82 to 120.97) | 91.57 (60.48 to 134.01) | 100.09 (66.49 to 145.69) | 108.99 (72.14 to 156.89) | 120.32 (78.73 to 172.81) | 130.31 (86.71 to 184.05) | 131.58 (88.53 to 188.37) | 123.59 (81.65 to 175.78) | 122.05 (81.50 to 173.78) | 130.04 (87.41 to 181.26) | 113.51 (77.81 to 158.91) | 95.54 (65.63 to 132.46) | 78.88 (54.34 to 108.72) |
| Low-middle SDI | Female | Prevalence | 1393.55 (1220.90 to 1641.29) | 3415.30 (2975.01 to 4036.81) | 4813.18 (4136.70 to 5730.21) | 6464.77 (5528.63 to 7738.13) | 7986.72 (6825.98 to 9565.69) | 9135.71 (7798.59 to 10960.71) | 10137.69 (8701.70 to 12118.62) | 11184.68 (9720.30 to 13159.57) | 11692.22 (10181.81 to 13759.06) | 12195.78 (10585.26 to 14492.79) | 12737.00 (11022.73 to 15396.99) | 13492.26 (11674.64 to 16313.58) | 14058.49 (12147.09 to 16911.62) | 14179.92 (12183.23 to 17063.91) | 13749.04 (11752.72 to 16534.81) | 13690.02 (11776.86 to 16191.32) | 14307.29 (12439.81 to 16832.42) | 13095.23 (11184.74 to 15839.20) | 11745.77 (9783.68 to 14688.47) | 10149.16 (8305.65 to 12761.06) |
| Low-middle SDI | Male | DALY rates | 7.96 (5.38 to 11.50) | 147.26 (94.99 to 224.38) | 198.25 (129.60 to 291.14) | 258.53 (171.10 to 375.85) | 305.58 (202.28 to 436.52) | 333.99 (223.48 to 474.43) | 362.12 (240.45 to 515.66) | 391.76 (262.77 to 556.94) | 408.52 (275.59 to 574.72) | 425.06 (286.75 to 593.73) | 441.46 (300.82 to 611.52) | 467.66 (320.77 to 652.68) | 487.50 (335.36 to 675.82) | 472.53 (328.18 to 648.70) | 437.00 (305.71 to 596.59) | 419.70 (294.25 to 569.87) | 424.14 (301.23 to 575.84) | 374.92 (265.61 to 505.99) | 330.09 (233.88 to 442.88) | 284.94 (203.51 to 381.51) |
| Low-middle SDI | Male | Prevalence | 1380.87 (1212.37 to 1625.09) | 4487.31 (3921.18 to 5207.25) | 6327.35 (5524.47 to 7346.84) | 8562.47 (7534.24 to 9895.47) | 10382.96 (9188.25 to 11987.85) | 11487.90 (10134.66 to 13296.43) | 12621.69 (11119.64 to 14654.45) | 13848.28 (12275.31 to 15922.22) | 14610.16 (13018.96 to 16729.30) | 15355.82 (13714.37 to 17634.01) | 16051.65 (14291.90 to 18534.65) | 17170.07 (15334.58 to 19805.84) | 18074.82 (16160.07 to 20699.78) | 17723.30 (15787.89 to 20386.16) | 16674.83 (14822.43 to 19093.22) | 16490.65 (14768.07 to 18718.98) | 17191.83 (15467.79 to 19418.55) | 15313.26 (13632.14 to 17730.77) | 13567.48 (11925.39 to 15989.14) | 11910.68 (10492.00 to 14015.95) |
| Middle SDI | Both | DALY rates | 4.15 (2.82 to 5.91) | 32.66 (20.09 to 51.77) | 46.14 (29.35 to 70.28) | 55.88 (36.63 to 82.06) | 65.16 (43.69 to 92.99) | 79.12 (52.92 to 112.65) | 92.45 (61.54 to 131.20) | 88.34 (59.15 to 124.16) | 94.35 (63.22 to 132.47) | 100.68 (68.14 to 140.35) | 98.01 (66.30 to 135.45) | 95.82 (64.80 to 132.55) | 95.42 (64.95 to 131.82) | 87.70 (60.23 to 119.73) | 79.00 (54.29 to 107.04) | 75.27 (52.52 to 101.52) | 78.59 (54.82 to 105.50) | 80.62 (56.61 to 108.06) | 76.63 (54.19 to 102.72) | 67.71 (48.02 to 89.40) |
| Middle SDI | Both | Prevalence | 570.32 (384.76 to 884.42) | 1613.96 (1120.70 to 2381.14) | 2284.28 (1592.11 to 3396.65) | 2729.50 (1912.63 to 4091.30) | 3174.87 (2229.60 to 4735.94) | 3833.60 (2699.99 to 5708.78) | 4421.05 (3117.20 to 6613.17) | 4202.67 (2998.89 to 6258.45) | 4493.72 (3254.12 to 6624.35) | 4816.49 (3463.48 to 7201.34) | 4732.45 (3329.62 to 7174.58) | 4693.27 (3261.46 to 7120.95) | 4754.51 (3275.37 to 7278.46) | 4541.31 (3082.09 to 7041.27) | 4274.17 (2866.37 to 6642.03) | 4254.81 (2815.72 to 6464.56) | 4634.61 (3029.99 to 7153.59) | 4964.36 (3103.68 to 7898.45) | 5322.10 (3134.63 to 9297.41) | 5894.84 (3040.97 to 11392.27) |
| Middle SDI | Female | DALY rates | 4.91 (3.33 to 7.06) | 9.51 (6.47 to 13.60) | 11.03 (7.39 to 15.94) | 12.13 (8.14 to 17.18) | 14.21 (9.57 to 20.40) | 17.68 (11.93 to 25.97) | 20.91 (14.04 to 29.92) | 20.54 (13.76 to 29.43) | 22.90 (15.43 to 32.94) | 24.81 (16.63 to 35.96) | 24.57 (16.50 to 35.27) | 24.99 (16.61 to 35.84) | 25.70 (17.29 to 37.06) | 24.03 (16.25 to 34.53) | 21.70 (14.85 to 31.10) | 20.50 (14.11 to 29.06) | 21.15 (14.74 to 29.73) | 20.52 (14.38 to 28.71) | 22.01 (15.74 to 30.30) | 24.89 (17.36 to 33.90) |
| Middle SDI | Female | Prevalence | 580.62 (392.87 to 897.36) | 1425.22 (955.35 to 2230.22) | 1993.83 (1319.71 to 3146.61) | 2378.21 (1552.78 to 3787.57) | 2778.67 (1802.75 to 4417.67) | 3351.43 (2173.37 to 5340.31) | 3839.63 (2488.94 to 6115.57) | 3612.11 (2380.46 to 5718.61) | 3847.26 (2570.45 to 6076.70) | 4122.02 (2698.78 to 6650.93) | 4092.87 (2621.17 to 6731.26) | 4091.02 (2592.79 to 6720.06) | 4153.53 (2619.46 to 6856.96) | 3992.97 (2490.28 to 6532.48) | 3749.31 (2308.86 to 6092.14) | 3687.49 (2237.44 to 6020.61) | 3958.90 (2344.58 to 6599.77) | 4212.57 (2341.41 to 7444.40) | 4907.34 (2439.26 to 9537.42) | 6052.66 (2623.57 to 13088.07) |
| Middle SDI | Male | DALY rates | 3.45 (2.32 to 4.90) | 54.52 (31.83 to 89.55) | 79.68 (49.24 to 123.88) | 98.28 (63.93 to 145.65) | 115.17 (75.87 to 166.05) | 139.61 (92.19 to 199.90) | 161.15 (106.72 to 228.58) | 153.12 (102.21 to 216.22) | 160.97 (107.52 to 227.62) | 171.16 (115.48 to 240.52) | 166.49 (112.95 to 232.54) | 163.16 (110.18 to 227.87) | 163.81 (110.45 to 225.91) | 155.14 (105.04 to 213.01) | 145.32 (99.16 to 199.42) | 145.37 (99.19 to 198.41) | 160.49 (110.40 to 219.14) | 174.42 (122.41 to 235.90) | 167.65 (117.60 to 225.17) | 152.13 (107.84 to 203.30) |
| Middle SDI | Male | Prevalence | 560.75 (377.55 to 872.39) | 1792.20 (1275.88 to 2559.29) | 2561.71 (1833.00 to 3666.09) | 3069.97 (2228.53 to 4377.81) | 3563.85 (2609.63 to 5063.22) | 4308.45 (3172.41 to 6129.25) | 4979.37 (3681.87 to 7115.17) | 4766.85 (3543.25 to 6785.87) | 5096.40 (3861.20 to 7151.89) | 5461.47 (4137.17 to 7702.50) | 5328.89 (3969.63 to 7595.87) | 5265.82 (3893.48 to 7526.25) | 5344.05 (3919.93 to 7702.19) | 5122.03 (3694.51 to 7505.44) | 4881.68 (3484.49 to 7167.64) | 4980.86 (3575.73 to 7220.61) | 5598.04 (4007.58 to 8182.94) | 6137.63 (4295.77 to 9211.32) | 6013.32 (4156.65 to 9216.50) | 5583.65 (3878.80 to 8466.92) |
| High-middle SDI | Both | DALY rates | 0.65 (0.44 to 0.93) | 3.47 (1.96 to 5.93) | 4.58 (2.65 to 7.49) | 5.46 (3.39 to 8.66) | 6.57 (4.21 to 9.92) | 7.61 (4.93 to 11.26) | 7.96 (5.15 to 11.47) | 7.37 (4.82 to 10.67) | 7.85 (5.20 to 11.28) | 7.86 (5.22 to 11.23) | 5.99 (3.99 to 8.46) | 5.11 (3.45 to 7.21) | 4.32 (2.89 to 6.10) | 3.63 (2.47 to 5.07) | 3.33 (2.28 to 4.72) | 2.38 (1.63 to 3.28) | 2.47 (1.67 to 3.50) | 2.45 (1.67 to 3.47) | 3.15 (2.15 to 4.53) | 4.92 (3.18 to 7.20) |
| High-middle SDI | Both | Prevalence | 67.67 (29.23 to 162.03) | 179.80 (83.11 to 407.92) | 237.11 (107.34 to 547.49) | 273.29 (124.14 to 625.29) | 318.03 (147.93 to 748.39) | 358.46 (170.72 to 826.13) | 365.89 (175.75 to 797.19) | 334.46 (161.91 to 732.81) | 354.66 (174.23 to 775.68) | 354.04 (174.91 to 761.48) | 275.49 (132.92 to 607.62) | 239.68 (119.06 to 512.86) | 206.36 (101.16 to 452.94) | 178.73 (86.15 to 395.45) | 172.42 (78.56 to 386.17) | 128.56 (58.41 to 298.19) | 138.63 (59.44 to 350.46) | 140.17 (56.46 to 376.74) | 201.28 (67.04 to 630.34) | 328.35 (93.14 to 1129.14) |
| High-middle SDI | Female | DALY rates | 0.77 (0.52 to 1.11) | 1.34 (0.86 to 1.93) | 1.36 (0.88 to 1.97) | 1.31 (0.87 to 1.86) | 1.34 (0.93 to 1.98) | 1.41 (0.95 to 1.99) | 1.36 (0.89 to 1.92) | 1.19 (0.77 to 1.71) | 1.22 (0.82 to 1.70) | 1.18 (0.81 to 1.71) | 0.90 (0.59 to 1.29) | 0.78 (0.52 to 1.13) | 0.64 (0.44 to 0.90) | 0.52 (0.36 to 0.73) | 0.49 (0.33 to 0.69) | 0.35 (0.24 to 0.49) | 0.36 (0.24 to 0.51) | 0.33 (0.23 to 0.46) | 0.51 (0.34 to 0.72) | 0.79 (0.52 to 1.11) |
| High-middle SDI | Female | Prevalence | 68.73 (30.10 to 164.68) | 160.42 (67.78 to 387.79) | 209.03 (84.07 to 513.16) | 239.26 (92.44 to 617.93) | 274.18 (104.70 to 719.41) | 302.16 (116.67 to 773.76) | 300.68 (115.24 to 736.58) | 267.46 (103.81 to 665.94) | 280.50 (111.46 to 692.48) | 274.02 (108.64 to 670.31) | 212.25 (81.42 to 531.64) | 186.94 (75.22 to 450.18) | 154.36 (61.04 to 381.71) | 128.48 (50.26 to 316.24) | 123.30 (46.03 to 315.37) | 89.05 (33.41 to 230.62) | 93.37 (32.86 to 264.62) | 86.79 (26.81 to 262.11) | 132.91 (30.42 to 467.16) | 211.13 (38.93 to 802.52) |
| High-middle SDI | Male | DALY rates | 0.54 (0.35 to 0.80) | 5.48 (2.78 to 9.97) | 7.64 (4.20 to 12.87) | 9.45 (5.68 to 15.43) | 11.65 (7.32 to 17.90) | 13.62 (8.68 to 20.45) | 14.30 (9.12 to 20.79) | 13.37 (8.71 to 19.52) | 14.28 (9.38 to 20.73) | 14.44 (9.43 to 20.80) | 11.18 (7.39 to 15.94) | 9.65 (6.47 to 13.77) | 8.57 (5.67 to 12.20) | 7.72 (5.19 to 10.89) | 7.45 (5.07 to 10.63) | 5.88 (3.99 to 8.18) | 6.67 (4.48 to 9.53) | 7.66 (5.14 to 11.00) | 10.75 (7.20 to 15.83) | 18.49 (11.70 to 27.93) |
| High-middle SDI | Male | Prevalence | 66.70 (28.45 to 159.58) | 198.06 (95.12 to 428.76) | 263.82 (129.69 to 575.14) | 306.06 (153.54 to 644.26) | 360.73 (187.24 to 771.33) | 413.04 (218.70 to 868.91) | 428.46 (227.66 to 871.13) | 399.44 (215.23 to 801.25) | 426.53 (233.88 to 852.58) | 432.86 (239.56 to 856.95) | 339.96 (186.30 to 680.35) | 295.07 (164.59 to 574.77) | 266.41 (146.10 to 538.09) | 245.00 (133.91 to 495.25) | 243.89 (128.36 to 500.47) | 196.91 (102.65 to 415.28) | 228.87 (116.63 to 519.70) | 271.16 (129.76 to 654.58) | 397.79 (170.35 to 1096.69) | 713.44 (267.05 to 2194.86) |
| High SDI | Both | DALY rates | 0.00 (0.00 to 0.00) | 0.00 (0.00 to 0.00) | 0.00 (0.00 to 0.00) | 0.00 (0.00 to 0.00) | 0.00 (0.00 to 0.00) | 0.00 (0.00 to 0.00) | 0.00 (0.00 to 0.00) | 0.00 (0.00 to 0.00) | 0.00 (0.00 to 0.00) | 0.00 (0.00 to 0.00) | 0.00 (0.00 to 0.00) | 0.00 (0.00 to 0.00) | 0.00 (0.00 to 0.00) | 0.00 (0.00 to 0.00) | 0.00 (0.00 to 0.00) | 0.00 (0.00 to 0.00) | 0.00 (0.00 to 0.00) | 0.00 (0.00 to 0.00) | 0.00 (0.00 to 0.00) | 0.00 (0.00 to 0.00) |
| High SDI | Both | Prevalence | 0.00 (0.00 to 0.00) | 0.00 (0.00 to 0.00) | 0.00 (0.00 to 0.00) | 0.00 (0.00 to 0.00) | 0.00 (0.00 to 0.00) | 0.00 (0.00 to 0.00) | 0.00 (0.00 to 0.00) | 0.00 (0.00 to 0.00) | 0.00 (0.00 to 0.00) | 0.00 (0.00 to 0.00) | 0.00 (0.00 to 0.00) | 0.00 (0.00 to 0.00) | 0.00 (0.00 to 0.00) | 0.00 (0.00 to 0.00) | 0.00 (0.00 to 0.00) | 0.00 (0.00 to 0.00) | 0.00 (0.00 to 0.00) | 0.00 (0.00 to 0.00) | 0.00 (0.00 to 0.00) | 0.00 (0.00 to 0.00) |
| High SDI | Female | DALY rates | 0.00 (0.00 to 0.00) | 0.00 (0.00 to 0.00) | 0.00 (0.00 to 0.00) | 0.00 (0.00 to 0.00) | 0.00 (0.00 to 0.00) | 0.00 (0.00 to 0.00) | 0.00 (0.00 to 0.00) | 0.00 (0.00 to 0.00) | 0.00 (0.00 to 0.00) | 0.00 (0.00 to 0.00) | 0.00 (0.00 to 0.00) | 0.00 (0.00 to 0.00) | 0.00 (0.00 to 0.00) | 0.00 (0.00 to 0.00) | 0.00 (0.00 to 0.00) | 0.00 (0.00 to 0.00) | 0.00 (0.00 to 0.00) | 0.00 (0.00 to 0.00) | 0.00 (0.00 to 0.00) | 0.00 (0.00 to 0.00) |
| High SDI | Female | Prevalence | 0.00 (0.00 to 0.00) | 0.00 (0.00 to 0.00) | 0.00 (0.00 to 0.00) | 0.00 (0.00 to 0.00) | 0.00 (0.00 to 0.00) | 0.00 (0.00 to 0.00) | 0.00 (0.00 to 0.00) | 0.00 (0.00 to 0.00) | 0.00 (0.00 to 0.00) | 0.00 (0.00 to 0.00) | 0.00 (0.00 to 0.00) | 0.00 (0.00 to 0.00) | 0.00 (0.00 to 0.00) | 0.00 (0.00 to 0.00) | 0.00 (0.00 to 0.00) | 0.00 (0.00 to 0.00) | 0.00 (0.00 to 0.00) | 0.00 (0.00 to 0.00) | 0.00 (0.00 to 0.00) | 0.00 (0.00 to 0.00) |
| High SDI | Male | DALY rates | 0.00 (0.00 to 0.00) | 0.00 (0.00 to 0.00) | 0.00 (0.00 to 0.00) | 0.00 (0.00 to 0.00) | 0.00 (0.00 to 0.00) | 0.00 (0.00 to 0.00) | 0.00 (0.00 to 0.00) | 0.00 (0.00 to 0.00) | 0.00 (0.00 to 0.00) | 0.00 (0.00 to 0.00) | 0.00 (0.00 to 0.00) | 0.00 (0.00 to 0.00) | 0.00 (0.00 to 0.00) | 0.00 (0.00 to 0.00) | 0.00 (0.00 to 0.00) | 0.00 (0.00 to 0.00) | 0.00 (0.00 to 0.00) | 0.00 (0.00 to 0.00) | 0.00 (0.00 to 0.00) | 0.00 (0.00 to 0.00) |
| High SDI | Male | Prevalence | 0.00 (0.00 to 0.00) | 0.00 (0.00 to 0.00) | 0.00 (0.00 to 0.00) | 0.00 (0.00 to 0.00) | 0.00 (0.00 to 0.00) | 0.00 (0.00 to 0.00) | 0.00 (0.00 to 0.00) | 0.00 (0.00 to 0.00) | 0.00 (0.00 to 0.00) | 0.00 (0.00 to 0.00) | 0.00 (0.00 to 0.00) | 0.00 (0.00 to 0.00) | 0.00 (0.00 to 0.00) | 0.00 (0.00 to 0.00) | 0.00 (0.00 to 0.00) | 0.00 (0.00 to 0.00) | 0.00 (0.00 to 0.00) | 0.00 (0.00 to 0.00) | 0.00 (0.00 to 0.00) | 0.00 (0.00 to 0.00) |
| **GBD region** |  |  |  |  |  |  |  |  |  |  |  |  |  |  |  |  |  |  |  |  |  |  |
| Caribbean | Both | DALY rates | 6.96 (4.49 to 9.97) | 75.85 (49.21 to 114.14) | 107.32 (72.09 to 150.96) | 112.19 (74.23 to 156.67) | 116.43 (78.88 to 161.42) | 119.45 (82.30 to 169.30) | 135.69 (91.60 to 187.28) | 143.46 (96.84 to 200.83) | 132.29 (90.57 to 183.25) | 134.78 (92.22 to 190.09) | 138.51 (94.77 to 191.50) | 143.97 (99.70 to 195.99) | 144.59 (100.53 to 199.68) | 132.43 (91.72 to 184.31) | 111.80 (77.82 to 150.84) | 93.41 (64.90 to 129.49) | 77.06 (53.28 to 103.54) | 63.00 (44.02 to 86.16) | 59.08 (41.55 to 78.99) | 73.56 (52.19 to 98.29) |
| Caribbean | Both | Prevalence | 1298.02 (352.33 to 3174.23) | 3594.62 (1310.82 to 8098.60) | 4954.87 (1888.26 to 10942.67) | 5588.01 (2118.31 to 12222.88) | 6350.89 (2364.03 to 13984.54) | 6832.88 (2508.34 to 15107.08) | 7887.80 (2859.90 to 17625.79) | 8357.08 (3000.29 to 18861.90) | 7641.43 (2771.19 to 17144.35) | 7774.39 (2803.97 to 17574.40) | 8042.99 (2880.99 to 18139.07) | 8356.74 (2975.54 to 18663.74) | 8397.29 (3016.50 to 18472.85) | 7841.70 (2831.73 to 17120.52) | 6761.99 (2433.13 to 14648.61) | 5691.08 (2099.09 to 12020.81) | 4752.04 (1781.30 to 10355.50) | 3986.18 (1556.21 to 8703.87) | 3920.80 (1600.67 to 8241.66) | 4970.71 (2010.51 to 11478.75) |
| Caribbean | Female | DALY rates | 8.24 (5.15 to 11.95) | 16.02 (10.35 to 23.70) | 19.83 (12.98 to 28.44) | 23.03 (14.91 to 33.96) | 28.83 (19.20 to 41.02) | 33.77 (22.31 to 49.34) | 41.17 (26.80 to 60.14) | 45.66 (31.23 to 67.21) | 43.13 (28.32 to 62.26) | 45.08 (29.82 to 66.42) | 48.39 (31.92 to 70.44) | 51.51 (34.58 to 74.06) | 54.75 (36.03 to 79.20) | 53.11 (34.89 to 75.09) | 45.12 (30.00 to 65.33) | 38.12 (25.02 to 53.75) | 32.49 (21.65 to 46.12) | 28.21 (18.98 to 39.65) | 28.34 (19.23 to 40.13) | 28.16 (19.39 to 40.03) |
| Caribbean | Female | Prevalence | 1324.82 (363.85 to 3227.04) | 3156.23 (837.58 to 7739.82) | 4326.39 (1187.84 to 10477.82) | 4984.99 (1404.15 to 11857.29) | 5807.66 (1670.27 to 13687.38) | 6332.90 (1824.55 to 14988.58) | 7278.21 (2086.70 to 17357.03) | 7634.15 (2202.16 to 18313.62) | 6905.25 (2014.87 to 16422.99) | 6951.37 (2038.46 to 16611.73) | 7250.08 (2113.15 to 17422.05) | 7451.97 (2172.77 to 17714.12) | 7500.06 (2232.41 to 17503.78) | 7094.96 (2134.78 to 16450.41) | 6180.72 (1842.46 to 14360.00) | 5240.96 (1611.80 to 11943.66) | 4481.61 (1406.84 to 9900.02) | 3802.76 (1259.16 to 8417.57) | 3791.93 (1311.27 to 8170.06) | 4330.43 (1332.37 to 10355.33) |
| Caribbean | Male | DALY rates | 5.71 (3.53 to 8.62) | 134.95 (85.37 to 207.28) | 194.72 (130.50 to 276.81) | 202.10 (133.93 to 280.88) | 207.09 (140.60 to 292.91) | 210.03 (142.74 to 297.90) | 237.00 (160.56 to 329.20) | 247.35 (166.63 to 346.57) | 225.30 (151.48 to 313.00) | 227.46 (155.94 to 319.18) | 231.66 (157.03 to 318.38) | 239.43 (165.23 to 326.56) | 237.20 (164.25 to 327.53) | 216.03 (150.59 to 296.84) | 184.30 (127.85 to 249.81) | 155.13 (107.42 to 211.39) | 128.15 (89.05 to 174.08) | 105.89 (73.98 to 143.91) | 99.95 (70.90 to 134.42) | 151.85 (107.26 to 204.51) |
| Caribbean | Male | Prevalence | 1272.03 (337.49 to 3123.03) | 4027.65 (1751.64 to 8484.17) | 5582.64 (2549.04 to 11488.15) | 6196.09 (2820.30 to 12664.10) | 6913.07 (3068.25 to 14274.00) | 7361.52 (3214.19 to 15292.49) | 8541.12 (3674.79 to 17936.08) | 9125.12 (3863.79 to 19444.38) | 8409.45 (3552.43 to 17896.92) | 8624.70 (3594.68 to 18451.71) | 8862.68 (3668.26 to 18875.20) | 9290.78 (3792.14 to 19702.95) | 9322.11 (3816.85 to 19377.94) | 8628.76 (3564.49 to 17692.02) | 7394.05 (3074.97 to 15002.12) | 6193.58 (2630.93 to 12372.75) | 5062.09 (2228.33 to 10547.06) | 4212.33 (1915.87 to 8929.79) | 4092.10 (1975.90 to 8356.96) | 6074.92 (3017.85 to 13059.02) |
| Central Sub-Saharan Africa | Both | DALY rates | 11.69 (7.48 to 17.08) | 110.77 (64.92 to 179.66) | 184.66 (119.93 to 278.09) | 274.31 (181.78 to 390.66) | 333.43 (220.48 to 462.50) | 360.06 (240.19 to 498.01) | 374.76 (251.65 to 512.48) | 384.12 (259.52 to 531.57) | 377.75 (258.82 to 513.57) | 379.46 (258.78 to 519.68) | 381.45 (261.88 to 518.18) | 386.50 (267.30 to 521.18) | 389.57 (268.10 to 525.52) | 389.51 (274.07 to 519.46) | 390.05 (273.74 to 523.11) | 390.31 (275.63 to 527.37) | 373.21 (263.67 to 497.15) | 349.09 (249.25 to 460.89) | 329.67 (236.87 to 432.52) | 281.08 (203.47 to 367.46) |
| Central Sub-Saharan Africa | Both | Prevalence | 2003.81 (1094.50 to 3281.40) | 5901.51 (3474.93 to 9351.06) | 8742.12 (5246.05 to 13754.22) | 12103.41 (7351.74 to 18881.31) | 14861.59 (9074.41 to 23230.63) | 16706.49 (10160.22 to 26124.51) | 17983.12 (10896.98 to 28099.47) | 18897.29 (11473.64 to 29493.04) | 19771.00 (11908.65 to 30938.31) | 20402.96 (12267.76 to 31976.04) | 21165.69 (12670.78 to 33346.86) | 22091.47 (13103.62 to 34996.65) | 22888.16 (13518.52 to 36350.70) | 23323.14 (13723.29 to 37037.27) | 23358.89 (13709.40 to 37043.43) | 23137.27 (13752.61 to 36730.80) | 22155.92 (13311.29 to 34801.53) | 22207.75 (13349.85 to 34749.88) | 22779.16 (13595.22 to 36022.53) | 22694.95 (13299.20 to 36181.44) |
| Central Sub-Saharan Africa | Female | DALY rates | 13.67 (8.49 to 20.28) | 28.01 (17.59 to 42.93) | 33.49 (21.35 to 48.19) | 41.62 (28.09 to 60.59) | 50.26 (31.94 to 72.71) | 57.48 (38.68 to 82.47) | 63.10 (40.90 to 89.55) | 67.43 (45.80 to 97.02) | 73.09 (48.57 to 104.17) | 77.03 (51.37 to 106.46) | 82.04 (55.25 to 117.12) | 87.48 (60.07 to 124.62) | 92.99 (62.56 to 134.34) | 94.12 (64.20 to 133.06) | 92.35 (61.38 to 132.77) | 89.15 (60.58 to 123.41) | 80.21 (55.75 to 110.34) | 81.39 (56.70 to 111.80) | 82.92 (58.25 to 113.84) | 82.86 (57.20 to 114.85) |
| Central Sub-Saharan Africa | Female | Prevalence | 2030.10 (1122.31 to 3314.41) | 5227.43 (2843.74 to 8610.77) | 7500.91 (4045.84 to 12422.72) | 10187.62 (5478.10 to 16940.77) | 12537.32 (6759.08 to 20854.04) | 14231.05 (7695.55 to 23604.68) | 15457.98 (8356.81 to 25599.13) | 16337.52 (8845.52 to 26990.95) | 17363.47 (9417.92 to 28700.32) | 18025.99 (9798.81 to 29805.65) | 18802.47 (10146.52 to 31237.62) | 19664.92 (10550.65 to 32699.34) | 20390.78 (10916.51 to 33923.65) | 20739.66 (11087.36 to 34548.75) | 20695.92 (11091.04 to 34626.33) | 20278.41 (11070.97 to 33442.86) | 18994.33 (10359.26 to 31303.24) | 19303.56 (10827.85 to 31577.16) | 20077.56 (11186.95 to 32851.15) | 20504.17 (11372.87 to 33603.04) |
| Central Sub-Saharan Africa | Male | DALY rates | 9.77 (6.09 to 14.93) | 192.98 (108.61 to 325.44) | 334.44 (212.85 to 512.38) | 504.02 (329.69 to 729.78) | 616.99 (409.83 to 855.81) | 670.55 (446.26 to 932.70) | 696.64 (467.09 to 958.34) | 710.14 (481.99 to 988.08) | 714.70 (490.06 to 985.86) | 717.50 (490.42 to 986.61) | 724.67 (499.58 to 989.97) | 733.47 (506.30 to 993.99) | 736.84 (501.55 to 999.87) | 732.90 (508.69 to 991.31) | 723.12 (508.28 to 979.89) | 702.83 (498.80 to 951.99) | 671.01 (475.91 to 901.14) | 662.96 (470.01 to 887.25) | 673.08 (484.91 to 884.44) | 664.09 (477.91 to 877.72) |
| Central Sub-Saharan Africa | Male | Prevalence | 1978.15 (1069.75 to 3249.26) | 6571.05 (4082.79 to 10154.38) | 9972.01 (6371.29 to 15039.69) | 13994.57 (9176.51 to 20836.81) | 17189.07 (11312.61 to 25537.17) | 19246.65 (12603.41 to 28581.07) | 20591.20 (13440.36 to 30553.31) | 21532.49 (14090.00 to 31937.45) | 22433.74 (14602.28 to 33274.74) | 23059.77 (15006.91 to 34249.53) | 23874.66 (15499.19 to 35610.98) | 24907.05 (16031.03 to 37526.64) | 25812.28 (16531.42 to 39048.06) | 26326.36 (16778.78 to 39967.82) | 26338.26 (16690.19 to 39864.83) | 26103.98 (16505.05 to 39544.61) | 25369.31 (16062.41 to 38394.52) | 25612.78 (16234.11 to 38532.82) | 26539.14 (16729.33 to 40311.32) | 26928.33 (16936.32 to 41163.82) |
| Eastern Sub-Saharan Africa | Both | DALY rates | 10.63 (7.16 to 15.18) | 110.19 (71.22 to 165.98) | 161.19 (107.48 to 233.53) | 215.46 (145.18 to 305.46) | 245.75 (164.35 to 343.59) | 271.75 (182.29 to 382.33) | 301.04 (204.68 to 420.39) | 324.35 (219.65 to 451.97) | 356.42 (240.78 to 501.15) | 373.95 (256.55 to 525.45) | 396.32 (270.67 to 559.62) | 416.61 (286.41 to 583.62) | 439.22 (303.20 to 614.35) | 449.14 (310.62 to 628.18) | 435.04 (303.54 to 598.82) | 426.90 (297.95 to 583.31) | 430.27 (303.06 to 585.04) | 424.80 (300.97 to 575.16) | 408.11 (291.65 to 553.47) | 352.78 (253.48 to 477.00) |
| Eastern Sub-Saharan Africa | Both | Prevalence | 1795.17 (1202.31 to 2582.30) | 5406.89 (3898.87 to 7453.68) | 7894.49 (5683.50 to 10849.31) | 10774.11 (7697.82 to 14814.04) | 12893.44 (9149.34 to 17787.44) | 14550.67 (10336.36 to 20091.28) | 15961.31 (11410.83 to 21837.57) | 17084.54 (12250.23 to 23307.54) | 18574.60 (13349.08 to 25053.33) | 19339.76 (13933.20 to 26116.20) | 20373.48 (14718.06 to 27439.54) | 21273.27 (15412.50 to 28675.05) | 22250.47 (16162.94 to 29964.23) | 22815.18 (16584.44 to 30882.43) | 22413.99 (16207.21 to 30558.53) | 22819.78 (16535.34 to 30925.40) | 23945.72 (17283.38 to 32227.58) | 24702.62 (17747.38 to 33213.63) | 25160.50 (18146.51 to 32981.30) | 24136.96 (17185.20 to 32669.24) |
| Eastern Sub-Saharan Africa | Female | DALY rates | 12.52 (8.32 to 17.81) | 26.45 (18.11 to 37.76) | 34.70 (23.38 to 49.62) | 48.91 (32.91 to 71.38) | 65.69 (44.02 to 94.68) | 83.14 (55.34 to 121.36) | 103.34 (68.98 to 149.09) | 122.97 (80.76 to 180.70) | 146.27 (96.77 to 213.88) | 157.38 (104.75 to 229.68) | 173.85 (114.30 to 252.27) | 184.84 (121.63 to 266.58) | 201.01 (132.33 to 289.47) | 211.71 (141.51 to 303.63) | 207.73 (137.59 to 296.91) | 201.96 (135.53 to 283.65) | 206.10 (139.11 to 290.01) | 208.03 (141.90 to 294.88) | 212.91 (144.78 to 296.47) | 199.50 (136.05 to 275.88) |
| Eastern Sub-Saharan Africa | Female | Prevalence | 1824.79 (1230.73 to 2612.88) | 4765.15 (3190.00 to 6780.40) | 6950.77 (4664.41 to 9908.97) | 9584.29 (6487.02 to 13577.07) | 11665.79 (7842.93 to 16645.03) | 13217.80 (8916.76 to 18794.51) | 14550.62 (9921.07 to 20384.64) | 15685.65 (10790.73 to 21974.86) | 17207.50 (11914.71 to 23838.27) | 17811.11 (12329.87 to 24644.72) | 18712.59 (13027.52 to 25775.08) | 19465.69 (13570.76 to 26823.44) | 20362.98 (14212.44 to 28137.35) | 21087.63 (14758.57 to 29128.57) | 21037.44 (14719.22 to 28967.30) | 21081.99 (14657.45 to 28993.54) | 21771.74 (15216.82 to 29899.62) | 22461.81 (15634.91 to 30937.97) | 23118.78 (16190.27 to 31114.48) | 22603.87 (15544.37 to 30675.83) |
| Eastern Sub-Saharan Africa | Male | DALY rates | 8.80 (5.77 to 12.59) | 191.50 (118.44 to 294.47) | 283.91 (186.76 to 414.59) | 386.28 (259.27 to 550.03) | 444.84 (294.70 to 625.82) | 484.43 (323.83 to 680.11) | 516.54 (349.27 to 724.10) | 540.32 (364.94 to 752.97) | 573.58 (387.50 to 804.53) | 595.40 (402.87 to 825.48) | 624.61 (423.42 to 869.74) | 649.28 (443.31 to 893.83) | 675.02 (464.72 to 930.18) | 676.38 (467.35 to 935.01) | 645.77 (446.11 to 874.22) | 654.53 (454.96 to 883.45) | 688.98 (479.31 to 928.84) | 701.88 (498.34 to 941.07) | 703.85 (501.08 to 933.21) | 669.24 (478.68 to 880.59) |
| Eastern Sub-Saharan Africa | Male | Prevalence | 1766.46 (1171.49 to 2550.67) | 6029.98 (4509.43 to 8103.94) | 8810.04 (6616.48 to 11756.10) | 11994.40 (8931.83 to 15886.60) | 14250.87 (10573.16 to 19123.78) | 16053.63 (11903.78 to 21563.18) | 17499.02 (13057.82 to 23471.14) | 18584.81 (13828.19 to 24896.51) | 19987.40 (14857.76 to 26404.16) | 20902.79 (15559.02 to 27582.23) | 22077.90 (16499.16 to 29056.30) | 23087.97 (17292.82 to 30434.54) | 24118.81 (18121.20 to 31762.68) | 24468.55 (18328.83 to 32356.66) | 23690.15 (17656.41 to 31588.58) | 24578.26 (18384.26 to 32951.67) | 26454.69 (19726.00 to 34885.11) | 27566.73 (20619.01 to 36351.43) | 28253.74 (21242.99 to 36538.52) | 27302.09 (20160.32 to 36096.41) |
| High-income Asia Pacific | Both | DALY rates | 0.03 (0.02 to 0.05) | 0.08 (0.04 to 0.15) | 0.07 (0.04 to 0.12) | 0.06 (0.03 to 0.10) | 0.07 (0.04 to 0.13) | 0.09 (0.05 to 0.16) | 0.09 (0.05 to 0.16) | 0.07 (0.04 to 0.13) | 0.05 (0.02 to 0.09) | 0.04 (0.02 to 0.07) | 0.03 (0.02 to 0.05) | 0.02 (0.01 to 0.04) | 0.02 (0.01 to 0.04) | 0.02 (0.01 to 0.04) | 0.02 (0.01 to 0.03) | 0.02 (0.01 to 0.03) | 0.02 (0.01 to 0.03) | 0.02 (0.01 to 0.04) | 0.00 (0.00 to 0.01) | 0.00 (0.00 to 0.00) |
| High-income Asia Pacific | Both | Prevalence | 1.09 (0.32 to 7.30) | 2.13 (0.54 to 13.56) | 2.23 (0.45 to 15.33) | 2.34 (0.40 to 16.91) | 3.19 (0.50 to 23.62) | 4.07 (0.60 to 30.45) | 4.18 (0.59 to 31.38) | 3.49 (0.48 to 26.30) | 2.31 (0.32 to 17.46) | 1.94 (0.26 to 14.69) | 1.56 (0.20 to 11.87) | 1.31 (0.17 to 10.08) | 1.15 (0.15 to 8.85) | 1.21 (0.15 to 9.30) | 1.09 (0.14 to 8.39) | 0.97 (0.12 to 7.50) | 1.12 (0.14 to 8.65) | 1.33 (0.17 to 10.25) | 0.47 (0.05 to 3.83) | 0.31 (0.03 to 2.59) |
| High-income Asia Pacific | Female | DALY rates | 0.04 (0.02 to 0.06) | 0.05 (0.03 to 0.08) | 0.04 (0.02 to 0.06) | 0.03 (0.02 to 0.05) | 0.04 (0.02 to 0.06) | 0.04 (0.02 to 0.06) | 0.04 (0.02 to 0.06) | 0.03 (0.02 to 0.04) | 0.02 (0.01 to 0.03) | 0.02 (0.01 to 0.02) | 0.01 (0.01 to 0.02) | 0.01 (0.01 to 0.02) | 0.01 (0.00 to 0.01) | 0.01 (0.00 to 0.01) | 0.01 (0.00 to 0.01) | 0.01 (0.00 to 0.01) | 0.01 (0.00 to 0.01) | 0.01 (0.00 to 0.01) | 0.00 (0.00 to 0.01) | 0.00 (0.00 to 0.00) |
| High-income Asia Pacific | Female | Prevalence | 1.14 (0.36 to 7.28) | 1.91 (0.47 to 13.35) | 2.02 (0.38 to 15.14) | 2.13 (0.32 to 16.66) | 2.88 (0.37 to 23.01) | 3.51 (0.41 to 28.36) | 3.52 (0.39 to 28.66) | 2.84 (0.30 to 23.25) | 1.83 (0.19 to 15.07) | 1.58 (0.15 to 12.98) | 1.29 (0.12 to 10.70) | 1.12 (0.10 to 9.31) | 0.97 (0.09 to 8.02) | 0.89 (0.08 to 7.43) | 0.79 (0.07 to 6.60) | 0.74 (0.07 to 6.14) | 0.83 (0.07 to 6.88) | 0.86 (0.08 to 7.14) | 0.57 (0.05 to 4.72) | 0.41 (0.04 to 3.39) |
| High-income Asia Pacific | Male | DALY rates | 0.03 (0.01 to 0.04) | 0.11 (0.05 to 0.21) | 0.09 (0.04 to 0.19) | 0.08 (0.04 to 0.17) | 0.11 (0.05 to 0.22) | 0.13 (0.06 to 0.27) | 0.14 (0.06 to 0.26) | 0.11 (0.05 to 0.23) | 0.07 (0.03 to 0.15) | 0.06 (0.03 to 0.12) | 0.05 (0.02 to 0.09) | 0.04 (0.02 to 0.08) | 0.03 (0.02 to 0.06) | 0.04 (0.02 to 0.08) | 0.04 (0.02 to 0.07) | 0.03 (0.02 to 0.06) | 0.04 (0.02 to 0.07) | 0.05 (0.03 to 0.10) | 0.00 (0.00 to 0.01) | 0.00 (0.00 to 0.00) |
| High-income Asia Pacific | Male | Prevalence | 1.05 (0.26 to 7.31) | 2.35 (0.59 to 13.81) | 2.42 (0.51 to 15.52) | 2.54 (0.47 to 17.15) | 3.49 (0.60 to 24.20) | 4.63 (0.76 to 32.50) | 4.81 (0.77 to 34.02) | 4.12 (0.66 to 29.27) | 2.78 (0.44 to 19.80) | 2.30 (0.36 to 16.41) | 1.82 (0.28 to 13.06) | 1.52 (0.23 to 10.88) | 1.36 (0.21 to 9.77) | 1.64 (0.25 to 11.78) | 1.53 (0.23 to 10.99) | 1.33 (0.20 to 9.61) | 1.63 (0.25 to 11.75) | 2.34 (0.35 to 16.82) | 0.21 (0.03 to 1.52) | 0.03 (0.00 to 0.22) |
| North Africa and Middle East | Both | DALY rates | 2.80 (1.86 to 4.06) | 9.35 (5.16 to 17.12) | 11.00 (6.03 to 19.68) | 12.81 (7.12 to 23.09) | 14.71 (8.04 to 25.19) | 16.42 (9.40 to 28.12) | 17.70 (10.13 to 29.69) | 19.45 (11.11 to 32.73) | 21.49 (12.22 to 35.97) | 22.60 (13.09 to 37.26) | 22.42 (13.18 to 36.96) | 21.38 (12.25 to 34.53) | 22.29 (12.88 to 36.15) | 23.39 (13.86 to 37.50) | 23.78 (14.33 to 38.00) | 22.59 (13.74 to 35.58) | 19.74 (11.80 to 30.78) | 16.06 (9.56 to 25.55) | 12.82 (7.80 to 20.45) | 7.98 (4.86 to 12.51) |
| North Africa and Middle East | Both | Prevalence | 148.99 (44.01 to 462.05) | 365.76 (116.96 to 1116.21) | 514.12 (149.33 to 1597.93) | 676.53 (185.74 to 2148.87) | 815.99 (219.63 to 2631.79) | 930.65 (247.77 to 3024.57) | 1008.64 (268.29 to 3288.32) | 1109.75 (294.88 to 3658.24) | 1208.71 (324.11 to 3984.55) | 1285.38 (341.53 to 4255.19) | 1262.56 (336.85 to 4203.76) | 1201.66 (322.03 to 4007.61) | 1247.65 (337.10 to 4169.09) | 1321.41 (361.29 to 4280.05) | 1356.47 (375.54 to 4466.92) | 1294.27 (364.36 to 4144.97) | 1176.49 (322.73 to 3901.38) | 978.04 (267.18 to 3123.75) | 733.48 (210.25 to 2324.90) | 471.40 (134.67 to 1464.82) |
| North Africa and Middle East | Female | DALY rates | 3.28 (2.07 to 5.09) | 5.27 (3.33 to 7.80) | 5.45 (3.32 to 8.01) | 5.57 (3.43 to 8.17) | 5.80 (3.67 to 8.64) | 6.06 (3.93 to 8.71) | 6.26 (4.17 to 9.05) | 6.62 (4.29 to 9.80) | 6.70 (4.26 to 10.07) | 6.93 (4.40 to 10.12) | 6.50 (4.22 to 9.22) | 6.04 (3.92 to 8.85) | 5.99 (3.91 to 8.81) | 6.17 (4.02 to 9.14) | 6.11 (4.04 to 8.79) | 5.40 (3.63 to 7.57) | 4.83 (3.23 to 6.96) | 3.75 (2.55 to 5.34) | 2.28 (1.55 to 3.20) | 1.31 (0.89 to 1.77) |
| North Africa and Middle East | Female | Prevalence | 152.46 (48.02 to 465.51) | 332.14 (89.28 to 1049.17) | 466.56 (108.65 to 1530.87) | 617.31 (130.66 to 2067.35) | 754.54 (151.45 to 2551.11) | 870.13 (170.61 to 2997.70) | 951.92 (183.91 to 3298.14) | 1045.79 (197.03 to 3643.13) | 1110.07 (203.67 to 3926.21) | 1181.53 (214.99 to 4181.27) | 1139.63 (205.40 to 4088.49) | 1081.41 (194.13 to 3930.09) | 1102.19 (195.04 to 4065.25) | 1158.94 (207.07 to 4163.45) | 1182.23 (212.96 to 4233.77) | 1068.10 (192.60 to 3587.66) | 960.84 (172.74 to 3380.26) | 766.11 (139.53 to 2726.99) | 470.71 (84.29 to 1582.68) | 277.29 (48.48 to 967.01) |
| North Africa and Middle East | Male | DALY rates | 2.34 (1.45 to 3.35) | 13.25 (6.35 to 27.58) | 16.26 (7.89 to 32.61) | 19.70 (9.92 to 38.05) | 23.33 (11.50 to 42.69) | 26.34 (13.64 to 48.56) | 28.43 (15.13 to 49.52) | 31.52 (16.49 to 55.95) | 35.18 (18.57 to 61.34) | 37.60 (20.56 to 64.31) | 37.68 (20.98 to 64.92) | 35.87 (19.77 to 59.74) | 37.59 (20.69 to 62.62) | 39.91 (22.49 to 66.03) | 40.77 (23.61 to 67.02) | 39.85 (23.01 to 64.70) | 36.13 (20.77 to 59.12) | 30.77 (17.54 to 50.82) | 26.25 (15.07 to 42.92) | 19.40 (11.31 to 31.46) |
| North Africa and Middle East | Male | Prevalence | 145.69 (39.89 to 458.76) | 397.82 (136.53 to 1173.16) | 559.16 (179.48 to 1680.92) | 732.93 (225.47 to 2221.23) | 875.49 (268.41 to 2695.53) | 988.67 (305.24 to 3080.15) | 1061.84 (330.24 to 3305.92) | 1169.93 (366.20 to 3673.41) | 1300.06 (410.04 to 4040.21) | 1384.74 (441.20 to 4326.97) | 1380.33 (440.60 to 4316.80) | 1315.32 (423.57 to 4093.76) | 1384.10 (450.44 to 4293.83) | 1477.31 (486.78 to 4418.01) | 1524.07 (504.08 to 4624.89) | 1521.31 (498.96 to 4730.62) | 1413.59 (458.88 to 4287.27) | 1231.20 (393.22 to 3719.71) | 1068.33 (339.42 to 3230.13) | 804.03 (255.81 to 2419.37) |
| Oceania | Both | DALY rates | 16.77 (11.05 to 24.51) | 339.81 (226.03 to 474.57) | 416.41 (283.92 to 578.07) | 473.02 (322.14 to 655.29) | 540.11 (367.43 to 743.47) | 612.90 (418.39 to 868.90) | 676.58 (461.35 to 951.33) | 734.96 (501.87 to 1051.84) | 799.06 (542.50 to 1130.65) | 853.48 (580.48 to 1210.38) | 907.58 (616.80 to 1299.22) | 965.83 (655.15 to 1380.64) | 1043.05 (719.77 to 1462.04) | 1058.34 (724.74 to 1479.67) | 1020.28 (707.42 to 1416.10) | 956.22 (661.51 to 1333.95) | 885.13 (610.50 to 1218.43) | 796.39 (555.32 to 1085.49) | 697.55 (487.21 to 947.71) | 724.13 (504.10 to 978.43) |
| Oceania | Both | Prevalence | 4612.18 (2052.80 to 8087.77) | 14069.49 (7549.24 to 22964.64) | 19769.82 (10396.18 to 32361.27) | 26106.70 (13415.19 to 43022.56) | 31893.36 (16074.31 to 52815.66) | 36326.35 (18395.91 to 60057.93) | 39327.54 (20047.19 to 64792.00) | 41794.79 (21548.12 to 68706.17) | 44342.76 (22996.47 to 72220.63) | 46293.59 (24319.17 to 74034.82) | 48023.79 (25422.57 to 74964.47) | 49823.51 (26404.18 to 76035.32) | 52434.16 (28060.84 to 77619.75) | 53163.53 (28860.90 to 77374.52) | 52499.84 (28646.77 to 76324.07) | 50622.74 (28276.64 to 73665.87) | 48397.13 (27320.27 to 71210.90) | 45829.31 (26474.34 to 67439.39) | 44074.21 (25520.67 to 65149.31) | 49126.57 (27691.12 to 73881.83) |
| Oceania | Female | DALY rates | 19.72 (12.60 to 28.73) | 47.74 (31.57 to 70.58) | 75.97 (49.62 to 114.69) | 131.01 (84.03 to 189.79) | 208.65 (132.05 to 302.99) | 287.72 (188.22 to 421.14) | 354.57 (226.11 to 522.14) | 416.96 (271.62 to 611.39) | 484.46 (311.65 to 705.80) | 539.07 (350.16 to 783.52) | 594.11 (382.16 to 878.43) | 656.77 (417.74 to 969.13) | 748.15 (484.23 to 1084.49) | 770.23 (502.16 to 1123.29) | 735.89 (481.56 to 1076.78) | 678.87 (448.61 to 965.36) | 602.98 (401.41 to 859.56) | 532.35 (356.67 to 754.84) | 470.98 (316.90 to 673.72) | 558.91 (378.40 to 793.96) |
| Oceania | Female | Prevalence | 4631.51 (2082.09 to 8091.08) | 11665.17 (5304.91 to 20292.24) | 16957.45 (7727.73 to 29406.72) | 23326.40 (10736.74 to 40255.03) | 29285.80 (13454.09 to 50232.64) | 33746.29 (15799.09 to 57440.74) | 36798.20 (17537.57 to 62168.58) | 39342.95 (19040.27 to 66143.30) | 42000.10 (20513.56 to 70429.38) | 43916.59 (21774.02 to 72666.77) | 45537.63 (22841.50 to 73473.49) | 47407.90 (24060.88 to 74694.17) | 50452.67 (25819.36 to 77275.74) | 51318.13 (26652.79 to 77087.93) | 50388.17 (26343.19 to 75383.48) | 48254.07 (25952.89 to 71960.80) | 45155.10 (24918.58 to 67525.17) | 42276.57 (23755.91 to 63885.03) | 40903.51 (22374.18 to 63638.40) | 47300.91 (25770.87 to 73689.97) |
| Oceania | Male | DALY rates | 14.04 (8.88 to 21.21) | 606.22 (403.30 to 844.19) | 726.30 (490.79 to 1014.89) | 791.94 (537.41 to 1095.82) | 857.73 (584.70 to 1202.05) | 927.88 (639.41 to 1292.27) | 984.98 (682.37 to 1341.72) | 1034.82 (708.97 to 1460.32) | 1091.48 (757.61 to 1519.87) | 1141.79 (789.53 to 1589.29) | 1191.56 (819.96 to 1687.58) | 1243.37 (868.28 to 1788.09) | 1314.43 (914.26 to 1846.87) | 1324.76 (932.76 to 1871.96) | 1286.87 (901.22 to 1819.86) | 1223.91 (857.27 to 1713.56) | 1170.80 (828.60 to 1613.69) | 1115.62 (797.29 to 1532.17) | 1097.85 (781.72 to 1511.00) | 1170.17 (843.61 to 1586.26) |
| Oceania | Male | Prevalence | 4594.34 (2025.76 to 8084.72) | 16262.63 (9635.01 to 25178.92) | 22329.77 (12848.74 to 35050.65) | 28699.27 (15912.79 to 45603.22) | 34392.12 (18620.58 to 55290.89) | 38825.56 (20896.66 to 62593.11) | 41750.03 (22450.78 to 67315.13) | 44106.75 (23801.14 to 70920.29) | 46520.23 (25115.96 to 74182.12) | 48473.34 (26389.52 to 75396.95) | 50276.02 (27478.53 to 76373.84) | 51992.75 (28593.97 to 77190.80) | 54257.63 (30200.37 to 78035.12) | 54870.01 (30898.54 to 77878.72) | 54479.40 (30769.52 to 77639.16) | 52908.97 (30426.61 to 75678.21) | 51679.45 (30113.20 to 74611.03) | 50124.83 (29531.18 to 71825.73) | 49676.25 (28632.89 to 69782.94) | 54055.47 (30063.10 to 76210.01) |
| South Asia | Both | DALY rates | 10.68 (7.33 to 14.95) | 98.85 (64.79 to 147.26) | 128.89 (84.93 to 188.48) | 166.01 (111.23 to 237.55) | 199.77 (133.39 to 286.39) | 224.67 (150.80 to 318.97) | 249.00 (169.13 to 351.60) | 276.80 (188.07 to 389.46) | 294.46 (201.73 to 410.26) | 313.49 (214.67 to 438.01) | 330.51 (227.17 to 458.88) | 346.92 (237.32 to 485.10) | 367.26 (252.75 to 510.45) | 358.84 (247.57 to 493.67) | 330.10 (229.34 to 453.68) | 322.01 (224.25 to 437.91) | 327.23 (230.85 to 443.90) | 293.40 (208.13 to 391.56) | 259.14 (185.00 to 346.71) | 233.99 (167.75 to 311.79) |
| South Asia | Both | Prevalence | 1608.96 (1482.33 to 1738.93) | 4532.01 (4166.07 to 4935.97) | 6376.61 (5897.69 to 6886.64) | 8572.79 (7954.15 to 9240.89) | 10416.73 (9694.08 to 11218.04) | 11715.49 (10925.77 to 12624.38) | 12891.84 (12032.14 to 13877.27) | 14179.33 (13225.89 to 15243.92) | 14927.93 (13919.92 to 16028.42) | 15701.15 (14638.56 to 16860.98) | 16498.51 (15375.07 to 17706.89) | 17335.79 (16151.00 to 18606.11) | 18262.38 (17018.09 to 19590.47) | 18189.30 (16926.01 to 19528.03) | 17340.95 (16129.76 to 18645.14) | 17458.22 (16227.08 to 18752.92) | 18300.92 (17002.87 to 19624.79) | 16840.20 (15625.40 to 18133.97) | 15378.37 (14237.47 to 16625.87) | 14463.62 (13326.38 to 15755.17) |
| South Asia | Female | DALY rates | 12.51 (8.53 to 17.67) | 24.52 (16.56 to 34.71) | 30.63 (20.79 to 44.32) | 41.03 (27.53 to 59.84) | 54.65 (36.76 to 79.41) | 68.20 (45.70 to 99.14) | 81.62 (54.30 to 118.17) | 97.41 (64.38 to 143.42) | 107.63 (71.10 to 157.87) | 118.77 (78.75 to 173.32) | 130.78 (86.73 to 189.01) | 142.86 (93.60 to 205.67) | 156.13 (103.77 to 221.49) | 160.04 (107.31 to 229.50) | 150.39 (99.07 to 212.78) | 150.06 (100.42 to 213.90) | 160.70 (107.95 to 224.64) | 148.77 (101.67 to 208.51) | 132.30 (90.36 to 184.25) | 123.49 (84.65 to 170.93) |
| South Asia | Female | Prevalence | 1612.66 (1487.45 to 1739.03) | 3893.27 (3586.54 to 4203.82) | 5499.41 (5061.93 to 5938.69) | 7388.54 (6809.45 to 7974.43) | 9081.08 (8382.31 to 9795.11) | 10388.08 (9593.68 to 11205.48) | 11515.48 (10631.31 to 12427.03) | 12735.49 (11752.60 to 13729.91) | 13313.25 (12282.75 to 14341.17) | 13928.11 (12855.92 to 14994.21) | 14639.01 (13511.19 to 15761.01) | 15366.93 (14195.13 to 16541.52) | 16077.65 (14854.51 to 17291.96) | 16310.25 (15074.01 to 17542.18) | 15790.31 (14587.53 to 16981.30) | 15961.24 (14747.69 to 17154.01) | 16924.39 (15638.52 to 18185.89) | 16067.65 (14840.31 to 17260.37) | 15020.74 (13856.24 to 16203.42) | 14435.03 (13243.08 to 15626.01) |
| South Asia | Male | DALY rates | 8.97 (6.10 to 12.87) | 167.29 (108.76 to 253.07) | 219.65 (143.88 to 324.54) | 282.70 (188.29 to 411.25) | 336.78 (222.87 to 484.23) | 373.14 (249.38 to 528.99) | 405.44 (269.29 to 577.37) | 442.13 (295.97 to 621.84) | 464.10 (314.96 to 656.62) | 485.44 (327.69 to 677.79) | 506.11 (346.46 to 702.96) | 526.97 (361.25 to 731.71) | 552.75 (380.14 to 763.44) | 538.21 (373.15 to 737.23) | 499.91 (349.49 to 683.68) | 486.76 (342.51 to 660.45) | 489.91 (349.25 to 662.42) | 433.57 (307.72 to 582.23) | 384.98 (273.38 to 517.75) | 348.19 (249.10 to 465.57) |
| South Asia | Male | Prevalence | 1605.52 (1477.55 to 1740.07) | 5120.16 (4667.94 to 5656.19) | 7186.78 (6589.12 to 7850.95) | 9678.49 (8935.69 to 10489.04) | 11677.83 (10817.54 to 12589.04) | 12975.11 (12060.01 to 13962.86) | 14178.25 (13203.52 to 15248.26) | 15510.01 (14461.46 to 16649.70) | 16393.99 (15285.26 to 17583.70) | 17266.81 (16092.68 to 18513.97) | 18133.29 (16884.77 to 19417.10) | 19073.04 (17768.33 to 20408.55) | 20181.79 (18823.11 to 21588.79) | 19884.74 (18519.09 to 21298.18) | 18806.15 (17501.49 to 20142.94) | 18892.58 (17608.94 to 20195.74) | 19645.68 (18304.91 to 21015.22) | 17588.88 (16383.88 to 18911.79) | 15733.17 (14620.81 to 16976.33) | 14493.16 (13340.21 to 15791.90) |
| Southeast Asia | Both | DALY rates | 11.70 (8.06 to 16.64) | 86.90 (50.16 to 142.65) | 137.63 (85.64 to 211.54) | 198.12 (129.64 to 287.54) | 239.50 (158.65 to 343.73) | 268.88 (179.30 to 380.82) | 292.26 (194.50 to 407.80) | 307.63 (204.63 to 429.63) | 313.33 (208.66 to 438.62) | 318.27 (213.92 to 443.41) | 322.45 (217.54 to 447.04) | 327.25 (220.31 to 450.19) | 331.59 (224.98 to 449.72) | 324.95 (221.25 to 440.81) | 306.63 (212.27 to 413.86) | 287.75 (197.91 to 387.83) | 269.20 (187.28 to 364.16) | 259.34 (182.79 to 349.55) | 218.65 (155.04 to 292.71) | 154.86 (110.22 to 204.30) |
| Southeast Asia | Both | Prevalence | 1659.53 (818.43 to 3002.95) | 4582.65 (2321.03 to 8034.98) | 6699.70 (3483.62 to 11709.86) | 9126.43 (4829.84 to 15862.94) | 11010.82 (5883.06 to 19058.27) | 12421.47 (6725.66 to 21660.38) | 13439.05 (7272.70 to 23097.36) | 14208.36 (7639.53 to 24242.19) | 14752.83 (7849.48 to 25704.40) | 15387.67 (8144.48 to 27197.52) | 15877.12 (8375.53 to 28242.23) | 16413.10 (8800.97 to 28852.07) | 17011.68 (9084.83 to 29941.16) | 17294.39 (9212.41 to 30426.67) | 17013.30 (9089.50 to 29703.86) | 16647.69 (8954.29 to 29102.00) | 16276.11 (8775.53 to 28633.75) | 16347.93 (8535.98 to 29508.26) | 15750.63 (7634.69 to 31079.07) | 14221.17 (6357.11 to 28396.30) |
| Southeast Asia | Female | DALY rates | 13.78 (9.41 to 19.79) | 25.90 (17.41 to 37.31) | 29.88 (19.98 to 43.08) | 35.39 (23.74 to 49.82) | 40.97 (27.15 to 58.79) | 45.56 (30.74 to 66.03) | 48.99 (33.12 to 70.21) | 51.82 (35.51 to 73.98) | 54.43 (37.14 to 77.83) | 56.83 (38.94 to 82.27) | 58.58 (39.41 to 83.24) | 60.49 (40.75 to 87.39) | 62.91 (42.55 to 90.07) | 63.21 (43.35 to 89.51) | 61.42 (42.43 to 86.19) | 58.31 (40.02 to 81.80) | 56.05 (39.42 to 79.15) | 55.04 (38.57 to 76.08) | 51.91 (37.40 to 71.53) | 46.12 (32.54 to 62.15) |
| Southeast Asia | Female | Prevalence | 1680.82 (835.13 to 3022.62) | 4089.98 (1990.77 to 7536.12) | 5827.12 (2756.56 to 10780.37) | 7806.80 (3630.39 to 14511.24) | 9445.16 (4348.54 to 17624.45) | 10634.23 (4960.12 to 19910.13) | 11441.85 (5355.83 to 21136.04) | 12082.49 (5674.02 to 22085.59) | 12613.00 (5835.88 to 23674.39) | 13236.41 (6060.19 to 25239.07) | 13670.92 (6283.38 to 26282.61) | 14148.72 (6547.29 to 26831.70) | 14709.40 (6769.96 to 27814.77) | 15016.92 (6917.85 to 28361.23) | 14847.10 (6888.94 to 27801.01) | 14575.75 (6745.12 to 27303.08) | 14360.50 (6607.83 to 27376.17) | 14430.53 (6367.81 to 28950.48) | 14119.50 (6006.27 to 30303.06) | 13154.63 (5008.69 to 28502.59) |
| Southeast Asia | Male | DALY rates | 9.73 (6.47 to 13.81) | 145.25 (79.23 to 247.37) | 241.33 (146.21 to 382.14) | 362.10 (233.34 to 531.75) | 447.24 (292.71 to 644.74) | 506.44 (336.42 to 723.41) | 543.26 (359.90 to 760.57) | 566.76 (376.70 to 796.31) | 576.43 (382.59 to 811.58) | 592.86 (397.14 to 831.38) | 604.08 (407.15 to 840.65) | 615.25 (416.29 to 847.01) | 622.00 (422.78 to 852.23) | 619.67 (419.44 to 847.32) | 600.17 (409.85 to 812.93) | 581.79 (396.11 to 790.45) | 560.20 (388.06 to 757.80) | 557.31 (387.47 to 750.27) | 539.34 (377.49 to 725.26) | 490.62 (346.95 to 653.31) |
| Southeast Asia | Male | Prevalence | 1639.27 (797.45 to 2984.24) | 5053.91 (2728.36 to 8492.37) | 7539.49 (4221.73 to 12504.67) | 10456.18 (6087.86 to 17194.33) | 12649.12 (7475.03 to 20672.10) | 14322.65 (8531.90 to 23461.16) | 15499.81 (9220.99 to 25157.93) | 16361.75 (9660.33 to 26426.73) | 16927.27 (10033.75 to 27767.24) | 17647.10 (10433.03 to 29256.81) | 18231.75 (10773.19 to 30357.20) | 18857.83 (11114.86 to 31241.48) | 19500.10 (11482.32 to 32285.04) | 19858.87 (11690.58 to 32815.09) | 19606.33 (11634.38 to 32378.29) | 19303.08 (11593.97 to 31557.03) | 18891.45 (11294.95 to 30918.48) | 19144.42 (11084.77 to 31619.00) | 18887.73 (10693.46 to 32217.05) | 17514.29 (10019.21 to 30536.18) |
| Southern Sub-Saharan Africa | Both | DALY rates | 2.27 (1.38 to 3.60) | 7.10 (3.87 to 12.87) | 7.36 (3.96 to 12.96) | 7.35 (3.92 to 13.08) | 7.00 (3.80 to 12.62) | 6.40 (3.40 to 11.39) | 6.47 (3.45 to 11.83) | 6.78 (3.64 to 11.99) | 6.49 (3.57 to 11.44) | 6.81 (3.65 to 12.04) | 7.23 (3.97 to 13.20) | 7.10 (3.68 to 13.21) | 7.04 (3.87 to 12.55) | 7.43 (4.16 to 12.97) | 6.95 (3.67 to 12.12) | 6.04 (3.35 to 10.43) | 3.99 (2.31 to 6.83) | 3.00 (1.76 to 4.89) | 2.29 (1.36 to 3.53) | 2.03 (1.25 to 3.09) |
| Southern Sub-Saharan Africa | Both | Prevalence | 75.39 (27.58 to 253.35) | 197.15 (69.63 to 643.61) | 253.57 (77.84 to 881.70) | 299.93 (82.73 to 1085.19) | 324.54 (83.95 to 1200.48) | 320.36 (79.17 to 1199.34) | 338.83 (82.08 to 1275.67) | 360.06 (86.03 to 1359.87) | 349.45 (82.68 to 1323.32) | 373.55 (88.36 to 1415.16) | 400.96 (94.23 to 1521.26) | 389.77 (92.27 to 1477.06) | 394.25 (93.07 to 1496.26) | 436.45 (100.95 to 1664.28) | 419.05 (96.45 to 1599.81) | 387.11 (88.39 to 1484.95) | 282.99 (63.41 to 1095.13) | 234.68 (51.67 to 914.37) | 197.25 (42.23 to 774.74) | 195.18 (40.45 to 772.42) |
| Southern Sub-Saharan Africa | Female | DALY rates | 2.67 (1.59 to 4.15) | 4.61 (2.78 to 7.14) | 4.44 (2.62 to 6.81) | 4.21 (2.47 to 6.47) | 3.90 (2.31 to 5.88) | 3.56 (2.06 to 5.43) | 3.48 (1.94 to 5.34) | 3.51 (2.14 to 5.43) | 3.28 (1.99 to 4.90) | 3.19 (1.86 to 4.93) | 3.21 (1.86 to 4.96) | 2.81 (1.76 to 4.25) | 2.67 (1.64 to 4.12) | 3.04 (1.78 to 4.53) | 2.87 (1.74 to 4.24) | 2.72 (1.69 to 4.22) | 2.10 (1.25 to 3.22) | 1.78 (1.03 to 2.66) | 1.56 (0.94 to 2.42) | 1.54 (0.90 to 2.31) |
| Southern Sub-Saharan Africa | Female | Prevalence | 79.58 (31.37 to 258.94) | 178.23 (58.37 to 625.29) | 230.51 (63.19 to 854.91) | 273.47 (65.34 to 1050.51) | 302.46 (66.06 to 1183.85) | 305.75 (63.35 to 1208.95) | 324.78 (65.19 to 1291.85) | 340.98 (66.81 to 1362.04) | 331.10 (63.69 to 1326.59) | 336.93 (63.81 to 1353.35) | 346.63 (64.65 to 1395.49) | 311.31 (57.33 to 1255.93) | 306.35 (55.77 to 1238.74) | 358.91 (64.90 to 1452.78) | 342.11 (61.83 to 1384.82) | 330.71 (59.83 to 1338.56) | 264.78 (47.93 to 1071.70) | 231.90 (41.96 to 938.63) | 203.90 (36.81 to 825.32) | 205.31 (37.17 to 831.04) |
| Southern Sub-Saharan Africa | Male | DALY rates | 1.88 (0.96 to 3.11) | 9.60 (4.09 to 21.10) | 10.36 (4.61 to 20.59) | 10.66 (4.70 to 21.65) | 10.35 (4.69 to 20.31) | 9.50 (4.00 to 19.20) | 9.67 (4.38 to 19.14) | 10.23 (4.76 to 19.53) | 9.84 (4.47 to 19.10) | 10.67 (5.06 to 20.55) | 11.82 (5.62 to 23.43) | 12.24 (5.64 to 23.99) | 12.49 (6.04 to 24.53) | 13.12 (6.50 to 24.97) | 12.62 (5.91 to 24.27) | 11.12 (5.50 to 21.29) | 7.27 (3.45 to 13.96) | 5.37 (2.44 to 10.21) | 4.01 (1.99 to 7.55) | 3.54 (1.70 to 6.84) |
| Southern Sub-Saharan Africa | Male | Prevalence | 71.26 (23.57 to 247.86) | 216.19 (75.61 to 655.23) | 277.16 (86.89 to 903.66) | 327.81 (95.29 to 1118.54) | 348.39 (96.76 to 1216.71) | 336.25 (91.36 to 1188.18) | 353.81 (94.78 to 1258.09) | 380.17 (101.01 to 1357.57) | 368.57 (97.72 to 1319.91) | 412.71 (108.82 to 1481.24) | 463.02 (121.58 to 1664.92) | 483.79 (126.93 to 1742.08) | 503.91 (131.69 to 1817.56) | 536.89 (140.27 to 1938.22) | 526.19 (137.28 to 1899.20) | 473.34 (123.51 to 1708.75) | 314.68 (82.21 to 1135.91) | 240.13 (62.63 to 866.74) | 181.56 (47.37 to 655.24) | 164.05 (42.74 to 592.13) |
| Tropical Latin America | Both | DALY rates | 1.90 (1.27 to 2.76) | 4.20 (2.67 to 6.30) | 4.52 (2.84 to 6.82) | 4.83 (3.00 to 7.48) | 4.74 (2.91 to 7.25) | 4.51 (2.83 to 6.98) | 4.48 (2.87 to 6.79) | 4.57 (2.81 to 6.94) | 4.89 (3.03 to 7.28) | 5.21 (3.35 to 7.86) | 5.32 (3.46 to 8.04) | 5.30 (3.41 to 8.06) | 5.61 (3.54 to 8.49) | 6.10 (3.80 to 9.34) | 6.32 (4.04 to 9.67) | 6.37 (4.12 to 9.48) | 5.94 (3.85 to 8.80) | 6.24 (3.92 to 9.28) | 5.60 (3.76 to 7.87) | 6.88 (4.70 to 9.64) |
| Tropical Latin America | Both | Prevalence | 44.51 (35.23 to 75.01) | 100.06 (76.44 to 169.34) | 132.12 (97.65 to 235.76) | 169.01 (122.25 to 312.85) | 184.39 (132.06 to 347.74) | 186.46 (132.95 to 354.75) | 190.79 (135.63 to 364.68) | 202.03 (143.13 to 387.77) | 220.26 (155.83 to 423.82) | 240.84 (170.09 to 464.57) | 251.11 (177.06 to 485.73) | 258.81 (182.31 to 502.21) | 279.61 (196.81 to 543.01) | 307.16 (216.16 to 596.64) | 324.36 (228.25 to 629.95) | 323.77 (228.52 to 626.83) | 319.36 (224.66 to 620.71) | 357.53 (250.37 to 698.75) | 309.87 (218.67 to 601.12) | 298.28 (218.59 to 554.18) |
| Tropical Latin America | Female | DALY rates | 2.26 (1.42 to 3.31) | 3.84 (2.43 to 5.62) | 3.85 (2.51 to 5.68) | 3.74 (2.44 to 5.71) | 3.47 (2.18 to 5.06) | 3.10 (2.03 to 4.52) | 2.98 (1.98 to 4.24) | 2.95 (1.94 to 4.36) | 3.05 (1.99 to 4.49) | 3.16 (2.07 to 4.62) | 3.11 (2.03 to 4.48) | 2.97 (1.96 to 4.36) | 2.96 (2.00 to 4.32) | 3.11 (2.06 to 4.50) | 3.20 (2.12 to 4.55) | 3.32 (2.22 to 4.70) | 3.24 (2.17 to 4.59) | 3.34 (2.23 to 4.76) | 3.81 (2.54 to 5.54) | 5.74 (3.83 to 8.43) |
| Tropical Latin America | Female | Prevalence | 48.03 (38.67 to 78.16) | 98.71 (76.99 to 170.64) | 128.26 (96.27 to 235.67) | 162.32 (117.78 to 312.15) | 178.00 (126.79 to 350.64) | 180.63 (127.40 to 360.30) | 186.31 (130.74 to 374.17) | 198.01 (138.23 to 400.28) | 216.91 (150.73 to 440.63) | 236.27 (163.73 to 481.82) | 245.91 (169.79 to 503.43) | 250.56 (172.46 to 514.86) | 263.99 (181.33 to 544.06) | 284.56 (195.13 to 587.50) | 295.16 (202.50 to 609.10) | 289.16 (199.26 to 593.59) | 294.03 (202.39 to 604.40) | 328.16 (225.13 to 677.06) | 304.85 (212.30 to 617.77) | 289.55 (211.19 to 553.15) |
| Tropical Latin America | Male | DALY rates | 1.56 (0.98 to 2.35) | 4.54 (2.58 to 7.70) | 5.18 (2.95 to 8.57) | 5.93 (3.38 to 10.16) | 6.05 (3.43 to 10.13) | 5.98 (3.47 to 10.04) | 6.03 (3.49 to 10.18) | 6.27 (3.55 to 10.42) | 6.78 (3.84 to 10.91) | 7.33 (4.26 to 11.96) | 7.63 (4.49 to 12.32) | 7.84 (4.58 to 12.74) | 8.53 (4.95 to 14.04) | 9.43 (5.48 to 15.51) | 9.95 (5.69 to 15.89) | 10.11 (6.00 to 16.20) | 9.63 (5.71 to 15.43) | 10.71 (6.34 to 17.44) | 8.86 (5.57 to 13.88) | 9.17 (5.83 to 13.65) |
| Tropical Latin America | Male | Prevalence | 41.12 (31.77 to 71.54) | 101.38 (75.59 to 169.67) | 135.94 (99.48 to 239.00) | 175.80 (126.62 to 317.35) | 190.95 (137.01 to 348.80) | 192.55 (138.11 to 353.09) | 195.48 (140.20 to 358.98) | 206.25 (147.93 to 379.18) | 223.71 (160.57 to 411.39) | 245.56 (176.22 to 452.04) | 256.56 (184.16 to 472.73) | 267.78 (192.11 to 494.19) | 296.87 (213.04 to 547.83) | 332.33 (238.74 to 613.23) | 358.21 (257.13 to 661.15) | 366.06 (263.24 to 674.76) | 353.89 (254.74 to 651.13) | 402.93 (289.61 to 742.48) | 319.01 (232.12 to 579.93) | 315.70 (232.47 to 565.09) |
| Western Sub-Saharan Africa | Both | DALY rates | 12.11 (8.25 to 17.21) | 150.90 (97.32 to 222.92) | 223.74 (149.55 to 316.79) | 287.07 (190.88 to 399.99) | 326.30 (219.72 to 450.31) | 356.12 (241.17 to 490.56) | 386.95 (263.87 to 529.32) | 416.31 (284.31 to 573.77) | 446.17 (305.02 to 607.68) | 458.76 (315.06 to 628.07) | 477.05 (329.92 to 656.85) | 494.04 (343.44 to 677.24) | 503.07 (350.45 to 694.19) | 463.64 (323.57 to 633.72) | 435.53 (305.76 to 593.93) | 408.03 (288.39 to 557.86) | 382.29 (271.63 to 516.43) | 349.93 (249.24 to 467.74) | 321.55 (230.80 to 431.06) | 283.43 (205.34 to 378.12) |
| Western Sub-Saharan Africa | Both | Prevalence | 2359.70 (1440.60 to 3674.09) | 7192.14 (4724.58 to 10658.80) | 10429.47 (6844.14 to 15515.76) | 14004.08 (9152.76 to 20859.25) | 17027.90 (11043.92 to 25539.43) | 19271.31 (12504.75 to 28980.69) | 20921.96 (13552.88 to 31261.84) | 22178.17 (14292.28 to 33325.64) | 23411.68 (15177.34 to 34993.22) | 24251.63 (15592.18 to 36399.82) | 25195.19 (16441.16 to 37287.11) | 26125.30 (17073.91 to 37697.00) | 27153.47 (17868.20 to 39265.58) | 27048.51 (17570.82 to 39834.17) | 26543.93 (16950.09 to 39356.02) | 26110.47 (16384.04 to 39156.78) | 25554.13 (15701.72 to 39019.19) | 24636.14 (14834.85 to 38586.18) | 24191.38 (14229.29 to 38574.21) | 24150.55 (13812.51 to 38908.76) |
| Western Sub-Saharan Africa | Female | DALY rates | 14.25 (9.72 to 19.97) | 30.54 (20.66 to 43.54) | 39.46 (27.26 to 57.10) | 54.33 (36.95 to 79.45) | 73.39 (49.92 to 107.00) | 91.54 (61.49 to 131.82) | 106.65 (71.29 to 153.23) | 118.60 (79.51 to 172.50) | 131.64 (87.43 to 191.81) | 141.04 (94.51 to 205.67) | 153.01 (101.31 to 222.35) | 164.71 (108.69 to 239.96) | 175.89 (116.80 to 251.53) | 170.05 (114.18 to 242.89) | 160.87 (107.39 to 229.59) | 153.36 (102.70 to 216.04) | 146.53 (99.37 to 206.67) | 138.82 (94.85 to 195.79) | 133.46 (91.09 to 186.61) | 131.57 (90.42 to 182.21) |
| Western Sub-Saharan Africa | Female | Prevalence | 2393.82 (1460.93 to 3727.81) | 6215.92 (3770.11 to 9731.46) | 8937.58 (5327.91 to 14038.34) | 12137.27 (7160.74 to 19083.12) | 14994.06 (8952.35 to 23624.43) | 17089.05 (10348.56 to 26700.70) | 18536.85 (11314.83 to 28891.76) | 19552.84 (12043.72 to 30411.33) | 20550.45 (12946.21 to 31671.00) | 21314.98 (13285.27 to 32863.96) | 22157.40 (14030.47 to 33663.46) | 23020.55 (14668.47 to 34015.02) | 24050.23 (15232.88 to 36276.45) | 24345.13 (14834.50 to 37993.76) | 24050.72 (14254.04 to 38592.87) | 23859.49 (13815.67 to 38435.25) | 23496.87 (13351.99 to 38165.08) | 22906.06 (12837.82 to 37729.35) | 22635.02 (12479.04 to 37415.19) | 22777.81 (12279.31 to 37755.18) |
| Western Sub-Saharan Africa | Male | DALY rates | 9.99 (6.60 to 14.25) | 273.68 (174.94 to 410.70) | 416.47 (276.86 to 595.05) | 539.94 (359.42 to 756.33) | 612.42 (411.26 to 838.20) | 651.98 (440.16 to 895.62) | 679.45 (461.06 to 927.46) | 699.45 (477.81 to 957.88) | 719.70 (492.37 to 980.52) | 730.38 (499.72 to 995.17) | 743.81 (510.86 to 1017.74) | 756.77 (522.74 to 1027.74) | 771.59 (532.65 to 1053.11) | 757.83 (524.84 to 1020.30) | 732.15 (509.89 to 978.18) | 704.53 (494.30 to 942.21) | 682.38 (481.37 to 909.03) | 647.41 (459.72 to 857.75) | 629.77 (448.40 to 834.40) | 623.60 (450.83 to 820.47) |
| Western Sub-Saharan Africa | Male | Prevalence | 2325.93 (1417.73 to 3615.37) | 8187.97 (5713.39 to 11595.93) | 11989.81 (8410.13 to 16987.26) | 16032.35 (11185.06 to 22919.63) | 19328.81 (13380.61 to 27775.16) | 21711.52 (14860.54 to 31415.40) | 23410.91 (15816.28 to 34153.97) | 24675.06 (16529.38 to 36060.45) | 25899.95 (17136.89 to 37909.42) | 26762.24 (17657.87 to 39296.32) | 27695.98 (18328.68 to 40447.60) | 28602.16 (19046.82 to 40706.37) | 29700.36 (19950.60 to 41980.54) | 29757.44 (20186.63 to 41811.05) | 29236.50 (19911.90 to 41005.60) | 28731.09 (19507.04 to 40480.97) | 28172.78 (18917.58 to 40352.66) | 27074.03 (17764.06 to 39468.64) | 26741.91 (17184.50 to 39810.24) | 27225.39 (17364.48 to 41432.10) |
| **Country and territory** |  |  |  |  |  |  |  |  |  |  |  |  |  |  |  |  |  |  |  |  |  |  |
| American Samoa | Both | DALY rates | 10.54 (6.43 to 15.79) | 63.70 (34.65 to 110.38) | 95.16 (53.32 to 158.04) | 140.78 (83.23 to 224.15) | 180.00 (110.33 to 277.79) | 213.93 (136.00 to 314.82) | 247.00 (160.49 to 363.63) | 272.73 (176.65 to 403.56) | 298.46 (194.61 to 434.43) | 316.91 (212.31 to 452.08) | 335.03 (222.49 to 476.76) | 342.95 (227.72 to 480.95) | 330.67 (220.69 to 461.93) | 323.90 (216.34 to 447.57) | 319.88 (216.08 to 450.97) | 304.42 (211.23 to 419.27) | 291.08 (199.72 to 396.29) | 262.59 (183.30 to 358.09) | 232.70 (164.36 to 315.66) | 178.30 (122.39 to 240.29) |
| American Samoa | Both | Prevalence | 1258.70 (1068.46 to 1485.91) | 3560.94 (3017.44 to 4243.57) | 5213.54 (4420.27 to 6186.18) | 7183.85 (6129.47 to 8463.00) | 8858.40 (7575.82 to 10374.95) | 10123.40 (8695.97 to 11816.57) | 11108.57 (9576.43 to 12903.66) | 11902.86 (10285.22 to 13789.25) | 12612.89 (10918.97 to 14576.63) | 13206.73 (11435.14 to 15244.84) | 13772.65 (11946.26 to 15879.46) | 14269.88 (12395.66 to 16428.49) | 14678.29 (12755.56 to 16924.17) | 14892.09 (12932.03 to 17183.34) | 14910.92 (12962.87 to 17197.31) | 14831.75 (12883.96 to 17125.36) | 14758.34 (12809.06 to 17046.69) | 14547.89 (12620.01 to 16834.06) | 14319.52 (12395.35 to 16611.69) | 13819.75 (11901.46 to 16123.68) |
| American Samoa | Female | DALY rates | 12.29 (7.15 to 18.85) | 24.16 (14.80 to 37.59) | 26.76 (16.83 to 41.94) | 30.07 (17.71 to 46.37) | 33.70 (20.47 to 51.99) | 36.11 (22.85 to 53.37) | 38.02 (23.81 to 56.88) | 39.66 (24.73 to 58.19) | 41.12 (25.48 to 62.11) | 41.76 (25.93 to 62.07) | 43.28 (27.57 to 64.00) | 44.13 (28.06 to 63.11) | 45.41 (29.03 to 68.68) | 45.66 (29.42 to 64.67) | 44.93 (29.34 to 66.68) | 43.65 (28.86 to 64.13) | 42.97 (28.63 to 61.51) | 42.21 (28.15 to 60.48) | 40.96 (27.60 to 59.26) | 40.31 (26.30 to 56.36) |
| American Samoa | Female | Prevalence | 1275.56 (1087.14 to 1502.15) | 3250.29 (2759.83 to 3844.66) | 4669.40 (3952.91 to 5537.12) | 6294.87 (5320.50 to 7473.71) | 7676.29 (6483.08 to 9118.31) | 8675.79 (7323.61 to 10306.75) | 9404.28 (7937.91 to 11174.37) | 9989.72 (8430.69 to 11870.97) | 10486.29 (8851.33 to 12461.25) | 10915.14 (9212.24 to 12971.47) | 11321.29 (9553.02 to 13455.05) | 11739.12 (9906.21 to 13952.05) | 12222.58 (10314.14 to 14524.63) | 12450.04 (10509.06 to 14796.59) | 12450.06 (10505.91 to 14796.12) | 12450.05 (10506.97 to 14795.73) | 12450.05 (10506.26 to 14794.90) | 12450.09 (10506.86 to 14795.95) | 12450.07 (10506.02 to 14795.24) | 12450.07 (10505.78 to 14793.82) |
| American Samoa | Male | DALY rates | 8.93 (4.98 to 14.25) | 99.89 (50.05 to 182.91) | 156.17 (82.18 to 268.41) | 243.54 (136.58 to 397.01) | 333.81 (202.01 to 522.66) | 402.78 (249.63 to 601.60) | 454.97 (295.11 to 670.93) | 494.24 (319.79 to 738.27) | 525.86 (342.22 to 767.20) | 550.29 (368.71 to 783.37) | 572.33 (378.03 to 816.11) | 593.46 (391.09 to 840.63) | 612.15 (404.22 to 861.05) | 612.99 (404.38 to 853.82) | 601.32 (404.16 to 850.16) | 588.92 (401.59 to 821.48) | 578.18 (395.22 to 792.55) | 565.40 (389.67 to 774.26) | 551.73 (382.06 to 749.44) | 542.02 (368.68 to 738.40) |
| American Samoa | Male | Prevalence | 1243.16 (1052.29 to 1469.01) | 3845.29 (3209.13 to 4661.75) | 5698.88 (4782.19 to 6815.24) | 8008.99 (6831.78 to 9408.83) | 10101.22 (8697.48 to 11717.52) | 11660.74 (10142.78 to 13557.82) | 12804.70 (11136.96 to 14782.40) | 13721.14 (11941.24 to 15804.52) | 14492.10 (12611.38 to 16621.79) | 15150.49 (13218.39 to 17342.47) | 15766.61 (13795.86 to 17999.45) | 16391.55 (14392.89 to 18706.79) | 17101.50 (15051.69 to 19511.27) | 17429.41 (15367.38 to 19823.18) | 17429.96 (15355.72 to 19821.95) | 17430.22 (15365.11 to 19824.40) | 17429.37 (15358.98 to 19829.79) | 17430.40 (15374.37 to 19851.41) | 17429.87 (15380.00 to 19874.95) | 17429.98 (15356.77 to 19834.93) |
| Angola | Both | DALY rates | 10.31 (6.37 to 15.62) | 57.12 (29.71 to 100.29) | 82.24 (46.21 to 137.76) | 120.63 (67.50 to 194.39) | 160.72 (98.46 to 246.05) | 190.33 (119.20 to 289.07) | 216.88 (141.45 to 322.00) | 237.94 (154.84 to 348.37) | 254.76 (170.05 to 375.00) | 268.93 (178.57 to 386.45) | 281.40 (189.37 to 404.66) | 292.65 (193.97 to 414.27) | 298.44 (199.58 to 420.36) | 295.73 (195.46 to 414.36) | 288.38 (195.75 to 409.18) | 276.50 (186.61 to 384.15) | 258.06 (174.40 to 355.96) | 237.79 (164.84 to 324.01) | 218.78 (149.11 to 301.17) | 187.33 (131.72 to 253.49) |
| Angola | Both | Prevalence | 1140.71 (231.63 to 3615.18) | 3265.24 (812.24 to 9908.92) | 4748.37 (1180.35 to 14453.19) | 6526.13 (1684.15 to 19719.23) | 8114.09 (2175.54 to 24295.43) | 9285.43 (2534.97 to 27638.04) | 10154.41 (2821.33 to 30061.00) | 10857.83 (3066.65 to 32013.33) | 11447.07 (3273.74 to 33630.61) | 11970.58 (3462.24 to 35091.65) | 12443.43 (3635.27 to 36405.53) | 12950.67 (3824.32 to 37817.21) | 13461.72 (3982.84 to 39325.82) | 13685.95 (4037.62 to 40039.46) | 13661.89 (4007.62 to 40018.04) | 13606.82 (3936.88 to 39944.25) | 13484.87 (3790.42 to 39825.46) | 13339.73 (3652.58 to 39660.74) | 13204.33 (3514.67 to 39517.85) | 12943.13 (3259.40 to 39238.87) |
| Angola | Female | DALY rates | 12.22 (7.17 to 18.70) | 23.15 (14.21 to 34.78) | 25.65 (15.96 to 39.29) | 28.67 (17.80 to 42.44) | 31.16 (19.23 to 48.77) | 33.55 (20.94 to 48.63) | 34.86 (21.42 to 50.86) | 36.51 (22.98 to 52.60) | 37.23 (23.34 to 55.31) | 39.04 (25.12 to 56.95) | 39.87 (26.02 to 58.58) | 40.74 (25.46 to 59.62) | 41.89 (27.35 to 63.43) | 42.06 (27.29 to 60.63) | 41.51 (26.22 to 61.62) | 40.49 (27.73 to 58.55) | 39.49 (25.73 to 57.70) | 38.94 (25.51 to 52.73) | 37.85 (24.59 to 53.96) | 36.71 (24.30 to 52.50) |
| Angola | Female | Prevalence | 1162.81 (247.73 to 3649.27) | 2997.84 (574.83 to 9591.62) | 4295.86 (761.54 to 13920.25) | 5779.24 (976.99 to 18861.17) | 7056.88 (1165.10 to 23108.43) | 7986.13 (1304.79 to 26193.69) | 8648.85 (1404.97 to 28389.59) | 9186.61 (1487.16 to 30173.79) | 9632.02 (1554.48 to 31648.08) | 10037.72 (1618.06 to 32989.10) | 10399.43 (1673.11 to 34186.79) | 10788.82 (1733.78 to 35472.19) | 11228.48 (1802.89 to 36924.19) | 11440.91 (1835.77 to 37626.39) | 11440.94 (1835.73 to 37627.02) | 11440.96 (1834.90 to 37626.39) | 11440.95 (1836.77 to 37626.03) | 11440.89 (1835.86 to 37625.02) | 11440.93 (1835.88 to 37625.76) | 11440.93 (1834.96 to 37624.71) |
| Angola | Male | DALY rates | 8.44 (4.57 to 13.40) | 91.06 (42.21 to 174.92) | 138.51 (73.73 to 238.78) | 210.97 (114.54 to 351.33) | 288.51 (171.31 to 453.93) | 347.70 (214.15 to 532.64) | 394.79 (250.87 to 593.14) | 432.38 (277.11 to 638.84) | 461.31 (303.27 to 686.78) | 487.09 (318.61 to 712.89) | 508.56 (339.38 to 736.11) | 527.56 (346.88 to 750.95) | 548.19 (364.42 to 771.05) | 552.27 (356.78 to 784.55) | 543.39 (366.50 to 778.07) | 532.45 (353.36 to 750.53) | 522.28 (348.22 to 727.53) | 511.78 (351.31 to 702.94) | 501.08 (338.21 to 690.49) | 489.38 (335.01 to 677.93) |
| Angola | Male | Prevalence | 1119.02 (215.79 to 3581.74) | 3532.33 (993.05 to 10225.85) | 5198.28 (1520.82 to 14982.14) | 7259.84 (2293.02 to 20562.15) | 9156.82 (3128.52 to 25466.18) | 10589.52 (3766.13 to 29087.73) | 11625.98 (4270.51 to 31694.67) | 12471.19 (4667.15 to 33789.19) | 13170.51 (5001.13 to 35513.08) | 13804.89 (5298.98 to 37086.99) | 14365.77 (5536.71 to 38492.19) | 14966.66 (5827.80 to 40004.01) | 15635.68 (6110.37 to 41663.71) | 15956.50 (6242.51 to 42479.94) | 15956.05 (6249.54 to 42487.89) | 15955.59 (6245.98 to 42457.83) | 15955.70 (6240.25 to 42484.30) | 15955.94 (6248.38 to 42465.54) | 15955.63 (6254.21 to 42469.93) | 15955.74 (6243.76 to 42476.04) |
| Bangladesh | Both | DALY rates | 10.10 (6.47 to 14.70) | 40.70 (21.58 to 71.60) | 49.60 (26.41 to 89.70) | 61.39 (32.36 to 105.34) | 74.37 (40.76 to 128.79) | 88.11 (49.04 to 150.16) | 99.06 (55.89 to 165.15) | 109.03 (62.88 to 179.09) | 115.78 (68.85 to 189.44) | 121.71 (70.40 to 198.51) | 127.45 (74.41 to 205.21) | 133.41 (78.53 to 212.57) | 139.90 (86.42 to 224.17) | 143.97 (89.81 to 224.83) | 143.29 (85.38 to 225.75) | 143.20 (89.28 to 224.90) | 142.57 (85.98 to 224.34) | 142.40 (86.81 to 216.23) | 140.25 (86.47 to 216.81) | 136.77 (85.73 to 212.31) |
| Bangladesh | Both | Prevalence | 724.67 (631.67 to 825.56) | 1924.71 (1662.22 to 2214.20) | 2714.02 (2346.27 to 3118.16) | 3638.28 (3153.62 to 4170.32) | 4453.11 (3866.75 to 5097.02) | 5071.91 (4403.92 to 5802.97) | 5537.69 (4817.18 to 6337.01) | 5913.32 (5153.41 to 6767.80) | 6227.15 (5431.57 to 7133.06) | 6504.67 (5677.85 to 7453.27) | 6761.62 (5904.38 to 7743.09) | 7041.13 (6148.57 to 8063.59) | 7349.32 (6419.19 to 8417.39) | 7514.12 (6564.65 to 8600.78) | 7533.22 (6573.93 to 8622.70) | 7553.72 (6591.58 to 8642.94) | 7569.23 (6605.96 to 8658.37) | 7592.27 (6620.91 to 8689.15) | 7600.29 (6628.53 to 8697.06) | 7590.89 (6620.13 to 8684.03) |
| Bangladesh | Female | DALY rates | 11.85 (6.96 to 18.47) | 21.37 (12.54 to 34.13) | 22.29 (12.96 to 34.44) | 23.56 (13.62 to 37.90) | 24.67 (14.16 to 38.71) | 25.77 (15.29 to 41.06) | 26.29 (15.60 to 39.80) | 26.89 (16.15 to 40.96) | 27.08 (16.50 to 40.45) | 27.29 (16.50 to 40.61) | 27.66 (16.94 to 40.61) | 27.94 (17.08 to 41.30) | 28.30 (17.85 to 42.39) | 28.17 (17.91 to 41.44) | 27.69 (17.83 to 40.51) | 27.29 (16.99 to 39.95) | 26.59 (16.69 to 38.47) | 25.99 (16.43 to 37.74) | 25.44 (16.24 to 38.04) | 24.94 (15.82 to 36.50) |
| Bangladesh | Female | Prevalence | 739.95 (648.90 to 841.54) | 1771.63 (1537.94 to 2024.51) | 2499.65 (2154.52 to 2863.57) | 3337.76 (2867.46 to 3834.89) | 4054.81 (3475.83 to 4664.13) | 4571.91 (3915.64 to 5262.32) | 4945.81 (4231.76 to 5695.10) | 5244.45 (4486.76 to 6040.43) | 5497.17 (4703.14 to 6331.63) | 5720.57 (4892.00 to 6590.68) | 5928.16 (5067.64 to 6830.81) | 6145.49 (5254.18 to 7082.75) | 6385.81 (5457.43 to 7359.72) | 6507.44 (5561.16 to 7500.21) | 6507.44 (5560.11 to 7500.76) | 6507.46 (5561.46 to 7500.29) | 6507.44 (5560.71 to 7501.28) | 6507.45 (5560.82 to 7501.04) | 6507.46 (5560.16 to 7500.48) | 6507.51 (5561.02 to 7501.08) |
| Bangladesh | Male | DALY rates | 8.44 (4.56 to 13.66) | 58.69 (27.66 to 115.08) | 76.42 (36.59 to 151.83) | 101.29 (49.87 to 191.16) | 127.33 (63.80 to 234.27) | 149.80 (79.51 to 268.79) | 165.50 (87.97 to 284.42) | 180.38 (98.47 to 306.02) | 191.63 (108.36 to 322.30) | 201.95 (114.54 to 339.92) | 212.41 (118.35 to 351.73) | 221.48 (125.58 to 362.01) | 231.88 (139.13 to 377.68) | 237.14 (145.67 to 380.82) | 232.43 (136.98 to 375.98) | 228.57 (139.47 to 363.10) | 225.02 (133.91 to 361.60) | 220.95 (130.04 to 343.50) | 216.29 (130.71 to 339.52) | 212.51 (129.29 to 338.39) |
| Bangladesh | Male | Prevalence | 710.24 (614.21 to 812.09) | 2067.15 (1728.84 to 2551.02) | 2924.59 (2466.42 to 3524.42) | 3955.22 (3355.50 to 4660.80) | 4877.43 (4147.56 to 5658.42) | 5566.71 (4739.23 to 6444.54) | 6078.02 (5169.32 to 7033.25) | 6494.26 (5530.60 to 7490.33) | 6851.40 (5850.27 to 7889.38) | 7171.02 (6123.72 to 8247.84) | 7471.23 (6398.61 to 8586.63) | 7788.88 (6664.85 to 8937.11) | 8143.47 (6976.32 to 9335.31) | 8324.02 (7143.65 to 9533.86) | 8324.30 (7137.95 to 9539.45) | 8324.27 (7140.66 to 9542.64) | 8324.15 (7137.38 to 9534.43) | 8324.31 (7144.47 to 9538.64) | 8324.06 (7141.49 to 9530.96) | 8324.52 (7140.35 to 9544.41) |
| Benin | Both | DALY rates | 9.57 (6.01 to 14.30) | 34.78 (18.07 to 62.67) | 39.53 (21.20 to 69.57) | 44.34 (22.81 to 80.43) | 47.74 (25.57 to 81.59) | 51.54 (28.87 to 87.65) | 55.52 (31.52 to 96.00) | 59.86 (34.02 to 104.22) | 62.62 (35.24 to 108.32) | 65.28 (37.28 to 110.99) | 68.07 (37.94 to 115.05) | 70.37 (38.82 to 116.69) | 71.98 (40.26 to 120.76) | 73.76 (42.09 to 121.98) | 74.45 (43.10 to 120.48) | 73.40 (41.91 to 120.42) | 69.81 (40.79 to 118.17) | 68.15 (39.26 to 112.72) | 57.29 (34.13 to 92.14) | 46.34 (27.95 to 71.90) |
| Benin | Both | Prevalence | 483.74 (119.40 to 2118.87) | 1315.99 (347.92 to 5513.40) | 1809.53 (420.45 to 7900.66) | 2390.82 (499.64 to 10730.78) | 2873.80 (555.83 to 13109.67) | 3235.48 (610.45 to 14842.39) | 3505.94 (663.81 to 16085.17) | 3734.50 (713.49 to 17107.24) | 3919.67 (747.44 to 17964.07) | 4079.99 (776.47 to 18694.11) | 4241.92 (813.91 to 19404.40) | 4400.25 (843.23 to 20130.55) | 4582.29 (879.66 to 20963.17) | 4680.91 (911.76 to 21362.12) | 4697.56 (926.46 to 21374.39) | 4700.82 (930.56 to 21379.45) | 4680.21 (908.55 to 21362.06) | 4679.72 (908.95 to 21361.31) | 4584.18 (815.47 to 21278.80) | 4493.92 (744.54 to 21202.72) |
| Benin | Female | DALY rates | 11.17 (6.65 to 17.56) | 20.32 (12.05 to 32.36) | 20.94 (12.56 to 32.60) | 21.35 (12.35 to 33.91) | 22.19 (13.23 to 32.75) | 22.53 (13.44 to 33.30) | 22.96 (14.47 to 34.96) | 23.00 (13.37 to 35.08) | 23.28 (14.04 to 36.14) | 23.47 (14.30 to 36.32) | 23.59 (13.26 to 35.32) | 23.62 (13.93 to 34.80) | 23.60 (13.98 to 35.71) | 23.62 (14.84 to 35.23) | 23.26 (14.83 to 35.31) | 22.68 (14.06 to 34.83) | 22.52 (14.42 to 32.65) | 21.98 (13.14 to 32.50) | 21.80 (13.69 to 31.76) | 20.81 (12.59 to 30.71) |
| Benin | Female | Prevalence | 499.47 (133.16 to 2135.21) | 1203.50 (258.67 to 5424.83) | 1663.35 (295.32 to 7783.43) | 2209.50 (337.45 to 10581.76) | 2670.33 (372.85 to 12941.58) | 3004.07 (397.63 to 14651.41) | 3241.66 (416.12 to 15865.76) | 3435.03 (430.21 to 16855.84) | 3598.93 (442.32 to 17693.89) | 3738.15 (452.81 to 18405.71) | 3873.02 (463.51 to 19095.39) | 4011.53 (473.73 to 19804.45) | 4170.14 (486.34 to 20614.88) | 4243.63 (491.34 to 20991.03) | 4243.60 (491.67 to 20989.89) | 4243.61 (490.92 to 20990.88) | 4243.66 (491.76 to 20990.04) | 4243.64 (492.26 to 20991.11) | 4243.66 (490.92 to 20989.56) | 4243.63 (491.88 to 20989.76) |
| Benin | Male | DALY rates | 8.05 (4.54 to 12.63) | 48.21 (21.14 to 98.72) | 56.67 (26.26 to 111.96) | 68.63 (30.50 to 135.77) | 80.61 (37.91 to 154.64) | 90.41 (45.85 to 165.75) | 96.92 (49.36 to 178.17) | 103.17 (53.30 to 193.58) | 108.76 (54.86 to 196.83) | 113.63 (57.88 to 204.84) | 117.37 (58.16 to 210.27) | 122.32 (61.84 to 215.42) | 125.93 (64.98 to 220.15) | 126.37 (65.79 to 220.39) | 124.32 (66.70 to 211.41) | 122.10 (64.68 to 207.54) | 119.59 (65.65 to 209.90) | 116.84 (62.59 to 201.82) | 115.18 (62.21 to 196.70) | 112.23 (59.22 to 192.74) |
| Benin | Male | Prevalence | 468.75 (102.53 to 2103.29) | 1420.52 (402.87 to 5596.12) | 1944.40 (496.15 to 8008.82) | 2582.49 (630.24 to 10888.31) | 3135.66 (759.20 to 13326.01) | 3545.52 (858.74 to 15098.25) | 3841.93 (933.40 to 16364.13) | 4086.48 (996.88 to 17402.72) | 4295.84 (1049.76 to 18280.95) | 4475.34 (1099.23 to 19027.66) | 4650.79 (1146.77 to 19746.88) | 4832.21 (1195.11 to 20492.92) | 5041.86 (1255.40 to 21351.55) | 5139.69 (1285.55 to 21751.45) | 5139.89 (1285.48 to 21749.06) | 5139.81 (1284.68 to 21752.54) | 5139.79 (1283.95 to 21753.70) | 5139.69 (1284.38 to 21751.79) | 5139.59 (1284.46 to 21750.58) | 5139.70 (1283.53 to 21752.21) |
| Brazil | Both | DALY rates | 1.98 (1.31 to 2.86) | 4.33 (2.76 to 6.50) | 4.65 (2.92 to 7.01) | 4.95 (3.08 to 7.67) | 4.86 (2.98 to 7.43) | 4.62 (2.89 to 7.15) | 4.58 (2.94 to 6.96) | 4.68 (2.88 to 7.12) | 5.00 (3.10 to 7.45) | 5.34 (3.43 to 8.06) | 5.44 (3.54 to 8.23) | 5.43 (3.49 to 8.25) | 5.74 (3.62 to 8.68) | 6.25 (3.89 to 9.57) | 6.48 (4.14 to 9.91) | 6.54 (4.23 to 9.73) | 6.12 (3.97 to 9.06) | 6.44 (4.05 to 9.58) | 5.88 (3.95 to 8.27) | 7.47 (5.10 to 10.46) |
| Brazil | Both | Prevalence | 46.18 (36.55 to 77.82) | 103.20 (78.83 to 174.65) | 135.81 (100.37 to 242.35) | 173.44 (125.46 to 321.05) | 188.98 (135.35 to 356.40) | 191.02 (136.21 to 363.43) | 195.44 (138.93 to 373.55) | 207.00 (146.65 to 397.30) | 225.21 (159.33 to 433.33) | 246.71 (174.23 to 475.89) | 256.94 (181.17 to 497.00) | 264.80 (186.53 to 513.84) | 286.08 (201.36 to 555.59) | 314.62 (221.41 to 611.13) | 332.40 (233.91 to 645.56) | 332.18 (234.46 to 643.12) | 328.89 (231.37 to 639.24) | 369.32 (258.62 to 721.79) | 325.50 (229.70 to 631.44) | 323.82 (237.30 to 601.63) |
| Brazil | Female | DALY rates | 2.34 (1.47 to 3.44) | 3.96 (2.50 to 5.80) | 3.96 (2.58 to 5.83) | 3.84 (2.50 to 5.85) | 3.55 (2.23 to 5.19) | 3.17 (2.08 to 4.63) | 3.05 (2.03 to 4.34) | 3.02 (1.98 to 4.47) | 3.11 (2.04 to 4.58) | 3.23 (2.12 to 4.73) | 3.18 (2.08 to 4.58) | 3.04 (2.01 to 4.46) | 3.03 (2.04 to 4.41) | 3.18 (2.11 to 4.61) | 3.28 (2.17 to 4.66) | 3.41 (2.28 to 4.82) | 3.33 (2.23 to 4.72) | 3.45 (2.30 to 4.91) | 3.98 (2.66 to 5.80) | 6.18 (4.13 to 9.07) |
| Brazil | Female | Prevalence | 49.82 (40.11 to 81.07) | 101.78 (79.39 to 175.97) | 131.82 (98.94 to 242.21) | 166.54 (120.85 to 320.27) | 182.41 (129.93 to 359.32) | 184.96 (130.46 to 368.94) | 190.69 (133.82 to 382.97) | 202.66 (141.48 to 409.68) | 221.58 (153.99 to 450.14) | 241.89 (167.63 to 493.29) | 251.46 (173.63 to 514.80) | 256.13 (176.29 to 526.31) | 269.94 (185.41 to 556.32) | 291.39 (199.81 to 601.59) | 302.38 (207.45 to 624.00) | 296.58 (204.37 to 608.84) | 302.69 (208.36 to 622.22) | 338.71 (232.36 to 698.83) | 319.14 (222.25 to 646.71) | 311.74 (227.37 to 595.53) |
| Brazil | Male | DALY rates | 1.62 (1.02 to 2.44) | 4.68 (2.66 to 7.94) | 5.32 (3.03 to 8.82) | 6.08 (3.46 to 10.43) | 6.21 (3.51 to 10.38) | 6.13 (3.56 to 10.29) | 6.19 (3.58 to 10.44) | 6.43 (3.64 to 10.69) | 6.94 (3.93 to 11.17) | 7.52 (4.37 to 12.26) | 7.82 (4.59 to 12.62) | 8.03 (4.69 to 13.05) | 8.74 (5.07 to 14.37) | 9.66 (5.61 to 15.89) | 10.20 (5.83 to 16.29) | 10.37 (6.15 to 16.63) | 9.92 (5.88 to 15.90) | 11.08 (6.56 to 18.03) | 9.37 (5.89 to 14.67) | 10.12 (6.44 to 15.07) |
| Brazil | Male | Prevalence | 42.67 (32.96 to 74.24) | 104.57 (77.97 to 175.01) | 139.76 (102.28 to 245.72) | 180.44 (129.96 to 325.73) | 195.73 (140.45 to 357.53) | 197.35 (141.56 to 361.90) | 200.40 (143.74 to 368.03) | 211.55 (151.73 to 388.93) | 228.94 (164.32 to 421.00) | 251.68 (180.61 to 463.30) | 262.68 (188.55 to 484.00) | 274.25 (196.75 to 506.13) | 303.95 (218.12 to 560.89) | 340.51 (244.62 to 628.32) | 367.23 (263.60 to 677.80) | 375.71 (270.18 to 692.54) | 364.64 (262.48 to 670.90) | 416.73 (299.53 to 767.91) | 337.21 (245.36 to 613.00) | 348.55 (256.66 to 623.89) |
| Brunei Darussalam | Both | DALY rates | 9.77 (6.20 to 15.06) | 31.00 (16.62 to 57.35) | 33.73 (17.62 to 62.27) | 36.16 (19.07 to 63.83) | 39.53 (21.52 to 72.52) | 42.05 (23.01 to 77.69) | 43.77 (23.16 to 78.73) | 45.41 (23.55 to 83.75) | 47.00 (24.79 to 85.72) | 47.23 (24.12 to 85.36) | 46.98 (25.77 to 84.98) | 46.70 (25.33 to 84.61) | 47.00 (24.52 to 81.56) | 47.78 (25.52 to 84.29) | 46.55 (24.87 to 83.78) | 43.68 (24.31 to 75.87) | 42.55 (22.82 to 76.13) | 43.55 (24.19 to 75.77) | 24.30 (15.10 to 37.50) | 19.73 (12.23 to 29.84) |
| Brunei Darussalam | Both | Prevalence | 324.08 (93.70 to 2162.11) | 842.71 (212.94 to 5358.75) | 1121.94 (228.53 to 7719.67) | 1448.69 (249.27 to 10462.81) | 1726.36 (268.02 to 12766.23) | 1932.76 (282.65 to 14446.29) | 2079.71 (293.97 to 15628.31) | 2202.37 (305.17 to 16596.48) | 2304.61 (314.01 to 17406.56) | 2388.62 (319.20 to 18137.14) | 2464.08 (322.68 to 18790.80) | 2542.24 (326.84 to 19485.32) | 2636.12 (334.11 to 20261.46) | 2694.92 (343.07 to 20670.46) | 2690.58 (340.74 to 20667.91) | 2676.44 (334.44 to 20656.70) | 2673.08 (332.09 to 20654.64) | 2687.59 (339.69 to 20664.98) | 2509.21 (251.23 to 20515.94) | 2471.39 (225.92 to 20484.44) |
| Brunei Darussalam | Female | DALY rates | 11.49 (6.89 to 18.39) | 19.80 (12.34 to 31.62) | 19.97 (11.18 to 30.65) | 20.41 (12.38 to 32.15) | 20.83 (12.37 to 33.34) | 20.92 (12.09 to 32.23) | 21.00 (11.91 to 32.22) | 21.03 (12.63 to 31.27) | 21.48 (11.98 to 32.91) | 21.37 (12.16 to 32.23) | 21.22 (12.50 to 32.96) | 21.35 (12.60 to 33.10) | 21.22 (12.39 to 32.09) | 20.67 (12.55 to 31.95) | 20.65 (12.48 to 31.16) | 20.05 (12.09 to 30.69) | 19.67 (11.95 to 29.89) | 19.23 (11.74 to 29.15) | 19.35 (11.31 to 29.21) | 18.77 (11.47 to 28.61) |
| Brunei Darussalam | Female | Prevalence | 340.09 (108.23 to 2171.43) | 756.66 (185.67 to 5296.54) | 1020.11 (191.03 to 7643.05) | 1326.25 (196.94 to 10368.16) | 1582.90 (202.07 to 12653.04) | 1769.65 (205.68 to 14314.88) | 1901.04 (208.26 to 15482.43) | 2008.30 (210.45 to 16437.46) | 2098.13 (212.03 to 17236.79) | 2179.82 (213.91 to 17964.74) | 2252.98 (214.62 to 18615.26) | 2330.93 (216.77 to 19309.39) | 2417.47 (218.09 to 20078.87) | 2462.17 (219.42 to 20476.55) | 2462.15 (218.95 to 20476.49) | 2462.19 (219.03 to 20476.46) | 2462.18 (218.71 to 20477.04) | 2462.18 (218.74 to 20476.82) | 2462.19 (219.21 to 20476.54) | 2462.19 (219.00 to 20476.68) |
| Brunei Darussalam | Male | DALY rates | 8.19 (4.38 to 13.19) | 41.52 (18.47 to 83.85) | 46.68 (20.26 to 97.75) | 50.99 (22.44 to 102.32) | 56.74 (25.20 to 115.30) | 60.48 (29.02 to 122.59) | 62.75 (28.76 to 121.84) | 64.59 (29.50 to 128.82) | 66.29 (30.75 to 131.06) | 68.07 (30.99 to 134.72) | 68.90 (33.95 to 134.93) | 69.97 (32.16 to 136.60) | 71.00 (33.91 to 130.95) | 70.90 (34.05 to 137.41) | 69.53 (32.35 to 133.92) | 67.60 (33.34 to 126.11) | 66.44 (31.13 to 131.61) | 65.74 (32.27 to 124.84) | 64.75 (32.57 to 119.09) | 63.60 (31.31 to 117.81) |
| Brunei Darussalam | Male | Prevalence | 309.34 (77.54 to 2153.53) | 923.57 (232.34 to 5440.28) | 1217.69 (255.72 to 7791.71) | 1563.96 (289.15 to 10551.91) | 1858.31 (318.91 to 12870.33) | 2075.00 (342.59 to 14560.90) | 2228.68 (357.16 to 15749.95) | 2355.05 (375.72 to 16721.58) | 2460.65 (387.21 to 17534.87) | 2556.89 (398.48 to 18276.08) | 2643.67 (405.47 to 18940.14) | 2736.27 (418.83 to 19646.85) | 2839.73 (432.19 to 20431.49) | 2893.33 (438.41 to 20835.77) | 2893.27 (438.27 to 20837.75) | 2893.39 (438.66 to 20839.20) | 2893.30 (438.56 to 20840.09) | 2893.31 (435.99 to 20836.72) | 2893.28 (437.63 to 20837.73) | 2893.30 (438.26 to 20840.56) |
| Burkina Faso | Both | DALY rates | 13.68 (9.02 to 20.17) | 205.65 (129.97 to 305.72) | 342.75 (225.21 to 485.25) | 431.90 (284.83 to 605.53) | 437.12 (295.68 to 603.04) | 434.73 (295.47 to 599.31) | 447.45 (307.29 to 613.12) | 466.63 (321.76 to 654.16) | 484.53 (331.51 to 669.98) | 501.47 (348.16 to 682.67) | 520.57 (359.71 to 710.93) | 538.20 (367.06 to 740.77) | 555.08 (384.35 to 771.14) | 553.05 (385.12 to 757.16) | 542.00 (379.69 to 739.68) | 514.63 (362.43 to 697.63) | 496.69 (357.29 to 670.87) | 486.42 (345.68 to 648.70) | 440.18 (315.51 to 586.65) | 380.75 (273.90 to 504.52) |
| Burkina Faso | Both | Prevalence | 3166.07 (743.97 to 7430.20) | 9662.32 (3245.60 to 20701.10) | 14375.79 (5067.16 to 30547.07) | 19294.80 (6685.66 to 41292.64) | 22946.78 (7524.27 to 50003.44) | 25565.94 (8091.27 to 56241.21) | 27564.53 (8622.10 to 60809.92) | 29244.74 (9119.77 to 64548.05) | 30684.81 (9547.76 to 67741.52) | 31942.94 (9952.19 to 70504.48) | 33174.88 (10362.53 to 73182.92) | 34438.01 (10786.76 to 75955.55) | 35855.28 (11266.20 to 79091.52) | 36503.54 (11457.30 to 80574.02) | 36498.60 (11451.01 to 80579.91) | 36375.04 (11324.18 to 80466.59) | 36281.08 (11228.31 to 80352.22) | 36284.76 (11216.58 to 80359.61) | 35932.93 (10881.65 to 80022.70) | 35476.98 (10428.74 to 79573.34) |
| Burkina Faso | Female | DALY rates | 16.12 (9.99 to 23.68) | 35.01 (21.49 to 51.06) | 47.09 (29.21 to 68.21) | 65.48 (43.74 to 95.19) | 86.64 (57.38 to 127.67) | 105.17 (69.51 to 153.51) | 122.00 (81.72 to 171.37) | 135.90 (90.67 to 195.33) | 149.75 (98.91 to 214.71) | 162.45 (108.97 to 236.65) | 174.86 (114.24 to 250.53) | 189.19 (122.86 to 278.74) | 204.92 (135.64 to 294.46) | 212.18 (140.43 to 306.94) | 208.49 (136.30 to 303.98) | 204.10 (138.72 to 290.17) | 200.86 (139.63 to 284.05) | 197.31 (134.38 to 281.61) | 192.40 (132.71 to 268.70) | 187.06 (126.58 to 262.93) |
| Burkina Faso | Female | Prevalence | 3191.32 (770.49 to 7452.88) | 8262.71 (1940.23 to 19393.02) | 11952.76 (2775.35 to 28112.63) | 16298.85 (3802.31 to 38310.54) | 20079.31 (4738.68 to 47101.89) | 22873.69 (5466.78 to 53539.07) | 24892.00 (6009.20 to 58155.48) | 26522.86 (6449.11 to 61861.14) | 27921.62 (6845.53 to 65020.27) | 29133.10 (7195.68 to 67739.98) | 30297.76 (7536.24 to 70351.94) | 31515.42 (7916.27 to 73073.96) | 32908.82 (8368.72 to 76189.32) | 33587.62 (8583.76 to 77700.06) | 33587.45 (8588.27 to 77708.58) | 33587.52 (8583.26 to 77715.31) | 33587.18 (8583.23 to 77699.22) | 33587.37 (8583.23 to 77697.90) | 33587.38 (8586.79 to 77704.50) | 33587.35 (8583.11 to 77708.65) |
| Burkina Faso | Male | DALY rates | 11.32 (6.41 to 17.65) | 370.97 (229.86 to 561.62) | 627.83 (410.12 to 896.76) | 812.13 (533.19 to 1135.07) | 858.17 (579.71 to 1198.35) | 873.36 (598.03 to 1203.01) | 881.60 (594.60 to 1214.56) | 889.35 (606.40 to 1253.11) | 896.49 (612.80 to 1256.56) | 900.99 (618.58 to 1226.64) | 905.56 (610.32 to 1240.09) | 909.82 (611.38 to 1246.71) | 915.21 (633.92 to 1273.88) | 907.40 (631.65 to 1246.89) | 889.84 (617.50 to 1213.59) | 866.71 (601.67 to 1171.06) | 853.96 (608.60 to 1152.54) | 834.70 (590.76 to 1114.02) | 820.70 (575.34 to 1098.44) | 796.69 (575.17 to 1057.34) |
| Burkina Faso | Male | Prevalence | 3141.60 (718.24 to 7408.22) | 11018.24 (4672.22 to 22264.52) | 16712.07 (7546.45 to 33006.44) | 22403.61 (9773.70 to 44428.64) | 26391.63 (10933.94 to 53340.69) | 29149.13 (11682.56 to 59683.02) | 31129.71 (12211.93 to 64304.58) | 32723.59 (12650.53 to 67979.97) | 34085.03 (12982.96 to 71094.33) | 35254.26 (13312.69 to 73766.00) | 36379.02 (13653.64 to 76339.25) | 37549.97 (14013.58 to 79026.02) | 38885.65 (14442.92 to 82078.62) | 39534.68 (14649.57 to 83562.92) | 39534.85 (14640.00 to 83577.53) | 39535.47 (14646.21 to 83588.52) | 39534.36 (14652.67 to 83556.71) | 39534.14 (14620.83 to 83568.26) | 39535.03 (14645.94 to 83584.75) | 39534.88 (14639.34 to 83580.80) |
| Cambodia | Both | DALY rates | 9.76 (5.98 to 14.67) | 36.18 (19.03 to 62.90) | 42.37 (23.34 to 75.63) | 49.93 (26.99 to 87.08) | 56.78 (31.04 to 97.88) | 61.72 (33.51 to 103.97) | 62.75 (35.99 to 103.31) | 68.93 (39.25 to 118.38) | 74.07 (42.19 to 123.45) | 76.78 (44.13 to 128.30) | 78.46 (45.18 to 130.72) | 81.67 (47.09 to 135.54) | 84.48 (48.83 to 136.89) | 84.40 (50.20 to 137.16) | 79.10 (45.38 to 128.16) | 75.58 (45.53 to 120.71) | 72.23 (44.40 to 112.01) | 69.35 (41.67 to 110.34) | 65.32 (40.09 to 101.73) | 54.94 (34.27 to 85.97) |
| Cambodia | Both | Prevalence | 562.94 (102.80 to 3332.25) | 1519.61 (289.83 to 8746.26) | 2104.59 (342.50 to 12525.91) | 2813.18 (416.01 to 17055.41) | 3409.10 (481.60 to 20820.23) | 3841.57 (531.44 to 23549.73) | 4127.52 (550.12 to 25469.35) | 4404.29 (599.68 to 27096.28) | 4639.22 (645.48 to 28458.85) | 4832.34 (671.11 to 29645.78) | 5005.70 (693.64 to 30737.12) | 5198.88 (729.01 to 31896.21) | 5416.14 (766.76 to 33197.03) | 5521.19 (782.79 to 33821.90) | 5488.72 (755.50 to 33774.57) | 5467.84 (737.20 to 33743.07) | 5451.13 (723.19 to 33718.26) | 5440.20 (714.99 to 33705.38) | 5414.11 (694.47 to 33669.44) | 5327.23 (630.31 to 33544.30) |
| Cambodia | Female | DALY rates | 11.56 (6.49 to 18.16) | 20.48 (12.15 to 32.66) | 21.19 (12.15 to 33.32) | 22.41 (13.47 to 34.19) | 23.13 (13.71 to 34.54) | 23.75 (14.47 to 35.01) | 24.59 (14.91 to 39.11) | 24.21 (15.10 to 36.87) | 24.50 (14.98 to 37.67) | 24.91 (14.58 to 38.22) | 25.18 (14.95 to 38.60) | 25.52 (15.85 to 38.48) | 25.51 (15.69 to 39.04) | 25.41 (15.56 to 38.20) | 24.71 (15.40 to 35.65) | 24.39 (15.37 to 35.42) | 23.62 (14.68 to 35.18) | 23.08 (14.12 to 33.39) | 22.68 (13.89 to 33.57) | 22.06 (14.16 to 32.63) |
| Cambodia | Female | Prevalence | 580.61 (119.13 to 3352.61) | 1396.97 (217.09 to 8482.60) | 1942.24 (234.23 to 12205.88) | 2594.30 (255.07 to 16656.64) | 3137.44 (271.87 to 20360.36) | 3531.75 (284.01 to 23048.83) | 3814.82 (293.37 to 24977.02) | 4044.21 (300.18 to 26538.55) | 4236.02 (306.52 to 27845.93) | 4407.53 (311.65 to 29013.15) | 4565.17 (317.52 to 30085.78) | 4729.15 (323.27 to 31201.77) | 4915.27 (328.60 to 32467.54) | 5005.56 (331.94 to 33081.49) | 5005.56 (332.20 to 33082.34) | 5005.59 (332.00 to 33081.57) | 5005.59 (332.14 to 33081.91) | 5005.58 (332.86 to 33081.56) | 5005.60 (332.94 to 33081.88) | 5005.55 (331.41 to 33081.93) |
| Cambodia | Male | DALY rates | 8.02 (4.45 to 12.72) | 51.61 (23.10 to 101.80) | 63.83 (30.12 to 122.86) | 79.10 (36.23 to 147.47) | 94.54 (45.60 to 173.14) | 107.24 (52.67 to 192.31) | 117.80 (60.67 to 210.44) | 127.95 (65.69 to 233.14) | 134.92 (70.36 to 238.16) | 141.74 (74.23 to 251.91) | 147.68 (78.92 to 262.63) | 153.83 (81.74 to 268.15) | 160.18 (87.51 to 272.14) | 160.25 (88.73 to 268.51) | 157.39 (85.11 to 268.84) | 154.88 (88.08 to 264.96) | 152.20 (85.62 to 247.31) | 148.54 (83.97 to 250.02) | 145.70 (82.04 to 248.55) | 142.56 (80.92 to 239.96) |
| Cambodia | Male | Prevalence | 545.88 (85.91 to 3312.59) | 1640.17 (347.05 to 9008.24) | 2269.04 (435.28 to 12854.13) | 3045.10 (559.16 to 17483.57) | 3713.97 (681.30 to 21342.87) | 4213.05 (793.06 to 24157.36) | 4578.58 (875.10 to 26186.99) | 4879.51 (951.51 to 27840.18) | 5134.22 (1015.25 to 29219.56) | 5364.43 (1080.87 to 30447.35) | 5578.12 (1139.16 to 31592.23) | 5802.54 (1205.33 to 32800.03) | 6059.12 (1278.70 to 34145.13) | 6184.17 (1313.31 to 34784.87) | 6184.13 (1312.75 to 34781.40) | 6184.01 (1310.18 to 34777.94) | 6184.09 (1314.81 to 34774.94) | 6184.24 (1312.85 to 34784.33) | 6184.16 (1316.47 to 34787.91) | 6184.13 (1313.44 to 34787.10) |
| Cameroon | Both | DALY rates | 9.79 (5.81 to 14.59) | 40.17 (21.22 to 70.10) | 49.19 (26.69 to 88.66) | 60.75 (34.27 to 104.10) | 70.77 (38.84 to 120.19) | 80.58 (44.84 to 134.16) | 91.45 (53.38 to 149.98) | 99.90 (55.91 to 163.75) | 106.69 (60.95 to 172.56) | 111.82 (64.13 to 181.49) | 117.21 (67.59 to 185.05) | 123.21 (74.05 to 198.27) | 130.59 (79.43 to 207.51) | 128.81 (81.93 to 198.34) | 126.09 (76.37 to 196.19) | 123.49 (76.85 to 187.39) | 121.93 (76.06 to 189.92) | 114.81 (71.49 to 174.96) | 104.78 (64.47 to 156.63) | 86.19 (54.04 to 127.12) |
| Cameroon | Both | Prevalence | 693.72 (207.58 to 1852.34) | 1907.73 (643.43 to 4904.90) | 2695.05 (863.66 to 7075.41) | 3610.27 (1130.82 to 9584.91) | 4393.68 (1362.78 to 11728.84) | 4988.15 (1544.41 to 13312.58) | 5439.41 (1697.01 to 14461.40) | 5804.57 (1819.18 to 15390.11) | 6118.70 (1925.61 to 16185.13) | 6376.02 (1991.03 to 16851.63) | 6627.62 (2070.06 to 17488.98) | 6897.80 (2166.99 to 18160.56) | 7216.81 (2274.61 to 18938.78) | 7335.26 (2293.56 to 19277.26) | 7335.63 (2293.71 to 19277.85) | 7333.30 (2290.55 to 19273.10) | 7329.16 (2287.02 to 19268.89) | 7292.48 (2251.46 to 19228.59) | 7213.16 (2176.44 to 19147.70) | 7056.34 (2033.34 to 18987.23) |
| Cameroon | Female | DALY rates | 11.52 (5.92 to 18.05) | 21.10 (12.35 to 31.95) | 22.03 (13.08 to 32.93) | 23.50 (13.69 to 34.99) | 24.64 (15.12 to 37.35) | 25.35 (15.15 to 38.26) | 26.21 (15.25 to 39.26) | 26.60 (16.00 to 39.11) | 26.95 (17.05 to 39.48) | 27.54 (17.33 to 40.98) | 27.88 (17.61 to 40.61) | 27.89 (17.40 to 42.33) | 28.39 (18.26 to 41.67) | 27.86 (17.16 to 41.66) | 27.40 (17.10 to 39.67) | 27.14 (17.55 to 40.17) | 26.90 (17.82 to 39.11) | 26.28 (17.02 to 37.45) | 25.61 (16.04 to 37.72) | 24.92 (15.59 to 36.28) |
| Cameroon | Female | Prevalence | 710.89 (223.33 to 1868.77) | 1757.65 (496.18 to 4760.12) | 2479.78 (641.61 to 6856.85) | 3311.10 (808.26 to 9269.36) | 4020.96 (952.39 to 11327.45) | 4539.52 (1057.81 to 12830.41) | 4907.82 (1133.43 to 13897.31) | 5204.39 (1193.67 to 14755.32) | 5458.80 (1245.72 to 15492.24) | 5676.30 (1290.87 to 16120.96) | 5881.03 (1332.54 to 16713.88) | 6092.77 (1376.28 to 17325.43) | 6340.62 (1426.05 to 18042.20) | 6457.55 (1451.60 to 18379.84) | 6457.56 (1451.82 to 18380.44) | 6457.53 (1451.07 to 18380.36) | 6457.59 (1451.50 to 18379.77) | 6457.52 (1451.26 to 18379.96) | 6457.57 (1450.42 to 18380.54) | 6457.54 (1452.17 to 18380.98) |
| Cameroon | Male | DALY rates | 8.12 (4.38 to 13.14) | 58.45 (26.36 to 114.60) | 75.27 (35.56 to 147.48) | 99.75 (49.33 to 182.60) | 125.46 (64.09 to 227.98) | 146.87 (73.63 to 257.54) | 163.89 (88.02 to 278.54) | 177.83 (95.98 to 302.97) | 189.26 (101.61 to 315.92) | 200.62 (113.04 to 339.44) | 210.68 (115.68 to 341.23) | 220.28 (128.58 to 361.38) | 231.64 (137.49 to 377.96) | 234.81 (141.65 to 369.50) | 229.63 (133.55 to 371.99) | 225.09 (136.84 to 351.15) | 223.06 (134.18 to 358.63) | 217.07 (130.10 to 342.38) | 214.11 (127.38 to 337.72) | 209.05 (124.53 to 322.64) |
| Cameroon | Male | Prevalence | 677.19 (191.17 to 1836.53) | 2051.59 (750.97 to 5064.16) | 2901.72 (1027.04 to 7291.95) | 3923.53 (1382.50 to 9916.69) | 4835.68 (1731.40 to 12184.23) | 5526.49 (2011.09 to 13835.74) | 6029.67 (2212.49 to 15030.08) | 6442.73 (2391.10 to 16009.58) | 6802.01 (2557.22 to 16846.91) | 7113.11 (2698.60 to 17591.90) | 7408.89 (2835.49 to 18260.75) | 7717.64 (2974.80 to 18968.23) | 8083.12 (3131.71 to 19790.86) | 8256.91 (3209.43 to 20171.27) | 8256.83 (3210.75 to 20165.13) | 8256.73 (3207.10 to 20173.84) | 8256.84 (3209.23 to 20170.56) | 8256.91 (3211.74 to 20170.54) | 8256.54 (3207.78 to 20167.51) | 8256.97 (3207.28 to 20164.56) |
| Central African Republic | Both | DALY rates | 14.03 (9.03 to 21.29) | 225.79 (142.56 to 336.90) | 368.76 (239.96 to 519.72) | 446.69 (299.50 to 615.70) | 462.72 (315.01 to 634.48) | 480.56 (329.53 to 664.15) | 502.15 (339.06 to 679.61) | 513.33 (355.04 to 708.05) | 523.27 (359.79 to 717.64) | 525.48 (363.86 to 730.17) | 531.08 (371.16 to 732.51) | 549.53 (378.32 to 745.62) | 568.28 (394.57 to 783.17) | 573.50 (399.93 to 791.34) | 534.80 (372.89 to 718.40) | 494.96 (352.07 to 680.28) | 492.71 (350.39 to 665.72) | 409.75 (291.59 to 547.24) | 376.04 (267.92 to 509.28) | 341.51 (244.81 to 462.91) |
| Central African Republic | Both | Prevalence | 3348.15 (926.02 to 8131.99) | 10341.62 (3862.29 to 22891.39) | 15396.41 (5957.91 to 33659.66) | 20548.66 (7773.76 to 45462.44) | 24506.78 (8954.60 to 55080.57) | 27468.52 (9821.18 to 62164.33) | 29650.47 (10477.02 to 67274.10) | 31389.70 (10998.23 to 71393.92) | 32852.66 (11438.02 to 74866.63) | 34089.94 (11766.72 to 77894.46) | 35278.35 (12115.79 to 80744.31) | 36599.41 (12603.60 to 83793.38) | 38115.93 (13199.40 to 87296.69) | 38851.78 (13469.23 to 88992.77) | 38606.58 (13234.66 to 88746.69) | 38340.06 (12982.59 to 88484.03) | 38416.43 (13045.64 to 88556.28) | 37740.80 (12389.63 to 87869.77) | 37494.48 (12147.82 to 87630.15) | 37249.92 (11907.41 to 87378.78) |
| Central African Republic | Female | DALY rates | 16.50 (10.16 to 25.25) | 36.60 (22.06 to 55.90) | 50.47 (32.42 to 77.99) | 71.31 (46.27 to 103.19) | 95.55 (63.24 to 134.16) | 117.70 (78.52 to 175.01) | 137.93 (88.13 to 198.31) | 153.79 (102.77 to 223.25) | 170.31 (110.63 to 253.95) | 185.78 (121.37 to 272.92) | 201.50 (136.65 to 290.24) | 219.09 (146.17 to 319.89) | 238.30 (158.63 to 341.59) | 246.99 (163.76 to 359.14) | 242.24 (161.57 to 354.06) | 237.11 (158.94 to 336.25) | 231.42 (157.14 to 325.70) | 227.51 (155.64 to 319.67) | 222.36 (151.89 to 311.22) | 216.56 (150.23 to 301.81) |
| Central African Republic | Female | Prevalence | 3378.36 (948.20 to 8173.95) | 8797.25 (2420.93 to 21378.57) | 12790.64 (3492.03 to 31131.33) | 17466.41 (4781.00 to 42462.97) | 21494.71 (5933.26 to 52134.16) | 24473.90 (6810.54 to 59210.43) | 26638.38 (7468.65 to 64305.63) | 28403.09 (8019.98 to 68436.07) | 29914.05 (8519.88 to 71951.41) | 31248.69 (8975.25 to 75064.70) | 32502.48 (9412.74 to 77981.82) | 33794.47 (9880.93 to 80993.86) | 35293.36 (10432.71 to 84469.56) | 36015.93 (10695.14 to 86148.20) | 36016.08 (10704.69 to 86144.16) | 36015.77 (10705.52 to 86145.43) | 36015.32 (10699.36 to 86146.69) | 36015.91 (10702.78 to 86143.00) | 36016.14 (10703.35 to 86144.05) | 36015.57 (10702.06 to 86142.14) |
| Central African Republic | Male | DALY rates | 11.62 (6.72 to 18.91) | 411.10 (257.96 to 618.81) | 676.73 (439.16 to 961.73) | 830.90 (562.23 to 1150.43) | 863.11 (589.30 to 1191.50) | 874.57 (596.93 to 1221.93) | 887.42 (598.58 to 1205.02) | 893.33 (613.84 to 1236.27) | 901.67 (615.34 to 1247.66) | 906.90 (625.56 to 1247.06) | 912.24 (631.40 to 1266.49) | 918.21 (634.16 to 1245.06) | 924.45 (639.18 to 1257.11) | 918.78 (642.97 to 1263.65) | 901.14 (622.96 to 1210.66) | 884.45 (621.06 to 1208.56) | 866.27 (609.92 to 1170.06) | 843.97 (599.84 to 1115.03) | 828.92 (591.66 to 1086.82) | 807.20 (577.37 to 1076.07) |
| Central African Republic | Male | Prevalence | 3318.65 (901.61 to 8091.00) | 11854.38 (5316.00 to 24373.25) | 17917.61 (8358.81 to 36105.04) | 23703.56 (10802.11 to 48483.23) | 27791.35 (12066.60 to 58359.52) | 30720.24 (13061.08 to 65439.01) | 32836.75 (13658.77 to 70475.63) | 34546.25 (14131.76 to 74620.58) | 36002.98 (14538.74 to 78059.57) | 37280.28 (14888.61 to 81126.72) | 38488.52 (15227.41 to 84011.42) | 39728.94 (15633.80 to 87011.39) | 41162.55 (16097.69 to 90407.80) | 41850.61 (16322.65 to 92089.00) | 41850.42 (16349.09 to 92069.25) | 41850.99 (16355.74 to 92062.61) | 41849.27 (16329.69 to 92034.14) | 41850.77 (16345.80 to 92041.02) | 41850.99 (16361.24 to 92056.73) | 41850.27 (16326.76 to 92049.63) |
| Chad | Both | DALY rates | 9.36 (5.95 to 14.28) | 37.05 (20.47 to 68.59) | 44.24 (23.83 to 79.65) | 53.01 (28.40 to 92.70) | 61.54 (32.60 to 102.40) | 68.87 (37.97 to 118.21) | 73.69 (41.68 to 123.38) | 78.34 (44.22 to 131.44) | 83.07 (46.98 to 134.58) | 87.07 (50.56 to 141.24) | 91.21 (52.08 to 153.76) | 97.32 (57.18 to 159.96) | 99.09 (58.40 to 157.63) | 106.67 (63.32 to 170.54) | 108.12 (63.40 to 175.91) | 101.58 (59.50 to 158.50) | 93.42 (56.39 to 145.99) | 85.71 (51.12 to 134.43) | 76.57 (46.62 to 119.20) | 64.35 (41.02 to 97.82) |
| Chad | Both | Prevalence | 587.12 (166.26 to 1808.19) | 1653.16 (516.00 to 4888.14) | 2314.57 (667.22 to 7034.81) | 3087.95 (859.59 to 9521.73) | 3756.02 (1025.23 to 11648.17) | 4248.90 (1140.96 to 13210.65) | 4604.69 (1236.16 to 14322.69) | 4895.47 (1313.39 to 15228.85) | 5152.51 (1379.42 to 16007.41) | 5364.50 (1434.71 to 16660.81) | 5576.14 (1499.69 to 17289.26) | 5816.40 (1582.15 to 17963.02) | 6045.54 (1627.21 to 18700.55) | 6221.96 (1721.28 to 19098.79) | 6249.31 (1748.38 to 19121.47) | 6213.64 (1713.87 to 19092.38) | 6151.40 (1651.54 to 19040.13) | 6100.67 (1599.16 to 18997.62) | 6026.10 (1524.43 to 18932.54) | 5927.07 (1421.49 to 18852.02) |
| Chad | Female | DALY rates | 10.97 (6.75 to 17.22) | 20.44 (12.56 to 31.43) | 21.52 (13.22 to 32.96) | 22.38 (13.29 to 34.26) | 23.57 (14.17 to 34.94) | 24.56 (14.95 to 36.68) | 24.63 (15.09 to 37.15) | 25.26 (15.84 to 39.32) | 25.27 (14.57 to 37.42) | 25.61 (14.93 to 37.30) | 25.90 (16.19 to 38.68) | 25.97 (16.04 to 38.06) | 26.46 (16.62 to 39.80) | 26.35 (16.76 to 39.37) | 25.64 (15.02 to 38.51) | 25.57 (15.99 to 38.16) | 24.98 (15.71 to 37.17) | 24.34 (15.08 to 35.07) | 23.98 (15.05 to 34.92) | 23.37 (15.59 to 34.67) |
| Chad | Female | Prevalence | 605.64 (184.56 to 1830.77) | 1523.33 (400.14 to 4785.58) | 2137.43 (497.96 to 6895.60) | 2845.30 (612.09 to 9327.13) | 3449.27 (710.20 to 11400.57) | 3891.45 (781.81 to 12916.98) | 4206.53 (833.95 to 13997.39) | 4461.04 (875.07 to 14870.23) | 4678.21 (911.22 to 15614.09) | 4858.40 (939.77 to 16231.91) | 5032.90 (968.06 to 16829.31) | 5216.50 (998.73 to 17458.59) | 5427.60 (1034.89 to 18180.27) | 5523.85 (1050.87 to 18510.87) | 5523.85 (1049.65 to 18510.34) | 5523.81 (1050.89 to 18510.64) | 5523.85 (1049.40 to 18510.35) | 5523.83 (1050.33 to 18510.16) | 5523.85 (1049.95 to 18509.62) | 5523.79 (1051.01 to 18511.15) |
| Chad | Male | DALY rates | 7.79 (4.42 to 12.85) | 53.61 (24.26 to 111.62) | 67.38 (31.07 to 133.45) | 85.57 (41.83 to 162.10) | 104.02 (50.45 to 184.49) | 120.82 (60.59 to 221.01) | 132.52 (68.19 to 234.25) | 142.71 (75.42 to 255.21) | 151.82 (81.41 to 258.26) | 159.85 (87.27 to 267.84) | 166.86 (89.62 to 290.66) | 174.12 (95.17 to 295.45) | 183.11 (104.18 to 302.36) | 184.38 (104.03 to 305.28) | 181.83 (102.00 to 305.80) | 176.92 (100.72 to 288.28) | 174.79 (99.37 to 286.15) | 170.50 (95.28 to 276.31) | 167.81 (96.05 to 277.17) | 162.97 (95.59 to 263.45) |
| Chad | Male | Prevalence | 569.21 (149.89 to 1786.36) | 1782.52 (586.65 to 5034.42) | 2495.04 (772.65 to 7193.57) | 3345.89 (1023.49 to 9736.44) | 4099.18 (1259.34 to 11932.94) | 4667.89 (1464.53 to 13563.08) | 5082.10 (1610.06 to 14720.72) | 5422.33 (1739.49 to 15670.97) | 5716.77 (1852.85 to 16482.87) | 5963.85 (1949.35 to 17174.97) | 6205.41 (2048.35 to 17827.96) | 6462.22 (2155.00 to 18512.35) | 6760.34 (2279.28 to 19307.65) | 6897.43 (2337.23 to 19673.32) | 6897.61 (2336.01 to 19672.91) | 6897.43 (2337.07 to 19674.74) | 6897.46 (2335.04 to 19675.73) | 6897.57 (2338.73 to 19676.51) | 6897.44 (2332.89 to 19671.35) | 6897.49 (2333.92 to 19677.63) |
| Comoros | Both | DALY rates | 11.38 (7.30 to 17.27) | 88.25 (48.53 to 144.84) | 144.39 (88.36 to 227.96) | 224.66 (143.73 to 331.30) | 291.93 (191.61 to 422.37) | 333.00 (217.54 to 467.35) | 352.53 (235.38 to 494.77) | 371.26 (247.34 to 514.48) | 388.20 (257.87 to 546.93) | 399.03 (271.56 to 549.53) | 411.91 (277.90 to 571.50) | 422.57 (278.06 to 580.14) | 417.98 (285.53 to 573.40) | 416.68 (287.96 to 567.33) | 414.90 (276.81 to 558.98) | 401.86 (275.33 to 545.08) | 357.97 (247.73 to 486.87) | 323.43 (228.88 to 429.08) | 283.12 (202.72 to 373.68) | 239.00 (169.86 to 318.39) |
| Comoros | Both | Prevalence | 1743.58 (147.64 to 8887.49) | 4988.87 (786.50 to 23393.73) | 7390.18 (1309.50 to 34248.68) | 10267.25 (2021.17 to 46857.63) | 12730.69 (2674.04 to 57730.51) | 14445.29 (3075.81 to 65530.47) | 15590.84 (3307.75 to 71065.92) | 16513.66 (3507.41 to 75536.54) | 17303.61 (3665.08 to 79365.76) | 17946.69 (3827.43 to 82548.45) | 18592.52 (3972.88 to 85712.38) | 19230.50 (4126.62 to 88908.38) | 19809.99 (4140.11 to 92417.12) | 20124.83 (4213.26 to 94109.40) | 20174.03 (4256.30 to 94151.65) | 20129.17 (4209.21 to 94105.63) | 19819.93 (3889.76 to 93794.09) | 19573.40 (3628.61 to 93529.26) | 19242.01 (3268.18 to 93179.07) | 18877.65 (2867.33 to 92797.37) |
| Comoros | Female | DALY rates | 13.37 (7.61 to 20.51) | 26.38 (15.35 to 40.20) | 30.72 (18.69 to 45.52) | 36.72 (24.02 to 54.66) | 42.19 (26.74 to 63.21) | 47.13 (30.17 to 71.47) | 50.24 (31.82 to 72.23) | 53.63 (34.53 to 79.08) | 55.98 (35.36 to 81.30) | 58.43 (38.31 to 88.02) | 60.19 (39.89 to 86.34) | 62.10 (40.79 to 90.11) | 64.51 (42.08 to 90.96) | 65.37 (41.95 to 93.57) | 64.41 (42.49 to 92.84) | 63.11 (41.20 to 90.66) | 61.33 (41.14 to 87.11) | 60.14 (40.20 to 83.80) | 59.01 (40.31 to 83.49) | 57.24 (38.83 to 79.26) |
| Comoros | Female | Prevalence | 1768.26 (166.97 to 8934.22) | 4495.24 (349.56 to 23047.19) | 6474.10 (437.90 to 33496.18) | 8743.83 (546.95 to 45438.90) | 10699.09 (648.84 to 55692.33) | 12121.06 (728.83 to 63164.47) | 13122.69 (786.82 to 68543.48) | 13911.98 (839.31 to 72850.60) | 14587.42 (881.80 to 76548.25) | 15133.23 (920.14 to 79615.81) | 15671.30 (956.81 to 82656.15) | 16211.61 (998.72 to 85752.07) | 16824.28 (1042.45 to 89280.73) | 17107.28 (1065.11 to 90930.71) | 17107.40 (1065.65 to 90931.32) | 17107.47 (1067.93 to 90930.62) | 17107.37 (1065.86 to 90929.42) | 17107.22 (1065.43 to 90929.26) | 17107.41 (1067.23 to 90931.48) | 17107.37 (1065.68 to 90930.97) |
| Comoros | Male | DALY rates | 9.45 (5.32 to 15.45) | 148.50 (78.88 to 255.92) | 255.72 (152.45 to 417.10) | 412.23 (261.70 to 617.63) | 550.91 (358.14 to 804.39) | 641.11 (414.70 to 902.31) | 691.75 (459.23 to 976.83) | 726.07 (479.86 to 1001.26) | 752.15 (499.66 to 1063.09) | 762.81 (513.42 to 1060.16) | 772.77 (516.80 to 1076.90) | 778.80 (511.29 to 1080.04) | 783.87 (533.95 to 1083.09) | 775.74 (535.47 to 1061.63) | 761.86 (505.46 to 1034.50) | 747.19 (513.32 to 1014.66) | 728.58 (503.59 to 990.56) | 711.73 (500.89 to 947.07) | 699.65 (496.27 to 924.24) | 683.71 (474.80 to 912.21) |
| Comoros | Male | Prevalence | 1719.73 (127.19 to 8842.34) | 5469.57 (1161.70 to 23656.68) | 8287.49 (2082.23 to 34878.88) | 11787.65 (3391.74 to 48126.78) | 14837.56 (4549.49 to 59662.76) | 16950.23 (5387.11 to 67991.70) | 18360.58 (5857.14 to 73862.95) | 19419.81 (6197.87 to 78498.47) | 20279.20 (6514.91 to 82442.09) | 20951.54 (6689.52 to 85654.85) | 21589.67 (6850.82 to 88823.04) | 22213.92 (7039.81 to 92027.60) | 22900.53 (7207.87 to 95663.65) | 23208.99 (7275.81 to 97358.26) | 23209.85 (7284.25 to 97339.62) | 23209.41 (7281.46 to 97342.15) | 23208.86 (7266.16 to 97373.08) | 23210.53 (7282.44 to 97363.74) | 23209.44 (7277.53 to 97356.52) | 23209.09 (7283.16 to 97363.98) |
| Congo | Both | DALY rates | 10.00 (6.40 to 14.44) | 41.82 (21.58 to 75.11) | 52.17 (27.99 to 92.24) | 67.10 (35.99 to 116.75) | 82.69 (45.24 to 140.05) | 96.64 (55.24 to 158.72) | 105.64 (62.32 to 170.88) | 112.10 (65.52 to 178.34) | 118.84 (69.65 to 187.21) | 126.28 (76.87 to 201.15) | 129.40 (78.74 to 200.19) | 130.32 (80.04 to 203.94) | 131.42 (81.75 to 199.83) | 132.33 (82.99 to 198.35) | 129.78 (81.88 to 194.68) | 128.86 (78.49 to 193.99) | 123.49 (77.67 to 184.93) | 114.24 (73.09 to 166.50) | 93.05 (61.70 to 136.10) | 80.01 (53.58 to 115.16) |
| Congo | Both | Prevalence | 763.11 (173.84 to 2717.80) | 2075.57 (516.95 to 7240.32) | 2941.90 (688.09 to 10435.13) | 3961.38 (914.15 to 14099.36) | 4853.99 (1133.12 to 17239.57) | 5527.29 (1308.53 to 19565.11) | 6007.64 (1440.10 to 21210.91) | 6390.57 (1544.25 to 22538.10) | 6727.60 (1640.00 to 23678.85) | 7026.55 (1739.22 to 24662.81) | 7284.93 (1804.97 to 25564.99) | 7530.39 (1845.92 to 26478.80) | 7820.69 (1906.33 to 27519.73) | 7966.04 (1945.37 to 28021.03) | 7969.70 (1947.36 to 28032.84) | 7976.63 (1957.02 to 28030.72) | 7951.32 (1929.12 to 28009.35) | 7887.78 (1868.49 to 27934.31) | 7707.34 (1704.34 to 27719.60) | 7601.70 (1602.04 to 27590.59) |
| Congo | Female | DALY rates | 11.78 (6.98 to 18.35) | 21.41 (12.03 to 32.53) | 22.60 (12.82 to 34.80) | 24.01 (14.06 to 37.67) | 25.39 (15.23 to 39.97) | 26.37 (16.31 to 40.46) | 26.94 (16.46 to 40.70) | 27.57 (16.95 to 40.16) | 27.84 (16.86 to 42.67) | 28.65 (17.78 to 43.27) | 28.86 (18.38 to 44.08) | 29.34 (18.13 to 43.96) | 29.77 (19.01 to 43.74) | 29.72 (19.53 to 43.58) | 29.17 (18.67 to 41.79) | 28.90 (18.72 to 42.05) | 28.24 (18.26 to 41.24) | 27.69 (17.18 to 40.79) | 26.89 (17.26 to 38.77) | 26.18 (16.57 to 38.16) |
| Congo | Female | Prevalence | 782.99 (189.58 to 2748.95) | 1916.81 (400.33 to 6946.37) | 2708.75 (499.10 to 10027.21) | 3617.64 (614.41 to 13560.43) | 4391.43 (712.17 to 16566.50) | 4960.65 (784.44 to 18776.26) | 5366.59 (838.00 to 20350.32) | 5695.51 (880.62 to 21624.47) | 5976.19 (917.07 to 22713.61) | 6212.14 (947.30 to 23626.71) | 6438.90 (977.06 to 24505.06) | 6673.82 (1008.99 to 25415.56) | 6939.46 (1043.38 to 26443.03) | 7068.67 (1060.04 to 26942.14) | 7068.68 (1059.87 to 26941.99) | 7068.68 (1060.38 to 26943.33) | 7068.66 (1060.24 to 26943.85) | 7068.68 (1060.72 to 26942.51) | 7068.65 (1061.01 to 26941.70) | 7068.71 (1060.30 to 26942.65) |
| Congo | Male | DALY rates | 8.23 (4.66 to 12.80) | 62.50 (27.89 to 125.27) | 82.24 (39.28 to 156.79) | 111.25 (54.89 to 202.49) | 141.45 (73.34 to 248.27) | 168.24 (92.77 to 288.94) | 187.34 (103.93 to 318.71) | 203.56 (115.54 to 340.63) | 218.41 (124.32 to 350.97) | 230.52 (133.48 to 374.88) | 241.94 (142.96 to 382.49) | 254.45 (154.70 to 402.11) | 265.66 (159.56 to 417.05) | 271.22 (164.79 to 421.23) | 265.02 (163.37 to 407.78) | 261.42 (153.51 to 407.14) | 256.14 (158.01 to 389.19) | 250.87 (159.08 to 379.26) | 245.69 (151.23 to 378.62) | 239.51 (147.39 to 362.54) |
| Congo | Male | Prevalence | 743.42 (154.06 to 2686.97) | 2236.47 (628.42 to 7496.54) | 3178.97 (862.94 to 10727.75) | 4313.49 (1198.53 to 14541.79) | 5328.39 (1520.10 to 17829.76) | 6104.66 (1800.75 to 20286.81) | 6673.11 (2028.05 to 22035.75) | 7142.65 (2211.28 to 23474.14) | 7549.74 (2381.63 to 24691.28) | 7896.12 (2519.06 to 25733.20) | 8231.80 (2653.49 to 26728.08) | 8583.31 (2799.07 to 27785.69) | 8984.46 (2965.11 to 28941.61) | 9180.70 (3064.00 to 29481.39) | 9180.86 (3062.58 to 29499.18) | 9180.71 (3056.52 to 29472.77) | 9180.56 (3057.61 to 29493.23) | 9180.78 (3058.27 to 29499.94) | 9180.76 (3059.67 to 29514.15) | 9180.78 (3054.40 to 29510.27) |
| Côte d'Ivoire | Both | DALY rates | 18.52 (11.89 to 26.95) | 394.15 (260.29 to 557.17) | 479.26 (328.45 to 656.32) | 524.25 (354.77 to 724.19) | 594.63 (407.23 to 817.71) | 679.15 (461.46 to 946.98) | 763.91 (521.26 to 1091.32) | 840.15 (580.15 to 1176.20) | 902.51 (612.11 to 1264.09) | 960.39 (654.24 to 1356.08) | 1020.05 (692.69 to 1450.90) | 1089.53 (749.32 to 1545.70) | 1165.98 (802.12 to 1617.35) | 1191.98 (810.24 to 1658.59) | 1167.01 (802.66 to 1631.02) | 1125.54 (785.46 to 1557.37) | 1095.67 (756.88 to 1498.18) | 1055.17 (744.20 to 1443.18) | 979.29 (688.28 to 1348.57) | 912.23 (637.38 to 1231.86) |
| Côte d'Ivoire | Both | Prevalence | 5247.34 (1697.56 to 11035.35) | 16370.35 (7148.24 to 31421.02) | 23068.86 (9748.61 to 44793.72) | 30502.24 (12480.80 to 60072.83) | 37062.81 (15046.71 to 73265.84) | 42175.80 (17167.65 to 83197.62) | 45998.35 (18852.32 to 90545.30) | 49121.28 (20338.47 to 96532.95) | 51762.08 (21620.56 to 99468.00) | 54068.19 (22860.10 to 100000.00) | 56268.01 (24103.85 to 100000.00) | 58540.06 (25453.37 to 100000.00) | 61069.08 (27025.13 to 100000.00) | 62307.37 (27820.80 to 100000.00) | 62286.83 (27802.47 to 100000.00) | 62174.52 (27685.34 to 100000.00) | 62067.80 (27559.93 to 100000.00) | 61906.99 (27388.66 to 100000.00) | 61486.52 (26920.74 to 100000.00) | 61128.27 (26539.81 to 100000.00) |
| Côte d'Ivoire | Female | DALY rates | 21.70 (13.88 to 31.19) | 53.76 (33.35 to 80.80) | 85.85 (56.91 to 129.24) | 148.60 (99.42 to 218.68) | 233.91 (155.72 to 342.57) | 318.68 (207.00 to 466.21) | 397.12 (258.17 to 579.53) | 468.88 (302.26 to 693.91) | 534.92 (348.31 to 779.88) | 601.20 (387.80 to 881.35) | 666.25 (431.87 to 977.77) | 735.19 (480.06 to 1080.10) | 815.36 (531.30 to 1191.45) | 845.95 (554.70 to 1233.69) | 830.64 (533.11 to 1201.85) | 814.38 (527.02 to 1195.49) | 804.92 (524.28 to 1171.87) | 791.29 (527.87 to 1135.14) | 772.24 (519.28 to 1100.64) | 752.79 (506.69 to 1046.87) |
| Côte d'Ivoire | Female | Prevalence | 5287.26 (1729.05 to 11080.23) | 13599.83 (4436.38 to 28529.41) | 19853.95 (6543.32 to 41554.83) | 27445.35 (9293.01 to 57081.95) | 34161.75 (11979.62 to 70464.10) | 39304.45 (14225.72 to 80431.60) | 43106.04 (15991.65 to 87694.46) | 46226.75 (17501.95 to 93667.83) | 48933.30 (18872.08 to 98844.96) | 51322.70 (20137.99 to 100000.00) | 53583.21 (21408.66 to 100000.00) | 55895.62 (22736.68 to 100000.00) | 58495.78 (24295.00 to 100000.00) | 59769.58 (25108.32 to 100000.00) | 59769.18 (25103.04 to 100000.00) | 59768.81 (25094.71 to 100000.00) | 59768.57 (25102.37 to 100000.00) | 59768.59 (25104.50 to 100000.00) | 59768.40 (25088.15 to 100000.00) | 59768.68 (25118.08 to 100000.00) |
| Côte d'Ivoire | Male | DALY rates | 15.46 (9.34 to 23.73) | 721.64 (476.73 to 1018.81) | 858.46 (582.71 to 1183.22) | 907.89 (611.80 to 1240.42) | 975.95 (665.42 to 1375.44) | 1041.59 (711.87 to 1433.70) | 1102.48 (749.67 to 1537.01) | 1162.41 (805.58 to 1603.17) | 1216.40 (829.76 to 1687.23) | 1270.82 (867.77 to 1782.42) | 1323.29 (914.48 to 1852.38) | 1384.53 (959.97 to 1940.75) | 1454.15 (998.66 to 2027.96) | 1472.76 (1020.63 to 2041.51) | 1444.90 (1007.20 to 1995.17) | 1409.05 (1003.35 to 1973.30) | 1386.39 (969.65 to 1909.60) | 1358.68 (976.87 to 1872.25) | 1326.23 (953.57 to 1794.28) | 1291.96 (932.86 to 1725.13) |
| Côte d'Ivoire | Male | Prevalence | 5208.94 (1667.27 to 10992.17) | 19035.84 (9786.40 to 34059.12) | 26167.76 (12835.82 to 47915.72) | 33624.12 (15635.09 to 63108.77) | 40129.61 (18091.83 to 76176.52) | 45062.82 (20053.40 to 85902.77) | 48668.22 (21478.02 to 93069.29) | 51633.75 (22794.53 to 98900.03) | 54177.54 (23970.89 to 100000.00) | 56440.97 (25211.64 to 100000.00) | 58569.22 (26412.23 to 100000.00) | 60741.55 (27717.33 to 100000.00) | 63184.09 (29249.65 to 100000.00) | 64366.61 (30024.83 to 100000.00) | 64366.71 (30034.08 to 100000.00) | 64366.47 (30070.52 to 100000.00) | 64366.73 (30026.08 to 100000.00) | 64366.54 (30017.65 to 100000.00) | 64365.57 (30042.45 to 100000.00) | 64366.28 (30049.27 to 100000.00) |
| Democratic Republic of the Congo | Both | DALY rates | 12.04 (7.60 to 17.87) | 122.75 (72.71 to 200.00) | 212.09 (136.62 to 320.22) | 323.27 (215.52 to 458.35) | 393.39 (259.49 to 544.30) | 422.22 (280.81 to 584.94) | 434.14 (291.82 to 592.86) | 442.96 (299.00 to 621.11) | 425.22 (291.25 to 578.94) | 424.95 (293.62 to 584.72) | 421.82 (290.68 to 575.35) | 421.62 (295.29 to 574.32) | 424.11 (291.61 to 579.76) | 424.85 (296.35 to 567.52) | 431.65 (305.48 to 578.30) | 445.16 (315.85 to 601.92) | 449.19 (320.29 to 609.25) | 422.88 (304.03 to 560.31) | 385.37 (278.40 to 506.50) | 321.81 (233.90 to 422.63) |
| Democratic Republic of the Congo | Both | Prevalence | 2237.64 (1099.60 to 3862.31) | 6610.36 (3605.24 to 11070.75) | 9905.34 (5533.32 to 16398.18) | 13787.25 (7821.97 to 22528.86) | 16890.91 (9553.62 to 27473.02) | 18976.86 (10633.18 to 30885.04) | 20399.86 (11348.22 to 33275.51) | 21543.16 (11949.79 to 35220.19) | 22340.37 (12282.44 to 36695.32) | 23129.18 (12680.31 to 38076.59) | 23859.12 (13030.19 to 39369.87) | 24645.41 (13408.48 to 40713.70) | 25562.19 (13866.65 to 42296.54) | 26039.81 (14119.38 to 43084.94) | 26153.23 (14226.19 to 43202.51) | 26362.92 (14431.88 to 43428.83) | 26504.29 (14576.74 to 43558.05) | 26346.35 (14419.36 to 43402.71) | 26051.16 (14123.41 to 43101.73) | 25507.08 (13588.25 to 42531.87) |
| Democratic Republic of the Congo | Female | DALY rates | 14.01 (8.45 to 21.27) | 29.27 (17.63 to 47.63) | 35.50 (21.67 to 52.20) | 44.71 (30.00 to 66.59) | 54.26 (33.31 to 79.66) | 62.19 (41.26 to 89.86) | 68.34 (42.72 to 98.95) | 73.72 (49.54 to 106.92) | 79.00 (51.87 to 114.63) | 83.26 (54.68 to 114.98) | 88.56 (58.37 to 125.48) | 93.77 (63.93 to 134.23) | 100.61 (67.21 to 145.12) | 102.01 (70.14 to 144.96) | 100.08 (66.17 to 143.88) | 98.30 (65.80 to 138.56) | 95.62 (64.55 to 131.38) | 93.40 (64.78 to 130.13) | 91.43 (64.24 to 125.81) | 89.71 (60.68 to 127.79) |
| Democratic Republic of the Congo | Female | Prevalence | 2266.94 (1122.88 to 3895.52) | 5852.32 (2859.08 to 10115.94) | 8464.15 (4100.94 to 14679.22) | 11497.39 (5551.00 to 19964.97) | 14085.36 (6800.91 to 24461.30) | 15990.81 (7727.15 to 27764.24) | 17359.51 (8399.50 to 30127.44) | 18469.77 (8943.29 to 32044.77) | 19424.13 (9417.60 to 33685.30) | 20233.57 (9817.11 to 35079.76) | 21010.58 (10199.88 to 36413.12) | 21813.62 (10597.62 to 37790.41) | 22734.68 (11056.65 to 39366.95) | 23179.87 (11279.35 to 40124.45) | 23179.89 (11279.83 to 40128.08) | 23179.87 (11280.49 to 40126.88) | 23179.95 (11279.88 to 40124.18) | 23179.90 (11276.02 to 40125.03) | 23179.80 (11277.56 to 40128.39) | 23179.90 (11279.87 to 40125.61) |
| Democratic Republic of the Congo | Male | DALY rates | 10.11 (5.71 to 15.93) | 215.47 (121.60 to 364.13) | 386.70 (243.94 to 593.61) | 596.41 (393.05 to 857.90) | 730.68 (480.66 to 1009.98) | 792.10 (522.52 to 1098.18) | 817.56 (550.27 to 1130.24) | 829.86 (558.12 to 1164.30) | 828.44 (566.19 to 1133.92) | 828.19 (569.06 to 1133.68) | 826.06 (565.14 to 1139.91) | 822.37 (575.32 to 1116.12) | 818.82 (562.33 to 1123.08) | 809.89 (558.70 to 1094.51) | 799.45 (561.09 to 1079.31) | 781.64 (553.84 to 1058.26) | 762.58 (542.82 to 1029.98) | 745.96 (533.44 to 1001.14) | 733.42 (525.38 to 959.28) | 715.13 (514.21 to 948.44) |
| Democratic Republic of the Congo | Male | Prevalence | 2209.11 (1076.93 to 3829.97) | 7362.26 (4395.27 to 11944.25) | 11330.43 (6984.52 to 17956.37) | 16032.69 (10134.59 to 24839.28) | 19681.28 (12449.51 to 30497.76) | 22044.54 (13781.19 to 34286.27) | 23586.78 (14528.76 to 36730.82) | 24763.60 (15136.59 to 38620.41) | 25736.69 (15629.45 to 40244.36) | 26546.34 (16008.99 to 41614.16) | 27314.39 (16378.04 to 42956.39) | 28106.78 (16788.22 to 44286.91) | 29012.14 (17257.97 to 45871.05) | 29450.75 (17475.21 to 46615.79) | 29451.40 (17466.18 to 46612.82) | 29450.70 (17452.28 to 46631.95) | 29450.86 (17470.68 to 46601.70) | 29451.28 (17473.07 to 46616.70) | 29451.08 (17472.97 to 46622.40) | 29450.73 (17474.73 to 46609.55) |
| Dominican Republic | Both | DALY rates | 10.45 (6.54 to 15.74) | 49.70 (27.14 to 92.28) | 66.91 (37.54 to 114.80) | 92.47 (52.23 to 154.16) | 118.38 (70.65 to 191.98) | 140.77 (86.87 to 221.27) | 158.26 (96.52 to 240.75) | 172.14 (108.99 to 262.30) | 186.52 (119.86 to 279.05) | 198.68 (127.90 to 292.49) | 208.89 (133.57 to 301.29) | 219.08 (142.68 to 319.72) | 232.47 (151.18 to 338.68) | 234.33 (153.62 to 337.85) | 228.33 (149.88 to 330.38) | 227.14 (152.35 to 329.86) | 222.99 (147.40 to 314.79) | 215.68 (143.21 to 312.82) | 204.51 (139.26 to 290.56) | 196.84 (133.83 to 282.83) |
| Dominican Republic | Both | Prevalence | 993.69 (169.07 to 4045.77) | 2723.89 (620.21 to 10600.82) | 3917.11 (869.27 to 15406.49) | 5326.68 (1195.87 to 20934.45) | 6560.08 (1503.09 to 25638.45) | 7493.97 (1747.73 to 29121.72) | 8175.44 (1930.54 to 31628.55) | 8728.22 (2085.23 to 33665.71) | 9219.38 (2233.44 to 35393.81) | 9646.69 (2360.07 to 36901.97) | 10038.85 (2480.67 to 38308.46) | 10453.93 (2626.03 to 39771.63) | 10933.73 (2795.51 to 41457.72) | 11148.63 (2862.13 to 42232.68) | 11124.82 (2831.86 to 42212.98) | 11149.25 (2861.29 to 42238.99) | 11152.65 (2865.12 to 42249.52) | 11123.32 (2831.84 to 42211.39) | 11062.83 (2769.89 to 42152.15) | 11034.77 (2736.13 to 42117.61) |
| Dominican Republic | Female | DALY rates | 12.28 (7.33 to 18.64) | 22.68 (13.31 to 33.66) | 24.53 (14.09 to 37.48) | 26.64 (15.40 to 40.95) | 28.71 (17.60 to 42.69) | 29.98 (17.82 to 45.39) | 31.51 (19.43 to 45.75) | 32.56 (19.56 to 49.56) | 33.37 (21.03 to 49.25) | 34.00 (20.45 to 51.76) | 35.05 (22.15 to 51.22) | 35.54 (21.83 to 52.57) | 36.59 (23.45 to 53.48) | 36.49 (23.00 to 54.75) | 36.19 (23.98 to 53.25) | 35.81 (22.51 to 52.64) | 34.81 (22.95 to 51.05) | 34.23 (21.47 to 51.20) | 32.86 (21.79 to 47.97) | 32.58 (21.24 to 46.90) |
| Dominican Republic | Female | Prevalence | 1012.83 (186.09 to 4072.77) | 2508.54 (390.92 to 10344.94) | 3579.26 (487.06 to 15011.60) | 4802.26 (602.57 to 20335.36) | 5836.44 (700.15 to 24828.97) | 6596.33 (774.95 to 28128.23) | 7142.42 (827.95 to 30496.35) | 7582.77 (872.32 to 32404.01) | 7956.14 (909.31 to 34021.75) | 8279.78 (942.74 to 35421.96) | 8583.86 (972.91 to 36737.84) | 8900.98 (1005.71 to 38110.53) | 9261.31 (1041.81 to 39666.24) | 9432.80 (1060.03 to 40406.98) | 9432.84 (1059.84 to 40406.59) | 9432.79 (1059.55 to 40407.28) | 9432.77 (1060.17 to 40406.75) | 9432.78 (1060.25 to 40406.70) | 9432.82 (1060.12 to 40407.48) | 9432.80 (1060.17 to 40406.84) |
| Dominican Republic | Male | DALY rates | 8.67 (4.96 to 13.99) | 76.52 (35.29 to 157.34) | 110.70 (56.02 to 198.54) | 163.07 (88.00 to 279.57) | 216.42 (125.45 to 362.08) | 261.57 (154.67 to 419.90) | 296.22 (176.73 to 459.16) | 324.34 (201.25 to 501.86) | 348.63 (218.67 to 528.68) | 369.30 (231.45 to 548.96) | 388.30 (246.53 to 570.56) | 406.40 (259.97 to 603.05) | 428.31 (273.97 to 634.61) | 433.55 (277.43 to 627.22) | 427.17 (277.86 to 623.19) | 419.56 (276.93 to 616.29) | 411.53 (267.75 to 587.96) | 403.74 (264.35 to 588.74) | 395.46 (264.95 to 569.38) | 385.61 (258.20 to 557.37) |
| Dominican Republic | Male | Prevalence | 975.05 (153.31 to 4019.45) | 2937.59 (773.74 to 10737.58) | 4266.23 (1139.17 to 15666.36) | 5889.13 (1670.46 to 21388.72) | 7351.23 (2233.97 to 26317.14) | 8472.69 (2700.86 to 29986.48) | 9299.87 (3020.96 to 32636.59) | 9977.13 (3296.44 to 34797.11) | 10556.56 (3524.71 to 36626.64) | 11062.79 (3737.28 to 38216.61) | 11540.45 (3934.19 to 39717.59) | 12038.96 (4141.08 to 41302.18) | 12605.86 (4382.73 to 43096.78) | 12876.48 (4507.55 to 43922.53) | 12875.79 (4498.08 to 43938.48) | 12875.40 (4494.31 to 43941.31) | 12875.73 (4500.54 to 43944.59) | 12875.54 (4492.57 to 43933.83) | 12875.99 (4495.26 to 43938.51) | 12875.82 (4511.14 to 43951.87) |
| Egypt | Both | DALY rates | 9.86 (6.22 to 14.56) | 35.75 (19.01 to 65.89) | 41.48 (22.31 to 77.16) | 48.88 (26.38 to 89.76) | 56.56 (30.15 to 99.60) | 62.12 (34.37 to 106.81) | 66.42 (36.61 to 113.42) | 70.46 (38.14 to 122.37) | 73.80 (40.59 to 127.35) | 76.24 (42.49 to 127.07) | 79.54 (45.50 to 136.46) | 81.87 (45.72 to 134.12) | 84.73 (46.34 to 140.26) | 83.89 (47.75 to 137.98) | 84.09 (47.91 to 138.48) | 88.22 (50.75 to 146.66) | 84.92 (49.13 to 139.22) | 84.07 (47.45 to 136.76) | 100.20 (57.31 to 164.74) | 109.35 (62.04 to 186.06) |
| Egypt | Both | Prevalence | 532.70 (120.80 to 2193.75) | 1424.17 (368.61 to 5596.85) | 1979.21 (452.36 to 8070.22) | 2623.61 (555.71 to 10909.22) | 3180.49 (644.35 to 13338.92) | 3591.55 (714.11 to 15112.61) | 3888.62 (767.76 to 16383.37) | 4130.22 (809.33 to 17413.03) | 4334.02 (850.95 to 18262.40) | 4512.98 (883.48 to 19026.79) | 4686.06 (919.68 to 19745.17) | 4860.93 (954.25 to 20480.72) | 5068.57 (1000.31 to 21328.47) | 5153.24 (1008.46 to 21718.53) | 5168.56 (1025.77 to 21730.22) | 5219.36 (1080.09 to 21775.98) | 5201.91 (1062.11 to 21757.56) | 5209.57 (1070.71 to 21761.56) | 5371.46 (1224.49 to 21886.88) | 5476.47 (1305.94 to 21967.69) |
| Egypt | Female | DALY rates | 11.63 (6.89 to 18.52) | 20.37 (11.63 to 31.87) | 21.14 (11.77 to 33.27) | 21.98 (12.37 to 33.06) | 22.84 (13.22 to 34.95) | 22.98 (13.05 to 34.54) | 23.29 (14.52 to 35.50) | 23.91 (15.00 to 36.82) | 23.99 (14.43 to 36.81) | 24.26 (14.95 to 37.73) | 24.33 (14.80 to 37.10) | 24.62 (15.32 to 37.16) | 24.78 (15.21 to 36.95) | 24.72 (15.69 to 37.79) | 24.33 (14.89 to 36.04) | 23.81 (14.98 to 34.94) | 23.70 (14.58 to 35.63) | 22.69 (14.32 to 34.13) | 22.61 (14.02 to 33.00) | 21.88 (13.77 to 31.16) |
| Egypt | Female | Prevalence | 549.63 (138.22 to 2213.79) | 1305.79 (260.68 to 5520.25) | 1820.58 (295.40 to 7962.42) | 2410.49 (336.18 to 10760.16) | 2913.63 (372.10 to 13145.45) | 3280.60 (398.22 to 14884.33) | 3543.19 (416.76 to 16127.28) | 3755.49 (431.50 to 17131.91) | 3930.40 (444.03 to 17959.48) | 4088.36 (455.40 to 18705.94) | 4235.09 (465.77 to 19401.38) | 4386.21 (475.86 to 20115.44) | 4559.64 (488.65 to 20935.09) | 4642.29 (494.05 to 21325.87) | 4642.28 (493.94 to 21327.22) | 4642.31 (495.26 to 21326.95) | 4642.26 (494.67 to 21327.25) | 4642.24 (495.42 to 21326.52) | 4642.28 (495.00 to 21326.15) | 4642.29 (494.64 to 21326.26) |
| Egypt | Male | DALY rates | 8.17 (4.38 to 12.87) | 50.26 (22.02 to 106.78) | 60.28 (27.53 to 126.86) | 73.84 (35.71 to 147.56) | 88.41 (42.65 to 167.83) | 99.64 (49.08 to 184.67) | 108.15 (55.05 to 197.76) | 115.90 (57.60 to 211.29) | 121.89 (61.87 to 219.24) | 127.44 (65.71 to 221.77) | 133.38 (70.59 to 240.13) | 138.23 (72.93 to 232.67) | 142.91 (74.58 to 245.47) | 144.30 (75.95 to 239.73) | 141.60 (78.03 to 243.68) | 139.05 (74.53 to 238.76) | 136.66 (74.53 to 231.72) | 134.43 (71.89 to 228.32) | 132.49 (71.62 to 222.02) | 130.16 (71.53 to 225.13) |
| Egypt | Male | Prevalence | 516.55 (103.57 to 2174.63) | 1535.84 (426.83 to 5673.46) | 2125.89 (533.03 to 8169.91) | 2821.29 (676.68 to 11047.48) | 3432.50 (821.15 to 13521.62) | 3889.63 (933.21 to 15331.45) | 4222.85 (1022.63 to 16631.16) | 4496.06 (1097.42 to 17687.48) | 4723.68 (1164.89 to 18554.84) | 4931.25 (1225.64 to 19342.84) | 5125.88 (1284.67 to 20080.47) | 5328.25 (1345.27 to 20840.31) | 5562.47 (1418.09 to 21710.25) | 5674.91 (1456.51 to 22119.44) | 5675.05 (1451.35 to 22118.06) | 5674.79 (1456.22 to 22130.38) | 5674.94 (1452.15 to 22121.27) | 5675.00 (1455.12 to 22118.46) | 5674.99 (1455.48 to 22120.29) | 5674.91 (1456.04 to 22120.27) |
| Equatorial Guinea | Both | DALY rates | 11.44 (6.97 to 17.15) | 102.55 (59.25 to 170.23) | 169.52 (104.91 to 263.21) | 258.14 (169.95 to 371.28) | 320.31 (213.14 to 460.02) | 343.77 (230.06 to 476.03) | 360.24 (241.12 to 502.72) | 381.82 (256.96 to 534.61) | 380.14 (253.86 to 519.89) | 404.43 (273.17 to 550.61) | 416.27 (284.12 to 568.38) | 418.40 (286.12 to 567.87) | 414.10 (285.85 to 561.27) | 394.82 (269.03 to 538.86) | 375.03 (263.12 to 498.03) | 359.31 (256.08 to 477.86) | 341.00 (241.49 to 450.65) | 323.83 (228.23 to 432.36) | 292.67 (212.41 to 390.30) | 226.36 (165.00 to 299.16) |
| Equatorial Guinea | Both | Prevalence | 1934.93 (213.50 to 6956.52) | 5686.84 (1104.12 to 19023.92) | 8455.61 (1776.88 to 27852.08) | 11738.90 (2636.49 to 38088.24) | 14404.19 (3332.68 to 46607.88) | 16202.65 (3700.72 to 52732.96) | 17490.60 (3950.99 to 57148.35) | 18599.63 (4210.72 to 60774.36) | 19369.12 (4236.61 to 63698.31) | 20231.56 (4508.43 to 66355.25) | 20970.37 (4668.51 to 68828.59) | 21645.65 (4741.15 to 71313.83) | 22366.11 (4793.67 to 74125.34) | 22599.01 (4713.43 to 75356.03) | 22494.63 (4612.23 to 75248.79) | 22409.71 (4529.41 to 75156.01) | 22323.90 (4437.78 to 75064.24) | 22233.28 (4350.54 to 74976.28) | 21992.70 (4113.10 to 74733.43) | 21399.18 (3519.80 to 74132.14) |
| Equatorial Guinea | Female | DALY rates | 13.42 (7.97 to 20.37) | 27.34 (16.22 to 41.64) | 32.34 (20.56 to 48.52) | 39.60 (25.47 to 59.17) | 46.96 (30.05 to 69.67) | 52.68 (33.36 to 76.17) | 57.33 (37.00 to 86.27) | 60.66 (40.01 to 86.59) | 64.88 (42.67 to 96.28) | 67.95 (43.76 to 98.86) | 71.43 (47.14 to 100.46) | 74.28 (48.76 to 107.79) | 78.02 (51.60 to 113.41) | 78.88 (53.56 to 115.37) | 77.44 (52.30 to 109.53) | 76.17 (52.56 to 107.50) | 74.08 (48.87 to 104.54) | 72.10 (48.86 to 100.53) | 70.84 (47.63 to 98.06) | 68.62 (48.17 to 95.99) |
| Equatorial Guinea | Female | Prevalence | 1950.29 (231.53 to 6959.95) | 5075.11 (531.72 to 18319.73) | 7327.33 (703.72 to 26631.81) | 9939.77 (916.85 to 36240.62) | 12154.28 (1107.99 to 44352.44) | 13794.08 (1255.76 to 50334.77) | 14978.88 (1370.33 to 54641.58) | 15930.78 (1463.73 to 58115.12) | 16733.49 (1542.53 to 61059.64) | 17401.93 (1613.39 to 63515.41) | 18041.29 (1680.71 to 65877.34) | 18698.74 (1751.03 to 68332.66) | 19449.87 (1832.99 to 71160.65) | 19808.53 (1873.38 to 72512.58) | 19808.70 (1874.80 to 72507.89) | 19808.65 (1875.45 to 72509.14) | 19808.53 (1878.04 to 72513.12) | 19808.41 (1875.84 to 72509.41) | 19808.45 (1877.54 to 72511.87) | 19808.51 (1877.08 to 72512.81) |
| Equatorial Guinea | Male | DALY rates | 9.59 (5.42 to 15.40) | 173.39 (96.82 to 297.28) | 305.64 (180.06 to 479.81) | 491.40 (317.61 to 712.06) | 637.87 (422.76 to 916.37) | 719.09 (477.52 to 1003.85) | 762.72 (511.57 to 1069.82) | 787.20 (528.43 to 1110.13) | 800.28 (531.16 to 1095.93) | 805.83 (540.57 to 1099.44) | 805.41 (542.58 to 1100.74) | 804.49 (543.56 to 1099.61) | 800.11 (549.69 to 1072.48) | 787.93 (537.61 to 1072.81) | 771.32 (532.44 to 1035.01) | 757.87 (529.80 to 1016.33) | 738.61 (524.47 to 989.49) | 722.30 (508.09 to 969.07) | 706.84 (506.61 to 938.70) | 689.59 (495.35 to 900.27) |
| Equatorial Guinea | Male | Prevalence | 1920.58 (197.00 to 6953.32) | 6262.98 (1579.56 to 19687.14) | 9575.30 (2871.92 to 29063.07) | 13659.20 (4542.19 to 40060.29) | 17018.03 (5909.16 to 49228.15) | 19308.19 (6805.21 to 55825.12) | 20827.88 (7267.85 to 60479.04) | 21968.45 (7578.19 to 64131.07) | 22881.56 (7768.63 to 67214.79) | 23607.06 (7926.31 to 69742.94) | 24275.79 (8017.25 to 72159.03) | 24951.95 (8096.50 to 74658.57) | 25715.61 (8161.29 to 77530.51) | 26071.16 (8174.89 to 78894.09) | 26071.33 (8207.75 to 78898.70) | 26071.18 (8194.66 to 78881.95) | 26070.89 (8182.03 to 78864.49) | 26071.68 (8208.37 to 78881.17) | 26070.98 (8183.78 to 78881.37) | 26070.36 (8165.50 to 78887.52) |
| Eritrea | Both | DALY rates | 9.15 (5.69 to 13.69) | 29.89 (16.03 to 55.58) | 31.64 (16.89 to 57.65) | 33.48 (17.32 to 61.88) | 34.37 (17.52 to 61.95) | 34.89 (18.66 to 59.27) | 36.09 (19.19 to 64.23) | 36.84 (20.21 to 67.19) | 37.38 (19.68 to 64.62) | 38.62 (21.06 to 67.96) | 39.30 (20.89 to 69.33) | 38.62 (20.85 to 69.26) | 37.93 (21.26 to 67.79) | 36.15 (19.78 to 63.36) | 33.92 (19.13 to 56.77) | 31.79 (18.30 to 52.00) | 30.15 (17.08 to 47.69) | 28.33 (17.51 to 44.45) | 27.94 (17.15 to 44.24) | 26.29 (15.81 to 41.08) |
| Eritrea | Both | Prevalence | 269.05 (96.48 to 991.93) | 732.95 (251.17 to 2587.30) | 960.72 (276.31 to 3667.72) | 1222.05 (304.44 to 4916.48) | 1441.28 (326.11 to 5973.05) | 1601.97 (342.79 to 6749.31) | 1716.07 (356.53 to 7299.28) | 1810.48 (368.27 to 7743.92) | 1890.52 (378.14 to 8123.00) | 1966.65 (390.48 to 8455.65) | 2031.48 (397.88 to 8760.67) | 2093.46 (404.20 to 9074.84) | 2162.80 (408.99 to 9432.36) | 2187.77 (404.26 to 9602.12) | 2172.51 (390.84 to 9589.68) | 2159.56 (380.00 to 9579.10) | 2149.55 (371.11 to 9570.67) | 2141.73 (363.71 to 9565.50) | 2142.82 (364.67 to 9566.19) | 2134.81 (357.05 to 9559.82) |
| Eritrea | Female | DALY rates | 10.79 (6.10 to 16.81) | 19.37 (11.06 to 31.01) | 19.65 (11.19 to 31.31) | 19.84 (11.52 to 30.35) | 20.18 (12.05 to 31.72) | 20.07 (11.61 to 31.14) | 20.14 (11.68 to 30.81) | 20.14 (11.50 to 31.59) | 20.15 (11.70 to 31.17) | 20.05 (12.16 to 30.30) | 20.32 (11.15 to 31.12) | 19.99 (12.29 to 29.39) | 20.23 (12.48 to 30.94) | 19.97 (11.54 to 30.53) | 19.81 (11.98 to 30.34) | 19.22 (11.73 to 27.92) | 19.06 (11.77 to 29.51) | 18.31 (11.54 to 28.48) | 17.92 (11.01 to 25.98) | 17.51 (10.03 to 26.34) |
| Eritrea | Female | Prevalence | 284.78 (110.82 to 1003.89) | 652.66 (201.61 to 2524.68) | 867.75 (214.03 to 3598.37) | 1115.42 (226.75 to 4835.49) | 1325.61 (238.44 to 5883.61) | 1479.92 (246.45 to 6654.13) | 1589.21 (253.17 to 7199.85) | 1677.26 (258.30 to 7638.45) | 1752.55 (262.31 to 8013.15) | 1817.30 (266.89 to 8337.19) | 1877.85 (270.49 to 8638.54) | 1940.86 (273.55 to 8952.80) | 2012.83 (277.59 to 9311.59) | 2048.58 (279.53 to 9490.06) | 2048.58 (279.50 to 9490.29) | 2048.55 (279.15 to 9490.00) | 2048.53 (278.94 to 9489.43) | 2048.56 (279.65 to 9490.19) | 2048.56 (279.50 to 9490.50) | 2048.53 (279.24 to 9489.93) |
| Eritrea | Male | DALY rates | 7.61 (4.38 to 12.42) | 39.62 (17.66 to 84.26) | 42.53 (19.19 to 89.64) | 46.25 (19.54 to 98.31) | 48.82 (21.56 to 100.55) | 51.02 (22.63 to 100.97) | 54.18 (24.30 to 109.05) | 55.77 (26.11 to 117.02) | 57.12 (24.62 to 111.66) | 58.13 (26.70 to 113.52) | 59.38 (27.31 to 117.93) | 59.88 (27.54 to 117.67) | 60.26 (28.43 to 120.41) | 60.13 (27.20 to 117.61) | 59.11 (28.77 to 114.15) | 58.34 (27.82 to 112.69) | 57.00 (26.96 to 107.83) | 55.45 (26.64 to 105.25) | 54.63 (26.26 to 102.64) | 52.69 (26.00 to 99.48) |
| Eritrea | Male | Prevalence | 254.28 (81.76 to 980.69) | 807.20 (277.16 to 2709.79) | 1045.10 (312.66 to 3790.30) | 1321.92 (350.00 to 5012.46) | 1559.11 (384.79 to 6064.16) | 1734.86 (411.14 to 6852.94) | 1859.98 (430.82 to 7412.07) | 1961.54 (447.25 to 7863.52) | 2048.52 (460.71 to 8248.80) | 2123.57 (475.55 to 8580.12) | 2194.06 (488.58 to 8889.91) | 2267.61 (500.30 to 9214.11) | 2351.94 (516.28 to 9584.68) | 2393.96 (524.96 to 9768.10) | 2393.90 (525.32 to 9767.23) | 2393.93 (523.62 to 9767.22) | 2393.94 (525.46 to 9767.22) | 2393.88 (525.54 to 9769.28) | 2393.98 (524.52 to 9767.85) | 2393.94 (525.35 to 9769.75) |
| Ethiopia | Both | DALY rates | 9.82 (6.38 to 14.31) | 45.83 (25.27 to 82.47) | 60.13 (33.30 to 104.56) | 78.77 (44.63 to 131.63) | 96.25 (56.27 to 157.44) | 109.61 (65.47 to 173.08) | 123.13 (73.81 to 190.81) | 134.81 (83.67 to 206.24) | 153.29 (95.92 to 232.95) | 161.86 (102.59 to 244.06) | 167.62 (105.35 to 251.87) | 178.42 (116.48 to 265.21) | 189.53 (120.63 to 279.32) | 197.95 (128.90 to 287.39) | 207.17 (135.23 to 301.99) | 193.59 (125.94 to 276.54) | 170.61 (113.81 to 244.54) | 146.39 (100.24 to 206.42) | 122.42 (84.18 to 170.57) | 96.71 (66.88 to 132.34) |
| Ethiopia | Both | Prevalence | 843.02 (268.69 to 2037.60) | 2364.63 (818.41 to 5539.93) | 3339.47 (1125.79 to 7868.03) | 4485.52 (1506.56 to 10591.63) | 5507.42 (1831.19 to 13054.51) | 6279.70 (2092.32 to 14928.06) | 6862.30 (2316.56 to 16254.99) | 7338.47 (2502.41 to 17336.57) | 7794.02 (2708.06 to 18315.43) | 8140.55 (2829.65 to 19260.63) | 8438.62 (2916.46 to 20372.56) | 8812.33 (3060.81 to 21559.13) | 9229.90 (3219.45 to 22487.49) | 9509.78 (3333.26 to 23160.74) | 9690.39 (3448.84 to 23486.56) | 9750.78 (3306.68 to 24156.19) | 9667.98 (3088.71 to 24196.95) | 9419.13 (2878.78 to 24013.01) | 9169.19 (2704.04 to 24123.31) | 8943.30 (2566.81 to 23536.17) |
| Ethiopia | Female | DALY rates | 11.56 (7.32 to 16.98) | 21.66 (14.12 to 30.76) | 23.48 (15.36 to 35.00) | 25.22 (16.72 to 37.44) | 26.89 (17.89 to 39.47) | 28.05 (18.80 to 40.47) | 29.05 (18.87 to 42.29) | 29.83 (20.21 to 42.67) | 30.34 (20.27 to 42.96) | 30.86 (21.11 to 43.51) | 31.28 (20.89 to 44.14) | 31.56 (21.20 to 46.83) | 32.05 (21.36 to 45.82) | 31.89 (22.04 to 45.62) | 31.37 (21.61 to 44.03) | 30.72 (21.17 to 42.31) | 30.32 (20.92 to 41.73) | 29.62 (20.50 to 40.64) | 29.07 (20.01 to 40.25) | 28.31 (19.96 to 39.10) |
| Ethiopia | Female | Prevalence | 860.66 (283.97 to 2055.96) | 2173.84 (660.64 to 5281.27) | 3041.64 (871.21 to 7478.72) | 4046.14 (1124.14 to 10061.59) | 4946.19 (1344.42 to 12414.79) | 5613.37 (1508.22 to 14175.79) | 6088.38 (1630.00 to 15398.82) | 6474.96 (1739.88 to 16411.25) | 6786.46 (1825.81 to 17281.32) | 7050.96 (1880.25 to 18165.48) | 7268.67 (1886.72 to 19201.17) | 7518.36 (1918.48 to 20169.41) | 7815.41 (1970.29 to 20963.65) | 7965.94 (2001.79 to 21244.96) | 7973.99 (2026.85 to 20978.80) | 8075.72 (2028.61 to 21832.22) | 8221.27 (1897.27 to 22202.09) | 8260.75 (1861.42 to 22484.07) | 8275.07 (1867.30 to 22903.34) | 8293.88 (1932.07 to 23160.20) |
| Ethiopia | Male | DALY rates | 8.16 (5.12 to 12.02) | 68.52 (33.13 to 134.20) | 94.01 (46.96 to 174.62) | 131.96 (70.53 to 231.02) | 173.39 (95.79 to 292.56) | 207.27 (117.39 to 339.97) | 233.76 (135.60 to 371.85) | 255.22 (154.34 to 404.12) | 271.87 (167.47 to 421.69) | 286.54 (178.38 to 442.03) | 299.20 (185.03 to 457.19) | 314.37 (197.47 to 472.28) | 330.33 (206.26 to 491.79) | 336.97 (213.23 to 495.11) | 336.76 (215.98 to 496.21) | 342.71 (220.28 to 494.07) | 339.04 (222.71 to 488.94) | 324.15 (217.96 to 463.41) | 309.64 (206.11 to 445.40) | 296.93 (197.67 to 422.69) |
| Ethiopia | Male | Prevalence | 826.17 (253.34 to 2020.07) | 2543.72 (937.24 to 5785.84) | 3614.81 (1326.83 to 8236.83) | 4921.91 (1866.13 to 11122.08) | 6131.49 (2375.74 to 13775.25) | 7077.55 (2794.38 to 15844.74) | 7772.34 (3130.86 to 17277.85) | 8328.94 (3409.50 to 18411.73) | 8765.82 (3617.40 to 19312.85) | 9177.50 (3821.65 to 20302.86) | 9567.72 (3972.83 to 21503.07) | 10010.12 (4162.41 to 22845.56) | 10494.54 (4364.03 to 23849.91) | 10802.26 (4436.38 to 24565.97) | 10955.64 (4436.94 to 25046.42) | 11284.38 (4465.65 to 25947.31) | 11404.86 (4461.54 to 26371.13) | 11182.64 (4393.76 to 26786.61) | 10962.36 (4362.74 to 26325.47) | 10844.03 (4381.69 to 25943.28) |
| Fiji | Both | DALY rates | 15.83 (10.17 to 23.24) | 253.70 (164.88 to 373.37) | 396.36 (267.19 to 557.67) | 474.61 (319.97 to 654.18) | 498.26 (338.94 to 693.12) | 521.83 (358.98 to 719.82) | 541.08 (373.15 to 744.00) | 556.12 (382.86 to 766.36) | 571.64 (392.31 to 789.53) | 582.40 (400.78 to 823.47) | 588.78 (399.38 to 822.13) | 606.75 (416.47 to 828.81) | 619.86 (431.38 to 850.88) | 617.67 (431.85 to 857.50) | 606.30 (423.19 to 823.36) | 598.22 (418.67 to 808.04) | 572.63 (402.46 to 777.07) | 513.42 (366.35 to 692.71) | 304.42 (210.11 to 416.62) | 273.32 (188.03 to 379.55) |
| Fiji | Both | Prevalence | 3838.24 (589.39 to 10858.15) | 11057.22 (3127.95 to 28163.63) | 16274.08 (4843.96 to 40889.63) | 21655.91 (6136.12 to 55249.53) | 25901.89 (6811.48 to 67238.26) | 29034.54 (7314.03 to 75949.13) | 31306.22 (7788.90 to 82192.73) | 33114.70 (8161.88 to 87210.72) | 34659.36 (8519.63 to 91497.07) | 35986.93 (8813.13 to 95239.13) | 37190.28 (9085.36 to 97730.68) | 38521.33 (9446.62 to 99608.56) | 39928.19 (9793.89 to 100000.00) | 40617.95 (9975.78 to 100000.00) | 40630.13 (9985.89 to 100000.00) | 40680.39 (10032.54 to 100000.00) | 40575.62 (9926.68 to 100000.00) | 40147.70 (9517.25 to 100000.00) | 38318.74 (7609.96 to 100000.00) | 38086.63 (7366.63 to 100000.00) |
| Fiji | Female | DALY rates | 18.70 (11.49 to 28.48) | 39.26 (24.66 to 56.87) | 53.85 (32.93 to 81.83) | 77.63 (49.97 to 113.57) | 106.87 (68.74 to 159.95) | 133.53 (87.45 to 199.32) | 156.20 (102.28 to 229.96) | 176.75 (116.91 to 258.98) | 195.82 (123.29 to 285.59) | 213.80 (137.53 to 312.89) | 230.89 (149.24 to 345.27) | 249.21 (164.10 to 356.51) | 271.59 (177.29 to 390.44) | 278.91 (181.61 to 398.36) | 273.85 (181.61 to 397.32) | 269.60 (179.64 to 387.58) | 263.33 (175.04 to 374.73) | 258.77 (174.09 to 371.30) | 252.29 (168.40 to 357.16) | 246.53 (166.91 to 347.50) |
| Fiji | Female | Prevalence | 3865.05 (617.72 to 10877.79) | 9327.60 (1444.46 to 26366.73) | 13505.02 (2074.36 to 38218.54) | 18447.52 (2870.99 to 52102.48) | 22738.95 (3624.28 to 64003.12) | 25902.41 (4225.19 to 72655.76) | 28198.94 (4696.30 to 78877.19) | 30062.07 (5106.88 to 83912.06) | 31639.63 (5472.78 to 88214.28) | 33024.16 (5816.17 to 91984.95) | 34296.92 (6143.55 to 95485.82) | 35631.47 (6494.19 to 99211.31) | 37099.12 (6902.21 to 100000.00) | 37836.95 (7120.30 to 100000.00) | 37836.59 (7121.37 to 100000.00) | 37837.58 (7120.95 to 100000.00) | 37837.19 (7120.51 to 100000.00) | 37837.09 (7123.18 to 100000.00) | 37836.99 (7134.63 to 100000.00) | 37837.20 (7121.02 to 100000.00) |
| Fiji | Male | DALY rates | 13.14 (7.80 to 20.33) | 456.27 (289.63 to 681.56) | 722.49 (487.45 to 1017.91) | 856.27 (571.50 to 1175.85) | 883.84 (600.79 to 1248.95) | 904.09 (620.35 to 1266.99) | 916.90 (626.31 to 1268.64) | 929.41 (634.41 to 1264.80) | 940.82 (640.77 to 1297.66) | 947.89 (644.09 to 1320.27) | 950.57 (638.82 to 1314.89) | 959.06 (655.25 to 1324.74) | 968.36 (674.58 to 1324.27) | 962.82 (669.17 to 1356.79) | 942.07 (657.75 to 1279.94) | 918.61 (638.80 to 1236.21) | 897.39 (632.63 to 1213.26) | 877.50 (627.06 to 1175.36) | 859.81 (608.96 to 1132.03) | 849.47 (611.82 to 1134.80) |
| Fiji | Male | Prevalence | 3813.04 (562.77 to 10839.69) | 12691.11 (4720.23 to 29865.91) | 18910.69 (7406.44 to 43457.52) | 24740.51 (9201.79 to 58291.55) | 29017.78 (9937.01 to 70450.17) | 32117.97 (10420.71 to 79213.64) | 34340.35 (10808.25 to 85460.59) | 36118.49 (11151.83 to 90484.76) | 37625.75 (11465.06 to 94754.65) | 38924.70 (11738.57 to 98498.55) | 40115.17 (12036.52 to 100000.00) | 41368.94 (12347.81 to 100000.00) | 42759.21 (12716.85 to 100000.00) | 43451.41 (12863.08 to 100000.00) | 43451.57 (12862.04 to 100000.00) | 43452.04 (12861.91 to 100000.00) | 43450.99 (12881.83 to 100000.00) | 43451.41 (12867.72 to 100000.00) | 43450.58 (12867.67 to 100000.00) | 43450.78 (12884.68 to 100000.00) |
| Gabon | Both | DALY rates | 9.88 (6.16 to 14.74) | 39.95 (20.71 to 72.60) | 48.31 (25.59 to 85.51) | 60.46 (33.59 to 105.48) | 73.62 (42.09 to 125.31) | 86.66 (48.74 to 146.45) | 98.54 (56.73 to 165.39) | 109.42 (59.93 to 181.79) | 112.44 (65.37 to 185.73) | 112.79 (66.79 to 178.36) | 112.37 (67.07 to 179.82) | 113.95 (70.21 to 177.77) | 118.78 (72.27 to 184.53) | 121.04 (74.22 to 184.95) | 118.98 (75.05 to 188.78) | 115.57 (73.50 to 177.97) | 107.28 (69.12 to 160.50) | 88.80 (58.10 to 133.18) | 68.68 (43.71 to 101.97) | 50.92 (34.29 to 73.58) |
| Gabon | Both | Prevalence | 703.55 (153.02 to 2773.22) | 1904.21 (482.28 to 7341.85) | 2683.43 (620.08 to 10547.41) | 3603.07 (784.22 to 14278.91) | 4408.39 (951.05 to 17455.94) | 5024.05 (1113.91 to 19794.24) | 5483.91 (1250.21 to 21469.03) | 5872.00 (1375.92 to 22872.89) | 6162.38 (1441.19 to 24014.92) | 6373.99 (1461.78 to 24910.92) | 6579.96 (1478.01 to 25788.14) | 6816.46 (1522.50 to 26721.55) | 7102.88 (1595.95 to 27806.00) | 7250.23 (1642.71 to 28337.17) | 7257.18 (1648.51 to 28343.86) | 7245.38 (1634.33 to 28330.77) | 7188.68 (1576.44 to 28264.40) | 7036.76 (1425.27 to 28090.13) | 6855.16 (1235.58 to 27882.04) | 6698.04 (1109.71 to 27703.21) |
| Gabon | Female | DALY rates | 11.63 (6.92 to 17.42) | 21.17 (12.18 to 33.57) | 22.19 (13.30 to 34.12) | 23.48 (13.76 to 37.73) | 24.57 (14.55 to 38.19) | 25.53 (15.73 to 39.76) | 25.79 (16.12 to 39.22) | 26.65 (16.74 to 40.61) | 26.99 (17.46 to 40.70) | 26.99 (16.66 to 38.24) | 27.71 (17.38 to 41.46) | 27.79 (17.15 to 41.72) | 28.41 (18.08 to 42.67) | 28.45 (18.31 to 41.38) | 27.78 (17.48 to 40.38) | 27.39 (16.40 to 40.30) | 26.75 (15.97 to 38.55) | 26.16 (17.26 to 38.18) | 25.64 (16.04 to 37.81) | 25.09 (16.46 to 36.74) |
| Gabon | Female | Prevalence | 722.36 (171.79 to 2794.98) | 1757.54 (351.42 to 7050.51) | 2476.15 (428.63 to 10181.20) | 3307.22 (518.52 to 13798.85) | 4013.11 (598.04 to 16868.84) | 4528.18 (655.81 to 19108.03) | 4894.59 (698.75 to 20698.48) | 5197.00 (732.27 to 22011.71) | 5456.18 (762.36 to 23136.52) | 5665.42 (787.02 to 24044.10) | 5871.19 (809.36 to 24936.71) | 6082.80 (833.33 to 25855.04) | 6325.59 (861.19 to 26907.19) | 6441.35 (875.23 to 27408.72) | 6441.36 (874.97 to 27408.53) | 6441.35 (875.42 to 27407.72) | 6441.35 (876.08 to 27409.04) | 6441.34 (874.67 to 27408.12) | 6441.35 (876.15 to 27408.21) | 6441.38 (874.92 to 27408.19) |
| Gabon | Male | DALY rates | 8.14 (4.42 to 13.09) | 58.81 (26.93 to 119.11) | 75.31 (35.39 to 144.51) | 99.94 (48.19 to 181.46) | 125.44 (64.89 to 226.44) | 146.81 (76.36 to 260.61) | 163.77 (88.65 to 282.62) | 178.22 (93.62 to 299.43) | 189.50 (103.74 to 318.00) | 200.52 (112.98 to 326.78) | 209.82 (119.74 to 347.59) | 218.22 (128.67 to 353.09) | 230.29 (134.77 to 370.51) | 233.67 (141.35 to 366.76) | 228.17 (141.02 to 374.49) | 224.00 (136.08 to 354.89) | 219.95 (135.44 to 347.95) | 214.78 (132.73 to 337.52) | 212.11 (127.68 to 335.73) | 205.50 (121.91 to 319.50) |
| Gabon | Male | Prevalence | 684.92 (137.09 to 2751.66) | 2051.55 (586.32 to 7526.76) | 2897.65 (794.70 to 10880.49) | 3918.96 (1083.43 to 14788.50) | 4825.98 (1354.16 to 18088.28) | 5511.87 (1592.32 to 20476.22) | 6012.25 (1766.48 to 22161.26) | 6433.05 (1924.61 to 23588.69) | 6799.21 (2062.31 to 24807.02) | 7098.45 (2176.28 to 25797.18) | 7395.70 (2295.44 to 26768.07) | 7704.40 (2423.01 to 27770.28) | 8062.04 (2574.60 to 28915.12) | 8234.19 (2641.82 to 29466.58) | 8233.87 (2639.13 to 29463.62) | 8233.98 (2644.65 to 29465.70) | 8234.28 (2639.40 to 29461.15) | 8234.11 (2645.62 to 29461.61) | 8234.11 (2643.45 to 29461.03) | 8234.55 (2644.34 to 29469.35) |
| Ghana | Both | DALY rates | 10.51 (6.61 to 15.55) | 56.89 (30.71 to 100.87) | 81.79 (45.66 to 141.45) | 118.91 (70.89 to 190.41) | 151.79 (96.07 to 230.73) | 176.15 (112.05 to 269.79) | 203.36 (130.16 to 300.94) | 228.85 (147.48 to 338.87) | 247.06 (159.60 to 363.65) | 260.28 (172.48 to 378.11) | 271.06 (179.77 to 391.61) | 283.90 (186.76 to 408.22) | 287.85 (188.52 to 413.21) | 285.34 (191.14 to 405.18) | 277.32 (186.27 to 390.15) | 272.34 (187.89 to 381.33) | 262.29 (176.32 to 362.39) | 246.06 (166.21 to 336.49) | 231.67 (158.46 to 322.34) | 197.35 (137.36 to 273.02) |
| Ghana | Both | Prevalence | 1175.28 (287.90 to 3353.22) | 3239.08 (933.41 to 8890.09) | 4695.61 (1350.44 to 12910.11) | 6453.23 (1882.48 to 17631.67) | 7955.48 (2381.54 to 21641.64) | 9062.89 (2725.65 to 24561.67) | 9933.04 (3067.07 to 26733.67) | 10662.51 (3366.29 to 28509.99) | 11264.35 (3612.85 to 29991.63) | 11764.33 (3799.73 to 31263.65) | 12237.24 (3975.15 to 32470.78) | 12733.71 (4181.13 to 33696.83) | 13222.36 (4336.31 to 35035.15) | 13437.99 (4389.49 to 35651.44) | 13410.40 (4380.81 to 35620.16) | 13410.78 (4366.75 to 35630.27) | 13356.04 (4319.77 to 35571.30) | 13254.90 (4233.85 to 35455.08) | 13159.92 (4149.81 to 35358.22) | 12872.82 (3905.91 to 35052.95) |
| Ghana | Female | DALY rates | 12.29 (7.34 to 18.63) | 23.27 (13.53 to 35.07) | 25.58 (14.67 to 38.60) | 28.62 (17.28 to 44.13) | 31.11 (19.34 to 46.05) | 33.34 (21.19 to 50.57) | 34.95 (22.01 to 52.54) | 35.96 (22.55 to 51.19) | 37.24 (23.80 to 55.17) | 38.55 (24.97 to 55.42) | 39.59 (25.85 to 56.94) | 40.62 (27.03 to 57.93) | 41.10 (25.97 to 60.89) | 41.15 (27.09 to 58.84) | 40.79 (25.73 to 58.17) | 40.49 (26.12 to 56.57) | 40.26 (25.75 to 56.69) | 39.29 (25.53 to 55.65) | 38.41 (24.86 to 54.00) | 37.20 (23.87 to 53.19) |
| Ghana | Female | Prevalence | 1194.94 (308.14 to 3376.40) | 2969.94 (703.68 to 8545.66) | 4243.38 (948.19 to 12356.23) | 5720.44 (1231.58 to 16768.51) | 6975.98 (1477.13 to 20512.81) | 7896.05 (1657.33 to 23253.56) | 8553.21 (1788.24 to 25207.90) | 9077.08 (1892.99 to 26764.88) | 9524.38 (1983.23 to 28094.64) | 9918.69 (2061.30 to 29264.34) | 10288.71 (2136.15 to 30360.91) | 10668.56 (2214.21 to 31487.74) | 11102.66 (2302.98 to 32772.27) | 11311.35 (2344.74 to 33389.84) | 11311.39 (2345.89 to 33390.53) | 11311.36 (2345.75 to 33390.54) | 11311.42 (2345.52 to 33391.15) | 11311.39 (2345.03 to 33390.82) | 11311.39 (2345.54 to 33391.29) | 11311.37 (2346.60 to 33389.82) |
| Ghana | Male | DALY rates | 8.80 (5.06 to 14.23) | 89.22 (42.19 to 169.74) | 135.61 (70.71 to 241.59) | 207.86 (120.36 to 342.16) | 284.71 (177.49 to 439.19) | 345.82 (212.33 to 544.43) | 391.73 (249.10 to 590.23) | 427.91 (270.30 to 641.45) | 456.57 (290.65 to 679.77) | 483.08 (317.43 to 706.81) | 503.59 (328.32 to 734.13) | 525.15 (343.37 to 759.06) | 546.68 (356.39 to 794.77) | 552.07 (364.20 to 785.08) | 542.22 (361.73 to 771.36) | 531.95 (363.50 to 755.06) | 523.43 (347.77 to 729.44) | 512.63 (343.03 to 715.66) | 503.62 (338.21 to 717.03) | 493.59 (341.35 to 686.51) |
| Ghana | Male | Prevalence | 1156.40 (268.46 to 3330.96) | 3497.94 (1156.92 to 9226.62) | 5128.69 (1742.58 to 13448.26) | 7175.10 (2520.19 to 18492.48) | 9034.33 (3301.20 to 22896.44) | 10449.22 (3984.42 to 26127.06) | 11476.50 (4481.28 to 28449.84) | 12298.77 (4893.37 to 30321.93) | 13001.69 (5258.24 to 31893.90) | 13618.82 (5593.34 to 33281.29) | 14194.70 (5904.61 to 34598.77) | 14781.77 (6177.89 to 35894.34) | 15445.80 (6492.04 to 37415.29) | 15760.97 (6603.17 to 38126.62) | 15761.24 (6605.08 to 38122.62) | 15761.52 (6604.56 to 38145.04) | 15760.79 (6607.95 to 38142.10) | 15760.49 (6616.95 to 38121.47) | 15760.96 (6599.93 to 38132.88) | 15761.09 (6606.61 to 38136.61) |
| Guinea | Both | DALY rates | 10.06 (6.44 to 14.93) | 49.34 (25.44 to 86.39) | 67.71 (36.64 to 116.97) | 91.51 (52.50 to 151.78) | 112.04 (65.92 to 185.04) | 127.95 (77.08 to 197.52) | 143.62 (89.14 to 220.17) | 164.26 (104.57 to 248.33) | 175.05 (109.95 to 265.15) | 193.52 (122.71 to 285.00) | 210.14 (134.10 to 306.70) | 221.19 (145.35 to 322.46) | 231.26 (148.89 to 337.37) | 229.94 (151.03 to 335.50) | 225.75 (147.73 to 327.20) | 227.51 (152.56 to 326.41) | 226.67 (151.83 to 320.52) | 218.37 (148.33 to 306.36) | 205.37 (138.07 to 295.92) | 178.13 (120.38 to 250.46) |
| Guinea | Both | Prevalence | 958.04 (194.51 to 2865.37) | 2694.34 (648.86 to 7642.45) | 3878.58 (903.74 to 11112.51) | 5269.50 (1218.38 to 15158.70) | 6467.39 (1503.09 to 18659.56) | 7344.07 (1724.10 to 21206.71) | 8007.12 (1919.02 to 23073.46) | 8605.38 (2140.94 to 24632.07) | 9062.81 (2284.74 to 25905.11) | 9544.54 (2480.42 to 27120.69) | 9987.84 (2654.79 to 28240.85) | 10408.21 (2811.36 to 29377.32) | 10867.01 (2953.76 to 30643.39) | 11050.56 (2978.41 to 31200.15) | 11051.48 (2987.09 to 31199.63) | 11107.85 (3043.52 to 31270.51) | 11130.59 (3056.05 to 31292.71) | 11092.49 (3021.21 to 31245.95) | 11013.86 (2945.67 to 31160.49) | 10790.52 (2732.77 to 30906.93) |
| Guinea | Female | DALY rates | 11.83 (6.99 to 17.79) | 22.04 (13.49 to 33.23) | 24.52 (14.83 to 38.82) | 26.66 (15.80 to 41.06) | 28.36 (18.49 to 43.83) | 29.99 (18.81 to 43.46) | 31.32 (20.67 to 47.40) | 31.97 (19.44 to 48.71) | 33.04 (21.26 to 49.31) | 33.69 (22.09 to 49.74) | 34.67 (21.75 to 52.63) | 34.97 (21.90 to 51.08) | 35.96 (23.30 to 54.22) | 35.58 (22.52 to 53.17) | 34.81 (21.61 to 49.08) | 34.46 (22.74 to 51.05) | 33.88 (21.34 to 49.16) | 33.49 (21.43 to 48.45) | 32.42 (21.48 to 46.30) | 31.69 (20.42 to 46.47) |
| Guinea | Female | Prevalence | 976.78 (212.91 to 2890.65) | 2479.11 (470.13 to 7500.64) | 3534.59 (602.65 to 10855.22) | 4747.78 (756.97 to 14705.40) | 5791.26 (890.31 to 18014.26) | 6547.19 (987.98 to 20408.18) | 7090.72 (1059.11 to 22128.23) | 7521.80 (1116.36 to 23489.64) | 7888.61 (1165.80 to 24648.65) | 8214.25 (1209.34 to 25675.95) | 8514.38 (1249.18 to 26623.13) | 8830.48 (1292.07 to 27622.75) | 9190.92 (1342.43 to 28758.07) | 9357.65 (1362.93 to 29282.66) | 9357.69 (1364.60 to 29283.67) | 9357.66 (1363.34 to 29283.25) | 9357.66 (1363.09 to 29284.03) | 9357.61 (1362.95 to 29284.14) | 9357.68 (1365.10 to 29284.50) | 9357.63 (1365.02 to 29283.32) |
| Guinea | Male | DALY rates | 8.35 (4.63 to 13.59) | 75.65 (35.47 to 145.95) | 109.08 (52.94 to 198.68) | 159.04 (83.41 to 275.30) | 213.02 (119.89 to 357.56) | 257.29 (147.12 to 413.63) | 291.89 (177.94 to 458.35) | 319.73 (198.54 to 491.33) | 342.56 (209.07 to 534.89) | 363.09 (224.97 to 544.89) | 381.42 (241.40 to 562.15) | 399.99 (260.39 to 587.61) | 420.38 (269.44 to 618.17) | 425.33 (278.21 to 630.00) | 417.51 (268.26 to 613.56) | 408.91 (271.56 to 593.97) | 403.05 (264.08 to 573.07) | 395.26 (265.29 to 558.21) | 386.91 (256.10 to 567.63) | 378.66 (247.48 to 542.39) |
| Guinea | Male | Prevalence | 940.00 (178.21 to 2841.05) | 2901.80 (810.27 to 7806.56) | 4208.06 (1174.82 to 11387.53) | 5812.76 (1690.61 to 15630.72) | 7283.26 (2215.02 to 19438.22) | 8396.24 (2647.19 to 22261.06) | 9217.11 (2988.64 to 24321.52) | 9878.78 (3283.40 to 25974.63) | 10447.83 (3519.88 to 27387.14) | 10955.98 (3747.03 to 28653.58) | 11426.10 (3945.99 to 29819.93) | 11923.10 (4162.34 to 31062.03) | 12490.09 (4445.26 to 32469.08) | 12752.44 (4568.18 to 33127.81) | 12752.60 (4573.81 to 33123.88) | 12752.37 (4567.36 to 33137.80) | 12752.49 (4564.25 to 33130.28) | 12752.37 (4557.58 to 33122.97) | 12752.29 (4568.41 to 33129.65) | 12752.66 (4580.78 to 33130.25) |
| Guinea-Bissau | Both | DALY rates | 12.68 (8.00 to 19.00) | 146.23 (87.94 to 227.29) | 252.48 (167.38 to 369.64) | 362.03 (237.70 to 512.94) | 412.34 (273.35 to 578.72) | 429.52 (288.65 to 593.04) | 432.07 (293.57 to 602.21) | 434.77 (295.02 to 593.44) | 437.99 (296.75 to 594.68) | 444.72 (301.14 to 610.25) | 454.14 (311.97 to 626.97) | 467.55 (323.06 to 641.77) | 480.68 (328.44 to 662.05) | 493.32 (343.67 to 670.49) | 488.64 (343.12 to 657.93) | 475.22 (334.11 to 633.72) | 435.65 (307.36 to 590.37) | 381.33 (272.44 to 511.90) | 343.51 (245.76 to 453.55) | 299.81 (215.82 to 399.97) |
| Guinea-Bissau | Both | Prevalence | 2552.20 (215.58 to 9679.71) | 7556.24 (1434.59 to 26075.77) | 11291.69 (2482.60 to 38058.36) | 15535.69 (3433.48 to 51980.80) | 18796.23 (3945.13 to 63570.91) | 21035.54 (4129.16 to 71952.78) | 22554.75 (4243.33 to 77820.70) | 23776.76 (4360.85 to 82595.27) | 24817.61 (4484.19 to 86674.10) | 25707.78 (4625.06 to 90137.40) | 26572.63 (4776.07 to 93477.26) | 27505.88 (4977.94 to 96838.26) | 28530.99 (5180.33 to 98914.59) | 29097.32 (5368.20 to 99855.74) | 29132.89 (5400.41 to 99855.79) | 29114.40 (5378.58 to 99855.84) | 28817.19 (5083.20 to 99843.37) | 28411.08 (4675.81 to 99821.23) | 28126.05 (4387.21 to 99809.51) | 27787.39 (4048.40 to 99788.94) |
| Guinea-Bissau | Female | DALY rates | 14.92 (9.17 to 23.16) | 30.83 (18.64 to 44.93) | 39.22 (23.83 to 59.84) | 50.65 (32.71 to 75.10) | 63.60 (41.28 to 92.79) | 74.00 (48.00 to 108.12) | 83.02 (54.70 to 123.39) | 90.64 (59.55 to 129.87) | 97.38 (63.55 to 140.15) | 103.99 (69.64 to 153.29) | 110.21 (72.80 to 162.54) | 116.05 (75.30 to 167.31) | 123.74 (80.18 to 178.28) | 125.58 (83.71 to 178.74) | 123.96 (83.43 to 180.05) | 121.84 (81.91 to 175.55) | 119.97 (82.20 to 167.93) | 117.65 (79.08 to 165.04) | 115.12 (78.32 to 162.68) | 112.25 (78.92 to 157.63) |
| Guinea-Bissau | Female | Prevalence | 2583.65 (241.65 to 9740.11) | 6618.33 (545.63 to 25160.44) | 9552.25 (730.06 to 36471.86) | 12987.41 (971.55 to 49642.49) | 15943.21 (1204.65 to 60888.55) | 18132.21 (1393.48 to 69162.50) | 19691.95 (1540.94 to 75017.60) | 20960.63 (1663.08 to 79780.98) | 22030.19 (1775.38 to 83835.38) | 22911.37 (1875.71 to 87249.97) | 23737.82 (1963.82 to 90524.89) | 24595.82 (2062.89 to 93955.40) | 25556.88 (2180.81 to 97861.59) | 26008.88 (2238.41 to 99703.05) | 26008.57 (2235.00 to 99699.47) | 26008.91 (2236.35 to 99701.49) | 26008.91 (2238.37 to 99705.82) | 26008.75 (2238.25 to 99702.07) | 26008.86 (2237.93 to 99705.81) | 26008.99 (2235.85 to 99700.15) |
| Guinea-Bissau | Male | DALY rates | 10.47 (6.10 to 16.59) | 261.68 (155.11 to 417.60) | 467.52 (306.23 to 688.05) | 687.49 (445.15 to 974.83) | 802.89 (528.70 to 1127.20) | 843.10 (563.88 to 1177.26) | 852.17 (578.62 to 1188.72) | 857.14 (577.87 to 1176.70) | 857.37 (583.45 to 1176.99) | 856.68 (580.59 to 1189.17) | 855.24 (589.88 to 1180.21) | 853.06 (589.56 to 1185.32) | 848.61 (578.89 to 1157.75) | 840.76 (581.07 to 1152.60) | 825.07 (579.17 to 1123.16) | 805.26 (556.70 to 1083.25) | 795.11 (561.60 to 1063.95) | 776.96 (550.84 to 1041.31) | 763.04 (542.44 to 1006.63) | 745.70 (532.92 to 996.85) |
| Guinea-Bissau | Male | Prevalence | 2521.15 (188.43 to 9620.10) | 8494.56 (2204.19 to 27007.52) | 13045.73 (4126.77 to 39661.73) | 18199.17 (6146.66 to 54575.95) | 21991.33 (7136.51 to 66666.93) | 24413.07 (7539.55 to 75209.31) | 26000.28 (7672.35 to 81203.17) | 27233.09 (7759.37 to 86049.33) | 28249.65 (7849.54 to 90169.29) | 29088.71 (7920.44 to 93628.37) | 29878.74 (8026.83 to 96920.49) | 30697.46 (8157.91 to 100000.00) | 31596.63 (8252.08 to 100000.00) | 32015.27 (8304.97 to 100000.00) | 32015.19 (8304.92 to 100000.00) | 32014.85 (8308.05 to 100000.00) | 32014.93 (8303.15 to 100000.00) | 32015.47 (8315.45 to 100000.00) | 32015.13 (8312.11 to 100000.00) | 32015.07 (8306.64 to 100000.00) |
| Guyana | Both | DALY rates | 19.12 (12.63 to 28.15) | 404.51 (268.07 to 564.94) | 479.35 (321.54 to 656.79) | 541.01 (366.34 to 744.83) | 622.78 (424.41 to 857.75) | 713.74 (489.62 to 1019.84) | 798.98 (540.62 to 1132.90) | 882.14 (601.72 to 1250.69) | 954.54 (647.73 to 1368.66) | 1029.88 (699.62 to 1439.47) | 1100.64 (742.40 to 1540.64) | 1178.28 (802.50 to 1682.67) | 1276.65 (878.81 to 1802.24) | 1313.00 (886.76 to 1846.92) | 1292.40 (893.67 to 1794.27) | 1272.14 (871.16 to 1777.48) | 1239.25 (846.26 to 1723.54) | 1201.11 (840.21 to 1655.37) | 1198.39 (830.85 to 1636.80) | 1240.16 (871.61 to 1684.35) |
| Guyana | Both | Prevalence | 5411.70 (2582.68 to 9370.89) | 17088.51 (9625.95 to 27697.43) | 24047.98 (13226.46 to 39225.43) | 31828.26 (17172.32 to 52243.57) | 38730.86 (20808.97 to 63559.07) | 44123.88 (23718.23 to 72141.58) | 48185.18 (25949.66 to 78458.89) | 51611.75 (27896.17 to 83649.10) | 54535.51 (29620.22 to 88066.54) | 57223.93 (31348.61 to 92118.88) | 59740.04 (33007.24 to 95878.04) | 62385.03 (34816.58 to 98703.70) | 65507.86 (37041.51 to 100000.00) | 66873.10 (38033.52 to 100000.00) | 66847.91 (37992.91 to 100000.00) | 66824.44 (37989.27 to 100000.00) | 66719.88 (37892.21 to 100000.00) | 66584.55 (37726.44 to 100000.00) | 66760.92 (37919.52 to 100000.00) | 67348.20 (38468.37 to 100000.00) |
| Guyana | Female | DALY rates | 22.54 (14.66 to 34.77) | 56.65 (35.31 to 83.50) | 94.02 (61.40 to 138.66) | 164.41 (106.68 to 244.97) | 261.60 (171.41 to 382.99) | 362.70 (234.15 to 550.75) | 454.55 (296.00 to 675.76) | 541.57 (347.16 to 810.21) | 620.32 (399.44 to 927.38) | 700.39 (448.29 to 1018.05) | 778.84 (500.65 to 1124.35) | 862.26 (556.66 to 1251.59) | 969.35 (636.26 to 1398.78) | 1015.14 (659.67 to 1490.44) | 1004.92 (665.85 to 1460.52) | 991.60 (646.07 to 1451.62) | 977.23 (643.22 to 1420.65) | 959.21 (631.39 to 1374.62) | 939.37 (614.07 to 1370.42) | 917.62 (615.87 to 1311.21) |
| Guyana | Female | Prevalence | 5475.85 (2628.81 to 9459.86) | 14278.05 (6853.80 to 24667.33) | 20926.70 (10133.06 to 36041.05) | 28795.44 (14186.45 to 49242.32) | 35838.20 (17926.10 to 60712.84) | 41332.53 (20963.14 to 69412.95) | 45458.24 (23315.92 to 75780.76) | 48938.04 (25360.60 to 81029.71) | 51921.50 (27167.59 to 85508.96) | 54673.04 (28936.45 to 89601.82) | 57237.91 (30637.81 to 93419.92) | 59938.38 (32508.56 to 97445.55) | 63145.21 (34761.73 to 100000.00) | 64561.04 (35777.65 to 100000.00) | 64560.62 (35795.09 to 100000.00) | 64560.12 (35816.95 to 100000.00) | 64560.17 (35808.17 to 100000.00) | 64560.10 (35774.31 to 100000.00) | 64561.44 (35792.95 to 100000.00) | 64560.66 (35802.14 to 100000.00) |
| Guyana | Male | DALY rates | 15.83 (9.78 to 24.35) | 756.46 (496.99 to 1055.22) | 872.37 (586.37 to 1208.97) | 927.05 (629.21 to 1271.74) | 999.24 (675.79 to 1389.81) | 1078.65 (739.08 to 1519.29) | 1152.95 (792.50 to 1609.37) | 1227.58 (844.53 to 1706.85) | 1292.40 (877.95 to 1806.81) | 1363.34 (926.95 to 1910.74) | 1427.26 (983.13 to 1998.90) | 1503.89 (1053.35 to 2123.14) | 1601.00 (1116.89 to 2268.53) | 1635.56 (1127.75 to 2316.74) | 1610.18 (1121.19 to 2282.68) | 1588.49 (1107.23 to 2213.21) | 1561.49 (1092.10 to 2172.39) | 1534.69 (1069.94 to 2132.10) | 1506.37 (1082.33 to 2039.63) | 1474.89 (1050.16 to 2010.44) |
| Guyana | Male | Prevalence | 5350.00 (2538.31 to 9285.29) | 19932.00 (12720.62 to 30764.06) | 27231.56 (16293.45 to 42488.23) | 34937.16 (20082.34 to 55524.20) | 41745.91 (23717.26 to 66593.39) | 47025.48 (26559.54 to 74978.19) | 50987.61 (28656.32 to 81211.17) | 54323.63 (30467.94 to 86305.89) | 57178.00 (32151.92 to 90651.98) | 59805.51 (33789.79 to 94666.22) | 62279.66 (35412.17 to 98482.67) | 64905.84 (37194.56 to 100000.00) | 68001.59 (39447.77 to 100000.00) | 69376.84 (40475.25 to 100000.00) | 69376.32 (40422.42 to 100000.00) | 69377.68 (40438.77 to 100000.00) | 69375.95 (40455.22 to 100000.00) | 69376.24 (40418.41 to 100000.00) | 69376.18 (40448.07 to 100000.00) | 69376.87 (40408.76 to 100000.00) |
| Haiti | Both | DALY rates | 14.33 (9.08 to 21.13) | 218.15 (142.01 to 325.11) | 351.80 (237.77 to 497.91) | 438.26 (292.06 to 597.93) | 458.93 (312.02 to 623.70) | 468.29 (320.38 to 646.21) | 484.44 (330.12 to 661.03) | 507.11 (343.96 to 699.95) | 528.86 (361.70 to 728.15) | 546.13 (376.84 to 775.18) | 553.91 (377.51 to 765.94) | 569.58 (395.93 to 778.80) | 583.05 (406.56 to 804.27) | 575.37 (398.64 to 796.63) | 556.13 (381.49 to 751.35) | 534.04 (376.87 to 722.88) | 502.63 (350.69 to 678.74) | 472.41 (335.94 to 640.36) | 430.67 (306.43 to 578.66) | 369.02 (262.38 to 493.53) |
| Haiti | Both | Prevalence | 3376.11 (440.22 to 9751.83) | 10189.13 (2543.78 to 26697.99) | 15111.62 (4088.53 to 39170.64) | 20235.85 (5279.81 to 52958.77) | 24189.10 (5868.62 to 64272.94) | 27041.21 (6199.75 to 72501.64) | 29146.21 (6531.44 to 78456.95) | 30931.12 (6897.25 to 83378.90) | 32432.25 (7238.09 to 87490.69) | 33733.67 (7547.08 to 91105.40) | 34874.54 (7748.76 to 94446.90) | 36118.19 (8050.03 to 97432.72) | 37481.54 (8362.92 to 99423.29) | 38040.39 (8435.18 to 99863.15) | 37945.40 (8321.41 to 99856.82) | 37828.62 (8192.04 to 99849.86) | 37632.53 (8000.79 to 99840.04) | 37443.75 (7810.20 to 99831.37) | 37152.33 (7501.43 to 99815.30) | 36663.39 (7049.67 to 99796.40) |
| Haiti | Female | DALY rates | 16.86 (10.27 to 24.92) | 36.21 (22.74 to 54.84) | 49.36 (31.40 to 71.24) | 69.63 (44.01 to 104.23) | 93.73 (62.07 to 134.21) | 115.82 (75.35 to 167.92) | 133.53 (86.04 to 194.22) | 151.37 (103.32 to 219.70) | 165.53 (105.83 to 238.58) | 179.06 (118.33 to 259.38) | 193.21 (126.56 to 284.56) | 209.55 (139.01 to 300.17) | 228.36 (150.14 to 336.34) | 236.54 (154.57 to 339.48) | 234.42 (154.98 to 341.39) | 231.71 (152.26 to 327.42) | 227.54 (150.38 to 326.49) | 224.21 (149.67 to 324.44) | 219.02 (148.14 to 312.91) | 214.24 (143.58 to 307.24) |
| Haiti | Female | Prevalence | 3413.12 (468.52 to 9812.13) | 8706.43 (1135.88 to 25155.65) | 12637.57 (1620.40 to 36577.18) | 17228.28 (2242.60 to 49800.98) | 21217.82 (2842.30 to 61168.14) | 24168.62 (3320.88 to 69492.74) | 26284.72 (3684.40 to 75445.11) | 28014.47 (4018.35 to 80307.54) | 29464.57 (4316.20 to 84370.21) | 30724.18 (4591.88 to 87929.24) | 31922.31 (4855.55 to 91324.45) | 33153.31 (5147.28 to 94807.32) | 34552.02 (5499.45 to 98830.69) | 35209.09 (5665.43 to 99729.71) | 35208.81 (5663.53 to 99726.18) | 35208.95 (5671.78 to 99723.66) | 35208.55 (5666.95 to 99722.98) | 35208.99 (5665.57 to 99723.72) | 35208.61 (5662.61 to 99720.64) | 35208.55 (5668.25 to 99727.22) |
| Haiti | Male | DALY rates | 11.81 (7.05 to 18.69) | 405.24 (259.74 to 609.86) | 668.61 (446.99 to 949.75) | 831.56 (557.11 to 1142.26) | 868.16 (588.64 to 1199.92) | 881.51 (608.87 to 1243.24) | 891.89 (605.69 to 1220.15) | 898.52 (601.72 to 1231.70) | 907.28 (615.29 to 1263.32) | 911.73 (620.18 to 1289.09) | 919.82 (628.33 to 1260.44) | 921.65 (632.90 to 1269.69) | 928.24 (643.16 to 1272.79) | 922.87 (633.61 to 1263.82) | 908.74 (625.54 to 1227.64) | 893.68 (619.49 to 1223.37) | 878.51 (615.97 to 1162.59) | 861.20 (602.58 to 1168.45) | 843.66 (594.42 to 1126.29) | 824.54 (584.83 to 1084.37) |
| Haiti | Male | Prevalence | 3339.41 (412.10 to 9692.03) | 11713.84 (3978.62 to 28221.16) | 17703.16 (6531.16 to 41660.94) | 23444.68 (8384.65 to 56180.25) | 27518.60 (9140.03 to 67752.07) | 30408.85 (9531.47 to 76029.08) | 32468.69 (9807.52 to 81954.01) | 34140.22 (10096.70 to 86758.21) | 35523.09 (10330.62 to 90740.67) | 36731.05 (10569.31 to 94268.78) | 37869.35 (10777.74 to 97614.37) | 39017.43 (10995.46 to 100000.00) | 40332.57 (11303.53 to 100000.00) | 40944.10 (11432.18 to 100000.00) | 40944.78 (11422.23 to 100000.00) | 40944.96 (11406.12 to 100000.00) | 40944.59 (11413.43 to 100000.00) | 40944.54 (11413.55 to 100000.00) | 40945.09 (11416.55 to 100000.00) | 40944.82 (11423.68 to 100000.00) |
| India | Both | DALY rates | 12.45 (8.45 to 17.60) | 121.66 (79.71 to 180.51) | 158.02 (104.72 to 231.71) | 201.44 (134.93 to 288.45) | 238.39 (160.30 to 341.52) | 264.33 (178.15 to 373.79) | 290.46 (197.32 to 408.96) | 319.36 (217.96 to 447.84) | 341.11 (233.56 to 473.83) | 364.36 (249.65 to 510.18) | 383.68 (264.02 to 534.37) | 399.30 (273.37 to 559.53) | 424.50 (292.17 to 590.11) | 420.83 (290.13 to 578.20) | 396.76 (275.02 to 546.80) | 397.30 (277.10 to 540.34) | 403.98 (285.97 to 545.53) | 373.29 (264.25 to 499.78) | 329.00 (234.00 to 439.99) | 313.13 (224.48 to 417.32) |
| India | Both | Prevalence | 1999.82 (1836.86 to 2166.58) | 5545.74 (5094.53 to 6026.06) | 7783.80 (7189.02 to 8426.01) | 10380.77 (9606.61 to 11221.06) | 12411.51 (11520.60 to 13385.08) | 13784.67 (12819.64 to 14857.98) | 15051.34 (14010.86 to 16208.48) | 16378.57 (15230.97 to 17629.40) | 17299.97 (16068.49 to 18603.62) | 18244.53 (16938.76 to 19611.66) | 19147.16 (17758.20 to 20578.12) | 19937.40 (18480.79 to 21417.82) | 21068.75 (19528.58 to 22614.57) | 21304.94 (19714.08 to 22874.80) | 20840.38 (19261.26 to 22387.35) | 21563.62 (19928.82 to 23166.17) | 22648.61 (20946.23 to 24313.46) | 21535.49 (19859.58 to 23196.14) | 19633.23 (18102.41 to 21278.42) | 19418.51 (17859.28 to 21166.14) |
| India | Female | DALY rates | 14.60 (9.85 to 20.73) | 28.51 (19.34 to 40.27) | 36.27 (24.49 to 52.50) | 49.37 (33.12 to 72.31) | 65.76 (44.15 to 94.88) | 81.44 (54.55 to 118.97) | 97.08 (64.45 to 140.37) | 115.19 (75.84 to 168.95) | 128.46 (84.68 to 189.11) | 142.57 (94.45 to 207.61) | 156.25 (103.49 to 225.52) | 168.33 (109.77 to 242.20) | 184.05 (122.20 to 261.46) | 190.61 (127.62 to 273.94) | 182.22 (119.87 to 257.35) | 185.42 (124.33 to 265.04) | 197.81 (132.89 to 276.72) | 189.72 (129.95 to 267.49) | 169.03 (115.73 to 235.58) | 165.23 (112.71 to 229.43) |
| India | Female | Prevalence | 2004.98 (1845.11 to 2166.09) | 4747.97 (4365.75 to 5132.60) | 6694.13 (6152.27 to 7247.12) | 8956.82 (8236.06 to 9695.55) | 10829.99 (9971.50 to 11720.89) | 12190.86 (11236.87 to 13189.28) | 13404.98 (12367.27 to 14487.01) | 14721.55 (13584.36 to 15898.62) | 15498.27 (14312.31 to 16730.51) | 16284.30 (15046.16 to 17563.49) | 17029.39 (15731.68 to 18353.81) | 17624.14 (16277.88 to 18984.75) | 18417.98 (17017.21 to 19839.69) | 18871.99 (17435.99 to 20316.69) | 18552.66 (17113.01 to 20002.38) | 19105.48 (17629.02 to 20559.03) | 20205.54 (18647.64 to 21788.45) | 19691.81 (18141.45 to 21260.79) | 18332.74 (16876.72 to 19840.85) | 18386.93 (16840.32 to 20003.92) |
| India | Male | DALY rates | 10.45 (6.99 to 15.19) | 207.11 (135.09 to 312.25) | 269.88 (177.44 to 397.04) | 341.22 (226.88 to 491.24) | 399.11 (264.06 to 571.93) | 437.93 (291.96 to 621.12) | 472.37 (314.23 to 669.35) | 507.95 (341.11 to 716.55) | 533.12 (363.09 to 749.71) | 558.33 (377.82 to 777.58) | 583.31 (399.62 to 805.88) | 604.96 (415.72 to 840.43) | 639.19 (440.16 to 881.47) | 634.96 (440.24 to 869.81) | 611.83 (427.90 to 835.57) | 617.44 (434.40 to 832.87) | 625.45 (444.56 to 844.90) | 574.85 (408.58 to 771.61) | 511.30 (366.23 to 680.39) | 491.38 (351.67 to 651.35) |
| India | Male | Prevalence | 1995.04 (1829.21 to 2164.46) | 6277.53 (5724.66 to 6921.87) | 8785.01 (8052.51 to 9568.64) | 11689.64 (10774.00 to 12655.21) | 13883.99 (12848.20 to 14973.71) | 15297.43 (14183.03 to 16489.14) | 16600.11 (15414.59 to 17869.71) | 17909.09 (16651.60 to 19281.76) | 18926.78 (17603.25 to 20368.19) | 19958.87 (18576.78 to 21440.52) | 21006.01 (19554.11 to 22545.23) | 21997.21 (20484.48 to 23606.77) | 23435.45 (21836.54 to 25137.65) | 23567.84 (21904.03 to 25311.09) | 23133.77 (21477.95 to 24892.40) | 24117.47 (22413.79 to 25922.00) | 25273.13 (23479.65 to 27159.33) | 23559.94 (21838.52 to 25365.23) | 21115.35 (19414.73 to 22881.35) | 20661.78 (18821.92 to 22599.59) |
| Indonesia | Both | DALY rates | 12.40 (8.45 to 17.97) | 107.56 (62.53 to 174.20) | 176.09 (111.53 to 261.70) | 253.82 (167.63 to 364.31) | 302.36 (201.49 to 427.73) | 336.85 (224.20 to 474.24) | 370.14 (245.56 to 513.30) | 394.22 (264.79 to 547.95) | 399.42 (268.57 to 551.55) | 398.70 (266.59 to 544.62) | 402.32 (272.76 to 546.81) | 402.30 (274.61 to 546.91) | 405.37 (276.33 to 545.38) | 400.51 (275.52 to 540.47) | 384.57 (267.54 to 513.92) | 367.14 (255.19 to 485.71) | 347.57 (242.15 to 462.25) | 339.88 (238.29 to 450.57) | 317.09 (223.34 to 419.47) | 275.71 (196.06 to 359.70) |
| Indonesia | Both | Prevalence | 2058.74 (698.36 to 4989.43) | 5671.02 (2300.68 to 13117.31) | 8320.57 (3438.02 to 19217.30) | 11309.19 (4729.22 to 26296.35) | 13641.10 (5597.55 to 32363.12) | 15391.99 (6290.16 to 36549.25) | 16752.56 (6903.35 to 39347.64) | 17815.11 (7371.83 to 41684.56) | 18559.80 (7626.18 to 43004.64) | 19104.33 (7719.57 to 44784.58) | 19616.52 (7770.62 to 46691.31) | 20113.42 (7786.03 to 48534.26) | 20756.56 (7924.15 to 50369.19) | 21050.81 (8037.41 to 51193.85) | 20941.23 (8000.31 to 50973.43) | 20827.78 (7938.73 to 50486.34) | 20606.42 (7676.87 to 50391.48) | 20422.67 (7494.48 to 49846.92) | 20272.32 (7315.90 to 50579.95) | 19859.06 (6732.45 to 52269.67) |
| Indonesia | Female | DALY rates | 14.59 (9.88 to 21.02) | 27.92 (18.83 to 40.78) | 33.08 (22.03 to 46.79) | 40.12 (26.87 to 56.52) | 47.48 (31.90 to 68.96) | 53.73 (36.56 to 78.57) | 59.05 (39.08 to 83.61) | 63.07 (42.75 to 91.63) | 66.40 (45.20 to 95.14) | 68.76 (46.52 to 99.57) | 71.02 (48.60 to 101.07) | 73.12 (49.56 to 105.27) | 76.10 (50.99 to 107.86) | 76.41 (51.98 to 108.29) | 74.84 (51.58 to 105.05) | 72.82 (50.79 to 99.77) | 70.62 (49.39 to 97.04) | 68.10 (47.09 to 93.74) | 66.18 (47.20 to 92.04) | 63.45 (43.98 to 86.84) |
| Indonesia | Female | Prevalence | 2080.34 (719.64 to 5015.17) | 5031.22 (1669.61 to 12528.42) | 7162.20 (2281.88 to 18169.92) | 9568.93 (2988.26 to 24717.33) | 11584.90 (3575.74 to 30296.70) | 13125.11 (4061.16 to 34450.06) | 14246.31 (4440.92 to 37051.84) | 15139.68 (4745.67 to 39400.30) | 15844.93 (4933.74 to 40459.30) | 16373.54 (4945.56 to 42912.40) | 16834.11 (4877.45 to 44613.97) | 17302.71 (4895.63 to 46554.85) | 17908.04 (5048.23 to 48222.32) | 18195.82 (5165.49 to 48971.18) | 18135.56 (5166.22 to 48895.14) | 18069.26 (5130.56 to 48497.39) | 17962.86 (5004.50 to 48592.06) | 17793.73 (4795.45 to 48339.61) | 17773.88 (4692.94 to 49196.78) | 17701.62 (4318.99 to 51103.26) |
| Indonesia | Male | DALY rates | 10.32 (6.81 to 14.85) | 182.92 (102.04 to 308.19) | 312.72 (195.78 to 469.97) | 469.10 (308.44 to 679.59) | 577.79 (384.97 to 821.90) | 643.44 (426.73 to 906.94) | 684.75 (453.74 to 951.41) | 712.80 (476.48 to 990.15) | 729.80 (487.68 to 1017.47) | 739.68 (491.53 to 1019.44) | 746.79 (507.49 to 1028.16) | 750.73 (511.44 to 1030.05) | 753.29 (512.48 to 1024.14) | 745.14 (510.16 to 1005.78) | 730.32 (503.85 to 989.70) | 716.58 (496.19 to 960.09) | 699.51 (490.73 to 940.11) | 684.41 (478.11 to 914.53) | 673.52 (471.18 to 888.68) | 656.34 (469.53 to 872.01) |
| Indonesia | Male | Prevalence | 2038.23 (679.92 to 4965.01) | 6276.43 (2807.24 to 13732.22) | 9427.22 (4436.71 to 20356.74) | 13062.19 (6365.47 to 27963.66) | 15863.06 (7720.13 to 34675.31) | 17846.79 (8699.99 to 39019.97) | 19287.08 (9329.97 to 42157.94) | 20389.03 (9851.90 to 43993.85) | 21253.16 (10252.37 to 45422.23) | 21926.54 (10512.55 to 47002.90) | 22509.49 (10616.56 to 49022.83) | 23088.47 (10740.97 to 50633.75) | 23766.42 (10947.85 to 52637.67) | 24086.59 (11089.65 to 53378.81) | 24073.26 (11189.41 to 53293.48) | 24102.89 (11278.26 to 52847.76) | 23965.82 (11203.69 to 52678.16) | 23755.28 (11039.77 to 51890.46) | 23821.38 (11043.23 to 52544.77) | 23727.85 (10840.29 to 54150.74) |
| Kenya | Both | DALY rates | 12.23 (8.24 to 17.45) | 116.85 (73.29 to 179.67) | 176.00 (114.36 to 260.16) | 235.74 (157.55 to 336.51) | 268.84 (178.28 to 382.08) | 296.67 (198.37 to 418.52) | 335.97 (223.35 to 466.16) | 365.55 (244.79 to 512.79) | 384.70 (259.29 to 539.20) | 399.41 (270.76 to 556.61) | 414.16 (281.32 to 579.16) | 432.53 (295.25 to 600.59) | 441.05 (302.88 to 608.76) | 432.79 (298.35 to 594.42) | 430.11 (298.51 to 589.27) | 426.88 (297.33 to 576.78) | 410.59 (287.43 to 550.39) | 394.23 (278.66 to 525.12) | 363.67 (258.43 to 482.98) | 318.17 (227.43 to 421.63) |
| Kenya | Both | Prevalence | 2048.76 (626.41 to 5349.85) | 5884.85 (2238.83 to 14300.68) | 8544.45 (3263.53 to 20689.92) | 11487.74 (4419.61 to 27706.09) | 13681.67 (5240.16 to 32329.05) | 15431.38 (6019.84 to 36200.59) | 17183.65 (6873.69 to 39727.79) | 18568.06 (7520.96 to 42742.91) | 19543.69 (8027.08 to 44145.80) | 20466.27 (8378.72 to 46644.82) | 21458.19 (8715.44 to 48925.65) | 22624.88 (9126.85 to 51551.13) | 23305.30 (9250.44 to 52948.72) | 23111.76 (8964.75 to 52876.10) | 22931.33 (8846.85 to 52718.56) | 22691.62 (8697.08 to 52388.16) | 22621.28 (8719.53 to 51999.84) | 22606.15 (8958.25 to 50864.58) | 22157.83 (8704.55 to 49610.31) | 21490.89 (8109.39 to 48829.22) |
| Kenya | Female | DALY rates | 14.37 (9.75 to 20.57) | 28.79 (19.29 to 41.45) | 35.75 (24.52 to 51.06) | 46.56 (31.44 to 66.78) | 59.46 (40.41 to 86.07) | 73.48 (49.16 to 106.71) | 88.93 (59.40 to 128.13) | 101.57 (68.30 to 147.81) | 111.83 (74.33 to 163.35) | 118.79 (79.14 to 173.65) | 127.96 (85.48 to 184.87) | 138.52 (92.60 to 202.05) | 142.43 (95.52 to 204.03) | 136.25 (92.67 to 194.75) | 130.46 (87.63 to 187.74) | 122.28 (82.92 to 172.79) | 117.99 (80.68 to 164.51) | 118.24 (80.96 to 162.92) | 110.49 (76.21 to 153.58) | 96.94 (67.58 to 132.87) |
| Kenya | Female | Prevalence | 2071.30 (640.00 to 5382.11) | 5173.65 (1510.40 to 13645.78) | 7399.87 (2078.26 to 19602.84) | 9973.82 (2811.25 to 26306.98) | 12152.07 (3557.21 to 31336.19) | 13985.79 (4265.88 to 35467.44) | 15689.02 (4976.49 to 39551.60) | 16945.50 (5410.78 to 42677.10) | 17811.36 (5768.28 to 43820.76) | 18519.21 (5780.70 to 46009.67) | 19425.46 (6003.83 to 48254.56) | 20395.99 (6214.31 to 50291.66) | 20747.86 (6202.79 to 51035.31) | 20429.41 (5957.66 to 50575.10) | 20100.81 (5864.33 to 50293.60) | 19553.19 (5537.75 to 49090.72) | 19474.52 (5465.20 to 49336.62) | 19558.75 (5637.41 to 49205.39) | 19080.83 (5454.33 to 47938.42) | 18393.70 (4940.78 to 46627.54) |
| Kenya | Male | DALY rates | 10.15 (6.76 to 14.48) | 203.83 (122.72 to 323.97) | 315.51 (203.79 to 469.65) | 431.28 (284.22 to 617.63) | 497.65 (326.35 to 706.07) | 539.04 (357.40 to 762.47) | 585.75 (390.17 to 823.45) | 626.29 (418.37 to 873.68) | 652.47 (432.68 to 910.16) | 681.96 (457.87 to 946.91) | 708.51 (480.59 to 984.25) | 738.30 (504.44 to 1027.09) | 760.53 (520.43 to 1046.03) | 751.96 (519.77 to 1024.89) | 734.65 (511.81 to 995.17) | 718.17 (502.42 to 968.33) | 706.71 (494.11 to 946.28) | 694.72 (490.15 to 924.46) | 682.63 (483.70 to 902.77) | 675.55 (479.65 to 886.73) |
| Kenya | Male | Prevalence | 2026.74 (612.10 to 5318.35) | 6587.32 (2908.36 to 15051.69) | 9682.96 (4391.41 to 21857.66) | 13052.53 (6037.40 to 29152.22) | 15353.24 (7093.59 to 33565.33) | 17001.24 (7935.57 to 37136.68) | 18694.77 (8758.30 to 40498.36) | 20170.64 (9417.65 to 43504.55) | 21243.60 (10038.23 to 45436.61) | 22426.70 (10610.68 to 47374.87) | 23548.82 (11089.43 to 50187.63) | 24942.86 (11847.69 to 52734.83) | 26041.39 (12375.33 to 55095.95) | 25998.76 (12145.63 to 55441.04) | 25808.04 (11828.52 to 55349.81) | 25692.95 (11664.27 to 55264.91) | 25805.88 (12051.49 to 54748.96) | 25924.08 (12594.35 to 53229.72) | 26034.22 (12853.04 to 52769.41) | 26494.26 (13173.14 to 53095.43) |
| Kiribati | Both | DALY rates | 19.12 (12.31 to 27.17) | 411.11 (272.66 to 577.96) | 493.89 (336.63 to 697.00) | 549.94 (371.82 to 766.42) | 622.69 (420.66 to 871.12) | 705.99 (478.00 to 974.18) | 784.81 (542.09 to 1105.88) | 865.06 (589.76 to 1238.08) | 949.88 (645.40 to 1355.44) | 1020.95 (696.70 to 1431.45) | 1091.88 (738.11 to 1541.70) | 1160.73 (785.33 to 1642.24) | 1244.21 (840.59 to 1733.38) | 1251.87 (859.93 to 1764.69) | 1206.49 (822.22 to 1676.14) | 1162.80 (788.29 to 1615.56) | 1133.81 (773.64 to 1583.73) | 1106.31 (761.73 to 1523.93) | 1061.22 (735.66 to 1469.92) | 1009.24 (698.59 to 1384.24) |
| Kiribati | Both | Prevalence | 5466.18 (260.27 to 10685.67) | 17021.22 (3560.99 to 30353.74) | 23968.61 (4402.90 to 43524.36) | 31732.77 (5156.05 to 58513.54) | 38647.16 (5953.77 to 71483.75) | 43851.43 (6861.17 to 80954.03) | 47832.31 (7745.31 to 88018.01) | 51235.00 (8580.89 to 93895.83) | 54235.41 (9445.76 to 98294.50) | 56711.68 (10251.17 to 100000.00) | 58799.62 (10975.16 to 100000.00) | 60618.06 (11827.49 to 100000.00) | 62421.39 (12816.41 to 100000.00) | 63087.32 (13136.13 to 100000.00) | 62960.24 (12983.99 to 100000.00) | 62840.28 (12779.26 to 100000.00) | 62802.34 (12723.15 to 100000.00) | 62754.52 (12657.16 to 100000.00) | 62633.09 (12488.02 to 100000.00) | 62460.96 (12238.99 to 100000.00) |
| Kiribati | Female | DALY rates | 22.43 (14.05 to 32.70) | 57.19 (37.52 to 85.51) | 93.00 (61.49 to 134.52) | 162.54 (104.81 to 243.56) | 260.40 (167.80 to 392.18) | 358.72 (231.61 to 532.45) | 450.97 (291.32 to 654.86) | 536.20 (348.38 to 774.08) | 618.19 (399.73 to 926.81) | 692.43 (445.11 to 1017.15) | 773.93 (499.79 to 1167.48) | 853.93 (553.45 to 1227.47) | 946.81 (610.61 to 1376.33) | 978.79 (633.69 to 1432.82) | 961.07 (627.13 to 1401.60) | 943.06 (613.53 to 1364.63) | 923.46 (602.83 to 1331.23) | 908.94 (597.82 to 1318.79) | 885.16 (582.77 to 1290.87) | 862.58 (579.25 to 1216.66) |
| Kiribati | Female | Prevalence | 5497.52 (297.92 to 10716.48) | 14147.23 (754.68 to 27597.02) | 20723.42 (1194.81 to 40327.13) | 28614.40 (1970.70 to 55324.66) | 35744.16 (3064.93 to 68542.03) | 41085.65 (4143.65 to 78260.50) | 45185.03 (5100.64 to 85509.51) | 48645.62 (5987.22 to 91517.00) | 51672.70 (6826.97 to 96635.86) | 54303.21 (7664.58 to 100000.00) | 56671.06 (8526.42 to 100000.00) | 58770.62 (9504.60 to 100000.00) | 60735.32 (10576.15 to 100000.00) | 61558.20 (11068.55 to 100000.00) | 61556.86 (11050.40 to 100000.00) | 61557.47 (11062.82 to 100000.00) | 61558.00 (11078.22 to 100000.00) | 61556.51 (11034.12 to 100000.00) | 61557.50 (11060.59 to 100000.00) | 61557.38 (11055.54 to 100000.00) |
| Kiribati | Male | DALY rates | 16.01 (9.37 to 25.88) | 748.51 (495.75 to 1044.61) | 872.23 (591.85 to 1235.89) | 926.18 (631.53 to 1300.77) | 997.97 (677.26 to 1377.42) | 1074.89 (736.47 to 1470.65) | 1149.75 (788.73 to 1585.70) | 1222.51 (834.56 to 1727.93) | 1290.94 (888.25 to 1814.29) | 1357.13 (931.18 to 1930.98) | 1424.22 (980.50 to 2000.02) | 1496.74 (1037.89 to 2135.18) | 1578.42 (1094.92 to 2200.56) | 1601.64 (1110.52 to 2248.69) | 1571.42 (1101.11 to 2183.37) | 1540.63 (1077.78 to 2185.37) | 1513.34 (1062.41 to 2112.09) | 1483.74 (1047.63 to 2047.47) | 1456.35 (1038.20 to 1991.54) | 1428.83 (1024.21 to 1938.23) |
| Kiribati | Male | Prevalence | 5436.73 (228.26 to 10656.72) | 19761.05 (6083.45 to 33108.62) | 27031.28 (7495.24 to 46630.31) | 34761.22 (8099.41 to 61498.85) | 41654.35 (8995.69 to 74366.19) | 46789.39 (9806.19 to 83765.49) | 50726.19 (10576.17 to 90724.12) | 54049.42 (11330.28 to 96470.61) | 56870.50 (12091.18 to 100000.00) | 59176.32 (12832.53 to 100000.00) | 61024.51 (13613.38 to 100000.00) | 62641.40 (14414.56 to 100000.00) | 64316.08 (15492.09 to 100000.00) | 65045.93 (15915.22 to 100000.00) | 65047.13 (15935.31 to 100000.00) | 65045.99 (15933.18 to 100000.00) | 65047.44 (15933.85 to 100000.00) | 65045.53 (15946.89 to 100000.00) | 65046.95 (15957.44 to 100000.00) | 65045.95 (15917.43 to 100000.00) |
| Lao People's Democratic Republic | Both | DALY rates | 9.79 (6.02 to 14.94) | 37.77 (19.80 to 69.37) | 43.93 (23.27 to 78.28) | 52.59 (28.89 to 93.13) | 60.98 (33.93 to 104.63) | 68.93 (37.95 to 118.32) | 75.61 (41.62 to 127.56) | 80.95 (45.12 to 138.62) | 85.25 (48.54 to 140.56) | 87.93 (49.52 to 146.96) | 90.25 (53.05 to 149.80) | 93.51 (55.31 to 153.12) | 100.54 (58.07 to 161.25) | 102.15 (59.91 to 163.77) | 96.94 (57.83 to 155.08) | 93.94 (56.86 to 147.83) | 86.78 (51.52 to 138.49) | 68.45 (42.27 to 105.77) | 61.45 (38.61 to 92.90) | 52.64 (33.66 to 78.02) |
| Lao People's Democratic Republic | Both | Prevalence | 599.99 (118.19 to 2622.23) | 1628.42 (351.37 to 6765.36) | 2273.21 (438.24 to 9758.26) | 3023.50 (543.87 to 13218.66) | 3672.81 (638.05 to 16186.99) | 4169.36 (726.83 to 18380.27) | 4529.78 (800.83 to 19932.47) | 4816.97 (857.72 to 21184.79) | 5060.87 (908.48 to 22240.42) | 5269.55 (939.34 to 23182.24) | 5462.73 (976.66 to 24038.35) | 5673.58 (1021.01 to 24941.39) | 5944.80 (1098.63 to 26006.48) | 6063.22 (1128.35 to 26495.55) | 6037.36 (1101.76 to 26477.44) | 6018.85 (1084.43 to 26463.20) | 5967.93 (1035.26 to 26425.99) | 5811.50 (881.62 to 26308.53) | 5758.59 (834.55 to 26267.55) | 5687.11 (768.66 to 26214.78) |
| Lao People's Democratic Republic | Female | DALY rates | 11.48 (6.38 to 18.13) | 20.86 (12.12 to 33.42) | 21.42 (12.37 to 33.64) | 22.81 (13.86 to 35.50) | 24.00 (13.90 to 37.68) | 24.51 (14.98 to 38.68) | 24.68 (15.44 to 37.30) | 24.97 (15.21 to 38.51) | 25.42 (15.18 to 38.92) | 25.81 (16.13 to 38.58) | 25.99 (15.42 to 39.64) | 26.05 (15.74 to 39.33) | 26.33 (16.26 to 39.47) | 26.15 (16.48 to 39.13) | 25.69 (15.23 to 38.35) | 25.30 (15.96 to 36.75) | 24.84 (15.24 to 37.00) | 24.66 (16.32 to 35.64) | 24.01 (15.12 to 34.82) | 23.30 (15.15 to 33.52) |
| Lao People's Democratic Republic | Female | Prevalence | 616.95 (135.01 to 2641.17) | 1498.52 (259.57 to 6700.20) | 2096.50 (295.88 to 9660.28) | 2787.37 (338.86 to 13075.41) | 3378.55 (372.70 to 15997.95) | 3812.54 (399.10 to 18141.17) | 4117.92 (418.01 to 19649.02) | 4364.64 (432.82 to 20865.41) | 4572.83 (445.85 to 21891.89) | 4759.40 (456.76 to 22812.99) | 4928.82 (466.99 to 23647.46) | 5106.37 (478.30 to 24522.89) | 5310.95 (491.91 to 25531.44) | 5406.62 (496.07 to 26002.18) | 5406.62 (497.24 to 26002.26) | 5406.55 (496.88 to 26002.19) | 5406.59 (496.68 to 26002.50) | 5406.55 (497.34 to 26001.84) | 5406.58 (496.28 to 26002.29) | 5406.61 (496.14 to 26003.16) |
| Lao People's Democratic Republic | Male | DALY rates | 8.16 (4.60 to 13.02) | 54.17 (24.08 to 112.50) | 66.10 (29.83 to 129.27) | 84.34 (40.45 to 161.05) | 103.38 (52.10 to 188.77) | 118.30 (58.18 to 214.52) | 129.51 (66.15 to 231.62) | 139.96 (72.26 to 251.03) | 148.22 (78.15 to 250.79) | 156.06 (81.32 to 269.86) | 162.41 (92.18 to 282.94) | 169.08 (93.02 to 288.48) | 176.84 (98.06 to 297.47) | 179.58 (99.84 to 297.72) | 175.40 (97.52 to 293.65) | 173.89 (98.13 to 282.34) | 171.09 (93.53 to 283.46) | 168.01 (95.45 to 280.73) | 164.96 (92.51 to 269.64) | 161.94 (92.72 to 270.54) |
| Lao People's Democratic Republic | Male | Prevalence | 583.53 (99.79 to 2603.85) | 1754.40 (424.70 to 6828.54) | 2447.24 (540.82 to 9854.76) | 3275.28 (700.45 to 13371.41) | 4010.10 (863.98 to 16403.67) | 4566.02 (1003.27 to 18646.06) | 4965.75 (1114.98 to 20232.51) | 5293.86 (1203.64 to 21521.51) | 5574.58 (1287.99 to 22607.29) | 5829.05 (1363.76 to 23587.22) | 6062.20 (1430.88 to 24477.23) | 6309.02 (1509.23 to 25410.24) | 6596.63 (1602.53 to 26494.99) | 6732.12 (1639.99 to 26998.17) | 6731.89 (1644.03 to 27000.68) | 6732.01 (1642.36 to 27000.15) | 6732.08 (1641.82 to 27002.48) | 6732.12 (1643.08 to 27005.76) | 6731.80 (1642.34 to 27000.94) | 6732.07 (1641.95 to 27003.12) |
| Liberia | Both | DALY rates | 14.76 (9.45 to 22.16) | 245.57 (155.64 to 357.13) | 386.92 (256.89 to 539.00) | 448.38 (299.80 to 620.63) | 463.27 (310.19 to 637.21) | 478.45 (324.96 to 663.00) | 517.43 (357.87 to 713.86) | 549.64 (376.48 to 761.23) | 573.86 (392.09 to 796.15) | 593.92 (409.42 to 825.89) | 606.95 (417.51 to 840.25) | 633.32 (438.06 to 877.52) | 639.11 (441.53 to 879.11) | 653.83 (457.75 to 894.93) | 653.79 (457.55 to 883.29) | 642.89 (453.74 to 877.04) | 624.75 (443.48 to 831.43) | 594.52 (421.42 to 794.50) | 557.01 (397.09 to 737.34) | 484.18 (350.33 to 646.82) |
| Liberia | Both | Prevalence | 3586.78 (610.98 to 9404.20) | 11049.31 (3410.17 to 26448.60) | 16274.74 (5228.76 to 38424.22) | 21494.85 (6328.04 to 51455.94) | 25728.44 (7078.51 to 62500.38) | 28926.20 (7652.87 to 70769.61) | 31348.99 (8318.96 to 76668.73) | 33338.54 (8845.84 to 81507.96) | 35012.60 (9310.27 to 85622.92) | 36410.83 (9693.36 to 89100.15) | 37698.62 (10051.71 to 92392.96) | 39123.03 (10520.52 to 95915.62) | 40591.31 (10843.23 to 98447.66) | 41382.20 (11146.45 to 99313.27) | 41471.51 (11232.94 to 99333.98) | 41491.62 (11275.25 to 99336.75) | 41426.96 (11193.43 to 99319.05) | 41262.08 (11025.78 to 99277.77) | 41033.87 (10786.59 to 99215.07) | 40483.65 (10193.81 to 99061.87) |
| Liberia | Female | DALY rates | 17.30 (10.67 to 26.12) | 38.39 (24.67 to 58.59) | 53.05 (34.04 to 78.47) | 75.98 (49.96 to 111.01) | 104.23 (68.08 to 153.55) | 130.97 (84.65 to 188.21) | 153.75 (101.35 to 220.74) | 174.15 (115.38 to 251.12) | 193.32 (128.75 to 280.60) | 210.41 (140.11 to 303.87) | 229.01 (151.51 to 338.06) | 248.47 (161.78 to 360.48) | 271.99 (180.05 to 383.23) | 280.97 (187.91 to 403.63) | 277.43 (187.31 to 398.43) | 273.20 (183.28 to 386.28) | 269.04 (178.25 to 383.34) | 264.56 (176.39 to 371.72) | 258.61 (171.91 to 370.77) | 251.21 (167.63 to 350.85) |
| Liberia | Female | Prevalence | 3621.78 (635.19 to 9458.28) | 9355.37 (1590.17 to 24532.27) | 13532.78 (2283.57 to 35523.92) | 18433.14 (3161.04 to 48304.10) | 22766.50 (4012.54 to 59465.11) | 26055.40 (4721.58 to 67828.89) | 28346.71 (5246.37 to 73597.19) | 30251.14 (5717.60 to 78345.46) | 31888.81 (6129.27 to 82402.89) | 33278.41 (6482.64 to 85851.37) | 34600.98 (6844.39 to 89165.38) | 35964.56 (7222.06 to 92614.35) | 37580.10 (7704.44 to 96702.98) | 38288.56 (7926.43 to 98481.11) | 38288.30 (7920.70 to 98473.42) | 38288.42 (7922.66 to 98466.96) | 38288.44 (7931.79 to 98466.53) | 38288.55 (7926.65 to 98474.34) | 38288.26 (7924.84 to 98472.23) | 38288.48 (7928.79 to 98465.03) |
| Liberia | Male | DALY rates | 12.28 (7.25 to 19.68) | 451.33 (281.82 to 667.18) | 712.17 (470.58 to 989.74) | 841.35 (559.91 to 1181.14) | 865.22 (575.22 to 1180.75) | 882.95 (600.13 to 1232.99) | 899.11 (611.04 to 1239.75) | 914.77 (620.15 to 1277.65) | 926.10 (634.06 to 1288.42) | 936.20 (635.85 to 1302.94) | 943.42 (644.87 to 1301.42) | 953.67 (653.73 to 1319.72) | 965.75 (669.81 to 1339.45) | 961.52 (674.36 to 1327.25) | 945.06 (649.51 to 1273.56) | 924.79 (650.39 to 1264.79) | 908.88 (641.50 to 1213.15) | 891.14 (628.98 to 1201.52) | 872.33 (621.51 to 1150.47) | 850.38 (616.70 to 1133.21) |
| Liberia | Male | Prevalence | 3552.52 (587.49 to 9351.27) | 12731.68 (5079.68 to 28175.78) | 18945.86 (7894.00 to 41000.67) | 24725.69 (9718.00 to 54776.35) | 29044.36 (10435.11 to 65898.40) | 32268.03 (11002.64 to 74192.86) | 34499.85 (11476.82 to 79892.28) | 36340.69 (11831.24 to 84583.14) | 37904.08 (12205.60 to 88603.47) | 39206.52 (12506.74 to 91999.69) | 40456.39 (12837.33 to 95266.41) | 41752.14 (13194.45 to 98663.58) | 43270.53 (13564.60 to 100000.00) | 43935.19 (13727.10 to 100000.00) | 43935.15 (13726.50 to 100000.00) | 43934.20 (13772.87 to 100000.00) | 43933.87 (13723.72 to 100000.00) | 43935.11 (13741.41 to 100000.00) | 43935.03 (13755.99 to 100000.00) | 43934.09 (13766.08 to 100000.00) |
| Madagascar | Both | DALY rates | 11.81 (7.79 to 17.35) | 110.32 (65.76 to 184.45) | 188.32 (119.89 to 283.30) | 287.48 (191.42 to 412.90) | 358.72 (239.64 to 508.39) | 401.67 (265.86 to 563.05) | 427.64 (287.48 to 587.75) | 439.16 (291.91 to 608.45) | 443.58 (304.22 to 621.83) | 441.95 (304.67 to 610.84) | 442.49 (302.80 to 608.28) | 450.50 (310.57 to 621.68) | 457.84 (320.23 to 617.27) | 466.93 (319.72 to 626.73) | 466.24 (323.69 to 626.81) | 447.52 (310.46 to 601.38) | 422.46 (295.25 to 564.60) | 417.29 (297.05 to 553.59) | 406.52 (289.11 to 545.34) | 350.29 (252.01 to 464.56) |
| Madagascar | Both | Prevalence | 2087.65 (697.73 to 4418.92) | 6076.12 (2410.25 to 12255.64) | 9069.96 (3731.29 to 17998.25) | 12598.09 (5349.96 to 24716.49) | 15494.25 (6689.20 to 30335.26) | 17562.83 (7614.56 to 34398.38) | 18986.93 (8217.67 to 37252.18) | 20056.18 (8616.73 to 39465.26) | 20938.95 (8917.71 to 41328.43) | 21663.93 (9117.09 to 42910.65) | 22368.94 (9347.79 to 44404.36) | 23167.59 (9649.88 to 46031.31) | 24073.05 (9986.49 to 47887.81) | 24581.39 (10215.14 to 48832.42) | 24650.39 (10303.86 to 48917.09) | 24563.58 (10219.72 to 48846.94) | 24409.86 (10062.37 to 48680.45) | 24449.56 (10098.43 to 48724.95) | 24411.97 (10061.78 to 48671.29) | 23952.94 (9602.45 to 48203.47) |
| Madagascar | Female | DALY rates | 13.82 (8.66 to 20.77) | 28.14 (18.10 to 43.81) | 33.86 (20.97 to 52.50) | 42.07 (26.62 to 61.38) | 50.19 (32.27 to 74.63) | 57.27 (36.45 to 83.99) | 62.52 (41.07 to 89.50) | 66.86 (43.62 to 98.00) | 71.78 (46.92 to 106.71) | 75.27 (50.24 to 109.31) | 78.86 (51.03 to 114.59) | 83.67 (54.67 to 122.78) | 87.55 (58.22 to 123.25) | 89.58 (61.79 to 125.35) | 88.07 (58.84 to 124.46) | 86.38 (58.48 to 121.77) | 85.02 (56.51 to 120.57) | 83.22 (55.59 to 118.35) | 81.05 (55.48 to 113.01) | 79.05 (53.84 to 109.05) |
| Madagascar | Female | Prevalence | 2113.93 (714.95 to 4455.31) | 5411.02 (1780.44 to 11489.01) | 7813.01 (2528.12 to 16663.56) | 10585.48 (3403.41 to 22621.37) | 12968.05 (4169.37 to 27727.02) | 14725.74 (4740.95 to 31483.14) | 15976.46 (5152.84 to 34149.58) | 16984.55 (5487.90 to 36298.08) | 17852.70 (5779.56 to 38137.68) | 18609.24 (6035.53 to 39741.77) | 19310.91 (6274.07 to 41229.26) | 20048.67 (6526.12 to 42788.98) | 20894.77 (6820.07 to 44585.25) | 21295.48 (6960.93 to 45426.69) | 21295.52 (6960.31 to 45430.36) | 21295.37 (6960.13 to 45425.34) | 21295.41 (6959.94 to 45428.88) | 21295.41 (6959.30 to 45424.26) | 21295.57 (6960.40 to 45426.87) | 21295.48 (6957.55 to 45425.83) |
| Madagascar | Male | DALY rates | 9.86 (5.68 to 15.40) | 191.17 (104.74 to 319.11) | 341.41 (212.15 to 521.56) | 537.46 (355.99 to 776.32) | 685.23 (456.99 to 973.30) | 759.08 (500.19 to 1063.98) | 796.49 (533.32 to 1103.87) | 817.24 (539.36 to 1133.95) | 826.30 (561.89 to 1160.91) | 831.25 (571.33 to 1158.39) | 829.47 (566.56 to 1130.41) | 826.21 (563.34 to 1142.96) | 822.07 (571.37 to 1120.15) | 812.97 (552.96 to 1092.86) | 798.29 (557.08 to 1080.58) | 782.56 (542.96 to 1054.21) | 767.54 (533.43 to 1028.13) | 750.44 (530.21 to 988.36) | 739.07 (524.54 to 993.15) | 722.15 (517.85 to 956.21) |
| Madagascar | Male | Prevalence | 2062.13 (681.01 to 4383.58) | 6730.46 (2971.69 to 12966.28) | 10315.75 (4949.05 to 19336.88) | 14648.13 (7380.80 to 26772.48) | 18167.74 (9320.31 to 33118.45) | 20507.18 (10497.08 to 37437.40) | 22028.17 (11170.48 to 40394.66) | 23175.60 (11650.86 to 42683.38) | 24115.85 (12026.73 to 44612.91) | 24907.06 (12369.77 to 46275.02) | 25623.26 (12613.44 to 47783.26) | 26361.94 (12848.72 to 49352.06) | 27199.28 (13098.81 to 51136.28) | 27594.69 (13204.09 to 51955.60) | 27596.08 (13238.14 to 51978.58) | 27595.57 (13240.90 to 52021.23) | 27594.81 (13231.63 to 52005.61) | 27595.03 (13226.05 to 52016.55) | 27596.05 (13228.57 to 51986.17) | 27596.34 (13238.11 to 52011.64) |
| Malawi | Both | DALY rates | 13.14 (8.41 to 19.98) | 194.21 (121.46 to 290.59) | 327.04 (217.84 to 470.37) | 425.99 (285.06 to 599.00) | 452.88 (305.15 to 631.05) | 472.66 (322.47 to 657.46) | 490.66 (334.74 to 675.99) | 499.38 (343.40 to 679.37) | 507.93 (347.81 to 705.86) | 517.61 (356.90 to 713.01) | 520.43 (354.47 to 709.13) | 522.69 (359.03 to 723.00) | 525.71 (364.31 to 721.79) | 522.50 (363.35 to 712.16) | 507.56 (353.41 to 685.71) | 490.36 (341.16 to 664.50) | 483.14 (339.41 to 656.97) | 475.65 (340.80 to 643.31) | 449.36 (322.39 to 596.25) | 394.71 (285.77 to 521.81) |
| Malawi | Both | Prevalence | 2963.71 (605.30 to 7946.59) | 9304.37 (2807.03 to 23004.12) | 13903.04 (4529.73 to 33871.96) | 18805.81 (6176.91 to 45869.05) | 22487.31 (7045.41 to 55420.57) | 25172.20 (7669.06 to 62404.49) | 27169.33 (8184.35 to 67513.47) | 28699.87 (8524.39 to 71523.16) | 30018.66 (8845.75 to 74986.71) | 31199.04 (9172.33 to 78039.80) | 32249.22 (9397.13 to 80836.55) | 33346.06 (9653.15 to 83766.24) | 34591.04 (9938.30 to 87124.84) | 35178.71 (10085.08 to 88700.58) | 35129.04 (10026.73 to 88653.09) | 35075.10 (9983.96 to 88604.15) | 35108.21 (10008.12 to 88631.19) | 35137.44 (10046.68 to 88664.88) | 34982.60 (9876.23 to 88505.17) | 34554.01 (9425.30 to 88089.20) |
| Malawi | Female | DALY rates | 15.48 (9.54 to 23.71) | 34.74 (22.32 to 52.56) | 45.44 (29.50 to 69.55) | 63.07 (40.33 to 91.14) | 81.65 (52.61 to 118.54) | 99.40 (65.55 to 143.39) | 114.10 (74.71 to 164.64) | 127.48 (84.28 to 183.14) | 138.60 (92.52 to 204.97) | 151.03 (97.96 to 222.32) | 162.46 (108.03 to 232.23) | 175.19 (115.12 to 260.07) | 192.12 (123.00 to 280.56) | 196.52 (131.50 to 280.60) | 192.92 (130.99 to 274.94) | 189.58 (126.78 to 277.93) | 184.73 (125.33 to 268.35) | 180.17 (123.44 to 256.15) | 176.07 (120.20 to 248.20) | 171.27 (116.94 to 236.91) |
| Malawi | Female | Prevalence | 2996.92 (626.06 to 7998.56) | 8005.13 (1617.73 to 21484.40) | 11610.38 (2312.39 to 31234.62) | 15844.15 (3164.27 to 42606.46) | 19469.88 (3935.70 to 52262.20) | 22134.87 (4528.64 to 59294.58) | 24087.18 (4984.39 to 64408.62) | 25662.88 (5358.04 to 68513.87) | 26996.14 (5688.93 to 71993.49) | 28174.40 (5995.57 to 75056.84) | 29281.73 (6282.43 to 77938.17) | 30440.02 (6591.59 to 80938.95) | 31780.78 (6957.64 to 84390.98) | 32405.47 (7137.77 to 85994.81) | 32405.82 (7139.92 to 86002.97) | 32405.72 (7136.51 to 86000.81) | 32405.55 (7134.12 to 86000.19) | 32405.66 (7142.12 to 85997.24) | 32405.58 (7129.88 to 85999.00) | 32405.37 (7131.56 to 86000.72) |
| Malawi | Male | DALY rates | 10.83 (6.03 to 17.42) | 355.14 (219.60 to 538.66) | 609.80 (403.72 to 882.47) | 801.87 (537.24 to 1134.89) | 858.31 (577.50 to 1198.56) | 872.09 (592.53 to 1204.54) | 878.19 (598.85 to 1216.63) | 887.93 (602.78 to 1210.04) | 892.62 (604.32 to 1243.86) | 894.46 (607.91 to 1235.67) | 898.68 (607.80 to 1234.29) | 900.29 (615.82 to 1255.06) | 904.12 (622.86 to 1247.16) | 897.60 (619.26 to 1233.83) | 882.00 (608.83 to 1185.80) | 861.63 (595.03 to 1160.71) | 843.31 (588.80 to 1152.52) | 825.43 (581.69 to 1099.02) | 808.56 (566.08 to 1082.28) | 791.60 (561.98 to 1052.25) |
| Malawi | Male | Prevalence | 2930.94 (584.82 to 7895.29) | 10615.42 (4107.24 to 24445.97) | 16205.11 (6855.41 to 36432.06) | 21873.27 (9264.11 to 49135.01) | 25782.74 (10347.80 to 58869.92) | 28422.51 (11028.80 to 65732.48) | 30341.19 (11476.77 to 70708.69) | 31872.81 (11829.99 to 74667.17) | 33166.85 (12091.56 to 78104.39) | 34308.50 (12281.78 to 81106.41) | 35384.86 (12503.75 to 83899.16) | 36503.88 (12775.66 to 86838.77) | 37778.83 (13120.98 to 90228.47) | 38369.96 (13295.98 to 91818.60) | 38369.74 (13262.76 to 91810.95) | 38369.98 (13289.68 to 91821.56) | 38370.31 (13277.19 to 91810.88) | 38371.30 (13277.24 to 91827.36) | 38369.67 (13259.75 to 91804.21) | 38370.67 (13274.22 to 91805.21) |
| Malaysia | Both | DALY rates | 10.45 (6.43 to 15.65) | 40.96 (21.67 to 73.98) | 51.13 (27.92 to 93.16) | 63.98 (33.56 to 111.23) | 77.99 (43.09 to 132.50) | 89.08 (49.11 to 149.86) | 99.23 (57.41 to 164.86) | 107.37 (61.91 to 174.13) | 114.83 (67.37 to 185.72) | 121.61 (70.50 to 198.07) | 124.46 (72.74 to 197.84) | 126.59 (78.24 to 200.65) | 133.22 (80.08 to 209.68) | 130.05 (78.76 to 198.57) | 127.17 (79.02 to 197.78) | 125.04 (75.62 to 190.17) | 121.51 (75.36 to 188.61) | 120.13 (73.87 to 185.77) | 105.24 (65.93 to 160.87) | 106.28 (66.94 to 162.41) |
| Malaysia | Both | Prevalence | 743.28 (150.74 to 2848.18) | 1950.44 (447.23 to 7290.16) | 2753.98 (568.88 to 10488.75) | 3702.62 (732.83 to 14202.08) | 4528.32 (898.26 to 17349.73) | 5144.69 (1035.25 to 19664.97) | 5594.31 (1145.24 to 21321.81) | 5960.85 (1239.71 to 22664.47) | 6282.64 (1332.53 to 23816.85) | 6557.67 (1417.79 to 24794.38) | 6812.13 (1481.72 to 25736.98) | 7046.34 (1532.52 to 26639.27) | 7363.50 (1620.10 to 27767.03) | 7473.53 (1634.77 to 28242.20) | 7474.28 (1634.50 to 28247.89) | 7474.25 (1635.53 to 28247.27) | 7462.92 (1624.43 to 28228.53) | 7471.68 (1634.90 to 28240.97) | 7360.96 (1547.11 to 28110.98) | 7396.78 (1575.36 to 28150.69) |
| Malaysia | Female | DALY rates | 12.33 (7.30 to 19.11) | 21.16 (12.18 to 33.13) | 22.51 (13.28 to 35.14) | 23.99 (14.83 to 36.03) | 25.06 (14.90 to 37.95) | 26.16 (16.15 to 38.67) | 26.71 (15.78 to 40.75) | 27.49 (16.37 to 42.14) | 27.74 (16.74 to 41.03) | 28.19 (16.95 to 42.48) | 28.04 (17.38 to 41.90) | 28.75 (18.35 to 42.50) | 29.12 (18.82 to 42.35) | 28.74 (18.20 to 42.84) | 27.98 (17.36 to 41.18) | 27.70 (17.83 to 40.28) | 26.84 (17.19 to 38.46) | 26.23 (16.88 to 37.63) | 25.89 (16.39 to 38.16) | 24.75 (16.04 to 35.52) |
| Malaysia | Female | Prevalence | 760.95 (171.62 to 2864.89) | 1796.26 (335.28 to 7003.54) | 2533.04 (403.06 to 10115.84) | 3384.31 (484.12 to 13709.60) | 4108.03 (553.99 to 16761.70) | 4635.13 (604.95 to 18983.06) | 5010.47 (642.34 to 20563.63) | 5313.54 (671.45 to 21839.73) | 5570.89 (696.64 to 22922.42) | 5789.74 (718.23 to 23844.59) | 6007.98 (739.37 to 24761.84) | 6218.83 (761.38 to 25647.24) | 6473.14 (786.85 to 26716.50) | 6591.02 (799.44 to 27212.92) | 6591.02 (798.35 to 27212.84) | 6591.02 (799.53 to 27212.24) | 6591.01 (798.91 to 27212.73) | 6591.02 (799.69 to 27212.69) | 6591.04 (800.11 to 27213.18) | 6590.97 (798.68 to 27211.72) |
| Malaysia | Male | DALY rates | 8.67 (4.65 to 14.05) | 59.73 (26.80 to 118.37) | 78.65 (36.39 to 157.60) | 103.49 (51.16 to 191.33) | 131.36 (66.16 to 236.35) | 152.55 (79.05 to 268.74) | 171.36 (94.05 to 293.03) | 186.20 (102.88 to 311.27) | 198.39 (111.45 to 329.23) | 209.58 (116.78 to 351.69) | 218.67 (122.82 to 360.03) | 228.43 (132.72 to 373.73) | 240.95 (138.30 to 383.05) | 243.48 (140.24 to 383.87) | 237.99 (142.69 to 377.51) | 233.78 (139.01 to 362.84) | 229.93 (137.27 to 374.12) | 225.65 (134.21 to 364.57) | 218.64 (133.69 to 350.92) | 213.98 (129.69 to 337.83) |
| Malaysia | Male | Prevalence | 726.61 (133.56 to 2832.43) | 2096.63 (543.31 to 7595.44) | 2966.51 (737.66 to 10886.02) | 4017.14 (1007.18 to 14642.67) | 4952.16 (1276.99 to 17866.87) | 5658.65 (1487.52 to 20267.48) | 6174.94 (1659.27 to 21985.12) | 6599.71 (1794.10 to 23382.08) | 6965.45 (1941.84 to 24579.86) | 7280.80 (2064.14 to 25595.03) | 7597.91 (2191.00 to 26600.94) | 7907.67 (2310.15 to 27589.76) | 8284.88 (2451.87 to 28789.30) | 8461.63 (2521.28 to 29335.61) | 8461.02 (2524.72 to 29339.67) | 8460.94 (2524.99 to 29342.13) | 8461.46 (2518.21 to 29332.02) | 8461.24 (2530.07 to 29328.62) | 8461.28 (2525.61 to 29330.29) | 8461.26 (2521.57 to 29332.15) |
| Maldives | Both | DALY rates | 9.92 (6.20 to 15.15) | 37.67 (20.07 to 68.91) | 44.82 (23.36 to 78.49) | 54.73 (29.01 to 95.43) | 63.75 (36.09 to 109.09) | 72.30 (40.26 to 125.96) | 80.03 (44.92 to 137.03) | 86.31 (47.44 to 147.23) | 91.97 (51.02 to 155.26) | 96.28 (55.61 to 158.62) | 103.10 (59.49 to 170.07) | 111.94 (61.92 to 186.56) | 120.01 (69.79 to 196.86) | 121.42 (72.37 to 201.05) | 120.11 (69.93 to 195.39) | 122.31 (70.81 to 198.42) | 128.40 (76.04 to 213.86) | 136.31 (81.15 to 225.60) | 136.75 (79.99 to 214.93) | 129.53 (76.65 to 210.23) |
| Maldives | Both | Prevalence | 631.50 (566.83 to 700.42) | 1660.40 (1448.75 to 1882.43) | 2325.18 (2037.36 to 2618.74) | 3110.52 (2736.61 to 3468.44) | 3776.04 (3336.50 to 4207.56) | 4283.40 (3794.31 to 4771.25) | 4659.08 (4126.36 to 5182.65) | 4958.89 (4396.36 to 5506.82) | 5226.47 (4638.99 to 5797.85) | 5454.56 (4841.34 to 6037.89) | 5686.32 (5050.04 to 6294.19) | 5948.48 (5282.13 to 6581.77) | 6232.58 (5538.52 to 6886.63) | 6363.34 (5659.89 to 7030.82) | 6369.12 (5660.78 to 7038.93) | 6409.82 (5693.35 to 7087.77) | 6483.90 (5754.07 to 7181.13) | 6575.89 (5827.15 to 7297.28) | 6610.37 (5855.01 to 7345.84) | 6565.47 (5820.51 to 7287.19) |
| Maldives | Female | DALY rates | 11.70 (7.20 to 18.05) | 20.72 (12.09 to 31.67) | 21.67 (12.37 to 33.42) | 22.93 (13.69 to 35.47) | 23.60 (14.89 to 35.60) | 24.29 (15.18 to 36.31) | 24.58 (15.32 to 38.80) | 25.12 (15.77 to 39.53) | 25.75 (15.06 to 38.14) | 25.72 (15.28 to 38.32) | 25.95 (16.10 to 39.00) | 26.54 (16.18 to 40.15) | 26.50 (16.02 to 38.80) | 26.27 (16.34 to 40.08) | 26.32 (16.03 to 39.56) | 25.31 (15.71 to 36.82) | 24.92 (16.14 to 36.00) | 24.54 (15.74 to 35.32) | 24.07 (14.92 to 35.49) | 23.00 (14.99 to 32.91) |
| Maldives | Female | Prevalence | 649.42 (585.70 to 717.88) | 1527.93 (1370.77 to 1698.67) | 2143.07 (1914.95 to 2393.61) | 2860.29 (2548.17 to 3202.56) | 3460.04 (3079.15 to 3877.91) | 3901.75 (3469.00 to 4376.20) | 4218.86 (3750.23 to 4732.94) | 4466.77 (3968.67 to 5012.81) | 4687.29 (4163.99 to 5261.64) | 4879.89 (4334.04 to 5478.90) | 5053.32 (4488.39 to 5673.47) | 5232.68 (4646.06 to 5875.96) | 5440.61 (4830.25 to 6110.55) | 5541.03 (4918.21 to 6222.64) | 5541.01 (4918.39 to 6222.01) | 5541.06 (4918.61 to 6223.88) | 5541.05 (4919.18 to 6222.95) | 5541.00 (4918.25 to 6223.95) | 5541.06 (4918.67 to 6223.42) | 5541.03 (4918.26 to 6223.56) |
| Maldives | Male | DALY rates | 8.18 (4.47 to 13.36) | 53.87 (24.45 to 111.98) | 67.09 (31.78 to 132.01) | 86.69 (41.63 to 165.69) | 106.11 (50.43 to 198.10) | 121.81 (61.34 to 225.08) | 134.74 (69.51 to 239.78) | 144.22 (74.98 to 256.75) | 152.96 (83.13 to 269.29) | 161.60 (86.76 to 276.89) | 168.96 (91.78 to 287.70) | 175.01 (93.02 to 299.63) | 183.53 (102.03 to 310.37) | 185.06 (105.68 to 314.23) | 181.77 (101.56 to 300.89) | 178.53 (101.03 to 298.15) | 175.55 (100.74 to 296.56) | 172.76 (100.41 to 290.95) | 168.67 (97.18 to 269.32) | 165.72 (96.34 to 273.16) |
| Maldives | Male | Prevalence | 614.06 (551.23 to 682.00) | 1787.09 (1512.82 to 2204.73) | 2500.38 (2137.78 to 2983.42) | 3361.96 (2898.11 to 3924.64) | 4109.39 (3562.15 to 4725.23) | 4677.03 (4074.81 to 5326.65) | 5093.42 (4452.53 to 5798.13) | 5424.55 (4745.78 to 6162.04) | 5723.06 (5009.42 to 6500.79) | 5986.58 (5246.06 to 6795.80) | 6226.65 (5454.68 to 7052.78) | 6477.03 (5681.99 to 7334.38) | 6770.63 (5942.27 to 7643.15) | 6913.30 (6072.68 to 7784.23) | 6913.45 (6070.18 to 7786.50) | 6913.42 (6070.85 to 7784.88) | 6913.46 (6075.11 to 7782.14) | 6913.38 (6072.17 to 7782.20) | 6913.38 (6070.20 to 7792.29) | 6913.60 (6070.17 to 7785.87) |
| Mali | Both | DALY rates | 16.99 (10.63 to 24.92) | 365.03 (238.19 to 517.52) | 469.95 (318.93 to 642.73) | 506.43 (346.05 to 699.32) | 543.67 (371.68 to 759.92) | 593.36 (404.10 to 831.71) | 653.41 (449.00 to 920.94) | 719.62 (492.01 to 1009.53) | 777.06 (536.14 to 1101.36) | 832.75 (563.17 to 1177.99) | 883.64 (607.15 to 1230.54) | 945.41 (650.67 to 1315.01) | 1011.74 (688.94 to 1441.85) | 1036.55 (702.95 to 1458.19) | 1016.22 (703.31 to 1416.61) | 993.79 (690.76 to 1368.27) | 971.45 (680.54 to 1343.86) | 937.21 (655.54 to 1275.11) | 894.45 (626.77 to 1223.02) | 829.53 (589.47 to 1134.28) |
| Mali | Both | Prevalence | 4723.52 (1769.67 to 8911.68) | 15248.87 (7398.61 to 26296.90) | 21674.48 (10305.77 to 37773.55) | 28504.58 (12922.37 to 50632.05) | 34384.87 (15319.31 to 61621.33) | 38913.99 (17377.59 to 69874.04) | 42370.34 (19078.11 to 75959.41) | 45335.14 (20663.35 to 81055.24) | 47860.54 (22050.45 to 85389.16) | 50098.82 (23334.67 to 89203.65) | 52211.27 (24518.61 to 92861.49) | 54492.81 (25861.40 to 96655.20) | 57002.78 (27367.65 to 99206.22) | 58235.90 (28128.25 to 99857.27) | 58229.50 (28136.90 to 99870.23) | 58226.82 (28116.79 to 99880.26) | 58175.24 (28052.94 to 99866.87) | 58040.21 (27888.53 to 99863.71) | 57832.29 (27626.55 to 99853.33) | 57451.91 (27170.33 to 99841.38) |
| Mali | Female | DALY rates | 19.96 (12.76 to 29.88) | 49.88 (32.17 to 72.56) | 77.46 (49.69 to 113.65) | 129.06 (84.26 to 190.51) | 197.68 (130.95 to 293.37) | 265.73 (177.33 to 386.32) | 328.84 (215.90 to 486.70) | 386.00 (250.51 to 570.67) | 440.45 (280.03 to 644.64) | 494.25 (322.37 to 727.16) | 549.57 (350.00 to 815.75) | 610.92 (395.31 to 892.50) | 681.86 (437.06 to 988.36) | 712.24 (459.60 to 1036.11) | 699.38 (456.37 to 1032.13) | 688.41 (450.00 to 978.28) | 675.75 (447.99 to 971.38) | 665.71 (435.73 to 959.63) | 651.85 (436.31 to 932.88) | 632.80 (422.13 to 893.93) |
| Mali | Female | Prevalence | 4764.57 (1799.71 to 8965.53) | 12672.17 (4769.06 to 23872.19) | 18464.89 (7001.15 to 34742.21) | 25433.71 (9832.01 to 47645.33) | 31596.51 (12511.78 to 58851.56) | 36298.53 (14703.93 to 67226.48) | 39780.12 (16371.69 to 73321.62) | 42684.57 (17815.21 to 78342.04) | 45202.30 (19107.54 to 82644.67) | 47430.79 (20298.91 to 86444.76) | 49571.92 (21486.65 to 90071.73) | 51854.93 (22766.31 to 93987.57) | 54410.74 (24245.89 to 98377.47) | 55668.74 (25002.61 to 99707.31) | 55669.88 (25028.46 to 99734.62) | 55670.59 (25023.71 to 99755.44) | 55668.71 (24991.44 to 99733.64) | 55669.27 (25001.16 to 99741.22) | 55668.65 (24979.12 to 99741.79) | 55669.37 (24993.55 to 99753.74) |
| Mali | Male | DALY rates | 14.12 (8.37 to 21.28) | 670.71 (436.08 to 956.74) | 847.04 (568.31 to 1159.09) | 894.29 (603.54 to 1230.31) | 949.01 (638.64 to 1334.19) | 1003.51 (677.91 to 1395.90) | 1049.89 (717.10 to 1462.77) | 1094.67 (749.14 to 1513.74) | 1139.54 (773.46 to 1573.78) | 1179.88 (808.76 to 1631.87) | 1220.33 (844.40 to 1691.85) | 1269.01 (872.84 to 1757.81) | 1327.71 (916.79 to 1879.85) | 1345.23 (936.75 to 1865.96) | 1319.41 (932.43 to 1815.75) | 1286.76 (909.56 to 1795.62) | 1266.90 (904.89 to 1724.07) | 1239.28 (893.09 to 1667.18) | 1213.48 (873.93 to 1646.80) | 1185.59 (850.87 to 1602.78) |
| Mali | Male | Prevalence | 4683.80 (1740.59 to 8859.58) | 17748.17 (9806.11 to 28638.76) | 24758.21 (13305.96 to 40707.26) | 31660.81 (16203.18 to 53701.79) | 37651.56 (18707.27 to 64866.24) | 42188.16 (20767.82 to 73188.39) | 45534.46 (22315.97 to 79181.63) | 48314.81 (23749.70 to 84105.31) | 50723.09 (25049.74 to 88344.59) | 52834.87 (26266.05 to 92065.23) | 54871.24 (27433.11 to 95679.68) | 57044.90 (28674.98 to 99236.08) | 59485.44 (30154.06 to 100000.00) | 60679.32 (30879.92 to 100000.00) | 60678.87 (30895.71 to 100000.00) | 60679.12 (30863.17 to 100000.00) | 60679.64 (30864.18 to 100000.00) | 60678.17 (30868.31 to 100000.00) | 60677.45 (30861.62 to 100000.00) | 60678.12 (30883.83 to 100000.00) |
| Marshall Islands | Both | DALY rates | 9.79 (5.95 to 14.48) | 28.71 (15.75 to 51.90) | 29.70 (15.89 to 54.20) | 30.54 (15.71 to 56.72) | 31.41 (17.00 to 57.27) | 32.29 (17.33 to 56.97) | 33.06 (17.72 to 60.20) | 33.34 (17.42 to 60.39) | 34.24 (18.20 to 62.38) | 34.52 (18.10 to 65.02) | 34.39 (18.32 to 61.84) | 34.11 (18.09 to 62.60) | 33.67 (18.75 to 61.71) | 32.30 (17.39 to 57.18) | 31.71 (17.65 to 54.66) | 31.17 (16.91 to 54.68) | 27.37 (15.40 to 46.80) | 25.85 (15.30 to 42.07) | 24.78 (14.89 to 39.81) | 24.90 (15.06 to 40.98) |
| Marshall Islands | Both | Prevalence | 219.14 (99.34 to 359.00) | 557.42 (235.12 to 929.64) | 699.40 (250.44 to 1214.53) | 867.22 (266.02 to 1550.53) | 1009.41 (277.55 to 1843.08) | 1114.79 (287.92 to 2057.77) | 1192.39 (297.52 to 2213.90) | 1254.23 (305.23 to 2339.56) | 1309.92 (313.09 to 2449.73) | 1353.38 (318.27 to 2538.95) | 1393.39 (321.81 to 2621.99) | 1435.43 (324.22 to 2710.50) | 1483.32 (327.59 to 2811.82) | 1497.94 (321.67 to 2852.11) | 1496.06 (320.65 to 2850.90) | 1497.87 (321.63 to 2852.23) | 1472.06 (300.04 to 2824.44) | 1459.60 (289.12 to 2813.06) | 1456.76 (287.35 to 2809.32) | 1462.65 (292.05 to 2815.45) |
| Marshall Islands | Female | DALY rates | 11.49 (6.80 to 18.26) | 19.37 (11.79 to 29.77) | 19.53 (11.70 to 30.62) | 19.38 (11.66 to 29.53) | 19.61 (11.86 to 31.81) | 19.57 (11.54 to 29.90) | 19.68 (11.37 to 32.30) | 19.47 (11.19 to 29.51) | 19.34 (11.31 to 30.16) | 19.30 (11.02 to 29.96) | 19.32 (10.77 to 29.85) | 19.33 (11.50 to 29.51) | 19.29 (11.51 to 30.11) | 18.74 (10.72 to 28.12) | 18.58 (10.83 to 28.25) | 18.30 (10.99 to 27.81) | 17.72 (10.46 to 26.99) | 17.57 (11.17 to 25.27) | 16.95 (9.95 to 25.39) | 16.56 (10.38 to 24.62) |
| Marshall Islands | Female | Prevalence | 236.59 (116.77 to 376.66) | 486.83 (197.88 to 824.98) | 621.98 (204.00 to 1109.91) | 781.76 (210.69 to 1447.12) | 918.53 (215.54 to 1734.50) | 1018.30 (219.20 to 1945.12) | 1088.81 (221.79 to 2094.53) | 1145.63 (223.78 to 2214.05) | 1193.40 (226.05 to 2315.15) | 1235.01 (227.39 to 2403.21) | 1274.32 (228.21 to 2486.90) | 1316.01 (229.83 to 2573.97) | 1362.91 (231.41 to 2672.90) | 1384.38 (233.21 to 2718.15) | 1384.42 (232.50 to 2718.39) | 1384.40 (232.98 to 2718.93) | 1384.41 (232.90 to 2718.72) | 1384.41 (232.39 to 2718.83) | 1384.41 (232.85 to 2717.96) | 1384.38 (232.82 to 2718.89) |
| Marshall Islands | Male | DALY rates | 8.21 (4.65 to 13.13) | 37.61 (17.28 to 79.51) | 39.34 (17.00 to 83.35) | 41.20 (17.36 to 86.89) | 43.24 (18.99 to 88.22) | 45.07 (20.94 to 89.54) | 45.73 (21.20 to 94.35) | 46.11 (20.14 to 93.80) | 46.78 (21.37 to 94.21) | 47.54 (21.17 to 100.59) | 47.79 (21.53 to 94.70) | 47.83 (21.29 to 100.94) | 47.53 (21.62 to 98.03) | 47.32 (21.82 to 96.09) | 46.73 (20.59 to 90.66) | 45.43 (20.03 to 90.87) | 44.05 (19.55 to 87.60) | 43.90 (19.70 to 85.57) | 42.84 (20.81 to 86.24) | 42.07 (19.75 to 83.78) |
| Marshall Islands | Male | Prevalence | 202.86 (81.67 to 344.16) | 624.76 (251.53 to 1088.33) | 772.79 (272.55 to 1358.14) | 948.88 (299.46 to 1696.66) | 1100.48 (319.04 to 1984.74) | 1211.71 (336.88 to 2203.67) | 1290.53 (350.37 to 2357.80) | 1354.25 (361.92 to 2483.45) | 1407.96 (370.24 to 2591.05) | 1454.75 (377.78 to 2683.64) | 1499.20 (384.60 to 2771.74) | 1546.30 (391.98 to 2864.58) | 1599.34 (400.14 to 2967.71) | 1623.74 (402.68 to 3015.41) | 1623.70 (402.14 to 3016.08) | 1623.69 (403.35 to 3015.05) | 1623.70 (403.66 to 3016.56) | 1623.75 (403.24 to 3015.72) | 1623.68 (403.92 to 3014.89) | 1623.77 (403.57 to 3015.71) |
| Micronesia (Federated States of) | Both | DALY rates | 12.70 (7.83 to 19.10) | 108.57 (60.96 to 182.44) | 183.50 (115.26 to 278.78) | 278.88 (180.88 to 404.18) | 347.97 (232.97 to 491.82) | 397.62 (266.21 to 562.06) | 428.28 (285.86 to 592.99) | 454.15 (305.18 to 622.25) | 476.20 (320.10 to 655.18) | 463.18 (310.57 to 636.00) | 444.25 (300.49 to 614.75) | 453.87 (312.91 to 627.83) | 463.35 (317.71 to 637.13) | 453.47 (313.59 to 620.06) | 420.45 (291.66 to 565.50) | 375.61 (262.70 to 504.66) | 345.82 (240.78 to 465.05) | 321.37 (226.64 to 427.75) | 299.87 (213.39 to 395.46) | 259.63 (185.16 to 340.45) |
| Micronesia (Federated States of) | Both | Prevalence | 2150.62 (524.76 to 5656.57) | 5847.94 (1835.19 to 14383.74) | 8705.27 (2914.35 to 21041.18) | 12036.43 (4246.43 to 28679.45) | 14845.83 (5262.57 to 35284.95) | 16909.83 (5994.89 to 40118.36) | 18316.77 (6470.62 to 43440.49) | 19483.44 (6875.02 to 46246.59) | 20487.06 (7253.92 to 48612.09) | 21065.69 (7313.95 to 50355.39) | 21568.39 (7325.02 to 51976.31) | 22382.24 (7615.06 to 53986.22) | 23271.03 (7914.02 to 56215.34) | 23596.46 (7990.05 to 57149.58) | 23380.58 (7771.27 to 56945.14) | 23044.23 (7438.69 to 56613.96) | 22845.93 (7233.69 to 56427.79) | 22679.44 (7061.21 to 56265.90) | 22538.63 (6920.30 to 56140.15) | 22184.50 (6553.15 to 55793.79) |
| Micronesia (Federated States of) | Female | DALY rates | 14.87 (8.85 to 22.83) | 28.12 (16.69 to 43.02) | 33.47 (20.71 to 50.24) | 40.81 (26.38 to 61.40) | 49.03 (31.98 to 73.98) | 55.11 (34.11 to 82.14) | 59.91 (38.75 to 88.34) | 64.61 (43.57 to 92.50) | 68.19 (42.98 to 100.77) | 71.43 (44.83 to 102.97) | 75.00 (49.14 to 113.26) | 78.53 (51.77 to 111.88) | 82.59 (54.84 to 118.76) | 83.23 (53.77 to 119.57) | 82.28 (54.40 to 118.38) | 80.35 (54.03 to 110.79) | 78.22 (52.06 to 109.49) | 76.67 (51.15 to 107.00) | 75.09 (51.22 to 104.04) | 73.88 (50.29 to 104.84) |
| Micronesia (Federated States of) | Female | Prevalence | 2169.59 (546.25 to 5671.05) | 5203.79 (1248.65 to 13730.78) | 7495.30 (1743.63 to 19890.46) | 10107.01 (2317.67 to 26889.47) | 12408.92 (2833.97 to 33033.13) | 14114.46 (3221.80 to 37571.10) | 15291.26 (3494.35 to 40689.66) | 16276.71 (3726.06 to 43298.89) | 17099.54 (3918.87 to 45472.40) | 17793.04 (4085.51 to 47298.14) | 18461.57 (4244.97 to 49056.72) | 19193.18 (4422.70 to 50977.35) | 19998.86 (4619.22 to 53089.85) | 20368.04 (4712.22 to 54054.89) | 20367.96 (4711.01 to 54054.37) | 20368.04 (4714.84 to 54052.72) | 20367.94 (4708.54 to 54056.09) | 20368.01 (4710.66 to 54052.35) | 20367.97 (4711.68 to 54056.66) | 20367.93 (4711.57 to 54053.34) |
| Micronesia (Federated States of) | Male | DALY rates | 10.70 (6.37 to 16.98) | 182.47 (97.59 to 313.85) | 322.61 (197.04 to 499.54) | 510.08 (325.47 to 739.73) | 658.55 (436.82 to 942.83) | 742.38 (500.83 to 1048.08) | 780.63 (521.93 to 1085.51) | 805.84 (534.94 to 1107.73) | 815.42 (543.39 to 1123.63) | 820.85 (550.04 to 1142.90) | 823.04 (551.52 to 1145.97) | 821.35 (560.34 to 1132.13) | 817.31 (556.76 to 1130.12) | 807.01 (553.94 to 1099.45) | 790.76 (546.64 to 1071.19) | 776.53 (538.38 to 1035.25) | 759.64 (533.43 to 1022.10) | 744.72 (521.67 to 1005.65) | 728.59 (516.11 to 970.19) | 719.21 (509.29 to 942.42) |
| Micronesia (Federated States of) | Male | Prevalence | 2133.12 (507.47 to 5643.19) | 6439.59 (2402.05 to 14923.86) | 9827.16 (3905.93 to 22206.51) | 13910.12 (5897.57 to 30584.16) | 17377.71 (7491.80 to 37803.08) | 19723.56 (8563.02 to 42842.87) | 21210.76 (9183.95 to 46194.31) | 22378.54 (9652.08 to 48946.25) | 23303.46 (10033.94 to 51222.44) | 24053.67 (10268.10 to 53146.69) | 24755.44 (10500.89 to 54971.30) | 25504.48 (10745.50 to 56932.04) | 26312.85 (11000.41 to 59120.80) | 26679.22 (11115.09 to 60104.65) | 26679.48 (11116.56 to 60110.61) | 26677.98 (11132.97 to 60091.64) | 26677.94 (11123.94 to 60095.43) | 26678.57 (11121.90 to 60095.66) | 26678.70 (11130.33 to 60113.98) | 26679.06 (11105.73 to 60100.03) |
| Mozambique | Both | DALY rates | 21.42 (13.85 to 30.63) | 446.39 (298.47 to 619.83) | 501.59 (340.95 to 694.53) | 580.12 (396.15 to 807.93) | 703.60 (474.29 to 985.90) | 871.73 (589.39 to 1254.45) | 1035.53 (696.08 to 1467.22) | 1180.10 (790.53 to 1678.21) | 1332.31 (899.15 to 1895.54) | 1470.20 (989.25 to 2105.01) | 1602.90 (1076.28 to 2282.39) | 1746.06 (1186.85 to 2463.78) | 1916.56 (1299.64 to 2732.82) | 1968.90 (1341.82 to 2788.09) | 1919.13 (1318.28 to 2702.76) | 1872.17 (1285.52 to 2646.07) | 1821.13 (1248.68 to 2534.04) | 1768.87 (1223.59 to 2439.93) | 1694.52 (1175.51 to 2328.02) | 1581.54 (1099.82 to 2179.17) |
| Mozambique | Both | Prevalence | 6315.34 (3565.86 to 9610.22) | 19841.65 (12556.69 to 28580.42) | 27595.58 (17151.13 to 40221.39) | 36778.13 (22490.76 to 54009.39) | 45366.65 (27735.83 to 66678.50) | 52359.30 (32291.21 to 76749.34) | 57718.89 (36000.83 to 84379.76) | 62192.10 (39104.70 to 90726.86) | 66237.27 (42036.98 to 96408.82) | 69788.63 (44665.26 to 99558.97) | 73089.34 (47220.17 to 100000.00) | 76503.26 (49897.63 to 100000.00) | 80339.01 (53088.43 to 100000.00) | 81960.70 (54531.91 to 100000.00) | 81849.71 (54394.51 to 100000.00) | 81786.09 (54289.19 to 100000.00) | 81748.01 (54201.44 to 100000.00) | 81654.83 (54118.15 to 100000.00) | 81366.08 (53746.19 to 100000.00) | 80844.53 (53074.81 to 100000.00) |
| Mozambique | Female | DALY rates | 25.11 (15.71 to 37.25) | 68.34 (44.53 to 100.15) | 121.22 (79.59 to 182.91) | 231.52 (154.94 to 340.33) | 395.57 (253.19 to 564.65) | 570.67 (368.24 to 847.74) | 731.01 (474.95 to 1069.38) | 877.92 (562.38 to 1296.95) | 1017.60 (660.86 to 1472.22) | 1142.38 (742.34 to 1666.71) | 1270.02 (822.11 to 1853.43) | 1400.72 (902.70 to 2048.74) | 1554.46 (1019.21 to 2259.22) | 1610.81 (1059.54 to 2306.84) | 1586.12 (1046.21 to 2274.20) | 1555.87 (1025.13 to 2217.28) | 1517.62 (1002.97 to 2152.81) | 1485.28 (1005.72 to 2114.51) | 1451.12 (977.43 to 2030.46) | 1409.32 (959.75 to 1957.79) |
| Mozambique | Female | Prevalence | 6362.25 (3614.86 to 9656.66) | 16756.70 (9516.86 to 25429.70) | 24500.31 (13984.04 to 37094.66) | 33981.62 (19613.88 to 51251.19) | 42893.06 (25146.07 to 64339.72) | 49987.17 (29817.98 to 74587.20) | 55388.78 (33491.76 to 82321.23) | 59923.69 (36637.44 to 88688.94) | 63910.83 (39520.25 to 94279.16) | 67404.86 (42057.11 to 99153.54) | 70684.59 (44535.74 to 100000.00) | 74052.68 (47082.69 to 100000.00) | 77879.06 (50195.13 to 100000.00) | 79550.20 (51618.31 to 100000.00) | 79551.06 (51618.28 to 100000.00) | 79551.36 (51583.17 to 100000.00) | 79551.63 (51570.64 to 100000.00) | 79550.54 (51604.73 to 100000.00) | 79550.64 (51614.12 to 100000.00) | 79552.45 (51601.90 to 100000.00) |
| Mozambique | Male | DALY rates | 17.78 (11.02 to 26.48) | 823.76 (553.30 to 1148.59) | 888.81 (603.56 to 1241.99) | 976.18 (651.93 to 1352.66) | 1096.75 (756.27 to 1528.98) | 1251.31 (860.84 to 1750.14) | 1409.38 (970.96 to 1974.64) | 1555.36 (1060.91 to 2232.86) | 1698.10 (1172.24 to 2428.13) | 1826.81 (1249.07 to 2642.47) | 1959.54 (1339.81 to 2820.66) | 2101.70 (1439.91 to 2996.37) | 2282.85 (1570.43 to 3273.45) | 2344.54 (1611.62 to 3350.76) | 2301.30 (1582.76 to 3261.91) | 2254.77 (1553.81 to 3193.19) | 2199.89 (1547.34 to 3069.37) | 2150.87 (1513.55 to 2993.57) | 2113.02 (1494.61 to 2901.85) | 2067.40 (1448.92 to 2829.73) |
| Mozambique | Male | Prevalence | 6269.03 (3517.49 to 9564.39) | 22921.01 (15670.50 to 31582.28) | 30746.51 (20351.77 to 43399.69) | 39955.29 (25790.13 to 57146.46) | 48523.84 (31063.41 to 69664.41) | 55350.17 (35339.60 to 79477.83) | 60579.38 (38920.49 to 86905.75) | 65009.12 (41967.11 to 93169.77) | 68941.29 (44794.98 to 98740.11) | 72381.71 (47397.26 to 100000.00) | 75665.77 (49930.54 to 100000.00) | 79026.88 (52560.97 to 100000.00) | 82827.35 (56003.61 to 100000.00) | 84489.41 (57674.98 to 100000.00) | 84487.71 (57691.39 to 100000.00) | 84489.20 (57625.47 to 100000.00) | 84488.87 (57673.55 to 100000.00) | 84489.34 (57674.01 to 100000.00) | 84487.49 (57682.22 to 100000.00) | 84489.58 (57634.07 to 100000.00) |
| Myanmar | Both | DALY rates | 12.76 (8.30 to 19.14) | 125.14 (74.49 to 206.58) | 214.87 (137.63 to 315.17) | 322.97 (212.22 to 459.35) | 390.59 (261.85 to 551.75) | 425.40 (286.76 to 589.07) | 444.24 (297.52 to 609.23) | 454.07 (306.90 to 626.66) | 460.90 (315.45 to 634.85) | 453.98 (307.82 to 629.52) | 453.73 (309.37 to 620.44) | 454.28 (307.34 to 624.40) | 456.91 (320.35 to 623.11) | 437.72 (307.78 to 590.37) | 413.55 (288.48 to 561.71) | 401.10 (279.03 to 534.18) | 384.52 (273.20 to 518.54) | 359.64 (256.60 to 479.94) | 308.76 (221.23 to 411.16) | 229.55 (165.51 to 302.97) |
| Myanmar | Both | Prevalence | 2370.87 (306.06 to 7403.37) | 6677.17 (1422.71 to 19180.13) | 9947.65 (2388.11 to 28079.40) | 13793.49 (3564.83 to 38531.23) | 16858.63 (4402.97 to 47197.96) | 18983.15 (4866.91 to 53433.46) | 20457.86 (5123.15 to 57849.58) | 21605.43 (5286.34 to 61358.31) | 22579.86 (5440.80 to 64324.79) | 23333.19 (5474.79 to 66823.22) | 24099.92 (5539.54 to 69227.22) | 24888.03 (5617.00 to 71698.33) | 25827.30 (5764.15 to 74554.40) | 26154.66 (5697.36 to 75811.67) | 26008.57 (5554.64 to 75670.46) | 25964.56 (5500.77 to 75619.53) | 25879.44 (5413.29 to 75537.30) | 25722.63 (5249.31 to 75370.95) | 25306.29 (4827.34 to 74939.06) | 24593.35 (4107.53 to 74211.60) |
| Myanmar | Female | DALY rates | 15.09 (9.29 to 23.73) | 29.46 (18.15 to 44.99) | 35.70 (22.04 to 51.96) | 45.56 (29.68 to 69.44) | 55.46 (36.23 to 82.86) | 63.69 (40.57 to 95.58) | 71.11 (45.83 to 101.45) | 76.34 (50.60 to 111.48) | 82.09 (53.90 to 119.92) | 86.27 (57.61 to 125.74) | 90.95 (58.91 to 129.25) | 96.04 (63.33 to 138.86) | 101.99 (66.76 to 150.19) | 102.71 (68.80 to 148.24) | 101.53 (68.20 to 148.56) | 98.83 (66.37 to 144.07) | 96.93 (66.50 to 138.69) | 94.61 (64.83 to 133.11) | 92.57 (63.27 to 131.10) | 90.19 (62.71 to 124.87) |
| Myanmar | Female | Prevalence | 2397.22 (326.70 to 7434.71) | 5901.93 (731.70 to 18473.09) | 8495.00 (998.74 to 26731.18) | 11541.36 (1325.64 to 36387.54) | 14128.08 (1617.06 to 44541.88) | 16031.41 (1843.24 to 50512.20) | 17402.88 (2010.78 to 54798.96) | 18511.00 (2151.78 to 58246.80) | 19455.96 (2275.45 to 61179.02) | 20280.69 (2388.92 to 63731.63) | 21063.06 (2495.07 to 66148.85) | 21864.61 (2607.98 to 68617.70) | 22783.16 (2744.34 to 71449.58) | 23226.39 (2806.62 to 72815.84) | 23226.26 (2806.06 to 72818.25) | 23226.32 (2802.17 to 72818.20) | 23226.28 (2803.96 to 72816.70) | 23226.38 (2806.38 to 72822.34) | 23226.39 (2805.64 to 72817.60) | 23226.43 (2805.87 to 72816.11) |
| Myanmar | Male | DALY rates | 10.49 (6.14 to 16.67) | 219.01 (125.02 to 369.42) | 392.22 (246.88 to 579.69) | 608.02 (398.91 to 872.78) | 744.70 (494.11 to 1062.87) | 806.87 (538.99 to 1117.67) | 832.40 (554.38 to 1157.08) | 844.93 (570.85 to 1178.57) | 847.68 (576.88 to 1169.87) | 846.54 (567.86 to 1190.64) | 843.80 (572.83 to 1167.48) | 840.62 (567.57 to 1151.70) | 832.15 (579.35 to 1131.70) | 817.70 (576.47 to 1123.08) | 802.28 (559.72 to 1082.87) | 788.65 (548.11 to 1047.84) | 774.33 (543.78 to 1054.52) | 758.06 (538.19 to 1033.54) | 742.06 (526.71 to 993.32) | 727.24 (519.51 to 961.17) |
| Myanmar | Male | Prevalence | 2345.17 (285.34 to 7372.79) | 7437.75 (2106.04 to 19850.73) | 11385.56 (3745.47 to 29381.72) | 16107.57 (5809.78 to 40721.18) | 19743.92 (7239.54 to 49978.52) | 22096.20 (7972.17 to 56468.33) | 23635.98 (8348.70 to 60977.99) | 24807.37 (8546.68 to 64555.55) | 25769.49 (8655.26 to 67536.75) | 26591.97 (8774.90 to 70123.73) | 27365.13 (8887.13 to 72537.06) | 28148.55 (8975.21 to 75020.55) | 29045.77 (9070.25 to 77837.02) | 29476.02 (9150.99 to 79209.68) | 29474.84 (9136.07 to 79223.81) | 29475.43 (9153.02 to 79211.27) | 29475.72 (9161.71 to 79224.98) | 29475.39 (9145.23 to 79202.42) | 29474.92 (9133.85 to 79191.00) | 29475.21 (9138.31 to 79195.51) |
| Nepal | Both | DALY rates | 11.66 (7.62 to 16.98) | 102.07 (59.37 to 171.26) | 172.15 (104.05 to 260.12) | 261.67 (171.74 to 384.77) | 324.62 (216.43 to 470.59) | 366.39 (242.96 to 514.50) | 398.03 (268.70 to 556.21) | 418.99 (278.99 to 582.47) | 430.78 (291.87 to 599.33) | 442.30 (303.81 to 612.17) | 455.10 (306.64 to 622.28) | 457.19 (314.01 to 621.02) | 452.47 (312.24 to 612.80) | 448.12 (310.65 to 598.55) | 437.17 (304.58 to 582.88) | 418.81 (292.33 to 558.51) | 410.77 (285.25 to 545.69) | 400.41 (283.95 to 535.34) | 365.53 (257.40 to 482.29) | 284.12 (202.34 to 373.00) |
| Nepal | Both | Prevalence | 1962.08 (1682.69 to 2277.54) | 5652.71 (4862.50 to 6481.57) | 8407.72 (7284.98 to 9628.24) | 11673.55 (10208.53 to 13359.48) | 14359.01 (12586.32 to 16376.49) | 16285.02 (14333.13 to 18622.60) | 17684.01 (15586.79 to 20217.66) | 18786.09 (16518.59 to 21482.61) | 19668.52 (17271.08 to 22478.47) | 20467.00 (17933.30 to 23361.20) | 21229.76 (18601.95 to 24210.91) | 21946.86 (19207.96 to 25046.06) | 22697.16 (19815.79 to 25912.77) | 23072.26 (20127.71 to 26345.37) | 23049.12 (20097.39 to 26320.87) | 22948.03 (20003.70 to 26216.80) | 22947.77 (19997.47 to 26218.73) | 22910.84 (19961.41 to 26169.89) | 22645.83 (19702.52 to 25898.89) | 21910.19 (18966.32 to 25142.26) |
| Nepal | Female | DALY rates | 13.75 (8.60 to 20.60) | 27.53 (16.54 to 43.32) | 32.65 (19.68 to 49.38) | 39.58 (25.21 to 58.57) | 46.53 (30.24 to 68.98) | 52.36 (33.25 to 77.03) | 57.57 (36.95 to 83.82) | 61.15 (39.80 to 86.00) | 64.59 (42.83 to 92.81) | 67.30 (44.61 to 99.57) | 70.75 (46.67 to 103.42) | 73.94 (48.60 to 106.45) | 77.47 (52.48 to 109.25) | 77.82 (52.25 to 112.94) | 77.29 (51.21 to 110.89) | 75.50 (51.58 to 108.58) | 73.80 (50.82 to 103.80) | 72.70 (49.07 to 100.08) | 71.35 (47.68 to 98.91) | 69.96 (47.00 to 99.31) |
| Nepal | Female | Prevalence | 1982.39 (1704.67 to 2298.61) | 5052.02 (4329.24 to 5872.87) | 7276.20 (6222.73 to 8472.33) | 9863.41 (8427.47 to 11492.58) | 12084.73 (10323.96 to 14087.13) | 13704.96 (11706.10 to 15973.96) | 14868.19 (12699.62 to 17332.40) | 15810.03 (13503.82 to 18431.48) | 16600.99 (14181.40 to 19351.97) | 17299.59 (14779.40 to 20164.38) | 17955.81 (15342.01 to 20926.73) | 18635.88 (15925.92 to 21714.29) | 19420.80 (16595.98 to 22624.60) | 19790.76 (16913.58 to 23047.39) | 19790.78 (16913.10 to 23052.48) | 19790.85 (16914.06 to 23051.62) | 19790.87 (16914.25 to 23053.69) | 19790.82 (16915.70 to 23049.60) | 19790.75 (16914.41 to 23048.14) | 19790.72 (16914.97 to 23052.90) |
| Nepal | Male | DALY rates | 9.67 (5.66 to 15.09) | 173.14 (95.09 to 299.88) | 305.07 (182.26 to 472.06) | 488.66 (315.85 to 722.20) | 635.36 (419.11 to 922.07) | 718.22 (473.65 to 1013.28) | 760.70 (506.76 to 1064.80) | 785.14 (519.61 to 1088.56) | 798.14 (538.02 to 1124.46) | 803.80 (548.26 to 1104.57) | 806.78 (542.95 to 1096.36) | 803.03 (555.85 to 1094.52) | 800.10 (549.43 to 1097.66) | 790.34 (547.50 to 1058.38) | 774.68 (539.23 to 1029.76) | 762.16 (533.36 to 1023.01) | 747.89 (521.25 to 998.59) | 735.89 (522.20 to 986.78) | 722.05 (507.26 to 951.30) | 708.10 (502.51 to 928.03) |
| Nepal | Male | Prevalence | 1942.72 (1659.51 to 2257.48) | 6225.38 (5244.50 to 7230.30) | 9485.90 (8122.27 to 10899.55) | 13523.68 (11804.18 to 15309.90) | 16900.21 (14953.05 to 19131.72) | 19175.55 (17009.63 to 21763.73) | 20683.56 (18382.22 to 23407.65) | 21831.23 (19438.49 to 24621.97) | 22745.91 (20253.81 to 25660.17) | 23520.37 (20933.21 to 26553.27) | 24225.41 (21548.11 to 27371.79) | 24934.63 (22157.43 to 28140.30) | 25734.35 (22812.28 to 29050.03) | 26104.96 (23161.69 to 29449.83) | 26104.94 (23133.91 to 29475.33) | 26105.52 (23154.92 to 29439.05) | 26106.01 (23176.43 to 29444.06) | 26104.87 (23162.42 to 29468.18) | 26105.92 (23143.06 to 29453.20) | 26106.04 (23148.14 to 29457.55) |
| Niger | Both | DALY rates | 13.13 (8.54 to 19.74) | 203.12 (131.22 to 306.44) | 330.18 (221.25 to 472.12) | 421.89 (277.73 to 586.13) | 447.42 (303.88 to 621.86) | 463.61 (318.87 to 635.47) | 488.14 (329.26 to 669.68) | 512.86 (347.20 to 716.51) | 521.20 (354.09 to 709.20) | 544.44 (376.00 to 743.10) | 560.39 (388.97 to 777.99) | 577.62 (398.67 to 788.71) | 594.35 (411.18 to 816.81) | 587.79 (403.27 to 805.80) | 572.82 (402.41 to 772.82) | 549.98 (386.50 to 750.06) | 504.64 (356.10 to 676.72) | 435.00 (306.33 to 594.39) | 357.01 (250.83 to 482.22) | 313.50 (225.78 to 418.49) |
| Niger | Both | Prevalence | 3024.32 (803.69 to 6455.26) | 9554.91 (3507.18 to 18975.88) | 14136.64 (5530.57 to 27587.50) | 19078.30 (7185.27 to 37415.37) | 22852.07 (8253.56 to 45453.39) | 25580.25 (9047.49 to 51254.81) | 27658.80 (9758.91 to 55556.75) | 29353.31 (10370.49 to 59000.05) | 30691.86 (10793.69 to 61797.20) | 32048.52 (11310.12 to 64494.32) | 33273.69 (11763.72 to 66981.28) | 34532.41 (12229.56 to 69488.26) | 35921.65 (12747.74 to 72273.57) | 36567.38 (12943.34 to 73625.88) | 36536.50 (12912.63 to 73618.66) | 36433.78 (12806.47 to 73491.58) | 36100.15 (12469.10 to 73132.80) | 35548.24 (11894.20 to 72521.72) | 34892.94 (11210.83 to 71820.06) | 34555.06 (10858.60 to 71466.95) |
| Niger | Female | DALY rates | 15.45 (9.47 to 23.35) | 35.14 (21.53 to 53.95) | 46.55 (29.96 to 69.22) | 65.21 (41.59 to 95.53) | 85.20 (56.13 to 122.95) | 103.67 (68.14 to 147.24) | 120.05 (79.79 to 176.81) | 133.55 (88.42 to 196.34) | 146.46 (96.55 to 215.11) | 159.68 (104.88 to 232.26) | 171.98 (111.42 to 250.92) | 185.33 (124.13 to 274.84) | 201.37 (133.45 to 293.88) | 207.50 (138.97 to 300.27) | 204.35 (136.87 to 288.71) | 201.20 (131.29 to 282.57) | 197.66 (133.23 to 280.26) | 194.82 (133.26 to 279.28) | 189.98 (128.96 to 269.80) | 184.80 (126.07 to 257.31) |
| Niger | Female | Prevalence | 3049.67 (828.77 to 6485.46) | 8188.65 (2174.91 to 17490.51) | 11829.34 (3115.44 to 25311.27) | 16173.58 (4277.52 to 34588.00) | 19913.66 (5322.42 to 42511.96) | 22662.20 (6123.46 to 48288.53) | 24664.62 (6730.03 to 52473.28) | 26262.68 (7223.50 to 55785.61) | 27617.19 (7652.92 to 58585.47) | 28872.13 (8061.52 to 61167.80) | 30051.59 (8450.98 to 63576.11) | 31256.20 (8850.14 to 66031.91) | 32606.80 (9316.26 to 68764.47) | 33304.75 (9558.31 to 70162.73) | 33304.64 (9553.94 to 70166.65) | 33304.56 (9556.89 to 70177.17) | 33304.39 (9557.23 to 70175.59) | 33305.01 (9560.03 to 70176.75) | 33304.60 (9556.91 to 70175.15) | 33304.55 (9556.12 to 70169.45) |
| Niger | Male | DALY rates | 10.91 (6.56 to 16.76) | 369.07 (229.51 to 556.35) | 624.85 (416.75 to 892.89) | 812.51 (535.39 to 1142.92) | 863.45 (586.02 to 1196.26) | 878.18 (600.19 to 1208.17) | 887.41 (594.60 to 1214.56) | 895.37 (601.63 to 1266.34) | 898.77 (604.37 to 1232.82) | 903.21 (616.32 to 1234.59) | 907.59 (633.66 to 1268.40) | 911.76 (623.62 to 1243.87) | 915.21 (625.55 to 1263.39) | 906.05 (616.47 to 1234.41) | 887.69 (619.87 to 1193.66) | 869.22 (603.34 to 1194.19) | 855.79 (602.11 to 1150.15) | 836.50 (588.38 to 1139.50) | 820.20 (579.90 to 1101.15) | 801.69 (559.83 to 1069.13) |
| Niger | Male | Prevalence | 3000.05 (779.69 to 6427.64) | 10904.63 (4786.00 to 20415.49) | 16533.80 (7870.42 to 29952.37) | 22259.40 (10451.10 to 40511.77) | 26226.89 (11616.39 to 48882.98) | 28941.13 (12383.01 to 54779.47) | 30906.70 (13018.18 to 59013.25) | 32469.95 (13518.34 to 62342.56) | 33789.80 (13932.49 to 65145.18) | 35010.35 (14315.41 to 67702.61) | 36153.90 (14670.78 to 70128.67) | 37323.04 (15036.15 to 72534.19) | 38628.07 (15467.37 to 75235.29) | 39297.81 (15685.36 to 76612.45) | 39298.21 (15686.84 to 76665.46) | 39297.87 (15683.31 to 76634.32) | 39298.10 (15699.15 to 76646.11) | 39298.14 (15689.73 to 76633.86) | 39297.56 (15703.90 to 76628.71) | 39298.46 (15698.92 to 76646.36) |
| Nigeria | Both | DALY rates | 11.88 (7.98 to 16.76) | 142.23 (88.04 to 217.34) | 234.53 (155.74 to 337.36) | 325.19 (217.84 to 451.34) | 374.85 (250.26 to 516.31) | 408.00 (275.74 to 561.07) | 439.42 (301.24 to 600.23) | 472.78 (325.18 to 650.27) | 505.28 (349.64 to 692.49) | 505.62 (349.17 to 691.58) | 523.92 (363.49 to 713.56) | 540.97 (376.26 to 735.87) | 536.38 (373.09 to 727.17) | 463.34 (323.67 to 625.40) | 430.11 (300.69 to 576.52) | 399.82 (281.60 to 541.14) | 373.99 (264.47 to 500.25) | 348.90 (249.60 to 457.85) | 324.50 (233.22 to 428.94) | 276.09 (201.22 to 363.85) |
| Nigeria | Both | Prevalence | 2429.38 (869.71 to 4717.59) | 7383.73 (3207.39 to 13652.19) | 10886.02 (4990.53 to 19891.33) | 14737.16 (6804.52 to 26655.75) | 17739.33 (7974.41 to 32202.69) | 19983.25 (8867.31 to 36440.32) | 21675.40 (9620.64 to 39807.63) | 23110.37 (10240.63 to 42703.63) | 24226.54 (10801.91 to 44591.12) | 24952.51 (11014.95 to 45353.58) | 26035.94 (11677.96 to 46637.71) | 27249.05 (12325.02 to 48695.17) | 28202.25 (12502.60 to 50138.20) | 28051.24 (12015.27 to 50742.41) | 27723.41 (11712.31 to 51276.90) | 27403.94 (11289.00 to 51250.59) | 27067.79 (11096.81 to 51074.17) | 26706.24 (10897.81 to 50804.93) | 26328.87 (10508.44 to 50356.68) | 25711.80 (9859.25 to 50402.35) |
| Nigeria | Female | DALY rates | 14.02 (9.49 to 20.06) | 29.91 (20.09 to 42.32) | 37.65 (25.88 to 54.19) | 49.35 (33.55 to 70.92) | 62.15 (42.33 to 90.13) | 73.71 (49.99 to 105.16) | 83.00 (55.61 to 119.78) | 90.51 (61.39 to 130.45) | 96.41 (65.32 to 140.97) | 102.23 (69.04 to 146.25) | 108.18 (73.82 to 156.23) | 115.18 (78.11 to 166.77) | 122.02 (83.08 to 174.85) | 123.74 (84.07 to 176.12) | 121.46 (82.85 to 173.41) | 119.33 (80.84 to 168.26) | 116.27 (80.57 to 163.92) | 113.52 (78.24 to 158.85) | 109.58 (75.52 to 152.23) | 105.09 (73.84 to 145.77) |
| Nigeria | Female | Prevalence | 2472.09 (895.16 to 4780.37) | 6463.20 (2275.45 to 12612.41) | 9285.90 (3223.54 to 18314.38) | 12530.76 (4380.37 to 24649.25) | 15300.39 (5394.67 to 30018.70) | 17402.24 (6137.01 to 34475.75) | 18893.84 (6654.02 to 37407.28) | 20033.29 (6999.58 to 39582.13) | 20792.34 (7388.63 to 40664.98) | 21520.04 (7768.44 to 41511.60) | 22415.63 (8253.65 to 43100.33) | 23594.94 (8685.69 to 44982.99) | 24680.74 (8714.28 to 46930.56) | 25173.54 (8863.76 to 49700.19) | 25127.80 (8780.76 to 50359.89) | 25041.00 (8737.04 to 50419.50) | 24901.79 (8596.37 to 50031.06) | 24705.78 (8585.00 to 49310.59) | 24460.09 (8536.81 to 48553.34) | 24197.87 (8295.07 to 48532.42) |
| Nigeria | Male | DALY rates | 9.69 (6.39 to 13.93) | 264.09 (159.70 to 414.38) | 457.56 (302.48 to 665.35) | 640.41 (427.63 to 894.44) | 725.39 (477.67 to 1006.79) | 758.63 (514.17 to 1044.98) | 776.55 (532.39 to 1080.63) | 789.35 (543.89 to 1093.02) | 794.10 (547.94 to 1084.33) | 791.82 (545.87 to 1081.29) | 803.41 (556.31 to 1100.18) | 816.50 (567.76 to 1111.50) | 822.50 (569.84 to 1119.33) | 815.30 (566.58 to 1103.90) | 797.81 (556.56 to 1063.02) | 774.60 (545.05 to 1030.72) | 755.57 (531.20 to 999.98) | 733.00 (521.40 to 969.40) | 711.58 (511.86 to 939.97) | 686.34 (502.21 to 901.91) |
| Nigeria | Male | Prevalence | 2385.78 (843.79 to 4653.51) | 8382.48 (4099.34 to 14709.69) | 12698.66 (6714.35 to 21677.74) | 17258.54 (9464.46 to 29012.50) | 20473.38 (10982.67 to 34656.90) | 22690.48 (11798.70 to 38793.15) | 24306.37 (12462.21 to 42044.47) | 25658.61 (12924.66 to 45378.75) | 26652.49 (13142.49 to 47223.74) | 27387.73 (13144.29 to 48748.50) | 28469.81 (13914.68 to 49360.31) | 29613.68 (14666.22 to 51033.18) | 30633.87 (15055.80 to 51767.87) | 31033.66 (15210.61 to 52296.47) | 30815.50 (15127.65 to 52230.26) | 30561.18 (15008.68 to 52285.96) | 30274.86 (14740.88 to 52618.64) | 29970.67 (14316.64 to 53255.65) | 29694.68 (13788.41 to 53494.71) | 29343.85 (13405.15 to 54339.78) |
| Niue | Both | DALY rates | 11.02 (6.47 to 16.34) | 44.75 (23.36 to 78.92) | 58.41 (31.17 to 102.23) | 80.01 (41.86 to 137.74) | 99.17 (55.67 to 170.31) | 113.34 (65.65 to 185.43) | 123.42 (72.37 to 195.48) | 133.29 (80.01 to 213.32) | 142.59 (87.71 to 225.84) | 146.70 (87.34 to 226.78) | 149.17 (91.46 to 230.89) | 160.98 (101.02 to 245.41) | 170.30 (109.32 to 257.95) | 168.80 (109.10 to 252.27) | 149.92 (97.12 to 222.20) | 122.77 (80.67 to 183.13) | 109.06 (71.68 to 159.63) | 104.74 (69.32 to 151.53) | 92.18 (62.13 to 132.04) | 79.93 (53.75 to 112.35) |
| Niue | Both | Prevalence | 906.51 (260.60 to 2515.77) | 2270.54 (727.39 to 6043.28) | 3220.36 (1001.79 to 8671.42) | 4375.22 (1358.74 to 11791.10) | 5384.79 (1667.51 to 14513.32) | 6122.18 (1896.04 to 16494.76) | 6639.59 (2053.72 to 17879.49) | 7084.24 (2204.98 to 19033.38) | 7471.15 (2340.17 to 20031.01) | 7771.24 (2429.78 to 20857.30) | 8037.52 (2505.28 to 21595.97) | 8403.14 (2662.71 to 22465.62) | 8796.68 (2820.43 to 23458.17) | 8939.62 (2863.29 to 23866.91) | 8796.81 (2731.96 to 23721.14) | 8579.41 (2534.47 to 23506.73) | 8475.88 (2439.89 to 23402.63) | 8456.25 (2422.80 to 23382.08) | 8356.33 (2331.95 to 23286.84) | 8251.68 (2237.67 to 23179.24) |
| Niue | Female | DALY rates | 13.09 (7.05 to 20.05) | 21.99 (13.07 to 33.93) | 23.23 (13.68 to 35.58) | 25.10 (14.87 to 37.57) | 26.77 (16.91 to 42.20) | 28.03 (16.69 to 42.33) | 28.94 (17.87 to 43.79) | 29.15 (17.38 to 44.30) | 29.96 (18.47 to 44.87) | 30.48 (19.14 to 45.61) | 30.64 (19.40 to 46.51) | 31.16 (18.07 to 46.73) | 31.47 (19.25 to 47.11) | 31.37 (19.81 to 48.25) | 31.19 (20.53 to 44.80) | 30.54 (18.47 to 44.78) | 29.91 (19.33 to 43.67) | 29.09 (18.64 to 42.04) | 28.47 (19.09 to 40.04) | 27.73 (17.64 to 41.14) |
| Niue | Female | Prevalence | 927.96 (280.97 to 2539.50) | 2094.98 (572.44 to 5885.34) | 2943.56 (745.35 to 8415.82) | 3939.86 (949.67 to 11383.84) | 4806.16 (1128.69 to 13961.52) | 5434.12 (1259.30 to 15828.60) | 5874.84 (1351.82 to 17138.11) | 6233.39 (1425.29 to 18202.68) | 6543.11 (1490.59 to 19121.06) | 6805.87 (1545.79 to 19901.22) | 7047.72 (1595.13 to 20619.30) | 7304.32 (1650.72 to 21379.15) | 7607.19 (1713.24 to 22277.21) | 7741.58 (1741.68 to 22674.72) | 7741.53 (1741.85 to 22675.18) | 7741.57 (1740.84 to 22675.31) | 7741.56 (1741.53 to 22675.77) | 7741.53 (1742.84 to 22673.73) | 7741.55 (1741.76 to 22675.19) | 7741.53 (1742.59 to 22675.20) |
| Niue | Male | DALY rates | 9.13 (5.09 to 14.57) | 67.33 (30.04 to 132.20) | 89.64 (43.11 to 166.83) | 124.74 (62.70 to 223.32) | 162.44 (84.74 to 287.29) | 193.37 (107.63 to 322.83) | 217.43 (121.04 to 355.30) | 236.26 (138.23 to 387.61) | 253.58 (149.11 to 406.09) | 268.17 (157.44 to 426.58) | 282.00 (168.00 to 447.00) | 295.15 (178.89 to 458.25) | 311.37 (196.49 to 480.48) | 314.94 (198.25 to 476.69) | 309.31 (197.05 to 472.37) | 302.64 (191.01 to 464.81) | 296.36 (188.08 to 447.85) | 290.76 (184.14 to 436.47) | 284.62 (180.76 to 431.06) | 280.60 (178.42 to 414.94) |
| Niue | Male | Prevalence | 887.03 (243.11 to 2494.21) | 2444.79 (897.28 to 6151.23) | 3466.10 (1229.74 to 8834.72) | 4729.91 (1699.38 to 12042.80) | 5890.44 (2180.77 to 14888.82) | 6767.53 (2566.29 to 16990.15) | 7400.60 (2861.84 to 18468.87) | 7925.64 (3095.17 to 19698.66) | 8385.68 (3295.81 to 20761.17) | 8780.25 (3481.32 to 21674.33) | 9146.82 (3645.26 to 22510.63) | 9538.75 (3808.47 to 23404.25) | 10005.32 (4036.85 to 24459.86) | 10213.56 (4146.44 to 24936.23) | 10213.46 (4138.26 to 24920.46) | 10213.46 (4134.18 to 24932.48) | 10213.57 (4144.71 to 24927.03) | 10213.63 (4135.81 to 24923.87) | 10213.21 (4142.14 to 24927.01) | 10213.03 (4146.72 to 24921.25) |
| Palau | Both | DALY rates | 9.39 (5.78 to 14.05) | 15.98 (10.08 to 24.34) | 15.99 (9.92 to 23.96) | 15.70 (9.71 to 24.18) | 15.68 (10.02 to 23.39) | 15.69 (10.21 to 23.51) | 15.66 (9.80 to 23.20) | 15.48 (9.44 to 24.41) | 15.57 (9.64 to 23.46) | 15.23 (9.20 to 22.98) | 15.34 (9.57 to 22.61) | 15.14 (9.08 to 23.68) | 25.02 (13.74 to 44.01) | 24.16 (13.02 to 43.03) | 23.85 (12.85 to 43.08) | 23.27 (12.90 to 40.91) | 22.13 (12.28 to 38.94) | 21.61 (12.49 to 37.46) | 21.17 (12.38 to 37.16) | 18.92 (11.56 to 30.42) |
| Palau | Both | Prevalence | 97.59 (90.91 to 105.46) | 168.55 (156.20 to 185.84) | 174.52 (159.81 to 197.66) | 181.14 (162.93 to 210.62) | 186.28 (165.05 to 221.11) | 190.71 (167.11 to 230.78) | 194.41 (168.93 to 237.76) | 196.98 (169.99 to 243.12) | 199.03 (170.62 to 247.29) | 200.71 (171.85 to 251.07) | 203.72 (173.59 to 255.62) | 205.22 (174.09 to 259.49) | 292.50 (221.01 to 434.13) | 291.44 (220.89 to 430.43) | 292.35 (221.09 to 432.38) | 291.21 (220.81 to 429.71) | 283.92 (220.00 to 410.71) | 283.24 (220.16 to 408.89) | 284.61 (220.17 to 412.36) | 270.30 (216.08 to 365.60) |
| Palau | Female | DALY rates | 10.99 (6.22 to 16.97) | 18.58 (10.89 to 29.20) | 18.77 (10.68 to 28.92) | 18.51 (10.84 to 29.41) | 18.27 (10.69 to 28.01) | 18.55 (10.71 to 28.99) | 18.26 (10.38 to 27.95) | 18.06 (10.29 to 28.60) | 18.10 (10.47 to 27.97) | 17.92 (10.38 to 28.27) | 17.80 (10.09 to 26.98) | 17.74 (10.52 to 28.51) | 17.49 (10.83 to 27.63) | 17.07 (10.17 to 25.72) | 16.82 (10.38 to 25.55) | 16.42 (9.77 to 25.21) | 16.31 (9.60 to 24.38) | 15.99 (10.29 to 24.81) | 15.49 (9.28 to 23.05) | 14.93 (8.78 to 22.06) |
| Palau | Female | Prevalence | 114.20 (105.83 to 124.65) | 195.69 (180.09 to 214.82) | 201.73 (183.31 to 226.52) | 208.62 (186.74 to 239.31) | 214.53 (189.89 to 251.75) | 218.81 (192.36 to 260.78) | 221.84 (193.67 to 267.01) | 224.26 (194.59 to 271.26) | 226.34 (195.44 to 275.59) | 228.17 (196.40 to 279.74) | 229.89 (197.10 to 282.72) | 231.78 (198.03 to 287.58) | 233.82 (199.08 to 291.49) | 234.76 (199.65 to 293.27) | 234.73 (199.60 to 293.75) | 234.72 (199.85 to 294.10) | 234.74 (199.68 to 293.71) | 234.73 (199.92 to 293.59) | 234.71 (199.60 to 293.32) | 234.74 (199.62 to 293.94) |
| Palau | Male | DALY rates | 7.88 (4.34 to 12.88) | 13.50 (7.24 to 22.27) | 13.35 (7.08 to 22.01) | 13.10 (7.14 to 21.14) | 13.40 (7.33 to 21.46) | 13.16 (7.18 to 20.89) | 13.23 (7.10 to 21.84) | 13.04 (6.94 to 21.90) | 13.18 (7.31 to 21.31) | 12.71 (7.12 to 20.75) | 12.82 (7.25 to 19.95) | 12.54 (6.66 to 21.00) | 32.35 (14.42 to 64.07) | 31.55 (13.90 to 66.32) | 30.94 (13.02 to 67.45) | 30.45 (13.45 to 64.63) | 30.01 (12.79 to 63.19) | 29.40 (13.54 to 63.26) | 28.66 (13.22 to 61.22) | 27.92 (12.68 to 55.95) |
| Palau | Male | Prevalence | 82.04 (74.37 to 90.30) | 142.72 (128.78 to 159.60) | 148.71 (132.54 to 171.76) | 155.63 (136.47 to 184.31) | 161.48 (139.84 to 197.51) | 165.78 (141.95 to 206.51) | 168.78 (143.47 to 212.77) | 171.19 (144.66 to 218.29) | 173.25 (145.40 to 222.75) | 175.09 (146.10 to 225.09) | 176.81 (146.91 to 229.01) | 178.67 (148.02 to 232.69) | 349.48 (221.78 to 635.94) | 350.45 (222.47 to 641.73) | 350.46 (222.48 to 639.00) | 350.42 (222.09 to 639.01) | 350.47 (222.37 to 638.93) | 350.45 (222.07 to 638.84) | 350.48 (222.61 to 641.59) | 350.46 (222.21 to 640.58) |
| Papua New Guinea | Both | DALY rates | 19.66 (12.85 to 28.86) | 427.79 (286.29 to 593.55) | 505.42 (343.88 to 704.18) | 566.88 (385.99 to 782.62) | 652.21 (444.09 to 899.10) | 748.36 (507.08 to 1064.28) | 842.82 (572.93 to 1181.68) | 930.17 (630.79 to 1328.17) | 1015.49 (689.97 to 1428.22) | 1094.19 (742.38 to 1546.70) | 1176.58 (803.08 to 1691.90) | 1261.61 (856.83 to 1804.76) | 1353.19 (933.03 to 1900.49) | 1386.50 (951.22 to 1942.50) | 1359.31 (942.37 to 1889.74) | 1331.78 (924.33 to 1849.11) | 1309.83 (902.89 to 1806.19) | 1269.47 (884.29 to 1718.50) | 1221.07 (852.35 to 1659.14) | 1114.13 (775.02 to 1510.32) |
| Papua New Guinea | Both | Prevalence | 5664.58 (2090.22 to 10800.85) | 17544.32 (8251.91 to 30652.46) | 24607.85 (11140.75 to 43666.77) | 32550.02 (14344.39 to 58426.92) | 39676.69 (17373.49 to 71390.67) | 45167.72 (19914.46 to 81108.61) | 49329.95 (21969.76 to 88297.38) | 52806.92 (23772.46 to 94266.75) | 55864.09 (25432.81 to 98382.98) | 58384.37 (26907.24 to 100000.00) | 60864.55 (28377.32 to 100000.00) | 63405.60 (29911.21 to 100000.00) | 66199.02 (31748.75 to 100000.00) | 67549.55 (32649.58 to 100000.00) | 67552.62 (32662.62 to 100000.00) | 67544.27 (32659.00 to 100000.00) | 67572.17 (32699.26 to 100000.00) | 67464.78 (32550.74 to 100000.00) | 67316.86 (32388.17 to 100000.00) | 66710.11 (31619.26 to 100000.00) |
| Papua New Guinea | Female | DALY rates | 23.12 (14.57 to 35.01) | 58.15 (37.70 to 86.52) | 96.26 (62.36 to 144.93) | 171.42 (109.58 to 249.26) | 275.80 (173.91 to 402.53) | 385.25 (250.50 to 563.94) | 483.37 (308.35 to 715.17) | 575.03 (373.00 to 841.03) | 665.63 (429.19 to 977.51) | 746.14 (486.12 to 1088.93) | 829.85 (531.24 to 1223.38) | 919.03 (583.64 to 1359.83) | 1021.46 (659.17 to 1480.97) | 1059.77 (689.87 to 1543.80) | 1040.93 (679.53 to 1521.86) | 1020.34 (672.38 to 1448.48) | 999.92 (663.53 to 1425.01) | 976.85 (650.42 to 1380.86) | 946.85 (635.90 to 1358.21) | 919.19 (618.25 to 1302.97) |
| Papua New Guinea | Female | Prevalence | 5689.97 (2121.68 to 10817.92) | 14534.13 (5412.18 to 27639.48) | 21290.03 (7997.21 to 40356.35) | 29370.81 (11270.38 to 55258.35) | 36674.15 (14449.39 to 68329.18) | 42297.05 (17092.26 to 78097.19) | 46530.77 (19186.19 to 85308.35) | 50075.07 (20957.45 to 91308.39) | 53202.82 (22551.63 to 96637.10) | 55777.98 (23907.18 to 100000.00) | 58300.88 (25364.88 to 100000.00) | 60905.67 (26917.98 to 100000.00) | 63819.17 (28720.38 to 100000.00) | 65197.72 (29588.79 to 100000.00) | 65199.01 (29621.59 to 100000.00) | 65199.74 (29629.25 to 100000.00) | 65199.44 (29600.82 to 100000.00) | 65198.27 (29601.22 to 100000.00) | 65199.00 (29602.98 to 100000.00) | 65198.26 (29610.34 to 100000.00) |
| Papua New Guinea | Male | DALY rates | 16.47 (10.12 to 25.82) | 761.54 (508.87 to 1058.40) | 873.17 (588.44 to 1226.91) | 933.16 (629.00 to 1280.25) | 1012.32 (689.95 to 1414.32) | 1098.36 (752.98 to 1538.20) | 1184.30 (816.15 to 1629.91) | 1262.08 (863.69 to 1797.53) | 1339.53 (926.77 to 1880.00) | 1409.43 (974.62 to 1984.13) | 1483.12 (1015.11 to 2122.60) | 1562.65 (1086.20 to 2269.66) | 1658.02 (1143.25 to 2325.34) | 1688.78 (1192.39 to 2386.66) | 1653.32 (1153.94 to 2332.26) | 1621.69 (1139.19 to 2258.15) | 1591.22 (1122.12 to 2197.49) | 1561.44 (1108.30 to 2128.12) | 1532.81 (1084.74 to 2090.74) | 1502.98 (1075.92 to 2034.03) |
| Papua New Guinea | Male | Prevalence | 5641.24 (2061.31 to 10785.17) | 20262.21 (10981.91 to 33386.93) | 27589.83 (13998.13 to 46682.53) | 35494.67 (17191.60 to 61361.71) | 42549.27 (20171.02 to 74319.67) | 47934.68 (22605.31 to 84011.23) | 51989.21 (24524.96 to 91156.53) | 55360.07 (26236.24 to 97133.77) | 58328.93 (27823.49 to 100000.00) | 60745.08 (29281.79 to 100000.00) | 63131.00 (30789.78 to 100000.00) | 65602.34 (32374.17 to 100000.00) | 68385.92 (34273.76 to 100000.00) | 69725.39 (35182.82 to 100000.00) | 69726.15 (35167.18 to 100000.00) | 69726.84 (35180.24 to 100000.00) | 69726.56 (35212.35 to 100000.00) | 69726.21 (35187.76 to 100000.00) | 69724.46 (35205.83 to 100000.00) | 69725.89 (35171.89 to 100000.00) |
| Philippines | Both | DALY rates | 11.28 (7.71 to 16.21) | 80.61 (46.27 to 132.18) | 126.11 (77.88 to 195.23) | 182.01 (117.94 to 267.67) | 225.79 (149.10 to 324.60) | 257.93 (170.86 to 365.55) | 281.37 (187.60 to 395.29) | 299.52 (201.62 to 418.92) | 311.60 (207.72 to 435.86) | 318.48 (213.55 to 443.85) | 321.39 (215.87 to 445.59) | 323.44 (215.79 to 446.52) | 325.15 (218.04 to 445.46) | 316.14 (213.40 to 433.87) | 306.67 (207.89 to 419.27) | 297.35 (203.66 to 403.20) | 291.24 (200.36 to 392.02) | 311.67 (216.44 to 418.99) | 392.52 (273.29 to 519.75) | 448.68 (317.89 to 593.79) |
| Philippines | Both | Prevalence | 1525.87 (253.62 to 4562.49) | 4280.24 (1031.00 to 11920.87) | 6303.68 (1591.17 to 17609.79) | 8620.15 (2234.59 to 24386.31) | 10478.18 (2730.23 to 30544.51) | 11839.03 (3098.96 to 34827.59) | 12819.70 (3371.54 to 37490.72) | 13628.23 (3604.86 to 39406.13) | 14284.30 (3795.16 to 40688.90) | 14840.22 (3916.11 to 41390.31) | 15254.03 (3997.01 to 42375.22) | 15736.30 (4106.74 to 43528.55) | 16287.09 (4216.39 to 44860.96) | 16442.02 (4219.24 to 45114.65) | 16419.93 (4210.06 to 45124.19) | 16347.35 (4190.62 to 45488.31) | 16213.24 (4154.84 to 45476.73) | 16362.67 (4383.85 to 44562.94) | 17226.85 (5154.75 to 44040.02) | 18402.31 (5857.58 to 45657.36) |
| Philippines | Female | DALY rates | 13.32 (9.13 to 19.01) | 25.31 (17.28 to 36.23) | 29.13 (20.10 to 42.62) | 34.05 (23.24 to 49.21) | 38.85 (26.36 to 56.11) | 42.73 (29.05 to 61.53) | 45.94 (30.89 to 66.22) | 48.59 (33.07 to 69.36) | 50.92 (34.77 to 72.44) | 53.28 (36.20 to 76.88) | 54.99 (38.00 to 77.96) | 57.11 (38.59 to 81.68) | 59.31 (40.04 to 84.46) | 59.38 (40.83 to 83.84) | 58.15 (40.26 to 81.61) | 56.45 (39.27 to 78.38) | 54.35 (37.71 to 75.35) | 52.51 (36.99 to 72.84) | 49.17 (34.56 to 67.67) | 46.62 (33.24 to 63.34) |
| Philippines | Female | Prevalence | 1548.77 (274.09 to 4579.62) | 3839.72 (612.85 to 11576.47) | 5521.29 (817.23 to 16931.59) | 7406.94 (1043.28 to 23314.30) | 8933.79 (1192.17 to 29152.15) | 10071.81 (1322.93 to 33178.10) | 10899.63 (1424.92 to 35759.55) | 11583.60 (1512.34 to 37710.88) | 12164.07 (1606.42 to 38918.83) | 12678.53 (1710.03 to 39805.37) | 13069.86 (1721.48 to 40950.61) | 13538.06 (1777.42 to 41979.42) | 14064.32 (1848.05 to 43408.79) | 14261.59 (1858.78 to 44230.02) | 14252.41 (1843.70 to 44361.80) | 14170.01 (1819.48 to 44322.65) | 13990.93 (1794.90 to 44248.13) | 13814.23 (1804.79 to 42754.56) | 13321.26 (1899.49 to 39277.63) | 12904.79 (1825.44 to 39343.53) |
| Philippines | Male | DALY rates | 9.37 (6.34 to 13.46) | 133.35 (72.05 to 229.01) | 220.03 (131.95 to 350.15) | 329.04 (208.70 to 491.10) | 414.87 (268.57 to 601.03) | 474.66 (311.16 to 677.23) | 514.74 (342.71 to 729.49) | 544.70 (365.42 to 766.10) | 566.68 (376.66 to 794.24) | 581.78 (389.02 to 812.68) | 591.82 (397.30 to 823.85) | 600.05 (400.30 to 828.18) | 609.69 (407.82 to 838.78) | 603.14 (406.82 to 827.12) | 590.73 (400.72 to 807.54) | 581.05 (397.30 to 790.16) | 566.72 (389.27 to 765.43) | 551.53 (383.61 to 740.86) | 541.11 (376.76 to 717.41) | 545.90 (386.98 to 721.97) |
| Philippines | Male | Prevalence | 1504.43 (233.57 to 4546.76) | 4700.39 (1386.21 to 12348.48) | 7061.39 (2290.59 to 18272.25) | 9825.73 (3368.36 to 25483.39) | 12040.34 (4210.70 to 31952.90) | 13618.76 (4825.66 to 36488.74) | 14723.04 (5260.33 to 39206.81) | 15626.01 (5616.65 to 41063.26) | 16359.00 (5933.58 to 42511.70) | 16986.46 (6201.03 to 43141.03) | 17471.16 (6352.16 to 43958.28) | 18019.42 (6505.77 to 44994.29) | 18666.12 (6689.02 to 46531.97) | 18879.31 (6726.06 to 46571.10) | 18897.37 (6709.55 to 46211.91) | 18911.53 (6720.13 to 46541.86) | 18797.55 (6678.91 to 47002.76) | 18721.26 (6629.64 to 46851.69) | 18916.97 (6646.46 to 46403.81) | 19731.65 (6909.49 to 46610.72) |
| Samoa | Both | DALY rates | 13.34 (8.36 to 19.78) | 156.87 (92.79 to 249.37) | 273.89 (177.98 to 396.56) | 400.41 (262.69 to 563.67) | 472.26 (314.92 to 661.77) | 492.50 (334.08 to 678.17) | 484.64 (327.26 to 678.43) | 482.13 (324.46 to 671.91) | 483.81 (333.72 to 684.43) | 482.71 (331.19 to 662.55) | 490.06 (337.25 to 674.93) | 495.93 (340.78 to 677.62) | 498.90 (344.08 to 684.82) | 500.34 (348.75 to 685.25) | 484.75 (340.21 to 658.59) | 431.64 (298.24 to 574.32) | 383.28 (272.81 to 524.04) | 330.76 (232.59 to 446.95) | 280.23 (199.32 to 373.92) | 247.59 (177.03 to 333.59) |
| Samoa | Both | Prevalence | 2666.07 (561.47 to 7537.32) | 7662.82 (2425.67 to 19951.20) | 11511.42 (3788.19 to 29396.35) | 15859.45 (5444.36 to 40165.09) | 19280.04 (6539.67 to 48973.23) | 21559.48 (7127.30 to 55211.66) | 22985.41 (7360.21 to 59414.45) | 24180.39 (7550.80 to 62907.61) | 25210.86 (7769.49 to 65820.50) | 26115.40 (7950.76 to 68431.76) | 27035.91 (8197.50 to 70933.78) | 27950.12 (8462.64 to 73457.81) | 28989.25 (8731.79 to 76385.26) | 29512.37 (8892.21 to 77795.94) | 29466.26 (8844.72 to 77728.75) | 29067.33 (8433.60 to 77321.35) | 28700.17 (8060.44 to 76933.61) | 28279.77 (7615.30 to 76486.84) | 27866.33 (7198.35 to 76055.35) | 27606.76 (6954.92 to 75783.89) |
| Samoa | Female | DALY rates | 15.75 (9.13 to 23.46) | 31.54 (19.84 to 47.21) | 39.57 (25.21 to 58.89) | 51.21 (33.23 to 76.91) | 64.54 (41.85 to 93.09) | 75.79 (49.17 to 111.58) | 84.41 (57.26 to 126.51) | 92.68 (59.31 to 139.99) | 99.42 (63.96 to 144.03) | 105.72 (68.77 to 153.49) | 111.59 (72.43 to 162.11) | 118.53 (77.44 to 170.74) | 126.75 (84.14 to 183.56) | 129.04 (87.24 to 184.06) | 127.51 (86.48 to 183.46) | 124.79 (84.92 to 176.29) | 122.28 (80.84 to 172.69) | 120.00 (81.36 to 170.73) | 117.61 (78.67 to 170.20) | 113.90 (77.88 to 163.10) |
| Samoa | Female | Prevalence | 2692.78 (590.12 to 7573.25) | 6658.26 (1395.67 to 18859.44) | 9617.74 (1971.72 to 27340.86) | 13036.78 (2663.46 to 37089.97) | 15969.35 (3284.93 to 45395.71) | 18163.46 (3768.14 to 51571.09) | 19721.08 (4122.31 to 55929.96) | 21000.84 (4419.05 to 59506.82) | 22058.30 (4675.16 to 62469.36) | 22999.80 (4910.05 to 65123.61) | 23875.07 (5125.97 to 67583.83) | 24779.64 (5357.16 to 70116.56) | 25828.36 (5632.05 to 73060.62) | 26316.80 (5758.45 to 74420.28) | 26316.75 (5756.93 to 74421.28) | 26316.80 (5760.70 to 74418.46) | 26316.80 (5757.55 to 74425.10) | 26316.78 (5759.70 to 74422.52) | 26316.78 (5760.62 to 74426.13) | 26316.87 (5758.99 to 74419.56) |
| Samoa | Male | DALY rates | 11.06 (6.48 to 17.29) | 268.50 (153.75 to 433.89) | 477.00 (308.16 to 701.13) | 698.99 (459.78 to 983.54) | 810.26 (537.91 to 1138.88) | 846.71 (573.01 to 1172.41) | 858.05 (576.26 to 1204.30) | 862.66 (580.96 to 1208.99) | 863.11 (589.66 to 1228.18) | 861.77 (587.39 to 1174.69) | 857.50 (590.48 to 1183.49) | 857.13 (585.98 to 1174.67) | 852.91 (587.24 to 1185.64) | 843.35 (583.28 to 1140.12) | 824.98 (579.76 to 1117.13) | 810.76 (555.41 to 1107.71) | 795.52 (557.99 to 1088.03) | 780.23 (550.90 to 1061.78) | 762.93 (544.96 to 1030.76) | 751.18 (534.58 to 1011.32) |
| Samoa | Male | Prevalence | 2640.81 (537.60 to 7503.37) | 8557.64 (3310.03 to 20951.67) | 13152.86 (5482.53 to 31207.99) | 18272.93 (7861.11 to 42797.79) | 22024.52 (9240.84 to 51938.91) | 24446.04 (9910.54 to 58306.09) | 26031.01 (10336.94 to 62665.47) | 27287.12 (10698.75 to 66230.53) | 28321.68 (10983.13 to 69127.27) | 29248.16 (11211.23 to 71758.14) | 30104.70 (11406.40 to 74186.19) | 30984.52 (11633.73 to 76655.65) | 31996.04 (11895.00 to 79547.81) | 32464.48 (11991.10 to 80914.41) | 32465.84 (11997.08 to 80878.80) | 32465.69 (11993.37 to 80907.96) | 32464.72 (12007.21 to 80895.82) | 32465.98 (12002.84 to 80889.14) | 32465.89 (11984.37 to 80891.37) | 32465.60 (12002.06 to 80923.09) |
| Sao Tome and Principe | Both | DALY rates | 10.61 (6.73 to 15.49) | 54.37 (28.92 to 95.27) | 75.84 (42.71 to 128.59) | 109.86 (63.29 to 180.48) | 144.42 (84.94 to 230.10) | 169.52 (105.84 to 259.56) | 185.03 (117.45 to 278.82) | 197.30 (127.73 to 293.00) | 213.35 (138.39 to 308.34) | 228.23 (147.35 to 336.57) | 245.90 (158.80 to 358.68) | 254.92 (170.71 to 369.28) | 266.18 (174.36 to 387.27) | 265.93 (176.71 to 380.08) | 256.24 (174.45 to 363.19) | 238.80 (161.98 to 337.84) | 195.58 (134.29 to 275.50) | 144.41 (99.14 to 199.53) | 63.60 (43.54 to 86.59) | 57.52 (38.14 to 79.23) |
| Sao Tome and Principe | Both | Prevalence | 1116.54 (116.79 to 6354.95) | 3038.71 (437.57 to 16262.28) | 4371.59 (620.38 to 23557.01) | 5993.88 (918.67 to 32124.15) | 7428.69 (1220.11 to 39505.74) | 8480.94 (1450.48 to 44913.33) | 9208.49 (1596.85 to 48723.07) | 9802.79 (1721.74 to 51840.05) | 10346.43 (1860.26 to 54529.41) | 10832.30 (2008.10 to 56902.10) | 11302.01 (2163.68 to 59098.71) | 11720.28 (2264.36 to 61277.41) | 12210.28 (2403.53 to 63855.06) | 12412.94 (2437.45 to 65029.92) | 12370.43 (2395.76 to 64988.35) | 12260.36 (2301.64 to 64873.95) | 11904.72 (1979.69 to 64498.21) | 11467.22 (1543.23 to 64050.00) | 10725.77 (808.60 to 63273.20) | 10683.22 (766.50 to 63230.97) |
| Sao Tome and Principe | Female | DALY rates | 12.55 (7.71 to 19.16) | 22.83 (13.63 to 34.90) | 25.29 (15.46 to 37.93) | 27.83 (16.71 to 41.42) | 30.45 (19.63 to 44.77) | 32.30 (20.29 to 49.32) | 34.13 (21.59 to 51.69) | 34.96 (21.23 to 50.47) | 35.91 (22.69 to 53.20) | 36.79 (23.44 to 56.51) | 37.45 (23.88 to 55.32) | 38.22 (24.31 to 55.25) | 38.79 (24.68 to 57.33) | 39.13 (24.87 to 56.61) | 38.77 (25.55 to 55.44) | 37.94 (23.90 to 54.33) | 37.13 (24.38 to 53.11) | 36.44 (23.65 to 51.67) | 35.87 (23.56 to 50.88) | 34.79 (21.82 to 50.02) |
| Sao Tome and Principe | Female | Prevalence | 1135.85 (135.02 to 6383.22) | 2790.95 (255.76 to 16076.03) | 3966.56 (293.22 to 23218.23) | 5336.41 (339.95 to 31527.72) | 6509.82 (380.78 to 38631.98) | 7369.57 (411.03 to 43828.10) | 7977.95 (434.22 to 47501.21) | 8475.90 (454.83 to 50503.13) | 8893.98 (471.22 to 53039.00) | 9248.27 (484.40 to 55258.20) | 9573.44 (499.95 to 57312.79) | 9902.20 (512.63 to 59390.78) | 10283.35 (529.81 to 61847.92) | 10462.05 (538.19 to 62999.08) | 10462.09 (539.35 to 62999.87) | 10462.06 (539.34 to 62998.43) | 10462.04 (540.32 to 62998.02) | 10462.06 (539.82 to 62998.82) | 10462.07 (539.21 to 62998.93) | 10462.03 (537.24 to 63000.26) |
| Sao Tome and Principe | Male | DALY rates | 8.73 (4.80 to 13.80) | 85.26 (41.24 to 158.17) | 125.59 (65.67 to 230.41) | 190.27 (105.21 to 324.73) | 258.66 (149.39 to 414.67) | 314.46 (192.67 to 487.94) | 354.94 (220.42 to 543.50) | 389.03 (247.88 to 585.52) | 417.40 (267.88 to 608.27) | 438.16 (281.57 to 653.98) | 459.91 (293.01 to 676.82) | 476.90 (316.83 to 699.92) | 498.11 (326.29 to 729.43) | 502.80 (330.20 to 727.59) | 493.34 (332.69 to 704.15) | 483.47 (323.49 to 689.21) | 475.15 (321.44 to 668.41) | 464.91 (307.86 to 653.73) | 455.23 (309.91 to 644.01) | 444.62 (299.43 to 626.39) |
| Sao Tome and Principe | Male | Prevalence | 1097.89 (97.86 to 6327.66) | 3281.38 (600.98 to 16444.70) | 4770.21 (909.46 to 23890.42) | 6638.49 (1428.91 to 32708.91) | 8349.71 (1950.25 to 40381.54) | 9654.83 (2395.10 to 46059.61) | 10594.01 (2755.07 to 50098.83) | 11369.94 (3043.37 to 53419.03) | 12016.75 (3305.78 to 56243.38) | 12569.41 (3516.99 to 58704.86) | 13076.73 (3716.67 to 60932.31) | 13582.76 (3893.52 to 63210.10) | 14175.63 (4133.93 to 65902.22) | 14450.51 (4233.19 to 67150.99) | 14450.93 (4240.24 to 67156.21) | 14450.93 (4231.83 to 67158.59) | 14450.27 (4236.96 to 67145.23) | 14450.92 (4226.83 to 67170.33) | 14450.22 (4229.48 to 67146.98) | 14450.56 (4234.00 to 67160.39) |
| Senegal | Both | DALY rates | 10.27 (6.36 to 16.10) | 50.21 (27.49 to 87.81) | 68.21 (37.90 to 115.46) | 95.87 (53.86 to 158.29) | 123.02 (72.67 to 193.42) | 146.46 (88.98 to 225.22) | 165.63 (104.57 to 250.84) | 180.81 (114.44 to 274.33) | 195.16 (124.57 to 289.89) | 206.58 (132.69 to 300.25) | 220.94 (145.98 to 326.88) | 236.30 (151.76 to 343.92) | 252.75 (168.80 to 361.79) | 261.96 (172.09 to 376.84) | 249.84 (166.73 to 353.91) | 242.97 (162.59 to 347.11) | 232.26 (157.75 to 330.85) | 216.92 (147.54 to 307.02) | 194.15 (130.32 to 274.08) | 136.07 (93.45 to 190.18) |
| Senegal | Both | Prevalence | 1020.57 (184.77 to 3793.35) | 2830.52 (668.76 to 9925.41) | 4067.42 (927.89 to 14406.75) | 5555.95 (1280.26 to 19664.00) | 6858.17 (1605.26 to 24178.75) | 7834.00 (1853.51 to 27513.33) | 8549.53 (2050.27 to 29913.31) | 9135.58 (2223.33 to 31849.33) | 9645.26 (2381.35 to 33508.75) | 10087.99 (2526.58 to 34947.25) | 10521.88 (2677.51 to 36318.01) | 10998.90 (2859.14 to 37784.02) | 11539.42 (3052.49 to 39450.92) | 11818.72 (3168.55 to 40256.42) | 11758.20 (3112.47 to 40197.62) | 11749.31 (3108.63 to 40178.23) | 11687.00 (3046.62 to 40132.04) | 11592.19 (2962.81 to 40033.70) | 11405.14 (2780.27 to 39861.78) | 10879.20 (2241.61 to 39352.90) |
| Senegal | Female | DALY rates | 12.10 (7.05 to 18.89) | 22.62 (13.86 to 34.92) | 24.48 (14.55 to 36.48) | 27.20 (16.42 to 41.52) | 28.79 (17.24 to 42.77) | 30.92 (19.74 to 48.23) | 32.30 (20.34 to 46.82) | 33.09 (20.92 to 49.98) | 33.73 (21.22 to 48.69) | 34.18 (21.33 to 50.57) | 35.63 (22.85 to 53.02) | 36.33 (22.85 to 54.24) | 37.02 (23.48 to 53.43) | 36.94 (23.70 to 54.76) | 36.19 (23.25 to 53.26) | 35.68 (22.75 to 51.71) | 35.25 (23.17 to 52.73) | 34.08 (21.80 to 48.91) | 33.43 (21.96 to 47.49) | 32.66 (21.45 to 46.72) |
| Senegal | Female | Prevalence | 1043.25 (198.81 to 3831.67) | 2608.32 (431.00 to 9804.74) | 3714.25 (549.55 to 14180.40) | 4999.28 (688.46 to 19256.57) | 6092.67 (810.44 to 23569.63) | 6892.68 (898.46 to 26721.95) | 7464.93 (962.78 to 28973.86) | 7922.54 (1014.88 to 30773.52) | 8312.01 (1060.66 to 32304.29) | 8651.92 (1098.94 to 33638.71) | 8968.47 (1135.85 to 34880.97) | 9301.20 (1175.10 to 36186.38) | 9683.76 (1219.93 to 37688.15) | 9861.02 (1243.38 to 38381.29) | 9861.05 (1242.07 to 38382.45) | 9861.03 (1242.30 to 38383.33) | 9861.00 (1242.46 to 38382.44) | 9861.01 (1240.93 to 38382.11) | 9861.03 (1241.16 to 38383.26) | 9861.01 (1241.99 to 38382.48) |
| Senegal | Male | DALY rates | 8.49 (4.49 to 14.50) | 78.37 (37.75 to 152.23) | 114.07 (57.44 to 206.46) | 170.18 (92.51 to 291.20) | 229.16 (131.05 to 377.51) | 279.00 (165.78 to 442.35) | 317.91 (195.49 to 486.82) | 346.66 (215.94 to 527.04) | 371.91 (235.54 to 561.33) | 392.78 (246.87 to 573.55) | 413.55 (269.08 to 612.75) | 431.87 (273.04 to 633.07) | 452.79 (296.55 to 654.63) | 459.48 (296.82 to 665.73) | 450.25 (293.72 to 644.83) | 439.29 (289.63 to 633.20) | 431.91 (289.37 to 618.80) | 422.41 (281.50 to 599.15) | 416.11 (274.43 to 592.03) | 406.05 (272.24 to 577.13) |
| Senegal | Male | Prevalence | 998.29 (170.66 to 3755.70) | 3057.23 (848.72 to 10048.53) | 4437.86 (1242.59 to 14644.16) | 6158.33 (1830.47 to 20104.89) | 7720.35 (2420.05 to 24864.78) | 8913.79 (2913.32 to 28421.12) | 9788.22 (3264.94 to 30986.23) | 10497.50 (3578.16 to 33057.16) | 11105.11 (3840.22 to 34827.58) | 11638.94 (4068.02 to 36360.46) | 12136.53 (4272.22 to 37811.70) | 12659.23 (4490.84 to 39346.48) | 13260.13 (4717.98 to 41085.49) | 13537.16 (4830.85 to 41902.37) | 13537.73 (4823.54 to 41900.25) | 13537.62 (4833.40 to 41878.12) | 13537.58 (4823.79 to 41905.20) | 13537.77 (4831.64 to 41889.82) | 13537.52 (4834.11 to 41903.58) | 13537.47 (4834.23 to 41886.43) |
| Sierra Leone | Both | DALY rates | 16.41 (10.50 to 23.88) | 338.86 (223.93 to 478.03) | 455.71 (304.13 to 633.02) | 489.47 (338.64 to 683.11) | 512.80 (351.41 to 709.77) | 552.41 (379.14 to 774.60) | 607.95 (419.40 to 851.87) | 667.17 (456.45 to 930.85) | 718.30 (491.42 to 1004.56) | 764.90 (523.74 to 1085.57) | 796.78 (547.59 to 1106.28) | 825.70 (565.54 to 1171.02) | 869.18 (592.47 to 1228.83) | 883.10 (610.03 to 1221.84) | 871.67 (597.33 to 1222.27) | 854.51 (593.29 to 1202.95) | 832.06 (579.93 to 1138.76) | 780.68 (548.93 to 1066.21) | 745.26 (530.27 to 1012.51) | 700.96 (495.42 to 938.75) |
| Sierra Leone | Both | Prevalence | 4429.13 (587.73 to 11212.69) | 14258.46 (4029.61 to 32380.15) | 20466.16 (5545.58 to 46944.75) | 26902.93 (6558.79 to 63263.25) | 32308.93 (7342.54 to 76842.67) | 36429.82 (8175.85 to 86923.90) | 39579.19 (9041.20 to 94383.49) | 42172.02 (9886.26 to 98749.21) | 44327.62 (10601.00 to 100000.00) | 46152.85 (11194.46 to 100000.00) | 47760.06 (11720.75 to 100000.00) | 49407.44 (12218.26 to 100000.00) | 51293.82 (12941.53 to 100000.00) | 52174.82 (13330.35 to 100000.00) | 52201.20 (13361.97 to 100000.00) | 52193.45 (13341.28 to 100000.00) | 52130.55 (13266.51 to 100000.00) | 51846.38 (12963.11 to 100000.00) | 51690.40 (12798.68 to 100000.00) | 51463.30 (12547.62 to 100000.00) |
| Sierra Leone | Female | DALY rates | 19.28 (11.99 to 28.33) | 46.87 (28.38 to 70.72) | 71.42 (47.21 to 108.31) | 114.78 (75.27 to 170.59) | 171.46 (109.64 to 257.09) | 226.96 (149.38 to 331.71) | 277.13 (177.85 to 410.72) | 322.42 (209.01 to 464.39) | 362.57 (234.52 to 526.77) | 402.13 (260.01 to 593.71) | 438.73 (280.40 to 637.42) | 481.37 (311.94 to 698.13) | 530.20 (343.39 to 773.29) | 546.54 (355.71 to 792.24) | 537.60 (354.40 to 780.29) | 530.75 (342.75 to 787.43) | 521.35 (338.72 to 747.11) | 511.56 (339.27 to 736.48) | 500.03 (334.68 to 713.13) | 485.66 (328.58 to 686.53) |
| Sierra Leone | Female | Prevalence | 4468.35 (614.35 to 11272.07) | 11881.55 (1598.12 to 30041.43) | 17323.06 (2360.79 to 43763.18) | 23850.62 (3414.43 to 59991.11) | 29536.86 (4493.26 to 73858.30) | 33810.93 (5445.37 to 84089.25) | 36934.00 (6226.91 to 91532.63) | 39465.37 (7006.40 to 97580.55) | 41573.03 (7695.45 to 100000.00) | 43367.21 (8333.31 to 100000.00) | 45027.56 (8882.65 to 100000.00) | 46787.45 (9451.85 to 100000.00) | 48750.84 (10113.00 to 100000.00) | 49634.29 (10416.46 to 100000.00) | 49633.49 (10420.48 to 100000.00) | 49633.39 (10404.41 to 100000.00) | 49633.95 (10416.23 to 100000.00) | 49634.28 (10424.51 to 100000.00) | 49633.80 (10418.41 to 100000.00) | 49633.59 (10406.17 to 100000.00) |
| Sierra Leone | Male | DALY rates | 13.61 (8.15 to 21.81) | 627.08 (410.26 to 897.76) | 832.42 (553.57 to 1164.48) | 883.10 (608.59 to 1235.71) | 925.06 (623.77 to 1279.65) | 966.82 (649.46 to 1338.41) | 1002.48 (679.04 to 1381.60) | 1036.15 (712.37 to 1431.99) | 1065.02 (727.24 to 1472.73) | 1093.18 (751.84 to 1522.38) | 1119.39 (773.19 to 1530.53) | 1147.25 (797.27 to 1612.77) | 1184.76 (813.80 to 1640.15) | 1187.94 (836.20 to 1620.73) | 1167.74 (810.13 to 1616.98) | 1143.29 (802.40 to 1569.47) | 1123.91 (792.14 to 1519.90) | 1100.62 (778.82 to 1482.08) | 1077.50 (765.95 to 1453.31) | 1055.44 (754.79 to 1420.03) |
| Sierra Leone | Male | Prevalence | 4390.89 (561.77 to 11154.80) | 16604.60 (6324.15 to 34729.09) | 23547.32 (8604.59 to 50063.62) | 30109.65 (9737.29 to 66700.92) | 35657.03 (10783.89 to 80447.19) | 39764.57 (11541.25 to 90537.92) | 42733.82 (12167.02 to 97790.44) | 45068.89 (12709.28 to 100000.00) | 47012.49 (13260.06 to 100000.00) | 48673.71 (13732.18 to 100000.00) | 50222.12 (14219.60 to 100000.00) | 51854.07 (14707.44 to 100000.00) | 53661.23 (15316.97 to 100000.00) | 54475.95 (15663.53 to 100000.00) | 54476.81 (15642.62 to 100000.00) | 54476.91 (15666.35 to 100000.00) | 54475.56 (15651.43 to 100000.00) | 54476.15 (15655.23 to 100000.00) | 54476.78 (15658.43 to 100000.00) | 54475.91 (15640.57 to 100000.00) |
| South Sudan | Both | DALY rates | 9.54 (6.15 to 14.53) | 40.38 (21.65 to 74.90) | 49.79 (26.79 to 89.92) | 62.68 (34.30 to 108.15) | 76.09 (41.17 to 128.40) | 87.14 (48.00 to 145.32) | 95.16 (55.01 to 159.37) | 103.57 (58.66 to 170.30) | 107.75 (62.94 to 177.46) | 119.39 (69.23 to 191.62) | 130.12 (77.23 to 211.38) | 140.11 (82.14 to 225.98) | 152.48 (91.26 to 243.63) | 157.59 (94.49 to 245.56) | 148.53 (90.49 to 231.35) | 134.64 (84.49 to 211.25) | 120.75 (76.10 to 185.09) | 107.34 (66.03 to 164.78) | 95.82 (60.47 to 144.63) | 80.28 (51.94 to 118.40) |
| South Sudan | Both | Prevalence | 677.64 (191.56 to 2001.71) | 1893.86 (584.00 to 5384.83) | 2672.55 (777.40 to 7734.43) | 3588.64 (1026.09 to 10455.83) | 4384.83 (1254.36 to 12799.25) | 4978.27 (1436.33 to 14520.09) | 5410.42 (1569.41 to 15762.37) | 5772.33 (1697.02 to 16772.81) | 6055.08 (1777.91 to 17611.21) | 6375.51 (1925.85 to 18401.52) | 6669.93 (2062.47 to 19145.37) | 6981.58 (2194.58 to 19915.30) | 7329.03 (2329.20 to 20804.90) | 7509.53 (2408.44 to 21233.89) | 7462.42 (2376.11 to 21189.19) | 7355.23 (2283.03 to 21088.86) | 7260.04 (2188.62 to 21002.68) | 7163.80 (2104.79 to 20907.88) | 7070.94 (2021.68 to 20826.20) | 6941.00 (1913.42 to 20703.25) |
| South Sudan | Female | DALY rates | 11.26 (6.76 to 17.67) | 20.97 (12.81 to 33.00) | 21.96 (13.05 to 33.34) | 23.37 (13.88 to 35.11) | 24.90 (14.69 to 39.05) | 25.42 (15.17 to 37.97) | 26.03 (16.45 to 38.95) | 26.28 (16.29 to 40.80) | 26.84 (17.27 to 38.89) | 27.06 (17.16 to 41.44) | 27.29 (17.55 to 41.88) | 27.56 (16.71 to 41.60) | 27.84 (17.27 to 41.20) | 27.78 (17.33 to 40.34) | 26.96 (16.97 to 41.01) | 26.57 (16.72 to 37.96) | 25.89 (16.50 to 38.38) | 25.32 (15.70 to 35.67) | 24.80 (15.83 to 35.33) | 23.95 (15.89 to 34.90) |
| South Sudan | Female | Prevalence | 693.15 (209.81 to 2014.01) | 1740.79 (465.70 to 5226.32) | 2453.01 (597.14 to 7530.03) | 3272.06 (748.55 to 10177.07) | 3969.33 (877.93 to 12429.56) | 4478.11 (973.18 to 14071.81) | 4844.14 (1041.49 to 15251.95) | 5137.13 (1096.68 to 16196.66) | 5388.48 (1143.34 to 17006.84) | 5602.17 (1183.79 to 17695.25) | 5806.25 (1222.72 to 18353.00) | 6016.55 (1261.41 to 19031.24) | 6262.08 (1308.59 to 19820.59) | 6373.51 (1329.92 to 20179.42) | 6373.52 (1328.90 to 20178.99) | 6373.56 (1330.67 to 20179.37) | 6373.52 (1328.82 to 20180.69) | 6373.51 (1328.64 to 20179.44) | 6373.51 (1330.26 to 20179.64) | 6373.52 (1329.95 to 20178.14) |
| South Sudan | Male | DALY rates | 7.95 (4.53 to 12.80) | 57.98 (26.90 to 114.24) | 74.90 (34.73 to 151.02) | 98.34 (47.29 to 178.92) | 123.59 (62.44 to 216.93) | 144.95 (74.36 to 255.78) | 160.47 (86.62 to 277.04) | 174.15 (93.27 to 294.85) | 186.81 (100.21 to 318.13) | 195.32 (108.79 to 324.94) | 205.82 (117.97 to 347.84) | 213.60 (120.57 to 347.51) | 227.70 (133.51 to 370.87) | 229.40 (134.46 to 362.71) | 223.93 (132.59 to 354.29) | 220.80 (132.42 to 355.68) | 214.66 (130.55 to 336.48) | 208.45 (124.98 to 330.52) | 204.50 (125.07 to 317.71) | 199.09 (121.55 to 303.39) |
| South Sudan | Male | Prevalence | 663.34 (174.74 to 1990.38) | 2032.54 (689.98 to 5595.27) | 2870.63 (932.29 to 8012.95) | 3875.88 (1249.94 to 10834.25) | 4770.35 (1573.63 to 13271.00) | 5446.64 (1816.29 to 15063.89) | 5945.44 (2006.06 to 16371.29) | 6352.30 (2165.23 to 17411.73) | 6706.52 (2317.31 to 18311.40) | 7011.41 (2446.00 to 19091.69) | 7305.66 (2575.22 to 19824.15) | 7611.65 (2707.82 to 20586.41) | 7972.89 (2868.06 to 21489.84) | 8138.06 (2938.77 to 21894.18) | 8137.77 (2943.85 to 21893.44) | 8137.91 (2946.64 to 21900.21) | 8137.76 (2931.98 to 21900.07) | 8137.95 (2936.53 to 21888.46) | 8137.99 (2938.36 to 21896.85) | 8137.74 (2938.73 to 21897.33) |
| Sri Lanka | Both | DALY rates | 9.74 (6.07 to 14.80) | 28.80 (15.08 to 51.44) | 29.99 (15.43 to 55.24) | 31.12 (16.56 to 55.05) | 32.18 (16.95 to 59.18) | 32.77 (18.47 to 58.27) | 33.78 (17.76 to 60.60) | 33.96 (18.23 to 60.32) | 34.33 (18.11 to 61.76) | 34.58 (19.00 to 62.36) | 34.82 (18.96 to 63.71) | 34.63 (18.54 to 63.68) | 35.16 (18.82 to 61.30) | 34.69 (17.96 to 61.02) | 34.02 (18.28 to 59.44) | 33.08 (17.64 to 58.93) | 32.57 (18.37 to 57.06) | 31.57 (17.96 to 54.41) | 31.86 (17.25 to 57.16) | 34.18 (17.81 to 61.95) |
| Sri Lanka | Both | Prevalence | 232.49 (194.30 to 279.82) | 594.80 (473.71 to 759.95) | 754.67 (593.21 to 975.81) | 943.38 (737.70 to 1216.60) | 1101.17 (855.30 to 1416.21) | 1218.25 (941.28 to 1570.08) | 1301.42 (1003.82 to 1672.60) | 1369.70 (1054.43 to 1758.12) | 1425.79 (1095.82 to 1828.55) | 1475.90 (1133.10 to 1895.49) | 1523.11 (1168.24 to 1959.50) | 1571.85 (1204.24 to 2024.32) | 1629.36 (1247.28 to 2097.12) | 1655.95 (1267.53 to 2131.15) | 1654.48 (1265.66 to 2127.99) | 1654.00 (1266.06 to 2127.46) | 1653.46 (1265.24 to 2127.61) | 1650.48 (1263.08 to 2120.21) | 1657.09 (1268.65 to 2128.27) | 1685.92 (1289.05 to 2178.93) |
| Sri Lanka | Female | DALY rates | 11.36 (6.55 to 17.88) | 19.23 (11.50 to 29.22) | 19.22 (11.18 to 29.82) | 19.63 (11.72 to 29.33) | 19.60 (11.43 to 30.89) | 19.52 (12.00 to 29.09) | 19.80 (11.25 to 29.97) | 19.73 (11.79 to 29.33) | 19.53 (11.77 to 29.85) | 19.67 (11.51 to 30.70) | 19.64 (11.40 to 29.22) | 19.31 (11.53 to 30.02) | 19.47 (10.94 to 30.30) | 18.94 (11.29 to 28.08) | 18.86 (10.69 to 28.53) | 18.08 (10.95 to 27.12) | 17.98 (11.02 to 27.70) | 17.58 (10.79 to 26.11) | 17.23 (10.61 to 26.23) | 16.95 (10.41 to 25.88) |
| Sri Lanka | Female | Prevalence | 249.36 (211.17 to 298.67) | 523.32 (430.75 to 640.39) | 675.59 (541.44 to 843.33) | 855.20 (672.27 to 1082.89) | 1005.41 (782.35 to 1283.53) | 1116.22 (863.25 to 1431.74) | 1194.23 (919.50 to 1535.35) | 1258.23 (966.38 to 1620.13) | 1311.06 (1004.06 to 1691.24) | 1357.69 (1037.48 to 1752.87) | 1402.03 (1070.80 to 1811.93) | 1447.62 (1102.29 to 1872.86) | 1499.83 (1139.60 to 1942.10) | 1524.35 (1157.41 to 1975.25) | 1524.36 (1157.57 to 1975.38) | 1524.35 (1156.93 to 1974.45) | 1524.35 (1157.46 to 1974.46) | 1524.33 (1157.15 to 1974.07) | 1524.34 (1157.45 to 1975.24) | 1524.35 (1157.31 to 1975.43) |
| Sri Lanka | Male | DALY rates | 8.16 (4.53 to 12.86) | 38.17 (16.52 to 79.06) | 40.49 (17.11 to 84.81) | 42.44 (18.39 to 89.22) | 44.82 (20.55 to 92.94) | 46.14 (21.54 to 92.56) | 47.80 (22.00 to 97.49) | 48.17 (20.73 to 98.18) | 49.17 (22.37 to 101.53) | 49.40 (22.85 to 100.38) | 49.94 (22.73 to 102.06) | 49.89 (22.36 to 102.14) | 50.39 (22.85 to 100.35) | 49.90 (21.81 to 96.41) | 49.00 (22.70 to 96.06) | 48.01 (22.15 to 95.46) | 47.21 (21.76 to 93.46) | 46.28 (20.93 to 90.62) | 45.76 (20.67 to 91.29) | 44.54 (20.16 to 86.83) |
| Sri Lanka | Male | Prevalence | 216.15 (176.40 to 262.62) | 664.67 (491.19 to 944.09) | 831.77 (620.81 to 1140.06) | 1030.23 (773.38 to 1366.28) | 1197.31 (903.59 to 1589.08) | 1321.23 (999.55 to 1757.79) | 1408.87 (1067.45 to 1879.30) | 1480.99 (1120.87 to 1970.20) | 1540.73 (1166.77 to 2045.08) | 1593.43 (1206.54 to 2113.65) | 1643.75 (1244.40 to 2178.63) | 1695.65 (1282.75 to 2241.91) | 1755.12 (1328.37 to 2318.34) | 1783.05 (1350.45 to 2353.09) | 1783.01 (1349.46 to 2355.34) | 1783.06 (1349.78 to 2352.93) | 1783.09 (1349.30 to 2353.63) | 1783.08 (1350.16 to 2353.10) | 1783.07 (1349.98 to 2362.29) | 1783.04 (1350.32 to 2352.25) |
| Sudan | Both | DALY rates | 9.65 (5.92 to 14.77) | 41.88 (22.90 to 78.32) | 52.84 (28.63 to 93.14) | 67.05 (36.43 to 118.49) | 80.42 (45.41 to 133.04) | 91.80 (52.72 to 154.54) | 101.47 (57.79 to 162.97) | 109.22 (65.80 to 174.08) | 122.62 (72.96 to 195.70) | 126.00 (76.06 to 196.52) | 135.45 (82.08 to 212.99) | 144.74 (86.15 to 223.56) | 157.19 (95.96 to 244.54) | 161.85 (99.83 to 249.90) | 152.48 (95.38 to 233.44) | 143.42 (88.57 to 215.17) | 137.69 (85.86 to 206.15) | 133.48 (83.86 to 199.90) | 126.79 (81.17 to 188.57) | 110.09 (72.02 to 159.50) |
| Sudan | Both | Prevalence | 729.96 (126.07 to 2922.37) | 2054.67 (430.62 to 7905.35) | 2905.54 (563.52 to 11387.07) | 3910.91 (729.83 to 15437.55) | 4770.39 (881.74 to 18875.50) | 5419.64 (1013.14 to 21413.98) | 5899.68 (1120.29 to 23246.98) | 6283.17 (1203.67 to 24712.13) | 6667.62 (1315.25 to 26015.66) | 6929.06 (1369.43 to 27063.74) | 7233.26 (1459.55 to 28114.72) | 7553.64 (1557.84 to 29209.20) | 7931.77 (1697.03 to 30475.52) | 8124.18 (1773.33 to 31089.57) | 8066.92 (1715.74 to 31032.54) | 8010.59 (1659.79 to 30977.76) | 7985.47 (1636.28 to 30951.49) | 7972.10 (1624.22 to 30937.49) | 7929.28 (1593.34 to 30892.51) | 7795.72 (1466.78 to 30764.96) |
| Sudan | Female | DALY rates | 11.35 (6.60 to 18.02) | 21.16 (12.41 to 32.22) | 22.52 (13.46 to 34.86) | 24.14 (14.26 to 36.76) | 25.28 (15.87 to 38.42) | 26.25 (16.38 to 38.75) | 26.94 (15.91 to 39.84) | 27.52 (17.17 to 41.80) | 28.03 (17.79 to 42.93) | 28.46 (17.43 to 43.41) | 29.05 (18.45 to 43.33) | 29.09 (18.21 to 42.77) | 29.52 (18.68 to 44.59) | 29.59 (17.74 to 44.71) | 29.36 (18.75 to 44.45) | 28.86 (18.18 to 42.60) | 28.18 (18.20 to 41.88) | 27.59 (17.46 to 40.10) | 27.00 (16.65 to 39.32) | 26.06 (16.97 to 37.06) |
| Sudan | Female | Prevalence | 745.96 (142.05 to 2937.59) | 1891.75 (291.91 to 7706.16) | 2667.51 (342.03 to 11113.48) | 3566.79 (401.44 to 15061.61) | 4330.26 (453.19 to 18411.02) | 4890.17 (491.75 to 20864.39) | 5292.38 (520.30 to 22625.51) | 5613.73 (541.61 to 24032.42) | 5886.99 (561.37 to 25228.00) | 6122.07 (577.68 to 26257.41) | 6344.69 (594.15 to 27230.54) | 6576.26 (610.32 to 28241.72) | 6841.06 (629.52 to 29399.49) | 6966.92 (638.70 to 29949.52) | 6966.92 (639.13 to 29949.53) | 6966.87 (638.47 to 29948.75) | 6966.95 (638.85 to 29948.96) | 6966.92 (639.43 to 29949.06) | 6966.91 (638.00 to 29948.55) | 6966.91 (638.76 to 29949.36) |
| Sudan | Male | DALY rates | 8.11 (4.41 to 12.91) | 61.35 (29.37 to 125.45) | 81.37 (39.06 to 156.20) | 109.33 (52.01 to 203.39) | 140.33 (73.21 to 243.65) | 164.84 (86.34 to 290.20) | 183.93 (99.86 to 311.44) | 200.22 (115.74 to 331.12) | 214.21 (123.52 to 347.25) | 227.32 (131.67 to 364.93) | 238.81 (141.60 to 385.28) | 249.81 (145.52 to 391.14) | 263.53 (157.74 to 417.56) | 265.49 (161.18 to 415.45) | 260.40 (157.73 to 408.47) | 255.42 (154.27 to 390.99) | 250.07 (155.58 to 386.73) | 245.04 (146.96 to 374.25) | 241.02 (150.68 to 365.60) | 235.35 (146.61 to 352.16) |
| Sudan | Male | Prevalence | 715.49 (114.65 to 2908.60) | 2207.72 (515.96 to 8099.44) | 3129.53 (694.68 to 11652.13) | 4249.89 (954.28 to 15814.26) | 5248.52 (1221.91 to 19385.26) | 6009.56 (1448.94 to 22029.84) | 6571.52 (1634.43 to 23936.45) | 7028.77 (1791.30 to 25470.00) | 7423.44 (1932.58 to 26779.00) | 7767.21 (2059.78 to 27901.23) | 8096.42 (2184.08 to 28973.61) | 8441.60 (2310.52 to 30088.18) | 8840.28 (2455.25 to 31371.81) | 9031.04 (2517.28 to 31982.95) | 9031.06 (2521.29 to 31981.79) | 9031.04 (2518.30 to 31983.83) | 9030.75 (2516.71 to 31980.37) | 9031.15 (2513.15 to 31978.90) | 9031.06 (2522.89 to 31973.20) | 9031.10 (2523.81 to 31980.65) |
| Thailand | Both | DALY rates | 10.71 (6.73 to 16.47) | 46.84 (25.25 to 82.75) | 61.64 (33.83 to 107.58) | 82.63 (45.30 to 139.53) | 106.64 (59.70 to 175.98) | 121.66 (71.21 to 197.92) | 135.67 (80.08 to 209.27) | 147.74 (92.08 to 230.36) | 158.19 (97.77 to 247.42) | 165.35 (105.64 to 251.32) | 172.14 (106.90 to 255.87) | 178.04 (114.54 to 267.45) | 188.04 (120.40 to 274.52) | 186.99 (122.31 to 272.73) | 177.65 (115.95 to 261.77) | 166.91 (109.15 to 241.26) | 163.99 (103.68 to 239.70) | 146.90 (97.28 to 212.33) | 111.85 (76.98 to 160.65) | 66.49 (44.84 to 94.69) |
| Thailand | Both | Prevalence | 942.30 (165.74 to 3900.51) | 2451.92 (538.30 to 9739.81) | 3505.24 (734.35 to 14108.83) | 4740.93 (983.89 to 19116.16) | 5847.85 (1255.15 to 23406.98) | 6631.66 (1430.09 to 26506.93) | 7227.48 (1587.00 to 28759.03) | 7711.49 (1713.50 to 30570.69) | 8132.85 (1829.73 to 32132.72) | 8486.71 (1920.60 to 33468.64) | 8820.36 (2012.00 to 34722.20) | 9162.24 (2096.22 to 36008.07) | 9572.68 (2217.41 to 37507.01) | 9742.26 (2245.69 to 38200.39) | 9685.83 (2196.61 to 38145.41) | 9622.04 (2142.98 to 38082.61) | 9614.50 (2133.76 to 38074.42) | 9485.58 (2019.05 to 37944.07) | 9188.52 (1738.58 to 37644.91) | 8761.70 (1304.56 to 37214.78) |
| Thailand | Female | DALY rates | 12.58 (7.34 to 18.99) | 22.08 (13.42 to 35.57) | 23.80 (14.56 to 36.66) | 25.77 (15.45 to 40.03) | 27.48 (16.38 to 42.21) | 29.06 (17.76 to 45.30) | 29.68 (18.20 to 45.11) | 30.58 (19.87 to 46.62) | 31.71 (19.06 to 46.68) | 31.67 (19.70 to 47.56) | 32.14 (19.61 to 46.89) | 32.62 (20.04 to 47.29) | 33.42 (20.55 to 50.53) | 33.43 (21.26 to 50.09) | 32.98 (20.91 to 48.35) | 31.70 (21.44 to 46.53) | 31.31 (20.46 to 46.27) | 30.92 (19.28 to 45.39) | 30.05 (19.12 to 44.02) | 29.48 (18.56 to 42.27) |
| Thailand | Female | Prevalence | 961.50 (183.05 to 3924.48) | 2258.74 (360.69 to 9485.83) | 3204.64 (444.89 to 13717.76) | 4288.85 (543.12 to 18562.78) | 5209.43 (628.47 to 22671.77) | 5882.91 (691.78 to 25673.67) | 6366.09 (736.91 to 27826.50) | 6753.91 (773.61 to 29551.90) | 7085.17 (805.69 to 31028.03) | 7372.80 (834.40 to 32306.36) | 7642.57 (860.92 to 33505.43) | 7921.97 (889.07 to 34746.91) | 8239.64 (918.79 to 36157.89) | 8394.95 (933.60 to 36846.50) | 8394.95 (934.33 to 36848.11) | 8394.94 (935.25 to 36847.66) | 8394.99 (934.19 to 36847.47) | 8394.90 (934.00 to 36848.03) | 8394.95 (933.36 to 36848.58) | 8394.92 (934.74 to 36846.17) |
| Thailand | Male | DALY rates | 8.92 (5.13 to 14.22) | 70.86 (33.11 to 139.18) | 97.80 (47.12 to 182.00) | 139.67 (70.55 to 246.87) | 182.14 (96.11 to 308.82) | 219.20 (122.61 to 368.77) | 246.88 (138.71 to 390.73) | 270.22 (158.92 to 429.14) | 288.87 (173.41 to 456.05) | 305.66 (191.70 to 471.86) | 321.01 (197.02 to 482.45) | 335.74 (213.35 to 510.52) | 353.97 (223.47 to 527.90) | 358.49 (229.00 to 528.08) | 352.57 (224.87 to 527.03) | 345.98 (224.90 to 510.67) | 341.61 (212.00 to 508.61) | 334.24 (212.49 to 489.98) | 324.01 (210.05 to 476.38) | 317.22 (208.01 to 456.18) |
| Thailand | Male | Prevalence | 923.83 (149.11 to 3877.46) | 2639.37 (679.68 to 9922.70) | 3792.54 (963.25 to 14389.88) | 5194.52 (1359.32 to 19591.54) | 6456.74 (1770.57 to 24073.59) | 7420.42 (2097.73 to 27390.37) | 8131.15 (2359.34 to 29746.92) | 8712.62 (2585.43 to 31645.65) | 9215.32 (2777.86 to 33283.75) | 9655.95 (2965.03 to 34695.79) | 10072.78 (3138.75 to 36023.85) | 10507.13 (3322.50 to 37383.47) | 11003.25 (3540.98 to 38962.03) | 11247.04 (3635.09 to 39719.65) | 11246.58 (3630.47 to 39720.30) | 11247.07 (3640.04 to 39725.25) | 11247.12 (3635.69 to 39724.13) | 11247.21 (3634.18 to 39722.11) | 11247.00 (3628.90 to 39717.91) | 11246.60 (3637.44 to 39718.94) |
| Timor-Leste | Both | DALY rates | 19.90 (13.35 to 29.81) | 446.30 (299.00 to 620.92) | 523.80 (357.43 to 736.31) | 597.30 (407.04 to 838.43) | 685.29 (468.64 to 963.47) | 775.66 (532.60 to 1090.22) | 864.58 (585.04 to 1214.67) | 941.02 (641.42 to 1347.43) | 992.43 (677.18 to 1411.92) | 1049.77 (708.81 to 1477.75) | 1107.22 (751.03 to 1563.20) | 1145.11 (783.04 to 1608.48) | 1187.73 (796.28 to 1652.82) | 1198.25 (823.72 to 1677.70) | 1167.41 (804.71 to 1627.10) | 1144.63 (792.83 to 1588.63) | 1134.61 (785.16 to 1561.82) | 1062.39 (738.57 to 1477.95) | 1008.66 (708.19 to 1383.62) | 945.86 (656.27 to 1295.09) |
| Timor-Leste | Both | Prevalence | 5743.20 (334.98 to 15851.22) | 18443.46 (4020.97 to 45268.63) | 25904.93 (4924.89 to 65034.81) | 34569.04 (5860.61 to 88112.07) | 41704.44 (7035.89 to 100000.00) | 46407.69 (8138.71 to 100000.00) | 49605.88 (9169.80 to 100000.00) | 52001.97 (9992.93 to 100000.00) | 53824.58 (10621.85 to 100000.00) | 55434.67 (11273.58 to 100000.00) | 56977.94 (11980.03 to 100000.00) | 58347.17 (12569.58 to 100000.00) | 59793.11 (13237.47 to 100000.00) | 60512.07 (13575.69 to 100000.00) | 60474.84 (13529.70 to 100000.00) | 60485.66 (13523.99 to 100000.00) | 60562.62 (13665.53 to 100000.00) | 60241.03 (13178.06 to 100000.00) | 60050.04 (12957.29 to 100000.00) | 59799.20 (12616.36 to 100000.00) |
| Timor-Leste | Female | DALY rates | 23.42 (15.29 to 34.95) | 62.52 (39.96 to 92.04) | 106.34 (69.37 to 158.95) | 196.79 (129.41 to 286.43) | 320.62 (208.33 to 478.50) | 430.53 (276.87 to 638.90) | 516.50 (332.53 to 746.31) | 590.41 (379.54 to 879.69) | 651.97 (418.77 to 946.64) | 706.97 (450.27 to 1025.13) | 759.17 (484.62 to 1106.21) | 814.38 (522.65 to 1205.02) | 864.84 (559.62 to 1259.97) | 879.14 (559.40 to 1284.02) | 860.83 (556.24 to 1255.81) | 839.50 (554.49 to 1196.81) | 821.28 (538.37 to 1181.86) | 804.29 (539.44 to 1149.24) | 783.03 (528.99 to 1126.05) | 762.05 (513.43 to 1087.57) |
| Timor-Leste | Female | Prevalence | 5786.46 (370.54 to 15910.31) | 15343.52 (989.22 to 42187.08) | 22533.78 (1583.15 to 61660.38) | 31345.49 (2660.64 to 84831.54) | 38896.11 (4074.40 to 100000.00) | 43889.32 (5317.30 to 100000.00) | 47179.91 (6308.70 to 100000.00) | 49689.57 (7191.21 to 100000.00) | 51646.89 (7927.83 to 100000.00) | 53296.99 (8606.82 to 100000.00) | 54858.90 (9290.11 to 100000.00) | 56335.83 (9954.25 to 100000.00) | 57845.00 (10673.53 to 100000.00) | 58582.66 (11053.48 to 100000.00) | 58581.91 (11040.64 to 100000.00) | 58583.32 (11043.04 to 100000.00) | 58581.94 (11037.91 to 100000.00) | 58583.44 (11065.29 to 100000.00) | 58583.10 (11054.34 to 100000.00) | 58581.57 (11040.38 to 100000.00) |
| Timor-Leste | Male | DALY rates | 16.50 (9.72 to 24.94) | 796.45 (530.59 to 1108.23) | 884.26 (603.32 to 1247.53) | 950.08 (638.26 to 1328.97) | 1035.31 (707.86 to 1443.24) | 1118.49 (763.56 to 1555.10) | 1198.84 (824.63 to 1675.07) | 1272.80 (869.25 to 1808.62) | 1325.15 (917.93 to 1857.55) | 1372.00 (942.66 to 1940.70) | 1418.75 (977.23 to 2032.90) | 1456.21 (1017.17 to 2032.27) | 1497.40 (1019.95 to 2078.94) | 1501.06 (1057.31 to 2103.11) | 1470.11 (1038.09 to 2065.26) | 1442.78 (1018.43 to 1997.62) | 1416.27 (995.81 to 1962.20) | 1389.77 (989.71 to 1910.15) | 1361.43 (972.47 to 1851.62) | 1330.02 (949.43 to 1810.36) |
| Timor-Leste | Male | Prevalence | 5701.53 (300.71 to 15794.28) | 21271.76 (6784.54 to 48244.78) | 28815.84 (7776.41 to 67956.45) | 37408.31 (8607.26 to 90978.60) | 44399.99 (9764.15 to 100000.00) | 48909.26 (10925.95 to 100000.00) | 51935.55 (11840.01 to 100000.00) | 54190.14 (12566.65 to 100000.00) | 55952.84 (13222.65 to 100000.00) | 57444.03 (13813.09 to 100000.00) | 58874.61 (14434.51 to 100000.00) | 60239.13 (15040.17 to 100000.00) | 61661.44 (15698.91 to 100000.00) | 62342.86 (16056.12 to 100000.00) | 62343.81 (16037.15 to 100000.00) | 62344.56 (16046.29 to 100000.00) | 62343.11 (16075.83 to 100000.00) | 62343.52 (16101.15 to 100000.00) | 62343.62 (16061.14 to 100000.00) | 62344.12 (16053.25 to 100000.00) |
| Togo | Both | DALY rates | 9.87 (5.96 to 14.81) | 40.52 (21.37 to 75.33) | 50.41 (26.41 to 87.98) | 63.60 (35.64 to 111.94) | 76.18 (41.76 to 127.61) | 85.75 (49.23 to 138.73) | 92.05 (52.39 to 150.25) | 99.82 (56.18 to 161.14) | 104.04 (60.67 to 163.98) | 111.28 (69.08 to 180.37) | 119.03 (71.01 to 192.14) | 125.44 (75.84 to 196.95) | 135.14 (83.84 to 210.32) | 136.32 (84.55 to 208.06) | 133.03 (79.57 to 208.66) | 129.00 (80.18 to 198.85) | 121.06 (78.51 to 186.19) | 113.71 (71.48 to 170.04) | 101.46 (63.54 to 152.31) | 85.27 (57.11 to 124.38) |
| Togo | Both | Prevalence | 722.08 (134.61 to 2915.74) | 1961.11 (416.57 to 7627.26) | 2769.78 (541.57 to 11016.14) | 3724.21 (701.05 to 14935.58) | 4545.86 (848.37 to 18279.51) | 5150.32 (963.48 to 20709.22) | 5582.82 (1046.82 to 22443.44) | 5949.56 (1135.12 to 23862.78) | 6243.64 (1198.17 to 25025.20) | 6530.77 (1272.41 to 26097.64) | 6812.91 (1363.23 to 27103.17) | 7097.03 (1444.04 to 28165.45) | 7433.48 (1555.78 to 29345.32) | 7584.76 (1590.19 to 29904.59) | 7577.27 (1586.01 to 29898.39) | 7560.17 (1571.39 to 29883.21) | 7507.04 (1524.30 to 29829.19) | 7462.76 (1486.53 to 29780.46) | 7366.20 (1397.70 to 29679.17) | 7232.86 (1275.54 to 29538.45) |
| Togo | Female | DALY rates | 11.53 (6.63 to 17.94) | 20.98 (12.05 to 32.47) | 22.42 (13.44 to 36.11) | 23.62 (14.39 to 36.18) | 25.21 (14.78 to 38.45) | 25.88 (15.93 to 38.41) | 26.53 (16.34 to 38.43) | 27.19 (16.45 to 40.71) | 27.33 (17.36 to 40.52) | 28.00 (17.85 to 42.78) | 28.23 (17.66 to 41.89) | 28.55 (17.02 to 42.18) | 28.86 (18.18 to 42.82) | 28.86 (18.75 to 42.34) | 28.34 (16.99 to 40.50) | 27.66 (17.31 to 41.10) | 27.29 (17.70 to 38.82) | 27.03 (17.09 to 39.90) | 26.12 (16.39 to 36.71) | 25.55 (16.90 to 36.21) |
| Togo | Female | Prevalence | 740.98 (151.70 to 2940.46) | 1808.65 (303.62 to 7431.99) | 2547.41 (359.55 to 10720.19) | 3405.37 (426.65 to 14535.65) | 4137.87 (485.23 to 17790.60) | 4670.49 (527.47 to 20153.98) | 5050.55 (557.95 to 21839.79) | 5358.48 (582.16 to 23205.92) | 5612.98 (601.98 to 24333.46) | 5841.55 (621.29 to 25346.91) | 6053.62 (637.97 to 26285.84) | 6279.35 (657.40 to 27286.34) | 6525.51 (677.31 to 28375.22) | 6648.67 (687.24 to 28919.81) | 6648.66 (687.40 to 28921.89) | 6648.67 (687.96 to 28920.05) | 6648.69 (687.78 to 28920.46) | 6648.67 (688.12 to 28920.57) | 6648.69 (688.05 to 28920.25) | 6648.69 (687.43 to 28920.28) |
| Togo | Male | DALY rates | 8.23 (4.50 to 13.28) | 59.78 (28.08 to 119.84) | 77.42 (36.25 to 147.95) | 103.79 (53.36 to 198.74) | 132.08 (66.32 to 230.29) | 155.46 (83.24 to 266.87) | 172.09 (92.52 to 291.22) | 187.84 (101.74 to 307.75) | 199.54 (110.76 to 322.68) | 211.27 (123.45 to 356.05) | 221.30 (126.04 to 366.30) | 232.54 (133.17 to 376.96) | 244.43 (146.59 to 391.35) | 247.38 (149.09 to 386.13) | 242.87 (142.66 to 387.28) | 239.24 (144.14 to 379.03) | 235.19 (147.54 to 370.98) | 229.64 (139.85 to 357.15) | 225.92 (137.82 to 350.23) | 220.11 (137.41 to 344.50) |
| Togo | Male | Prevalence | 703.58 (117.36 to 2891.56) | 2111.28 (529.12 to 7709.89) | 2984.43 (713.71 to 11148.67) | 4044.73 (962.52 to 15185.47) | 4993.23 (1214.00 to 18664.86) | 5708.97 (1421.72 to 21226.21) | 6232.96 (1588.30 to 23066.60) | 6665.92 (1730.41 to 24562.50) | 7028.84 (1865.08 to 25803.93) | 7358.36 (1971.79 to 26923.66) | 7668.04 (2079.60 to 27962.57) | 8000.90 (2197.57 to 29072.69) | 8367.11 (2334.95 to 30285.27) | 8552.20 (2402.67 to 30894.87) | 8551.55 (2402.88 to 30900.58) | 8551.73 (2403.88 to 30891.21) | 8551.74 (2402.61 to 30900.29) | 8551.68 (2407.08 to 30901.05) | 8551.47 (2403.84 to 30898.66) | 8551.75 (2406.95 to 30906.84) |
| Tonga | Both | DALY rates | 9.86 (5.88 to 15.04) | 34.39 (18.73 to 62.06) | 38.55 (20.43 to 69.33) | 44.23 (23.61 to 78.99) | 49.20 (25.62 to 87.16) | 53.19 (28.74 to 92.11) | 54.77 (29.95 to 94.82) | 55.45 (30.34 to 98.44) | 57.27 (30.33 to 98.57) | 59.61 (32.89 to 104.93) | 62.17 (34.15 to 107.42) | 64.48 (35.53 to 110.21) | 66.52 (36.53 to 111.76) | 67.00 (36.86 to 111.02) | 65.69 (36.62 to 111.82) | 64.20 (34.87 to 112.85) | 59.34 (33.96 to 97.81) | 50.90 (30.34 to 82.68) | 44.31 (26.79 to 69.76) | 42.22 (25.46 to 66.52) |
| Tonga | Both | Prevalence | 455.41 (93.55 to 3583.09) | 1212.61 (236.53 to 9071.96) | 1667.58 (265.64 to 13156.43) | 2187.14 (304.38 to 17766.22) | 2634.80 (340.49 to 21720.81) | 2967.15 (368.01 to 24651.68) | 3191.99 (385.36 to 26719.95) | 3360.23 (397.37 to 28399.78) | 3511.57 (412.11 to 29813.71) | 3648.54 (429.23 to 31053.97) | 3771.60 (444.63 to 32211.41) | 3894.57 (460.83 to 33411.58) | 4030.88 (480.07 to 34781.22) | 4096.48 (489.79 to 35437.36) | 4098.53 (491.37 to 35439.20) | 4093.84 (489.00 to 35436.56) | 4064.03 (474.26 to 35409.64) | 3995.61 (431.09 to 35353.96) | 3943.27 (396.50 to 35309.15) | 3933.13 (391.32 to 35299.77) |
| Tonga | Female | DALY rates | 11.65 (6.36 to 18.14) | 20.38 (11.86 to 31.89) | 20.61 (12.04 to 33.67) | 21.69 (12.95 to 34.47) | 22.09 (13.33 to 32.99) | 22.86 (13.88 to 34.95) | 22.79 (13.61 to 36.30) | 22.92 (13.73 to 34.92) | 23.02 (13.93 to 33.71) | 22.98 (14.35 to 34.57) | 23.41 (14.13 to 35.23) | 23.36 (14.38 to 34.87) | 23.16 (14.30 to 35.72) | 23.23 (14.32 to 34.68) | 22.27 (13.86 to 32.68) | 21.97 (13.41 to 32.19) | 21.61 (12.57 to 32.39) | 21.48 (13.00 to 31.64) | 21.01 (12.75 to 30.74) | 20.35 (12.85 to 30.22) |
| Tonga | Female | Prevalence | 472.33 (107.68 to 3600.68) | 1105.79 (189.34 to 9009.06) | 1530.79 (197.50 to 13068.54) | 2009.93 (206.40 to 17643.51) | 2420.78 (214.03 to 21563.15) | 2725.05 (219.50 to 24465.64) | 2937.60 (224.11 to 26520.40) | 3099.97 (227.39 to 28192.00) | 3235.90 (230.53 to 29591.20) | 3352.86 (232.00 to 30812.47) | 3455.90 (234.68 to 31951.76) | 3557.32 (236.90 to 33130.29) | 3670.84 (239.47 to 34478.94) | 3725.18 (240.36 to 35125.72) | 3725.22 (240.44 to 35125.24) | 3725.21 (240.44 to 35125.14) | 3725.23 (240.84 to 35125.11) | 3725.19 (240.23 to 35125.28) | 3725.22 (239.71 to 35125.68) | 3725.20 (240.32 to 35124.95) |
| Tonga | Male | DALY rates | 8.21 (4.50 to 13.61) | 47.39 (21.95 to 94.75) | 55.16 (24.98 to 109.90) | 65.00 (28.52 to 127.78) | 75.05 (34.40 to 145.93) | 83.37 (39.96 to 155.98) | 89.47 (43.80 to 166.49) | 94.17 (45.58 to 184.63) | 98.17 (46.07 to 185.31) | 101.57 (51.33 to 188.42) | 104.66 (51.57 to 191.19) | 107.33 (55.41 to 197.47) | 110.02 (55.40 to 195.68) | 110.11 (56.89 to 195.08) | 107.99 (54.87 to 195.59) | 106.40 (53.46 to 198.89) | 103.67 (53.43 to 180.56) | 101.67 (52.38 to 177.31) | 99.76 (52.86 to 175.69) | 97.85 (49.55 to 175.71) |
| Tonga | Male | Prevalence | 439.75 (77.21 to 3566.81) | 1311.79 (261.13 to 9130.36) | 1794.22 (307.71 to 13237.80) | 2350.43 (369.45 to 17879.28) | 2838.77 (430.47 to 21871.08) | 3207.92 (481.61 to 24836.70) | 3467.93 (520.62 to 26936.41) | 3670.00 (553.37 to 28647.08) | 3840.77 (582.57 to 30079.44) | 3987.16 (605.54 to 31330.54) | 4117.65 (628.04 to 32496.02) | 4246.01 (651.15 to 33704.71) | 4392.20 (680.79 to 35084.58) | 4462.12 (694.79 to 35744.26) | 4462.19 (696.44 to 35745.05) | 4462.19 (696.35 to 35747.74) | 4462.16 (697.36 to 35744.01) | 4462.27 (696.77 to 35748.58) | 4462.20 (697.15 to 35745.79) | 4462.09 (695.30 to 35744.50) |
| Uganda | Both | DALY rates | 10.13 (6.33 to 15.40) | 57.30 (31.56 to 101.76) | 82.52 (44.72 to 139.89) | 119.00 (70.20 to 196.13) | 154.50 (96.21 to 234.89) | 185.56 (119.30 to 279.05) | 213.88 (136.75 to 314.87) | 234.33 (152.41 to 338.10) | 248.82 (164.06 to 360.60) | 264.11 (174.47 to 388.49) | 277.35 (183.84 to 399.78) | 288.27 (193.42 to 412.65) | 298.82 (201.80 to 426.49) | 304.12 (206.03 to 422.98) | 291.99 (196.91 to 405.16) | 279.41 (191.43 to 389.25) | 259.96 (178.54 to 357.85) | 239.37 (166.00 to 326.91) | 208.61 (144.85 to 285.41) | 170.66 (118.72 to 230.31) |
| Uganda | Both | Prevalence | 1143.74 (223.47 to 3323.66) | 3306.12 (794.18 to 9137.70) | 4808.85 (1147.88 to 13336.50) | 6588.70 (1618.66 to 18212.21) | 8148.88 (2055.70 to 22408.09) | 9349.16 (2438.09 to 25545.00) | 10238.10 (2766.63 to 27796.97) | 10958.63 (3028.10 to 29657.48) | 11538.42 (3214.90 to 31199.91) | 12059.10 (3407.33 to 32507.47) | 12555.95 (3590.72 to 33761.11) | 13044.37 (3743.73 to 35040.13) | 13598.01 (3916.24 to 36498.10) | 13889.73 (4033.37 to 37214.95) | 13834.44 (3977.80 to 37161.94) | 13760.23 (3909.04 to 37094.23) | 13639.30 (3776.18 to 36957.81) | 13502.73 (3637.22 to 36813.68) | 13242.17 (3382.50 to 36540.67) | 12917.45 (3069.48 to 36196.03) |
| Uganda | Female | DALY rates | 11.94 (7.01 to 18.73) | 23.30 (13.79 to 36.17) | 25.65 (15.31 to 38.45) | 28.61 (17.84 to 42.18) | 31.63 (19.99 to 45.80) | 33.24 (20.62 to 50.38) | 35.12 (21.79 to 51.17) | 36.63 (23.67 to 53.43) | 38.25 (24.32 to 54.57) | 39.13 (25.63 to 56.73) | 39.84 (25.39 to 61.79) | 41.20 (25.63 to 60.09) | 42.29 (26.87 to 62.28) | 42.10 (27.60 to 61.12) | 41.76 (27.65 to 59.49) | 40.96 (27.04 to 59.48) | 40.38 (26.55 to 59.56) | 39.22 (25.06 to 54.81) | 38.37 (24.71 to 54.85) | 37.14 (24.59 to 52.85) |
| Uganda | Female | Prevalence | 1164.74 (243.46 to 3355.31) | 3034.33 (565.95 to 8898.03) | 4348.98 (743.19 to 12904.18) | 5855.66 (950.66 to 17489.02) | 7140.92 (1131.81 to 21393.58) | 8082.78 (1265.25 to 24251.34) | 8751.47 (1362.47 to 26277.00) | 9307.24 (1443.04 to 27958.16) | 9773.45 (1509.47 to 29368.87) | 10162.96 (1566.92 to 30547.71) | 10538.76 (1622.51 to 31684.24) | 10926.83 (1680.59 to 32854.44) | 11376.95 (1747.50 to 34211.64) | 11588.73 (1780.00 to 34851.20) | 11588.75 (1779.38 to 34851.45) | 11588.76 (1779.37 to 34850.96) | 11588.73 (1779.18 to 34850.25) | 11588.74 (1779.50 to 34849.95) | 11588.81 (1779.30 to 34852.33) | 11588.77 (1778.84 to 34850.77) |
| Uganda | Male | DALY rates | 8.34 (4.80 to 13.07) | 91.27 (43.19 to 177.15) | 139.50 (72.20 to 246.46) | 215.45 (120.56 to 369.29) | 293.10 (177.69 to 458.38) | 352.91 (225.42 to 537.42) | 400.23 (254.67 to 589.94) | 438.27 (278.93 to 636.25) | 470.10 (304.49 to 685.63) | 494.48 (324.01 to 735.95) | 516.06 (335.95 to 739.41) | 537.32 (357.76 to 776.31) | 560.18 (376.64 to 804.79) | 564.70 (379.79 to 790.33) | 553.11 (369.94 to 768.88) | 544.89 (370.88 to 763.51) | 531.81 (364.52 to 738.67) | 519.19 (355.32 to 713.16) | 510.85 (350.54 to 709.85) | 498.38 (339.53 to 691.11) |
| Uganda | Male | Prevalence | 1123.03 (205.55 to 3292.44) | 3577.71 (1021.53 to 9389.00) | 5269.64 (1549.96 to 13787.14) | 7370.92 (2320.44 to 19009.60) | 9285.88 (3139.37 to 23585.42) | 10740.54 (3831.51 to 27003.86) | 11787.98 (4334.09 to 29419.64) | 12662.07 (4730.89 to 31450.07) | 13393.11 (5049.43 to 33166.95) | 14000.61 (5334.41 to 34557.93) | 14583.34 (5591.28 to 35894.52) | 15178.86 (5851.27 to 37290.69) | 15860.98 (6156.91 to 38876.87) | 16178.06 (6291.60 to 39616.57) | 16177.91 (6293.36 to 39624.62) | 16177.90 (6304.13 to 39647.14) | 16178.00 (6287.34 to 39628.14) | 16178.46 (6285.78 to 39629.23) | 16177.58 (6287.34 to 39626.74) | 16178.45 (6290.17 to 39619.16) |
| United Republic of Tanzania | Both | DALY rates | 12.98 (8.26 to 18.95) | 168.19 (102.55 to 255.89) | 289.22 (190.72 to 414.35) | 389.76 (261.17 to 543.41) | 420.59 (279.97 to 585.57) | 433.83 (297.48 to 600.96) | 455.63 (312.29 to 626.00) | 475.19 (326.60 to 659.26) | 475.07 (324.71 to 663.28) | 483.57 (334.04 to 669.23) | 492.53 (340.53 to 680.64) | 503.92 (347.77 to 686.90) | 514.61 (354.92 to 701.33) | 518.61 (361.89 to 714.95) | 506.89 (356.68 to 696.02) | 485.19 (338.69 to 656.60) | 451.60 (315.42 to 604.34) | 413.34 (294.15 to 550.43) | 373.39 (269.40 to 491.97) | 312.64 (223.47 to 409.20) |
| United Republic of Tanzania | Both | Prevalence | 2778.08 (1362.78 to 4732.90) | 8437.29 (4585.14 to 13721.28) | 12640.28 (7054.75 to 20444.74) | 17149.32 (9523.72 to 27667.73) | 20558.52 (11266.30 to 33329.76) | 22991.11 (12480.29 to 37333.36) | 24827.66 (13488.58 to 40299.73) | 26351.76 (14317.18 to 42744.66) | 27503.57 (14885.84 to 44667.74) | 28560.61 (15446.85 to 46394.55) | 29580.84 (16006.15 to 48059.96) | 30663.86 (16592.59 to 49787.88) | 31893.27 (17265.20 to 51778.21) | 32515.96 (17613.34 to 52762.90) | 32488.68 (17598.74 to 52733.24) | 32396.47 (17510.01 to 52649.50) | 32197.20 (17309.91 to 52453.81) | 31928.82 (17041.00 to 52199.30) | 31628.08 (16755.96 to 51905.78) | 31112.70 (16253.91 to 51417.90) |
| United Republic of Tanzania | Female | DALY rates | 15.28 (9.38 to 23.48) | 32.31 (20.35 to 47.55) | 42.46 (26.67 to 63.68) | 55.87 (35.87 to 80.67) | 71.31 (46.61 to 108.47) | 84.91 (57.91 to 125.07) | 96.34 (63.50 to 141.25) | 106.05 (71.01 to 155.04) | 116.29 (78.84 to 167.88) | 124.77 (81.60 to 183.25) | 134.35 (87.57 to 195.70) | 143.81 (95.92 to 205.70) | 156.51 (101.96 to 225.12) | 160.56 (107.04 to 232.75) | 157.97 (106.37 to 231.33) | 153.95 (103.66 to 221.79) | 149.85 (101.37 to 211.47) | 147.63 (101.99 to 207.47) | 144.02 (100.67 to 198.78) | 139.84 (95.77 to 197.32) |
| United Republic of Tanzania | Female | Prevalence | 2804.06 (1389.31 to 4764.25) | 7316.56 (3586.89 to 12480.04) | 10611.43 (5170.93 to 18132.76) | 14411.13 (7018.46 to 24625.95) | 17698.33 (8636.91 to 30209.47) | 20134.53 (9846.67 to 34329.01) | 21883.20 (10728.58 to 37269.94) | 23320.90 (11453.21 to 39679.63) | 24543.54 (12078.35 to 41723.79) | 25585.29 (12612.60 to 43456.07) | 26588.58 (13128.32 to 45127.84) | 27633.16 (13671.70 to 46849.07) | 28847.62 (14314.27 to 48856.37) | 29419.65 (14601.34 to 49792.54) | 29419.68 (14609.17 to 49794.61) | 29419.41 (14607.76 to 49799.15) | 29419.49 (14606.78 to 49790.14) | 29419.72 (14604.05 to 49794.85) | 29419.45 (14607.49 to 49794.71) | 29419.63 (14609.09 to 49793.14) |
| United Republic of Tanzania | Male | DALY rates | 10.73 (6.18 to 16.39) | 302.91 (183.43 to 474.26) | 538.09 (347.75 to 782.39) | 750.02 (500.20 to 1045.14) | 834.95 (553.85 to 1155.65) | 856.04 (586.42 to 1198.72) | 864.08 (589.12 to 1191.11) | 869.45 (589.39 to 1196.91) | 873.21 (603.34 to 1223.99) | 875.23 (597.81 to 1221.40) | 876.39 (598.43 to 1222.39) | 877.31 (607.73 to 1195.60) | 878.32 (599.25 to 1209.06) | 868.66 (601.59 to 1195.10) | 854.03 (595.36 to 1174.14) | 835.19 (571.29 to 1119.11) | 815.02 (573.86 to 1092.26) | 796.03 (558.07 to 1067.45) | 779.88 (559.44 to 1024.49) | 764.72 (541.51 to 1010.12) |
| United Republic of Tanzania | Male | Prevalence | 2752.70 (1335.86 to 4702.27) | 9548.52 (5673.08 to 14904.34) | 14686.45 (9008.20 to 22574.58) | 20103.89 (12525.15 to 30733.20) | 23951.53 (14593.06 to 36837.61) | 26447.63 (15795.26 to 40886.92) | 28174.92 (16691.33 to 43694.60) | 29588.84 (17413.95 to 46028.46) | 30788.28 (18021.10 to 48015.46) | 31808.49 (18551.39 to 49698.01) | 32787.67 (19053.02 to 51282.41) | 33806.45 (19585.91 to 52927.78) | 34986.69 (20229.79 to 54854.40) | 35543.05 (20524.38 to 55741.38) | 35542.14 (20525.73 to 55750.53) | 35542.19 (20528.18 to 55743.54) | 35542.48 (20520.76 to 55757.39) | 35542.48 (20502.82 to 55745.90) | 35542.18 (20515.97 to 55738.61) | 35542.20 (20507.80 to 55760.66) |
| Vanuatu | Both | DALY rates | 16.88 (10.64 to 24.45) | 342.34 (226.01 to 492.85) | 468.54 (313.77 to 657.73) | 502.76 (343.76 to 711.13) | 529.72 (361.91 to 732.05) | 546.10 (378.09 to 755.41) | 561.00 (381.61 to 775.90) | 590.90 (405.09 to 824.47) | 618.22 (421.91 to 854.87) | 626.60 (424.89 to 883.79) | 640.49 (437.87 to 896.53) | 642.05 (442.36 to 897.08) | 651.06 (452.68 to 891.32) | 651.43 (453.32 to 902.05) | 635.80 (443.61 to 877.14) | 616.70 (428.01 to 851.10) | 591.45 (414.77 to 799.84) | 596.55 (418.94 to 794.13) | 557.85 (400.10 to 752.23) | 499.64 (356.78 to 668.61) |
| Vanuatu | Both | Prevalence | 4468.56 (165.78 to 18456.88) | 13918.33 (2708.03 to 49698.92) | 20041.89 (3822.73 to 72407.04) | 26192.00 (4178.52 to 96973.05) | 30278.19 (4393.94 to 100000.00) | 32557.23 (4584.14 to 100000.00) | 34016.86 (4773.60 to 100000.00) | 35178.19 (5045.40 to 100000.00) | 36170.74 (5343.44 to 100000.00) | 36873.15 (5446.54 to 100000.00) | 37569.99 (5636.81 to 100000.00) | 38200.83 (5729.39 to 100000.00) | 38921.83 (5878.21 to 100000.00) | 39275.36 (5969.64 to 100000.00) | 39245.59 (5937.34 to 100000.00) | 39192.44 (5878.58 to 100000.00) | 39095.21 (5744.67 to 100000.00) | 39216.04 (5902.96 to 100000.00) | 39016.48 (5653.66 to 100000.00) | 38638.49 (5161.69 to 100000.00) |
| Vanuatu | Female | DALY rates | 19.88 (12.05 to 29.16) | 46.19 (29.05 to 69.18) | 69.79 (44.91 to 103.09) | 109.33 (70.25 to 160.43) | 151.68 (100.31 to 223.35) | 180.39 (117.20 to 258.78) | 200.27 (128.10 to 282.43) | 214.65 (139.79 to 321.10) | 226.57 (148.19 to 329.32) | 238.11 (149.31 to 338.79) | 246.48 (159.81 to 355.09) | 255.60 (167.52 to 377.65) | 265.90 (173.19 to 391.22) | 269.18 (176.69 to 394.46) | 264.39 (173.92 to 380.97) | 259.60 (171.93 to 372.55) | 255.48 (170.68 to 365.86) | 250.32 (166.22 to 360.68) | 244.35 (164.56 to 349.93) | 240.14 (160.36 to 343.76) |
| Vanuatu | Female | Prevalence | 4491.58 (194.00 to 18459.26) | 11531.39 (458.77 to 47536.15) | 16823.36 (687.36 to 69276.67) | 23027.85 (1075.14 to 93857.11) | 27519.83 (1468.81 to 100000.00) | 29971.63 (1734.03 to 100000.00) | 31518.10 (1926.93 to 100000.00) | 32616.36 (2064.42 to 100000.00) | 33515.21 (2194.47 to 100000.00) | 34230.22 (2297.56 to 100000.00) | 34900.34 (2394.52 to 100000.00) | 35577.08 (2505.85 to 100000.00) | 36316.93 (2623.48 to 100000.00) | 36649.24 (2680.33 to 100000.00) | 36649.04 (2689.22 to 100000.00) | 36649.47 (2676.37 to 100000.00) | 36649.44 (2679.47 to 100000.00) | 36649.88 (2684.80 to 100000.00) | 36649.36 (2688.96 to 100000.00) | 36648.82 (2683.54 to 100000.00) |
| Vanuatu | Male | DALY rates | 14.10 (8.44 to 21.23) | 611.48 (404.19 to 885.04) | 834.64 (556.57 to 1172.47) | 884.96 (600.99 to 1261.70) | 920.59 (620.72 to 1294.17) | 938.50 (645.30 to 1298.62) | 948.06 (645.62 to 1314.95) | 959.34 (654.50 to 1354.36) | 962.99 (653.57 to 1335.13) | 964.23 (647.91 to 1353.45) | 966.64 (662.11 to 1355.76) | 964.05 (656.90 to 1339.16) | 965.79 (667.03 to 1307.95) | 953.85 (652.05 to 1313.02) | 937.29 (647.44 to 1283.84) | 920.06 (631.97 to 1249.47) | 901.62 (630.54 to 1199.01) | 884.90 (618.91 to 1167.18) | 867.34 (616.96 to 1153.69) | 853.64 (601.81 to 1141.32) |
| Vanuatu | Male | Prevalence | 4447.28 (139.17 to 18454.68) | 16087.61 (4695.85 to 51847.97) | 22996.93 (6643.69 to 75281.12) | 29265.78 (7182.97 to 100000.00) | 33130.22 (7381.75 to 100000.00) | 35331.44 (7598.42 to 100000.00) | 36697.95 (7772.20 to 100000.00) | 37686.75 (7866.04 to 100000.00) | 38508.36 (7953.70 to 100000.00) | 39170.08 (8032.19 to 100000.00) | 39779.82 (8087.48 to 100000.00) | 40386.99 (8207.75 to 100000.00) | 41050.37 (8293.01 to 100000.00) | 41353.09 (8351.53 to 100000.00) | 41353.25 (8367.28 to 100000.00) | 41352.78 (8342.46 to 100000.00) | 41353.12 (8373.85 to 100000.00) | 41353.27 (8338.89 to 100000.00) | 41353.35 (8356.49 to 100000.00) | 41352.78 (8350.73 to 100000.00) |
| Viet Nam | Both | DALY rates | 11.69 (7.48 to 17.57) | 75.91 (41.94 to 130.12) | 119.86 (68.87 to 199.13) | 181.73 (115.15 to 274.63) | 238.24 (153.98 to 348.21) | 281.33 (187.80 to 405.53) | 310.22 (203.71 to 443.51) | 324.32 (211.63 to 458.73) | 340.75 (227.41 to 483.92) | 342.47 (229.32 to 478.42) | 346.53 (234.63 to 489.39) | 362.57 (237.15 to 500.48) | 366.46 (248.33 to 501.94) | 347.52 (234.91 to 476.04) | 326.24 (222.34 to 443.62) | 302.83 (207.37 to 417.36) | 272.37 (188.73 to 366.82) | 227.98 (160.24 to 307.33) | 187.44 (132.34 to 250.90) | 138.45 (100.07 to 183.40) |
| Viet Nam | Both | Prevalence | 1615.81 (254.78 to 5190.24) | 4343.74 (910.74 to 13060.21) | 6376.20 (1403.46 to 19047.25) | 8836.33 (2108.45 to 26171.41) | 10948.37 (2760.63 to 32270.42) | 12515.08 (3270.82 to 36749.28) | 13618.50 (3648.02 to 39843.00) | 14440.67 (3840.37 to 42302.70) | 15189.99 (4048.52 to 44450.32) | 15719.26 (4128.72 to 46164.80) | 16259.28 (4242.47 to 47833.71) | 16929.17 (4468.32 to 49672.39) | 17558.47 (4576.90 to 51605.67) | 17717.39 (4467.63 to 52410.12) | 17583.43 (4328.73 to 52268.56) | 17427.19 (4173.74 to 52106.30) | 17198.51 (3931.80 to 51885.47) | 16832.52 (3560.27 to 51508.18) | 16494.94 (3202.16 to 51187.97) | 16059.96 (2788.95 to 50769.90) |
| Viet Nam | Female | DALY rates | 13.73 (8.30 to 21.68) | 25.48 (15.84 to 38.45) | 28.92 (16.64 to 44.54) | 33.50 (20.34 to 50.27) | 38.23 (24.73 to 56.82) | 42.33 (26.72 to 62.10) | 44.45 (29.15 to 64.19) | 47.56 (30.32 to 69.49) | 49.10 (31.01 to 73.19) | 50.89 (32.40 to 72.64) | 52.94 (33.61 to 80.26) | 54.85 (35.33 to 82.39) | 56.57 (38.00 to 83.25) | 56.94 (38.05 to 81.21) | 56.03 (36.13 to 82.97) | 54.18 (35.55 to 77.46) | 53.18 (34.68 to 76.63) | 52.28 (35.91 to 74.66) | 50.59 (34.45 to 70.64) | 49.26 (33.38 to 69.26) |
| Viet Nam | Female | Prevalence | 1637.05 (273.19 to 5216.28) | 3940.55 (589.48 to 12736.19) | 5648.14 (780.86 to 18423.42) | 7639.90 (1012.92 to 25043.28) | 9330.30 (1214.63 to 30644.41) | 10572.60 (1365.30 to 34753.31) | 11447.66 (1473.88 to 37642.47) | 12166.54 (1565.88 to 40011.13) | 12785.49 (1644.88 to 42050.09) | 13308.84 (1714.69 to 43771.33) | 13810.22 (1778.43 to 45419.79) | 14325.71 (1847.13 to 47109.87) | 14910.83 (1927.74 to 49027.94) | 15192.22 (1963.55 to 49950.56) | 15192.19 (1966.58 to 49950.60) | 15192.24 (1962.64 to 49947.87) | 15192.21 (1967.26 to 49949.64) | 15192.14 (1965.69 to 49947.63) | 15192.18 (1966.59 to 49947.04) | 15192.22 (1966.90 to 49946.35) |
| Viet Nam | Male | DALY rates | 9.74 (5.29 to 15.21) | 124.15 (63.71 to 223.10) | 206.61 (116.06 to 353.40) | 332.25 (206.56 to 507.77) | 454.34 (291.71 to 661.37) | 541.98 (355.87 to 793.14) | 597.54 (392.58 to 861.23) | 639.17 (416.52 to 905.16) | 671.28 (447.44 to 957.44) | 696.30 (465.06 to 976.45) | 713.73 (483.12 to 1011.91) | 725.39 (475.12 to 999.92) | 739.11 (497.43 to 1016.55) | 735.67 (495.36 to 995.63) | 722.54 (491.64 to 984.14) | 710.48 (483.42 to 979.57) | 697.56 (480.95 to 944.35) | 684.14 (470.32 to 926.32) | 670.30 (459.75 to 891.76) | 655.55 (461.88 to 883.88) |
| Viet Nam | Male | Prevalence | 1595.62 (236.65 to 5165.48) | 4729.43 (1214.99 to 13401.90) | 7070.71 (1988.59 to 19662.12) | 10051.13 (3216.00 to 27331.00) | 12696.47 (4402.37 to 34042.71) | 14633.57 (5312.51 to 38981.55) | 15965.37 (5853.39 to 42342.83) | 17027.80 (6308.53 to 45046.78) | 17915.03 (6674.50 to 47316.17) | 18644.30 (6966.09 to 49231.95) | 19322.28 (7234.15 to 51028.33) | 19998.87 (7416.31 to 52867.07) | 20742.31 (7644.91 to 54885.02) | 21090.45 (7729.83 to 55868.76) | 21090.51 (7742.02 to 55865.80) | 21091.29 (7746.35 to 55855.30) | 21090.41 (7732.22 to 55879.79) | 21091.32 (7746.28 to 55845.49) | 21091.45 (7743.51 to 55867.94) | 21090.74 (7753.60 to 55848.91) |
| Yemen | Both | DALY rates | 9.21 (5.58 to 13.56) | 28.16 (14.75 to 52.61) | 29.20 (15.78 to 54.09) | 29.99 (15.36 to 52.84) | 30.63 (16.38 to 55.74) | 30.43 (17.09 to 55.31) | 30.70 (16.47 to 54.22) | 30.96 (16.72 to 57.29) | 32.68 (17.83 to 58.58) | 32.61 (17.79 to 58.57) | 32.80 (17.40 to 59.56) | 33.05 (17.30 to 61.33) | 33.45 (18.62 to 63.34) | 31.63 (16.96 to 55.66) | 29.88 (16.45 to 52.56) | 28.32 (15.93 to 47.00) | 26.20 (15.18 to 43.48) | 24.21 (14.24 to 37.19) | 22.31 (13.57 to 34.12) | 20.34 (12.76 to 29.71) |
| Yemen | Both | Prevalence | 195.31 (95.17 to 600.45) | 523.02 (232.43 to 1536.67) | 651.88 (248.27 to 2130.57) | 799.64 (266.88 to 2821.16) | 924.41 (278.11 to 3417.21) | 1014.59 (288.76 to 3849.74) | 1081.11 (296.84 to 4156.79) | 1135.63 (303.68 to 4402.49) | 1190.35 (312.23 to 4620.58) | 1228.01 (316.64 to 4801.25) | 1267.24 (322.63 to 4974.28) | 1306.78 (327.70 to 5152.82) | 1352.54 (334.03 to 5357.47) | 1364.03 (333.60 to 5446.30) | 1353.35 (328.86 to 5438.13) | 1342.60 (321.60 to 5429.61) | 1330.18 (316.81 to 5420.07) | 1317.07 (307.05 to 5409.82) | 1305.27 (300.09 to 5400.68) | 1291.08 (284.95 to 5389.20) |
| Yemen | Female | DALY rates | 10.84 (6.04 to 16.60) | 18.94 (10.80 to 30.19) | 18.83 (10.78 to 29.20) | 19.00 (10.90 to 29.29) | 19.30 (11.20 to 30.78) | 19.04 (11.49 to 28.57) | 18.82 (11.19 to 28.40) | 18.61 (10.88 to 28.21) | 18.71 (11.26 to 28.41) | 18.83 (10.80 to 28.56) | 18.55 (10.87 to 27.54) | 18.53 (10.92 to 28.41) | 18.73 (11.10 to 29.02) | 18.34 (10.97 to 28.78) | 18.22 (11.33 to 28.14) | 18.15 (10.05 to 27.44) | 17.42 (10.27 to 26.52) | 17.26 (10.22 to 26.00) | 16.79 (10.35 to 24.67) | 16.31 (10.05 to 23.27) |
| Yemen | Female | Prevalence | 211.03 (110.40 to 615.52) | 452.73 (197.43 to 1487.04) | 574.21 (204.71 to 2074.17) | 715.98 (214.30 to 2759.45) | 838.78 (222.40 to 3352.68) | 927.96 (228.06 to 3783.99) | 990.60 (232.17 to 4087.18) | 1040.90 (234.86 to 4329.86) | 1084.10 (237.71 to 4538.44) | 1121.23 (239.33 to 4718.33) | 1156.40 (241.88 to 4888.06) | 1192.95 (244.56 to 5064.70) | 1234.38 (247.73 to 5264.62) | 1254.10 (248.34 to 5359.76) | 1254.14 (248.27 to 5360.14) | 1254.12 (249.03 to 5359.86) | 1254.11 (248.94 to 5360.20) | 1254.11 (248.54 to 5360.18) | 1254.10 (249.14 to 5360.55) | 1254.13 (248.46 to 5360.29) |
| Yemen | Male | DALY rates | 7.67 (4.39 to 12.18) | 36.65 (15.88 to 77.72) | 38.39 (16.41 to 83.11) | 40.12 (17.86 to 84.08) | 42.28 (19.56 to 88.29) | 43.19 (19.38 to 87.71) | 43.88 (19.90 to 92.54) | 44.28 (20.43 to 93.93) | 45.26 (20.31 to 93.54) | 45.47 (20.65 to 92.96) | 45.67 (20.17 to 93.66) | 46.01 (20.55 to 93.44) | 46.24 (22.04 to 96.42) | 45.33 (19.99 to 90.79) | 44.47 (20.54 to 91.13) | 43.81 (19.98 to 86.40) | 43.18 (20.78 to 83.49) | 41.92 (19.66 to 82.45) | 40.89 (19.01 to 83.19) | 40.69 (18.59 to 79.12) |
| Yemen | Male | Prevalence | 180.41 (79.03 to 586.54) | 587.72 (249.24 to 1604.55) | 720.68 (273.30 to 2184.46) | 876.67 (295.39 to 2877.97) | 1012.52 (315.43 to 3483.60) | 1111.65 (330.40 to 3923.41) | 1181.49 (340.64 to 4234.00) | 1237.71 (349.36 to 4480.77) | 1286.04 (356.77 to 4694.55) | 1327.75 (361.88 to 4878.70) | 1367.28 (369.13 to 5052.10) | 1408.37 (375.39 to 5231.48) | 1455.18 (381.80 to 5438.12) | 1477.41 (385.45 to 5535.55) | 1477.40 (385.12 to 5535.67) | 1477.42 (385.10 to 5535.88) | 1477.34 (385.70 to 5535.90) | 1477.38 (385.10 to 5536.23) | 1477.40 (385.63 to 5535.66) | 1477.35 (385.25 to 5534.98) |
| Zambia | Both | DALY rates | 12.62 (7.94 to 18.29) | 160.81 (100.90 to 246.65) | 274.94 (181.88 to 396.30) | 384.24 (256.12 to 537.36) | 426.26 (287.84 to 591.86) | 445.89 (302.52 to 621.82) | 463.97 (312.99 to 640.84) | 476.74 (325.56 to 655.16) | 476.87 (327.86 to 656.69) | 480.06 (331.05 to 662.74) | 492.25 (337.98 to 680.75) | 510.19 (351.84 to 696.38) | 527.13 (369.30 to 722.26) | 533.16 (375.27 to 735.04) | 539.89 (373.70 to 742.74) | 539.61 (375.29 to 728.87) | 501.17 (350.48 to 673.27) | 462.69 (330.04 to 613.33) | 437.08 (314.93 to 583.59) | 377.58 (271.55 to 499.93) |
| Zambia | Both | Prevalence | 2654.78 (1039.53 to 5595.32) | 8146.47 (3748.49 to 16121.56) | 12227.25 (5731.98 to 23794.84) | 16652.50 (7862.78 to 32298.40) | 20044.61 (9450.94 to 39202.80) | 22451.35 (10483.95 to 44207.75) | 24227.81 (11235.52 to 47841.82) | 25636.69 (11841.17 to 50736.69) | 26759.08 (12280.19 to 53128.12) | 27753.94 (12689.09 to 55200.34) | 28762.30 (13141.74 to 57210.41) | 29870.86 (13656.43 to 59387.73) | 31122.89 (14251.17 to 61835.35) | 31761.80 (14579.66 to 63048.12) | 31896.78 (14718.65 to 63190.04) | 31988.31 (14803.89 to 63280.93) | 31755.84 (14583.55 to 63045.68) | 31498.82 (14309.68 to 62777.07) | 31349.20 (14167.35 to 62631.40) | 30854.94 (13673.69 to 62125.90) |
| Zambia | Female | DALY rates | 14.83 (8.93 to 21.79) | 32.13 (20.37 to 49.06) | 41.22 (26.60 to 60.89) | 54.41 (33.93 to 80.18) | 68.54 (43.91 to 100.58) | 82.53 (55.45 to 116.44) | 92.52 (60.40 to 134.54) | 102.18 (68.65 to 150.29) | 110.95 (73.11 to 164.13) | 118.80 (80.82 to 173.23) | 126.70 (84.20 to 184.07) | 134.90 (88.92 to 195.38) | 144.99 (95.54 to 207.33) | 149.04 (100.12 to 214.89) | 147.42 (97.45 to 210.07) | 145.31 (98.02 to 209.69) | 141.26 (96.71 to 195.38) | 138.92 (93.49 to 198.53) | 135.79 (94.05 to 193.45) | 131.40 (89.72 to 182.61) |
| Zambia | Female | Prevalence | 2684.01 (1067.96 to 5636.17) | 7095.31 (2773.57 to 14980.81) | 10297.75 (3987.04 to 21800.36) | 13964.37 (5395.00 to 29573.26) | 17122.96 (6629.56 to 36231.28) | 19477.64 (7563.61 to 41168.06) | 21174.51 (8248.79 to 44707.77) | 22552.83 (8806.52 to 47567.61) | 23735.21 (9291.87 to 50013.38) | 24750.04 (9714.76 to 52111.39) | 25711.30 (10115.35 to 54084.45) | 26718.25 (10540.40 to 56148.45) | 27877.06 (11026.51 to 58510.09) | 28437.75 (11275.60 to 59659.74) | 28437.91 (11277.71 to 59648.69) | 28437.68 (11271.48 to 59648.62) | 28437.76 (11275.78 to 59656.08) | 28437.73 (11274.62 to 59656.11) | 28437.78 (11273.58 to 59653.98) | 28437.73 (11272.13 to 59651.95) |
| Zambia | Male | DALY rates | 10.42 (6.07 to 16.34) | 291.29 (176.89 to 450.02) | 514.38 (338.57 to 740.21) | 736.43 (482.52 to 1029.17) | 828.26 (552.60 to 1153.57) | 854.10 (576.92 to 1184.76) | 859.13 (581.32 to 1209.61) | 865.18 (590.80 to 1193.71) | 868.60 (598.47 to 1204.98) | 869.35 (596.40 to 1190.66) | 871.88 (591.45 to 1209.28) | 872.54 (601.25 to 1187.87) | 870.87 (606.83 to 1198.26) | 859.90 (600.06 to 1178.45) | 845.31 (579.75 to 1162.84) | 828.29 (576.27 to 1125.98) | 808.47 (563.45 to 1086.92) | 789.57 (558.52 to 1051.48) | 772.36 (555.43 to 1042.66) | 757.87 (542.24 to 1000.75) |
| Zambia | Male | Prevalence | 2625.73 (1011.29 to 5554.72) | 9212.23 (4763.47 to 17092.34) | 14203.97 (7686.70 to 25777.32) | 19522.92 (10759.34 to 35243.42) | 23327.87 (12546.47 to 42542.10) | 25792.12 (13718.97 to 47622.67) | 27475.92 (14434.51 to 51175.83) | 28834.89 (15010.51 to 54023.26) | 29996.22 (15516.42 to 56462.55) | 30990.86 (15949.71 to 58528.92) | 31930.93 (16344.57 to 60456.91) | 32914.82 (16732.45 to 62515.37) | 34042.51 (17208.85 to 64826.42) | 34589.31 (17454.73 to 65930.34) | 34588.41 (17470.98 to 65945.87) | 34587.86 (17459.05 to 65940.27) | 34588.83 (17481.02 to 65939.73) | 34589.27 (17461.56 to 65927.98) | 34589.20 (17461.44 to 65944.85) | 34589.03 (17455.66 to 65947.66) |
| Zimbabwe | Both | DALY rates | 9.62 (5.84 to 15.25) | 30.26 (16.51 to 54.87) | 32.43 (17.43 to 57.08) | 35.17 (18.77 to 62.62) | 36.77 (19.98 to 66.30) | 38.07 (20.23 to 67.72) | 39.17 (20.89 to 71.60) | 40.87 (21.91 to 72.25) | 42.19 (23.21 to 74.29) | 43.04 (23.07 to 76.10) | 44.08 (24.21 to 80.54) | 46.26 (23.97 to 86.07) | 47.14 (25.95 to 84.06) | 45.50 (25.49 to 79.42) | 44.22 (23.37 to 77.14) | 41.38 (22.95 to 71.37) | 36.88 (21.40 to 63.17) | 33.12 (19.49 to 54.08) | 29.78 (17.67 to 45.86) | 26.40 (16.32 to 40.23) |
| Zimbabwe | Both | Prevalence | 319.45 (116.87 to 1073.57) | 840.47 (296.84 to 2743.79) | 1116.69 (342.78 to 3882.81) | 1435.26 (395.90 to 5192.97) | 1704.92 (441.03 to 6306.54) | 1904.31 (470.61 to 7129.27) | 2049.85 (496.59 to 7717.49) | 2169.68 (518.40 to 8194.43) | 2269.83 (537.02 to 8595.60) | 2361.97 (558.69 to 8948.04) | 2445.71 (574.76 to 9279.12) | 2539.41 (601.15 to 9623.36) | 2640.68 (623.42 to 10022.03) | 2672.09 (618.04 to 10189.18) | 2667.43 (613.95 to 10183.54) | 2650.04 (605.10 to 10165.44) | 2618.00 (586.60 to 10131.41) | 2593.71 (571.11 to 10105.88) | 2565.37 (549.24 to 10075.85) | 2539.30 (526.28 to 10049.07) |
| Zimbabwe | Female | DALY rates | 11.29 (6.75 to 17.56) | 19.63 (11.82 to 30.36) | 19.64 (11.58 to 30.13) | 20.33 (11.92 to 31.26) | 20.32 (12.05 to 30.66) | 20.58 (11.90 to 31.37) | 20.36 (11.34 to 31.28) | 20.68 (12.62 to 31.99) | 20.84 (12.60 to 31.11) | 20.63 (12.06 to 31.87) | 20.88 (12.10 to 32.29) | 21.04 (13.16 to 31.83) | 21.12 (12.94 to 32.58) | 20.84 (12.21 to 31.05) | 20.67 (12.51 to 30.51) | 20.29 (12.56 to 31.41) | 19.56 (11.61 to 29.91) | 18.95 (10.93 to 28.22) | 18.86 (11.31 to 29.28) | 18.44 (10.83 to 27.73) |
| Zimbabwe | Female | Prevalence | 336.64 (132.70 to 1095.32) | 758.27 (248.32 to 2660.21) | 1020.05 (279.64 to 3783.23) | 1321.04 (315.63 to 5074.67) | 1577.08 (344.48 to 6172.85) | 1766.51 (366.01 to 6984.88) | 1901.09 (381.58 to 7561.69) | 2009.45 (393.74 to 8026.79) | 2101.12 (404.16 to 8418.46) | 2180.08 (412.86 to 8756.83) | 2255.03 (420.56 to 9078.53) | 2331.30 (429.30 to 9405.14) | 2421.65 (440.88 to 9792.19) | 2462.69 (445.32 to 9968.36) | 2462.72 (445.12 to 9968.91) | 2462.72 (445.55 to 9967.97) | 2462.71 (445.75 to 9967.81) | 2462.71 (445.57 to 9968.12) | 2462.71 (444.56 to 9968.35) | 2462.73 (445.84 to 9968.65) |
| Zimbabwe | Male | DALY rates | 7.98 (4.08 to 13.20) | 41.00 (17.47 to 90.14) | 45.38 (20.21 to 90.23) | 50.50 (22.29 to 102.59) | 54.82 (24.83 to 107.58) | 58.29 (24.56 to 117.84) | 60.67 (27.50 to 120.06) | 63.16 (29.40 to 120.54) | 65.53 (29.75 to 127.21) | 65.88 (31.21 to 126.84) | 67.28 (32.00 to 133.38) | 69.01 (31.79 to 135.23) | 70.25 (33.96 to 138.04) | 70.49 (34.90 to 134.15) | 69.19 (32.40 to 133.05) | 67.77 (33.51 to 129.74) | 66.60 (31.66 to 127.93) | 64.55 (29.32 to 122.64) | 63.73 (31.55 to 119.96) | 62.29 (29.85 to 120.19) |
| Zimbabwe | Male | Prevalence | 302.48 (100.03 to 1052.10) | 923.54 (322.99 to 2799.10) | 1214.51 (380.75 to 3959.85) | 1553.31 (451.54 to 5300.16) | 1845.20 (512.48 to 6444.13) | 2063.53 (560.67 to 7291.76) | 2219.91 (594.67 to 7893.54) | 2346.58 (623.49 to 8379.47) | 2454.31 (650.69 to 8789.30) | 2547.46 (671.68 to 9143.04) | 2636.35 (692.25 to 9479.67) | 2727.15 (715.52 to 9820.22) | 2835.17 (740.90 to 10226.14) | 2884.43 (753.58 to 10413.10) | 2884.50 (752.53 to 10411.13) | 2884.38 (752.62 to 10412.50) | 2884.45 (753.55 to 10412.09) | 2884.51 (752.39 to 10411.67) | 2884.50 (752.64 to 10410.01) | 2884.51 (751.50 to 10411.63) |
| **Abbreviations:** GBD, Global Burden of Disease, DALYs, disability-adjusted life years; SDI, socio-demographic index; UI, uncertainty interval. | | | | | | | | | | | | | | | | | | | | | | |
