# Supplementary material for: Global, regional, and national disability-adjusted life years and prevalence of lymphatic filariasis from 1990 to 2021: A trend and health inequality analysis based on the global burden of disease study 2021
Source: PLoS Negl Trop Dis. 2025 Apr 29;19(4):e0013017. doi: 10.1371/journal.pntd.0013017 (PMC12040265; doi:10.1371/journal.pntd.0013017)
Supplement: S7 Table — Abbreviations: GBD, Global Burden of Disease, DALYs, disability-adjusted life years; SDI, socio-demographic index; UI, uncertainty interval. (DOCX) [file pntd.0013017.s007.docx]

**S7 Table The crude prevalence and DALY rates (per 100,000) of lymphatic filariasis, by age group , sex, SDI levels, GBD regions, among 67 countries and territories, in 2021.**

| **Location** | **Sex** | **Metric** | **Age group (95% UI)** | | | | | | | | | | | | | | | | | | | |
| --- | --- | --- | --- | --- | --- | --- | --- | --- | --- | --- | --- | --- | --- | --- | --- | --- | --- | --- | --- | --- | --- | --- |
|  |  |  | **<5 years** | **5-9 years** | **10-14 years** | **15-19 years** | **20-24 years** | **25-29 years** | **30-34 years** | **35-39 years** | **40-44 years** | **45-49 years** | **50-54 years** | **55-59 years** | **60-64 years** | **65-69 years** | **70-74 years** | **75-79 years** | **80-84 years** | **85-89 years** | **90-94 years** | **95+ years** |
| Global | Both | DALY rates | 5.23 (3.58 to 7.38) | 15.45 (8.88 to 27.90) | 17.73 (10.04 to 31.67) | 20.14 (11.33 to 35.64) | 21.49 (12.28 to 37.24) | 21.10 (12.03 to 36.09) | 19.44 (11.14 to 33.18) | 19.43 (11.24 to 32.78) | 19.38 (11.34 to 32.38) | 17.85 (10.41 to 29.89) | 16.03 (9.45 to 26.58) | 14.80 (8.72 to 24.26) | 15.19 (9.10 to 24.56) | 13.63 (8.30 to 21.96) | 12.50 (7.60 to 20.04) | 11.70 (7.27 to 18.77) | 9.20 (5.74 to 14.58) | 7.01 (4.38 to 11.11) | 5.12 (3.25 to 8.09) | 3.24 (2.08 to 5.04) |
| Global | Both | Prevalence | 173.48 (152.85 to 202.73) | 428.33 (360.31 to 524.83) | 594.32 (503.51 to 712.64) | 787.61 (671.62 to 939.57) | 918.11 (786.19 to 1088.59) | 938.03 (803.31 to 1110.65) | 882.84 (754.10 to 1055.51) | 903.87 (773.28 to 1081.45) | 913.61 (780.21 to 1093.74) | 852.37 (725.76 to 1027.53) | 782.38 (663.59 to 945.10) | 741.67 (629.51 to 906.18) | 783.83 (666.47 to 966.55) | 721.61 (617.68 to 881.22) | 680.60 (587.22 to 816.57) | 656.51 (567.07 to 784.29) | 543.19 (469.60 to 652.18) | 431.71 (369.78 to 523.66) | 330.75 (279.76 to 410.80) | 242.37 (201.48 to 314.28) |
| Global | Female | DALY rates | 6.17 (4.18 to 8.72) | 10.01 (6.82 to 14.30) | 10.36 (7.10 to 14.97) | 10.73 (7.29 to 15.17) | 10.56 (7.14 to 14.96) | 9.88 (6.87 to 14.00) | 8.77 (5.94 to 12.43) | 8.47 (5.89 to 11.93) | 8.21 (5.65 to 11.63) | 7.34 (5.05 to 10.29) | 6.60 (4.60 to 9.20) | 6.07 (4.23 to 8.55) | 6.12 (4.22 to 8.66) | 5.36 (3.67 to 7.43) | 4.83 (3.34 to 6.59) | 4.56 (3.17 to 6.25) | 3.75 (2.64 to 5.19) | 2.93 (2.07 to 3.94) | 2.13 (1.51 to 2.91) | 1.57 (1.13 to 2.11) |
| Global | Female | Prevalence | 182.46 (161.55 to 211.28) | 386.67 (338.92 to 456.54) | 536.53 (468.96 to 632.92) | 712.95 (621.37 to 842.77) | 831.46 (723.08 to 983.37) | 850.07 (737.77 to 1001.19) | 797.19 (690.48 to 944.62) | 814.31 (704.26 to 974.15) | 817.44 (704.35 to 994.44) | 755.99 (650.27 to 929.54) | 697.42 (597.99 to 866.77) | 664.21 (567.40 to 834.31) | 704.15 (601.74 to 887.89) | 643.22 (550.03 to 806.14) | 600.39 (516.60 to 745.74) | 573.77 (493.85 to 707.90) | 474.88 (409.64 to 589.94) | 368.27 (316.66 to 462.58) | 270.41 (228.73 to 350.89) | 209.72 (174.95 to 279.44) |
| Global | Male | DALY rates | 4.35 (2.92 to 6.20) | 20.56 (9.88 to 41.76) | 24.66 (11.90 to 49.24) | 29.05 (14.43 to 57.39) | 32.08 (16.28 to 60.26) | 32.09 (16.67 to 59.42) | 29.88 (15.53 to 54.58) | 30.19 (15.86 to 54.55) | 30.38 (16.10 to 54.77) | 28.25 (15.33 to 50.92) | 25.51 (13.89 to 45.48) | 23.80 (12.97 to 42.26) | 24.79 (13.64 to 43.25) | 22.66 (12.64 to 38.99) | 21.20 (11.85 to 36.15) | 20.32 (11.49 to 34.75) | 16.78 (9.45 to 28.64) | 13.74 (7.82 to 23.75) | 11.31 (6.41 to 19.54) | 7.59 (4.25 to 13.17) |
| Global | Male | Prevalence | 165.08 (144.00 to 192.04) | 467.42 (373.26 to 636.65) | 648.62 (527.47 to 850.01) | 858.39 (712.71 to 1081.33) | 1002.01 (836.76 to 1235.08) | 1024.11 (856.72 to 1248.09) | 966.64 (810.41 to 1183.33) | 991.77 (829.71 to 1212.19) | 1008.23 (841.86 to 1238.10) | 947.85 (792.18 to 1167.12) | 867.71 (724.20 to 1069.17) | 821.64 (685.16 to 1018.52) | 868.10 (726.65 to 1077.41) | 807.25 (677.34 to 992.27) | 771.68 (653.88 to 937.11) | 756.28 (646.09 to 911.63) | 638.11 (543.79 to 772.01) | 536.39 (455.00 to 648.50) | 455.63 (384.53 to 556.93) | 327.41 (267.71 to 426.67) |
| **SDI region** |  |  |  |  |  |  |  |  |  |  |  |  |  |  |  |  |  |  |  |  |  |  |
| Low SDI | Both | DALY rates | 8.28 (5.69 to 11.88) | 26.28 (14.99 to 47.33) | 29.42 (16.65 to 52.44) | 33.08 (18.88 to 58.10) | 36.58 (21.00 to 62.56) | 39.56 (22.91 to 67.07) | 42.44 (25.19 to 71.16) | 45.05 (26.51 to 74.04) | 47.26 (28.21 to 76.63) | 48.79 (29.54 to 79.37) | 49.88 (29.28 to 81.39) | 51.42 (31.01 to 83.37) | 52.73 (31.95 to 84.71) | 52.18 (31.66 to 84.23) | 50.75 (30.71 to 81.24) | 48.53 (29.81 to 78.73) | 45.53 (28.31 to 72.01) | 43.09 (26.78 to 68.23) | 41.12 (26.26 to 63.68) | 35.94 (23.73 to 54.68) |
| Low SDI | Both | Prevalence | 267.96 (218.03 to 355.71) | 713.42 (573.54 to 931.83) | 969.64 (784.37 to 1253.48) | 1265.02 (1026.35 to 1623.91) | 1542.01 (1253.97 to 1973.20) | 1752.71 (1424.08 to 2237.83) | 1941.03 (1577.91 to 2485.48) | 2110.57 (1724.66 to 2673.30) | 2228.83 (1824.72 to 2806.48) | 2328.97 (1910.03 to 2920.71) | 2438.20 (1998.98 to 3067.48) | 2585.63 (2127.82 to 3240.73) | 2733.01 (2261.86 to 3372.26) | 2787.78 (2327.55 to 3418.19) | 2804.43 (2348.03 to 3390.70) | 2739.34 (2292.74 to 3324.51) | 2676.75 (2242.31 to 3215.32) | 2617.20 (2178.25 to 3193.81) | 2519.92 (2087.10 to 3108.92) | 2384.76 (1961.69 to 2973.08) |
| Low SDI | Female | DALY rates | 9.75 (6.65 to 14.15) | 16.84 (11.23 to 23.92) | 17.24 (11.51 to 24.75) | 17.68 (12.12 to 25.51) | 17.87 (12.21 to 25.17) | 18.03 (12.36 to 25.62) | 18.22 (12.14 to 25.67) | 18.43 (12.73 to 25.86) | 18.53 (12.82 to 26.15) | 18.49 (12.67 to 25.78) | 18.65 (12.65 to 26.02) | 19.02 (12.96 to 26.70) | 19.21 (13.23 to 26.85) | 18.96 (13.13 to 26.97) | 18.76 (12.71 to 25.72) | 18.23 (12.69 to 25.03) | 17.97 (12.44 to 24.67) | 17.68 (12.41 to 24.02) | 17.29 (12.16 to 23.23) | 16.85 (11.89 to 22.60) |
| Low SDI | Female | Prevalence | 281.94 (231.60 to 370.44) | 640.04 (519.71 to 849.08) | 872.14 (703.40 to 1149.29) | 1137.76 (916.78 to 1483.23) | 1381.26 (1113.10 to 1804.38) | 1562.69 (1250.58 to 2048.44) | 1720.84 (1377.86 to 2251.12) | 1858.76 (1499.07 to 2420.14) | 1947.77 (1578.00 to 2519.17) | 2034.32 (1653.72 to 2617.58) | 2138.50 (1737.79 to 2741.30) | 2279.75 (1861.67 to 2900.11) | 2425.06 (2001.85 to 3022.54) | 2477.85 (2070.51 to 3039.93) | 2507.88 (2099.76 to 3037.62) | 2445.11 (2034.97 to 2970.78) | 2418.71 (2007.22 to 2949.83) | 2395.64 (1980.41 to 2972.66) | 2305.98 (1878.55 to 2922.54) | 2220.52 (1797.56 to 2835.60) |
| Low SDI | Male | DALY rates | 6.87 (4.62 to 9.94) | 35.38 (17.12 to 72.46) | 41.25 (19.65 to 82.24) | 48.32 (24.02 to 94.14) | 55.72 (28.24 to 103.91) | 62.10 (32.35 to 111.70) | 67.91 (36.25 to 123.07) | 72.96 (39.22 to 128.33) | 76.68 (42.01 to 132.54) | 78.86 (43.53 to 135.92) | 80.79 (44.56 to 140.30) | 84.17 (47.16 to 145.22) | 87.04 (48.92 to 148.54) | 86.69 (48.57 to 147.45) | 84.31 (47.51 to 142.74) | 81.07 (46.06 to 137.81) | 77.67 (44.40 to 131.30) | 75.09 (42.24 to 127.68) | 72.58 (41.78 to 121.70) | 69.53 (40.46 to 114.55) |
| Low SDI | Male | Prevalence | 254.58 (207.06 to 340.58) | 784.17 (599.38 to 1075.62) | 1064.29 (833.07 to 1418.50) | 1390.92 (1112.15 to 1817.58) | 1706.45 (1381.84 to 2209.13) | 1951.59 (1585.21 to 2508.67) | 2172.70 (1767.83 to 2794.31) | 2374.52 (1938.70 to 3034.25) | 2516.73 (2060.17 to 3215.96) | 2621.34 (2145.78 to 3331.26) | 2734.85 (2231.90 to 3470.10) | 2894.88 (2371.88 to 3649.11) | 3048.36 (2519.21 to 3819.70) | 3109.81 (2584.31 to 3836.30) | 3115.39 (2586.49 to 3777.74) | 3055.37 (2533.36 to 3671.74) | 2977.68 (2470.59 to 3603.68) | 2896.29 (2404.18 to 3522.54) | 2802.31 (2303.39 to 3434.87) | 2673.65 (2186.83 to 3300.11) |
| Low-middle SDI | Both | DALY rates | 6.83 (4.60 to 9.61) | 20.83 (11.80 to 36.75) | 25.17 (14.12 to 44.25) | 29.15 (16.36 to 51.63) | 32.34 (18.61 to 55.36) | 33.68 (19.43 to 57.36) | 34.47 (19.79 to 58.61) | 35.36 (20.62 to 59.74) | 36.51 (21.47 to 60.63) | 37.64 (21.99 to 63.06) | 37.47 (22.18 to 61.69) | 37.06 (21.86 to 60.43) | 38.46 (23.33 to 61.32) | 40.43 (24.96 to 63.89) | 41.14 (25.30 to 65.45) | 39.27 (24.08 to 61.79) | 35.92 (22.48 to 56.24) | 32.32 (20.29 to 50.44) | 29.84 (18.87 to 46.70) | 19.86 (12.71 to 30.74) |
| Low-middle SDI | Both | Prevalence | 241.86 (208.72 to 291.04) | 617.70 (516.57 to 766.56) | 885.00 (741.69 to 1090.09) | 1191.81 (998.84 to 1457.97) | 1429.20 (1202.16 to 1742.16) | 1536.08 (1291.17 to 1868.84) | 1597.50 (1341.04 to 1939.90) | 1677.86 (1407.58 to 2049.72) | 1756.46 (1469.76 to 2164.13) | 1827.53 (1529.22 to 2260.51) | 1855.51 (1556.28 to 2312.44) | 1889.86 (1584.45 to 2370.61) | 2018.15 (1693.45 to 2524.98) | 2164.62 (1825.83 to 2668.61) | 2248.32 (1910.35 to 2723.17) | 2209.74 (1887.03 to 2651.90) | 2131.88 (1829.98 to 2544.29) | 1993.69 (1706.75 to 2403.47) | 1895.08 (1609.83 to 2297.29) | 1501.95 (1265.75 to 1843.30) |
| Low-middle SDI | Female | DALY rates | 8.01 (5.42 to 11.47) | 13.65 (9.31 to 19.52) | 14.34 (9.70 to 20.57) | 14.99 (9.98 to 21.12) | 15.16 (10.10 to 21.51) | 15.05 (10.46 to 21.32) | 14.93 (10.11 to 20.89) | 14.83 (10.22 to 20.93) | 14.89 (10.08 to 21.08) | 14.94 (10.20 to 21.02) | 14.89 (10.35 to 20.83) | 14.78 (10.12 to 20.78) | 15.04 (10.35 to 21.18) | 15.29 (10.60 to 21.12) | 15.30 (10.60 to 21.07) | 14.81 (10.00 to 20.40) | 14.42 (10.09 to 19.96) | 13.70 (9.56 to 18.58) | 12.79 (9.10 to 17.34) | 10.28 (7.29 to 13.89) |
| Low-middle SDI | Female | Prevalence | 251.41 (218.99 to 300.98) | 559.23 (481.48 to 677.59) | 793.95 (678.99 to 957.21) | 1065.93 (906.77 to 1289.42) | 1272.19 (1079.75 to 1540.85) | 1364.12 (1155.37 to 1661.28) | 1417.69 (1195.81 to 1742.26) | 1496.18 (1260.35 to 1843.02) | 1561.56 (1307.48 to 1928.07) | 1611.40 (1342.17 to 2023.07) | 1652.45 (1369.32 to 2112.47) | 1708.65 (1413.26 to 2217.52) | 1839.56 (1517.71 to 2356.91) | 1964.36 (1634.95 to 2463.26) | 2028.21 (1711.14 to 2479.49) | 1988.26 (1693.18 to 2398.90) | 1954.08 (1675.10 to 2357.27) | 1841.72 (1578.30 to 2273.89) | 1732.85 (1471.92 to 2177.28) | 1469.50 (1237.51 to 1835.81) |
| Low-middle SDI | Male | DALY rates | 5.73 (3.79 to 8.17) | 27.60 (13.60 to 55.55) | 35.40 (17.06 to 68.44) | 42.74 (21.41 to 83.79) | 49.36 (25.15 to 93.61) | 52.46 (27.22 to 96.33) | 54.01 (28.22 to 97.36) | 55.82 (29.45 to 101.18) | 58.17 (30.87 to 103.46) | 60.51 (33.13 to 108.23) | 60.41 (32.63 to 106.07) | 60.09 (32.80 to 104.18) | 63.27 (35.17 to 107.95) | 67.52 (38.66 to 113.78) | 69.58 (39.76 to 116.18) | 67.46 (38.48 to 113.09) | 63.26 (35.57 to 106.23) | 57.41 (32.82 to 97.31) | 53.91 (30.72 to 91.30) | 36.88 (20.75 to 63.95) |
| Low-middle SDI | Male | Prevalence | 232.87 (200.86 to 281.30) | 672.83 (533.52 to 894.59) | 970.97 (781.50 to 1266.45) | 1312.70 (1071.45 to 1660.89) | 1584.81 (1306.15 to 1979.91) | 1709.37 (1416.94 to 2128.44) | 1777.28 (1478.87 to 2236.72) | 1858.83 (1543.77 to 2323.12) | 1951.69 (1618.65 to 2422.85) | 2045.34 (1698.28 to 2537.98) | 2061.71 (1708.44 to 2563.32) | 2077.18 (1718.12 to 2591.43) | 2207.30 (1828.39 to 2738.47) | 2380.48 (1992.07 to 2933.33) | 2490.56 (2099.90 to 3043.61) | 2465.01 (2088.26 to 2982.55) | 2358.05 (1999.29 to 2855.00) | 2198.51 (1845.19 to 2691.46) | 2124.12 (1763.63 to 2597.26) | 1559.61 (1278.90 to 2014.41) |
| Middle SDI | Both | DALY rates | 3.97 (2.68 to 5.66) | 11.80 (6.74 to 21.67) | 13.35 (7.54 to 24.22) | 15.60 (8.89 to 27.92) | 17.52 (9.94 to 30.70) | 17.85 (10.13 to 30.80) | 16.60 (9.51 to 28.82) | 16.88 (9.55 to 29.20) | 17.10 (9.71 to 29.28) | 15.43 (8.96 to 26.25) | 13.93 (8.20 to 23.57) | 13.29 (7.73 to 22.51) | 14.85 (8.82 to 24.87) | 12.30 (7.39 to 20.49) | 11.38 (6.79 to 19.04) | 10.54 (6.49 to 17.29) | 9.43 (5.86 to 15.20) | 8.30 (5.17 to 13.50) | 6.92 (4.40 to 10.96) | 5.28 (3.40 to 8.17) |
| Middle SDI | Both | Prevalence | 123.70 (102.40 to 171.12) | 304.72 (239.62 to 431.73) | 425.71 (335.58 to 596.34) | 587.06 (465.87 to 818.99) | 724.13 (580.91 to 1003.32) | 772.16 (619.67 to 1089.15) | 733.35 (585.71 to 1037.34) | 765.16 (606.61 to 1085.46) | 788.52 (624.83 to 1128.52) | 725.84 (572.42 to 1034.46) | 670.44 (525.62 to 964.53) | 654.73 (510.74 to 955.38) | 751.12 (584.31 to 1110.28) | 640.93 (499.28 to 949.58) | 614.54 (483.78 to 894.05) | 590.52 (466.08 to 852.64) | 557.32 (440.67 to 815.52) | 519.28 (403.80 to 774.23) | 479.02 (358.94 to 787.28) | 420.30 (292.21 to 741.24) |
| Middle SDI | Female | DALY rates | 4.68 (3.17 to 6.69) | 7.49 (5.04 to 10.55) | 7.92 (5.35 to 11.29) | 8.51 (5.70 to 12.04) | 8.87 (6.06 to 12.51) | 8.67 (5.89 to 12.41) | 7.82 (5.34 to 11.25) | 7.77 (5.23 to 11.04) | 7.77 (5.28 to 10.90) | 6.88 (4.65 to 9.74) | 6.26 (4.31 to 8.75) | 5.96 (4.13 to 8.40) | 6.59 (4.49 to 9.37) | 5.47 (3.74 to 7.71) | 5.04 (3.47 to 6.92) | 4.74 (3.23 to 6.58) | 4.51 (3.24 to 6.29) | 4.11 (2.84 to 5.58) | 3.75 (2.63 to 5.11) | 3.24 (2.32 to 4.38) |
| Middle SDI | Female | Prevalence | 130.74 (109.01 to 180.19) | 271.44 (222.01 to 394.21) | 383.34 (311.54 to 547.79) | 531.52 (431.03 to 764.44) | 654.04 (532.02 to 942.97) | 697.46 (566.06 to 1006.75) | 657.94 (531.75 to 962.41) | 685.22 (550.35 to 1015.57) | 704.69 (558.24 to 1050.18) | 647.48 (507.29 to 966.90) | 601.54 (465.82 to 902.66) | 586.23 (449.40 to 897.75) | 675.42 (515.06 to 1044.01) | 577.18 (440.10 to 895.32) | 552.17 (423.21 to 848.41) | 527.37 (401.66 to 820.45) | 504.06 (383.32 to 779.90) | 461.49 (347.71 to 727.98) | 433.27 (314.62 to 722.90) | 401.70 (271.52 to 744.04) |
| Middle SDI | Male | DALY rates | 3.31 (2.19 to 4.87) | 15.78 (7.55 to 31.84) | 18.36 (9.01 to 36.97) | 22.19 (10.87 to 43.70) | 25.80 (12.97 to 49.74) | 26.78 (13.57 to 50.36) | 25.21 (12.67 to 47.08) | 25.87 (13.17 to 48.27) | 26.30 (13.60 to 49.08) | 23.93 (12.51 to 44.30) | 21.70 (11.44 to 39.96) | 20.94 (11.20 to 38.25) | 23.53 (12.74 to 42.70) | 19.64 (10.73 to 35.17) | 18.37 (9.88 to 33.08) | 17.36 (9.54 to 31.13) | 15.95 (8.84 to 27.99) | 15.00 (8.21 to 26.74) | 13.08 (7.11 to 23.46) | 9.60 (5.22 to 17.33) |
| Middle SDI | Male | Prevalence | 117.24 (95.62 to 162.86) | 335.44 (249.44 to 486.62) | 464.78 (356.15 to 653.90) | 638.68 (496.15 to 884.09) | 791.21 (618.28 to 1088.16) | 844.78 (658.53 to 1159.45) | 807.29 (629.27 to 1118.68) | 843.95 (656.71 to 1181.39) | 871.13 (674.23 to 1222.89) | 803.80 (620.61 to 1134.58) | 740.29 (569.82 to 1060.39) | 726.16 (558.19 to 1051.82) | 830.71 (638.62 to 1202.10) | 709.38 (547.02 to 1026.53) | 683.42 (531.37 to 964.60) | 664.73 (522.81 to 917.91) | 627.81 (496.12 to 861.92) | 611.68 (481.66 to 859.48) | 567.97 (430.91 to 870.86) | 459.72 (315.97 to 824.54) |
| High-middle SDI | Both | DALY rates | 0.84 (0.56 to 1.20) | 2.28 (1.27 to 4.10) | 2.66 (1.47 to 4.73) | 3.12 (1.74 to 5.52) | 3.30 (1.83 to 6.04) | 3.06 (1.66 to 5.63) | 2.41 (1.34 to 4.33) | 2.39 (1.32 to 4.25) | 2.34 (1.32 to 4.14) | 1.93 (1.07 to 3.51) | 1.68 (0.97 to 2.95) | 1.52 (0.85 to 2.67) | 1.46 (0.82 to 2.58) | 1.18 (0.67 to 2.09) | 0.98 (0.57 to 1.72) | 0.96 (0.55 to 1.66) | 0.64 (0.37 to 1.09) | 0.52 (0.29 to 0.89) | 0.42 (0.24 to 0.72) | 0.37 (0.20 to 0.65) |
| High-middle SDI | Both | Prevalence | 21.08 (12.16 to 47.75) | 48.42 (27.84 to 103.33) | 69.68 (37.85 to 158.39) | 97.46 (50.40 to 231.05) | 114.82 (57.46 to 270.70) | 113.53 (55.61 to 265.10) | 93.64 (45.18 to 220.20) | 95.87 (45.90 to 232.00) | 96.07 (45.67 to 244.00) | 81.54 (38.61 to 210.68) | 72.45 (34.07 to 186.16) | 66.98 (31.32 to 168.55) | 66.53 (31.28 to 154.81) | 54.95 (26.19 to 128.79) | 46.99 (22.50 to 112.91) | 46.96 (22.19 to 114.87) | 32.44 (15.43 to 79.00) | 27.05 (12.71 to 69.41) | 21.44 (9.50 to 58.63) | 17.74 (7.50 to 52.32) |
| High-middle SDI | Female | DALY rates | 0.99 (0.64 to 1.43) | 1.49 (0.97 to 2.21) | 1.67 (1.10 to 2.42) | 1.88 (1.23 to 2.74) | 1.92 (1.24 to 2.86) | 1.73 (1.13 to 2.53) | 1.33 (0.89 to 1.91) | 1.28 (0.83 to 1.81) | 1.23 (0.80 to 1.76) | 0.99 (0.67 to 1.40) | 0.87 (0.58 to 1.25) | 0.77 (0.52 to 1.16) | 0.74 (0.49 to 1.06) | 0.59 (0.39 to 0.83) | 0.49 (0.32 to 0.69) | 0.48 (0.33 to 0.68) | 0.32 (0.21 to 0.44) | 0.24 (0.16 to 0.34) | 0.16 (0.11 to 0.23) | 0.10 (0.07 to 0.14) |
| High-middle SDI | Female | Prevalence | 22.59 (13.56 to 49.45) | 42.16 (23.35 to 97.48) | 62.09 (30.56 to 153.45) | 88.39 (40.43 to 228.01) | 105.11 (46.10 to 270.13) | 104.09 (44.81 to 265.31) | 84.95 (36.05 to 219.18) | 86.29 (36.06 to 227.44) | 85.88 (34.80 to 232.12) | 72.33 (29.09 to 198.76) | 64.51 (25.76 to 175.26) | 58.93 (23.57 to 156.42) | 57.82 (23.95 to 137.89) | 46.75 (19.51 to 113.63) | 39.45 (16.91 to 99.36) | 39.09 (16.71 to 100.49) | 26.23 (11.42 to 66.07) | 20.52 (8.91 to 53.06) | 13.94 (5.90 to 38.45) | 8.53 (3.67 to 23.57) |
| High-middle SDI | Male | DALY rates | 0.70 (0.43 to 1.07) | 3.00 (1.40 to 6.22) | 3.56 (1.71 to 7.24) | 4.24 (2.04 to 8.61) | 4.54 (2.08 to 9.30) | 4.26 (1.96 to 8.61) | 3.43 (1.59 to 6.81) | 3.45 (1.66 to 6.90) | 3.41 (1.64 to 6.82) | 2.87 (1.39 to 5.72) | 2.49 (1.20 to 4.86) | 2.29 (1.09 to 4.43) | 2.25 (1.09 to 4.37) | 1.86 (0.92 to 3.68) | 1.58 (0.79 to 3.06) | 1.58 (0.78 to 3.04) | 1.15 (0.57 to 2.18) | 1.05 (0.51 to 2.02) | 1.04 (0.50 to 1.97) | 1.16 (0.57 to 2.26) |
| High-middle SDI | Male | Prevalence | 19.71 (10.75 to 46.20) | 54.11 (29.98 to 106.56) | 76.58 (40.92 to 164.88) | 105.67 (55.04 to 235.84) | 123.59 (62.89 to 273.63) | 122.10 (61.70 to 267.39) | 101.74 (51.10 to 225.50) | 105.02 (52.18 to 238.50) | 105.96 (52.32 to 252.48) | 90.67 (44.76 to 221.13) | 80.39 (39.19 to 196.62) | 75.35 (36.62 to 180.55) | 76.00 (37.67 to 169.68) | 64.35 (31.95 to 145.43) | 56.22 (28.03 to 129.15) | 57.22 (28.35 to 134.82) | 42.34 (20.89 to 100.02) | 39.43 (18.79 to 100.42) | 39.66 (17.13 to 110.56) | 45.29 (17.75 to 139.87) |
| High SDI | Both | DALY rates | 0.00 (0.00 to 0.00) | 0.00 (0.00 to 0.00) | 0.00 (0.00 to 0.00) | 0.00 (0.00 to 0.00) | 0.00 (0.00 to 0.00) | 0.00 (0.00 to 0.00) | 0.00 (0.00 to 0.00) | 0.00 (0.00 to 0.00) | 0.00 (0.00 to 0.00) | 0.00 (0.00 to 0.00) | 0.00 (0.00 to 0.00) | 0.00 (0.00 to 0.00) | 0.00 (0.00 to 0.00) | 0.00 (0.00 to 0.00) | 0.00 (0.00 to 0.00) | 0.00 (0.00 to 0.00) | 0.00 (0.00 to 0.00) | 0.00 (0.00 to 0.00) | 0.00 (0.00 to 0.00) | 0.00 (0.00 to 0.00) |
| High SDI | Both | Prevalence | 0.00 (0.00 to 0.00) | 0.00 (0.00 to 0.00) | 0.00 (0.00 to 0.00) | 0.00 (0.00 to 0.00) | 0.00 (0.00 to 0.00) | 0.00 (0.00 to 0.00) | 0.00 (0.00 to 0.00) | 0.00 (0.00 to 0.00) | 0.00 (0.00 to 0.00) | 0.00 (0.00 to 0.00) | 0.00 (0.00 to 0.00) | 0.00 (0.00 to 0.00) | 0.00 (0.00 to 0.00) | 0.00 (0.00 to 0.00) | 0.00 (0.00 to 0.00) | 0.00 (0.00 to 0.00) | 0.00 (0.00 to 0.00) | 0.00 (0.00 to 0.00) | 0.00 (0.00 to 0.00) | 0.00 (0.00 to 0.00) |
| High SDI | Female | DALY rates | 0.00 (0.00 to 0.00) | 0.00 (0.00 to 0.00) | 0.00 (0.00 to 0.00) | 0.00 (0.00 to 0.00) | 0.00 (0.00 to 0.00) | 0.00 (0.00 to 0.00) | 0.00 (0.00 to 0.00) | 0.00 (0.00 to 0.00) | 0.00 (0.00 to 0.00) | 0.00 (0.00 to 0.00) | 0.00 (0.00 to 0.00) | 0.00 (0.00 to 0.00) | 0.00 (0.00 to 0.00) | 0.00 (0.00 to 0.00) | 0.00 (0.00 to 0.00) | 0.00 (0.00 to 0.00) | 0.00 (0.00 to 0.00) | 0.00 (0.00 to 0.00) | 0.00 (0.00 to 0.00) | 0.00 (0.00 to 0.00) |
| High SDI | Female | Prevalence | 0.00 (0.00 to 0.00) | 0.00 (0.00 to 0.00) | 0.00 (0.00 to 0.00) | 0.00 (0.00 to 0.00) | 0.00 (0.00 to 0.00) | 0.00 (0.00 to 0.00) | 0.00 (0.00 to 0.00) | 0.00 (0.00 to 0.00) | 0.00 (0.00 to 0.00) | 0.00 (0.00 to 0.00) | 0.00 (0.00 to 0.00) | 0.00 (0.00 to 0.00) | 0.00 (0.00 to 0.00) | 0.00 (0.00 to 0.00) | 0.00 (0.00 to 0.00) | 0.00 (0.00 to 0.00) | 0.00 (0.00 to 0.00) | 0.00 (0.00 to 0.00) | 0.00 (0.00 to 0.00) | 0.00 (0.00 to 0.00) |
| High SDI | Male | DALY rates | 0.00 (0.00 to 0.00) | 0.00 (0.00 to 0.00) | 0.00 (0.00 to 0.00) | 0.00 (0.00 to 0.00) | 0.00 (0.00 to 0.00) | 0.00 (0.00 to 0.00) | 0.00 (0.00 to 0.00) | 0.00 (0.00 to 0.00) | 0.00 (0.00 to 0.00) | 0.00 (0.00 to 0.00) | 0.00 (0.00 to 0.00) | 0.00 (0.00 to 0.00) | 0.00 (0.00 to 0.00) | 0.00 (0.00 to 0.00) | 0.00 (0.00 to 0.00) | 0.00 (0.00 to 0.00) | 0.00 (0.00 to 0.00) | 0.00 (0.00 to 0.00) | 0.00 (0.00 to 0.00) | 0.00 (0.00 to 0.00) |
| High SDI | Male | Prevalence | 0.00 (0.00 to 0.00) | 0.00 (0.00 to 0.00) | 0.00 (0.00 to 0.00) | 0.00 (0.00 to 0.00) | 0.00 (0.00 to 0.00) | 0.00 (0.00 to 0.00) | 0.00 (0.00 to 0.00) | 0.00 (0.00 to 0.00) | 0.00 (0.00 to 0.00) | 0.00 (0.00 to 0.00) | 0.00 (0.00 to 0.00) | 0.00 (0.00 to 0.00) | 0.00 (0.00 to 0.00) | 0.00 (0.00 to 0.00) | 0.00 (0.00 to 0.00) | 0.00 (0.00 to 0.00) | 0.00 (0.00 to 0.00) | 0.00 (0.00 to 0.00) | 0.00 (0.00 to 0.00) | 0.00 (0.00 to 0.00) |
| **GBD region** |  |  |  |  |  |  |  |  |  |  |  |  |  |  |  |  |  |  |  |  |  |  |
| Caribbean | Both | DALY rates | 6.99 (4.39 to 10.13) | 21.08 (11.79 to 37.04) | 22.34 (12.87 to 38.35) | 24.93 (14.72 to 42.48) | 26.96 (16.45 to 44.02) | 27.36 (16.68 to 45.15) | 25.23 (15.24 to 41.19) | 25.08 (15.22 to 41.43) | 25.75 (15.76 to 41.03) | 22.43 (14.07 to 36.16) | 20.16 (12.52 to 31.74) | 18.34 (11.60 to 29.11) | 19.08 (12.05 to 30.19) | 17.94 (11.46 to 28.29) | 15.05 (9.54 to 23.77) | 13.18 (8.20 to 21.14) | 11.76 (7.36 to 18.26) | 10.67 (6.80 to 16.93) | 9.04 (5.68 to 14.41) | 4.66 (3.02 to 7.15) |
| Caribbean | Both | Prevalence | 212.94 (131.45 to 373.39) | 527.90 (320.95 to 914.23) | 675.09 (396.19 to 1208.16) | 860.95 (485.74 to 1602.78) | 1020.82 (563.80 to 1910.30) | 1114.81 (608.10 to 2120.85) | 1067.77 (570.99 to 2085.28) | 1103.94 (586.87 to 2172.08) | 1179.19 (631.42 to 2314.91) | 1060.35 (561.94 to 2110.17) | 981.57 (512.95 to 1974.99) | 930.98 (481.89 to 1875.61) | 1004.75 (516.69 to 2029.93) | 975.70 (497.77 to 2003.74) | 831.93 (417.07 to 1815.26) | 746.84 (364.14 to 1735.07) | 690.87 (328.68 to 1691.45) | 649.19 (291.85 to 1689.38) | 558.37 (235.27 to 1545.27) | 288.61 (121.89 to 797.21) |
| Caribbean | Female | DALY rates | 8.22 (5.18 to 12.18) | 13.02 (7.95 to 19.66) | 12.62 (8.14 to 18.86) | 12.79 (8.45 to 18.90) | 12.62 (8.02 to 18.18) | 12.45 (7.87 to 18.53) | 11.88 (7.73 to 17.43) | 11.91 (7.71 to 17.58) | 11.71 (7.46 to 16.87) | 9.90 (5.94 to 14.19) | 8.68 (5.75 to 12.32) | 7.91 (5.25 to 11.51) | 8.19 (5.37 to 11.63) | 7.82 (5.15 to 11.41) | 6.70 (4.28 to 9.69) | 5.92 (3.91 to 8.55) | 5.46 (3.58 to 7.77) | 4.95 (3.30 to 7.28) | 4.11 (2.68 to 5.83) | 1.89 (1.23 to 2.71) |
| Caribbean | Female | Prevalence | 225.21 (142.86 to 387.02) | 468.23 (276.17 to 845.59) | 601.92 (334.22 to 1131.25) | 769.85 (403.93 to 1490.76) | 914.01 (461.58 to 1799.73) | 1007.34 (500.67 to 2002.05) | 972.33 (484.60 to 1973.93) | 1006.53 (500.75 to 2043.42) | 1070.50 (521.25 to 2167.34) | 958.32 (458.49 to 1974.90) | 883.03 (417.23 to 1853.27) | 842.26 (397.78 to 1764.78) | 908.72 (425.74 to 1906.92) | 887.91 (410.91 to 1891.38) | 755.23 (334.92 to 1724.60) | 676.00 (291.38 to 1641.85) | 631.69 (263.18 to 1603.60) | 582.21 (230.95 to 1570.96) | 482.86 (174.87 to 1400.32) | 225.85 (77.98 to 680.24) |
| Caribbean | Male | DALY rates | 5.81 (3.43 to 9.12) | 28.85 (13.97 to 57.05) | 31.76 (16.39 to 61.68) | 36.78 (19.66 to 68.30) | 41.13 (23.24 to 73.47) | 42.33 (23.57 to 75.56) | 38.78 (21.71 to 67.90) | 38.66 (21.56 to 68.11) | 40.29 (22.73 to 69.35) | 35.39 (20.77 to 61.48) | 32.10 (18.77 to 54.43) | 29.40 (17.27 to 50.15) | 30.90 (18.34 to 51.27) | 29.19 (17.29 to 47.97) | 24.52 (14.22 to 41.57) | 21.86 (12.72 to 38.02) | 19.78 (11.54 to 34.04) | 18.58 (10.73 to 31.83) | 16.53 (9.36 to 28.88) | 9.69 (5.86 to 16.16) |
| Caribbean | Male | Prevalence | 201.17 (119.22 to 360.51) | 585.47 (353.10 to 994.18) | 746.06 (446.19 to 1317.40) | 949.94 (553.12 to 1724.86) | 1126.37 (652.52 to 2030.73) | 1222.59 (699.27 to 2251.40) | 1164.56 (651.55 to 2223.01) | 1204.40 (667.62 to 2333.36) | 1291.71 (713.19 to 2495.09) | 1165.80 (647.23 to 2269.33) | 1084.01 (595.52 to 2119.87) | 1025.04 (558.18 to 2010.63) | 1109.02 (601.89 to 2182.38) | 1073.31 (579.47 to 2151.01) | 918.95 (488.97 to 1935.48) | 831.51 (435.26 to 1845.37) | 766.25 (390.90 to 1773.18) | 741.97 (361.11 to 1814.72) | 673.20 (313.03 to 1741.19) | 402.86 (192.88 to 996.96) |
| Central Sub-Saharan Africa | Both | DALY rates | 9.86 (6.38 to 14.61) | 31.31 (17.12 to 57.56) | 33.76 (18.72 to 61.65) | 37.36 (20.76 to 66.44) | 40.64 (21.79 to 70.81) | 43.28 (23.88 to 76.29) | 44.81 (24.46 to 79.53) | 46.37 (25.83 to 79.54) | 47.77 (26.22 to 84.63) | 49.20 (27.11 to 86.02) | 50.12 (28.25 to 85.77) | 49.92 (28.66 to 86.53) | 49.84 (28.69 to 86.16) | 48.64 (27.79 to 81.34) | 45.30 (26.58 to 75.75) | 42.72 (25.14 to 70.69) | 39.86 (24.01 to 64.05) | 37.66 (22.50 to 61.36) | 35.13 (21.42 to 55.15) | 31.63 (19.42 to 48.52) |
| Central Sub-Saharan Africa | Both | Prevalence | 356.81 (209.30 to 658.68) | 926.35 (536.74 to 1655.07) | 1249.93 (696.34 to 2317.06) | 1634.76 (886.27 to 3099.15) | 1963.86 (1051.97 to 3734.64) | 2188.64 (1164.30 to 4165.20) | 2353.93 (1243.52 to 4497.54) | 2496.11 (1312.60 to 4777.65) | 2603.93 (1363.24 to 5019.06) | 2712.21 (1418.65 to 5231.00) | 2825.42 (1479.71 to 5436.31) | 2917.12 (1527.11 to 5612.77) | 3031.04 (1588.45 to 5834.15) | 3059.55 (1603.22 to 5868.13) | 3024.16 (1575.49 to 5814.95) | 2985.77 (1537.41 to 5760.03) | 2958.70 (1517.98 to 5700.43) | 2979.16 (1499.31 to 5839.34) | 2961.09 (1472.08 to 5903.13) | 2954.17 (1453.84 to 5952.46) |
| Central Sub-Saharan Africa | Female | DALY rates | 11.65 (7.30 to 17.66) | 19.55 (11.79 to 29.07) | 19.97 (12.53 to 30.15) | 20.56 (13.45 to 30.55) | 20.67 (13.50 to 30.82) | 21.04 (13.22 to 31.32) | 20.91 (12.90 to 30.46) | 21.11 (13.32 to 30.24) | 21.20 (13.61 to 31.12) | 21.40 (13.74 to 30.82) | 21.27 (13.60 to 31.11) | 21.13 (13.95 to 30.53) | 21.23 (13.87 to 29.74) | 21.21 (13.66 to 31.54) | 20.86 (13.45 to 31.14) | 20.51 (13.34 to 29.61) | 20.05 (13.16 to 29.06) | 19.75 (12.72 to 28.77) | 19.35 (12.71 to 27.27) | 18.60 (12.22 to 26.62) |
| Central Sub-Saharan Africa | Female | Prevalence | 373.52 (223.69 to 674.08) | 836.51 (469.04 to 1586.35) | 1140.35 (607.43 to 2237.58) | 1498.56 (767.51 to 2983.26) | 1802.96 (900.40 to 3579.55) | 2006.42 (991.74 to 4064.10) | 2153.87 (1056.46 to 4386.29) | 2279.18 (1110.44 to 4613.36) | 2373.23 (1152.30 to 4824.69) | 2466.92 (1193.85 to 5026.59) | 2573.20 (1240.68 to 5164.95) | 2663.41 (1281.80 to 5383.89) | 2783.41 (1330.59 to 5559.69) | 2827.47 (1347.34 to 5622.96) | 2824.62 (1342.72 to 5610.28) | 2813.09 (1326.31 to 5586.98) | 2811.17 (1334.76 to 5615.92) | 2842.51 (1332.62 to 5770.02) | 2833.19 (1319.08 to 5837.26) | 2839.13 (1322.49 to 5889.88) |
| Central Sub-Saharan Africa | Male | DALY rates | 8.13 (4.89 to 12.51) | 42.83 (19.58 to 91.61) | 47.33 (21.45 to 99.51) | 53.96 (25.01 to 108.55) | 60.57 (27.97 to 116.68) | 65.75 (31.74 to 126.31) | 69.15 (32.08 to 136.30) | 71.97 (34.69 to 134.84) | 74.62 (37.02 to 143.45) | 76.85 (37.03 to 144.17) | 79.04 (39.51 to 146.26) | 80.41 (40.57 to 149.35) | 82.02 (40.98 to 152.30) | 81.75 (41.91 to 147.89) | 79.32 (39.90 to 142.41) | 77.48 (38.92 to 140.46) | 75.00 (38.58 to 136.35) | 73.80 (37.33 to 132.77) | 72.55 (35.93 to 131.61) | 71.10 (36.05 to 130.51) |
| Central Sub-Saharan Africa | Male | Prevalence | 340.61 (191.73 to 643.89) | 1014.36 (575.46 to 1718.84) | 1357.76 (752.51 to 2412.75) | 1769.37 (960.04 to 3195.38) | 2124.49 (1153.04 to 3884.55) | 2372.77 (1288.45 to 4370.80) | 2557.60 (1389.47 to 4732.87) | 2715.93 (1473.94 to 5020.02) | 2837.08 (1533.38 to 5216.35) | 2956.11 (1594.38 to 5413.14) | 3078.29 (1659.24 to 5639.05) | 3185.82 (1715.84 to 5856.98) | 3309.52 (1780.87 to 6103.22) | 3339.58 (1796.74 to 6167.01) | 3301.99 (1779.12 to 6124.89) | 3255.97 (1761.19 to 6073.97) | 3220.47 (1747.09 to 6011.19) | 3254.99 (1752.25 to 6078.44) | 3264.47 (1764.38 to 6098.96) | 3302.58 (1782.78 to 6183.95) |
| Eastern Sub-Saharan Africa | Both | DALY rates | 8.35 (5.67 to 12.13) | 23.33 (13.87 to 42.07) | 25.25 (14.34 to 45.33) | 26.04 (14.63 to 47.51) | 26.35 (15.11 to 46.27) | 26.76 (15.67 to 48.07) | 26.99 (15.45 to 48.12) | 26.99 (15.73 to 48.14) | 27.33 (15.68 to 47.37) | 27.97 (15.89 to 49.52) | 28.20 (16.00 to 49.86) | 27.89 (16.16 to 48.98) | 27.46 (15.67 to 48.69) | 26.95 (15.90 to 47.44) | 26.72 (15.70 to 47.13) | 26.12 (15.34 to 44.88) | 25.00 (14.99 to 42.25) | 24.02 (14.23 to 40.74) | 22.78 (14.29 to 37.76) | 21.17 (13.41 to 34.14) |
| Eastern Sub-Saharan Africa | Both | Prevalence | 157.19 (123.31 to 210.00) | 404.44 (297.10 to 563.78) | 508.91 (362.12 to 743.65) | 612.74 (422.87 to 926.03) | 704.01 (474.12 to 1078.75) | 775.62 (515.04 to 1204.04) | 820.59 (538.31 to 1297.91) | 854.03 (559.88 to 1365.04) | 886.65 (580.59 to 1419.61) | 928.99 (606.37 to 1484.92) | 959.57 (627.75 to 1522.03) | 971.17 (632.52 to 1537.81) | 986.63 (637.80 to 1585.30) | 988.52 (641.42 to 1631.58) | 1002.16 (648.14 to 1639.75) | 994.85 (644.65 to 1614.36) | 968.05 (629.75 to 1571.86) | 947.49 (618.55 to 1534.36) | 929.52 (610.15 to 1517.62) | 925.39 (606.20 to 1529.88) |
| Eastern Sub-Saharan Africa | Female | DALY rates | 9.80 (6.66 to 14.22) | 16.74 (11.13 to 24.20) | 16.86 (11.41 to 24.29) | 17.00 (11.37 to 24.50) | 16.98 (11.64 to 23.81) | 17.00 (11.46 to 24.18) | 16.90 (11.26 to 23.59) | 16.69 (11.34 to 23.35) | 16.63 (11.15 to 23.14) | 16.73 (11.36 to 23.36) | 16.74 (11.35 to 23.96) | 16.55 (11.31 to 23.16) | 16.28 (11.12 to 22.44) | 16.17 (11.08 to 23.13) | 16.02 (10.95 to 22.20) | 15.85 (11.00 to 21.59) | 15.69 (10.74 to 21.80) | 15.47 (10.44 to 21.22) | 15.15 (10.57 to 20.52) | 14.93 (10.52 to 20.29) |
| Eastern Sub-Saharan Africa | Female | Prevalence | 171.69 (136.56 to 224.19) | 356.31 (267.32 to 499.74) | 446.06 (317.01 to 666.12) | 545.75 (371.98 to 850.05) | 635.95 (420.32 to 1016.73) | 702.98 (455.90 to 1139.14) | 742.55 (476.69 to 1200.67) | 772.59 (493.55 to 1264.28) | 797.92 (509.73 to 1312.49) | 830.78 (529.11 to 1356.57) | 857.43 (545.05 to 1405.51) | 867.02 (547.35 to 1437.99) | 884.76 (555.93 to 1488.83) | 891.64 (564.86 to 1519.54) | 904.11 (576.23 to 1548.98) | 899.15 (571.65 to 1525.66) | 882.08 (561.17 to 1473.96) | 866.02 (551.01 to 1440.46) | 851.47 (545.09 to 1427.05) | 858.65 (556.26 to 1434.62) |
| Eastern Sub-Saharan Africa | Male | DALY rates | 6.94 (4.58 to 10.12) | 29.73 (14.40 to 61.65) | 33.43 (16.13 to 68.87) | 35.08 (16.55 to 70.68) | 36.22 (17.46 to 73.85) | 37.34 (18.04 to 75.42) | 37.88 (18.15 to 76.72) | 38.02 (18.41 to 76.89) | 38.53 (18.37 to 77.54) | 39.40 (19.11 to 78.08) | 39.90 (19.19 to 78.81) | 39.72 (19.63 to 79.43) | 39.37 (19.11 to 78.24) | 38.84 (19.27 to 77.22) | 38.81 (18.93 to 75.57) | 38.45 (19.22 to 73.70) | 37.27 (18.48 to 72.76) | 36.46 (18.24 to 71.69) | 35.64 (17.69 to 69.12) | 35.26 (17.72 to 69.34) |
| Eastern Sub-Saharan Africa | Male | Prevalence | 143.20 (108.94 to 196.05) | 451.21 (310.50 to 692.14) | 570.22 (382.05 to 879.72) | 679.70 (451.65 to 1040.99) | 775.69 (505.07 to 1195.98) | 854.25 (543.35 to 1322.74) | 904.81 (576.25 to 1425.01) | 941.19 (603.10 to 1493.71) | 979.43 (628.52 to 1559.13) | 1028.95 (658.84 to 1627.45) | 1063.77 (684.39 to 1680.32) | 1079.81 (698.53 to 1698.63) | 1095.11 (712.89 to 1739.80) | 1095.27 (716.96 to 1769.11) | 1112.93 (727.32 to 1786.45) | 1109.73 (725.29 to 1764.29) | 1081.52 (711.04 to 1746.38) | 1066.11 (699.29 to 1721.58) | 1060.87 (693.92 to 1708.39) | 1076.18 (698.71 to 1732.37) |
| High-income Asia Pacific | Both | DALY rates | 0.05 (0.03 to 0.07) | 0.12 (0.06 to 0.22) | 0.12 (0.06 to 0.21) | 0.13 (0.07 to 0.23) | 0.13 (0.07 to 0.25) | 0.13 (0.07 to 0.25) | 0.14 (0.07 to 0.26) | 0.12 (0.06 to 0.23) | 0.10 (0.05 to 0.18) | 0.07 (0.04 to 0.13) | 0.06 (0.03 to 0.11) | 0.05 (0.03 to 0.10) | 0.04 (0.02 to 0.07) | 0.03 (0.02 to 0.05) | 0.02 (0.01 to 0.03) | 0.01 (0.01 to 0.03) | 0.01 (0.01 to 0.02) | 0.00 (0.00 to 0.01) | 0.00 (0.00 to 0.00) | 0.00 (0.00 to 0.00) |
| High-income Asia Pacific | Both | Prevalence | 0.98 (0.44 to 4.27) | 2.17 (0.84 to 9.04) | 2.56 (0.82 to 12.02) | 3.37 (0.92 to 17.13) | 3.93 (0.97 to 20.88) | 4.30 (0.99 to 23.39) | 4.69 (1.03 to 25.89) | 4.18 (0.89 to 23.28) | 3.40 (0.71 to 19.11) | 2.61 (0.53 to 14.77) | 2.25 (0.45 to 12.84) | 2.09 (0.41 to 12.01) | 1.78 (0.34 to 10.27) | 1.34 (0.25 to 7.77) | 0.76 (0.14 to 4.42) | 0.62 (0.12 to 3.61) | 0.45 (0.08 to 2.62) | 0.23 (0.04 to 1.39) | 0.09 (0.02 to 0.56) | 0.01 (0.00 to 0.07) |
| High-income Asia Pacific | Female | DALY rates | 0.05 (0.03 to 0.09) | 0.08 (0.04 to 0.12) | 0.07 (0.04 to 0.12) | 0.08 (0.05 to 0.12) | 0.08 (0.04 to 0.12) | 0.08 (0.04 to 0.12) | 0.08 (0.05 to 0.12) | 0.07 (0.04 to 0.10) | 0.05 (0.03 to 0.08) | 0.04 (0.02 to 0.06) | 0.03 (0.02 to 0.05) | 0.03 (0.02 to 0.05) | 0.02 (0.02 to 0.04) | 0.02 (0.01 to 0.03) | 0.01 (0.01 to 0.02) | 0.01 (0.00 to 0.01) | 0.01 (0.00 to 0.01) | 0.00 (0.00 to 0.00) | 0.00 (0.00 to 0.00) | 0.00 (0.00 to 0.00) |
| High-income Asia Pacific | Female | Prevalence | 1.04 (0.51 to 4.26) | 1.86 (0.73 to 8.67) | 2.19 (0.69 to 11.39) | 2.89 (0.75 to 16.11) | 3.33 (0.75 to 19.30) | 3.72 (0.77 to 22.04) | 4.03 (0.79 to 24.16) | 3.51 (0.65 to 21.22) | 2.88 (0.52 to 17.55) | 2.30 (0.40 to 14.09) | 2.05 (0.35 to 12.64) | 1.90 (0.32 to 11.76) | 1.63 (0.26 to 10.15) | 1.22 (0.19 to 7.63) | 0.69 (0.11 to 4.29) | 0.52 (0.08 to 3.23) | 0.37 (0.06 to 2.32) | 0.23 (0.04 to 1.45) | 0.12 (0.02 to 0.72) | 0.01 (0.00 to 0.07) |
| High-income Asia Pacific | Male | DALY rates | 0.04 (0.02 to 0.07) | 0.16 (0.06 to 0.33) | 0.16 (0.07 to 0.33) | 0.18 (0.08 to 0.36) | 0.19 (0.08 to 0.39) | 0.19 (0.09 to 0.39) | 0.20 (0.09 to 0.43) | 0.18 (0.08 to 0.36) | 0.14 (0.06 to 0.29) | 0.10 (0.04 to 0.21) | 0.08 (0.04 to 0.17) | 0.07 (0.03 to 0.15) | 0.06 (0.03 to 0.12) | 0.04 (0.02 to 0.09) | 0.03 (0.01 to 0.05) | 0.02 (0.01 to 0.05) | 0.02 (0.01 to 0.03) | 0.01 (0.00 to 0.01) | 0.00 (0.00 to 0.00) | 0.00 (0.00 to 0.00) |
| High-income Asia Pacific | Male | Prevalence | 0.92 (0.37 to 4.28) | 2.46 (0.87 to 9.39) | 2.91 (0.89 to 12.61) | 3.83 (1.02 to 18.09) | 4.51 (1.10 to 22.37) | 4.84 (1.12 to 24.65) | 5.31 (1.19 to 27.50) | 4.82 (1.05 to 25.22) | 3.90 (0.84 to 20.60) | 2.91 (0.61 to 15.43) | 2.44 (0.51 to 13.04) | 2.28 (0.46 to 12.26) | 1.92 (0.39 to 10.40) | 1.46 (0.29 to 7.93) | 0.84 (0.17 to 4.56) | 0.75 (0.15 to 4.09) | 0.56 (0.11 to 3.05) | 0.24 (0.05 to 1.29) | 0.03 (0.01 to 0.16) | 0.01 (0.00 to 0.06) |
| North Africa and Middle East | Both | DALY rates | 1.61 (1.04 to 2.36) | 4.51 (2.48 to 8.18) | 4.68 (2.53 to 8.38) | 4.60 (2.53 to 8.54) | 4.18 (2.29 to 7.73) | 3.74 (2.03 to 6.98) | 3.25 (1.82 to 5.86) | 2.79 (1.55 to 4.95) | 2.63 (1.49 to 4.48) | 2.45 (1.31 to 4.47) | 2.35 (1.28 to 4.30) | 2.18 (1.20 to 4.01) | 2.12 (1.16 to 3.88) | 2.08 (1.15 to 3.72) | 2.19 (1.21 to 3.98) | 2.23 (1.23 to 3.91) | 2.01 (1.13 to 3.58) | 1.73 (0.99 to 3.00) | 1.69 (0.95 to 2.98) | 1.66 (0.96 to 2.88) |
| North Africa and Middle East | Both | Prevalence | 49.10 (20.93 to 132.58) | 117.89 (49.87 to 309.67) | 151.17 (56.58 to 419.62) | 185.63 (60.42 to 542.91) | 206.21 (59.65 to 627.05) | 208.83 (57.58 to 646.02) | 198.59 (52.77 to 636.16) | 191.56 (48.37 to 619.34) | 190.31 (46.89 to 619.19) | 188.25 (45.05 to 618.52) | 199.48 (45.72 to 661.07) | 208.25 (45.67 to 706.02) | 218.74 (46.33 to 724.44) | 211.85 (45.70 to 707.03) | 198.88 (45.46 to 659.18) | 183.76 (44.49 to 605.62) | 153.87 (38.78 to 488.94) | 124.84 (32.51 to 411.03) | 121.16 (32.22 to 387.34) | 152.00 (36.47 to 502.81) |
| North Africa and Middle East | Female | DALY rates | 1.89 (1.21 to 2.84) | 3.08 (1.91 to 4.49) | 3.14 (1.88 to 4.73) | 3.04 (1.92 to 4.65) | 2.71 (1.69 to 4.12) | 2.47 (1.55 to 3.83) | 2.24 (1.42 to 3.30) | 1.99 (1.26 to 2.96) | 1.83 (1.15 to 2.68) | 1.60 (1.02 to 2.43) | 1.46 (0.92 to 2.18) | 1.31 (0.84 to 1.97) | 1.23 (0.80 to 1.81) | 1.22 (0.80 to 1.79) | 1.15 (0.74 to 1.70) | 1.21 (0.78 to 1.73) | 1.08 (0.70 to 1.53) | 0.98 (0.64 to 1.41) | 1.05 (0.67 to 1.54) | 1.08 (0.71 to 1.54) |
| North Africa and Middle East | Female | Prevalence | 51.90 (24.04 to 135.91) | 107.04 (42.16 to 298.66) | 138.86 (47.56 to 405.17) | 173.31 (51.83 to 528.87) | 196.31 (52.13 to 614.85) | 205.17 (51.23 to 654.01) | 200.14 (48.59 to 651.35) | 196.68 (45.87 to 651.02) | 194.20 (43.87 to 646.87) | 188.68 (40.82 to 633.43) | 194.60 (40.04 to 658.75) | 197.81 (39.42 to 666.04) | 202.92 (39.67 to 685.52) | 193.49 (38.53 to 644.64) | 173.96 (35.30 to 595.07) | 158.41 (34.18 to 530.88) | 123.36 (28.60 to 404.68) | 84.46 (22.98 to 260.49) | 67.33 (19.38 to 223.00) | 69.42 (19.10 to 246.92) |
| North Africa and Middle East | Male | DALY rates | 1.35 (0.80 to 2.09) | 5.86 (2.73 to 12.01) | 6.11 (2.77 to 12.83) | 6.07 (2.86 to 13.17) | 5.56 (2.47 to 12.03) | 4.93 (2.17 to 10.33) | 4.17 (1.86 to 8.59) | 3.50 (1.62 to 7.31) | 3.34 (1.56 to 6.56) | 3.19 (1.36 to 6.58) | 3.14 (1.50 to 6.37) | 3.00 (1.39 to 6.22) | 2.97 (1.39 to 6.07) | 2.93 (1.37 to 6.01) | 3.26 (1.47 to 6.47) | 3.33 (1.59 to 6.65) | 3.02 (1.41 to 6.17) | 2.49 (1.16 to 4.77) | 2.27 (1.05 to 4.66) | 2.30 (1.11 to 4.68) |
| North Africa and Middle East | Male | Prevalence | 46.45 (18.42 to 129.20) | 128.10 (53.55 to 320.08) | 162.69 (62.40 to 435.33) | 197.16 (69.18 to 557.88) | 215.57 (68.19 to 634.97) | 212.24 (64.15 to 636.25) | 197.18 (56.78 to 619.00) | 187.04 (50.68 to 592.18) | 186.88 (49.69 to 597.34) | 187.88 (48.54 to 605.48) | 203.89 (50.75 to 666.52) | 218.05 (52.20 to 737.16) | 233.79 (54.53 to 783.18) | 229.70 (54.26 to 773.80) | 224.41 (55.61 to 733.47) | 210.84 (54.06 to 676.78) | 187.38 (49.23 to 586.43) | 166.02 (41.95 to 526.84) | 171.07 (41.11 to 568.64) | 243.75 (50.29 to 834.52) |
| Oceania | Both | DALY rates | 9.90 (6.27 to 14.17) | 54.70 (29.64 to 95.16) | 79.22 (44.61 to 133.64) | 116.68 (68.64 to 190.27) | 154.49 (97.53 to 235.39) | 183.15 (116.46 to 278.87) | 200.85 (128.98 to 293.12) | 218.62 (141.86 to 321.80) | 231.45 (152.92 to 332.28) | 243.82 (159.82 to 348.97) | 257.98 (170.27 to 367.67) | 267.73 (178.08 to 379.96) | 276.36 (186.80 to 394.70) | 275.62 (184.79 to 386.36) | 263.83 (179.48 to 368.08) | 254.18 (173.08 to 356.57) | 235.18 (161.26 to 326.27) | 211.45 (144.32 to 293.39) | 179.71 (125.44 to 246.35) | 125.79 (88.49 to 171.14) |
| Oceania | Both | Prevalence | 1102.57 (328.95 to 2829.28) | 3009.68 (1085.96 to 7358.14) | 4342.17 (1557.62 to 10624.39) | 5959.21 (2158.06 to 14483.82) | 7463.32 (2748.27 to 18063.22) | 8565.02 (3186.70 to 20654.93) | 9310.68 (3515.19 to 22377.03) | 9918.82 (3829.25 to 23699.34) | 10388.33 (4065.51 to 24706.69) | 10764.63 (4282.85 to 25476.04) | 11182.39 (4566.91 to 26027.37) | 11588.55 (4796.52 to 26362.34) | 11953.67 (5025.36 to 26720.36) | 12089.25 (5086.23 to 27000.85) | 11930.21 (5024.35 to 26492.70) | 11870.73 (4983.39 to 26230.48) | 11388.11 (4830.81 to 24772.71) | 10773.58 (4530.41 to 23613.13) | 9883.21 (4003.09 to 22689.21) | 7424.63 (2776.40 to 17621.27) |
| Oceania | Female | DALY rates | 11.69 (7.22 to 17.08) | 21.72 (13.30 to 33.34) | 24.02 (13.95 to 37.27) | 26.82 (16.89 to 39.75) | 29.27 (17.96 to 43.98) | 31.60 (19.95 to 45.88) | 33.28 (21.26 to 47.93) | 34.00 (21.53 to 49.77) | 34.89 (21.93 to 52.41) | 35.63 (22.87 to 53.89) | 36.44 (24.15 to 53.42) | 36.51 (23.52 to 52.45) | 37.07 (24.15 to 52.76) | 36.69 (23.60 to 52.04) | 35.76 (23.07 to 51.12) | 35.16 (23.81 to 50.59) | 33.14 (21.82 to 45.55) | 30.90 (20.76 to 42.70) | 28.09 (19.64 to 39.60) | 19.79 (13.84 to 27.45) |
| Oceania | Female | Prevalence | 1119.70 (344.87 to 2846.03) | 2747.66 (813.22 to 7073.58) | 3896.73 (1121.10 to 10108.97) | 5225.94 (1466.48 to 13639.79) | 6430.11 (1753.92 to 16906.89) | 7320.76 (1984.09 to 19285.19) | 7935.19 (2161.40 to 20887.50) | 8391.80 (2313.12 to 21998.08) | 8760.45 (2426.49 to 22920.63) | 8996.09 (2517.10 to 23475.45) | 9284.31 (2643.46 to 23771.25) | 9533.34 (2787.40 to 23734.28) | 9799.47 (2969.91 to 23849.31) | 9840.44 (3026.86 to 23694.27) | 9733.10 (3036.05 to 23198.61) | 9686.07 (3048.99 to 22841.23) | 9307.50 (3023.13 to 21476.62) | 8852.52 (2917.65 to 20358.75) | 8311.76 (2609.11 to 19659.58) | 6027.49 (1783.89 to 15252.76) |
| Oceania | Male | DALY rates | 8.26 (4.74 to 12.78) | 84.80 (43.40 to 158.83) | 129.04 (67.75 to 231.32) | 198.01 (111.43 to 332.85) | 270.86 (164.42 to 421.95) | 330.42 (206.60 to 506.03) | 370.96 (234.13 to 550.97) | 404.39 (262.35 to 600.66) | 425.62 (278.78 to 617.95) | 444.98 (288.15 to 640.50) | 460.42 (301.14 to 662.97) | 476.08 (315.88 to 678.09) | 487.91 (328.99 to 702.89) | 489.09 (327.91 to 691.14) | 472.99 (315.96 to 661.60) | 464.88 (316.69 to 657.00) | 442.74 (300.63 to 618.32) | 418.95 (285.42 to 587.60) | 379.40 (259.67 to 525.48) | 306.80 (212.80 to 419.28) |
| Oceania | Male | Prevalence | 1086.87 (313.10 to 2813.93) | 3248.80 (1299.29 to 7613.62) | 4744.14 (1935.72 to 11079.64) | 6622.85 (2809.77 to 15257.45) | 8423.52 (3664.08 to 19121.50) | 9774.18 (4336.09 to 21901.86) | 10706.92 (4876.78 to 23729.63) | 11455.36 (5334.05 to 25139.65) | 11996.40 (5661.38 to 26155.07) | 12473.41 (5927.87 to 27068.08) | 12916.91 (6243.71 to 27714.36) | 13440.56 (6582.41 to 28388.84) | 13858.14 (6843.06 to 29050.00) | 14098.49 (6953.66 to 29676.21) | 13945.10 (6877.56 to 29318.17) | 13972.39 (6855.46 to 29367.34) | 13525.47 (6677.96 to 28300.83) | 12981.48 (6348.88 to 27412.14) | 11952.85 (5586.14 to 25872.53) | 9810.32 (4453.66 to 21667.87) |
| South Asia | Both | DALY rates | 8.19 (5.53 to 11.57) | 26.37 (14.94 to 47.18) | 31.60 (17.61 to 56.32) | 37.14 (20.85 to 65.66) | 41.78 (23.91 to 72.51) | 44.31 (25.21 to 75.09) | 46.18 (26.11 to 78.43) | 47.74 (27.44 to 80.71) | 49.39 (28.60 to 81.77) | 50.87 (29.69 to 84.91) | 51.26 (29.98 to 84.88) | 51.31 (30.01 to 83.56) | 52.67 (31.76 to 84.36) | 53.90 (32.74 to 86.47) | 53.80 (32.48 to 85.66) | 51.71 (31.92 to 82.51) | 47.75 (29.67 to 74.77) | 43.35 (26.98 to 68.33) | 40.02 (25.08 to 63.22) | 32.61 (20.73 to 50.40) |
| South Asia | Both | Prevalence | 335.92 (298.91 to 376.80) | 856.25 (739.95 to 1031.31) | 1211.44 (1042.64 to 1443.46) | 1623.12 (1399.75 to 1922.76) | 1944.35 (1680.21 to 2280.58) | 2110.49 (1830.22 to 2461.08) | 2221.54 (1938.80 to 2582.31) | 2337.37 (2047.27 to 2706.91) | 2440.93 (2137.82 to 2819.92) | 2527.47 (2215.07 to 2913.11) | 2582.78 (2270.59 to 2962.80) | 2641.41 (2329.36 to 3021.45) | 2777.72 (2449.09 to 3172.06) | 2907.48 (2560.76 to 3313.53) | 2977.81 (2617.28 to 3390.62) | 2944.30 (2588.95 to 3349.75) | 2870.99 (2530.75 to 3273.33) | 2715.94 (2408.09 to 3089.58) | 2597.94 (2292.53 to 2949.38) | 2498.42 (2189.55 to 2878.05) |
| South Asia | Female | DALY rates | 9.61 (6.39 to 13.57) | 16.35 (11.22 to 23.23) | 17.23 (11.51 to 24.57) | 18.08 (12.24 to 25.44) | 18.50 (12.41 to 26.12) | 18.62 (12.86 to 26.36) | 18.81 (12.70 to 26.44) | 18.92 (12.98 to 26.66) | 19.08 (12.92 to 26.75) | 19.13 (13.12 to 26.73) | 19.31 (13.47 to 26.86) | 19.42 (13.38 to 27.81) | 19.56 (13.42 to 27.81) | 19.59 (13.41 to 27.35) | 19.44 (13.38 to 26.63) | 18.95 (13.00 to 26.18) | 18.65 (12.92 to 25.92) | 18.02 (12.66 to 24.30) | 17.46 (12.48 to 23.55) | 17.07 (12.31 to 22.96) |
| South Asia | Female | Prevalence | 347.97 (310.35 to 388.52) | 776.08 (685.81 to 870.71) | 1094.05 (959.98 to 1234.89) | 1459.81 (1274.79 to 1656.77) | 1738.16 (1514.73 to 1977.81) | 1879.48 (1642.59 to 2136.03) | 1976.15 (1732.94 to 2242.61) | 2087.69 (1833.16 to 2367.67) | 2171.69 (1905.17 to 2462.38) | 2230.89 (1956.79 to 2528.97) | 2299.25 (2020.52 to 2605.90) | 2379.64 (2094.21 to 2698.97) | 2518.14 (2215.88 to 2859.92) | 2637.20 (2318.25 to 2995.81) | 2696.39 (2371.07 to 3060.80) | 2666.49 (2348.51 to 3020.65) | 2632.71 (2326.03 to 2972.18) | 2517.83 (2233.38 to 2844.54) | 2419.93 (2144.04 to 2737.52) | 2471.60 (2189.61 to 2794.63) |
| South Asia | Male | DALY rates | 6.88 (4.53 to 9.85) | 35.60 (17.10 to 71.19) | 44.83 (21.66 to 87.45) | 54.91 (27.40 to 105.51) | 64.16 (32.62 to 120.54) | 69.39 (36.13 to 127.26) | 72.74 (37.65 to 131.19) | 75.70 (39.60 to 136.03) | 78.92 (41.37 to 140.68) | 81.89 (44.83 to 146.26) | 82.73 (44.88 to 145.87) | 83.15 (45.33 to 144.91) | 86.48 (47.91 to 147.82) | 89.28 (50.50 to 152.13) | 89.72 (50.36 to 151.45) | 86.81 (49.15 to 147.39) | 83.03 (46.79 to 139.21) | 76.51 (43.43 to 130.75) | 71.83 (40.91 to 122.91) | 61.97 (34.68 to 107.53) |
| South Asia | Male | Prevalence | 324.86 (286.68 to 366.81) | 930.04 (763.64 to 1219.31) | 1319.43 (1096.60 to 1688.85) | 1775.33 (1489.85 to 2199.50) | 2142.58 (1802.99 to 2618.56) | 2336.06 (1969.77 to 2834.30) | 2459.64 (2085.32 to 2969.74) | 2579.56 (2196.65 to 3100.58) | 2703.26 (2303.82 to 3246.02) | 2817.22 (2404.29 to 3380.52) | 2861.99 (2448.49 to 3428.75) | 2902.75 (2491.35 to 3473.12) | 3042.76 (2616.18 to 3634.88) | 3186.18 (2741.77 to 3795.93) | 3272.06 (2815.69 to 3887.42) | 3241.99 (2787.52 to 3849.58) | 3159.73 (2719.08 to 3758.98) | 2975.30 (2564.21 to 3542.25) | 2848.96 (2451.27 to 3402.66) | 2549.11 (2173.03 to 3125.82) |
| Southeast Asia | Both | DALY rates | 8.76 (5.92 to 12.55) | 26.06 (14.55 to 47.99) | 27.41 (15.43 to 49.32) | 29.15 (16.39 to 52.00) | 30.68 (17.23 to 54.21) | 31.72 (17.57 to 56.20) | 32.57 (18.34 to 57.46) | 32.46 (18.46 to 57.11) | 32.49 (18.57 to 57.09) | 32.22 (18.56 to 56.85) | 31.48 (18.14 to 55.41) | 30.76 (17.77 to 53.88) | 30.18 (17.26 to 52.12) | 29.40 (17.24 to 49.58) | 27.66 (16.32 to 46.80) | 25.86 (15.63 to 42.44) | 23.06 (14.24 to 38.13) | 20.23 (12.53 to 33.14) | 17.44 (10.76 to 28.33) | 14.31 (8.86 to 23.28) |
| Southeast Asia | Both | Prevalence | 230.50 (140.34 to 400.94) | 582.77 (341.61 to 1055.84) | 758.71 (420.18 to 1327.84) | 960.62 (513.61 to 1747.31) | 1125.02 (576.09 to 2115.15) | 1230.08 (619.29 to 2343.43) | 1309.05 (646.21 to 2552.73) | 1364.27 (663.08 to 2729.24) | 1417.77 (679.17 to 2919.36) | 1459.02 (687.66 to 3077.12) | 1473.86 (691.58 to 3138.26) | 1490.00 (697.98 to 3216.43) | 1517.69 (712.89 to 3357.83) | 1536.72 (722.50 to 3428.57) | 1513.58 (714.40 to 3378.05) | 1488.34 (695.59 to 3328.97) | 1406.76 (659.83 to 3325.55) | 1320.56 (598.71 to 3059.43) | 1247.79 (549.89 to 2913.37) | 1138.78 (504.80 to 2855.62) |
| Southeast Asia | Female | DALY rates | 10.26 (6.86 to 14.64) | 17.09 (11.38 to 24.56) | 17.10 (11.48 to 24.56) | 17.15 (11.61 to 24.30) | 17.26 (11.59 to 24.39) | 17.31 (11.78 to 24.93) | 17.44 (11.94 to 24.93) | 17.03 (11.62 to 24.39) | 16.85 (11.31 to 23.80) | 16.44 (11.04 to 23.68) | 16.05 (10.84 to 22.64) | 15.62 (10.52 to 22.36) | 15.21 (10.08 to 21.37) | 14.89 (10.21 to 20.94) | 14.14 (9.76 to 19.31) | 13.63 (9.17 to 18.91) | 12.88 (9.03 to 18.00) | 11.81 (8.19 to 16.22) | 10.48 (7.06 to 14.50) | 8.56 (5.86 to 11.87) |
| Southeast Asia | Female | Prevalence | 245.71 (154.80 to 416.30) | 516.03 (293.81 to 1020.68) | 681.34 (363.75 to 1287.04) | 869.19 (435.60 to 1691.09) | 1021.58 (490.30 to 2050.53) | 1117.82 (528.81 to 2273.00) | 1191.84 (557.69 to 2490.47) | 1244.09 (571.10 to 2666.84) | 1295.26 (577.62 to 2861.45) | 1332.88 (588.10 to 3011.45) | 1344.31 (594.20 to 3054.90) | 1358.76 (589.41 to 3116.19) | 1381.03 (590.97 to 3217.98) | 1396.57 (588.52 to 3259.91) | 1377.67 (598.80 to 3208.47) | 1361.21 (595.94 to 3194.44) | 1292.40 (552.81 to 3159.20) | 1222.71 (506.23 to 2909.79) | 1173.79 (474.51 to 2900.75) | 1085.06 (402.74 to 2883.06) |
| Southeast Asia | Male | DALY rates | 7.35 (4.86 to 11.02) | 34.54 (16.88 to 71.81) | 37.14 (17.62 to 74.86) | 40.57 (19.79 to 80.69) | 43.61 (21.25 to 85.07) | 45.76 (22.79 to 89.67) | 47.32 (23.37 to 93.23) | 47.62 (23.58 to 94.30) | 47.98 (24.29 to 93.49) | 48.13 (23.90 to 91.79) | 47.36 (23.96 to 90.37) | 46.78 (23.95 to 90.26) | 46.52 (23.31 to 88.33) | 46.02 (24.04 to 86.44) | 43.97 (22.68 to 82.69) | 42.29 (22.19 to 78.74) | 38.68 (20.56 to 72.03) | 34.72 (18.24 to 65.70) | 29.99 (15.68 to 56.11) | 24.00 (12.72 to 44.03) |
| Southeast Asia | Male | Prevalence | 216.18 (124.52 to 387.77) | 645.79 (365.25 to 1162.28) | 831.75 (461.57 to 1456.87) | 1047.72 (562.15 to 1844.45) | 1224.73 (644.37 to 2198.06) | 1339.44 (700.54 to 2452.29) | 1423.22 (743.25 to 2656.76) | 1482.25 (768.22 to 2792.12) | 1539.22 (798.10 to 2978.42) | 1586.12 (814.28 to 3143.75) | 1607.20 (815.19 to 3224.93) | 1628.93 (819.45 to 3323.01) | 1666.86 (830.15 to 3512.97) | 1697.21 (838.72 to 3625.39) | 1677.57 (813.45 to 3582.68) | 1659.13 (805.87 to 3472.60) | 1582.16 (774.71 to 3531.44) | 1489.00 (728.69 to 3220.69) | 1381.24 (678.50 to 2984.02) | 1229.14 (616.98 to 2729.45) |
| Southern Sub-Saharan Africa | Both | DALY rates | 2.66 (1.69 to 3.97) | 7.26 (3.76 to 13.09) | 7.16 (3.95 to 12.97) | 6.96 (3.69 to 12.78) | 6.49 (3.51 to 11.89) | 5.28 (2.94 to 9.49) | 4.60 (2.43 to 8.34) | 4.62 (2.55 to 8.26) | 4.88 (2.60 to 8.70) | 4.65 (2.58 to 8.69) | 4.15 (2.36 to 7.17) | 3.51 (1.93 to 6.22) | 3.44 (1.94 to 5.73) | 3.63 (2.04 to 6.33) | 3.27 (1.90 to 5.51) | 2.87 (1.69 to 4.78) | 2.03 (1.20 to 3.40) | 1.54 (0.86 to 2.55) | 1.20 (0.69 to 2.02) | 1.35 (0.80 to 2.19) |
| Southern Sub-Saharan Africa | Both | Prevalence | 54.18 (28.12 to 147.30) | 132.74 (63.82 to 346.70) | 157.21 (66.26 to 455.46) | 181.85 (67.67 to 567.97) | 192.80 (65.92 to 628.73) | 170.70 (55.43 to 570.55) | 157.30 (49.48 to 533.43) | 165.29 (50.92 to 565.63) | 180.18 (54.57 to 620.09) | 176.22 (52.62 to 609.07) | 163.36 (48.02 to 568.71) | 144.94 (41.74 to 510.06) | 149.36 (41.84 to 530.73) | 162.54 (45.10 to 579.19) | 149.44 (41.28 to 533.04) | 134.15 (37.15 to 478.19) | 96.98 (26.93 to 345.58) | 73.94 (20.50 to 263.50) | 59.82 (16.34 to 213.96) | 72.84 (19.40 to 262.94) |
| Southern Sub-Saharan Africa | Female | DALY rates | 3.16 (1.91 to 4.92) | 4.96 (2.82 to 7.88) | 4.79 (2.77 to 7.38) | 4.62 (2.67 to 7.21) | 4.36 (2.61 to 6.86) | 3.54 (2.16 to 5.26) | 3.11 (1.83 to 4.90) | 3.05 (1.89 to 4.45) | 3.12 (1.82 to 4.95) | 2.80 (1.67 to 4.35) | 2.50 (1.49 to 3.86) | 2.27 (1.38 to 3.42) | 2.33 (1.25 to 3.67) | 2.39 (1.43 to 3.60) | 2.19 (1.33 to 3.36) | 1.81 (1.13 to 2.80) | 1.18 (0.70 to 1.76) | 0.84 (0.48 to 1.22) | 0.66 (0.40 to 1.02) | 0.87 (0.52 to 1.31) |
| Southern Sub-Saharan Africa | Female | Prevalence | 58.88 (32.53 to 151.70) | 115.87 (54.32 to 335.91) | 140.68 (55.09 to 448.19) | 166.82 (55.80 to 567.03) | 183.12 (55.25 to 644.86) | 164.73 (46.58 to 591.66) | 154.26 (41.65 to 560.75) | 161.90 (42.30 to 593.37) | 172.81 (44.04 to 637.47) | 161.38 (40.27 to 598.40) | 148.65 (36.39 to 553.65) | 137.69 (33.05 to 515.07) | 146.58 (34.44 to 551.03) | 156.70 (36.58 to 590.21) | 143.86 (33.54 to 541.83) | 122.37 (28.50 to 460.90) | 81.96 (19.13 to 308.70) | 58.39 (13.60 to 219.92) | 47.13 (10.99 to 177.52) | 62.80 (14.62 to 236.52) |
| Southern Sub-Saharan Africa | Male | DALY rates | 2.18 (1.20 to 3.50) | 9.53 (4.10 to 20.31) | 9.50 (4.34 to 20.01) | 9.29 (4.14 to 19.35) | 8.60 (3.70 to 18.13) | 7.06 (3.20 to 14.37) | 6.13 (2.62 to 12.68) | 6.22 (2.72 to 12.97) | 6.66 (2.99 to 13.76) | 6.60 (3.07 to 14.04) | 6.03 (2.80 to 11.91) | 5.06 (2.21 to 10.28) | 4.92 (2.37 to 10.09) | 5.38 (2.39 to 10.61) | 4.85 (2.19 to 9.44) | 4.61 (2.18 to 9.16) | 3.75 (1.83 to 7.61) | 3.31 (1.47 to 6.71) | 2.84 (1.33 to 5.55) | 2.95 (1.35 to 6.11) |
| Southern Sub-Saharan Africa | Male | Prevalence | 49.58 (23.56 to 143.07) | 149.36 (66.56 to 375.95) | 173.45 (69.82 to 470.60) | 196.72 (73.73 to 571.94) | 202.43 (71.94 to 613.87) | 176.81 (60.75 to 549.40) | 160.40 (53.81 to 506.10) | 168.72 (55.65 to 538.04) | 187.59 (61.07 to 603.11) | 191.86 (61.84 to 620.79) | 180.11 (57.42 to 586.29) | 154.00 (48.55 to 504.14) | 153.06 (47.71 to 503.98) | 170.80 (53.01 to 563.96) | 157.63 (49.01 to 520.46) | 153.56 (47.75 to 506.97) | 127.27 (39.49 to 420.24) | 113.13 (35.15 to 373.51) | 98.50 (30.57 to 325.21) | 106.61 (33.13 to 352.02) |
| Tropical Latin America | Both | DALY rates | 0.49 (0.29 to 0.76) | 0.79 (0.49 to 1.16) | 0.78 (0.50 to 1.22) | 0.75 (0.46 to 1.12) | 0.72 (0.46 to 1.10) | 0.69 (0.42 to 1.01) | 0.69 (0.41 to 1.05) | 0.69 (0.43 to 1.05) | 0.69 (0.43 to 1.06) | 0.69 (0.44 to 1.03) | 0.67 (0.41 to 0.98) | 0.63 (0.39 to 0.94) | 0.60 (0.39 to 0.87) | 0.59 (0.38 to 0.86) | 0.62 (0.39 to 0.92) | 0.62 (0.39 to 0.90) | 0.62 (0.39 to 0.93) | 0.56 (0.37 to 0.86) | 0.52 (0.33 to 0.74) | 0.58 (0.36 to 0.84) |
| Tropical Latin America | Both | Prevalence | 5.01 (4.60 to 6.08) | 8.17 (7.41 to 10.72) | 8.37 (7.44 to 12.05) | 8.34 (7.25 to 13.17) | 8.20 (7.00 to 13.82) | 8.11 (6.85 to 14.26) | 8.29 (6.91 to 15.00) | 8.34 (6.88 to 15.40) | 8.41 (6.89 to 15.80) | 8.56 (6.98 to 16.33) | 8.42 (6.83 to 16.24) | 8.12 (6.56 to 15.87) | 7.94 (6.38 to 15.78) | 7.91 (6.33 to 15.80) | 8.35 (6.69 to 16.69) | 8.51 (6.81 to 16.95) | 8.61 (6.89 to 17.08) | 7.94 (6.39 to 15.73) | 7.48 (6.00 to 14.75) | 8.55 (6.85 to 17.03) |
| Tropical Latin America | Female | DALY rates | 0.58 (0.32 to 0.93) | 0.93 (0.54 to 1.49) | 0.91 (0.54 to 1.50) | 0.87 (0.51 to 1.34) | 0.84 (0.51 to 1.29) | 0.81 (0.45 to 1.25) | 0.82 (0.46 to 1.28) | 0.82 (0.49 to 1.30) | 0.81 (0.46 to 1.28) | 0.81 (0.50 to 1.31) | 0.79 (0.44 to 1.23) | 0.75 (0.41 to 1.17) | 0.71 (0.44 to 1.10) | 0.71 (0.42 to 1.09) | 0.74 (0.42 to 1.12) | 0.72 (0.42 to 1.07) | 0.73 (0.43 to 1.14) | 0.64 (0.39 to 1.00) | 0.59 (0.35 to 0.88) | 0.60 (0.36 to 0.90) |
| Tropical Latin America | Female | Prevalence | 5.85 (5.33 to 7.00) | 9.52 (8.58 to 12.19) | 9.72 (8.60 to 13.50) | 9.65 (8.35 to 14.59) | 9.51 (8.07 to 15.33) | 9.43 (7.93 to 15.85) | 9.68 (8.07 to 16.74) | 9.69 (8.01 to 17.12) | 9.73 (8.00 to 17.49) | 9.93 (8.13 to 18.13) | 9.80 (7.97 to 18.11) | 9.43 (7.64 to 17.67) | 9.23 (7.44 to 17.57) | 9.22 (7.40 to 17.67) | 9.69 (7.79 to 18.59) | 9.81 (7.88 to 18.77) | 9.93 (7.96 to 19.01) | 8.88 (7.11 to 17.00) | 8.28 (6.66 to 15.85) | 8.65 (6.95 to 16.57) |
| Tropical Latin America | Male | DALY rates | 0.41 (0.22 to 0.68) | 0.66 (0.36 to 1.06) | 0.65 (0.36 to 1.07) | 0.64 (0.33 to 1.01) | 0.60 (0.31 to 1.00) | 0.57 (0.30 to 0.93) | 0.56 (0.31 to 0.89) | 0.56 (0.31 to 0.91) | 0.57 (0.31 to 0.94) | 0.56 (0.32 to 0.89) | 0.54 (0.30 to 0.85) | 0.49 (0.28 to 0.77) | 0.48 (0.27 to 0.74) | 0.46 (0.26 to 0.73) | 0.49 (0.27 to 0.78) | 0.48 (0.27 to 0.76) | 0.46 (0.24 to 0.74) | 0.44 (0.26 to 0.67) | 0.40 (0.22 to 0.64) | 0.54 (0.30 to 0.83) |
| Tropical Latin America | Male | Prevalence | 4.20 (3.73 to 5.23) | 6.88 (6.03 to 9.33) | 7.08 (6.06 to 10.58) | 7.06 (5.89 to 11.62) | 6.92 (5.65 to 12.24) | 6.77 (5.45 to 12.55) | 6.86 (5.46 to 13.12) | 6.92 (5.47 to 13.55) | 7.01 (5.49 to 14.00) | 7.08 (5.51 to 14.37) | 6.92 (5.35 to 14.21) | 6.64 (5.09 to 13.85) | 6.47 (4.93 to 13.73) | 6.34 (4.82 to 13.57) | 6.70 (5.10 to 14.34) | 6.77 (5.15 to 14.49) | 6.68 (5.07 to 14.28) | 6.32 (4.81 to 13.54) | 5.87 (4.46 to 12.56) | 8.37 (6.36 to 17.90) |
| Western Sub-Saharan Africa | Both | DALY rates | 9.19 (6.18 to 13.09) | 28.83 (16.61 to 50.84) | 32.62 (18.42 to 57.82) | 36.60 (20.98 to 63.80) | 40.71 (23.96 to 68.57) | 44.05 (25.92 to 73.19) | 47.43 (28.78 to 77.44) | 51.13 (31.18 to 81.74) | 54.28 (33.51 to 85.79) | 55.16 (34.57 to 87.48) | 53.43 (33.01 to 83.72) | 53.45 (33.33 to 84.23) | 54.27 (34.49 to 84.11) | 54.47 (34.44 to 84.99) | 52.67 (33.17 to 82.77) | 50.30 (31.66 to 78.06) | 48.15 (30.29 to 74.91) | 47.49 (30.03 to 72.60) | 45.91 (29.50 to 70.17) | 38.47 (25.54 to 57.19) |
| Western Sub-Saharan Africa | Both | Prevalence | 298.91 (205.51 to 444.97) | 785.92 (521.82 to 1149.76) | 1057.48 (689.03 to 1568.38) | 1371.98 (883.01 to 2063.95) | 1661.57 (1054.47 to 2523.15) | 1887.32 (1202.41 to 2894.19) | 2077.86 (1312.42 to 3173.65) | 2255.61 (1432.83 to 3433.20) | 2381.12 (1519.91 to 3622.41) | 2439.60 (1565.97 to 3729.54) | 2425.75 (1535.44 to 3760.17) | 2497.33 (1591.53 to 3828.21) | 2588.95 (1646.71 to 3979.22) | 2643.26 (1702.52 to 4078.05) | 2616.97 (1656.49 to 4068.41) | 2562.33 (1624.41 to 4039.05) | 2516.25 (1582.21 to 4017.32) | 2555.48 (1603.48 to 4089.27) | 2631.46 (1624.28 to 4343.97) | 2646.42 (1533.70 to 4705.93) |
| Western Sub-Saharan Africa | Female | DALY rates | 10.79 (7.31 to 15.54) | 18.69 (12.62 to 27.06) | 18.91 (12.83 to 26.96) | 19.34 (12.88 to 28.08) | 19.60 (13.17 to 27.87) | 19.84 (13.51 to 28.37) | 19.97 (13.44 to 28.13) | 20.08 (13.75 to 28.15) | 20.20 (13.77 to 28.66) | 20.15 (13.76 to 28.19) | 20.01 (13.74 to 28.04) | 20.05 (13.80 to 27.71) | 20.10 (13.68 to 28.15) | 19.91 (13.94 to 27.83) | 19.61 (13.70 to 27.14) | 19.09 (13.39 to 25.73) | 18.58 (12.85 to 25.72) | 18.46 (12.94 to 25.14) | 18.39 (12.89 to 25.17) | 17.86 (12.67 to 24.14) |
| Western Sub-Saharan Africa | Female | Prevalence | 314.50 (218.73 to 463.70) | 705.72 (466.56 to 1077.49) | 944.89 (599.74 to 1466.19) | 1226.21 (752.46 to 1916.58) | 1481.78 (903.94 to 2356.22) | 1680.36 (1018.63 to 2703.82) | 1837.82 (1103.07 to 2952.53) | 1963.75 (1185.44 to 3167.77) | 2031.07 (1218.16 to 3294.00) | 2072.24 (1242.24 to 3388.92) | 2079.95 (1214.10 to 3492.08) | 2165.67 (1268.48 to 3606.03) | 2261.82 (1323.01 to 3805.85) | 2326.68 (1375.72 to 3970.46) | 2316.41 (1365.20 to 3906.74) | 2256.80 (1319.67 to 3795.35) | 2213.82 (1293.09 to 3681.73) | 2262.48 (1323.41 to 3795.11) | 2392.78 (1388.96 to 4164.40) | 2487.42 (1318.09 to 4900.53) |
| Western Sub-Saharan Africa | Male | DALY rates | 7.63 (5.11 to 11.04) | 38.80 (19.07 to 77.97) | 46.52 (22.55 to 92.03) | 54.82 (28.00 to 104.96) | 64.01 (33.89 to 116.98) | 71.60 (38.27 to 127.92) | 78.50 (43.32 to 138.89) | 85.58 (47.72 to 145.08) | 90.68 (51.88 to 153.17) | 92.38 (54.76 to 154.76) | 90.42 (51.72 to 149.58) | 91.87 (52.52 to 151.13) | 93.74 (54.51 to 152.62) | 92.95 (54.00 to 152.10) | 88.62 (52.32 to 146.19) | 84.60 (49.82 to 139.71) | 81.74 (47.71 to 135.04) | 81.30 (48.25 to 133.16) | 79.61 (46.49 to 130.11) | 78.46 (47.02 to 127.39) |
| Western Sub-Saharan Africa | Male | Prevalence | 283.78 (191.91 to 428.22) | 864.81 (553.35 to 1265.50) | 1171.49 (746.91 to 1700.73) | 1525.92 (980.93 to 2237.70) | 1859.95 (1195.74 to 2725.99) | 2122.86 (1370.06 to 3131.94) | 2349.45 (1526.19 to 3456.29) | 2579.55 (1678.54 to 3805.09) | 2755.16 (1794.78 to 4063.61) | 2830.09 (1851.42 to 4161.98) | 2808.55 (1824.50 to 4134.83) | 2878.90 (1882.81 to 4189.33) | 2966.77 (1951.43 to 4290.42) | 2995.79 (1987.10 to 4311.19) | 2943.81 (1931.14 to 4311.65) | 2898.12 (1903.76 to 4408.59) | 2859.80 (1869.50 to 4441.07) | 2896.67 (1902.91 to 4420.12) | 2923.82 (1875.01 to 4544.35) | 2954.88 (1923.31 to 4589.21) |
| **Country and territory** |  |  |  |  |  |  |  |  |  |  |  |  |  |  |  |  |  |  |  |  |  |  |
| American Samoa | Both | DALY rates | 10.84 (7.11 to 16.20) | 39.19 (21.04 to 70.63) | 47.76 (25.02 to 85.40) | 59.10 (31.45 to 103.65) | 68.75 (38.22 to 119.57) | 77.59 (42.30 to 130.95) | 85.36 (48.50 to 145.21) | 92.28 (51.68 to 157.18) | 97.37 (55.40 to 162.95) | 101.49 (57.80 to 169.96) | 105.73 (61.22 to 174.31) | 109.95 (63.58 to 178.08) | 113.02 (68.91 to 182.32) | 112.33 (68.11 to 178.53) | 110.06 (65.11 to 172.64) | 107.50 (64.09 to 169.68) | 104.68 (63.35 to 167.83) | 98.71 (61.36 to 157.92) | 90.31 (56.64 to 139.60) | 84.31 (52.45 to 126.50) |
| American Samoa | Both | Prevalence | 711.73 (302.55 to 1624.03) | 1760.61 (769.98 to 3921.53) | 2479.27 (1045.39 to 5623.19) | 3304.03 (1371.86 to 7548.62) | 4007.92 (1658.17 to 9184.04) | 4542.48 (1879.54 to 10411.97) | 4943.96 (2058.61 to 11304.79) | 5273.52 (2207.19 to 12038.20) | 5545.55 (2328.80 to 12644.57) | 5783.17 (2435.87 to 13169.43) | 6012.46 (2540.01 to 13673.22) | 6244.07 (2646.41 to 14181.99) | 6499.01 (2754.76 to 14759.55) | 6615.80 (2807.23 to 15029.37) | 6614.08 (2806.55 to 15030.83) | 6609.60 (2800.92 to 15027.20) | 6600.27 (2793.68 to 15018.64) | 6561.66 (2760.30 to 14982.30) | 6502.42 (2710.36 to 14930.04) | 6461.90 (2669.62 to 14893.23) |
| American Samoa | Female | DALY rates | 12.74 (7.85 to 19.39) | 21.23 (12.21 to 32.93) | 22.51 (13.38 to 33.27) | 23.29 (12.29 to 36.16) | 24.36 (14.65 to 37.91) | 24.91 (15.32 to 37.47) | 25.39 (15.04 to 38.40) | 26.13 (15.56 to 40.12) | 26.18 (15.36 to 40.67) | 26.08 (16.13 to 40.13) | 26.35 (16.13 to 39.61) | 26.45 (15.81 to 38.51) | 26.87 (15.98 to 41.78) | 26.66 (17.06 to 37.92) | 26.27 (16.40 to 39.00) | 25.91 (16.09 to 37.84) | 25.05 (15.85 to 37.74) | 24.71 (15.91 to 36.22) | 23.92 (15.01 to 35.55) | 23.46 (15.63 to 33.68) |
| American Samoa | Female | Prevalence | 731.53 (321.16 to 1644.48) | 1620.93 (664.25 to 3752.78) | 2280.11 (886.15 to 5386.11) | 3022.69 (1136.97 to 7226.13) | 3654.10 (1350.46 to 8789.60) | 4121.95 (1508.93 to 9946.80) | 4457.09 (1623.49 to 10775.39) | 4731.37 (1714.64 to 11453.87) | 4958.92 (1793.45 to 12017.81) | 5155.33 (1858.86 to 12502.14) | 5342.61 (1922.37 to 12964.96) | 5532.73 (1987.81 to 13434.81) | 5753.37 (2063.64 to 13979.57) | 5858.79 (2099.54 to 14239.34) | 5858.79 (2098.18 to 14241.09) | 5858.83 (2099.70 to 14238.53) | 5858.80 (2098.96 to 14241.05) | 5858.82 (2099.18 to 14240.38) | 5858.75 (2099.45 to 14240.71) | 5858.78 (2098.82 to 14241.19) |
| American Samoa | Male | DALY rates | 9.06 (5.06 to 14.66) | 56.36 (25.92 to 110.88) | 71.12 (32.41 to 137.61) | 92.14 (43.61 to 171.55) | 112.86 (56.41 to 212.05) | 130.96 (65.65 to 234.13) | 143.41 (75.48 to 256.64) | 154.96 (82.48 to 272.16) | 164.67 (88.18 to 283.68) | 172.41 (92.95 to 298.02) | 179.78 (99.52 to 306.43) | 187.83 (103.37 to 312.51) | 196.78 (115.33 to 322.99) | 198.40 (114.66 to 324.32) | 194.65 (110.26 to 316.78) | 190.84 (109.49 to 308.17) | 188.02 (107.65 to 309.45) | 184.49 (106.69 to 303.16) | 180.46 (107.77 to 286.04) | 176.60 (104.83 to 279.00) |
| American Samoa | Male | Prevalence | 693.06 (283.91 to 1604.76) | 1894.13 (897.51 to 4065.13) | 2663.39 (1217.05 to 5809.00) | 3563.56 (1604.10 to 7797.12) | 4359.52 (1952.72 to 9511.50) | 4968.50 (2226.28 to 10806.42) | 5415.16 (2438.85 to 11737.46) | 5787.22 (2627.38 to 12508.09) | 6100.21 (2780.09 to 13150.65) | 6373.59 (2925.01 to 13713.30) | 6637.31 (3057.82 to 14262.90) | 6907.51 (3204.00 to 14818.94) | 7223.91 (3369.15 to 15464.48) | 7376.39 (3455.33 to 15775.05) | 7376.45 (3453.90 to 15780.22) | 7376.51 (3454.72 to 15781.46) | 7376.30 (3454.77 to 15778.28) | 7376.43 (3452.15 to 15778.13) | 7376.44 (3457.61 to 15779.65) | 7376.48 (3453.81 to 15778.80) |
| Angola | Both | DALY rates | 9.75 (5.87 to 14.41) | 28.85 (15.32 to 50.84) | 29.70 (16.04 to 54.03) | 30.59 (16.47 to 55.07) | 31.91 (16.46 to 55.63) | 32.41 (17.83 to 58.90) | 32.65 (16.86 to 59.05) | 33.21 (17.89 to 58.89) | 33.60 (18.45 to 60.25) | 33.83 (18.54 to 58.59) | 34.05 (18.37 to 58.58) | 33.53 (18.51 to 59.15) | 33.71 (17.99 to 60.49) | 33.32 (18.90 to 58.22) | 32.26 (17.77 to 55.48) | 31.14 (17.30 to 53.99) | 29.74 (16.23 to 50.23) | 28.55 (16.67 to 47.65) | 26.88 (15.66 to 44.43) | 24.45 (14.61 to 39.43) |
| Angola | Both | Prevalence | 234.49 (107.53 to 620.30) | 608.16 (265.57 to 1596.22) | 775.77 (290.97 to 2217.49) | 969.53 (324.35 to 2915.68) | 1137.00 (352.23 to 3520.82) | 1257.12 (371.56 to 3956.09) | 1343.69 (385.90 to 4267.60) | 1413.92 (398.40 to 4517.49) | 1474.38 (407.98 to 4733.31) | 1526.73 (417.89 to 4919.12) | 1576.37 (426.13 to 5095.56) | 1623.38 (432.41 to 5270.50) | 1679.76 (441.97 to 5473.50) | 1706.26 (445.70 to 5572.84) | 1700.82 (441.96 to 5564.89) | 1695.08 (438.39 to 5557.53) | 1690.95 (436.19 to 5552.24) | 1684.68 (431.55 to 5543.05) | 1672.42 (421.46 to 5525.73) | 1655.46 (412.14 to 5503.74) |
| Angola | Female | DALY rates | 11.39 (6.60 to 18.55) | 19.22 (10.83 to 29.46) | 19.39 (11.35 to 30.98) | 19.53 (11.69 to 30.19) | 19.30 (11.35 to 28.80) | 19.48 (11.35 to 30.14) | 19.36 (11.91 to 29.65) | 19.52 (11.57 to 30.37) | 19.36 (11.34 to 29.54) | 19.49 (11.11 to 30.76) | 19.38 (11.32 to 29.78) | 19.47 (11.89 to 29.53) | 19.45 (11.13 to 29.60) | 19.26 (11.61 to 29.91) | 18.83 (11.04 to 29.36) | 18.61 (11.30 to 27.67) | 18.01 (10.09 to 27.42) | 17.69 (10.62 to 26.75) | 17.51 (10.62 to 27.28) | 17.03 (10.11 to 26.21) |
| Angola | Female | Prevalence | 251.17 (124.85 to 640.52) | 537.03 (224.12 to 1502.76) | 697.39 (243.51 to 2101.20) | 882.12 (265.69 to 2788.53) | 1041.92 (284.41 to 3384.66) | 1157.47 (298.19 to 3814.62) | 1240.42 (308.21 to 4123.43) | 1305.98 (316.10 to 4368.17) | 1362.93 (323.14 to 4579.54) | 1410.99 (329.00 to 4758.82) | 1457.41 (334.74 to 4932.57) | 1504.88 (340.51 to 5108.74) | 1558.62 (346.47 to 5308.88) | 1585.27 (349.74 to 5407.63) | 1585.29 (350.01 to 5407.32) | 1585.28 (349.12 to 5408.31) | 1585.27 (349.81 to 5408.54) | 1585.27 (350.33 to 5407.27) | 1585.29 (350.01 to 5407.30) | 1585.29 (349.75 to 5408.42) |
| Angola | Male | DALY rates | 8.14 (4.31 to 12.82) | 38.58 (16.59 to 78.87) | 40.29 (17.91 to 84.09) | 42.14 (18.83 to 87.36) | 45.43 (19.59 to 91.49) | 46.88 (21.99 to 96.24) | 47.89 (20.25 to 101.04) | 48.74 (22.12 to 97.69) | 49.75 (23.35 to 102.00) | 49.77 (22.65 to 99.33) | 50.32 (22.90 to 98.48) | 50.03 (22.20 to 94.74) | 50.68 (22.48 to 104.60) | 50.55 (23.85 to 100.31) | 50.14 (21.13 to 97.70) | 49.34 (24.21 to 98.51) | 47.90 (21.56 to 93.51) | 47.12 (22.37 to 92.22) | 46.48 (21.35 to 92.64) | 45.49 (21.32 to 89.11) |
| Angola | Male | Prevalence | 218.05 (90.32 to 600.38) | 679.98 (291.71 to 1645.55) | 856.22 (326.21 to 2279.60) | 1060.77 (369.24 to 3006.78) | 1239.01 (413.25 to 3636.77) | 1368.65 (439.66 to 4093.53) | 1462.07 (459.88 to 4420.90) | 1536.31 (474.93 to 4679.40) | 1600.75 (488.36 to 4905.79) | 1655.43 (499.78 to 5094.98) | 1708.28 (509.92 to 5280.84) | 1762.48 (521.69 to 5465.65) | 1824.01 (534.53 to 5674.67) | 1854.55 (541.31 to 5780.48) | 1854.64 (540.86 to 5779.74) | 1854.55 (541.50 to 5779.29) | 1854.53 (541.44 to 5779.74) | 1854.54 (542.52 to 5780.21) | 1854.59 (540.92 to 5778.36) | 1854.61 (540.08 to 5779.52) |
| Bangladesh | Both | DALY rates | 9.78 (6.06 to 14.36) | 15.93 (10.16 to 24.47) | 26.72 (14.52 to 49.23) | 26.46 (14.29 to 46.64) | 26.04 (14.40 to 46.58) | 26.07 (14.01 to 46.71) | 26.26 (14.33 to 44.88) | 25.97 (14.23 to 45.96) | 25.73 (13.74 to 43.47) | 25.39 (14.62 to 46.43) | 25.75 (14.32 to 46.03) | 25.67 (13.85 to 45.74) | 25.57 (13.93 to 45.84) | 25.67 (14.41 to 46.53) | 25.04 (13.98 to 46.23) | 24.84 (13.99 to 44.77) | 24.28 (13.52 to 44.51) | 23.88 (13.39 to 41.92) | 23.75 (13.05 to 42.13) | 22.97 (13.09 to 39.76) |
| Bangladesh | Both | Prevalence | 112.11 (105.60 to 118.72) | 197.14 (185.19 to 209.95) | 301.76 (239.37 to 439.65) | 322.40 (260.10 to 458.73) | 339.65 (277.68 to 474.82) | 354.11 (290.62 to 491.49) | 365.55 (299.74 to 507.38) | 374.31 (307.14 to 518.33) | 379.81 (313.28 to 521.87) | 384.87 (318.63 to 525.39) | 392.46 (323.35 to 537.68) | 400.11 (328.90 to 549.69) | 408.50 (334.88 to 563.23) | 413.07 (337.86 to 571.54) | 413.64 (338.10 to 573.10) | 414.05 (337.87 to 574.69) | 413.86 (338.11 to 573.78) | 412.94 (337.86 to 571.60) | 415.57 (338.11 to 579.78) | 411.11 (337.07 to 564.60) |
| Bangladesh | Female | DALY rates | 11.43 (6.66 to 17.91) | 18.65 (10.67 to 29.60) | 18.72 (10.79 to 29.47) | 18.67 (11.07 to 28.42) | 18.30 (10.95 to 27.84) | 18.42 (10.65 to 28.92) | 18.17 (10.96 to 27.37) | 17.95 (10.59 to 27.62) | 17.78 (10.32 to 27.76) | 17.66 (10.20 to 27.70) | 17.64 (10.62 to 26.68) | 17.65 (10.09 to 27.17) | 17.51 (10.60 to 27.43) | 17.27 (10.46 to 26.55) | 16.92 (10.14 to 25.23) | 16.76 (10.11 to 26.41) | 16.39 (10.10 to 24.71) | 16.32 (9.79 to 24.62) | 15.90 (9.68 to 24.53) | 15.52 (9.99 to 22.56) |
| Bangladesh | Female | Prevalence | 128.84 (119.79 to 138.43) | 224.24 (208.26 to 241.32) | 243.31 (225.86 to 262.82) | 265.11 (245.92 to 287.79) | 283.64 (261.15 to 308.75) | 297.15 (273.03 to 324.73) | 306.83 (281.18 to 336.11) | 314.58 (287.68 to 344.58) | 321.13 (293.53 to 352.95) | 326.90 (298.87 to 360.03) | 332.30 (303.77 to 366.80) | 337.89 (308.48 to 373.05) | 344.30 (313.21 to 381.69) | 347.31 (316.11 to 384.29) | 347.28 (315.97 to 384.87) | 347.30 (316.55 to 384.42) | 347.30 (316.83 to 384.90) | 347.28 (315.93 to 384.89) | 347.26 (316.46 to 384.58) | 347.28 (316.08 to 385.38) |
| Bangladesh | Male | DALY rates | 8.22 (4.27 to 13.46) | 13.33 (7.11 to 21.87) | 34.65 (15.48 to 73.07) | 34.75 (14.86 to 74.69) | 34.86 (14.74 to 71.51) | 34.68 (15.91 to 73.10) | 34.95 (15.68 to 69.29) | 34.39 (14.63 to 70.90) | 34.46 (14.70 to 66.82) | 34.13 (15.87 to 73.90) | 34.36 (15.75 to 72.64) | 33.70 (14.70 to 69.52) | 33.22 (14.96 to 68.26) | 33.27 (15.66 to 68.11) | 32.26 (14.60 to 67.17) | 31.92 (14.14 to 65.84) | 31.25 (14.17 to 64.84) | 30.75 (13.62 to 64.10) | 30.30 (13.80 to 61.18) | 30.15 (14.03 to 61.11) |
| Bangladesh | Male | Prevalence | 96.25 (88.51 to 104.60) | 171.16 (157.14 to 186.70) | 359.76 (238.73 to 634.18) | 383.36 (260.73 to 660.70) | 403.43 (278.81 to 683.96) | 418.13 (293.38 to 700.85) | 428.62 (302.97 to 712.74) | 437.03 (310.43 to 721.45) | 444.21 (316.96 to 730.11) | 450.41 (323.21 to 737.40) | 456.31 (328.81 to 743.51) | 462.35 (334.10 to 750.33) | 469.37 (341.15 to 759.05) | 472.63 (343.52 to 763.05) | 472.60 (343.65 to 763.22) | 472.61 (343.74 to 763.06) | 472.61 (343.84 to 761.91) | 472.63 (343.95 to 764.38) | 472.58 (343.94 to 763.14) | 472.62 (344.05 to 762.31) |
| Benin | Both | DALY rates | 9.36 (5.58 to 14.26) | 27.84 (14.39 to 50.95) | 28.19 (15.28 to 50.46) | 28.91 (15.60 to 51.85) | 29.42 (15.45 to 51.68) | 29.94 (16.60 to 53.26) | 29.92 (16.75 to 53.16) | 29.96 (16.51 to 54.33) | 30.00 (16.11 to 54.56) | 30.41 (16.61 to 54.17) | 30.52 (17.09 to 53.58) | 30.79 (16.20 to 55.41) | 30.40 (17.21 to 53.08) | 29.75 (15.93 to 52.86) | 28.80 (16.25 to 49.72) | 27.81 (16.10 to 47.71) | 26.62 (15.15 to 44.41) | 25.44 (14.63 to 44.16) | 24.03 (14.46 to 39.88) | 22.47 (13.46 to 35.71) |
| Benin | Both | Prevalence | 177.12 (136.72 to 246.46) | 468.70 (336.04 to 682.08) | 573.19 (395.49 to 869.92) | 693.09 (467.73 to 1084.93) | 794.54 (527.06 to 1237.51) | 870.41 (572.53 to 1365.60) | 925.35 (604.10 to 1460.85) | 967.56 (628.47 to 1534.48) | 1002.96 (650.62 to 1594.73) | 1036.83 (670.08 to 1650.41) | 1069.36 (688.89 to 1707.39) | 1100.59 (707.25 to 1759.27) | 1134.29 (727.55 to 1813.64) | 1150.30 (736.05 to 1836.97) | 1147.11 (733.65 to 1830.38) | 1143.12 (730.32 to 1824.17) | 1136.92 (724.70 to 1816.54) | 1129.93 (719.01 to 1812.76) | 1122.67 (712.43 to 1808.92) | 1113.34 (706.28 to 1804.13) |
| Benin | Female | DALY rates | 10.95 (6.48 to 17.61) | 18.85 (10.58 to 29.75) | 18.69 (11.19 to 28.67) | 19.15 (10.66 to 29.56) | 19.29 (11.09 to 30.18) | 18.90 (11.26 to 28.93) | 18.86 (11.34 to 29.03) | 18.88 (11.22 to 29.77) | 18.80 (11.67 to 30.82) | 18.79 (11.17 to 29.44) | 18.82 (11.25 to 28.07) | 18.83 (10.58 to 29.71) | 18.61 (10.91 to 27.54) | 18.38 (11.03 to 28.61) | 17.98 (11.39 to 27.29) | 17.74 (10.43 to 26.79) | 17.54 (10.70 to 26.84) | 17.11 (10.53 to 25.59) | 16.76 (9.82 to 25.23) | 16.29 (10.01 to 24.55) |
| Benin | Female | Prevalence | 193.28 (151.96 to 262.27) | 402.73 (300.04 to 577.16) | 503.14 (353.45 to 756.40) | 619.31 (414.70 to 964.58) | 718.05 (466.59 to 1142.38) | 789.75 (505.43 to 1271.08) | 841.15 (533.26 to 1363.19) | 882.57 (556.54 to 1437.86) | 917.48 (574.82 to 1499.83) | 947.82 (591.49 to 1554.18) | 976.43 (607.50 to 1605.42) | 1006.03 (622.27 to 1658.35) | 1039.97 (641.25 to 1719.46) | 1056.22 (650.33 to 1748.43) | 1056.22 (650.07 to 1749.14) | 1056.24 (649.50 to 1749.29) | 1056.25 (649.69 to 1748.55) | 1056.23 (649.63 to 1748.52) | 1056.21 (649.71 to 1748.89) | 1056.23 (649.48 to 1749.04) |
| Benin | Male | DALY rates | 7.83 (4.10 to 12.85) | 36.61 (15.48 to 75.02) | 37.59 (16.97 to 78.27) | 39.07 (16.86 to 80.15) | 40.55 (16.98 to 83.90) | 41.89 (18.64 to 87.29) | 41.64 (18.31 to 89.09) | 42.10 (19.35 to 86.72) | 42.63 (19.13 to 89.50) | 42.99 (19.27 to 87.27) | 42.56 (18.48 to 86.03) | 43.11 (20.22 to 90.44) | 43.10 (20.16 to 89.29) | 42.30 (18.85 to 90.27) | 41.55 (18.70 to 84.90) | 40.68 (19.05 to 83.98) | 39.81 (18.60 to 81.73) | 39.48 (18.06 to 84.38) | 38.40 (17.49 to 79.72) | 37.71 (17.85 to 75.99) |
| Benin | Male | Prevalence | 161.57 (119.79 to 232.56) | 532.97 (358.08 to 845.60) | 642.58 (423.76 to 1028.54) | 769.94 (498.16 to 1215.73) | 878.56 (564.21 to 1381.49) | 957.80 (611.36 to 1514.17) | 1014.63 (645.28 to 1620.30) | 1060.61 (672.59 to 1701.88) | 1099.39 (695.49 to 1774.17) | 1133.17 (715.02 to 1832.65) | 1165.02 (734.66 to 1887.16) | 1198.03 (753.99 to 1947.60) | 1235.91 (775.86 to 2016.89) | 1254.11 (787.97 to 2050.34) | 1254.12 (787.42 to 2050.21) | 1254.15 (788.09 to 2049.38) | 1254.12 (787.44 to 2049.66) | 1254.16 (787.51 to 2049.34) | 1254.12 (787.87 to 2050.54) | 1254.11 (788.50 to 2050.00) |
| Brazil | Both | DALY rates | 0.51 (0.30 to 0.79) | 0.82 (0.51 to 1.21) | 0.81 (0.52 to 1.27) | 0.78 (0.48 to 1.17) | 0.75 (0.47 to 1.14) | 0.72 (0.44 to 1.05) | 0.71 (0.43 to 1.08) | 0.72 (0.45 to 1.08) | 0.71 (0.45 to 1.09) | 0.71 (0.45 to 1.06) | 0.69 (0.42 to 1.00) | 0.65 (0.40 to 0.96) | 0.62 (0.40 to 0.89) | 0.61 (0.39 to 0.88) | 0.64 (0.40 to 0.94) | 0.63 (0.40 to 0.92) | 0.63 (0.40 to 0.95) | 0.58 (0.37 to 0.87) | 0.53 (0.34 to 0.76) | 0.60 (0.37 to 0.86) |
| Brazil | Both | Prevalence | 5.20 (4.78 to 6.31) | 8.52 (7.72 to 11.17) | 8.74 (7.77 to 12.58) | 8.69 (7.55 to 13.72) | 8.51 (7.27 to 14.34) | 8.41 (7.10 to 14.77) | 8.58 (7.14 to 15.51) | 8.58 (7.09 to 15.86) | 8.63 (7.07 to 16.22) | 8.77 (7.15 to 16.74) | 8.62 (7.00 to 16.64) | 8.31 (6.71 to 16.25) | 8.13 (6.53 to 16.15) | 8.09 (6.48 to 16.17) | 8.53 (6.83 to 17.04) | 8.70 (6.96 to 17.32) | 8.79 (7.04 to 17.44) | 8.11 (6.52 to 16.07) | 7.62 (6.12 to 15.04) | 8.80 (7.06 to 17.53) |
| Brazil | Female | DALY rates | 0.60 (0.33 to 0.96) | 0.97 (0.56 to 1.55) | 0.95 (0.56 to 1.56) | 0.90 (0.53 to 1.40) | 0.87 (0.53 to 1.34) | 0.84 (0.47 to 1.30) | 0.84 (0.47 to 1.33) | 0.84 (0.50 to 1.33) | 0.83 (0.48 to 1.31) | 0.83 (0.51 to 1.34) | 0.81 (0.45 to 1.26) | 0.77 (0.42 to 1.19) | 0.73 (0.45 to 1.13) | 0.72 (0.43 to 1.12) | 0.75 (0.43 to 1.15) | 0.74 (0.43 to 1.09) | 0.75 (0.44 to 1.16) | 0.65 (0.40 to 1.02) | 0.60 (0.36 to 0.89) | 0.62 (0.37 to 0.93) |
| Brazil | Female | Prevalence | 6.08 (5.53 to 7.27) | 9.92 (8.94 to 12.70) | 10.14 (8.97 to 14.09) | 10.05 (8.70 to 15.20) | 9.86 (8.37 to 15.90) | 9.76 (8.21 to 16.41) | 10.00 (8.34 to 17.30) | 9.96 (8.23 to 17.61) | 9.98 (8.21 to 17.94) | 10.18 (8.33 to 18.57) | 10.03 (8.16 to 18.53) | 9.65 (7.81 to 18.07) | 9.43 (7.61 to 17.96) | 9.42 (7.56 to 18.05) | 9.88 (7.95 to 18.96) | 10.02 (8.05 to 19.18) | 10.13 (8.13 to 19.39) | 9.06 (7.26 to 17.34) | 8.44 (6.79 to 16.16) | 8.91 (7.16 to 17.06) |
| Brazil | Male | DALY rates | 0.43 (0.23 to 0.70) | 0.68 (0.38 to 1.10) | 0.68 (0.38 to 1.11) | 0.67 (0.34 to 1.05) | 0.62 (0.32 to 1.03) | 0.59 (0.31 to 0.96) | 0.58 (0.32 to 0.93) | 0.58 (0.32 to 0.94) | 0.59 (0.32 to 0.97) | 0.57 (0.32 to 0.91) | 0.55 (0.31 to 0.87) | 0.51 (0.29 to 0.79) | 0.49 (0.27 to 0.76) | 0.47 (0.26 to 0.75) | 0.50 (0.27 to 0.79) | 0.49 (0.28 to 0.77) | 0.47 (0.24 to 0.76) | 0.45 (0.26 to 0.69) | 0.41 (0.23 to 0.65) | 0.56 (0.31 to 0.85) |
| Brazil | Male | Prevalence | 4.37 (3.87 to 5.43) | 7.17 (6.28 to 9.72) | 7.39 (6.32 to 11.04) | 7.36 (6.14 to 12.11) | 7.18 (5.86 to 12.70) | 7.03 (5.66 to 13.02) | 7.10 (5.65 to 13.59) | 7.14 (5.64 to 13.97) | 7.21 (5.65 to 14.39) | 7.27 (5.65 to 14.75) | 7.09 (5.48 to 14.57) | 6.81 (5.22 to 14.20) | 6.63 (5.06 to 14.07) | 6.50 (4.94 to 13.91) | 6.85 (5.21 to 14.66) | 6.93 (5.27 to 14.83) | 6.83 (5.18 to 14.59) | 6.47 (4.92 to 13.85) | 5.98 (4.55 to 12.80) | 8.61 (6.54 to 18.41) |
| Brunei Darussalam | Both | DALY rates | 9.84 (5.78 to 14.76) | 28.67 (14.61 to 54.11) | 29.75 (15.81 to 53.93) | 31.02 (17.00 to 56.22) | 32.29 (16.89 to 59.42) | 32.58 (17.50 to 59.34) | 33.50 (16.89 to 60.61) | 34.08 (17.95 to 62.67) | 34.28 (18.16 to 65.14) | 33.60 (17.28 to 61.09) | 33.20 (17.03 to 60.05) | 33.04 (18.20 to 59.97) | 32.42 (17.48 to 56.93) | 32.02 (18.58 to 54.48) | 31.31 (17.05 to 55.09) | 31.25 (17.26 to 55.61) | 29.69 (15.60 to 52.42) | 25.74 (14.89 to 41.66) | 19.42 (11.94 to 28.86) | 21.87 (13.17 to 34.45) |
| Brunei Darussalam | Both | Prevalence | 204.85 (92.81 to 891.11) | 527.97 (204.06 to 2202.06) | 660.81 (211.89 to 3099.48) | 816.38 (223.55 to 4149.00) | 945.30 (232.88 to 5014.89) | 1038.39 (238.44 to 5648.99) | 1107.46 (243.59 to 6106.87) | 1162.92 (248.21 to 6470.88) | 1207.66 (251.25 to 6779.40) | 1244.12 (252.82 to 7048.15) | 1277.55 (254.32 to 7296.74) | 1317.02 (257.56 to 7563.09) | 1358.86 (259.78 to 7859.45) | 1379.77 (260.80 to 8004.85) | 1377.92 (259.96 to 8002.74) | 1381.99 (261.24 to 8006.92) | 1375.97 (259.27 to 8000.73) | 1341.92 (242.73 to 7968.47) | 1286.53 (216.83 to 7914.11) | 1312.14 (230.72 to 7939.24) |
| Brunei Darussalam | Female | DALY rates | 11.59 (6.87 to 18.10) | 19.19 (10.71 to 29.92) | 19.36 (11.36 to 30.61) | 19.47 (11.67 to 29.33) | 19.89 (11.34 to 30.61) | 19.58 (11.33 to 31.00) | 19.77 (11.58 to 30.18) | 19.68 (11.24 to 30.06) | 19.71 (11.88 to 30.59) | 19.65 (11.49 to 31.69) | 19.29 (11.20 to 29.11) | 19.01 (10.82 to 29.92) | 19.05 (11.64 to 29.11) | 18.60 (11.12 to 28.49) | 18.37 (11.14 to 28.61) | 18.15 (11.19 to 26.54) | 17.55 (10.56 to 26.50) | 17.56 (10.00 to 26.45) | 17.35 (10.38 to 26.67) | 17.04 (10.38 to 25.05) |
| Brunei Darussalam | Female | Prevalence | 221.25 (107.27 to 901.85) | 457.34 (180.93 to 2134.76) | 582.36 (184.49 to 3024.65) | 728.43 (188.23 to 4064.92) | 849.00 (190.94 to 4922.26) | 937.42 (193.89 to 5551.77) | 1001.10 (195.17 to 6004.06) | 1051.64 (196.09 to 6364.01) | 1094.61 (197.40 to 6669.69) | 1132.58 (197.94 to 6939.35) | 1167.79 (198.74 to 7189.82) | 1204.76 (200.06 to 7453.70) | 1246.40 (200.94 to 7749.31) | 1266.96 (201.61 to 7894.29) | 1266.96 (201.51 to 7894.72) | 1266.95 (201.54 to 7895.22) | 1266.95 (201.03 to 7895.14) | 1266.93 (200.96 to 7895.05) | 1266.95 (201.37 to 7894.90) | 1266.94 (201.22 to 7895.21) |
| Brunei Darussalam | Male | DALY rates | 8.22 (4.35 to 13.70) | 37.50 (15.47 to 79.15) | 39.08 (17.52 to 82.88) | 41.05 (17.94 to 83.20) | 42.79 (18.68 to 89.38) | 43.82 (20.11 to 90.72) | 45.08 (19.35 to 95.74) | 45.84 (20.91 to 94.51) | 46.49 (21.39 to 96.61) | 46.26 (19.55 to 97.22) | 46.84 (20.38 to 95.80) | 46.79 (21.55 to 95.28) | 46.13 (20.83 to 90.86) | 46.05 (22.29 to 91.35) | 45.27 (20.33 to 89.50) | 44.42 (19.97 to 91.35) | 43.24 (19.78 to 88.84) | 42.75 (20.12 to 85.05) | 41.75 (19.20 to 85.08) | 41.70 (19.98 to 80.95) |
| Brunei Darussalam | Male | Prevalence | 189.67 (76.40 to 881.17) | 593.73 (210.69 to 2264.71) | 731.18 (222.48 to 3166.58) | 892.79 (238.05 to 4222.06) | 1026.86 (250.73 to 5093.33) | 1125.70 (261.13 to 5733.05) | 1197.11 (268.06 to 6193.52) | 1253.85 (274.15 to 6558.20) | 1302.39 (278.55 to 6871.33) | 1345.31 (282.45 to 7146.86) | 1385.16 (287.60 to 7401.56) | 1427.02 (290.56 to 7670.29) | 1474.24 (296.11 to 7972.45) | 1497.65 (298.12 to 8120.38) | 1497.66 (297.76 to 8119.28) | 1497.67 (298.15 to 8119.25) | 1497.65 (297.99 to 8118.60) | 1497.68 (297.46 to 8120.97) | 1497.64 (297.74 to 8121.24) | 1497.62 (297.67 to 8119.96) |
| Burkina Faso | Both | DALY rates | 9.10 (5.66 to 14.08) | 27.13 (15.16 to 49.58) | 27.51 (15.20 to 48.96) | 27.96 (14.65 to 50.21) | 27.88 (14.94 to 48.85) | 27.90 (15.57 to 50.86) | 28.49 (15.67 to 51.04) | 28.63 (15.43 to 53.14) | 29.14 (15.73 to 51.61) | 29.41 (15.54 to 54.49) | 29.14 (15.91 to 52.29) | 28.90 (15.39 to 51.83) | 28.88 (15.71 to 52.49) | 28.50 (15.74 to 49.50) | 27.65 (15.70 to 48.85) | 26.96 (15.31 to 47.29) | 25.97 (14.75 to 44.27) | 24.84 (14.21 to 42.50) | 23.73 (13.74 to 39.28) | 22.06 (13.10 to 35.47) |
| Burkina Faso | Both | Prevalence | 154.06 (110.09 to 277.77) | 412.65 (270.59 to 736.97) | 491.43 (304.02 to 945.88) | 581.51 (339.17 to 1177.01) | 655.41 (365.57 to 1393.71) | 711.20 (388.17 to 1553.39) | 754.92 (405.47 to 1668.67) | 789.76 (419.35 to 1761.79) | 819.20 (430.53 to 1839.81) | 844.30 (440.55 to 1907.83) | 866.14 (448.67 to 1971.21) | 889.08 (456.67 to 2036.22) | 916.29 (466.60 to 2111.37) | 929.28 (471.94 to 2147.56) | 928.72 (471.59 to 2147.87) | 925.65 (469.22 to 2145.64) | 920.90 (464.89 to 2142.89) | 915.31 (461.17 to 2140.29) | 909.02 (457.83 to 2135.72) | 898.69 (450.04 to 2130.36) |
| Burkina Faso | Female | DALY rates | 10.70 (6.10 to 17.19) | 18.65 (11.24 to 29.93) | 18.74 (11.52 to 28.59) | 18.89 (10.95 to 30.04) | 18.80 (10.40 to 29.78) | 18.74 (11.38 to 28.72) | 18.77 (10.70 to 28.68) | 18.47 (10.87 to 28.99) | 18.88 (11.14 to 29.22) | 18.80 (10.79 to 28.88) | 18.50 (10.41 to 28.93) | 18.40 (10.74 to 28.27) | 18.52 (11.02 to 28.44) | 18.17 (10.39 to 27.39) | 17.78 (9.77 to 27.59) | 17.62 (10.95 to 26.60) | 17.38 (10.41 to 27.09) | 17.03 (9.90 to 25.94) | 16.61 (9.97 to 25.46) | 16.15 (9.52 to 24.53) |
| Burkina Faso | Female | Prevalence | 169.90 (125.67 to 295.06) | 349.62 (237.21 to 668.41) | 425.79 (262.47 to 888.45) | 513.69 (293.25 to 1143.28) | 588.56 (319.38 to 1359.42) | 643.43 (336.92 to 1517.61) | 682.56 (350.06 to 1629.98) | 713.81 (360.35 to 1720.74) | 740.23 (369.37 to 1796.75) | 763.32 (377.05 to 1863.64) | 785.04 (383.80 to 1926.51) | 807.54 (392.51 to 1991.00) | 833.18 (400.81 to 2064.99) | 845.53 (404.37 to 2100.91) | 845.55 (404.63 to 2101.53) | 845.54 (403.80 to 2101.15) | 845.55 (404.39 to 2100.96) | 845.55 (404.80 to 2101.28) | 845.55 (403.98 to 2100.00) | 845.57 (404.40 to 2100.62) |
| Burkina Faso | Male | DALY rates | 7.54 (3.89 to 12.81) | 35.55 (15.84 to 76.54) | 36.43 (16.10 to 77.02) | 37.68 (15.62 to 78.27) | 38.87 (17.02 to 77.54) | 39.44 (18.18 to 83.21) | 39.87 (17.83 to 82.95) | 39.91 (17.30 to 83.00) | 40.06 (17.76 to 80.19) | 40.48 (17.91 to 87.07) | 40.51 (17.67 to 84.53) | 40.30 (18.59 to 83.05) | 40.07 (17.77 to 83.38) | 39.65 (18.19 to 79.25) | 38.47 (17.60 to 76.08) | 37.93 (17.25 to 78.62) | 37.25 (16.78 to 73.43) | 36.52 (16.30 to 75.58) | 36.17 (16.00 to 72.04) | 35.54 (16.61 to 72.24) |
| Burkina Faso | Male | Prevalence | 138.63 (94.02 to 260.30) | 475.22 (278.64 to 863.11) | 558.21 (319.79 to 1024.67) | 654.22 (366.21 to 1292.34) | 736.28 (402.92 to 1532.57) | 796.62 (428.61 to 1668.98) | 839.62 (447.68 to 1765.95) | 874.04 (462.17 to 1861.73) | 903.24 (475.07 to 1942.38) | 928.76 (485.43 to 2008.40) | 952.75 (494.82 to 2063.36) | 977.63 (505.14 to 2117.93) | 1006.08 (518.34 to 2183.51) | 1019.74 (524.13 to 2214.69) | 1019.79 (523.66 to 2216.33) | 1019.77 (523.78 to 2215.47) | 1019.80 (523.84 to 2215.47) | 1019.76 (524.03 to 2214.76) | 1019.75 (524.15 to 2213.05) | 1019.74 (523.91 to 2214.71) |
| Cambodia | Both | DALY rates | 0.00 (0.00 to 0.00) | 0.00 (0.00 to 0.00) | 0.00 (0.00 to 0.00) | 0.00 (0.00 to 0.00) | 0.00 (0.00 to 0.00) | 0.00 (0.00 to 0.00) | 0.00 (0.00 to 0.00) | 0.00 (0.00 to 0.00) | 0.00 (0.00 to 0.00) | 0.00 (0.00 to 0.00) | 0.00 (0.00 to 0.00) | 0.00 (0.00 to 0.00) | 0.00 (0.00 to 0.00) | 0.00 (0.00 to 0.00) | 0.00 (0.00 to 0.00) | 0.00 (0.00 to 0.00) | 0.00 (0.00 to 0.00) | 0.00 (0.00 to 0.00) | 0.00 (0.00 to 0.00) | 0.00 (0.00 to 0.00) |
| Cambodia | Both | Prevalence | 19.58 (0.33 to 127.19) | 48.18 (0.81 to 312.89) | 70.09 (1.18 to 455.19) | 95.33 (1.61 to 619.13) | 116.94 (1.98 to 759.44) | 132.55 (2.24 to 860.81) | 143.62 (2.43 to 932.72) | 152.54 (2.58 to 990.64) | 160.07 (2.71 to 1039.54) | 166.83 (2.82 to 1083.45) | 173.10 (2.93 to 1124.18) | 179.46 (3.03 to 1165.48) | 186.82 (3.16 to 1213.28) | 190.35 (3.22 to 1236.19) | 190.35 (3.22 to 1236.19) | 190.35 (3.22 to 1236.19) | 190.35 (3.22 to 1236.19) | 190.35 (3.22 to 1236.19) | 190.35 (3.22 to 1236.19) | 190.35 (3.22 to 1236.19) |
| Cambodia | Female | DALY rates | 0.00 (0.00 to 0.00) | 0.00 (0.00 to 0.00) | 0.00 (0.00 to 0.00) | 0.00 (0.00 to 0.00) | 0.00 (0.00 to 0.00) | 0.00 (0.00 to 0.00) | 0.00 (0.00 to 0.00) | 0.00 (0.00 to 0.00) | 0.00 (0.00 to 0.00) | 0.00 (0.00 to 0.00) | 0.00 (0.00 to 0.00) | 0.00 (0.00 to 0.00) | 0.00 (0.00 to 0.00) | 0.00 (0.00 to 0.00) | 0.00 (0.00 to 0.00) | 0.00 (0.00 to 0.00) | 0.00 (0.00 to 0.00) | 0.00 (0.00 to 0.00) | 0.00 (0.00 to 0.00) | 0.00 (0.00 to 0.00) |
| Cambodia | Female | Prevalence | 19.58 (0.33 to 127.14) | 48.18 (0.81 to 312.89) | 70.09 (1.18 to 455.19) | 95.33 (1.61 to 619.13) | 116.94 (1.98 to 759.44) | 132.55 (2.24 to 860.81) | 143.62 (2.43 to 932.72) | 152.54 (2.58 to 990.64) | 160.07 (2.71 to 1039.54) | 166.83 (2.82 to 1083.45) | 173.10 (2.93 to 1124.18) | 179.46 (3.03 to 1165.48) | 186.82 (3.16 to 1213.28) | 190.35 (3.22 to 1236.19) | 190.35 (3.22 to 1236.19) | 190.35 (3.22 to 1236.19) | 190.35 (3.22 to 1236.19) | 190.35 (3.22 to 1236.19) | 190.35 (3.22 to 1236.19) | 190.35 (3.22 to 1236.19) |
| Cambodia | Male | DALY rates | 0.00 (0.00 to 0.00) | 0.00 (0.00 to 0.00) | 0.00 (0.00 to 0.00) | 0.00 (0.00 to 0.00) | 0.00 (0.00 to 0.00) | 0.00 (0.00 to 0.00) | 0.00 (0.00 to 0.00) | 0.00 (0.00 to 0.00) | 0.00 (0.00 to 0.00) | 0.00 (0.00 to 0.00) | 0.00 (0.00 to 0.00) | 0.00 (0.00 to 0.00) | 0.00 (0.00 to 0.00) | 0.00 (0.00 to 0.00) | 0.00 (0.00 to 0.00) | 0.00 (0.00 to 0.00) | 0.00 (0.00 to 0.00) | 0.00 (0.00 to 0.00) | 0.00 (0.00 to 0.00) | 0.00 (0.00 to 0.00) |
| Cambodia | Male | Prevalence | 19.59 (0.33 to 127.24) | 48.18 (0.81 to 312.89) | 70.09 (1.18 to 455.19) | 95.33 (1.61 to 619.13) | 116.94 (1.98 to 759.44) | 132.55 (2.24 to 860.81) | 143.62 (2.43 to 932.72) | 152.54 (2.58 to 990.64) | 160.07 (2.71 to 1039.54) | 166.83 (2.82 to 1083.45) | 173.10 (2.93 to 1124.18) | 179.46 (3.03 to 1165.48) | 186.82 (3.16 to 1213.28) | 190.35 (3.22 to 1236.19) | 190.35 (3.22 to 1236.19) | 190.35 (3.22 to 1236.19) | 190.35 (3.22 to 1236.19) | 190.35 (3.22 to 1236.19) | 190.35 (3.22 to 1236.19) | 190.35 (3.22 to 1236.19) |
| Cameroon | Both | DALY rates | 9.49 (5.76 to 14.23) | 15.93 (9.59 to 24.69) | 26.66 (14.21 to 46.99) | 26.45 (14.43 to 49.27) | 26.25 (14.15 to 46.44) | 26.06 (13.95 to 47.88) | 25.94 (13.99 to 43.72) | 25.96 (14.08 to 45.75) | 26.19 (14.65 to 47.47) | 25.91 (13.64 to 48.61) | 25.79 (14.11 to 44.72) | 25.52 (13.66 to 46.40) | 25.28 (13.52 to 45.97) | 24.75 (13.31 to 42.86) | 24.53 (13.16 to 42.42) | 23.77 (13.55 to 41.61) | 22.69 (12.85 to 39.19) | 21.77 (11.96 to 36.56) | 20.82 (12.07 to 34.16) | 19.44 (11.98 to 31.63) |
| Cameroon | Both | Prevalence | 106.19 (94.64 to 130.40) | 190.50 (164.83 to 249.49) | 292.25 (212.75 to 444.10) | 310.50 (222.71 to 461.49) | 325.54 (232.09 to 492.24) | 336.94 (238.20 to 517.98) | 345.56 (242.65 to 548.68) | 352.91 (247.30 to 549.65) | 359.75 (250.47 to 563.14) | 365.51 (253.11 to 575.32) | 369.54 (255.44 to 587.28) | 373.82 (258.24 to 598.37) | 379.67 (260.34 to 612.19) | 381.87 (261.14 to 618.52) | 381.01 (260.82 to 616.02) | 378.55 (260.64 to 612.88) | 374.17 (259.28 to 606.41) | 370.26 (257.83 to 601.05) | 364.98 (255.83 to 583.85) | 357.31 (253.22 to 566.98) |
| Cameroon | Female | DALY rates | 11.12 (6.32 to 17.32) | 18.58 (11.15 to 29.75) | 18.34 (10.38 to 28.11) | 18.54 (10.37 to 30.42) | 18.39 (10.51 to 28.86) | 18.20 (10.61 to 28.45) | 18.21 (10.18 to 28.03) | 18.01 (10.73 to 27.19) | 18.04 (10.67 to 28.54) | 17.72 (10.03 to 27.93) | 17.88 (10.74 to 28.54) | 17.71 (10.40 to 27.08) | 17.54 (9.96 to 26.20) | 17.24 (9.79 to 27.14) | 17.13 (10.22 to 26.89) | 17.01 (10.77 to 26.32) | 16.35 (9.96 to 24.46) | 16.04 (9.50 to 25.85) | 15.88 (9.69 to 24.20) | 15.44 (9.42 to 23.58) |
| Cameroon | Female | Prevalence | 122.33 (109.63 to 146.33) | 217.54 (189.91 to 277.31) | 233.52 (197.09 to 319.80) | 251.89 (204.69 to 367.46) | 267.59 (211.21 to 407.48) | 279.10 (214.88 to 437.80) | 287.24 (217.81 to 459.64) | 293.77 (219.94 to 476.68) | 299.31 (221.62 to 490.87) | 304.15 (223.94 to 503.77) | 308.72 (224.83 to 516.18) | 313.44 (226.88 to 528.38) | 318.81 (228.84 to 542.83) | 321.42 (229.83 to 549.26) | 321.40 (229.44 to 549.27) | 321.43 (229.62 to 549.42) | 321.40 (229.53 to 549.36) | 321.40 (229.98 to 549.47) | 321.41 (229.57 to 549.29) | 321.40 (229.07 to 549.44) |
| Cameroon | Male | DALY rates | 7.93 (4.47 to 12.55) | 13.38 (7.19 to 22.14) | 34.72 (15.36 to 71.24) | 34.35 (15.90 to 75.65) | 34.46 (15.34 to 71.72) | 34.42 (15.20 to 73.11) | 34.14 (15.25 to 69.05) | 34.25 (14.76 to 69.68) | 34.38 (15.76 to 72.20) | 33.96 (14.92 to 74.14) | 33.76 (14.66 to 70.83) | 33.54 (14.17 to 71.64) | 33.19 (13.77 to 71.97) | 32.52 (14.77 to 65.14) | 32.41 (14.45 to 67.69) | 31.59 (14.72 to 67.50) | 31.14 (14.26 to 65.49) | 30.48 (13.95 to 61.97) | 29.84 (13.75 to 61.30) | 29.16 (13.84 to 62.75) |
| Cameroon | Male | Prevalence | 90.73 (78.38 to 114.56) | 164.49 (137.59 to 221.32) | 349.17 (215.56 to 622.68) | 369.06 (227.25 to 665.68) | 386.07 (237.45 to 680.78) | 398.54 (245.79 to 701.04) | 407.43 (251.53 to 716.25) | 414.55 (255.74 to 728.81) | 420.54 (258.65 to 735.42) | 425.82 (261.74 to 744.11) | 430.78 (264.47 to 749.51) | 435.88 (267.17 to 752.29) | 441.78 (269.63 to 755.38) | 444.52 (270.69 to 760.11) | 444.51 (270.86 to 760.11) | 444.55 (271.33 to 759.86) | 444.55 (271.25 to 760.40) | 444.52 (271.40 to 759.85) | 444.59 (271.57 to 759.16) | 444.52 (271.35 to 757.79) |
| Central African Republic | Both | DALY rates | 10.03 (6.43 to 15.12) | 39.73 (21.23 to 72.67) | 48.16 (24.71 to 87.19) | 59.83 (32.24 to 101.53) | 70.43 (39.60 to 117.10) | 79.07 (44.87 to 132.63) | 86.04 (49.28 to 138.62) | 94.26 (54.76 to 157.05) | 101.54 (58.58 to 165.84) | 109.51 (62.35 to 176.20) | 113.90 (67.74 to 181.72) | 115.19 (69.50 to 186.25) | 115.43 (70.19 to 179.21) | 110.64 (68.62 to 167.80) | 102.34 (63.77 to 158.88) | 93.11 (57.84 to 141.95) | 84.33 (54.96 to 126.65) | 79.82 (50.00 to 119.64) | 77.70 (50.18 to 118.09) | 71.33 (47.13 to 105.53) |
| Central African Republic | Both | Prevalence | 693.29 (179.91 to 2198.30) | 1859.17 (520.41 to 5630.47) | 2621.95 (680.38 to 8138.28) | 3512.52 (883.01 to 11014.47) | 4275.74 (1063.53 to 13464.21) | 4838.24 (1192.33 to 15247.04) | 5258.04 (1294.92 to 16561.06) | 5613.10 (1398.62 to 17618.19) | 5919.80 (1493.61 to 18516.31) | 6202.09 (1590.64 to 19311.71) | 6444.76 (1656.76 to 20041.47) | 6670.94 (1713.61 to 20776.11) | 6916.69 (1776.78 to 21605.20) | 7005.20 (1781.02 to 21980.99) | 6946.58 (1740.01 to 21930.98) | 6872.27 (1687.16 to 21867.09) | 6808.99 (1633.60 to 21812.95) | 6781.16 (1610.38 to 21789.57) | 6774.68 (1603.46 to 21785.44) | 6735.39 (1568.49 to 21750.36) |
| Central African Republic | Female | DALY rates | 11.88 (7.04 to 18.70) | 20.86 (11.92 to 32.06) | 22.18 (13.00 to 35.71) | 23.48 (12.87 to 35.31) | 24.57 (15.32 to 37.61) | 25.42 (14.76 to 39.44) | 25.91 (16.13 to 40.05) | 26.01 (15.42 to 40.01) | 26.45 (16.38 to 40.63) | 26.60 (16.86 to 39.79) | 26.92 (17.05 to 40.54) | 27.33 (17.09 to 42.23) | 27.43 (17.24 to 42.26) | 27.78 (18.07 to 41.31) | 26.94 (16.92 to 40.63) | 26.98 (16.65 to 39.52) | 25.95 (17.01 to 38.07) | 25.65 (16.28 to 36.75) | 25.05 (15.78 to 36.36) | 24.44 (16.41 to 34.65) |
| Central African Republic | Female | Prevalence | 711.81 (199.11 to 2219.22) | 1713.92 (416.21 to 5524.66) | 2416.23 (522.50 to 7972.22) | 3220.97 (646.36 to 10773.96) | 3904.22 (751.45 to 13151.49) | 4401.50 (828.82 to 14879.63) | 4764.77 (885.19 to 16142.80) | 5052.61 (930.62 to 17142.24) | 5296.05 (969.57 to 17988.17) | 5508.18 (1003.19 to 18725.12) | 5707.83 (1034.32 to 19416.15) | 5913.35 (1065.92 to 20129.37) | 6150.17 (1103.13 to 20951.66) | 6264.60 (1121.06 to 21348.87) | 6264.68 (1121.13 to 21347.72) | 6264.62 (1121.11 to 21347.20) | 6264.67 (1121.63 to 21347.09) | 6264.64 (1122.29 to 21347.78) | 6264.62 (1121.16 to 21347.97) | 6264.61 (1121.96 to 21347.95) |
| Central African Republic | Male | DALY rates | 8.25 (4.68 to 13.07) | 58.16 (28.15 to 118.49) | 73.86 (33.78 to 143.63) | 96.93 (47.56 to 178.56) | 120.69 (63.11 to 211.72) | 140.73 (74.85 to 247.09) | 156.08 (83.43 to 262.41) | 169.38 (92.43 to 286.56) | 179.93 (97.60 to 305.74) | 189.53 (103.35 to 314.42) | 198.07 (112.81 to 323.32) | 206.14 (121.13 to 343.53) | 217.02 (128.16 to 347.64) | 218.57 (129.97 to 341.04) | 215.55 (128.68 to 349.39) | 212.59 (123.27 to 340.28) | 208.86 (127.09 to 331.55) | 204.50 (119.33 to 321.26) | 201.10 (121.91 to 314.65) | 194.31 (121.31 to 304.42) |
| Central African Republic | Male | Prevalence | 675.46 (165.27 to 2178.17) | 2001.03 (636.83 to 5693.83) | 2825.49 (844.10 to 8245.89) | 3810.20 (1125.70 to 11204.97) | 4682.91 (1406.36 to 13747.86) | 5340.21 (1633.86 to 15620.01) | 5832.66 (1811.49 to 16998.99) | 6229.97 (1966.83 to 18099.27) | 6571.08 (2104.22 to 19036.19) | 6871.80 (2226.96 to 19853.93) | 7157.77 (2345.77 to 20627.72) | 7455.20 (2464.98 to 21425.41) | 7801.49 (2617.08 to 22359.00) | 7969.92 (2694.97 to 22804.40) | 7970.43 (2691.41 to 22806.71) | 7970.13 (2691.31 to 22806.36) | 7970.07 (2698.37 to 22806.68) | 7970.17 (2695.74 to 22806.55) | 7970.26 (2691.43 to 22808.61) | 7970.07 (2693.07 to 22805.70) |
| Chad | Both | DALY rates | 9.08 (5.61 to 13.65) | 27.67 (14.78 to 49.43) | 28.41 (14.61 to 51.17) | 28.72 (15.64 to 52.10) | 29.26 (15.58 to 51.64) | 29.40 (16.62 to 53.41) | 29.48 (15.63 to 52.55) | 29.61 (15.78 to 51.38) | 30.10 (16.19 to 54.03) | 30.33 (16.56 to 58.20) | 31.02 (16.52 to 55.79) | 31.51 (16.70 to 57.73) | 32.01 (17.17 to 57.42) | 31.77 (16.98 to 58.84) | 31.31 (16.42 to 56.91) | 30.31 (15.83 to 54.77) | 29.62 (16.36 to 52.54) | 28.16 (15.66 to 51.27) | 26.25 (14.41 to 48.51) | 24.81 (14.50 to 40.51) |
| Chad | Both | Prevalence | 172.33 (99.71 to 411.98) | 465.27 (249.04 to 1118.90) | 568.01 (268.15 to 1535.43) | 686.49 (293.07 to 2011.81) | 786.15 (312.72 to 2396.84) | 859.67 (324.51 to 2681.64) | 911.35 (334.01 to 2883.30) | 954.06 (342.38 to 3046.49) | 993.11 (351.02 to 3190.23) | 1028.09 (359.20 to 3315.27) | 1062.70 (367.28 to 3435.82) | 1097.59 (375.34 to 3559.19) | 1136.05 (383.84 to 3697.93) | 1155.13 (388.74 to 3765.65) | 1155.51 (388.73 to 3766.16) | 1152.85 (387.60 to 3762.06) | 1148.60 (384.62 to 3756.05) | 1141.84 (381.61 to 3746.44) | 1131.33 (377.04 to 3732.29) | 1123.16 (372.87 to 3721.02) |
| Chad | Female | DALY rates | 10.64 (6.22 to 16.62) | 19.01 (11.08 to 29.32) | 19.15 (10.83 to 30.31) | 19.01 (10.28 to 28.99) | 19.08 (10.89 to 29.40) | 18.91 (11.64 to 28.60) | 18.87 (10.56 to 29.21) | 18.86 (10.77 to 29.12) | 18.87 (10.83 to 29.40) | 18.51 (10.70 to 27.93) | 18.58 (11.32 to 27.73) | 18.89 (10.91 to 29.12) | 18.72 (11.28 to 28.47) | 18.24 (10.78 to 26.71) | 18.14 (11.27 to 27.84) | 17.49 (10.50 to 26.28) | 17.56 (10.47 to 26.67) | 16.92 (9.66 to 25.99) | 16.38 (9.78 to 24.87) | 16.21 (9.20 to 24.63) |
| Chad | Female | Prevalence | 188.24 (114.57 to 430.46) | 399.48 (212.57 to 1026.30) | 498.40 (228.10 to 1411.26) | 612.56 (247.39 to 1858.14) | 709.35 (261.60 to 2235.33) | 780.91 (273.18 to 2513.78) | 831.68 (281.16 to 2711.50) | 872.45 (287.02 to 2870.16) | 906.90 (293.66 to 3005.04) | 936.88 (297.93 to 3121.75) | 965.07 (303.17 to 3231.39) | 994.27 (307.07 to 3345.48) | 1027.65 (312.72 to 3476.23) | 1043.67 (315.60 to 3538.33) | 1043.67 (315.08 to 3538.02) | 1043.70 (315.79 to 3537.72) | 1043.69 (315.27 to 3538.40) | 1043.66 (315.02 to 3538.11) | 1043.68 (314.99 to 3538.32) | 1043.64 (314.95 to 3538.87) |
| Chad | Male | DALY rates | 7.58 (4.21 to 11.86) | 36.13 (15.52 to 73.95) | 37.64 (16.35 to 79.67) | 38.72 (17.10 to 82.23) | 40.26 (16.70 to 85.89) | 41.18 (19.18 to 85.47) | 41.86 (18.00 to 86.06) | 42.19 (17.63 to 88.74) | 42.44 (17.89 to 86.31) | 42.41 (18.31 to 93.21) | 42.45 (18.65 to 86.75) | 42.20 (18.29 to 86.12) | 42.60 (19.23 to 83.60) | 42.10 (19.48 to 87.89) | 41.29 (19.07 to 81.43) | 40.58 (18.19 to 83.22) | 40.15 (18.67 to 79.80) | 39.44 (18.75 to 80.78) | 38.53 (17.22 to 82.24) | 37.48 (17.11 to 72.73) |
| Chad | Male | Prevalence | 157.04 (84.93 to 394.21) | 529.50 (270.18 to 1158.98) | 637.48 (299.05 to 1575.37) | 762.58 (327.50 to 2043.77) | 869.12 (352.17 to 2454.10) | 948.09 (370.56 to 2758.45) | 1004.28 (383.97 to 2975.19) | 1049.56 (394.85 to 3147.75) | 1087.82 (404.34 to 3294.65) | 1121.22 (412.42 to 3422.60) | 1152.52 (418.78 to 3541.72) | 1185.10 (427.32 to 3666.18) | 1222.39 (436.49 to 3808.39) | 1240.30 (440.34 to 3876.55) | 1240.31 (440.62 to 3876.76) | 1240.28 (440.29 to 3875.81) | 1240.30 (440.41 to 3876.57) | 1240.33 (440.70 to 3876.33) | 1240.34 (441.12 to 3877.55) | 1240.35 (440.01 to 3876.79) |
| Comoros | Both | DALY rates | 10.20 (6.27 to 15.86) | 37.64 (20.08 to 67.06) | 44.64 (24.16 to 79.38) | 53.89 (28.36 to 96.53) | 63.21 (34.58 to 108.45) | 71.04 (38.31 to 118.15) | 77.84 (42.33 to 134.91) | 82.97 (45.40 to 138.81) | 87.69 (49.58 to 142.90) | 90.65 (53.08 to 150.00) | 93.59 (53.03 to 152.86) | 95.43 (55.48 to 157.08) | 94.97 (56.18 to 156.10) | 93.91 (55.71 to 145.96) | 91.10 (55.19 to 145.68) | 89.28 (52.62 to 140.54) | 88.61 (53.35 to 139.19) | 86.15 (51.01 to 136.46) | 82.43 (51.00 to 127.88) | 74.76 (46.24 to 115.70) |
| Comoros | Both | Prevalence | 622.30 (108.47 to 2981.07) | 1626.94 (312.85 to 7515.81) | 2273.64 (379.66 to 10831.48) | 3029.41 (467.49 to 14653.61) | 3683.68 (552.51 to 17913.52) | 4170.55 (620.67 to 20304.38) | 4523.93 (677.03 to 22010.07) | 4813.26 (727.25 to 23392.63) | 5058.87 (771.17 to 24565.20) | 5272.58 (810.39 to 25582.95) | 5468.00 (839.60 to 26532.28) | 5661.60 (866.26 to 27501.29) | 5874.30 (885.16 to 28611.43) | 5968.22 (890.96 to 29127.59) | 5960.22 (883.22 to 29118.64) | 5958.93 (879.25 to 29114.69) | 5968.19 (890.19 to 29125.91) | 5962.69 (885.58 to 29117.77) | 5940.55 (864.17 to 29092.41) | 5883.60 (816.96 to 29025.16) |
| Comoros | Female | DALY rates | 12.03 (7.06 to 19.42) | 20.93 (12.42 to 32.98) | 21.89 (13.08 to 34.68) | 22.79 (13.61 to 34.68) | 23.51 (13.88 to 35.48) | 24.29 (15.27 to 36.92) | 24.88 (15.03 to 38.47) | 25.23 (15.33 to 37.20) | 25.56 (15.20 to 40.61) | 25.89 (15.81 to 39.52) | 25.80 (15.67 to 38.72) | 25.79 (15.67 to 37.49) | 26.12 (16.26 to 39.08) | 26.06 (15.93 to 39.51) | 25.61 (16.02 to 38.72) | 25.05 (15.68 to 36.87) | 24.76 (15.80 to 36.56) | 24.25 (15.17 to 37.15) | 23.96 (15.84 to 36.96) | 23.06 (15.01 to 32.65) |
| Comoros | Female | Prevalence | 640.09 (126.40 to 3002.73) | 1496.41 (225.66 to 7338.61) | 2095.23 (245.52 to 10595.42) | 2783.01 (269.00 to 14332.81) | 3367.92 (289.47 to 17509.19) | 3795.30 (303.55 to 19828.29) | 4100.34 (314.44 to 21483.12) | 4346.41 (323.80 to 22817.75) | 4554.78 (331.81 to 23947.64) | 4736.06 (338.93 to 24929.21) | 4905.86 (345.12 to 25850.05) | 5081.85 (352.11 to 26803.15) | 5285.11 (360.15 to 27903.41) | 5381.38 (363.99 to 28425.50) | 5381.42 (363.98 to 28425.08) | 5381.40 (363.64 to 28425.56) | 5381.40 (364.03 to 28425.79) | 5381.40 (363.91 to 28424.61) | 5381.45 (363.74 to 28425.00) | 5381.39 (364.09 to 28425.02) |
| Comoros | Male | DALY rates | 8.44 (4.77 to 13.63) | 53.72 (24.77 to 109.17) | 66.73 (31.62 to 132.56) | 84.51 (40.28 to 160.43) | 102.92 (51.53 to 184.52) | 118.00 (57.18 to 210.58) | 130.73 (66.06 to 237.80) | 140.04 (71.85 to 242.44) | 148.91 (80.68 to 253.54) | 154.67 (86.77 to 267.18) | 162.23 (87.40 to 277.82) | 169.94 (92.89 to 287.67) | 176.01 (96.56 to 298.27) | 179.01 (99.85 to 294.32) | 175.30 (98.89 to 293.64) | 172.15 (95.80 to 281.51) | 168.68 (93.64 to 277.94) | 165.10 (92.71 to 273.77) | 162.28 (95.44 to 267.70) | 159.24 (91.02 to 261.15) |
| Comoros | Male | Prevalence | 605.28 (90.61 to 2960.34) | 1752.60 (375.38 to 7686.41) | 2446.90 (479.65 to 11060.74) | 3272.11 (628.40 to 14969.58) | 3999.62 (774.91 to 18318.07) | 4547.49 (891.91 to 20782.60) | 4947.01 (977.64 to 22536.38) | 5274.64 (1060.24 to 23960.78) | 5555.55 (1137.14 to 25173.68) | 5802.93 (1205.73 to 26229.17) | 6037.12 (1274.64 to 27222.97) | 6281.91 (1352.74 to 28249.96) | 6567.80 (1442.18 to 29455.81) | 6704.16 (1487.68 to 30025.93) | 6704.36 (1483.84 to 30026.25) | 6704.10 (1481.67 to 30022.33) | 6704.16 (1484.91 to 30021.97) | 6704.01 (1485.59 to 30020.56) | 6704.26 (1485.27 to 30026.23) | 6704.21 (1487.20 to 30024.53) |
| Congo | Both | DALY rates | 9.91 (5.92 to 15.11) | 28.31 (15.33 to 50.73) | 28.89 (15.78 to 52.83) | 29.64 (15.65 to 52.89) | 30.60 (17.09 to 54.81) | 30.96 (16.55 to 56.56) | 31.07 (16.66 to 57.13) | 31.47 (17.66 to 55.23) | 31.76 (17.18 to 56.73) | 32.80 (17.51 to 61.25) | 33.15 (17.94 to 62.04) | 32.80 (17.84 to 58.83) | 32.25 (18.13 to 60.48) | 31.29 (17.12 to 54.85) | 30.19 (17.19 to 51.15) | 29.59 (16.28 to 51.41) | 28.32 (15.79 to 49.54) | 27.56 (15.74 to 48.46) | 26.88 (15.39 to 46.64) | 25.71 (15.10 to 42.41) |
| Congo | Both | Prevalence | 206.92 (110.58 to 474.87) | 525.95 (251.13 to 1202.76) | 655.74 (274.41 to 1620.05) | 805.02 (302.87 to 2103.23) | 932.85 (327.59 to 2518.80) | 1027.05 (345.63 to 2825.89) | 1093.48 (357.69 to 3045.00) | 1147.82 (369.08 to 3221.73) | 1196.76 (380.05 to 3373.05) | 1241.00 (390.16 to 3507.11) | 1280.44 (398.48 to 3630.86) | 1317.91 (405.33 to 3755.25) | 1357.77 (411.82 to 3894.17) | 1373.56 (412.37 to 3957.70) | 1369.62 (410.63 to 3953.19) | 1367.90 (410.21 to 3950.88) | 1364.42 (408.45 to 3947.67) | 1361.59 (407.19 to 3944.91) | 1358.34 (405.98 to 3940.91) | 1352.16 (402.85 to 3934.33) |
| Congo | Female | DALY rates | 11.63 (6.36 to 18.94) | 19.32 (11.56 to 30.59) | 19.17 (10.71 to 30.39) | 19.31 (10.49 to 30.32) | 19.40 (10.78 to 30.79) | 19.13 (11.06 to 30.03) | 19.08 (10.80 to 29.48) | 19.04 (11.53 to 28.71) | 18.77 (11.41 to 28.36) | 18.99 (10.86 to 29.57) | 18.95 (11.16 to 28.73) | 18.68 (11.12 to 28.64) | 18.53 (11.05 to 28.04) | 18.63 (11.46 to 27.61) | 18.24 (10.99 to 27.76) | 17.83 (10.58 to 26.87) | 17.52 (10.72 to 25.82) | 17.05 (9.64 to 25.62) | 17.10 (9.75 to 25.00) | 16.62 (10.70 to 24.01) |
| Congo | Female | Prevalence | 223.61 (126.21 to 490.98) | 457.75 (223.50 to 1111.18) | 582.06 (241.24 to 1534.55) | 725.10 (262.94 to 2018.74) | 847.56 (280.73 to 2434.26) | 937.38 (294.67 to 2739.14) | 1001.29 (305.00 to 2955.65) | 1052.50 (311.94 to 3128.69) | 1095.61 (318.68 to 3275.75) | 1133.27 (324.33 to 3402.73) | 1168.64 (329.96 to 3522.67) | 1205.35 (335.84 to 3647.13) | 1247.38 (342.17 to 3790.28) | 1267.80 (345.83 to 3859.10) | 1267.82 (344.75 to 3859.44) | 1267.82 (344.85 to 3859.61) | 1267.83 (345.42 to 3859.18) | 1267.81 (345.68 to 3859.20) | 1267.81 (345.24 to 3859.18) | 1267.81 (345.45 to 3858.88) |
| Congo | Male | DALY rates | 8.23 (4.63 to 13.38) | 37.16 (16.56 to 76.75) | 38.59 (16.46 to 80.77) | 40.19 (17.77 to 81.73) | 42.32 (19.52 to 90.14) | 43.46 (19.41 to 88.89) | 44.04 (19.05 to 90.94) | 44.83 (21.44 to 89.98) | 44.84 (19.89 to 92.43) | 45.60 (20.69 to 95.45) | 45.87 (20.28 to 95.44) | 45.85 (20.96 to 93.70) | 46.10 (21.29 to 96.12) | 45.50 (20.05 to 91.96) | 44.60 (20.28 to 86.89) | 44.23 (21.18 to 89.98) | 42.63 (20.00 to 84.38) | 42.22 (18.91 to 85.63) | 41.37 (19.84 to 83.26) | 40.81 (18.99 to 83.59) |
| Congo | Male | Prevalence | 190.66 (95.29 to 456.55) | 593.07 (265.00 to 1278.22) | 729.22 (301.37 to 1724.68) | 886.57 (334.41 to 2226.72) | 1022.07 (366.05 to 2643.47) | 1121.85 (389.51 to 2953.77) | 1193.11 (406.62 to 3174.97) | 1250.31 (421.12 to 3354.84) | 1298.61 (432.32 to 3505.16) | 1340.84 (441.76 to 3637.62) | 1380.57 (451.33 to 3763.29) | 1421.93 (459.91 to 3891.55) | 1469.34 (471.13 to 4042.27) | 1492.36 (475.54 to 4112.89) | 1492.41 (476.13 to 4110.41) | 1492.45 (477.02 to 4111.82) | 1492.41 (475.29 to 4112.20) | 1492.40 (475.41 to 4111.92) | 1492.42 (476.45 to 4112.15) | 1492.36 (475.48 to 4112.25) |
| Côte d'Ivoire | Both | DALY rates | 10.71 (6.49 to 16.69) | 54.21 (29.60 to 94.97) | 76.18 (40.07 to 127.98) | 109.20 (62.47 to 180.37) | 142.03 (85.50 to 222.49) | 166.73 (102.01 to 259.01) | 186.63 (117.58 to 289.91) | 209.10 (130.92 to 305.08) | 233.58 (149.76 to 348.20) | 253.32 (168.45 to 369.93) | 260.84 (170.00 to 380.61) | 266.76 (175.71 to 385.76) | 277.79 (184.43 to 394.72) | 280.90 (186.19 to 406.78) | 271.73 (182.63 to 383.72) | 255.05 (169.44 to 363.61) | 236.09 (161.61 to 333.90) | 214.90 (145.56 to 299.35) | 188.99 (128.87 to 262.04) | 151.24 (103.31 to 204.71) |
| Côte d'Ivoire | Both | Prevalence | 1110.02 (365.68 to 2742.39) | 2992.76 (1155.04 to 7181.58) | 4322.93 (1623.17 to 10386.87) | 5925.87 (2231.37 to 14150.47) | 7326.15 (2756.34 to 17382.49) | 8360.86 (3134.11 to 19747.94) | 9123.16 (3429.57 to 21460.72) | 9789.42 (3734.88 to 22868.56) | 10406.19 (4045.82 to 24115.59) | 10924.00 (4299.68 to 25187.46) | 11340.76 (4463.53 to 26116.61) | 11750.55 (4620.86 to 27057.28) | 12254.40 (4833.94 to 28165.74) | 12511.01 (4946.77 to 28715.93) | 12474.35 (4911.48 to 28682.88) | 12376.12 (4814.76 to 28579.26) | 12232.53 (4664.68 to 28440.78) | 12073.90 (4505.07 to 28284.04) | 11859.42 (4298.97 to 28064.96) | 11540.49 (3991.74 to 27743.40) |
| Côte d'Ivoire | Female | DALY rates | 12.57 (7.23 to 19.09) | 23.01 (13.82 to 35.64) | 24.92 (15.20 to 38.24) | 27.86 (17.05 to 42.73) | 30.22 (19.28 to 44.38) | 32.02 (20.04 to 48.20) | 33.29 (20.68 to 48.93) | 34.41 (22.06 to 50.69) | 35.32 (21.74 to 52.95) | 36.31 (22.39 to 54.99) | 37.05 (22.98 to 54.29) | 37.52 (24.15 to 54.25) | 38.54 (25.01 to 57.85) | 38.31 (25.21 to 55.96) | 38.02 (23.84 to 53.14) | 37.47 (24.41 to 53.52) | 36.79 (23.88 to 52.98) | 35.88 (23.90 to 51.12) | 35.60 (23.18 to 51.63) | 34.39 (22.80 to 48.44) |
| Côte d'Ivoire | Female | Prevalence | 1126.92 (382.86 to 2755.19) | 2746.33 (874.16 to 6838.19) | 3916.22 (1191.12 to 9872.58) | 5271.08 (1558.60 to 13380.09) | 6423.01 (1873.84 to 16359.49) | 7267.96 (2103.70 to 18543.37) | 7870.87 (2270.31 to 20099.11) | 8352.31 (2403.58 to 21340.13) | 8760.03 (2517.79 to 22391.49) | 9118.36 (2614.81 to 23314.09) | 9455.36 (2709.31 to 24182.97) | 9805.48 (2806.94 to 25082.56) | 10202.98 (2919.14 to 26104.21) | 10396.64 (2972.17 to 26603.46) | 10396.60 (2971.57 to 26601.81) | 10396.64 (2973.60 to 26602.54) | 10396.59 (2971.76 to 26603.71) | 10396.54 (2972.21 to 26602.50) | 10396.61 (2972.05 to 26603.87) | 10396.60 (2972.87 to 26603.23) |
| Côte d'Ivoire | Male | DALY rates | 8.94 (5.16 to 14.18) | 83.43 (38.93 to 158.23) | 123.40 (60.18 to 216.65) | 184.70 (100.54 to 317.84) | 250.42 (147.75 to 403.21) | 304.36 (182.05 to 477.38) | 343.88 (210.71 to 539.18) | 375.53 (233.86 to 554.01) | 401.20 (253.98 to 601.96) | 426.05 (279.42 to 623.39) | 444.79 (285.32 to 652.94) | 465.73 (299.45 to 678.65) | 488.24 (317.72 to 698.68) | 492.97 (320.98 to 716.48) | 483.74 (321.47 to 692.28) | 472.97 (313.44 to 681.07) | 466.95 (315.23 to 664.57) | 458.83 (309.48 to 645.98) | 451.09 (299.44 to 625.95) | 439.17 (296.35 to 602.86) |
| Côte d'Ivoire | Male | Prevalence | 1093.90 (345.64 to 2730.19) | 3223.46 (1368.98 to 7472.39) | 4697.54 (2004.98 to 10861.07) | 6533.76 (2850.15 to 14829.57) | 8201.72 (3618.15 to 18305.96) | 9477.51 (4248.53 to 20892.92) | 10407.38 (4729.79 to 22762.42) | 11158.49 (5095.50 to 24240.03) | 11798.01 (5410.18 to 25521.20) | 12361.22 (5710.86 to 26627.28) | 12890.49 (5989.16 to 27675.03) | 13438.86 (6265.70 to 28748.92) | 14058.98 (6567.62 to 29976.68) | 14359.34 (6727.58 to 30563.02) | 14359.09 (6727.29 to 30571.72) | 14358.79 (6727.84 to 30559.95) | 14359.23 (6729.24 to 30568.91) | 14359.36 (6718.03 to 30574.41) | 14359.01 (6727.09 to 30561.61) | 14358.99 (6761.41 to 30554.28) |
| Democratic Republic of the Congo | Both | DALY rates | 9.90 (6.06 to 15.19) | 32.02 (16.81 to 61.30) | 34.79 (18.69 to 62.91) | 38.91 (21.04 to 70.16) | 42.49 (22.33 to 74.19) | 45.92 (24.75 to 80.37) | 47.76 (25.67 to 83.32) | 49.27 (26.30 to 86.15) | 50.89 (27.69 to 91.15) | 52.28 (28.42 to 91.29) | 52.96 (28.92 to 92.40) | 52.86 (29.79 to 94.44) | 52.57 (28.70 to 92.01) | 51.65 (28.47 to 86.53) | 47.87 (27.40 to 81.61) | 45.41 (26.44 to 75.47) | 42.48 (24.74 to 69.34) | 39.73 (23.53 to 65.23) | 37.02 (22.52 to 58.15) | 33.15 (20.02 to 50.93) |
| Democratic Republic of the Congo | Both | Prevalence | 396.27 (208.07 to 756.66) | 1029.04 (548.14 to 1965.59) | 1392.12 (707.46 to 2724.94) | 1814.43 (887.57 to 3610.06) | 2177.75 (1044.27 to 4351.74) | 2446.39 (1158.53 to 4896.88) | 2639.15 (1239.85 to 5288.38) | 2794.39 (1306.32 to 5612.62) | 2926.08 (1361.90 to 5883.20) | 3042.95 (1412.63 to 6121.70) | 3149.50 (1458.86 to 6340.19) | 3254.79 (1501.40 to 6563.34) | 3373.35 (1551.32 to 6816.72) | 3424.10 (1570.04 to 6932.35) | 3400.21 (1551.38 to 6908.58) | 3382.43 (1537.86 to 6891.51) | 3362.97 (1527.00 to 6871.96) | 3345.92 (1517.14 to 6855.71) | 3327.27 (1506.45 to 6837.40) | 3302.03 (1487.14 to 6811.40) |
| Democratic Republic of the Congo | Female | DALY rates | 11.74 (6.70 to 18.21) | 19.63 (10.98 to 31.28) | 20.12 (12.08 to 31.25) | 20.85 (12.70 to 31.78) | 21.00 (12.37 to 33.01) | 21.50 (12.33 to 32.81) | 21.33 (12.09 to 32.18) | 21.56 (13.05 to 32.44) | 21.80 (12.95 to 33.19) | 22.02 (13.06 to 33.10) | 21.79 (12.85 to 33.15) | 21.54 (13.40 to 32.09) | 21.66 (12.93 to 31.64) | 21.68 (13.00 to 33.23) | 21.40 (12.82 to 33.10) | 21.02 (12.66 to 31.49) | 20.64 (12.67 to 31.33) | 20.28 (12.19 to 30.61) | 19.83 (12.23 to 28.94) | 18.99 (11.33 to 27.48) |
| Democratic Republic of the Congo | Female | Prevalence | 413.46 (223.75 to 775.31) | 935.61 (468.89 to 1824.54) | 1277.72 (600.91 to 2569.70) | 1671.99 (753.69 to 3430.24) | 2008.00 (882.50 to 4161.74) | 2254.35 (977.08 to 4698.65) | 2429.93 (1045.13 to 5080.67) | 2570.64 (1098.96 to 5387.61) | 2689.70 (1145.04 to 5646.97) | 2794.17 (1186.17 to 5874.19) | 2891.80 (1222.70 to 6086.89) | 2992.80 (1261.98 to 6306.87) | 3107.98 (1306.60 to 6557.68) | 3163.92 (1328.08 to 6679.52) | 3163.94 (1328.10 to 6679.59) | 3163.96 (1327.40 to 6679.91) | 3163.92 (1327.83 to 6679.74) | 3163.92 (1327.43 to 6679.31) | 3163.95 (1327.94 to 6679.92) | 3163.93 (1327.78 to 6679.49) |
| Democratic Republic of the Congo | Male | DALY rates | 8.11 (4.39 to 13.14) | 44.03 (18.96 to 95.68) | 49.02 (21.95 to 103.64) | 56.42 (25.96 to 111.48) | 63.33 (28.43 to 122.32) | 69.58 (31.78 to 133.16) | 73.33 (33.63 to 139.93) | 76.04 (36.59 to 144.35) | 79.04 (38.20 to 152.28) | 81.36 (37.38 to 154.52) | 83.44 (40.55 to 156.36) | 85.27 (41.69 to 160.31) | 86.98 (42.30 to 165.35) | 87.86 (43.41 to 160.93) | 85.76 (42.85 to 155.54) | 85.15 (42.38 to 153.95) | 83.65 (41.14 to 152.94) | 81.69 (41.89 to 147.24) | 80.28 (39.76 to 145.54) | 77.88 (39.20 to 141.63) |
| Democratic Republic of the Congo | Male | Prevalence | 379.69 (191.34 to 741.09) | 1119.65 (608.05 to 2078.27) | 1503.14 (781.06 to 2904.24) | 1952.56 (980.46 to 3835.39) | 2342.39 (1163.95 to 4619.08) | 2632.50 (1299.77 to 5197.66) | 2841.62 (1395.83 to 5612.24) | 3010.66 (1477.96 to 5945.58) | 3154.77 (1546.07 to 6229.92) | 3281.98 (1606.83 to 6479.67) | 3401.52 (1663.95 to 6707.92) | 3525.94 (1721.17 to 6949.94) | 3668.75 (1793.22 to 7226.27) | 3738.41 (1824.98 to 7357.71) | 3738.44 (1826.62 to 7366.29) | 3738.46 (1824.50 to 7355.50) | 3738.33 (1826.84 to 7351.95) | 3738.39 (1824.81 to 7358.37) | 3738.38 (1825.39 to 7358.76) | 3738.36 (1824.90 to 7367.38) |
| Dominican Republic | Both | DALY rates | 9.82 (5.89 to 14.44) | 29.58 (16.20 to 54.26) | 30.90 (16.65 to 56.59) | 32.76 (17.69 to 60.29) | 34.16 (18.75 to 60.80) | 35.15 (19.12 to 65.54) | 36.30 (19.85 to 64.87) | 37.14 (19.94 to 65.98) | 37.59 (20.43 to 66.83) | 37.70 (20.34 to 66.37) | 37.66 (21.23 to 66.48) | 37.55 (19.85 to 69.12) | 37.75 (21.33 to 65.92) | 37.28 (20.77 to 64.98) | 36.49 (20.25 to 64.78) | 35.81 (19.88 to 63.17) | 34.65 (19.69 to 58.23) | 33.29 (18.83 to 55.93) | 32.63 (18.79 to 55.57) | 31.14 (18.02 to 52.56) |
| Dominican Republic | Both | Prevalence | 263.47 (110.82 to 804.72) | 682.63 (263.31 to 2060.69) | 887.22 (294.47 to 2914.52) | 1122.79 (332.70 to 3863.10) | 1321.46 (365.40 to 4658.07) | 1467.91 (389.83 to 5241.42) | 1574.24 (407.69 to 5663.03) | 1660.32 (422.14 to 6001.27) | 1731.88 (434.05 to 6285.06) | 1793.32 (444.68 to 6533.53) | 1850.51 (455.95 to 6764.93) | 1910.36 (465.83 to 7000.00) | 1979.28 (476.88 to 7275.15) | 2010.76 (481.86 to 7409.85) | 2008.22 (480.77 to 7399.93) | 2005.63 (478.84 to 7400.55) | 1999.54 (475.76 to 7384.98) | 1996.11 (472.77 to 7382.25) | 1993.74 (470.82 to 7376.93) | 1988.80 (467.12 to 7374.51) |
| Dominican Republic | Female | DALY rates | 11.42 (6.61 to 17.57) | 19.38 (11.50 to 29.43) | 19.53 (11.51 to 30.38) | 19.89 (11.72 to 31.04) | 19.90 (11.95 to 31.09) | 19.56 (11.40 to 30.33) | 19.96 (12.06 to 29.87) | 20.14 (11.98 to 31.00) | 20.18 (11.82 to 30.45) | 20.13 (11.71 to 30.35) | 20.09 (11.81 to 30.21) | 19.99 (12.01 to 30.70) | 19.99 (12.19 to 30.29) | 19.69 (11.93 to 29.84) | 19.52 (11.40 to 29.90) | 19.19 (11.92 to 28.84) | 19.00 (11.20 to 28.61) | 18.59 (10.94 to 29.03) | 18.36 (11.31 to 27.33) | 17.70 (10.74 to 26.42) |
| Dominican Republic | Female | Prevalence | 279.98 (128.23 to 820.40) | 606.35 (229.35 to 1948.43) | 801.12 (250.46 to 2761.25) | 1024.63 (273.38 to 3695.33) | 1212.39 (291.66 to 4479.58) | 1349.69 (306.49 to 5052.31) | 1447.74 (318.01 to 5462.49) | 1527.01 (326.50 to 5793.03) | 1594.01 (333.56 to 6072.56) | 1652.50 (339.62 to 6316.95) | 1707.33 (345.66 to 6546.38) | 1764.10 (352.06 to 6783.07) | 1829.14 (359.40 to 7053.82) | 1860.15 (362.81 to 7184.18) | 1860.09 (362.67 to 7182.94) | 1860.10 (362.66 to 7183.85) | 1860.13 (363.23 to 7183.50) | 1860.11 (362.28 to 7184.10) | 1860.10 (362.34 to 7183.88) | 1860.11 (362.30 to 7183.62) |
| Dominican Republic | Male | DALY rates | 8.28 (4.32 to 13.41) | 39.37 (17.24 to 83.71) | 41.93 (17.98 to 87.95) | 45.43 (19.57 to 97.05) | 48.36 (22.33 to 96.59) | 50.62 (22.92 to 103.95) | 52.14 (23.92 to 106.09) | 53.33 (24.28 to 105.09) | 54.27 (25.56 to 107.97) | 54.90 (24.15 to 108.74) | 55.31 (25.27 to 109.49) | 55.50 (25.01 to 112.90) | 56.19 (27.54 to 107.16) | 56.03 (27.18 to 109.16) | 55.16 (25.66 to 110.50) | 54.73 (24.65 to 109.40) | 53.92 (25.64 to 101.98) | 52.22 (24.63 to 99.32) | 51.57 (24.22 to 103.64) | 50.18 (23.83 to 95.27) |
| Dominican Republic | Male | Prevalence | 247.66 (93.53 to 789.70) | 755.91 (284.63 to 2146.51) | 970.70 (323.49 to 2984.79) | 1219.36 (366.65 to 3954.60) | 1430.07 (409.14 to 4780.77) | 1585.28 (441.01 to 5391.56) | 1696.77 (464.08 to 5829.30) | 1787.20 (482.82 to 6181.07) | 1863.94 (498.05 to 6479.68) | 1931.18 (512.01 to 6740.25) | 1994.38 (524.48 to 6982.25) | 2059.95 (535.46 to 7231.30) | 2135.27 (551.94 to 7514.90) | 2171.27 (559.57 to 7660.02) | 2171.24 (559.24 to 7649.32) | 2171.26 (560.04 to 7658.16) | 2171.25 (560.57 to 7645.02) | 2171.20 (560.23 to 7649.76) | 2171.26 (560.19 to 7646.23) | 2171.20 (561.03 to 7657.83) |
| Egypt | Both | DALY rates | 0.00 (0.00 to 0.00) | 0.00 (0.00 to 0.00) | 0.00 (0.00 to 0.00) | 0.00 (0.00 to 0.00) | 0.00 (0.00 to 0.00) | 0.00 (0.00 to 0.00) | 0.00 (0.00 to 0.00) | 0.00 (0.00 to 0.00) | 0.00 (0.00 to 0.00) | 0.00 (0.00 to 0.00) | 0.00 (0.00 to 0.00) | 0.00 (0.00 to 0.00) | 0.00 (0.00 to 0.00) | 0.00 (0.00 to 0.00) | 0.00 (0.00 to 0.00) | 0.00 (0.00 to 0.00) | 0.00 (0.00 to 0.00) | 0.00 (0.00 to 0.00) | 0.00 (0.00 to 0.00) | 0.00 (0.00 to 0.00) |
| Egypt | Both | Prevalence | 95.83 (10.32 to 416.09) | 233.88 (25.20 to 1015.48) | 339.35 (36.56 to 1473.40) | 461.92 (49.76 to 2005.60) | 566.20 (61.00 to 2458.36) | 641.92 (69.15 to 2787.12) | 696.44 (75.03 to 3023.83) | 739.64 (79.68 to 3211.39) | 776.09 (83.61 to 3369.66) | 808.42 (87.09 to 3510.02) | 838.80 (90.37 to 3641.96) | 869.92 (93.72 to 3777.07) | 905.55 (97.56 to 3931.78) | 922.65 (99.40 to 4006.01) | 922.65 (99.40 to 4006.01) | 922.65 (99.40 to 4006.01) | 922.65 (99.40 to 4006.01) | 922.65 (99.40 to 4006.01) | 922.65 (99.40 to 4006.01) | 922.65 (99.40 to 4006.01) |
| Egypt | Female | DALY rates | 0.00 (0.00 to 0.00) | 0.00 (0.00 to 0.00) | 0.00 (0.00 to 0.00) | 0.00 (0.00 to 0.00) | 0.00 (0.00 to 0.00) | 0.00 (0.00 to 0.00) | 0.00 (0.00 to 0.00) | 0.00 (0.00 to 0.00) | 0.00 (0.00 to 0.00) | 0.00 (0.00 to 0.00) | 0.00 (0.00 to 0.00) | 0.00 (0.00 to 0.00) | 0.00 (0.00 to 0.00) | 0.00 (0.00 to 0.00) | 0.00 (0.00 to 0.00) | 0.00 (0.00 to 0.00) | 0.00 (0.00 to 0.00) | 0.00 (0.00 to 0.00) | 0.00 (0.00 to 0.00) | 0.00 (0.00 to 0.00) |
| Egypt | Female | Prevalence | 95.76 (10.32 to 415.78) | 233.88 (25.20 to 1015.48) | 339.35 (36.56 to 1473.40) | 461.92 (49.76 to 2005.60) | 566.20 (61.00 to 2458.36) | 641.92 (69.15 to 2787.12) | 696.44 (75.03 to 3023.83) | 739.64 (79.68 to 3211.39) | 776.09 (83.61 to 3369.66) | 808.42 (87.09 to 3510.02) | 838.80 (90.37 to 3641.96) | 869.92 (93.72 to 3777.07) | 905.55 (97.56 to 3931.78) | 922.65 (99.40 to 4006.01) | 922.65 (99.40 to 4006.01) | 922.65 (99.40 to 4006.01) | 922.65 (99.40 to 4006.01) | 922.65 (99.40 to 4006.01) | 922.65 (99.40 to 4006.01) | 922.65 (99.40 to 4006.01) |
| Egypt | Male | DALY rates | 0.00 (0.00 to 0.00) | 0.00 (0.00 to 0.00) | 0.00 (0.00 to 0.00) | 0.00 (0.00 to 0.00) | 0.00 (0.00 to 0.00) | 0.00 (0.00 to 0.00) | 0.00 (0.00 to 0.00) | 0.00 (0.00 to 0.00) | 0.00 (0.00 to 0.00) | 0.00 (0.00 to 0.00) | 0.00 (0.00 to 0.00) | 0.00 (0.00 to 0.00) | 0.00 (0.00 to 0.00) | 0.00 (0.00 to 0.00) | 0.00 (0.00 to 0.00) | 0.00 (0.00 to 0.00) | 0.00 (0.00 to 0.00) | 0.00 (0.00 to 0.00) | 0.00 (0.00 to 0.00) | 0.00 (0.00 to 0.00) |
| Egypt | Male | Prevalence | 95.90 (10.33 to 416.39) | 233.88 (25.20 to 1015.48) | 339.35 (36.56 to 1473.40) | 461.92 (49.76 to 2005.60) | 566.20 (61.00 to 2458.36) | 641.92 (69.15 to 2787.12) | 696.44 (75.03 to 3023.83) | 739.64 (79.68 to 3211.39) | 776.09 (83.61 to 3369.66) | 808.42 (87.09 to 3510.02) | 838.80 (90.37 to 3641.96) | 869.92 (93.72 to 3777.07) | 905.55 (97.56 to 3931.78) | 922.65 (99.40 to 4006.01) | 922.65 (99.40 to 4006.01) | 922.65 (99.40 to 4006.01) | 922.65 (99.40 to 4006.01) | 922.65 (99.40 to 4006.01) | 922.65 (99.40 to 4006.01) | 922.65 (99.40 to 4006.01) |
| Equatorial Guinea | Both | DALY rates | 9.77 (6.16 to 14.74) | 30.32 (16.05 to 56.35) | 32.22 (15.89 to 61.05) | 34.43 (17.86 to 62.79) | 36.22 (18.32 to 68.32) | 36.71 (19.18 to 67.62) | 36.97 (19.55 to 66.20) | 36.55 (19.84 to 66.00) | 35.86 (19.76 to 63.89) | 35.55 (19.69 to 64.44) | 35.62 (19.27 to 62.42) | 34.95 (19.13 to 62.19) | 34.95 (19.08 to 61.85) | 34.55 (19.80 to 61.11) | 33.16 (18.53 to 58.36) | 32.53 (18.41 to 55.03) | 32.06 (18.54 to 52.48) | 30.70 (17.64 to 50.91) | 29.16 (16.91 to 49.92) | 25.81 (15.62 to 40.39) |
| Equatorial Guinea | Both | Prevalence | 265.39 (102.14 to 1004.93) | 682.47 (252.35 to 2459.90) | 886.74 (275.75 to 3480.44) | 1119.52 (309.50 to 4645.49) | 1315.32 (335.73 to 5630.89) | 1456.62 (351.86 to 6354.64) | 1554.24 (361.57 to 6869.21) | 1629.94 (369.53 to 7279.64) | 1692.75 (372.21 to 7626.51) | 1748.93 (376.16 to 7931.81) | 1803.34 (379.58 to 8218.46) | 1858.33 (385.88 to 8513.26) | 1923.03 (393.32 to 8854.73) | 1956.09 (397.46 to 9019.26) | 1950.40 (394.01 to 9014.83) | 1951.51 (394.41 to 9015.32) | 1951.54 (394.53 to 9015.77) | 1945.24 (390.08 to 9011.19) | 1933.48 (381.66 to 9002.29) | 1910.47 (366.50 to 8983.68) |
| Equatorial Guinea | Female | DALY rates | 11.50 (6.82 to 17.87) | 19.21 (10.61 to 30.08) | 19.56 (11.66 to 31.94) | 19.81 (11.87 to 30.21) | 19.85 (11.59 to 29.88) | 19.53 (11.70 to 29.98) | 19.80 (11.86 to 30.73) | 19.95 (11.58 to 30.59) | 19.56 (11.57 to 30.29) | 19.58 (11.72 to 29.96) | 19.77 (11.40 to 30.58) | 19.60 (11.65 to 29.85) | 19.60 (11.79 to 30.16) | 19.52 (12.33 to 29.62) | 19.03 (11.38 to 29.71) | 18.60 (11.26 to 28.20) | 18.22 (10.81 to 27.62) | 17.88 (11.12 to 27.19) | 17.74 (10.97 to 26.26) | 16.95 (10.42 to 25.09) |
| Equatorial Guinea | Female | Prevalence | 280.10 (117.50 to 1013.40) | 599.79 (203.15 to 2399.72) | 789.52 (214.62 to 3408.52) | 1006.22 (225.89 to 4560.82) | 1189.87 (236.10 to 5535.75) | 1325.01 (243.08 to 6254.38) | 1421.63 (249.56 to 6767.81) | 1499.05 (254.71 to 7178.75) | 1564.56 (258.68 to 7526.96) | 1622.34 (261.81 to 7833.59) | 1676.12 (265.83 to 8118.93) | 1731.65 (269.05 to 8414.92) | 1795.75 (273.02 to 8755.29) | 1826.42 (275.56 to 8917.70) | 1826.42 (275.26 to 8917.33) | 1826.44 (274.97 to 8917.31) | 1826.42 (275.42 to 8917.52) | 1826.43 (275.19 to 8918.36) | 1826.43 (275.23 to 8918.39) | 1826.41 (275.03 to 8917.85) |
| Equatorial Guinea | Male | DALY rates | 8.23 (4.44 to 13.20) | 39.23 (17.72 to 82.30) | 41.52 (17.44 to 89.11) | 44.70 (20.69 to 91.99) | 47.94 (20.94 to 101.38) | 49.91 (22.43 to 102.52) | 51.63 (23.45 to 102.03) | 52.53 (24.72 to 106.03) | 53.41 (25.38 to 107.29) | 54.25 (23.94 to 110.65) | 55.03 (25.50 to 109.89) | 54.94 (25.13 to 107.34) | 55.97 (25.26 to 108.38) | 55.05 (23.88 to 107.80) | 53.95 (25.70 to 107.14) | 52.74 (25.54 to 103.77) | 52.12 (25.83 to 99.79) | 50.94 (23.89 to 101.07) | 50.44 (23.53 to 100.11) | 49.27 (23.42 to 92.95) |
| Equatorial Guinea | Male | Prevalence | 252.32 (87.92 to 997.41) | 748.85 (267.30 to 2508.20) | 958.08 (303.29 to 3533.22) | 1199.05 (343.68 to 4704.94) | 1405.11 (376.94 to 5698.98) | 1557.81 (398.56 to 6431.73) | 1667.46 (417.16 to 6955.79) | 1755.86 (431.47 to 7376.70) | 1830.83 (444.25 to 7733.73) | 1897.15 (455.52 to 8046.81) | 1959.06 (465.00 to 8340.28) | 2023.28 (476.76 to 8641.32) | 2097.34 (491.14 to 8990.91) | 2132.97 (497.45 to 9157.81) | 2132.92 (496.51 to 9158.36) | 2132.95 (496.84 to 9157.51) | 2133.00 (495.75 to 9158.26) | 2132.96 (496.43 to 9157.87) | 2132.97 (496.52 to 9158.64) | 2132.99 (496.59 to 9157.95) |
| Eritrea | Both | DALY rates | 9.30 (5.72 to 13.98) | 26.85 (14.41 to 49.42) | 27.10 (14.34 to 48.58) | 27.48 (14.53 to 49.62) | 27.31 (14.34 to 49.13) | 27.46 (15.48 to 47.68) | 27.54 (15.44 to 49.79) | 27.45 (14.62 to 50.80) | 27.48 (14.71 to 49.47) | 26.81 (14.31 to 47.96) | 25.89 (14.63 to 44.74) | 25.39 (14.47 to 44.94) | 24.63 (13.50 to 42.19) | 24.10 (12.87 to 41.42) | 23.20 (13.04 to 38.44) | 22.64 (13.18 to 37.31) | 21.85 (12.39 to 35.91) | 20.86 (12.28 to 34.67) | 19.94 (12.70 to 31.53) | 18.50 (11.48 to 27.87) |
| Eritrea | Both | Prevalence | 122.80 (90.99 to 238.11) | 321.01 (196.02 to 608.02) | 358.24 (201.82 to 763.12) | 401.17 (208.14 to 950.72) | 437.47 (209.87 to 1110.62) | 463.95 (212.96 to 1227.67) | 482.77 (214.98 to 1312.22) | 497.47 (215.90 to 1381.50) | 510.17 (217.35 to 1440.39) | 519.65 (219.30 to 1490.57) | 526.62 (220.63 to 1536.28) | 533.66 (221.27 to 1584.13) | 541.68 (222.19 to 1638.80) | 544.14 (221.20 to 1663.87) | 539.77 (219.22 to 1662.23) | 538.00 (218.41 to 1661.35) | 534.99 (218.20 to 1659.33) | 530.02 (216.91 to 1656.87) | 524.90 (215.40 to 1653.67) | 518.14 (212.49 to 1650.11) |
| Eritrea | Female | DALY rates | 10.93 (6.09 to 17.36) | 18.81 (10.76 to 29.70) | 18.72 (10.96 to 29.37) | 18.80 (10.92 to 29.35) | 18.47 (10.90 to 29.57) | 18.42 (10.32 to 29.29) | 18.44 (10.53 to 28.56) | 18.28 (11.32 to 29.49) | 18.39 (10.34 to 28.53) | 17.96 (10.76 to 27.23) | 17.94 (10.64 to 27.64) | 17.88 (10.56 to 27.06) | 17.66 (9.91 to 26.96) | 17.40 (10.13 to 27.04) | 17.29 (9.90 to 26.75) | 17.06 (10.12 to 26.93) | 16.75 (9.99 to 26.68) | 16.38 (9.23 to 25.05) | 16.06 (10.25 to 24.19) | 15.47 (9.54 to 23.14) |
| Eritrea | Female | Prevalence | 139.00 (104.57 to 255.89) | 260.08 (179.89 to 550.06) | 295.62 (183.28 to 718.02) | 336.58 (186.41 to 911.39) | 371.21 (189.24 to 1074.47) | 396.52 (191.47 to 1194.08) | 414.53 (192.18 to 1278.60) | 429.02 (192.87 to 1346.83) | 441.32 (193.92 to 1405.67) | 452.05 (194.84 to 1456.22) | 462.13 (194.90 to 1503.55) | 472.55 (195.46 to 1553.30) | 484.44 (196.69 to 1609.72) | 490.17 (197.31 to 1636.34) | 490.18 (197.24 to 1636.66) | 490.19 (197.87 to 1636.81) | 490.19 (197.72 to 1636.45) | 490.20 (197.58 to 1636.57) | 490.17 (197.56 to 1636.20) | 490.20 (197.48 to 1635.87) |
| Eritrea | Male | DALY rates | 7.77 (4.22 to 12.29) | 34.38 (16.00 to 73.83) | 34.90 (14.73 to 73.38) | 35.53 (15.76 to 76.11) | 35.50 (14.69 to 75.09) | 35.84 (16.65 to 71.65) | 35.99 (15.78 to 76.27) | 36.08 (15.93 to 78.26) | 36.08 (15.48 to 75.74) | 35.63 (15.40 to 77.60) | 34.69 (15.70 to 71.13) | 34.70 (15.51 to 74.17) | 34.47 (14.68 to 73.50) | 34.60 (14.82 to 71.33) | 33.81 (14.89 to 70.70) | 33.25 (15.14 to 68.12) | 32.53 (15.28 to 67.45) | 31.97 (14.96 to 67.13) | 31.55 (14.33 to 64.64) | 30.46 (14.38 to 62.19) |
| Eritrea | Male | Prevalence | 107.59 (75.34 to 221.76) | 378.02 (201.23 to 809.57) | 416.59 (206.46 to 896.86) | 461.10 (214.46 to 1020.82) | 498.85 (220.44 to 1167.49) | 526.42 (226.48 to 1285.77) | 546.07 (229.23 to 1368.41) | 561.88 (231.34 to 1435.93) | 575.34 (233.60 to 1491.73) | 587.08 (235.52 to 1543.41) | 598.03 (236.50 to 1589.47) | 609.47 (238.44 to 1637.72) | 622.47 (238.82 to 1694.14) | 628.75 (240.48 to 1721.13) | 628.77 (239.94 to 1720.23) | 628.77 (240.01 to 1719.52) | 628.75 (239.66 to 1719.22) | 628.76 (239.53 to 1719.61) | 628.74 (239.66 to 1720.79) | 628.75 (239.29 to 1719.91) |
| Ethiopia | Both | DALY rates | 9.45 (6.31 to 13.73) | 27.45 (15.58 to 48.85) | 27.81 (16.04 to 51.03) | 28.28 (16.10 to 51.36) | 28.44 (15.83 to 50.33) | 28.57 (16.41 to 51.20) | 28.45 (16.00 to 50.62) | 28.48 (16.35 to 51.14) | 28.56 (16.06 to 50.50) | 28.79 (16.33 to 50.55) | 28.85 (16.14 to 51.27) | 28.68 (16.27 to 51.30) | 28.20 (16.02 to 51.44) | 27.86 (15.97 to 50.37) | 27.74 (15.60 to 50.36) | 27.16 (15.39 to 47.06) | 26.38 (14.92 to 46.12) | 25.73 (14.90 to 45.26) | 24.85 (14.66 to 43.24) | 23.30 (13.96 to 39.98) |
| Ethiopia | Both | Prevalence | 153.36 (105.61 to 275.53) | 403.59 (257.19 to 724.13) | 477.44 (277.63 to 940.83) | 559.10 (303.19 to 1159.65) | 626.04 (324.59 to 1339.57) | 670.26 (337.39 to 1451.90) | 700.68 (345.76 to 1528.31) | 727.84 (355.56 to 1608.03) | 750.48 (363.66 to 1665.25) | 767.03 (370.80 to 1701.39) | 778.51 (374.30 to 1734.92) | 791.06 (377.95 to 1803.71) | 806.98 (380.64 to 1844.68) | 811.48 (380.08 to 1860.71) | 812.15 (381.23 to 1864.87) | 811.60 (380.15 to 1870.84) | 806.17 (376.11 to 1877.78) | 801.15 (373.01 to 1886.80) | 799.61 (371.35 to 1887.63) | 773.70 (358.33 to 1875.08) |
| Ethiopia | Female | DALY rates | 11.11 (7.44 to 16.61) | 18.87 (12.29 to 27.36) | 18.91 (12.09 to 27.82) | 18.86 (12.17 to 27.15) | 18.82 (12.70 to 26.80) | 18.86 (12.22 to 27.37) | 18.62 (12.26 to 26.54) | 18.79 (12.35 to 27.24) | 18.54 (12.17 to 26.48) | 18.47 (12.40 to 26.35) | 18.33 (12.17 to 26.46) | 18.25 (12.21 to 25.69) | 17.95 (11.89 to 25.29) | 17.93 (11.72 to 24.90) | 17.45 (11.61 to 24.48) | 17.01 (11.50 to 24.18) | 16.75 (11.25 to 23.76) | 16.40 (10.69 to 23.11) | 15.95 (10.96 to 22.36) | 15.47 (10.61 to 21.98) |
| Ethiopia | Female | Prevalence | 169.50 (120.63 to 292.92) | 339.89 (218.08 to 652.09) | 410.57 (236.48 to 868.00) | 489.59 (257.64 to 1113.70) | 555.27 (273.23 to 1303.05) | 598.50 (282.93 to 1413.03) | 627.42 (289.80 to 1493.98) | 653.17 (295.90 to 1569.23) | 672.49 (300.63 to 1622.00) | 685.09 (303.48 to 1654.51) | 696.59 (305.42 to 1688.35) | 709.48 (307.03 to 1727.01) | 724.88 (308.56 to 1803.46) | 729.47 (308.28 to 1816.14) | 724.43 (306.14 to 1795.64) | 721.33 (304.98 to 1785.81) | 712.55 (302.00 to 1759.71) | 707.19 (300.24 to 1741.74) | 705.18 (300.19 to 1745.28) | 684.87 (295.07 to 1750.31) |
| Ethiopia | Male | DALY rates | 7.86 (5.06 to 12.07) | 35.62 (16.46 to 74.31) | 36.27 (17.28 to 75.49) | 37.37 (18.07 to 76.04) | 38.03 (17.49 to 75.93) | 38.58 (18.14 to 78.83) | 38.90 (18.03 to 79.21) | 38.97 (18.41 to 80.89) | 39.00 (18.01 to 79.24) | 38.77 (18.55 to 76.33) | 38.83 (17.81 to 76.04) | 38.74 (18.44 to 80.14) | 38.34 (18.48 to 78.40) | 37.79 (18.11 to 77.26) | 37.03 (17.70 to 75.66) | 36.50 (17.47 to 71.59) | 35.61 (17.15 to 72.44) | 35.08 (17.16 to 70.79) | 34.28 (16.73 to 68.17) | 33.42 (16.07 to 68.93) |
| Ethiopia | Male | Prevalence | 137.98 (88.46 to 259.58) | 464.27 (273.91 to 836.11) | 541.00 (305.22 to 1031.80) | 626.25 (336.08 to 1251.44) | 696.59 (358.88 to 1441.47) | 744.32 (374.08 to 1559.04) | 778.49 (384.12 to 1637.13) | 808.68 (392.11 to 1710.66) | 831.74 (398.79 to 1771.06) | 846.19 (403.46 to 1815.95) | 856.23 (408.98 to 1854.52) | 869.67 (415.39 to 1895.72) | 888.24 (421.34 to 1935.04) | 893.59 (422.97 to 1954.97) | 891.36 (421.23 to 1960.61) | 894.72 (421.41 to 1982.77) | 895.93 (421.52 to 2013.41) | 895.33 (421.70 to 2020.44) | 899.58 (422.16 to 2043.68) | 888.55 (416.53 to 2036.39) |
| Fiji | Both | DALY rates | 11.47 (7.17 to 17.78) | 64.59 (34.32 to 114.43) | 95.79 (54.82 to 161.17) | 143.97 (86.93 to 227.65) | 193.75 (121.78 to 285.63) | 230.72 (151.13 to 346.98) | 260.44 (168.40 to 382.10) | 283.03 (183.46 to 411.83) | 300.26 (196.92 to 429.63) | 314.53 (212.66 to 455.06) | 320.54 (213.44 to 454.56) | 325.48 (219.66 to 457.03) | 328.93 (221.84 to 457.75) | 314.83 (213.37 to 433.53) | 296.35 (199.24 to 408.03) | 273.45 (186.69 to 377.75) | 247.75 (173.63 to 337.24) | 205.81 (142.26 to 278.15) | 120.38 (85.49 to 161.23) | 79.74 (57.25 to 107.92) |
| Fiji | Both | Prevalence | 1386.14 (262.52 to 4072.46) | 3662.97 (916.94 to 10353.35) | 5325.87 (1340.50 to 15039.72) | 7356.01 (1941.11 to 20562.03) | 9165.39 (2536.77 to 25334.86) | 10484.43 (2954.53 to 28788.44) | 11460.89 (3291.26 to 31309.93) | 12235.96 (3543.75 to 33297.90) | 12881.32 (3756.85 to 34966.95) | 13455.10 (3971.91 to 36476.05) | 13945.00 (4106.24 to 37797.68) | 14447.52 (4259.18 to 39162.48) | 14990.32 (4394.90 to 40681.17) | 15163.30 (4356.34 to 41312.56) | 15056.01 (4233.66 to 41193.64) | 14900.63 (4083.36 to 41021.74) | 14695.32 (3857.51 to 40786.45) | 14331.92 (3496.98 to 40375.77) | 13539.77 (2741.45 to 39481.77) | 13147.29 (2350.42 to 39036.80) |
| Fiji | Female | DALY rates | 13.45 (7.97 to 20.91) | 24.19 (14.80 to 38.04) | 27.24 (16.65 to 40.71) | 30.36 (19.11 to 45.66) | 34.00 (21.60 to 50.25) | 36.79 (23.16 to 54.98) | 38.70 (24.33 to 57.50) | 40.54 (26.37 to 58.13) | 41.28 (25.44 to 60.71) | 42.80 (27.93 to 64.53) | 43.21 (27.26 to 63.57) | 44.51 (28.02 to 65.16) | 45.69 (28.75 to 67.25) | 45.66 (28.77 to 65.05) | 45.29 (28.39 to 65.73) | 44.40 (28.80 to 63.47) | 43.88 (28.73 to 62.41) | 42.72 (28.96 to 61.94) | 41.66 (27.17 to 61.97) | 40.92 (27.65 to 56.45) |
| Fiji | Female | Prevalence | 1407.55 (279.07 to 4097.79) | 3344.74 (595.80 to 9898.39) | 4779.41 (789.00 to 14296.24) | 6442.43 (1018.39 to 19382.53) | 7869.34 (1220.47 to 23739.31) | 8902.32 (1368.52 to 26888.10) | 9648.40 (1478.35 to 29158.67) | 10243.76 (1565.16 to 30966.33) | 10743.57 (1638.94 to 32485.01) | 11189.28 (1705.51 to 33836.83) | 11608.45 (1769.47 to 35106.74) | 12036.42 (1833.68 to 36404.28) | 12524.27 (1908.22 to 37881.84) | 12762.93 (1947.07 to 38604.00) | 12762.95 (1947.55 to 38602.55) | 12762.96 (1946.71 to 38602.92) | 12762.94 (1945.98 to 38602.42) | 12762.92 (1946.08 to 38603.26) | 12762.89 (1947.51 to 38602.87) | 12762.95 (1948.21 to 38602.97) |
| Fiji | Male | DALY rates | 9.61 (5.25 to 15.68) | 102.92 (50.40 to 196.95) | 161.69 (87.46 to 282.98) | 252.78 (148.78 to 406.50) | 346.64 (215.53 to 519.62) | 419.26 (268.73 to 636.40) | 472.48 (301.37 to 696.35) | 512.62 (325.96 to 753.29) | 544.11 (352.38 to 784.14) | 568.65 (380.36 to 826.15) | 588.66 (388.29 to 839.03) | 603.85 (404.90 to 853.26) | 622.06 (417.49 to 869.22) | 619.64 (414.37 to 855.06) | 605.71 (405.93 to 841.13) | 592.85 (400.33 to 821.86) | 583.96 (406.93 to 801.23) | 574.84 (394.06 to 782.26) | 560.37 (392.38 to 759.05) | 557.91 (387.53 to 755.14) |
| Fiji | Male | Prevalence | 1366.10 (247.37 to 4048.76) | 3964.84 (1131.19 to 10659.10) | 5851.14 (1789.45 to 15591.22) | 8230.97 (2698.99 to 21531.26) | 10405.81 (3662.08 to 26756.03) | 12022.57 (4402.51 to 30578.58) | 13194.15 (4924.10 to 33352.67) | 14122.06 (5339.70 to 35506.24) | 14894.27 (5662.20 to 37303.98) | 15574.06 (5996.10 to 38944.21) | 16203.96 (6309.80 to 40399.26) | 16836.36 (6563.08 to 41895.23) | 17542.46 (6840.23 to 43578.22) | 17881.50 (6979.13 to 44379.76) | 17881.52 (6978.68 to 44386.40) | 17881.43 (6997.27 to 44394.58) | 17882.19 (7010.82 to 44388.33) | 17882.22 (6992.15 to 44386.54) | 17882.18 (7003.43 to 44394.38) | 17881.65 (6994.13 to 44380.93) |
| Gabon | Both | DALY rates | 9.90 (5.76 to 15.62) | 29.22 (16.11 to 52.46) | 30.37 (15.57 to 56.18) | 32.02 (17.26 to 59.34) | 33.47 (18.45 to 59.44) | 34.60 (18.69 to 60.08) | 35.88 (19.56 to 65.20) | 36.73 (18.92 to 64.90) | 37.55 (19.88 to 65.78) | 37.83 (20.06 to 65.83) | 38.35 (20.75 to 68.68) | 39.04 (21.00 to 71.03) | 39.87 (21.74 to 70.42) | 39.38 (21.28 to 68.75) | 37.59 (20.70 to 65.89) | 34.52 (19.25 to 58.10) | 31.45 (18.25 to 54.61) | 29.35 (17.56 to 48.23) | 27.41 (16.73 to 44.26) | 25.22 (15.31 to 39.00) |
| Gabon | Both | Prevalence | 280.46 (109.08 to 975.13) | 712.96 (261.24 to 2452.35) | 928.04 (286.18 to 3446.35) | 1177.16 (315.28 to 4592.50) | 1390.08 (340.29 to 5568.31) | 1547.28 (358.57 to 6281.68) | 1661.84 (372.75 to 6795.63) | 1755.40 (385.37 to 7208.87) | 1833.99 (396.88 to 7558.55) | 1902.16 (405.33 to 7862.15) | 1966.90 (414.29 to 8149.77) | 2035.50 (425.59 to 8447.59) | 2112.97 (437.34 to 8785.76) | 2149.12 (442.27 to 8947.39) | 2138.52 (434.21 to 8935.59) | 2117.38 (420.31 to 8905.38) | 2096.28 (407.16 to 8874.69) | 2082.55 (401.02 to 8852.93) | 2068.62 (392.11 to 8831.66) | 2052.93 (382.60 to 8804.16) |
| Gabon | Female | DALY rates | 11.62 (6.67 to 18.71) | 19.24 (10.89 to 29.43) | 19.49 (11.40 to 30.53) | 19.66 (11.82 to 31.01) | 19.94 (11.57 to 31.00) | 19.75 (11.92 to 29.63) | 19.81 (11.53 to 30.76) | 19.88 (12.14 to 30.10) | 20.05 (12.39 to 30.29) | 19.80 (11.71 to 29.42) | 19.95 (12.10 to 30.91) | 19.66 (11.40 to 29.66) | 20.03 (12.25 to 30.13) | 19.67 (12.14 to 29.79) | 19.46 (12.00 to 29.02) | 19.43 (11.76 to 29.97) | 18.53 (11.14 to 28.45) | 18.36 (11.07 to 28.02) | 17.99 (10.51 to 26.17) | 17.55 (10.63 to 26.00) |
| Gabon | Female | Prevalence | 298.44 (125.13 to 998.43) | 637.90 (219.77 to 2336.83) | 844.51 (237.42 to 3312.32) | 1082.65 (256.53 to 4435.41) | 1285.08 (274.29 to 5390.47) | 1432.92 (285.49 to 6087.87) | 1538.58 (294.77 to 6586.02) | 1623.61 (300.96 to 6987.87) | 1695.43 (307.03 to 7325.56) | 1758.28 (311.74 to 7622.21) | 1817.15 (316.90 to 7899.01) | 1878.01 (321.01 to 8186.65) | 1947.29 (326.81 to 8513.43) | 1980.96 (329.55 to 8671.50) | 1980.95 (330.26 to 8672.70) | 1980.95 (329.99 to 8672.37) | 1980.94 (330.07 to 8672.41) | 1980.95 (330.77 to 8671.93) | 1980.96 (329.23 to 8671.63) | 1980.97 (329.40 to 8671.96) |
| Gabon | Male | DALY rates | 8.21 (4.39 to 13.18) | 39.52 (18.24 to 80.56) | 42.18 (18.37 to 87.12) | 46.01 (20.87 to 96.08) | 49.18 (21.79 to 98.74) | 51.79 (23.76 to 103.50) | 53.91 (23.72 to 107.61) | 54.92 (23.73 to 109.29) | 56.03 (24.46 to 109.06) | 56.74 (25.04 to 110.75) | 57.31 (25.90 to 112.93) | 58.28 (26.44 to 115.60) | 58.98 (27.45 to 116.58) | 58.46 (27.33 to 113.88) | 57.54 (26.29 to 111.16) | 56.02 (26.57 to 107.54) | 55.61 (26.15 to 106.84) | 54.18 (25.85 to 103.18) | 53.55 (25.81 to 104.10) | 52.80 (25.72 to 102.43) |
| Gabon | Male | Prevalence | 262.75 (91.16 to 952.19) | 790.52 (294.39 to 2533.20) | 1018.72 (325.53 to 3561.32) | 1284.15 (366.67 to 4726.30) | 1511.98 (408.93 to 5719.71) | 1679.71 (440.30 to 6442.71) | 1800.20 (462.71 to 6964.28) | 1897.70 (480.23 to 7380.41) | 1980.37 (496.32 to 7735.17) | 2053.03 (510.36 to 8041.80) | 2121.19 (523.23 to 8334.89) | 2191.86 (538.26 to 8633.22) | 2272.61 (554.11 to 8973.84) | 2311.86 (559.12 to 9138.28) | 2311.86 (561.31 to 9139.13) | 2311.84 (560.99 to 9139.52) | 2311.93 (561.43 to 9141.60) | 2311.86 (560.88 to 9140.32) | 2311.93 (560.92 to 9139.47) | 2311.89 (561.32 to 9140.56) |
| Ghana | Both | DALY rates | 9.56 (5.67 to 14.39) | 27.85 (14.10 to 52.50) | 28.78 (15.40 to 52.27) | 29.28 (15.43 to 51.68) | 29.51 (16.21 to 55.48) | 29.63 (16.19 to 51.81) | 29.60 (15.44 to 54.86) | 29.94 (16.25 to 52.52) | 30.21 (16.79 to 51.57) | 30.19 (16.40 to 53.48) | 30.04 (16.44 to 54.88) | 29.58 (17.07 to 51.11) | 29.50 (16.42 to 52.46) | 29.23 (15.78 to 51.22) | 28.41 (16.26 to 50.08) | 27.41 (15.79 to 46.33) | 26.32 (14.96 to 45.52) | 25.57 (15.57 to 42.14) | 24.26 (14.34 to 40.09) | 22.18 (13.15 to 34.64) |
| Ghana | Both | Prevalence | 182.85 (108.50 to 399.75) | 473.01 (252.80 to 1038.07) | 580.32 (279.61 to 1371.54) | 702.82 (304.61 to 1779.37) | 804.60 (325.82 to 2123.85) | 878.33 (343.76 to 2369.31) | 932.71 (357.21 to 2548.45) | 976.83 (367.82 to 2690.87) | 1014.82 (377.41 to 2812.85) | 1047.52 (385.29 to 2919.93) | 1075.20 (391.42 to 3016.18) | 1104.65 (397.61 to 3117.21) | 1140.83 (405.66 to 3235.42) | 1157.08 (409.07 to 3290.09) | 1154.87 (407.99 to 3287.43) | 1150.17 (405.31 to 3281.99) | 1144.77 (402.72 to 3275.00) | 1140.35 (400.27 to 3269.68) | 1132.44 (390.21 to 3260.04) | 1120.89 (381.03 to 3247.53) |
| Ghana | Female | DALY rates | 11.30 (6.39 to 18.01) | 18.85 (11.06 to 29.85) | 19.16 (11.18 to 29.79) | 19.18 (10.84 to 29.66) | 18.81 (11.30 to 30.11) | 19.13 (10.94 to 29.68) | 18.80 (10.97 to 29.11) | 18.98 (11.38 to 29.41) | 18.88 (11.26 to 28.14) | 18.83 (10.85 to 28.72) | 18.88 (11.39 to 29.06) | 18.70 (11.24 to 28.27) | 18.77 (10.81 to 28.49) | 18.52 (10.85 to 28.27) | 18.26 (10.96 to 28.22) | 17.85 (10.97 to 26.63) | 17.64 (10.68 to 26.00) | 17.52 (11.14 to 26.51) | 17.11 (10.22 to 25.61) | 16.37 (9.53 to 24.66) |
| Ghana | Female | Prevalence | 199.36 (124.84 to 415.13) | 406.37 (222.44 to 943.75) | 508.87 (239.19 to 1295.67) | 626.49 (258.99 to 1699.20) | 726.21 (277.61 to 2041.76) | 798.94 (289.27 to 2291.39) | 850.92 (299.04 to 2469.67) | 892.68 (305.99 to 2613.11) | 928.02 (312.61 to 2734.30) | 958.92 (317.68 to 2840.12) | 987.88 (322.89 to 2939.96) | 1018.03 (327.88 to 3043.67) | 1052.56 (333.65 to 3161.54) | 1068.84 (336.92 to 3217.44) | 1068.84 (336.47 to 3217.35) | 1068.83 (336.25 to 3217.29) | 1068.84 (336.44 to 3217.44) | 1068.84 (336.89 to 3217.36) | 1068.83 (336.50 to 3217.20) | 1068.85 (336.49 to 3217.16) |
| Ghana | Male | DALY rates | 7.90 (4.29 to 12.99) | 36.49 (15.12 to 78.20) | 38.04 (15.83 to 82.34) | 39.24 (17.78 to 81.43) | 40.89 (18.23 to 90.98) | 41.53 (18.83 to 83.16) | 41.92 (18.11 to 94.64) | 42.38 (17.47 to 86.64) | 42.84 (18.99 to 86.81) | 42.80 (19.35 to 87.75) | 43.20 (19.04 to 89.26) | 43.06 (20.17 to 86.11) | 42.84 (19.32 to 86.14) | 42.80 (18.79 to 84.49) | 41.85 (18.86 to 85.71) | 41.37 (18.59 to 81.55) | 40.50 (18.68 to 87.25) | 40.04 (18.67 to 80.93) | 39.58 (17.60 to 78.70) | 38.67 (18.23 to 79.06) |
| Ghana | Male | Prevalence | 167.07 (92.16 to 385.15) | 537.05 (269.79 to 1103.46) | 649.07 (300.44 to 1489.22) | 778.09 (332.19 to 1904.85) | 887.93 (362.50 to 2236.27) | 968.27 (385.97 to 2476.81) | 1025.92 (404.07 to 2650.53) | 1072.28 (417.64 to 2790.28) | 1111.56 (427.81 to 2908.64) | 1145.99 (437.12 to 3011.52) | 1178.27 (446.43 to 3113.94) | 1211.95 (454.93 to 3222.18) | 1250.63 (465.38 to 3345.96) | 1268.83 (470.02 to 3404.19) | 1268.82 (469.41 to 3404.58) | 1268.88 (470.19 to 3404.23) | 1268.82 (469.63 to 3403.73) | 1268.84 (469.40 to 3404.01) | 1268.85 (469.23 to 3404.09) | 1268.85 (469.21 to 3403.61) |
| Guinea | Both | DALY rates | 9.40 (5.94 to 13.79) | 27.78 (14.19 to 51.43) | 28.17 (14.91 to 51.52) | 28.42 (16.00 to 50.85) | 28.54 (15.37 to 50.80) | 28.74 (14.74 to 53.71) | 28.58 (15.71 to 50.90) | 28.71 (16.35 to 49.54) | 29.05 (15.69 to 50.59) | 29.43 (16.39 to 53.30) | 29.96 (16.27 to 52.93) | 30.22 (16.09 to 55.11) | 30.74 (16.59 to 54.34) | 30.44 (16.68 to 58.72) | 29.79 (16.08 to 54.48) | 29.36 (15.39 to 51.46) | 28.08 (15.49 to 52.46) | 26.80 (15.29 to 45.50) | 25.64 (15.05 to 44.44) | 24.15 (14.26 to 41.07) |
| Guinea | Both | Prevalence | 170.61 (103.36 to 377.44) | 448.16 (246.11 to 973.57) | 542.47 (268.25 to 1298.31) | 650.05 (293.19 to 1677.12) | 740.21 (311.28 to 1999.55) | 806.28 (320.95 to 2236.44) | 854.15 (328.62 to 2402.94) | 892.75 (334.90 to 2537.14) | 927.42 (342.84 to 2653.41) | 959.48 (350.30 to 2757.95) | 990.92 (359.22 to 2857.36) | 1022.70 (367.03 to 2959.94) | 1058.38 (375.88 to 3076.05) | 1076.46 (380.95 to 3131.76) | 1074.64 (379.80 to 3128.41) | 1073.71 (379.50 to 3128.48) | 1069.01 (376.40 to 3120.54) | 1061.85 (372.44 to 3110.78) | 1054.42 (367.24 to 3104.42) | 1045.58 (361.48 to 3096.50) |
| Guinea | Female | DALY rates | 11.09 (6.54 to 16.52) | 19.08 (10.74 to 29.80) | 19.10 (10.80 to 30.45) | 18.96 (10.63 to 29.26) | 19.11 (10.91 to 28.57) | 18.98 (10.46 to 30.17) | 19.03 (11.06 to 30.39) | 18.85 (11.12 to 28.69) | 19.08 (11.39 to 29.97) | 18.77 (11.39 to 29.17) | 18.83 (11.07 to 28.28) | 18.40 (10.91 to 27.77) | 18.39 (11.52 to 29.16) | 18.02 (10.88 to 28.78) | 18.12 (10.86 to 27.87) | 17.96 (11.40 to 27.42) | 17.37 (10.21 to 26.78) | 17.03 (10.09 to 25.76) | 16.70 (10.39 to 24.89) | 16.32 (9.56 to 24.69) |
| Guinea | Female | Prevalence | 186.84 (118.64 to 395.42) | 382.81 (211.17 to 907.65) | 473.60 (227.05 to 1234.31) | 578.53 (244.88 to 1610.86) | 668.25 (259.55 to 1934.19) | 733.84 (270.60 to 2169.46) | 780.35 (278.98 to 2336.91) | 817.67 (284.43 to 2470.41) | 849.28 (289.68 to 2583.93) | 877.12 (295.11 to 2684.74) | 903.04 (298.41 to 2778.07) | 929.98 (303.37 to 2874.81) | 960.87 (307.54 to 2985.59) | 975.36 (309.60 to 3037.70) | 975.38 (309.37 to 3037.12) | 975.38 (310.32 to 3038.08) | 975.38 (309.81 to 3038.03) | 975.36 (310.99 to 3037.60) | 975.38 (310.64 to 3037.62) | 975.37 (309.64 to 3037.93) |
| Guinea | Male | DALY rates | 7.77 (4.25 to 12.87) | 36.19 (16.33 to 78.49) | 37.11 (16.04 to 79.39) | 38.35 (16.15 to 78.50) | 39.51 (17.23 to 83.23) | 40.84 (17.57 to 87.67) | 40.65 (17.99 to 84.68) | 41.31 (19.30 to 86.50) | 41.34 (18.06 to 83.42) | 41.75 (19.51 to 87.56) | 41.66 (19.62 to 84.15) | 41.80 (18.48 to 86.97) | 42.07 (19.80 to 87.63) | 41.20 (18.25 to 89.14) | 40.29 (18.47 to 80.18) | 39.83 (17.84 to 79.01) | 38.95 (18.31 to 83.23) | 38.35 (17.25 to 76.94) | 38.02 (18.09 to 75.98) | 37.34 (17.02 to 75.69) |
| Guinea | Male | Prevalence | 155.06 (88.60 to 359.56) | 511.28 (260.35 to 1090.65) | 610.36 (291.77 to 1382.51) | 725.25 (323.14 to 1768.31) | 823.80 (346.10 to 2099.80) | 896.11 (365.39 to 2343.24) | 947.47 (378.23 to 2512.54) | 988.76 (390.56 to 2648.42) | 1023.84 (400.23 to 2763.33) | 1054.69 (408.38 to 2866.56) | 1083.44 (415.66 to 2963.98) | 1113.50 (424.37 to 3064.37) | 1147.83 (433.58 to 3177.16) | 1164.02 (438.35 to 3228.96) | 1163.99 (437.96 to 3228.53) | 1164.02 (437.78 to 3228.75) | 1164.04 (438.49 to 3227.52) | 1163.99 (437.97 to 3227.63) | 1164.03 (438.05 to 3229.11) | 1164.00 (438.21 to 3229.64) |
| Guinea-Bissau | Both | DALY rates | 9.50 (5.85 to 14.53) | 29.15 (15.98 to 54.53) | 30.28 (16.03 to 55.77) | 31.95 (16.89 to 59.49) | 32.82 (17.95 to 58.17) | 33.82 (17.78 to 60.93) | 33.91 (18.16 to 58.51) | 34.02 (18.42 to 60.50) | 34.67 (18.74 to 60.89) | 34.65 (19.15 to 62.29) | 35.53 (19.72 to 62.37) | 35.92 (19.31 to 63.69) | 35.54 (19.49 to 60.91) | 35.63 (19.79 to 62.87) | 34.45 (18.90 to 60.14) | 32.93 (18.55 to 55.69) | 31.01 (17.26 to 52.38) | 30.04 (17.02 to 49.46) | 28.76 (16.41 to 48.16) | 26.84 (15.32 to 45.00) |
| Guinea-Bissau | Both | Prevalence | 247.26 (98.51 to 905.24) | 647.43 (243.29 to 2322.09) | 833.68 (262.22 to 3249.70) | 1048.66 (283.41 to 4319.91) | 1232.09 (304.35 to 5233.34) | 1364.78 (315.99 to 5899.17) | 1457.88 (324.37 to 6368.92) | 1533.53 (332.10 to 6747.12) | 1599.80 (340.20 to 7071.19) | 1659.06 (347.23 to 7359.05) | 1714.51 (353.53 to 7627.28) | 1772.22 (360.91 to 7904.83) | 1837.11 (368.49 to 8223.06) | 1867.06 (370.65 to 8372.97) | 1860.39 (366.20 to 8366.82) | 1851.38 (360.56 to 8357.85) | 1842.12 (355.20 to 8348.79) | 1835.40 (351.49 to 8341.49) | 1828.85 (347.48 to 8336.13) | 1816.03 (338.48 to 8322.27) |
| Guinea-Bissau | Female | DALY rates | 11.20 (6.61 to 16.96) | 19.32 (11.35 to 29.30) | 19.09 (11.13 to 30.64) | 19.54 (11.37 to 30.52) | 19.56 (11.67 to 30.86) | 19.80 (11.97 to 30.52) | 19.81 (11.91 to 31.05) | 19.69 (11.20 to 30.38) | 19.71 (12.20 to 30.78) | 19.30 (12.04 to 28.53) | 19.66 (11.18 to 30.50) | 19.76 (11.01 to 31.40) | 19.16 (11.61 to 29.23) | 19.09 (11.59 to 28.43) | 19.07 (11.11 to 28.75) | 18.96 (11.52 to 27.93) | 18.30 (10.67 to 28.23) | 18.38 (11.33 to 28.12) | 17.93 (10.60 to 26.99) | 17.56 (11.01 to 27.14) |
| Guinea-Bissau | Female | Prevalence | 263.77 (114.13 to 926.45) | 573.13 (200.98 to 2225.19) | 750.74 (210.73 to 3148.43) | 955.68 (222.69 to 4213.66) | 1130.81 (230.43 to 5123.76) | 1258.64 (237.76 to 5787.20) | 1349.20 (240.55 to 6258.04) | 1421.97 (243.78 to 6636.39) | 1483.57 (247.15 to 6955.59) | 1537.75 (249.37 to 7238.50) | 1588.53 (251.65 to 7501.47) | 1641.06 (254.48 to 7774.82) | 1701.05 (257.36 to 8085.94) | 1729.87 (258.97 to 8235.31) | 1729.86 (258.83 to 8235.70) | 1729.90 (258.93 to 8235.79) | 1729.87 (258.35 to 8235.43) | 1729.86 (259.27 to 8235.41) | 1729.84 (258.78 to 8235.80) | 1729.86 (259.04 to 8234.83) |
| Guinea-Bissau | Male | DALY rates | 7.86 (4.23 to 12.95) | 38.69 (17.30 to 82.40) | 41.28 (18.04 to 85.71) | 44.50 (19.78 to 95.48) | 46.78 (20.88 to 94.84) | 49.41 (21.75 to 102.60) | 50.42 (22.03 to 100.32) | 51.29 (24.03 to 102.05) | 52.48 (23.04 to 101.69) | 52.51 (24.86 to 105.16) | 53.65 (23.92 to 105.01) | 53.96 (23.82 to 106.86) | 53.67 (25.80 to 105.35) | 54.21 (24.37 to 108.37) | 53.40 (24.43 to 106.78) | 52.46 (24.38 to 104.78) | 51.28 (23.79 to 99.93) | 50.56 (23.77 to 99.50) | 49.80 (23.28 to 95.49) | 48.92 (22.99 to 98.76) |
| Guinea-Bissau | Male | Prevalence | 231.38 (82.08 to 884.83) | 719.56 (258.25 to 2438.69) | 915.17 (293.20 to 3379.35) | 1142.67 (329.53 to 4466.48) | 1338.71 (360.12 to 5397.37) | 1482.73 (381.07 to 6080.62) | 1585.19 (396.46 to 6563.25) | 1667.92 (406.92 to 6950.31) | 1738.17 (418.80 to 7276.92) | 1800.14 (428.39 to 7567.38) | 1858.35 (437.16 to 7839.03) | 1918.69 (446.97 to 8116.40) | 1987.82 (457.62 to 8439.95) | 2021.11 (463.93 to 8592.65) | 2021.14 (464.14 to 8593.16) | 2021.20 (464.34 to 8594.04) | 2021.07 (463.62 to 8594.14) | 2021.10 (464.64 to 8592.81) | 2021.12 (464.40 to 8595.78) | 2021.13 (462.54 to 8595.41) |
| Guyana | Both | DALY rates | 12.89 (8.29 to 18.92) | 118.93 (68.41 to 199.38) | 203.96 (129.19 to 304.91) | 310.73 (205.08 to 445.54) | 385.54 (254.46 to 542.06) | 413.67 (279.85 to 571.02) | 425.10 (286.42 to 595.45) | 430.90 (290.54 to 596.40) | 436.63 (297.05 to 588.35) | 443.74 (305.29 to 603.03) | 447.88 (307.77 to 612.58) | 437.56 (303.05 to 588.17) | 434.70 (299.08 to 590.99) | 421.44 (293.00 to 570.06) | 412.67 (291.90 to 559.47) | 391.67 (274.54 to 523.93) | 367.04 (258.81 to 484.97) | 351.68 (247.04 to 461.89) | 355.26 (252.87 to 469.24) | 419.18 (295.42 to 566.07) |
| Guyana | Both | Prevalence | 2310.62 (641.84 to 5073.17) | 6353.69 (2216.38 to 13162.40) | 9509.33 (3484.06 to 19435.82) | 13225.93 (5058.38 to 26714.56) | 16248.84 (6183.96 to 32727.53) | 18199.86 (6804.91 to 36785.13) | 19570.92 (7266.14 to 39715.98) | 20664.30 (7609.65 to 42084.17) | 21608.57 (7924.70 to 44092.45) | 22439.21 (8199.26 to 45838.65) | 23214.10 (8458.13 to 47486.24) | 23900.04 (8611.56 to 49077.36) | 24737.43 (8836.36 to 50941.38) | 25073.97 (8890.05 to 51775.58) | 25047.34 (8865.01 to 51738.10) | 24902.29 (8720.21 to 51586.95) | 24721.79 (8534.85 to 51411.17) | 24635.00 (8454.29 to 51319.37) | 24732.80 (8547.17 to 51419.72) | 25434.14 (9242.13 to 52126.77) |
| Guyana | Female | DALY rates | 15.24 (9.43 to 22.50) | 28.79 (17.87 to 42.71) | 34.93 (21.10 to 52.28) | 43.48 (27.60 to 65.22) | 52.81 (33.89 to 76.30) | 60.03 (38.94 to 88.21) | 65.46 (42.69 to 96.55) | 71.10 (46.73 to 102.63) | 74.72 (48.25 to 107.69) | 78.86 (50.67 to 109.44) | 82.35 (53.63 to 117.82) | 85.94 (56.83 to 122.96) | 91.45 (60.37 to 135.17) | 93.02 (61.35 to 129.66) | 92.74 (60.67 to 130.00) | 91.43 (61.63 to 128.89) | 90.86 (61.78 to 130.04) | 88.89 (61.76 to 124.66) | 87.35 (59.99 to 123.13) | 85.04 (59.08 to 120.30) |
| Guyana | Female | Prevalence | 2337.99 (659.44 to 5103.70) | 5635.52 (1535.18 to 12400.34) | 8146.93 (2173.45 to 18006.24) | 11052.20 (2920.12 to 24471.34) | 13529.05 (3568.98 to 29957.44) | 15293.09 (4039.03 to 33849.13) | 16600.95 (4390.78 to 36726.03) | 17671.60 (4681.94 to 39070.05) | 18567.01 (4927.74 to 41033.74) | 19348.41 (5143.46 to 42739.07) | 20084.55 (5349.07 to 44343.24) | 20853.18 (5566.05 to 46012.85) | 21728.68 (5815.36 to 47911.76) | 22150.59 (5932.57 to 48826.25) | 22150.74 (5934.78 to 48825.41) | 22150.76 (5933.54 to 48826.04) | 22150.56 (5930.60 to 48823.66) | 22150.72 (5931.09 to 48821.31) | 22150.61 (5931.62 to 48823.67) | 22150.67 (5934.02 to 48824.24) |
| Guyana | Male | DALY rates | 10.64 (6.28 to 17.05) | 206.43 (113.19 to 357.10) | 368.62 (229.22 to 564.02) | 573.62 (377.32 to 820.14) | 714.94 (470.27 to 1010.36) | 780.21 (523.85 to 1078.70) | 809.70 (535.21 to 1148.36) | 822.71 (552.82 to 1145.24) | 824.05 (556.28 to 1128.57) | 824.16 (563.49 to 1120.47) | 819.89 (558.72 to 1130.14) | 813.65 (559.51 to 1098.58) | 809.39 (553.11 to 1094.53) | 799.38 (554.15 to 1086.54) | 787.17 (549.65 to 1065.86) | 777.52 (544.31 to 1042.88) | 766.23 (534.55 to 1027.59) | 753.97 (527.21 to 988.31) | 739.72 (520.93 to 993.89) | 724.80 (511.65 to 989.71) |
| Guyana | Male | Prevalence | 2284.58 (622.17 to 5044.14) | 7050.83 (2914.51 to 14080.23) | 10836.45 (4859.53 to 20983.48) | 15364.20 (7188.71 to 29047.84) | 18941.40 (8924.14 to 35536.92) | 21212.72 (9891.31 to 39836.04) | 22747.04 (10442.18 to 42921.85) | 23923.33 (10805.93 to 45367.23) | 24864.63 (11038.06 to 47366.88) | 25661.64 (11287.87 to 49070.23) | 26399.07 (11564.52 to 50684.90) | 27158.89 (11803.82 to 52355.10) | 28021.90 (12065.93 to 54248.62) | 28438.22 (12217.18 to 55169.70) | 28437.93 (12221.84 to 55147.52) | 28438.40 (12217.65 to 55135.11) | 28438.12 (12203.04 to 55151.05) | 28438.12 (12214.47 to 55143.58) | 28438.30 (12210.00 to 55145.10) | 28437.39 (12209.22 to 55147.45) |
| Haiti | Both | DALY rates | 9.57 (5.80 to 14.29) | 28.51 (14.79 to 51.28) | 29.21 (15.88 to 53.21) | 30.57 (16.65 to 55.76) | 32.08 (17.25 to 59.86) | 32.32 (16.84 to 57.90) | 32.83 (17.80 to 60.66) | 32.84 (17.04 to 59.82) | 32.86 (17.10 to 57.18) | 33.09 (17.94 to 60.07) | 33.49 (18.13 to 59.70) | 33.48 (18.76 to 58.26) | 33.40 (19.11 to 57.16) | 33.11 (18.25 to 57.91) | 32.91 (18.04 to 58.44) | 32.59 (17.58 to 57.86) | 32.05 (17.64 to 56.59) | 31.82 (17.97 to 55.42) | 31.99 (16.82 to 55.80) | 30.46 (17.12 to 53.22) |
| Haiti | Both | Prevalence | 223.88 (142.37 to 415.49) | 586.60 (354.80 to 1090.42) | 746.51 (427.67 to 1475.14) | 929.03 (505.12 to 1905.64) | 1084.65 (572.23 to 2272.21) | 1198.26 (619.06 to 2540.66) | 1278.16 (652.25 to 2729.12) | 1341.45 (679.54 to 2878.18) | 1396.97 (704.62 to 3008.16) | 1447.23 (727.16 to 3124.16) | 1494.23 (748.07 to 3231.82) | 1541.32 (769.24 to 3342.07) | 1594.67 (792.49 to 3466.57) | 1619.92 (803.37 to 3525.78) | 1621.44 (803.91 to 3527.06) | 1623.01 (805.14 to 3532.59) | 1623.04 (805.70 to 3530.27) | 1627.37 (807.33 to 3537.56) | 1633.09 (810.09 to 3546.30) | 1626.56 (807.53 to 3536.42) |
| Haiti | Female | DALY rates | 11.27 (6.68 to 17.65) | 18.94 (10.82 to 29.96) | 18.93 (10.89 to 29.77) | 19.30 (11.24 to 30.16) | 19.53 (11.69 to 29.76) | 19.39 (11.49 to 30.21) | 19.42 (11.27 to 29.73) | 19.67 (11.49 to 31.10) | 19.37 (11.54 to 30.00) | 19.24 (11.17 to 29.43) | 19.10 (11.71 to 28.64) | 19.19 (11.26 to 29.74) | 18.91 (11.86 to 28.59) | 18.77 (11.77 to 27.90) | 18.79 (11.17 to 28.99) | 18.47 (11.03 to 27.72) | 18.11 (10.54 to 27.67) | 17.75 (10.99 to 27.25) | 17.76 (10.63 to 25.99) | 17.18 (10.95 to 25.02) |
| Haiti | Female | Prevalence | 240.23 (158.11 to 433.17) | 515.57 (311.38 to 997.84) | 668.27 (370.85 to 1369.76) | 842.15 (438.00 to 1793.08) | 990.54 (495.87 to 2155.33) | 1099.34 (537.97 to 2420.30) | 1176.51 (568.34 to 2607.84) | 1238.20 (592.66 to 2757.98) | 1290.59 (612.74 to 2886.01) | 1336.55 (630.05 to 2997.46) | 1379.87 (647.42 to 3103.87) | 1424.62 (664.41 to 3212.20) | 1475.74 (684.55 to 3336.91) | 1500.06 (692.80 to 3396.19) | 1500.05 (692.88 to 3395.84) | 1500.06 (693.04 to 3396.20) | 1500.04 (692.79 to 3395.44) | 1500.05 (693.10 to 3396.59) | 1500.05 (692.94 to 3396.30) | 1500.03 (692.95 to 3396.66) |
| Haiti | Male | DALY rates | 7.92 (4.16 to 12.90) | 37.90 (17.12 to 79.36) | 39.37 (17.27 to 83.03) | 41.84 (18.47 to 87.25) | 44.91 (19.61 to 94.57) | 45.95 (18.92 to 95.61) | 47.46 (21.20 to 97.76) | 47.79 (20.82 to 97.93) | 48.16 (21.04 to 96.39) | 48.41 (21.99 to 98.83) | 49.17 (22.11 to 98.64) | 49.20 (23.50 to 98.12) | 49.62 (22.35 to 96.36) | 49.34 (22.34 to 95.68) | 48.52 (22.46 to 96.17) | 47.80 (21.75 to 96.27) | 47.08 (21.62 to 95.46) | 45.98 (22.40 to 93.38) | 45.10 (19.94 to 88.67) | 43.99 (20.16 to 87.50) |
| Haiti | Male | Prevalence | 207.99 (124.95 to 398.35) | 656.24 (383.52 to 1190.63) | 823.83 (463.50 to 1593.05) | 1015.84 (550.22 to 2008.50) | 1180.83 (628.82 to 2395.42) | 1302.51 (686.44 to 2680.02) | 1389.08 (728.16 to 2879.86) | 1458.55 (760.18 to 3041.82) | 1517.68 (788.41 to 3177.94) | 1569.73 (813.52 to 3298.34) | 1618.82 (837.09 to 3407.45) | 1669.65 (861.63 to 3523.95) | 1727.84 (888.89 to 3654.07) | 1755.52 (900.75 to 3717.00) | 1755.55 (900.46 to 3717.48) | 1755.53 (901.10 to 3720.20) | 1755.59 (901.62 to 3716.78) | 1755.55 (901.26 to 3717.32) | 1755.60 (900.83 to 3719.51) | 1755.58 (901.56 to 3717.85) |
| India | Both | DALY rates | 10.12 (6.84 to 14.28) | 33.75 (18.70 to 61.47) | 38.48 (21.39 to 68.16) | 44.85 (25.11 to 79.25) | 50.50 (28.74 to 87.30) | 53.74 (30.61 to 90.69) | 55.53 (31.37 to 93.88) | 56.90 (32.61 to 95.58) | 58.89 (33.89 to 97.23) | 60.41 (35.25 to 99.70) | 60.35 (35.35 to 99.62) | 60.01 (35.20 to 97.87) | 60.98 (36.72 to 98.10) | 61.91 (37.51 to 98.89) | 61.73 (37.23 to 98.68) | 59.64 (36.93 to 95.77) | 54.44 (33.95 to 84.80) | 49.09 (30.47 to 76.29) | 45.28 (28.61 to 70.90) | 36.49 (23.44 to 55.92) |
| India | Both | Prevalence | 451.70 (398.68 to 508.68) | 1134.57 (973.46 to 1375.14) | 1557.90 (1338.78 to 1859.17) | 2048.67 (1760.16 to 2429.02) | 2438.58 (2102.06 to 2865.54) | 2649.53 (2291.02 to 3091.70) | 2761.05 (2398.64 to 3203.95) | 2869.98 (2499.54 to 3314.35) | 2995.87 (2611.18 to 3453.52) | 3086.31 (2692.50 to 3551.13) | 3127.75 (2734.71 to 3588.17) | 3181.57 (2788.29 to 3643.69) | 3307.18 (2902.96 to 3780.27) | 3433.68 (3010.16 to 3922.78) | 3517.09 (3075.52 to 4016.38) | 3514.01 (3072.90 to 4008.66) | 3395.90 (2977.37 to 3875.29) | 3205.74 (2822.26 to 3659.10) | 3078.20 (2701.30 to 3506.30) | 2914.32 (2542.87 to 3368.15) |
| India | Female | DALY rates | 11.93 (7.96 to 16.82) | 19.86 (13.58 to 28.15) | 20.45 (13.65 to 29.24) | 21.15 (14.32 to 29.71) | 21.58 (14.58 to 31.01) | 21.75 (14.80 to 30.66) | 21.83 (14.73 to 30.93) | 21.77 (14.95 to 30.66) | 21.93 (15.04 to 30.69) | 21.84 (14.81 to 30.48) | 21.75 (15.13 to 30.16) | 21.61 (14.92 to 30.71) | 21.56 (14.70 to 30.77) | 21.46 (14.74 to 29.94) | 21.21 (14.67 to 29.11) | 20.66 (14.16 to 28.36) | 20.12 (14.09 to 27.76) | 19.32 (13.70 to 25.98) | 18.71 (13.40 to 25.13) | 18.34 (13.19 to 24.65) |
| India | Female | Prevalence | 468.56 (414.83 to 525.28) | 1025.58 (899.78 to 1151.97) | 1414.47 (1232.10 to 1597.52) | 1854.74 (1607.00 to 2105.07) | 2197.66 (1902.10 to 2503.06) | 2380.18 (2065.56 to 2719.21) | 2478.16 (2158.33 to 2823.58) | 2582.97 (2253.90 to 2939.58) | 2689.01 (2345.29 to 3060.89) | 2748.02 (2401.28 to 3128.56) | 2781.96 (2438.71 to 3163.89) | 2843.02 (2496.10 to 3235.19) | 2966.03 (2604.52 to 3377.65) | 3080.34 (2702.49 to 3509.40) | 3141.80 (2757.21 to 3575.64) | 3127.47 (2748.44 to 3545.97) | 3037.51 (2678.39 to 3438.24) | 2888.78 (2549.72 to 3276.20) | 2761.27 (2437.03 to 3140.26) | 2766.88 (2440.70 to 3145.34) |
| India | Male | DALY rates | 8.49 (5.58 to 12.11) | 46.44 (22.25 to 93.30) | 54.91 (26.37 to 105.84) | 66.60 (33.09 to 127.75) | 77.69 (39.48 to 146.16) | 84.21 (43.89 to 153.58) | 87.39 (45.32 to 156.28) | 90.20 (47.17 to 161.00) | 93.99 (49.64 to 166.07) | 97.17 (53.16 to 171.51) | 98.28 (53.62 to 172.48) | 98.94 (54.15 to 170.95) | 102.15 (56.47 to 173.02) | 104.62 (59.04 to 176.71) | 105.39 (59.35 to 177.60) | 103.14 (58.79 to 175.16) | 98.84 (56.07 to 164.81) | 91.28 (52.08 to 153.95) | 87.09 (49.56 to 148.34) | 76.23 (42.55 to 131.87) |
| India | Male | Prevalence | 436.38 (383.42 to 494.83) | 1234.11 (1006.60 to 1626.99) | 1688.66 (1402.25 to 2144.22) | 2226.68 (1861.87 to 2752.50) | 2665.19 (2235.75 to 3252.35) | 2906.05 (2450.43 to 3520.70) | 3028.41 (2566.35 to 3660.36) | 3142.01 (2674.29 to 3787.05) | 3287.34 (2801.29 to 3958.68) | 3408.76 (2911.82 to 4101.65) | 3467.52 (2965.87 to 4163.74) | 3524.84 (3020.23 to 4224.49) | 3663.45 (3147.86 to 4364.22) | 3806.80 (3277.56 to 4514.93) | 3921.42 (3377.23 to 4646.83) | 3945.28 (3396.68 to 4671.43) | 3859.56 (3318.91 to 4571.46) | 3654.95 (3144.21 to 4338.16) | 3576.66 (3071.93 to 4255.74) | 3237.22 (2749.66 to 3974.89) |
| Indonesia | Both | DALY rates | 9.90 (6.67 to 14.25) | 30.13 (16.99 to 56.31) | 32.53 (18.22 to 58.71) | 35.26 (19.86 to 63.05) | 37.64 (21.18 to 66.15) | 39.71 (22.21 to 69.45) | 41.02 (23.01 to 72.54) | 41.94 (23.43 to 73.46) | 42.48 (23.99 to 73.72) | 42.78 (24.47 to 74.64) | 43.08 (24.60 to 75.19) | 43.30 (24.95 to 74.90) | 43.72 (24.83 to 75.09) | 43.15 (24.99 to 72.71) | 42.04 (24.46 to 72.31) | 40.39 (23.95 to 67.61) | 38.24 (23.22 to 63.06) | 35.68 (21.74 to 58.77) | 33.31 (20.36 to 52.58) | 30.12 (19.37 to 47.40) |
| Indonesia | Both | Prevalence | 288.82 (145.98 to 644.15) | 734.32 (354.04 to 1799.73) | 978.77 (444.75 to 2234.54) | 1244.60 (537.07 to 2906.45) | 1468.22 (614.19 to 3496.40) | 1640.58 (673.19 to 3960.29) | 1759.76 (716.93 to 4301.10) | 1845.73 (742.13 to 4603.64) | 1915.21 (765.38 to 4889.18) | 1972.35 (780.51 to 5167.79) | 2029.41 (795.90 to 5436.58) | 2085.57 (806.46 to 5779.00) | 2162.58 (817.24 to 6388.83) | 2193.51 (821.09 to 6613.55) | 2212.44 (808.39 to 6957.82) | 2222.75 (798.30 to 7124.44) | 2191.55 (766.55 to 7368.85) | 2147.31 (729.61 to 7604.35) | 2115.67 (704.01 to 7675.67) | 2078.86 (669.00 to 7621.47) |
| Indonesia | Female | DALY rates | 11.59 (7.66 to 16.72) | 19.44 (12.90 to 28.19) | 19.81 (13.23 to 28.70) | 20.07 (13.23 to 29.17) | 20.27 (13.21 to 28.88) | 20.46 (14.13 to 29.61) | 20.72 (13.72 to 29.73) | 20.62 (13.77 to 29.18) | 20.64 (13.93 to 29.39) | 20.58 (13.90 to 29.56) | 20.55 (13.64 to 29.51) | 20.36 (13.84 to 28.98) | 20.33 (13.29 to 29.22) | 20.02 (13.17 to 28.07) | 19.57 (13.71 to 26.99) | 19.24 (12.67 to 27.01) | 18.84 (12.98 to 26.67) | 18.23 (12.53 to 25.23) | 17.93 (12.18 to 24.56) | 17.32 (12.27 to 23.79) |
| Indonesia | Female | Prevalence | 305.67 (162.00 to 663.07) | 653.38 (300.15 to 1710.69) | 882.48 (369.23 to 2143.04) | 1128.53 (443.81 to 2812.79) | 1333.04 (497.11 to 3383.77) | 1490.09 (536.73 to 3829.06) | 1598.49 (565.02 to 4147.63) | 1677.95 (586.19 to 4413.61) | 1741.78 (603.29 to 4663.15) | 1793.72 (611.43 to 4948.59) | 1845.10 (616.37 to 5242.22) | 1896.62 (614.89 to 5603.87) | 1966.72 (614.85 to 6241.01) | 1995.70 (612.81 to 6470.31) | 2011.72 (602.44 to 6800.18) | 2025.47 (588.77 to 7128.14) | 2001.71 (552.20 to 7343.02) | 1968.61 (532.08 to 7576.32) | 1959.82 (518.02 to 7840.17) | 1952.18 (506.71 to 7973.90) |
| Indonesia | Male | DALY rates | 8.29 (5.46 to 12.20) | 40.35 (19.67 to 82.99) | 44.48 (21.35 to 89.62) | 49.53 (24.17 to 99.63) | 54.08 (26.24 to 104.21) | 58.06 (28.88 to 113.06) | 60.62 (30.68 to 117.41) | 62.75 (31.60 to 119.26) | 64.05 (32.03 to 120.54) | 64.88 (32.52 to 121.70) | 65.59 (32.91 to 124.80) | 66.41 (34.12 to 125.69) | 67.33 (34.20 to 124.61) | 67.02 (34.81 to 121.80) | 66.44 (34.53 to 123.48) | 66.02 (34.89 to 121.79) | 64.34 (34.32 to 117.65) | 62.17 (33.41 to 113.74) | 60.60 (31.45 to 109.22) | 59.31 (31.82 to 108.00) |
| Indonesia | Male | Prevalence | 272.81 (129.65 to 626.16) | 811.65 (388.08 to 1881.07) | 1069.33 (491.04 to 2357.42) | 1353.60 (596.28 to 3014.36) | 1596.15 (692.77 to 3648.31) | 1784.05 (767.62 to 4146.07) | 1915.44 (823.95 to 4515.39) | 2009.47 (853.03 to 4831.78) | 2086.51 (878.06 to 5119.13) | 2150.13 (895.00 to 5367.70) | 2213.50 (917.06 to 5630.71) | 2275.94 (938.91 to 5955.44) | 2360.23 (957.99 to 6550.39) | 2397.62 (969.63 to 6761.36) | 2430.40 (965.58 to 7142.23) | 2461.81 (979.69 to 7170.37) | 2446.89 (962.90 to 7426.86) | 2418.64 (935.71 to 7565.92) | 2392.21 (927.42 to 7502.90) | 2367.81 (935.62 to 6982.98) |
| Kenya | Both | DALY rates | 9.94 (6.61 to 14.21) | 29.05 (16.70 to 53.11) | 30.20 (17.20 to 54.99) | 31.52 (17.81 to 56.88) | 32.58 (18.52 to 58.79) | 33.49 (19.11 to 58.96) | 34.20 (19.36 to 60.38) | 34.63 (19.55 to 62.25) | 35.18 (19.71 to 63.56) | 35.52 (20.07 to 64.00) | 35.24 (19.96 to 62.22) | 34.89 (19.94 to 61.17) | 34.52 (19.73 to 60.36) | 33.62 (19.55 to 58.24) | 33.44 (19.85 to 57.29) | 32.47 (19.51 to 55.15) | 29.07 (17.70 to 48.40) | 26.39 (16.46 to 42.26) | 24.13 (15.70 to 37.33) | 21.47 (14.38 to 31.23) |
| Kenya | Both | Prevalence | 245.29 (128.03 to 561.88) | 603.85 (309.00 to 1414.20) | 749.22 (344.92 to 1909.86) | 918.81 (383.73 to 2506.09) | 1084.09 (418.86 to 3124.02) | 1211.20 (447.47 to 3573.04) | 1269.44 (457.65 to 3766.98) | 1310.45 (464.39 to 3967.02) | 1345.20 (472.58 to 4082.55) | 1371.60 (474.54 to 4177.09) | 1390.02 (477.24 to 4262.30) | 1412.68 (481.72 to 4352.91) | 1441.71 (490.08 to 4338.87) | 1446.46 (490.01 to 4287.81) | 1487.78 (503.10 to 4385.81) | 1506.28 (510.12 to 4296.22) | 1415.97 (473.56 to 4145.00) | 1331.82 (438.18 to 3904.62) | 1306.88 (417.78 to 3864.39) | 1301.85 (396.35 to 4108.57) |
| Kenya | Female | DALY rates | 11.67 (7.78 to 16.97) | 19.32 (12.97 to 27.62) | 19.40 (12.88 to 27.66) | 19.42 (13.21 to 27.78) | 19.54 (13.21 to 27.88) | 19.53 (13.35 to 27.44) | 19.52 (13.50 to 27.82) | 19.40 (13.16 to 27.51) | 19.37 (13.31 to 27.59) | 19.26 (13.29 to 27.02) | 19.16 (12.97 to 26.89) | 19.04 (13.02 to 26.45) | 18.96 (13.15 to 26.91) | 18.72 (12.96 to 26.09) | 18.42 (12.92 to 25.09) | 18.14 (12.68 to 24.73) | 17.64 (12.37 to 24.26) | 17.26 (12.12 to 23.51) | 16.84 (11.83 to 23.13) | 16.28 (11.60 to 21.97) |
| Kenya | Female | Prevalence | 262.07 (143.78 to 578.93) | 529.37 (255.26 to 1299.87) | 661.14 (279.71 to 1827.02) | 815.31 (305.34 to 2390.99) | 969.01 (330.65 to 2974.58) | 1085.33 (351.76 to 3397.03) | 1138.12 (354.81 to 3568.93) | 1174.91 (354.69 to 3744.45) | 1203.44 (358.03 to 3843.57) | 1224.78 (362.23 to 3926.00) | 1232.27 (363.81 to 4007.24) | 1246.66 (365.38 to 4125.81) | 1282.32 (374.10 to 4093.09) | 1297.09 (376.42 to 4085.63) | 1321.29 (377.89 to 4120.66) | 1332.77 (380.62 to 4112.14) | 1286.12 (371.18 to 3973.06) | 1230.18 (356.62 to 3828.23) | 1225.49 (349.23 to 3828.63) | 1241.02 (341.29 to 4038.08) |
| Kenya | Male | DALY rates | 8.27 (5.45 to 11.72) | 38.56 (18.44 to 79.00) | 40.61 (19.65 to 83.64) | 43.38 (21.20 to 87.95) | 46.64 (22.86 to 94.58) | 49.05 (23.62 to 96.81) | 49.81 (24.00 to 99.80) | 50.32 (24.84 to 101.63) | 50.53 (24.76 to 102.66) | 50.74 (24.76 to 101.05) | 50.94 (25.07 to 98.75) | 51.17 (24.82 to 100.02) | 51.31 (25.34 to 99.96) | 50.84 (25.32 to 99.26) | 51.37 (25.79 to 98.03) | 51.54 (26.40 to 96.98) | 48.31 (24.40 to 93.86) | 45.73 (23.37 to 88.18) | 44.44 (22.71 to 85.16) | 43.74 (22.13 to 83.25) |
| Kenya | Male | Prevalence | 229.06 (108.92 to 545.38) | 676.63 (339.37 to 1525.29) | 834.16 (387.24 to 2014.67) | 1020.26 (439.91 to 2621.58) | 1208.14 (495.48 to 3275.66) | 1351.49 (542.04 to 3769.23) | 1409.00 (552.63 to 4040.77) | 1450.12 (560.49 to 4226.73) | 1482.86 (568.81 to 4318.47) | 1508.99 (574.99 to 4386.36) | 1544.00 (585.65 to 4498.98) | 1583.15 (598.26 to 4654.45) | 1613.63 (609.70 to 4708.90) | 1618.99 (610.07 to 4612.17) | 1686.58 (645.55 to 4653.94) | 1737.04 (674.94 to 4607.66) | 1634.48 (620.60 to 4353.78) | 1547.07 (580.75 to 4213.78) | 1533.74 (559.15 to 4200.44) | 1562.46 (547.49 to 4276.55) |
| Kiribati | Both | DALY rates | 9.80 (5.92 to 14.52) | 29.83 (15.59 to 55.57) | 31.53 (16.67 to 57.76) | 33.48 (17.23 to 61.99) | 34.96 (18.17 to 61.01) | 36.34 (19.25 to 66.92) | 36.98 (19.38 to 64.93) | 37.53 (20.32 to 66.90) | 37.69 (20.37 to 67.42) | 37.68 (21.25 to 65.72) | 38.38 (20.49 to 68.31) | 38.96 (21.13 to 67.30) | 38.52 (20.78 to 69.30) | 35.65 (19.81 to 61.54) | 33.03 (19.04 to 56.44) | 32.71 (19.91 to 54.61) | 31.78 (18.61 to 52.09) | 30.43 (18.07 to 49.66) | 28.81 (17.03 to 46.08) | 26.49 (15.60 to 41.33) |
| Kiribati | Both | Prevalence | 284.31 (98.53 to 866.43) | 733.49 (242.52 to 2159.68) | 957.02 (265.89 to 3012.78) | 1215.29 (285.88 to 3997.08) | 1439.20 (303.77 to 4861.47) | 1601.07 (319.66 to 5485.47) | 1716.42 (331.27 to 5928.33) | 1809.69 (339.95 to 6289.12) | 1886.89 (347.69 to 6588.97) | 1955.34 (353.80 to 6854.03) | 2024.01 (361.73 to 7110.58) | 2093.53 (370.04 to 7368.48) | 2165.21 (374.72 to 7655.18) | 2181.61 (365.26 to 7784.00) | 2163.88 (355.66 to 7771.40) | 2166.26 (357.77 to 7773.29) | 2163.50 (355.33 to 7771.03) | 2155.83 (350.26 to 7765.61) | 2145.91 (341.68 to 7758.56) | 2129.42 (326.29 to 7747.07) |
| Kiribati | Female | DALY rates | 11.57 (6.63 to 17.79) | 19.27 (10.92 to 29.47) | 19.61 (11.92 to 29.94) | 20.07 (12.21 to 31.14) | 20.07 (11.38 to 30.99) | 20.50 (12.19 to 30.74) | 20.34 (12.13 to 31.65) | 20.22 (12.13 to 30.70) | 20.30 (11.73 to 31.23) | 20.01 (12.17 to 30.09) | 20.13 (11.80 to 30.94) | 20.29 (12.19 to 31.49) | 19.75 (11.88 to 29.37) | 19.54 (11.82 to 29.40) | 19.47 (11.28 to 29.90) | 18.98 (11.76 to 28.61) | 18.73 (10.77 to 28.33) | 18.33 (11.32 to 27.42) | 17.91 (10.83 to 27.12) | 17.51 (10.22 to 26.11) |
| Kiribati | Female | Prevalence | 301.40 (115.52 to 884.23) | 654.01 (197.85 to 2086.09) | 867.22 (205.55 to 2944.91) | 1113.10 (216.48 to 3935.85) | 1325.30 (224.72 to 4790.09) | 1478.05 (230.82 to 5405.94) | 1586.51 (234.75 to 5842.60) | 1674.83 (237.75 to 6197.91) | 1748.51 (240.44 to 6495.06) | 1813.48 (243.70 to 6756.13) | 1875.94 (245.41 to 7007.76) | 1938.51 (248.39 to 7259.42) | 2009.42 (250.05 to 7544.93) | 2044.58 (251.97 to 7686.55) | 2044.57 (251.86 to 7687.04) | 2044.56 (252.24 to 7686.62) | 2044.58 (251.71 to 7686.18) | 2044.60 (252.27 to 7686.02) | 2044.56 (251.54 to 7686.47) | 2044.57 (251.71 to 7686.54) |
| Kiribati | Male | DALY rates | 8.15 (4.48 to 13.05) | 39.74 (17.18 to 86.32) | 43.05 (18.70 to 89.47) | 46.97 (19.95 to 98.34) | 50.39 (22.10 to 97.82) | 53.05 (22.61 to 104.48) | 54.73 (23.79 to 108.04) | 56.37 (25.51 to 113.76) | 57.10 (26.68 to 112.03) | 57.77 (27.05 to 115.85) | 58.72 (26.35 to 115.79) | 59.24 (26.96 to 114.85) | 60.17 (27.33 to 120.33) | 59.67 (27.61 to 117.16) | 58.30 (27.31 to 113.31) | 57.51 (27.70 to 110.42) | 56.20 (26.84 to 110.99) | 55.48 (26.26 to 103.78) | 54.63 (24.84 to 103.55) | 53.67 (25.53 to 102.68) |
| Kiribati | Male | Prevalence | 268.45 (83.39 to 849.91) | 808.18 (262.60 to 2203.72) | 1043.84 (293.35 to 3100.62) | 1318.16 (331.28 to 4135.12) | 1557.31 (366.93 to 5005.76) | 1730.92 (391.36 to 5632.42) | 1854.90 (409.77 to 6078.27) | 1956.39 (424.84 to 6441.90) | 2041.33 (436.43 to 6747.95) | 2116.51 (447.28 to 7015.49) | 2189.06 (460.90 to 7275.01) | 2261.94 (472.46 to 7535.31) | 2344.90 (486.79 to 7828.72) | 2385.97 (492.32 to 7974.66) | 2386.08 (493.67 to 7974.67) | 2386.04 (492.83 to 7974.18) | 2386.03 (493.33 to 7975.87) | 2385.95 (492.57 to 7972.85) | 2385.97 (493.60 to 7974.29) | 2386.07 (493.54 to 7975.55) |
| Lao People's Democratic Republic | Both | DALY rates | 9.59 (5.99 to 14.44) | 27.29 (14.52 to 49.39) | 27.37 (14.55 to 49.45) | 27.65 (14.13 to 50.59) | 27.90 (15.63 to 49.94) | 28.35 (15.46 to 51.55) | 28.25 (15.20 to 49.80) | 28.31 (15.80 to 50.75) | 28.43 (15.05 to 52.60) | 28.02 (15.19 to 51.97) | 27.84 (15.09 to 48.58) | 27.55 (15.12 to 52.44) | 27.39 (14.73 to 49.10) | 26.88 (15.27 to 47.77) | 26.14 (14.47 to 46.66) | 25.41 (14.14 to 45.43) | 24.48 (13.64 to 42.93) | 23.62 (13.63 to 40.56) | 22.78 (12.03 to 38.36) | 20.89 (12.50 to 33.21) |
| Lao People's Democratic Republic | Both | Prevalence | 136.15 (92.68 to 346.66) | 354.35 (205.59 to 839.42) | 406.98 (211.02 to 1129.15) | 467.35 (218.05 to 1462.66) | 517.96 (224.18 to 1743.22) | 555.92 (228.49 to 1949.18) | 583.06 (231.35 to 2095.89) | 604.42 (233.83 to 2214.17) | 622.68 (236.04 to 2315.14) | 637.80 (236.68 to 2402.92) | 651.40 (238.11 to 2485.04) | 667.13 (239.35 to 2571.27) | 685.30 (241.22 to 2668.97) | 693.40 (241.25 to 2715.43) | 690.24 (240.32 to 2714.43) | 688.84 (239.69 to 2714.58) | 684.24 (238.01 to 2713.30) | 679.31 (236.15 to 2711.71) | 675.15 (234.00 to 2710.81) | 664.06 (230.61 to 2707.60) |
| Lao People's Democratic Republic | Female | DALY rates | 11.25 (6.59 to 17.57) | 18.82 (10.70 to 28.82) | 18.91 (10.93 to 29.74) | 18.52 (10.55 to 30.35) | 18.65 (11.28 to 28.52) | 18.91 (11.10 to 28.97) | 18.55 (11.47 to 28.92) | 18.72 (10.98 to 28.48) | 18.78 (11.02 to 30.30) | 18.53 (10.93 to 28.81) | 18.48 (10.83 to 28.27) | 18.37 (10.90 to 28.64) | 18.07 (10.65 to 27.83) | 17.77 (10.67 to 27.35) | 17.37 (10.28 to 27.22) | 16.90 (10.06 to 26.11) | 16.81 (10.11 to 25.83) | 16.63 (9.86 to 25.79) | 16.32 (9.44 to 24.95) | 15.86 (9.48 to 23.91) |
| Lao People's Democratic Republic | Female | Prevalence | 152.28 (106.80 to 361.83) | 292.88 (183.67 to 816.65) | 343.42 (187.67 to 1107.81) | 401.54 (190.55 to 1442.49) | 450.39 (193.63 to 1723.88) | 486.36 (195.64 to 1930.75) | 511.77 (196.65 to 2077.62) | 532.28 (198.56 to 2195.72) | 549.72 (199.09 to 2296.13) | 565.02 (200.14 to 2383.81) | 579.28 (201.04 to 2465.61) | 594.07 (201.64 to 2551.50) | 610.92 (202.42 to 2648.69) | 619.04 (202.64 to 2695.44) | 619.06 (202.84 to 2695.32) | 619.05 (203.43 to 2695.41) | 619.07 (202.83 to 2695.50) | 619.08 (202.94 to 2695.47) | 619.07 (202.89 to 2695.83) | 619.08 (202.86 to 2695.53) |
| Lao People's Democratic Republic | Male | DALY rates | 8.00 (4.42 to 13.09) | 35.44 (15.49 to 75.93) | 35.55 (15.95 to 75.40) | 36.60 (15.40 to 75.81) | 37.11 (16.09 to 80.24) | 37.65 (16.87 to 77.00) | 37.69 (16.39 to 74.75) | 37.66 (16.89 to 77.54) | 37.85 (16.87 to 79.29) | 37.53 (16.74 to 80.58) | 37.56 (15.76 to 76.08) | 37.03 (16.50 to 80.22) | 36.88 (16.16 to 75.39) | 36.28 (16.51 to 72.62) | 35.97 (16.02 to 77.75) | 35.31 (15.76 to 74.83) | 34.59 (16.34 to 75.03) | 34.14 (15.54 to 73.76) | 33.71 (14.77 to 67.47) | 32.74 (14.57 to 67.66) |
| Lao People's Democratic Republic | Male | Prevalence | 120.65 (76.29 to 332.08) | 413.59 (213.87 to 907.69) | 468.52 (222.67 to 1158.09) | 531.87 (234.66 to 1492.44) | 585.21 (243.93 to 1774.66) | 624.47 (250.10 to 1981.73) | 652.34 (255.34 to 2129.06) | 674.81 (258.37 to 2246.48) | 693.97 (261.61 to 2347.70) | 710.71 (263.85 to 2435.23) | 726.35 (265.94 to 2518.24) | 742.60 (268.78 to 2603.74) | 761.10 (271.54 to 2700.66) | 770.06 (272.21 to 2748.25) | 770.05 (272.16 to 2746.83) | 770.06 (272.23 to 2747.84) | 770.01 (272.37 to 2747.62) | 770.02 (272.36 to 2747.14) | 770.07 (273.03 to 2748.15) | 770.08 (272.76 to 2747.84) |
| Liberia | Both | DALY rates | 11.56 (7.16 to 17.18) | 78.32 (44.37 to 133.56) | 123.60 (74.02 to 200.50) | 190.61 (121.05 to 285.49) | 253.45 (163.32 to 375.44) | 294.88 (194.01 to 426.47) | 325.64 (217.51 to 464.06) | 350.27 (233.72 to 497.31) | 386.46 (257.85 to 533.80) | 396.67 (269.63 to 553.86) | 412.03 (273.72 to 574.40) | 404.90 (276.01 to 555.56) | 421.84 (286.01 to 576.33) | 412.73 (277.90 to 557.60) | 394.05 (270.73 to 537.31) | 374.18 (258.42 to 506.23) | 371.76 (256.16 to 502.80) | 395.34 (273.79 to 532.50) | 357.30 (246.26 to 472.06) | 349.77 (242.01 to 465.98) |
| Liberia | Both | Prevalence | 1653.89 (1276.99 to 2144.99) | 4504.84 (3440.78 to 5834.44) | 6641.58 (5095.95 to 8577.14) | 9181.44 (7105.52 to 11766.07) | 11425.34 (8901.51 to 14579.35) | 13031.79 (10190.36 to 16588.35) | 14202.35 (11142.28 to 17989.57) | 15152.02 (11929.68 to 19133.65) | 16082.51 (12727.78 to 20235.49) | 16739.82 (13265.45 to 21051.46) | 17380.37 (13797.81 to 21829.92) | 17867.07 (14185.32 to 22500.77) | 18641.06 (14845.34 to 23458.93) | 18900.81 (15030.72 to 23800.46) | 18799.34 (14928.46 to 23702.25) | 18687.90 (14819.93 to 23581.32) | 18711.11 (14842.93 to 23608.78) | 18985.23 (15108.07 to 23892.10) | 18684.63 (14821.71 to 23583.69) | 18692.30 (14829.20 to 23595.80) |
| Liberia | Female | DALY rates | 13.54 (8.07 to 20.90) | 25.29 (15.07 to 39.98) | 29.07 (17.67 to 43.42) | 33.43 (20.76 to 49.86) | 38.48 (23.83 to 55.15) | 42.02 (26.26 to 61.68) | 44.68 (28.02 to 62.69) | 46.87 (29.68 to 68.43) | 48.99 (31.28 to 70.36) | 51.27 (32.97 to 75.15) | 52.35 (34.47 to 77.04) | 54.57 (36.08 to 76.82) | 56.58 (37.17 to 81.44) | 57.44 (37.51 to 81.75) | 56.70 (37.46 to 80.74) | 56.05 (38.17 to 79.99) | 54.86 (36.91 to 78.61) | 54.26 (35.82 to 77.59) | 53.25 (36.58 to 74.16) | 51.51 (35.05 to 72.62) |
| Liberia | Female | Prevalence | 1676.61 (1297.26 to 2169.35) | 4082.04 (3141.28 to 5306.37) | 5871.82 (4504.70 to 7654.56) | 7904.11 (6054.46 to 10320.05) | 9673.78 (7406.91 to 12636.48) | 10956.56 (8387.52 to 14316.95) | 11885.90 (9099.85 to 15532.38) | 12633.45 (9672.66 to 16508.78) | 13259.17 (10153.36 to 17323.98) | 13807.04 (10574.06 to 18042.62) | 14312.03 (10960.56 to 18698.94) | 14858.83 (11379.59 to 19412.49) | 15474.84 (11854.64 to 20217.60) | 15772.82 (12081.83 to 20601.74) | 15772.74 (12082.37 to 20602.86) | 15772.73 (12081.29 to 20602.93) | 15772.72 (12083.25 to 20605.24) | 15772.71 (12082.05 to 20603.45) | 15772.75 (12082.89 to 20604.15) | 15772.73 (12083.70 to 20603.67) |
| Liberia | Male | DALY rates | 9.67 (5.29 to 15.43) | 129.47 (66.28 to 234.19) | 215.29 (122.60 to 359.11) | 346.98 (216.49 to 525.58) | 474.20 (300.03 to 701.54) | 560.35 (368.54 to 812.46) | 617.15 (407.28 to 884.57) | 655.82 (435.28 to 930.13) | 683.21 (454.94 to 948.88) | 699.15 (469.00 to 976.16) | 715.97 (473.84 to 1008.79) | 731.36 (495.94 to 1008.19) | 743.33 (496.42 to 1020.67) | 740.25 (497.15 to 1006.97) | 726.81 (499.40 to 997.15) | 712.11 (489.22 to 960.11) | 703.10 (481.66 to 952.43) | 692.61 (479.38 to 931.38) | 681.01 (464.61 to 909.91) | 665.69 (457.62 to 890.35) |
| Liberia | Male | Prevalence | 1632.24 (1257.67 to 2121.96) | 4912.63 (3772.08 to 6287.65) | 7388.13 (5753.95 to 9395.47) | 10452.24 (8319.24 to 13130.51) | 13223.99 (10631.51 to 16456.83) | 15210.48 (12309.25 to 18856.58) | 16605.89 (13535.09 to 20504.05) | 17688.33 (14490.94 to 21785.73) | 18565.17 (15292.01 to 22822.34) | 19308.10 (15911.29 to 23727.62) | 19973.15 (16455.01 to 24494.54) | 20670.29 (17030.11 to 25385.03) | 21428.00 (17661.02 to 26321.09) | 21784.28 (17922.43 to 26754.49) | 21784.82 (17920.38 to 26765.99) | 21784.43 (17902.51 to 26752.02) | 21783.43 (17906.89 to 26758.41) | 21785.01 (17884.41 to 26763.68) | 21784.82 (17895.75 to 26764.67) | 21784.84 (17888.45 to 26772.88) |
| Madagascar | Both | DALY rates | 9.81 (6.04 to 14.77) | 28.40 (13.49 to 54.31) | 28.85 (15.62 to 50.74) | 30.77 (16.48 to 55.55) | 31.31 (16.78 to 55.60) | 31.92 (17.25 to 57.15) | 32.46 (17.96 to 59.46) | 32.70 (17.49 to 57.90) | 33.49 (17.69 to 60.02) | 33.46 (17.93 to 60.84) | 33.42 (18.26 to 58.09) | 33.71 (18.07 to 59.53) | 34.12 (18.11 to 60.69) | 33.50 (18.04 to 58.10) | 32.57 (18.17 to 58.29) | 31.51 (17.36 to 55.50) | 30.54 (17.40 to 52.92) | 29.51 (16.77 to 50.88) | 28.68 (16.31 to 48.64) | 26.91 (15.16 to 44.59) |
| Madagascar | Both | Prevalence | 221.87 (123.75 to 461.41) | 569.08 (287.44 to 1171.30) | 719.41 (333.80 to 1579.71) | 892.98 (385.62 to 2070.46) | 1040.25 (425.76 to 2473.11) | 1148.65 (454.72 to 2761.64) | 1226.61 (474.37 to 2965.88) | 1289.53 (491.36 to 3130.60) | 1342.98 (505.87 to 3272.16) | 1389.07 (518.85 to 3395.16) | 1432.31 (529.80 to 3512.45) | 1479.14 (544.07 to 3637.25) | 1532.49 (559.05 to 3779.61) | 1555.34 (564.78 to 3846.83) | 1552.43 (561.82 to 3845.17) | 1547.84 (558.23 to 3842.67) | 1542.92 (555.99 to 3840.90) | 1540.44 (555.79 to 3838.90) | 1536.94 (554.33 to 3836.89) | 1527.92 (549.58 to 3832.21) |
| Madagascar | Female | DALY rates | 11.50 (6.52 to 18.08) | 19.07 (10.96 to 30.73) | 18.86 (11.14 to 29.73) | 19.54 (11.76 to 30.76) | 19.32 (10.97 to 30.17) | 19.38 (11.01 to 30.72) | 19.57 (11.34 to 30.43) | 19.26 (11.37 to 30.13) | 19.61 (11.43 to 28.83) | 19.52 (11.24 to 28.79) | 19.20 (11.48 to 29.35) | 19.34 (11.01 to 30.00) | 19.30 (11.41 to 30.19) | 19.29 (11.82 to 29.89) | 18.51 (10.97 to 28.68) | 18.38 (11.41 to 28.28) | 17.95 (10.26 to 28.10) | 17.74 (10.68 to 26.67) | 17.43 (10.94 to 26.79) | 16.68 (9.99 to 24.47) |
| Madagascar | Female | Prevalence | 238.83 (140.14 to 477.66) | 499.20 (258.23 to 1088.46) | 643.31 (295.84 to 1502.29) | 809.18 (337.44 to 1980.41) | 949.71 (371.11 to 2384.87) | 1052.82 (396.33 to 2682.38) | 1126.55 (413.29 to 2894.24) | 1185.81 (429.00 to 3065.03) | 1235.84 (440.89 to 3208.96) | 1279.47 (451.97 to 3334.85) | 1320.37 (461.17 to 3452.96) | 1362.84 (470.90 to 3574.61) | 1411.31 (482.91 to 3714.05) | 1434.71 (488.35 to 3781.68) | 1434.68 (488.38 to 3780.83) | 1434.69 (488.39 to 3781.24) | 1434.72 (488.60 to 3781.49) | 1434.73 (489.05 to 3780.93) | 1434.71 (489.07 to 3781.06) | 1434.72 (488.85 to 3781.49) |
| Madagascar | Male | DALY rates | 8.17 (4.60 to 13.22) | 37.65 (15.07 to 83.43) | 38.97 (17.52 to 81.95) | 42.35 (18.49 to 89.03) | 43.93 (19.13 to 89.63) | 45.25 (20.31 to 90.87) | 46.20 (19.82 to 97.41) | 47.00 (20.31 to 95.74) | 48.15 (21.46 to 100.72) | 48.27 (23.04 to 98.11) | 48.58 (21.38 to 97.03) | 48.62 (22.10 to 96.97) | 49.09 (22.71 to 97.30) | 48.36 (21.47 to 96.67) | 47.96 (21.68 to 96.47) | 47.02 (21.02 to 93.33) | 46.66 (22.34 to 92.34) | 45.22 (20.59 to 90.29) | 44.57 (20.41 to 90.88) | 43.73 (20.36 to 86.30) |
| Madagascar | Male | Prevalence | 205.43 (105.67 to 445.67) | 638.35 (301.93 to 1291.46) | 796.46 (357.02 to 1695.50) | 979.45 (414.93 to 2187.43) | 1135.54 (462.58 to 2610.27) | 1250.52 (499.89 to 2912.19) | 1333.20 (527.13 to 3133.50) | 1399.79 (548.31 to 3307.33) | 1456.16 (566.21 to 3456.16) | 1505.43 (580.60 to 3586.21) | 1551.64 (595.85 to 3704.57) | 1599.75 (610.27 to 3830.21) | 1654.81 (628.04 to 3972.46) | 1681.41 (637.37 to 4048.72) | 1681.43 (638.20 to 4042.13) | 1681.42 (637.52 to 4045.90) | 1681.46 (637.34 to 4046.29) | 1681.41 (638.69 to 4045.97) | 1681.45 (637.88 to 4040.72) | 1681.34 (637.29 to 4045.20) |
| Malawi | Both | DALY rates | 9.56 (6.00 to 14.03) | 26.64 (14.37 to 48.64) | 26.89 (14.66 to 47.29) | 27.10 (14.33 to 48.96) | 26.73 (14.35 to 49.09) | 26.74 (15.26 to 49.06) | 27.00 (14.56 to 47.64) | 26.80 (14.18 to 47.16) | 26.87 (14.25 to 47.30) | 27.06 (14.10 to 50.19) | 26.87 (14.42 to 48.98) | 26.38 (14.54 to 47.48) | 25.96 (14.20 to 46.58) | 25.45 (14.06 to 43.94) | 24.59 (14.35 to 40.62) | 23.60 (13.67 to 40.68) | 22.36 (13.18 to 36.21) | 21.32 (11.87 to 34.29) | 20.11 (12.04 to 31.28) | 18.73 (11.19 to 28.76) |
| Malawi | Both | Prevalence | 127.17 (106.38 to 165.74) | 326.19 (240.82 to 489.71) | 366.29 (263.67 to 556.06) | 411.79 (291.29 to 635.23) | 449.30 (312.43 to 710.42) | 477.45 (328.28 to 764.40) | 498.00 (338.80 to 805.93) | 514.24 (347.35 to 840.07) | 530.94 (356.46 to 873.42) | 545.91 (363.19 to 901.06) | 554.98 (368.85 to 923.71) | 564.36 (373.59 to 945.15) | 575.89 (379.44 to 969.86) | 580.13 (381.42 to 978.88) | 576.82 (378.73 to 973.66) | 572.69 (377.70 to 967.06) | 567.86 (375.38 to 958.05) | 561.30 (372.41 to 946.07) | 553.59 (365.45 to 936.89) | 545.00 (360.15 to 931.20) |
| Malawi | Female | DALY rates | 11.15 (6.73 to 17.03) | 18.69 (10.54 to 29.78) | 18.71 (11.03 to 27.73) | 18.78 (10.72 to 28.74) | 18.59 (11.06 to 29.09) | 18.40 (10.84 to 29.16) | 18.60 (10.76 to 28.86) | 18.05 (10.87 to 27.67) | 17.89 (10.36 to 27.78) | 17.78 (10.25 to 27.74) | 18.02 (9.99 to 27.69) | 17.89 (10.12 to 27.58) | 17.78 (10.81 to 28.63) | 17.75 (10.47 to 28.13) | 17.35 (10.17 to 26.45) | 17.15 (10.80 to 25.91) | 16.27 (9.81 to 25.26) | 16.42 (9.36 to 24.95) | 15.94 (9.42 to 23.42) | 15.63 (9.08 to 23.19) |
| Malawi | Female | Prevalence | 143.53 (121.40 to 184.32) | 267.35 (216.17 to 365.34) | 306.03 (234.56 to 446.47) | 350.04 (255.25 to 540.65) | 387.47 (271.76 to 620.67) | 415.32 (283.93 to 679.58) | 435.23 (293.09 to 721.67) | 450.98 (299.82 to 755.74) | 464.32 (306.89 to 784.57) | 476.03 (311.70 to 809.71) | 486.90 (317.18 to 832.67) | 498.40 (321.58 to 856.55) | 511.55 (327.47 to 884.53) | 517.61 (330.15 to 897.26) | 517.60 (329.61 to 897.74) | 517.62 (329.53 to 897.44) | 517.60 (329.53 to 897.74) | 517.62 (330.07 to 897.33) | 517.61 (329.63 to 898.25) | 517.64 (329.82 to 897.50) |
| Malawi | Male | DALY rates | 8.00 (4.43 to 12.98) | 34.69 (15.43 to 74.64) | 35.23 (14.76 to 74.55) | 35.70 (15.41 to 76.86) | 35.55 (15.91 to 75.50) | 36.03 (15.86 to 78.85) | 36.44 (16.25 to 77.21) | 36.68 (15.97 to 77.17) | 36.19 (15.64 to 73.81) | 35.97 (16.40 to 76.32) | 35.96 (15.44 to 75.29) | 35.78 (16.13 to 75.90) | 35.62 (15.24 to 77.36) | 35.09 (15.02 to 73.93) | 34.57 (15.89 to 68.47) | 33.63 (14.87 to 70.51) | 33.32 (14.76 to 67.75) | 32.22 (14.41 to 64.72) | 32.27 (14.32 to 67.03) | 31.59 (14.22 to 68.49) |
| Malawi | Male | Prevalence | 111.06 (90.01 to 148.22) | 385.82 (243.69 to 671.23) | 427.78 (271.66 to 734.37) | 475.59 (301.43 to 813.47) | 516.36 (326.21 to 889.20) | 546.71 (347.32 to 936.79) | 568.45 (359.84 to 970.39) | 585.58 (369.83 to 994.39) | 600.16 (378.10 to 1011.69) | 612.98 (385.89 to 1027.48) | 624.87 (393.85 to 1048.90) | 637.43 (400.70 to 1071.50) | 651.78 (408.80 to 1117.05) | 658.46 (411.47 to 1105.26) | 658.46 (412.46 to 1106.06) | 658.42 (411.98 to 1105.93) | 658.44 (412.23 to 1105.13) | 658.44 (412.26 to 1105.37) | 658.43 (412.03 to 1105.66) | 658.44 (412.21 to 1106.01) |
| Malaysia | Both | DALY rates | 9.88 (5.89 to 15.50) | 28.78 (14.82 to 52.26) | 29.73 (15.52 to 52.13) | 30.96 (16.22 to 56.46) | 31.99 (16.58 to 61.95) | 33.25 (16.76 to 61.84) | 34.00 (18.20 to 61.59) | 34.26 (18.19 to 62.25) | 34.65 (18.22 to 62.48) | 34.26 (17.04 to 64.15) | 34.17 (18.43 to 59.76) | 34.05 (18.20 to 61.15) | 33.77 (18.20 to 60.23) | 32.93 (17.69 to 59.10) | 32.49 (18.03 to 57.65) | 31.62 (17.21 to 56.20) | 30.80 (17.31 to 52.83) | 30.53 (16.10 to 53.96) | 31.34 (16.88 to 54.58) | 34.01 (17.95 to 62.85) |
| Malaysia | Both | Prevalence | 222.54 (108.21 to 671.07) | 562.49 (253.92 to 1686.38) | 710.63 (275.48 to 2331.86) | 880.99 (301.98 to 3069.87) | 1025.91 (327.69 to 3696.44) | 1133.54 (347.13 to 4155.84) | 1210.96 (360.23 to 4485.00) | 1271.95 (371.53 to 4746.79) | 1322.38 (379.06 to 4966.77) | 1365.67 (384.37 to 5161.57) | 1406.33 (391.32 to 5341.18) | 1448.91 (397.53 to 5527.95) | 1497.57 (403.89 to 5742.69) | 1519.91 (405.82 to 5845.76) | 1519.86 (406.74 to 5842.26) | 1518.36 (405.12 to 5840.52) | 1515.51 (403.50 to 5835.97) | 1521.83 (407.79 to 5846.47) | 1534.71 (417.21 to 5865.25) | 1567.10 (437.87 to 5910.60) |
| Malaysia | Female | DALY rates | 11.61 (6.82 to 18.11) | 19.32 (10.98 to 30.47) | 19.31 (11.19 to 29.93) | 19.43 (10.94 to 30.18) | 19.32 (10.87 to 29.29) | 19.53 (11.61 to 30.45) | 19.56 (11.62 to 29.96) | 19.36 (11.73 to 29.04) | 19.79 (11.52 to 30.71) | 19.13 (11.38 to 28.45) | 19.38 (11.34 to 29.56) | 19.41 (11.39 to 29.27) | 19.14 (11.13 to 28.96) | 18.73 (11.10 to 27.74) | 18.55 (11.61 to 27.67) | 18.11 (10.98 to 27.51) | 17.87 (10.73 to 26.45) | 17.33 (10.02 to 25.67) | 17.00 (11.02 to 25.57) | 16.24 (9.96 to 24.33) |
| Malaysia | Female | Prevalence | 239.61 (125.17 to 688.27) | 491.09 (215.62 to 1573.35) | 632.32 (228.46 to 2210.53) | 794.28 (245.34 to 2939.87) | 930.68 (257.74 to 3555.62) | 1030.44 (266.60 to 4004.25) | 1101.61 (271.75 to 4324.86) | 1158.49 (277.12 to 4581.93) | 1207.02 (281.45 to 4800.16) | 1249.88 (285.78 to 4993.42) | 1289.88 (288.97 to 5173.47) | 1331.18 (292.86 to 5359.44) | 1378.40 (297.74 to 5571.97) | 1400.64 (299.76 to 5672.68) | 1400.68 (300.69 to 5672.55) | 1400.66 (300.91 to 5672.46) | 1400.64 (300.61 to 5672.63) | 1400.63 (300.07 to 5673.15) | 1400.65 (299.95 to 5672.90) | 1400.63 (300.75 to 5672.13) |
| Malaysia | Male | DALY rates | 8.26 (4.34 to 13.53) | 37.69 (16.24 to 78.85) | 39.57 (17.81 to 80.05) | 41.92 (18.35 to 86.78) | 43.84 (18.46 to 96.03) | 45.60 (19.46 to 92.43) | 46.56 (20.74 to 91.03) | 47.16 (21.11 to 95.69) | 47.84 (21.01 to 98.61) | 48.29 (21.58 to 100.86) | 48.39 (22.61 to 98.99) | 48.50 (21.33 to 98.25) | 48.64 (21.59 to 97.18) | 47.71 (21.15 to 96.50) | 47.01 (21.74 to 96.39) | 46.03 (21.12 to 92.95) | 45.25 (21.39 to 88.29) | 43.83 (19.61 to 87.43) | 43.04 (19.94 to 85.23) | 42.22 (20.20 to 84.90) |
| Malaysia | Male | Prevalence | 206.53 (90.67 to 654.94) | 629.72 (278.93 to 1727.67) | 784.64 (307.12 to 2413.08) | 963.39 (341.31 to 3198.01) | 1115.00 (371.55 to 3831.79) | 1226.37 (393.07 to 4295.07) | 1306.06 (407.64 to 4626.34) | 1370.06 (422.52 to 4890.76) | 1424.73 (433.49 to 5115.47) | 1473.08 (442.86 to 5318.29) | 1518.34 (452.64 to 5502.50) | 1565.07 (461.87 to 5694.19) | 1618.68 (473.07 to 5916.19) | 1643.98 (479.04 to 6025.78) | 1643.96 (479.33 to 6018.98) | 1643.97 (477.83 to 6019.89) | 1643.98 (478.70 to 6018.64) | 1643.98 (478.55 to 6021.14) | 1644.05 (478.80 to 6022.14) | 1644.00 (478.78 to 6020.74) |
| Maldives | Both | DALY rates | 9.84 (6.06 to 15.72) | 27.16 (14.56 to 49.50) | 27.06 (14.11 to 48.46) | 27.66 (14.51 to 51.42) | 28.82 (15.44 to 54.88) | 30.62 (15.46 to 57.73) | 31.02 (15.61 to 58.90) | 30.67 (15.12 to 58.82) | 30.36 (15.85 to 56.22) | 29.90 (15.32 to 58.52) | 28.80 (15.30 to 56.09) | 28.00 (14.68 to 49.98) | 27.25 (15.19 to 48.88) | 26.92 (14.38 to 48.54) | 26.06 (13.97 to 48.91) | 25.41 (13.78 to 44.17) | 24.55 (13.76 to 43.91) | 24.81 (13.77 to 43.01) | 24.65 (13.59 to 44.24) | 23.41 (12.96 to 42.13) |
| Maldives | Both | Prevalence | 122.22 (91.05 to 211.33) | 306.91 (183.80 to 544.50) | 337.39 (185.21 to 675.16) | 375.17 (187.14 to 825.67) | 413.88 (189.35 to 963.70) | 445.46 (189.87 to 1063.63) | 465.31 (190.47 to 1132.50) | 476.70 (191.18 to 1178.88) | 483.74 (191.89 to 1218.06) | 488.61 (192.64 to 1246.47) | 492.33 (193.28 to 1273.65) | 496.81 (193.70 to 1304.71) | 503.44 (193.31 to 1341.39) | 507.41 (193.82 to 1358.16) | 507.06 (193.51 to 1359.82) | 504.84 (193.09 to 1354.88) | 504.62 (193.04 to 1354.81) | 506.21 (193.15 to 1356.27) | 508.98 (193.58 to 1361.44) | 507.03 (192.99 to 1359.53) |
| Maldives | Female | DALY rates | 11.56 (6.73 to 18.43) | 18.99 (10.90 to 31.81) | 18.85 (10.56 to 30.34) | 18.79 (11.09 to 29.29) | 18.51 (10.82 to 28.56) | 18.48 (11.19 to 29.00) | 18.46 (10.94 to 28.41) | 18.31 (10.68 to 28.19) | 18.44 (10.64 to 29.00) | 18.32 (11.05 to 29.30) | 18.08 (9.93 to 27.80) | 17.99 (10.08 to 27.37) | 18.05 (10.66 to 28.91) | 17.83 (10.84 to 27.32) | 17.32 (10.64 to 26.70) | 16.79 (9.93 to 25.64) | 16.36 (9.70 to 25.88) | 16.62 (9.69 to 25.31) | 16.20 (9.70 to 23.97) | 15.43 (9.32 to 23.10) |
| Maldives | Female | Prevalence | 139.00 (105.21 to 231.80) | 246.17 (171.47 to 461.75) | 274.99 (171.68 to 584.21) | 308.83 (172.67 to 728.41) | 337.84 (173.14 to 853.06) | 358.43 (174.14 to 941.74) | 372.75 (174.24 to 1003.81) | 384.29 (174.64 to 1052.30) | 394.27 (174.60 to 1095.72) | 403.16 (174.81 to 1133.47) | 411.38 (174.98 to 1168.73) | 420.00 (175.12 to 1206.57) | 429.65 (175.54 to 1246.90) | 434.41 (175.59 to 1267.77) | 434.43 (175.85 to 1267.22) | 434.39 (175.28 to 1267.75) | 434.43 (175.95 to 1268.15) | 434.41 (175.41 to 1267.29) | 434.41 (175.36 to 1267.58) | 434.42 (175.22 to 1267.06) |
| Maldives | Male | DALY rates | 8.21 (4.37 to 13.26) | 34.83 (15.42 to 75.62) | 34.75 (15.37 to 71.37) | 35.40 (15.54 to 74.48) | 35.79 (15.46 to 75.03) | 36.57 (16.52 to 74.92) | 36.27 (16.29 to 73.95) | 36.04 (15.29 to 76.32) | 36.23 (16.65 to 72.78) | 36.56 (16.56 to 79.87) | 36.02 (16.67 to 76.17) | 35.78 (15.32 to 72.64) | 35.20 (16.09 to 71.60) | 35.02 (16.21 to 74.40) | 33.94 (14.78 to 73.02) | 33.70 (13.85 to 69.25) | 32.47 (15.64 to 67.81) | 32.38 (15.47 to 64.91) | 31.85 (14.17 to 66.21) | 30.61 (15.01 to 64.38) |
| Maldives | Male | Prevalence | 106.32 (73.99 to 192.72) | 364.00 (185.38 to 735.13) | 395.82 (187.39 to 806.78) | 433.11 (189.99 to 898.29) | 465.31 (191.85 to 1030.08) | 488.13 (192.90 to 1123.50) | 503.98 (194.25 to 1188.04) | 516.81 (195.09 to 1241.15) | 527.87 (195.39 to 1288.62) | 537.70 (196.04 to 1323.85) | 546.92 (197.05 to 1358.40) | 556.47 (197.11 to 1396.83) | 567.23 (198.20 to 1440.22) | 572.54 (198.12 to 1456.83) | 572.51 (198.45 to 1461.60) | 572.52 (198.15 to 1457.05) | 572.54 (198.47 to 1458.37) | 572.54 (198.93 to 1457.26) | 572.52 (198.50 to 1458.43) | 572.55 (198.46 to 1461.01) |
| Mali | Both | DALY rates | 9.25 (5.83 to 13.61) | 29.60 (16.02 to 53.83) | 31.45 (16.38 to 57.95) | 33.90 (17.81 to 60.67) | 35.31 (18.91 to 65.07) | 36.24 (19.51 to 65.20) | 37.15 (20.03 to 68.23) | 37.65 (20.31 to 65.92) | 39.21 (21.90 to 69.46) | 40.59 (21.11 to 71.95) | 41.52 (22.42 to 71.29) | 42.16 (22.93 to 76.13) | 42.47 (22.83 to 74.56) | 42.11 (22.65 to 73.84) | 41.33 (22.25 to 73.97) | 40.20 (21.51 to 69.18) | 39.73 (22.01 to 70.49) | 39.48 (22.13 to 67.53) | 39.50 (21.64 to 69.69) | 37.58 (20.77 to 64.36) |
| Mali | Both | Prevalence | 284.38 (120.11 to 811.64) | 764.11 (311.30 to 2145.83) | 1005.68 (363.14 to 3015.03) | 1284.18 (416.03 to 4000.73) | 1519.48 (465.05 to 4836.27) | 1690.65 (499.49 to 5450.48) | 1813.63 (524.84 to 5892.73) | 1915.48 (546.51 to 6250.97) | 2007.44 (567.29 to 6559.73) | 2090.23 (588.32 to 6831.01) | 2164.51 (605.90 to 7083.74) | 2238.54 (623.18 to 7341.73) | 2323.12 (641.68 to 7634.87) | 2363.44 (650.60 to 7775.95) | 2363.15 (650.21 to 7774.69) | 2362.54 (649.88 to 7773.35) | 2361.58 (649.06 to 7773.12) | 2363.72 (650.91 to 7774.69) | 2369.56 (653.92 to 7780.82) | 2363.82 (650.77 to 7776.18) |
| Mali | Female | DALY rates | 10.82 (6.35 to 16.54) | 19.18 (11.18 to 29.54) | 19.25 (11.09 to 29.63) | 19.98 (11.71 to 30.96) | 20.01 (11.45 to 31.02) | 20.02 (12.13 to 30.90) | 20.26 (11.54 to 31.70) | 20.12 (11.77 to 30.17) | 20.29 (12.70 to 30.59) | 20.28 (11.21 to 31.30) | 20.30 (11.58 to 31.46) | 20.58 (12.10 to 31.03) | 20.12 (12.14 to 30.11) | 19.80 (11.95 to 30.10) | 19.58 (12.27 to 29.46) | 19.29 (11.22 to 28.52) | 18.87 (11.53 to 27.52) | 18.89 (11.77 to 27.07) | 18.50 (11.63 to 27.36) | 17.62 (10.67 to 26.32) |
| Mali | Female | Prevalence | 300.69 (135.32 to 828.55) | 684.32 (262.96 to 2039.21) | 913.76 (300.74 to 2888.44) | 1177.95 (342.12 to 3865.50) | 1401.76 (378.58 to 4693.54) | 1566.19 (406.07 to 5301.23) | 1684.01 (424.80 to 5737.49) | 1778.38 (440.14 to 6086.28) | 1858.12 (453.73 to 6381.14) | 1927.68 (464.90 to 6638.65) | 1992.89 (475.77 to 6879.66) | 2060.49 (486.64 to 7129.63) | 2137.82 (499.27 to 7415.85) | 2174.68 (505.52 to 7551.39) | 2174.68 (505.30 to 7551.42) | 2174.66 (504.67 to 7551.29) | 2174.68 (504.77 to 7551.26) | 2174.70 (504.98 to 7551.91) | 2174.64 (505.13 to 7551.61) | 2174.69 (505.50 to 7551.89) |
| Mali | Male | DALY rates | 7.74 (4.44 to 12.64) | 39.71 (17.52 to 83.08) | 43.35 (19.02 to 88.50) | 47.87 (21.04 to 100.09) | 51.48 (20.90 to 105.00) | 54.58 (25.22 to 109.47) | 57.07 (25.11 to 112.76) | 58.13 (26.08 to 112.03) | 59.57 (27.60 to 117.64) | 60.41 (26.48 to 117.26) | 61.36 (29.27 to 115.68) | 62.25 (28.70 to 125.62) | 63.22 (29.78 to 119.74) | 62.83 (28.18 to 118.69) | 61.59 (28.93 to 123.38) | 59.80 (28.44 to 116.73) | 59.51 (28.18 to 112.84) | 58.54 (28.15 to 110.90) | 57.72 (28.41 to 112.00) | 56.03 (27.53 to 105.13) |
| Mali | Male | Prevalence | 268.64 (104.29 to 795.33) | 841.46 (346.01 to 2218.80) | 1095.35 (412.29 to 3109.96) | 1390.88 (475.99 to 4131.70) | 1643.87 (533.63 to 5003.26) | 1831.33 (576.59 to 5646.03) | 1966.60 (606.25 to 6105.44) | 2075.55 (632.93 to 6476.83) | 2168.02 (654.16 to 6789.04) | 2248.91 (674.54 to 7058.52) | 2325.08 (693.77 to 7314.93) | 2404.26 (716.19 to 7576.51) | 2495.11 (740.05 to 7879.52) | 2538.69 (751.84 to 8028.66) | 2538.68 (751.80 to 8026.13) | 2538.65 (751.96 to 8023.48) | 2538.66 (751.69 to 8025.99) | 2538.70 (752.60 to 8020.49) | 2538.71 (752.12 to 8021.55) | 2538.67 (752.68 to 8025.76) |
| Marshall Islands | Both | DALY rates | 9.56 (5.84 to 13.98) | 15.82 (9.39 to 23.55) | 15.89 (9.77 to 24.44) | 15.94 (9.80 to 24.79) | 15.64 (9.50 to 23.81) | 15.63 (9.45 to 22.68) | 15.43 (9.82 to 24.24) | 15.33 (9.66 to 23.33) | 15.24 (9.13 to 23.08) | 15.06 (9.28 to 21.78) | 14.99 (9.19 to 22.95) | 14.85 (9.64 to 21.90) | 14.76 (9.04 to 21.40) | 14.40 (9.22 to 21.33) | 14.16 (8.97 to 20.65) | 13.88 (8.74 to 21.02) | 13.85 (9.25 to 20.28) | 13.57 (8.67 to 19.89) | 13.08 (8.38 to 18.78) | 13.06 (8.70 to 18.79) |
| Marshall Islands | Both | Prevalence | 93.79 (87.96 to 99.28) | 156.33 (146.27 to 165.96) | 157.02 (146.87 to 167.94) | 157.63 (147.07 to 169.00) | 157.94 (147.26 to 170.50) | 157.87 (146.70 to 171.10) | 157.69 (146.59 to 171.10) | 158.07 (146.66 to 172.62) | 158.78 (147.45 to 174.05) | 159.16 (148.08 to 174.86) | 159.57 (148.03 to 175.22) | 160.03 (148.29 to 176.70) | 160.31 (148.50 to 177.21) | 159.77 (147.85 to 176.72) | 158.51 (146.73 to 175.19) | 159.04 (147.32 to 175.62) | 159.59 (147.78 to 176.44) | 159.17 (147.30 to 176.31) | 158.53 (146.68 to 175.59) | 161.29 (149.40 to 178.41) |
| Marshall Islands | Female | DALY rates | 11.33 (6.59 to 17.89) | 18.39 (10.27 to 29.35) | 18.61 (10.41 to 28.12) | 18.64 (11.07 to 30.51) | 18.20 (9.83 to 28.94) | 18.27 (10.15 to 27.99) | 18.04 (11.29 to 27.79) | 17.86 (10.56 to 28.18) | 17.67 (10.23 to 26.29) | 17.47 (9.77 to 26.21) | 17.36 (10.56 to 26.83) | 17.17 (10.40 to 26.41) | 16.96 (9.75 to 26.00) | 16.84 (9.52 to 25.43) | 16.60 (9.67 to 23.64) | 16.19 (9.74 to 24.94) | 16.08 (9.33 to 24.07) | 15.75 (9.65 to 23.92) | 15.38 (9.42 to 23.40) | 15.10 (9.56 to 22.29) |
| Marshall Islands | Female | Prevalence | 110.45 (102.29 to 119.83) | 183.42 (169.74 to 199.17) | 183.90 (170.17 to 199.47) | 184.44 (170.08 to 201.73) | 184.87 (170.04 to 202.88) | 185.22 (170.08 to 204.13) | 185.46 (170.44 to 204.52) | 185.66 (170.36 to 205.42) | 185.83 (171.06 to 205.80) | 185.96 (170.92 to 206.55) | 186.08 (170.73 to 206.22) | 186.23 (170.86 to 207.30) | 186.40 (171.11 to 207.89) | 186.48 (171.03 to 208.32) | 186.49 (171.20 to 208.59) | 186.48 (171.02 to 208.10) | 186.47 (171.29 to 207.61) | 186.48 (171.00 to 209.19) | 186.47 (170.99 to 208.50) | 186.48 (170.84 to 208.43) |
| Marshall Islands | Male | DALY rates | 7.90 (4.31 to 12.57) | 13.37 (7.21 to 22.18) | 13.25 (7.62 to 21.67) | 13.30 (6.66 to 21.93) | 13.18 (7.23 to 22.02) | 13.17 (7.39 to 20.66) | 13.06 (6.73 to 22.10) | 13.01 (7.23 to 20.60) | 12.92 (6.77 to 21.29) | 12.72 (6.83 to 20.21) | 12.62 (6.88 to 20.84) | 12.49 (6.73 to 20.06) | 12.49 (6.74 to 19.98) | 12.01 (6.85 to 18.82) | 11.99 (6.29 to 18.81) | 11.73 (6.58 to 19.19) | 11.70 (6.81 to 18.51) | 11.53 (6.67 to 18.17) | 11.01 (6.03 to 17.76) | 10.81 (6.07 to 16.50) |
| Marshall Islands | Male | Prevalence | 78.21 (71.53 to 85.84) | 130.49 (119.15 to 143.44) | 130.98 (119.27 to 144.80) | 131.51 (119.19 to 146.26) | 131.95 (119.42 to 148.08) | 132.30 (119.46 to 149.90) | 132.54 (119.57 to 150.42) | 132.72 (119.67 to 150.76) | 132.90 (119.68 to 151.23) | 133.04 (119.74 to 152.13) | 133.19 (119.91 to 152.69) | 133.32 (119.84 to 153.16) | 133.49 (119.93 to 154.26) | 133.56 (120.03 to 154.65) | 133.56 (119.74 to 154.56) | 133.54 (120.15 to 154.47) | 133.57 (120.06 to 154.53) | 133.57 (119.93 to 154.48) | 133.55 (119.97 to 154.68) | 133.57 (120.04 to 154.31) |
| Micronesia (Federated States of) | Both | DALY rates | 10.12 (6.36 to 15.33) | 32.62 (17.45 to 59.11) | 35.84 (18.88 to 63.41) | 40.27 (21.33 to 73.29) | 44.49 (23.32 to 81.50) | 47.28 (25.11 to 85.14) | 49.59 (26.01 to 88.02) | 51.48 (28.11 to 90.61) | 52.93 (28.67 to 90.52) | 54.05 (29.08 to 96.19) | 53.96 (28.77 to 95.36) | 55.28 (29.44 to 95.79) | 57.04 (31.30 to 99.14) | 55.33 (31.29 to 95.68) | 51.78 (29.00 to 90.50) | 47.48 (27.89 to 77.02) | 44.29 (26.35 to 73.23) | 42.69 (24.35 to 67.88) | 42.49 (24.80 to 67.34) | 41.56 (24.53 to 70.69) |
| Micronesia (Federated States of) | Both | Prevalence | 412.86 (104.58 to 2637.29) | 1055.26 (267.56 to 6401.27) | 1431.22 (301.92 to 9230.23) | 1862.73 (345.35 to 12448.94) | 2234.29 (387.02 to 15195.30) | 2510.64 (418.57 to 17218.94) | 2709.65 (441.98 to 18665.02) | 2870.08 (462.29 to 19829.53) | 3005.75 (479.96 to 20814.76) | 3122.38 (491.94 to 21669.37) | 3228.38 (501.75 to 22472.67) | 3345.04 (516.31 to 23312.20) | 3479.79 (538.42 to 24253.02) | 3530.38 (537.10 to 24705.82) | 3508.90 (518.67 to 24692.79) | 3480.79 (498.53 to 24676.24) | 3456.65 (483.70 to 24660.64) | 3449.53 (479.54 to 24657.46) | 3460.45 (484.95 to 24663.77) | 3455.09 (482.88 to 24659.31) |
| Micronesia (Federated States of) | Female | DALY rates | 11.87 (7.08 to 18.62) | 19.74 (11.18 to 29.44) | 20.38 (11.66 to 31.13) | 21.04 (12.13 to 33.70) | 21.42 (12.27 to 33.67) | 21.73 (13.02 to 34.29) | 21.88 (12.32 to 33.34) | 21.84 (13.11 to 33.45) | 21.90 (13.27 to 33.27) | 22.10 (13.53 to 33.48) | 22.00 (12.64 to 34.06) | 22.04 (13.26 to 33.42) | 22.25 (13.22 to 34.35) | 21.59 (13.17 to 31.68) | 21.65 (12.91 to 32.61) | 21.02 (12.84 to 31.68) | 20.76 (12.46 to 30.78) | 20.24 (12.31 to 30.31) | 19.56 (12.08 to 27.90) | 19.29 (11.64 to 28.07) |
| Micronesia (Federated States of) | Female | Prevalence | 430.50 (121.84 to 2653.90) | 958.79 (207.27 to 6364.33) | 1312.19 (220.68 to 9179.02) | 1714.04 (233.60 to 12378.82) | 2056.65 (244.58 to 15104.91) | 2308.93 (254.52 to 17112.84) | 2488.99 (259.98 to 18544.91) | 2634.08 (265.19 to 19698.50) | 2756.82 (270.52 to 20673.41) | 2863.25 (273.84 to 21519.77) | 2963.65 (277.21 to 22317.73) | 3068.07 (281.09 to 23147.96) | 3184.68 (285.94 to 24075.50) | 3242.07 (287.39 to 24530.46) | 3242.06 (287.86 to 24530.91) | 3242.07 (288.01 to 24531.04) | 3242.06 (287.80 to 24530.04) | 3242.09 (287.61 to 24531.36) | 3242.07 (287.26 to 24530.78) | 3242.09 (288.08 to 24529.97) |
| Micronesia (Federated States of) | Male | DALY rates | 8.49 (4.78 to 13.35) | 44.76 (20.20 to 92.02) | 50.40 (22.46 to 100.23) | 58.41 (26.09 to 120.98) | 66.29 (29.34 to 134.25) | 71.34 (32.55 to 138.79) | 75.54 (35.17 to 143.38) | 79.29 (37.63 to 151.56) | 82.27 (39.64 to 153.56) | 84.77 (39.30 to 159.96) | 86.18 (41.50 to 157.80) | 88.87 (43.19 to 166.94) | 91.32 (45.57 to 167.53) | 91.84 (45.75 to 172.06) | 89.43 (44.77 to 166.26) | 87.55 (45.47 to 158.78) | 86.59 (44.35 to 157.07) | 85.21 (42.02 to 153.12) | 82.60 (42.29 to 146.77) | 82.06 (40.79 to 156.06) |
| Micronesia (Federated States of) | Male | Prevalence | 396.36 (88.18 to 2621.75) | 1146.16 (305.23 to 6436.08) | 1543.34 (359.03 to 9278.46) | 2002.97 (416.48 to 12515.08) | 2402.08 (477.80 to 15280.68) | 2700.68 (523.81 to 17318.89) | 2916.25 (558.44 to 18777.47) | 3091.53 (587.35 to 19952.48) | 3241.11 (615.88 to 20948.39) | 3371.55 (639.53 to 21813.22) | 3495.31 (663.14 to 22628.90) | 3624.93 (689.62 to 23478.18) | 3770.51 (722.01 to 24427.89) | 3842.37 (736.45 to 24895.59) | 3842.48 (734.90 to 24895.15) | 3842.38 (734.76 to 24896.17) | 3842.44 (732.78 to 24895.44) | 3842.45 (736.69 to 24896.29) | 3842.42 (734.34 to 24896.36) | 3842.44 (736.68 to 24894.52) |
| Mozambique | Both | DALY rates | 9.73 (6.01 to 14.57) | 28.33 (15.69 to 53.20) | 29.01 (15.22 to 51.40) | 29.63 (15.90 to 52.68) | 30.14 (16.57 to 55.35) | 30.73 (16.90 to 53.92) | 31.35 (16.71 to 56.88) | 31.65 (17.72 to 54.42) | 31.83 (17.82 to 56.70) | 31.68 (17.07 to 59.03) | 31.50 (16.89 to 56.35) | 31.89 (17.61 to 55.06) | 32.43 (18.01 to 58.20) | 31.40 (17.81 to 55.99) | 30.04 (16.96 to 52.39) | 28.46 (16.43 to 48.02) | 26.51 (15.47 to 44.20) | 24.64 (14.17 to 39.10) | 22.99 (13.41 to 36.72) | 21.34 (12.58 to 32.55) |
| Mozambique | Both | Prevalence | 212.74 (124.16 to 436.19) | 549.87 (306.28 to 1106.53) | 690.79 (349.90 to 1482.33) | 852.83 (393.60 to 1930.28) | 989.05 (424.39 to 2313.13) | 1092.12 (451.36 to 2594.55) | 1167.07 (473.82 to 2797.59) | 1226.76 (490.64 to 2961.34) | 1276.64 (505.85 to 3097.42) | 1319.62 (517.05 to 3216.51) | 1359.80 (529.15 to 3328.71) | 1402.85 (541.22 to 3444.69) | 1453.69 (556.49 to 3579.77) | 1472.65 (560.66 to 3639.61) | 1465.50 (555.71 to 3634.40) | 1454.94 (543.61 to 3624.62) | 1443.00 (529.44 to 3613.50) | 1431.36 (516.63 to 3604.02) | 1422.28 (511.07 to 3596.53) | 1410.22 (504.05 to 3584.75) |
| Mozambique | Female | DALY rates | 11.37 (6.73 to 18.24) | 19.03 (11.09 to 29.31) | 19.13 (11.03 to 29.85) | 19.07 (11.39 to 29.65) | 19.19 (11.39 to 30.02) | 18.92 (11.22 to 28.49) | 18.93 (10.80 to 29.82) | 18.71 (11.10 to 28.86) | 18.90 (11.66 to 28.50) | 18.79 (11.21 to 29.44) | 19.03 (11.22 to 29.42) | 18.87 (11.06 to 28.65) | 18.71 (11.27 to 28.35) | 18.62 (10.89 to 27.99) | 18.28 (10.93 to 29.32) | 18.19 (10.68 to 27.42) | 17.63 (10.37 to 27.46) | 17.30 (10.05 to 26.44) | 16.74 (9.90 to 24.63) | 16.53 (9.88 to 24.43) |
| Mozambique | Female | Prevalence | 229.36 (138.75 to 449.55) | 481.26 (260.33 to 1029.65) | 616.32 (295.24 to 1414.04) | 773.29 (334.17 to 1861.81) | 906.20 (367.47 to 2239.92) | 1003.74 (392.35 to 2518.08) | 1073.30 (409.32 to 2716.48) | 1129.72 (423.08 to 2876.61) | 1176.51 (435.39 to 3009.98) | 1218.04 (445.06 to 3128.14) | 1256.47 (454.22 to 3238.33) | 1296.54 (465.46 to 3352.42) | 1342.72 (476.41 to 3483.64) | 1364.44 (481.96 to 3545.29) | 1364.45 (481.36 to 3544.97) | 1364.42 (482.48 to 3545.21) | 1364.43 (481.45 to 3544.75) | 1364.42 (481.40 to 3544.42) | 1364.43 (481.10 to 3545.88) | 1364.42 (481.44 to 3544.68) |
| Mozambique | Male | DALY rates | 8.11 (4.59 to 12.44) | 37.67 (17.60 to 80.28) | 39.06 (17.36 to 81.38) | 41.16 (17.98 to 84.66) | 43.08 (18.77 to 92.80) | 44.56 (20.57 to 89.45) | 45.41 (20.38 to 93.87) | 46.31 (21.85 to 91.41) | 46.37 (21.05 to 92.74) | 46.44 (20.30 to 99.59) | 45.91 (20.18 to 93.58) | 46.79 (21.12 to 93.74) | 47.66 (21.10 to 95.88) | 46.59 (20.95 to 95.33) | 45.84 (20.72 to 94.23) | 45.05 (21.10 to 92.26) | 44.40 (21.59 to 89.58) | 43.28 (20.22 to 86.89) | 42.34 (18.88 to 82.32) | 41.41 (18.41 to 82.08) |
| Mozambique | Male | Prevalence | 196.39 (108.90 to 423.35) | 618.71 (328.15 to 1205.04) | 766.66 (390.72 to 1592.71) | 939.64 (456.26 to 2059.81) | 1086.88 (506.82 to 2442.84) | 1195.59 (543.52 to 2722.34) | 1273.34 (568.59 to 2925.91) | 1336.65 (591.42 to 3084.01) | 1389.17 (610.24 to 3220.21) | 1435.88 (627.35 to 3339.80) | 1479.28 (642.19 to 3452.19) | 1524.54 (657.89 to 3566.01) | 1576.79 (675.95 to 3700.24) | 1601.38 (683.67 to 3763.75) | 1601.36 (683.31 to 3765.84) | 1601.34 (684.01 to 3764.41) | 1601.35 (684.99 to 3765.03) | 1601.35 (684.35 to 3767.11) | 1601.40 (684.93 to 3764.99) | 1601.38 (683.86 to 3764.59) |
| Myanmar | Both | DALY rates | 9.90 (5.94 to 14.77) | 30.56 (16.26 to 55.95) | 32.24 (16.93 to 58.55) | 34.67 (17.99 to 62.68) | 36.72 (18.96 to 67.70) | 37.95 (20.83 to 68.31) | 39.30 (20.90 to 70.39) | 40.27 (21.33 to 73.12) | 40.94 (22.34 to 73.57) | 40.90 (22.66 to 73.37) | 40.82 (23.06 to 70.91) | 40.92 (22.26 to 72.28) | 41.12 (22.50 to 70.59) | 40.08 (22.28 to 67.71) | 38.77 (21.78 to 67.44) | 36.84 (21.84 to 61.28) | 35.34 (20.31 to 58.78) | 33.52 (19.96 to 56.51) | 31.34 (18.34 to 51.36) | 27.47 (16.85 to 43.34) |
| Myanmar | Both | Prevalence | 312.63 (109.13 to 1078.15) | 806.14 (267.39 to 2703.57) | 1065.79 (301.17 to 3820.49) | 1363.78 (341.70 to 5098.24) | 1616.38 (374.53 to 6179.57) | 1802.54 (399.72 to 6974.44) | 1937.30 (418.01 to 7546.31) | 2046.21 (433.37 to 8007.29) | 2136.22 (446.88 to 8391.17) | 2213.91 (455.66 to 8727.39) | 2286.25 (464.45 to 9041.87) | 2361.13 (474.60 to 9367.44) | 2447.69 (487.38 to 9740.07) | 2485.38 (491.58 to 9914.49) | 2480.97 (489.62 to 9908.60) | 2471.85 (484.35 to 9898.28) | 2464.18 (478.56 to 9889.87) | 2454.06 (469.60 to 9877.55) | 2438.11 (456.31 to 9858.83) | 2409.83 (436.11 to 9826.74) |
| Myanmar | Female | DALY rates | 11.54 (6.57 to 17.58) | 19.68 (11.89 to 31.81) | 19.36 (11.25 to 31.18) | 20.08 (11.70 to 30.90) | 20.23 (11.81 to 32.22) | 20.56 (12.45 to 32.82) | 20.93 (12.59 to 32.33) | 20.92 (12.53 to 32.92) | 20.84 (11.83 to 32.86) | 20.59 (12.05 to 31.20) | 20.79 (12.25 to 31.86) | 20.69 (11.80 to 31.34) | 20.64 (11.40 to 32.26) | 20.43 (13.11 to 29.99) | 19.95 (12.34 to 30.55) | 19.35 (11.77 to 29.19) | 19.18 (11.39 to 28.96) | 18.55 (11.15 to 27.43) | 18.25 (11.12 to 28.07) | 17.68 (11.29 to 26.60) |
| Myanmar | Female | Prevalence | 329.69 (124.92 to 1096.06) | 724.04 (220.87 to 2608.25) | 970.59 (239.34 to 3710.66) | 1252.52 (261.92 to 4969.62) | 1491.11 (281.60 to 6035.17) | 1666.54 (294.07 to 6818.56) | 1792.48 (303.88 to 7381.32) | 1893.51 (312.39 to 7832.35) | 1978.26 (318.07 to 8210.97) | 2052.87 (325.20 to 8543.17) | 2122.65 (330.34 to 8855.11) | 2194.84 (335.34 to 9176.68) | 2277.61 (342.29 to 9545.83) | 2316.84 (345.75 to 9721.37) | 2316.85 (345.84 to 9721.41) | 2316.87 (345.65 to 9722.19) | 2316.86 (345.62 to 9721.35) | 2316.83 (345.85 to 9720.86) | 2316.85 (344.84 to 9720.80) | 2316.85 (346.49 to 9721.16) |
| Myanmar | Male | DALY rates | 8.33 (4.42 to 13.50) | 41.06 (17.93 to 89.39) | 44.81 (19.47 to 90.01) | 49.25 (21.14 to 102.59) | 53.75 (24.80 to 108.95) | 56.40 (26.42 to 108.28) | 58.96 (25.69 to 118.51) | 60.97 (26.65 to 121.17) | 62.85 (29.12 to 124.30) | 63.83 (29.18 to 125.30) | 64.27 (29.78 to 120.81) | 65.47 (30.12 to 130.29) | 66.80 (31.50 to 126.61) | 66.01 (30.50 to 127.70) | 64.77 (31.70 to 127.22) | 63.46 (30.96 to 122.35) | 62.07 (29.96 to 118.17) | 61.18 (30.61 to 119.23) | 60.46 (28.64 to 114.24) | 58.83 (27.33 to 114.98) |
| Myanmar | Male | Prevalence | 296.32 (94.38 to 1062.15) | 885.39 (306.13 to 2798.48) | 1158.80 (349.49 to 3928.94) | 1475.02 (396.78 to 5226.84) | 1745.80 (441.66 to 6328.74) | 1946.87 (479.44 to 7139.87) | 2092.24 (506.14 to 7722.85) | 2209.54 (530.70 to 8194.42) | 2308.39 (549.44 to 8587.58) | 2395.73 (568.04 to 8935.38) | 2477.80 (583.84 to 9260.55) | 2563.00 (599.03 to 9599.02) | 2661.00 (616.66 to 9983.69) | 2707.68 (626.39 to 10169.22) | 2707.72 (626.37 to 10167.22) | 2707.70 (625.62 to 10166.24) | 2707.70 (626.72 to 10168.43) | 2707.67 (626.37 to 10167.13) | 2707.78 (626.13 to 10165.79) | 2707.69 (625.79 to 10164.96) |
| Nepal | Both | DALY rates | 10.03 (6.16 to 15.25) | 33.46 (17.33 to 62.58) | 36.90 (19.48 to 66.55) | 41.43 (21.76 to 69.73) | 44.91 (24.45 to 79.63) | 46.63 (25.60 to 81.97) | 48.02 (26.46 to 82.95) | 49.38 (26.80 to 85.96) | 51.95 (28.55 to 89.62) | 54.53 (29.56 to 95.05) | 56.62 (30.09 to 97.20) | 58.75 (32.99 to 100.59) | 60.09 (33.48 to 104.08) | 59.89 (34.39 to 99.55) | 58.69 (32.59 to 100.72) | 57.98 (32.84 to 99.58) | 57.00 (30.75 to 96.77) | 55.35 (31.77 to 92.22) | 52.42 (30.14 to 85.50) | 47.46 (27.96 to 78.96) |
| Nepal | Both | Prevalence | 442.60 (356.69 to 577.30) | 1151.72 (896.34 to 1563.24) | 1575.54 (1222.37 to 2142.52) | 2061.01 (1592.81 to 2790.07) | 2466.77 (1909.25 to 3342.45) | 2763.47 (2140.68 to 3748.61) | 2976.54 (2307.54 to 4039.70) | 3151.97 (2442.77 to 4278.46) | 3308.93 (2563.17 to 4493.59) | 3452.30 (2673.14 to 4688.41) | 3589.73 (2778.94 to 4873.62) | 3726.05 (2881.40 to 5058.61) | 3874.13 (2997.51 to 5259.99) | 3941.92 (3050.91 to 5351.73) | 3941.49 (3049.34 to 5351.79) | 3943.32 (3051.98 to 5353.89) | 3942.43 (3050.65 to 5351.74) | 3934.92 (3045.30 to 5342.27) | 3913.88 (3029.71 to 5315.74) | 3877.35 (3003.09 to 5268.55) |
| Nepal | Female | DALY rates | 11.89 (6.43 to 18.46) | 19.91 (12.04 to 30.24) | 20.67 (11.08 to 33.34) | 21.02 (12.86 to 32.60) | 21.74 (13.17 to 33.36) | 21.86 (13.70 to 32.47) | 22.18 (13.39 to 35.18) | 22.36 (13.58 to 35.02) | 22.41 (13.29 to 35.91) | 22.39 (13.90 to 34.82) | 22.17 (14.04 to 33.17) | 22.22 (13.85 to 33.47) | 22.59 (13.56 to 34.09) | 22.15 (13.41 to 33.63) | 21.68 (13.58 to 32.67) | 21.31 (13.27 to 31.95) | 21.21 (12.99 to 32.17) | 20.97 (13.33 to 30.58) | 20.77 (13.08 to 32.55) | 20.11 (11.84 to 29.71) |
| Nepal | Female | Prevalence | 460.20 (374.14 to 595.95) | 1050.47 (837.55 to 1385.15) | 1449.13 (1137.99 to 1937.64) | 1904.09 (1484.17 to 2567.98) | 2286.15 (1772.74 to 3096.44) | 2567.17 (1985.06 to 3484.82) | 2769.66 (2137.98 to 3764.68) | 2932.30 (2261.67 to 3990.32) | 3069.37 (2365.38 to 4180.03) | 3189.33 (2456.91 to 4346.28) | 3301.98 (2541.58 to 4501.81) | 3418.42 (2629.79 to 4662.95) | 3552.13 (2730.45 to 4847.02) | 3614.76 (2778.05 to 4934.39) | 3614.71 (2777.68 to 4934.46) | 3614.77 (2777.66 to 4934.88) | 3614.77 (2778.33 to 4934.17) | 3614.75 (2778.34 to 4934.19) | 3614.76 (2778.97 to 4934.33) | 3614.77 (2777.80 to 4934.85) |
| Nepal | Male | DALY rates | 8.29 (4.87 to 13.32) | 46.30 (20.35 to 97.84) | 52.53 (23.32 to 106.59) | 62.22 (27.55 to 117.27) | 70.96 (33.33 to 138.68) | 77.23 (36.31 to 147.00) | 82.37 (40.28 to 155.24) | 86.14 (40.43 to 167.83) | 90.32 (44.46 to 170.64) | 93.26 (45.67 to 176.58) | 94.99 (45.40 to 176.33) | 98.07 (48.74 to 180.28) | 101.20 (50.02 to 185.98) | 102.02 (53.00 to 187.29) | 100.10 (52.46 to 176.80) | 98.59 (51.50 to 177.71) | 96.83 (46.96 to 175.42) | 95.32 (49.87 to 171.66) | 94.03 (50.21 to 167.35) | 92.23 (48.16 to 162.64) |
| Nepal | Male | Prevalence | 426.11 (339.87 to 559.83) | 1247.67 (930.99 to 1753.50) | 1697.34 (1273.04 to 2347.68) | 2220.81 (1673.89 to 3075.48) | 2669.81 (2015.01 to 3687.15) | 3005.95 (2271.15 to 4140.22) | 3251.42 (2457.04 to 4459.94) | 3450.76 (2607.82 to 4727.66) | 3620.00 (2738.68 to 4962.40) | 3769.21 (2851.23 to 5162.44) | 3910.30 (2959.28 to 5351.84) | 4057.25 (3073.61 to 5543.77) | 4227.18 (3202.91 to 5768.29) | 4307.17 (3268.11 to 5876.20) | 4307.14 (3267.30 to 5878.06) | 4307.19 (3267.62 to 5877.98) | 4307.03 (3268.15 to 5875.83) | 4307.12 (3268.03 to 5874.74) | 4307.18 (3265.57 to 5874.09) | 4307.05 (3268.06 to 5874.78) |
| Niger | Both | DALY rates | 9.29 (5.59 to 13.96) | 29.70 (15.45 to 52.30) | 31.64 (17.03 to 60.88) | 33.49 (18.25 to 60.32) | 35.46 (18.92 to 63.59) | 36.88 (19.33 to 67.72) | 38.12 (20.03 to 69.78) | 39.01 (20.85 to 70.97) | 38.90 (21.98 to 69.96) | 38.28 (21.35 to 69.02) | 39.11 (20.60 to 68.55) | 40.69 (21.78 to 75.22) | 41.95 (23.04 to 74.73) | 41.10 (22.23 to 72.03) | 39.58 (21.66 to 69.05) | 38.95 (21.73 to 68.41) | 38.25 (21.75 to 66.73) | 37.83 (20.77 to 63.64) | 38.08 (21.19 to 64.62) | 35.83 (20.47 to 60.97) |
| Niger | Both | Prevalence | 279.21 (157.07 to 611.30) | 753.16 (420.35 to 1626.06) | 988.68 (523.24 to 2275.18) | 1258.77 (630.33 to 3014.66) | 1492.10 (729.05 to 3630.86) | 1665.23 (801.73 to 4083.39) | 1791.50 (855.44 to 4412.87) | 1890.62 (896.71 to 4672.97) | 1967.41 (925.84 to 4880.53) | 2033.89 (953.94 to 5062.79) | 2107.38 (986.52 to 5250.80) | 2186.55 (1022.74 to 5449.38) | 2275.35 (1062.08 to 5672.93) | 2310.47 (1075.83 to 5768.68) | 2304.62 (1072.37 to 5759.84) | 2305.99 (1073.89 to 5761.86) | 2304.03 (1072.93 to 5759.55) | 2306.85 (1074.77 to 5763.23) | 2314.08 (1078.77 to 5774.62) | 2299.22 (1070.08 to 5752.22) |
| Niger | Female | DALY rates | 10.86 (6.26 to 17.06) | 19.40 (10.70 to 30.56) | 19.77 (11.54 to 32.69) | 19.95 (11.24 to 30.62) | 20.38 (11.94 to 31.44) | 20.35 (11.39 to 32.88) | 20.18 (12.01 to 30.35) | 20.21 (12.32 to 31.01) | 20.46 (12.32 to 31.86) | 20.29 (11.73 to 30.81) | 20.32 (12.25 to 31.13) | 20.19 (12.05 to 30.06) | 20.50 (11.87 to 31.22) | 19.87 (12.11 to 30.49) | 19.61 (11.28 to 30.06) | 19.40 (11.75 to 29.29) | 18.89 (11.58 to 28.37) | 18.69 (11.74 to 28.32) | 18.39 (10.98 to 27.27) | 18.09 (11.14 to 26.67) |
| Niger | Female | Prevalence | 295.21 (171.83 to 627.01) | 673.68 (358.18 to 1526.46) | 898.27 (441.92 to 2139.19) | 1156.07 (536.28 to 2843.64) | 1376.50 (619.88 to 3444.62) | 1537.22 (679.57 to 3882.47) | 1652.89 (722.10 to 4198.58) | 1745.79 (757.46 to 4451.56) | 1823.19 (785.90 to 4662.07) | 1891.46 (811.82 to 4849.32) | 1956.22 (836.08 to 5025.30) | 2021.68 (859.97 to 5204.18) | 2097.83 (888.98 to 5412.08) | 2133.65 (901.99 to 5509.88) | 2133.66 (901.96 to 5509.07) | 2133.67 (902.79 to 5508.91) | 2133.64 (901.68 to 5508.45) | 2133.66 (902.07 to 5509.33) | 2133.64 (901.73 to 5509.51) | 2133.67 (901.83 to 5508.90) |
| Niger | Male | DALY rates | 7.78 (4.17 to 12.80) | 39.65 (17.30 to 82.16) | 43.40 (18.92 to 95.24) | 47.67 (22.71 to 96.91) | 51.53 (23.06 to 101.43) | 54.10 (22.89 to 109.03) | 56.19 (25.65 to 114.77) | 58.17 (25.68 to 117.05) | 59.42 (27.33 to 117.63) | 60.18 (27.46 to 116.67) | 60.92 (25.56 to 119.52) | 62.18 (28.99 to 126.26) | 62.90 (29.33 to 123.20) | 62.75 (28.54 to 120.09) | 61.35 (28.37 to 119.46) | 59.93 (28.05 to 115.87) | 59.47 (29.38 to 114.10) | 58.18 (26.92 to 108.41) | 57.37 (27.02 to 106.61) | 56.36 (26.30 to 107.66) |
| Niger | Male | Prevalence | 263.83 (142.67 to 596.20) | 829.85 (450.88 to 1751.48) | 1078.28 (559.79 to 2331.01) | 1366.43 (691.90 to 3092.50) | 1615.35 (805.73 to 3745.43) | 1798.61 (891.49 to 4224.04) | 1931.17 (953.52 to 4570.01) | 2038.29 (1004.07 to 4847.37) | 2127.89 (1046.94 to 5079.76) | 2207.20 (1085.19 to 5284.07) | 2282.78 (1120.25 to 5480.41) | 2359.32 (1156.24 to 5678.66) | 2448.75 (1197.62 to 5908.44) | 2490.90 (1218.23 to 6015.19) | 2490.93 (1218.17 to 6015.32) | 2490.89 (1218.08 to 6015.50) | 2490.79 (1218.33 to 6016.93) | 2490.88 (1218.45 to 6015.87) | 2490.93 (1217.17 to 6015.84) | 2490.86 (1217.52 to 6013.65) |
| Nigeria | Both | DALY rates | 9.35 (6.42 to 13.23) | 28.46 (16.25 to 50.87) | 30.54 (17.37 to 54.95) | 32.50 (18.31 to 58.05) | 34.42 (19.52 to 61.16) | 35.99 (20.60 to 62.99) | 37.56 (21.29 to 66.63) | 38.87 (22.14 to 67.81) | 40.16 (22.81 to 69.50) | 40.49 (23.01 to 70.51) | 40.01 (23.14 to 68.99) | 39.62 (22.91 to 67.49) | 40.23 (23.54 to 67.66) | 41.50 (24.08 to 69.92) | 42.04 (24.31 to 71.84) | 41.10 (23.93 to 70.10) | 40.00 (22.96 to 68.39) | 40.43 (23.57 to 68.42) | 40.92 (24.09 to 68.83) | 33.41 (21.06 to 52.22) |
| Nigeria | Both | Prevalence | 276.68 (142.52 to 514.22) | 721.26 (365.63 to 1295.22) | 950.24 (449.62 to 1797.68) | 1214.90 (533.49 to 2394.11) | 1449.77 (612.18 to 2890.82) | 1634.66 (673.19 to 3312.52) | 1770.59 (726.26 to 3656.24) | 1876.86 (767.90 to 3946.02) | 1958.12 (798.89 to 4104.62) | 2021.72 (819.76 to 4193.47) | 2080.06 (834.62 to 4306.16) | 2151.36 (854.57 to 4517.99) | 2284.84 (903.62 to 4963.12) | 2405.66 (953.00 to 5194.37) | 2436.99 (963.07 to 5247.24) | 2368.98 (945.03 to 5116.63) | 2300.69 (900.16 to 5010.48) | 2369.26 (949.85 to 5159.92) | 2502.91 (989.56 to 5690.68) | 2606.94 (967.38 to 6351.57) |
| Nigeria | Female | DALY rates | 11.00 (7.26 to 15.99) | 18.92 (12.76 to 27.24) | 19.03 (12.98 to 27.11) | 19.33 (13.09 to 27.91) | 19.46 (13.00 to 27.52) | 19.66 (13.24 to 27.53) | 19.71 (13.46 to 28.42) | 19.72 (13.47 to 27.70) | 19.81 (13.55 to 27.53) | 19.80 (13.49 to 27.99) | 19.73 (13.69 to 27.69) | 19.74 (13.74 to 27.14) | 19.88 (13.59 to 27.61) | 19.95 (13.75 to 27.30) | 19.64 (13.59 to 27.00) | 18.90 (13.01 to 25.86) | 18.16 (12.30 to 25.30) | 18.26 (12.61 to 25.37) | 18.27 (12.56 to 25.13) | 17.94 (12.93 to 24.31) |
| Nigeria | Female | Prevalence | 292.92 (157.93 to 533.51) | 647.09 (311.87 to 1244.59) | 859.64 (375.92 to 1734.28) | 1112.94 (454.81 to 2308.68) | 1340.66 (524.29 to 2822.86) | 1516.12 (572.66 to 3222.46) | 1637.36 (608.40 to 3567.16) | 1725.95 (632.27 to 3774.75) | 1792.98 (650.56 to 3911.57) | 1857.14 (671.41 to 4088.03) | 1926.60 (696.15 to 4287.39) | 2008.39 (725.52 to 4566.16) | 2164.80 (782.51 to 4994.91) | 2309.36 (821.03 to 5340.49) | 2329.05 (805.20 to 5434.98) | 2205.15 (780.63 to 4931.48) | 2100.98 (733.59 to 4806.66) | 2198.27 (765.99 to 5156.26) | 2385.44 (815.88 to 5937.97) | 2569.80 (838.70 to 6773.77) |
| Nigeria | Male | DALY rates | 7.73 (5.25 to 10.98) | 38.06 (18.73 to 77.04) | 42.72 (20.44 to 85.90) | 47.19 (22.82 to 94.94) | 51.66 (24.77 to 103.08) | 55.29 (26.90 to 108.39) | 58.28 (29.06 to 115.10) | 60.62 (30.06 to 118.18) | 62.40 (31.10 to 119.97) | 63.46 (31.49 to 122.87) | 64.47 (32.60 to 120.21) | 65.59 (32.78 to 124.45) | 67.55 (34.76 to 125.77) | 68.08 (34.54 to 125.94) | 67.04 (34.35 to 124.42) | 64.62 (33.35 to 118.98) | 62.56 (32.19 to 114.52) | 62.67 (32.70 to 114.30) | 63.55 (33.43 to 116.15) | 63.56 (34.09 to 111.63) |
| Nigeria | Male | Prevalence | 260.74 (123.84 to 497.46) | 795.80 (414.97 to 1371.14) | 1046.00 (519.30 to 1935.10) | 1328.52 (622.82 to 2510.04) | 1575.57 (719.59 to 2978.25) | 1774.73 (796.20 to 3418.82) | 1925.17 (862.69 to 3774.87) | 2048.22 (916.45 to 4136.36) | 2138.65 (955.44 to 4310.12) | 2204.56 (985.08 to 4344.65) | 2265.09 (1008.61 to 4373.48) | 2338.12 (1046.48 to 4460.66) | 2445.97 (1096.27 to 4705.99) | 2524.35 (1126.29 to 4848.03) | 2557.52 (1129.72 to 4929.36) | 2542.52 (1104.02 to 5211.06) | 2506.93 (1069.93 to 5231.35) | 2540.77 (1122.81 to 5214.37) | 2620.27 (1168.45 to 5443.40) | 2679.32 (1182.48 to 5483.87) |
| Niue | Both | DALY rates | 9.99 (6.23 to 14.70) | 30.10 (15.92 to 56.12) | 32.31 (17.22 to 59.66) | 34.94 (17.43 to 66.98) | 35.62 (18.80 to 64.35) | 36.26 (19.30 to 66.38) | 37.20 (19.31 to 66.42) | 38.22 (20.33 to 68.50) | 38.72 (20.80 to 70.23) | 39.28 (21.04 to 71.54) | 39.92 (21.51 to 71.76) | 40.62 (21.28 to 74.31) | 40.80 (21.69 to 73.47) | 38.62 (21.60 to 67.56) | 35.42 (19.99 to 61.08) | 33.08 (19.33 to 54.91) | 32.66 (18.39 to 55.92) | 31.64 (18.72 to 54.55) | 28.84 (17.16 to 47.03) | 27.15 (17.20 to 43.06) |
| Niue | Both | Prevalence | 290.18 (122.92 to 840.51) | 733.67 (298.71 to 2023.89) | 965.92 (341.52 to 2853.62) | 1227.02 (392.33 to 3795.94) | 1442.44 (429.46 to 4599.93) | 1601.92 (456.94 to 5191.76) | 1718.21 (480.24 to 5612.01) | 1816.54 (499.34 to 5963.40) | 1895.79 (514.84 to 6246.70) | 1967.01 (529.03 to 6499.38) | 2036.33 (544.83 to 6739.33) | 2107.09 (559.16 to 6983.32) | 2183.44 (575.36 to 7256.33) | 2203.93 (568.83 to 7380.58) | 2183.40 (556.51 to 7365.92) | 2167.65 (546.36 to 7355.30) | 2169.30 (546.78 to 7356.27) | 2167.73 (546.06 to 7355.50) | 2147.21 (532.58 to 7341.75) | 2134.67 (524.41 to 7332.95) |
| Niue | Female | DALY rates | 11.77 (6.91 to 17.93) | 19.55 (11.20 to 30.36) | 19.75 (11.17 to 30.25) | 20.13 (12.08 to 32.00) | 20.23 (11.78 to 30.61) | 20.28 (11.63 to 31.20) | 20.37 (12.48 to 32.32) | 20.30 (12.26 to 32.02) | 20.25 (12.02 to 31.92) | 20.27 (11.39 to 31.23) | 20.05 (11.77 to 30.18) | 20.21 (11.98 to 30.93) | 19.96 (11.67 to 30.00) | 19.82 (11.24 to 30.15) | 19.28 (11.47 to 29.21) | 19.20 (11.76 to 29.38) | 18.59 (11.63 to 28.03) | 18.27 (11.15 to 27.44) | 17.76 (11.07 to 25.98) | 17.68 (10.61 to 26.56) |
| Niue | Female | Prevalence | 309.42 (141.89 to 864.79) | 655.50 (251.43 to 1987.54) | 870.33 (282.11 to 2805.19) | 1114.09 (316.82 to 3732.94) | 1323.19 (347.59 to 4529.08) | 1477.27 (370.82 to 5115.59) | 1586.00 (386.08 to 5528.35) | 1676.59 (398.54 to 5872.81) | 1749.51 (410.03 to 6150.38) | 1814.57 (418.88 to 6397.68) | 1875.93 (427.70 to 6631.91) | 1938.36 (437.01 to 6868.25) | 2008.61 (447.64 to 7136.13) | 2043.77 (451.87 to 7269.49) | 2043.83 (452.35 to 7268.85) | 2043.81 (453.07 to 7269.15) | 2043.83 (451.73 to 7269.54) | 2043.82 (452.58 to 7269.92) | 2043.81 (452.48 to 7269.99) | 2043.83 (452.07 to 7269.65) |
| Niue | Male | DALY rates | 8.33 (4.42 to 14.24) | 40.38 (17.03 to 85.67) | 43.01 (18.55 to 90.53) | 47.03 (20.06 to 102.28) | 50.14 (21.91 to 100.34) | 52.68 (23.68 to 108.69) | 54.54 (23.73 to 111.36) | 56.37 (24.18 to 108.98) | 57.26 (26.60 to 116.37) | 58.08 (26.68 to 119.12) | 58.86 (26.76 to 116.69) | 59.34 (25.83 to 116.93) | 59.93 (27.54 to 122.61) | 59.90 (28.19 to 114.82) | 58.77 (28.02 to 116.35) | 57.45 (27.74 to 110.96) | 56.87 (26.75 to 112.33) | 55.11 (26.37 to 106.95) | 54.35 (26.43 to 105.34) | 53.25 (25.40 to 101.50) |
| Niue | Male | Prevalence | 272.18 (105.89 to 817.79) | 809.87 (333.61 to 2064.67) | 1047.29 (388.02 to 2887.54) | 1319.28 (447.65 to 3843.29) | 1554.98 (503.09 to 4665.27) | 1730.09 (541.76 to 5270.08) | 1854.39 (570.12 to 5698.18) | 1958.44 (593.84 to 6055.25) | 2042.56 (613.57 to 6343.34) | 2117.80 (631.00 to 6599.98) | 2189.12 (647.48 to 6841.65) | 2261.76 (664.36 to 7088.80) | 2343.95 (684.18 to 7366.70) | 2385.16 (694.55 to 7506.28) | 2385.19 (694.80 to 7506.28) | 2385.11 (694.64 to 7506.60) | 2385.13 (693.90 to 7505.45) | 2385.17 (695.15 to 7505.69) | 2385.23 (695.03 to 7506.94) | 2385.22 (694.22 to 7507.54) |
| Palau | Both | DALY rates | 9.75 (5.94 to 14.75) | 15.92 (9.63 to 25.35) | 15.86 (9.91 to 24.79) | 15.81 (9.60 to 24.21) | 15.69 (9.13 to 24.32) | 15.16 (9.51 to 22.97) | 15.13 (9.19 to 23.12) | 14.82 (9.21 to 21.97) | 14.94 (9.36 to 23.14) | 14.78 (9.39 to 21.64) | 15.01 (9.46 to 21.72) | 14.86 (9.30 to 22.27) | 14.74 (9.01 to 21.70) | 14.67 (9.23 to 22.10) | 14.44 (9.16 to 21.36) | 14.01 (8.77 to 20.39) | 13.76 (8.33 to 20.33) | 13.38 (8.39 to 19.61) | 12.80 (8.25 to 18.93) | 12.06 (7.39 to 17.88) |
| Palau | Both | Prevalence | 98.67 (92.44 to 104.98) | 163.01 (152.36 to 174.68) | 167.10 (155.30 to 181.31) | 170.61 (157.50 to 188.38) | 171.18 (156.81 to 191.57) | 171.08 (155.79 to 194.90) | 172.92 (156.84 to 198.67) | 175.27 (158.56 to 202.33) | 177.73 (160.43 to 205.65) | 180.19 (162.38 to 209.06) | 182.50 (164.21 to 212.23) | 184.97 (166.33 to 215.78) | 186.82 (167.55 to 218.30) | 187.88 (168.31 to 220.27) | 187.10 (167.52 to 219.23) | 187.39 (167.83 to 218.96) | 187.12 (167.68 to 219.52) | 184.25 (164.55 to 217.26) | 180.78 (160.91 to 214.43) | 176.59 (156.90 to 209.49) |
| Palau | Female | DALY rates | 11.51 (6.65 to 18.06) | 18.66 (10.72 to 29.77) | 18.64 (10.80 to 28.74) | 18.36 (10.39 to 28.78) | 18.57 (10.02 to 29.00) | 18.40 (10.31 to 27.73) | 18.34 (10.71 to 29.26) | 17.96 (9.89 to 28.32) | 17.92 (10.20 to 28.84) | 17.50 (9.66 to 26.39) | 17.56 (10.03 to 26.63) | 17.37 (9.97 to 27.14) | 17.08 (10.16 to 26.49) | 17.05 (10.06 to 25.19) | 16.93 (9.55 to 26.09) | 16.19 (9.64 to 23.90) | 16.02 (9.37 to 25.15) | 15.69 (9.29 to 24.49) | 15.15 (9.03 to 22.74) | 14.60 (8.88 to 22.06) |
| Palau | Female | Prevalence | 115.76 (107.28 to 125.95) | 190.42 (176.24 to 207.76) | 194.01 (178.48 to 213.15) | 198.20 (181.77 to 219.23) | 201.73 (183.72 to 225.26) | 204.32 (185.17 to 228.99) | 206.21 (186.72 to 232.19) | 207.69 (187.49 to 234.86) | 208.91 (187.95 to 236.89) | 210.03 (188.80 to 239.14) | 211.09 (189.33 to 240.80) | 212.12 (190.14 to 243.25) | 213.32 (190.67 to 244.90) | 213.90 (191.22 to 246.55) | 213.94 (191.12 to 246.10) | 213.92 (191.02 to 245.99) | 213.91 (190.99 to 246.35) | 213.91 (190.97 to 245.89) | 213.91 (190.92 to 246.37) | 213.90 (191.36 to 246.98) |
| Palau | Male | DALY rates | 8.14 (4.45 to 12.88) | 13.36 (7.35 to 21.50) | 13.17 (7.16 to 22.35) | 13.46 (7.33 to 22.40) | 13.57 (6.56 to 23.34) | 13.24 (7.39 to 21.23) | 13.22 (7.21 to 21.90) | 12.83 (6.88 to 20.21) | 12.86 (6.80 to 21.13) | 12.67 (6.85 to 21.25) | 12.83 (7.12 to 20.12) | 12.47 (6.89 to 19.66) | 12.39 (6.67 to 20.32) | 12.20 (6.75 to 19.58) | 12.01 (6.81 to 18.76) | 11.83 (6.58 to 18.56) | 11.55 (6.44 to 18.59) | 11.56 (6.75 to 18.49) | 11.39 (6.43 to 18.35) | 10.99 (6.15 to 17.44) |
| Palau | Male | Prevalence | 83.03 (75.63 to 90.53) | 137.48 (124.50 to 151.20) | 141.07 (127.15 to 156.63) | 145.21 (130.61 to 165.18) | 148.74 (132.95 to 172.27) | 151.34 (134.51 to 177.07) | 153.19 (135.49 to 180.27) | 154.69 (136.23 to 182.80) | 155.89 (137.20 to 184.41) | 157.03 (137.85 to 186.55) | 158.02 (137.96 to 188.35) | 159.07 (138.49 to 190.31) | 160.27 (139.27 to 192.79) | 160.88 (139.32 to 193.34) | 160.86 (139.37 to 193.14) | 160.86 (139.20 to 193.82) | 160.88 (139.66 to 193.49) | 160.87 (139.15 to 193.74) | 160.87 (139.27 to 193.44) | 160.86 (139.99 to 193.78) |
| Papua New Guinea | Both | DALY rates | 10.54 (6.55 to 15.26) | 60.20 (32.92 to 105.17) | 88.54 (50.01 to 149.10) | 131.73 (77.55 to 213.97) | 172.94 (109.16 to 264.27) | 203.94 (129.93 to 310.11) | 223.22 (143.17 to 326.55) | 243.73 (159.42 to 360.28) | 259.91 (171.89 to 372.56) | 277.22 (181.49 to 397.72) | 296.74 (195.29 to 424.68) | 311.03 (205.63 to 445.53) | 327.72 (218.75 to 469.64) | 332.73 (222.11 to 472.82) | 323.91 (219.38 to 455.84) | 315.77 (214.25 to 443.50) | 307.75 (209.36 to 427.30) | 293.95 (201.82 to 407.06) | 270.95 (188.96 to 370.77) | 243.42 (171.02 to 331.05) |
| Papua New Guinea | Both | Prevalence | 1214.97 (294.53 to 3292.79) | 3360.10 (1019.24 to 8571.80) | 4889.29 (1488.45 to 12486.66) | 6741.41 (2113.15 to 17095.61) | 8361.32 (2698.24 to 21065.74) | 9540.66 (3132.86 to 23942.89) | 10373.69 (3435.64 to 26015.88) | 11084.50 (3697.87 to 27715.33) | 11690.92 (3919.14 to 29165.04) | 12238.69 (4121.73 to 30442.39) | 12795.01 (4363.26 to 31679.23) | 13333.81 (4565.12 to 32926.23) | 13946.86 (4804.39 to 34355.83) | 14248.96 (4927.67 to 35057.73) | 14236.36 (4924.12 to 35046.63) | 14215.81 (4902.92 to 35023.87) | 14189.05 (4875.70 to 34996.18) | 14100.00 (4786.15 to 34906.51) | 13929.42 (4611.01 to 34744.11) | 13707.37 (4392.14 to 34524.70) |
| Papua New Guinea | Female | DALY rates | 12.45 (7.58 to 18.38) | 23.45 (13.90 to 36.13) | 26.20 (14.62 to 41.51) | 29.54 (18.21 to 44.04) | 32.01 (19.26 to 48.20) | 34.45 (21.37 to 50.44) | 36.35 (23.10 to 53.22) | 37.21 (23.05 to 55.92) | 38.43 (23.44 to 57.86) | 39.80 (25.05 to 60.62) | 40.98 (26.49 to 61.02) | 41.28 (25.85 to 61.52) | 42.36 (26.83 to 61.89) | 42.57 (26.67 to 61.81) | 41.88 (26.87 to 61.01) | 41.41 (26.89 to 59.37) | 40.26 (25.69 to 57.50) | 39.32 (25.57 to 56.56) | 37.94 (25.59 to 52.87) | 36.74 (25.30 to 52.00) |
| Papua New Guinea | Female | Prevalence | 1233.46 (310.89 to 3313.60) | 3069.89 (710.94 to 8386.51) | 4393.11 (959.87 to 12133.76) | 5922.15 (1248.50 to 16458.11) | 7217.95 (1497.90 to 20116.07) | 8161.43 (1679.36 to 22775.87) | 8839.77 (1811.08 to 24685.98) | 9386.57 (1917.35 to 26225.29) | 9848.85 (2007.90 to 27523.80) | 10252.17 (2086.20 to 28655.32) | 10632.40 (2161.63 to 29724.37) | 11023.75 (2238.20 to 30820.89) | 11473.65 (2329.48 to 32083.83) | 11692.52 (2373.57 to 32696.66) | 11692.55 (2374.07 to 32696.95) | 11692.48 (2372.66 to 32697.73) | 11692.54 (2373.99 to 32695.97) | 11692.55 (2372.90 to 32695.63) | 11692.51 (2374.39 to 32698.00) | 11692.48 (2373.21 to 32695.97) |
| Papua New Guinea | Male | DALY rates | 8.80 (4.75 to 13.91) | 93.57 (48.07 to 174.70) | 144.21 (75.94 to 258.92) | 223.11 (125.62 to 375.75) | 302.60 (184.59 to 473.00) | 367.97 (230.80 to 565.19) | 413.98 (261.32 to 616.20) | 452.40 (294.54 to 672.71) | 480.42 (315.06 to 697.34) | 506.12 (327.15 to 733.10) | 526.92 (343.83 to 761.71) | 545.28 (359.84 to 785.52) | 566.68 (378.09 to 816.63) | 570.44 (381.06 to 812.42) | 557.47 (371.45 to 785.68) | 547.09 (374.26 to 774.18) | 538.58 (361.03 to 756.39) | 531.16 (362.11 to 746.52) | 522.41 (356.69 to 720.80) | 513.81 (356.55 to 701.45) |
| Papua New Guinea | Male | Prevalence | 1198.07 (279.49 to 3273.77) | 3623.53 (1218.56 to 8894.21) | 5332.45 (1852.37 to 13013.65) | 7474.03 (2738.31 to 17931.23) | 9413.27 (3698.66 to 22200.59) | 10875.46 (4420.21 to 25319.77) | 11939.53 (4957.92 to 27582.52) | 12800.15 (5359.45 to 29406.46) | 13524.96 (5716.75 to 30943.32) | 14153.87 (6024.02 to 32300.74) | 14741.31 (6345.52 to 33567.71) | 15339.87 (6646.16 to 34880.02) | 16017.91 (6967.83 to 36382.91) | 16343.25 (7114.08 to 37115.98) | 16342.96 (7110.50 to 37117.83) | 16343.32 (7112.74 to 37113.66) | 16343.35 (7110.24 to 37111.09) | 16342.76 (7136.55 to 37106.46) | 16343.48 (7128.41 to 37115.17) | 16343.33 (7120.52 to 37114.58) |
| Philippines | Both | DALY rates | 9.71 (6.50 to 14.24) | 28.40 (16.25 to 51.16) | 29.65 (16.77 to 53.32) | 30.66 (17.37 to 54.62) | 31.55 (17.92 to 56.19) | 32.25 (18.39 to 57.99) | 32.95 (18.81 to 59.05) | 33.38 (18.90 to 59.48) | 33.63 (19.19 to 59.73) | 33.97 (19.62 to 59.97) | 33.71 (19.46 to 60.32) | 33.62 (19.62 to 59.24) | 33.15 (19.50 to 57.67) | 32.07 (18.97 to 54.72) | 30.93 (18.56 to 52.80) | 29.55 (18.19 to 49.20) | 28.03 (17.46 to 45.69) | 27.81 (17.35 to 45.61) | 30.56 (18.74 to 51.54) | 39.17 (22.58 to 68.35) |
| Philippines | Both | Prevalence | 205.44 (142.96 to 374.35) | 524.83 (347.39 to 928.24) | 653.98 (415.60 to 1245.74) | 784.33 (473.60 to 1595.59) | 877.53 (511.08 to 1880.60) | 963.19 (554.10 to 2107.67) | 1023.69 (585.43 to 2266.65) | 1070.11 (604.23 to 2435.22) | 1108.53 (620.97 to 2559.46) | 1153.54 (651.60 to 2662.92) | 1170.18 (658.51 to 2774.59) | 1204.07 (679.30 to 2884.95) | 1228.23 (681.70 to 3003.12) | 1233.10 (681.98 to 3050.37) | 1249.87 (690.76 to 3176.94) | 1268.33 (710.54 to 3138.21) | 1255.33 (700.00 to 3082.48) | 1266.08 (701.53 to 3126.40) | 1374.57 (786.43 to 3285.41) | 1556.40 (900.97 to 3460.34) |
| Philippines | Female | DALY rates | 11.43 (7.69 to 17.02) | 19.16 (12.78 to 27.39) | 19.28 (12.82 to 27.86) | 19.24 (13.01 to 26.87) | 19.32 (13.17 to 27.11) | 19.32 (13.09 to 26.68) | 19.32 (12.85 to 27.25) | 19.31 (13.15 to 27.16) | 19.21 (13.26 to 27.11) | 19.21 (12.95 to 27.06) | 19.05 (13.04 to 27.17) | 18.95 (12.84 to 26.89) | 18.78 (12.90 to 26.23) | 18.47 (12.71 to 25.92) | 18.15 (12.60 to 24.89) | 17.81 (12.23 to 24.27) | 17.48 (12.22 to 23.99) | 17.12 (11.86 to 23.47) | 16.82 (11.86 to 23.16) | 16.61 (11.83 to 22.26) |
| Philippines | Female | Prevalence | 222.61 (160.28 to 389.86) | 456.62 (308.60 to 869.67) | 577.40 (364.21 to 1181.93) | 699.80 (415.27 to 1533.90) | 786.89 (441.78 to 1824.36) | 866.93 (470.65 to 2053.60) | 922.04 (490.92 to 2213.55) | 963.45 (505.58 to 2381.36) | 996.00 (516.13 to 2497.33) | 1036.64 (539.85 to 2604.41) | 1047.28 (542.79 to 2704.83) | 1074.80 (561.33 to 2826.10) | 1097.35 (569.79 to 2942.09) | 1103.26 (566.96 to 2997.87) | 1122.06 (577.10 to 3063.08) | 1143.47 (594.96 to 3029.99) | 1133.83 (583.06 to 3034.88) | 1133.30 (575.80 to 3058.90) | 1218.77 (639.17 to 3158.21) | 1352.17 (722.15 to 3105.80) |
| Philippines | Male | DALY rates | 8.11 (5.42 to 11.61) | 37.02 (18.15 to 76.63) | 39.40 (18.60 to 80.98) | 41.53 (20.07 to 84.02) | 43.33 (20.68 to 87.10) | 44.79 (21.92 to 90.91) | 45.86 (22.67 to 89.95) | 46.66 (23.23 to 92.60) | 47.26 (23.51 to 94.68) | 48.07 (24.13 to 95.68) | 48.08 (24.38 to 95.45) | 48.55 (24.56 to 95.91) | 48.57 (24.85 to 94.39) | 47.97 (24.35 to 92.39) | 47.74 (24.75 to 92.41) | 48.11 (24.89 to 90.91) | 47.70 (24.85 to 90.82) | 47.40 (25.04 to 89.65) | 47.95 (25.77 to 89.74) | 48.66 (26.02 to 88.59) |
| Philippines | Male | Prevalence | 189.56 (127.25 to 360.87) | 588.39 (363.76 to 1046.55) | 726.00 (437.66 to 1328.01) | 864.78 (515.54 to 1679.28) | 964.76 (556.26 to 1957.25) | 1056.46 (597.34 to 2187.87) | 1119.91 (630.69 to 2343.81) | 1170.72 (657.00 to 2506.27) | 1214.93 (678.82 to 2635.69) | 1265.24 (713.40 to 2750.25) | 1290.69 (722.89 to 2870.49) | 1335.62 (747.83 to 2981.88) | 1368.66 (767.49 to 3098.89) | 1384.95 (777.75 to 3140.30) | 1417.86 (801.98 to 3246.17) | 1465.87 (848.08 to 3257.97) | 1482.06 (864.18 to 3264.70) | 1509.44 (886.76 to 3351.51) | 1571.75 (908.91 to 3432.50) | 1642.26 (957.95 to 3656.37) |
| Samoa | Both | DALY rates | 10.67 (6.80 to 15.52) | 50.88 (25.87 to 88.30) | 68.45 (36.12 to 121.23) | 95.12 (54.06 to 161.91) | 123.35 (71.27 to 200.84) | 146.23 (88.76 to 228.70) | 162.83 (98.28 to 250.98) | 177.41 (111.39 to 269.91) | 194.58 (125.38 to 298.51) | 208.55 (131.48 to 306.85) | 217.50 (141.87 to 318.73) | 222.76 (145.37 to 320.58) | 227.76 (151.95 to 330.44) | 225.92 (149.08 to 324.43) | 215.12 (143.59 to 312.38) | 203.49 (136.72 to 297.27) | 191.46 (130.15 to 272.08) | 184.10 (123.96 to 264.30) | 173.40 (116.46 to 242.11) | 162.42 (109.96 to 226.53) |
| Samoa | Both | Prevalence | 1013.15 (176.80 to 3985.72) | 2715.31 (612.16 to 10249.01) | 3886.45 (841.58 to 14792.24) | 5295.17 (1163.06 to 20093.58) | 6541.83 (1475.62 to 24641.02) | 7482.88 (1726.02 to 28021.38) | 8148.96 (1909.93 to 30402.87) | 8706.14 (2071.35 to 32334.69) | 9214.35 (2252.58 to 34020.99) | 9662.27 (2406.84 to 35493.55) | 10046.54 (2522.44 to 36823.25) | 10419.32 (2603.69 to 38173.54) | 10842.18 (2707.65 to 39703.79) | 11026.86 (2738.43 to 40440.10) | 10962.24 (2678.39 to 40372.00) | 10894.96 (2601.45 to 40296.23) | 10813.11 (2521.69 to 40218.22) | 10777.64 (2480.07 to 40180.42) | 10722.55 (2428.82 to 40121.59) | 10638.03 (2335.72 to 40033.63) |
| Samoa | Female | DALY rates | 12.57 (7.68 to 19.51) | 22.68 (13.33 to 36.27) | 24.40 (14.15 to 36.76) | 26.47 (16.03 to 43.45) | 28.89 (17.62 to 44.17) | 30.09 (18.71 to 43.76) | 31.72 (20.04 to 48.54) | 32.40 (20.19 to 49.13) | 33.49 (19.89 to 50.08) | 33.64 (20.71 to 50.71) | 34.35 (21.32 to 51.82) | 34.51 (22.15 to 50.97) | 35.48 (22.38 to 51.92) | 34.98 (21.33 to 50.25) | 34.68 (22.31 to 51.38) | 34.05 (22.04 to 49.15) | 33.51 (21.67 to 48.46) | 32.48 (21.09 to 46.08) | 31.58 (19.79 to 44.26) | 31.27 (20.35 to 44.53) |
| Samoa | Female | Prevalence | 1033.68 (196.77 to 4010.16) | 2495.47 (403.21 to 9930.94) | 3540.08 (501.65 to 14331.39) | 4748.87 (617.82 to 19416.30) | 5778.66 (718.29 to 23740.87) | 6542.62 (793.98 to 26947.34) | 7082.60 (848.77 to 29209.07) | 7517.08 (892.79 to 31027.90) | 7886.23 (930.64 to 32574.75) | 8210.42 (962.04 to 33930.90) | 8508.79 (994.06 to 35177.61) | 8821.49 (1026.42 to 36484.42) | 9174.99 (1064.27 to 37961.25) | 9348.79 (1081.13 to 38686.90) | 9348.75 (1081.85 to 38686.66) | 9348.77 (1081.49 to 38686.85) | 9348.77 (1081.90 to 38687.31) | 9348.80 (1082.34 to 38687.39) | 9348.74 (1081.22 to 38686.57) | 9348.74 (1081.63 to 38688.14) |
| Samoa | Male | DALY rates | 8.91 (4.94 to 14.19) | 77.15 (35.16 to 142.41) | 109.94 (53.53 to 207.14) | 159.70 (85.49 to 278.15) | 211.91 (118.14 to 357.10) | 257.00 (151.66 to 410.31) | 291.28 (175.14 to 461.45) | 318.05 (194.30 to 500.54) | 342.22 (216.99 to 534.63) | 362.03 (227.25 to 534.40) | 379.11 (244.39 to 556.89) | 396.48 (255.45 to 578.24) | 413.18 (270.63 to 604.59) | 418.81 (274.38 to 609.82) | 411.95 (266.43 to 602.70) | 403.80 (266.97 to 593.88) | 397.39 (263.21 to 574.99) | 390.50 (259.10 to 565.99) | 379.89 (248.41 to 537.81) | 374.51 (247.20 to 532.79) |
| Samoa | Male | Prevalence | 994.15 (156.97 to 3963.08) | 2920.12 (737.69 to 10545.35) | 4212.66 (1068.65 to 15226.26) | 5809.05 (1562.96 to 20730.66) | 7257.26 (2077.07 to 25484.86) | 8379.71 (2532.37 to 29045.80) | 9193.76 (2859.44 to 31572.54) | 9859.40 (3138.51 to 33602.13) | 10431.58 (3387.36 to 35346.50) | 10936.21 (3607.37 to 36864.71) | 11403.37 (3818.59 to 38275.27) | 11893.79 (4024.02 to 39732.25) | 12449.95 (4254.80 to 41384.24) | 12722.02 (4387.30 to 42211.14) | 12722.40 (4394.55 to 42210.55) | 12722.75 (4386.06 to 42198.73) | 12722.32 (4388.17 to 42214.21) | 12722.79 (4385.84 to 42212.95) | 12722.79 (4394.57 to 42210.94) | 12723.03 (4387.55 to 42209.52) |
| Sao Tome and Principe | Both | DALY rates | 10.22 (6.39 to 15.17) | 34.82 (18.93 to 62.98) | 39.94 (21.29 to 69.76) | 45.98 (24.68 to 82.80) | 52.94 (27.91 to 93.00) | 58.30 (31.95 to 103.73) | 62.28 (33.07 to 109.07) | 66.24 (36.59 to 115.30) | 69.84 (37.88 to 120.94) | 72.55 (39.11 to 121.42) | 73.70 (40.17 to 124.59) | 75.05 (42.12 to 122.59) | 76.66 (41.33 to 129.81) | 76.83 (44.78 to 127.25) | 72.85 (41.56 to 121.10) | 67.16 (38.40 to 109.32) | 64.18 (37.65 to 103.46) | 62.64 (37.51 to 101.11) | 61.30 (35.89 to 99.78) | 54.99 (33.48 to 87.96) |
| Sao Tome and Principe | Both | Prevalence | 526.93 (106.42 to 2794.78) | 1348.11 (280.30 to 6797.19) | 1858.65 (325.91 to 9788.77) | 2447.90 (387.09 to 13209.33) | 2963.05 (448.59 to 16152.68) | 3348.42 (501.05 to 18325.56) | 3626.50 (542.65 to 19877.41) | 3852.49 (576.42 to 21120.35) | 4045.68 (606.31 to 22170.34) | 4214.36 (632.92 to 23090.69) | 4366.35 (655.91 to 23948.89) | 4522.66 (678.71 to 24847.52) | 4704.36 (704.63 to 25862.73) | 4788.64 (718.33 to 26354.15) | 4767.10 (704.21 to 26337.94) | 4729.78 (679.21 to 26311.59) | 4710.62 (663.49 to 26295.36) | 4707.03 (658.78 to 26294.02) | 4706.50 (658.86 to 26295.62) | 4662.55 (619.00 to 26263.57) |
| Sao Tome and Principe | Female | DALY rates | 11.91 (7.13 to 18.56) | 20.29 (11.88 to 32.17) | 21.02 (11.97 to 32.57) | 21.78 (12.41 to 33.96) | 22.72 (14.33 to 33.99) | 22.92 (14.01 to 33.39) | 23.22 (13.71 to 35.44) | 23.56 (14.13 to 34.59) | 23.91 (14.59 to 37.30) | 23.89 (14.46 to 37.14) | 23.76 (14.48 to 36.51) | 24.10 (14.34 to 36.99) | 23.84 (14.46 to 34.62) | 24.09 (14.54 to 35.90) | 23.58 (14.09 to 34.83) | 23.13 (13.85 to 35.34) | 22.78 (13.94 to 35.35) | 22.36 (14.13 to 32.91) | 21.97 (14.16 to 32.73) | 21.22 (13.00 to 31.89) |
| Sao Tome and Principe | Female | Prevalence | 545.49 (122.58 to 2819.68) | 1236.32 (213.60 to 6752.56) | 1713.94 (231.68 to 9728.60) | 2257.70 (249.83 to 13112.96) | 2724.90 (267.25 to 16019.99) | 3069.16 (279.82 to 18160.16) | 3314.59 (288.07 to 19685.48) | 3510.35 (295.29 to 20901.85) | 3675.55 (301.94 to 21929.10) | 3820.67 (306.91 to 22829.95) | 3956.33 (312.63 to 23672.89) | 4098.32 (317.39 to 24554.30) | 4258.55 (322.29 to 25549.27) | 4336.58 (325.80 to 26032.87) | 4336.57 (327.12 to 26033.22) | 4336.58 (326.32 to 26033.01) | 4336.57 (326.31 to 26033.17) | 4336.58 (326.08 to 26032.46) | 4336.54 (326.94 to 26033.17) | 4336.59 (326.25 to 26033.48) |
| Sao Tome and Principe | Male | DALY rates | 8.59 (4.69 to 13.98) | 49.07 (22.20 to 101.87) | 58.85 (26.59 to 115.20) | 70.47 (32.03 to 139.59) | 83.41 (38.47 to 159.94) | 93.68 (45.11 to 177.21) | 100.98 (49.19 to 185.55) | 107.62 (54.07 to 199.11) | 113.35 (54.86 to 209.54) | 118.28 (58.10 to 211.45) | 121.87 (60.96 to 219.48) | 126.26 (66.21 to 217.52) | 130.89 (66.99 to 231.69) | 132.59 (70.55 to 228.40) | 130.02 (69.83 to 226.49) | 127.28 (66.78 to 222.11) | 125.73 (65.69 to 218.74) | 123.50 (67.61 to 208.58) | 120.85 (65.03 to 204.68) | 117.54 (63.82 to 203.20) |
| Sao Tome and Principe | Male | Prevalence | 509.14 (88.77 to 2770.92) | 1457.82 (316.95 to 6884.46) | 2003.25 (385.23 to 9903.00) | 2640.37 (481.22 to 13360.55) | 3203.13 (578.60 to 16338.07) | 3627.67 (658.83 to 18539.34) | 3935.48 (717.43 to 20115.53) | 4184.12 (771.21 to 21378.62) | 4396.28 (816.75 to 22443.34) | 4584.41 (861.85 to 23378.52) | 4761.80 (900.75 to 24258.92) | 4949.11 (942.02 to 25182.16) | 5162.09 (995.04 to 26222.93) | 5266.55 (1019.95 to 26735.14) | 5266.68 (1019.36 to 26732.38) | 5266.62 (1018.31 to 26732.29) | 5266.65 (1018.90 to 26725.69) | 5266.62 (1021.27 to 26731.98) | 5266.62 (1016.69 to 26730.85) | 5266.47 (1020.38 to 26731.00) |
| Senegal | Both | DALY rates | 9.46 (5.95 to 14.24) | 27.21 (14.63 to 48.41) | 27.72 (14.76 to 50.07) | 28.38 (14.39 to 53.08) | 28.64 (15.24 to 51.96) | 28.40 (15.45 to 52.80) | 28.21 (15.28 to 51.58) | 28.41 (15.32 to 52.27) | 28.32 (15.26 to 50.48) | 28.28 (15.45 to 53.04) | 28.17 (15.28 to 50.98) | 28.05 (15.56 to 51.01) | 28.14 (15.81 to 49.69) | 27.73 (15.10 to 47.88) | 27.21 (15.05 to 48.75) | 26.41 (14.74 to 46.50) | 25.64 (14.86 to 43.82) | 24.92 (14.05 to 44.52) | 23.92 (13.73 to 40.91) | 22.61 (13.42 to 37.62) |
| Senegal | Both | Prevalence | 150.80 (96.19 to 384.45) | 393.75 (216.89 to 965.14) | 464.58 (230.25 to 1297.46) | 545.11 (243.43 to 1673.27) | 611.97 (253.95 to 1992.22) | 659.37 (260.25 to 2230.73) | 693.38 (263.47 to 2401.77) | 722.51 (267.57 to 2539.93) | 747.33 (270.80 to 2656.12) | 768.02 (272.76 to 2756.89) | 787.85 (275.50 to 2852.51) | 809.80 (279.04 to 2952.37) | 834.67 (283.34 to 3065.18) | 845.55 (284.68 to 3118.98) | 844.72 (284.38 to 3119.06) | 842.10 (282.95 to 3117.47) | 839.74 (280.95 to 3115.78) | 837.94 (279.72 to 3115.27) | 832.55 (277.41 to 3111.35) | 823.87 (272.76 to 3106.47) |
| Senegal | Female | DALY rates | 11.13 (6.64 to 18.05) | 19.03 (10.45 to 30.61) | 18.72 (11.01 to 29.52) | 18.84 (10.57 to 29.08) | 18.80 (10.79 to 28.85) | 18.57 (10.48 to 29.46) | 18.63 (10.75 to 28.58) | 18.60 (10.55 to 28.06) | 18.55 (10.91 to 29.55) | 18.27 (10.61 to 27.87) | 18.28 (11.06 to 28.16) | 18.20 (10.90 to 27.79) | 18.04 (9.62 to 28.39) | 17.70 (10.35 to 27.44) | 17.66 (10.71 to 27.80) | 17.18 (10.40 to 25.17) | 17.08 (10.10 to 25.91) | 16.64 (10.54 to 24.57) | 16.34 (9.51 to 24.85) | 16.09 (9.71 to 24.12) |
| Senegal | Female | Prevalence | 166.81 (111.52 to 402.14) | 329.86 (194.61 to 916.84) | 396.88 (200.33 to 1249.52) | 473.62 (207.10 to 1630.39) | 539.12 (212.10 to 1955.93) | 587.13 (215.74 to 2193.73) | 621.42 (218.24 to 2364.05) | 648.88 (220.99 to 2500.78) | 672.16 (222.95 to 2615.48) | 692.49 (224.39 to 2716.47) | 711.59 (226.36 to 2811.30) | 731.31 (227.78 to 2909.88) | 753.90 (230.23 to 3021.43) | 764.63 (231.02 to 3075.10) | 764.63 (231.16 to 3074.39) | 764.61 (230.83 to 3074.82) | 764.59 (230.87 to 3074.13) | 764.58 (231.43 to 3075.07) | 764.61 (231.18 to 3074.12) | 764.61 (230.68 to 3073.65) |
| Senegal | Male | DALY rates | 7.87 (4.26 to 12.88) | 34.91 (15.35 to 72.51) | 35.98 (15.90 to 74.22) | 37.10 (16.30 to 79.25) | 38.13 (16.51 to 80.97) | 38.69 (17.84 to 80.40) | 38.76 (17.30 to 82.30) | 39.12 (17.61 to 84.16) | 38.86 (17.60 to 79.53) | 39.25 (17.48 to 85.80) | 39.07 (17.12 to 84.08) | 38.58 (17.13 to 79.53) | 38.63 (17.73 to 76.51) | 38.26 (17.26 to 75.85) | 37.43 (16.31 to 76.99) | 36.95 (16.87 to 76.77) | 35.96 (16.24 to 71.24) | 35.36 (15.13 to 75.14) | 34.86 (15.61 to 69.95) | 34.35 (15.30 to 70.05) |
| Senegal | Male | Prevalence | 135.54 (79.68 to 367.59) | 453.78 (224.96 to 1017.00) | 526.76 (240.71 to 1336.65) | 610.54 (256.68 to 1727.94) | 682.22 (274.58 to 2053.48) | 734.93 (286.17 to 2294.44) | 772.61 (294.82 to 2466.20) | 802.80 (302.16 to 2602.82) | 828.46 (307.01 to 2719.84) | 850.84 (311.88 to 2820.54) | 871.92 (315.66 to 2917.26) | 893.73 (319.49 to 3015.61) | 918.63 (323.25 to 3129.52) | 930.51 (325.61 to 3182.40) | 930.48 (325.70 to 3183.08) | 930.52 (325.06 to 3182.51) | 930.46 (325.98 to 3182.33) | 930.46 (325.71 to 3182.00) | 930.49 (325.08 to 3182.73) | 930.44 (325.15 to 3183.08) |
| Sierra Leone | Both | DALY rates | 9.93 (6.04 to 14.52) | 39.12 (21.50 to 70.20) | 47.10 (25.78 to 82.75) | 58.24 (31.15 to 100.31) | 69.16 (36.26 to 118.09) | 78.70 (44.25 to 130.97) | 86.50 (47.46 to 137.90) | 93.44 (53.53 to 155.70) | 99.22 (57.35 to 163.81) | 107.63 (63.93 to 176.18) | 113.77 (68.68 to 185.77) | 117.69 (69.81 to 193.36) | 123.84 (72.38 to 201.40) | 123.08 (74.68 to 195.28) | 117.99 (72.04 to 184.23) | 113.14 (69.79 to 179.04) | 107.47 (66.05 to 168.88) | 104.49 (63.91 to 158.87) | 102.45 (62.82 to 160.90) | 93.77 (57.74 to 142.11) |
| Sierra Leone | Both | Prevalence | 674.07 (154.19 to 2173.01) | 1805.11 (451.11 to 5749.05) | 2541.43 (581.03 to 8213.16) | 3402.80 (742.38 to 11075.36) | 4141.76 (895.64 to 13501.99) | 4702.44 (1017.20 to 15316.16) | 5106.11 (1110.70 to 16605.49) | 5444.21 (1195.19 to 17659.10) | 5732.75 (1269.64 to 18549.22) | 6005.44 (1351.86 to 19355.02) | 6250.95 (1422.44 to 20098.57) | 6494.56 (1491.56 to 20848.96) | 6780.81 (1577.58 to 21717.48) | 6901.13 (1607.84 to 22115.19) | 6871.20 (1589.68 to 22075.49) | 6850.59 (1572.92 to 22054.72) | 6810.15 (1544.07 to 22008.31) | 6803.83 (1538.96 to 21998.21) | 6801.49 (1536.38 to 21996.33) | 6742.47 (1495.23 to 21920.52) |
| Sierra Leone | Female | DALY rates | 11.66 (6.79 to 18.59) | 20.95 (12.61 to 32.18) | 21.91 (13.47 to 33.93) | 23.40 (13.94 to 35.41) | 24.19 (14.31 to 35.97) | 25.12 (16.08 to 39.50) | 25.68 (16.01 to 37.97) | 26.04 (16.03 to 39.05) | 26.34 (16.63 to 39.97) | 27.17 (16.41 to 40.85) | 27.10 (16.25 to 40.03) | 27.12 (17.22 to 42.12) | 27.53 (16.67 to 41.36) | 27.22 (17.55 to 40.89) | 26.99 (16.82 to 39.53) | 26.61 (16.61 to 39.41) | 26.44 (15.91 to 38.45) | 25.69 (16.31 to 37.66) | 25.11 (15.97 to 36.82) | 24.43 (15.69 to 36.40) |
| Sierra Leone | Female | Prevalence | 692.70 (170.05 to 2194.49) | 1664.86 (343.57 to 5463.06) | 2344.64 (421.03 to 7883.20) | 3126.09 (510.38 to 10663.45) | 3783.24 (587.68 to 12999.66) | 4271.25 (644.40 to 14733.56) | 4616.20 (686.03 to 15958.96) | 4894.02 (718.24 to 16944.63) | 5130.30 (745.68 to 17783.23) | 5336.25 (770.67 to 18513.93) | 5530.17 (795.49 to 19202.67) | 5729.82 (818.46 to 19909.58) | 5959.40 (846.31 to 20724.35) | 6068.79 (859.16 to 21111.01) | 6068.78 (858.94 to 21111.01) | 6068.80 (858.08 to 21110.92) | 6068.75 (857.55 to 21111.68) | 6068.79 (859.35 to 21110.27) | 6068.79 (858.40 to 21110.76) | 6068.71 (857.86 to 21110.19) |
| Sierra Leone | Male | DALY rates | 8.25 (4.44 to 13.19) | 57.20 (26.36 to 115.96) | 72.56 (34.34 to 139.79) | 94.70 (46.64 to 177.80) | 117.43 (57.55 to 212.52) | 136.45 (69.93 to 235.68) | 151.68 (77.92 to 255.18) | 162.95 (89.01 to 283.68) | 172.66 (95.21 to 299.11) | 182.68 (99.73 to 307.63) | 192.38 (112.53 to 321.33) | 200.20 (114.67 to 334.20) | 210.99 (120.92 to 349.67) | 213.23 (125.18 to 350.22) | 210.17 (123.05 to 333.35) | 205.39 (121.23 to 332.64) | 202.97 (119.55 to 330.19) | 198.85 (115.73 to 314.23) | 195.61 (115.19 to 313.78) | 190.66 (112.97 to 299.17) |
| Sierra Leone | Male | Prevalence | 655.89 (138.67 to 2152.05) | 1944.67 (544.76 to 6101.16) | 2740.34 (726.33 to 8668.21) | 3692.43 (953.48 to 11632.41) | 4526.65 (1175.19 to 14135.68) | 5167.17 (1365.10 to 15968.48) | 5631.16 (1509.30 to 17323.45) | 6011.70 (1634.87 to 18421.76) | 6339.86 (1745.47 to 19346.39) | 6629.56 (1845.02 to 20162.93) | 6904.76 (1942.33 to 20935.30) | 7191.27 (2049.86 to 21729.06) | 7524.02 (2177.23 to 22641.60) | 7683.93 (2240.34 to 23085.81) | 7684.03 (2248.86 to 23080.43) | 7684.07 (2246.81 to 23091.14) | 7683.89 (2239.93 to 23097.04) | 7684.14 (2253.49 to 23094.46) | 7684.15 (2244.45 to 23097.11) | 7683.88 (2242.08 to 23090.73) |
| South Sudan | Both | DALY rates | 8.91 (5.43 to 13.10) | 28.95 (15.50 to 52.70) | 30.05 (16.33 to 54.72) | 31.42 (16.64 to 58.35) | 31.14 (17.40 to 56.00) | 31.69 (17.55 to 56.24) | 31.32 (17.30 to 54.48) | 32.00 (17.77 to 57.22) | 33.05 (17.08 to 58.17) | 34.14 (18.11 to 60.44) | 34.64 (18.02 to 63.58) | 35.34 (18.30 to 63.33) | 35.86 (19.47 to 66.54) | 35.36 (19.58 to 64.53) | 33.96 (17.79 to 62.92) | 33.80 (18.31 to 61.28) | 33.19 (18.09 to 58.65) | 32.66 (17.88 to 58.01) | 31.55 (17.58 to 55.14) | 29.29 (16.76 to 50.29) |
| South Sudan | Both | Prevalence | 215.90 (113.42 to 473.30) | 598.69 (307.56 to 1252.58) | 763.94 (349.72 to 1727.55) | 950.97 (388.72 to 2265.72) | 1102.86 (420.03 to 2716.75) | 1217.35 (446.83 to 3051.97) | 1299.77 (465.76 to 3300.32) | 1369.39 (484.26 to 3493.94) | 1430.23 (501.28 to 3659.79) | 1486.19 (518.28 to 3807.08) | 1538.21 (533.93 to 3944.99) | 1592.04 (549.84 to 4087.60) | 1651.21 (566.62 to 4250.48) | 1678.86 (573.71 to 4327.77) | 1672.54 (569.67 to 4322.78) | 1677.32 (573.05 to 4326.11) | 1676.51 (572.33 to 4325.81) | 1677.40 (572.84 to 4326.31) | 1673.78 (570.08 to 4323.17) | 1657.69 (559.15 to 4311.22) |
| South Sudan | Female | DALY rates | 10.51 (6.11 to 16.07) | 19.21 (11.01 to 30.41) | 19.13 (10.61 to 30.15) | 19.55 (11.42 to 29.88) | 19.14 (11.48 to 30.89) | 19.30 (11.48 to 29.69) | 19.16 (11.64 to 29.39) | 19.10 (10.97 to 29.39) | 19.64 (11.24 to 30.97) | 19.30 (11.31 to 30.14) | 19.14 (11.42 to 28.99) | 19.25 (11.54 to 29.69) | 18.98 (11.04 to 28.86) | 18.67 (10.88 to 28.04) | 18.24 (10.90 to 26.97) | 17.61 (10.14 to 26.29) | 17.39 (10.45 to 26.61) | 17.23 (10.50 to 25.96) | 17.08 (10.19 to 25.59) | 16.68 (10.42 to 25.07) |
| South Sudan | Female | Prevalence | 231.17 (129.09 to 485.88) | 524.92 (253.66 to 1200.59) | 681.74 (285.83 to 1668.12) | 859.72 (320.52 to 2198.25) | 1010.74 (350.85 to 2647.41) | 1122.39 (373.35 to 2979.79) | 1205.87 (390.62 to 3228.14) | 1268.99 (403.07 to 3416.68) | 1322.69 (413.95 to 3576.56) | 1369.78 (423.76 to 3716.51) | 1413.97 (432.29 to 3848.30) | 1459.59 (440.95 to 3983.91) | 1512.30 (451.96 to 4141.08) | 1537.74 (457.61 to 4216.31) | 1537.76 (457.28 to 4216.84) | 1537.73 (457.52 to 4216.22) | 1537.77 (457.71 to 4216.64) | 1537.75 (457.04 to 4215.93) | 1537.73 (457.88 to 4216.47) | 1537.75 (457.33 to 4216.09) |
| South Sudan | Male | DALY rates | 7.41 (3.84 to 12.07) | 37.88 (16.50 to 80.19) | 39.96 (18.63 to 81.47) | 42.41 (17.64 to 90.77) | 44.24 (19.00 to 92.69) | 46.18 (20.88 to 94.05) | 47.18 (21.22 to 94.67) | 47.93 (20.77 to 99.15) | 48.47 (20.90 to 94.07) | 49.60 (22.55 to 97.39) | 49.55 (22.34 to 98.85) | 49.61 (22.35 to 101.17) | 50.25 (23.01 to 102.82) | 49.52 (23.03 to 99.32) | 48.67 (21.07 to 100.01) | 47.87 (22.41 to 96.68) | 47.11 (22.20 to 89.77) | 46.05 (21.36 to 91.12) | 44.82 (21.08 to 83.97) | 44.12 (20.70 to 85.90) |
| South Sudan | Male | Prevalence | 201.62 (97.96 to 460.77) | 666.42 (340.42 to 1341.50) | 838.62 (396.24 to 1784.61) | 1035.37 (456.62 to 2328.13) | 1203.39 (508.84 to 2792.43) | 1328.36 (542.92 to 3136.36) | 1422.14 (570.97 to 3394.39) | 1493.35 (589.31 to 3589.32) | 1554.00 (605.47 to 3755.59) | 1607.40 (621.03 to 3901.40) | 1657.62 (634.44 to 4037.93) | 1709.47 (649.95 to 4179.52) | 1769.58 (665.14 to 4343.70) | 1798.65 (673.60 to 4422.39) | 1798.66 (674.60 to 4421.92) | 1798.64 (675.00 to 4421.63) | 1798.68 (674.30 to 4421.94) | 1798.65 (675.32 to 4422.13) | 1798.65 (674.91 to 4421.10) | 1798.65 (674.02 to 4423.02) |
| Sri Lanka | Both | DALY rates | 0.00 (0.00 to 0.00) | 0.00 (0.00 to 0.00) | 0.00 (0.00 to 0.00) | 0.00 (0.00 to 0.00) | 0.00 (0.00 to 0.00) | 0.00 (0.00 to 0.00) | 0.00 (0.00 to 0.00) | 0.00 (0.00 to 0.00) | 0.00 (0.00 to 0.00) | 0.00 (0.00 to 0.00) | 0.00 (0.00 to 0.00) | 0.00 (0.00 to 0.00) | 0.00 (0.00 to 0.00) | 0.00 (0.00 to 0.00) | 0.00 (0.00 to 0.00) | 0.00 (0.00 to 0.00) | 0.00 (0.00 to 0.00) | 0.00 (0.00 to 0.00) | 0.00 (0.00 to 0.00) | 0.00 (0.00 to 0.00) |
| Sri Lanka | Both | Prevalence | 22.42 (16.38 to 30.48) | 54.22 (39.61 to 73.72) | 78.77 (57.55 to 107.10) | 106.82 (78.04 to 145.25) | 130.67 (95.47 to 177.68) | 148.33 (108.37 to 201.69) | 160.83 (117.50 to 218.69) | 170.96 (124.90 to 232.45) | 179.30 (131.00 to 243.80) | 186.77 (136.46 to 253.96) | 193.74 (141.54 to 263.43) | 200.99 (146.84 to 273.30) | 209.19 (152.83 to 284.44) | 213.07 (155.67 to 289.72) | 213.07 (155.67 to 289.72) | 213.07 (155.67 to 289.72) | 213.07 (155.67 to 289.72) | 213.07 (155.67 to 289.72) | 213.07 (155.67 to 289.72) | 213.07 (155.67 to 289.72) |
| Sri Lanka | Female | DALY rates | 0.00 (0.00 to 0.00) | 0.00 (0.00 to 0.00) | 0.00 (0.00 to 0.00) | 0.00 (0.00 to 0.00) | 0.00 (0.00 to 0.00) | 0.00 (0.00 to 0.00) | 0.00 (0.00 to 0.00) | 0.00 (0.00 to 0.00) | 0.00 (0.00 to 0.00) | 0.00 (0.00 to 0.00) | 0.00 (0.00 to 0.00) | 0.00 (0.00 to 0.00) | 0.00 (0.00 to 0.00) | 0.00 (0.00 to 0.00) | 0.00 (0.00 to 0.00) | 0.00 (0.00 to 0.00) | 0.00 (0.00 to 0.00) | 0.00 (0.00 to 0.00) | 0.00 (0.00 to 0.00) | 0.00 (0.00 to 0.00) |
| Sri Lanka | Female | Prevalence | 22.45 (16.40 to 30.52) | 54.22 (39.61 to 73.72) | 78.77 (57.55 to 107.10) | 106.82 (78.04 to 145.25) | 130.67 (95.47 to 177.68) | 148.33 (108.37 to 201.69) | 160.83 (117.50 to 218.69) | 170.96 (124.90 to 232.45) | 179.30 (131.00 to 243.80) | 186.77 (136.46 to 253.96) | 193.74 (141.54 to 263.43) | 200.99 (146.84 to 273.30) | 209.19 (152.83 to 284.44) | 213.07 (155.67 to 289.72) | 213.07 (155.67 to 289.72) | 213.07 (155.67 to 289.72) | 213.07 (155.67 to 289.72) | 213.07 (155.67 to 289.72) | 213.07 (155.67 to 289.72) | 213.07 (155.67 to 289.72) |
| Sri Lanka | Male | DALY rates | 0.00 (0.00 to 0.00) | 0.00 (0.00 to 0.00) | 0.00 (0.00 to 0.00) | 0.00 (0.00 to 0.00) | 0.00 (0.00 to 0.00) | 0.00 (0.00 to 0.00) | 0.00 (0.00 to 0.00) | 0.00 (0.00 to 0.00) | 0.00 (0.00 to 0.00) | 0.00 (0.00 to 0.00) | 0.00 (0.00 to 0.00) | 0.00 (0.00 to 0.00) | 0.00 (0.00 to 0.00) | 0.00 (0.00 to 0.00) | 0.00 (0.00 to 0.00) | 0.00 (0.00 to 0.00) | 0.00 (0.00 to 0.00) | 0.00 (0.00 to 0.00) | 0.00 (0.00 to 0.00) | 0.00 (0.00 to 0.00) |
| Sri Lanka | Male | Prevalence | 22.39 (16.36 to 30.44) | 54.22 (39.61 to 73.72) | 78.77 (57.55 to 107.10) | 106.82 (78.04 to 145.25) | 130.67 (95.47 to 177.68) | 148.33 (108.37 to 201.69) | 160.83 (117.50 to 218.69) | 170.96 (124.90 to 232.45) | 179.30 (131.00 to 243.80) | 186.77 (136.46 to 253.96) | 193.74 (141.54 to 263.43) | 200.99 (146.84 to 273.30) | 209.19 (152.83 to 284.44) | 213.07 (155.67 to 289.72) | 213.07 (155.67 to 289.72) | 213.07 (155.67 to 289.72) | 213.07 (155.67 to 289.72) | 213.07 (155.67 to 289.72) | 213.07 (155.67 to 289.72) | 213.07 (155.67 to 289.72) |
| Sudan | Both | DALY rates | 9.69 (5.88 to 14.95) | 28.59 (15.12 to 53.64) | 29.30 (14.93 to 54.00) | 30.29 (16.14 to 55.70) | 31.28 (16.64 to 59.83) | 31.56 (17.11 to 56.15) | 31.76 (17.59 to 56.50) | 31.09 (17.40 to 55.88) | 31.78 (17.76 to 54.09) | 32.48 (17.36 to 58.53) | 32.95 (17.23 to 60.77) | 33.73 (17.86 to 60.94) | 34.11 (18.00 to 63.55) | 33.89 (17.39 to 62.81) | 34.01 (17.67 to 62.58) | 32.80 (17.69 to 57.99) | 32.08 (17.40 to 58.02) | 31.27 (17.43 to 54.78) | 30.33 (16.38 to 56.21) | 28.72 (15.98 to 51.25) |
| Sudan | Both | Prevalence | 208.90 (98.60 to 629.87) | 537.34 (222.98 to 1600.50) | 673.20 (235.51 to 2210.99) | 828.18 (256.31 to 2908.23) | 961.66 (273.99 to 3508.70) | 1056.63 (283.97 to 3941.42) | 1122.76 (288.72 to 4247.69) | 1174.82 (291.42 to 4492.39) | 1223.32 (295.92 to 4706.40) | 1271.06 (304.67 to 4900.84) | 1315.08 (311.80 to 5082.04) | 1359.39 (318.50 to 5265.46) | 1409.26 (324.39 to 5478.13) | 1430.95 (326.74 to 5575.72) | 1437.49 (331.50 to 5583.51) | 1432.17 (327.29 to 5576.52) | 1432.33 (328.10 to 5577.60) | 1431.22 (327.33 to 5575.14) | 1427.56 (324.47 to 5570.77) | 1416.35 (315.65 to 5555.80) |
| Sudan | Female | DALY rates | 11.36 (6.49 to 17.50) | 19.14 (11.17 to 29.57) | 19.19 (10.73 to 29.36) | 19.20 (11.05 to 31.62) | 19.43 (12.05 to 31.02) | 19.32 (10.79 to 30.48) | 19.46 (11.73 to 30.91) | 19.18 (11.38 to 30.56) | 19.29 (11.24 to 29.44) | 18.81 (11.21 to 29.47) | 18.94 (11.49 to 29.06) | 19.05 (11.32 to 30.26) | 18.69 (11.20 to 29.36) | 18.69 (11.39 to 27.34) | 18.33 (11.12 to 27.66) | 17.94 (10.76 to 26.94) | 17.89 (10.64 to 27.02) | 17.26 (10.16 to 25.92) | 16.92 (10.25 to 25.86) | 16.57 (9.77 to 24.50) |
| Sudan | Female | Prevalence | 225.41 (114.89 to 645.81) | 467.06 (195.91 to 1503.69) | 596.27 (204.58 to 2105.06) | 743.80 (216.16 to 2792.41) | 870.80 (223.54 to 3383.44) | 962.87 (229.97 to 3812.98) | 1029.05 (233.56 to 4120.48) | 1081.93 (237.55 to 4366.60) | 1126.59 (240.30 to 4575.38) | 1165.98 (242.75 to 4758.15) | 1202.61 (244.98 to 4928.97) | 1240.32 (247.47 to 5104.74) | 1284.30 (250.15 to 5309.17) | 1305.05 (251.35 to 5405.75) | 1305.06 (251.50 to 5405.17) | 1305.04 (251.27 to 5405.74) | 1305.06 (251.20 to 5406.28) | 1305.07 (251.57 to 5405.75) | 1305.06 (251.25 to 5406.44) | 1305.03 (251.45 to 5405.65) |
| Sudan | Male | DALY rates | 8.12 (4.38 to 13.26) | 37.46 (16.48 to 80.46) | 38.71 (16.07 to 83.52) | 40.69 (18.22 to 85.50) | 42.53 (18.61 to 92.11) | 43.80 (18.80 to 91.37) | 45.07 (19.61 to 91.01) | 45.01 (20.58 to 94.53) | 46.01 (21.01 to 90.60) | 46.35 (19.84 to 92.17) | 45.90 (20.47 to 95.22) | 46.31 (20.88 to 95.85) | 46.69 (21.43 to 97.48) | 46.42 (20.64 to 93.74) | 45.51 (20.32 to 92.44) | 44.78 (20.68 to 87.31) | 43.49 (19.69 to 88.04) | 42.75 (19.44 to 82.32) | 42.05 (18.57 to 86.63) | 41.63 (19.55 to 85.53) |
| Sudan | Male | Prevalence | 193.44 (83.59 to 614.94) | 603.27 (232.31 to 1738.45) | 744.84 (254.89 to 2309.65) | 907.27 (281.72 to 3016.79) | 1047.93 (297.31 to 3627.63) | 1150.34 (311.19 to 4069.78) | 1224.20 (319.19 to 4385.37) | 1283.43 (329.42 to 4639.46) | 1333.53 (336.92 to 4855.68) | 1377.74 (344.02 to 5045.70) | 1418.91 (349.20 to 5223.37) | 1461.50 (355.58 to 5403.28) | 1511.25 (362.60 to 5616.02) | 1534.70 (367.89 to 5715.80) | 1534.67 (366.67 to 5714.38) | 1534.65 (366.74 to 5714.19) | 1534.61 (367.59 to 5715.29) | 1534.65 (366.00 to 5714.04) | 1534.63 (366.26 to 5714.40) | 1534.62 (367.33 to 5715.33) |
| Thailand | Both | DALY rates | 0.00 (0.00 to 0.00) | 0.00 (0.00 to 0.00) | 0.00 (0.00 to 0.00) | 0.00 (0.00 to 0.00) | 0.00 (0.00 to 0.00) | 0.00 (0.00 to 0.00) | 0.00 (0.00 to 0.00) | 0.00 (0.00 to 0.00) | 0.00 (0.00 to 0.00) | 0.00 (0.00 to 0.00) | 0.00 (0.00 to 0.00) | 0.00 (0.00 to 0.00) | 0.00 (0.00 to 0.00) | 0.00 (0.00 to 0.00) | 0.00 (0.00 to 0.00) | 0.00 (0.00 to 0.00) | 0.00 (0.00 to 0.00) | 0.00 (0.00 to 0.00) | 0.00 (0.00 to 0.00) | 0.00 (0.00 to 0.00) |
| Thailand | Both | Prevalence | 73.46 (3.93 to 366.72) | 176.38 (9.43 to 880.56) | 256.54 (13.72 to 1280.71) | 348.43 (18.63 to 1739.47) | 426.75 (22.82 to 2130.41) | 482.72 (25.81 to 2409.86) | 523.59 (28.00 to 2613.88) | 556.49 (29.76 to 2778.12) | 583.93 (31.23 to 2915.11) | 608.05 (32.51 to 3035.52) | 630.68 (33.73 to 3148.50) | 654.08 (34.98 to 3265.35) | 680.88 (36.41 to 3399.10) | 693.54 (37.09 to 3462.30) | 693.54 (37.09 to 3462.30) | 693.54 (37.09 to 3462.30) | 693.54 (37.09 to 3462.30) | 693.54 (37.09 to 3462.30) | 693.54 (37.09 to 3462.30) | 693.54 (37.09 to 3462.30) |
| Thailand | Female | DALY rates | 0.00 (0.00 to 0.00) | 0.00 (0.00 to 0.00) | 0.00 (0.00 to 0.00) | 0.00 (0.00 to 0.00) | 0.00 (0.00 to 0.00) | 0.00 (0.00 to 0.00) | 0.00 (0.00 to 0.00) | 0.00 (0.00 to 0.00) | 0.00 (0.00 to 0.00) | 0.00 (0.00 to 0.00) | 0.00 (0.00 to 0.00) | 0.00 (0.00 to 0.00) | 0.00 (0.00 to 0.00) | 0.00 (0.00 to 0.00) | 0.00 (0.00 to 0.00) | 0.00 (0.00 to 0.00) | 0.00 (0.00 to 0.00) | 0.00 (0.00 to 0.00) | 0.00 (0.00 to 0.00) | 0.00 (0.00 to 0.00) |
| Thailand | Female | Prevalence | 73.46 (3.93 to 366.74) | 176.38 (9.43 to 880.56) | 256.54 (13.72 to 1280.71) | 348.43 (18.63 to 1739.47) | 426.75 (22.82 to 2130.41) | 482.72 (25.81 to 2409.86) | 523.59 (28.00 to 2613.88) | 556.49 (29.76 to 2778.12) | 583.93 (31.23 to 2915.11) | 608.05 (32.51 to 3035.52) | 630.68 (33.73 to 3148.50) | 654.08 (34.98 to 3265.35) | 680.88 (36.41 to 3399.10) | 693.54 (37.09 to 3462.30) | 693.54 (37.09 to 3462.30) | 693.54 (37.09 to 3462.30) | 693.54 (37.09 to 3462.30) | 693.54 (37.09 to 3462.30) | 693.54 (37.09 to 3462.30) | 693.54 (37.09 to 3462.30) |
| Thailand | Male | DALY rates | 0.00 (0.00 to 0.00) | 0.00 (0.00 to 0.00) | 0.00 (0.00 to 0.00) | 0.00 (0.00 to 0.00) | 0.00 (0.00 to 0.00) | 0.00 (0.00 to 0.00) | 0.00 (0.00 to 0.00) | 0.00 (0.00 to 0.00) | 0.00 (0.00 to 0.00) | 0.00 (0.00 to 0.00) | 0.00 (0.00 to 0.00) | 0.00 (0.00 to 0.00) | 0.00 (0.00 to 0.00) | 0.00 (0.00 to 0.00) | 0.00 (0.00 to 0.00) | 0.00 (0.00 to 0.00) | 0.00 (0.00 to 0.00) | 0.00 (0.00 to 0.00) | 0.00 (0.00 to 0.00) | 0.00 (0.00 to 0.00) |
| Thailand | Male | Prevalence | 73.45 (3.93 to 366.70) | 176.38 (9.43 to 880.56) | 256.54 (13.72 to 1280.71) | 348.43 (18.63 to 1739.47) | 426.75 (22.82 to 2130.41) | 482.72 (25.81 to 2409.86) | 523.59 (28.00 to 2613.88) | 556.49 (29.76 to 2778.12) | 583.93 (31.23 to 2915.11) | 608.05 (32.51 to 3035.52) | 630.68 (33.73 to 3148.50) | 654.08 (34.98 to 3265.35) | 680.88 (36.41 to 3399.10) | 693.54 (37.09 to 3462.30) | 693.54 (37.09 to 3462.30) | 693.54 (37.09 to 3462.30) | 693.54 (37.09 to 3462.30) | 693.54 (37.09 to 3462.30) | 693.54 (37.09 to 3462.30) | 693.54 (37.09 to 3462.30) |
| Timor-Leste | Both | DALY rates | 11.22 (7.05 to 16.49) | 71.13 (37.77 to 121.08) | 108.63 (59.96 to 181.81) | 164.91 (99.41 to 255.90) | 216.97 (139.17 to 321.66) | 251.63 (166.30 to 367.06) | 278.55 (186.68 to 404.48) | 302.03 (201.71 to 429.88) | 324.13 (213.76 to 465.27) | 347.29 (230.67 to 492.51) | 365.91 (244.53 to 514.57) | 374.04 (253.02 to 516.88) | 364.68 (247.30 to 508.59) | 347.83 (234.77 to 486.44) | 334.79 (230.59 to 463.63) | 336.30 (227.98 to 459.25) | 349.80 (239.80 to 477.49) | 321.22 (223.88 to 439.28) | 316.78 (220.86 to 429.77) | 302.13 (213.18 to 401.91) |
| Timor-Leste | Both | Prevalence | 1442.97 (125.56 to 7337.31) | 3992.71 (571.89 to 18968.43) | 5856.73 (874.28 to 27685.27) | 8086.78 (1344.97 to 37680.81) | 10007.12 (1793.17 to 46167.42) | 11408.04 (2127.76 to 52416.93) | 12433.58 (2388.97 to 56913.76) | 13287.13 (2607.34 to 60576.74) | 14034.73 (2843.23 to 63714.78) | 14692.06 (3072.57 to 66431.49) | 15280.62 (3257.56 to 68950.33) | 15824.09 (3398.07 to 71548.18) | 16254.84 (3354.28 to 74357.92) | 16368.44 (3258.48 to 75538.63) | 16321.00 (3213.33 to 75483.48) | 16397.74 (3290.12 to 75548.37) | 16584.40 (3467.70 to 75732.30) | 16365.33 (3262.11 to 75528.01) | 16387.77 (3280.72 to 75550.35) | 16313.94 (3214.76 to 75478.27) |
| Timor-Leste | Female | DALY rates | 13.08 (7.91 to 19.75) | 24.93 (15.43 to 38.35) | 28.03 (17.82 to 44.92) | 31.43 (19.01 to 48.18) | 35.93 (21.90 to 54.68) | 39.12 (24.01 to 60.63) | 41.94 (25.53 to 63.65) | 44.12 (28.43 to 65.22) | 45.18 (29.03 to 66.33) | 47.03 (29.34 to 71.04) | 47.60 (30.73 to 69.64) | 48.87 (32.29 to 70.65) | 50.85 (32.20 to 76.21) | 50.58 (32.56 to 75.14) | 49.51 (31.80 to 70.07) | 47.84 (31.62 to 69.44) | 47.31 (30.41 to 70.18) | 46.63 (31.17 to 65.27) | 45.23 (29.74 to 64.41) | 44.19 (29.53 to 62.56) |
| Timor-Leste | Female | Prevalence | 1462.75 (143.69 to 7357.05) | 3630.80 (283.01 to 18608.28) | 5213.93 (336.47 to 27044.60) | 7017.56 (403.22 to 36633.61) | 8545.47 (462.76 to 44736.94) | 9679.96 (512.12 to 50738.41) | 10494.92 (548.81 to 55040.87) | 11153.40 (579.73 to 58514.10) | 11713.33 (608.23 to 61463.89) | 12176.91 (629.87 to 63990.69) | 12598.08 (651.75 to 66343.83) | 13042.01 (675.74 to 68848.34) | 13551.51 (702.98 to 71731.79) | 13775.77 (715.58 to 73016.29) | 13775.91 (715.85 to 73014.50) | 13775.81 (716.00 to 73014.08) | 13775.92 (715.51 to 73016.31) | 13775.86 (716.27 to 73013.12) | 13775.86 (714.80 to 73013.68) | 13775.92 (715.65 to 73015.44) |
| Timor-Leste | Male | DALY rates | 9.47 (5.16 to 15.09) | 114.35 (56.15 to 206.60) | 184.17 (96.67 to 316.02) | 292.03 (171.49 to 460.32) | 398.68 (250.15 to 594.51) | 479.07 (313.63 to 705.69) | 534.05 (353.20 to 778.38) | 573.75 (381.58 to 818.43) | 602.86 (393.88 to 874.84) | 625.32 (413.92 to 887.43) | 643.59 (428.53 to 910.95) | 656.36 (442.80 to 913.45) | 675.48 (455.86 to 947.77) | 675.81 (455.58 to 949.07) | 660.86 (446.74 to 913.00) | 647.79 (434.23 to 890.36) | 634.64 (434.76 to 871.26) | 625.01 (432.80 to 860.99) | 612.15 (426.12 to 830.81) | 598.35 (422.41 to 794.07) |
| Timor-Leste | Male | Prevalence | 1424.36 (108.79 to 7318.74) | 4331.26 (813.68 to 19305.33) | 6459.13 (1348.14 to 28285.67) | 9104.98 (2196.34 to 38678.04) | 11474.29 (3029.39 to 47603.29) | 13257.47 (3756.20 to 54213.32) | 14526.91 (4268.19 to 58936.08) | 15535.06 (4636.89 to 62749.78) | 16354.36 (4977.11 to 65963.94) | 17021.07 (5210.56 to 68691.66) | 17620.75 (5403.46 to 71224.13) | 18239.59 (5613.38 to 73892.29) | 18932.04 (5852.21 to 76958.67) | 19229.13 (5925.46 to 78321.70) | 19230.03 (5944.49 to 78305.52) | 19228.95 (5926.88 to 78284.95) | 19228.92 (5931.80 to 78289.75) | 19230.11 (5942.23 to 78310.28) | 19228.77 (5932.85 to 78309.53) | 19228.49 (5936.67 to 78306.45) |
| Togo | Both | DALY rates | 0.00 (0.00 to 0.00) | 0.00 (0.00 to 0.00) | 0.00 (0.00 to 0.00) | 0.00 (0.00 to 0.00) | 0.00 (0.00 to 0.00) | 0.00 (0.00 to 0.00) | 0.00 (0.00 to 0.00) | 0.00 (0.00 to 0.00) | 0.00 (0.00 to 0.00) | 0.00 (0.00 to 0.00) | 0.00 (0.00 to 0.00) | 0.00 (0.00 to 0.00) | 0.00 (0.00 to 0.00) | 0.00 (0.00 to 0.00) | 0.00 (0.00 to 0.00) | 0.00 (0.00 to 0.00) | 0.00 (0.00 to 0.00) | 0.00 (0.00 to 0.00) | 0.00 (0.00 to 0.00) | 0.00 (0.00 to 0.00) |
| Togo | Both | Prevalence | 32.34 (3.86 to 137.30) | 79.70 (9.51 to 338.38) | 115.84 (13.82 to 491.82) | 157.45 (18.78 to 668.49) | 193.01 (23.02 to 819.43) | 219.01 (26.12 to 929.84) | 237.50 (28.33 to 1008.33) | 252.33 (30.10 to 1071.30) | 264.89 (31.60 to 1124.61) | 275.83 (32.90 to 1171.08) | 286.12 (34.13 to 1214.73) | 296.76 (35.40 to 1259.93) | 308.91 (36.85 to 1311.49) | 314.83 (37.55 to 1336.66) | 314.83 (37.55 to 1336.66) | 314.83 (37.55 to 1336.66) | 314.83 (37.55 to 1336.66) | 314.83 (37.55 to 1336.66) | 314.83 (37.55 to 1336.66) | 314.83 (37.55 to 1336.66) |
| Togo | Female | DALY rates | 0.00 (0.00 to 0.00) | 0.00 (0.00 to 0.00) | 0.00 (0.00 to 0.00) | 0.00 (0.00 to 0.00) | 0.00 (0.00 to 0.00) | 0.00 (0.00 to 0.00) | 0.00 (0.00 to 0.00) | 0.00 (0.00 to 0.00) | 0.00 (0.00 to 0.00) | 0.00 (0.00 to 0.00) | 0.00 (0.00 to 0.00) | 0.00 (0.00 to 0.00) | 0.00 (0.00 to 0.00) | 0.00 (0.00 to 0.00) | 0.00 (0.00 to 0.00) | 0.00 (0.00 to 0.00) | 0.00 (0.00 to 0.00) | 0.00 (0.00 to 0.00) | 0.00 (0.00 to 0.00) | 0.00 (0.00 to 0.00) |
| Togo | Female | Prevalence | 32.33 (3.86 to 137.27) | 79.70 (9.51 to 338.38) | 115.84 (13.82 to 491.82) | 157.45 (18.78 to 668.49) | 193.01 (23.02 to 819.43) | 219.01 (26.12 to 929.84) | 237.50 (28.33 to 1008.33) | 252.33 (30.10 to 1071.30) | 264.89 (31.60 to 1124.61) | 275.83 (32.90 to 1171.08) | 286.12 (34.13 to 1214.73) | 296.76 (35.40 to 1259.93) | 308.91 (36.85 to 1311.49) | 314.83 (37.55 to 1336.66) | 314.83 (37.55 to 1336.66) | 314.83 (37.55 to 1336.66) | 314.83 (37.55 to 1336.66) | 314.83 (37.55 to 1336.66) | 314.83 (37.55 to 1336.66) | 314.83 (37.55 to 1336.66) |
| Togo | Male | DALY rates | 0.00 (0.00 to 0.00) | 0.00 (0.00 to 0.00) | 0.00 (0.00 to 0.00) | 0.00 (0.00 to 0.00) | 0.00 (0.00 to 0.00) | 0.00 (0.00 to 0.00) | 0.00 (0.00 to 0.00) | 0.00 (0.00 to 0.00) | 0.00 (0.00 to 0.00) | 0.00 (0.00 to 0.00) | 0.00 (0.00 to 0.00) | 0.00 (0.00 to 0.00) | 0.00 (0.00 to 0.00) | 0.00 (0.00 to 0.00) | 0.00 (0.00 to 0.00) | 0.00 (0.00 to 0.00) | 0.00 (0.00 to 0.00) | 0.00 (0.00 to 0.00) | 0.00 (0.00 to 0.00) | 0.00 (0.00 to 0.00) |
| Togo | Male | Prevalence | 32.35 (3.86 to 137.33) | 79.70 (9.51 to 338.38) | 115.84 (13.82 to 491.82) | 157.45 (18.78 to 668.49) | 193.01 (23.02 to 819.43) | 219.01 (26.12 to 929.84) | 237.50 (28.33 to 1008.33) | 252.33 (30.10 to 1071.30) | 264.89 (31.60 to 1124.61) | 275.83 (32.90 to 1171.08) | 286.12 (34.13 to 1214.73) | 296.76 (35.40 to 1259.93) | 308.91 (36.85 to 1311.49) | 314.83 (37.55 to 1336.66) | 314.83 (37.55 to 1336.66) | 314.83 (37.55 to 1336.66) | 314.83 (37.55 to 1336.66) | 314.83 (37.55 to 1336.66) | 314.83 (37.55 to 1336.66) | 314.83 (37.55 to 1336.66) |
| Tonga | Both | DALY rates | 9.53 (5.86 to 14.45) | 27.32 (14.70 to 51.26) | 27.67 (14.69 to 49.16) | 27.83 (15.04 to 51.51) | 27.62 (14.92 to 49.76) | 27.12 (14.84 to 48.76) | 27.11 (14.40 to 48.21) | 26.87 (14.80 to 48.33) | 27.25 (14.95 to 51.30) | 27.05 (14.56 to 48.05) | 27.25 (15.41 to 50.80) | 27.21 (15.01 to 49.47) | 26.62 (14.50 to 48.35) | 26.13 (14.14 to 46.28) | 25.38 (14.35 to 45.49) | 24.48 (13.78 to 41.41) | 23.90 (13.00 to 43.05) | 22.67 (13.05 to 36.85) | 21.29 (12.54 to 34.25) | 19.23 (11.83 to 29.58) |
| Tonga | Both | Prevalence | 126.88 (88.35 to 348.37) | 332.53 (190.50 to 862.80) | 374.24 (193.62 to 1140.42) | 421.27 (197.60 to 1474.79) | 460.29 (199.74 to 1759.06) | 487.52 (201.49 to 1968.20) | 506.41 (202.03 to 2117.45) | 523.15 (203.16 to 2237.90) | 539.04 (203.73 to 2339.31) | 553.96 (205.29 to 2429.47) | 567.73 (206.12 to 2512.73) | 580.40 (206.64 to 2598.13) | 593.18 (206.97 to 2696.48) | 597.39 (206.69 to 2742.05) | 595.40 (206.19 to 2741.53) | 593.16 (205.74 to 2740.57) | 589.63 (204.98 to 2738.80) | 584.34 (204.37 to 2737.03) | 575.73 (203.23 to 2732.79) | 562.56 (199.02 to 2727.67) |
| Tonga | Female | DALY rates | 11.31 (6.40 to 17.21) | 19.11 (10.52 to 29.46) | 19.22 (10.79 to 29.95) | 19.03 (11.12 to 30.79) | 18.80 (11.24 to 29.14) | 18.53 (10.69 to 29.71) | 18.75 (10.34 to 29.28) | 18.51 (10.71 to 29.49) | 18.63 (10.85 to 30.43) | 18.23 (10.79 to 28.52) | 17.89 (10.46 to 27.49) | 18.18 (10.93 to 28.08) | 17.73 (10.30 to 28.04) | 17.51 (10.05 to 27.45) | 17.38 (10.25 to 27.23) | 16.92 (10.33 to 25.50) | 16.87 (9.95 to 25.73) | 16.30 (9.29 to 24.47) | 15.91 (9.50 to 23.77) | 15.51 (9.49 to 23.21) |
| Tonga | Female | Prevalence | 143.49 (103.03 to 366.62) | 270.20 (174.62 to 824.75) | 310.25 (175.95 to 1116.91) | 355.94 (177.62 to 1449.56) | 394.82 (178.43 to 1734.03) | 423.49 (178.86 to 1942.93) | 444.03 (179.42 to 2092.67) | 460.48 (180.10 to 2212.36) | 474.25 (180.66 to 2312.76) | 486.41 (181.06 to 2401.75) | 497.66 (181.08 to 2483.82) | 509.33 (181.56 to 2568.23) | 522.79 (182.24 to 2667.13) | 529.19 (181.94 to 2713.24) | 529.22 (182.11 to 2713.52) | 529.20 (182.04 to 2713.41) | 529.18 (182.18 to 2713.46) | 529.20 (182.22 to 2713.57) | 529.18 (181.58 to 2713.32) | 529.19 (182.06 to 2713.60) |
| Tonga | Male | DALY rates | 7.90 (4.17 to 13.23) | 34.79 (14.91 to 75.42) | 35.42 (16.07 to 73.69) | 36.12 (15.99 to 72.93) | 36.38 (16.09 to 76.77) | 36.39 (16.22 to 75.89) | 36.86 (15.94 to 77.82) | 36.73 (16.67 to 75.68) | 37.00 (16.55 to 78.10) | 36.40 (15.55 to 75.63) | 36.64 (16.42 to 78.11) | 36.15 (16.63 to 74.47) | 35.78 (16.41 to 74.10) | 35.65 (16.36 to 74.71) | 34.72 (16.38 to 74.65) | 33.88 (15.49 to 71.57) | 33.55 (15.51 to 71.85) | 32.88 (15.16 to 65.67) | 32.49 (14.72 to 68.26) | 31.50 (14.50 to 65.10) |
| Tonga | Male | Prevalence | 111.72 (73.63 to 332.08) | 389.25 (193.45 to 966.96) | 432.89 (197.09 to 1223.76) | 482.73 (200.80 to 1528.78) | 525.26 (205.95 to 1789.85) | 556.63 (209.32 to 1995.47) | 579.14 (211.72 to 2146.34) | 597.17 (214.76 to 2268.05) | 612.31 (216.56 to 2369.33) | 625.61 (218.63 to 2458.87) | 638.05 (219.96 to 2541.74) | 650.82 (221.94 to 2627.76) | 665.63 (223.71 to 2726.69) | 672.70 (224.20 to 2773.87) | 672.71 (224.26 to 2774.25) | 672.71 (223.62 to 2774.34) | 672.67 (224.73 to 2773.60) | 672.74 (223.79 to 2774.65) | 672.71 (224.55 to 2773.34) | 672.71 (224.21 to 2774.13) |
| Uganda | Both | DALY rates | 9.49 (5.80 to 14.10) | 15.91 (9.70 to 24.56) | 26.77 (14.44 to 47.76) | 26.59 (14.03 to 49.91) | 26.12 (14.44 to 45.93) | 25.94 (14.51 to 47.35) | 25.92 (14.12 to 48.69) | 25.56 (13.89 to 45.62) | 25.75 (14.28 to 44.81) | 25.66 (14.38 to 44.49) | 25.37 (13.14 to 45.93) | 24.88 (13.73 to 43.80) | 24.94 (13.99 to 43.53) | 24.16 (13.90 to 41.93) | 23.37 (12.93 to 39.90) | 22.64 (12.71 to 38.89) | 21.71 (12.20 to 35.53) | 20.87 (12.42 to 33.26) | 19.20 (11.25 to 29.46) | 17.80 (11.12 to 27.88) |
| Uganda | Both | Prevalence | 107.42 (92.02 to 153.05) | 195.46 (159.70 to 310.05) | 300.61 (207.84 to 530.13) | 321.42 (213.85 to 605.40) | 337.95 (219.20 to 657.46) | 350.04 (222.43 to 694.43) | 359.35 (224.83 to 736.99) | 367.20 (227.39 to 767.29) | 373.99 (228.84 to 791.26) | 380.32 (230.15 to 813.63) | 386.04 (230.79 to 834.47) | 390.31 (231.91 to 851.49) | 395.11 (233.03 to 871.81) | 396.62 (233.52 to 881.86) | 393.26 (233.30 to 876.09) | 390.53 (233.03 to 872.47) | 387.39 (233.26 to 867.89) | 381.89 (231.64 to 860.60) | 372.20 (228.17 to 841.79) | 363.63 (224.79 to 819.30) |
| Uganda | Female | DALY rates | 11.11 (6.32 to 17.44) | 18.81 (10.45 to 30.14) | 18.73 (10.95 to 29.85) | 18.51 (10.94 to 29.68) | 18.31 (10.78 to 27.99) | 18.28 (10.57 to 28.58) | 18.28 (11.27 to 29.44) | 18.10 (11.11 to 28.22) | 18.15 (10.71 to 27.67) | 17.92 (10.43 to 29.15) | 17.69 (10.38 to 27.51) | 17.62 (10.30 to 25.66) | 17.91 (10.70 to 27.45) | 17.25 (9.68 to 26.76) | 17.14 (9.96 to 26.16) | 16.56 (9.59 to 25.17) | 16.31 (9.80 to 24.81) | 16.32 (9.95 to 24.79) | 15.68 (9.47 to 23.75) | 15.38 (9.37 to 22.44) |
| Uganda | Female | Prevalence | 123.27 (106.18 to 168.17) | 222.66 (184.09 to 334.73) | 241.02 (188.60 to 404.58) | 262.00 (193.18 to 486.01) | 279.92 (196.70 to 554.09) | 293.06 (198.72 to 604.37) | 302.45 (200.50 to 638.85) | 309.98 (202.33 to 667.79) | 316.35 (203.54 to 691.48) | 321.93 (204.56 to 712.91) | 327.07 (206.11 to 731.50) | 332.52 (207.41 to 753.10) | 338.67 (208.36 to 775.69) | 341.63 (209.20 to 787.01) | 341.66 (208.80 to 787.73) | 341.65 (209.19 to 787.15) | 341.65 (208.59 to 787.19) | 341.65 (208.73 to 787.55) | 341.63 (208.85 to 787.88) | 341.64 (208.65 to 787.26) |
| Uganda | Male | DALY rates | 7.93 (4.29 to 13.02) | 13.15 (7.48 to 21.93) | 34.42 (15.10 to 70.75) | 34.56 (14.52 to 77.00) | 34.40 (16.06 to 69.33) | 34.51 (15.93 to 71.42) | 34.58 (15.67 to 77.20) | 34.04 (14.01 to 71.56) | 34.33 (14.89 to 70.62) | 34.24 (14.82 to 70.80) | 33.79 (14.33 to 71.78) | 33.22 (14.74 to 72.29) | 33.44 (15.51 to 68.52) | 32.96 (15.18 to 68.48) | 32.23 (14.12 to 65.70) | 32.10 (14.42 to 69.71) | 31.05 (13.89 to 63.27) | 30.45 (13.81 to 63.84) | 30.05 (13.64 to 61.61) | 29.14 (13.29 to 61.57) |
| Uganda | Male | Prevalence | 92.20 (76.33 to 137.22) | 169.60 (133.03 to 283.25) | 357.28 (213.49 to 668.02) | 380.03 (221.79 to 735.97) | 399.43 (228.29 to 799.95) | 413.78 (232.94 to 854.51) | 423.91 (237.16 to 894.30) | 432.12 (239.87 to 919.03) | 439.02 (241.88 to 939.55) | 445.08 (244.15 to 968.30) | 450.75 (245.70 to 967.63) | 456.62 (248.11 to 1012.46) | 463.31 (249.36 to 1008.98) | 466.55 (250.67 to 1046.54) | 466.58 (250.97 to 1045.26) | 466.58 (250.54 to 1021.30) | 466.57 (251.11 to 1043.88) | 466.56 (250.65 to 1045.74) | 466.50 (250.50 to 1045.12) | 466.56 (250.73 to 1044.00) |
| United Republic of Tanzania | Both | DALY rates | 9.49 (5.83 to 14.61) | 27.16 (14.24 to 49.73) | 27.34 (14.78 to 49.75) | 27.98 (14.75 to 50.38) | 27.81 (14.92 to 50.06) | 27.79 (15.67 to 50.45) | 28.06 (15.32 to 48.53) | 28.25 (15.52 to 50.14) | 28.13 (15.53 to 48.81) | 28.39 (15.40 to 51.19) | 28.42 (15.20 to 51.51) | 28.62 (15.69 to 52.50) | 28.61 (15.35 to 52.46) | 28.35 (15.46 to 51.04) | 27.40 (15.01 to 48.16) | 26.25 (14.47 to 46.03) | 25.14 (14.36 to 43.79) | 24.22 (13.77 to 41.68) | 22.94 (13.47 to 38.10) | 21.36 (13.08 to 34.28) |
| United Republic of Tanzania | Both | Prevalence | 151.24 (120.73 to 211.63) | 392.65 (277.39 to 607.71) | 462.49 (313.89 to 730.22) | 542.03 (355.59 to 880.51) | 608.27 (387.40 to 1010.81) | 657.16 (413.43 to 1104.06) | 694.83 (432.72 to 1179.52) | 725.07 (448.27 to 1239.91) | 748.86 (460.07 to 1287.83) | 770.59 (470.30 to 1331.17) | 792.28 (480.68 to 1373.27) | 814.13 (491.62 to 1415.34) | 838.97 (503.65 to 1465.16) | 851.26 (510.97 to 1489.97) | 847.87 (508.06 to 1485.80) | 844.11 (506.44 to 1481.54) | 839.38 (504.32 to 1476.53) | 834.82 (502.04 to 1472.85) | 827.87 (497.69 to 1468.56) | 817.31 (490.31 to 1452.67) |
| United Republic of Tanzania | Female | DALY rates | 11.15 (6.43 to 17.35) | 18.99 (11.29 to 29.77) | 18.76 (11.27 to 29.29) | 18.99 (10.27 to 29.79) | 18.75 (11.47 to 29.38) | 18.68 (10.77 to 28.50) | 18.64 (10.76 to 29.79) | 18.54 (11.01 to 28.56) | 18.31 (10.52 to 28.55) | 18.60 (11.35 to 28.64) | 18.26 (10.55 to 28.41) | 18.40 (10.89 to 27.50) | 18.24 (10.53 to 28.00) | 18.06 (10.70 to 29.41) | 17.80 (10.53 to 28.69) | 17.25 (9.90 to 25.92) | 17.10 (10.33 to 25.91) | 16.52 (9.98 to 24.78) | 16.27 (9.60 to 25.63) | 15.87 (9.69 to 23.22) |
| United Republic of Tanzania | Female | Prevalence | 167.30 (134.68 to 228.29) | 330.47 (250.13 to 484.34) | 397.77 (283.52 to 618.92) | 475.27 (320.59 to 773.92) | 541.19 (350.35 to 905.50) | 589.49 (372.46 to 1002.63) | 624.06 (388.79 to 1070.94) | 651.75 (402.05 to 1126.72) | 675.10 (412.76 to 1173.44) | 695.51 (421.88 to 1214.00) | 714.63 (431.16 to 1252.15) | 734.46 (440.34 to 1293.08) | 757.13 (451.57 to 1338.53) | 768.06 (455.90 to 1359.39) | 768.07 (456.11 to 1360.26) | 768.09 (456.58 to 1360.25) | 768.07 (456.26 to 1360.33) | 768.09 (455.86 to 1360.07) | 768.07 (456.06 to 1360.18) | 768.10 (455.77 to 1360.31) |
| United Republic of Tanzania | Male | DALY rates | 7.86 (4.39 to 12.82) | 35.26 (14.99 to 77.37) | 35.96 (15.62 to 74.75) | 37.41 (15.90 to 82.34) | 38.07 (16.11 to 79.32) | 38.57 (16.93 to 80.99) | 38.75 (16.84 to 79.84) | 38.91 (15.91 to 80.40) | 39.11 (17.04 to 77.90) | 39.23 (17.09 to 79.11) | 39.22 (17.99 to 79.78) | 39.21 (17.62 to 82.31) | 39.11 (17.43 to 81.14) | 38.57 (17.02 to 77.82) | 37.74 (16.92 to 77.07) | 36.87 (16.67 to 76.26) | 35.79 (16.18 to 73.22) | 35.64 (15.66 to 73.50) | 34.77 (15.15 to 71.07) | 34.37 (16.47 to 70.99) |
| United Republic of Tanzania | Male | Prevalence | 135.46 (102.70 to 193.72) | 454.33 (291.73 to 784.79) | 527.58 (335.54 to 894.62) | 612.16 (376.11 to 1034.59) | 684.26 (408.60 to 1177.91) | 737.19 (438.02 to 1269.06) | 775.14 (459.51 to 1330.61) | 805.59 (475.86 to 1385.72) | 831.32 (490.09 to 1431.58) | 853.73 (501.64 to 1470.23) | 874.85 (512.08 to 1508.23) | 896.69 (523.26 to 1546.47) | 921.81 (535.33 to 1597.22) | 933.85 (542.01 to 1620.94) | 933.87 (542.18 to 1620.72) | 933.82 (542.01 to 1620.73) | 933.84 (542.41 to 1619.55) | 933.86 (540.97 to 1620.13) | 933.83 (541.77 to 1620.09) | 933.82 (542.02 to 1621.21) |
| Vanuatu | Both | DALY rates | 9.94 (6.10 to 15.13) | 34.34 (18.12 to 64.21) | 38.91 (21.17 to 68.19) | 44.32 (23.66 to 77.52) | 49.26 (26.50 to 85.67) | 53.07 (28.88 to 94.96) | 55.79 (29.79 to 97.34) | 58.41 (31.40 to 99.83) | 61.49 (33.92 to 108.74) | 62.95 (33.37 to 108.21) | 63.62 (33.19 to 106.50) | 64.64 (35.00 to 109.22) | 65.32 (35.61 to 111.03) | 65.48 (35.26 to 109.49) | 64.17 (37.31 to 108.22) | 60.66 (34.92 to 104.70) | 59.89 (34.40 to 101.38) | 57.89 (33.91 to 95.98) | 54.33 (32.52 to 88.39) | 49.36 (29.15 to 78.92) |
| Vanuatu | Both | Prevalence | 470.33 (95.93 to 3356.96) | 1230.08 (232.93 to 8405.88) | 1687.74 (258.20 to 12116.34) | 2218.80 (293.66 to 16392.08) | 2676.96 (326.82 to 20077.89) | 3011.32 (355.34 to 22729.88) | 3241.29 (376.56 to 24627.39) | 3424.85 (395.32 to 26164.39) | 3579.86 (415.43 to 27474.65) | 3699.62 (429.73 to 28609.36) | 3803.10 (439.99 to 29663.64) | 3906.25 (451.22 to 30766.19) | 4020.53 (466.96 to 32023.56) | 4074.36 (473.01 to 32615.61) | 4075.16 (473.75 to 32616.31) | 4050.30 (459.09 to 32586.49) | 4056.80 (463.00 to 32597.53) | 4044.54 (455.21 to 32579.34) | 4022.89 (443.33 to 32554.08) | 3986.79 (421.34 to 32511.37) |
| Vanuatu | Female | DALY rates | 11.78 (6.73 to 19.12) | 20.33 (11.95 to 31.66) | 20.89 (11.66 to 33.88) | 21.49 (13.15 to 34.36) | 22.34 (13.84 to 34.22) | 22.67 (13.66 to 34.77) | 22.61 (14.23 to 33.84) | 22.81 (13.60 to 34.26) | 23.09 (13.71 to 36.68) | 22.77 (13.66 to 34.13) | 22.81 (13.55 to 34.44) | 22.99 (13.74 to 34.37) | 22.96 (14.16 to 35.39) | 22.67 (14.25 to 33.73) | 22.43 (13.78 to 34.26) | 21.87 (13.42 to 31.84) | 21.69 (12.53 to 32.19) | 21.37 (13.10 to 32.38) | 21.00 (13.41 to 30.55) | 20.30 (12.87 to 30.69) |
| Vanuatu | Female | Prevalence | 487.33 (110.06 to 3373.85) | 1122.01 (190.57 to 8279.15) | 1548.86 (197.84 to 11952.66) | 2040.46 (206.72 to 16179.69) | 2464.77 (215.31 to 19826.66) | 2769.48 (221.06 to 22444.19) | 2978.19 (225.24 to 24316.40) | 3140.78 (227.81 to 25827.72) | 3270.53 (230.90 to 27106.28) | 3374.60 (233.03 to 28221.40) | 3470.32 (235.05 to 29267.74) | 3563.27 (236.96 to 30355.29) | 3665.26 (238.75 to 31597.15) | 3711.19 (239.66 to 32180.40) | 3711.15 (239.30 to 32180.50) | 3711.15 (239.79 to 32180.36) | 3711.15 (240.11 to 32180.85) | 3711.17 (239.78 to 32180.49) | 3711.17 (239.73 to 32180.40) | 3711.18 (239.44 to 32180.03) |
| Vanuatu | Male | DALY rates | 8.22 (4.62 to 12.56) | 47.37 (19.88 to 100.48) | 55.52 (26.10 to 108.38) | 65.95 (29.74 to 126.29) | 76.79 (35.42 to 147.14) | 84.95 (42.03 to 165.19) | 91.03 (42.26 to 170.71) | 95.69 (48.00 to 176.68) | 99.21 (49.37 to 187.39) | 101.98 (50.28 to 186.18) | 104.39 (50.73 to 184.80) | 106.98 (51.91 to 198.68) | 108.69 (54.61 to 192.87) | 109.00 (56.71 to 191.32) | 106.42 (55.91 to 189.09) | 105.64 (54.54 to 190.25) | 102.63 (52.31 to 183.87) | 101.60 (53.41 to 182.11) | 99.31 (53.00 to 179.56) | 97.51 (52.17 to 168.33) |
| Vanuatu | Male | Prevalence | 454.44 (78.61 to 3341.19) | 1330.57 (260.14 to 8523.72) | 1815.86 (302.01 to 12267.34) | 2387.78 (361.43 to 16593.33) | 2893.89 (422.88 to 20334.75) | 3265.02 (476.11 to 23029.59) | 3520.70 (516.24 to 24957.66) | 3722.31 (545.76 to 26516.91) | 3883.71 (572.73 to 27836.48) | 4015.29 (596.58 to 28986.17) | 4135.47 (617.72 to 30059.07) | 4254.86 (639.26 to 31183.83) | 4384.36 (667.73 to 32460.25) | 4443.52 (679.98 to 33057.99) | 4443.59 (678.91 to 33057.42) | 4443.57 (680.12 to 33057.42) | 4443.48 (679.93 to 33063.68) | 4443.49 (675.63 to 33056.66) | 4443.52 (681.06 to 33058.31) | 4443.47 (678.60 to 33060.39) |
| Viet Nam | Both | DALY rates | 9.87 (6.07 to 15.13) | 28.21 (14.80 to 52.65) | 28.90 (15.14 to 52.96) | 29.34 (15.23 to 50.85) | 30.07 (16.28 to 54.97) | 30.82 (16.13 to 58.73) | 31.19 (16.53 to 57.14) | 30.92 (16.44 to 57.32) | 31.18 (17.21 to 56.02) | 31.12 (16.50 to 55.32) | 30.66 (16.30 to 54.53) | 30.28 (16.78 to 54.45) | 29.85 (16.72 to 55.34) | 28.63 (16.27 to 50.72) | 28.08 (15.57 to 49.12) | 26.73 (15.61 to 44.99) | 24.83 (14.93 to 41.49) | 23.32 (13.55 to 37.17) | 21.49 (13.11 to 34.33) | 18.90 (11.52 to 29.44) |
| Viet Nam | Both | Prevalence | 182.18 (99.91 to 554.57) | 459.80 (223.88 to 1347.89) | 557.06 (236.65 to 1841.56) | 671.93 (255.17 to 2425.16) | 770.50 (267.93 to 2924.15) | 842.43 (278.60 to 3287.42) | 891.88 (286.04 to 3537.05) | 932.41 (291.30 to 3742.21) | 966.35 (296.48 to 3916.10) | 996.63 (301.50 to 4069.63) | 1023.33 (304.75 to 4210.45) | 1049.34 (306.83 to 4356.39) | 1079.69 (310.04 to 4521.44) | 1091.31 (307.69 to 4597.84) | 1088.78 (304.91 to 4595.51) | 1082.21 (299.87 to 4588.88) | 1071.13 (295.94 to 4577.55) | 1062.12 (289.85 to 4570.79) | 1050.45 (279.48 to 4564.05) | 1032.85 (263.69 to 4553.73) |
| Viet Nam | Female | DALY rates | 11.57 (6.78 to 17.36) | 19.13 (11.20 to 29.92) | 19.35 (11.39 to 29.72) | 19.06 (11.27 to 29.22) | 19.43 (11.24 to 30.26) | 19.58 (11.19 to 32.06) | 19.23 (11.56 to 30.55) | 19.19 (11.41 to 30.23) | 19.47 (11.81 to 30.30) | 19.01 (11.06 to 29.36) | 19.04 (10.62 to 29.12) | 18.91 (11.23 to 29.98) | 18.60 (10.92 to 29.69) | 18.33 (10.73 to 27.77) | 18.30 (11.13 to 28.70) | 17.82 (10.36 to 26.89) | 17.33 (9.90 to 26.58) | 16.94 (9.86 to 25.61) | 16.47 (9.72 to 24.82) | 15.95 (9.58 to 23.90) |
| Viet Nam | Female | Prevalence | 199.07 (116.05 to 570.28) | 391.97 (195.22 to 1286.68) | 485.02 (203.55 to 1778.73) | 594.86 (211.83 to 2359.56) | 688.90 (218.51 to 2857.97) | 757.37 (223.25 to 3220.64) | 804.26 (227.02 to 3467.52) | 843.09 (229.13 to 3674.02) | 875.80 (231.78 to 3847.28) | 904.84 (233.70 to 4000.28) | 931.62 (235.74 to 4142.44) | 959.68 (236.95 to 4291.31) | 991.34 (239.40 to 4458.14) | 1006.58 (240.06 to 4538.22) | 1006.59 (240.08 to 4539.47) | 1006.62 (240.86 to 4538.67) | 1006.63 (240.01 to 4538.11) | 1006.59 (240.05 to 4538.44) | 1006.61 (240.25 to 4538.57) | 1006.62 (240.01 to 4538.55) |
| Viet Nam | Male | DALY rates | 8.29 (4.33 to 13.61) | 36.50 (16.16 to 81.95) | 37.72 (16.38 to 80.48) | 38.98 (16.06 to 81.29) | 40.16 (17.84 to 85.20) | 41.55 (17.73 to 85.81) | 42.63 (18.96 to 88.94) | 42.27 (18.16 to 88.67) | 42.67 (18.93 to 90.90) | 43.13 (19.50 to 88.91) | 42.60 (18.55 to 87.04) | 42.90 (19.21 to 87.70) | 43.17 (19.46 to 93.09) | 42.01 (19.45 to 83.94) | 41.48 (18.67 to 86.05) | 40.79 (18.98 to 83.21) | 39.99 (18.73 to 78.93) | 39.31 (18.44 to 83.41) | 38.79 (17.83 to 79.81) | 37.85 (15.98 to 78.21) |
| Viet Nam | Male | Prevalence | 166.59 (83.55 to 540.07) | 521.70 (232.97 to 1412.12) | 623.62 (252.90 to 1909.50) | 744.23 (275.98 to 2498.11) | 847.91 (292.87 to 3001.58) | 923.67 (305.53 to 3368.16) | 975.71 (315.54 to 3621.01) | 1018.90 (323.00 to 3827.76) | 1055.27 (328.45 to 4004.48) | 1087.62 (333.78 to 4159.75) | 1117.54 (338.89 to 4303.77) | 1148.88 (343.16 to 4455.08) | 1184.34 (348.73 to 4624.91) | 1201.40 (349.61 to 4707.62) | 1201.38 (348.97 to 4706.84) | 1201.37 (348.93 to 4706.89) | 1201.39 (349.68 to 4706.71) | 1201.37 (348.71 to 4707.41) | 1201.41 (350.54 to 4706.66) | 1201.37 (349.05 to 4706.48) |
| Yemen | Both | DALY rates | 9.33 (5.92 to 14.22) | 26.77 (13.99 to 49.72) | 26.79 (14.51 to 48.19) | 27.05 (14.36 to 48.37) | 26.78 (14.37 to 48.15) | 26.72 (14.20 to 50.39) | 26.79 (14.26 to 48.98) | 26.86 (14.19 to 48.65) | 26.85 (14.05 to 48.05) | 26.78 (13.51 to 49.02) | 26.69 (14.56 to 47.39) | 26.19 (14.21 to 47.92) | 25.84 (14.61 to 46.35) | 25.54 (13.71 to 46.44) | 25.27 (14.12 to 45.20) | 24.96 (13.82 to 44.10) | 24.33 (13.51 to 42.87) | 23.60 (13.37 to 41.78) | 22.52 (13.26 to 38.58) | 20.63 (12.63 to 33.81) |
| Yemen | Both | Prevalence | 121.66 (93.36 to 245.90) | 313.54 (200.14 to 634.19) | 347.24 (206.65 to 807.86) | 385.20 (213.21 to 994.28) | 417.09 (218.01 to 1160.67) | 440.74 (220.80 to 1287.53) | 457.63 (223.58 to 1376.03) | 471.86 (225.35 to 1446.80) | 484.43 (227.17 to 1508.32) | 494.74 (228.16 to 1562.16) | 503.22 (228.87 to 1612.69) | 512.02 (230.09 to 1664.86) | 523.39 (231.52 to 1724.99) | 528.12 (232.36 to 1753.00) | 530.98 (232.53 to 1754.60) | 529.57 (232.26 to 1754.30) | 528.94 (232.46 to 1753.40) | 526.07 (231.66 to 1751.62) | 520.80 (230.76 to 1749.05) | 507.82 (225.38 to 1741.30) |
| Yemen | Female | DALY rates | 11.01 (6.53 to 17.48) | 18.71 (11.08 to 29.90) | 18.56 (11.00 to 30.45) | 18.56 (11.10 to 28.98) | 18.05 (10.43 to 28.29) | 18.08 (10.98 to 28.51) | 18.05 (10.41 to 28.06) | 17.95 (10.57 to 28.30) | 17.79 (10.73 to 27.46) | 17.71 (9.42 to 26.59) | 17.63 (10.65 to 27.33) | 17.60 (11.00 to 27.63) | 17.44 (10.35 to 26.52) | 17.40 (10.17 to 27.12) | 17.06 (10.47 to 26.04) | 16.94 (10.47 to 25.48) | 16.67 (10.29 to 24.44) | 16.43 (9.80 to 24.46) | 15.89 (9.77 to 24.14) | 15.55 (9.81 to 23.57) |
| Yemen | Female | Prevalence | 138.00 (107.52 to 263.97) | 253.35 (182.68 to 561.06) | 285.53 (185.06 to 729.87) | 322.40 (188.57 to 927.12) | 354.02 (191.63 to 1095.78) | 377.36 (193.43 to 1221.02) | 393.80 (194.51 to 1308.99) | 406.93 (196.05 to 1379.37) | 418.04 (196.28 to 1439.20) | 427.76 (197.50 to 1491.82) | 437.01 (197.18 to 1541.00) | 446.54 (198.63 to 1592.13) | 457.43 (199.17 to 1650.14) | 462.60 (199.06 to 1678.08) | 462.60 (199.57 to 1678.12) | 462.59 (199.90 to 1678.56) | 462.61 (199.64 to 1677.53) | 462.59 (199.72 to 1678.21) | 462.58 (199.64 to 1678.34) | 462.56 (199.33 to 1678.43) |
| Yemen | Male | DALY rates | 7.74 (4.32 to 12.64) | 34.41 (14.79 to 68.67) | 34.56 (14.59 to 72.07) | 35.23 (15.21 to 74.46) | 35.50 (15.35 to 73.96) | 35.55 (15.38 to 78.27) | 35.81 (15.34 to 75.48) | 35.90 (15.61 to 76.85) | 35.79 (14.76 to 71.82) | 35.69 (15.51 to 77.95) | 35.91 (16.77 to 75.09) | 35.23 (15.77 to 77.02) | 34.70 (16.20 to 72.04) | 34.29 (14.89 to 75.49) | 33.39 (14.43 to 68.60) | 33.21 (15.22 to 66.84) | 32.36 (14.79 to 67.30) | 31.78 (14.60 to 65.29) | 31.38 (15.35 to 66.19) | 30.82 (14.00 to 64.90) |
| Yemen | Male | Prevalence | 106.15 (76.44 to 228.86) | 370.65 (205.83 to 744.96) | 405.59 (215.80 to 881.57) | 445.66 (223.61 to 1070.11) | 480.13 (229.42 to 1247.15) | 505.48 (233.39 to 1378.63) | 523.43 (236.51 to 1465.98) | 537.78 (239.03 to 1519.80) | 549.85 (241.07 to 1578.70) | 560.49 (243.64 to 1629.35) | 570.57 (245.17 to 1679.68) | 580.99 (246.54 to 1730.39) | 592.88 (248.01 to 1788.48) | 598.53 (248.17 to 1815.17) | 598.55 (248.39 to 1815.25) | 598.53 (248.62 to 1814.85) | 598.53 (249.02 to 1816.65) | 598.49 (248.50 to 1815.66) | 598.50 (249.22 to 1815.19) | 598.57 (248.74 to 1815.85) |
| Zambia | Both | DALY rates | 9.89 (6.07 to 15.52) | 31.51 (16.42 to 55.52) | 34.21 (18.69 to 62.07) | 37.89 (19.72 to 67.97) | 40.82 (21.91 to 72.12) | 43.11 (23.72 to 77.00) | 45.48 (24.71 to 81.87) | 47.26 (25.64 to 85.02) | 49.76 (27.23 to 90.13) | 51.68 (27.37 to 90.52) | 52.56 (29.17 to 91.67) | 52.67 (28.41 to 90.17) | 52.09 (28.63 to 93.00) | 51.21 (29.08 to 83.85) | 49.55 (26.91 to 82.18) | 47.49 (26.36 to 80.63) | 45.78 (26.50 to 76.84) | 43.94 (24.23 to 73.03) | 41.82 (24.17 to 67.59) | 37.58 (22.10 to 60.54) |
| Zambia | Both | Prevalence | 386.98 (154.52 to 1045.76) | 998.84 (415.49 to 2678.78) | 1347.25 (511.05 to 3773.23) | 1751.16 (621.60 to 5042.55) | 2097.40 (713.58 to 6127.73) | 2353.23 (787.33 to 6918.03) | 2539.69 (841.73 to 7492.11) | 2691.74 (887.98 to 7952.05) | 2824.99 (930.93 to 8349.98) | 2944.15 (971.05 to 8698.61) | 3048.70 (1003.22 to 9015.90) | 3147.48 (1027.75 to 9335.43) | 3259.07 (1056.13 to 9697.38) | 3308.31 (1068.26 to 9859.68) | 3303.04 (1064.89 to 9850.72) | 3293.20 (1055.75 to 9834.40) | 3284.49 (1047.59 to 9818.77) | 3277.63 (1041.74 to 9808.14) | 3261.75 (1027.37 to 9780.83) | 3231.21 (995.16 to 9728.81) |
| Zambia | Female | DALY rates | 11.58 (6.37 to 18.54) | 19.61 (11.48 to 30.35) | 20.08 (11.86 to 31.68) | 20.69 (12.15 to 31.94) | 20.90 (12.31 to 32.67) | 21.01 (12.74 to 32.17) | 21.64 (12.49 to 32.96) | 21.14 (13.05 to 32.31) | 21.38 (13.30 to 32.08) | 21.64 (12.65 to 32.67) | 21.32 (12.80 to 32.02) | 21.61 (13.20 to 32.80) | 21.62 (13.00 to 33.43) | 21.66 (13.72 to 31.86) | 20.99 (12.67 to 31.65) | 20.49 (12.09 to 30.66) | 20.46 (12.66 to 30.23) | 19.88 (12.13 to 29.99) | 19.80 (12.24 to 29.74) | 19.30 (11.56 to 28.31) |
| Zambia | Female | Prevalence | 403.99 (172.42 to 1061.49) | 908.21 (336.91 to 2530.35) | 1237.66 (407.45 to 3598.53) | 1617.81 (490.88 to 4829.45) | 1941.98 (560.98 to 5880.59) | 2178.18 (612.37 to 6645.89) | 2347.14 (649.01 to 7192.91) | 2483.09 (678.79 to 7632.82) | 2597.78 (703.39 to 8004.22) | 2697.71 (725.28 to 8327.05) | 2791.52 (746.22 to 8631.18) | 2889.15 (766.84 to 8947.11) | 3001.13 (792.17 to 9309.54) | 3054.62 (803.72 to 9482.16) | 3054.61 (803.50 to 9482.10) | 3054.62 (803.39 to 9482.72) | 3054.62 (803.28 to 9481.79) | 3054.66 (803.02 to 9483.36) | 3054.65 (803.35 to 9482.52) | 3054.61 (803.91 to 9482.45) |
| Zambia | Male | DALY rates | 8.22 (4.72 to 13.37) | 43.37 (18.50 to 88.11) | 48.49 (21.47 to 99.69) | 55.85 (25.60 to 113.04) | 62.35 (28.60 to 126.25) | 67.00 (31.32 to 135.33) | 70.63 (33.05 to 143.63) | 74.02 (34.65 to 145.75) | 77.06 (36.71 to 149.29) | 78.55 (37.31 to 147.13) | 80.53 (37.91 to 148.87) | 82.87 (41.19 to 150.53) | 84.87 (40.10 to 165.29) | 85.51 (41.42 to 156.41) | 84.00 (40.49 to 154.17) | 82.52 (40.95 to 154.18) | 80.83 (41.67 to 149.98) | 79.03 (39.21 to 143.52) | 78.07 (39.32 to 143.01) | 76.05 (39.28 to 142.97) |
| Zambia | Male | Prevalence | 370.20 (138.27 to 1030.25) | 1089.19 (453.69 to 2808.17) | 1458.00 (577.37 to 3926.56) | 1890.37 (716.71 to 5230.88) | 2265.39 (839.63 to 6352.91) | 2542.52 (929.43 to 7165.53) | 2742.80 (995.08 to 7758.47) | 2905.43 (1052.00 to 8227.78) | 3043.55 (1099.74 to 8633.21) | 3164.67 (1141.58 to 8982.96) | 3278.89 (1180.91 to 9310.30) | 3398.52 (1224.84 to 9656.11) | 3536.54 (1272.32 to 10058.55) | 3602.73 (1298.59 to 10245.64) | 3602.73 (1296.20 to 10247.12) | 3602.68 (1294.63 to 10246.51) | 3602.72 (1296.14 to 10245.65) | 3602.80 (1295.32 to 10245.37) | 3602.61 (1295.90 to 10245.09) | 3602.71 (1296.79 to 10246.84) |
| Zimbabwe | Both | DALY rates | 9.68 (6.14 to 14.43) | 27.86 (14.43 to 50.23) | 28.81 (15.90 to 52.16) | 29.66 (15.73 to 54.42) | 30.05 (16.27 to 55.11) | 30.30 (16.87 to 54.50) | 30.53 (16.11 to 55.30) | 30.62 (16.92 to 54.69) | 30.91 (16.46 to 55.05) | 31.15 (17.28 to 58.23) | 30.83 (17.54 to 53.25) | 30.05 (16.57 to 53.27) | 29.39 (16.62 to 48.93) | 28.94 (16.24 to 50.41) | 28.31 (16.48 to 47.70) | 27.67 (16.26 to 46.09) | 27.15 (16.00 to 45.35) | 26.95 (15.00 to 44.72) | 25.88 (14.89 to 43.63) | 23.59 (14.05 to 38.38) |
| Zimbabwe | Both | Prevalence | 196.86 (102.16 to 535.23) | 509.45 (244.95 to 1330.63) | 632.41 (266.56 to 1832.19) | 774.40 (288.16 to 2418.73) | 893.24 (305.41 to 2912.90) | 980.16 (318.29 to 3276.19) | 1043.19 (328.13 to 3537.58) | 1094.86 (337.28 to 3746.72) | 1140.17 (345.29 to 3923.91) | 1180.25 (352.40 to 4079.24) | 1212.86 (356.52 to 4222.25) | 1241.11 (357.39 to 4367.74) | 1276.60 (357.63 to 4536.27) | 1295.33 (359.43 to 4615.79) | 1293.64 (357.33 to 4614.47) | 1294.78 (358.53 to 4615.21) | 1295.32 (359.63 to 4615.77) | 1295.25 (359.17 to 4615.61) | 1289.36 (352.15 to 4611.74) | 1274.55 (339.51 to 4600.99) |
| Zimbabwe | Female | DALY rates | 11.44 (6.90 to 17.80) | 18.93 (10.77 to 30.08) | 19.05 (11.03 to 29.38) | 19.26 (11.12 to 30.09) | 19.31 (11.55 to 30.42) | 19.24 (11.73 to 28.62) | 19.27 (11.34 to 30.42) | 18.91 (11.73 to 27.64) | 18.87 (11.03 to 29.95) | 18.78 (11.21 to 29.13) | 18.78 (11.18 to 28.94) | 18.98 (11.54 to 28.61) | 18.91 (10.19 to 29.79) | 18.48 (11.01 to 27.79) | 18.44 (11.17 to 28.22) | 17.86 (11.16 to 27.72) | 17.39 (10.37 to 26.04) | 17.32 (9.97 to 25.26) | 17.01 (10.35 to 26.18) | 16.76 (10.09 to 25.32) |
| Zimbabwe | Female | Prevalence | 213.23 (117.78 to 549.33) | 442.29 (207.34 to 1282.16) | 559.93 (219.26 to 1783.84) | 696.22 (232.88 to 2366.44) | 811.41 (244.82 to 2857.40) | 896.16 (253.40 to 3218.75) | 957.00 (258.36 to 3478.75) | 1005.46 (262.68 to 3685.10) | 1046.07 (266.57 to 3858.86) | 1081.80 (269.92 to 4011.33) | 1115.37 (273.00 to 4154.13) | 1150.51 (276.13 to 4303.81) | 1190.68 (279.73 to 4475.90) | 1209.25 (282.26 to 4554.68) | 1209.26 (281.92 to 4554.66) | 1209.27 (281.65 to 4554.85) | 1209.29 (282.28 to 4554.56) | 1209.27 (281.76 to 4554.68) | 1209.30 (281.93 to 4555.14) | 1209.28 (281.45 to 4554.69) |
| Zimbabwe | Male | DALY rates | 7.95 (4.38 to 12.78) | 36.76 (15.83 to 78.37) | 38.60 (17.63 to 81.36) | 40.37 (18.01 to 84.11) | 41.75 (17.98 to 87.96) | 43.00 (19.46 to 87.51) | 43.72 (18.68 to 90.42) | 44.17 (19.31 to 92.13) | 44.16 (19.85 to 91.22) | 44.16 (20.56 to 93.96) | 44.25 (20.56 to 87.37) | 44.69 (19.57 to 90.89) | 45.20 (21.80 to 92.71) | 44.95 (19.96 to 88.70) | 43.92 (19.79 to 85.51) | 42.84 (20.23 to 85.16) | 42.11 (20.49 to 85.37) | 41.72 (18.59 to 84.68) | 41.15 (19.30 to 80.46) | 39.55 (18.00 to 81.86) |
| Zimbabwe | Male | Prevalence | 180.75 (85.90 to 521.60) | 576.36 (256.84 to 1450.75) | 705.16 (283.87 to 1913.22) | 855.04 (320.45 to 2485.87) | 982.40 (349.14 to 2979.11) | 1076.52 (369.88 to 3345.12) | 1144.16 (383.86 to 3610.03) | 1198.32 (395.28 to 3821.41) | 1243.79 (404.88 to 3998.79) | 1283.78 (413.81 to 4153.84) | 1321.40 (421.27 to 4301.26) | 1360.98 (429.09 to 4455.40) | 1406.25 (438.37 to 4630.48) | 1427.20 (442.93 to 4712.43) | 1427.18 (443.76 to 4712.08) | 1427.18 (443.80 to 4711.71) | 1427.18 (442.90 to 4712.58) | 1427.17 (443.44 to 4712.09) | 1427.13 (442.99 to 4712.13) | 1427.16 (443.48 to 4712.20) |
| **Abbreviations:** GBD, Global Burden of Disease, DALYs, disability-adjusted life years; SDI, socio-demographic index; UI, uncertainty interval. | | | | | | | | | | | | | | | | | | | | | | |
