# Supplementary material for: Global, regional, and national disability-adjusted life years and prevalence of lymphatic filariasis from 1990 to 2021: A trend and health inequality analysis based on the global burden of disease study 2021
Source: PLoS Negl Trop Dis. 2025 Apr 29;19(4):e0013017. doi: 10.1371/journal.pntd.0013017 (PMC12040265; doi:10.1371/journal.pntd.0013017)
Supplement: S8 Table — Abbreviations: GBD, Global Burden of Disease, DALYs, disability-adjusted life years; SDI, socio-demographic index; UI, uncertainty interval. (DOCX) [file pntd.0013017.s008.docx]

**S8 Table The number of prevalence and DALYs of lymphatic filariasis, by age group , sex, SDI levels, GBD regions, among 67 countries and territories, in 1990.**

| **Location** | **Sex** | **Metric** | **Age group (95% UI)** | | | | | | | | | | | | | | | | | | | |
| --- | --- | --- | --- | --- | --- | --- | --- | --- | --- | --- | --- | --- | --- | --- | --- | --- | --- | --- | --- | --- | --- | --- |
|  |  |  | **<5 years** | **5-9 years** | **10-14 years** | **15-19 years** | **20-24 years** | **25-29 years** | **30-34 years** | **35-39 years** | **40-44 years** | **45-49 years** | **50-54 years** | **55-59 years** | **60-64 years** | **65-69 years** | **70-74 years** | **75-79 years** | **80-84 years** | **85-89 years** | **90-94 years** | **95+ years** |
| Global | Both | Number of DALYs | 35354.20 (24272.08 to 49911.83) | 294106.29 (188298.99 to 448230.66) | 357340.30 (235900.28 to 522277.48) | 407370.01 (271162.03 to 584157.30) | 427940.28 (285534.37 to 606692.50) | 416163.20 (279279.25 to 585243.64) | 386617.61 (259577.77 to 543781.01) | 355249.89 (239530.82 to 499232.70) | 298574.38 (202488.98 to 419571.15) | 260310.57 (177038.75 to 364847.21) | 225676.49 (153974.97 to 312202.81) | 195567.11 (132852.50 to 272500.75) | 163065.96 (112020.95 to 226827.85) | 114183.95 (78702.36 to 157014.85) | 71696.18 (49762.45 to 97888.76) | 41481.84 (29003.74 to 56357.58) | 21898.75 (15308.18 to 29746.92) | 7850.28 (5545.19 to 10512.62) | 1814.08 (1285.57 to 2421.01) | 347.06 (248.06 to 462.77) |
| Global | Both | Number of prevalence | 5382795.13 (4605954.52 to 6354774.86) | 14014338.22 (11982139.93 to 16399469.90) | 17366051.83 (14856785.03 to 20536959.65) | 20136302.78 (17234651.57 to 23887378.43) | 21585199.97 (18482218.76 to 25740468.23) | 21179843.09 (18082089.27 to 25393686.04) | 19582974.60 (16737291.26 to 23530607.76) | 17915450.28 (15437737.18 to 21264373.32) | 15043201.65 (13057180.27 to 17851863.02) | 13120520.90 (11386359.91 to 15605852.85) | 11396700.05 (9842759.92 to 13647999.27) | 9921494.63 (8556365.29 to 11844287.73) | 8307106.05 (7167511.30 to 9864403.06) | 5987898.22 (5147832.36 to 7133799.49) | 3896796.59 (3339474.33 to 4641264.06) | 2333226.06 (1993187.18 to 2766542.95) | 1273941.21 (1089489.36 to 1521070.15) | 474860.71 (396951.75 to 589975.15) | 117938.76 (96157.55 to 152389.61) | 25536.83 (19948.34 to 34374.09) |
| Global | Female | Number of DALYs | 20221.11 (13854.85 to 28857.88) | 36500.81 (25059.70 to 52199.91) | 39059.88 (26423.67 to 56254.70) | 44364.47 (29872.95 to 64189.83) | 51309.07 (34639.34 to 74794.68) | 54489.36 (36624.41 to 78867.99) | 53221.36 (35659.67 to 76763.25) | 51767.34 (34860.32 to 75296.73) | 45404.54 (30229.39 to 66442.18) | 40761.34 (27271.12 to 59362.06) | 36545.33 (24281.74 to 52724.02) | 32800.42 (21662.16 to 47384.29) | 28387.93 (19004.06 to 40756.50) | 21138.10 (14319.67 to 30177.71) | 13776.54 (9190.99 to 19599.59) | 8250.06 (5530.83 to 11649.98) | 4637.65 (3152.63 to 6508.65) | 1691.28 (1158.03 to 2359.93) | 420.62 (291.36 to 586.10) | 94.16 (65.41 to 129.51) |
| Global | Female | Number of prevalence | 2647235.07 (2265249.04 to 3124666.77) | 5945249.27 (5059636.13 to 7094719.79) | 7353220.77 (6198632.22 to 8884510.86) | 8630875.72 (7232139.10 to 10511252.90) | 9472977.39 (7921473.25 to 11607400.33) | 9413195.30 (7840541.94 to 11615545.22) | 8608106.44 (7192078.89 to 10582437.71) | 7795032.75 (6587601.54 to 9441543.57) | 6463723.19 (5503212.71 to 7811414.10) | 5596886.66 (4760743.08 to 6860032.24) | 4860079.97 (4107269.07 to 6017573.99) | 4225959.85 (3576278.19 to 5201568.84) | 3529424.17 (2982259.59 to 4336540.28) | 2638355.44 (2217596.74 to 3255721.58) | 1774188.30 (1484528.19 to 2188127.96) | 1084210.63 (904700.17 to 1332695.03) | 609212.13 (505927.66 to 756574.30) | 236062.89 (190427.40 to 309512.64) | 63154.49 (48931.15 to 85627.24) | 15392.15 (11133.10 to 22595.20) |
| Global | Male | Number of DALYs | 15133.09 (10209.23 to 21520.24) | 257605.47 (160485.39 to 404313.05) | 318280.42 (206971.53 to 473947.46) | 363005.54 (240749.68 to 521448.93) | 376631.21 (248061.43 to 536348.36) | 361673.84 (240984.44 to 512068.93) | 333396.25 (220949.74 to 468172.98) | 303482.55 (202576.21 to 426335.32) | 253169.84 (169084.76 to 354626.55) | 219549.23 (147588.04 to 305440.24) | 189131.16 (128659.21 to 263123.08) | 162766.69 (110339.70 to 225849.24) | 134678.03 (92128.87 to 186008.67) | 93045.85 (63872.03 to 127838.47) | 57919.64 (39879.12 to 79012.70) | 33231.79 (23104.16 to 45158.81) | 17261.09 (12029.78 to 23378.45) | 6159.00 (4323.76 to 8268.00) | 1393.46 (983.57 to 1865.57) | 252.90 (178.98 to 337.50) |
| Global | Male | Number of prevalence | 2735560.06 (2340185.07 to 3229432.54) | 8069088.95 (6861395.02 to 9402907.70) | 10012831.06 (8528879.77 to 11707655.54) | 11505427.06 (9926874.35 to 13438263.74) | 12112222.58 (10486657.41 to 14167817.02) | 11766647.79 (10160380.19 to 13796134.88) | 10974868.16 (9471988.17 to 12917453.44) | 10120417.54 (8821466.23 to 11860834.36) | 8579478.46 (7565408.75 to 9988366.01) | 7523634.25 (6636257.46 to 8711635.44) | 6536620.08 (5754536.27 to 7612600.12) | 5695534.78 (5016900.64 to 6630365.84) | 4777681.88 (4202578.78 to 5539292.97) | 3349542.78 (2940415.98 to 3886402.12) | 2122608.28 (1860708.32 to 2457577.62) | 1249015.43 (1095602.06 to 1433328.04) | 664729.08 (581701.74 to 764552.45) | 238797.82 (206841.40 to 284132.22) | 54784.28 (46999.50 to 66366.07) | 10144.68 (8698.37 to 12178.61) |
| **SDI region** |  |  |  |  |  |  |  |  |  |  |  |  |  |  |  |  |  |  |  |  |  |  |
| Low SDI | Both | Number of DALYs | 9872.04 (6634.99 to 13987.26) | 88976.90 (57018.18 to 134699.01) | 109390.15 (72853.61 to 156694.04) | 120706.54 (80842.42 to 170390.21) | 121304.93 (80539.67 to 168072.90) | 113891.47 (76909.59 to 157999.07) | 102440.80 (69166.13 to 142339.24) | 92707.19 (62835.33 to 129445.75) | 76115.22 (51495.65 to 105797.84) | 66101.04 (45217.42 to 91627.29) | 57121.55 (39053.04 to 78917.40) | 48979.98 (33622.20 to 67218.99) | 40399.59 (28005.31 to 55712.46) | 29579.55 (20597.90 to 40570.46) | 19257.27 (13390.92 to 26264.09) | 10521.22 (7410.70 to 14307.17) | 4829.82 (3424.94 to 6520.84) | 1611.40 (1147.62 to 2147.26) | 379.86 (271.80 to 509.63) | 70.71 (50.95 to 94.79) |
| Low SDI | Both | Number of prevalence | 1763253.27 (1425240.59 to 2156828.28) | 4358422.13 (3632618.86 to 5306537.10) | 5204192.86 (4341261.03 to 6312001.78) | 5782987.94 (4822351.62 to 6958199.25) | 6007565.72 (5007371.82 to 7197293.16) | 5789844.43 (4823544.07 to 6907066.12) | 5226172.49 (4381454.66 to 6202298.10) | 4697783.45 (3950258.86 to 5551342.09) | 3870710.87 (3257283.88 to 4573808.65) | 3372360.53 (2838826.93 to 3995190.22) | 2922515.45 (2470140.77 to 3453915.99) | 2518160.88 (2136846.01 to 2959497.89) | 2087823.53 (1777458.45 to 2443311.23) | 1573294.78 (1341609.13 to 1833536.90) | 1053463.82 (897676.39 to 1232262.27) | 597253.42 (507517.41 to 700791.20) | 284606.90 (242721.59 to 331542.52) | 99625.14 (84903.55 to 116929.02) | 25174.80 (21201.19 to 29906.14) | 5227.14 (4372.47 to 6263.19) |
| Low SDI | Female | Number of DALYs | 5692.41 (3837.41 to 8146.85) | 9903.29 (6742.55 to 14179.57) | 10424.54 (7009.88 to 14987.71) | 11843.93 (8030.33 to 17014.28) | 13807.36 (9288.73 to 20022.66) | 14725.37 (9883.16 to 21261.15) | 14255.32 (9620.08 to 20506.90) | 13299.60 (8871.86 to 19575.64) | 11522.55 (7692.71 to 16878.99) | 10276.71 (6901.57 to 14860.27) | 9186.82 (6084.17 to 13260.42) | 7944.29 (5300.39 to 11546.83) | 6823.52 (4544.30 to 9853.85) | 5304.51 (3564.36 to 7623.54) | 3540.11 (2346.33 to 4991.38) | 2006.39 (1356.50 to 2837.75) | 970.66 (658.49 to 1355.22) | 357.80 (244.03 to 502.46) | 96.85 (66.79 to 134.71) | 22.44 (15.51 to 30.94) |
| Low SDI | Female | Number of prevalence | 876716.89 (709446.69 to 1072012.78) | 1874118.47 (1520742.35 to 2281879.25) | 2216920.24 (1791477.33 to 2715198.99) | 2514936.49 (2027659.90 to 3089457.37) | 2708102.54 (2180399.29 to 3316244.13) | 2650967.02 (2145422.46 to 3231991.78) | 2369456.21 (1928957.97 to 2866705.82) | 2093823.82 (1724566.62 to 2516399.19) | 1709750.11 (1413497.22 to 2043908.48) | 1478249.51 (1219211.70 to 1788090.35) | 1271731.55 (1052970.01 to 1525888.22) | 1081203.35 (898736.24 to 1286305.36) | 890741.70 (742290.26 to 1056753.33) | 689299.17 (574849.13 to 815598.50) | 470622.68 (393449.39 to 557488.79) | 274423.06 (228587.92 to 326649.34) | 135870.83 (113427.63 to 160895.17) | 50205.51 (41672.33 to 59995.61) | 13745.30 (11308.30 to 16664.77) | 3225.87 (2632.45 to 3946.83) |
| Low SDI | Male | Number of DALYs | 4179.64 (2804.40 to 6005.52) | 79073.61 (49133.34 to 122727.51) | 98965.62 (64825.31 to 143487.46) | 108862.61 (72709.91 to 153742.36) | 107497.57 (71100.20 to 150305.75) | 99166.10 (66166.14 to 137273.64) | 88185.48 (59678.13 to 121512.18) | 79407.59 (53562.08 to 109925.28) | 64592.67 (43894.81 to 88552.09) | 55824.33 (37904.47 to 76607.61) | 47934.74 (32524.26 to 65513.31) | 41035.69 (28253.50 to 55396.71) | 33576.07 (23226.22 to 45526.37) | 24275.04 (16800.09 to 32641.78) | 15717.16 (10947.18 to 21160.80) | 8514.83 (5984.92 to 11324.35) | 3859.17 (2705.78 to 5129.97) | 1253.60 (891.94 to 1646.29) | 283.01 (201.65 to 370.97) | 48.28 (34.71 to 63.10) |
| Low SDI | Male | Number of prevalence | 886536.38 (715794.64 to 1082400.42) | 2484303.66 (2114071.55 to 2947775.91) | 2987272.61 (2550055.00 to 3540858.68) | 3268051.45 (2797746.11 to 3868058.44) | 3299463.19 (2807975.22 to 3908083.60) | 3138877.41 (2666387.58 to 3704604.20) | 2856716.28 (2440087.50 to 3353181.70) | 2603959.63 (2241578.37 to 3033281.86) | 2160960.77 (1854198.19 to 2513350.27) | 1894111.02 (1625323.59 to 2207179.87) | 1650783.90 (1418344.62 to 1925492.11) | 1436957.54 (1239349.42 to 1671152.67) | 1197081.83 (1034099.73 to 1386044.16) | 883995.61 (766343.83 to 1019510.88) | 582841.14 (504174.85 to 670388.72) | 322830.36 (279069.81 to 372524.25) | 148736.07 (128993.70 to 170680.82) | 49419.63 (43131.67 to 56737.72) | 11429.50 (9883.91 to 13133.57) | 2001.27 (1732.87 to 2315.23) |
| Low-middle SDI | Both | Number of DALYs | 16514.07 (11212.87 to 23102.72) | 138390.86 (90526.96 to 207356.84) | 158831.42 (104984.94 to 231245.75) | 176194.40 (117717.19 to 252073.74) | 183510.51 (123064.49 to 261161.19) | 175235.80 (118093.85 to 248017.35) | 163598.82 (111389.23 to 229587.65) | 155967.92 (106136.23 to 218847.21) | 134565.45 (91754.48 to 187854.96) | 119155.88 (81458.45 to 165939.42) | 104814.11 (71956.61 to 145774.93) | 93044.62 (63630.81 to 130264.24) | 79744.12 (54649.00 to 110809.40) | 55369.38 (38163.47 to 76537.01) | 34397.04 (23870.89 to 47310.45) | 20453.22 (14226.58 to 27919.74) | 11370.22 (7996.65 to 15473.16) | 3939.11 (2791.38 to 5276.58) | 895.78 (636.92 to 1196.26) | 167.21 (119.53 to 221.85) |
| Low-middle SDI | Both | Number of prevalence | 2406267.80 (2106606.88 to 2834684.61) | 6351632.05 (5541113.16 to 7360325.51) | 7742023.76 (6755455.69 to 9041791.80) | 8949339.16 (7783173.00 to 10484048.73) | 9577584.38 (8363464.96 to 11195539.12) | 9240644.83 (8064039.16 to 10844937.06) | 8600280.56 (7516806.79 to 10095427.73) | 8158215.10 (7190219.14 to 9454052.48) | 6996385.61 (6199316.24 to 8080819.93) | 6166948.65 (5463842.23 to 7165901.81) | 5402361.53 (4775358.06 to 6354584.50) | 4784760.40 (4238861.89 to 5633919.89) | 4083909.82 (3622655.31 to 4762249.62) | 2903471.27 (2552462.97 to 3401913.04) | 1868692.16 (1636865.34 to 2191018.54) | 1142997.27 (1008307.85 to 1323726.63) | 653914.02 (580644.23 to 749273.07) | 234034.73 (204205.56 to 276673.62) | 55534.62 (47705.60 to 67443.19) | 11001.12 (9408.99 to 13393.67) |
| Low-middle SDI | Female | Number of DALYs | 9419.21 (6406.55 to 13328.12) | 17031.67 (11561.99 to 24245.43) | 18119.90 (12309.59 to 26056.00) | 20660.98 (13809.73 to 30125.10) | 24224.21 (16171.87 to 35435.21) | 25789.24 (17565.51 to 37342.21) | 25759.05 (17139.49 to 37246.76) | 26535.00 (17560.37 to 38750.12) | 23758.43 (15691.56 to 34767.86) | 21724.98 (14431.39 to 31621.53) | 19766.56 (13084.01 to 28453.44) | 18145.56 (11874.34 to 26062.70) | 15919.59 (10593.28 to 22485.53) | 11766.54 (7917.08 to 16844.75) | 7610.00 (5027.57 to 10822.95) | 4656.90 (3109.63 to 6630.56) | 2777.94 (1867.22 to 3872.25) | 981.52 (672.79 to 1374.11) | 228.70 (157.11 to 317.08) | 46.18 (31.81 to 63.65) |
| Low-middle SDI | Female | Number of prevalence | 1175529.19 (1029889.97 to 1384515.72) | 2653524.25 (2311439.06 to 3136410.41) | 3250978.48 (2794058.18 to 3870365.16) | 3798171.94 (3248169.39 to 4546293.07) | 4165428.36 (3560049.65 to 4988929.32) | 4100358.21 (3500222.48 to 4919471.67) | 3795927.66 (3258241.34 to 4537661.79) | 3582909.54 (3113808.38 to 4215545.90) | 3033489.00 (2641620.25 to 3569719.46) | 2647111.67 (2297546.78 to 3145681.48) | 2310033.99 (1999126.42 to 2792459.42) | 2034853.28 (1760725.82 to 2460354.32) | 1717512.77 (1483999.55 to 2066077.80) | 1268036.83 (1089483.13 to 1525936.77) | 846556.86 (723639.84 to 1018082.78) | 522336.91 (449341.09 to 617772.75) | 305638.90 (265745.11 to 359582.09) | 113235.85 (96715.59 to 136963.20) | 28116.48 (23419.70 to 35160.57) | 5942.06 (4862.73 to 7471.26) |
| Low-middle SDI | Male | Number of DALYs | 7094.85 (4791.44 to 10251.50) | 121359.19 (78287.76 to 184920.91) | 140711.52 (91984.92 to 206644.11) | 155533.42 (102935.27 to 226108.73) | 159286.30 (105437.40 to 227536.19) | 149446.57 (99997.11 to 212283.68) | 137839.77 (91524.81 to 196281.94) | 129432.92 (86816.36 to 184005.26) | 110807.02 (74752.36 to 155888.96) | 97430.90 (65727.55 to 136094.50) | 85047.55 (57952.79 to 117809.11) | 74899.06 (51373.24 to 104531.92) | 63824.53 (43905.48 to 88480.55) | 43602.84 (30283.54 to 59859.11) | 26787.04 (18739.19 to 36569.91) | 15796.32 (11074.85 to 21448.28) | 8592.28 (6102.45 to 11665.43) | 2957.59 (2095.25 to 3991.48) | 667.08 (472.65 to 895.00) | 121.03 (86.44 to 162.05) |
| Low-middle SDI | Male | Number of prevalence | 1230738.61 (1080562.69 to 1448407.43) | 3698107.79 (3231547.78 to 4291426.79) | 4491045.28 (3921171.04 to 5214663.02) | 5151167.22 (4532586.97 to 5953096.98) | 5412156.01 (4789409.92 to 6248711.23) | 5140286.62 (4534773.58 to 5949517.61) | 4804352.90 (4232608.39 to 5578107.68) | 4575305.56 (4055614.87 to 5260509.97) | 3962896.60 (3531295.06 to 4537698.53) | 3519836.99 (3143586.47 to 4042039.99) | 3092327.54 (2753315.22 to 3570674.48) | 2749907.12 (2455939.35 to 3172044.12) | 2366397.05 (2115713.49 to 2710063.60) | 1635434.44 (1456843.03 to 1881152.23) | 1022135.30 (908587.16 to 1170378.03) | 620660.36 (555827.11 to 704528.00) | 348275.12 (313349.28 to 393384.56) | 120798.88 (107537.36 to 139869.39) | 27418.14 (24099.70 to 32312.02) | 5059.05 (4456.47 to 5953.27) |
| Middle SDI | Both | Number of DALYs | 8327.88 (5648.02 to 11843.91) | 63194.12 (38867.63 to 100175.62) | 84529.05 (53762.72 to 128742.16) | 104677.87 (68616.10 to 153737.06) | 116180.92 (77899.79 to 165801.58) | 119440.30 (79891.78 to 170061.55) | 113308.83 (75427.35 to 160800.05) | 100232.19 (67114.28 to 140865.16) | 82634.55 (55364.82 to 116021.85) | 70868.98 (47963.80 to 98790.84) | 60305.57 (40795.83 to 83345.90) | 50943.10 (34453.61 to 70469.81) | 40869.20 (27816.66 to 56460.35) | 27949.56 (19194.61 to 38154.67) | 17266.08 (11866.09 to 23395.12) | 10064.71 (7022.25 to 13574.05) | 5446.42 (3799.20 to 7311.69) | 2199.63 (1544.62 to 2948.29) | 507.28 (358.72 to 680.02) | 99.44 (70.53 to 131.31) |
| Middle SDI | Both | Number of prevalence | 1143700.62 (771581.38 to 1773576.38) | 3122963.98 (2168509.99 to 4607431.72) | 4184377.38 (2916455.96 to 6222043.60) | 5113445.57 (3583124.37 to 7664633.10) | 5660968.22 (3975499.25 to 8444426.23) | 5787601.24 (4076186.83 to 8618564.00) | 5418493.73 (3820478.45 to 8105186.74) | 4768165.11 (3402403.66 to 7100560.63) | 3935653.44 (2849997.82 to 5801686.63) | 3390187.28 (2437844.62 to 5068810.35) | 2911970.84 (2048783.45 to 4414666.68) | 2495238.78 (1733999.57 to 3785944.26) | 2036403.05 (1402871.24 to 3117430.45) | 1447233.09 (982206.66 to 2243926.03) | 934194.32 (626495.40 to 1451731.71) | 568905.77 (376486.61 to 864368.10) | 321188.60 (209985.08 to 495759.35) | 135444.87 (84679.05 to 215497.11) | 35232.95 (20751.68 to 61550.02) | 8658.05 (4466.42 to 16732.39) |
| Middle SDI | Female | Number of DALYs | 4746.18 (3215.08 to 6820.60) | 8938.59 (6076.92 to 12780.11) | 9874.34 (6613.56 to 14262.59) | 11182.41 (7501.48 to 15843.52) | 12555.19 (8452.25 to 18020.90) | 13246.11 (8939.20 to 19454.83) | 12555.14 (8428.50 to 17965.12) | 11383.85 (7628.42 to 16314.41) | 9676.53 (6519.33 to 13918.93) | 8407.32 (5637.21 to 12189.06) | 7294.76 (4898.04 to 10472.44) | 6474.54 (4302.82 to 9286.84) | 5450.93 (3667.97 to 7859.46) | 3938.70 (2662.99 to 5660.17) | 2544.40 (1741.83 to 3646.44) | 1538.53 (1058.73 to 2181.56) | 861.44 (600.43 to 1211.11) | 341.18 (239.19 to 477.45) | 91.06 (65.13 to 125.38) | 24.26 (16.91 to 33.03) |
| Middle SDI | Female | Number of prevalence | 561041.96 (379622.80 to 867097.24) | 1339375.17 (897805.47 to 2095896.06) | 1784309.09 (1181025.28 to 2815949.09) | 2192807.63 (1431731.08 to 3492297.93) | 2454456.46 (1592404.26 to 3902223.81) | 2510488.15 (1628028.50 to 4000312.47) | 2305275.56 (1494335.96 to 3671722.44) | 2002245.54 (1319523.87 to 3169914.75) | 1625705.12 (1086170.55 to 2567776.16) | 1397077.53 (914697.46 to 2254202.27) | 1215272.19 (778287.90 to 1998670.81) | 1060019.89 (671815.44 to 1741228.88) | 880939.58 (555571.35 to 1454321.24) | 654494.21 (408186.44 to 1070750.27) | 439647.25 (270739.16 to 714368.80) | 276778.67 (167939.18 to 451899.40) | 161258.88 (95502.18 to 268830.03) | 70048.88 (38934.18 to 123789.46) | 20304.21 (10092.49 to 39461.20) | 5898.58 (2556.78 to 12754.89) |
| Middle SDI | Male | Number of DALYs | 3581.70 (2405.76 to 5096.49) | 54255.53 (31673.62 to 89120.37) | 74654.71 (46136.56 to 116065.52) | 93495.46 (60822.35 to 138562.76) | 103625.73 (68263.35 to 149397.63) | 106194.19 (70124.85 to 152047.02) | 100753.69 (66725.81 to 142913.64) | 88848.33 (59308.46 to 125459.61) | 72958.02 (48733.03 to 103167.84) | 62461.66 (42144.21 to 87775.52) | 53010.81 (35964.25 to 74040.45) | 44468.56 (30030.85 to 62106.25) | 35418.27 (23880.46 to 48845.15) | 24010.86 (16257.72 to 32967.07) | 14721.68 (10045.64 to 20202.51) | 8526.18 (5817.66 to 11636.80) | 4584.98 (3153.93 to 6260.65) | 1858.46 (1304.27 to 2513.52) | 416.22 (291.97 to 559.02) | 75.18 (53.30 to 100.47) |
| Middle SDI | Male | Number of prevalence | 582658.66 (392308.03 to 906479.14) | 1783588.81 (1269753.46 to 2546995.85) | 2400068.29 (1717337.12 to 3434763.55) | 2920637.94 (2120125.87 to 4164852.23) | 3206511.76 (2347967.81 to 4555547.20) | 3277113.09 (2413012.62 to 4662056.34) | 3113218.17 (2301987.76 to 4448564.81) | 2765919.57 (2055935.24 to 3937436.53) | 2309948.32 (1750094.63 to 3241603.73) | 1993109.75 (1509818.87 to 2810953.86) | 1696698.65 (1263913.82 to 2418495.63) | 1435218.88 (1061184.61 to 2051308.42) | 1155463.47 (847548.03 to 1665329.15) | 792738.89 (571799.67 to 1161618.46) | 494547.07 (353002.62 to 726129.83) | 292127.10 (209716.22 to 423488.35) | 159929.72 (114492.21 to 233777.50) | 65395.99 (45771.04 to 98145.88) | 14928.74 (10319.36 to 22881.01) | 2759.47 (1916.93 to 4184.41) |
| High-middle SDI | Both | Number of DALYs | 606.20 (405.73 to 864.56) | 3157.41 (1787.26 to 5399.72) | 4108.15 (2379.08 to 6719.99) | 5267.40 (3272.25 to 8358.03) | 6409.09 (4106.85 to 9678.46) | 7070.55 (4581.52 to 10465.15) | 6787.51 (4390.14 to 9782.54) | 5916.30 (3865.30 to 8557.75) | 4905.99 (3251.41 to 7048.54) | 3882.55 (2581.01 to 5549.37) | 3172.75 (2114.03 to 4479.18) | 2373.57 (1604.23 to 3351.45) | 1859.82 (1245.31 to 2627.60) | 1143.73 (778.64 to 1598.80) | 687.63 (470.97 to 974.68) | 395.58 (271.78 to 545.34) | 229.69 (155.39 to 325.96) | 91.77 (62.45 to 129.71) | 29.33 (20.03 to 42.13) | 9.27 (6.00 to 13.58) |
| High-middle SDI | Both | Number of prevalence | 62871.03 (27157.73 to 150526.16) | 163721.45 (75683.49 to 371448.73) | 212595.59 (96240.05 to 490883.57) | 263841.20 (119845.62 to 603670.17) | 310340.09 (144353.58 to 730283.31) | 333102.14 (158646.55 to 767697.07) | 311946.94 (149839.52 to 679664.89) | 268339.27 (129898.29 to 587930.58) | 221549.25 (108837.94 to 484553.98) | 174917.02 (86418.63 to 376219.65) | 145933.90 (70408.54 to 321872.26) | 111426.27 (55350.19 to 238427.55) | 88830.99 (43548.02 to 194976.75) | 56346.39 (27158.24 to 124666.25) | 35607.35 (16222.78 to 79746.92) | 21399.81 (9721.66 to 49633.78) | 12903.43 (5532.04 to 32619.48) | 5242.09 (2111.40 to 14089.07) | 1872.36 (623.64 to 5863.54) | 619.19 (175.65 to 2129.27) |
| High-middle SDI | Female | Number of DALYs | 343.78 (230.64 to 496.00) | 590.25 (381.59 to 851.46) | 595.47 (385.50 to 860.40) | 618.08 (413.46 to 878.75) | 646.94 (446.68 to 952.12) | 642.73 (434.40 to 911.04) | 567.09 (372.10 to 802.45) | 469.23 (305.83 to 675.65) | 376.18 (251.87 to 522.34) | 289.60 (199.40 to 419.73) | 241.25 (158.72 to 344.52) | 186.49 (124.88 to 269.36) | 148.32 (101.24 to 208.78) | 93.73 (63.99 to 131.06) | 60.53 (40.24 to 84.39) | 37.01 (25.52 to 51.72) | 22.39 (15.15 to 31.60) | 8.81 (6.04 to 12.16) | 3.53 (2.33 to 4.99) | 1.14 (0.75 to 1.61) |
| High-middle SDI | Female | Number of prevalence | 30652.76 (13426.22 to 73446.96) | 70878.71 (29946.27 to 171342.07) | 91367.66 (36746.01 to 224304.11) | 113313.88 (43781.14 to 292654.41) | 131993.59 (50404.35 to 346330.44) | 138225.13 (53369.78 to 353960.18) | 125528.67 (48109.63 to 307512.25) | 105637.09 (41001.84 to 263025.56) | 86237.80 (34266.24 to 212898.53) | 67182.38 (26635.94 to 164343.10) | 56758.12 (21771.98 to 142166.05) | 44520.25 (17913.97 to 107212.14) | 35613.46 (14083.93 to 88067.20) | 23034.84 (9010.86 to 56697.00) | 15088.46 (5633.37 to 38594.08) | 9391.13 (3523.96 to 24322.04) | 5787.07 (2036.93 to 16401.72) | 2305.94 (712.20 to 6964.16) | 917.20 (209.94 to 3223.86) | 305.23 (56.29 to 1160.19) |
| High-middle SDI | Male | Number of DALYs | 262.42 (167.47 to 387.47) | 2567.16 (1301.72 to 4671.60) | 3512.68 (1928.00 to 5913.80) | 4649.32 (2792.09 to 7589.51) | 5762.15 (3616.55 to 8849.66) | 6427.82 (4095.01 to 9649.68) | 6220.43 (3967.29 to 9046.49) | 5447.07 (3546.16 to 7951.10) | 4529.81 (2974.63 to 6575.91) | 3592.95 (2346.99 to 5177.70) | 2931.51 (1938.68 to 4180.07) | 2187.08 (1468.13 to 3122.90) | 1711.50 (1132.05 to 2437.88) | 1050.00 (706.34 to 1481.20) | 627.09 (426.49 to 894.24) | 358.57 (243.04 to 499.11) | 207.30 (139.45 to 296.22) | 82.96 (55.68 to 119.12) | 25.81 (17.29 to 38.00) | 8.14 (5.15 to 12.29) |
| High-middle SDI | Male | Number of prevalence | 32218.27 (13742.75 to 77079.20) | 92842.74 (44588.73 to 200982.05) | 121227.92 (59595.01 to 264281.97) | 150527.32 (75513.48 to 316864.11) | 178346.50 (92570.98 to 381344.62) | 194877.01 (103183.51 to 409960.42) | 186418.26 (99054.39 to 379019.54) | 162702.18 (87667.43 to 326367.51) | 135311.45 (74196.56 to 270471.52) | 107734.63 (59623.61 to 213287.99) | 89175.78 (48868.95 to 178462.51) | 66906.02 (37320.21 to 130329.36) | 53217.53 (29183.77 to 107487.75) | 33311.55 (18206.97 to 67337.37) | 20518.89 (10799.65 to 42105.76) | 12008.68 (6260.53 to 25326.46) | 7116.35 (3626.33 to 16159.32) | 2936.14 (1405.04 to 7087.79) | 955.16 (409.05 to 2633.34) | 313.95 (117.52 to 965.86) |
| High SDI | Both | DALY rates | 0.00 (0.00 to 0.00) | 0.00 (0.00 to 0.00) | 0.00 (0.00 to 0.00) | 0.00 (0.00 to 0.00) | 0.00 (0.00 to 0.00) | 0.00 (0.00 to 0.00) | 0.00 (0.00 to 0.00) | 0.00 (0.00 to 0.00) | 0.00 (0.00 to 0.00) | 0.00 (0.00 to 0.00) | 0.00 (0.00 to 0.00) | 0.00 (0.00 to 0.00) | 0.00 (0.00 to 0.00) | 0.00 (0.00 to 0.00) | 0.00 (0.00 to 0.00) | 0.00 (0.00 to 0.00) | 0.00 (0.00 to 0.00) | 0.00 (0.00 to 0.00) | 0.00 (0.00 to 0.00) | 0.00 (0.00 to 0.00) |
| High SDI | Both | Number of Prevalence | 0.00 (0.00 to 0.00) | 0.00 (0.00 to 0.00) | 0.00 (0.00 to 0.00) | 0.00 (0.00 to 0.00) | 0.00 (0.00 to 0.00) | 0.00 (0.00 to 0.00) | 0.00 (0.00 to 0.00) | 0.00 (0.00 to 0.00) | 0.00 (0.00 to 0.00) | 0.00 (0.00 to 0.00) | 0.00 (0.00 to 0.00) | 0.00 (0.00 to 0.00) | 0.00 (0.00 to 0.00) | 0.00 (0.00 to 0.00) | 0.00 (0.00 to 0.00) | 0.00 (0.00 to 0.00) | 0.00 (0.00 to 0.00) | 0.00 (0.00 to 0.00) | 0.00 (0.00 to 0.00) | 0.00 (0.00 to 0.00) |
| High SDI | Female | DALY rates | 0.00 (0.00 to 0.00) | 0.00 (0.00 to 0.00) | 0.00 (0.00 to 0.00) | 0.00 (0.00 to 0.00) | 0.00 (0.00 to 0.00) | 0.00 (0.00 to 0.00) | 0.00 (0.00 to 0.00) | 0.00 (0.00 to 0.00) | 0.00 (0.00 to 0.00) | 0.00 (0.00 to 0.00) | 0.00 (0.00 to 0.00) | 0.00 (0.00 to 0.00) | 0.00 (0.00 to 0.00) | 0.00 (0.00 to 0.00) | 0.00 (0.00 to 0.00) | 0.00 (0.00 to 0.00) | 0.00 (0.00 to 0.00) | 0.00 (0.00 to 0.00) | 0.00 (0.00 to 0.00) | 0.00 (0.00 to 0.00) |
| High SDI | Female | Number of Prevalence | 0.00 (0.00 to 0.00) | 0.00 (0.00 to 0.00) | 0.00 (0.00 to 0.00) | 0.00 (0.00 to 0.00) | 0.00 (0.00 to 0.00) | 0.00 (0.00 to 0.00) | 0.00 (0.00 to 0.00) | 0.00 (0.00 to 0.00) | 0.00 (0.00 to 0.00) | 0.00 (0.00 to 0.00) | 0.00 (0.00 to 0.00) | 0.00 (0.00 to 0.00) | 0.00 (0.00 to 0.00) | 0.00 (0.00 to 0.00) | 0.00 (0.00 to 0.00) | 0.00 (0.00 to 0.00) | 0.00 (0.00 to 0.00) | 0.00 (0.00 to 0.00) | 0.00 (0.00 to 0.00) | 0.00 (0.00 to 0.00) |
| High SDI | Male | DALY rates | 0.00 (0.00 to 0.00) | 0.00 (0.00 to 0.00) | 0.00 (0.00 to 0.00) | 0.00 (0.00 to 0.00) | 0.00 (0.00 to 0.00) | 0.00 (0.00 to 0.00) | 0.00 (0.00 to 0.00) | 0.00 (0.00 to 0.00) | 0.00 (0.00 to 0.00) | 0.00 (0.00 to 0.00) | 0.00 (0.00 to 0.00) | 0.00 (0.00 to 0.00) | 0.00 (0.00 to 0.00) | 0.00 (0.00 to 0.00) | 0.00 (0.00 to 0.00) | 0.00 (0.00 to 0.00) | 0.00 (0.00 to 0.00) | 0.00 (0.00 to 0.00) | 0.00 (0.00 to 0.00) | 0.00 (0.00 to 0.00) |
| High SDI | Male | Number of Prevalence | 0.00 (0.00 to 0.00) | 0.00 (0.00 to 0.00) | 0.00 (0.00 to 0.00) | 0.00 (0.00 to 0.00) | 0.00 (0.00 to 0.00) | 0.00 (0.00 to 0.00) | 0.00 (0.00 to 0.00) | 0.00 (0.00 to 0.00) | 0.00 (0.00 to 0.00) | 0.00 (0.00 to 0.00) | 0.00 (0.00 to 0.00) | 0.00 (0.00 to 0.00) | 0.00 (0.00 to 0.00) | 0.00 (0.00 to 0.00) | 0.00 (0.00 to 0.00) | 0.00 (0.00 to 0.00) | 0.00 (0.00 to 0.00) | 0.00 (0.00 to 0.00) | 0.00 (0.00 to 0.00) | 0.00 (0.00 to 0.00) |
| **GBD region** |  |  |  |  |  |  |  |  |  |  |  |  |  |  |  |  |  |  |  |  |  |  |
| Caribbean | Both | Number of DALYs | 287.38 (185.32 to 412.09) | 2817.36 (1827.87 to 4239.69) | 3827.56 (2571.16 to 5383.72) | 4135.87 (2736.65 to 5775.86) | 3988.68 (2702.16 to 5529.80) | 3728.96 (2569.18 to 5285.45) | 3366.42 (2272.56 to 4646.18) | 3083.57 (2081.55 to 4316.89) | 2444.26 (1673.40 to 3385.82) | 2094.66 (1433.15 to 2954.16) | 1806.03 (1235.74 to 2497.09) | 1592.23 (1102.57 to 2167.46) | 1366.71 (950.24 to 1887.40) | 1036.16 (717.63 to 1442.13) | 679.04 (472.70 to 916.19) | 401.40 (278.89 to 556.43) | 212.01 (146.59 to 284.88) | 80.10 (55.97 to 109.55) | 18.39 (12.93 to 24.58) | 4.15 (2.95 to 5.55) |
| Caribbean | Both | Number of prevalence | 53626.46 (14556.11 to 131140.83) | 133525.54 (48691.80 to 300830.04) | 176711.09 (67343.10 to 390260.72) | 206005.37 (78092.89 to 450603.72) | 217568.84 (80986.79 to 479082.35) | 213314.84 (78307.68 to 471626.28) | 195688.39 (70951.34 to 437278.26) | 179635.31 (64491.21 to 405436.08) | 141189.33 (51202.75 to 316773.03) | 120822.83 (43576.83 to 273126.13) | 104874.97 (37566.10 to 236520.70) | 92419.49 (32907.33 to 206407.42) | 79371.69 (28512.12 to 174606.61) | 61356.76 (22156.68 to 133958.11) | 41071.44 (14778.51 to 88973.70) | 24455.64 (9020.19 to 51655.65) | 13074.67 (4901.03 to 28491.90) | 5068.00 (1978.56 to 11066.04) | 1220.17 (498.14 to 2564.84) | 280.74 (113.55 to 648.31) |
| Caribbean | Female | Number of DALYs | 167.56 (104.66 to 243.10) | 295.63 (191.03 to 437.44) | 353.37 (231.41 to 506.85) | 426.25 (276.04 to 628.67) | 502.23 (334.47 to 714.64) | 541.86 (357.93 to 791.60) | 528.37 (343.90 to 771.81) | 505.60 (345.75 to 744.17) | 406.91 (267.15 to 587.34) | 356.02 (235.47 to 524.55) | 320.75 (211.59 to 466.87) | 289.37 (194.28 to 416.05) | 262.67 (172.85 to 379.99) | 213.24 (140.09 to 301.47) | 142.75 (94.92 to 206.70) | 86.41 (56.71 to 121.84) | 47.75 (31.82 to 67.77) | 19.81 (13.33 to 27.84) | 5.03 (3.42 to 7.13) | 1.01 (0.69 to 1.43) |
| Caribbean | Female | Number of prevalence | 26944.45 (7400.05 to 65632.31) | 58260.31 (15460.75 to 142867.99) | 77104.83 (21169.68 to 186735.55) | 92270.99 (25990.52 to 219475.53) | 101183.90 (29100.24 to 238468.50) | 101608.75 (29274.14 to 240485.53) | 93408.35 (26780.70 to 222759.75) | 84529.88 (24383.65 to 202779.38) | 65144.04 (19008.20 to 154934.18) | 54896.97 (16098.31 to 131187.71) | 48052.66 (14005.70 to 115471.23) | 41862.50 (12205.86 to 99511.63) | 35982.32 (10710.25 to 83976.29) | 28486.72 (8571.29 to 66049.41) | 19556.07 (5829.63 to 45435.68) | 11879.83 (3653.52 to 27072.99) | 6586.07 (2067.46 to 14548.85) | 2669.62 (883.96 to 5909.32) | 673.40 (232.87 to 1450.91) | 154.81 (47.63 to 370.20) |
| Caribbean | Male | Number of DALYs | 119.82 (74.10 to 180.79) | 2521.73 (1595.35 to 3873.39) | 3474.19 (2328.36 to 4938.87) | 3709.63 (2458.39 to 5155.78) | 3486.45 (2367.07 to 4931.32) | 3187.10 (2166.00 to 4520.49) | 2838.05 (1922.73 to 3942.12) | 2577.97 (1736.66 to 3612.07) | 2037.35 (1369.85 to 2830.42) | 1738.65 (1192.00 to 2439.77) | 1485.28 (1006.80 to 2041.26) | 1302.86 (899.15 to 1777.01) | 1104.04 (764.51 to 1524.49) | 822.93 (573.64 to 1130.77) | 536.29 (372.02 to 726.89) | 314.99 (218.12 to 429.22) | 164.26 (114.14 to 223.14) | 60.29 (42.12 to 81.94) | 13.35 (9.47 to 17.96) | 3.15 (2.22 to 4.24) |
| Caribbean | Male | Number of prevalence | 26682.02 (7079.26 to 65508.52) | 75265.22 (32733.10 to 158544.65) | 99606.27 (45480.44 to 204973.33) | 113734.38 (51769.03 to 232460.16) | 116384.94 (51655.56 to 240309.73) | 111706.09 (48773.12 to 232053.03) | 102280.04 (44005.64 to 214784.81) | 95105.43 (40269.92 to 202656.70) | 76045.29 (32123.99 to 161838.85) | 65925.86 (27477.19 to 141041.96) | 56822.31 (23518.77 to 121016.76) | 50556.99 (20635.43 to 107216.17) | 43389.37 (17765.38 to 90193.80) | 32870.04 (13578.43 to 67395.23) | 21515.37 (8947.63 to 43653.52) | 12575.81 (5341.99 to 25122.39) | 6488.60 (2856.28 to 13519.25) | 2398.38 (1090.84 to 5084.36) | 546.77 (264.01 to 1116.62) | 125.93 (62.56 to 270.71) |
| Central Sub-Saharan Africa | Both | Number of DALYs | 1214.35 (776.63 to 1773.49) | 9038.99 (5297.45 to 14660.20) | 12472.45 (8100.55 to 18782.98) | 15607.18 (10342.24 to 22226.57) | 16217.76 (10723.85 to 22495.41) | 14836.05 (9896.93 to 20520.18) | 12503.50 (8396.05 to 17098.75) | 10570.22 (7141.50 to 14627.77) | 7372.28 (5051.14 to 10023.00) | 6460.85 (4406.07 to 8848.27) | 5673.43 (3895.00 to 7707.02) | 4867.21 (3366.09 to 6563.26) | 3821.04 (2629.60 to 5154.50) | 2763.84 (1944.73 to 3685.94) | 1795.81 (1260.31 to 2408.41) | 898.42 (634.46 to 1213.92) | 307.16 (217.01 to 409.17) | 97.92 (69.92 to 129.28) | 25.72 (18.48 to 33.74) | 5.23 (3.79 to 6.84) |
| Central Sub-Saharan Africa | Both | Number of prevalence | 208083.35 (113656.99 to 340753.90) | 481553.45 (283548.54 to 763031.64) | 590476.14 (354338.48 to 929012.93) | 688631.18 (418281.98 to 1074263.79) | 722855.50 (441372.03 to 1129918.88) | 688385.46 (418648.44 to 1076451.54) | 599997.27 (363572.04 to 937523.79) | 520014.85 (315731.18 to 811588.45) | 385855.96 (232412.25 to 603800.00) | 347386.77 (208874.43 to 544433.46) | 314803.41 (188456.20 to 495977.45) | 278197.75 (165013.87 to 440712.63) | 224493.75 (132593.60 to 356538.23) | 165494.26 (97376.53 to 262805.81) | 107545.28 (63118.62 to 170549.41) | 53257.78 (31655.99 to 84547.59) | 18234.72 (10955.43 to 28642.28) | 6229.43 (3744.72 to 9747.58) | 1776.95 (1060.53 to 2810.03) | 422.60 (247.65 to 673.74) |
| Central Sub-Saharan Africa | Female | Number of DALYs | 700.93 (435.55 to 1040.23) | 1138.92 (715.28 to 1745.42) | 1126.00 (717.65 to 1619.91) | 1176.27 (793.87 to 1712.42) | 1223.19 (777.39 to 1769.60) | 1199.40 (807.10 to 1721.05) | 1069.69 (693.29 to 1518.02) | 941.26 (639.26 to 1354.26) | 749.15 (497.81 to 1067.68) | 692.23 (461.62 to 956.74) | 651.68 (438.89 to 930.33) | 591.67 (406.27 to 842.91) | 491.91 (330.95 to 710.67) | 358.99 (244.88 to 507.53) | 224.52 (149.22 to 322.78) | 104.50 (71.01 to 144.67) | 33.28 (23.13 to 45.78) | 12.32 (8.58 to 16.93) | 3.76 (2.64 to 5.17) | 1.02 (0.70 to 1.41) |
| Central Sub-Saharan Africa | Female | Number of prevalence | 104118.56 (57560.58 to 169987.53) | 212554.66 (115630.48 to 350125.91) | 252160.38 (136010.08 to 417618.14) | 287940.14 (154831.39 to 478809.20) | 305113.31 (164491.74 to 507512.39) | 296975.44 (160591.70 to 492585.71) | 262040.37 (141662.89 to 433950.87) | 228051.82 (123472.58 to 376760.55) | 177963.39 (96527.07 to 294158.21) | 161989.21 (88056.23 to 267846.14) | 149358.63 (80599.53 to 248138.06) | 133009.29 (71362.31 to 221171.32) | 107870.34 (57750.00 to 179461.30) | 79109.71 (42291.81 to 131783.32) | 50313.95 (26963.49 to 84180.23) | 23770.68 (12977.56 to 39202.25) | 7879.85 (4297.57 to 12986.23) | 2922.31 (1639.20 to 4780.38) | 911.37 (507.80 to 1491.20) | 251.60 (139.56 to 412.34) |
| Central Sub-Saharan Africa | Male | Number of DALYs | 513.43 (319.85 to 784.52) | 7900.07 (4446.04 to 13322.34) | 11346.45 (7221.22 to 17383.27) | 14430.91 (9439.77 to 20894.88) | 14994.57 (9960.07 to 20798.46) | 13636.65 (9075.41 to 18967.90) | 11433.81 (7666.15 to 15728.87) | 9628.95 (6535.46 to 13397.50) | 6623.12 (4541.40 to 9135.92) | 5768.62 (3942.94 to 7932.19) | 5021.75 (3461.92 to 6860.21) | 4275.53 (2951.30 to 5794.18) | 3329.12 (2266.05 to 4517.54) | 2404.85 (1669.16 to 3252.78) | 1571.29 (1104.47 to 2129.24) | 793.91 (563.45 to 1075.37) | 273.88 (194.25 to 367.81) | 85.60 (60.69 to 114.56) | 21.95 (15.82 to 28.85) | 4.22 (3.03 to 5.57) |
| Central Sub-Saharan Africa | Male | Number of prevalence | 103964.79 (56222.26 to 170770.16) | 268998.79 (167137.16 to 415689.53) | 338315.76 (216155.67 to 510244.48) | 400691.04 (262740.87 to 596597.43) | 417742.19 (274927.87 to 620624.16) | 391410.02 (256309.63 to 581239.84) | 337956.90 (220592.43 to 501461.87) | 291963.02 (191049.00 to 433045.82) | 207892.57 (135318.68 to 308355.65) | 185397.56 (120653.64 to 275361.80) | 165444.78 (107405.10 to 246774.28) | 145188.46 (93448.25 to 218750.66) | 116623.41 (74691.25 to 176424.52) | 86384.55 (55056.15 to 131146.21) | 57231.33 (36266.71 to 86623.69) | 29487.10 (18644.13 to 44669.65) | 10354.87 (6556.12 to 15671.31) | 3307.11 (2096.14 to 4975.34) | 865.58 (545.63 to 1314.76) | 171.00 (107.55 to 261.40) |
| Eastern Sub-Saharan Africa | Both | Number of DALYs | 3836.53 (2582.61 to 5477.33) | 32397.75 (20939.37 to 48800.00) | 40432.45 (26960.48 to 58577.97) | 43796.46 (29510.10 to 62088.85) | 40851.97 (27321.30 to 57117.19) | 37455.87 (25125.43 to 52697.56) | 33361.41 (22682.54 to 46588.26) | 29431.63 (19931.68 to 41012.47) | 24325.15 (16433.37 to 34203.32) | 21307.40 (14617.61 to 29939.46) | 18531.21 (12656.02 to 26167.21) | 15662.87 (10768.00 to 21942.06) | 13173.56 (9093.96 to 18426.10) | 10295.37 (7120.09 to 14399.36) | 6991.38 (4878.19 to 9623.46) | 3891.49 (2715.98 to 5317.22) | 1763.88 (1242.38 to 2398.39) | 618.54 (438.23 to 837.47) | 137.05 (97.94 to 185.86) | 23.62 (16.97 to 31.94) |
| Eastern Sub-Saharan Africa | Both | Number of prevalence | 647812.45 (433871.42 to 931858.72) | 1589682.31 (1146308.84 to 2191458.06) | 1980197.02 (1425608.12 to 2721362.67) | 2190016.41 (1564710.36 to 3011200.75) | 2143349.15 (1520947.24 to 2956907.59) | 2005560.84 (1424690.99 to 2769239.37) | 1768858.65 (1264566.62 to 2420075.30) | 1550278.14 (1111605.59 to 2114963.93) | 1267706.98 (911067.95 to 1709877.30) | 1101954.73 (793895.79 to 1488067.21) | 952636.48 (688196.58 to 1283035.98) | 799798.17 (579454.50 to 1078078.48) | 667355.68 (484773.06 to 898713.53) | 522976.47 (380153.49 to 707896.28) | 360210.23 (260462.36 to 491099.31) | 208016.68 (150730.06 to 281904.49) | 98165.46 (70853.19 to 132116.94) | 35968.69 (25841.39 to 48361.29) | 8449.18 (6093.80 to 11075.50) | 1616.38 (1150.84 to 2187.76) |
| Eastern Sub-Saharan Africa | Female | Number of DALYs | 2224.35 (1477.83 to 3163.08) | 3831.30 (2623.37 to 5469.04) | 4285.67 (2888.19 to 6128.29) | 5034.15 (3387.22 to 7346.59) | 5733.84 (3842.73 to 8264.76) | 6073.12 (4042.76 to 8865.70) | 5972.60 (3986.67 to 8617.03) | 5774.22 (3792.26 to 8485.10) | 5073.65 (3356.61 to 7418.51) | 4533.43 (3017.49 to 6616.21) | 4117.09 (2706.90 to 5974.25) | 3481.51 (2290.89 to 5021.03) | 2999.04 (1974.35 to 4318.80) | 2373.21 (1586.33 to 3403.57) | 1605.99 (1063.76 to 2295.46) | 925.92 (621.39 to 1300.49) | 452.67 (305.54 to 636.97) | 169.94 (115.92 to 240.90) | 43.07 (29.29 to 59.97) | 9.00 (6.14 to 12.45) |
| Eastern Sub-Saharan Africa | Female | Number of prevalence | 324079.41 (218575.00 to 464043.23) | 690166.92 (462027.99 to 982048.86) | 858529.64 (576128.14 to 1223913.99) | 986399.27 (667633.38 to 1397329.25) | 1018314.68 (684614.96 to 1452956.13) | 965561.09 (651369.91 to 1372939.52) | 840992.05 (573414.77 to 1178185.19) | 736558.67 (506705.64 to 1031884.43) | 596855.61 (413270.82 to 826848.87) | 513067.70 (355174.75 to 709917.09) | 443146.37 (308514.04 to 610398.30) | 366639.54 (255607.44 to 505223.90) | 303814.21 (212048.69 to 419807.30) | 236385.30 (165438.69 to 326521.60) | 162646.78 (113798.75 to 223954.87) | 96656.39 (67201.27 to 132929.14) | 47818.93 (33421.86 to 65670.80) | 18349.62 (12772.55 to 25274.01) | 4676.67 (3275.11 to 6294.11) | 1019.77 (701.28 to 1383.94) |
| Eastern Sub-Saharan Africa | Male | Number of DALYs | 1612.19 (1058.20 to 2308.07) | 28566.45 (17668.29 to 43927.20) | 36146.78 (23777.23 to 52784.92) | 38762.31 (26017.24 to 55194.34) | 35118.13 (23264.79 to 49405.64) | 31382.75 (20978.67 to 44059.74) | 27388.80 (18519.55 to 38394.50) | 23657.42 (15978.60 to 32968.05) | 19251.49 (13005.89 to 27003.18) | 16773.97 (11350.03 to 23256.08) | 14414.12 (9771.26 to 20070.92) | 12181.36 (8317.09 to 16769.31) | 10174.53 (7004.74 to 14020.57) | 7922.17 (5473.84 to 10951.39) | 5385.39 (3720.30 to 7290.54) | 2965.57 (2061.36 to 4002.77) | 1311.21 (912.19 to 1767.70) | 448.60 (318.51 to 601.48) | 93.98 (66.91 to 124.60) | 14.62 (10.46 to 19.24) |
| Eastern Sub-Saharan Africa | Male | Number of prevalence | 323733.04 (214694.01 to 467451.36) | 899515.39 (672689.04 to 1208897.46) | 1121667.38 (842389.83 to 1496750.44) | 1203617.14 (896294.31 to 1594193.04) | 1125034.47 (834697.96 to 1509726.73) | 1039999.75 (771160.90 to 1396924.54) | 927866.60 (692376.69 to 1244531.83) | 813719.47 (605454.95 to 1090071.57) | 670851.36 (498681.76 to 886221.63) | 588887.03 (438338.86 to 777064.80) | 509490.11 (380749.98 to 670530.33) | 433158.63 (324434.43 to 570989.22) | 363541.48 (273139.83 to 478757.11) | 286591.17 (214678.84 to 378981.62) | 197563.45 (147245.23 to 263432.22) | 111360.29 (83296.23 to 149298.92) | 50346.53 (37541.01 to 66390.67) | 17619.07 (13178.49 to 23233.75) | 3772.52 (2836.42 to 4878.72) | 596.61 (440.55 to 788.79) |
| High-income Asia Pacific | Both | Number of DALYs | 3.37 (2.14 to 5.19) | 9.33 (5.00 to 17.25) | 8.77 (4.58 to 16.20) | 8.84 (4.66 to 15.60) | 10.17 (5.54 to 18.66) | 11.57 (6.33 to 21.38) | 11.09 (5.87 to 19.95) | 9.20 (4.77 to 16.97) | 6.48 (3.41 to 11.81) | 4.44 (2.27 to 8.02) | 3.08 (1.69 to 5.58) | 2.31 (1.25 to 4.18) | 1.65 (0.86 to 2.87) | 1.31 (0.70 to 2.32) | 0.85 (0.45 to 1.53) | 0.55 (0.30 to 0.95) | 0.37 (0.20 to 0.66) | 0.20 (0.11 to 0.35) | 0.01 (0.01 to 0.02) | 0.00 (0.00 to 0.00) |
| High-income Asia Pacific | Both | Number of prevalence | 111.76 (32.31 to 745.63) | 253.52 (64.06 to 1612.13) | 291.81 (59.44 to 2007.81) | 354.15 (60.94 to 2557.79) | 444.18 (68.96 to 3284.63) | 531.84 (77.78 to 3975.18) | 526.95 (74.48 to 3959.85) | 446.17 (61.82 to 3362.19) | 317.49 (43.26 to 2398.01) | 224.43 (29.99 to 1704.14) | 161.66 (21.17 to 1232.76) | 125.49 (16.13 to 961.83) | 92.67 (11.75 to 712.27) | 74.15 (9.44 to 568.74) | 49.13 (6.22 to 377.39) | 33.49 (4.19 to 258.49) | 23.02 (2.86 to 177.91) | 12.25 (1.55 to 94.17) | 1.31 (0.13 to 10.73) | 0.17 (0.02 to 1.41) |
| High-income Asia Pacific | Female | Number of DALYs | 1.90 (1.14 to 3.04) | 2.89 (1.80 to 4.61) | 2.52 (1.41 to 3.86) | 2.42 (1.47 to 3.81) | 2.57 (1.52 to 4.11) | 2.68 (1.55 to 4.13) | 2.42 (1.37 to 3.71) | 1.88 (1.13 to 2.79) | 1.27 (0.71 to 1.95) | 0.90 (0.51 to 1.35) | 0.64 (0.38 to 0.99) | 0.50 (0.30 to 0.78) | 0.36 (0.21 to 0.54) | 0.26 (0.16 to 0.40) | 0.18 (0.11 to 0.27) | 0.13 (0.08 to 0.19) | 0.09 (0.05 to 0.13) | 0.04 (0.03 to 0.06) | 0.01 (0.01 to 0.01) | 0.00 (0.00 to 0.00) |
| High-income Asia Pacific | Female | Number of prevalence | 56.21 (17.89 to 358.93) | 110.27 (27.06 to 771.91) | 128.57 (24.08 to 963.33) | 157.22 (23.35 to 1229.11) | 195.12 (24.91 to 1559.68) | 226.84 (26.36 to 1834.90) | 219.00 (23.99 to 1783.60) | 179.14 (18.77 to 1466.22) | 124.42 (12.57 to 1022.13) | 91.40 (8.97 to 753.27) | 67.94 (6.47 to 561.39) | 55.07 (5.12 to 456.24) | 40.98 (3.70 to 340.34) | 31.18 (2.78 to 259.28) | 21.14 (1.88 to 175.78) | 15.50 (1.38 to 128.92) | 10.83 (0.96 to 90.10) | 5.35 (0.48 to 44.53) | 1.15 (0.10 to 9.54) | 0.17 (0.01 to 1.38) |
| High-income Asia Pacific | Male | Number of DALYs | 1.47 (0.79 to 2.37) | 6.44 (2.87 to 13.00) | 6.26 (2.72 to 13.10) | 6.42 (2.83 to 12.88) | 7.60 (3.38 to 15.45) | 8.89 (4.27 to 18.02) | 8.67 (3.97 to 16.83) | 7.32 (3.35 to 14.61) | 5.20 (2.41 to 10.28) | 3.54 (1.61 to 7.01) | 2.44 (1.20 to 4.78) | 1.80 (0.83 to 3.52) | 1.29 (0.62 to 2.38) | 1.05 (0.51 to 2.04) | 0.67 (0.31 to 1.30) | 0.42 (0.21 to 0.78) | 0.28 (0.13 to 0.55) | 0.16 (0.08 to 0.30) | 0.00 (0.00 to 0.01) | 0.00 (0.00 to 0.00) |
| High-income Asia Pacific | Male | Number of prevalence | 55.55 (13.92 to 386.70) | 143.25 (36.04 to 843.80) | 163.23 (34.28 to 1044.48) | 196.93 (36.41 to 1328.68) | 249.06 (42.74 to 1724.95) | 305.00 (50.36 to 2140.28) | 307.95 (49.35 to 2176.25) | 267.03 (42.60 to 1895.98) | 193.08 (30.38 to 1375.88) | 133.03 (20.73 to 950.87) | 93.71 (14.37 to 671.38) | 70.41 (10.78 to 505.59) | 51.69 (7.87 to 371.92) | 42.97 (6.51 to 309.47) | 27.99 (4.24 to 201.61) | 17.99 (2.73 to 129.57) | 12.19 (1.85 to 87.81) | 6.89 (1.04 to 49.64) | 0.17 (0.02 to 1.19) | 0.00 (0.00 to 0.03) |
| North Africa and Middle East | Both | Number of DALYs | 1433.60 (951.48 to 2081.31) | 4441.38 (2450.60 to 8132.22) | 4594.47 (2516.75 to 8218.58) | 4647.13 (2583.22 to 8380.02) | 4538.54 (2479.62 to 7772.52) | 4312.27 (2467.96 to 7385.63) | 3905.49 (2234.57 to 6550.26) | 3570.22 (2040.03 to 6007.93) | 3143.16 (1787.44 to 5261.61) | 2670.80 (1546.51 to 4403.48) | 2280.09 (1340.31 to 3758.96) | 1878.77 (1076.68 to 3034.74) | 1584.23 (915.37 to 2568.82) | 1203.85 (713.40 to 1929.90) | 814.61 (491.14 to 1301.95) | 458.39 (278.73 to 721.94) | 212.51 (127.02 to 331.40) | 79.38 (47.25 to 126.30) | 19.90 (12.11 to 31.75) | 3.19 (1.94 to 5.01) |
| North Africa and Middle East | Both | Number of prevalence | 76328.14 (22547.50 to 236708.24) | 173705.31 (55543.47 to 530098.99) | 214721.19 (62368.62 to 667370.80) | 245490.13 (67398.89 to 779755.14) | 251769.84 (67765.49 to 812023.96) | 244463.78 (65084.13 to 794495.60) | 222530.30 (59191.57 to 725482.16) | 203726.48 (54132.86 to 671573.54) | 176785.92 (47404.73 to 582778.68) | 151893.94 (40359.43 to 502839.12) | 128403.12 (34258.12 to 427525.39) | 105620.18 (28305.28 to 352249.53) | 88654.86 (23953.77 to 296245.78) | 68011.53 (18595.22 to 220289.04) | 46476.69 (12867.06 to 153050.44) | 26264.25 (7393.94 to 84112.83) | 12665.49 (3474.32 to 42000.13) | 4833.80 (1320.48 to 15438.62) | 1138.79 (326.43 to 3609.61) | 188.70 (53.91 to 586.37) |
| North Africa and Middle East | Female | Number of DALYs | 818.94 (517.00 to 1272.65) | 1220.70 (771.52 to 1808.69) | 1106.64 (674.60 to 1627.05) | 985.36 (606.58 to 1446.64) | 880.75 (556.31 to 1311.32) | 779.59 (505.61 to 1119.89) | 668.95 (445.72 to 966.78) | 588.94 (381.40 to 872.34) | 471.30 (299.30 to 707.90) | 400.44 (254.33 to 584.78) | 323.28 (209.80 to 458.73) | 257.81 (167.36 to 377.80) | 206.08 (134.35 to 303.08) | 155.49 (101.35 to 230.39) | 102.60 (67.81 to 147.71) | 54.87 (36.87 to 76.97) | 27.22 (18.22 to 39.22) | 10.09 (6.85 to 14.38) | 1.98 (1.35 to 2.78) | 0.33 (0.22 to 0.45) |
| North Africa and Middle East | Female | Number of prevalence | 38101.84 (12001.22 to 116338.63) | 76984.20 (20692.85 to 243181.65) | 94772.87 (22071.27 to 310969.70) | 109281.17 (23130.28 to 365978.41) | 114524.35 (22986.50 to 387208.82) | 111870.46 (21934.59 to 385405.37) | 101650.93 (19638.48 to 352190.94) | 93060.73 (17532.99 to 324188.32) | 78062.50 (14322.62 to 276099.85) | 68270.24 (12422.64 to 241599.51) | 56710.04 (10220.83 to 203450.13) | 46187.42 (8291.19 to 167855.05) | 37907.06 (6707.91 to 139814.80) | 29209.02 (5218.87 to 104932.21) | 19860.20 (3577.56 to 71122.88) | 10858.40 (1958.00 to 36472.38) | 5416.88 (973.84 to 19056.71) | 2061.03 (375.37 to 7336.28) | 409.49 (73.32 to 1376.83) | 70.09 (12.25 to 244.45) |
| North Africa and Middle East | Male | Number of DALYs | 614.66 (381.38 to 880.15) | 3220.67 (1544.13 to 6704.88) | 3487.82 (1693.42 to 6994.91) | 3661.77 (1842.73 to 7072.16) | 3657.79 (1803.05 to 6692.87) | 3532.68 (1829.91 to 6512.36) | 3236.54 (1722.21 to 5637.14) | 2981.27 (1560.19 to 5292.71) | 2671.86 (1409.92 to 4658.00) | 2270.37 (1241.67 to 3883.42) | 1956.82 (1089.46 to 3371.63) | 1620.96 (893.23 to 2699.49) | 1378.15 (758.46 to 2296.02) | 1048.36 (590.75 to 1734.40) | 712.01 (412.36 to 1170.40) | 403.52 (232.98 to 655.19) | 185.28 (106.48 to 303.17) | 69.29 (39.50 to 114.44) | 17.92 (10.29 to 29.30) | 2.86 (1.67 to 4.64) |
| North Africa and Middle East | Male | Number of prevalence | 38226.31 (10465.28 to 120369.61) | 96721.12 (33194.04 to 285225.73) | 119948.32 (38500.44 to 360584.87) | 136208.96 (41901.99 to 412794.46) | 137245.50 (42076.54 to 422562.03) | 132593.32 (40936.46 to 413089.29) | 120879.36 (37594.52 to 376342.78) | 110665.75 (34639.38 to 347474.92) | 98723.41 (31137.62 to 306802.73) | 83623.71 (26643.70 to 261303.02) | 71693.08 (22884.42 to 224210.45) | 59432.76 (19138.99 to 184976.39) | 50747.80 (16515.24 to 157433.19) | 38802.51 (12785.49 to 116041.93) | 26616.48 (8803.35 to 80769.59) | 15405.85 (5052.79 to 47905.60) | 7248.61 (2353.05 to 21984.29) | 2772.78 (885.57 to 8377.11) | 729.30 (231.71 to 2205.05) | 118.60 (37.74 to 356.89) |
| Oceania | Both | Number of DALYs | 168.38 (111.01 to 246.15) | 3008.34 (2001.07 to 4201.36) | 3291.22 (2244.07 to 4568.95) | 3247.42 (2211.57 to 4498.74) | 3321.74 (2259.73 to 4572.47) | 3320.54 (2266.74 to 4707.49) | 3026.76 (2063.92 to 4255.87) | 2687.79 (1835.35 to 3846.65) | 2386.53 (1620.26 to 3376.87) | 2049.22 (1393.75 to 2906.14) | 1759.29 (1195.62 to 2518.44) | 1510.34 (1024.50 to 2159.00) | 1337.12 (922.69 to 1874.23) | 979.60 (670.82 to 1369.57) | 582.65 (403.99 to 808.70) | 275.41 (190.53 to 384.20) | 111.12 (76.64 to 152.96) | 34.25 (23.89 to 46.69) | 6.51 (4.54 to 8.84) | 1.90 (1.32 to 2.57) |
| Oceania | Both | Number of prevalence | 46314.88 (20613.92 to 81216.24) | 124557.85 (66833.74 to 203307.04) | 156255.57 (82168.75 to 255775.18) | 179228.90 (92098.57 to 295360.42) | 196148.88 (98859.39 to 324824.11) | 196807.78 (99664.79 to 325380.01) | 175936.54 (89683.52 to 289854.91) | 152845.74 (78802.61 to 251262.07) | 132436.92 (68682.72 to 215698.72) | 111151.98 (58390.89 to 177759.31) | 93090.70 (49279.85 to 145313.31) | 77912.16 (41289.87 to 118901.20) | 67216.71 (35971.91 to 99502.77) | 49207.95 (26713.53 to 71617.56) | 29981.26 (16359.40 to 43586.64) | 14580.34 (8144.22 to 21217.21) | 6075.83 (3429.82 to 8939.89) | 1971.24 (1138.73 to 2900.75) | 411.02 (238.00 to 607.56) | 128.98 (72.70 to 193.97) |
| Oceania | Female | Number of DALYs | 95.07 (60.73 to 138.48) | 201.61 (133.31 to 298.09) | 286.10 (186.88 to 431.96) | 434.00 (278.38 to 628.71) | 627.96 (397.41 to 911.86) | 767.01 (501.76 to 1122.66) | 775.99 (494.85 to 1142.72) | 740.03 (482.07 to 1085.13) | 697.01 (448.38 to 1015.48) | 619.15 (402.18 to 899.91) | 547.39 (352.11 to 809.35) | 485.92 (309.07 to 717.03) | 459.62 (297.48 to 666.25) | 342.52 (223.31 to 499.52) | 203.34 (133.06 to 297.53) | 96.03 (63.46 to 136.56) | 38.08 (25.35 to 54.29) | 12.53 (8.40 to 17.77) | 2.80 (1.89 to 4.01) | 1.07 (0.72 to 1.52) |
| Oceania | Female | Number of prevalence | 22325.05 (10036.19 to 39001.03) | 49264.41 (22403.70 to 85698.29) | 63865.11 (29104.17 to 110751.50) | 77272.92 (35567.39 to 133352.07) | 88137.00 (40490.74 to 151177.54) | 89959.77 (42116.70 to 153123.67) | 80534.37 (38381.70 to 136058.51) | 69827.26 (33793.35 to 117393.47) | 60427.73 (29513.93 to 101330.43) | 50440.23 (25008.47 to 83461.14) | 41956.75 (21045.34 to 67695.87) | 35075.48 (17801.82 to 55263.65) | 30995.24 (15861.94 to 47473.79) | 22821.04 (11852.43 to 34280.81) | 13923.08 (7279.06 to 20829.70) | 6825.99 (3671.28 to 10179.52) | 2851.92 (1573.81 to 4264.77) | 995.26 (559.25 to 1503.96) | 243.58 (133.24 to 378.97) | 90.62 (49.37 to 141.18) |
| Oceania | Male | Number of DALYs | 73.31 (46.35 to 110.73) | 2806.73 (1867.20 to 3908.47) | 3005.12 (2030.65 to 4199.17) | 2813.42 (1909.19 to 3892.97) | 2693.79 (1836.30 to 3775.17) | 2553.53 (1759.65 to 3556.32) | 2250.77 (1559.27 to 3065.94) | 1947.76 (1334.44 to 2748.65) | 1689.52 (1172.72 to 2352.62) | 1430.07 (988.86 to 1990.55) | 1211.89 (833.95 to 1716.38) | 1024.41 (715.37 to 1473.20) | 877.49 (610.34 to 1232.94) | 637.08 (448.56 to 900.22) | 379.31 (265.64 to 536.42) | 179.38 (125.64 to 251.14) | 73.04 (51.69 to 100.67) | 21.72 (15.52 to 29.83) | 3.70 (2.63 to 5.09) | 0.83 (0.60 to 1.13) |
| Oceania | Male | Number of prevalence | 23989.82 (10577.73 to 42215.21) | 75293.44 (44608.59 to 116574.41) | 92390.45 (53162.24 to 145023.68) | 101955.99 (56531.19 to 162008.35) | 108011.88 (58479.80 to 173646.57) | 106848.01 (57507.64 to 172256.34) | 95402.17 (51301.83 to 153820.48) | 83018.48 (44798.91 to 133487.37) | 72009.19 (38877.28 to 114827.34) | 60711.75 (33052.27 to 94432.95) | 51133.95 (27947.44 to 77677.12) | 42836.68 (23558.49 to 63597.28) | 36221.48 (20161.25 to 52094.92) | 26386.91 (14859.07 to 37451.77) | 16058.18 (9069.53 to 22884.67) | 7754.35 (4459.33 to 11091.42) | 3223.91 (1878.55 to 4654.45) | 975.98 (575.00 to 1398.51) | 167.43 (96.51 to 235.20) | 38.36 (21.33 to 54.08) |
| South Asia | Both | Number of DALYs | 16764.72 (11508.55 to 23480.33) | 147809.90 (96886.49 to 220203.48) | 163442.07 (107691.70 to 239000.82) | 181058.80 (121318.36 to 259088.41) | 196967.31 (131517.21 to 282380.02) | 193482.64 (129871.55 to 274694.96) | 182592.64 (124026.45 to 257829.08) | 178542.36 (121308.68 to 251209.65) | 156710.49 (107359.34 to 218335.00) | 138560.19 (94881.20 to 193595.19) | 119230.34 (81951.56 to 165538.64) | 105273.79 (72014.78 to 147207.60) | 89002.65 (61251.21 to 123702.66) | 60553.44 (41776.72 to 83306.32) | 36926.96 (25656.03 to 50751.73) | 21834.99 (15206.56 to 29694.24) | 12181.86 (8593.87 to 16525.07) | 4040.02 (2865.93 to 5391.57) | 901.24 (643.39 to 1205.79) | 170.01 (121.89 to 226.54) |
| South Asia | Both | Number of prevalence | 2526486.72 (2327634.11 to 2730566.94) | 6776765.95 (6229571.75 to 7380815.52) | 8085862.32 (7478556.35 to 8732598.08) | 9350032.28 (8675305.51 to 10078712.39) | 10270715.07 (9558195.97 to 11060792.88) | 10089330.61 (9409223.12 to 10872057.72) | 9453681.30 (8823255.53 to 10176303.82) | 9145881.70 (8530897.60 to 9832555.83) | 7944502.62 (7408050.01 to 8530169.70) | 6939758.00 (6470106.68 to 7452392.43) | 5951750.00 (5546476.15 to 6387665.78) | 5260637.17 (4901107.02 to 5646124.51) | 4425722.48 (4124181.45 to 4747575.13) | 3069432.27 (2856253.42 to 3295341.55) | 1939872.22 (1804381.03 to 2085768.59) | 1183830.57 (1100347.95 to 1271623.35) | 681290.88 (632968.11 to 730574.71) | 231883.26 (215155.91 to 249698.03) | 53482.39 (49514.62 to 57820.92) | 10508.95 (9682.66 to 11447.36) |
| South Asia | Female | Number of DALYs | 9473.28 (6462.03 to 13379.39) | 17573.73 (11874.06 to 24880.81) | 18648.09 (12659.82 to 26986.09) | 21607.82 (14496.03 to 31514.61) | 26169.03 (17603.85 to 38024.70) | 28597.91 (19163.49 to 41569.00) | 28915.74 (19236.89 to 41862.18) | 30135.58 (19915.60 to 44367.66) | 27258.35 (18005.50 to 39980.83) | 24616.98 (16321.82 to 35923.16) | 22071.22 (14637.48 to 31899.13) | 20321.71 (13314.11 to 29255.97) | 17695.63 (11761.51 to 25103.54) | 12809.46 (8589.05 to 18368.97) | 8173.49 (5384.09 to 11564.40) | 4978.95 (3331.82 to 7097.37) | 2956.35 (1985.86 to 4132.58) | 1008.15 (688.97 to 1413.01) | 229.15 (156.51 to 319.12) | 45.60 (31.26 to 63.12) |
| South Asia | Female | Number of prevalence | 1221194.72 (1126374.39 to 1316885.57) | 2790802.93 (2570927.05 to 3013413.37) | 3348231.55 (3081879.65 to 3615681.64) | 3891000.49 (3586037.39 to 4199547.87) | 4348351.46 (4013753.15 to 4690252.56) | 4355857.97 (4022756.09 to 4698603.61) | 4079554.43 (3766321.82 to 4402486.90) | 3939770.25 (3635711.28 to 4247398.34) | 3371683.91 (3110703.54 to 3632014.44) | 2886862.54 (2664631.95 to 3107832.96) | 2470660.42 (2280316.44 to 2660022.97) | 2185878.12 (2019194.83 to 2352959.32) | 1822199.06 (1683572.13 to 1959825.40) | 1305483.99 (1206534.22 to 1404088.35) | 858185.29 (792815.54 to 922914.10) | 529599.52 (489333.62 to 569175.99) | 311345.06 (287689.79 to 334551.89) | 108886.86 (100569.44 to 116969.69) | 26015.96 (23999.05 to 28064.37) | 5330.29 (4890.15 to 5770.08) |
| South Asia | Male | Number of DALYs | 7291.44 (4959.01 to 10462.77) | 130236.17 (84667.50 to 197008.91) | 144793.98 (94848.27 to 213944.25) | 159450.98 (106205.02 to 231961.33) | 170798.28 (113026.41 to 245574.18) | 164884.72 (110197.05 to 233750.02) | 153676.90 (102070.62 to 218844.78) | 148406.78 (99346.07 to 208727.39) | 129452.14 (87851.97 to 183151.75) | 113943.21 (76916.88 to 159091.35) | 97159.12 (66510.71 to 134948.82) | 84952.08 (58236.43 to 117959.50) | 71307.02 (49039.62 to 98486.75) | 47743.98 (33101.72 to 65398.69) | 28753.48 (20101.90 to 39323.84) | 16856.05 (11860.78 to 22870.71) | 9225.52 (6576.76 to 12473.97) | 3031.87 (2151.84 to 4071.41) | 672.09 (477.26 to 903.87) | 124.42 (89.01 to 166.36) |
| South Asia | Male | Number of prevalence | 1305292.00 (1201255.09 to 1414681.55) | 3985963.02 (3633917.62 to 4403259.93) | 4737630.76 (4343640.55 to 5175460.65) | 5459031.78 (5040065.24 to 5916213.37) | 5922363.61 (5486071.53 to 6384482.13) | 5733472.64 (5329106.87 to 6169940.82) | 5374126.87 (5004665.99 to 5779704.20) | 5206111.45 (4854151.79 to 5588659.52) | 4572818.71 (4263556.60 to 4904667.35) | 4052895.47 (3777300.22 to 4345631.11) | 3481089.58 (3241407.67 to 3727545.14) | 3074759.05 (2864427.68 to 3290056.80) | 2603523.43 (2428248.46 to 2785031.58) | 1763948.28 (1642802.62 to 1889332.53) | 1081686.94 (1006645.89 to 1158576.46) | 654231.05 (609779.91 to 699358.26) | 369945.82 (344697.80 to 395735.36) | 122996.40 (114570.00 to 132247.32) | 27466.43 (25524.51 to 29636.70) | 5178.66 (4766.69 to 5642.72) |
| Southeast Asia | Both | Number of DALYs | 6822.33 (4699.61 to 9701.42) | 50238.03 (28996.80 to 82465.01) | 75213.62 (46803.01 to 115607.00) | 97382.47 (63724.61 to 141334.34) | 106728.14 (70698.38 to 153178.60) | 107968.89 (71999.75 to 152919.39) | 101681.83 (67671.85 to 141883.38) | 87185.42 (57992.03 to 121759.79) | 67704.37 (45086.15 to 94774.52) | 57239.15 (38471.97 to 79744.82) | 51016.23 (34418.54 to 70727.97) | 43308.23 (29156.66 to 59579.47) | 35122.36 (23830.25 to 47633.87) | 24690.77 (16811.52 to 33494.67) | 15398.37 (10659.71 to 20782.78) | 8822.72 (6068.05 to 11891.30) | 4753.54 (3307.10 to 6430.38) | 2042.33 (1439.48 to 2752.74) | 472.99 (335.39 to 633.20) | 91.93 (65.43 to 121.28) |
| Southeast Asia | Both | Number of prevalence | 967335.01 (477059.03 to 1750411.10) | 2649167.51 (1341757.64 to 4644917.74) | 3661333.90 (1903770.64 to 6399343.94) | 4485969.15 (2374041.61 to 7797205.99) | 4906795.73 (2621691.64 to 8493013.06) | 4987849.75 (2700693.23 to 8697741.02) | 4675712.50 (2530316.97 to 8036027.88) | 4026732.96 (2165089.36 to 6870381.90) | 3187742.62 (1696088.85 to 5554120.41) | 2767364.94 (1464727.71 to 4891284.30) | 2511991.76 (1325131.16 to 4468331.29) | 2172134.99 (1164734.15 to 3818328.01) | 1801868.70 (962260.14 to 3171351.84) | 1314096.06 (699995.25 to 2311939.19) | 854365.02 (456451.92 to 1491653.54) | 510438.48 (274549.40 to 892302.98) | 287407.05 (154960.11 to 505620.85) | 128742.19 (67222.05 to 232381.66) | 34072.22 (16515.59 to 67231.16) | 8441.94 (3773.69 to 16856.55) |
| Southeast Asia | Female | Number of DALYs | 3917.03 (2675.26 to 5626.58) | 7321.15 (4921.80 to 10545.10) | 8009.04 (5353.89 to 11544.82) | 8729.98 (5855.68 to 12290.69) | 9335.02 (6187.42 to 13395.91) | 9430.36 (6363.05 to 13666.15) | 8655.94 (5851.38 to 12405.35) | 7391.00 (5064.62 to 10550.23) | 5927.37 (4045.11 to 8475.89) | 5235.33 (3587.49 to 7579.64) | 4784.64 (3218.86 to 6798.88) | 4155.93 (2799.56 to 6003.90) | 3461.21 (2341.08 to 4955.24) | 2543.87 (1744.68 to 3602.30) | 1680.37 (1160.86 to 2358.18) | 1004.34 (689.19 to 1408.82) | 571.29 (401.79 to 806.71) | 257.13 (180.22 to 355.46) | 73.88 (53.22 to 101.81) | 20.68 (14.59 to 27.87) |
| Southeast Asia | Female | Number of prevalence | 477793.74 (237395.29 to 859217.55) | 1155935.15 (562644.44 to 2129904.32) | 1561736.51 (738792.23 to 2889268.11) | 1925994.79 (895644.25 to 3580029.07) | 2152261.18 (990897.42 to 4016068.04) | 2201051.48 (1026635.64 to 4120958.27) | 2021597.90 (946293.11 to 3734411.32) | 1723139.47 (809198.30 to 3149727.53) | 1373619.83 (635556.68 to 2578260.74) | 1219427.65 (558305.73 to 2325194.44) | 1116664.12 (513237.05 to 2146808.81) | 972088.57 (449832.03 to 1843473.27) | 809274.09 (372465.80 to 1530297.91) | 604341.03 (278402.08 to 1141369.94) | 406225.99 (188485.80 to 760653.34) | 251034.51 (116169.49 to 470234.07) | 146370.76 (67350.96 to 279034.25) | 67417.79 (29749.70 to 135253.37) | 20095.26 (8548.28 to 43128.14) | 5898.49 (2245.88 to 12780.45) |
| Southeast Asia | Male | Number of DALYs | 2905.30 (1931.82 to 4124.50) | 42916.88 (23409.16 to 73087.83) | 67204.58 (40715.27 to 106418.98) | 88652.49 (57127.57 to 130186.86) | 97393.12 (63740.98 to 140401.88) | 98538.53 (65458.49 to 140756.75) | 93025.89 (61626.96 to 130236.66) | 79794.42 (53035.93 to 112113.15) | 61777.00 (41003.04 to 86978.52) | 52003.82 (34835.86 to 72925.72) | 46231.59 (31160.70 to 64337.20) | 39152.30 (26491.46 to 53900.89) | 31661.15 (21520.40 to 43380.33) | 22146.90 (14990.76 to 30283.33) | 13718.00 (9367.83 to 18580.96) | 7818.38 (5323.14 to 10622.49) | 4182.24 (2897.12 to 5657.46) | 1785.21 (1241.17 to 2403.30) | 399.11 (279.34 to 536.69) | 71.25 (50.38 to 94.87) |
| Southeast Asia | Male | Number of prevalence | 489541.27 (238144.92 to 891193.54) | 1493232.36 (806124.64 to 2509163.07) | 2099597.38 (1175668.24 to 3482302.52) | 2559974.36 (1490484.19 to 4209666.77) | 2754534.54 (1627799.27 to 4501658.35) | 2786798.28 (1660074.94 to 4564903.32) | 2654114.60 (1578958.47 to 4307925.15) | 2303593.49 (1360091.60 to 3720654.37) | 1814122.79 (1075333.48 to 2975859.67) | 1547937.29 (915146.24 to 2566296.95) | 1395327.64 (824502.64 to 2323322.66) | 1200046.42 (707310.95 to 1988098.83) | 992594.61 (584473.35 to 1643373.82) | 709755.03 (417820.84 to 1172809.61) | 448139.04 (265925.30 to 740065.97) | 259403.97 (155805.24 to 424078.40) | 141036.30 (84323.71 to 230825.45) | 61324.39 (35507.29 to 101283.59) | 13976.96 (7913.18 to 23840.68) | 2543.46 (1455.01 to 4434.52) |
| Southern Sub-Saharan Africa | Both | Number of DALYs | 169.68 (103.00 to 268.89) | 489.05 (266.91 to 886.91) | 465.82 (250.36 to 819.87) | 424.13 (226.43 to 755.18) | 349.04 (189.70 to 629.29) | 278.54 (148.01 to 495.47) | 230.95 (123.20 to 422.15) | 199.50 (106.95 to 352.68) | 149.35 (82.15 to 262.99) | 125.38 (67.20 to 221.69) | 111.81 (61.40 to 204.27) | 88.45 (45.83 to 164.58) | 74.84 (41.21 to 133.45) | 60.74 (34.03 to 106.04) | 41.42 (21.89 to 72.26) | 20.65 (11.46 to 35.62) | 8.45 (4.90 to 14.47) | 3.19 (1.88 to 5.21) | 0.79 (0.47 to 1.21) | 0.15 (0.09 to 0.23) |
| Southern Sub-Saharan Africa | Both | Number of prevalence | 5633.57 (2061.10 to 18932.75) | 13584.92 (4797.92 to 44349.40) | 16040.39 (4923.77 to 55773.52) | 17309.95 (4774.72 to 62629.98) | 16183.41 (4186.32 to 59862.96) | 13931.97 (3442.95 to 52157.83) | 12086.63 (2928.06 to 45504.97) | 10590.59 (2530.39 to 39998.35) | 8035.79 (1901.19 to 30430.64) | 6880.96 (1627.59 to 26067.66) | 6203.36 (1457.85 to 23535.80) | 4855.70 (1149.49 to 18401.18) | 4192.43 (989.76 to 15911.35) | 3567.65 (825.18 to 13604.12) | 2498.49 (575.07 to 9538.58) | 1322.54 (301.99 to 5073.22) | 599.87 (134.41 to 2321.44) | 249.93 (55.03 to 973.79) | 67.66 (14.49 to 265.76) | 14.64 (3.04 to 57.95) |
| Southern Sub-Saharan Africa | Female | Number of DALYs | 98.86 (59.09 to 153.84) | 159.49 (96.06 to 246.67) | 141.89 (83.68 to 217.74) | 124.60 (73.06 to 191.60) | 100.93 (59.82 to 152.29) | 80.70 (46.66 to 123.03) | 64.03 (35.67 to 98.39) | 52.96 (32.33 to 81.93) | 38.53 (23.30 to 57.52) | 30.35 (17.73 to 46.88) | 26.48 (15.35 to 40.95) | 19.08 (11.94 to 28.87) | 15.77 (9.66 to 24.33) | 14.01 (8.21 to 20.87) | 9.97 (6.03 to 14.71) | 5.63 (3.48 to 8.71) | 2.83 (1.68 to 4.33) | 1.26 (0.73 to 1.87) | 0.38 (0.23 to 0.58) | 0.09 (0.05 to 0.13) |
| Southern Sub-Saharan Africa | Female | Number of prevalence | 2948.74 (1162.39 to 9594.25) | 6160.55 (2017.45 to 21612.87) | 7370.52 (2020.57 to 27336.19) | 8097.67 (1934.74 to 31106.60) | 7832.51 (1710.82 to 30657.23) | 6927.81 (1435.40 to 27392.88) | 5979.14 (1200.10 to 23782.35) | 5146.56 (1008.45 to 20558.06) | 3885.34 (747.37 to 15567.19) | 3206.67 (607.27 to 12880.38) | 2859.58 (533.31 to 11512.39) | 2114.22 (389.32 to 8529.34) | 1808.32 (329.22 to 7312.14) | 1655.53 (299.36 to 6701.18) | 1187.20 (214.58 to 4805.68) | 683.07 (123.58 to 2764.76) | 356.50 (64.53 to 1442.94) | 163.60 (29.60 to 662.20) | 49.15 (8.87 to 198.93) | 11.62 (2.10 to 47.05) |
| Southern Sub-Saharan Africa | Male | Number of DALYs | 70.82 (36.25 to 117.20) | 329.56 (140.46 to 724.67) | 323.92 (144.28 to 644.09) | 299.53 (132.18 to 608.46) | 248.11 (112.38 to 486.89) | 197.84 (83.36 to 399.98) | 166.92 (75.65 to 330.31) | 146.54 (68.21 to 279.64) | 110.82 (50.30 to 215.12) | 95.02 (45.01 to 182.94) | 85.33 (40.58 to 169.17) | 69.37 (31.96 to 135.94) | 59.07 (28.56 to 116.08) | 46.73 (23.13 to 88.93) | 31.45 (14.73 to 60.48) | 15.02 (7.43 to 28.76) | 5.62 (2.67 to 10.79) | 1.93 (0.88 to 3.67) | 0.41 (0.20 to 0.77) | 0.07 (0.03 to 0.13) |
| Southern Sub-Saharan Africa | Male | Number of prevalence | 2684.84 (887.89 to 9338.49) | 7424.37 (2596.55 to 22502.14) | 8669.87 (2718.01 to 28267.79) | 9212.29 (2677.96 to 31433.92) | 8350.91 (2319.36 to 29164.45) | 7004.16 (1903.06 to 24750.18) | 6107.48 (1636.08 to 21716.99) | 5444.03 (1446.49 to 19440.26) | 4150.45 (1100.37 to 14863.45) | 3674.29 (968.78 to 13187.29) | 3343.78 (878.01 to 12023.42) | 2741.49 (719.28 to 9871.84) | 2384.11 (623.03 to 8599.21) | 1912.12 (499.55 to 6902.95) | 1311.29 (342.10 to 4732.90) | 639.47 (166.86 to 2308.46) | 243.37 (63.58 to 878.50) | 86.33 (22.52 to 311.60) | 18.52 (4.83 to 66.83) | 3.02 (0.79 to 10.90) |
| Tropical Latin America | Both | Number of DALYs | 325.34 (216.35 to 471.60) | 784.33 (499.51 to 1177.55) | 806.33 (507.25 to 1216.65) | 755.25 (469.00 to 1169.86) | 681.62 (418.11 to 1041.60) | 594.21 (372.28 to 919.16) | 510.15 (327.13 to 774.09) | 444.49 (273.30 to 675.10) | 387.30 (239.84 to 577.03) | 327.60 (210.23 to 494.12) | 280.61 (182.76 to 424.29) | 231.70 (148.91 to 352.26) | 205.48 (129.57 to 310.87) | 167.38 (104.26 to 256.35) | 122.97 (78.48 to 187.99) | 84.12 (54.42 to 125.14) | 41.17 (26.70 to 60.97) | 18.82 (11.83 to 28.01) | 4.89 (3.28 to 6.87) | 1.40 (0.95 to 1.95) |
| Tropical Latin America | Both | Number of prevalence | 7602.23 (6017.32 to 12811.55) | 18703.83 (14287.59 to 31653.74) | 23574.13 (17422.80 to 42065.82) | 26446.84 (19130.11 to 48954.00) | 26494.83 (18975.65 to 49967.51) | 24570.31 (17519.91 to 46747.09) | 21746.70 (15459.29 to 41565.93) | 19639.36 (13913.44 to 37694.37) | 17450.42 (12345.65 to 33577.30) | 15133.68 (10687.57 to 29191.95) | 13250.87 (9343.32 to 25631.81) | 11304.82 (7963.33 to 21936.58) | 10240.65 (7208.05 to 19887.94) | 8430.22 (5932.70 to 16375.10) | 6306.55 (4437.88 to 12248.08) | 4272.40 (3015.53 to 8271.51) | 2213.08 (1556.88 to 4301.41) | 1079.17 (755.72 to 2109.13) | 270.54 (190.91 to 524.82) | 60.46 (44.31 to 112.34) |
| Tropical Latin America | Female | Number of DALYs | 189.56 (119.17 to 278.02) | 353.70 (223.53 to 517.40) | 341.43 (222.15 to 503.13) | 294.92 (192.00 to 449.53) | 252.43 (158.39 to 368.50) | 208.46 (136.62 to 304.37) | 173.75 (115.27 to 246.70) | 146.85 (96.30 to 217.02) | 122.39 (80.09 to 180.21) | 100.63 (65.98 to 147.48) | 83.92 (54.86 to 120.86) | 67.66 (44.68 to 99.27) | 57.01 (38.41 to 83.03) | 44.93 (29.85 to 65.12) | 33.42 (22.15 to 47.51) | 24.09 (16.13 to 34.13) | 12.94 (8.67 to 18.34) | 6.13 (4.08 to 8.72) | 2.14 (1.43 to 3.12) | 0.78 (0.52 to 1.14) |
| Tropical Latin America | Female | Number of prevalence | 4030.71 (3245.58 to 6559.45) | 9087.49 (7087.92 to 15710.70) | 11367.89 (8532.49 to 20888.05) | 12788.68 (9279.91 to 24593.18) | 12959.75 (9231.64 to 25529.59) | 12157.47 (8575.18 to 24251.11) | 10849.71 (7613.72 to 21789.37) | 9844.49 (6872.38 to 19900.90) | 8711.98 (6054.21 to 17697.91) | 7534.71 (5221.44 to 15365.42) | 6640.01 (4584.73 to 13593.58) | 5700.12 (3923.44 to 11713.01) | 5076.46 (3486.84 to 10461.97) | 4114.87 (2821.69 to 8495.38) | 3081.04 (2113.84 to 6358.16) | 2098.24 (1445.88 to 4307.33) | 1175.50 (809.15 to 2416.36) | 601.49 (412.64 to 1241.00) | 171.83 (119.66 to 348.21) | 39.11 (28.52 to 74.71) |
| Tropical Latin America | Male | Number of DALYs | 135.79 (85.36 to 204.29) | 430.63 (245.05 to 730.18) | 464.90 (264.80 to 769.91) | 460.33 (262.23 to 789.17) | 429.19 (242.86 to 717.80) | 385.75 (223.84 to 647.26) | 336.40 (194.47 to 567.63) | 297.64 (168.43 to 494.73) | 264.91 (149.95 to 426.24) | 226.97 (131.88 to 370.19) | 196.70 (115.61 to 317.49) | 164.04 (95.81 to 266.62) | 148.47 (86.13 to 244.20) | 122.45 (71.14 to 201.43) | 89.55 (51.24 to 143.10) | 60.03 (35.61 to 96.23) | 28.23 (16.74 to 45.24) | 12.70 (7.51 to 20.67) | 2.74 (1.72 to 4.30) | 0.62 (0.39 to 0.92) |
| Tropical Latin America | Male | Number of prevalence | 3571.52 (2759.51 to 6214.44) | 9616.33 (7170.35 to 16094.08) | 12206.24 (8932.54 to 21460.33) | 13658.16 (9836.77 to 24655.02) | 13535.08 (9711.87 to 24723.59) | 12412.83 (8903.38 to 22762.38) | 10896.98 (7815.80 to 20011.69) | 9794.87 (7025.32 to 18007.60) | 8738.44 (6271.94 to 16069.42) | 7598.97 (5453.15 to 13988.57) | 6610.86 (4745.38 to 12181.08) | 5604.71 (4020.84 to 10343.49) | 5164.19 (3705.96 to 9529.87) | 4315.35 (3100.06 to 7962.88) | 3225.51 (2315.30 to 5953.34) | 2174.16 (1563.49 to 4007.64) | 1037.58 (746.88 to 1909.03) | 477.68 (343.33 to 880.21) | 98.70 (71.82 to 179.43) | 21.35 (15.72 to 38.22) |
| Western Sub-Saharan Africa | Both | Number of DALYs | 4328.50 (2948.84 to 6151.07) | 43071.83 (27777.24 to 63629.02) | 52785.55 (35282.97 to 74738.03) | 56306.46 (37439.78 to 78456.11) | 54285.30 (36553.56 to 74917.26) | 50173.66 (33977.30 to 69113.76) | 45427.38 (30977.53 to 62141.21) | 39525.49 (26992.84 to 54475.42) | 33945.02 (23205.93 to 46232.54) | 29470.88 (20239.91 to 40347.71) | 24984.37 (17278.96 to 34400.92) | 21151.22 (14703.65 to 28994.53) | 17376.32 (12104.88 to 23977.69) | 12431.47 (8675.94 to 16991.96) | 8342.10 (5856.41 to 11376.10) | 4793.71 (3388.10 to 6553.93) | 2306.68 (1638.97 to 3116.08) | 835.52 (595.10 to 1116.80) | 226.61 (162.65 to 303.78) | 45.46 (32.93 to 60.64) |
| Western Sub-Saharan Africa | Both | Number of prevalence | 843460.56 (514932.39 to 1313281.21) | 2052838.04 (1348525.93 to 3042319.22) | 2460588.29 (1614713.71 to 3660580.65) | 2746818.40 (1795261.64 to 4091422.00) | 2832874.53 (1837339.54 to 4248909.48) | 2715095.92 (1761769.04 to 4083030.61) | 2456209.39 (1591088.99 to 3670097.28) | 2105658.99 (1356949.55 to 3164030.88) | 1781177.62 (1154703.21 to 2662310.51) | 1557948.63 (1001657.07 to 2338359.99) | 1319533.72 (861063.92 to 1952817.40) | 1118488.71 (730976.59 to 1613901.84) | 937896.41 (617177.99 to 1356255.65) | 725250.89 (471125.78 to 1068072.30) | 508420.27 (324660.58 to 753821.94) | 306753.89 (192484.75 to 460025.99) | 154191.13 (94742.65 to 235438.00) | 58822.76 (35420.59 to 92130.74) | 17048.54 (10027.89 to 27184.64) | 3873.25 (2215.24 to 6240.16) |
| Western Sub-Saharan Africa | Female | Number of DALYs | 2533.64 (1728.46 to 3551.35) | 4401.69 (2978.08 to 6276.22) | 4759.14 (3288.38 to 6886.85) | 5548.71 (3773.67 to 8114.89) | 6481.13 (4408.09 to 9448.60) | 6808.27 (4573.48 to 9804.31) | 6393.88 (4273.78 to 9185.94) | 5489.02 (3679.93 to 7983.69) | 4658.60 (3093.95 to 6787.95) | 4175.89 (2798.34 to 6089.45) | 3618.24 (2395.81 to 5257.89) | 3129.24 (2064.94 to 4558.73) | 2738.63 (1818.58 to 3916.29) | 2282.11 (1532.39 to 3259.67) | 1599.92 (1068.03 to 2283.30) | 969.19 (649.06 to 1365.35) | 495.15 (335.80 to 698.38) | 193.87 (132.46 to 273.44) | 58.41 (39.87 to 81.67) | 14.59 (10.03 to 20.20) |
| Western Sub-Saharan Africa | Female | Number of prevalence | 425641.64 (259765.35 to 662835.81) | 895922.38 (543399.26 to 1402628.33) | 1077952.90 (642593.27 to 1693150.06) | 1239672.38 (731380.97 to 1949105.06) | 1324104.13 (790568.90 to 2086239.41) | 1270998.23 (769674.34 to 1985864.43) | 1111280.18 (678321.59 to 1732054.99) | 904924.48 (557395.25 to 1407465.97) | 727244.45 (458143.95 to 1120781.38) | 631099.33 (393353.68 to 973044.43) | 523963.44 (331783.11 to 796051.13) | 437349.53 (278674.78 to 646225.01) | 374456.09 (237172.13 to 564815.17) | 326717.06 (199082.20 to 509884.64) | 239187.57 (141758.30 to 383811.11) | 150788.52 (87313.03 to 242905.20) | 79399.83 (45118.59 to 128966.17) | 31989.95 (17928.93 to 52691.72) | 9906.62 (5461.67 to 16375.43) | 2525.57 (1361.51 to 4186.24) |
| Western Sub-Saharan Africa | Male | Number of DALYs | 1794.87 (1186.08 to 2560.55) | 38670.14 (24718.57 to 58029.47) | 48026.42 (31926.47 to 68619.43) | 50757.75 (33788.09 to 71100.08) | 47804.17 (32102.04 to 65428.61) | 43365.39 (29276.25 to 59570.55) | 39033.50 (26487.30 to 53281.74) | 34036.47 (23251.05 to 46612.09) | 29286.42 (20035.91 to 39899.91) | 25294.98 (17306.72 to 34465.62) | 21366.13 (14674.49 to 29234.77) | 18021.98 (12448.65 to 24474.97) | 14637.69 (10104.80 to 19978.44) | 10149.37 (7029.00 to 13664.67) | 6742.18 (4695.43 to 9007.82) | 3824.52 (2683.29 to 5114.75) | 1811.53 (1277.92 to 2413.23) | 641.65 (455.62 to 850.10) | 168.19 (119.75 to 222.84) | 30.87 (22.32 to 40.61) |
| Western Sub-Saharan Africa | Male | Number of prevalence | 417818.92 (254674.10 to 649449.29) | 1156915.66 (807270.59 to 1638442.05) | 1382635.39 (969835.91 to 1958929.90) | 1507146.02 (1051468.73 to 2154595.15) | 1508770.40 (1044464.93 to 2168076.71) | 1444097.69 (988418.90 to 2089531.57) | 1344929.20 (908626.72 to 1962105.42) | 1200734.51 (804350.37 to 1754769.02) | 1053933.17 (697342.64 to 1542628.57) | 926849.30 (611540.20 to 1360938.96) | 795570.28 (526493.44 to 1161862.23) | 681139.18 (453585.89 to 969391.87) | 563440.33 (378479.40 to 796405.53) | 398533.84 (270354.46 to 559964.82) | 269232.70 (183364.46 to 377611.92) | 155965.38 (105893.06 to 219749.08) | 74791.29 (50221.18 to 107125.64) | 26832.81 (17605.79 to 39116.99) | 7141.92 (4589.43 to 10632.05) | 1347.68 (859.56 to 2050.93) |
| **Country and territory** |  |  |  |  |  |  |  |  |  |  |  |  |  |  |  |  |  |  |  |  |  |  |
| American Samoa | Both | Number of DALYs | 0.80 (0.49 to 1.20) | 3.87 (2.11 to 6.71) | 5.07 (2.84 to 8.42) | 6.90 (4.08 to 10.99) | 8.66 (5.31 to 13.36) | 9.08 (5.77 to 13.36) | 8.47 (5.51 to 12.47) | 7.75 (5.02 to 11.47) | 6.78 (4.42 to 9.86) | 5.85 (3.92 to 8.34) | 4.94 (3.28 to 7.03) | 4.03 (2.68 to 5.65) | 3.04 (2.03 to 4.24) | 2.22 (1.48 to 3.07) | 1.41 (0.95 to 1.98) | 0.79 (0.55 to 1.09) | 0.34 (0.23 to 0.46) | 0.13 (0.09 to 0.17) | 0.03 (0.02 to 0.04) | 0.00 (0.00 to 0.01) |
| American Samoa | Both | Number of prevalence | 95.94 (81.44 to 113.25) | 216.39 (183.36 to 257.87) | 277.63 (235.39 to 329.42) | 352.23 (300.53 to 414.94) | 426.14 (364.44 to 499.09) | 429.72 (369.13 to 501.60) | 381.08 (328.52 to 442.66) | 338.34 (292.36 to 391.96) | 286.41 (247.94 to 331.00) | 243.68 (210.99 to 281.29) | 203.19 (176.24 to 234.27) | 167.74 (145.71 to 193.12) | 134.83 (117.17 to 155.46) | 101.99 (88.57 to 117.68) | 65.57 (57.00 to 75.62) | 38.45 (33.40 to 44.39) | 17.27 (14.99 to 19.95) | 7.04 (6.11 to 8.15) | 1.62 (1.41 to 1.88) | 0.30 (0.26 to 0.35) |
| American Samoa | Female | Number of DALYs | 0.45 (0.26 to 0.69) | 0.70 (0.43 to 1.09) | 0.67 (0.42 to 1.05) | 0.71 (0.42 to 1.09) | 0.83 (0.50 to 1.28) | 0.79 (0.50 to 1.17) | 0.65 (0.41 to 0.97) | 0.55 (0.34 to 0.81) | 0.44 (0.27 to 0.66) | 0.35 (0.22 to 0.53) | 0.29 (0.18 to 0.42) | 0.24 (0.15 to 0.34) | 0.21 (0.13 to 0.31) | 0.16 (0.10 to 0.23) | 0.10 (0.07 to 0.15) | 0.06 (0.04 to 0.09) | 0.03 (0.02 to 0.04) | 0.01 (0.01 to 0.02) | 0.00 (0.00 to 0.00) | 0.00 (0.00 to 0.00) |
| American Samoa | Female | Number of prevalence | 46.65 (39.76 to 54.93) | 94.39 (80.15 to 111.65) | 117.23 (99.24 to 139.01) | 148.57 (125.57 to 176.40) | 189.26 (159.84 to 224.81) | 189.67 (160.11 to 225.33) | 160.92 (135.83 to 191.21) | 138.37 (116.77 to 164.43) | 111.71 (94.29 to 132.74) | 92.43 (78.01 to 109.84) | 74.92 (63.22 to 89.04) | 62.93 (53.10 to 74.79) | 55.76 (47.06 to 66.26) | 43.45 (36.68 to 51.64) | 27.69 (23.37 to 32.91) | 16.84 (14.21 to 20.01) | 7.82 (6.60 to 9.29) | 3.49 (2.94 to 4.15) | 0.88 (0.74 to 1.05) | 0.20 (0.17 to 0.24) |
| American Samoa | Male | Number of DALYs | 0.35 (0.20 to 0.56) | 3.17 (1.59 to 5.80) | 4.40 (2.31 to 7.55) | 6.19 (3.47 to 10.10) | 7.83 (4.74 to 12.26) | 8.29 (5.14 to 12.38) | 7.82 (5.07 to 11.54) | 7.20 (4.66 to 10.76) | 6.34 (4.13 to 9.25) | 5.49 (3.68 to 7.82) | 4.66 (3.08 to 6.64) | 3.79 (2.50 to 5.38) | 2.83 (1.87 to 3.98) | 2.06 (1.36 to 2.87) | 1.31 (0.88 to 1.85) | 0.73 (0.50 to 1.02) | 0.31 (0.21 to 0.43) | 0.12 (0.08 to 0.16) | 0.02 (0.02 to 0.03) | 0.00 (0.00 to 0.00) |
| American Samoa | Male | Number of prevalence | 49.29 (41.72 to 58.24) | 122.00 (101.82 to 147.90) | 160.40 (134.60 to 191.82) | 203.65 (173.72 to 239.25) | 236.88 (203.96 to 274.78) | 240.05 (208.80 to 279.10) | 220.16 (191.49 to 254.17) | 199.97 (174.03 to 230.33) | 174.70 (152.03 to 200.38) | 151.25 (131.96 to 173.14) | 128.27 (112.24 to 146.44) | 104.81 (92.03 to 119.62) | 79.07 (69.59 to 90.21) | 58.54 (51.62 to 66.58) | 37.88 (33.37 to 43.07) | 21.61 (19.05 to 24.58) | 9.46 (8.33 to 10.76) | 3.55 (3.14 to 4.05) | 0.74 (0.65 to 0.85) | 0.11 (0.09 to 0.12) |
| Angola | Both | Number of DALYs | 201.33 (124.41 to 304.98) | 855.80 (445.04 to 1502.54) | 1039.90 (584.35 to 1741.84) | 1268.06 (709.53 to 2043.46) | 1428.31 (875.01 to 2186.62) | 1479.83 (926.75 to 2247.47) | 1395.83 (910.33 to 2072.34) | 1312.60 (854.19 to 1921.84) | 1004.64 (670.61 to 1478.84) | 934.51 (620.52 to 1342.89) | 799.34 (537.91 to 1149.48) | 631.06 (418.28 to 893.34) | 493.68 (330.13 to 695.34) | 335.97 (222.06 to 470.75) | 198.42 (134.68 to 281.53) | 100.97 (68.14 to 140.28) | 40.37 (27.28 to 55.68) | 13.18 (9.14 to 17.96) | 3.14 (2.14 to 4.32) | 0.57 (0.40 to 0.78) |
| Angola | Both | Number of prevalence | 22268.44 (4521.77 to 70574.32) | 48919.74 (12168.96 to 148455.03) | 60040.08 (14924.75 to 182751.19) | 68604.24 (17704.18 to 207293.32) | 72108.42 (19333.62 to 215909.06) | 72193.12 (19709.11 to 214882.61) | 65353.16 (18157.92 to 193470.73) | 59898.47 (16917.52 to 176605.26) | 45142.04 (12910.15 to 132623.86) | 41597.22 (12031.12 to 121941.88) | 35346.59 (10326.28 to 103412.89) | 27926.69 (8246.72 to 81548.65) | 22267.82 (6588.24 to 65051.16) | 15548.57 (4587.13 to 45488.71) | 9399.93 (2757.41 to 27534.02) | 4968.77 (1437.62 to 14586.33) | 2109.45 (592.94 to 6229.94) | 739.41 (202.46 to 2198.36) | 189.33 (50.40 to 566.64) | 39.62 (9.98 to 120.10) |
| Angola | Female | Number of DALYs | 118.15 (69.31 to 180.84) | 173.29 (106.39 to 260.42) | 161.72 (100.61 to 247.65) | 149.34 (92.71 to 221.06) | 137.49 (84.85 to 215.23) | 130.66 (81.55 to 189.40) | 110.89 (68.14 to 161.80) | 98.95 (62.26 to 142.53) | 71.50 (44.83 to 106.23) | 66.07 (42.51 to 96.36) | 54.89 (35.83 to 80.64) | 42.39 (26.49 to 62.04) | 34.18 (22.31 to 51.76) | 24.03 (15.59 to 34.63) | 14.51 (9.17 to 21.54) | 7.69 (5.27 to 11.12) | 3.38 (2.20 to 4.94) | 1.25 (0.82 to 1.69) | 0.33 (0.21 to 0.47) | 0.07 (0.05 to 0.11) |
| Angola | Female | Number of prevalence | 11242.08 (2395.02 to 35281.32) | 22443.54 (4303.49 to 71808.34) | 27080.94 (4800.70 to 87752.76) | 30106.17 (5089.51 to 98254.70) | 31140.61 (5141.36 to 101972.87) | 31102.84 (5081.66 to 102014.12) | 27513.87 (4469.51 to 90313.48) | 24893.17 (4029.79 to 81762.63) | 18500.47 (2985.74 to 60787.32) | 16984.08 (2737.79 to 55818.40) | 14317.15 (2303.41 to 47065.79) | 11226.32 (1804.08 to 36910.63) | 9161.94 (1471.07 to 30128.50) | 6535.70 (1048.70 to 21494.34) | 3999.74 (641.77 to 13154.36) | 2173.56 (348.60 to 7148.29) | 979.48 (157.25 to 3221.22) | 367.46 (58.96 to 1208.44) | 99.97 (16.04 to 328.78) | 23.37 (3.75 to 76.84) |
| Angola | Male | Number of DALYs | 83.18 (45.01 to 132.08) | 682.51 (316.36 to 1311.09) | 878.18 (467.48 to 1513.94) | 1118.73 (607.40 to 1863.05) | 1290.82 (766.45 to 2030.90) | 1349.17 (830.97 to 2066.80) | 1284.94 (816.52 to 1930.50) | 1213.65 (777.82 to 1793.17) | 933.14 (613.47 to 1389.22) | 868.44 (568.06 to 1271.04) | 744.45 (496.80 to 1077.56) | 588.68 (387.06 to 837.94) | 459.49 (305.46 to 646.30) | 311.95 (201.52 to 443.15) | 183.90 (124.04 to 263.33) | 93.28 (61.90 to 131.48) | 36.99 (24.66 to 51.52) | 11.93 (8.19 to 16.39) | 2.81 (1.89 to 3.87) | 0.50 (0.34 to 0.69) |
| Angola | Male | Number of prevalence | 11026.36 (2126.29 to 35293.00) | 26476.20 (7443.29 to 76646.69) | 32959.13 (9642.56 to 94992.55) | 38498.08 (12159.62 to 109038.63) | 40967.80 (13997.07 to 113936.20) | 41090.28 (14613.62 to 112868.49) | 37839.29 (13899.32 to 103157.25) | 35005.30 (13100.18 to 94842.64) | 26641.56 (10116.39 to 71836.54) | 24613.14 (9447.70 to 66123.47) | 21029.44 (8104.96 to 56347.10) | 16700.37 (6502.88 to 44638.02) | 13105.88 (5121.74 to 34922.66) | 9012.87 (3526.02 to 23994.38) | 5400.19 (2115.10 to 14379.66) | 2795.21 (1094.21 to 7438.04) | 1129.97 (441.93 to 3008.71) | 371.95 (145.66 to 989.92) | 89.36 (35.03 to 237.86) | 16.25 (6.36 to 43.26) |
| Bangladesh | Both | Number of DALYs | 1911.82 (1225.02 to 2783.78) | 6718.41 (3561.24 to 11817.46) | 6683.16 (3558.69 to 12086.32) | 7027.66 (3705.00 to 12059.65) | 7420.69 (4067.37 to 12849.80) | 7433.65 (4137.23 to 12668.05) | 6827.32 (3851.82 to 11382.18) | 5949.03 (3430.96 to 9771.85) | 5062.37 (3010.58 to 8283.14) | 4132.68 (2390.50 to 6740.62) | 3386.29 (1977.18 to 5452.45) | 2993.57 (1762.04 to 4769.70) | 2568.22 (1586.57 to 4115.29) | 1756.12 (1095.44 to 2742.40) | 1394.13 (830.72 to 2196.44) | 967.54 (603.23 to 1519.50) | 549.61 (331.47 to 864.85) | 262.62 (160.10 to 398.80) | 65.45 (40.35 to 101.17) | 13.83 (8.67 to 21.46) |
| Bangladesh | Both | Number of prevalence | 137186.21 (119580.49 to 156285.48) | 317688.48 (274362.42 to 365471.14) | 365707.22 (316154.07 to 420163.99) | 416503.75 (361020.32 to 477410.79) | 444317.87 (385812.80 to 508565.13) | 427886.39 (371531.92 to 489561.08) | 381648.22 (331991.85 to 436735.37) | 322647.42 (281184.55 to 369270.28) | 272283.87 (237496.84 to 311894.89) | 220875.39 (192799.67 to 253086.53) | 179655.04 (156878.15 to 205732.25) | 157989.43 (137962.03 to 180931.57) | 134917.78 (117842.52 to 154525.11) | 91653.69 (80072.47 to 104908.26) | 73295.60 (63962.09 to 83895.87) | 51035.79 (44535.16 to 58394.96) | 29179.98 (25466.49 to 33378.72) | 14002.50 (12211.00 to 16025.49) | 3546.58 (3093.12 to 4058.37) | 767.42 (669.28 to 877.93) |
| Bangladesh | Female | Number of DALYs | 1089.48 (639.37 to 1697.97) | 1699.95 (997.60 to 2715.35) | 1488.58 (865.46 to 2299.36) | 1384.29 (800.25 to 2227.21) | 1269.50 (728.72 to 1992.26) | 1081.51 (641.75 to 1722.73) | 864.53 (513.14 to 1309.08) | 681.91 (409.69 to 1038.70) | 545.83 (332.59 to 815.38) | 425.68 (257.34 to 633.55) | 337.98 (206.97 to 496.16) | 285.22 (174.34 to 421.70) | 234.75 (148.04 to 351.62) | 153.18 (97.40 to 225.35) | 117.32 (75.54 to 171.63) | 78.19 (48.68 to 114.47) | 42.60 (26.74 to 61.62) | 19.31 (12.21 to 28.05) | 4.73 (3.02 to 7.07) | 1.02 (0.65 to 1.49) |
| Bangladesh | Female | Number of prevalence | 68007.81 (59639.10 to 77344.56) | 140944.39 (122353.47 to 161062.75) | 166906.34 (143860.87 to 191205.84) | 196129.36 (168493.83 to 225341.15) | 208686.51 (178888.50 to 240046.02) | 191842.64 (164304.67 to 220812.99) | 162666.15 (139181.54 to 187310.13) | 133009.83 (113793.16 to 153197.43) | 110800.06 (94795.65 to 127619.27) | 89240.72 (76315.06 to 102814.43) | 72433.10 (61918.85 to 83462.14) | 62742.13 (53642.33 to 72311.00) | 52966.67 (45266.28 to 61044.69) | 35388.30 (30242.30 to 40787.09) | 27568.02 (23554.75 to 31776.10) | 18647.24 (15936.47 to 21492.22) | 10424.54 (8907.94 to 12016.62) | 4835.72 (4132.27 to 5574.06) | 1209.84 (1033.72 to 1394.46) | 265.63 (226.99 to 306.18) |
| Bangladesh | Male | Number of DALYs | 822.34 (444.54 to 1330.36) | 5018.46 (2365.05 to 9839.71) | 5194.58 (2487.47 to 10321.06) | 5643.37 (2778.89 to 10651.01) | 6151.18 (3082.26 to 11317.47) | 6352.14 (3371.40 to 11397.37) | 5962.79 (3169.28 to 10247.09) | 5267.12 (2875.31 to 8935.97) | 4516.55 (2554.03 to 7596.54) | 3707.00 (2102.55 to 6239.76) | 3048.32 (1698.50 to 5047.72) | 2708.36 (1535.64 to 4426.84) | 2333.47 (1400.11 to 3800.79) | 1602.94 (984.65 to 2574.15) | 1276.81 (752.45 to 2065.36) | 889.35 (542.64 to 1412.76) | 507.01 (301.71 to 814.73) | 243.31 (143.20 to 378.26) | 60.72 (36.69 to 95.31) | 12.81 (7.79 to 20.40) |
| Bangladesh | Male | Number of prevalence | 69178.40 (59824.88 to 79098.31) | 176744.10 (147818.61 to 218115.79) | 198800.89 (167656.24 to 239574.66) | 220374.40 (186959.63 to 259687.69) | 235631.37 (200370.65 to 273361.01) | 236043.76 (200956.56 to 273266.32) | 218982.07 (186243.17 to 253397.69) | 189637.60 (161497.94 to 218723.80) | 161483.81 (137887.70 to 185948.27) | 131634.67 (112409.99 to 151401.41) | 107221.94 (91828.41 to 123229.48) | 95247.29 (81501.95 to 109288.61) | 81951.11 (70205.58 to 93945.12) | 56265.38 (48286.80 to 64443.23) | 45727.58 (39210.67 to 52402.76) | 32388.55 (27783.29 to 37129.06) | 18755.44 (16081.50 to 21482.37) | 9166.78 (7867.53 to 10504.00) | 2336.74 (2004.77 to 2675.55) | 501.79 (430.41 to 575.32) |
| Benin | Both | Number of DALYs | 94.46 (59.27 to 141.11) | 288.27 (149.82 to 519.48) | 239.58 (128.47 to 421.71) | 198.59 (102.17 to 360.26) | 185.59 (99.39 to 317.20) | 180.68 (101.19 to 307.27) | 158.37 (89.91 to 273.84) | 137.84 (78.35 to 240.01) | 101.52 (57.14 to 175.62) | 90.26 (51.55 to 153.46) | 71.98 (40.12 to 121.66) | 60.07 (33.13 to 99.61) | 52.55 (29.39 to 88.16) | 43.50 (24.82 to 71.93) | 33.08 (19.15 to 53.53) | 22.75 (12.99 to 37.33) | 11.36 (6.64 to 19.23) | 5.30 (3.05 to 8.76) | 1.42 (0.85 to 2.29) | 0.29 (0.17 to 0.45) |
| Benin | Both | Number of prevalence | 4773.17 (1178.17 to 20907.18) | 10908.60 (2884.01 to 45701.97) | 10968.30 (2548.52 to 47889.03) | 10709.00 (2238.00 to 48065.40) | 11173.08 (2161.00 to 50969.26) | 11342.01 (2139.94 to 52030.14) | 10000.45 (1893.48 to 45881.83) | 8600.30 (1643.12 to 39396.74) | 6354.75 (1211.79 to 29124.19) | 5641.30 (1073.60 to 25847.89) | 4485.56 (860.66 to 20518.93) | 3756.11 (719.79 to 17183.71) | 3345.35 (642.20 to 15304.39) | 2760.48 (537.69 to 12597.90) | 2087.13 (411.63 to 9496.65) | 1457.35 (288.49 to 6628.05) | 761.49 (147.83 to 3475.71) | 363.80 (70.66 to 1660.60) | 113.77 (20.24 to 528.11) | 28.05 (4.65 to 132.35) |
| Benin | Female | Number of DALYs | 53.81 (32.05 to 84.58) | 81.12 (48.11 to 129.22) | 60.92 (36.52 to 94.83) | 49.15 (28.42 to 78.04) | 48.56 (28.95 to 71.64) | 45.22 (26.98 to 66.85) | 36.66 (23.11 to 55.82) | 28.62 (16.63 to 43.65) | 20.37 (12.29 to 31.63) | 17.40 (10.60 to 26.93) | 13.12 (7.37 to 19.63) | 10.61 (6.26 to 15.64) | 9.08 (5.38 to 13.74) | 7.13 (4.48 to 10.64) | 5.10 (3.25 to 7.74) | 3.44 (2.13 to 5.29) | 1.88 (1.20 to 2.72) | 0.88 (0.52 to 1.30) | 0.34 (0.21 to 0.49) | 0.09 (0.06 to 0.14) |
| Benin | Female | Number of prevalence | 2405.81 (641.38 to 10284.76) | 4805.13 (1032.76 to 21659.27) | 4838.18 (858.99 to 22639.60) | 5085.64 (776.70 to 24356.17) | 5842.40 (815.75 to 28314.82) | 6030.04 (798.15 to 29409.58) | 5175.71 (664.39 to 25331.61) | 4274.11 (535.30 to 20973.21) | 3149.46 (387.08 to 15484.09) | 2771.92 (335.77 to 13648.25) | 2152.96 (257.66 to 10614.87) | 1802.36 (212.84 to 8898.06) | 1605.04 (187.19 to 7934.43) | 1281.32 (148.36 to 6338.04) | 930.50 (107.81 to 4602.46) | 644.44 (74.55 to 3187.69) | 354.10 (41.03 to 1751.47) | 169.34 (19.64 to 837.66) | 65.29 (7.55 to 322.94) | 19.09 (2.21 to 94.43) |
| Benin | Male | Number of DALYs | 40.65 (22.95 to 63.80) | 207.15 (90.85 to 424.18) | 178.66 (82.78 to 352.98) | 149.44 (66.40 to 295.64) | 137.04 (64.44 to 262.90) | 135.46 (68.69 to 248.33) | 121.71 (61.99 to 223.75) | 109.22 (56.42 to 204.94) | 81.15 (40.93 to 146.86) | 72.86 (37.11 to 131.33) | 58.87 (29.17 to 105.46) | 49.46 (25.00 to 87.10) | 43.47 (22.43 to 75.99) | 36.37 (18.93 to 63.42) | 27.98 (15.01 to 47.57) | 19.31 (10.23 to 32.83) | 9.48 (5.20 to 16.64) | 4.42 (2.37 to 7.64) | 1.09 (0.59 to 1.86) | 0.20 (0.10 to 0.34) |
| Benin | Male | Number of prevalence | 2367.36 (517.83 to 10622.42) | 6103.47 (1731.00 to 24044.48) | 6130.11 (1564.23 to 25249.43) | 5623.35 (1372.34 to 23709.23) | 5330.68 (1290.66 to 22654.44) | 5311.97 (1286.58 to 22620.56) | 4824.74 (1172.18 to 20550.22) | 4326.19 (1055.36 to 18423.53) | 3205.29 (783.26 to 13640.10) | 2869.38 (704.78 to 12199.64) | 2332.61 (575.16 to 9904.06) | 1953.75 (483.20 to 8285.65) | 1740.31 (433.33 to 7369.96) | 1479.15 (369.97 to 6259.86) | 1156.63 (289.27 to 4894.19) | 812.91 (203.18 to 3440.36) | 407.39 (101.77 to 1724.25) | 194.45 (48.59 to 822.94) | 48.48 (12.12 to 205.17) | 8.96 (2.24 to 37.93) |
| Brazil | Both | Number of DALYs | 325.34 (216.35 to 471.60) | 784.33 (499.51 to 1177.55) | 806.33 (507.25 to 1216.65) | 755.25 (469.00 to 1169.86) | 681.62 (418.11 to 1041.60) | 594.21 (372.28 to 919.16) | 510.15 (327.13 to 774.09) | 444.49 (273.30 to 675.10) | 387.30 (239.84 to 577.03) | 327.60 (210.23 to 494.12) | 280.61 (182.76 to 424.29) | 231.70 (148.91 to 352.26) | 205.48 (129.57 to 310.87) | 167.38 (104.26 to 256.35) | 122.97 (78.48 to 187.99) | 84.12 (54.42 to 125.14) | 41.17 (26.70 to 60.97) | 18.82 (11.83 to 28.01) | 4.89 (3.28 to 6.87) | 1.40 (0.95 to 1.95) |
| Brazil | Both | Number of prevalence | 7602.23 (6017.32 to 12811.55) | 18703.83 (14287.59 to 31653.74) | 23574.13 (17422.80 to 42065.82) | 26446.84 (19130.11 to 48954.00) | 26494.83 (18975.65 to 49967.51) | 24570.31 (17519.91 to 46747.09) | 21746.70 (15459.29 to 41565.93) | 19639.36 (13913.44 to 37694.37) | 17450.42 (12345.65 to 33577.30) | 15133.68 (10687.57 to 29191.95) | 13250.87 (9343.32 to 25631.81) | 11304.82 (7963.33 to 21936.58) | 10240.65 (7208.05 to 19887.94) | 8430.22 (5932.70 to 16375.10) | 6306.55 (4437.88 to 12248.08) | 4272.40 (3015.53 to 8271.51) | 2213.08 (1556.88 to 4301.41) | 1079.17 (755.72 to 2109.13) | 270.54 (190.91 to 524.82) | 60.46 (44.31 to 112.34) |
| Brazil | Female | Number of DALYs | 189.56 (119.17 to 278.02) | 353.70 (223.53 to 517.40) | 341.43 (222.15 to 503.13) | 294.92 (192.00 to 449.53) | 252.43 (158.39 to 368.50) | 208.46 (136.62 to 304.37) | 173.75 (115.27 to 246.70) | 146.85 (96.30 to 217.02) | 122.39 (80.09 to 180.21) | 100.63 (65.98 to 147.48) | 83.92 (54.86 to 120.86) | 67.66 (44.68 to 99.27) | 57.01 (38.41 to 83.03) | 44.93 (29.85 to 65.12) | 33.42 (22.15 to 47.51) | 24.09 (16.13 to 34.13) | 12.94 (8.67 to 18.34) | 6.13 (4.08 to 8.72) | 2.14 (1.43 to 3.12) | 0.78 (0.52 to 1.14) |
| Brazil | Female | Number of prevalence | 4030.71 (3245.58 to 6559.45) | 9087.49 (7087.92 to 15710.70) | 11367.89 (8532.49 to 20888.05) | 12788.68 (9279.91 to 24593.18) | 12959.75 (9231.64 to 25529.59) | 12157.47 (8575.18 to 24251.11) | 10849.71 (7613.72 to 21789.37) | 9844.49 (6872.38 to 19900.90) | 8711.98 (6054.21 to 17697.91) | 7534.71 (5221.44 to 15365.42) | 6640.01 (4584.73 to 13593.58) | 5700.12 (3923.44 to 11713.01) | 5076.46 (3486.84 to 10461.97) | 4114.87 (2821.69 to 8495.38) | 3081.04 (2113.84 to 6358.16) | 2098.24 (1445.88 to 4307.33) | 1175.50 (809.15 to 2416.36) | 601.49 (412.64 to 1241.00) | 171.83 (119.66 to 348.21) | 39.11 (28.52 to 74.71) |
| Brazil | Male | Number of DALYs | 135.79 (85.36 to 204.29) | 430.63 (245.05 to 730.18) | 464.90 (264.80 to 769.91) | 460.33 (262.23 to 789.17) | 429.19 (242.86 to 717.80) | 385.75 (223.84 to 647.26) | 336.40 (194.47 to 567.63) | 297.64 (168.43 to 494.73) | 264.91 (149.95 to 426.24) | 226.97 (131.88 to 370.19) | 196.70 (115.61 to 317.49) | 164.04 (95.81 to 266.62) | 148.47 (86.13 to 244.20) | 122.45 (71.14 to 201.43) | 89.55 (51.24 to 143.10) | 60.03 (35.61 to 96.23) | 28.23 (16.74 to 45.24) | 12.70 (7.51 to 20.67) | 2.74 (1.72 to 4.30) | 0.62 (0.39 to 0.92) |
| Brazil | Male | Number of prevalence | 3571.52 (2759.51 to 6214.44) | 9616.33 (7170.35 to 16094.08) | 12206.24 (8932.54 to 21460.33) | 13658.16 (9836.77 to 24655.02) | 13535.08 (9711.87 to 24723.59) | 12412.83 (8903.38 to 22762.38) | 10896.98 (7815.80 to 20011.69) | 9794.87 (7025.32 to 18007.60) | 8738.44 (6271.94 to 16069.42) | 7598.97 (5453.15 to 13988.57) | 6610.86 (4745.38 to 12181.08) | 5604.71 (4020.84 to 10343.49) | 5164.19 (3705.96 to 9529.87) | 4315.35 (3100.06 to 7962.88) | 3225.51 (2315.30 to 5953.34) | 2174.16 (1563.49 to 4007.64) | 1037.58 (746.88 to 1909.03) | 477.68 (343.33 to 880.21) | 98.70 (71.82 to 179.43) | 21.35 (15.72 to 38.22) |
| Brunei Darussalam | Both | Number of DALYs | 3.37 (2.14 to 5.19) | 9.33 (5.00 to 17.25) | 8.77 (4.58 to 16.20) | 8.84 (4.66 to 15.60) | 10.17 (5.54 to 18.66) | 11.57 (6.33 to 21.38) | 11.09 (5.87 to 19.95) | 9.20 (4.77 to 16.97) | 6.48 (3.41 to 11.81) | 4.44 (2.27 to 8.02) | 3.08 (1.69 to 5.58) | 2.31 (1.25 to 4.18) | 1.65 (0.86 to 2.87) | 1.31 (0.70 to 2.32) | 0.85 (0.45 to 1.53) | 0.55 (0.30 to 0.95) | 0.37 (0.20 to 0.66) | 0.20 (0.11 to 0.35) | 0.01 (0.01 to 0.02) | 0.00 (0.00 to 0.00) |
| Brunei Darussalam | Both | Number of prevalence | 111.76 (32.31 to 745.63) | 253.52 (64.06 to 1612.13) | 291.81 (59.44 to 2007.81) | 354.15 (60.94 to 2557.79) | 444.18 (68.96 to 3284.63) | 531.84 (77.78 to 3975.18) | 526.95 (74.48 to 3959.85) | 446.17 (61.82 to 3362.19) | 317.49 (43.26 to 2398.01) | 224.43 (29.99 to 1704.14) | 161.66 (21.17 to 1232.76) | 125.49 (16.13 to 961.83) | 92.67 (11.75 to 712.27) | 74.15 (9.44 to 568.74) | 49.13 (6.22 to 377.39) | 33.49 (4.19 to 258.49) | 23.02 (2.86 to 177.91) | 12.25 (1.55 to 94.17) | 1.31 (0.13 to 10.73) | 0.17 (0.02 to 1.41) |
| Brunei Darussalam | Female | Number of DALYs | 1.90 (1.14 to 3.04) | 2.89 (1.80 to 4.61) | 2.52 (1.41 to 3.86) | 2.42 (1.47 to 3.81) | 2.57 (1.52 to 4.11) | 2.68 (1.55 to 4.13) | 2.42 (1.37 to 3.71) | 1.88 (1.13 to 2.79) | 1.27 (0.71 to 1.95) | 0.90 (0.51 to 1.35) | 0.64 (0.38 to 0.99) | 0.50 (0.30 to 0.78) | 0.36 (0.21 to 0.54) | 0.26 (0.16 to 0.40) | 0.18 (0.11 to 0.27) | 0.13 (0.08 to 0.19) | 0.09 (0.05 to 0.13) | 0.04 (0.03 to 0.06) | 0.01 (0.01 to 0.01) | 0.00 (0.00 to 0.00) |
| Brunei Darussalam | Female | Number of prevalence | 56.21 (17.89 to 358.93) | 110.27 (27.06 to 771.91) | 128.57 (24.08 to 963.33) | 157.22 (23.35 to 1229.11) | 195.12 (24.91 to 1559.68) | 226.84 (26.36 to 1834.90) | 219.00 (23.99 to 1783.60) | 179.14 (18.77 to 1466.22) | 124.42 (12.57 to 1022.13) | 91.40 (8.97 to 753.27) | 67.94 (6.47 to 561.39) | 55.07 (5.12 to 456.24) | 40.98 (3.70 to 340.34) | 31.18 (2.78 to 259.28) | 21.14 (1.88 to 175.78) | 15.50 (1.38 to 128.92) | 10.83 (0.96 to 90.10) | 5.35 (0.48 to 44.53) | 1.15 (0.10 to 9.54) | 0.17 (0.01 to 1.38) |
| Brunei Darussalam | Male | Number of DALYs | 1.47 (0.79 to 2.37) | 6.44 (2.87 to 13.00) | 6.26 (2.72 to 13.10) | 6.42 (2.83 to 12.88) | 7.60 (3.38 to 15.45) | 8.89 (4.27 to 18.02) | 8.67 (3.97 to 16.83) | 7.32 (3.35 to 14.61) | 5.20 (2.41 to 10.28) | 3.54 (1.61 to 7.01) | 2.44 (1.20 to 4.78) | 1.80 (0.83 to 3.52) | 1.29 (0.62 to 2.38) | 1.05 (0.51 to 2.04) | 0.67 (0.31 to 1.30) | 0.42 (0.21 to 0.78) | 0.28 (0.13 to 0.55) | 0.16 (0.08 to 0.30) | 0.00 (0.00 to 0.01) | 0.00 (0.00 to 0.00) |
| Brunei Darussalam | Male | Number of prevalence | 55.55 (13.92 to 386.70) | 143.25 (36.04 to 843.80) | 163.23 (34.28 to 1044.48) | 196.93 (36.41 to 1328.68) | 249.06 (42.74 to 1724.95) | 305.00 (50.36 to 2140.28) | 307.95 (49.35 to 2176.25) | 267.03 (42.60 to 1895.98) | 193.08 (30.38 to 1375.88) | 133.03 (20.73 to 950.87) | 93.71 (14.37 to 671.38) | 70.41 (10.78 to 505.59) | 51.69 (7.87 to 371.92) | 42.97 (6.51 to 309.47) | 27.99 (4.24 to 201.61) | 17.99 (2.73 to 129.57) | 12.19 (1.85 to 87.81) | 6.89 (1.04 to 49.64) | 0.17 (0.02 to 1.19) | 0.00 (0.00 to 0.03) |
| Burkina Faso | Both | Number of DALYs | 256.72 (169.19 to 378.49) | 3239.56 (2047.41 to 4815.89) | 4343.52 (2853.97 to 6149.29) | 4051.94 (2672.17 to 5680.85) | 3171.94 (2145.56 to 4375.97) | 2631.90 (1788.79 to 3628.31) | 2245.29 (1541.97 to 3076.62) | 1945.77 (1341.69 to 2727.72) | 1680.30 (1149.65 to 2323.44) | 1498.75 (1040.56 to 2040.31) | 1312.62 (907.02 to 1792.64) | 1146.50 (781.92 to 1578.02) | 969.74 (671.47 to 1347.20) | 769.80 (536.05 to 1053.90) | 519.90 (364.21 to 709.52) | 320.54 (225.74 to 434.52) | 131.59 (94.65 to 177.73) | 43.48 (30.90 to 57.98) | 9.85 (7.06 to 13.13) | 1.69 (1.22 to 2.24) |
| Burkina Faso | Both | Number of prevalence | 59412.08 (13960.70 to 139429.54) | 152208.65 (51127.34 to 326100.50) | 182177.36 (64213.63 to 387108.15) | 181016.37 (62722.30 to 387391.55) | 166512.88 (54599.72 to 362848.99) | 154778.89 (48985.42 to 340490.16) | 138318.64 (43265.65 to 305143.77) | 121945.05 (38027.71 to 269153.23) | 106412.11 (33110.76 to 234921.36) | 95468.92 (29744.44 to 210719.08) | 83651.48 (26129.44 to 184532.97) | 73361.48 (22978.46 to 161804.11) | 62639.95 (19682.29 to 138174.59) | 50809.45 (15947.47 to 112151.36) | 35010.25 (10984.05 to 77294.00) | 22656.42 (7053.34 to 50119.12) | 9611.73 (2974.65 to 21287.23) | 3243.27 (1002.58 to 7182.85) | 804.07 (243.50 to 1790.66) | 157.77 (46.38 to 353.88) |
| Burkina Faso | Female | Number of DALYs | 148.88 (92.22 to 218.69) | 271.36 (166.54 to 395.79) | 292.94 (181.71 to 424.34) | 312.83 (208.96 to 454.78) | 343.10 (227.24 to 505.61) | 363.54 (240.28 to 530.64) | 349.89 (234.39 to 491.50) | 317.92 (212.12 to 456.97) | 286.50 (189.23 to 410.78) | 262.65 (176.18 to 382.62) | 232.32 (151.77 to 332.84) | 207.84 (134.97 to 306.21) | 181.51 (120.15 to 260.83) | 150.53 (99.63 to 217.75) | 102.10 (66.75 to 148.86) | 67.55 (45.91 to 96.03) | 29.11 (20.24 to 41.16) | 9.64 (6.56 to 13.75) | 2.61 (1.80 to 3.64) | 0.57 (0.38 to 0.80) |
| Burkina Faso | Female | Number of prevalence | 29475.11 (7116.25 to 68835.03) | 64048.76 (15039.79 to 150325.82) | 74355.12 (17264.75 to 174881.67) | 77868.13 (18165.60 to 183029.47) | 79516.29 (18765.69 to 186528.68) | 79070.00 (18897.63 to 185074.37) | 71391.30 (17234.64 to 166792.36) | 62048.32 (15087.24 to 144719.72) | 53418.74 (13096.64 to 124394.69) | 47102.23 (11633.93 to 109521.64) | 40252.43 (10012.35 to 93466.86) | 34621.22 (8696.41 to 80275.31) | 29149.77 (7412.79 to 67486.50) | 23828.29 (6089.63 to 55123.28) | 16447.75 (4205.67 to 38053.84) | 11115.90 (2840.66 to 25720.13) | 4867.49 (1243.89 to 11260.26) | 1640.42 (419.21 to 3794.79) | 455.18 (116.37 to 1053.07) | 101.91 (26.04 to 235.79) |
| Burkina Faso | Male | Number of DALYs | 107.84 (61.04 to 168.23) | 2968.20 (1839.15 to 4493.67) | 4050.58 (2646.02 to 5785.70) | 3739.11 (2454.85 to 5225.99) | 2828.84 (1910.95 to 3950.20) | 2268.37 (1553.25 to 3124.56) | 1895.40 (1278.36 to 2611.25) | 1627.85 (1109.95 to 2293.66) | 1393.80 (952.75 to 1953.63) | 1236.11 (848.66 to 1682.87) | 1080.30 (728.09 to 1479.38) | 938.66 (630.75 to 1286.23) | 788.23 (545.96 to 1097.12) | 619.27 (431.08 to 850.96) | 417.80 (289.93 to 569.81) | 252.99 (175.63 to 341.84) | 102.48 (73.03 to 138.31) | 33.84 (23.95 to 45.17) | 7.24 (5.08 to 9.69) | 1.13 (0.81 to 1.49) |
| Burkina Faso | Male | Number of prevalence | 29936.96 (6844.25 to 70594.51) | 88159.89 (37383.68 to 178144.45) | 107822.24 (48687.91 to 212949.66) | 103148.23 (44999.02 to 204553.48) | 86996.59 (36042.33 to 175830.66) | 75708.88 (30343.06 to 155014.37) | 66927.34 (26255.06 to 138251.70) | 59896.73 (23155.32 to 124429.43) | 52993.36 (20185.13 to 110533.19) | 48366.68 (18264.19 to 101202.43) | 43399.05 (16288.37 to 91070.38) | 38740.25 (14457.79 to 81531.03) | 33490.18 (12438.93 to 70690.03) | 26981.15 (9997.86 to 57029.02) | 18562.49 (6873.81 to 39241.51) | 11540.52 (4275.27 to 24399.74) | 4744.24 (1758.36 to 10027.04) | 1602.85 (592.78 to 3388.15) | 348.88 (129.25 to 737.61) | 55.86 (20.68 to 118.10) |
| Cambodia | Both | Number of DALYs | 178.13 (109.06 to 267.77) | 562.05 (295.72 to 977.20) | 543.34 (299.28 to 969.80) | 515.55 (278.63 to 899.08) | 506.55 (276.88 to 873.20) | 490.92 (266.57 to 827.01) | 387.25 (222.11 to 637.51) | 353.71 (201.43 to 607.47) | 313.42 (178.53 to 522.37) | 245.92 (141.36 to 410.97) | 217.50 (125.24 to 362.38) | 191.29 (110.29 to 317.47) | 156.49 (90.46 to 253.57) | 113.91 (67.75 to 185.13) | 72.80 (41.77 to 117.95) | 42.16 (25.40 to 67.34) | 18.84 (11.58 to 29.22) | 6.62 (3.98 to 10.54) | 1.65 (1.02 to 2.58) | 0.28 (0.17 to 0.43) |
| Cambodia | Both | Number of prevalence | 10273.85 (1876.20 to 60814.99) | 23608.24 (4502.71 to 135879.27) | 26987.80 (4391.92 to 160623.55) | 29045.36 (4295.21 to 176092.82) | 30414.20 (4296.55 to 185747.17) | 30556.61 (4227.20 to 187319.11) | 25470.65 (3394.75 to 157169.66) | 22600.78 (3077.27 to 139045.68) | 19630.84 (2731.34 to 120423.45) | 15478.73 (2149.65 to 94959.99) | 13876.82 (1922.90 to 85209.64) | 12176.85 (1707.48 to 74707.46) | 10032.97 (1420.37 to 61494.84) | 7452.00 (1056.54 to 45649.68) | 5051.68 (695.35 to 31085.25) | 3050.20 (411.24 to 18823.34) | 1421.90 (188.64 to 8795.23) | 519.49 (68.28 to 3218.58) | 137.10 (17.59 to 852.60) | 26.67 (3.16 to 167.96) |
| Cambodia | Female | Number of DALYs | 103.64 (58.15 to 162.84) | 157.69 (93.56 to 251.51) | 136.73 (78.38 to 215.01) | 119.02 (71.55 to 181.63) | 109.12 (64.66 to 162.93) | 103.00 (62.77 to 151.85) | 89.63 (54.34 to 142.52) | 70.67 (44.08 to 107.63) | 57.14 (34.93 to 87.85) | 44.37 (25.97 to 68.07) | 39.45 (23.42 to 60.47) | 33.61 (20.87 to 50.69) | 26.57 (16.34 to 40.66) | 19.29 (11.82 to 29.00) | 13.42 (8.36 to 19.36) | 8.27 (5.21 to 12.01) | 3.83 (2.38 to 5.71) | 1.39 (0.85 to 2.01) | 0.38 (0.23 to 0.56) | 0.08 (0.05 to 0.12) |
| Cambodia | Female | Number of prevalence | 5205.32 (1068.00 to 30057.13) | 10758.58 (1671.92 to 65327.43) | 12532.93 (1511.42 to 78762.51) | 13780.53 (1354.89 to 88477.42) | 14801.30 (1282.58 to 96052.83) | 15317.18 (1231.74 to 99962.71) | 13902.79 (1069.16 to 91026.63) | 11806.98 (876.37 to 77478.63) | 9878.32 (714.81 to 64936.20) | 7850.39 (555.08 to 51676.20) | 7151.71 (497.42 to 47131.85) | 6229.29 (425.82 to 41099.38) | 5118.21 (342.17 to 33807.99) | 3800.31 (252.01 to 25116.08) | 2718.34 (180.41 to 17965.80) | 1697.00 (112.55 to 11215.37) | 812.07 (53.88 to 5366.93) | 301.74 (20.06 to 1994.15) | 82.82 (5.51 to 547.36) | 18.22 (1.21 to 120.43) |
| Cambodia | Male | Number of DALYs | 74.50 (41.28 to 118.13) | 404.37 (181.01 to 797.55) | 406.61 (191.87 to 782.68) | 396.52 (181.61 to 739.23) | 397.43 (191.69 to 727.85) | 387.92 (190.51 to 695.62) | 297.63 (153.28 to 531.67) | 283.04 (145.31 to 515.73) | 256.28 (133.65 to 452.38) | 201.55 (105.55 to 358.22) | 178.04 (95.15 to 316.63) | 157.68 (83.79 to 274.85) | 129.93 (70.99 to 220.74) | 94.63 (52.39 to 158.55) | 59.38 (32.11 to 101.44) | 33.89 (19.27 to 57.98) | 15.01 (8.44 to 24.39) | 5.23 (2.96 to 8.80) | 1.28 (0.72 to 2.18) | 0.19 (0.11 to 0.33) |
| Cambodia | Male | Number of prevalence | 5068.54 (797.68 to 30757.86) | 12849.66 (2718.92 to 70573.75) | 14454.87 (2772.93 to 81886.91) | 15264.83 (2803.01 to 87643.54) | 15612.90 (2864.06 to 89721.95) | 15239.43 (2868.67 to 87381.97) | 11567.86 (2210.97 to 66161.92) | 10793.80 (2104.81 to 61584.34) | 9752.52 (1928.49 to 55502.93) | 7628.34 (1537.03 to 43296.85) | 6725.11 (1373.40 to 38088.34) | 5947.56 (1235.46 to 33619.76) | 4914.76 (1037.20 to 27696.28) | 3651.69 (775.50 to 20540.09) | 2333.34 (495.32 to 13123.41) | 1353.19 (286.70 to 7610.16) | 609.83 (129.66 to 3429.26) | 217.76 (46.23 to 1224.82) | 54.28 (11.56 to 305.34) | 8.45 (1.79 to 47.54) |
| Cameroon | Both | Number of DALYs | 196.37 (116.64 to 292.72) | 638.98 (337.60 to 1115.08) | 632.10 (342.96 to 1139.22) | 640.94 (361.51 to 1098.27) | 625.72 (343.46 to 1062.74) | 605.31 (336.81 to 1007.75) | 569.21 (332.23 to 933.56) | 489.91 (274.21 to 803.06) | 420.91 (240.48 to 680.80) | 369.27 (211.77 to 599.31) | 335.68 (193.57 to 529.97) | 290.72 (174.73 to 467.83) | 252.94 (153.84 to 401.91) | 163.20 (103.80 to 251.30) | 110.98 (67.21 to 172.67) | 63.91 (39.77 to 96.98) | 34.61 (21.59 to 53.90) | 13.58 (8.46 to 20.70) | 3.61 (2.22 to 5.39) | 0.59 (0.37 to 0.87) |
| Cameroon | Both | Number of prevalence | 13918.85 (4164.80 to 37165.44) | 30347.04 (10235.27 to 78024.16) | 34628.69 (11097.16 to 90911.91) | 38089.36 (11930.49 to 101123.40) | 38849.96 (12050.05 to 103709.15) | 37468.79 (11600.95 to 99998.23) | 33856.95 (10562.83 to 90013.25) | 28467.09 (8921.72 to 75476.97) | 24139.28 (7596.85 to 63852.96) | 21054.84 (6574.77 to 55647.36) | 18981.32 (5928.60 to 50087.96) | 16275.66 (5113.10 to 42850.62) | 13978.09 (4405.65 to 36682.12) | 9293.88 (2905.97 to 24424.59) | 6456.19 (2018.73 to 16966.69) | 3795.21 (1185.43 to 9974.42) | 2080.20 (649.11 to 5468.99) | 862.71 (266.35 to 2274.78) | 248.30 (74.92 to 659.14) | 48.05 (13.85 to 129.30) |
| Cameroon | Female | Number of DALYs | 113.36 (58.22 to 177.68) | 164.23 (96.15 to 248.76) | 138.67 (82.32 to 207.27) | 126.82 (73.87 to 188.80) | 118.21 (72.53 to 179.18) | 103.85 (62.09 to 156.75) | 85.83 (49.95 to 128.57) | 67.23 (40.43 to 98.83) | 54.09 (34.22 to 79.24) | 46.65 (29.35 to 69.43) | 40.84 (25.79 to 59.48) | 33.20 (20.72 to 50.40) | 27.34 (17.58 to 40.13) | 18.08 (11.14 to 27.04) | 12.35 (7.71 to 17.88) | 7.21 (4.66 to 10.67) | 3.94 (2.61 to 5.72) | 1.67 (1.08 to 2.37) | 0.51 (0.32 to 0.75) | 0.11 (0.07 to 0.16) |
| Cameroon | Female | Number of prevalence | 6996.39 (2197.93 to 18391.85) | 13683.47 (3862.79 to 37058.10) | 15606.57 (4037.97 to 43153.78) | 17867.96 (4361.70 to 50021.04) | 19288.86 (4568.71 to 54338.64) | 18599.02 (4334.01 to 52567.91) | 16073.03 (3711.95 to 45513.43) | 13153.13 (3016.79 to 37291.36) | 10955.61 (2500.12 to 31092.34) | 9615.95 (2186.81 to 27309.74) | 8612.74 (1951.50 to 24477.38) | 7253.58 (1638.49 to 20626.33) | 6105.68 (1373.21 to 17373.67) | 4190.84 (942.07 to 11928.21) | 2909.79 (654.19 to 8282.26) | 1715.24 (385.43 to 4882.16) | 944.99 (212.41 to 2689.65) | 409.45 (92.02 to 1165.42) | 128.93 (28.96 to 366.97) | 29.34 (6.60 to 83.51) |
| Cameroon | Male | Number of DALYs | 83.01 (44.82 to 134.36) | 474.75 (214.09 to 930.80) | 493.43 (233.10 to 966.79) | 514.12 (254.24 to 941.09) | 507.51 (259.25 to 922.20) | 501.46 (251.41 to 879.36) | 483.38 (259.59 to 821.53) | 422.68 (228.14 to 720.14) | 366.82 (196.95 to 612.31) | 322.62 (181.79 to 545.86) | 294.84 (161.89 to 477.55) | 257.51 (150.31 to 422.46) | 225.60 (133.90 to 368.11) | 145.12 (87.54 to 228.36) | 98.63 (57.36 to 159.77) | 56.70 (34.47 to 88.46) | 30.67 (18.45 to 49.31) | 11.92 (7.14 to 18.79) | 3.10 (1.84 to 4.88) | 0.47 (0.28 to 0.73) |
| Cameroon | Male | Number of prevalence | 6922.46 (1954.16 to 18773.59) | 16663.57 (6099.55 to 41132.52) | 19022.13 (6732.70 to 47802.07) | 20221.40 (7125.23 to 51109.46) | 19561.10 (7003.77 to 49287.17) | 18869.77 (6866.72 to 47241.06) | 17783.92 (6525.51 to 44329.70) | 15313.96 (5683.50 to 38053.75) | 13183.67 (4956.41 to 32652.71) | 11438.89 (4339.73 to 28290.27) | 10368.58 (3968.21 to 25555.53) | 9022.08 (3477.60 to 22174.24) | 7872.41 (3050.08 to 19274.96) | 5103.04 (1983.53 to 12466.50) | 3546.40 (1379.05 to 8661.15) | 2079.97 (807.91 to 5082.04) | 1135.21 (441.23 to 2773.20) | 453.26 (176.31 to 1107.26) | 119.38 (46.38 to 291.59) | 18.71 (7.27 to 45.69) |
| Central African Republic | Both | Number of DALYs | 70.66 (45.50 to 107.26) | 897.03 (566.39 to 1338.49) | 1186.26 (771.94 to 1671.86) | 1226.01 (822.04 to 1689.90) | 1138.55 (775.09 to 1561.16) | 1033.56 (708.73 to 1428.42) | 873.19 (589.59 to 1181.78) | 676.82 (468.12 to 933.55) | 563.57 (387.50 to 772.91) | 486.57 (336.92 to 676.11) | 411.68 (287.71 to 567.82) | 362.42 (249.51 to 491.74) | 285.75 (198.40 to 393.80) | 203.16 (141.67 to 280.33) | 115.42 (80.47 to 155.04) | 52.67 (37.46 to 72.38) | 15.14 (10.76 to 20.45) | 5.61 (3.99 to 7.49) | 1.48 (1.06 to 2.01) | 0.33 (0.24 to 0.45) |
| Central African Republic | Both | Number of prevalence | 16864.48 (4664.34 to 40960.44) | 41086.44 (15344.57 to 90945.71) | 49528.32 (19165.84 to 108278.93) | 56399.36 (21336.44 to 124779.54) | 60300.14 (22033.24 to 135528.46) | 59078.11 (21122.97 to 133700.37) | 51559.73 (18218.69 to 116984.14) | 41386.96 (14501.05 to 94132.09) | 35382.84 (12318.94 to 80632.55) | 31565.92 (10895.51 to 72127.15) | 27346.60 (9391.75 to 62590.29) | 24137.55 (8312.16 to 55262.28) | 19165.93 (6637.09 to 43895.61) | 13762.94 (4771.37 to 31525.01) | 8331.73 (2856.19 to 19152.52) | 4079.50 (1381.39 to 9414.97) | 1180.22 (400.79 to 2720.61) | 516.60 (169.59 to 1202.76) | 147.81 (47.89 to 345.45) | 36.47 (11.66 to 85.54) |
| Central African Republic | Female | Number of DALYs | 41.06 (25.28 to 62.86) | 71.95 (43.36 to 109.90) | 79.83 (51.29 to 123.38) | 99.01 (64.23 to 143.25) | 122.64 (81.17 to 172.20) | 131.78 (87.91 to 195.95) | 123.29 (78.78 to 177.27) | 104.19 (69.63 to 151.25) | 94.90 (61.65 to 141.51) | 90.99 (59.45 to 133.67) | 83.76 (56.81 to 120.66) | 76.20 (50.83 to 111.25) | 62.20 (41.40 to 89.16) | 44.97 (29.82 to 65.39) | 29.07 (19.39 to 42.48) | 15.18 (10.18 to 21.53) | 4.18 (2.84 to 5.89) | 2.19 (1.50 to 3.08) | 0.65 (0.45 to 0.92) | 0.17 (0.12 to 0.23) |
| Central African Republic | Female | Number of prevalence | 8408.83 (2360.10 to 20345.21) | 17294.79 (4759.37 to 42028.77) | 20233.66 (5524.08 to 49246.98) | 24249.00 (6637.57 to 58952.26) | 27588.87 (7615.45 to 66915.19) | 27401.96 (7625.36 to 66294.39) | 23811.90 (6676.18 to 57482.43) | 19242.59 (5433.39 to 46364.22) | 16669.07 (4747.55 to 40093.63) | 15304.83 (4395.86 to 36764.82) | 13511.39 (3912.91 to 32417.32) | 11753.38 (3436.49 to 28168.85) | 9212.04 (2723.08 to 22047.69) | 6557.38 (1947.25 to 15684.90) | 4321.53 (1284.44 to 10336.34) | 2305.75 (685.37 to 5515.08) | 651.07 (193.42 to 1557.32) | 347.25 (103.19 to 830.55) | 106.01 (31.50 to 253.55) | 27.80 (8.26 to 66.49) |
| Central African Republic | Male | Number of DALYs | 29.61 (17.12 to 48.19) | 825.07 (517.73 to 1241.95) | 1106.43 (718.01 to 1572.40) | 1127.00 (762.58 to 1560.38) | 1015.91 (693.62 to 1402.43) | 901.78 (615.51 to 1259.95) | 749.89 (505.81 to 1018.27) | 572.63 (393.48 to 792.46) | 468.67 (319.84 to 648.51) | 395.58 (272.86 to 543.95) | 327.92 (226.96 to 455.26) | 286.22 (197.68 to 388.11) | 223.55 (154.57 to 303.99) | 158.19 (110.70 to 217.57) | 86.35 (59.69 to 116.01) | 37.49 (26.32 to 51.22) | 10.95 (7.71 to 14.79) | 3.42 (2.43 to 4.51) | 0.83 (0.59 to 1.09) | 0.17 (0.12 to 0.22) |
| Central African Republic | Male | Number of prevalence | 8455.65 (2297.23 to 20615.23) | 23791.65 (10669.17 to 48916.94) | 29294.67 (13666.35 to 59030.46) | 32150.36 (14651.46 to 65760.30) | 32711.27 (14202.75 to 68690.92) | 31676.14 (13467.50 to 67475.25) | 27747.83 (11541.98 to 59553.58) | 22144.37 (9058.55 to 47832.28) | 18713.77 (7557.00 to 40574.11) | 16261.10 (6494.19 to 35386.25) | 13835.20 (5473.69 to 30199.00) | 12384.17 (4873.32 to 27122.90) | 9953.89 (3892.73 to 21862.33) | 7205.57 (2810.33 to 15855.29) | 4010.20 (1566.61 to 8822.28) | 1773.75 (693.20 to 3901.85) | 529.16 (206.48 to 1163.71) | 169.35 (66.14 to 372.44) | 41.80 (16.34 to 91.95) | 8.67 (3.38 to 19.06) |
| Chad | Both | Number of DALYs | 116.28 (74.01 to 177.50) | 347.95 (192.17 to 644.07) | 329.32 (177.43 to 592.92) | 317.50 (170.11 to 555.22) | 296.82 (157.24 to 493.87) | 275.93 (152.11 to 473.57) | 242.63 (137.23 to 406.21) | 225.61 (127.34 to 378.53) | 176.43 (99.79 to 285.84) | 156.52 (90.90 to 253.91) | 132.68 (75.75 to 223.66) | 126.24 (74.18 to 207.51) | 93.94 (55.37 to 149.43) | 94.16 (55.89 to 150.54) | 76.43 (44.82 to 124.34) | 45.67 (26.75 to 71.26) | 21.40 (12.91 to 33.43) | 7.64 (4.56 to 11.99) | 1.98 (1.20 to 3.08) | 0.36 (0.23 to 0.55) |
| Chad | Both | Number of prevalence | 7297.47 (2066.52 to 22474.39) | 15523.43 (4845.30 to 45900.38) | 17230.36 (4967.02 to 52369.25) | 18494.35 (5148.24 to 57027.45) | 18114.91 (4944.58 to 56177.95) | 17022.61 (4571.08 to 52926.55) | 15160.67 (4069.98 to 47156.65) | 14098.07 (3782.31 to 43856.35) | 10943.57 (2929.79 to 33998.60) | 9643.89 (2579.21 to 29951.56) | 8111.01 (2181.44 to 25148.82) | 7545.36 (2052.45 to 23302.63) | 5731.09 (1542.58 to 17727.88) | 5492.17 (1519.39 to 16858.64) | 4417.38 (1235.86 to 13516.18) | 2793.82 (770.60 to 8584.45) | 1408.82 (378.24 to 4360.64) | 543.89 (142.57 to 1693.69) | 155.52 (39.34 to 488.60) | 33.40 (8.01 to 106.23) |
| Chad | Female | Number of DALYs | 67.06 (41.28 to 105.24) | 95.79 (58.87 to 147.30) | 80.86 (49.67 to 123.84) | 69.08 (41.02 to 105.72) | 60.02 (36.08 to 88.97) | 53.09 (32.31 to 79.30) | 44.22 (27.10 to 66.68) | 39.88 (25.01 to 62.06) | 29.16 (16.81 to 43.18) | 24.96 (14.55 to 36.36) | 20.22 (12.64 to 30.20) | 17.47 (10.79 to 25.60) | 13.45 (8.45 to 20.24) | 11.44 (7.28 to 17.09) | 8.55 (5.01 to 12.85) | 5.72 (3.58 to 8.54) | 3.11 (1.95 to 4.62) | 1.26 (0.78 to 1.81) | 0.39 (0.25 to 0.57) | 0.09 (0.06 to 0.14) |
| Chad | Female | Number of prevalence | 3701.31 (1127.91 to 11188.55) | 7139.08 (1875.27 to 22427.65) | 8029.95 (1870.75 to 25905.53) | 8780.72 (1888.94 to 28783.93) | 8783.74 (1808.55 to 29032.14) | 8413.04 (1690.22 to 27925.61) | 7551.71 (1497.13 to 25128.63) | 7041.13 (1381.18 to 23470.60) | 5398.42 (1051.50 to 18017.87) | 4735.42 (915.98 to 15821.05) | 3929.00 (755.73 to 13138.01) | 3508.29 (671.68 to 11741.54) | 2759.61 (526.18 to 9243.60) | 2397.78 (456.16 to 8035.16) | 1842.62 (350.14 to 6174.60) | 1236.37 (235.22 to 4143.15) | 687.12 (130.54 to 2302.54) | 285.68 (54.32 to 957.29) | 90.43 (17.19 to 303.02) | 21.99 (4.18 to 73.69) |
| Chad | Male | Number of DALYs | 49.22 (27.93 to 81.17) | 252.16 (114.09 to 525.02) | 248.47 (114.57 to 492.08) | 248.42 (121.43 to 470.59) | 236.80 (114.84 to 419.96) | 222.84 (111.75 to 407.63) | 198.41 (102.10 to 350.72) | 185.73 (98.15 to 332.14) | 147.26 (78.97 to 250.51) | 131.56 (71.82 to 220.44) | 112.46 (60.40 to 195.88) | 108.78 (59.46 to 184.57) | 80.49 (45.79 to 132.90) | 82.72 (46.67 to 136.96) | 67.87 (38.07 to 114.15) | 39.95 (22.74 to 65.09) | 18.29 (10.40 to 29.94) | 6.38 (3.57 to 10.34) | 1.58 (0.91 to 2.62) | 0.27 (0.16 to 0.44) |
| Chad | Male | Number of prevalence | 3596.16 (947.00 to 11285.84) | 8384.35 (2759.39 to 23680.11) | 9200.41 (2849.12 to 26526.12) | 9713.63 (2971.34 to 28266.33) | 9331.17 (2866.69 to 27163.52) | 8609.57 (2701.21 to 25016.08) | 7608.96 (2410.60 to 22039.96) | 7056.94 (2263.88 to 20395.11) | 5545.15 (1797.23 to 15988.06) | 4908.46 (1604.38 to 14135.63) | 4182.01 (1380.45 to 12014.80) | 4037.07 (1346.27 to 11565.01) | 2971.48 (1001.85 to 8486.59) | 3094.39 (1048.55 to 8826.03) | 2574.76 (871.99 to 7343.56) | 1557.45 (527.71 to 4442.58) | 721.70 (244.32 to 2058.71) | 258.21 (87.55 to 736.60) | 65.09 (22.01 to 185.63) | 11.41 (3.86 to 32.55) |
| Comoros | Both | Number of DALYs | 9.38 (6.02 to 14.23) | 60.93 (33.51 to 100.00) | 88.41 (54.10 to 139.59) | 115.98 (74.20 to 171.04) | 120.33 (78.98 to 174.10) | 109.99 (71.85 to 154.36) | 90.67 (60.54 to 127.26) | 78.51 (52.30 to 108.80) | 67.01 (44.51 to 94.40) | 59.26 (40.33 to 81.61) | 51.77 (34.93 to 71.83) | 42.01 (27.64 to 57.68) | 34.05 (23.26 to 46.71) | 26.10 (18.04 to 35.54) | 17.09 (11.40 to 23.02) | 9.69 (6.64 to 13.14) | 3.79 (2.62 to 5.15) | 1.31 (0.93 to 1.74) | 0.26 (0.18 to 0.34) | 0.04 (0.03 to 0.06) |
| Comoros | Both | Number of prevalence | 1437.01 (121.68 to 7324.85) | 3444.45 (543.02 to 16151.67) | 4525.13 (801.83 to 20971.05) | 5300.55 (1043.44 to 24190.61) | 5247.47 (1102.21 to 23796.00) | 4771.05 (1015.89 to 21643.66) | 4010.14 (850.79 to 18278.96) | 3492.18 (741.72 to 15973.89) | 2986.72 (632.62 to 13699.08) | 2665.15 (568.39 to 12258.77) | 2336.93 (499.36 to 10773.35) | 1911.88 (410.27 to 8839.20) | 1613.78 (337.26 to 7528.55) | 1260.63 (263.92 to 5895.05) | 830.96 (175.31 to 3878.05) | 485.40 (101.50 to 2269.29) | 209.63 (41.14 to 992.03) | 79.27 (14.70 to 378.78) | 17.54 (2.98 to 84.93) | 3.43 (0.52 to 16.85) |
| Comoros | Female | Number of DALYs | 5.42 (3.08 to 8.31) | 8.99 (5.23 to 13.69) | 9.31 (5.66 to 13.79) | 9.47 (6.20 to 14.10) | 8.85 (5.61 to 13.26) | 8.07 (5.17 to 12.24) | 6.83 (4.33 to 9.82) | 5.98 (3.85 to 8.82) | 5.05 (3.19 to 7.34) | 4.48 (2.94 to 6.75) | 3.83 (2.54 to 5.50) | 3.07 (2.02 to 4.45) | 2.67 (1.74 to 3.77) | 2.07 (1.33 to 2.96) | 1.32 (0.87 to 1.90) | 0.77 (0.50 to 1.10) | 0.36 (0.24 to 0.51) | 0.15 (0.10 to 0.20) | 0.03 (0.02 to 0.05) | 0.01 (0.01 to 0.01) |
| Comoros | Female | Number of prevalence | 716.13 (67.62 to 3618.25) | 1531.23 (119.07 to 7850.66) | 1961.59 (132.68 to 10149.02) | 2254.79 (141.04 to 11717.44) | 2245.14 (136.15 to 11686.71) | 2076.60 (124.86 to 10821.46) | 1784.82 (107.02 to 9322.63) | 1552.32 (93.65 to 8128.76) | 1316.32 (79.57 to 6907.46) | 1160.63 (70.57 to 6106.09) | 997.51 (60.90 to 5261.24) | 801.11 (49.35 to 4237.50) | 697.10 (43.19 to 3699.27) | 541.65 (33.72 to 2879.06) | 350.54 (21.84 to 1863.25) | 208.25 (13.00 to 1106.88) | 100.50 (6.26 to 534.17) | 41.29 (2.57 to 219.45) | 10.14 (0.63 to 53.88) | 2.21 (0.14 to 11.72) |
| Comoros | Male | Number of DALYs | 3.96 (2.23 to 6.48) | 51.94 (27.59 to 89.52) | 79.10 (47.16 to 129.02) | 106.51 (67.62 to 159.59) | 111.48 (72.47 to 162.76) | 101.91 (65.92 to 143.43) | 83.84 (55.66 to 118.39) | 72.53 (47.93 to 100.02) | 61.95 (41.16 to 87.57) | 54.78 (36.87 to 76.13) | 47.94 (32.06 to 66.81) | 38.94 (25.57 to 54.01) | 31.38 (21.37 to 43.35) | 24.03 (16.59 to 32.89) | 15.77 (10.46 to 21.41) | 8.92 (6.13 to 12.12) | 3.43 (2.37 to 4.66) | 1.16 (0.82 to 1.55) | 0.22 (0.16 to 0.29) | 0.04 (0.03 to 0.05) |
| Comoros | Male | Number of prevalence | 720.89 (53.32 to 3706.59) | 1913.22 (406.35 to 8274.95) | 2563.54 (644.09 to 10788.97) | 3045.76 (876.38 to 12435.25) | 3002.33 (920.57 to 12072.57) | 2694.44 (856.35 to 10808.10) | 2225.32 (709.89 to 8952.26) | 1939.87 (619.11 to 7841.30) | 1670.40 (536.64 to 6790.78) | 1504.52 (480.37 to 6150.83) | 1339.42 (425.02 to 5510.55) | 1110.77 (352.01 to 4601.70) | 916.68 (288.52 to 3829.28) | 718.97 (225.39 to 3015.99) | 480.41 (150.77 to 2014.80) | 277.15 (86.95 to 1162.41) | 109.13 (34.17 to 457.86) | 37.98 (11.92 to 159.33) | 7.40 (2.32 to 31.04) | 1.22 (0.38 to 5.13) |
| Congo | Both | Number of DALYs | 39.43 (25.25 to 56.98) | 144.25 (74.45 to 259.11) | 163.57 (87.76 to 289.18) | 183.68 (98.50 to 319.57) | 188.15 (102.94 to 318.67) | 177.84 (101.65 to 292.09) | 152.61 (90.03 to 246.86) | 131.82 (77.05 to 209.72) | 99.99 (58.60 to 157.51) | 93.95 (57.19 to 149.65) | 84.03 (51.13 to 129.99) | 72.18 (44.33 to 112.96) | 59.00 (36.70 to 89.71) | 45.16 (28.32 to 67.69) | 31.01 (19.56 to 46.51) | 16.80 (10.24 to 25.30) | 6.45 (4.06 to 9.66) | 1.45 (0.93 to 2.12) | 0.13 (0.09 to 0.19) | 0.03 (0.02 to 0.04) |
| Congo | Both | Number of prevalence | 3010.40 (685.78 to 10721.51) | 7159.98 (1783.29 to 24976.49) | 9223.26 (2157.26 to 32715.62) | 10842.88 (2502.17 to 38592.01) | 11044.72 (2578.29 to 39226.69) | 10171.35 (2407.97 to 36003.82) | 8678.86 (2080.43 to 30642.06) | 7514.93 (1815.95 to 26503.45) | 5660.22 (1379.81 to 19922.05) | 5227.59 (1293.94 to 18348.53) | 4730.33 (1172.02 to 16600.13) | 4170.84 (1022.40 to 14665.75) | 3511.18 (855.87 to 12355.25) | 2718.62 (663.91 to 9562.93) | 1903.97 (465.23 to 6697.06) | 1040.23 (255.21 to 3655.47) | 415.44 (100.79 to 1463.44) | 100.45 (23.80 to 355.76) | 10.86 (2.40 to 39.05) | 2.43 (0.51 to 8.82) |
| Congo | Female | Number of DALYs | 23.12 (13.71 to 36.02) | 37.17 (20.88 to 56.48) | 35.73 (20.26 to 55.00) | 33.25 (19.47 to 52.18) | 29.25 (17.55 to 46.05) | 24.49 (15.15 to 37.58) | 19.82 (12.11 to 29.94) | 16.85 (10.36 to 24.54) | 12.24 (7.41 to 18.76) | 11.01 (6.83 to 16.62) | 9.90 (6.30 to 15.11) | 8.96 (5.54 to 13.43) | 7.61 (4.86 to 11.17) | 5.83 (3.83 to 8.55) | 4.00 (2.56 to 5.72) | 2.15 (1.39 to 3.13) | 0.86 (0.56 to 1.25) | 0.22 (0.13 to 0.32) | 0.03 (0.02 to 0.04) | 0.01 (0.00 to 0.01) |
| Congo | Female | Number of prevalence | 1536.68 (372.07 to 5395.00) | 3328.23 (695.12 to 12061.24) | 4281.59 (788.90 to 15849.52) | 5010.52 (850.98 to 18781.54) | 5059.15 (820.45 to 19085.48) | 4607.09 (728.53 to 17438.04) | 3948.85 (616.62 to 14974.19) | 3480.76 (538.18 to 13215.59) | 2627.02 (403.12 to 9984.46) | 2386.53 (363.93 to 9076.72) | 2208.06 (335.06 to 8403.41) | 2038.26 (308.16 to 7762.19) | 1772.99 (266.58 to 6756.02) | 1387.39 (208.06 to 5288.02) | 968.34 (145.19 to 3690.78) | 525.54 (78.84 to 2003.16) | 214.97 (32.24 to 819.40) | 55.11 (8.27 to 210.06) | 6.95 (1.04 to 26.48) | 1.69 (0.25 to 6.44) |
| Congo | Male | Number of DALYs | 16.31 (9.25 to 25.36) | 107.09 (47.79 to 214.63) | 127.85 (61.05 to 243.73) | 150.43 (74.22 to 273.79) | 158.90 (82.38 to 278.89) | 153.35 (84.56 to 263.36) | 132.79 (73.67 to 225.91) | 114.97 (65.26 to 192.39) | 87.75 (49.95 to 141.01) | 82.94 (48.03 to 134.88) | 74.13 (43.80 to 117.20) | 63.22 (38.44 to 99.91) | 51.40 (30.87 to 80.68) | 39.33 (23.89 to 61.08) | 27.01 (16.65 to 41.56) | 14.66 (8.61 to 22.83) | 5.59 (3.45 to 8.50) | 1.24 (0.79 to 1.87) | 0.10 (0.06 to 0.16) | 0.02 (0.01 to 0.03) |
| Congo | Male | Number of prevalence | 1473.72 (305.40 to 5326.51) | 3831.74 (1076.67 to 12843.82) | 4941.67 (1341.44 to 16676.18) | 5832.36 (1620.55 to 19662.26) | 5985.56 (1707.58 to 20028.78) | 5564.26 (1641.34 to 18490.98) | 4730.01 (1437.52 to 15619.30) | 4034.17 (1248.93 to 13258.19) | 3033.20 (956.85 to 9920.04) | 2841.05 (906.37 to 9258.90) | 2522.27 (813.04 to 8189.62) | 2132.58 (695.45 to 6903.54) | 1738.19 (573.65 to 5599.23) | 1331.23 (444.29 to 4274.90) | 935.63 (312.11 to 3006.28) | 514.69 (171.36 to 1652.31) | 200.47 (66.77 to 644.04) | 45.34 (15.10 to 145.70) | 3.91 (1.30 to 12.57) | 0.74 (0.25 to 2.38) |
| Côte d'Ivoire | Both | Number of DALYs | 427.88 (274.74 to 622.77) | 7525.04 (4969.49 to 10637.36) | 7110.59 (4873.10 to 9737.57) | 6370.70 (4311.16 to 8800.42) | 6680.49 (4575.12 to 9186.74) | 6748.67 (4585.47 to 9410.03) | 6120.63 (4176.50 to 8743.99) | 5011.10 (3460.35 to 7015.51) | 4192.83 (2843.70 to 5872.62) | 3546.70 (2416.08 to 5007.97) | 2957.93 (2008.67 to 4207.30) | 2449.81 (1684.84 to 3475.49) | 1980.27 (1362.30 to 2746.88) | 1340.27 (911.04 to 1864.93) | 815.22 (560.70 to 1139.36) | 412.25 (287.69 to 570.42) | 198.75 (137.29 to 271.76) | 74.01 (52.20 to 101.23) | 16.95 (11.91 to 23.34) | 3.99 (2.78 to 5.38) |
| Côte d'Ivoire | Both | Number of prevalence | 121237.32 (39221.32 to 254966.48) | 312538.96 (136472.48 to 599882.82) | 342266.39 (144637.45 to 664592.08) | 370663.33 (151666.70 to 730005.33) | 416391.24 (169046.00 to 823123.01) | 419097.47 (170593.49 to 826727.83) | 368551.35 (151049.94 to 725473.76) | 292986.29 (121309.78 to 575775.53) | 240472.30 (100443.17 to 462100.84) | 199672.36 (84421.72 to 369297.31) | 163165.78 (69896.25 to 289979.65) | 131626.97 (57231.75 to 224849.41) | 103718.48 (45898.94 to 169837.97) | 70058.75 (31281.86 to 112440.55) | 43510.76 (19421.55 to 69855.48) | 22772.75 (10140.35 to 36627.15) | 11258.69 (4999.19 to 18139.34) | 4342.37 (1921.14 to 7014.35) | 1064.38 (466.02 to 1731.07) | 267.06 (115.95 to 436.89) |
| Côte d'Ivoire | Female | Number of DALYs | 245.88 (157.19 to 353.35) | 503.24 (312.21 to 756.39) | 625.16 (414.45 to 941.11) | 912.37 (610.45 to 1342.69) | 1350.45 (899.02 to 1977.77) | 1587.63 (1031.27 to 2322.66) | 1527.31 (992.92 to 2228.83) | 1299.52 (837.72 to 1923.22) | 1144.62 (745.30 to 1668.78) | 1029.27 (663.92 to 1508.90) | 891.68 (577.99 to 1308.60) | 750.98 (490.37 to 1103.31) | 624.72 (407.07 to 912.87) | 426.09 (279.39 to 621.38) | 262.50 (168.47 to 379.81) | 142.21 (92.03 to 208.76) | 73.00 (47.55 to 106.28) | 29.69 (19.81 to 42.59) | 8.37 (5.63 to 11.93) | 2.32 (1.56 to 3.22) |
| Côte d'Ivoire | Female | Number of prevalence | 59897.71 (19587.85 to 125524.36) | 127314.10 (41530.93 to 267076.61) | 144577.27 (47648.71 to 302603.95) | 168512.32 (57058.36 to 350478.76) | 197228.63 (69162.83 to 406815.74) | 195813.69 (70872.17 to 400708.00) | 165783.13 (61502.87 to 337267.36) | 128119.76 (48507.53 to 259605.08) | 104706.92 (40382.27 to 211507.35) | 87865.90 (34476.80 to 171202.80) | 71713.14 (28652.30 to 133835.10) | 57096.43 (23225.13 to 102148.31) | 44818.40 (18614.39 to 76618.18) | 30104.62 (12646.50 to 50367.79) | 18888.26 (7933.06 to 31602.01) | 10436.89 (4382.06 to 17462.10) | 5420.46 (2276.55 to 9069.08) | 2242.60 (941.96 to 3752.14) | 647.96 (271.98 to 1084.11) | 183.91 (77.29 to 307.69) |
| Côte d'Ivoire | Male | Number of DALYs | 182.01 (109.97 to 279.45) | 7021.80 (4638.76 to 9913.33) | 6485.43 (4402.20 to 8938.84) | 5458.33 (3678.21 to 7457.50) | 5330.04 (3634.10 to 7511.80) | 5161.04 (3527.30 to 7103.91) | 4593.31 (3123.37 to 6403.72) | 3711.58 (2572.21 to 5118.93) | 3048.21 (2079.33 to 4228.10) | 2517.43 (1719.01 to 3530.87) | 2066.25 (1427.91 to 2892.39) | 1698.83 (1177.90 to 2381.32) | 1355.56 (930.94 to 1890.46) | 914.18 (633.54 to 1267.22) | 552.72 (385.29 to 763.22) | 270.05 (192.29 to 378.18) | 125.75 (87.95 to 173.21) | 44.32 (31.87 to 61.08) | 8.58 (6.17 to 11.61) | 1.67 (1.21 to 2.23) |
| Côte d'Ivoire | Male | Number of prevalence | 61339.61 (19633.47 to 129442.12) | 185224.86 (95224.78 to 331406.17) | 197689.12 (96970.58 to 361988.13) | 202151.01 (93999.46 to 379415.17) | 219162.61 (98806.16 to 416028.08) | 223283.78 (99363.49 to 425643.49) | 202768.22 (89484.67 to 387758.04) | 164866.53 (72782.90 to 315787.71) | 135765.38 (60069.50 to 250593.49) | 111806.47 (49942.87 to 198094.51) | 91452.64 (41241.26 to 156144.54) | 74530.55 (34009.47 to 122701.10) | 58900.08 (27266.46 to 93219.79) | 39954.13 (18637.24 to 62072.76) | 24622.50 (11489.08 to 38253.48) | 12335.87 (5763.03 to 19165.05) | 5838.23 (2723.44 to 9070.26) | 2099.77 (979.24 to 3262.21) | 416.42 (194.36 to 646.96) | 83.16 (38.82 to 129.20) |
| Democratic Republic of the Congo | Both | Number of DALYs | 878.08 (554.20 to 1303.46) | 7023.55 (4160.47 to 11443.66) | 9938.76 (6402.07 to 15006.00) | 12757.40 (8505.39 to 18088.48) | 13282.48 (8761.70 to 18378.04) | 11980.98 (7968.13 to 16598.25) | 9938.39 (6680.57 to 13572.07) | 8307.88 (5607.79 to 11649.25) | 5614.29 (3845.40 to 7643.90) | 4848.16 (3349.80 to 6670.99) | 4288.83 (2955.40 to 5849.84) | 3725.39 (2609.09 to 5074.61) | 2921.59 (2008.87 to 3993.86) | 2134.48 (1488.90 to 2851.25) | 1420.78 (1005.49 to 1903.46) | 710.56 (504.16 to 960.78) | 236.88 (168.91 to 321.29) | 75.08 (53.98 to 99.48) | 20.44 (14.77 to 26.87) | 4.22 (3.06 to 5.54) |
| Democratic Republic of the Congo | Both | Number of prevalence | 163249.48 (80222.36 to 281778.89) | 378242.15 (206290.43 to 633463.87) | 464184.23 (259302.70 to 768452.35) | 544101.86 (308687.32 to 889081.74) | 570313.38 (322573.29 to 927612.95) | 538484.61 (301725.71 to 876389.69) | 467001.91 (259788.07 to 761756.34) | 404051.38 (224123.58 to 660570.12) | 294963.68 (162167.08 to 484494.46) | 263876.08 (144667.04 to 434407.94) | 242584.61 (132482.85 to 400288.13) | 217762.83 (118475.10 to 359739.58) | 176092.86 (95524.60 to 291372.51) | 130825.75 (70936.70 to 216461.60) | 86082.74 (46825.18 to 142200.05) | 42080.33 (23036.07 to 69320.83) | 13977.15 (7687.10 to 22970.52) | 4677.53 (2560.01 to 7705.72) | 1382.02 (749.25 to 2286.56) | 334.09 (177.98 to 557.08) |
| Democratic Republic of the Congo | Female | Number of DALYs | 504.23 (304.15 to 765.58) | 833.89 (502.47 to 1357.02) | 827.21 (504.92 to 1216.16) | 873.67 (586.25 to 1301.01) | 913.50 (560.90 to 1341.19) | 894.27 (593.22 to 1292.16) | 800.63 (500.52 to 1159.30) | 707.48 (475.44 to 1026.13) | 561.20 (368.48 to 814.27) | 514.17 (337.66 to 710.09) | 493.57 (325.31 to 699.31) | 455.70 (310.71 to 652.32) | 380.91 (254.47 to 549.40) | 278.77 (191.66 to 396.15) | 173.24 (114.54 to 249.06) | 77.26 (51.71 to 108.90) | 23.69 (16.00 to 32.56) | 8.21 (5.69 to 11.44) | 2.63 (1.85 to 3.62) | 0.74 (0.50 to 1.05) |
| Democratic Republic of the Congo | Female | Number of prevalence | 81594.97 (40416.11 to 140213.04) | 166752.25 (81464.88 to 288237.16) | 197209.68 (95549.47 to 342017.32) | 224645.09 (108459.72 to 390091.33) | 237147.18 (114503.09 to 411841.01) | 229936.08 (111110.75 to 399229.32) | 203376.88 (98405.10 to 352960.73) | 177251.50 (85827.29 to 307528.60) | 137982.23 (66899.33 to 239288.60) | 124956.03 (60627.33 to 216641.30) | 117091.39 (56843.67 to 202929.34) | 106011.71 (51503.26 to 183657.13) | 86072.02 (41859.75 to 149040.72) | 63344.94 (30823.73 to 109650.35) | 40123.87 (19525.13 to 69460.80) | 18218.69 (8866.13 to 31538.54) | 5743.82 (2795.07 to 9942.48) | 2037.49 (991.15 to 3526.96) | 666.67 (324.35 to 1154.12) | 190.94 (92.91 to 330.52) |
| Democratic Republic of the Congo | Male | Number of DALYs | 373.85 (211.18 to 588.83) | 6189.66 (3493.16 to 10459.96) | 9111.55 (5747.96 to 13987.05) | 11883.73 (7831.57 to 17093.93) | 12368.98 (8136.63 to 17097.09) | 11086.71 (7313.56 to 15370.82) | 9137.76 (6150.21 to 12632.43) | 7600.40 (5111.58 to 10663.33) | 5053.09 (3453.47 to 6916.35) | 4333.99 (2977.95 to 5932.68) | 3795.27 (2596.46 to 5237.22) | 3269.69 (2287.46 to 4437.62) | 2540.68 (1744.83 to 3484.77) | 1855.71 (1280.14 to 2507.87) | 1247.54 (875.58 to 1684.26) | 633.31 (448.73 to 857.43) | 213.19 (151.75 to 287.94) | 66.87 (47.82 to 89.74) | 17.81 (12.76 to 23.30) | 3.48 (2.50 to 4.61) |
| Democratic Republic of the Congo | Male | Number of prevalence | 81654.51 (39806.25 to 141565.85) | 211489.89 (126259.38 to 343112.95) | 266974.55 (164573.65 to 423099.06) | 319456.78 (201935.20 to 494931.17) | 333166.20 (210746.22 to 516268.40) | 308548.54 (192889.75 to 479891.20) | 263625.03 (162385.29 to 410533.48) | 226799.88 (138629.95 to 353708.79) | 156981.45 (95332.14 to 245471.25) | 138920.05 (83776.87 to 217771.70) | 125493.21 (75247.23 to 197358.79) | 111751.12 (66749.10 to 176082.45) | 90020.84 (53549.19 to 142331.79) | 67480.81 (40041.13 to 106811.25) | 45958.87 (27255.95 to 72739.25) | 23861.63 (14140.24 to 37782.28) | 8233.33 (4884.13 to 13028.04) | 2640.04 (1566.30 to 4178.76) | 715.35 (424.41 to 1132.44) | 143.15 (84.94 to 226.56) |
| Dominican Republic | Both | Number of DALYs | 104.11 (65.09 to 156.71) | 429.86 (234.71 to 798.05) | 558.50 (313.33 to 958.29) | 745.92 (421.37 to 1243.60) | 876.70 (523.20 to 1421.76) | 879.95 (543.05 to 1383.15) | 785.90 (479.30 to 1195.55) | 693.80 (439.28 to 1057.15) | 588.95 (378.49 to 881.14) | 492.79 (317.22 to 725.46) | 434.95 (278.11 to 627.34) | 389.83 (253.88 to 568.92) | 334.09 (217.27 to 486.72) | 246.88 (161.85 to 355.95) | 178.31 (117.04 to 258.00) | 122.93 (82.45 to 178.52) | 76.53 (50.59 to 108.03) | 29.93 (19.87 to 43.41) | 6.21 (4.23 to 8.82) | 1.85 (1.26 to 2.66) |
| Dominican Republic | Both | Number of prevalence | 9895.70 (1683.68 to 40289.76) | 23557.62 (5363.91 to 91681.39) | 32698.32 (7256.31 to 128606.70) | 42969.64 (9646.95 to 168875.41) | 48582.65 (11131.58 to 189873.25) | 46845.20 (10925.15 to 182041.51) | 40599.06 (9587.02 to 157066.69) | 35177.59 (8404.15 to 135683.91) | 29111.27 (7052.34 to 111760.13) | 23926.51 (5853.63 to 91527.28) | 20902.70 (5165.21 to 79765.16) | 18602.15 (4672.87 to 70771.26) | 15713.13 (4017.49 to 59579.90) | 11745.96 (3015.48 to 44495.46) | 8687.57 (2211.45 to 32964.88) | 6034.12 (1548.57 to 22860.29) | 3827.43 (983.27 to 14499.42) | 1543.42 (392.93 to 5857.05) | 335.78 (84.07 to 1279.42) | 103.82 (25.74 to 396.26) |
| Dominican Republic | Female | Number of DALYs | 60.37 (36.06 to 91.64) | 97.69 (57.34 to 144.98) | 104.06 (59.77 to 158.99) | 111.22 (64.27 to 170.94) | 111.05 (68.06 to 165.12) | 97.75 (58.09 to 148.01) | 81.55 (50.30 to 118.40) | 68.45 (41.12 to 104.18) | 54.19 (34.14 to 79.96) | 42.90 (25.82 to 65.33) | 37.07 (23.43 to 54.16) | 31.94 (19.63 to 47.25) | 26.29 (16.85 to 38.42) | 19.29 (12.16 to 28.94) | 14.37 (9.52 to 21.15) | 9.72 (6.11 to 14.28) | 5.98 (3.94 to 8.77) | 2.42 (1.52 to 3.62) | 0.53 (0.35 to 0.77) | 0.16 (0.11 to 0.24) |
| Dominican Republic | Female | Number of prevalence | 4978.67 (914.73 to 20020.22) | 10805.58 (1683.91 to 44561.07) | 15184.35 (2066.26 to 63683.87) | 20047.07 (2515.41 to 84890.19) | 22574.83 (2708.11 to 96036.30) | 21507.96 (2526.78 to 91714.80) | 18485.95 (2142.89 to 78930.41) | 15940.82 (1833.84 to 68121.10) | 12918.40 (1476.46 to 55241.22) | 10449.56 (1189.80 to 44704.59) | 9077.49 (1028.86 to 38850.50) | 8000.34 (903.95 to 34254.35) | 6654.26 (748.54 to 28500.22) | 4986.45 (560.36 to 21360.29) | 3746.25 (420.91 to 16047.47) | 2559.76 (287.53 to 10965.26) | 1620.10 (182.09 to 6939.94) | 666.14 (74.88 to 2853.53) | 150.77 (16.94 to 645.85) | 47.46 (5.33 to 203.28) |
| Dominican Republic | Male | Number of DALYs | 43.74 (24.99 to 70.57) | 332.17 (153.19 to 683.02) | 454.44 (229.97 to 815.06) | 634.71 (342.52 to 1088.19) | 765.65 (443.81 to 1281.00) | 782.21 (462.53 to 1255.69) | 704.35 (420.22 to 1091.78) | 625.35 (388.02 to 967.63) | 534.76 (335.42 to 810.94) | 449.89 (281.96 to 668.75) | 397.88 (252.61 to 584.63) | 357.89 (228.94 to 531.07) | 307.80 (196.88 to 456.05) | 227.59 (145.64 to 329.26) | 163.93 (106.63 to 239.16) | 113.21 (74.73 to 166.30) | 70.55 (45.90 to 100.80) | 27.51 (18.01 to 40.11) | 5.68 (3.81 to 8.18) | 1.69 (1.13 to 2.44) |
| Dominican Republic | Male | Number of prevalence | 4917.03 (773.14 to 20269.55) | 12752.04 (3358.81 to 46611.75) | 17513.97 (4676.59 to 64314.39) | 22922.57 (6502.02 to 83252.46) | 26007.83 (7903.53 to 93107.10) | 25337.24 (8076.83 to 89673.36) | 22113.11 (7183.20 to 77602.85) | 19236.77 (6355.81 to 67091.84) | 16192.87 (5406.60 to 56182.15) | 13476.94 (4552.83 to 46556.35) | 11825.21 (4031.27 to 40697.61) | 10601.81 (3646.74 to 36371.73) | 9058.87 (3149.53 to 30970.36) | 6759.51 (2366.24 to 23057.15) | 4941.32 (1726.22 to 16862.20) | 3474.35 (1212.76 to 11857.32) | 2207.33 (771.54 to 7533.58) | 877.27 (306.10 to 2993.43) | 185.01 (64.59 to 631.35) | 56.36 (19.75 to 192.40) |
| Egypt | Both | Number of DALYs | 841.19 (530.37 to 1242.05) | 2550.46 (1356.19 to 4700.70) | 2702.11 (1453.27 to 5026.77) | 2806.41 (1514.55 to 5152.93) | 2797.18 (1490.87 to 4925.61) | 2699.86 (1493.63 to 4641.95) | 2431.77 (1340.24 to 4152.33) | 2275.00 (1231.65 to 3951.23) | 1981.23 (1089.66 to 3418.61) | 1701.79 (948.39 to 2836.42) | 1464.40 (837.77 to 2512.37) | 1198.08 (669.10 to 1962.71) | 1003.60 (548.92 to 1661.36) | 705.18 (401.38 to 1159.88) | 421.19 (239.97 to 693.61) | 240.16 (138.15 to 399.25) | 119.57 (69.18 to 196.04) | 46.91 (26.48 to 76.31) | 11.61 (6.64 to 19.08) | 1.76 (1.00 to 3.00) |
| Egypt | Both | Number of prevalence | 45454.44 (10307.81 to 187189.22) | 101604.04 (26297.54 to 399293.01) | 128938.14 (29469.47 to 525745.53) | 150622.02 (31903.29 to 626300.51) | 157286.69 (31865.19 to 659656.96) | 156087.48 (31035.11 to 656788.32) | 142368.83 (28109.06 to 599822.85) | 133362.25 (26132.89 to 562255.55) | 116344.19 (22843.12 to 490243.38) | 100738.21 (19721.02 to 424713.55) | 86276.29 (16932.50 to 363533.74) | 71132.84 (13964.16 to 299706.55) | 60036.16 (11848.51 to 252631.30) | 43320.25 (8477.54 to 182574.96) | 25888.88 (5137.98 to 108844.80) | 14208.43 (2940.28 to 59279.80) | 7324.86 (1495.57 to 30637.01) | 2906.95 (597.46 to 12142.98) | 622.18 (141.83 to 2535.19) | 88.34 (21.07 to 354.36) |
| Egypt | Female | Number of DALYs | 484.40 (287.04 to 771.44) | 705.29 (402.85 to 1103.66) | 661.74 (368.35 to 1041.36) | 607.23 (341.87 to 913.37) | 548.61 (317.56 to 839.43) | 488.87 (277.50 to 734.69) | 419.34 (261.35 to 639.17) | 381.34 (239.20 to 587.32) | 316.33 (190.23 to 485.38) | 268.72 (165.55 to 417.88) | 221.21 (134.49 to 337.30) | 178.76 (111.24 to 269.74) | 144.55 (88.75 to 215.56) | 104.98 (66.64 to 160.51) | 59.77 (36.57 to 88.52) | 28.60 (17.98 to 41.96) | 15.29 (9.40 to 22.98) | 5.70 (3.60 to 8.58) | 0.77 (0.48 to 1.12) | 0.07 (0.04 to 0.10) |
| Egypt | Female | Number of prevalence | 22896.54 (5757.76 to 92222.07) | 45219.81 (9027.57 to 191167.79) | 56982.69 (9245.79 to 249216.84) | 66592.63 (9287.41 to 297262.15) | 69982.92 (8937.63 to 315742.22) | 69780.22 (8470.30 to 316598.11) | 63792.83 (7503.53 to 290360.98) | 59902.95 (6882.71 to 273266.97) | 51825.80 (5854.93 to 236811.86) | 45285.69 (5044.34 to 207200.76) | 38498.87 (4234.02 to 176367.39) | 31840.80 (3454.40 to 146024.12) | 26599.35 (2850.62 to 122127.88) | 19715.23 (2098.15 to 90568.28) | 11403.53 (1213.33 to 52389.32) | 5574.50 (594.71 to 25609.45) | 2994.28 (319.07 to 13756.16) | 1167.39 (124.58 to 5363.02) | 158.04 (16.85 to 726.03) | 14.39 (1.53 to 66.11) |
| Egypt | Male | Number of DALYs | 356.80 (191.11 to 562.25) | 1845.17 (808.38 to 3920.16) | 2040.37 (931.96 to 4293.98) | 2199.18 (1063.45 to 4394.84) | 2248.57 (1084.81 to 4268.72) | 2210.99 (1088.97 to 4097.61) | 2012.43 (1024.27 to 3679.80) | 1893.67 (941.16 to 3452.24) | 1664.90 (845.10 to 2994.46) | 1433.07 (738.87 to 2493.87) | 1243.19 (657.93 to 2238.25) | 1019.32 (537.82 to 1715.76) | 859.05 (448.31 to 1475.54) | 600.20 (315.93 to 997.19) | 361.42 (199.18 to 621.98) | 211.56 (113.40 to 363.26) | 104.29 (56.87 to 176.82) | 41.21 (22.04 to 69.99) | 10.84 (5.86 to 18.16) | 1.70 (0.93 to 2.93) |
| Egypt | Male | Number of prevalence | 22557.89 (4523.12 to 94967.15) | 56384.23 (15669.82 to 208285.50) | 71955.44 (18041.63 to 276528.69) | 84029.38 (20154.19 to 329038.36) | 87303.77 (20885.60 to 343914.75) | 86307.26 (20707.13 to 340190.22) | 78576.00 (19028.51 to 309461.87) | 73459.30 (17930.27 to 288988.58) | 64518.38 (15910.68 to 253431.52) | 55452.52 (13782.48 to 217512.79) | 47777.41 (11974.18 to 187166.35) | 39292.04 (9920.41 to 153682.43) | 33436.81 (8524.37 to 130503.42) | 23605.02 (6058.41 to 92006.68) | 14485.34 (3704.51 to 56455.47) | 8633.93 (2215.58 to 33670.35) | 4330.58 (1108.14 to 16880.85) | 1739.55 (446.04 to 6779.96) | 464.14 (119.04 to 1809.15) | 73.95 (18.97 to 288.25) |
| Equatorial Guinea | Both | Number of DALYs | 9.41 (5.73 to 14.10) | 64.68 (37.37 to 107.37) | 87.44 (54.12 to 135.78) | 111.17 (73.19 to 159.89) | 114.39 (76.12 to 164.29) | 95.88 (64.17 to 132.77) | 81.86 (54.79 to 114.23) | 82.51 (55.53 to 115.53) | 55.46 (37.04 to 75.85) | 61.51 (41.55 to 83.74) | 54.80 (37.41 to 74.83) | 43.99 (30.08 to 59.71) | 33.34 (23.02 to 45.19) | 23.10 (15.74 to 31.53) | 14.63 (10.27 to 19.43) | 8.01 (5.71 to 10.65) | 3.33 (2.36 to 4.41) | 1.00 (0.70 to 1.33) | 0.21 (0.15 to 0.28) | 0.03 (0.02 to 0.04) |
| Equatorial Guinea | Both | Number of prevalence | 1591.67 (175.63 to 5722.44) | 3586.72 (696.37 to 11998.47) | 4361.81 (916.60 to 14367.45) | 5055.34 (1135.40 to 16402.65) | 5144.31 (1190.23 to 16645.55) | 4519.21 (1032.20 to 14708.16) | 3974.25 (897.75 to 12985.36) | 4019.37 (909.93 to 13133.31) | 2825.88 (618.10 to 9293.34) | 3076.95 (685.67 to 10091.74) | 2760.90 (614.64 to 9061.78) | 2275.87 (498.49 to 7498.07) | 1800.86 (385.97 to 5968.39) | 1322.34 (275.80 to 4409.32) | 877.73 (179.97 to 2936.18) | 499.52 (100.96 to 1675.24) | 218.29 (43.39 to 734.01) | 68.55 (13.41 to 231.18) | 15.71 (2.94 to 53.40) | 3.21 (0.53 to 11.13) |
| Equatorial Guinea | Female | Number of DALYs | 5.33 (3.16 to 8.09) | 8.36 (4.96 to 12.74) | 8.31 (5.28 to 12.47) | 8.81 (5.66 to 13.16) | 9.01 (5.77 to 13.37) | 8.28 (5.24 to 11.97) | 7.43 (4.80 to 11.18) | 7.31 (4.82 to 10.44) | 5.41 (3.56 to 8.02) | 5.62 (3.62 to 8.18) | 4.99 (3.29 to 7.01) | 4.13 (2.71 to 5.99) | 3.36 (2.22 to 4.88) | 2.56 (1.74 to 3.74) | 1.73 (1.17 to 2.44) | 0.99 (0.68 to 1.40) | 0.43 (0.29 to 0.61) | 0.14 (0.09 to 0.19) | 0.03 (0.02 to 0.05) | 0.01 (0.01 to 0.01) |
| Equatorial Guinea | Female | Number of prevalence | 774.61 (91.96 to 2764.33) | 1552.50 (162.65 to 5604.09) | 1882.68 (180.81 to 6842.76) | 2210.00 (203.85 to 8057.70) | 2332.79 (212.66 to 8512.65) | 2166.86 (197.26 to 7906.89) | 1941.96 (177.66 to 7084.09) | 1920.88 (176.49 to 7007.33) | 1394.76 (128.57 to 5089.42) | 1439.71 (133.48 to 5254.81) | 1259.32 (117.32 to 4598.39) | 1039.51 (97.34 to 3798.77) | 837.17 (78.90 to 3062.94) | 642.61 (60.77 to 2352.39) | 441.43 (41.78 to 1615.83) | 258.15 (24.44 to 944.96) | 115.90 (10.99 to 424.26) | 37.43 (3.54 to 137.02) | 9.22 (0.87 to 33.74) | 2.22 (0.21 to 8.12) |
| Equatorial Guinea | Male | Number of DALYs | 4.08 (2.31 to 6.55) | 56.32 (31.45 to 96.56) | 79.13 (46.62 to 124.23) | 102.36 (66.16 to 148.33) | 105.38 (69.84 to 151.39) | 87.61 (58.18 to 122.30) | 74.42 (49.92 to 104.39) | 75.20 (50.48 to 106.04) | 50.05 (33.22 to 68.54) | 55.89 (37.49 to 76.25) | 49.82 (33.56 to 68.09) | 39.86 (26.93 to 54.49) | 29.98 (20.60 to 40.19) | 20.54 (14.02 to 27.97) | 12.91 (8.91 to 17.32) | 7.02 (4.90 to 9.41) | 2.90 (2.06 to 3.89) | 0.86 (0.61 to 1.16) | 0.18 (0.13 to 0.23) | 0.03 (0.02 to 0.03) |
| Equatorial Guinea | Male | Number of prevalence | 817.06 (83.81 to 2958.11) | 2034.22 (513.04 to 6394.38) | 2479.13 (743.57 to 7524.70) | 2845.34 (946.18 to 8344.95) | 2811.52 (976.24 to 8132.90) | 2352.35 (829.09 to 6801.26) | 2032.29 (709.16 to 5901.27) | 2098.49 (723.89 to 6125.98) | 1431.12 (485.89 to 4203.92) | 1637.24 (549.72 to 4836.93) | 1501.58 (495.91 to 4463.39) | 1236.36 (401.18 to 3699.30) | 963.69 (305.84 to 2905.45) | 679.73 (213.14 to 2056.94) | 436.30 (137.36 to 1320.36) | 241.37 (75.87 to 730.29) | 102.40 (32.14 to 309.75) | 31.12 (9.80 to 94.17) | 6.50 (2.04 to 19.66) | 0.99 (0.31 to 3.01) |
| Eritrea | Both | Number of DALYs | 57.05 (35.48 to 85.33) | 155.42 (83.34 to 288.96) | 141.92 (75.74 to 258.53) | 125.51 (64.95 to 231.99) | 104.32 (53.17 to 188.04) | 86.69 (46.37 to 147.26) | 73.10 (38.88 to 130.09) | 61.31 (33.63 to 111.82) | 46.51 (24.48 to 80.39) | 46.79 (25.51 to 82.34) | 37.67 (20.03 to 66.46) | 27.41 (14.80 to 49.17) | 18.15 (10.17 to 32.44) | 10.74 (5.88 to 18.83) | 5.54 (3.12 to 9.27) | 2.46 (1.41 to 4.02) | 0.90 (0.51 to 1.42) | 0.25 (0.16 to 0.40) | 0.06 (0.03 to 0.09) | 0.01 (0.01 to 0.02) |
| Eritrea | Both | Number of prevalence | 1677.54 (601.58 to 6184.62) | 3810.58 (1305.82 to 13451.34) | 4308.73 (1239.24 to 16449.37) | 4581.54 (1141.38 to 18432.19) | 4374.86 (989.87 to 18130.61) | 3980.48 (851.74 to 16770.28) | 3475.88 (722.15 to 14784.61) | 3013.17 (612.91 to 12888.15) | 2351.85 (470.41 to 10105.21) | 2383.01 (473.15 to 10245.81) | 1947.38 (381.41 to 8397.96) | 1486.12 (286.94 to 6442.12) | 1034.83 (195.69 to 4513.07) | 650.26 (120.15 to 2853.99) | 354.89 (63.84 to 1566.50) | 166.81 (29.35 to 739.90) | 63.81 (11.02 to 284.10) | 19.11 (3.25 to 85.35) | 4.24 (0.72 to 18.94) | 0.95 (0.16 to 4.24) |
| Eritrea | Female | Number of DALYs | 32.59 (18.44 to 50.77) | 48.40 (27.62 to 77.46) | 41.93 (23.88 to 66.81) | 35.97 (20.89 to 55.03) | 30.91 (18.46 to 48.58) | 26.00 (15.04 to 40.33) | 21.68 (12.57 to 33.17) | 17.81 (10.17 to 27.94) | 13.38 (7.77 to 20.70) | 12.45 (7.55 to 18.81) | 10.01 (5.50 to 15.34) | 7.56 (4.65 to 11.12) | 5.40 (3.33 to 8.26) | 3.54 (2.05 to 5.42) | 2.07 (1.25 to 3.18) | 1.01 (0.61 to 1.46) | 0.40 (0.25 to 0.62) | 0.12 (0.08 to 0.19) | 0.03 (0.02 to 0.04) | 0.01 (0.00 to 0.01) |
| Eritrea | Female | Number of prevalence | 860.12 (334.70 to 3032.02) | 1630.37 (503.64 to 6306.77) | 1851.60 (456.69 to 7678.23) | 2022.41 (411.13 to 8767.40) | 2030.53 (365.23 to 9012.34) | 1916.78 (319.20 to 8618.40) | 1710.79 (272.54 to 7750.67) | 1483.29 (228.43 to 6755.10) | 1163.90 (174.20 to 5321.67) | 1128.28 (165.70 to 5176.18) | 925.50 (133.31 to 4257.54) | 734.32 (103.50 to 3387.29) | 537.16 (74.08 to 2484.97) | 363.50 (49.60 to 1683.92) | 214.54 (29.27 to 993.87) | 107.38 (14.63 to 497.42) | 43.03 (5.86 to 199.31) | 13.35 (1.82 to 61.83) | 2.95 (0.40 to 13.66) | 0.68 (0.09 to 3.15) |
| Eritrea | Male | Number of DALYs | 24.45 (14.07 to 39.94) | 107.02 (47.71 to 227.58) | 99.99 (45.12 to 210.75) | 89.54 (37.82 to 190.31) | 73.40 (32.42 to 151.19) | 60.69 (26.92 to 120.11) | 51.42 (23.06 to 103.49) | 43.50 (20.36 to 91.27) | 33.13 (14.28 to 64.75) | 34.34 (15.78 to 67.07) | 27.66 (12.72 to 54.93) | 19.85 (9.13 to 39.01) | 12.75 (6.02 to 25.48) | 7.20 (3.26 to 14.09) | 3.47 (1.69 to 6.69) | 1.45 (0.69 to 2.80) | 0.49 (0.23 to 0.94) | 0.13 (0.06 to 0.25) | 0.03 (0.01 to 0.06) | 0.01 (0.00 to 0.01) |
| Eritrea | Male | Number of prevalence | 817.42 (262.83 to 3152.61) | 2180.21 (748.60 to 7318.98) | 2457.13 (735.10 to 8911.37) | 2559.13 (677.58 to 9703.75) | 2344.33 (578.58 to 9118.28) | 2063.70 (489.07 to 8151.89) | 1765.09 (408.84 to 7033.94) | 1529.88 (348.83 to 6133.05) | 1187.95 (267.17 to 4783.55) | 1254.73 (280.98 to 5069.63) | 1021.87 (227.55 to 4140.42) | 751.80 (165.87 to 3054.83) | 497.67 (109.24 to 2028.10) | 286.76 (62.88 to 1170.07) | 140.35 (30.80 to 572.63) | 59.43 (13.00 to 242.48) | 20.78 (4.56 to 84.79) | 5.76 (1.27 to 23.52) | 1.29 (0.28 to 5.28) | 0.26 (0.06 to 1.08) |
| Ethiopia | Both | Number of DALYs | 946.90 (615.22 to 1379.49) | 3673.56 (2025.72 to 6610.66) | 4033.95 (2234.36 to 7014.76) | 4020.12 (2277.40 to 6717.37) | 4115.26 (2405.97 to 6731.16) | 3913.38 (2337.59 to 6179.31) | 3472.07 (2081.42 to 5380.52) | 3371.26 (2092.52 to 5157.65) | 2815.66 (1761.82 to 4278.90) | 2596.81 (1645.96 to 3915.54) | 2147.05 (1349.42 to 3226.31) | 1808.68 (1180.78 to 2688.45) | 1548.04 (985.26 to 2281.43) | 1206.73 (785.84 to 1752.02) | 947.54 (618.52 to 1381.25) | 426.08 (277.18 to 608.66) | 124.03 (82.74 to 177.78) | 27.47 (18.81 to 38.74) | 4.00 (2.75 to 5.58) | 0.61 (0.43 to 0.84) |
| Ethiopia | Both | Number of prevalence | 81261.26 (25900.20 to 196411.05) | 189537.71 (65600.16 to 444054.93) | 224047.17 (75530.13 to 527870.64) | 228911.19 (76884.79 to 540526.66) | 235465.52 (78291.20 to 558135.47) | 224201.45 (74701.07 to 532970.19) | 193506.64 (65323.56 to 458366.74) | 183520.62 (62580.39 to 433553.40) | 143163.04 (49742.49 to 336423.83) | 130601.85 (45397.05 to 309005.23) | 108093.44 (37357.97 to 260959.84) | 89332.29 (31027.96 to 218548.99) | 75388.14 (26295.89 to 183673.81) | 57974.37 (20320.52 to 141194.60) | 44322.16 (15774.40 to 107423.47) | 21461.04 (7277.86 to 53166.73) | 7028.67 (2245.50 to 17591.30) | 1767.63 (540.24 to 4506.37) | 299.91 (88.44 to 789.03) | 56.84 (16.31 to 149.57) |
| Ethiopia | Female | Number of DALYs | 544.41 (344.76 to 799.65) | 840.55 (547.92 to 1193.63) | 756.65 (495.10 to 1128.02) | 641.36 (425.14 to 952.18) | 605.25 (402.81 to 888.48) | 545.79 (365.76 to 787.42) | 442.66 (287.60 to 644.40) | 398.49 (270.07 to 570.13) | 273.65 (182.76 to 387.42) | 241.41 (165.14 to 340.36) | 196.75 (131.43 to 277.70) | 153.78 (103.33 to 228.21) | 123.56 (82.37 to 176.67) | 88.58 (61.23 to 126.72) | 60.88 (41.95 to 85.46) | 32.31 (22.27 to 44.50) | 12.02 (8.30 to 16.55) | 3.35 (2.32 to 4.60) | 0.63 (0.44 to 0.88) | 0.13 (0.09 to 0.19) |
| Ethiopia | Female | Number of prevalence | 40523.49 (13370.44 to 96803.03) | 84367.06 (25639.34 to 204966.38) | 98028.84 (28078.01 to 241031.05) | 102892.08 (28586.51 to 255862.72) | 111340.44 (30263.31 to 279461.39) | 109207.54 (29342.19 to 275788.33) | 92781.03 (24839.54 to 234662.93) | 86507.50 (23245.35 to 219259.38) | 61202.10 (16465.64 to 155847.62) | 55160.24 (14709.33 to 142110.02) | 45726.59 (11869.20 to 120792.93) | 36636.52 (9348.62 to 98284.33) | 30132.39 (7596.49 to 80825.54) | 22129.49 (5561.00 to 59018.76) | 15476.59 (3933.87 to 40717.41) | 8495.35 (2134.02 to 22966.67) | 3260.82 (752.52 to 8806.06) | 935.65 (210.83 to 2546.65) | 180.61 (40.75 to 499.88) | 39.29 (9.15 to 109.70) |
| Ethiopia | Male | Number of DALYs | 402.49 (252.43 to 592.57) | 2833.01 (1369.82 to 5548.51) | 3277.31 (1637.27 to 6087.50) | 3378.76 (1805.84 to 5914.99) | 3510.01 (1939.14 to 5922.46) | 3367.59 (1907.26 to 5523.72) | 3029.41 (1757.29 to 4818.99) | 2972.78 (1797.72 to 4707.09) | 2542.01 (1565.88 to 3942.87) | 2355.41 (1466.35 to 3633.62) | 1950.30 (1206.09 to 2980.16) | 1654.90 (1039.51 to 2486.18) | 1424.48 (889.46 to 2120.76) | 1118.15 (707.56 to 1642.93) | 886.67 (568.66 to 1306.48) | 393.77 (253.10 to 567.69) | 112.01 (73.58 to 161.53) | 24.12 (16.22 to 34.48) | 3.37 (2.24 to 4.85) | 0.48 (0.32 to 0.68) |
| Ethiopia | Male | Number of prevalence | 40737.77 (12491.72 to 99608.02) | 105170.66 (38750.38 to 239216.56) | 126018.32 (46255.33 to 287149.37) | 126019.12 (47779.82 to 284766.38) | 124125.07 (48094.27 to 278864.49) | 114993.91 (45402.19 to 257440.49) | 100725.61 (40574.42 to 223912.30) | 97013.11 (39712.92 to 214454.65) | 81960.94 (33822.88 to 180576.21) | 75441.61 (31415.02 to 166895.21) | 62366.85 (25896.74 to 140166.91) | 52695.77 (21911.96 to 120264.66) | 45255.75 (18819.08 to 102848.27) | 35844.89 (14721.14 to 81516.69) | 28845.57 (11682.22 to 65945.78) | 12965.69 (5131.00 to 29813.33) | 3767.85 (1473.97 to 8712.30) | 831.97 (326.89 to 1992.89) | 119.30 (47.48 to 286.50) | 17.55 (7.09 to 41.99) |
| Fiji | Both | Number of DALYs | 14.92 (9.58 to 21.91) | 247.92 (161.12 to 364.87) | 354.65 (239.08 to 498.98) | 366.72 (247.23 to 505.47) | 354.41 (241.09 to 493.01) | 349.27 (240.27 to 481.79) | 318.16 (219.42 to 437.48) | 268.76 (185.03 to 370.36) | 228.68 (156.94 to 315.85) | 190.36 (131.00 to 269.16) | 149.71 (101.55 to 209.04) | 117.67 (80.77 to 160.73) | 84.30 (58.66 to 115.71) | 60.84 (42.54 to 84.46) | 41.26 (28.80 to 56.02) | 22.99 (16.09 to 31.05) | 11.30 (7.94 to 15.34) | 3.58 (2.56 to 4.83) | 0.47 (0.33 to 0.65) | 0.14 (0.09 to 0.19) |
| Fiji | Both | Number of prevalence | 3617.14 (555.44 to 10232.69) | 10805.32 (3056.69 to 27522.03) | 14561.45 (4334.20 to 36586.53) | 16733.03 (4741.24 to 42690.04) | 18423.74 (4844.94 to 47825.86) | 19433.15 (4895.37 to 50833.62) | 18408.21 (4579.91 to 48329.74) | 16003.54 (3944.44 to 42146.84) | 13865.53 (3408.29 to 36603.54) | 11762.78 (2880.68 to 31130.10) | 9456.32 (2310.12 to 24849.85) | 7470.43 (1831.98 to 19317.06) | 5429.97 (1331.90 to 13599.33) | 4000.70 (982.57 to 9849.60) | 2764.65 (679.48 to 6804.42) | 1563.23 (385.52 to 3842.71) | 800.86 (195.93 to 1973.74) | 280.05 (66.39 to 697.56) | 59.39 (11.79 to 154.98) | 18.82 (3.64 to 49.41) |
| Fiji | Female | Number of DALYs | 8.54 (5.24 to 13.00) | 18.64 (11.71 to 27.00) | 23.50 (14.37 to 35.71) | 29.40 (18.93 to 43.02) | 37.72 (24.26 to 56.46) | 44.34 (29.04 to 66.18) | 45.38 (29.71 to 66.80) | 42.37 (28.02 to 62.07) | 38.82 (24.44 to 56.62) | 34.79 (22.38 to 50.92) | 29.51 (19.08 to 44.13) | 23.99 (15.80 to 34.31) | 18.47 (12.06 to 26.56) | 13.86 (9.03 to 19.80) | 9.36 (6.21 to 13.58) | 5.11 (3.41 to 7.35) | 2.66 (1.77 to 3.79) | 1.06 (0.71 to 1.52) | 0.36 (0.24 to 0.51) | 0.12 (0.08 to 0.16) |
| Fiji | Female | Number of prevalence | 1764.63 (282.03 to 4966.36) | 4427.84 (685.69 to 12516.37) | 5893.85 (905.29 to 16679.30) | 6986.79 (1087.35 to 19733.23) | 8026.38 (1279.30 to 22591.80) | 8600.46 (1402.90 to 24124.13) | 8191.83 (1364.28 to 22913.94) | 7205.56 (1224.06 to 20112.82) | 6272.34 (1084.94 to 17487.89) | 5374.31 (946.52 to 14969.51) | 4383.95 (785.29 to 12205.33) | 3429.57 (625.07 to 9549.19) | 2523.48 (469.49 to 6802.00) | 1880.80 (353.94 to 4970.81) | 1293.68 (243.49 to 3419.12) | 717.78 (135.08 to 1897.00) | 382.51 (71.98 to 1010.95) | 155.31 (29.24 to 410.47) | 53.61 (10.11 to 141.68) | 17.87 (3.36 to 47.22) |
| Fiji | Male | Number of DALYs | 6.38 (3.79 to 9.88) | 229.28 (145.54 to 342.50) | 331.15 (223.42 to 466.55) | 337.32 (225.14 to 463.21) | 316.69 (215.27 to 447.51) | 304.93 (209.23 to 427.33) | 272.78 (186.33 to 377.42) | 226.39 (154.53 to 308.09) | 189.87 (129.31 to 261.88) | 155.57 (105.71 to 216.69) | 120.20 (80.78 to 166.26) | 93.68 (64.00 to 129.40) | 65.82 (45.85 to 90.02) | 46.97 (32.65 to 66.19) | 31.89 (22.27 to 43.33) | 17.87 (12.43 to 24.05) | 8.64 (6.09 to 11.68) | 2.52 (1.80 to 3.37) | 0.11 (0.08 to 0.15) | 0.02 (0.01 to 0.02) |
| Fiji | Male | Number of prevalence | 1852.52 (273.41 to 5266.32) | 6377.48 (2371.99 to 15008.08) | 8667.60 (3394.70 to 19918.50) | 9746.23 (3624.94 to 22963.27) | 10397.35 (3560.53 to 25242.98) | 10832.69 (3514.68 to 26717.03) | 10216.38 (3215.49 to 25424.83) | 8797.98 (2716.44 to 22040.88) | 7593.18 (2313.74 to 19122.26) | 6388.47 (1926.58 to 16165.96) | 5072.37 (1521.96 to 12644.52) | 4040.87 (1206.12 to 9767.87) | 2906.49 (864.41 to 6797.33) | 2119.90 (627.56 to 4878.78) | 1470.97 (435.42 to 3385.31) | 845.45 (250.26 to 1945.71) | 418.35 (124.03 to 962.80) | 124.74 (36.94 to 287.08) | 5.78 (1.71 to 13.30) | 0.95 (0.28 to 2.20) |
| Gabon | Both | Number of DALYs | 15.43 (9.61 to 23.02) | 53.68 (27.82 to 97.54) | 56.51 (29.93 to 100.01) | 60.87 (33.82 to 106.20) | 65.87 (37.67 to 112.13) | 67.95 (38.22 to 114.82) | 61.62 (35.48 to 103.43) | 58.58 (32.08 to 97.33) | 34.33 (19.96 to 56.70) | 36.15 (21.41 to 57.17) | 34.74 (20.74 to 55.60) | 32.16 (19.82 to 50.18) | 27.68 (16.84 to 43.00) | 21.97 (13.47 to 33.57) | 15.56 (9.82 to 24.69) | 9.40 (5.98 to 14.48) | 4.99 (3.21 to 7.46) | 1.60 (1.05 to 2.40) | 0.31 (0.20 to 0.46) | 0.05 (0.03 to 0.07) |
| Gabon | Both | Number of prevalence | 1098.88 (239.01 to 4331.50) | 2558.44 (647.97 to 9864.26) | 3138.44 (725.22 to 12335.83) | 3627.49 (789.53 to 14375.70) | 3944.54 (850.98 to 15619.21) | 3939.06 (873.35 to 15519.50) | 3429.36 (781.82 to 13425.63) | 3143.74 (736.63 to 12245.63) | 1881.30 (439.98 to 7331.46) | 2043.02 (468.53 to 7984.56) | 2034.38 (456.97 to 7973.14) | 1923.97 (429.73 to 7542.27) | 1655.10 (371.89 to 6479.32) | 1316.03 (298.18 to 5143.63) | 949.18 (215.61 to 3707.14) | 589.44 (132.96 to 2304.81) | 334.16 (73.28 to 1313.84) | 126.88 (25.70 to 506.49) | 31.21 (5.63 to 126.95) | 6.79 (1.12 to 28.08) |
| Gabon | Female | Number of DALYs | 9.04 (5.38 to 13.54) | 14.25 (8.20 to 22.60) | 13.19 (7.91 to 20.28) | 12.20 (7.16 to 19.62) | 11.29 (6.69 to 17.56) | 9.93 (6.12 to 15.46) | 7.62 (4.77 to 11.59) | 6.48 (4.07 to 9.87) | 3.91 (2.53 to 5.89) | 4.37 (2.70 to 6.20) | 4.58 (2.88 to 6.86) | 4.30 (2.65 to 6.45) | 3.66 (2.33 to 5.49) | 2.83 (1.82 to 4.12) | 1.98 (1.25 to 2.88) | 1.23 (0.74 to 1.81) | 0.73 (0.43 to 1.05) | 0.32 (0.21 to 0.46) | 0.09 (0.06 to 0.13) | 0.02 (0.01 to 0.03) |
| Gabon | Female | Number of prevalence | 561.40 (133.51 to 2172.17) | 1183.35 (236.61 to 4747.09) | 1471.83 (254.78 to 6051.72) | 1719.37 (269.57 to 7173.80) | 1844.69 (274.90 to 7754.05) | 1760.62 (254.99 to 7429.46) | 1446.91 (206.56 to 6118.77) | 1262.92 (177.95 to 5349.07) | 789.83 (110.36 to 3349.23) | 918.03 (127.53 to 3896.11) | 971.30 (133.90 to 4125.41) | 940.12 (128.79 to 3995.98) | 814.18 (110.85 to 3463.29) | 641.69 (87.19 to 2730.46) | 459.04 (62.35 to 1953.25) | 288.99 (39.28 to 1229.64) | 174.62 (23.75 to 743.02) | 77.57 (10.53 to 330.06) | 22.56 (3.07 to 95.99) | 5.59 (0.76 to 23.80) |
| Gabon | Male | Number of DALYs | 6.39 (3.47 to 10.27) | 39.42 (18.05 to 79.84) | 43.32 (20.35 to 83.12) | 48.66 (23.46 to 88.35) | 54.58 (28.24 to 98.53) | 58.02 (30.18 to 103.00) | 54.00 (29.23 to 93.19) | 52.11 (27.37 to 87.54) | 30.42 (16.65 to 51.05) | 31.78 (17.90 to 51.79) | 30.16 (17.21 to 49.96) | 27.87 (16.43 to 45.09) | 24.02 (14.06 to 38.65) | 19.14 (11.58 to 30.04) | 13.58 (8.39 to 22.29) | 8.17 (4.97 to 12.95) | 4.26 (2.62 to 6.74) | 1.29 (0.79 to 2.02) | 0.22 (0.13 to 0.35) | 0.03 (0.02 to 0.05) |
| Gabon | Male | Number of prevalence | 537.48 (107.58 to 2159.33) | 1375.09 (393.00 to 5044.95) | 1666.61 (457.08 to 6258.01) | 1908.12 (527.52 to 7200.45) | 2099.84 (589.21 to 7870.43) | 2178.45 (629.33 to 8092.77) | 1982.45 (582.47 to 7307.33) | 1880.81 (562.69 to 6896.56) | 1091.47 (331.06 to 3982.23) | 1124.99 (344.91 to 4088.45) | 1063.08 (329.95 to 3847.73) | 983.86 (309.42 to 3546.29) | 840.92 (268.55 to 3016.02) | 674.34 (216.35 to 2413.17) | 490.14 (157.10 to 1753.89) | 300.45 (96.50 to 1075.17) | 159.54 (51.14 to 570.82) | 49.31 (15.84 to 176.43) | 8.65 (2.78 to 30.96) | 1.19 (0.38 to 4.27) |
| Ghana | Both | Number of DALYs | 276.90 (174.16 to 409.58) | 1270.91 (685.99 to 2253.34) | 1511.88 (844.15 to 2614.92) | 1834.84 (1093.83 to 2938.12) | 1998.61 (1264.89 to 3038.01) | 1998.34 (1271.14 to 3060.59) | 1951.97 (1249.40 to 2888.68) | 1799.02 (1159.41 to 2663.96) | 1505.75 (972.71 to 2216.32) | 1269.52 (841.28 to 1844.22) | 1074.39 (712.53 to 1552.20) | 933.40 (614.05 to 1342.16) | 726.16 (475.57 to 1042.39) | 532.86 (356.94 to 756.66) | 348.56 (234.12 to 490.37) | 199.72 (137.78 to 279.64) | 94.42 (63.47 to 130.45) | 34.24 (23.13 to 46.83) | 8.60 (5.88 to 11.96) | 1.50 (1.04 to 2.07) |
| Ghana | Both | Number of prevalence | 30956.98 (7583.31 to 88324.32) | 72360.42 (20852.20 to 198602.72) | 86802.52 (24964.10 to 238655.05) | 99574.23 (29046.92 to 272058.97) | 104748.69 (31357.39 to 284952.25) | 102814.18 (30921.26 to 278640.60) | 95345.39 (29440.23 to 256611.48) | 83821.12 (26463.36 to 224125.36) | 68651.84 (22018.90 to 182787.41) | 57380.00 (18533.02 to 152487.14) | 48504.32 (15756.16 to 128703.31) | 41866.23 (13746.83 to 110789.35) | 33355.77 (10939.12 to 88382.41) | 25094.96 (8197.21 to 66577.77) | 16855.37 (5506.19 to 44770.55) | 9834.41 (3202.23 to 26128.44) | 4807.73 (1554.97 to 12804.49) | 1844.69 (589.23 to 4934.29) | 488.37 (154.00 to 1312.16) | 97.74 (29.66 to 266.16) |
| Ghana | Female | Number of DALYs | 158.65 (94.66 to 240.33) | 254.88 (148.14 to 384.06) | 231.35 (132.67 to 349.08) | 219.13 (132.34 to 337.87) | 214.68 (133.45 to 317.82) | 205.38 (130.56 to 311.52) | 177.13 (111.54 to 266.30) | 143.59 (90.03 to 204.39) | 113.38 (72.48 to 167.99) | 94.25 (61.04 to 135.47) | 78.64 (51.36 to 113.10) | 66.51 (44.25 to 94.83) | 53.07 (33.54 to 78.64) | 40.12 (26.41 to 57.36) | 27.08 (17.09 to 38.63) | 15.68 (10.12 to 21.91) | 7.83 (5.01 to 11.03) | 3.08 (2.00 to 4.36) | 0.83 (0.54 to 1.17) | 0.18 (0.12 to 0.26) |
| Ghana | Female | Number of prevalence | 15418.84 (3976.13 to 43567.28) | 32528.01 (7706.97 to 93595.52) | 38373.25 (8574.58 to 111738.52) | 43802.16 (9430.38 to 128398.63) | 48142.28 (10193.89 to 141561.96) | 48638.85 (10209.00 to 143239.58) | 43348.11 (9062.90 to 127754.93) | 36241.72 (7558.06 to 106863.12) | 29001.82 (6038.94 to 85548.40) | 24246.86 (5038.98 to 71538.53) | 20437.14 (4243.18 to 60307.88) | 17465.34 (3624.85 to 51548.11) | 14338.71 (2974.22 to 42324.29) | 11027.78 (2285.96 to 32552.79) | 7510.87 (1557.69 to 22171.63) | 4381.66 (908.67 to 12934.43) | 2200.65 (456.32 to 6496.30) | 886.54 (183.79 to 2617.04) | 245.38 (50.88 to 724.36) | 55.75 (11.57 to 164.56) |
| Ghana | Male | Number of DALYs | 118.25 (67.96 to 191.19) | 1016.03 (480.43 to 1932.93) | 1280.52 (667.75 to 2281.33) | 1615.71 (935.55 to 2659.65) | 1783.93 (1112.11 to 2751.82) | 1792.97 (1100.84 to 2822.66) | 1774.84 (1128.59 to 2674.20) | 1655.43 (1045.68 to 2481.51) | 1392.37 (886.36 to 2073.02) | 1175.28 (772.28 to 1719.60) | 995.75 (649.19 to 1451.59) | 866.89 (566.81 to 1253.02) | 673.08 (438.80 to 978.53) | 492.74 (325.06 to 700.71) | 321.47 (214.46 to 457.32) | 184.03 (125.76 to 261.22) | 86.58 (57.53 to 120.66) | 31.16 (20.85 to 43.51) | 7.76 (5.21 to 11.05) | 1.32 (0.91 to 1.83) |
| Ghana | Male | Number of prevalence | 15538.14 (3607.19 to 44757.04) | 39832.41 (13174.34 to 105067.16) | 48429.28 (16454.90 to 126989.46) | 55772.06 (19589.48 to 143742.10) | 56606.42 (20684.33 to 143462.28) | 54175.32 (20657.72 to 135459.14) | 51997.29 (20303.60 to 128899.43) | 47579.40 (18930.66 to 117304.35) | 39650.03 (16035.55 to 97263.81) | 33133.14 (13608.01 to 80969.86) | 28067.17 (11675.18 to 68412.11) | 24400.89 (10198.11 to 59252.31) | 19017.06 (7993.07 to 46066.15) | 14067.18 (5893.55 to 34029.26) | 9344.50 (3916.01 to 22602.09) | 5452.75 (2284.87 to 13196.41) | 2607.08 (1093.06 to 6309.29) | 958.14 (402.27 to 2317.56) | 242.99 (101.75 to 587.90) | 41.99 (17.60 to 101.61) |
| Guinea | Both | Number of DALYs | 116.70 (74.75 to 173.25) | 443.68 (228.79 to 776.82) | 468.59 (253.56 to 809.54) | 489.89 (281.07 to 812.54) | 501.79 (295.24 to 828.73) | 514.17 (309.75 to 793.73) | 526.29 (326.65 to 806.81) | 497.56 (316.74 to 752.20) | 448.15 (281.49 to 678.82) | 400.93 (254.22 to 590.46) | 359.74 (229.56 to 525.05) | 323.77 (212.77 to 472.02) | 298.17 (191.97 to 434.98) | 258.14 (169.55 to 376.65) | 177.34 (116.05 to 257.04) | 104.29 (69.93 to 149.62) | 57.76 (38.69 to 81.68) | 25.34 (17.21 to 35.55) | 7.23 (4.86 to 10.42) | 1.44 (0.98 to 2.03) |
| Guinea | Both | Number of prevalence | 11118.41 (2257.37 to 33253.82) | 24227.97 (5834.66 to 68722.34) | 26842.91 (6254.61 to 76907.56) | 28209.89 (6522.51 to 81151.05) | 28965.18 (6731.82 to 83569.57) | 29512.29 (6928.34 to 85219.59) | 29342.32 (7032.29 to 84553.36) | 26065.94 (6484.96 to 74611.25) | 23201.93 (5849.23 to 66320.31) | 19774.34 (5138.92 to 56188.55) | 17098.50 (4544.83 to 48346.41) | 15235.64 (4115.30 to 43002.82) | 14011.15 (3808.37 to 39509.39) | 12405.98 (3343.73 to 35027.05) | 8681.75 (2346.58 to 24509.60) | 5091.64 (1395.10 to 14333.85) | 2836.43 (778.78 to 7974.40) | 1287.19 (350.59 to 3625.84) | 387.91 (103.75 to 1097.47) | 87.40 (22.13 to 250.34) |
| Guinea | Female | Number of DALYs | 67.32 (39.78 to 101.22) | 97.28 (59.54 to 146.65) | 83.01 (50.20 to 131.43) | 72.80 (43.13 to 112.14) | 69.45 (45.28 to 107.34) | 68.59 (43.01 to 99.37) | 65.31 (43.11 to 98.84) | 52.32 (31.81 to 79.71) | 45.78 (29.46 to 68.32) | 35.94 (23.56 to 53.05) | 29.31 (18.40 to 44.50) | 25.07 (15.70 to 36.63) | 22.81 (14.78 to 34.39) | 20.02 (12.67 to 29.93) | 13.70 (8.51 to 19.32) | 7.65 (5.05 to 11.34) | 4.12 (2.60 to 5.99) | 1.90 (1.22 to 2.75) | 0.58 (0.39 to 0.84) | 0.15 (0.10 to 0.22) |
| Guinea | Female | Number of prevalence | 5559.06 (1211.73 to 16451.32) | 10941.36 (2074.90 to 33103.52) | 11967.63 (2040.49 to 36754.28) | 12965.57 (2067.18 to 40158.54) | 14183.07 (2180.42 to 44117.77) | 14971.28 (2259.19 to 46666.82) | 14785.88 (2208.51 to 46142.77) | 12309.31 (1826.90 to 38440.41) | 10929.68 (1615.22 to 34150.76) | 8761.01 (1289.83 to 27384.97) | 7199.93 (1056.33 to 22513.04) | 6331.78 (926.46 to 19806.52) | 5829.84 (851.51 to 18241.37) | 5266.59 (767.07 to 16480.61) | 3683.52 (537.15 to 11527.09) | 2077.94 (302.74 to 6502.56) | 1139.27 (165.95 to 3565.26) | 530.94 (77.33 to 1661.56) | 168.78 (24.62 to 528.19) | 43.81 (6.39 to 137.08) |
| Guinea | Male | Number of DALYs | 49.38 (27.37 to 80.38) | 346.40 (162.42 to 668.27) | 385.58 (187.14 to 702.31) | 417.09 (218.75 to 721.99) | 432.34 (243.33 to 725.71) | 445.59 (254.79 to 716.34) | 460.98 (281.02 to 723.87) | 445.24 (276.48 to 684.20) | 402.38 (245.58 to 628.29) | 364.99 (226.15 to 547.75) | 330.43 (209.12 to 487.00) | 298.70 (194.45 to 438.81) | 275.36 (176.49 to 404.92) | 238.12 (155.75 to 352.70) | 163.64 (105.14 to 240.48) | 96.64 (64.18 to 140.37) | 53.64 (35.15 to 76.27) | 23.44 (15.73 to 33.10) | 6.65 (4.40 to 9.75) | 1.29 (0.85 to 1.85) |
| Guinea | Male | Number of prevalence | 5559.35 (1053.98 to 16802.50) | 13286.61 (3710.03 to 35744.32) | 14875.28 (4152.91 to 40254.31) | 15244.32 (4433.72 to 40992.51) | 14782.10 (4495.60 to 39451.80) | 14541.01 (4584.54 to 38552.77) | 14556.44 (4719.91 to 38410.59) | 13756.64 (4572.29 to 36170.83) | 12272.25 (4134.53 to 32169.56) | 11013.33 (3766.65 to 28803.58) | 9898.57 (3418.46 to 25833.37) | 8903.85 (3108.32 to 23196.30) | 8181.31 (2911.75 to 21268.01) | 7139.39 (2557.47 to 18546.44) | 4998.23 (1792.65 to 12982.51) | 3013.71 (1079.38 to 7831.30) | 1697.16 (607.43 to 4409.14) | 756.25 (270.28 to 1964.28) | 219.13 (78.50 to 569.28) | 43.60 (15.66 to 113.26) |
| Guinea-Bissau | Both | Number of DALYs | 23.86 (15.05 to 35.73) | 236.84 (142.43 to 368.13) | 334.17 (221.55 to 489.25) | 382.66 (251.24 to 542.16) | 351.41 (232.95 to 493.21) | 307.88 (206.91 to 425.10) | 257.64 (175.05 to 359.09) | 211.34 (143.41 to 288.47) | 158.89 (107.65 to 215.74) | 132.51 (89.73 to 181.83) | 108.83 (74.76 to 150.25) | 89.80 (62.05 to 123.26) | 75.86 (51.83 to 104.48) | 61.62 (42.92 to 83.74) | 41.38 (29.06 to 55.72) | 24.05 (16.91 to 32.07) | 8.87 (6.26 to 12.02) | 2.36 (1.69 to 3.17) | 0.49 (0.35 to 0.65) | 0.09 (0.06 to 0.11) |
| Guinea-Bissau | Both | Number of prevalence | 4800.08 (405.45 to 18205.24) | 12238.38 (2323.52 to 42233.34) | 14945.51 (3285.93 to 50373.49) | 16420.77 (3629.09 to 54942.19) | 16018.73 (3362.16 to 54177.08) | 15078.45 (2959.81 to 51576.36) | 13449.07 (2530.24 to 46403.37) | 11557.84 (2119.80 to 40149.41) | 9003.26 (1626.76 to 31443.37) | 7659.75 (1378.06 to 26856.85) | 6367.83 (1144.53 to 22400.77) | 5282.87 (956.08 to 18599.06) | 4502.62 (817.53 to 15610.20) | 3634.25 (670.49 to 12471.97) | 2467.26 (457.36 to 8456.78) | 1473.28 (272.17 to 5053.01) | 586.69 (103.49 to 2032.71) | 175.91 (28.95 to 618.04) | 40.01 (6.24 to 141.98) | 7.96 (1.16 to 28.60) |
| Guinea-Bissau | Female | Number of DALYs | 13.94 (8.57 to 21.64) | 24.97 (15.10 to 36.39) | 26.07 (15.83 to 39.77) | 27.36 (17.67 to 40.56) | 28.63 (18.59 to 41.77) | 28.52 (18.50 to 41.68) | 27.04 (17.81 to 40.19) | 24.28 (15.95 to 34.79) | 19.49 (12.72 to 28.06) | 16.96 (11.36 to 25.00) | 14.22 (9.39 to 20.97) | 11.66 (7.56 to 16.81) | 9.91 (6.42 to 14.28) | 7.62 (5.08 to 10.85) | 5.04 (3.39 to 7.32) | 2.98 (2.00 to 4.29) | 1.30 (0.89 to 1.82) | 0.44 (0.29 to 0.61) | 0.11 (0.07 to 0.15) | 0.02 (0.02 to 0.03) |
| Guinea-Bissau | Female | Number of prevalence | 2413.87 (225.77 to 9100.06) | 5360.85 (441.96 to 20379.97) | 6348.04 (485.17 to 24237.73) | 7015.35 (524.80 to 26815.18) | 7177.87 (542.35 to 27412.95) | 6989.29 (537.14 to 26659.56) | 6413.36 (501.86 to 24432.05) | 5614.42 (445.46 to 21369.78) | 4410.18 (355.41 to 16782.86) | 3736.25 (305.88 to 14228.19) | 3062.54 (253.36 to 11679.10) | 2470.95 (207.24 to 9438.97) | 2047.19 (174.69 to 7839.03) | 1578.15 (135.82 to 6049.71) | 1056.96 (90.83 to 4051.67) | 635.60 (54.65 to 2436.48) | 281.93 (24.26 to 1080.77) | 96.63 (8.32 to 370.42) | 23.96 (2.06 to 91.83) | 5.25 (0.45 to 20.11) |
| Guinea-Bissau | Male | Number of DALYs | 9.91 (5.77 to 15.71) | 211.87 (125.58 to 338.11) | 308.11 (201.81 to 453.44) | 355.30 (230.06 to 503.80) | 322.78 (212.55 to 453.15) | 279.36 (186.84 to 390.08) | 230.60 (156.57 to 321.67) | 187.06 (126.12 to 256.81) | 139.40 (94.86 to 191.37) | 115.55 (78.31 to 160.40) | 94.61 (65.25 to 130.56) | 78.14 (54.00 to 108.58) | 65.95 (44.99 to 89.97) | 54.00 (37.32 to 74.02) | 36.35 (25.51 to 49.48) | 21.07 (14.57 to 28.34) | 7.57 (5.35 to 10.13) | 1.92 (1.36 to 2.58) | 0.38 (0.27 to 0.50) | 0.06 (0.05 to 0.08) |
| Guinea-Bissau | Male | Number of prevalence | 2386.21 (178.34 to 9105.19) | 6877.53 (1784.60 to 21866.34) | 8597.47 (2719.65 to 26138.10) | 9405.42 (3176.62 to 28205.11) | 8840.85 (2868.99 to 26801.13) | 8089.17 (2498.20 to 24920.28) | 7035.71 (2076.15 to 21973.69) | 5943.42 (1693.42 to 18779.63) | 4593.08 (1276.25 to 14660.51) | 3923.50 (1068.31 to 12628.65) | 3305.28 (887.95 to 10721.67) | 2811.92 (747.27 to 9160.10) | 2455.43 (641.28 to 7771.17) | 2056.10 (533.37 to 6422.25) | 1410.30 (365.84 to 4405.11) | 837.68 (217.38 to 2616.53) | 304.76 (79.04 to 951.94) | 79.28 (20.59 to 247.62) | 16.05 (4.17 to 50.14) | 2.72 (0.70 to 8.49) |
| Guyana | Both | Number of DALYs | 21.57 (14.25 to 31.76) | 361.29 (239.42 to 504.57) | 440.13 (295.24 to 603.05) | 474.21 (321.11 to 652.87) | 496.98 (338.68 to 684.49) | 496.66 (340.70 to 709.66) | 453.93 (307.15 to 643.64) | 410.27 (279.85 to 581.67) | 334.78 (227.18 to 480.03) | 283.45 (192.56 to 396.18) | 236.37 (159.44 to 330.87) | 204.41 (139.22 to 291.91) | 187.84 (129.31 to 265.18) | 153.46 (103.64 to 215.86) | 97.16 (67.18 to 134.88) | 63.88 (43.74 to 89.25) | 38.13 (26.04 to 53.03) | 17.15 (12.00 to 23.63) | 5.04 (3.49 to 6.88) | 1.05 (0.74 to 1.43) |
| Guyana | Both | Number of prevalence | 6104.49 (2913.31 to 10570.51) | 15262.48 (8597.35 to 24737.76) | 22080.38 (12144.27 to 36016.01) | 27898.46 (15052.07 to 45793.12) | 30907.39 (16605.64 to 50720.41) | 30703.73 (16504.40 to 50199.93) | 27375.79 (14742.96 to 44575.41) | 24003.69 (12974.00 to 38903.68) | 19127.20 (10388.68 to 30887.52) | 15749.66 (8628.03 to 25353.74) | 12829.63 (7088.55 to 20590.54) | 10822.55 (6039.98 to 17123.11) | 9638.64 (5450.18 to 14713.71) | 7815.93 (4445.25 to 11687.71) | 5025.25 (2856.09 to 7517.43) | 3355.50 (1907.58 to 5021.36) | 2052.90 (1165.90 to 3076.89) | 950.65 (538.63 to 1427.74) | 280.51 (159.33 to 420.18) | 57.14 (32.64 to 84.85) |
| Guyana | Female | Number of DALYs | 12.47 (8.11 to 19.23) | 25.44 (15.86 to 37.51) | 43.59 (28.47 to 64.29) | 72.95 (47.33 to 108.69) | 106.54 (69.81 to 155.98) | 128.64 (83.04 to 195.33) | 130.89 (85.23 to 194.58) | 126.83 (81.30 to 189.74) | 109.37 (70.43 to 163.51) | 96.96 (62.06 to 140.94) | 84.25 (54.16 to 121.63) | 75.91 (49.01 to 110.18) | 73.24 (48.07 to 105.68) | 61.68 (40.08 to 90.57) | 39.66 (26.28 to 57.65) | 26.39 (17.19 to 38.63) | 16.58 (10.92 to 24.11) | 7.94 (5.23 to 11.38) | 2.14 (1.40 to 3.13) | 0.33 (0.22 to 0.47) |
| Guyana | Female | Number of prevalence | 3028.72 (1454.01 to 5232.31) | 6413.41 (3078.59 to 11080.08) | 9702.16 (4697.95 to 16709.57) | 12776.38 (6294.45 to 21848.54) | 14595.74 (7300.72 to 24726.38) | 14659.21 (7434.89 to 24618.35) | 13089.59 (6713.76 to 21820.88) | 11460.76 (5939.18 to 18976.28) | 9154.54 (4790.05 to 15076.51) | 7568.77 (4005.88 to 12404.21) | 6191.84 (3314.31 to 10105.91) | 5276.68 (2861.90 to 8578.63) | 4770.88 (2626.39 to 7555.40) | 3923.01 (2174.01 to 6076.44) | 2548.15 (1412.80 to 3946.92) | 1718.11 (953.18 to 2661.25) | 1095.60 (607.67 to 1697.01) | 534.29 (296.07 to 827.59) | 147.35 (81.69 to 228.23) | 23.07 (12.80 to 35.74) |
| Guyana | Male | Number of DALYs | 9.10 (5.63 to 14.00) | 335.84 (220.64 to 468.48) | 396.54 (266.54 to 549.54) | 401.26 (272.34 to 550.45) | 390.44 (264.06 to 543.05) | 368.02 (252.17 to 518.36) | 323.04 (222.05 to 450.93) | 283.44 (195.00 to 394.10) | 225.41 (153.13 to 315.13) | 186.49 (126.80 to 261.37) | 152.12 (104.78 to 213.04) | 128.50 (90.00 to 181.41) | 114.60 (79.95 to 162.39) | 91.78 (63.28 to 130.00) | 57.49 (40.03 to 81.50) | 37.49 (26.13 to 52.23) | 21.55 (15.07 to 29.98) | 9.21 (6.42 to 12.80) | 2.89 (2.08 to 3.91) | 0.72 (0.52 to 0.99) |
| Guyana | Male | Number of prevalence | 3075.76 (1459.30 to 5338.20) | 8849.06 (5647.48 to 13658.09) | 12378.21 (7406.25 to 19313.19) | 15122.09 (8692.38 to 24032.92) | 16311.65 (9267.20 to 26020.47) | 16044.52 (9061.79 to 25581.64) | 14286.20 (8029.20 to 22754.53) | 12542.93 (7034.83 to 19927.40) | 9972.67 (5607.76 to 15811.02) | 8180.88 (4622.16 to 12949.53) | 6637.79 (3774.24 to 10496.32) | 5545.87 (3178.08 to 8544.48) | 4867.76 (2823.79 to 7158.31) | 3892.92 (2271.18 to 5611.27) | 2477.09 (1443.29 to 3570.52) | 1637.39 (954.40 to 2360.12) | 957.30 (558.23 to 1379.88) | 416.36 (242.57 to 600.14) | 133.16 (77.64 to 191.95) | 34.07 (19.84 to 49.11) |
| Haiti | Both | Number of DALYs | 151.97 (96.28 to 224.17) | 1930.81 (1256.96 to 2877.59) | 2699.33 (1824.34 to 3820.38) | 2775.69 (1849.74 to 3786.94) | 2479.93 (1686.05 to 3370.30) | 2226.08 (1522.98 to 3071.84) | 2012.60 (1371.46 to 2746.23) | 1875.09 (1271.82 to 2588.14) | 1437.76 (983.33 to 1979.55) | 1247.49 (860.78 to 1770.69) | 1073.55 (731.67 to 1484.49) | 944.08 (656.25 to 1290.86) | 798.51 (556.79 to 1101.48) | 600.73 (416.22 to 831.75) | 380.59 (261.08 to 514.19) | 201.00 (141.85 to 272.08) | 90.18 (62.92 to 121.77) | 30.31 (21.55 to 41.08) | 6.52 (4.64 to 8.76) | 1.11 (0.79 to 1.48) |
| Haiti | Both | Number of prevalence | 35810.42 (4669.39 to 103437.80) | 90184.10 (22514.99 to 236304.26) | 115948.75 (31370.53 to 300549.35) | 128161.67 (33439.16 to 335409.00) | 130711.65 (31712.53 to 347314.42) | 128542.81 (29471.07 to 344643.01) | 121087.30 (27134.71 to 325947.75) | 114371.36 (25503.36 to 308303.01) | 88170.01 (19677.39 to 237851.38) | 77055.46 (17239.26 to 208105.67) | 67591.45 (15018.12 to 183050.49) | 59865.35 (13342.79 to 161493.23) | 51332.30 (11453.31 to 136163.71) | 39717.25 (8807.01 to 104265.23) | 25967.89 (5694.75 to 68336.90) | 14237.93 (3083.32 to 37581.47) | 6751.62 (1435.42 to 17912.21) | 2402.32 (501.09 to 6404.98) | 562.56 (113.59 to 1511.39) | 110.27 (21.20 to 300.16) |
| Haiti | Female | Number of DALYs | 89.05 (54.26 to 131.61) | 162.48 (102.05 to 246.09) | 193.76 (123.24 to 279.65) | 227.65 (143.87 to 340.75) | 267.63 (177.23 to 383.22) | 297.13 (193.31 to 430.78) | 298.04 (192.04 to 433.52) | 293.21 (200.13 to 425.57) | 229.58 (146.79 to 330.90) | 204.10 (134.87 to 295.64) | 188.57 (123.53 to 277.73) | 171.72 (113.92 to 245.98) | 154.25 (101.41 to 227.19) | 125.04 (81.71 to 179.46) | 83.89 (55.46 to 122.17) | 47.38 (31.14 to 66.95) | 23.57 (15.58 to 33.82) | 8.78 (5.86 to 12.71) | 2.19 (1.48 to 3.13) | 0.48 (0.32 to 0.69) |
| Haiti | Female | Number of prevalence | 18024.68 (2474.23 to 51817.91) | 39068.55 (5097.04 to 112881.53) | 49607.45 (6360.73 to 143579.89) | 56323.13 (7331.56 to 162810.64) | 60587.12 (8116.14 to 174664.59) | 62000.98 (8519.21 to 178273.21) | 58669.89 (8223.91 to 168400.37) | 54266.01 (7783.84 to 155561.40) | 40865.25 (5986.27 to 117015.44) | 35019.76 (5233.88 to 100222.70) | 31156.21 (4739.02 to 89132.78) | 27167.96 (4218.02 to 77691.22) | 23338.79 (3714.70 to 66756.98) | 18612.67 (2994.93 to 52720.36) | 12599.47 (2026.69 to 35687.01) | 7199.69 (1159.79 to 20391.97) | 3647.37 (587.06 to 10330.62) | 1378.78 (221.86 to 3905.17) | 352.48 (56.69 to 998.33) | 79.04 (12.72 to 223.88) |
| Haiti | Male | Number of DALYs | 62.93 (37.52 to 99.53) | 1768.33 (1133.41 to 2661.24) | 2505.57 (1675.06 to 3559.12) | 2548.04 (1707.09 to 3500.09) | 2212.30 (1500.01 to 3057.71) | 1928.95 (1332.36 to 2720.50) | 1714.56 (1164.38 to 2345.61) | 1581.89 (1059.35 to 2168.47) | 1208.18 (819.36 to 1682.32) | 1043.40 (709.75 to 1475.26) | 884.98 (604.53 to 1212.70) | 772.36 (530.39 to 1064.03) | 644.26 (446.40 to 883.40) | 475.69 (326.59 to 651.44) | 296.70 (204.24 to 400.82) | 153.62 (106.49 to 210.29) | 66.61 (46.70 to 88.14) | 21.53 (15.06 to 29.21) | 4.33 (3.05 to 5.78) | 0.63 (0.45 to 0.83) |
| Haiti | Male | Number of prevalence | 17785.74 (2194.87 to 51619.89) | 51115.55 (17361.46 to 123148.35) | 66341.29 (24475.03 to 156121.33) | 71838.54 (25692.01 to 172146.00) | 70124.53 (23291.17 to 172649.84) | 66541.83 (20857.14 to 166369.80) | 62417.41 (18853.86 to 157547.38) | 60105.34 (17775.68 to 152741.61) | 47304.76 (13756.90 to 120835.94) | 42035.70 (12095.72 to 107882.96) | 36435.23 (10369.59 to 93917.71) | 32697.39 (9214.42 to 83802.00) | 27993.52 (7845.41 to 69406.73) | 21104.58 (5892.70 to 51544.87) | 13368.42 (3729.35 to 32649.88) | 7038.23 (1960.65 to 17189.50) | 3104.25 (865.32 to 7581.59) | 1023.54 (285.32 to 2499.81) | 210.07 (58.57 to 513.06) | 31.23 (8.71 to 76.28) |
| India | Both | Number of DALYs | 14468.87 (9821.31 to 20461.24) | 138271.25 (90594.46 to 205155.14) | 152681.05 (101179.88 to 223882.47) | 168997.57 (113197.70 to 241998.91) | 184144.74 (123822.35 to 263814.13) | 180750.18 (121816.92 to 255595.65) | 170898.14 (116095.13 to 240622.54) | 168200.68 (114796.93 to 235867.65) | 147919.60 (101279.53 to 205472.79) | 131196.88 (89891.65 to 183701.71) | 113097.09 (77824.12 to 157515.69) | 100040.81 (68489.70 to 140184.92) | 84664.43 (58272.25 to 117693.70) | 57481.20 (39628.88 to 78975.86) | 34745.22 (24083.85 to 47884.85) | 20450.73 (14263.29 to 27813.09) | 11411.58 (8078.27 to 15410.27) | 3685.80 (2609.16 to 4934.77) | 809.98 (576.10 to 1083.23) | 152.50 (109.33 to 203.24) |
| India | Both | Number of prevalence | 2324681.92 (2135250.58 to 2518529.53) | 6302891.37 (5790073.70 to 6848789.92) | 7520995.23 (6946299.31 to 8141521.07) | 8708971.75 (8059488.62 to 9413937.98) | 9587457.27 (8899260.06 to 10339507.60) | 9425927.34 (8766044.64 to 10159855.33) | 8855788.48 (8243599.56 to 9536617.70) | 8626283.11 (8021867.41 to 9285073.99) | 7501981.73 (6967960.40 to 8067296.99) | 6569388.13 (6099215.39 to 7061658.58) | 5643953.05 (5234534.84 to 6065754.73) | 4995148.48 (4630207.72 to 5366056.01) | 4202017.23 (3894842.14 to 4510320.63) | 2910015.79 (2692721.83 to 3124440.85) | 1825050.71 (1686763.22 to 1960523.11) | 1109960.80 (1025811.82 to 1192450.02) | 639783.16 (591693.82 to 686812.13) | 212639.75 (196091.95 to 229036.93) | 48336.52 (44567.68 to 52386.92) | 9457.15 (8697.78 to 10308.28) |
| India | Female | Number of DALYs | 8162.89 (5509.74 to 11591.41) | 15502.59 (10516.68 to 21897.86) | 16782.13 (11332.87 to 24292.16) | 19838.68 (13306.20 to 29053.74) | 24490.91 (16442.49 to 35338.98) | 27116.33 (18165.02 to 39615.05) | 27688.12 (18382.26 to 40032.42) | 29129.47 (19179.71 to 42725.69) | 26432.55 (17423.25 to 38911.36) | 23950.02 (15865.90 to 34876.05) | 21529.21 (14259.83 to 31074.13) | 19864.69 (12954.29 to 28581.98) | 17315.09 (11495.61 to 24597.00) | 12546.51 (8399.96 to 18031.33) | 7988.78 (5255.40 to 11282.27) | 4863.20 (3261.09 to 6951.61) | 2893.92 (1944.19 to 4048.31) | 980.43 (671.56 to 1382.30) | 221.66 (151.75 to 308.93) | 43.98 (30.00 to 61.07) |
| India | Female | Number of prevalence | 1121333.02 (1031923.92 to 1211437.24) | 2581730.41 (2373894.48 to 2790871.81) | 3097227.36 (2846521.59 to 3353083.20) | 3598966.87 (3309355.29 to 3895798.86) | 4033543.99 (3713809.32 to 4365354.24) | 4059289.03 (3741632.93 to 4391743.74) | 3823110.26 (3527154.55 to 4131705.36) | 3722934.56 (3435350.44 to 4020602.62) | 3188925.26 (2944901.55 to 3442472.20) | 2735600.49 (2527604.28 to 2950491.13) | 2346442.58 (2167633.56 to 2528930.92) | 2079837.17 (1920963.81 to 2240402.79) | 1732685.90 (1600906.85 to 1866434.38) | 1242178.28 (1147658.82 to 1337269.66) | 813361.24 (750245.93 to 876917.73) | 501105.56 (462380.57 to 539229.94) | 295603.48 (272811.73 to 318761.11) | 101760.97 (93749.22 to 109868.94) | 24040.34 (22131.01 to 26017.97) | 4894.00 (4482.35 to 5324.39) |
| India | Male | Number of DALYs | 6305.98 (4214.18 to 9164.61) | 122768.66 (80075.50 to 185094.64) | 135898.92 (89350.19 to 199932.36) | 149158.89 (99177.97 to 214742.16) | 159653.83 (105631.80 to 228786.99) | 153633.84 (102426.61 to 217902.42) | 143210.02 (95266.27 to 202927.99) | 139071.21 (93392.25 to 196185.54) | 121487.05 (82740.31 to 170845.59) | 107246.86 (72573.31 to 149361.93) | 91567.89 (62732.87 to 126507.31) | 80176.11 (55095.45 to 111383.39) | 67349.35 (46378.95 to 92877.83) | 44934.70 (31154.37 to 61554.60) | 26756.44 (18712.97 to 36541.31) | 15587.53 (10966.63 to 21026.12) | 8517.67 (6054.16 to 11506.21) | 2705.37 (1922.90 to 3631.38) | 588.32 (421.40 to 782.88) | 108.52 (77.67 to 143.85) |
| India | Male | Number of prevalence | 1203348.90 (1103326.66 to 1305542.69) | 3721160.96 (3393433.52 to 4103103.73) | 4423767.87 (4054908.76 to 4818370.04) | 5110004.87 (4709744.06 to 5532095.69) | 5553913.28 (5139573.26 to 5989827.98) | 5366638.31 (4975687.55 to 5784715.15) | 5032678.22 (4673263.99 to 5417584.74) | 4903348.55 (4559060.08 to 5279175.39) | 4313056.47 (4011448.53 to 4641527.27) | 3833787.64 (3568310.24 to 4118390.47) | 3297510.46 (3069592.30 to 3539135.95) | 2915311.30 (2714828.11 to 3128628.34) | 2469331.33 (2300858.76 to 2648688.40) | 1667837.51 (1550094.18 to 1791203.29) | 1011689.47 (939277.06 to 1088598.20) | 608855.25 (565845.22 to 654411.31) | 344179.68 (319755.33 to 369866.72) | 110878.78 (102777.39 to 119374.92) | 24296.18 (22339.38 to 26328.21) | 4563.15 (4156.82 to 4991.12) |
| Indonesia | Both | Number of DALYs | 2767.85 (1886.71 to 4010.78) | 24973.05 (14517.83 to 40445.38) | 39089.97 (24757.18 to 58094.85) | 50069.57 (33066.71 to 71865.09) | 52996.78 (35316.51 to 74970.67) | 53196.38 (35405.88 to 74892.72) | 50930.38 (33788.61 to 70627.42) | 44303.36 (29757.69 to 61580.05) | 35549.17 (23903.52 to 49089.53) | 30206.41 (20197.32 to 41261.90) | 26432.71 (17920.21 to 35925.33) | 21161.75 (14444.99 to 28768.53) | 16815.60 (11462.44 to 22623.40) | 11772.27 (8098.21 to 15885.96) | 7060.83 (4912.21 to 9435.70) | 3780.15 (2627.50 to 5001.00) | 1989.36 (1385.98 to 2645.78) | 953.31 (668.35 to 1263.77) | 240.80 (169.61 to 318.54) | 37.55 (26.70 to 48.98) |
| Indonesia | Both | Number of prevalence | 459526.14 (155880.50 to 1113680.77) | 1316683.75 (534166.38 to 3045546.98) | 1847058.28 (763195.43 to 4265990.96) | 2230862.69 (932890.99 to 5187248.28) | 2390956.14 (981116.54 to 5672476.18) | 2430730.04 (993352.50 to 5771921.72) | 2305086.88 (949873.76 to 5414082.57) | 2002125.97 (828472.09 to 4684659.52) | 1651869.03 (678749.12 to 3827520.87) | 1447384.65 (584851.05 to 3392974.66) | 1288808.41 (510530.61 to 3067626.26) | 1058001.09 (409558.63 to 2552986.69) | 861016.85 (328707.13 to 2089398.34) | 618744.43 (236242.84 to 1504735.65) | 384489.47 (146888.99 to 935892.97) | 214447.74 (81738.99 to 519819.27) | 117944.47 (43939.89 to 288424.48) | 57281.90 (21020.68 to 139811.62) | 15394.53 (5555.60 to 38409.74) | 2704.42 (916.83 to 7118.11) |
| Indonesia | Female | Number of DALYs | 1585.54 (1073.59 to 2284.80) | 3151.85 (2125.84 to 4602.98) | 3587.43 (2389.44 to 5074.27) | 3971.18 (2660.30 to 5594.87) | 4322.15 (2903.60 to 6277.90) | 4411.52 (3001.79 to 6450.83) | 4085.33 (2703.92 to 5784.15) | 3475.24 (2355.92 to 5049.45) | 2942.93 (2003.58 to 4217.21) | 2647.69 (1791.19 to 3834.00) | 2378.52 (1627.46 to 3384.83) | 1977.71 (1340.41 to 2847.24) | 1621.94 (1086.76 to 2298.81) | 1157.38 (787.43 to 1640.25) | 724.85 (499.55 to 1017.37) | 406.96 (283.85 to 557.58) | 226.19 (158.21 to 310.84) | 106.78 (73.84 to 146.98) | 29.49 (21.03 to 41.02) | 5.55 (3.85 to 7.59) |
| Indonesia | Female | Number of prevalence | 226093.42 (78211.27 to 545052.52) | 567944.26 (188471.68 to 1414258.35) | 776804.68 (247489.92 to 1970690.98) | 947233.11 (295809.31 to 2446780.91) | 1054614.83 (325512.09 to 2758018.17) | 1077616.38 (333435.50 to 2828468.56) | 985613.32 (307239.99 to 2563386.43) | 834273.67 (261510.86 to 2171158.17) | 702313.50 (218684.17 to 1793325.39) | 630458.33 (190427.30 to 1652329.31) | 563774.55 (163345.96 to 1494122.59) | 468002.13 (132416.51 to 1259211.14) | 381657.21 (107588.09 to 1027716.60) | 275620.37 (78244.01 to 741788.88) | 175639.13 (50033.79 to 473539.24) | 100987.06 (28674.10 to 271046.44) | 57536.95 (16029.94 to 155645.54) | 27899.61 (7519.01 to 75793.89) | 7921.05 (2091.44 to 21924.89) | 1547.60 (377.60 to 4467.80) |
| Indonesia | Male | Number of DALYs | 1182.31 (780.00 to 1701.27) | 21821.20 (12172.76 to 36765.38) | 35502.54 (22226.72 to 53354.84) | 46098.39 (30310.87 to 66784.14) | 48674.63 (32430.96 to 69238.55) | 48784.86 (32353.77 to 68762.34) | 46845.05 (31041.51 to 65088.15) | 40828.12 (27292.32 to 56714.54) | 32606.25 (21788.68 to 45458.97) | 27558.72 (18313.28 to 37981.69) | 24054.19 (16346.42 to 33117.19) | 19184.04 (13069.21 to 26321.69) | 15193.66 (10336.53 to 20656.55) | 10614.89 (7267.44 to 14327.79) | 6335.98 (4371.25 to 8586.29) | 3373.19 (2335.73 to 4519.49) | 1763.17 (1236.93 to 2369.61) | 846.53 (591.37 to 1131.15) | 211.30 (147.82 to 278.81) | 32.00 (22.89 to 42.51) |
| Indonesia | Male | Number of prevalence | 233432.71 (77868.89 to 568628.25) | 748739.49 (334886.20 to 1638168.53) | 1070253.60 (503690.31 to 2311059.83) | 1283629.58 (625538.69 to 2748006.36) | 1336341.31 (650362.04 to 2921129.78) | 1353113.66 (659618.70 to 2958428.60) | 1319473.57 (638285.02 to 2884122.64) | 1167852.30 (564301.39 to 2519899.25) | 949555.53 (458058.58 to 2029389.30) | 816926.32 (391670.50 to 1751206.56) | 725033.85 (341960.80 to 1579032.31) | 589998.96 (274473.02 to 1293886.70) | 479359.64 (220813.88 to 1061681.74) | 343124.06 (157976.96 to 760404.80) | 208850.34 (97075.00 to 462353.73) | 113460.68 (53090.72 to 248772.83) | 60407.52 (28239.69 to 132778.95) | 29382.29 (13654.80 to 64181.95) | 7473.47 (3464.59 to 16484.85) | 1156.82 (528.50 to 2640.05) |
| Kenya | Both | Number of DALYs | 525.17 (353.95 to 749.02) | 4302.58 (2698.53 to 6615.72) | 5623.39 (3654.03 to 8312.18) | 6180.40 (4130.50 to 8822.16) | 5682.89 (3768.55 to 8076.59) | 5076.43 (3394.46 to 7161.59) | 4418.93 (2937.64 to 6131.17) | 3639.66 (2437.24 to 5105.64) | 2995.34 (2018.85 to 4198.30) | 2473.51 (1676.79 to 3447.01) | 2024.25 (1374.98 to 2830.72) | 1717.50 (1172.39 to 2384.81) | 1411.26 (969.16 to 1947.91) | 1061.10 (731.49 to 1457.37) | 725.41 (503.46 to 993.85) | 452.20 (314.97 to 610.98) | 259.46 (181.63 to 347.80) | 101.56 (71.79 to 135.28) | 21.11 (15.00 to 28.04) | 3.78 (2.70 to 5.00) |
| Kenya | Both | Number of prevalence | 87952.92 (26891.80 to 229668.66) | 216684.77 (82435.64 to 526562.37) | 273002.22 (104272.66 to 661060.20) | 301171.49 (115867.89 to 726364.46) | 289210.02 (110769.14 to 683387.78) | 264054.00 (103008.50 to 619446.26) | 226009.21 (90406.74 to 522522.66) | 184874.29 (74882.97 to 425572.89) | 152170.37 (62500.11 to 343726.34) | 126744.49 (51888.14 to 288864.33) | 104879.80 (42597.91 to 239130.76) | 89838.51 (36240.76 to 204698.43) | 74572.19 (29599.52 to 169425.06) | 56664.44 (21979.40 to 129639.40) | 38675.60 (14920.94 to 88914.25) | 24037.41 (9212.88 to 55495.18) | 14294.87 (5510.06 to 32859.83) | 5823.86 (2307.85 to 13103.88) | 1286.43 (505.36 to 2880.25) | 255.06 (96.25 to 579.52) |
| Kenya | Female | Number of DALYs | 304.70 (206.78 to 436.21) | 526.84 (352.95 to 758.41) | 569.63 (390.72 to 813.61) | 620.42 (418.91 to 889.81) | 656.36 (446.03 to 950.05) | 654.61 (437.97 to 950.65) | 588.02 (392.76 to 847.27) | 502.52 (337.90 to 731.31) | 431.23 (286.63 to 629.91) | 369.09 (245.88 to 539.54) | 317.09 (211.83 to 458.14) | 280.41 (187.46 to 409.00) | 235.56 (157.99 to 337.44) | 173.16 (117.78 to 247.52) | 110.90 (74.50 to 159.60) | 63.32 (42.94 to 89.47) | 37.50 (25.64 to 52.29) | 15.88 (10.87 to 21.88) | 3.58 (2.47 to 4.97) | 0.71 (0.50 to 0.97) |
| Kenya | Female | Number of prevalence | 43928.35 (13573.12 to 114144.22) | 94661.16 (27635.54 to 249673.88) | 117901.96 (33112.89 to 312331.53) | 132900.95 (37459.88 to 350540.00) | 134134.47 (39264.51 to 345888.63) | 124590.26 (38001.92 to 315956.26) | 103741.67 (32906.42 to 261529.94) | 83836.93 (26769.52 to 211142.61) | 68685.81 (22244.16 to 168985.68) | 57539.64 (17960.78 to 142953.17) | 48138.91 (14878.30 to 119581.35) | 41287.56 (12579.62 to 101805.30) | 34314.82 (10258.76 to 84407.11) | 25964.23 (7571.73 to 64277.12) | 17088.04 (4985.37 to 42755.43) | 10125.15 (2867.59 to 25420.45) | 6189.97 (1737.11 to 15681.63) | 2626.46 (757.03 to 6607.59) | 617.57 (176.54 to 1551.58) | 134.84 (36.22 to 341.81) |
| Kenya | Male | Number of DALYs | 220.47 (146.83 to 314.61) | 3775.75 (2273.26 to 6001.24) | 5053.76 (3264.25 to 7522.72) | 5559.98 (3664.12 to 7962.37) | 5026.53 (3296.35 to 7131.64) | 4421.82 (2931.77 to 6254.65) | 3830.91 (2551.78 to 5385.55) | 3137.14 (2095.66 to 4376.36) | 2564.11 (1700.39 to 3576.82) | 2104.41 (1412.89 to 2922.00) | 1707.15 (1157.97 to 2371.55) | 1437.09 (981.90 to 1999.22) | 1175.70 (804.53 to 1617.05) | 887.94 (613.76 to 1210.23) | 614.51 (428.11 to 832.42) | 388.87 (272.05 to 524.33) | 221.96 (155.19 to 297.20) | 85.68 (60.45 to 114.02) | 17.54 (12.43 to 23.19) | 3.07 (2.18 to 4.02) |
| Kenya | Male | Number of prevalence | 44024.57 (13296.04 to 115524.44) | 122023.61 (53874.49 to 278817.63) | 155100.26 (70340.95 to 350113.01) | 168270.54 (77832.98 to 375824.46) | 155075.56 (71648.93 to 339027.01) | 139463.74 (65096.69 to 304637.78) | 122267.54 (57281.05 to 264867.38) | 101037.36 (47174.21 to 217919.91) | 83484.56 (39448.95 to 178559.92) | 69204.85 (32742.69 to 146190.52) | 56740.88 (26719.97 to 120927.09) | 48550.95 (23061.38 to 102647.64) | 40257.37 (19131.02 to 85172.78) | 30700.21 (14341.97 to 65466.66) | 21587.57 (9894.16 to 46298.27) | 13912.26 (6315.99 to 29924.94) | 8104.90 (3785.03 to 17195.11) | 3197.40 (1553.35 to 6565.19) | 668.86 (330.21 to 1355.72) | 120.22 (59.78 to 240.93) |
| Kiribati | Both | Number of DALYs | 2.25 (1.45 to 3.20) | 40.62 (26.94 to 57.11) | 38.90 (26.51 to 54.90) | 39.16 (26.48 to 54.57) | 45.77 (30.92 to 64.03) | 46.05 (31.18 to 63.55) | 41.06 (28.36 to 57.86) | 37.24 (25.39 to 53.30) | 32.15 (21.84 to 45.88) | 28.14 (19.20 to 39.45) | 24.50 (16.56 to 34.59) | 21.34 (14.44 to 30.20) | 18.51 (12.51 to 25.79) | 14.89 (10.23 to 20.99) | 9.08 (6.19 to 12.62) | 4.87 (3.30 to 6.77) | 2.12 (1.44 to 2.95) | 0.70 (0.48 to 0.97) | 0.17 (0.12 to 0.24) | 0.04 (0.03 to 0.05) |
| Kiribati | Both | Number of prevalence | 643.71 (30.65 to 1258.36) | 1681.77 (351.84 to 2999.08) | 1887.81 (346.78 to 3428.05) | 2259.49 (367.13 to 4166.38) | 2840.81 (437.64 to 5254.51) | 2860.42 (447.55 to 5280.62) | 2502.75 (405.26 to 4605.40) | 2205.81 (369.43 to 4042.49) | 1835.69 (319.71 to 3326.95) | 1562.98 (282.52 to 2756.01) | 1319.25 (246.24 to 2243.64) | 1114.72 (217.50 to 1838.92) | 928.62 (190.67 to 1487.67) | 750.22 (156.21 to 1189.17) | 474.02 (97.75 to 752.88) | 263.22 (53.53 to 418.88) | 117.16 (23.73 to 186.55) | 39.77 (8.02 to 63.37) | 10.33 (2.06 to 16.49) | 2.43 (0.48 to 3.88) |
| Kiribati | Female | Number of DALYs | 1.28 (0.80 to 1.87) | 2.76 (1.81 to 4.12) | 3.56 (2.35 to 5.14) | 5.70 (3.68 to 8.54) | 9.74 (6.28 to 14.67) | 12.05 (7.78 to 17.89) | 12.32 (7.96 to 17.89) | 12.02 (7.81 to 17.36) | 10.61 (6.86 to 15.90) | 9.65 (6.20 to 14.18) | 8.87 (5.73 to 13.39) | 8.21 (5.32 to 11.80) | 7.45 (4.81 to 10.83) | 6.54 (4.23 to 9.57) | 4.33 (2.82 to 6.31) | 2.50 (1.62 to 3.61) | 1.11 (0.72 to 1.60) | 0.38 (0.25 to 0.55) | 0.10 (0.07 to 0.15) | 0.02 (0.02 to 0.04) |
| Kiribati | Female | Number of prevalence | 313.68 (17.00 to 611.47) | 682.20 (36.39 to 1330.77) | 792.49 (45.69 to 1542.16) | 1003.82 (69.13 to 1940.84) | 1336.87 (114.63 to 2563.55) | 1380.46 (139.22 to 2629.51) | 1234.72 (139.38 to 2336.63) | 1090.77 (134.25 to 2052.07) | 886.65 (117.14 to 1658.18) | 756.93 (106.84 to 1393.89) | 649.81 (97.77 to 1146.64) | 564.93 (91.36 to 961.24) | 478.09 (83.25 to 787.17) | 411.09 (73.92 to 667.80) | 277.11 (49.74 to 450.16) | 163.03 (29.30 to 264.85) | 73.88 (13.30 to 120.02) | 25.62 (4.59 to 41.61) | 7.02 (1.26 to 11.40) | 1.77 (0.32 to 2.88) |
| Kiribati | Male | Number of DALYs | 0.97 (0.57 to 1.57) | 37.86 (25.08 to 52.84) | 35.34 (23.98 to 50.08) | 33.46 (22.81 to 46.99) | 36.03 (24.45 to 49.73) | 34.00 (23.29 to 46.52) | 28.74 (19.72 to 39.64) | 25.22 (17.22 to 35.65) | 21.54 (14.82 to 30.28) | 18.49 (12.68 to 26.30) | 15.62 (10.76 to 21.94) | 13.14 (9.11 to 18.74) | 11.06 (7.67 to 15.41) | 8.35 (5.79 to 11.72) | 4.76 (3.33 to 6.61) | 2.37 (1.66 to 3.37) | 1.01 (0.71 to 1.41) | 0.32 (0.23 to 0.45) | 0.07 (0.05 to 0.10) | 0.01 (0.01 to 0.02) |
| Kiribati | Male | Number of prevalence | 330.02 (13.86 to 646.89) | 999.57 (307.72 to 1674.72) | 1095.32 (303.71 to 1889.47) | 1255.67 (292.57 to 2221.50) | 1503.94 (324.79 to 2685.01) | 1479.96 (310.17 to 2649.53) | 1268.02 (264.38 to 2267.87) | 1115.04 (233.74 to 1990.19) | 949.04 (201.77 to 1668.77) | 806.05 (174.79 to 1362.12) | 669.44 (149.34 to 1097.00) | 549.79 (126.51 to 877.68) | 450.53 (108.52 to 700.50) | 339.13 (82.98 to 521.36) | 196.91 (48.24 to 302.72) | 100.19 (24.54 to 154.03) | 43.27 (10.60 to 66.52) | 14.15 (3.47 to 21.76) | 3.31 (0.81 to 5.08) | 0.65 (0.16 to 1.01) |
| Lao People's Democratic Republic | Both | Number of DALYs | 69.63 (42.80 to 106.22) | 230.13 (120.63 to 422.68) | 229.72 (121.68 to 409.32) | 223.70 (122.88 to 396.16) | 208.37 (115.92 to 357.50) | 208.79 (114.96 to 358.42) | 199.81 (109.99 to 337.12) | 170.61 (95.10 to 292.15) | 143.82 (81.89 to 237.15) | 127.25 (71.67 to 212.66) | 112.83 (66.32 to 187.27) | 97.24 (57.52 to 159.23) | 83.88 (48.45 to 134.53) | 68.21 (40.01 to 109.36) | 45.58 (27.19 to 72.92) | 25.19 (15.25 to 39.64) | 9.35 (5.55 to 14.93) | 2.26 (1.40 to 3.49) | 0.42 (0.27 to 0.64) | 0.07 (0.04 to 0.10) |
| Lao People's Democratic Republic | Both | Number of prevalence | 4265.63 (840.26 to 18642.84) | 9921.74 (2140.84 to 41220.36) | 11885.90 (2291.41 to 51022.89) | 12861.09 (2313.45 to 56228.40) | 12549.51 (2180.13 to 55308.79) | 12629.62 (2201.67 to 55676.67) | 11971.19 (2116.43 to 52677.05) | 10152.44 (1807.77 to 44649.90) | 8538.29 (1532.71 to 37522.27) | 7625.36 (1359.29 to 33546.11) | 6829.29 (1220.99 to 30051.80) | 5899.96 (1061.75 to 25936.59) | 4959.71 (916.58 to 21697.03) | 4048.75 (753.46 to 17692.58) | 2838.93 (518.08 to 12450.39) | 1614.13 (290.82 to 7096.88) | 643.30 (111.59 to 2848.55) | 191.91 (29.11 to 868.78) | 39.77 (5.76 to 181.40) | 7.22 (0.98 to 33.28) |
| Lao People's Democratic Republic | Female | Number of DALYs | 40.18 (22.32 to 63.47) | 62.58 (36.36 to 100.26) | 55.58 (32.10 to 87.28) | 50.07 (30.43 to 77.91) | 43.79 (25.36 to 68.76) | 39.09 (23.89 to 61.68) | 33.54 (20.99 to 50.69) | 27.01 (16.46 to 41.65) | 22.00 (13.13 to 33.68) | 19.54 (12.21 to 29.20) | 17.18 (10.20 to 26.21) | 14.31 (8.65 to 21.61) | 11.14 (6.88 to 16.70) | 8.81 (5.55 to 13.19) | 6.33 (3.75 to 9.45) | 3.65 (2.30 to 5.30) | 1.54 (0.95 to 2.30) | 0.57 (0.37 to 0.82) | 0.12 (0.08 to 0.18) | 0.02 (0.02 to 0.03) |
| Lao People's Democratic Republic | Female | Number of prevalence | 2159.73 (472.61 to 9245.87) | 4494.99 (778.62 to 20098.09) | 5439.17 (767.64 to 25062.65) | 6118.45 (743.83 to 28701.38) | 6165.18 (680.10 to 29193.07) | 6079.70 (636.42 to 28929.00) | 5596.17 (568.06 to 26702.60) | 4721.05 (468.17 to 22569.26) | 3956.31 (385.74 to 18940.37) | 3602.46 (345.73 to 17267.46) | 3259.13 (308.79 to 15636.64) | 2805.70 (262.80 to 13474.10) | 2246.42 (208.07 to 10799.28) | 1821.90 (167.16 to 8762.12) | 1332.35 (122.54 to 6407.73) | 780.13 (71.70 to 3751.95) | 335.98 (30.87 to 1615.88) | 124.00 (11.41 to 596.34) | 27.42 (2.52 to 131.87) | 5.41 (0.50 to 26.03) |
| Lao People's Democratic Republic | Male | Number of DALYs | 29.45 (16.59 to 47.00) | 167.55 (74.48 to 348.00) | 174.13 (78.58 to 340.52) | 173.62 (83.26 to 331.54) | 164.58 (82.95 to 300.53) | 169.70 (83.46 to 307.72) | 166.27 (84.92 to 297.35) | 143.60 (74.14 to 257.55) | 121.83 (64.23 to 206.13) | 107.71 (56.12 to 186.24) | 95.65 (54.29 to 166.63) | 82.92 (45.62 to 141.49) | 72.74 (40.33 to 122.35) | 59.40 (33.02 to 98.48) | 39.25 (21.82 to 65.72) | 21.54 (12.16 to 34.98) | 7.81 (4.27 to 12.94) | 1.69 (0.96 to 2.83) | 0.30 (0.17 to 0.49) | 0.04 (0.02 to 0.07) |
| Lao People's Democratic Republic | Male | Number of prevalence | 2105.90 (360.15 to 9396.98) | 5426.75 (1313.68 to 21122.26) | 6446.73 (1424.67 to 25960.24) | 6742.64 (1441.99 to 27527.03) | 6384.34 (1375.51 to 26115.72) | 6549.92 (1439.18 to 26747.67) | 6375.02 (1431.41 to 25974.45) | 5431.39 (1234.91 to 22080.64) | 4581.99 (1058.66 to 18581.90) | 4022.90 (941.19 to 16278.65) | 3570.16 (842.68 to 14415.16) | 3094.27 (740.20 to 12462.49) | 2713.28 (659.14 to 10897.75) | 2226.85 (542.48 to 8930.46) | 1506.58 (367.93 to 6042.67) | 834.00 (203.46 to 3344.93) | 307.32 (74.95 to 1232.67) | 67.91 (16.58 to 272.44) | 12.35 (3.01 to 49.53) | 1.81 (0.44 to 7.25) |
| Liberia | Both | Number of DALYs | 68.56 (43.90 to 102.91) | 927.73 (588.00 to 1349.21) | 1114.20 (739.74 to 1552.12) | 1058.13 (707.49 to 1464.61) | 940.90 (629.99 to 1294.16) | 924.01 (627.57 to 1280.42) | 793.44 (548.77 to 1094.65) | 753.20 (515.91 to 1043.14) | 501.87 (342.91 to 696.28) | 432.96 (298.46 to 602.07) | 358.44 (246.56 to 496.22) | 308.29 (213.24 to 427.16) | 271.66 (187.67 to 373.67) | 246.87 (172.83 to 337.90) | 197.18 (137.99 to 266.39) | 111.20 (78.49 to 151.71) | 51.57 (36.61 to 68.64) | 17.10 (12.12 to 22.86) | 4.15 (2.96 to 5.49) | 0.79 (0.57 to 1.06) |
| Liberia | Both | Number of prevalence | 16657.88 (2837.55 to 43675.39) | 41742.97 (12883.21 to 99919.65) | 46865.52 (15056.98 to 110648.24) | 50725.40 (14933.45 to 121430.15) | 52254.06 (14376.35 to 126937.31) | 55863.59 (14779.57 to 136673.52) | 48071.08 (12756.43 to 117565.14) | 45685.22 (12121.83 to 111693.84) | 30620.54 (8142.37 to 74882.19) | 26543.26 (7066.39 to 64953.42) | 22263.31 (5936.14 to 54563.63) | 19044.51 (5121.23 to 46690.30) | 17253.57 (4608.98 to 41845.75) | 15624.77 (4208.59 to 37497.93) | 12507.54 (3387.78 to 29958.49) | 7177.05 (1950.35 to 17182.85) | 3419.89 (924.04 to 8199.02) | 1187.12 (317.21 to 2856.24) | 305.69 (80.36 to 739.13) | 66.35 (16.71 to 162.36) |
| Liberia | Female | Number of DALYs | 39.75 (24.51 to 60.00) | 72.27 (46.45 to 110.29) | 75.38 (48.37 to 111.50) | 92.07 (60.54 to 134.51) | 111.81 (73.04 to 164.72) | 136.06 (87.94 to 195.52) | 120.73 (79.58 to 173.33) | 117.65 (77.95 to 169.65) | 81.27 (54.13 to 117.96) | 72.34 (48.17 to 104.47) | 63.70 (42.14 to 94.03) | 54.94 (35.77 to 79.71) | 54.43 (36.03 to 76.70) | 47.97 (32.08 to 68.90) | 36.50 (24.65 to 52.43) | 20.45 (13.72 to 28.91) | 9.86 (6.53 to 14.05) | 3.60 (2.40 to 5.06) | 0.99 (0.66 to 1.42) | 0.25 (0.17 to 0.35) |
| Liberia | Female | Number of prevalence | 8320.42 (1459.24 to 21728.75) | 17611.18 (2993.45 to 46181.20) | 19229.85 (3244.92 to 50478.89) | 22334.62 (3830.09 to 58527.95) | 24422.83 (4304.46 to 63791.37) | 27067.33 (4904.95 to 70463.18) | 22258.46 (4119.56 to 57790.12) | 20437.05 (3862.69 to 52928.58) | 13405.77 (2576.69 to 34641.43) | 11440.84 (2228.68 to 29515.00) | 9623.94 (1903.70 to 24800.53) | 7952.86 (1597.02 to 20479.85) | 7520.87 (1541.88 to 19353.07) | 6536.25 (1353.12 to 16811.74) | 5038.00 (1042.21 to 12957.19) | 2865.35 (592.90 to 7368.87) | 1403.58 (290.76 to 3609.61) | 521.47 (107.96 to 1341.18) | 146.55 (30.33 to 376.90) | 38.35 (7.94 to 98.63) |
| Liberia | Male | Number of DALYs | 28.81 (17.01 to 46.19) | 855.46 (534.17 to 1264.58) | 1038.82 (686.42 to 1443.70) | 966.06 (642.91 to 1356.22) | 829.09 (551.19 to 1131.43) | 787.95 (535.56 to 1100.33) | 672.71 (457.18 to 927.58) | 635.55 (430.86 to 887.66) | 420.60 (287.97 to 585.16) | 360.62 (244.93 to 501.89) | 294.74 (201.47 to 406.59) | 253.35 (173.67 to 350.59) | 217.22 (150.66 to 301.28) | 198.90 (139.50 to 274.56) | 160.67 (110.43 to 216.52) | 90.76 (63.83 to 124.13) | 41.71 (29.44 to 55.68) | 13.50 (9.53 to 18.20) | 3.16 (2.25 to 4.17) | 0.54 (0.39 to 0.72) |
| Liberia | Male | Number of prevalence | 8337.46 (1378.78 to 21946.64) | 24131.79 (9628.10 to 53404.73) | 27635.67 (11514.70 to 59806.24) | 28390.78 (11158.50 to 62895.86) | 27831.23 (9999.26 to 63145.94) | 28796.27 (9818.85 to 66210.34) | 25812.62 (8586.90 to 59775.02) | 25248.18 (8219.91 to 58765.26) | 17214.78 (5543.38 to 40240.76) | 15102.41 (4817.62 to 35438.43) | 12639.37 (4010.63 to 29763.10) | 11091.65 (3505.17 to 26210.45) | 9732.70 (3051.04 to 22492.68) | 9088.52 (2839.61 to 20686.19) | 7469.55 (2333.68 to 17001.30) | 4311.69 (1351.67 to 9813.98) | 2016.31 (629.84 to 4589.42) | 665.65 (208.19 to 1515.07) | 159.14 (49.83 to 362.23) | 28.00 (8.77 to 63.73) |
| Madagascar | Both | Number of DALYs | 254.59 (167.83 to 374.02) | 1963.31 (1170.36 to 3282.47) | 2863.86 (1823.21 to 4308.23) | 3651.02 (2431.10 to 5243.86) | 3726.73 (2489.69 to 5281.71) | 3576.49 (2367.20 to 5013.44) | 3215.21 (2161.43 to 4418.97) | 2540.25 (1688.54 to 3519.50) | 1912.37 (1311.55 to 2680.85) | 1517.19 (1045.93 to 2096.98) | 1308.19 (895.21 to 1798.35) | 1173.74 (809.16 to 1619.73) | 964.11 (674.33 to 1299.82) | 742.99 (508.75 to 997.26) | 485.98 (337.40 to 653.34) | 261.68 (181.53 to 351.64) | 139.24 (97.31 to 186.09) | 59.55 (42.39 to 79.00) | 15.26 (10.85 to 20.47) | 3.44 (2.47 to 4.56) |
| Madagascar | Both | Number of prevalence | 45001.39 (15040.33 to 95254.27) | 108132.16 (42893.37 to 218104.36) | 137930.88 (56743.36 to 273707.14) | 159998.08 (67945.51 to 313904.04) | 160971.13 (69494.71 to 315155.77) | 156381.35 (67800.87 to 306286.87) | 142753.60 (61784.76 to 280081.33) | 116012.50 (49842.40 to 228282.00) | 90272.82 (38446.39 to 178176.78) | 74371.12 (31298.49 to 147309.98) | 66132.43 (27636.18 to 131278.82) | 60360.61 (25141.69 to 119929.50) | 50692.23 (21029.21 to 100840.54) | 39114.51 (16254.59 to 77703.35) | 25693.82 (10740.02 to 50987.71) | 14362.99 (5975.74 to 28562.12) | 8045.60 (3316.60 to 16045.30) | 3488.96 (1441.05 to 6953.07) | 916.19 (377.62 to 1826.65) | 235.09 (94.25 to 473.10) |
| Madagascar | Female | Number of DALYs | 146.74 (91.94 to 220.61) | 248.39 (159.73 to 386.68) | 256.30 (158.70 to 397.45) | 269.58 (170.61 to 393.36) | 268.09 (172.37 to 398.64) | 259.70 (165.27 to 380.87) | 236.22 (155.16 to 338.15) | 194.88 (127.14 to 285.63) | 156.96 (102.60 to 233.35) | 133.06 (88.82 to 193.25) | 120.19 (77.78 to 174.65) | 110.30 (72.07 to 161.86) | 91.42 (60.79 to 128.70) | 68.19 (47.03 to 95.42) | 42.92 (28.68 to 60.65) | 24.31 (16.46 to 34.27) | 14.17 (9.42 to 20.09) | 5.93 (3.96 to 8.43) | 1.54 (1.05 to 2.14) | 0.45 (0.31 to 0.62) |
| Madagascar | Female | Number of prevalence | 22451.97 (7593.49 to 47319.69) | 47754.76 (15713.23 to 101395.90) | 59142.89 (19137.31 to 126139.66) | 67838.17 (21811.13 to 144971.47) | 69271.21 (22271.48 to 148108.99) | 66775.98 (21498.50 to 142764.80) | 60365.07 (19469.39 to 129029.97) | 49501.73 (15994.56 to 105791.35) | 39040.65 (12638.87 to 83400.27) | 32898.05 (10669.81 to 70256.83) | 29433.44 (9562.85 to 62841.11) | 26429.35 (8603.11 to 56406.97) | 21818.09 (7121.45 to 46555.43) | 16209.68 (5298.52 to 34577.87) | 10377.73 (3391.89 to 22139.11) | 5992.55 (1958.59 to 12782.76) | 3548.81 (1159.85 to 7570.56) | 1517.34 (495.86 to 3236.56) | 403.91 (132.02 to 861.60) | 120.86 (39.49 to 257.80) |
| Madagascar | Male | Number of DALYs | 107.85 (62.16 to 168.39) | 1714.92 (939.59 to 2862.69) | 2607.56 (1620.34 to 3983.50) | 3381.44 (2239.72 to 4884.30) | 3458.64 (2306.60 to 4912.63) | 3316.79 (2185.56 to 4649.04) | 2978.99 (1994.70 to 4128.65) | 2345.37 (1547.88 to 3254.29) | 1755.41 (1193.69 to 2466.25) | 1384.12 (951.33 to 1928.85) | 1188.00 (811.46 to 1619.03) | 1063.44 (725.09 to 1471.13) | 872.69 (606.55 to 1189.13) | 674.80 (458.98 to 907.12) | 443.06 (309.18 to 599.73) | 237.37 (164.69 to 319.77) | 125.08 (86.93 to 167.54) | 53.62 (37.88 to 70.62) | 13.72 (9.74 to 18.44) | 2.99 (2.14 to 3.96) |
| Madagascar | Male | Number of prevalence | 22549.42 (7446.84 to 47934.58) | 60377.40 (26658.35 to 116317.54) | 78787.99 (37799.08 to 147688.15) | 92159.91 (46436.92 to 168441.21) | 91699.92 (47043.40 to 167162.22) | 89605.37 (45866.61 to 163581.35) | 82388.53 (41779.21 to 151081.85) | 66510.77 (33436.37 to 122495.39) | 51232.17 (25549.81 to 94776.51) | 41473.07 (20597.06 to 77053.15) | 36698.99 (18065.64 to 68437.72) | 33931.26 (16537.97 to 63522.53) | 28874.14 (13905.40 to 54285.11) | 22904.83 (10959.98 to 43125.48) | 15316.09 (7347.29 to 28848.60) | 8370.43 (4016.30 to 15779.35) | 4496.80 (2156.20 to 8474.74) | 1971.63 (944.98 to 3716.51) | 512.28 (245.57 to 965.05) | 114.23 (54.80 to 215.30) |
| Malawi | Both | Number of DALYs | 249.53 (159.61 to 379.38) | 2792.87 (1746.58 to 4178.72) | 3966.50 (2642.03 to 5704.84) | 4360.71 (2918.10 to 6131.81) | 4182.22 (2818.01 to 5827.61) | 3574.80 (2438.87 to 4972.51) | 2841.13 (1938.27 to 3914.22) | 2267.08 (1558.97 to 3084.21) | 1931.79 (1322.80 to 2684.59) | 1474.27 (1016.54 to 2030.83) | 1173.43 (799.25 to 1598.91) | 971.91 (667.59 to 1344.39) | 834.22 (578.10 to 1145.38) | 673.08 (468.07 to 917.40) | 417.38 (290.62 to 563.87) | 217.07 (151.03 to 294.17) | 94.26 (66.22 to 128.18) | 34.16 (24.47 to 46.20) | 8.63 (6.20 to 11.46) | 1.64 (1.19 to 2.17) |
| Malawi | Both | Number of prevalence | 56272.83 (11493.03 to 150884.10) | 133800.19 (40366.08 to 330807.48) | 168623.53 (54938.94 to 410817.36) | 192509.57 (63231.18 to 469547.94) | 207664.44 (65062.53 to 511794.51) | 190381.73 (58002.46 to 471976.06) | 157320.69 (47390.45 to 390928.48) | 130291.55 (38699.00 to 324700.57) | 114169.32 (33642.84 to 285195.38) | 88862.22 (26124.96 to 222275.77) | 72713.80 (21188.15 to 182265.89) | 62005.48 (17949.60 to 155759.51) | 54890.65 (15770.55 to 138253.70) | 45317.21 (12991.59 to 114264.08) | 28887.21 (8245.15 to 72900.96) | 15527.18 (4419.74 to 39223.61) | 6849.70 (1952.61 to 17292.16) | 2523.39 (721.50 to 6367.45) | 672.23 (189.78 to 1700.73) | 143.61 (39.17 to 366.10) |
| Malawi | Female | Number of DALYs | 146.02 (89.94 to 223.63) | 250.92 (161.18 to 379.61) | 276.10 (179.25 to 422.61) | 328.48 (210.03 to 474.67) | 393.61 (253.60 to 571.45) | 388.61 (256.28 to 560.62) | 335.07 (219.40 to 483.49) | 295.70 (195.49 to 424.81) | 268.93 (179.52 to 397.72) | 218.06 (141.43 to 320.98) | 188.20 (125.15 to 269.02) | 169.64 (111.48 to 251.83) | 162.02 (103.73 to 236.62) | 135.45 (90.64 to 193.40) | 86.20 (58.53 to 122.85) | 46.36 (31.00 to 67.97) | 19.71 (13.37 to 28.63) | 7.01 (4.81 to 9.97) | 1.92 (1.31 to 2.71) | 0.46 (0.31 to 0.63) |
| Malawi | Female | Number of prevalence | 28264.82 (5904.54 to 75436.84) | 57818.63 (11684.37 to 155175.41) | 70552.75 (14051.71 to 189803.20) | 82518.93 (16480.04 to 221901.45) | 93858.50 (18972.82 to 251940.54) | 86540.35 (17705.57 to 231823.07) | 70737.35 (14637.75 to 189150.18) | 59527.47 (12428.48 to 158924.38) | 52382.51 (11038.63 to 139694.01) | 40678.52 (8656.47 to 108367.92) | 33920.89 (7277.77 to 90286.06) | 29476.03 (6382.85 to 78375.73) | 26802.76 (5867.82 to 71172.29) | 22335.18 (4919.65 to 59271.16) | 14480.06 (3190.37 to 38429.15) | 7924.97 (1745.27 to 21031.90) | 3457.70 (761.22 to 9176.29) | 1261.53 (278.04 to 3347.82) | 353.64 (77.81 to 938.51) | 86.17 (18.96 to 228.68) |
| Malawi | Male | Number of DALYs | 103.51 (57.60 to 166.50) | 2541.95 (1571.81 to 3855.52) | 3690.40 (2443.26 to 5340.56) | 4032.23 (2701.53 to 5706.82) | 3788.61 (2549.11 to 5290.51) | 3186.18 (2164.82 to 4400.76) | 2506.05 (1708.91 to 3471.84) | 1971.38 (1338.30 to 2686.54) | 1662.87 (1125.80 to 2317.20) | 1256.21 (853.77 to 1735.40) | 985.23 (666.34 to 1353.17) | 802.27 (548.77 to 1118.41) | 672.20 (463.08 to 927.24) | 537.63 (370.91 to 739.01) | 331.17 (228.61 to 445.24) | 170.71 (117.89 to 229.97) | 74.55 (52.05 to 101.88) | 27.14 (19.13 to 36.14) | 6.71 (4.70 to 8.99) | 1.18 (0.84 to 1.58) |
| Malawi | Male | Number of prevalence | 28008.01 (5588.49 to 75447.26) | 75981.56 (29398.25 to 174975.89) | 98070.78 (41487.89 to 220481.17) | 109990.64 (46584.96 to 247077.46) | 113805.94 (45675.58 to 259853.97) | 103841.38 (40293.60 to 240152.99) | 86583.34 (32750.76 to 201778.30) | 70764.08 (26264.96 to 165776.18) | 61786.81 (22525.47 to 145501.37) | 48183.70 (17248.83 to 113907.85) | 38792.92 (13708.03 to 91979.82) | 32529.45 (11384.68 to 77384.03) | 28087.89 (9755.22 to 67083.26) | 22982.03 (7963.74 to 54995.56) | 14407.15 (4979.93 to 34473.36) | 7602.21 (2633.07 to 18192.52) | 3392.00 (1173.73 to 8116.23) | 1261.86 (436.63 to 3019.78) | 318.59 (110.10 to 762.26) | 57.44 (19.87 to 137.43) |
| Malaysia | Both | Number of DALYs | 248.90 (153.17 to 372.75) | 905.27 (478.98 to 1635.22) | 1012.53 (553.03 to 1845.06) | 1132.41 (594.01 to 1968.73) | 1264.28 (698.59 to 2147.99) | 1351.95 (745.30 to 2274.38) | 1349.24 (780.63 to 2241.55) | 1238.46 (714.10 to 2008.57) | 1056.10 (619.64 to 1708.08) | 811.22 (470.24 to 1321.23) | 758.38 (443.28 to 1205.58) | 538.17 (332.60 to 852.99) | 500.09 (300.60 to 787.12) | 312.44 (189.22 to 477.06) | 228.35 (141.88 to 355.13) | 142.92 (86.43 to 217.35) | 98.36 (61.00 to 152.67) | 43.32 (26.64 to 66.99) | 18.25 (11.44 to 27.90) | 7.38 (4.65 to 11.28) |
| Malaysia | Both | Number of prevalence | 17707.45 (3591.18 to 67853.48) | 43108.73 (9884.67 to 161127.78) | 54541.34 (11266.36 to 207725.09) | 65535.24 (12970.78 to 251372.20) | 73412.29 (14562.46 to 281270.59) | 78077.33 (15711.32 to 298441.34) | 76063.17 (15571.28 to 289902.37) | 68757.72 (14299.95 to 261432.12) | 57780.87 (12255.19 to 219041.34) | 43742.93 (9457.39 to 165390.86) | 41510.35 (9029.02 to 156830.77) | 29955.08 (6514.96 to 113247.70) | 27641.16 (6081.55 to 104232.18) | 17954.66 (3927.44 to 67850.03) | 13420.77 (2934.90 to 50721.74) | 8542.74 (1869.34 to 32285.39) | 6040.89 (1314.90 to 22849.68) | 2694.24 (589.54 to 10183.51) | 1276.73 (268.34 to 4875.75) | 513.56 (109.38 to 1954.49) |
| Malaysia | Female | Number of DALYs | 142.60 (84.46 to 220.94) | 227.61 (131.04 to 356.38) | 218.62 (128.96 to 341.22) | 211.00 (130.44 to 316.98) | 203.99 (121.32 to 308.90) | 199.37 (123.07 to 294.69) | 181.07 (106.97 to 276.24) | 157.51 (93.80 to 241.43) | 124.90 (75.40 to 184.76) | 91.20 (54.83 to 137.43) | 84.45 (52.33 to 126.19) | 62.34 (39.78 to 92.15) | 55.60 (35.93 to 80.85) | 36.48 (23.09 to 54.37) | 26.51 (16.45 to 39.02) | 16.71 (10.75 to 24.29) | 11.60 (7.43 to 16.62) | 5.00 (3.22 to 7.18) | 2.64 (1.67 to 3.89) | 0.98 (0.63 to 1.40) |
| Malaysia | Female | Number of prevalence | 8799.55 (1984.57 to 33129.48) | 19322.78 (3606.63 to 75338.59) | 24596.94 (3913.90 to 98229.14) | 29770.43 (4258.64 to 120598.08) | 33439.16 (4509.49 to 136439.54) | 35323.22 (4610.18 to 144665.31) | 33968.65 (4354.79 to 139411.71) | 30444.48 (3847.15 to 125132.88) | 25085.73 (3136.97 to 103219.77) | 18729.95 (2323.51 to 77137.88) | 18093.61 (2226.69 to 74572.74) | 13483.32 (1650.79 to 55606.91) | 12357.59 (1502.14 to 51003.30) | 8364.16 (1014.51 to 34533.85) | 6244.87 (756.42 to 25783.64) | 3975.03 (482.20 to 16411.67) | 2848.15 (345.23 to 11759.33) | 1257.53 (152.58 to 5192.02) | 672.58 (81.65 to 2776.95) | 260.45 (31.56 to 1075.29) |
| Malaysia | Male | Number of DALYs | 106.30 (57.00 to 172.29) | 677.66 (304.02 to 1342.86) | 793.91 (367.30 to 1590.83) | 921.41 (455.51 to 1703.41) | 1060.29 (534.04 to 1907.81) | 1152.58 (597.30 to 2030.48) | 1168.18 (641.16 to 1997.59) | 1080.95 (597.26 to 1807.00) | 931.20 (523.15 to 1545.40) | 720.02 (401.20 to 1208.24) | 673.93 (378.53 to 1109.60) | 475.83 (276.45 to 778.49) | 444.49 (255.13 to 706.63) | 275.97 (158.95 to 435.08) | 201.84 (121.02 to 320.17) | 126.21 (75.05 to 195.88) | 86.76 (51.79 to 141.16) | 38.31 (22.79 to 61.90) | 15.61 (9.55 to 25.06) | 6.40 (3.88 to 10.11) |
| Malaysia | Male | Number of prevalence | 8907.90 (1637.41 to 34724.00) | 23785.95 (6163.73 to 86169.18) | 29944.40 (7446.04 to 109884.96) | 35764.80 (8966.97 to 130364.58) | 39973.13 (10307.69 to 144218.84) | 42754.11 (11239.03 to 153131.54) | 42094.52 (11311.25 to 149872.33) | 38313.24 (10415.29 to 135739.76) | 32695.14 (9114.78 to 115375.46) | 25012.98 (7091.30 to 87930.98) | 23416.74 (6752.67 to 81984.04) | 16471.76 (4812.07 to 57469.80) | 15283.57 (4523.10 to 53109.18) | 9590.50 (2857.64 to 33249.28) | 7175.90 (2141.25 to 24883.36) | 4567.70 (1363.14 to 15840.56) | 3192.74 (950.19 to 11067.78) | 1436.71 (429.60 to 4979.98) | 604.16 (180.33 to 2094.25) | 253.11 (75.43 to 877.43) |
| Maldives | Both | Number of DALYs | 4.14 (2.59 to 6.33) | 13.25 (7.06 to 24.23) | 12.59 (6.56 to 22.05) | 12.75 (6.76 to 22.24) | 12.89 (7.30 to 22.05) | 11.97 (6.67 to 20.85) | 9.92 (5.57 to 16.99) | 7.59 (4.17 to 12.95) | 6.44 (3.57 to 10.87) | 6.70 (3.87 to 11.04) | 6.87 (3.96 to 11.33) | 6.20 (3.43 to 10.33) | 4.97 (2.89 to 8.15) | 3.22 (1.92 to 5.33) | 1.86 (1.08 to 3.02) | 1.01 (0.58 to 1.64) | 0.56 (0.33 to 0.92) | 0.23 (0.14 to 0.38) | 0.04 (0.02 to 0.06) | 0.01 (0.00 to 0.01) |
| Maldives | Both | Number of prevalence | 263.85 (236.83 to 292.65) | 583.88 (509.45 to 661.96) | 653.26 (572.40 to 735.73) | 724.82 (637.69 to 808.22) | 763.36 (674.51 to 850.60) | 709.14 (628.16 to 789.90) | 577.64 (511.59 to 642.55) | 436.11 (386.64 to 484.29) | 365.74 (324.63 to 405.72) | 379.71 (337.02 to 420.32) | 378.78 (336.40 to 419.28) | 329.50 (292.59 to 364.58) | 257.91 (229.19 to 284.98) | 168.78 (150.12 to 186.48) | 98.39 (87.45 to 108.74) | 52.94 (47.02 to 58.54) | 28.03 (24.88 to 31.05) | 10.97 (9.72 to 12.17) | 1.73 (1.53 to 1.92) | 0.27 (0.24 to 0.30) |
| Maldives | Female | Number of DALYs | 2.41 (1.48 to 3.72) | 3.56 (2.08 to 5.44) | 2.99 (1.70 to 4.60) | 2.68 (1.60 to 4.14) | 2.45 (1.55 to 3.69) | 2.04 (1.28 to 3.05) | 1.51 (0.94 to 2.39) | 1.07 (0.67 to 1.69) | 0.86 (0.51 to 1.28) | 0.86 (0.51 to 1.28) | 0.80 (0.49 to 1.20) | 0.62 (0.38 to 0.94) | 0.44 (0.27 to 0.65) | 0.28 (0.17 to 0.43) | 0.16 (0.10 to 0.24) | 0.08 (0.05 to 0.11) | 0.03 (0.02 to 0.05) | 0.01 (0.01 to 0.01) | 0.00 (0.00 to 0.00) | 0.00 (0.00 to 0.00) |
| Maldives | Female | Number of prevalence | 133.77 (120.64 to 147.87) | 262.66 (235.65 to 292.02) | 295.22 (263.79 to 329.73) | 334.07 (297.61 to 374.04) | 359.08 (319.55 to 402.45) | 327.96 (291.59 to 367.84) | 259.77 (230.92 to 291.43) | 190.99 (169.69 to 214.33) | 157.26 (139.70 to 176.53) | 163.31 (145.04 to 183.35) | 155.01 (137.69 to 174.04) | 123.11 (109.31 to 138.25) | 91.08 (80.86 to 102.29) | 58.90 (52.28 to 66.15) | 33.95 (30.14 to 38.12) | 16.79 (14.91 to 18.86) | 7.50 (6.66 to 8.42) | 2.27 (2.02 to 2.55) | 0.32 (0.28 to 0.36) | 0.06 (0.05 to 0.07) |
| Maldives | Male | Number of DALYs | 1.73 (0.95 to 2.83) | 9.68 (4.39 to 20.13) | 9.61 (4.55 to 18.90) | 10.08 (4.84 to 19.26) | 10.44 (4.96 to 19.49) | 9.93 (5.00 to 18.34) | 8.41 (4.34 to 14.96) | 6.52 (3.39 to 11.60) | 5.57 (3.03 to 9.81) | 5.84 (3.14 to 10.01) | 6.07 (3.30 to 10.34) | 5.58 (2.96 to 9.55) | 4.52 (2.51 to 7.65) | 2.94 (1.68 to 4.99) | 1.69 (0.95 to 2.80) | 0.93 (0.53 to 1.56) | 0.52 (0.30 to 0.88) | 0.22 (0.13 to 0.37) | 0.03 (0.02 to 0.05) | 0.01 (0.00 to 0.01) |
| Maldives | Male | Number of prevalence | 130.08 (116.77 to 144.47) | 321.21 (271.92 to 396.28) | 358.04 (306.12 to 427.21) | 390.75 (336.84 to 456.15) | 404.28 (350.44 to 464.87) | 381.17 (332.09 to 434.12) | 317.87 (277.87 to 361.85) | 245.12 (214.45 to 278.45) | 208.48 (182.48 to 236.81) | 216.40 (189.63 to 245.65) | 223.77 (196.03 to 253.46) | 206.38 (181.05 to 233.70) | 166.83 (146.42 to 188.33) | 109.88 (96.52 to 123.72) | 64.44 (56.58 to 72.58) | 36.14 (31.74 to 40.70) | 20.53 (18.04 to 23.11) | 8.70 (7.64 to 9.79) | 1.41 (1.24 to 1.59) | 0.21 (0.19 to 0.24) |
| Mali | Both | Number of DALYs | 293.79 (183.89 to 430.98) | 4916.04 (3207.77 to 6969.71) | 4952.71 (3361.16 to 6773.61) | 4132.77 (2823.98 to 5706.80) | 3704.16 (2532.35 to 5177.57) | 3425.65 (2332.98 to 4801.72) | 3226.56 (2217.17 to 4547.64) | 3002.00 (2052.48 to 4211.40) | 2705.30 (1866.56 to 3834.35) | 2367.84 (1601.32 to 3349.50) | 2175.53 (1494.80 to 3029.57) | 2002.21 (1378.01 to 2784.98) | 1697.15 (1155.67 to 2418.64) | 1312.63 (890.18 to 1846.57) | 868.60 (601.15 to 1210.84) | 459.47 (319.36 to 632.60) | 198.74 (139.23 to 274.93) | 72.72 (50.87 to 98.94) | 18.95 (13.28 to 25.91) | 3.73 (2.65 to 5.10) |
| Mali | Both | Number of prevalence | 81677.87 (30600.58 to 154098.40) | 205363.95 (99640.69 to 354153.15) | 228424.73 (108611.24 to 398090.94) | 232612.88 (105453.57 to 413185.06) | 234273.94 (104374.84 to 419843.69) | 224662.07 (100326.02 to 403403.65) | 209226.71 (94208.61 to 375091.11) | 189121.61 (86199.91 to 338132.76) | 166624.69 (76767.83 to 297279.19) | 142451.02 (66349.80 to 253641.71) | 128543.71 (60364.62 to 228624.23) | 115406.91 (54770.23 to 204699.99) | 95619.57 (45907.99 to 166413.93) | 73746.84 (35620.11 to 126453.92) | 49771.08 (24049.74 to 85363.08) | 26920.28 (12999.37 to 46178.11) | 11901.66 (5739.15 to 20431.06) | 4503.49 (2163.94 to 7748.69) | 1225.22 (585.29 to 2115.47) | 258.37 (122.19 to 449.01) |
| Mali | Female | Number of DALYs | 169.75 (108.50 to 254.14) | 330.77 (213.30 to 481.15) | 400.03 (256.62 to 586.88) | 533.83 (348.53 to 787.97) | 726.62 (481.34 to 1078.36) | 852.86 (569.13 to 1239.89) | 892.89 (586.24 to 1321.53) | 852.18 (553.05 to 1259.90) | 795.08 (505.50 to 1163.68) | 711.52 (464.08 to 1046.81) | 679.15 (432.52 to 1008.09) | 636.22 (411.68 to 929.47) | 559.57 (358.68 to 811.10) | 439.83 (283.82 to 639.84) | 292.32 (190.75 to 431.39) | 155.84 (101.87 to 221.45) | 69.09 (45.81 to 99.32) | 27.20 (17.81 to 39.21) | 7.84 (5.25 to 11.23) | 1.83 (1.22 to 2.59) |
| Mali | Female | Number of prevalence | 40518.81 (15305.09 to 76244.53) | 84029.84 (31623.93 to 158297.84) | 95353.95 (36154.44 to 179411.11) | 105198.88 (40667.17 to 197070.56) | 116141.01 (45990.23 to 216323.85) | 116500.13 (47192.25 to 215763.35) | 108013.68 (44453.54 to 199087.85) | 94236.31 (39331.30 to 172958.62) | 81597.10 (34492.05 to 149186.32) | 68281.07 (29222.18 to 124445.34) | 61260.29 (26552.90 to 111309.39) | 54002.44 (23709.14 to 97879.95) | 44652.20 (19897.40 to 80733.51) | 34377.50 (15440.03 to 61572.94) | 23268.00 (10460.99 to 41685.47) | 12602.17 (5664.63 to 22581.67) | 5692.01 (2555.32 to 10197.55) | 2274.90 (1021.66 to 4075.88) | 669.93 (300.60 to 1200.31) | 161.26 (72.40 to 288.96) |
| Mali | Male | Number of DALYs | 124.05 (73.58 to 187.03) | 4585.27 (2981.24 to 6540.71) | 4552.68 (3054.57 to 6229.92) | 3598.94 (2428.85 to 4951.18) | 2977.54 (2003.77 to 4186.07) | 2572.79 (1738.02 to 3578.80) | 2333.67 (1593.95 to 3251.41) | 2149.81 (1471.23 to 2972.83) | 1910.22 (1296.55 to 2638.15) | 1656.32 (1135.35 to 2290.83) | 1496.38 (1035.41 to 2074.56) | 1365.99 (939.55 to 1892.15) | 1137.58 (785.51 to 1610.66) | 872.80 (607.77 to 1210.65) | 576.29 (407.26 to 793.08) | 303.63 (214.62 to 423.70) | 129.65 (92.60 to 176.43) | 45.52 (32.80 to 61.23) | 11.11 (8.00 to 15.07) | 1.90 (1.36 to 2.57) |
| Mali | Male | Number of prevalence | 41159.06 (15295.49 to 77853.87) | 121334.12 (67038.76 to 195786.90) | 133070.77 (71517.05 to 218793.93) | 127414.00 (65207.19 to 216114.50) | 118132.94 (58694.63 to 203519.84) | 108161.94 (53244.50 to 187640.30) | 101213.03 (49603.46 to 176003.25) | 94885.30 (46641.95 to 165174.14) | 85027.59 (41991.12 to 148092.88) | 74169.96 (36872.47 to 129241.81) | 67283.42 (33638.63 to 117322.95) | 61404.47 (30866.42 to 106820.04) | 50967.38 (25836.13 to 85680.42) | 39369.34 (20035.19 to 64880.98) | 26503.08 (13494.51 to 43677.61) | 14318.11 (7282.61 to 23596.43) | 6209.65 (3158.49 to 10233.51) | 2228.59 (1133.73 to 3672.80) | 555.30 (282.43 to 915.16) | 97.11 (49.43 to 160.05) |
| Marshall Islands | Both | Number of DALYs | 0.73 (0.44 to 1.08) | 2.27 (1.24 to 4.10) | 1.96 (1.05 to 3.58) | 1.48 (0.76 to 2.74) | 1.19 (0.65 to 2.17) | 1.05 (0.56 to 1.85) | 0.94 (0.50 to 1.71) | 0.81 (0.42 to 1.47) | 0.61 (0.32 to 1.11) | 0.39 (0.21 to 0.74) | 0.30 (0.16 to 0.53) | 0.24 (0.13 to 0.44) | 0.20 (0.11 to 0.37) | 0.16 (0.09 to 0.29) | 0.12 (0.07 to 0.20) | 0.07 (0.04 to 0.13) | 0.03 (0.01 to 0.04) | 0.01 (0.00 to 0.01) | 0.00 (0.00 to 0.00) | 0.00 (0.00 to 0.00) |
| Marshall Islands | Both | Number of prevalence | 16.30 (7.39 to 26.70) | 44.00 (18.56 to 73.39) | 46.24 (16.56 to 80.30) | 41.97 (12.87 to 75.03) | 38.30 (10.53 to 69.93) | 36.19 (9.35 to 66.80) | 33.90 (8.46 to 62.95) | 30.47 (7.42 to 56.85) | 23.38 (5.59 to 43.73) | 15.35 (3.61 to 28.80) | 12.01 (2.77 to 22.59) | 10.19 (2.30 to 19.25) | 8.87 (1.96 to 16.82) | 7.61 (1.63 to 14.49) | 5.57 (1.19 to 10.62) | 3.43 (0.74 to 6.53) | 1.41 (0.29 to 2.70) | 0.44 (0.09 to 0.85) | 0.09 (0.02 to 0.16) | 0.01 (0.00 to 0.02) |
| Marshall Islands | Female | Number of DALYs | 0.41 (0.24 to 0.66) | 0.75 (0.45 to 1.15) | 0.63 (0.38 to 0.99) | 0.46 (0.28 to 0.70) | 0.37 (0.23 to 0.60) | 0.32 (0.19 to 0.49) | 0.27 (0.16 to 0.45) | 0.23 (0.13 to 0.34) | 0.16 (0.09 to 0.25) | 0.10 (0.06 to 0.16) | 0.08 (0.04 to 0.12) | 0.07 (0.04 to 0.10) | 0.06 (0.03 to 0.09) | 0.05 (0.03 to 0.08) | 0.04 (0.02 to 0.06) | 0.02 (0.01 to 0.03) | 0.01 (0.01 to 0.02) | 0.00 (0.00 to 0.01) | 0.00 (0.00 to 0.00) | 0.00 (0.00 to 0.00) |
| Marshall Islands | Female | Number of prevalence | 8.49 (4.19 to 13.52) | 18.76 (7.63 to 31.80) | 20.01 (6.56 to 35.71) | 18.48 (4.98 to 34.22) | 17.45 (4.09 to 32.94) | 16.57 (3.57 to 31.64) | 15.06 (3.07 to 28.97) | 13.35 (2.61 to 25.79) | 9.73 (1.84 to 18.88) | 6.46 (1.19 to 12.58) | 5.17 (0.93 to 10.08) | 4.50 (0.79 to 8.80) | 4.00 (0.68 to 7.85) | 3.70 (0.62 to 7.26) | 2.75 (0.46 to 5.40) | 1.67 (0.28 to 3.27) | 0.84 (0.14 to 1.65) | 0.29 (0.05 to 0.56) | 0.06 (0.01 to 0.11) | 0.01 (0.00 to 0.01) |
| Marshall Islands | Male | Number of DALYs | 0.32 (0.18 to 0.51) | 1.52 (0.70 to 3.21) | 1.34 (0.58 to 2.83) | 1.02 (0.43 to 2.15) | 0.82 (0.36 to 1.67) | 0.73 (0.34 to 1.45) | 0.67 (0.31 to 1.38) | 0.58 (0.25 to 1.19) | 0.45 (0.21 to 0.91) | 0.29 (0.13 to 0.61) | 0.22 (0.10 to 0.43) | 0.18 (0.08 to 0.37) | 0.14 (0.07 to 0.30) | 0.11 (0.05 to 0.23) | 0.08 (0.04 to 0.16) | 0.05 (0.02 to 0.10) | 0.02 (0.01 to 0.03) | 0.00 (0.00 to 0.01) | 0.00 (0.00 to 0.00) | 0.00 (0.00 to 0.00) |
| Marshall Islands | Male | Number of prevalence | 7.81 (3.14 to 13.24) | 25.24 (10.16 to 43.97) | 26.23 (9.25 to 46.09) | 23.48 (7.41 to 41.99) | 20.86 (6.05 to 37.61) | 19.62 (5.46 to 35.69) | 18.84 (5.12 to 34.42) | 17.13 (4.58 to 31.41) | 13.65 (3.59 to 25.12) | 8.89 (2.31 to 16.40) | 6.84 (1.75 to 12.64) | 5.69 (1.44 to 10.55) | 4.87 (1.22 to 9.04) | 3.91 (0.97 to 7.27) | 2.82 (0.70 to 5.24) | 1.76 (0.44 to 3.27) | 0.57 (0.14 to 1.06) | 0.15 (0.04 to 0.29) | 0.03 (0.01 to 0.05) | 0.00 (0.00 to 0.01) |
| Micronesia (Federated States of) | Both | Number of DALYs | 1.99 (1.23 to 2.99) | 17.17 (9.64 to 28.85) | 26.52 (16.66 to 40.29) | 32.27 (20.93 to 46.77) | 28.99 (19.41 to 40.98) | 29.51 (19.76 to 41.72) | 29.78 (19.87 to 41.23) | 26.28 (17.66 to 36.01) | 20.11 (13.52 to 27.67) | 14.13 (9.48 to 19.40) | 10.01 (6.77 to 13.85) | 9.36 (6.45 to 12.95) | 9.01 (6.18 to 12.40) | 7.28 (5.03 to 9.95) | 4.74 (3.29 to 6.38) | 2.61 (1.82 to 3.50) | 1.26 (0.87 to 1.69) | 0.42 (0.29 to 0.55) | 0.09 (0.06 to 0.12) | 0.01 (0.01 to 0.02) |
| Micronesia (Federated States of) | Both | Number of prevalence | 336.77 (82.17 to 885.76) | 924.80 (290.22 to 2274.65) | 1258.20 (421.22 to 3041.16) | 1392.78 (491.37 to 3318.60) | 1237.04 (438.51 to 2940.14) | 1255.15 (444.98 to 2977.82) | 1273.46 (449.86 to 3020.16) | 1127.48 (397.85 to 2676.23) | 865.29 (306.38 to 2053.17) | 642.73 (223.15 to 1536.38) | 485.99 (165.05 to 1171.17) | 461.57 (157.04 to 1113.32) | 452.72 (153.96 to 1093.64) | 378.66 (128.22 to 917.10) | 263.63 (87.63 to 642.10) | 159.90 (51.61 to 392.83) | 82.93 (26.26 to 204.83) | 29.36 (9.14 to 72.84) | 6.71 (2.06 to 16.71) | 1.15 (0.34 to 2.90) |
| Micronesia (Federated States of) | Female | Number of DALYs | 1.12 (0.67 to 1.72) | 2.13 (1.26 to 3.26) | 2.33 (1.44 to 3.49) | 2.33 (1.50 to 3.50) | 2.08 (1.36 to 3.14) | 2.05 (1.27 to 3.06) | 2.04 (1.32 to 3.00) | 1.77 (1.20 to 2.54) | 1.31 (0.82 to 1.93) | 1.04 (0.65 to 1.50) | 0.86 (0.56 to 1.29) | 0.80 (0.53 to 1.14) | 0.77 (0.51 to 1.11) | 0.65 (0.42 to 0.94) | 0.48 (0.32 to 0.70) | 0.32 (0.22 to 0.44) | 0.17 (0.11 to 0.24) | 0.06 (0.04 to 0.09) | 0.01 (0.01 to 0.02) | 0.00 (0.00 to 0.00) |
| Micronesia (Federated States of) | Female | Number of prevalence | 163.08 (41.06 to 426.26) | 393.98 (94.54 to 1039.57) | 521.20 (121.25 to 1383.12) | 576.19 (132.13 to 1532.94) | 526.87 (120.33 to 1402.56) | 525.54 (119.96 to 1398.94) | 519.75 (118.77 to 1383.03) | 446.90 (102.30 to 1188.84) | 327.86 (75.14 to 871.88) | 259.10 (59.49 to 688.74) | 210.65 (48.44 to 559.74) | 195.81 (45.12 to 520.07) | 187.44 (43.29 to 497.58) | 159.65 (36.94 to 423.71) | 120.04 (27.76 to 318.57) | 81.39 (18.84 to 215.99) | 44.90 (10.38 to 119.16) | 16.71 (3.86 to 44.34) | 3.98 (0.92 to 10.56) | 0.75 (0.17 to 2.00) |
| Micronesia (Federated States of) | Male | Number of DALYs | 0.87 (0.52 to 1.38) | 15.04 (8.04 to 25.87) | 24.19 (14.78 to 37.46) | 29.94 (19.11 to 43.43) | 26.91 (17.85 to 38.53) | 27.46 (18.53 to 38.77) | 27.74 (18.55 to 38.57) | 24.51 (16.27 to 33.69) | 18.81 (12.53 to 25.91) | 13.09 (8.77 to 18.23) | 9.15 (6.13 to 12.75) | 8.56 (5.84 to 11.80) | 8.24 (5.61 to 11.39) | 6.62 (4.55 to 9.03) | 4.26 (2.94 to 5.77) | 2.29 (1.58 to 3.05) | 1.08 (0.76 to 1.46) | 0.35 (0.25 to 0.48) | 0.07 (0.05 to 0.10) | 0.01 (0.01 to 0.01) |
| Micronesia (Federated States of) | Male | Number of prevalence | 173.69 (41.32 to 459.50) | 530.81 (198.00 to 1230.17) | 737.01 (292.93 to 1665.42) | 816.59 (346.21 to 1795.43) | 710.17 (306.16 to 1544.88) | 729.60 (316.76 to 1584.82) | 753.71 (326.35 to 1641.49) | 680.58 (293.54 to 1488.55) | 537.42 (231.40 to 1181.29) | 383.63 (163.77 to 847.64) | 275.35 (116.80 to 611.43) | 265.76 (111.97 to 593.25) | 265.29 (110.91 to 596.06) | 219.01 (91.24 to 493.39) | 143.59 (59.83 to 323.52) | 78.51 (32.76 to 176.84) | 38.03 (15.86 to 85.67) | 12.65 (5.27 to 28.49) | 2.73 (1.14 to 6.15) | 0.40 (0.17 to 0.90) |
| Mozambique | Both | Number of DALYs | 515.97 (333.63 to 737.77) | 9184.20 (6140.90 to 12752.67) | 8716.28 (5924.72 to 12069.03) | 7927.68 (5413.60 to 11040.78) | 7234.26 (4876.55 to 10136.89) | 7629.38 (5158.30 to 10978.94) | 8122.46 (5459.85 to 11508.53) | 8128.19 (5444.95 to 11559.04) | 7553.54 (5097.74 to 10746.76) | 6930.08 (4663.03 to 9922.37) | 6366.90 (4275.09 to 9065.92) | 5359.54 (3643.03 to 7562.57) | 4643.56 (3148.85 to 6621.24) | 3640.88 (2481.29 to 5155.73) | 2345.98 (1611.50 to 3303.92) | 1303.54 (895.07 to 1842.39) | 632.88 (433.94 to 880.63) | 225.84 (156.22 to 311.51) | 52.11 (36.15 to 71.60) | 8.98 (6.25 to 12.38) |
| Mozambique | Both | Number of prevalence | 152136.68 (85901.72 to 231510.53) | 408228.75 (258345.58 to 588023.21) | 479533.48 (298038.25 to 698934.34) | 502591.56 (307347.44 to 738065.42) | 466451.70 (285174.81 to 685576.25) | 458248.14 (282612.37 to 671709.52) | 452732.09 (282381.22 to 661853.07) | 428360.98 (269341.70 to 624900.02) | 375532.83 (238329.09 to 546590.78) | 328962.06 (210538.25 to 469290.24) | 290319.36 (187563.99 to 397211.61) | 234826.85 (153160.82 to 306950.11) | 194649.83 (128625.61 to 242285.58) | 151561.43 (100840.21 to 184919.64) | 100055.06 (66493.16 to 122242.42) | 56945.45 (37800.11 to 69627.31) | 28408.91 (18835.98 to 34751.81) | 10425.07 (6909.39 to 12767.24) | 2502.30 (1652.89 to 3075.36) | 459.10 (301.40 to 567.88) |
| Mozambique | Female | Number of DALYs | 300.45 (187.95 to 445.75) | 702.37 (457.67 to 1029.28) | 1062.61 (697.67 to 1603.37) | 1682.70 (1126.12 to 2473.55) | 2280.47 (1459.63 to 3255.24) | 2785.38 (1797.34 to 4137.69) | 3159.86 (2053.05 to 4622.50) | 3349.59 (2145.67 to 4948.35) | 3101.17 (2013.99 to 4486.63) | 2805.64 (1823.16 to 4093.39) | 2609.27 (1689.04 to 3807.88) | 2181.33 (1405.76 to 3190.48) | 1893.92 (1241.78 to 2752.58) | 1525.00 (1003.09 to 2183.95) | 1036.09 (683.41 to 1485.57) | 593.03 (390.74 to 845.14) | 292.78 (193.49 to 415.32) | 108.83 (73.69 to 154.94) | 28.22 (19.01 to 39.48) | 5.91 (4.02 to 8.21) |
| Mozambique | Female | Number of prevalence | 76135.29 (43258.04 to 115558.47) | 172222.85 (97812.88 to 261362.67) | 214769.70 (122584.11 to 325171.81) | 246983.53 (142556.62 to 372501.32) | 247279.96 (144967.94 to 370920.72) | 243980.65 (145537.53 to 364050.07) | 239424.39 (144772.00 to 355843.05) | 228630.96 (139785.33 to 338380.99) | 194770.27 (120439.21 to 287318.72) | 165544.00 (103290.81 to 243517.66) | 145221.80 (91498.87 to 205450.44) | 115321.21 (73321.23 to 155728.61) | 94886.03 (61156.58 to 121837.67) | 75312.25 (48868.40 to 94672.61) | 51964.93 (33718.47 to 65322.74) | 30321.80 (19661.44 to 38116.00) | 15347.21 (9949.08 to 19292.14) | 5829.03 (3781.31 to 7327.45) | 1546.82 (1003.61 to 1944.45) | 333.53 (216.35 to 419.26) |
| Mozambique | Male | Number of DALYs | 215.52 (133.55 to 320.99) | 8481.83 (5697.06 to 11826.42) | 7653.67 (5197.36 to 10695.00) | 6244.98 (4170.61 to 8653.41) | 4953.78 (3415.92 to 6906.06) | 4844.00 (3332.44 to 6775.01) | 4962.61 (3418.86 to 6952.94) | 4778.60 (3259.49 to 6860.11) | 4452.37 (3073.59 to 6366.51) | 4124.43 (2820.05 to 5965.97) | 3757.63 (2569.24 to 5408.93) | 3178.22 (2177.45 to 4531.16) | 2749.64 (1891.55 to 3942.80) | 2115.88 (1454.44 to 3023.96) | 1309.89 (900.90 to 1856.67) | 710.51 (489.63 to 1006.22) | 340.10 (239.21 to 474.51) | 117.00 (82.33 to 162.84) | 23.90 (16.90 to 32.82) | 3.07 (2.15 to 4.21) |
| Mozambique | Male | Number of prevalence | 76001.39 (42643.68 to 115952.06) | 236005.91 (161351.13 to 325186.52) | 264763.78 (175252.78 to 373722.60) | 255608.03 (164988.52 to 365585.94) | 219171.74 (140306.73 to 314659.10) | 214267.49 (136804.06 to 307668.70) | 213307.70 (137043.99 to 306006.19) | 199730.02 (128937.17 to 286249.07) | 180762.56 (117451.45 to 258894.42) | 163418.05 (107010.01 to 225772.58) | 145097.56 (95747.39 to 191761.17) | 119505.64 (79483.49 to 151221.50) | 99763.80 (67455.17 to 120447.90) | 76249.18 (52049.95 to 90247.03) | 48090.13 (32837.75 to 56919.68) | 26623.66 (18158.54 to 31511.32) | 13061.70 (8916.14 to 15459.67) | 4596.04 (3137.34 to 5439.78) | 955.48 (652.33 to 1130.91) | 125.57 (85.65 to 148.62) |
| Myanmar | Both | Number of DALYs | 643.43 (418.44 to 964.95) | 6216.31 (3700.30 to 10262.13) | 10241.92 (6560.12 to 15023.07) | 14000.61 (9199.50 to 19912.61) | 15474.39 (10374.07 to 21859.62) | 14862.15 (10018.40 to 20580.53) | 13078.80 (8759.19 to 17936.43) | 11046.74 (7466.38 to 15245.53) | 8187.78 (5603.87 to 11278.08) | 6687.74 (4534.67 to 9273.63) | 6037.42 (4116.49 to 8255.71) | 5433.95 (3676.26 to 7468.79) | 4614.34 (3235.28 to 6292.84) | 3196.96 (2247.93 to 4311.85) | 2029.44 (1415.69 to 2756.48) | 1116.84 (776.96 to 1487.40) | 535.77 (380.67 to 722.52) | 193.52 (138.08 to 258.25) | 40.52 (29.03 to 53.96) | 6.00 (4.33 to 7.92) |
| Myanmar | Both | Number of prevalence | 119544.58 (15432.01 to 373293.92) | 331689.60 (70673.35 to 952776.80) | 474164.16 (113831.55 to 1338431.66) | 597936.51 (154532.58 to 1670297.17) | 667913.12 (174438.75 to 1869910.59) | 663214.94 (170035.26 to 1866806.84) | 602298.28 (150830.18 to 1703145.06) | 525621.22 (128607.15 to 1492737.30) | 401129.04 (96655.22 to 1142723.78) | 343729.96 (80651.19 to 984397.85) | 320676.31 (73709.69 to 921145.29) | 297700.72 (67188.36 to 857626.95) | 260832.65 (58212.76 to 752932.66) | 191024.52 (41611.55 to 553702.13) | 127632.92 (27258.50 to 371340.76) | 72297.52 (15316.73 to 210560.23) | 36059.70 (7542.73 to 105251.58) | 13841.20 (2824.62 to 40556.67) | 3321.06 (633.51 to 9834.60) | 643.11 (107.41 to 1940.60) |
| Myanmar | Female | Number of DALYs | 375.81 (231.27 to 590.85) | 724.68 (446.51 to 1106.72) | 846.42 (522.62 to 1232.01) | 1000.86 (652.11 to 1525.41) | 1128.83 (737.47 to 1686.59) | 1142.24 (727.50 to 1714.02) | 1067.52 (688.01 to 1522.83) | 944.41 (625.98 to 1379.18) | 736.74 (483.71 to 1076.26) | 656.22 (438.20 to 956.40) | 627.00 (406.14 to 891.08) | 596.06 (393.05 to 861.85) | 529.32 (346.48 to 779.50) | 398.68 (267.06 to 575.38) | 276.38 (185.65 to 404.42) | 154.61 (103.83 to 225.37) | 77.72 (53.32 to 111.20) | 30.57 (20.95 to 43.01) | 8.10 (5.54 to 11.48) | 1.84 (1.28 to 2.55) |
| Myanmar | Female | Number of prevalence | 59687.61 (8134.30 to 185114.78) | 145191.53 (18000.22 to 454450.56) | 201429.47 (23681.77 to 633837.12) | 253547.68 (29122.52 to 799384.02) | 287577.59 (32915.41 to 906651.66) | 287494.19 (33055.08 to 905844.23) | 261239.87 (30184.43 to 822603.52) | 229014.44 (26621.37 to 720617.85) | 174616.15 (20422.08 to 549078.23) | 154263.67 (18171.16 to 484770.30) | 145211.12 (17201.29 to 456037.73) | 135701.90 (16186.32 to 425873.29) | 118247.09 (14243.44 to 370831.10) | 90153.84 (10893.96 to 282636.60) | 63227.54 (7638.78 to 198229.08) | 36334.44 (4383.62 to 113914.24) | 18623.40 (2248.28 to 58386.22) | 7505.50 (906.87 to 23532.20) | 2033.51 (245.64 to 6375.29) | 474.50 (57.32 to 1487.59) |
| Myanmar | Male | Number of DALYs | 267.62 (156.68 to 425.47) | 5491.63 (3134.75 to 9263.14) | 9395.51 (5913.98 to 13886.20) | 12999.75 (8529.01 to 18660.51) | 14345.56 (9518.15 to 20474.50) | 13719.91 (9164.93 to 19004.67) | 12011.27 (7999.53 to 16696.28) | 10102.33 (6825.29 to 14091.47) | 7451.04 (5070.73 to 10283.15) | 6031.52 (4045.96 to 8483.25) | 5410.42 (3673.01 to 7485.88) | 4837.89 (3266.43 to 6628.21) | 4085.02 (2844.05 to 5555.53) | 2798.28 (1972.74 to 3843.33) | 1753.06 (1223.03 to 2366.17) | 962.24 (668.75 to 1278.47) | 458.05 (321.67 to 623.80) | 162.94 (115.68 to 222.16) | 32.42 (23.01 to 43.39) | 4.16 (2.97 to 5.50) |
| Myanmar | Male | Number of prevalence | 59856.97 (7282.99 to 188179.14) | 186498.07 (52808.05 to 497747.35) | 272734.69 (89720.70 to 703822.28) | 344388.83 (124216.24 to 870641.52) | 380335.54 (139458.35 to 962757.32) | 375720.75 (135557.71 to 960179.66) | 341058.41 (120468.68 to 879889.61) | 296606.78 (102187.57 to 771851.94) | 226512.89 (76079.39 to 593645.54) | 189466.29 (62520.63 to 499627.55) | 175465.19 (56984.25 to 465107.56) | 161998.82 (51653.59 to 431753.67) | 142585.56 (44525.81 to 382101.56) | 100870.68 (31315.83 to 271065.53) | 64405.38 (19963.19 to 173111.69) | 35963.08 (11167.63 to 96645.98) | 17436.30 (5419.59 to 46865.36) | 6335.70 (1965.76 to 17024.47) | 1287.55 (398.99 to 3459.30) | 168.60 (52.27 to 453.01) |
| Nepal | Both | Number of DALYs | 384.02 (250.89 to 559.09) | 2820.24 (1640.28 to 4732.08) | 4077.86 (2464.64 to 6161.56) | 5033.57 (3303.58 to 7401.51) | 5401.88 (3601.50 to 7830.75) | 5298.81 (3513.69 to 7440.76) | 4867.18 (3285.75 to 6801.45) | 4392.65 (2924.93 to 6106.56) | 3728.52 (2526.26 to 5187.41) | 3230.63 (2219.09 to 4471.41) | 2746.95 (1850.89 to 3756.04) | 2239.41 (1538.05 to 3041.83) | 1770.00 (1221.44 to 2397.15) | 1316.12 (912.38 to 1757.93) | 787.61 (548.74 to 1050.13) | 416.73 (290.88 to 555.73) | 220.67 (153.24 to 293.15) | 91.60 (64.96 to 122.46) | 25.81 (18.18 to 34.06) | 3.69 (2.63 to 4.84) |
| Nepal | Both | Number of prevalence | 64618.58 (55417.35 to 75008.11) | 156186.09 (134352.31 to 179087.91) | 199159.86 (172564.49 to 228070.99) | 224556.78 (196375.01 to 256987.85) | 238939.92 (209441.61 to 272511.66) | 235516.87 (207288.30 to 269323.38) | 216244.60 (190599.29 to 247226.66) | 196951.17 (173178.94 to 225221.13) | 170237.01 (149486.40 to 194558.00) | 149494.49 (130987.93 to 170634.20) | 128141.92 (112280.55 to 146136.00) | 107499.27 (94083.72 to 122679.65) | 88787.47 (77516.04 to 101366.40) | 67762.79 (59114.71 to 77375.85) | 41525.90 (36208.00 to 47420.38) | 22833.98 (19904.28 to 26086.49) | 12327.74 (10742.81 to 14084.93) | 5241.01 (4566.31 to 5986.54) | 1599.28 (1391.42 to 1829.02) | 284.38 (246.17 to 326.33) |
| Nepal | Female | Number of DALYs | 220.90 (138.13 to 331.00) | 371.19 (223.04 to 584.22) | 377.38 (227.50 to 570.73) | 384.84 (245.14 to 569.53) | 408.62 (265.51 to 605.72) | 400.08 (254.05 to 588.62) | 363.09 (233.07 to 528.68) | 324.20 (211.05 to 455.96) | 279.97 (185.65 to 402.28) | 241.28 (159.93 to 356.97) | 204.03 (134.58 to 298.25) | 171.80 (112.91 to 247.32) | 145.79 (98.76 to 205.58) | 109.77 (73.71 to 159.31) | 67.39 (44.65 to 96.68) | 37.56 (25.66 to 54.02) | 19.83 (13.65 to 27.89) | 8.41 (5.68 to 11.58) | 2.76 (1.84 to 3.83) | 0.60 (0.41 to 0.86) |
| Nepal | Female | Number of prevalence | 31853.88 (27391.28 to 36934.95) | 68128.13 (58381.19 to 79197.59) | 84097.85 (71921.93 to 97922.60) | 95904.26 (81942.32 to 111745.03) | 106120.96 (90658.91 to 123704.89) | 104726.31 (89452.04 to 122064.84) | 93778.02 (80100.23 to 109320.50) | 83825.87 (71598.21 to 97724.97) | 71958.59 (61470.64 to 83882.98) | 62021.33 (52986.11 to 72292.00) | 51784.74 (44246.51 to 60352.89) | 43298.82 (37002.47 to 50451.23) | 36546.49 (31230.69 to 42575.48) | 27917.40 (23858.77 to 32511.29) | 17256.02 (14746.90 to 20099.97) | 9846.72 (8415.40 to 11469.08) | 5317.03 (4544.20 to 6193.63) | 2290.17 (1957.47 to 2667.27) | 765.78 (654.48 to 891.82) | 170.66 (145.87 to 198.80) |
| Nepal | Male | Number of DALYs | 163.12 (95.46 to 254.49) | 2449.05 (1345.11 to 4241.77) | 3700.49 (2210.72 to 5725.98) | 4648.73 (3004.74 to 6870.43) | 4993.27 (3293.77 to 7246.54) | 4898.73 (3230.62 to 6911.26) | 4504.09 (3000.49 to 6304.66) | 4068.45 (2692.54 to 5640.70) | 3448.54 (2324.64 to 4858.45) | 2989.35 (2038.99 to 4107.95) | 2542.92 (1711.34 to 3455.67) | 2067.61 (1431.19 to 2818.10) | 1624.20 (1115.34 to 2228.25) | 1206.34 (835.68 to 1615.46) | 720.22 (501.33 to 957.38) | 379.17 (265.34 to 508.94) | 200.84 (139.98 to 268.17) | 83.18 (59.03 to 111.54) | 23.05 (16.20 to 30.37) | 3.08 (2.19 to 4.04) |
| Nepal | Male | Number of prevalence | 32764.70 (27988.27 to 38073.16) | 88057.96 (74183.42 to 102272.53) | 115062.01 (98521.39 to 132209.20) | 128652.52 (112294.73 to 145645.12) | 132818.96 (117516.16 to 150356.40) | 130790.57 (116017.50 to 148443.74) | 122466.58 (108840.45 to 138595.84) | 113125.30 (100726.59 to 127586.36) | 98278.42 (87510.80 to 110870.06) | 87473.16 (77851.41 to 98752.63) | 76357.19 (67918.49 to 86274.42) | 64200.45 (57049.85 to 72454.24) | 52240.98 (46309.14 to 58971.83) | 39845.39 (35352.92 to 44950.84) | 24269.88 (21507.70 to 27403.34) | 12987.26 (11519.36 to 14645.66) | 7010.70 (6223.97 to 7907.13) | 2950.84 (2618.23 to 3331.02) | 833.51 (738.91 to 940.38) | 113.72 (100.83 to 128.32) |
| Niger | Both | Number of DALYs | 221.52 (143.99 to 332.93) | 2750.80 (1777.11 to 4149.99) | 3373.49 (2260.56 to 4823.76) | 3327.21 (2190.29 to 4622.49) | 2851.02 (1936.36 to 3962.54) | 2491.33 (1713.54 to 3414.83) | 2294.54 (1547.71 to 3147.91) | 1795.34 (1215.40 to 2508.21) | 1639.75 (1114.01 to 2231.23) | 1363.08 (941.35 to 1860.44) | 1016.90 (705.83 to 1411.76) | 759.30 (524.06 to 1036.79) | 707.15 (489.22 to 971.83) | 507.04 (347.86 to 695.09) | 296.98 (208.63 to 400.67) | 166.73 (117.17 to 227.38) | 72.38 (51.07 to 97.06) | 21.16 (14.90 to 28.91) | 4.34 (3.05 to 5.87) | 0.70 (0.50 to 0.93) |
| Niger | Both | Number of prevalence | 51019.92 (13558.24 to 108899.47) | 129397.07 (47495.90 to 256980.18) | 144436.12 (56506.64 to 281865.53) | 150459.94 (56666.27 to 295074.25) | 145614.96 (52592.26 to 289632.15) | 137461.68 (48618.88 to 275430.16) | 130013.48 (45872.94 to 261151.12) | 102754.54 (36303.06 to 206536.28) | 96560.29 (33958.24 to 194421.43) | 80237.50 (28316.30 to 161469.63) | 60379.09 (21346.68 to 121545.53) | 45394.24 (16076.25 to 91345.10) | 42738.99 (15167.06 to 85989.90) | 31543.59 (11165.13 to 63510.83) | 18942.22 (6694.51 to 38167.34) | 11045.10 (3882.35 to 22279.36) | 5177.52 (1788.33 to 10488.79) | 1729.10 (578.54 to 3527.52) | 424.45 (136.37 to 873.64) | 76.75 (24.12 to 158.74) |
| Niger | Female | Number of DALYs | 127.47 (78.10 to 192.64) | 236.50 (144.88 to 363.11) | 242.35 (155.99 to 360.39) | 268.81 (171.43 to 393.82) | 290.21 (191.20 to 418.80) | 298.19 (195.98 to 423.52) | 293.63 (195.15 to 432.46) | 234.74 (155.41 to 345.10) | 231.26 (152.45 to 339.65) | 192.90 (126.71 to 280.58) | 147.30 (95.43 to 214.91) | 112.06 (75.06 to 166.18) | 107.69 (71.36 to 157.16) | 81.55 (54.61 to 118.01) | 48.82 (32.70 to 68.97) | 29.15 (19.02 to 40.94) | 15.13 (10.20 to 21.45) | 5.93 (4.06 to 8.50) | 1.70 (1.15 to 2.41) | 0.32 (0.22 to 0.45) |
| Niger | Female | Number of prevalence | 25162.53 (6838.10 to 53510.96) | 55109.55 (14637.11 to 117710.93) | 61585.32 (16219.44 to 131774.30) | 66672.43 (17633.24 to 142582.29) | 67831.38 (18129.61 to 144807.36) | 65184.91 (17613.35 to 138895.74) | 60325.88 (16460.62 to 128341.59) | 46160.34 (12696.31 to 98051.05) | 43607.27 (12083.88 to 92505.86) | 34879.14 (9738.77 to 73894.11) | 25738.46 (7238.05 to 54451.39) | 18899.48 (5351.35 to 39927.08) | 17437.49 (4982.16 to 36773.90) | 13089.00 (3756.49 to 27574.46) | 7956.12 (2282.33 to 16762.05) | 4824.91 (1384.53 to 10166.72) | 2548.53 (731.34 to 5370.01) | 1013.63 (290.96 to 2135.80) | 297.75 (85.44 to 627.39) | 58.54 (16.80 to 123.34) |
| Niger | Male | Number of DALYs | 94.06 (56.56 to 144.49) | 2514.30 (1563.55 to 3790.15) | 3131.15 (2088.36 to 4474.29) | 3058.40 (2015.30 to 4302.13) | 2560.81 (1738.01 to 3547.87) | 2193.14 (1498.90 to 3017.25) | 2000.91 (1340.70 to 2738.55) | 1560.60 (1048.63 to 2207.19) | 1408.49 (947.12 to 1931.99) | 1170.18 (798.49 to 1599.50) | 869.61 (607.14 to 1215.31) | 647.24 (442.69 to 883.00) | 599.47 (409.73 to 827.52) | 425.49 (289.50 to 579.69) | 248.16 (173.29 to 333.70) | 137.58 (95.50 to 189.02) | 57.25 (40.28 to 76.94) | 15.23 (10.71 to 20.75) | 2.64 (1.87 to 3.55) | 0.37 (0.26 to 0.50) |
| Niger | Male | Number of prevalence | 25857.39 (6720.14 to 55399.68) | 74287.52 (32604.48 to 139080.01) | 82850.80 (39438.66 to 150091.23) | 83787.50 (39339.41 to 152491.96) | 77783.58 (34451.84 to 144976.90) | 72276.77 (30924.99 to 136804.70) | 69687.60 (29353.03 to 133061.48) | 56594.20 (23562.09 to 108661.32) | 52953.02 (21834.04 to 102090.99) | 45358.36 (18546.62 to 87713.47) | 34640.63 (14056.71 to 67193.35) | 26494.76 (10673.82 to 51490.33) | 25301.50 (10131.17 to 49279.34) | 18454.58 (7365.98 to 35977.85) | 10986.10 (4385.37 to 21432.39) | 6220.19 (2482.40 to 12129.92) | 2628.99 (1050.25 to 5127.52) | 715.47 (285.65 to 1395.22) | 126.69 (50.63 to 247.05) | 18.21 (7.27 to 35.52) |
| Nigeria | Both | Number of DALYs | 1887.93 (1268.75 to 2664.28) | 17650.86 (10926.44 to 26972.78) | 25373.77 (16850.20 to 36499.18) | 30549.94 (20465.19 to 42400.75) | 30032.96 (20050.85 to 41366.87) | 27206.35 (18386.90 to 37413.74) | 24390.74 (16720.63 to 33316.75) | 21363.89 (14694.20 to 29384.26) | 18480.36 (12788.06 to 25327.58) | 16255.71 (11225.89 to 22234.27) | 13703.50 (9507.31 to 18663.85) | 11490.07 (7991.64 to 15629.83) | 9222.31 (6414.76 to 12502.70) | 6239.02 (4358.33 to 8421.22) | 4224.22 (2953.16 to 5662.15) | 2451.63 (1726.72 to 3318.14) | 1203.64 (851.17 to 1610.02) | 450.71 (322.44 to 591.46) | 127.89 (91.92 to 169.05) | 26.03 (18.97 to 34.30) |
| Nigeria | Both | Number of prevalence | 386154.08 (138242.68 to 749870.00) | 916340.36 (398045.28 to 1694273.61) | 1177768.51 (539930.67 to 2152062.27) | 1384472.86 (639246.39 to 2504157.46) | 1421277.14 (638910.74 to 2580083.88) | 1332541.33 (591298.16 to 2429946.55) | 1203122.21 (534006.76 to 2209576.50) | 1044306.66 (462751.31 to 1929682.60) | 886076.57 (395075.58 to 1630903.10) | 802221.62 (354129.84 to 1458114.67) | 680992.70 (305447.23 to 1219850.04) | 578765.43 (261781.31 to 1034277.30) | 484899.46 (214965.20 to 862058.37) | 377717.55 (161788.87 to 683260.21) | 272276.10 (115028.46 to 503598.79) | 168034.43 (69221.49 to 314256.47) | 87114.88 (35713.93 to 164376.95) | 34499.42 (14077.92 to 65630.39) | 10376.69 (4141.57 to 19846.49) | 2424.13 (929.54 to 4751.97) |
| Nigeria | Female | Number of DALYs | 1125.79 (761.93 to 1610.57) | 1931.29 (1297.57 to 2732.98) | 2163.35 (1487.04 to 3114.22) | 2472.61 (1681.04 to 3553.29) | 2631.63 (1792.38 to 3816.69) | 2516.21 (1706.68 to 3589.94) | 2239.34 (1500.33 to 3231.71) | 1852.69 (1256.65 to 2670.35) | 1459.79 (989.02 to 2134.40) | 1363.98 (921.25 to 1951.37) | 1137.54 (776.25 to 1642.72) | 961.13 (651.79 to 1391.60) | 856.93 (583.49 to 1227.96) | 848.00 (576.12 to 1206.97) | 648.49 (442.34 to 925.87) | 418.51 (283.50 to 590.11) | 223.36 (154.77 to 314.90) | 90.93 (62.67 to 127.23) | 27.77 (19.14 to 38.58) | 6.99 (4.91 to 9.70) |
| Nigeria | Female | Number of prevalence | 198483.56 (71872.20 to 383814.74) | 417394.30 (146948.72 to 814511.47) | 533607.76 (185238.42 to 1052422.89) | 627812.10 (219464.09 to 1234968.75) | 647901.54 (228439.58 to 1271154.79) | 594064.55 (209500.48 to 1176907.23) | 509776.09 (179532.65 to 1009288.30) | 410078.12 (143280.16 to 810239.48) | 314815.12 (111870.66 to 615705.18) | 287140.29 (103653.70 to 553886.08) | 235701.80 (86787.63 to 453202.64) | 196891.09 (72478.88 to 375366.56) | 173331.19 (61199.79 to 329590.16) | 172512.66 (60742.78 to 340592.29) | 134163.08 (46882.47 to 268883.00) | 87819.74 (30641.11 to 176823.07) | 47836.22 (16513.58 to 96109.42) | 19788.65 (6876.34 to 39496.42) | 6198.57 (2163.36 to 12304.17) | 1610.21 (551.98 to 3229.52) |
| Nigeria | Male | Number of DALYs | 762.14 (502.88 to 1095.75) | 15719.56 (9505.91 to 24664.96) | 23210.42 (15343.69 to 33751.08) | 28077.33 (18748.66 to 39214.84) | 27401.32 (18043.67 to 38031.07) | 24690.14 (16733.87 to 34009.69) | 22151.40 (15186.57 to 30825.40) | 19511.21 (13443.75 to 27017.17) | 17020.57 (11744.44 to 23241.27) | 14891.73 (10266.19 to 20335.89) | 12565.96 (8701.13 to 17207.68) | 10528.94 (7321.33 to 14332.98) | 8365.38 (5795.63 to 11384.35) | 5391.03 (3746.43 to 7299.38) | 3575.74 (2494.47 to 4764.40) | 2033.13 (1430.62 to 2705.37) | 980.27 (689.19 to 1297.38) | 359.78 (255.93 to 475.82) | 100.12 (72.02 to 132.26) | 19.04 (13.93 to 25.02) |
| Nigeria | Male | Number of prevalence | 187670.53 (66374.76 to 366055.27) | 498946.06 (244002.61 to 875557.39) | 644160.75 (340596.30 to 1099639.38) | 756660.76 (414947.38 to 1271985.95) | 773375.59 (414867.02 to 1309153.82) | 738476.78 (383996.72 to 1262548.90) | 693346.12 (355488.06 to 1199330.79) | 634228.55 (319471.15 to 1121670.01) | 571261.45 (281692.20 to 1012179.49) | 515081.33 (247204.89 to 916813.65) | 445290.89 (217636.89 to 772035.12) | 381874.34 (189123.91 to 658083.13) | 311568.27 (153128.21 to 526516.11) | 205204.89 (100577.61 to 345801.67) | 138113.02 (67801.10 to 234092.55) | 80214.69 (39393.66 to 137236.27) | 39278.66 (19124.84 to 68267.52) | 14710.77 (7027.16 to 26139.95) | 4178.12 (1940.07 to 7526.86) | 813.92 (371.82 to 1507.23) |
| Niue | Both | Number of DALYs | 0.03 (0.02 to 0.04) | 0.13 (0.07 to 0.24) | 0.15 (0.08 to 0.26) | 0.17 (0.09 to 0.30) | 0.17 (0.10 to 0.30) | 0.17 (0.10 to 0.28) | 0.17 (0.10 to 0.27) | 0.17 (0.10 to 0.27) | 0.16 (0.10 to 0.25) | 0.16 (0.10 to 0.25) | 0.16 (0.10 to 0.25) | 0.14 (0.09 to 0.21) | 0.13 (0.08 to 0.19) | 0.10 (0.06 to 0.15) | 0.07 (0.05 to 0.11) | 0.05 (0.03 to 0.07) | 0.03 (0.02 to 0.05) | 0.02 (0.01 to 0.03) | 0.01 (0.00 to 0.01) | 0.00 (0.00 to 0.00) |
| Niue | Both | Number of prevalence | 2.31 (0.66 to 6.42) | 6.84 (2.19 to 18.21) | 8.08 (2.51 to 21.76) | 9.40 (2.92 to 25.34) | 9.42 (2.92 to 25.38) | 9.15 (2.83 to 24.65) | 9.16 (2.83 to 24.66) | 9.05 (2.82 to 24.31) | 8.32 (2.60 to 22.30) | 8.74 (2.73 to 23.45) | 8.62 (2.69 to 23.16) | 7.18 (2.28 to 19.20) | 6.47 (2.07 to 17.26) | 5.22 (1.67 to 13.94) | 4.35 (1.35 to 11.72) | 3.49 (1.03 to 9.57) | 2.68 (0.77 to 7.41) | 1.45 (0.42 to 4.02) | 0.54 (0.15 to 1.51) | 0.10 (0.03 to 0.29) |
| Niue | Female | Number of DALYs | 0.02 (0.01 to 0.02) | 0.03 (0.02 to 0.05) | 0.03 (0.02 to 0.04) | 0.02 (0.01 to 0.04) | 0.02 (0.01 to 0.03) | 0.02 (0.01 to 0.03) | 0.02 (0.01 to 0.03) | 0.02 (0.01 to 0.03) | 0.02 (0.01 to 0.02) | 0.02 (0.01 to 0.03) | 0.02 (0.01 to 0.03) | 0.01 (0.01 to 0.02) | 0.01 (0.01 to 0.02) | 0.01 (0.01 to 0.01) | 0.01 (0.01 to 0.01) | 0.01 (0.00 to 0.01) | 0.01 (0.00 to 0.01) | 0.00 (0.00 to 0.01) | 0.00 (0.00 to 0.00) | 0.00 (0.00 to 0.00) |
| Niue | Female | Number of prevalence | 1.13 (0.34 to 3.08) | 3.15 (0.86 to 8.84) | 3.47 (0.88 to 9.93) | 3.80 (0.92 to 10.98) | 3.92 (0.92 to 11.39) | 3.93 (0.91 to 11.45) | 4.04 (0.93 to 11.79) | 3.96 (0.91 to 11.56) | 3.61 (0.82 to 10.56) | 3.91 (0.89 to 11.43) | 3.99 (0.90 to 11.69) | 3.17 (0.72 to 9.29) | 2.82 (0.64 to 8.26) | 2.33 (0.52 to 6.83) | 2.19 (0.49 to 6.42) | 2.08 (0.47 to 6.10) | 1.72 (0.39 to 5.05) | 0.95 (0.21 to 2.77) | 0.38 (0.09 to 1.11) | 0.08 (0.02 to 0.22) |
| Niue | Male | Number of DALYs | 0.01 (0.01 to 0.02) | 0.10 (0.05 to 0.20) | 0.12 (0.06 to 0.22) | 0.15 (0.07 to 0.26) | 0.15 (0.08 to 0.27) | 0.15 (0.08 to 0.25) | 0.15 (0.08 to 0.25) | 0.15 (0.09 to 0.25) | 0.14 (0.08 to 0.23) | 0.15 (0.09 to 0.23) | 0.14 (0.08 to 0.23) | 0.12 (0.08 to 0.19) | 0.11 (0.07 to 0.18) | 0.09 (0.06 to 0.13) | 0.07 (0.04 to 0.10) | 0.04 (0.03 to 0.06) | 0.03 (0.02 to 0.04) | 0.01 (0.01 to 0.02) | 0.00 (0.00 to 0.01) | 0.00 (0.00 to 0.00) |
| Niue | Male | Number of prevalence | 1.19 (0.32 to 3.33) | 3.70 (1.36 to 9.30) | 4.61 (1.63 to 11.74) | 5.60 (2.01 to 14.26) | 5.50 (2.04 to 13.90) | 5.22 (1.98 to 13.10) | 5.12 (1.98 to 12.77) | 5.09 (1.99 to 12.65) | 4.70 (1.85 to 11.64) | 4.83 (1.91 to 11.91) | 4.63 (1.84 to 11.38) | 4.01 (1.60 to 9.84) | 3.65 (1.47 to 8.92) | 2.89 (1.17 to 7.06) | 2.16 (0.87 to 5.26) | 1.41 (0.57 to 3.44) | 0.96 (0.39 to 2.34) | 0.51 (0.21 to 1.24) | 0.17 (0.07 to 0.40) | 0.03 (0.01 to 0.06) |
| Palau | Both | Number of DALYs | 0.14 (0.09 to 0.21) | 0.24 (0.15 to 0.37) | 0.25 (0.15 to 0.37) | 0.25 (0.16 to 0.39) | 0.24 (0.15 to 0.35) | 0.22 (0.15 to 0.33) | 0.21 (0.13 to 0.30) | 0.17 (0.11 to 0.27) | 0.14 (0.09 to 0.21) | 0.11 (0.07 to 0.16) | 0.07 (0.04 to 0.11) | 0.06 (0.04 to 0.10) | 0.10 (0.06 to 0.18) | 0.07 (0.04 to 0.13) | 0.06 (0.03 to 0.10) | 0.03 (0.02 to 0.06) | 0.01 (0.01 to 0.03) | 0.00 (0.00 to 0.01) | 0.00 (0.00 to 0.00) | 0.00 (0.00 to 0.00) |
| Palau | Both | Number of prevalence | 1.44 (1.34 to 1.56) | 2.56 (2.37 to 2.82) | 2.73 (2.50 to 3.09) | 2.92 (2.62 to 3.39) | 2.82 (2.50 to 3.34) | 2.71 (2.37 to 3.28) | 2.55 (2.22 to 3.12) | 2.21 (1.91 to 2.73) | 1.76 (1.51 to 2.18) | 1.42 (1.22 to 1.78) | 0.96 (0.82 to 1.20) | 0.84 (0.71 to 1.06) | 1.20 (0.90 to 1.78) | 0.89 (0.67 to 1.31) | 0.68 (0.52 to 1.01) | 0.44 (0.33 to 0.64) | 0.19 (0.15 to 0.27) | 0.06 (0.05 to 0.09) | 0.01 (0.01 to 0.02) | 0.00 (0.00 to 0.00) |
| Palau | Female | Number of DALYs | 0.08 (0.04 to 0.12) | 0.14 (0.08 to 0.22) | 0.14 (0.08 to 0.22) | 0.14 (0.08 to 0.23) | 0.13 (0.08 to 0.20) | 0.12 (0.07 to 0.19) | 0.12 (0.07 to 0.18) | 0.10 (0.06 to 0.16) | 0.08 (0.04 to 0.12) | 0.06 (0.04 to 0.10) | 0.04 (0.02 to 0.06) | 0.04 (0.02 to 0.06) | 0.04 (0.02 to 0.06) | 0.03 (0.02 to 0.04) | 0.02 (0.01 to 0.03) | 0.01 (0.01 to 0.02) | 0.01 (0.00 to 0.01) | 0.00 (0.00 to 0.00) | 0.00 (0.00 to 0.00) | 0.00 (0.00 to 0.00) |
| Palau | Female | Number of prevalence | 0.82 (0.76 to 0.89) | 1.45 (1.33 to 1.59) | 1.53 (1.39 to 1.72) | 1.62 (1.45 to 1.85) | 1.52 (1.34 to 1.78) | 1.46 (1.28 to 1.74) | 1.41 (1.23 to 1.69) | 1.22 (1.06 to 1.48) | 0.97 (0.84 to 1.18) | 0.78 (0.67 to 0.96) | 0.55 (0.47 to 0.67) | 0.47 (0.41 to 0.59) | 0.47 (0.40 to 0.59) | 0.36 (0.31 to 0.45) | 0.27 (0.23 to 0.34) | 0.18 (0.15 to 0.23) | 0.09 (0.08 to 0.11) | 0.03 (0.03 to 0.04) | 0.01 (0.01 to 0.01) | 0.00 (0.00 to 0.00) |
| Palau | Male | Number of DALYs | 0.06 (0.03 to 0.10) | 0.10 (0.06 to 0.17) | 0.11 (0.06 to 0.18) | 0.11 (0.06 to 0.18) | 0.11 (0.06 to 0.17) | 0.10 (0.05 to 0.16) | 0.09 (0.05 to 0.15) | 0.08 (0.04 to 0.13) | 0.06 (0.03 to 0.10) | 0.05 (0.03 to 0.08) | 0.03 (0.02 to 0.05) | 0.03 (0.01 to 0.04) | 0.07 (0.03 to 0.13) | 0.05 (0.02 to 0.10) | 0.04 (0.02 to 0.08) | 0.02 (0.01 to 0.05) | 0.01 (0.00 to 0.02) | 0.00 (0.00 to 0.01) | 0.00 (0.00 to 0.00) | 0.00 (0.00 to 0.00) |
| Palau | Male | Number of prevalence | 0.63 (0.57 to 0.69) | 1.11 (1.00 to 1.24) | 1.19 (1.06 to 1.38) | 1.30 (1.14 to 1.54) | 1.30 (1.13 to 1.59) | 1.25 (1.07 to 1.55) | 1.15 (0.97 to 1.44) | 0.99 (0.83 to 1.26) | 0.79 (0.66 to 1.01) | 0.64 (0.53 to 0.82) | 0.41 (0.34 to 0.53) | 0.37 (0.30 to 0.48) | 0.73 (0.46 to 1.32) | 0.52 (0.33 to 0.95) | 0.41 (0.26 to 0.74) | 0.26 (0.16 to 0.47) | 0.10 (0.06 to 0.18) | 0.03 (0.02 to 0.06) | 0.01 (0.00 to 0.01) | 0.00 (0.00 to 0.00) |
| Papua New Guinea | Both | Number of DALYs | 127.31 (83.25 to 186.95) | 2386.93 (1597.38 to 3311.84) | 2498.83 (1700.15 to 3481.52) | 2429.53 (1654.29 to 3354.14) | 2520.60 (1716.31 to 3474.78) | 2541.11 (1721.84 to 3613.85) | 2329.75 (1583.72 to 3266.43) | 2085.10 (1414.01 to 2977.28) | 1874.72 (1273.77 to 2636.67) | 1618.56 (1098.16 to 2287.94) | 1405.92 (959.61 to 2021.68) | 1218.76 (827.72 to 1743.45) | 1100.19 (758.59 to 1545.17) | 803.19 (551.03 to 1125.27) | 471.39 (326.81 to 655.34) | 217.31 (150.83 to 301.73) | 84.94 (58.55 to 117.13) | 25.60 (17.83 to 34.65) | 4.95 (3.45 to 6.72) | 1.52 (1.06 to 2.06) |
| Papua New Guinea | Both | Number of prevalence | 36689.17 (13538.27 to 69956.58) | 97891.48 (46042.90 to 171030.59) | 121662.16 (55080.32 to 215890.22) | 139501.91 (61476.77 to 250404.37) | 153339.93 (67143.94 to 275906.13) | 153370.04 (67620.88 to 275409.77) | 136359.78 (60729.66 to 244075.07) | 118374.07 (53289.28 to 211312.04) | 103131.91 (46952.05 to 181626.96) | 86364.27 (39802.15 to 147923.60) | 72728.17 (33908.58 to 119491.83) | 61251.79 (28895.16 to 96603.12) | 53822.35 (25812.96 to 81303.85) | 39130.69 (18913.53 to 57928.87) | 23426.59 (11327.08 to 34679.03) | 11021.48 (5329.11 to 16317.42) | 4381.97 (2120.50 to 6484.87) | 1360.36 (656.36 to 2016.41) | 272.74 (131.22 to 405.15) | 90.82 (43.05 to 136.15) |
| Papua New Guinea | Female | Number of DALYs | 71.73 (45.21 to 108.61) | 153.94 (99.81 to 229.07) | 225.28 (145.95 to 339.16) | 353.27 (225.83 to 513.67) | 521.16 (328.63 to 760.63) | 642.03 (417.47 to 939.83) | 650.95 (415.25 to 963.12) | 622.71 (403.93 to 910.77) | 590.87 (380.99 to 867.72) | 524.56 (341.76 to 765.55) | 465.29 (297.87 to 685.94) | 415.25 (263.71 to 614.42) | 397.70 (256.65 to 576.61) | 295.03 (192.05 to 429.77) | 173.31 (113.14 to 253.39) | 80.27 (52.89 to 113.95) | 30.86 (20.48 to 43.98) | 9.84 (6.55 to 13.91) | 2.04 (1.37 to 2.93) | 0.83 (0.56 to 1.18) |
| Papua New Guinea | Female | Number of prevalence | 17650.65 (6581.59 to 33557.90) | 38478.71 (14328.59 to 73174.77) | 49823.78 (18715.38 to 94443.54) | 60527.63 (23226.11 to 113876.91) | 69300.38 (27303.93 to 129116.49) | 70490.29 (28485.16 to 130153.12) | 62662.56 (25837.87 to 114883.98) | 54227.44 (22695.30 to 98879.93) | 47227.55 (20018.83 to 85783.67) | 39213.83 (16807.57 to 70303.43) | 32688.99 (14221.95 to 56069.45) | 27519.33 (12162.50 to 45183.53) | 24847.66 (11182.13 to 38934.48) | 18150.14 (8237.11 to 27838.62) | 10855.50 (4931.93 to 16649.79) | 5129.16 (2330.88 to 7866.84) | 2012.09 (913.50 to 3086.05) | 656.60 (298.11 to 1007.08) | 140.53 (63.81 to 215.55) | 59.13 (26.85 to 90.69) |
| Papua New Guinea | Male | Number of DALYs | 55.58 (34.16 to 87.12) | 2232.99 (1492.09 to 3103.45) | 2273.55 (1532.18 to 3194.62) | 2076.25 (1399.49 to 2848.52) | 1999.44 (1362.73 to 2793.44) | 1899.07 (1301.91 to 2659.57) | 1678.80 (1156.93 to 2310.48) | 1462.40 (1000.77 to 2082.82) | 1283.85 (888.24 to 1801.85) | 1094.00 (756.50 to 1540.09) | 940.63 (643.80 to 1346.21) | 803.51 (558.52 to 1167.05) | 702.49 (484.39 to 985.23) | 508.16 (358.79 to 718.15) | 298.08 (208.05 to 420.49) | 137.04 (96.27 to 190.83) | 54.08 (38.14 to 74.69) | 15.76 (11.19 to 21.48) | 2.91 (2.06 to 3.96) | 0.68 (0.49 to 0.92) |
| Papua New Guinea | Male | Number of prevalence | 19038.53 (6956.68 to 36398.67) | 59412.77 (32201.13 to 97897.03) | 71838.38 (36448.32 to 121551.93) | 78974.28 (38250.66 to 136527.46) | 84039.56 (39840.02 to 146789.63) | 82879.76 (39084.90 to 145256.65) | 73697.22 (34765.31 to 129218.79) | 64146.63 (30400.36 to 112550.50) | 55904.36 (26666.95 to 95843.29) | 47150.44 (22728.58 to 77620.18) | 40039.19 (19527.61 to 63422.38) | 33732.45 (16646.66 to 51419.59) | 28974.69 (14521.58 to 42369.37) | 20980.54 (10586.60 to 30090.25) | 12571.09 (6340.37 to 18029.24) | 5892.33 (2972.94 to 8450.59) | 2369.88 (1196.80 to 3398.82) | 703.77 (355.16 to 1009.33) | 132.20 (66.75 to 189.61) | 31.70 (15.99 to 45.46) |
| Philippines | Both | Number of DALYs | 1043.59 (712.98 to 1499.37) | 6694.06 (3842.75 to 10977.07) | 9658.44 (5964.81 to 14951.79) | 12446.21 (8065.17 to 18303.70) | 13504.76 (8918.08 to 19414.80) | 13236.11 (8767.70 to 18758.52) | 12265.57 (8177.98 to 17231.59) | 10808.71 (7275.91 to 15117.71) | 8977.92 (5984.76 to 12557.96) | 7350.25 (4928.56 to 10243.86) | 6144.69 (4127.12 to 8519.13) | 4821.27 (3216.63 to 6656.08) | 3740.23 (2508.09 to 5124.12) | 2646.41 (1786.42 to 3631.95) | 1802.15 (1221.65 to 2463.84) | 1143.56 (783.25 to 1550.67) | 660.00 (454.06 to 888.40) | 286.23 (198.78 to 384.79) | 17.51 (12.19 to 23.18) | 3.60 (2.55 to 4.76) |
| Philippines | Both | Number of prevalence | 141160.89 (23463.03 to 422082.74) | 355456.04 (85620.70 to 989979.46) | 482773.85 (121861.31 to 1348664.61) | 589468.36 (152806.97 to 1667600.44) | 626722.80 (163300.74 to 1826933.48) | 607535.03 (159027.07 to 1787221.73) | 558843.21 (146974.16 to 1634315.89) | 491802.23 (130088.77 to 1422049.65) | 411558.91 (109346.12 to 1172327.25) | 342504.58 (90381.77 to 955267.20) | 291640.93 (76418.62 to 810169.30) | 234572.19 (61216.82 to 648855.69) | 187350.94 (48501.23 to 516037.18) | 137638.43 (35319.83 to 377660.97) | 96491.92 (24740.50 to 265172.89) | 62869.77 (16116.57 to 174942.11) | 36742.53 (9415.71 to 103059.61) | 15027.23 (4026.06 to 40925.94) | 768.40 (229.93 to 1964.39) | 147.51 (46.95 to 365.99) |
| Philippines | Female | Number of DALYs | 595.91 (408.31 to 850.55) | 1026.12 (700.35 to 1468.88) | 1097.75 (757.52 to 1605.85) | 1160.53 (792.06 to 1677.23) | 1168.50 (792.97 to 1687.61) | 1100.16 (747.90 to 1584.31) | 997.04 (670.35 to 1437.14) | 866.56 (589.87 to 1236.96) | 725.64 (495.52 to 1032.29) | 612.59 (416.26 to 884.03) | 529.61 (365.98 to 750.81) | 433.70 (293.09 to 620.28) | 352.68 (238.12 to 502.29) | 262.36 (180.42 to 370.45) | 182.25 (126.20 to 255.79) | 117.41 (81.67 to 163.02) | 66.22 (45.95 to 91.81) | 23.18 (16.33 to 32.16) | 0.66 (0.47 to 0.91) | 0.07 (0.05 to 0.10) |
| Philippines | Female | Number of prevalence | 69294.92 (12263.43 to 204901.10) | 155663.91 (24845.07 to 469315.50) | 208039.65 (30792.95 to 637974.02) | 252454.22 (35558.45 to 794632.68) | 268702.74 (35856.92 to 876812.76) | 259334.83 (34063.54 to 854289.01) | 236531.90 (30922.08 to 776014.78) | 206586.21 (26971.67 to 672550.00) | 173332.90 (22890.89 to 554577.21) | 145782.71 (19662.59 to 457697.79) | 125876.41 (16579.70 to 394397.14) | 102813.47 (13498.41 to 318808.48) | 83638.08 (10990.03 to 258144.55) | 63013.31 (8212.81 to 195425.61) | 44671.40 (5778.71 to 139043.42) | 29471.08 (3784.19 to 92183.19) | 17047.07 (2186.98 to 53913.60) | 6097.97 (796.68 to 18873.00) | 179.47 (25.59 to 529.16) | 20.14 (2.85 to 61.41) |
| Philippines | Male | Number of DALYs | 447.68 (302.74 to 643.18) | 5667.95 (3062.47 to 9734.34) | 8560.69 (5133.73 to 13623.24) | 11285.68 (7158.15 to 16844.42) | 12336.27 (7985.86 to 17871.72) | 12135.95 (7955.65 to 17315.32) | 11268.53 (7502.45 to 15969.70) | 9942.15 (6669.85 to 13983.33) | 8252.28 (5485.09 to 11566.06) | 6737.66 (4505.25 to 9411.73) | 5615.08 (3769.50 to 7816.60) | 4387.57 (2927.02 to 6055.68) | 3387.56 (2265.92 to 4660.44) | 2384.06 (1608.04 to 3269.41) | 1619.90 (1098.86 to 2214.43) | 1026.16 (701.65 to 1395.46) | 593.79 (407.86 to 802.00) | 263.05 (182.96 to 353.36) | 16.85 (11.73 to 22.33) | 3.52 (2.50 to 4.66) |
| Philippines | Male | Number of prevalence | 71865.97 (11157.30 to 217196.57) | 199792.13 (58921.38 to 524877.28) | 274734.21 (89119.12 to 710910.57) | 337014.13 (115531.75 to 874058.90) | 358020.06 (125205.22 to 950120.72) | 348200.19 (123380.91 to 932932.72) | 322311.31 (115157.28 to 858301.11) | 285216.02 (102518.71 to 749513.01) | 238226.01 (86407.13 to 619071.75) | 196721.86 (71814.72 to 499620.39) | 165764.52 (60268.59 to 417071.53) | 131758.72 (47570.47 to 329000.07) | 103712.86 (37165.59 to 258541.31) | 74625.11 (26586.40 to 184083.71) | 51820.52 (18398.99 to 126722.65) | 33398.69 (11868.07 to 82195.20) | 19695.46 (6997.94 to 49247.95) | 8929.26 (3162.06 to 22346.31) | 588.93 (206.92 to 1444.66) | 127.37 (44.60 to 300.88) |
| Samoa | Both | Number of DALYs | 3.40 (2.13 to 5.04) | 36.14 (21.38 to 57.44) | 62.25 (40.45 to 90.14) | 81.44 (53.43 to 114.64) | 75.23 (50.17 to 105.42) | 64.02 (43.43 to 88.16) | 47.39 (32.00 to 66.34) | 38.18 (25.69 to 53.21) | 29.12 (20.08 to 41.19) | 25.85 (17.74 to 35.48) | 24.14 (16.61 to 33.25) | 21.07 (14.48 to 28.79) | 17.88 (12.33 to 24.54) | 13.96 (9.73 to 19.12) | 8.70 (6.11 to 11.82) | 4.49 (3.10 to 5.97) | 2.12 (1.51 to 2.90) | 0.99 (0.69 to 1.33) | 0.23 (0.16 to 0.31) | 0.05 (0.03 to 0.06) |
| Samoa | Both | Number of prevalence | 679.60 (143.12 to 1921.31) | 1765.16 (558.76 to 4595.84) | 2616.55 (861.06 to 6681.81) | 3225.56 (1107.30 to 8168.94) | 3071.26 (1041.75 to 7801.32) | 2802.56 (926.49 to 7177.07) | 2247.54 (719.69 to 5809.61) | 1914.74 (597.91 to 4981.38) | 1517.23 (467.58 to 3961.20) | 1398.53 (425.78 to 3664.64) | 1331.79 (403.81 to 3494.20) | 1187.45 (359.53 to 3120.82) | 1038.88 (312.92 to 2737.40) | 823.57 (248.15 to 2170.97) | 528.94 (158.77 to 1395.28) | 302.22 (87.68 to 803.92) | 159.03 (44.66 to 426.28) | 84.44 (22.74 to 228.37) | 23.04 (5.95 to 62.87) | 5.28 (1.33 to 14.49) |
| Samoa | Female | Number of DALYs | 1.95 (1.13 to 2.91) | 3.42 (2.15 to 5.12) | 4.18 (2.66 to 6.22) | 4.80 (3.12 to 7.21) | 4.66 (3.02 to 6.72) | 4.53 (2.94 to 6.66) | 3.98 (2.70 to 5.97) | 3.63 (2.32 to 5.48) | 2.97 (1.91 to 4.31) | 2.84 (1.85 to 4.12) | 2.71 (1.76 to 3.93) | 2.46 (1.61 to 3.55) | 2.21 (1.47 to 3.21) | 1.73 (1.17 to 2.47) | 1.12 (0.76 to 1.61) | 0.72 (0.49 to 1.01) | 0.41 (0.27 to 0.59) | 0.24 (0.17 to 0.35) | 0.07 (0.05 to 0.11) | 0.02 (0.01 to 0.02) |
| Samoa | Female | Number of prevalence | 333.53 (73.09 to 938.03) | 722.57 (151.46 to 2046.67) | 1015.07 (208.10 to 2885.58) | 1222.13 (249.69 to 3477.00) | 1153.00 (237.17 to 3277.61) | 1084.82 (225.05 to 3080.11) | 930.75 (194.56 to 2639.66) | 821.85 (172.94 to 2328.75) | 659.33 (139.74 to 1867.23) | 617.53 (131.83 to 1748.53) | 579.35 (124.39 to 1640.00) | 514.83 (111.30 to 1456.77) | 451.24 (98.40 to 1276.42) | 352.66 (77.17 to 997.26) | 230.44 (50.41 to 651.67) | 151.22 (33.10 to 427.63) | 89.29 (19.53 to 252.51) | 53.49 (11.71 to 151.27) | 16.27 (3.56 to 46.02) | 3.98 (0.87 to 11.24) |
| Samoa | Male | Number of DALYs | 1.45 (0.85 to 2.27) | 32.71 (18.73 to 52.86) | 58.08 (37.52 to 85.37) | 76.64 (50.41 to 107.83) | 70.57 (46.85 to 99.19) | 59.50 (40.26 to 82.38) | 43.40 (29.15 to 60.92) | 34.55 (23.27 to 48.42) | 26.14 (17.86 to 37.20) | 23.01 (15.68 to 31.37) | 21.43 (14.76 to 29.58) | 18.61 (12.72 to 25.50) | 15.66 (10.79 to 21.78) | 12.23 (8.46 to 16.54) | 7.59 (5.33 to 10.27) | 3.77 (2.58 to 5.15) | 1.71 (1.20 to 2.34) | 0.74 (0.53 to 1.01) | 0.16 (0.11 to 0.21) | 0.03 (0.02 to 0.04) |
| Samoa | Male | Number of prevalence | 346.07 (70.45 to 983.28) | 1042.59 (403.27 to 2552.58) | 1601.49 (667.55 to 3799.87) | 2003.43 (861.88 to 4692.31) | 1918.26 (804.85 to 4523.71) | 1717.74 (696.38 to 4096.97) | 1316.79 (522.90 to 3169.95) | 1092.89 (428.50 to 2652.63) | 857.90 (332.69 to 2093.96) | 781.00 (299.37 to 1916.11) | 752.43 (285.09 to 1854.20) | 672.62 (252.55 to 1664.05) | 587.64 (218.46 to 1460.98) | 470.92 (173.94 to 1173.71) | 298.50 (110.30 to 743.61) | 150.99 (55.78 to 376.29) | 69.74 (25.79 to 173.77) | 30.94 (11.44 to 77.10) | 6.76 (2.50 to 16.85) | 1.30 (0.48 to 3.25) |
| Sao Tome and Principe | Both | Number of DALYs | 2.17 (1.38 to 3.17) | 10.47 (5.57 to 18.35) | 12.83 (7.23 to 21.76) | 14.52 (8.37 to 23.86) | 14.88 (8.75 to 23.71) | 14.10 (8.81 to 21.60) | 11.59 (7.35 to 17.46) | 9.52 (6.16 to 14.13) | 7.93 (5.14 to 11.46) | 7.69 (4.96 to 11.34) | 8.37 (5.40 to 12.21) | 7.88 (5.28 to 11.42) | 7.30 (4.78 to 10.62) | 5.48 (3.64 to 7.83) | 3.65 (2.48 to 5.17) | 2.19 (1.48 to 3.10) | 1.16 (0.80 to 1.64) | 0.38 (0.26 to 0.53) | 0.05 (0.03 to 0.07) | 0.01 (0.01 to 0.02) |
| Sao Tome and Principe | Both | Number of prevalence | 228.67 (23.92 to 1301.53) | 585.29 (84.28 to 3132.31) | 739.87 (105.00 to 3986.93) | 792.25 (121.43 to 4246.08) | 765.55 (125.74 to 4071.19) | 705.60 (120.68 to 3736.70) | 576.67 (100.00 to 3051.24) | 472.77 (83.04 to 2500.12) | 384.62 (69.15 to 2027.06) | 364.89 (67.64 to 1916.75) | 384.62 (73.63 to 2011.20) | 362.37 (70.01 to 1894.59) | 334.96 (65.94 to 1751.72) | 255.59 (50.19 to 1338.98) | 175.99 (34.08 to 924.59) | 112.38 (21.10 to 594.64) | 70.77 (11.77 to 383.43) | 30.55 (4.11 to 170.66) | 8.46 (0.64 to 49.91) | 2.62 (0.19 to 15.50) |
| Sao Tome and Principe | Female | Number of DALYs | 1.26 (0.78 to 1.93) | 2.18 (1.30 to 3.33) | 2.12 (1.30 to 3.18) | 1.82 (1.09 to 2.71) | 1.57 (1.01 to 2.31) | 1.38 (0.87 to 2.11) | 1.13 (0.72 to 1.71) | 0.91 (0.55 to 1.32) | 0.71 (0.45 to 1.06) | 0.65 (0.41 to 1.00) | 0.65 (0.41 to 0.95) | 0.60 (0.38 to 0.86) | 0.54 (0.34 to 0.79) | 0.41 (0.26 to 0.60) | 0.29 (0.19 to 0.41) | 0.19 (0.12 to 0.27) | 0.14 (0.09 to 0.20) | 0.07 (0.05 to 0.10) | 0.03 (0.02 to 0.04) | 0.01 (0.01 to 0.01) |
| Sao Tome and Principe | Female | Number of prevalence | 114.27 (13.58 to 642.16) | 266.00 (24.38 to 1532.16) | 332.99 (24.62 to 1949.13) | 349.19 (22.25 to 2063.04) | 335.82 (19.64 to 1992.89) | 314.95 (17.57 to 1873.08) | 264.61 (14.40 to 1575.48) | 221.35 (11.88 to 1318.92) | 176.84 (9.37 to 1054.61) | 162.94 (8.53 to 973.58) | 165.04 (8.62 to 988.06) | 154.93 (8.02 to 929.20) | 142.44 (7.34 to 856.71) | 110.05 (5.66 to 662.68) | 77.63 (4.00 to 467.49) | 52.66 (2.71 to 317.12) | 39.70 (2.05 to 239.04) | 20.85 (1.08 to 125.56) | 7.71 (0.40 to 46.41) | 2.42 (0.12 to 14.58) |
| Sao Tome and Principe | Male | Number of DALYs | 0.91 (0.50 to 1.44) | 8.30 (4.01 to 15.39) | 10.71 (5.60 to 19.65) | 12.70 (7.02 to 21.67) | 13.31 (7.69 to 21.34) | 12.72 (7.80 to 19.74) | 10.46 (6.49 to 16.01) | 8.60 (5.48 to 12.95) | 7.22 (4.63 to 10.52) | 7.04 (4.52 to 10.51) | 7.72 (4.92 to 11.36) | 7.28 (4.84 to 10.69) | 6.76 (4.43 to 9.91) | 5.06 (3.33 to 7.33) | 3.36 (2.26 to 4.79) | 2.00 (1.34 to 2.85) | 1.02 (0.69 to 1.44) | 0.31 (0.21 to 0.44) | 0.02 (0.02 to 0.03) | 0.01 (0.00 to 0.01) |
| Sao Tome and Principe | Male | Number of prevalence | 114.40 (10.20 to 659.37) | 319.30 (58.48 to 1600.15) | 406.89 (77.57 to 2037.79) | 443.06 (95.37 to 2183.04) | 429.73 (100.37 to 2078.30) | 390.64 (96.91 to 1863.62) | 312.07 (81.16 to 1475.76) | 251.41 (67.29 to 1181.20) | 207.77 (57.16 to 972.46) | 201.94 (56.50 to 943.16) | 219.58 (62.41 to 1023.14) | 207.44 (59.46 to 965.39) | 192.52 (56.14 to 895.02) | 145.54 (42.63 to 676.30) | 98.36 (28.86 to 457.10) | 59.72 (17.49 to 277.52) | 31.07 (9.11 to 144.39) | 9.70 (2.84 to 45.10) | 0.75 (0.22 to 3.50) | 0.20 (0.06 to 0.91) |
| Senegal | Both | Number of DALYs | 150.53 (93.23 to 235.89) | 606.63 (332.12 to 1060.78) | 667.16 (370.71 to 1129.40) | 762.83 (428.52 to 1259.43) | 797.84 (471.33 to 1254.40) | 783.71 (476.11 to 1205.14) | 719.61 (454.33 to 1089.80) | 625.04 (395.59 to 948.30) | 555.01 (354.26 to 824.42) | 456.17 (292.99 to 663.01) | 415.37 (274.43 to 614.54) | 359.40 (230.81 to 523.08) | 317.13 (211.79 to 453.95) | 261.03 (171.47 to 375.50) | 178.49 (119.12 to 252.84) | 107.18 (71.72 to 153.11) | 52.79 (35.85 to 75.20) | 20.78 (14.13 to 29.41) | 5.66 (3.80 to 8.00) | 0.61 (0.42 to 0.85) |
| Senegal | Both | Number of prevalence | 14951.94 (2707.05 to 55574.70) | 34194.40 (8078.98 to 119905.12) | 39786.22 (9076.30 to 140922.12) | 44206.36 (10186.51 to 156458.26) | 44478.17 (10410.78 to 156809.44) | 41919.67 (9918.11 to 147223.54) | 37144.98 (8907.76 to 129963.84) | 31580.48 (7685.75 to 110098.83) | 27430.26 (6772.36 to 95295.92) | 22276.01 (5579.12 to 77169.49) | 19781.11 (5033.71 to 68277.76) | 16728.51 (4348.53 to 57466.67) | 14478.80 (3830.04 to 49500.07) | 11776.45 (3157.22 to 40112.44) | 8400.25 (2223.60 to 28717.85) | 5182.63 (1371.22 to 17722.67) | 2656.34 (692.47 to 9121.61) | 1110.55 (283.84 to 3835.28) | 332.71 (81.11 to 1162.83) | 48.60 (10.01 to 175.81) |
| Senegal | Female | Number of DALYs | 87.84 (51.17 to 137.20) | 138.02 (84.56 to 213.04) | 122.60 (72.85 to 182.68) | 112.50 (67.90 to 171.70) | 98.89 (59.22 to 146.91) | 88.39 (56.44 to 137.89) | 74.81 (47.11 to 108.46) | 60.50 (38.26 to 91.38) | 50.14 (31.54 to 72.37) | 39.19 (24.46 to 57.98) | 34.14 (21.89 to 50.81) | 27.32 (17.18 to 40.79) | 22.35 (14.18 to 32.25) | 17.21 (11.04 to 25.51) | 12.51 (8.04 to 18.42) | 7.66 (4.88 to 11.10) | 4.03 (2.65 to 6.03) | 1.73 (1.11 to 2.48) | 0.57 (0.37 to 0.80) | 0.11 (0.07 to 0.15) |
| Senegal | Female | Number of prevalence | 7575.95 (1443.74 to 27825.21) | 15913.24 (2629.53 to 59818.38) | 18599.27 (2751.89 to 71008.96) | 20673.14 (2846.95 to 79630.21) | 20930.12 (2784.11 to 80968.67) | 19704.64 (2568.49 to 76392.16) | 17291.99 (2230.22 to 67115.94) | 14485.23 (1855.57 to 56265.01) | 12355.06 (1576.57 to 48017.44) | 9919.84 (1259.99 to 38568.37) | 8593.30 (1088.34 to 33421.82) | 6994.50 (883.67 to 27212.12) | 5845.98 (736.46 to 22751.91) | 4593.12 (579.15 to 17877.46) | 3409.76 (429.48 to 13271.90) | 2115.72 (266.54 to 8235.30) | 1128.15 (142.14 to 4391.13) | 499.89 (62.91 to 1945.73) | 166.85 (21.00 to 649.43) | 31.85 (4.01 to 123.99) |
| Senegal | Male | Number of DALYs | 62.69 (33.18 to 107.11) | 468.61 (225.73 to 910.28) | 544.57 (274.24 to 985.69) | 650.34 (353.52 to 1112.76) | 698.95 (399.71 to 1151.46) | 695.32 (413.15 to 1102.43) | 644.80 (396.50 to 987.39) | 564.54 (351.66 to 858.29) | 504.87 (319.74 to 762.00) | 416.99 (262.08 to 608.90) | 381.23 (248.04 to 564.85) | 332.07 (209.95 to 486.79) | 294.78 (193.06 to 426.19) | 243.82 (157.51 to 353.26) | 165.98 (108.28 to 237.71) | 99.52 (65.62 to 143.45) | 48.76 (32.67 to 69.85) | 19.05 (12.70 to 27.03) | 5.10 (3.36 to 7.25) | 0.50 (0.34 to 0.71) |
| Senegal | Male | Number of prevalence | 7375.98 (1260.94 to 27749.50) | 18281.16 (5075.06 to 60086.74) | 21186.95 (5932.30 to 69913.16) | 23533.22 (6994.89 to 76828.05) | 23548.05 (7381.48 to 75840.77) | 22215.03 (7260.61 to 70831.38) | 19852.99 (6622.12 to 62847.91) | 17095.25 (5827.07 to 53833.82) | 15075.20 (5213.10 to 47278.48) | 12356.18 (4318.70 to 38601.13) | 11187.82 (3938.26 to 34855.94) | 9734.01 (3453.13 to 30254.55) | 8632.83 (3071.58 to 26748.16) | 7183.32 (2563.43 to 22234.97) | 4990.50 (1778.13 to 15445.95) | 3066.91 (1094.99 to 9487.37) | 1528.19 (544.53 to 4730.48) | 610.65 (217.94 to 1889.54) | 165.86 (59.23 to 513.40) | 16.75 (5.98 to 51.83) |
| Sierra Leone | Both | Number of DALYs | 126.97 (81.28 to 184.75) | 1975.66 (1305.56 to 2787.02) | 2077.14 (1386.22 to 2885.35) | 1923.98 (1331.13 to 2685.14) | 1883.41 (1290.65 to 2606.84) | 1830.44 (1256.28 to 2566.68) | 1720.11 (1186.62 to 2410.25) | 1486.19 (1016.80 to 2073.57) | 1231.02 (842.19 to 1721.62) | 1005.45 (688.46 to 1426.97) | 855.26 (587.78 to 1187.48) | 725.53 (496.93 to 1028.95) | 640.22 (436.40 to 905.13) | 546.62 (377.60 to 756.29) | 417.56 (286.14 to 585.51) | 282.69 (196.28 to 397.97) | 158.50 (110.47 to 216.92) | 43.24 (30.40 to 59.05) | 14.62 (10.40 to 19.86) | 3.48 (2.46 to 4.66) |
| Sierra Leone | Both | Number of prevalence | 34272.40 (4547.80 to 86763.39) | 83130.40 (23493.62 to 188784.42) | 93286.11 (25277.11 to 213977.24) | 105749.35 (25781.11 to 248673.59) | 118664.88 (26967.82 to 282229.27) | 120711.73 (27091.03 to 288025.97) | 111983.94 (25580.83 to 267045.24) | 93942.58 (22022.67 to 219974.17) | 75968.82 (18168.04 to 171380.34) | 60667.38 (14714.98 to 131448.83) | 51265.39 (12580.99 to 107339.47) | 43413.53 (10736.00 to 87868.41) | 37782.06 (9532.49 to 73658.11) | 32295.20 (8251.23 to 61898.07) | 25006.30 (6400.88 to 47903.69) | 17266.94 (4413.64 to 33082.58) | 9930.33 (2527.13 to 19048.97) | 2871.43 (717.94 to 5538.35) | 1014.11 (251.10 to 1961.89) | 255.53 (62.30 to 496.53) |
| Sierra Leone | Female | Number of DALYs | 73.64 (45.79 to 108.23) | 135.74 (82.19 to 204.80) | 161.14 (106.51 to 244.38) | 231.16 (151.59 to 343.55) | 344.51 (220.30 to 516.55) | 421.23 (277.25 to 615.65) | 426.48 (273.70 to 632.07) | 371.30 (240.69 to 534.80) | 306.71 (198.39 to 445.60) | 251.11 (162.36 to 370.74) | 223.21 (142.66 to 324.29) | 204.25 (132.36 to 296.22) | 188.29 (121.95 to 274.61) | 160.79 (104.65 to 233.07) | 121.00 (79.77 to 175.62) | 82.78 (53.46 to 122.81) | 48.10 (31.25 to 68.93) | 15.39 (10.21 to 22.15) | 5.64 (3.78 to 8.05) | 1.50 (1.02 to 2.12) |
| Sierra Leone | Female | Number of prevalence | 17068.86 (2346.79 to 43058.75) | 34410.58 (4628.38 to 87004.06) | 39086.89 (5326.77 to 98745.04) | 48032.08 (6876.21 to 120814.39) | 59346.95 (9028.08 to 148399.82) | 62752.26 (10106.47 to 156067.61) | 56839.22 (9582.85 to 140863.24) | 45448.79 (8068.66 to 112374.91) | 35167.42 (6509.73 to 84591.90) | 27080.66 (5203.73 to 62445.01) | 22908.07 (4519.11 to 50875.66) | 19852.38 (4010.51 to 42430.99) | 17312.55 (3591.36 to 35512.31) | 14601.82 (3064.40 to 29418.82) | 11171.14 (2345.36 to 22507.26) | 7741.14 (1622.74 to 15596.63) | 4579.36 (961.03 to 9226.26) | 1493.02 (313.57 to 3008.04) | 560.25 (117.60 to 1128.76) | 153.32 (32.15 to 308.91) |
| Sierra Leone | Male | Number of DALYs | 53.33 (31.94 to 85.44) | 1839.93 (1203.75 to 2634.12) | 1916.00 (1274.16 to 2680.30) | 1692.82 (1166.61 to 2368.73) | 1538.90 (1037.69 to 2128.79) | 1409.21 (946.63 to 1950.82) | 1293.63 (876.25 to 1782.85) | 1114.88 (766.51 to 1540.81) | 924.32 (631.16 to 1278.16) | 754.34 (518.80 to 1050.50) | 632.05 (436.57 to 864.20) | 521.28 (362.26 to 732.80) | 451.94 (310.43 to 625.65) | 385.83 (271.59 to 526.40) | 296.56 (205.74 to 410.66) | 199.91 (140.31 to 274.44) | 110.40 (77.81 to 149.29) | 27.85 (19.71 to 37.50) | 8.98 (6.38 to 12.11) | 1.98 (1.42 to 2.66) |
| Sierra Leone | Male | Number of prevalence | 17203.55 (2201.01 to 43704.63) | 48719.82 (18555.78 to 101899.16) | 54199.22 (19805.32 to 115232.20) | 57717.27 (18665.44 to 127859.20) | 59317.94 (17939.74 to 133829.45) | 57959.48 (16822.13 to 131964.99) | 55144.72 (15700.61 to 126191.08) | 48493.79 (13675.10 to 107599.25) | 40801.40 (11508.20 to 86788.44) | 33586.72 (9475.73 to 69003.82) | 28357.32 (8028.93 to 56463.81) | 23561.15 (6682.68 to 45437.43) | 20469.50 (5842.78 to 38145.80) | 17693.38 (5087.40 to 32479.25) | 13835.17 (3972.67 to 25396.43) | 9525.81 (2739.41 to 17485.95) | 5350.97 (1537.39 to 9822.70) | 1378.41 (396.13 to 2530.30) | 453.86 (130.45 to 833.13) | 102.21 (29.34 to 187.62) |
| South Sudan | Both | Number of DALYs | 97.08 (62.58 to 147.94) | 342.32 (183.53 to 634.89) | 377.75 (203.25 to 682.19) | 414.89 (227.01 to 715.84) | 429.27 (232.26 to 724.35) | 398.07 (219.26 to 663.80) | 338.35 (195.62 to 566.68) | 279.71 (158.41 to 459.91) | 236.31 (138.04 to 389.21) | 205.58 (119.21 to 329.95) | 180.32 (107.02 to 292.93) | 154.68 (90.68 to 249.49) | 139.66 (83.58 to 223.13) | 137.17 (82.25 to 213.74) | 95.45 (58.15 to 148.67) | 48.79 (30.62 to 76.55) | 20.45 (12.89 to 31.34) | 6.67 (4.10 to 10.24) | 1.57 (0.99 to 2.37) | 0.30 (0.20 to 0.45) |
| South Sudan | Both | Number of prevalence | 6897.01 (1949.68 to 20373.52) | 16053.36 (4950.28 to 45644.72) | 20276.49 (5898.12 to 58680.76) | 23753.67 (6791.83 to 69208.53) | 24736.10 (7076.22 to 72204.22) | 22740.34 (6561.03 to 66326.64) | 19237.95 (5580.37 to 56046.59) | 15588.85 (4583.00 to 45296.96) | 13279.99 (3899.31 to 38624.85) | 10977.85 (3316.08 to 31685.20) | 9243.03 (2858.12 to 26531.21) | 7707.79 (2422.86 to 21986.84) | 6712.54 (2133.27 to 19054.88) | 6536.63 (2096.41 to 18482.93) | 4795.73 (1527.01 to 13617.23) | 2665.18 (827.26 to 7641.60) | 1229.40 (370.62 to 3556.56) | 445.28 (130.83 to 1299.58) | 115.81 (33.11 to 341.09) | 26.32 (7.26 to 78.51) |
| South Sudan | Female | Number of DALYs | 54.95 (32.99 to 86.24) | 84.48 (51.60 to 132.97) | 79.03 (46.97 to 119.98) | 73.60 (43.70 to 110.57) | 67.61 (39.89 to 106.01) | 56.15 (33.52 to 83.88) | 44.96 (28.42 to 67.29) | 33.88 (21.00 to 52.58) | 29.09 (18.72 to 42.16) | 21.03 (13.34 to 32.19) | 16.04 (10.31 to 24.60) | 12.02 (7.29 to 18.14) | 9.60 (5.95 to 14.20) | 8.61 (5.37 to 12.51) | 6.63 (4.17 to 10.09) | 4.27 (2.69 to 6.10) | 2.18 (1.39 to 3.23) | 0.87 (0.54 to 1.22) | 0.25 (0.16 to 0.35) | 0.06 (0.04 to 0.09) |
| South Sudan | Female | Number of prevalence | 3383.11 (1024.03 to 9829.94) | 7014.09 (1876.44 to 21058.20) | 8827.42 (2148.87 to 27097.64) | 10302.87 (2356.99 to 32044.96) | 10776.86 (2383.61 to 33746.68) | 9892.27 (2149.78 to 31085.02) | 8367.75 (1799.07 to 26346.18) | 6621.49 (1413.57 to 20876.63) | 5840.98 (1239.35 to 18434.99) | 4352.79 (919.79 to 13748.91) | 3411.48 (718.42 to 10783.36) | 2623.77 (550.09 to 8299.37) | 2158.51 (451.06 to 6832.04) | 1976.12 (412.34 to 6256.67) | 1567.91 (326.91 to 4964.10) | 1024.50 (213.89 to 3243.66) | 536.94 (111.95 to 1700.15) | 218.72 (45.60 to 692.50) | 63.13 (13.18 to 199.87) | 16.39 (3.42 to 51.90) |
| South Sudan | Male | Number of DALYs | 42.12 (23.97 to 67.82) | 257.85 (119.64 to 508.08) | 298.72 (138.51 to 602.31) | 341.29 (164.11 to 620.91) | 361.66 (182.70 to 634.79) | 341.92 (175.42 to 603.36) | 293.39 (158.36 to 506.53) | 245.84 (131.67 to 416.24) | 207.21 (111.16 to 352.88) | 184.55 (102.80 to 307.03) | 164.29 (94.17 to 277.65) | 142.67 (80.53 to 232.11) | 130.06 (76.26 to 211.84) | 128.56 (75.35 to 203.26) | 88.82 (52.59 to 140.53) | 44.52 (26.70 to 71.71) | 18.27 (11.11 to 28.63) | 5.80 (3.48 to 9.20) | 1.32 (0.81 to 2.06) | 0.24 (0.15 to 0.37) |
| South Sudan | Male | Number of prevalence | 3513.90 (925.65 to 10543.57) | 9039.27 (3068.54 to 24883.69) | 11449.07 (3718.29 to 31958.43) | 13450.80 (4337.76 to 37599.06) | 13959.24 (4604.84 to 38834.25) | 12848.07 (4284.44 to 35534.15) | 10870.20 (3667.73 to 29932.03) | 8967.36 (3056.60 to 24579.66) | 7439.01 (2570.41 to 20311.40) | 6625.06 (2311.22 to 18039.67) | 5831.55 (2055.60 to 15824.10) | 5084.02 (1808.63 to 13750.19) | 4554.03 (1638.20 to 12274.77) | 4560.51 (1646.86 to 12269.34) | 3227.82 (1167.67 to 8683.96) | 1640.69 (594.07 to 4415.32) | 692.46 (249.49 to 1863.52) | 226.56 (81.75 to 609.38) | 52.68 (19.02 to 141.75) | 9.93 (3.58 to 26.71) |
| Sri Lanka | Both | Number of DALYs | 172.14 (107.37 to 261.62) | 546.53 (286.17 to 976.04) | 560.14 (288.18 to 1031.71) | 531.29 (282.73 to 939.71) | 516.26 (271.85 to 949.42) | 497.96 (280.74 to 885.40) | 458.87 (241.24 to 823.10) | 409.43 (219.72 to 727.19) | 344.98 (181.94 to 620.56) | 271.71 (149.32 to 490.01) | 220.24 (119.91 to 402.99) | 185.16 (99.15 to 340.50) | 151.19 (80.93 to 263.62) | 111.67 (57.83 to 196.43) | 76.68 (41.20 to 133.95) | 47.76 (25.47 to 85.08) | 26.60 (15.01 to 46.60) | 10.47 (5.96 to 18.05) | 2.46 (1.33 to 4.41) | 0.28 (0.15 to 0.51) |
| Sri Lanka | Both | Number of prevalence | 4110.13 (3434.87 to 4946.84) | 11285.64 (8988.14 to 14419.17) | 14095.97 (11080.07 to 18226.38) | 16104.13 (12593.09 to 20768.18) | 17665.32 (13721.03 to 22719.30) | 18512.26 (14303.39 to 23858.53) | 17676.73 (13634.47 to 22718.28) | 16511.28 (12710.80 to 21193.60) | 14326.65 (11011.07 to 18373.70) | 11596.54 (8903.06 to 14893.40) | 9634.66 (7389.88 to 12395.15) | 8404.41 (6438.87 to 10823.64) | 7006.59 (5363.55 to 9018.02) | 5331.08 (4080.62 to 6860.91) | 3728.77 (2852.47 to 4795.94) | 2387.98 (1827.89 to 3071.56) | 1350.34 (1033.30 to 1737.58) | 547.57 (419.04 to 703.41) | 127.99 (97.99 to 164.39) | 13.88 (10.61 to 17.94) |
| Sri Lanka | Female | Number of DALYs | 98.83 (56.98 to 155.54) | 180.31 (107.85 to 274.03) | 177.21 (103.07 to 274.91) | 166.29 (99.30 to 248.48) | 157.48 (91.90 to 248.24) | 148.97 (91.62 to 222.01) | 134.63 (76.52 to 203.79) | 118.84 (71.00 to 176.62) | 98.19 (59.17 to 150.12) | 77.07 (45.07 to 120.26) | 62.00 (36.00 to 92.27) | 51.54 (30.78 to 80.12) | 41.24 (23.17 to 64.18) | 29.95 (17.85 to 44.41) | 21.12 (11.97 to 31.95) | 13.02 (7.89 to 19.53) | 7.36 (4.51 to 11.33) | 2.99 (1.83 to 4.44) | 0.65 (0.40 to 0.99) | 0.05 (0.03 to 0.08) |
| Sri Lanka | Female | Number of prevalence | 2169.42 (1837.16 to 2598.41) | 4907.92 (4039.73 to 6005.86) | 6228.89 (4992.05 to 7775.44) | 7244.42 (5694.83 to 9173.14) | 8080.39 (6287.73 to 10315.61) | 8519.64 (6588.88 to 10927.88) | 8120.30 (6252.21 to 10439.73) | 7577.61 (5819.99 to 9757.13) | 6592.93 (5049.12 to 8504.78) | 5318.40 (4064.07 to 6866.43) | 4426.45 (3380.69 to 5720.58) | 3863.21 (2941.63 to 4998.03) | 3177.10 (2414.03 to 4113.97) | 2410.97 (1830.60 to 3124.12) | 1707.20 (1296.41 to 2212.32) | 1097.90 (833.27 to 1422.09) | 623.72 (473.60 to 807.89) | 259.16 (196.74 to 335.63) | 57.33 (43.53 to 74.29) | 4.71 (3.58 to 6.10) |
| Sri Lanka | Male | Number of DALYs | 73.31 (40.69 to 115.48) | 366.22 (158.50 to 758.61) | 382.93 (161.80 to 802.20) | 365.00 (158.13 to 767.25) | 358.77 (164.50 to 744.05) | 348.99 (162.92 to 700.05) | 324.24 (149.20 to 661.26) | 290.59 (125.03 to 592.23) | 246.79 (112.28 to 509.63) | 194.64 (90.03 to 395.51) | 158.24 (72.02 to 323.37) | 133.62 (59.87 to 273.56) | 109.95 (49.85 to 218.96) | 81.71 (35.71 to 157.89) | 55.56 (25.74 to 108.91) | 34.73 (16.03 to 69.07) | 19.24 (8.87 to 38.08) | 7.49 (3.39 to 14.66) | 1.81 (0.82 to 3.62) | 0.23 (0.10 to 0.45) |
| Sri Lanka | Male | Number of prevalence | 1940.71 (1583.85 to 2357.96) | 6377.72 (4713.16 to 9058.85) | 7867.07 (5871.77 to 10783.05) | 8859.71 (6650.82 to 11749.62) | 9584.93 (7233.54 to 12721.16) | 9992.62 (7559.76 to 13294.39) | 9556.43 (7240.59 to 12747.41) | 8933.67 (6761.36 to 11884.73) | 7733.73 (5856.63 to 10265.33) | 6278.14 (4753.80 to 8327.84) | 5208.22 (3942.87 to 6902.96) | 4541.21 (3435.40 to 6004.17) | 3829.48 (2898.36 to 5058.37) | 2920.11 (2211.65 to 3853.68) | 2021.57 (1530.01 to 2670.46) | 1290.08 (976.60 to 1702.40) | 726.63 (549.85 to 959.13) | 288.41 (218.38 to 380.60) | 70.66 (53.50 to 93.62) | 9.17 (6.94 to 12.09) |
| Sudan | Both | Number of DALYs | 335.93 (205.93 to 514.25) | 1211.34 (662.31 to 2265.37) | 1331.07 (721.11 to 2346.21) | 1444.15 (784.50 to 2551.84) | 1436.87 (811.31 to 2376.88) | 1345.07 (772.40 to 2264.22) | 1230.82 (700.94 to 1976.74) | 1094.48 (659.40 to 1744.46) | 1007.53 (599.44 to 1607.95) | 844.41 (509.70 to 1316.95) | 717.24 (434.64 to 1127.80) | 597.39 (355.57 to 922.71) | 511.49 (312.25 to 795.76) | 449.32 (277.14 to 693.76) | 363.08 (227.12 to 555.84) | 202.13 (124.82 to 303.25) | 85.94 (53.59 to 128.68) | 30.10 (18.91 to 45.08) | 7.71 (4.93 to 11.46) | 1.31 (0.86 to 1.90) |
| Sudan | Both | Number of prevalence | 25409.09 (4388.45 to 101724.40) | 59431.14 (12455.77 to 228661.95) | 73192.12 (14195.49 to 286846.59) | 84228.72 (15718.17 to 332476.32) | 85229.52 (15753.53 to 337236.57) | 79407.53 (14844.34 to 313753.53) | 71561.11 (13588.69 to 281977.93) | 62962.46 (12061.74 to 247635.70) | 54784.18 (10806.70 to 213756.31) | 46434.60 (9177.11 to 181365.83) | 38301.09 (7728.52 to 148871.18) | 31176.33 (6429.71 to 120555.92) | 25810.39 (5522.22 to 99168.91) | 22554.26 (4923.09 to 86310.56) | 19208.14 (4085.36 to 73891.58) | 11289.95 (2339.27 to 43659.40) | 4984.38 (1021.33 to 19319.36) | 1797.76 (366.27 to 6976.60) | 481.97 (96.85 to 1877.76) | 92.84 (17.47 to 366.38) |
| Sudan | Female | Number of DALYs | 187.74 (109.15 to 298.02) | 296.45 (173.83 to 451.40) | 275.01 (164.33 to 425.71) | 257.97 (152.36 to 392.89) | 235.15 (147.66 to 357.46) | 202.67 (126.45 to 299.21) | 171.62 (101.34 to 253.81) | 145.31 (90.64 to 220.68) | 113.29 (71.92 to 173.52) | 97.16 (59.52 to 148.21) | 75.81 (48.13 to 113.05) | 57.16 (35.78 to 84.03) | 43.65 (27.62 to 65.94) | 36.09 (21.64 to 54.54) | 32.65 (20.86 to 49.44) | 20.11 (12.67 to 29.68) | 8.91 (5.75 to 13.24) | 3.19 (2.02 to 4.64) | 0.88 (0.54 to 1.28) | 0.19 (0.12 to 0.26) |
| Sudan | Female | Number of prevalence | 12334.54 (2348.82 to 48573.70) | 26505.70 (4089.97 to 107972.48) | 32576.91 (4177.00 to 135723.20) | 38119.90 (4290.33 to 160970.25) | 40284.00 (4215.96 to 171275.78) | 37759.23 (3797.02 to 161103.60) | 33716.89 (3314.78 to 144143.45) | 29640.89 (2859.76 to 126892.89) | 23794.31 (2268.99 to 101967.77) | 20902.01 (1972.32 to 89648.17) | 16554.28 (1550.24 to 71048.68) | 12920.73 (1199.14 to 55487.98) | 10116.16 (930.90 to 43474.26) | 8497.60 (779.02 to 36529.63) | 7748.49 (710.83 to 33309.40) | 4854.14 (444.85 to 20866.69) | 2202.53 (201.96 to 9468.04) | 806.05 (73.98 to 3464.99) | 226.04 (20.70 to 971.66) | 49.66 (4.55 to 213.46) |
| Sudan | Male | Number of DALYs | 148.19 (80.50 to 235.86) | 914.88 (437.98 to 1870.89) | 1056.06 (506.89 to 2027.22) | 1186.18 (564.32 to 2206.62) | 1201.72 (626.93 to 2086.49) | 1142.39 (598.34 to 2011.19) | 1059.21 (575.10 to 1793.52) | 949.17 (548.68 to 1569.74) | 894.25 (515.64 to 1449.64) | 747.25 (432.83 to 1199.61) | 641.43 (380.33 to 1034.86) | 540.23 (314.70 to 845.87) | 467.84 (280.05 to 741.30) | 413.23 (250.87 to 646.64) | 330.43 (200.15 to 518.32) | 182.02 (109.94 to 278.63) | 77.03 (47.93 to 119.13) | 26.91 (16.14 to 41.10) | 6.83 (4.27 to 10.36) | 1.13 (0.70 to 1.68) |
| Sudan | Male | Number of prevalence | 13074.55 (2095.02 to 53150.70) | 32925.44 (7694.89 to 120793.15) | 40615.21 (9015.54 to 151221.94) | 46108.82 (10353.41 to 171575.24) | 44945.52 (10463.79 to 166005.04) | 41648.30 (10041.66 to 152674.36) | 37844.22 (9412.39 to 137845.76) | 33321.57 (8492.08 to 120746.73) | 30989.87 (8067.72 to 111791.56) | 25532.59 (6770.97 to 91717.66) | 21746.81 (5866.40 to 77822.50) | 18255.60 (4996.67 to 65067.94) | 15694.23 (4358.82 to 55694.66) | 14056.66 (3918.11 to 49780.93) | 11459.65 (3199.30 to 40582.18) | 6435.81 (1794.62 to 22792.71) | 2781.86 (775.25 to 9851.32) | 991.71 (275.97 to 3511.61) | 255.93 (71.50 to 906.10) | 43.18 (12.07 to 152.92) |
| Thailand | Both | Number of DALYs | 557.13 (350.20 to 856.29) | 2626.27 (1415.61 to 4639.93) | 3729.73 (2047.16 to 6509.97) | 4865.21 (2667.67 to 8216.13) | 6375.85 (3569.64 to 10521.46) | 6464.33 (3783.60 to 10516.99) | 6508.18 (3841.34 to 10038.29) | 5842.17 (3641.04 to 9109.19) | 4945.64 (3056.66 to 7735.29) | 4296.55 (2745.02 to 6530.69) | 3984.64 (2474.58 to 5922.81) | 3437.72 (2211.50 to 5163.88) | 2799.64 (1792.59 to 4087.30) | 1843.75 (1205.98 to 2689.15) | 1189.88 (776.61 to 1753.35) | 714.35 (467.12 to 1032.51) | 440.53 (278.53 to 643.93) | 165.67 (109.71 to 239.45) | 39.06 (26.88 to 56.10) | 9.63 (6.50 to 13.72) |
| Thailand | Both | Number of prevalence | 49001.98 (8618.74 to 202836.82) | 137485.24 (30183.92 to 546134.98) | 212103.68 (44436.14 to 853732.88) | 279159.17 (57934.03 to 1125613.06) | 349631.70 (75042.87 to 1399458.36) | 352382.94 (75989.95 to 1408484.72) | 346695.57 (76127.14 to 1379544.60) | 304942.08 (67758.41 to 1208883.39) | 254266.36 (57205.02 to 1004600.94) | 220530.37 (49907.65 to 869695.64) | 204171.61 (46573.30 to 803740.85) | 176906.01 (40474.24 to 695249.54) | 142524.64 (33014.31 to 558430.37) | 96060.16 (22142.88 to 376661.59) | 64874.89 (14712.69 to 255494.90) | 41179.75 (9171.38 to 162983.30) | 25828.15 (5732.08 to 102282.11) | 10697.05 (2276.91 to 42790.18) | 3208.71 (607.13 to 13145.91) | 1269.40 (189.00 to 5391.68) |
| Thailand | Female | Number of DALYs | 320.56 (186.96 to 484.02) | 609.86 (370.45 to 982.25) | 703.91 (430.58 to 1084.19) | 760.07 (455.69 to 1180.50) | 801.94 (477.97 to 1231.91) | 792.17 (484.13 to 1234.73) | 728.82 (447.03 to 1107.86) | 618.16 (401.67 to 942.31) | 503.81 (302.82 to 741.60) | 421.50 (262.19 to 632.91) | 383.40 (233.99 to 559.36) | 327.68 (201.27 to 475.00) | 257.56 (158.38 to 389.44) | 173.94 (110.59 to 260.57) | 120.88 (76.65 to 177.23) | 77.29 (52.29 to 113.46) | 48.14 (31.46 to 71.15) | 21.53 (13.43 to 31.61) | 7.57 (4.82 to 11.09) | 3.72 (2.34 to 5.34) |
| Thailand | Female | Number of prevalence | 24506.63 (4665.45 to 100026.51) | 62374.78 (9960.43 to 261949.19) | 94765.59 (13156.06 to 405652.22) | 126480.05 (16016.89 to 547424.30) | 152042.98 (18342.50 to 661701.35) | 160365.74 (18857.67 to 699853.43) | 156345.46 (18097.92 to 683393.60) | 136507.26 (15635.91 to 597290.96) | 112564.51 (12800.33 to 492952.76) | 98113.98 (11103.83 to 429919.08) | 91170.32 (10270.18 to 399695.48) | 79574.59 (8930.54 to 349025.47) | 63503.53 (7081.17 to 278671.57) | 43672.84 (4856.83 to 191685.55) | 30775.08 (3425.18 to 135081.58) | 20470.39 (2280.53 to 89850.02) | 12909.23 (1436.53 to 56661.49) | 5847.03 (650.53 to 25664.54) | 2115.88 (235.25 to 9287.41) | 1059.82 (118.01 to 4651.67) |
| Thailand | Male | Number of DALYs | 236.56 (135.91 to 377.08) | 2016.41 (942.27 to 3960.87) | 3025.83 (1457.71 to 5630.78) | 4105.15 (2073.69 to 7255.96) | 5573.91 (2941.19 to 9450.58) | 5672.17 (3172.83 to 9542.73) | 5779.36 (3247.20 to 9147.05) | 5224.02 (3072.31 to 8296.18) | 4441.82 (2666.55 to 7012.58) | 3875.06 (2430.31 to 5982.20) | 3601.23 (2210.25 to 5412.36) | 3110.04 (1976.32 to 4729.16) | 2542.07 (1604.85 to 3791.20) | 1669.82 (1066.66 to 2459.75) | 1068.99 (681.82 to 1597.97) | 637.05 (414.11 to 940.30) | 392.38 (243.51 to 584.21) | 144.13 (91.63 to 211.29) | 31.48 (20.41 to 46.29) | 5.91 (3.88 to 8.50) |
| Thailand | Male | Number of prevalence | 24495.35 (3953.65 to 102810.30) | 75110.46 (19342.15 to 282376.89) | 117338.09 (29802.30 to 445211.17) | 152679.12 (39953.62 to 575841.87) | 197588.71 (54182.99 to 736698.86) | 192017.20 (54282.72 to 708777.34) | 190350.11 (55232.10 to 696375.42) | 168434.82 (49982.16 to 611782.39) | 141701.85 (42714.50 to 511796.74) | 122416.39 (37590.09 to 439867.15) | 113001.29 (35211.96 to 404132.84) | 97331.42 (30777.58 to 346296.70) | 79021.11 (25429.97 to 279810.45) | 52387.32 (16931.78 to 185009.22) | 34099.81 (11007.64 to 120432.63) | 20709.37 (6702.45 to 73146.57) | 12918.92 (4176.10 to 45628.83) | 4850.02 (1567.13 to 17128.98) | 1092.82 (352.60 to 3859.21) | 209.57 (67.78 to 740.14) |
| Timor-Leste | Both | Number of DALYs | 27.86 (18.69 to 41.75) | 492.86 (330.19 to 685.71) | 430.29 (293.62 to 604.86) | 442.21 (301.35 to 620.72) | 507.29 (346.92 to 713.22) | 530.26 (364.10 to 745.30) | 493.24 (333.76 to 692.96) | 421.83 (287.53 to 604.01) | 354.60 (241.96 to 504.49) | 302.34 (204.14 to 425.60) | 246.04 (166.89 to 347.37) | 185.54 (126.88 to 260.62) | 134.86 (90.41 to 187.67) | 88.33 (60.72 to 123.67) | 49.60 (34.19 to 69.13) | 21.05 (14.58 to 29.22) | 15.07 (10.43 to 20.74) | 8.52 (5.93 to 11.86) | 2.58 (1.81 to 3.54) | 0.55 (0.38 to 0.76) |
| Timor-Leste | Both | Number of prevalence | 8043.35 (469.13 to 22199.61) | 20367.74 (4440.49 to 49991.67) | 21280.20 (4045.67 to 53424.33) | 25592.68 (4338.82 to 65232.48) | 30872.19 (5208.40 to 74026.15) | 31725.28 (5563.79 to 68362.12) | 28299.90 (5231.32 to 57049.49) | 23310.73 (4479.49 to 44826.62) | 19231.82 (3795.25 to 35730.55) | 15965.38 (3246.83 to 28800.35) | 12661.47 (2662.17 to 22221.71) | 9454.05 (2036.66 to 16203.10) | 6789.31 (1503.07 to 11354.66) | 4460.62 (1000.72 to 7371.45) | 2569.44 (574.85 to 4248.77) | 1112.41 (248.72 to 1839.14) | 804.18 (181.46 to 1327.85) | 483.37 (105.74 to 802.39) | 153.65 (33.15 to 255.86) | 34.98 (7.38 to 58.49) |
| Timor-Leste | Female | Number of DALYs | 16.09 (10.51 to 24.02) | 32.94 (21.05 to 48.49) | 40.48 (26.41 to 60.50) | 68.23 (44.87 to 99.31) | 116.24 (75.53 to 173.48) | 146.67 (94.32 to 217.65) | 144.35 (92.93 to 208.57) | 128.68 (82.72 to 191.73) | 115.14 (73.95 to 167.18) | 98.65 (62.83 to 143.05) | 79.68 (50.86 to 116.10) | 63.96 (41.05 to 94.64) | 48.07 (31.11 to 70.04) | 31.55 (20.08 to 46.08) | 18.17 (11.74 to 26.51) | 7.63 (5.04 to 10.88) | 5.16 (3.38 to 7.43) | 3.61 (2.42 to 5.16) | 1.22 (0.83 to 1.76) | 0.30 (0.20 to 0.43) |
| Timor-Leste | Female | Number of prevalence | 3976.81 (254.66 to 10934.55) | 8083.96 (521.18 to 22226.88) | 8577.37 (602.62 to 23470.71) | 10867.64 (922.46 to 29411.53) | 14101.65 (1477.16 to 36254.66) | 14951.65 (1811.43 to 34066.71) | 13185.43 (1763.10 to 27947.13) | 10829.68 (1567.30 to 21794.68) | 9120.93 (1400.07 to 17660.17) | 7437.38 (1201.05 to 13954.59) | 5757.78 (975.05 to 10495.62) | 4424.49 (781.78 to 7853.78) | 3215.40 (593.30 to 5558.64) | 2102.56 (396.71 to 3589.05) | 1236.58 (233.05 to 2110.86) | 532.49 (100.38 to 908.95) | 368.24 (69.38 to 628.59) | 262.84 (49.65 to 448.67) | 91.42 (17.25 to 156.05) | 23.18 (4.37 to 39.56) |
| Timor-Leste | Male | Number of DALYs | 11.77 (6.93 to 17.79) | 459.93 (306.40 to 639.97) | 389.81 (265.96 to 549.95) | 373.98 (251.24 to 523.12) | 391.05 (267.37 to 545.13) | 383.59 (261.87 to 533.33) | 348.89 (239.99 to 487.49) | 293.15 (200.20 to 416.56) | 239.46 (165.87 to 335.67) | 203.68 (139.94 to 288.11) | 166.36 (114.59 to 238.38) | 121.58 (84.93 to 169.68) | 86.79 (59.12 to 120.50) | 56.78 (39.99 to 79.55) | 31.43 (22.19 to 44.15) | 13.42 (9.47 to 18.58) | 9.90 (6.96 to 13.72) | 4.92 (3.50 to 6.76) | 1.36 (0.97 to 1.85) | 0.25 (0.18 to 0.34) |
| Timor-Leste | Male | Number of prevalence | 4066.54 (214.48 to 11265.06) | 12283.78 (3917.86 to 27859.87) | 12702.83 (3428.06 to 29957.10) | 14725.04 (3388.08 to 35811.92) | 16770.54 (3688.07 to 37771.49) | 16773.63 (3747.10 to 34295.41) | 15114.47 (3445.72 to 29102.36) | 12481.04 (2894.34 to 23031.95) | 10110.89 (2389.38 to 18070.38) | 8528.01 (2050.66 to 14845.76) | 6903.69 (1692.60 to 11726.09) | 5029.56 (1255.75 to 8349.32) | 3573.91 (909.91 to 5796.02) | 2358.06 (607.31 to 3782.40) | 1332.86 (342.86 to 2137.91) | 579.92 (149.26 to 930.19) | 435.94 (112.41 to 699.26) | 220.52 (56.95 to 353.72) | 62.23 (16.03 to 99.81) | 11.80 (3.04 to 18.93) |
| Togo | Both | Number of DALYs | 67.70 (40.87 to 101.63) | 240.99 (127.06 to 447.98) | 242.75 (127.18 to 423.66) | 248.16 (139.07 to 436.76) | 245.98 (134.84 to 412.04) | 233.52 (134.07 to 377.81) | 197.27 (112.28 to 322.01) | 170.86 (96.16 to 275.83) | 137.87 (80.40 to 217.31) | 116.55 (72.36 to 188.91) | 96.35 (57.47 to 155.52) | 77.52 (46.87 to 121.72) | 63.19 (39.20 to 98.34) | 48.83 (30.29 to 74.53) | 32.27 (19.30 to 50.61) | 19.28 (11.99 to 29.72) | 9.08 (5.89 to 13.96) | 3.43 (2.16 to 5.13) | 0.80 (0.50 to 1.21) | 0.16 (0.11 to 0.23) |
| Togo | Both | Number of prevalence | 4955.60 (923.85 to 20010.70) | 11662.40 (2477.28 to 45358.15) | 13337.95 (2607.94 to 53048.59) | 14531.41 (2735.41 to 58276.85) | 14677.67 (2739.20 to 59020.82) | 14025.94 (2623.85 to 56397.67) | 11964.41 (2243.43 to 48098.03) | 10183.93 (1943.00 to 40846.17) | 8274.01 (1587.80 to 33163.12) | 6840.14 (1332.68 to 27333.90) | 5514.45 (1103.41 to 21937.63) | 4385.99 (892.42 to 17406.33) | 3475.57 (727.41 to 13720.58) | 2717.07 (569.65 to 10712.63) | 1837.91 (384.70 to 7252.02) | 1130.09 (234.89 to 4466.91) | 562.86 (114.29 to 2236.53) | 225.33 (44.88 to 899.17) | 58.32 (11.07 to 234.99) | 13.32 (2.35 to 54.40) |
| Togo | Female | Number of DALYs | 39.15 (22.50 to 60.88) | 61.91 (35.57 to 95.83) | 53.04 (31.79 to 85.41) | 46.20 (28.14 to 70.77) | 42.57 (24.96 to 64.93) | 37.91 (23.34 to 56.27) | 31.26 (19.26 to 45.28) | 25.50 (15.42 to 38.18) | 20.08 (12.76 to 29.78) | 16.00 (10.20 to 24.45) | 12.10 (7.57 to 17.96) | 9.26 (5.52 to 13.69) | 6.84 (4.31 to 10.15) | 5.25 (3.41 to 7.71) | 3.52 (2.11 to 5.03) | 2.15 (1.35 to 3.20) | 1.12 (0.73 to 1.60) | 0.47 (0.30 to 0.69) | 0.13 (0.08 to 0.18) | 0.03 (0.02 to 0.05) |
| Togo | Female | Number of prevalence | 2515.10 (514.91 to 9980.71) | 5337.36 (895.98 to 21931.95) | 6025.28 (850.44 to 25356.01) | 6661.16 (834.55 to 28432.83) | 6987.64 (819.41 to 30043.07) | 6842.30 (772.75 to 29525.70) | 5951.36 (657.46 to 25735.11) | 5025.52 (545.98 to 21763.97) | 4125.05 (442.40 to 17882.97) | 3338.19 (355.04 to 14484.65) | 2595.37 (273.52 to 11269.55) | 2037.47 (213.31 to 8853.64) | 1546.78 (160.55 to 6725.93) | 1210.48 (125.12 to 5265.25) | 825.69 (85.37 to 3591.76) | 517.82 (53.58 to 2252.40) | 273.66 (28.31 to 1190.36) | 114.87 (11.89 to 499.66) | 32.79 (3.39 to 142.63) | 8.49 (0.88 to 36.91) |
| Togo | Male | Number of DALYs | 28.55 (15.61 to 46.07) | 179.08 (84.11 to 359.02) | 189.71 (88.81 to 362.51) | 201.96 (103.83 to 386.71) | 203.41 (102.14 to 354.66) | 195.61 (104.74 to 335.81) | 166.01 (89.25 to 280.95) | 145.36 (78.73 to 238.15) | 117.79 (65.38 to 190.47) | 100.55 (58.75 to 169.45) | 84.24 (47.98 to 139.44) | 68.26 (39.09 to 110.65) | 56.35 (33.79 to 90.22) | 43.58 (26.26 to 68.02) | 28.75 (16.89 to 45.84) | 17.13 (10.32 to 27.14) | 7.95 (4.99 to 12.55) | 2.97 (1.81 to 4.61) | 0.67 (0.41 to 1.05) | 0.12 (0.08 to 0.19) |
| Togo | Male | Number of prevalence | 2440.50 (407.07 to 10029.98) | 6325.03 (1585.15 to 23097.47) | 7312.68 (1748.78 to 27317.34) | 7870.26 (1872.87 to 29547.96) | 7690.03 (1869.66 to 28745.59) | 7183.64 (1788.96 to 26709.09) | 6013.05 (1532.26 to 22252.78) | 5158.40 (1339.07 to 19007.63) | 4148.96 (1100.91 to 15231.45) | 3501.95 (938.41 to 12813.36) | 2919.08 (791.66 to 10644.82) | 2348.52 (645.06 to 8533.75) | 1928.79 (538.25 to 6981.38) | 1506.59 (423.26 to 5442.54) | 1012.22 (284.42 to 3657.62) | 612.26 (172.11 to 2211.66) | 289.20 (81.25 to 1044.99) | 110.46 (31.09 to 399.13) | 25.53 (7.18 to 92.25) | 4.83 (1.36 to 17.47) |
| Tonga | Both | Number of DALYs | 1.51 (0.90 to 2.30) | 4.66 (2.54 to 8.41) | 5.00 (2.65 to 9.00) | 5.11 (2.73 to 9.13) | 4.37 (2.27 to 7.74) | 3.66 (1.98 to 6.34) | 2.91 (1.59 to 5.03) | 2.37 (1.30 to 4.21) | 2.18 (1.16 to 3.76) | 2.13 (1.17 to 3.75) | 2.04 (1.12 to 3.52) | 1.85 (1.02 to 3.16) | 1.54 (0.85 to 2.59) | 1.15 (0.63 to 1.90) | 0.80 (0.45 to 1.37) | 0.47 (0.25 to 0.82) | 0.23 (0.13 to 0.38) | 0.10 (0.06 to 0.16) | 0.03 (0.02 to 0.04) | 0.00 (0.00 to 0.01) |
| Tonga | Both | Number of prevalence | 69.56 (14.29 to 547.30) | 164.34 (32.06 to 1229.51) | 216.43 (34.48 to 1707.51) | 252.75 (35.17 to 2053.11) | 233.92 (30.23 to 1928.39) | 204.09 (25.31 to 1695.60) | 169.36 (20.45 to 1417.74) | 143.76 (17.00 to 1215.01) | 133.78 (15.70 to 1135.82) | 130.28 (15.33 to 1108.90) | 123.67 (14.58 to 1056.17) | 111.69 (13.22 to 958.22) | 93.32 (11.11 to 805.19) | 70.01 (8.37 to 605.63) | 50.05 (6.00 to 432.82) | 29.85 (3.57 to 258.37) | 15.63 (1.82 to 136.16) | 7.53 (0.81 to 66.63) | 2.45 (0.25 to 21.90) | 0.46 (0.05 to 4.16) |
| Tonga | Female | Number of DALYs | 0.86 (0.47 to 1.33) | 1.33 (0.77 to 2.08) | 1.29 (0.75 to 2.10) | 1.20 (0.72 to 1.91) | 0.96 (0.58 to 1.43) | 0.78 (0.48 to 1.20) | 0.63 (0.38 to 1.00) | 0.53 (0.32 to 0.81) | 0.48 (0.29 to 0.70) | 0.44 (0.27 to 0.66) | 0.40 (0.24 to 0.60) | 0.34 (0.21 to 0.51) | 0.27 (0.17 to 0.41) | 0.20 (0.12 to 0.29) | 0.13 (0.08 to 0.20) | 0.08 (0.05 to 0.12) | 0.04 (0.03 to 0.07) | 0.03 (0.02 to 0.04) | 0.01 (0.01 to 0.01) | 0.00 (0.00 to 0.00) |
| Tonga | Female | Number of prevalence | 34.68 (7.91 to 264.36) | 72.15 (12.35 to 587.82) | 95.51 (12.32 to 815.37) | 111.39 (11.44 to 977.76) | 104.88 (9.27 to 934.21) | 93.46 (7.53 to 839.09) | 81.10 (6.19 to 732.17) | 72.07 (5.29 to 655.44) | 67.10 (4.78 to 613.56) | 63.92 (4.42 to 587.39) | 59.26 (4.02 to 547.86) | 52.06 (3.47 to 484.87) | 42.57 (2.78 to 399.81) | 31.59 (2.04 to 297.85) | 22.45 (1.45 to 211.68) | 13.58 (0.88 to 128.00) | 7.74 (0.50 to 72.97) | 4.44 (0.29 to 41.91) | 1.63 (0.10 to 15.34) | 0.32 (0.02 to 2.97) |
| Tonga | Male | Number of DALYs | 0.65 (0.36 to 1.08) | 3.33 (1.54 to 6.66) | 3.72 (1.68 to 7.41) | 3.91 (1.72 to 7.69) | 3.41 (1.56 to 6.63) | 2.87 (1.38 to 5.38) | 2.28 (1.11 to 4.24) | 1.84 (0.89 to 3.61) | 1.70 (0.80 to 3.22) | 1.69 (0.85 to 3.14) | 1.64 (0.81 to 2.99) | 1.51 (0.78 to 2.77) | 1.27 (0.64 to 2.26) | 0.95 (0.49 to 1.68) | 0.67 (0.34 to 1.21) | 0.39 (0.19 to 0.73) | 0.18 (0.09 to 0.32) | 0.07 (0.04 to 0.12) | 0.02 (0.01 to 0.03) | 0.00 (0.00 to 0.01) |
| Tonga | Male | Number of prevalence | 34.88 (6.12 to 282.93) | 92.19 (18.35 to 641.70) | 120.92 (20.74 to 892.14) | 141.37 (22.22 to 1075.35) | 129.04 (19.57 to 994.18) | 110.63 (16.61 to 856.51) | 88.26 (13.25 to 685.57) | 71.69 (10.81 to 559.57) | 66.69 (10.11 to 522.26) | 66.37 (10.08 to 521.51) | 64.41 (9.82 to 508.31) | 59.63 (9.14 to 473.35) | 50.75 (7.87 to 405.38) | 38.42 (5.98 to 307.78) | 27.61 (4.31 to 221.14) | 16.27 (2.54 to 130.37) | 7.89 (1.23 to 63.19) | 3.09 (0.48 to 24.72) | 0.82 (0.13 to 6.56) | 0.15 (0.02 to 1.19) |
| Uganda | Both | Number of DALYs | 363.72 (227.50 to 553.02) | 1501.41 (827.08 to 2666.60) | 1821.76 (987.29 to 3088.39) | 2235.01 (1318.40 to 3683.63) | 2415.92 (1504.38 to 3672.95) | 2390.59 (1536.92 to 3595.08) | 2036.11 (1301.81 to 2997.61) | 1715.55 (1115.78 to 2475.28) | 1351.27 (890.96 to 1958.26) | 1248.68 (824.88 to 1836.69) | 1096.19 (726.62 to 1580.07) | 896.05 (601.21 to 1282.65) | 744.23 (502.60 to 1062.20) | 587.98 (398.34 to 817.79) | 414.32 (279.41 to 574.90) | 244.51 (167.52 to 340.63) | 111.40 (76.51 to 153.34) | 38.16 (26.46 to 52.11) | 7.91 (5.49 to 10.82) | 1.19 (0.83 to 1.60) |
| Uganda | Both | Number of prevalence | 41076.96 (8025.97 to 119367.97) | 86634.77 (20811.02 to 239447.61) | 106166.08 (25341.99 to 294432.67) | 123746.51 (30401.10 to 342055.05) | 127424.49 (32145.09 to 350396.60) | 120448.13 (31410.71 to 329104.19) | 97466.65 (26338.31 to 264626.98) | 80228.91 (22168.93 to 217124.58) | 62660.77 (17458.87 to 169434.85) | 57013.23 (16109.21 to 153689.32) | 49625.43 (14191.76 to 133435.59) | 40546.62 (11636.88 to 108917.40) | 33866.79 (9753.66 to 90901.06) | 26854.36 (7798.11 to 71951.30) | 19630.37 (5644.30 to 52730.91) | 12041.52 (3420.79 to 32461.01) | 5844.55 (1618.12 to 15836.71) | 2152.41 (579.79 to 5868.30) | 502.08 (128.25 to 1385.44) | 89.86 (21.35 to 251.79) |
| Uganda | Female | Number of DALYs | 212.96 (124.96 to 334.06) | 305.14 (180.59 to 473.69) | 283.39 (169.17 to 424.83) | 277.40 (173.00 to 408.92) | 262.15 (165.66 to 379.62) | 224.20 (139.08 to 339.79) | 170.67 (105.88 to 248.65) | 136.16 (87.99 to 198.63) | 106.43 (67.69 to 151.85) | 93.59 (61.30 to 135.70) | 78.94 (50.30 to 122.42) | 64.29 (40.00 to 93.76) | 53.16 (33.77 to 78.28) | 40.58 (26.61 to 58.92) | 30.26 (20.04 to 43.10) | 18.88 (12.47 to 27.42) | 9.57 (6.29 to 14.12) | 3.64 (2.33 to 5.09) | 0.93 (0.60 to 1.33) | 0.18 (0.12 to 0.26) |
| Uganda | Female | Number of prevalence | 20769.14 (4341.36 to 59830.38) | 39742.18 (7412.50 to 116541.96) | 48054.55 (8211.91 to 142586.15) | 56774.09 (9217.21 to 169566.51) | 59190.17 (9381.42 to 177328.74) | 54515.34 (8533.61 to 163566.28) | 42524.62 (6620.44 to 127683.64) | 34598.08 (5364.24 to 103929.72) | 27195.70 (4200.26 to 81722.12) | 24308.28 (3747.84 to 73065.54) | 20878.98 (3214.46 to 62771.58) | 17049.97 (2622.36 to 51265.31) | 14299.97 (2196.47 to 43001.44) | 11171.91 (1715.98 to 33597.70) | 8397.10 (1289.32 to 25253.03) | 5342.66 (820.33 to 16067.02) | 2747.02 (421.74 to 8261.00) | 1076.95 (165.37 to 3238.62) | 281.08 (43.16 to 845.31) | 57.28 (8.79 to 172.25) |
| Uganda | Male | Number of DALYs | 150.76 (86.72 to 236.38) | 1196.27 (566.06 to 2321.83) | 1538.37 (796.19 to 2717.82) | 1957.61 (1095.41 to 3355.41) | 2153.77 (1305.67 to 3368.25) | 2166.39 (1383.76 to 3299.06) | 1865.43 (1186.97 to 2749.62) | 1579.40 (1005.21 to 2292.87) | 1244.84 (806.28 to 1815.54) | 1155.09 (756.87 to 1719.15) | 1017.25 (662.23 to 1457.51) | 831.76 (553.80 to 1201.71) | 691.07 (464.64 to 992.83) | 547.40 (368.16 to 766.12) | 384.06 (256.87 to 533.88) | 225.63 (153.57 to 316.15) | 101.82 (69.79 to 141.43) | 34.51 (23.62 to 47.41) | 6.98 (4.79 to 9.70) | 1.00 (0.68 to 1.39) |
| Uganda | Male | Number of prevalence | 20307.82 (3717.06 to 59537.59) | 46892.59 (13389.02 to 123060.37) | 58111.54 (17092.35 to 152039.16) | 66972.42 (21083.55 to 172721.76) | 68234.33 (23068.64 to 173309.90) | 65932.78 (23520.42 to 165768.18) | 54942.03 (20200.54 to 137120.63) | 45630.83 (17048.90 to 113337.97) | 35465.07 (13370.94 to 87826.37) | 32704.95 (12461.00 to 80726.14) | 28746.45 (11021.45 to 70754.73) | 23496.65 (9057.68 to 57725.41) | 19566.82 (7595.44 to 47960.25) | 15682.45 (6098.85 to 38402.91) | 11233.27 (4369.85 to 27513.70) | 6698.86 (2610.38 to 16416.88) | 3097.52 (1203.81 to 7587.40) | 1075.46 (417.85 to 2634.35) | 221.00 (85.89 to 541.34) | 32.58 (12.67 to 79.78) |
| United Republic of Tanzania | Both | Number of DALYs | 624.01 (397.20 to 911.19) | 6515.88 (3973.06 to 9913.60) | 9814.12 (6471.59 to 14060.21) | 11099.08 (7437.24 to 15474.61) | 9602.85 (6392.27 to 13369.46) | 8133.83 (5577.29 to 11267.26) | 6708.16 (4597.76 to 9216.39) | 5754.15 (3954.78 to 7983.03) | 4109.89 (2809.11 to 5738.09) | 3642.85 (2516.46 to 5041.49) | 3203.40 (2214.83 to 4426.91) | 2728.40 (1882.95 to 3719.13) | 2233.78 (1540.60 to 3044.28) | 1770.94 (1235.75 to 2441.39) | 1239.28 (872.04 to 1701.70) | 731.22 (510.43 to 989.57) | 287.68 (200.93 to 384.98) | 91.11 (64.84 to 121.33) | 19.96 (14.40 to 26.29) | 2.81 (2.01 to 3.67) |
| United Republic of Tanzania | Both | Number of prevalence | 133570.82 (65522.61 to 227558.90) | 326875.59 (177636.33 to 531586.26) | 428925.27 (239390.16 to 693755.55) | 488362.04 (271207.40 to 787895.34) | 469383.87 (257227.70 to 760971.88) | 431054.71 (233990.00 to 699954.18) | 365529.97 (198588.13 to 593320.46) | 319095.68 (173367.88 to 517598.67) | 237934.48 (128777.97 to 386422.37) | 215155.42 (116365.65 to 349503.76) | 192394.78 (104104.53 to 312583.58) | 166026.01 (89838.68 to 269570.85) | 138440.24 (74943.68 to 224755.49) | 111034.02 (60145.25 to 180172.38) | 79430.88 (43026.79 to 128926.36) | 48824.74 (26389.35 to 79348.11) | 20510.15 (11026.70 to 33413.95) | 7038.15 (3756.39 to 11506.42) | 1690.29 (895.49 to 2773.99) | 279.19 (145.85 to 461.39) |
| United Republic of Tanzania | Female | Number of DALYs | 362.97 (222.81 to 557.95) | 623.18 (392.52 to 917.17) | 723.45 (454.48 to 1085.02) | 825.77 (530.13 to 1192.30) | 883.43 (577.40 to 1343.77) | 871.57 (594.45 to 1283.92) | 754.59 (497.35 to 1106.37) | 663.23 (444.10 to 969.56) | 529.18 (358.75 to 763.94) | 490.55 (320.81 to 720.45) | 452.03 (294.63 to 658.45) | 396.39 (264.39 to 566.96) | 342.33 (223.01 to 492.40) | 271.04 (180.70 to 392.91) | 192.62 (129.70 to 282.07) | 119.20 (80.27 to 171.74) | 52.15 (35.28 to 73.60) | 19.21 (13.27 to 26.99) | 4.92 (3.44 to 6.79) | 0.91 (0.62 to 1.28) |
| United Republic of Tanzania | Female | Number of prevalence | 66625.59 (33010.69 to 113200.59) | 141124.10 (69185.09 to 240718.95) | 180805.30 (88106.02 to 308959.23) | 212992.36 (103730.80 to 363964.38) | 219256.46 (106998.67 to 374251.20) | 206685.84 (101078.48 to 352395.65) | 171402.75 (84032.85 to 291921.18) | 145842.57 (71625.28 to 248145.67) | 111683.34 (54961.55 to 189860.63) | 100591.77 (49588.02 to 170852.95) | 89459.47 (44171.29 to 151836.32) | 76164.17 (37682.77 to 129128.20) | 63097.23 (31309.03 to 106861.53) | 49662.66 (24648.20 to 84053.69) | 35872.45 (17813.48 to 60716.32) | 22779.71 (11310.92 to 38559.92) | 10238.94 (5083.63 to 17328.59) | 3827.49 (1899.98 to 6478.28) | 1005.10 (499.06 to 1701.22) | 190.99 (94.84 to 323.26) |
| United Republic of Tanzania | Male | Number of DALYs | 261.04 (150.36 to 398.71) | 5892.70 (3568.29 to 9225.91) | 9090.68 (5875.13 to 13218.01) | 10273.32 (6851.37 to 14315.57) | 8719.42 (5783.92 to 12068.52) | 7262.26 (4974.93 to 10169.38) | 5953.58 (4059.07 to 8206.84) | 5090.92 (3451.10 to 7008.33) | 3580.71 (2474.09 to 5019.14) | 3152.30 (2153.10 to 4399.07) | 2751.37 (1878.75 to 3837.62) | 2332.01 (1615.42 to 3178.05) | 1891.44 (1290.48 to 2603.68) | 1499.90 (1038.75 to 2063.55) | 1046.65 (729.64 to 1438.97) | 612.02 (418.64 to 820.07) | 235.53 (165.84 to 315.65) | 71.91 (50.41 to 96.43) | 15.03 (10.78 to 19.75) | 1.90 (1.34 to 2.51) |
| United Republic of Tanzania | Male | Number of prevalence | 66945.23 (32487.99 to 114358.32) | 185751.49 (110360.91 to 289940.77) | 248119.97 (152188.91 to 381385.80) | 275369.69 (171561.11 to 420962.97) | 250127.42 (152396.26 to 384697.59) | 224368.87 (133999.30 to 346864.81) | 194127.21 (115004.44 to 301058.89) | 173253.11 (101964.87 to 269512.95) | 126251.14 (73897.75 to 196893.28) | 114563.65 (66815.95 to 178995.74) | 102935.31 (59816.02 to 160998.65) | 89861.84 (52061.83 to 140688.77) | 75343.01 (43564.38 to 118127.66) | 61371.36 (35438.98 to 96247.34) | 43558.44 (25155.17 to 68324.69) | 26045.03 (15042.88 to 40848.42) | 10271.21 (5930.17 to 16113.00) | 3210.66 (1852.08 to 5035.70) | 685.19 (395.51 to 1074.54) | 88.19 (50.89 to 138.36) |
| Vanuatu | Both | Number of DALYs | 4.56 (2.88 to 6.61) | 76.47 (50.49 to 110.09) | 87.67 (58.71 to 123.07) | 77.22 (52.80 to 109.23) | 70.19 (47.96 to 97.00) | 64.57 (44.70 to 89.31) | 54.83 (37.30 to 75.84) | 49.48 (33.92 to 69.03) | 39.63 (27.05 to 54.80) | 32.80 (22.24 to 46.27) | 25.27 (17.27 to 35.37) | 19.47 (13.41 to 27.20) | 16.92 (11.76 to 23.16) | 13.26 (9.22 to 18.36) | 7.85 (5.48 to 10.83) | 4.16 (2.89 to 5.74) | 1.65 (1.16 to 2.23) | 0.53 (0.37 to 0.71) | 0.11 (0.08 to 0.15) | 0.02 (0.01 to 0.02) |
| Vanuatu | Both | Number of prevalence | 1208.30 (44.83 to 4990.73) | 3109.04 (604.91 to 11101.61) | 3750.00 (715.26 to 13547.95) | 4023.01 (641.81 to 14894.76) | 4012.23 (582.25 to 13251.21) | 3849.29 (541.99 to 11823.15) | 3324.91 (466.59 to 9774.30) | 2945.51 (422.46 to 8373.12) | 2318.83 (342.56 to 6410.80) | 1930.30 (285.13 to 5234.98) | 1482.04 (222.36 to 3944.74) | 1158.16 (173.70 to 3031.76) | 1011.41 (152.75 to 2598.56) | 799.19 (121.47 to 2034.83) | 484.56 (73.31 to 1234.68) | 264.49 (39.67 to 674.84) | 109.10 (16.03 to 279.06) | 34.97 (5.26 to 89.18) | 7.89 (1.14 to 20.23) | 1.37 (0.18 to 3.54) |
| Vanuatu | Female | Number of DALYs | 2.58 (1.57 to 3.79) | 4.91 (3.09 to 7.36) | 6.25 (4.02 to 9.23) | 8.27 (5.32 to 12.14) | 10.22 (6.76 to 15.05) | 11.04 (7.17 to 15.84) | 10.13 (6.48 to 14.29) | 8.89 (5.79 to 13.30) | 6.80 (4.45 to 9.88) | 5.80 (3.63 to 8.25) | 4.40 (2.86 to 6.34) | 3.52 (2.31 to 5.20) | 3.11 (2.02 to 4.57) | 2.42 (1.59 to 3.55) | 1.46 (0.96 to 2.11) | 0.80 (0.53 to 1.15) | 0.34 (0.23 to 0.49) | 0.10 (0.07 to 0.15) | 0.02 (0.02 to 0.04) | 0.00 (0.00 to 0.01) |
| Vanuatu | Female | Number of prevalence | 583.51 (25.20 to 2398.09) | 1226.40 (48.79 to 5055.60) | 1506.72 (61.56 to 6204.49) | 1742.88 (81.37 to 7103.65) | 1853.79 (98.94 to 6736.21) | 1834.14 (106.12 to 6119.60) | 1594.55 (97.49 to 5059.17) | 1351.16 (85.52 to 4142.58) | 1005.90 (65.86 to 3001.32) | 833.22 (55.93 to 2434.16) | 623.50 (42.78 to 1786.51) | 490.24 (34.53 to 1377.97) | 424.37 (30.66 to 1168.53) | 329.40 (24.09 to 898.80) | 202.74 (14.88 to 553.18) | 113.60 (8.30 to 309.97) | 49.10 (3.59 to 133.96) | 14.85 (1.09 to 40.52) | 3.68 (0.27 to 10.05) | 0.75 (0.05 to 2.04) |
| Vanuatu | Male | Number of DALYs | 1.98 (1.19 to 2.98) | 71.56 (47.30 to 103.57) | 81.42 (54.29 to 114.37) | 68.95 (46.82 to 98.30) | 59.98 (40.44 to 84.31) | 53.53 (36.80 to 74.07) | 44.70 (30.44 to 62.00) | 40.59 (27.69 to 57.30) | 32.83 (22.28 to 45.52) | 27.01 (18.15 to 37.91) | 20.86 (14.29 to 29.26) | 15.94 (10.86 to 22.15) | 13.81 (9.54 to 18.70) | 10.84 (7.41 to 14.92) | 6.39 (4.41 to 8.75) | 3.36 (2.31 to 4.56) | 1.31 (0.91 to 1.74) | 0.43 (0.30 to 0.57) | 0.09 (0.06 to 0.12) | 0.01 (0.01 to 0.02) |
| Vanuatu | Male | Number of prevalence | 624.78 (19.55 to 2592.64) | 1882.64 (549.53 to 6067.48) | 2243.29 (648.07 to 7343.46) | 2280.13 (559.63 to 7791.10) | 2158.43 (480.92 to 6515.00) | 2015.14 (433.38 to 5703.54) | 1730.36 (366.47 to 4715.13) | 1594.36 (332.78 to 4230.55) | 1312.94 (271.18 to 3409.48) | 1097.09 (224.97 to 2800.82) | 858.54 (174.55 to 2158.24) | 667.92 (135.74 to 1653.79) | 587.03 (118.59 to 1430.03) | 469.78 (94.88 to 1136.03) | 281.82 (57.02 to 681.50) | 150.88 (30.44 to 364.87) | 60.01 (12.15 to 145.10) | 20.12 (4.06 to 48.66) | 4.21 (0.85 to 10.18) | 0.62 (0.12 to 1.50) |
| Viet Nam | Both | Number of DALYs | 1099.66 (703.72 to 1653.40) | 6905.58 (3815.36 to 11836.93) | 9596.14 (5514.27 to 15943.01) | 13002.09 (8238.26 to 19647.93) | 15206.31 (9827.89 to 22225.02) | 16961.88 (11322.96 to 24450.47) | 15853.47 (10410.29 to 22665.51) | 12456.69 (8128.35 to 17619.16) | 7726.55 (5156.44 to 10972.93) | 6850.26 (4587.05 to 9569.65) | 6781.11 (4591.37 to 9576.63) | 7187.30 (4701.05 to 9921.28) | 6070.27 (4113.55 to 8314.47) | 4497.87 (3040.38 to 6161.20) | 2818.93 (1921.19 to 3833.15) | 1774.96 (1215.44 to 2446.25) | 952.22 (659.82 to 1282.43) | 369.22 (259.52 to 497.74) | 109.01 (76.97 to 145.92) | 26.46 (19.12 to 35.04) |
| Viet Nam | Both | Number of prevalence | 152037.81 (23972.95 to 488368.08) | 395144.64 (82849.02 to 1188071.59) | 510493.00 (112363.72 to 1524964.62) | 632189.73 (150847.57 to 1872417.77) | 698796.95 (176201.82 to 2059711.03) | 754561.19 (197205.09 to 2215693.47) | 695965.43 (186429.74 to 2036152.57) | 554647.36 (147503.75 to 1624791.63) | 344433.69 (91800.29 to 1007913.07) | 314423.49 (82584.51 to 923408.43) | 318169.29 (83018.69 to 936032.72) | 335592.94 (88577.09 to 984673.41) | 290849.42 (75814.56 to 854828.55) | 229311.69 (57823.46 to 678330.93) | 151931.93 (37402.99 to 451633.42) | 102144.90 (24463.28 to 305407.36) | 60127.80 (13745.98 to 181397.06) | 27261.01 (5766.01 to 83419.77) | 9593.28 (1862.34 to 29770.35) | 3068.72 (532.91 to 9701.06) |
| Viet Nam | Female | Number of DALYs | 629.80 (380.66 to 994.11) | 1133.37 (704.59 to 1710.06) | 1130.34 (650.23 to 1741.07) | 1207.43 (733.12 to 1812.10) | 1267.03 (819.83 to 1883.44) | 1331.49 (840.43 to 1953.13) | 1179.98 (773.75 to 1704.14) | 972.16 (619.72 to 1420.34) | 591.45 (373.60 to 881.65) | 558.06 (355.34 to 796.54) | 575.63 (365.42 to 872.71) | 588.38 (379.00 to 883.78) | 511.64 (343.66 to 752.90) | 421.48 (281.67 to 601.07) | 287.86 (185.65 to 426.28) | 197.26 (129.43 to 282.02) | 122.67 (80.00 to 176.77) | 61.12 (41.99 to 87.29) | 22.92 (15.61 to 32.01) | 8.03 (5.44 to 11.29) |
| Viet Nam | Female | Number of prevalence | 75075.37 (12528.63 to 239219.35) | 175257.61 (26217.25 to 566448.11) | 220767.40 (30521.41 to 720111.49) | 275378.04 (36510.29 to 902678.54) | 309262.83 (40260.17 to 1015742.36) | 332536.96 (42942.43 to 1093085.55) | 303909.82 (39128.16 to 999323.83) | 248694.43 (32007.94 to 817861.63) | 154014.23 (19814.26 to 506535.93) | 145943.07 (18803.09 to 479990.94) | 150172.66 (19338.72 to 493895.76) | 153661.14 (19812.77 to 505312.36) | 134851.69 (17434.19 to 443402.48) | 112447.64 (14533.56 to 369716.99) | 78051.90 (10103.59 to 256627.89) | 55309.05 (7145.20 to 181840.80) | 35046.71 (4538.25 to 115228.22) | 17762.62 (2298.28 to 58398.68) | 6884.39 (891.17 to 22633.69) | 2475.86 (320.54 to 8139.71) |
| Viet Nam | Male | Number of DALYs | 469.87 (255.14 to 733.75) | 5772.21 (2961.90 to 10372.71) | 8465.80 (4755.47 to 14480.68) | 11794.67 (7332.73 to 18025.53) | 13939.28 (8949.68 to 20291.26) | 15630.39 (10263.21 to 22873.83) | 14673.49 (9640.38 to 21148.88) | 11484.53 (7484.02 to 16263.76) | 7135.11 (4755.87 to 10176.71) | 6292.20 (4202.52 to 8823.78) | 6205.48 (4200.43 to 8797.98) | 6598.91 (4322.22 to 9096.42) | 5558.63 (3741.06 to 7645.19) | 4076.39 (2744.85 to 5516.87) | 2531.07 (1722.20 to 3447.45) | 1577.70 (1073.49 to 2175.27) | 829.55 (571.95 to 1123.03) | 308.10 (211.81 to 417.17) | 86.09 (59.05 to 114.53) | 18.43 (12.98 to 24.85) |
| Viet Nam | Male | Number of prevalence | 76962.44 (11414.61 to 249148.73) | 219887.04 (56488.77 to 623098.99) | 289725.60 (81483.58 to 805664.39) | 356811.69 (114166.78 to 970241.11) | 389534.12 (135066.88 to 1044447.48) | 422024.23 (153210.01 to 1124206.60) | 392055.61 (143739.55 to 1039797.18) | 305952.93 (113350.73 to 809393.73) | 190419.46 (70943.49 to 502925.20) | 168480.41 (62949.47 to 444887.63) | 167996.63 (62897.00 to 443663.42) | 181931.80 (67466.91 to 480937.21) | 155997.72 (57495.49 to 412776.54) | 116864.05 (42831.67 to 309573.68) | 73880.03 (27120.30 to 195697.85) | 46835.85 (17201.74 to 124033.69) | 25081.09 (9195.30 to 66453.23) | 9498.39 (3488.50 to 25149.79) | 2708.88 (994.54 to 7175.40) | 592.86 (217.95 to 1569.90) |
| Yemen | Both | Number of DALYs | 255.69 (155.03 to 376.41) | 677.15 (354.70 to 1264.95) | 558.77 (302.03 to 1035.06) | 394.03 (201.77 to 694.21) | 302.01 (161.56 to 549.65) | 264.99 (148.78 to 481.61) | 240.76 (129.20 to 425.26) | 198.78 (107.31 to 367.80) | 152.67 (83.30 to 273.65) | 123.15 (67.21 to 221.21) | 97.21 (51.56 to 176.52) | 82.28 (43.08 to 152.69) | 68.26 (38.00 to 129.24) | 48.69 (26.10 to 85.69) | 29.90 (16.46 to 52.60) | 15.85 (8.92 to 26.31) | 6.88 (3.99 to 11.42) | 2.32 (1.37 to 3.57) | 0.58 (0.35 to 0.89) | 0.12 (0.07 to 0.17) |
| Yemen | Both | Number of prevalence | 5422.86 (2642.49 to 16672.01) | 12575.11 (5588.34 to 36946.13) | 12473.47 (4750.60 to 40767.82) | 10505.11 (3506.06 to 37062.33) | 9115.91 (2742.54 to 33698.10) | 8835.04 (2514.54 to 33523.61) | 8478.62 (2327.98 to 32599.80) | 7290.32 (1949.52 to 28262.42) | 5560.84 (1458.61 to 21585.44) | 4638.04 (1195.90 to 18133.72) | 3755.50 (956.12 to 14741.46) | 3253.23 (815.81 to 12827.97) | 2759.81 (681.58 to 10931.72) | 2099.82 (513.55 to 8384.11) | 1354.24 (329.08 to 5441.74) | 751.50 (180.01 to 3039.15) | 349.33 (83.20 to 1423.39) | 126.45 (29.48 to 519.40) | 34.01 (7.82 to 140.72) | 7.41 (1.64 to 30.95) |
| Yemen | Female | Number of DALYs | 146.35 (81.54 to 224.22) | 218.29 (124.48 to 347.92) | 169.28 (96.90 to 262.43) | 119.63 (68.62 to 184.47) | 96.51 (56.00 to 153.92) | 87.62 (52.88 to 131.46) | 77.62 (46.15 to 117.12) | 61.98 (36.24 to 93.93) | 41.43 (24.93 to 62.88) | 34.34 (19.71 to 52.09) | 26.08 (15.28 to 38.72) | 21.75 (12.82 to 33.35) | 17.77 (10.53 to 27.52) | 14.34 (8.57 to 22.49) | 10.13 (6.30 to 15.65) | 6.13 (3.40 to 9.27) | 3.02 (1.78 to 4.59) | 1.19 (0.70 to 1.79) | 0.34 (0.21 to 0.50) | 0.08 (0.05 to 0.11) |
| Yemen | Female | Number of prevalence | 2849.90 (1490.85 to 8312.32) | 5216.58 (2274.86 to 17134.44) | 5161.42 (1840.06 to 18644.07) | 4508.86 (1349.52 to 17377.54) | 4194.78 (1112.23 to 16766.87) | 4269.81 (1049.37 to 17411.26) | 4085.61 (957.56 to 16856.99) | 3465.99 (782.03 to 14417.52) | 2399.69 (526.19 to 10046.01) | 2045.19 (436.55 to 8606.51) | 1625.86 (340.08 to 6872.45) | 1400.62 (287.14 to 5946.36) | 1170.81 (234.97 to 4993.53) | 980.21 (194.11 to 4189.20) | 697.31 (138.04 to 2980.28) | 423.82 (84.16 to 1811.30) | 217.11 (43.10 to 927.97) | 86.46 (17.13 to 369.53) | 25.19 (5.00 to 107.66) | 6.01 (1.19 to 25.69) |
| Yemen | Male | Number of DALYs | 109.34 (62.55 to 173.65) | 458.86 (198.82 to 973.14) | 389.49 (166.49 to 843.27) | 274.40 (122.16 to 575.06) | 205.50 (95.09 to 429.09) | 177.37 (79.60 to 360.22) | 163.14 (73.99 to 344.07) | 136.81 (63.12 to 290.23) | 111.25 (49.92 to 229.92) | 88.80 (40.33 to 181.54) | 71.13 (31.41 to 145.88) | 60.52 (27.03 to 122.92) | 50.50 (24.07 to 105.29) | 34.35 (15.15 to 68.80) | 19.77 (9.13 to 40.52) | 9.72 (4.43 to 19.16) | 3.86 (1.86 to 7.47) | 1.13 (0.53 to 2.23) | 0.24 (0.11 to 0.50) | 0.04 (0.02 to 0.08) |
| Yemen | Male | Number of prevalence | 2572.96 (1127.10 to 8364.84) | 7358.53 (3120.58 to 20089.86) | 7312.05 (2772.92 to 22163.62) | 5996.25 (2020.38 to 19684.80) | 4921.13 (1533.08 to 16931.23) | 4565.23 (1356.86 to 16112.35) | 4393.01 (1266.58 to 15742.81) | 3824.34 (1079.48 to 13844.90) | 3161.15 (876.97 to 11539.43) | 2592.85 (706.69 to 9527.21) | 2129.64 (574.95 to 7869.00) | 1852.61 (493.80 to 6881.62) | 1589.00 (416.91 to 5938.19) | 1119.60 (292.10 to 4194.91) | 656.93 (171.25 to 2461.46) | 327.69 (85.41 to 1227.84) | 132.21 (34.52 to 495.42) | 39.99 (10.42 to 149.87) | 8.82 (2.30 to 33.05) | 1.40 (0.37 to 5.26) |
| Zambia | Both | Number of DALYs | 190.40 (119.85 to 276.03) | 1882.12 (1180.95 to 2886.68) | 2955.61 (1955.20 to 4260.27) | 3634.75 (2422.79 to 5083.15) | 3208.74 (2166.71 to 4455.29) | 2539.45 (1722.94 to 3541.40) | 2021.37 (1363.57 to 2791.92) | 1574.92 (1075.49 to 2164.34) | 1288.08 (885.59 to 1773.79) | 1097.16 (756.60 to 1514.68) | 928.78 (637.70 to 1284.46) | 771.74 (532.22 to 1053.38) | 593.10 (415.51 to 812.64) | 430.31 (302.88 to 593.25) | 292.42 (202.40 to 402.29) | 191.47 (133.16 to 258.62) | 88.54 (61.92 to 118.94) | 32.02 (22.84 to 42.45) | 6.08 (4.38 to 8.12) | 0.81 (0.58 to 1.07) |
| Zambia | Both | Number of prevalence | 40065.06 (15688.31 to 84442.72) | 95343.89 (43871.22 to 188682.12) | 131442.84 (61618.79 to 255794.40) | 157525.08 (74378.32 to 305528.13) | 150887.76 (71142.89 to 295102.88) | 127866.17 (59708.80 to 251774.47) | 105551.68 (48949.03 to 208429.26) | 84691.46 (39117.60 to 167609.92) | 72278.78 (33169.96 to 143504.04) | 63430.80 (29000.53 to 126158.74) | 54269.28 (24796.11 to 107945.76) | 45184.43 (20657.53 to 89833.39) | 35017.54 (16034.53 to 69573.30) | 25634.85 (11767.20 to 50885.94) | 17276.13 (7972.01 to 34225.39) | 11350.30 (5252.81 to 22453.74) | 5610.01 (2576.34 to 11137.69) | 2179.86 (990.29 to 4344.46) | 436.13 (197.10 to 871.33) | 65.79 (29.16 to 132.47) |
| Zambia | Female | Number of DALYs | 111.54 (67.14 to 163.93) | 189.31 (120.00 to 289.08) | 224.22 (144.72 to 331.22) | 265.81 (165.74 to 391.69) | 273.01 (174.88 to 400.62) | 248.68 (167.09 to 350.86) | 207.78 (135.64 to 302.14) | 171.86 (115.45 to 252.77) | 154.95 (102.10 to 229.21) | 140.82 (95.80 to 205.35) | 121.80 (80.94 to 176.94) | 100.24 (66.07 to 145.18) | 77.25 (50.90 to 110.47) | 55.29 (37.14 to 79.72) | 34.94 (23.10 to 49.79) | 21.79 (14.70 to 31.45) | 11.49 (7.87 to 15.90) | 4.83 (3.25 to 6.90) | 1.00 (0.69 to 1.42) | 0.17 (0.12 to 0.24) |
| Zambia | Female | Number of prevalence | 20189.80 (8033.47 to 42396.76) | 41807.26 (16342.51 to 88270.47) | 56019.47 (21689.41 to 118593.38) | 68214.16 (26353.89 to 144461.61) | 68203.19 (26406.49 to 144314.35) | 58689.42 (22790.43 to 124046.28) | 47550.77 (18523.99 to 100398.50) | 37929.92 (14811.03 to 80000.39) | 33147.48 (12976.59 to 69846.33) | 29338.82 (11515.92 to 61773.09) | 24715.08 (9723.41 to 51988.88) | 19853.50 (7832.24 to 41722.16) | 14853.03 (5874.98 to 31174.44) | 10549.68 (4182.96 to 22132.24) | 6740.67 (2673.17 to 14138.60) | 4265.01 (1690.47 to 8945.95) | 2313.82 (917.45 to 4853.87) | 988.71 (391.99 to 2074.09) | 208.38 (82.61 to 437.12) | 36.81 (14.59 to 77.22) |
| Zambia | Male | Number of DALYs | 78.86 (45.92 to 123.69) | 1692.80 (1028.01 to 2615.30) | 2731.38 (1797.81 to 3930.52) | 3368.94 (2207.36 to 4708.11) | 2935.72 (1958.65 to 4088.76) | 2290.77 (1547.34 to 3177.62) | 1813.59 (1227.15 to 2553.46) | 1403.07 (958.09 to 1935.84) | 1133.13 (780.73 to 1571.94) | 956.34 (656.08 to 1309.81) | 806.99 (547.43 to 1119.27) | 671.50 (462.72 to 914.17) | 515.84 (359.45 to 709.77) | 375.02 (261.70 to 513.95) | 257.48 (176.59 to 354.20) | 169.67 (118.05 to 230.66) | 77.04 (53.69 to 103.58) | 27.19 (19.23 to 36.21) | 5.09 (3.66 to 6.87) | 0.64 (0.45 to 0.84) |
| Zambia | Male | Number of prevalence | 19875.26 (7654.84 to 42045.96) | 53536.63 (27682.81 to 99331.74) | 75423.37 (40816.53 to 136878.07) | 89310.91 (49220.44 to 161227.04) | 82684.57 (44470.39 to 150788.53) | 69176.74 (36795.49 to 127728.18) | 58000.90 (30470.84 to 108030.76) | 46761.54 (24342.54 to 87609.52) | 39131.30 (20241.81 to 73657.71) | 34091.98 (17545.72 to 64385.65) | 29554.20 (15127.98 to 55956.89) | 25330.93 (12877.14 to 48111.23) | 20164.52 (10193.38 to 38398.86) | 15085.17 (7612.40 to 28753.69) | 10535.46 (5321.57 to 20086.79) | 7085.29 (3576.47 to 13507.80) | 3296.19 (1665.88 to 6283.82) | 1191.15 (601.33 to 2270.37) | 227.75 (114.97 to 434.21) | 28.98 (14.63 to 55.26) |
| Zimbabwe | Both | Number of DALYs | 169.68 (103.00 to 268.89) | 489.05 (266.91 to 886.91) | 465.82 (250.36 to 819.87) | 424.13 (226.43 to 755.18) | 349.04 (189.70 to 629.29) | 278.54 (148.01 to 495.47) | 230.95 (123.20 to 422.15) | 199.50 (106.95 to 352.68) | 149.35 (82.15 to 262.99) | 125.38 (67.20 to 221.69) | 111.81 (61.40 to 204.27) | 88.45 (45.83 to 164.58) | 74.84 (41.21 to 133.45) | 60.74 (34.03 to 106.04) | 41.42 (21.89 to 72.26) | 20.65 (11.46 to 35.62) | 8.45 (4.90 to 14.47) | 3.19 (1.88 to 5.21) | 0.79 (0.47 to 1.21) | 0.15 (0.09 to 0.23) |
| Zimbabwe | Both | Number of prevalence | 5633.57 (2061.10 to 18932.75) | 13584.92 (4797.92 to 44349.40) | 16040.39 (4923.77 to 55773.52) | 17309.95 (4774.72 to 62629.98) | 16183.41 (4186.32 to 59862.96) | 13931.97 (3442.95 to 52157.83) | 12086.63 (2928.06 to 45504.97) | 10590.59 (2530.39 to 39998.35) | 8035.79 (1901.19 to 30430.64) | 6880.96 (1627.59 to 26067.66) | 6203.36 (1457.85 to 23535.80) | 4855.70 (1149.49 to 18401.18) | 4192.43 (989.76 to 15911.35) | 3567.65 (825.18 to 13604.12) | 2498.49 (575.07 to 9538.58) | 1322.54 (301.99 to 5073.22) | 599.87 (134.41 to 2321.44) | 249.93 (55.03 to 973.79) | 67.66 (14.49 to 265.76) | 14.64 (3.04 to 57.95) |
| Zimbabwe | Female | Number of DALYs | 98.86 (59.09 to 153.84) | 159.49 (96.06 to 246.67) | 141.89 (83.68 to 217.74) | 124.60 (73.06 to 191.60) | 100.93 (59.82 to 152.29) | 80.70 (46.66 to 123.03) | 64.03 (35.67 to 98.39) | 52.96 (32.33 to 81.93) | 38.53 (23.30 to 57.52) | 30.35 (17.73 to 46.88) | 26.48 (15.35 to 40.95) | 19.08 (11.94 to 28.87) | 15.77 (9.66 to 24.33) | 14.01 (8.21 to 20.87) | 9.97 (6.03 to 14.71) | 5.63 (3.48 to 8.71) | 2.83 (1.68 to 4.33) | 1.26 (0.73 to 1.87) | 0.38 (0.23 to 0.58) | 0.09 (0.05 to 0.13) |
| Zimbabwe | Female | Number of prevalence | 2948.74 (1162.39 to 9594.25) | 6160.55 (2017.45 to 21612.87) | 7370.52 (2020.57 to 27336.19) | 8097.67 (1934.74 to 31106.60) | 7832.51 (1710.82 to 30657.23) | 6927.81 (1435.40 to 27392.88) | 5979.14 (1200.10 to 23782.35) | 5146.56 (1008.45 to 20558.06) | 3885.34 (747.37 to 15567.19) | 3206.67 (607.27 to 12880.38) | 2859.58 (533.31 to 11512.39) | 2114.22 (389.32 to 8529.34) | 1808.32 (329.22 to 7312.14) | 1655.53 (299.36 to 6701.18) | 1187.20 (214.58 to 4805.68) | 683.07 (123.58 to 2764.76) | 356.50 (64.53 to 1442.94) | 163.60 (29.60 to 662.20) | 49.15 (8.87 to 198.93) | 11.62 (2.10 to 47.05) |
| Zimbabwe | Male | Number of DALYs | 70.82 (36.25 to 117.20) | 329.56 (140.46 to 724.67) | 323.92 (144.28 to 644.09) | 299.53 (132.18 to 608.46) | 248.11 (112.38 to 486.89) | 197.84 (83.36 to 399.98) | 166.92 (75.65 to 330.31) | 146.54 (68.21 to 279.64) | 110.82 (50.30 to 215.12) | 95.02 (45.01 to 182.94) | 85.33 (40.58 to 169.17) | 69.37 (31.96 to 135.94) | 59.07 (28.56 to 116.08) | 46.73 (23.13 to 88.93) | 31.45 (14.73 to 60.48) | 15.02 (7.43 to 28.76) | 5.62 (2.67 to 10.79) | 1.93 (0.88 to 3.67) | 0.41 (0.20 to 0.77) | 0.07 (0.03 to 0.13) |
| Zimbabwe | Male | Number of prevalence | 2684.84 (887.89 to 9338.49) | 7424.37 (2596.55 to 22502.14) | 8669.87 (2718.01 to 28267.79) | 9212.29 (2677.96 to 31433.92) | 8350.91 (2319.36 to 29164.45) | 7004.16 (1903.06 to 24750.18) | 6107.48 (1636.08 to 21716.99) | 5444.03 (1446.49 to 19440.26) | 4150.45 (1100.37 to 14863.45) | 3674.29 (968.78 to 13187.29) | 3343.78 (878.01 to 12023.42) | 2741.49 (719.28 to 9871.84) | 2384.11 (623.03 to 8599.21) | 1912.12 (499.55 to 6902.95) | 1311.29 (342.10 to 4732.90) | 639.47 (166.86 to 2308.46) | 243.37 (63.58 to 878.50) | 86.33 (22.52 to 311.60) | 18.52 (4.83 to 66.83) | 3.02 (0.79 to 10.90) |
| **Abbreviations:** GBD, Global Burden of Disease, DALYs, disability-adjusted life years; SDI, socio-demographic index; UI, uncertainty interval. | | | | | | | | | | | | | | | | | | | | | | |
