# Supplementary material for: Global, regional, and national disability-adjusted life years and prevalence of lymphatic filariasis from 1990 to 2021: A trend and health inequality analysis based on the global burden of disease study 2021
Source: PLoS Negl Trop Dis. 2025 Apr 29;19(4):e0013017. doi: 10.1371/journal.pntd.0013017 (PMC12040265; doi:10.1371/journal.pntd.0013017)
Supplement: S9 Table — Abbreviations: GBD, Global Burden of Disease, DALYs, disability-adjusted life years; SDI, socio-demographic index; UI, uncertainty interval. (DOCX) [file pntd.0013017.s009.docx]

**S9 Table The number of prevalence and DALYs of lymphatic filariasis, by age group , sex, SDI levels, GBD regions, among 67 countries and territories, in 2021.**

| **Location** | **Sex** | **Metric** | **Age group (95% UI)** | | | | | | | | | | | | | | | | | | | |
| --- | --- | --- | --- | --- | --- | --- | --- | --- | --- | --- | --- | --- | --- | --- | --- | --- | --- | --- | --- | --- | --- | --- |
|  |  |  | **<5 years** | **5-9 years** | **10-14 years** | **15-19 years** | **20-24 years** | **25-29 years** | **30-34 years** | **35-39 years** | **40-44 years** | **45-49 years** | **50-54 years** | **55-59 years** | **60-64 years** | **65-69 years** | **70-74 years** | **75-79 years** | **80-84 years** | **85-89 years** | **90-94 years** | **95+ years** |
| Global | Both | Number of DALYs | 34418.14 (23553.57 to 48541.55) | 106159.05 (61037.94 to 191699.32) | 118217.39 (66898.15 to 211149.90) | 125638.96 (70684.94 to 222371.48) | 128357.15 (73348.21 to 222357.91) | 124167.33 (70785.78 to 212345.91) | 117525.16 (67312.16 to 200567.16) | 108986.85 (63045.76 to 183871.14) | 96971.97 (56710.58 to 161981.02) | 84503.63 (49282.67 to 141532.44) | 71336.87 (42060.86 to 118274.53) | 58551.08 (34498.32 to 96010.77) | 48624.66 (29126.14 to 78591.86) | 37592.53 (22902.29 to 60586.48) | 25719.74 (15647.83 to 41250.14) | 15434.17 (9590.23 to 24760.13) | 8061.18 (5030.22 to 12770.90) | 3204.50 (2002.90 to 5077.43) | 916.47 (581.24 to 1447.54) | 176.58 (113.45 to 274.43) |
| Global | Both | Number of prevalence | 1141805.37 (1006037.36 to 1334305.81) | 2942880.96 (2475530.69 to 3605875.43) | 3961962.11 (3356608.85 to 4750746.37) | 4914545.35 (4190742.42 to 5862740.41) | 5482597.92 (4694805.84 to 6500597.90) | 5518849.24 (4726222.35 to 6534405.70) | 5336610.16 (4558392.22 to 6380319.18) | 5069507.43 (4337049.68 to 6065462.44) | 4570346.38 (3903000.69 to 5471431.60) | 4036023.67 (3436530.54 to 4865423.27) | 3480995.66 (2952453.30 to 4204948.27) | 2935005.41 (2491150.11 to 3586014.95) | 2508628.53 (2133025.48 to 3093417.14) | 1990509.02 (1703828.52 to 2430769.87) | 1400949.94 (1208724.76 to 1680822.58) | 865831.20 (747871.73 to 1034361.22) | 475744.02 (411292.91 to 571202.72) | 197383.70 (169068.29 to 239427.82) | 59169.95 (50047.92 to 73489.60) | 13209.76 (10981.50 to 17129.31) |
| Global | Female | Number of DALYs | 19632.10 (13301.53 to 27758.56) | 33291.82 (22690.37 to 47560.25) | 33446.91 (22932.04 to 48347.64) | 32595.54 (22145.67 to 46066.36) | 31014.82 (20959.37 to 43955.76) | 28744.71 (19984.77 to 40748.96) | 26225.53 (17756.26 to 37170.72) | 23522.36 (16375.00 to 33153.56) | 20368.12 (14014.69 to 28863.00) | 17305.83 (11896.31 to 24243.96) | 14705.71 (10263.07 to 20502.58) | 12201.71 (8504.28 to 17187.09) | 10064.56 (6947.08 to 14249.75) | 7714.48 (5281.64 to 10697.89) | 5283.57 (3650.68 to 7209.43) | 3284.73 (2282.38 to 4505.26) | 1911.14 (1344.44 to 2642.46) | 833.16 (588.58 to 1120.96) | 257.20 (182.68 to 350.60) | 61.76 (44.58 to 83.01) |
| Global | Female | Number of prevalence | 580581.52 (514039.78 to 672269.58) | 1285984.77 (1127181.40 to 1518336.73) | 1732590.21 (1514384.89 to 2043859.46) | 2164878.83 (1886801.78 to 2559073.28) | 2442426.98 (2124064.71 to 2888671.27) | 2473600.04 (2146804.79 to 2913330.59) | 2383046.21 (2064055.95 to 2823756.01) | 2262162.91 (1956464.43 to 2706219.44) | 2027985.70 (1747413.55 to 2467115.47) | 1781441.00 (1532308.58 to 2190396.14) | 1554842.92 (1333180.28 to 1932395.05) | 1335079.14 (1140497.03 to 1676987.07) | 1158406.39 (989923.91 to 1460682.62) | 926291.68 (792086.77 to 1160907.68) | 657118.24 (565405.80 to 816192.60) | 413673.30 (356056.35 to 510379.95) | 241864.31 (208633.15 to 300465.97) | 104842.92 (90151.19 to 131691.36) | 32613.33 (27586.89 to 42320.92) | 8259.20 (6890.16 to 11005.26) |
| Global | Male | Number of DALYs | 14786.04 (9936.24 to 21095.06) | 72867.23 (35019.41 to 148038.15) | 84770.48 (40909.55 to 169248.75) | 93043.42 (46209.59 to 183849.73) | 97342.33 (49404.31 to 182843.67) | 95422.62 (49575.05 to 176677.37) | 91299.64 (47460.25 to 166766.30) | 85464.49 (44899.68 to 154417.21) | 76603.85 (40585.36 to 138104.29) | 67197.79 (36474.13 to 121128.30) | 56631.16 (30828.10 to 100964.70) | 46349.37 (25246.90 to 82292.58) | 38560.10 (21222.94 to 67268.89) | 29878.05 (16659.62 to 51400.98) | 20436.17 (11422.83 to 34842.31) | 12149.44 (6866.64 to 20773.12) | 6150.04 (3465.33 to 10496.86) | 2371.34 (1348.32 to 4096.66) | 659.27 (373.62 to 1139.18) | 114.83 (64.19 to 199.11) |
| Global | Male | Number of prevalence | 561223.86 (489574.09 to 652909.64) | 1656896.20 (1323122.20 to 2256789.03) | 2229371.90 (1812955.04 to 2921563.71) | 2749666.52 (2283028.76 to 3463832.25) | 3040170.95 (2538777.85 to 3747297.72) | 3045249.20 (2547505.94 to 3711275.39) | 2953563.95 (2476205.33 to 3615668.37) | 2807344.51 (2348603.40 to 3431259.78) | 2542360.68 (2122841.76 to 3122016.04) | 2254582.67 (1884304.40 to 2776140.49) | 1926152.74 (1607581.75 to 2373353.60) | 1599926.27 (1334164.76 to 1983315.16) | 1350222.13 (1130203.54 to 1675768.44) | 1064217.34 (892953.71 to 1308144.08) | 743831.70 (630280.91 to 903296.61) | 452157.90 (386275.14 to 545033.69) | 233879.71 (199309.00 to 282957.86) | 92540.78 (78500.03 to 111883.08) | 26556.62 (22412.09 to 32460.77) | 4950.56 (4047.88 to 6451.36) |
| **SDI region** |  |  |  |  |  |  |  |  |  |  |  |  |  |  |  |  |  |  |  |  |  |  |
| Low SDI | Both | Number of DALYs | 13705.57 (9419.75 to 19676.57) | 40331.31 (23015.41 to 72642.14) | 41534.52 (23498.18 to 74029.36) | 41011.95 (23404.56 to 72036.96) | 38148.37 (21895.26 to 65233.31) | 34077.67 (19733.12 to 57776.24) | 30724.46 (18234.70 to 51520.30) | 28052.43 (16509.88 to 46101.17) | 24393.17 (14564.36 to 39555.37) | 20344.47 (12319.16 to 33094.76) | 16321.15 (9579.63 to 26629.22) | 13024.33 (7855.60 to 21118.20) | 10395.70 (6299.52 to 16701.88) | 7866.35 (4773.86 to 12698.34) | 5263.40 (3185.12 to 8425.38) | 3115.80 (1914.03 to 5054.98) | 1535.72 (954.82 to 2429.14) | 575.22 (357.44 to 910.89) | 147.40 (94.12 to 228.25) | 27.35 (18.06 to 41.60) |
| Low SDI | Both | Number of prevalence | 443680.43 (361000.19 to 588970.30) | 1095053.06 (880345.76 to 1430305.28) | 1368713.29 (1107182.24 to 1769368.73) | 1568373.25 (1272470.70 to 2013323.96) | 1608005.56 (1307642.61 to 2057654.24) | 1509737.97 (1226660.86 to 1927599.88) | 1405272.53 (1142378.41 to 1799443.00) | 1314172.34 (1073880.59 to 1664560.91) | 1150530.34 (941927.65 to 1448710.67) | 971114.93 (796429.86 to 1217853.07) | 797771.87 (654060.12 to 1003672.06) | 654967.74 (538999.06 to 820913.28) | 538859.56 (445965.00 to 664898.91) | 420293.34 (350907.81 to 515335.75) | 290831.86 (243500.73 to 351630.31) | 175888.56 (147212.95 to 213461.30) | 90289.52 (75635.32 to 108455.98) | 34938.63 (29078.80 to 42636.27) | 9032.99 (7481.49 to 11144.35) | 1814.32 (1492.45 to 2261.92) |
| Low SDI | Female | Number of DALYs | 7892.96 (5381.24 to 11456.75) | 12685.92 (8462.61 to 18021.42) | 11986.74 (8003.67 to 17208.12) | 10899.97 (7471.95 to 15726.98) | 9425.34 (6437.37 to 13270.99) | 7942.83 (5443.04 to 11286.80) | 6764.64 (4504.48 to 9528.03) | 5871.48 (4057.66 to 8239.15) | 4840.22 (3347.63 to 6829.53) | 3840.19 (2630.27 to 5354.40) | 3036.23 (2058.47 to 4234.54) | 2422.49 (1650.05 to 3400.05) | 1916.52 (1319.65 to 2678.41) | 1456.90 (1008.93 to 2071.97) | 995.60 (674.56 to 1365.21) | 606.07 (422.07 to 832.15) | 326.31 (225.84 to 448.07) | 131.60 (92.34 to 178.78) | 35.26 (24.80 to 47.38) | 8.17 (5.77 to 10.96) |
| Low SDI | Female | Number of prevalence | 228290.48 (187523.25 to 299944.61) | 482262.28 (391592.28 to 639770.20) | 606395.05 (489072.32 to 799095.94) | 701524.58 (565267.23 to 914530.98) | 728370.51 (586961.24 to 951491.80) | 688348.74 (550866.19 to 902316.28) | 638748.30 (511438.66 to 835580.41) | 592307.03 (477688.84 to 771195.44) | 508756.64 (412171.49 to 658004.55) | 422475.42 (343434.30 to 543604.43) | 348069.33 (282848.51 to 446183.59) | 290324.69 (237083.12 to 369327.72) | 241912.64 (199694.75 to 301514.25) | 190357.20 (159063.97 to 233538.50) | 133124.53 (111460.41 to 161244.49) | 81301.51 (67663.93 to 98780.46) | 43923.29 (36450.63 to 53568.36) | 17828.24 (14738.14 to 22122.39) | 4703.03 (3831.29 to 5960.50) | 1077.04 (871.89 to 1375.38) |
| Low SDI | Male | Number of DALYs | 5812.61 (3905.19 to 8413.25) | 27645.38 (13378.33 to 56623.92) | 29547.79 (14074.00 to 58902.73) | 30111.97 (14970.21 to 58669.91) | 28723.03 (14554.64 to 53563.23) | 26134.84 (13614.05 to 47013.56) | 23959.82 (12789.31 to 43418.45) | 22180.96 (11923.49 to 39012.28) | 19552.95 (10713.25 to 33799.04) | 16504.28 (9111.00 to 28447.16) | 13284.92 (7326.38 to 23070.28) | 10601.84 (5940.59 to 18292.60) | 8479.18 (4765.60 to 14469.35) | 6409.45 (3591.58 to 10902.66) | 4267.80 (2405.08 to 7225.65) | 2509.73 (1425.81 to 4266.15) | 1209.41 (691.43 to 2044.56) | 443.62 (249.56 to 754.32) | 112.14 (64.56 to 188.05) | 19.17 (11.16 to 31.59) |
| Low SDI | Male | Number of prevalence | 215389.96 (175182.07 to 288148.89) | 612790.78 (468385.86 to 840552.62) | 762318.25 (596697.21 to 1016026.58) | 866848.66 (693115.66 to 1132753.24) | 879635.05 (712305.85 to 1138751.82) | 821389.23 (667184.37 to 1055853.48) | 766524.23 (623686.94 to 985826.70) | 721865.31 (589374.47 to 922426.11) | 641773.70 (525349.68 to 820079.78) | 548639.51 (449107.73 to 697226.03) | 449702.54 (366999.56 to 570602.08) | 364643.05 (298765.79 to 459647.60) | 296946.92 (245402.08 to 372085.07) | 229936.14 (191081.56 to 283652.66) | 157707.33 (130933.32 to 191236.39) | 94587.05 (78426.96 to 113668.70) | 46366.22 (38470.14 to 56113.74) | 17110.38 (14203.14 to 20810.05) | 4329.96 (3559.06 to 5307.36) | 737.28 (603.04 to 910.03) |
| Low-middle SDI | Both | Number of DALYs | 13089.43 (8818.00 to 18404.10) | 40593.79 (22996.11 to 71620.60) | 48682.72 (27311.07 to 85579.51) | 53787.29 (30197.82 to 95285.73) | 56523.99 (32526.75 to 96779.17) | 54525.19 (31456.28 to 92855.18) | 50956.43 (29260.50 to 86642.00) | 47187.67 (27512.59 to 79718.54) | 42086.44 (24749.09 to 69881.55) | 37069.16 (21657.33 to 62105.68) | 31452.92 (18614.86 to 51777.77) | 25739.77 (15183.01 to 41974.01) | 21985.86 (13335.76 to 35055.07) | 17932.64 (11072.93 to 28343.81) | 12938.18 (7956.83 to 20581.94) | 7884.95 (4834.66 to 12407.14) | 4130.72 (2585.15 to 6468.41) | 1614.88 (1013.67 to 2520.63) | 482.86 (305.40 to 755.68) | 90.05 (57.65 to 139.42) |
| Low-middle SDI | Both | Number of prevalence | 463342.06 (399861.96 to 557569.98) | 1203703.45 (1006639.64 to 1493778.49) | 1711545.45 (1434396.53 to 2108180.69) | 2199450.63 (1843331.25 to 2690633.55) | 2498306.14 (2101430.71 to 3045367.47) | 2486542.50 (2090081.91 to 3025191.52) | 2361566.49 (1982450.90 to 2867737.63) | 2238901.04 (1878246.44 to 2735100.93) | 2024624.96 (1694145.95 to 2494533.20) | 1799960.32 (1506148.05 to 2226404.96) | 1557453.76 (1306289.40 to 1940985.42) | 1312731.74 (1100590.76 to 1646673.69) | 1153688.49 (968074.36 to 1443421.74) | 960228.21 (809942.79 to 1183799.28) | 707027.96 (600746.37 to 856351.69) | 443694.15 (378897.83 to 532476.32) | 245177.47 (210457.17 to 292607.36) | 99625.59 (85287.08 to 120102.74) | 30666.79 (26050.68 to 37175.47) | 6811.13 (5740.00 to 8359.08) |
| Low-middle SDI | Female | Number of DALYs | 7431.90 (5028.10 to 10649.14) | 12912.55 (8804.47 to 18455.81) | 13468.08 (9112.62 to 19319.27) | 13548.19 (9019.78 to 19096.80) | 13188.44 (8787.17 to 18712.22) | 12224.68 (8496.48 to 17325.10) | 11034.56 (7471.58 to 15436.82) | 9874.74 (6807.65 to 13938.96) | 8591.64 (5814.85 to 12161.13) | 7386.22 (5041.12 to 10391.19) | 6296.56 (4376.89 to 8810.90) | 5217.43 (3571.66 to 7336.75) | 4421.73 (3042.66 to 6226.51) | 3518.50 (2440.10 to 4859.18) | 2520.67 (1746.17 to 3470.85) | 1592.48 (1075.53 to 2193.25) | 928.69 (649.55 to 1285.23) | 392.88 (274.37 to 533.08) | 121.14 (86.22 to 164.21) | 29.82 (21.16 to 40.30) |
| Low-middle SDI | Female | Number of prevalence | 233411.91 (203306.11 to 279426.85) | 528838.12 (455309.88 to 640764.01) | 745699.38 (637728.54 to 899032.52) | 963609.25 (819731.57 to 1165649.78) | 1106923.55 (939481.33 to 1340674.51) | 1108302.83 (938705.76 to 1349735.17) | 1047783.22 (883796.64 to 1287666.31) | 996261.39 (839230.09 to 1227211.96) | 900734.41 (754178.51 to 1112146.55) | 796629.66 (663528.31 to 1000148.68) | 698840.05 (579101.73 to 893385.62) | 603272.60 (498980.29 to 782941.96) | 540913.13 (446273.10 to 693034.68) | 452039.63 (376236.89 to 566847.86) | 334165.26 (281924.95 to 408516.91) | 213759.80 (182035.88 to 257908.67) | 125819.81 (107856.79 to 151780.16) | 52832.89 (45276.14 to 65230.17) | 16414.58 (13942.93 to 20624.48) | 4263.90 (3590.77 to 5326.81) |
| Low-middle SDI | Male | Number of DALYs | 5657.53 (3737.57 to 8065.01) | 27681.24 (13642.45 to 55717.18) | 35214.64 (16972.76 to 68079.48) | 40239.09 (20156.87 to 78888.45) | 43335.55 (22082.95 to 82183.74) | 42300.50 (21944.00 to 77668.95) | 39921.87 (20858.01 to 71970.98) | 37312.93 (19685.90 to 67638.79) | 33494.80 (17774.10 to 59578.94) | 29682.94 (16251.52 to 53092.54) | 25156.36 (13588.91 to 44173.87) | 20522.34 (11202.31 to 35581.93) | 17564.13 (9762.90 to 29969.61) | 14414.14 (8254.00 to 24289.25) | 10417.51 (5953.08 to 17393.59) | 6292.47 (3588.95 to 10549.15) | 3202.03 (1800.65 to 5377.10) | 1222.00 (698.44 to 2071.15) | 361.72 (206.14 to 612.61) | 60.23 (33.89 to 104.45) |
| Low-middle SDI | Male | Number of prevalence | 229930.15 (198322.55 to 277752.91) | 674865.32 (535140.65 to 897303.14) | 965846.07 (777374.36 to 1259764.21) | 1235841.38 (1008723.60 to 1563651.81) | 1391382.59 (1146731.79 to 1738259.98) | 1378239.67 (1142462.68 to 1716133.95) | 1313783.27 (1093194.30 to 1653408.41) | 1242639.66 (1032018.74 to 1553024.39) | 1123890.55 (932106.41 to 1395210.74) | 1003330.67 (833078.71 to 1244992.41) | 858613.71 (711494.24 to 1067513.16) | 709459.15 (586819.81 to 885100.40) | 612775.36 (507584.05 to 760234.98) | 508188.58 (425269.27 to 626211.52) | 372862.70 (314376.55 to 455660.27) | 229934.36 (194790.98 to 278209.93) | 119357.66 (101198.69 to 144512.32) | 46792.69 (39272.79 to 57284.71) | 14252.21 (11833.46 to 17426.89) | 2547.24 (2088.77 to 3290.05) |
| Middle SDI | Both | Number of DALYs | 7005.35 (4738.41 to 9992.01) | 23248.30 (13278.23 to 42700.94) | 25794.84 (14573.00 to 46781.81) | 28442.28 (16213.58 to 50898.05) | 31057.16 (17617.18 to 54417.36) | 32810.73 (18613.62 to 56606.44) | 33110.94 (18963.46 to 57492.63) | 31173.39 (17637.22 to 53918.37) | 28200.09 (16017.43 to 48284.03) | 25098.03 (14575.99 to 42702.48) | 21834.69 (12860.39 to 36941.44) | 18338.42 (10661.76 to 31045.55) | 15111.14 (8972.90 to 25314.39) | 10964.56 (6589.02 to 18264.66) | 7010.58 (4182.14 to 11735.99) | 4130.67 (2542.17 to 6776.23) | 2247.23 (1395.97 to 3619.99) | 951.86 (592.45 to 1548.49) | 266.81 (169.57 to 422.51) | 54.90 (35.37 to 84.98) |
| Middle SDI | Both | Number of prevalence | 218483.91 (180855.79 to 302231.11) | 600523.73 (472228.03 to 850807.18) | 822355.80 (648255.84 to 1151963.89) | 1070380.02 (849427.38 to 1493263.39) | 1283471.62 (1029618.41 to 1778302.24) | 1419268.43 (1138972.38 to 2001894.61) | 1462951.65 (1168421.38 to 2069368.42) | 1412680.10 (1119957.96 to 2004048.30) | 1300419.30 (1030452.74 to 1861134.92) | 1180628.24 (931079.72 to 1682605.87) | 1050984.75 (823970.63 to 1512000.77) | 903171.93 (704547.37 to 1317899.02) | 764509.86 (594729.68 to 1130075.41) | 571304.98 (445044.43 to 846423.92) | 378726.47 (298145.02 to 550983.71) | 231375.63 (182616.15 to 334074.79) | 132758.06 (104970.28 to 194262.45) | 59550.68 (46307.10 to 88788.52) | 18468.07 (13838.35 to 30352.44) | 4372.68 (3040.02 to 7711.67) |
| Middle SDI | Female | Number of DALYs | 3959.23 (2678.74 to 5655.89) | 7079.60 (4770.52 to 9982.09) | 7339.73 (4955.89 to 10460.51) | 7472.42 (5009.36 to 10574.06) | 7689.75 (5249.00 to 10838.01) | 7854.06 (5340.50 to 11245.53) | 7718.48 (5271.52 to 11109.47) | 7118.59 (4790.57 to 10121.99) | 6356.61 (4319.35 to 8922.35) | 5583.90 (3770.59 to 7899.55) | 4938.98 (3398.06 to 6907.50) | 4194.98 (2906.56 to 5915.04) | 3435.63 (2340.49 to 4885.55) | 2524.19 (1727.08 to 3557.09) | 1631.49 (1123.37 to 2239.74) | 1002.62 (683.61 to 1392.53) | 612.25 (439.75 to 852.79) | 290.03 (200.66 to 393.87) | 95.46 (66.94 to 130.12) | 22.88 (16.37 to 30.94) |
| Middle SDI | Female | Number of prevalence | 110588.63 (92208.00 to 152415.94) | 256721.53 (209973.46 to 372837.95) | 355258.12 (288719.02 to 507662.45) | 466872.75 (378604.35 to 671468.09) | 566839.61 (461092.83 to 817245.15) | 631889.65 (512841.19 to 912100.16) | 649733.71 (525120.94 to 950411.53) | 627989.73 (504381.21 to 930746.00) | 576791.48 (456927.77 to 859580.41) | 525175.37 (411472.09 to 784264.48) | 474722.72 (367612.03 to 712354.36) | 412802.84 (316455.75 to 632163.00) | 352325.29 (268674.96 to 544597.88) | 266374.03 (203110.39 to 413199.88) | 178606.23 (136892.02 to 274427.63) | 111622.06 (85016.12 to 173656.03) | 68393.13 (52010.35 to 105820.05) | 32557.74 (24530.73 to 51359.11) | 11029.82 (8009.49 to 18403.26) | 2839.37 (1919.20 to 5259.12) |
| Middle SDI | Male | Number of DALYs | 3046.11 (2012.54 to 4482.09) | 16168.70 (7742.28 to 32631.81) | 18455.11 (9051.62 to 37151.81) | 20969.87 (10269.67 to 41295.88) | 23367.41 (11744.94 to 45054.35) | 24956.67 (12652.41 to 46939.57) | 25392.46 (12760.14 to 47426.85) | 24054.80 (12240.88 to 44881.25) | 21843.48 (11294.68 to 40770.10) | 19514.12 (10204.23 to 36122.96) | 16895.71 (8907.42 to 31106.29) | 14143.44 (7565.71 to 25829.24) | 11675.51 (6322.33 to 21185.18) | 8440.38 (4613.89 to 15119.25) | 5379.08 (2893.44 to 9685.26) | 3128.06 (1719.00 to 5607.85) | 1634.98 (906.65 to 2870.09) | 661.83 (362.45 to 1179.93) | 171.35 (93.06 to 307.23) | 32.02 (17.41 to 57.81) |
| Middle SDI | Male | Number of prevalence | 107895.28 (87996.46 to 149874.66) | 343802.21 (255656.07 to 498749.88) | 467097.68 (357923.11 to 657164.53) | 603507.27 (468827.15 to 835393.16) | 716632.01 (560000.48 to 985599.41) | 787378.78 (613786.80 to 1080670.81) | 813217.94 (633894.42 to 1126900.91) | 784690.37 (610599.82 to 1098432.46) | 723627.81 (560061.85 to 1015825.72) | 655452.87 (506072.51 to 925193.35) | 576262.03 (443563.96 to 825444.17) | 490369.09 (376936.61 to 710282.77) | 412184.58 (316872.73 to 596461.63) | 304930.95 (235141.21 to 441260.94) | 200120.24 (155596.81 to 282455.50) | 119753.56 (94185.90 to 165364.30) | 64364.93 (50863.67 to 88365.89) | 26992.94 (21255.55 to 37928.23) | 7438.25 (5643.31 to 11405.09) | 1533.32 (1053.86 to 2750.14) |
| High-middle SDI | Both | Number of DALYs | 587.53 (389.86 to 843.51) | 1881.04 (1050.64 to 3381.58) | 2086.97 (1150.49 to 3711.19) | 2260.68 (1261.98 to 3998.98) | 2470.82 (1369.79 to 4526.80) | 2589.40 (1402.81 to 4770.87) | 2576.46 (1430.97 to 4614.95) | 2426.35 (1341.57 to 4307.46) | 2157.77 (1215.52 to 3829.02) | 1873.73 (1038.92 to 3396.62) | 1625.90 (935.57 to 2860.92) | 1363.20 (768.74 to 2405.02) | 1066.46 (596.61 to 1878.80) | 783.06 (443.14 to 1390.82) | 479.01 (277.76 to 841.07) | 286.14 (163.30 to 495.65) | 139.35 (81.30 to 238.11) | 59.19 (33.28 to 101.69) | 18.33 (10.41 to 31.38) | 4.04 (2.25 to 7.14) |
| High-middle SDI | Both | Number of prevalence | 14763.58 (8519.23 to 33446.07) | 39899.59 (22939.87 to 85153.17) | 54650.36 (29688.43 to 124234.00) | 70632.30 (36528.90 to 167448.80) | 86083.71 (43081.38 to 202950.63) | 96145.66 (47098.76 to 224507.54) | 99910.57 (48211.08 to 234948.68) | 97247.21 (46558.47 to 235328.14) | 88767.79 (42197.10 to 225462.05) | 79022.31 (37410.99 to 204167.77) | 70192.17 (33008.61 to 180366.84) | 60236.63 (28168.52 to 151593.43) | 48523.97 (22810.48 to 112912.19) | 36489.63 (17388.28 to 85519.50) | 22955.94 (10989.82 to 55155.45) | 14025.82 (6626.93 to 34309.37) | 7081.40 (3369.46 to 17247.57) | 3077.68 (1445.98 to 7897.00) | 937.17 (415.41 to 2563.03) | 195.81 (82.78 to 577.49) |
| High-middle SDI | Female | Number of DALYs | 330.73 (214.48 to 476.30) | 585.68 (380.39 to 866.17) | 625.21 (409.85 to 905.99) | 648.41 (423.92 to 944.22) | 684.92 (441.84 to 1016.34) | 697.29 (456.76 to 1019.85) | 683.43 (458.32 to 982.37) | 635.53 (409.32 to 894.97) | 560.20 (365.22 to 801.76) | 479.01 (321.83 to 676.17) | 420.01 (280.64 to 603.72) | 355.26 (240.20 to 531.05) | 281.65 (185.40 to 403.78) | 208.29 (136.59 to 295.62) | 131.51 (86.10 to 184.20) | 80.90 (55.36 to 115.15) | 42.40 (28.11 to 59.39) | 17.95 (11.91 to 25.19) | 5.09 (3.54 to 7.16) | 0.82 (0.54 to 1.14) |
| High-middle SDI | Female | Number of prevalence | 7524.94 (4517.59 to 16477.11) | 16553.15 (9165.96 to 38270.78) | 23216.70 (11427.87 to 57373.21) | 30436.18 (13921.40 to 78511.80) | 37416.37 (16409.08 to 96153.14) | 41957.94 (18060.57 to 106943.73) | 43743.00 (18564.04 to 112857.66) | 42756.01 (17866.21 to 112700.89) | 39092.43 (15840.69 to 105660.62) | 34883.09 (14030.64 to 95857.88) | 31269.44 (12487.56 to 84950.66) | 27033.18 (10813.61 to 71758.51) | 21964.37 (9096.95 to 52376.96) | 16578.36 (6917.57 to 40296.67) | 10606.61 (4545.14 to 26711.17) | 6607.00 (2824.50 to 16987.04) | 3519.45 (1532.60 to 8865.79) | 1527.97 (663.67 to 3951.25) | 431.86 (182.74 to 1191.14) | 70.55 (30.40 to 195.02) |
| High-middle SDI | Male | Number of DALYs | 256.80 (159.27 to 393.10) | 1295.36 (603.61 to 2684.87) | 1461.76 (703.44 to 2970.60) | 1612.27 (777.03 to 3275.40) | 1785.90 (819.24 to 3661.52) | 1892.10 (870.08 to 3820.74) | 1893.03 (879.42 to 3756.89) | 1790.83 (862.59 to 3577.95) | 1597.57 (766.66 to 3196.43) | 1394.72 (674.31 to 2782.84) | 1205.88 (583.26 to 2352.28) | 1007.95 (480.14 to 1951.73) | 784.81 (381.76 to 1526.67) | 574.78 (283.20 to 1138.51) | 347.49 (173.82 to 671.86) | 205.25 (101.02 to 393.84) | 96.95 (47.55 to 183.56) | 41.24 (20.01 to 79.19) | 13.24 (6.38 to 25.08) | 3.21 (1.58 to 6.26) |
| High-middle SDI | Male | Number of prevalence | 7238.64 (3948.88 to 16968.96) | 23346.43 (12935.33 to 45982.21) | 31433.66 (16797.87 to 67675.21) | 40196.12 (20934.53 to 89708.42) | 48667.34 (24762.27 to 107747.88) | 54187.72 (27380.45 to 118668.99) | 56167.58 (28208.27 to 124490.09) | 54491.20 (27072.67 to 123742.54) | 49675.37 (24526.92 to 118369.00) | 44139.22 (21786.65 to 107644.05) | 38922.73 (18973.81 to 95193.19) | 33203.45 (16135.05 to 79555.51) | 26559.60 (13165.89 to 59301.79) | 19911.26 (9885.05 to 44994.63) | 12349.33 (6157.81 to 28367.84) | 7418.81 (3674.92 to 17478.14) | 3561.96 (1757.75 to 8414.90) | 1549.71 (738.61 to 3946.34) | 505.31 (218.22 to 1408.48) | 125.26 (49.08 to 386.82) |
| High SDI | Both | Number of DALYs | 0.00 (0.00 to 0.00) | 0.00 (0.00 to 0.00) | 0.00 (0.00 to 0.00) | 0.00 (0.00 to 0.00) | 0.00 (0.00 to 0.00) | 0.00 (0.00 to 0.00) | 0.00 (0.00 to 0.00) | 0.00 (0.00 to 0.00) | 0.00 (0.00 to 0.00) | 0.00 (0.00 to 0.00) | 0.00 (0.00 to 0.00) | 0.00 (0.00 to 0.00) | 0.00 (0.00 to 0.00) | 0.00 (0.00 to 0.00) | 0.00 (0.00 to 0.00) | 0.00 (0.00 to 0.00) | 0.00 (0.00 to 0.00) | 0.00 (0.00 to 0.00) | 0.00 (0.00 to 0.00) | 0.00 (0.00 to 0.00) |
| High SDI | Both | Number of prevalence | 0.00 (0.00 to 0.00) | 0.00 (0.00 to 0.00) | 0.00 (0.00 to 0.00) | 0.00 (0.00 to 0.00) | 0.00 (0.00 to 0.00) | 0.00 (0.00 to 0.00) | 0.00 (0.00 to 0.00) | 0.00 (0.00 to 0.00) | 0.00 (0.00 to 0.00) | 0.00 (0.00 to 0.00) | 0.00 (0.00 to 0.00) | 0.00 (0.00 to 0.00) | 0.00 (0.00 to 0.00) | 0.00 (0.00 to 0.00) | 0.00 (0.00 to 0.00) | 0.00 (0.00 to 0.00) | 0.00 (0.00 to 0.00) | 0.00 (0.00 to 0.00) | 0.00 (0.00 to 0.00) | 0.00 (0.00 to 0.00) |
| High SDI | Female | Number of DALYs | 0.00 (0.00 to 0.00) | 0.00 (0.00 to 0.00) | 0.00 (0.00 to 0.00) | 0.00 (0.00 to 0.00) | 0.00 (0.00 to 0.00) | 0.00 (0.00 to 0.00) | 0.00 (0.00 to 0.00) | 0.00 (0.00 to 0.00) | 0.00 (0.00 to 0.00) | 0.00 (0.00 to 0.00) | 0.00 (0.00 to 0.00) | 0.00 (0.00 to 0.00) | 0.00 (0.00 to 0.00) | 0.00 (0.00 to 0.00) | 0.00 (0.00 to 0.00) | 0.00 (0.00 to 0.00) | 0.00 (0.00 to 0.00) | 0.00 (0.00 to 0.00) | 0.00 (0.00 to 0.00) | 0.00 (0.00 to 0.00) |
| High SDI | Female | Number of prevalence | 0.00 (0.00 to 0.00) | 0.00 (0.00 to 0.00) | 0.00 (0.00 to 0.00) | 0.00 (0.00 to 0.00) | 0.00 (0.00 to 0.00) | 0.00 (0.00 to 0.00) | 0.00 (0.00 to 0.00) | 0.00 (0.00 to 0.00) | 0.00 (0.00 to 0.00) | 0.00 (0.00 to 0.00) | 0.00 (0.00 to 0.00) | 0.00 (0.00 to 0.00) | 0.00 (0.00 to 0.00) | 0.00 (0.00 to 0.00) | 0.00 (0.00 to 0.00) | 0.00 (0.00 to 0.00) | 0.00 (0.00 to 0.00) | 0.00 (0.00 to 0.00) | 0.00 (0.00 to 0.00) | 0.00 (0.00 to 0.00) |
| High SDI | Male | Number of DALYs | 0.00 (0.00 to 0.00) | 0.00 (0.00 to 0.00) | 0.00 (0.00 to 0.00) | 0.00 (0.00 to 0.00) | 0.00 (0.00 to 0.00) | 0.00 (0.00 to 0.00) | 0.00 (0.00 to 0.00) | 0.00 (0.00 to 0.00) | 0.00 (0.00 to 0.00) | 0.00 (0.00 to 0.00) | 0.00 (0.00 to 0.00) | 0.00 (0.00 to 0.00) | 0.00 (0.00 to 0.00) | 0.00 (0.00 to 0.00) | 0.00 (0.00 to 0.00) | 0.00 (0.00 to 0.00) | 0.00 (0.00 to 0.00) | 0.00 (0.00 to 0.00) | 0.00 (0.00 to 0.00) | 0.00 (0.00 to 0.00) |
| High SDI | Male | Number of prevalence | 0.00 (0.00 to 0.00) | 0.00 (0.00 to 0.00) | 0.00 (0.00 to 0.00) | 0.00 (0.00 to 0.00) | 0.00 (0.00 to 0.00) | 0.00 (0.00 to 0.00) | 0.00 (0.00 to 0.00) | 0.00 (0.00 to 0.00) | 0.00 (0.00 to 0.00) | 0.00 (0.00 to 0.00) | 0.00 (0.00 to 0.00) | 0.00 (0.00 to 0.00) | 0.00 (0.00 to 0.00) | 0.00 (0.00 to 0.00) | 0.00 (0.00 to 0.00) | 0.00 (0.00 to 0.00) | 0.00 (0.00 to 0.00) | 0.00 (0.00 to 0.00) | 0.00 (0.00 to 0.00) | 0.00 (0.00 to 0.00) |
| **GBD region** |  |  |  |  |  |  |  |  |  |  |  |  |  |  |  |  |  |  |  |  |  |  |
| Caribbean | Both | Number of DALYs | 270.39 (169.92 to 391.95) | 812.60 (454.58 to 1428.09) | 844.70 (486.61 to 1450.29) | 936.58 (553.11 to 1596.02) | 1021.26 (623.18 to 1667.80) | 1011.80 (616.93 to 1669.41) | 924.70 (558.53 to 1509.65) | 826.31 (501.55 to 1364.80) | 743.19 (454.72 to 1183.88) | 640.82 (401.95 to 1032.97) | 554.53 (344.36 to 873.07) | 466.89 (295.27 to 740.97) | 373.61 (236.04 to 591.17) | 278.97 (178.27 to 439.96) | 185.11 (117.40 to 292.38) | 115.98 (72.20 to 186.04) | 66.65 (41.74 to 103.50) | 33.49 (21.34 to 53.16) | 12.83 (8.06 to 20.46) | 3.10 (2.01 to 4.76) |
| Caribbean | Both | Number of prevalence | 8237.09 (5084.65 to 14443.57) | 20352.15 (12373.58 to 35246.12) | 25529.36 (14982.50 to 45687.93) | 32349.95 (18251.36 to 60223.74) | 38672.91 (21359.05 to 72370.29) | 41220.91 (22484.89 to 78420.15) | 39132.96 (20926.42 to 76424.35) | 36367.21 (19333.36 to 71554.89) | 34027.32 (18220.62 to 66800.34) | 30288.27 (16051.51 to 60275.92) | 26996.49 (14107.95 to 54318.93) | 23697.89 (12266.35 to 47743.32) | 19675.28 (10117.97 to 39750.47) | 15174.74 (7741.64 to 31163.58) | 10233.83 (5130.49 to 22330.15) | 6572.56 (3204.61 to 15269.38) | 3915.76 (1862.91 to 9586.89) | 2038.26 (916.32 to 5304.12) | 792.79 (334.05 to 2194.02) | 192.13 (81.14 to 530.70) |
| Caribbean | Female | Number of DALYs | 155.74 (98.13 to 230.83) | 246.45 (150.50 to 372.15) | 234.97 (151.65 to 351.25) | 237.51 (156.80 to 350.96) | 237.54 (151.02 to 342.25) | 230.43 (145.79 to 343.03) | 219.16 (142.59 to 321.62) | 199.22 (129.01 to 293.96) | 171.94 (109.48 to 247.63) | 143.70 (86.24 to 206.08) | 121.69 (80.62 to 172.75) | 103.60 (68.84 to 150.76) | 83.53 (54.76 to 118.52) | 64.02 (42.15 to 93.47) | 43.83 (27.98 to 63.37) | 28.35 (18.76 to 40.98) | 17.33 (11.38 to 24.68) | 9.03 (6.03 to 13.27) | 3.52 (2.30 to 5.00) | 0.81 (0.53 to 1.16) |
| Caribbean | Female | Number of prevalence | 4268.30 (2707.57 to 7335.05) | 8864.35 (5228.37 to 16008.39) | 11208.14 (6223.38 to 21064.42) | 14294.22 (7500.03 to 27679.72) | 17210.07 (8691.30 to 33887.55) | 18651.05 (9270.02 to 37068.24) | 17943.53 (8942.81 to 36426.98) | 16833.53 (8374.74 to 34174.88) | 15713.13 (7651.04 to 31812.89) | 13913.24 (6656.44 to 28672.25) | 12379.35 (5849.26 to 25981.28) | 11033.09 (5210.62 to 23117.54) | 9263.05 (4339.73 to 19438.19) | 7270.31 (3364.60 to 15486.85) | 4938.43 (2190.04 to 11277.07) | 3239.07 (1396.15 to 7866.94) | 2005.53 (835.58 to 5091.22) | 1061.58 (421.11 to 2864.45) | 413.59 (149.79 to 1199.46) | 97.04 (33.50 to 292.28) |
| Caribbean | Male | Number of DALYs | 114.65 (67.61 to 179.94) | 566.15 (274.17 to 1119.36) | 609.73 (314.69 to 1183.93) | 699.07 (373.62 to 1298.13) | 783.73 (442.89 to 1399.92) | 781.37 (435.16 to 1394.82) | 705.54 (394.99 to 1235.43) | 627.09 (349.73 to 1104.65) | 571.25 (322.23 to 983.27) | 497.12 (291.72 to 863.52) | 432.85 (253.06 to 733.96) | 363.29 (213.35 to 619.60) | 290.08 (172.16 to 481.33) | 214.96 (127.37 to 353.27) | 141.28 (81.97 to 239.56) | 87.63 (50.97 to 152.41) | 49.32 (28.77 to 84.86) | 24.46 (14.12 to 41.90) | 9.31 (5.27 to 16.27) | 2.29 (1.38 to 3.81) |
| Caribbean | Male | Number of prevalence | 3968.79 (2352.18 to 7112.58) | 11487.80 (6928.29 to 19507.10) | 14321.22 (8564.90 to 25288.38) | 18055.73 (10513.21 to 32784.78) | 21462.84 (12433.81 to 38695.37) | 22569.85 (12909.00 to 41562.32) | 21189.43 (11855.08 to 40448.31) | 19533.68 (10827.82 to 37844.01) | 18314.19 (10111.73 to 35376.01) | 16375.03 (9091.15 to 31875.51) | 14617.14 (8030.15 to 28585.06) | 12664.80 (6896.52 to 24842.18) | 10412.23 (5651.01 to 20489.71) | 7904.43 (4267.57 to 15841.25) | 5295.40 (2817.63 to 11153.02) | 3333.50 (1744.95 to 7397.99) | 1910.24 (974.49 to 4420.47) | 976.67 (475.33 to 2388.75) | 379.20 (176.32 to 980.78) | 95.08 (45.52 to 235.30) |
| Central Sub-Saharan Africa | Both | Number of DALYs | 2077.60 (1344.95 to 3078.87) | 6236.41 (3410.59 to 11465.76) | 5973.48 (3313.07 to 10909.22) | 5558.94 (3088.68 to 9885.22) | 5026.58 (2695.61 to 8758.82) | 4526.88 (2498.26 to 7980.39) | 4000.46 (2183.75 to 7099.63) | 3459.33 (1927.02 to 5933.62) | 2924.77 (1605.56 to 5181.42) | 2451.39 (1350.55 to 4285.89) | 2015.82 (1136.30 to 3449.49) | 1594.36 (915.49 to 2763.64) | 1165.78 (670.95 to 2015.13) | 781.00 (446.11 to 1305.93) | 418.35 (245.45 to 699.53) | 230.68 (135.72 to 381.69) | 110.39 (66.48 to 177.37) | 40.96 (24.47 to 66.74) | 10.53 (6.42 to 16.53) | 1.98 (1.21 to 3.03) |
| Central Sub-Saharan Africa | Both | Number of prevalence | 75167.02 (44091.49 to 138761.21) | 184529.75 (106918.89 to 329690.93) | 221175.92 (123217.61 to 410005.35) | 243238.69 (131869.35 to 461127.32) | 242925.45 (130125.81 to 461966.89) | 228930.15 (121784.66 to 435677.62) | 210135.81 (111009.79 to 401496.93) | 186215.34 (97922.65 to 356423.89) | 159432.39 (83467.79 to 307305.33) | 135133.80 (70683.33 to 260631.17) | 113635.69 (59512.57 to 218643.26) | 93165.71 (48772.18 to 179258.07) | 70890.91 (37151.19 to 136450.89) | 49123.03 (25740.78 to 94216.45) | 27928.81 (14550.03 to 53702.39) | 16121.08 (8300.95 to 31100.17) | 8193.64 (4203.81 to 15786.42) | 3240.26 (1630.71 to 6351.10) | 887.58 (441.25 to 1769.44) | 184.53 (90.81 to 371.82) |
| Central Sub-Saharan Africa | Female | Number of DALYs | 1207.59 (756.89 to 1830.91) | 1927.01 (1162.00 to 2865.79) | 1752.54 (1099.95 to 2645.77) | 1520.62 (994.41 to 2259.47) | 1277.50 (834.31 to 1904.46) | 1105.89 (695.02 to 1646.40) | 941.74 (580.85 to 1371.55) | 792.51 (500.16 to 1135.51) | 652.57 (418.82 to 957.64) | 531.49 (341.20 to 765.66) | 428.33 (273.79 to 626.40) | 347.14 (229.21 to 501.49) | 262.86 (171.71 to 368.18) | 186.17 (119.90 to 276.91) | 112.14 (72.30 to 167.39) | 67.57 (43.95 to 97.53) | 35.52 (23.30 to 51.47) | 14.37 (9.25 to 20.93) | 4.08 (2.68 to 5.75) | 0.87 (0.57 to 1.25) |
| Central Sub-Saharan Africa | Female | Number of prevalence | 38732.51 (23195.47 to 69899.21) | 82460.65 (46236.04 to 156376.94) | 100077.50 (53308.11 to 196370.53) | 110830.28 (56763.10 to 220635.11) | 111415.93 (55640.89 to 221202.38) | 105483.17 (52138.44 to 213661.09) | 96999.62 (47577.53 to 197536.46) | 85579.24 (41695.22 to 173223.67) | 73039.01 (35463.22 to 148485.30) | 61282.67 (29657.42 to 124869.47) | 51812.57 (24981.76 to 103998.71) | 43752.07 (21056.16 to 88441.55) | 34458.16 (16472.54 to 68828.08) | 24823.60 (11828.85 to 49366.40) | 15182.19 (7217.06 to 30154.94) | 9266.86 (4369.10 to 18404.56) | 4978.84 (2363.99 to 9946.32) | 2067.41 (969.24 to 4196.63) | 597.40 (278.14 to 1230.83) | 133.32 (62.10 to 276.59) |
| Central Sub-Saharan Africa | Male | Number of DALYs | 870.00 (522.89 to 1337.81) | 4309.40 (1969.77 to 9218.63) | 4220.95 (1913.25 to 8875.15) | 4038.32 (1871.58 to 8123.27) | 3749.08 (1731.21 to 7222.98) | 3420.99 (1651.15 to 6571.48) | 3058.72 (1419.29 to 6029.41) | 2666.82 (1285.24 to 4996.46) | 2272.20 (1127.31 to 4368.22) | 1919.90 (925.01 to 3601.84) | 1587.48 (793.58 to 2937.34) | 1247.22 (629.21 to 2316.50) | 902.92 (451.17 to 1676.55) | 594.83 (304.95 to 1076.11) | 306.21 (154.02 to 549.76) | 163.11 (81.94 to 295.69) | 74.87 (38.52 to 136.11) | 26.59 (13.45 to 47.84) | 6.45 (3.19 to 11.70) | 1.10 (0.56 to 2.02) |
| Central Sub-Saharan Africa | Male | Number of prevalence | 36434.51 (20509.12 to 68876.95) | 102069.10 (57905.59 to 172957.50) | 121098.43 (67116.28 to 215193.22) | 132408.41 (71843.16 to 239122.13) | 131509.52 (71375.46 to 240460.62) | 123446.98 (67033.58 to 227397.64) | 113136.18 (61463.75 to 209359.87) | 100636.10 (54615.56 to 186012.10) | 86393.38 (46693.56 to 158845.39) | 73851.13 (39831.67 to 135233.95) | 61823.12 (33323.43 to 113252.32) | 49413.64 (26613.56 to 90844.46) | 36432.75 (19604.63 to 67187.23) | 24299.42 (13073.40 to 44872.33) | 12746.62 (6867.92 to 23643.85) | 6854.22 (3707.51 to 12786.44) | 3214.81 (1744.02 to 6000.62) | 1172.85 (631.38 to 2190.21) | 290.18 (156.83 to 542.13) | 51.21 (27.64 to 95.88) |
| Eastern Sub-Saharan Africa | Both | Number of DALYs | 5325.68 (3615.02 to 7739.35) | 13871.85 (8249.77 to 25014.33) | 13928.37 (7911.72 to 25010.14) | 12800.00 (7190.00 to 23350.56) | 10832.09 (6213.13 to 19024.06) | 8986.28 (5262.04 to 16141.51) | 7520.24 (4306.01 to 13408.00) | 6338.07 (3694.69 to 11302.88) | 5267.92 (3021.36 to 9129.64) | 4177.92 (2373.53 to 7397.99) | 3167.85 (1796.76 to 5600.78) | 2392.51 (1385.97 to 4201.43) | 1817.67 (1037.20 to 3222.53) | 1334.36 (787.30 to 2348.46) | 861.50 (506.12 to 1519.58) | 526.42 (309.10 to 904.59) | 265.36 (159.18 to 448.53) | 104.77 (62.09 to 177.73) | 28.37 (17.79 to 47.01) | 5.99 (3.79 to 9.65) |
| Eastern Sub-Saharan Africa | Both | Number of prevalence | 100284.32 (78666.19 to 133969.51) | 240493.98 (176668.88 to 335241.61) | 280772.61 (199785.21 to 410280.58) | 301179.62 (207856.54 to 455171.05) | 289432.28 (194919.70 to 443492.87) | 260425.50 (172932.11 to 404275.95) | 228635.89 (149987.92 to 361630.81) | 200538.11 (131467.37 to 320530.17) | 170879.46 (111893.15 to 273592.66) | 138779.72 (90584.57 to 221829.42) | 107779.15 (70509.45 to 170955.19) | 83306.51 (54257.79 to 131913.35) | 65305.32 (42216.48 to 104931.88) | 48935.10 (31752.56 to 80768.97) | 32311.23 (20897.30 to 52868.25) | 20050.17 (12992.19 to 32535.71) | 10277.01 (6685.47 to 16687.13) | 4133.19 (2698.30 to 6693.32) | 1157.36 (759.71 to 1889.61) | 261.68 (171.42 to 432.62) |
| Eastern Sub-Saharan Africa | Female | Number of DALYs | 3071.50 (2086.86 to 4454.78) | 4904.83 (3261.23 to 7091.96) | 4593.08 (3107.74 to 6617.05) | 4177.12 (2793.81 to 6019.19) | 3580.00 (2455.37 to 5021.38) | 2966.83 (1999.56 to 4219.77) | 2443.86 (1628.20 to 3411.01) | 2025.99 (1376.93 to 2834.23) | 1637.99 (1098.02 to 2279.51) | 1261.00 (856.10 to 1760.01) | 949.46 (643.75 to 1359.19) | 724.81 (495.29 to 1014.34) | 555.73 (379.65 to 766.01) | 419.58 (287.56 to 600.14) | 273.98 (187.32 to 379.74) | 174.28 (120.97 to 237.43) | 94.78 (64.88 to 131.69) | 40.01 (27.00 to 54.88) | 11.83 (8.26 to 16.03) | 2.93 (2.06 to 3.98) |
| Eastern Sub-Saharan Africa | Female | Number of prevalence | 53791.29 (42785.65 to 70238.12) | 104414.85 (78338.51 to 146448.21) | 121524.26 (86365.72 to 181476.29) | 134098.93 (91400.81 to 208871.65) | 134104.13 (88633.26 to 214399.89) | 122700.00 (79573.97 to 198828.09) | 107389.30 (68940.97 to 173644.91) | 93781.01 (59910.40 to 153465.07) | 78602.59 (50213.74 to 129292.74) | 62606.94 (39872.72 to 102229.57) | 48636.85 (30917.31 to 79725.62) | 37972.21 (23972.05 to 62978.78) | 30202.34 (18977.21 to 50822.71) | 23138.88 (14658.58 to 39433.59) | 15463.33 (9855.52 to 26492.94) | 9886.18 (6285.24 to 16774.61) | 5327.46 (3389.30 to 8902.21) | 2239.65 (1425.00 to 3725.23) | 665.05 (425.75 to 1114.60) | 168.32 (109.04 to 281.22) |
| Eastern Sub-Saharan Africa | Male | Number of DALYs | 2254.18 (1488.01 to 3284.80) | 8967.02 (4341.78 to 18592.92) | 9335.29 (4503.50 to 19234.50) | 8622.88 (4068.36 to 17374.51) | 7252.09 (3495.71 to 14787.81) | 6019.45 (2908.55 to 12158.89) | 5076.37 (2432.60 to 10281.13) | 4312.08 (2088.74 to 8721.06) | 3629.93 (1730.87 to 7305.50) | 2916.92 (1414.52 to 5780.50) | 2218.39 (1067.03 to 4381.85) | 1667.71 (824.15 to 3334.75) | 1261.93 (612.64 to 2508.03) | 914.78 (453.94 to 1818.78) | 587.52 (286.53 to 1144.01) | 352.14 (176.08 to 674.99) | 170.58 (84.58 to 332.97) | 64.76 (32.40 to 127.32) | 16.54 (8.21 to 32.08) | 3.06 (1.54 to 6.02) |
| Eastern Sub-Saharan Africa | Male | Number of prevalence | 46493.03 (35367.41 to 63651.30) | 136079.13 (93641.97 to 208742.28) | 159248.35 (106698.17 to 245687.05) | 167080.69 (111022.69 to 255891.14) | 155328.16 (101136.84 to 239489.33) | 137725.50 (87600.69 to 213257.70) | 121246.59 (77219.52 to 190954.85) | 106757.10 (68408.23 to 169427.73) | 92276.87 (59215.82 to 146892.82) | 76172.78 (48773.69 to 120479.55) | 59142.30 (38049.94 to 93420.58) | 45334.30 (29326.82 to 71314.35) | 35102.98 (22851.36 to 55767.99) | 25796.22 (16886.10 to 41667.04) | 16847.90 (11010.40 to 27043.83) | 10163.99 (6642.91 to 16159.16) | 4949.55 (3254.06 to 7992.29) | 1893.54 (1242.02 to 3057.75) | 492.31 (322.02 to 792.80) | 93.36 (60.62 to 150.29) |
| High-income Asia Pacific | Both | Number of DALYs | 3.04 (1.79 to 4.56) | 9.18 (4.68 to 17.32) | 9.43 (5.01 to 17.09) | 10.71 (5.87 to 19.42) | 12.86 (6.72 to 23.66) | 13.90 (7.47 to 25.32) | 14.99 (7.56 to 27.12) | 14.40 (7.59 to 26.49) | 12.63 (6.69 to 24.01) | 10.29 (5.29 to 18.70) | 8.35 (4.28 to 15.11) | 6.55 (3.61 to 11.88) | 5.04 (2.72 to 8.85) | 3.48 (2.02 to 5.92) | 2.09 (1.14 to 3.67) | 1.19 (0.66 to 2.12) | 0.66 (0.35 to 1.16) | 0.20 (0.12 to 0.33) | 0.03 (0.02 to 0.04) | 0.00 (0.00 to 0.00) |
| High-income Asia Pacific | Both | Number of prevalence | 63.29 (28.67 to 275.31) | 169.00 (65.32 to 704.85) | 209.44 (67.16 to 982.35) | 281.97 (77.21 to 1433.05) | 376.40 (92.73 to 1996.85) | 443.06 (101.74 to 2410.32) | 495.56 (109.00 to 2732.68) | 491.52 (104.91 to 2734.97) | 445.07 (92.60 to 2498.49) | 380.85 (77.39 to 2157.56) | 321.40 (63.98 to 1835.68) | 260.99 (51.04 to 1498.74) | 211.16 (40.37 to 1221.33) | 150.02 (28.36 to 870.37) | 91.86 (17.33 to 533.50) | 52.70 (9.96 to 305.31) | 30.53 (5.75 to 177.50) | 10.53 (1.91 to 62.54) | 1.99 (0.33 to 12.22) | 0.11 (0.02 to 0.66) |
| High-income Asia Pacific | Female | Number of DALYs | 1.72 (1.02 to 2.69) | 2.96 (1.65 to 4.62) | 2.90 (1.70 to 4.59) | 3.13 (1.87 to 4.71) | 3.63 (2.07 to 5.59) | 3.87 (2.24 to 6.13) | 4.05 (2.37 to 6.18) | 3.74 (2.14 to 5.71) | 3.31 (2.00 to 5.14) | 2.86 (1.67 to 4.61) | 2.40 (1.39 to 3.63) | 1.86 (1.06 to 2.93) | 1.50 (0.92 to 2.29) | 1.03 (0.62 to 1.58) | 0.64 (0.39 to 0.99) | 0.35 (0.21 to 0.51) | 0.21 (0.12 to 0.31) | 0.09 (0.05 to 0.14) | 0.02 (0.01 to 0.04) | 0.00 (0.00 to 0.00) |
| High-income Asia Pacific | Female | Number of prevalence | 32.86 (15.93 to 133.93) | 70.58 (27.92 to 329.44) | 87.27 (27.65 to 453.25) | 116.97 (30.23 to 652.74) | 155.02 (34.86 to 898.74) | 185.48 (38.36 to 1098.49) | 204.88 (39.94 to 1228.78) | 199.87 (37.27 to 1209.55) | 183.92 (33.17 to 1120.65) | 164.92 (28.82 to 1010.49) | 145.45 (24.75 to 895.48) | 118.16 (19.62 to 731.02) | 98.08 (15.81 to 609.83) | 70.39 (11.20 to 438.61) | 43.83 (6.97 to 273.15) | 24.22 (3.85 to 150.94) | 14.83 (2.35 to 92.39) | 6.71 (1.06 to 41.83) | 1.79 (0.28 to 11.15) | 0.08 (0.01 to 0.53) |
| High-income Asia Pacific | Male | Number of DALYs | 1.32 (0.70 to 2.20) | 6.22 (2.57 to 13.12) | 6.53 (2.93 to 13.85) | 7.59 (3.32 to 15.38) | 9.23 (4.03 to 19.27) | 10.03 (4.60 to 20.76) | 10.95 (4.70 to 23.25) | 10.66 (4.86 to 21.98) | 9.32 (4.29 to 19.37) | 7.42 (3.14 to 15.60) | 5.95 (2.59 to 12.17) | 4.68 (2.16 to 9.54) | 3.54 (1.60 to 6.97) | 2.45 (1.19 to 4.86) | 1.45 (0.65 to 2.87) | 0.84 (0.38 to 1.74) | 0.45 (0.21 to 0.93) | 0.11 (0.05 to 0.22) | 0.01 (0.00 to 0.01) | 0.00 (0.00 to 0.00) |
| High-income Asia Pacific | Male | Number of prevalence | 30.43 (12.26 to 141.38) | 98.42 (34.92 to 375.41) | 122.17 (37.17 to 529.10) | 165.00 (44.00 to 780.31) | 221.39 (54.06 to 1098.11) | 257.58 (59.75 to 1311.83) | 290.68 (65.09 to 1503.91) | 291.64 (63.77 to 1525.42) | 261.15 (55.86 to 1377.83) | 215.92 (45.33 to 1147.07) | 175.95 (36.53 to 940.20) | 142.83 (29.08 to 767.72) | 113.08 (22.71 to 611.50) | 79.63 (15.85 to 431.75) | 48.02 (9.55 to 260.36) | 28.47 (5.67 to 154.37) | 15.70 (3.12 to 85.11) | 3.82 (0.76 to 20.71) | 0.20 (0.04 to 1.06) | 0.02 (0.00 to 0.13) |
| North Africa and Middle East | Both | Number of DALYs | 985.62 (637.89 to 1441.98) | 2850.47 (1565.31 to 5169.61) | 2757.51 (1489.46 to 4944.59) | 2448.42 (1346.50 to 4540.22) | 2094.58 (1146.71 to 3878.31) | 1849.65 (1003.46 to 3453.03) | 1654.26 (924.65 to 2981.86) | 1412.41 (786.19 to 2502.94) | 1154.57 (654.57 to 1963.56) | 885.35 (473.05 to 1617.23) | 683.29 (374.04 to 1252.93) | 518.26 (285.63 to 953.84) | 388.14 (212.71 to 709.51) | 288.30 (158.61 to 514.29) | 199.33 (110.13 to 361.25) | 125.12 (68.80 to 219.09) | 67.28 (37.75 to 120.15) | 28.01 (16.03 to 48.61) | 8.72 (4.94 to 15.41) | 2.47 (1.43 to 4.28) |
| North Africa and Middle East | Both | Number of prevalence | 30016.70 (12798.02 to 81054.26) | 74508.86 (31517.20 to 195714.01) | 89164.39 (33374.67 to 247504.49) | 98700.30 (32122.78 to 288662.84) | 103403.78 (29909.36 to 314431.04) | 103307.22 (28485.54 to 319589.97) | 101087.83 (26863.03 to 323823.84) | 96890.80 (24466.46 to 313256.55) | 83477.22 (20568.15 to 271602.69) | 68147.90 (16309.61 to 223907.80) | 58075.14 (13311.16 to 192458.39) | 49502.78 (10855.77 to 167830.33) | 40028.08 (8478.57 to 132571.67) | 29295.99 (6319.67 to 97772.73) | 18073.17 (4130.73 to 59902.72) | 10299.76 (2493.51 to 33944.74) | 5160.45 (1300.46 to 16397.76) | 2024.23 (527.18 to 6664.88) | 627.27 (166.84 to 2005.37) | 225.97 (54.22 to 747.50) |
| North Africa and Middle East | Female | Number of DALYs | 562.32 (358.41 to 841.71) | 944.00 (586.19 to 1374.89) | 895.92 (535.38 to 1350.09) | 780.52 (492.91 to 1194.45) | 660.43 (411.26 to 1003.47) | 590.39 (371.02 to 914.92) | 542.23 (343.62 to 800.02) | 472.13 (299.39 to 703.23) | 375.80 (236.98 to 550.96) | 270.09 (172.67 to 410.34) | 201.97 (127.61 to 300.85) | 150.26 (96.87 to 226.33) | 109.73 (71.09 to 161.88) | 82.98 (54.52 to 122.00) | 53.06 (34.00 to 78.27) | 34.94 (22.62 to 49.99) | 18.96 (12.23 to 26.89) | 8.02 (5.21 to 11.57) | 2.62 (1.68 to 3.83) | 0.85 (0.56 to 1.20) |
| North Africa and Middle East | Female | Number of prevalence | 15401.41 (7134.32 to 40329.23) | 32801.80 (12920.81 to 91523.86) | 39599.36 (13563.27 to 115541.34) | 44532.24 (13318.97 to 135895.84) | 47818.04 (12697.23 to 149766.12) | 49064.51 (12250.23 to 156397.24) | 48469.43 (11766.47 to 157744.72) | 46703.05 (10891.92 to 154588.76) | 39866.43 (9006.34 to 132793.29) | 31861.02 (6892.34 to 106962.81) | 26853.81 (5525.56 to 90906.49) | 22774.31 (4538.63 to 76682.83) | 18106.91 (3539.56 to 61171.04) | 13191.44 (2626.83 to 43948.64) | 8000.29 (1623.49 to 27366.63) | 4585.02 (989.33 to 15366.06) | 2165.57 (502.04 to 7103.85) | 691.55 (188.12 to 2132.87) | 167.71 (48.27 to 555.47) | 54.32 (14.94 to 193.21) |
| North Africa and Middle East | Male | Number of DALYs | 423.29 (252.58 to 656.88) | 1906.47 (888.22 to 3911.46) | 1861.59 (844.49 to 3907.98) | 1667.90 (786.85 to 3619.69) | 1434.15 (637.29 to 3102.81) | 1259.25 (555.72 to 2640.16) | 1112.03 (495.77 to 2291.24) | 940.28 (435.36 to 1961.57) | 778.76 (363.07 to 1531.39) | 615.26 (263.16 to 1270.31) | 481.32 (229.78 to 976.08) | 368.00 (170.54 to 762.83) | 278.41 (129.90 to 569.60) | 205.32 (95.96 to 421.05) | 146.28 (66.08 to 290.48) | 90.18 (43.17 to 180.16) | 48.32 (22.53 to 98.60) | 19.99 (9.33 to 38.26) | 6.10 (2.82 to 12.52) | 1.62 (0.78 to 3.30) |
| North Africa and Middle East | Male | Number of prevalence | 14615.29 (5794.98 to 40649.92) | 41707.06 (17435.77 to 104208.08) | 49565.02 (19010.83 to 132629.26) | 54168.07 (19007.78 to 153272.91) | 55585.74 (17584.52 to 163734.87) | 54242.71 (16393.54 to 162605.24) | 52618.40 (15151.94 to 165179.34) | 50187.75 (13598.49 to 158900.14) | 43610.79 (11594.45 to 139393.79) | 36286.88 (9374.60 to 116944.98) | 31221.32 (7771.05 to 102065.18) | 26728.47 (6398.81 to 90360.80) | 21921.17 (5113.28 to 73434.35) | 16104.55 (3804.37 to 54252.57) | 10072.88 (2495.90 to 32921.96) | 5714.73 (1465.31 to 18344.05) | 2994.88 (786.86 to 9372.93) | 1332.68 (336.78 to 4229.12) | 459.56 (110.44 to 1527.58) | 171.64 (35.41 to 587.64) |
| Oceania | Both | Number of DALYs | 191.53 (121.23 to 274.20) | 912.15 (494.30 to 1586.84) | 1171.66 (659.78 to 1976.44) | 1523.30 (896.05 to 2483.99) | 1927.88 (1217.09 to 2937.49) | 2120.94 (1348.65 to 3229.50) | 2054.20 (1319.15 to 2997.78) | 1968.02 (1277.03 to 2896.86) | 1811.15 (1196.64 to 2600.12) | 1604.23 (1051.52 to 2296.06) | 1387.04 (915.48 to 1976.80) | 1157.53 (769.95 to 1642.78) | 864.98 (584.68 to 1235.40) | 586.94 (393.53 to 822.78) | 358.87 (244.13 to 500.68) | 202.83 (138.12 to 284.54) | 90.25 (61.88 to 125.21) | 32.16 (21.95 to 44.63) | 8.86 (6.18 to 12.14) | 1.91 (1.34 to 2.60) |
| Oceania | Both | Number of prevalence | 21328.59 (6363.29 to 54730.98) | 50185.45 (18108.08 to 122694.62) | 64218.45 (23036.37 to 157129.20) | 77798.25 (28173.78 to 189088.02) | 93136.01 (34296.17 to 225414.06) | 99187.77 (36903.83 to 239195.87) | 95223.04 (35950.88 to 228856.44) | 89289.63 (34471.06 to 213342.45) | 81289.61 (31813.03 to 193332.15) | 70826.77 (28179.39 to 167621.70) | 60123.06 (24554.35 to 139938.34) | 50103.90 (20738.06 to 113979.35) | 37414.33 (15729.10 to 83633.27) | 25744.68 (10831.40 to 57499.72) | 16227.75 (6834.24 to 36035.99) | 9472.65 (3976.66 to 20931.49) | 4370.30 (1853.87 to 9506.78) | 1638.86 (689.16 to 3592.00) | 487.21 (197.34 to 1118.51) | 112.82 (42.19 to 267.76) |
| Oceania | Female | Number of DALYs | 108.11 (66.77 to 157.92) | 172.81 (105.80 to 265.30) | 168.50 (97.85 to 261.48) | 166.37 (104.73 to 246.52) | 175.94 (107.95 to 264.38) | 180.35 (113.89 to 261.83) | 171.45 (109.55 to 246.95) | 153.49 (97.23 to 224.72) | 135.69 (85.28 to 203.80) | 115.19 (73.93 to 174.24) | 93.56 (61.99 to 137.14) | 74.83 (48.20 to 107.49) | 54.44 (35.47 to 77.49) | 36.87 (23.71 to 52.29) | 23.27 (15.01 to 33.26) | 13.76 (9.32 to 19.79) | 6.44 (4.24 to 8.86) | 2.51 (1.69 to 3.47) | 0.79 (0.55 to 1.11) | 0.19 (0.13 to 0.26) |
| Oceania | Female | Number of prevalence | 10355.40 (3189.53 to 26321.22) | 21861.51 (6470.30 to 56280.38) | 27337.48 (7865.07 to 70919.35) | 32412.15 (9095.34 to 84596.19) | 38651.68 (10542.89 to 101628.13) | 41782.92 (11324.08 to 110069.39) | 40881.38 (11135.33 to 107610.56) | 37889.01 (10443.73 to 99321.41) | 34065.87 (9435.66 to 89129.15) | 29086.62 (8138.42 to 75902.01) | 23835.16 (6786.41 to 61026.74) | 19537.21 (5712.38 to 48639.98) | 14392.28 (4361.85 to 35026.99) | 9888.36 (3041.60 to 23809.65) | 6333.21 (1975.52 to 15095.06) | 3789.83 (1192.97 to 8936.99) | 1809.96 (587.88 to 4176.39) | 720.09 (237.33 to 1656.04) | 232.90 (73.11 to 550.88) | 57.76 (17.10 to 146.17) |
| Oceania | Male | Number of DALYs | 83.42 (47.90 to 129.01) | 739.34 (378.37 to 1384.69) | 1003.16 (526.68 to 1798.32) | 1356.93 (763.63 to 2281.03) | 1751.94 (1063.52 to 2729.20) | 1940.59 (1213.37 to 2971.99) | 1882.75 (1188.30 to 2796.40) | 1814.52 (1177.17 to 2695.20) | 1675.46 (1097.41 to 2432.56) | 1489.05 (964.24 to 2143.31) | 1293.48 (846.00 to 1862.50) | 1082.71 (718.37 to 1542.11) | 810.54 (546.55 to 1167.70) | 550.08 (368.79 to 777.31) | 335.61 (224.18 to 469.43) | 189.07 (128.80 to 267.22) | 83.81 (56.91 to 117.05) | 29.65 (20.20 to 41.59) | 8.07 (5.52 to 11.18) | 1.72 (1.19 to 2.35) |
| Oceania | Male | Number of prevalence | 10973.19 (3161.12 to 28409.76) | 28323.93 (11327.52 to 66377.58) | 36880.97 (15048.29 to 86133.11) | 45386.11 (19255.21 to 104558.73) | 54484.33 (23699.72 to 123680.19) | 57404.85 (25466.35 to 128632.05) | 54341.67 (24751.52 to 120436.90) | 51400.62 (23934.06 to 112802.54) | 47223.74 (22285.99 to 102959.23) | 41740.16 (19836.61 to 90578.78) | 36287.90 (17540.66 to 77858.87) | 30566.69 (14969.79 to 64562.26) | 23022.06 (11368.14 to 48259.79) | 15856.32 (7820.66 to 33376.31) | 9894.54 (4879.87 to 20802.28) | 5682.82 (2788.24 to 11944.22) | 2560.35 (1264.13 to 5357.29) | 918.77 (449.35 to 1940.11) | 254.31 (118.85 to 550.46) | 55.06 (24.99 to 121.60) |
| South Asia | Both | Number of DALYs | 12988.73 (8762.83 to 18353.02) | 44922.60 (25448.02 to 80355.43) | 56285.58 (31370.59 to 100314.40) | 65540.30 (36802.93 to 115864.10) | 71566.87 (40949.03 to 124203.63) | 70913.26 (40350.16 to 120172.10) | 68389.13 (38664.98 to 116149.37) | 64467.77 (37054.34 to 108981.79) | 57331.01 (33198.31 to 94919.12) | 50721.90 (29601.02 to 84650.45) | 43338.26 (25348.12 to 71759.72) | 35868.97 (20980.66 to 58411.38) | 30964.05 (18671.10 to 49593.50) | 25000.83 (15185.24 to 40110.10) | 17902.97 (10808.39 to 28505.50) | 10850.88 (6699.49 to 17314.48) | 5701.58 (3542.63 to 8927.23) | 2224.52 (1384.70 to 3506.48) | 619.99 (388.52 to 979.40) | 108.46 (68.97 to 167.64) |
| South Asia | Both | Number of prevalence | 532753.53 (474053.37 to 597574.12) | 1458436.24 (1260338.87 to 1756603.37) | 2157570.90 (1856937.75 to 2570806.01) | 2864364.11 (2470176.24 to 3393150.95) | 3330642.89 (2878168.80 to 3906597.06) | 3377635.36 (2929091.80 to 3938729.31) | 3289780.63 (2871089.23 to 3824030.39) | 3156187.00 (2764459.87 to 3655183.74) | 2833497.95 (2481631.22 to 3273433.58) | 2519865.23 (2208406.02 to 2904348.08) | 2183585.19 (1919644.93 to 2504863.45) | 1846524.15 (1628378.62 to 2112197.99) | 1632999.93 (1439799.47 to 1864827.31) | 1348621.60 (1187794.88 to 1536964.25) | 990902.22 (870930.34 to 1128267.61) | 617877.69 (543305.05 to 702962.71) | 342779.70 (302157.37 to 390817.39) | 139368.48 (123571.34 to 158542.16) | 40248.77 (35517.30 to 45693.55) | 8310.49 (7283.08 to 9573.26) |
| South Asia | Female | Number of DALYs | 7297.62 (4848.43 to 10304.78) | 13346.89 (9157.07 to 18965.59) | 14699.09 (9822.20 to 20962.97) | 15390.44 (10417.20 to 21660.02) | 15533.74 (10417.98 to 21932.08) | 14725.54 (10165.27 to 20843.31) | 13720.10 (9263.39 to 19282.51) | 12582.58 (8630.58 to 17727.08) | 10928.30 (7402.55 to 15321.96) | 9427.07 (6462.46 to 13167.71) | 8098.04 (5648.09 to 11267.32) | 6782.45 (4674.13 to 9712.43) | 5809.56 (3986.26 to 8258.54) | 4612.50 (3158.68 to 6440.67) | 3306.97 (2275.54 to 4528.63) | 2056.99 (1410.97 to 2842.24) | 1219.70 (845.24 to 1695.84) | 524.38 (368.40 to 707.11) | 158.28 (113.16 to 213.52) | 37.13 (26.77 to 49.95) |
| South Asia | Female | Number of prevalence | 264220.81 (235652.65 to 295013.01) | 633552.36 (559863.85 to 710803.52) | 933586.10 (819180.01 to 1053777.20) | 1242818.57 (1085300.01 to 1410501.04) | 1459404.89 (1271810.40 to 1660623.97) | 1486085.28 (1298780.30 to 1688938.05) | 1441125.95 (1263765.12 to 1635450.91) | 1388074.29 (1218839.81 to 1574224.18) | 1244079.58 (1091400.93 to 1410607.52) | 1099138.18 (964094.40 to 1245999.90) | 964449.30 (847535.67 to 1093079.02) | 831081.82 (731397.39 to 942607.34) | 747902.76 (658128.00 to 849412.00) | 620997.51 (545892.40 to 705442.40) | 458615.22 (403283.71 to 520596.18) | 289449.54 (254932.92 to 327893.62) | 172216.67 (152155.01 to 194422.28) | 73253.04 (64977.13 to 82758.17) | 21936.10 (19435.25 to 24815.01) | 5376.42 (4763.02 to 6079.12) |
| South Asia | Male | Number of DALYs | 5691.11 (3746.58 to 8141.93) | 31575.71 (15165.23 to 63143.53) | 41586.49 (20097.03 to 81119.96) | 50149.86 (25029.49 to 96369.19) | 56033.13 (28492.96 to 105277.22) | 56187.72 (29251.43 to 103040.95) | 54669.03 (28294.96 to 98604.01) | 51885.19 (27145.73 to 93236.24) | 46402.70 (24325.34 to 82714.50) | 41294.83 (22607.61 to 73758.26) | 35240.23 (19117.97 to 62135.39) | 29086.52 (15855.86 to 50693.12) | 25154.49 (13937.15 to 42999.43) | 20388.33 (11532.48 to 34741.50) | 14596.00 (8192.06 to 24637.87) | 8793.89 (4978.65 to 14931.42) | 4481.88 (2525.75 to 7514.85) | 1700.14 (965.13 to 2905.44) | 461.71 (262.98 to 790.04) | 71.33 (39.92 to 123.76) |
| South Asia | Male | Number of prevalence | 268532.72 (236971.22 to 303211.98) | 824883.88 (677293.64 to 1081442.92) | 1223984.80 (1017271.24 to 1566685.85) | 1621545.54 (1360790.71 to 2008966.61) | 1871238.00 (1574654.35 to 2286929.71) | 1891550.08 (1594954.85 to 2294979.35) | 1848654.68 (1567316.11 to 2232040.18) | 1768112.71 (1505654.27 to 2125238.68) | 1589418.37 (1354558.52 to 1908541.19) | 1420727.04 (1212487.16 to 1704801.87) | 1219135.88 (1042994.23 to 1460563.15) | 1015442.33 (871524.11 to 1214966.48) | 885097.17 (761010.82 to 1057339.12) | 727624.09 (626136.14 to 866872.44) | 532287.00 (458046.51 to 632391.38) | 328428.15 (282388.72 to 389980.49) | 170563.03 (146776.61 to 202910.50) | 66115.45 (56980.44 to 78713.76) | 18312.67 (15756.35 to 21871.75) | 2934.07 (2501.19 to 3597.86) |
| Southeast Asia | Both | Number of DALYs | 4931.30 (3333.14 to 7066.39) | 15192.14 (8483.11 to 27969.18) | 15919.23 (8964.15 to 28646.48) | 16502.00 (9277.30 to 29442.58) | 17274.08 (9703.85 to 30524.67) | 18030.06 (9985.18 to 31947.15) | 17993.74 (10133.38 to 31738.65) | 16983.67 (9657.65 to 29876.90) | 15940.28 (9113.59 to 28012.22) | 14309.69 (8240.68 to 25246.01) | 12685.20 (7308.44 to 22327.00) | 10716.32 (6190.56 to 18774.50) | 8618.08 (4928.36 to 14881.63) | 6198.52 (3635.17 to 10452.00) | 3756.25 (2215.81 to 6355.62) | 2137.08 (1291.76 to 3507.00) | 1112.99 (687.47 to 1840.28) | 471.42 (292.02 to 772.15) | 146.13 (90.16 to 237.40) | 34.19 (21.16 to 55.61) |
| Southeast Asia | Both | Number of prevalence | 129736.94 (78991.74 to 225668.79) | 339679.65 (199114.24 to 615420.10) | 440669.68 (244045.12 to 771235.03) | 543881.83 (290797.22 to 989290.60) | 633494.31 (324394.41 to 1191034.08) | 699230.04 (352029.81 to 1332105.37) | 723123.99 (356965.58 to 1410135.01) | 713707.64 (346887.84 to 1427784.70) | 695679.41 (333258.80 to 1432492.49) | 647926.50 (305378.53 to 1366499.42) | 593862.10 (278656.92 to 1264498.35) | 519152.22 (243193.11 to 1120687.19) | 433358.85 (203556.08 to 958786.23) | 323949.10 (152307.02 to 722763.48) | 205543.41 (97015.13 to 458738.66) | 122997.23 (57484.16 to 275108.56) | 67897.93 (31847.18 to 160509.48) | 30772.08 (13951.31 to 71291.70) | 10456.85 (4608.23 to 24414.84) | 2720.19 (1205.80 to 6821.18) |
| Southeast Asia | Female | Number of DALYs | 2801.63 (1871.85 to 3995.57) | 4838.92 (3222.67 to 6951.51) | 4823.89 (3239.47 to 6928.62) | 4738.46 (3206.62 to 6712.05) | 4770.68 (3202.90 to 6741.27) | 4854.84 (3304.73 to 6993.33) | 4752.77 (3254.02 to 6795.42) | 4413.12 (3010.99 to 6320.28) | 4116.94 (2761.79 to 5813.19) | 3663.26 (2459.92 to 5278.20) | 3281.04 (2215.62 to 4627.39) | 2798.35 (1884.59 to 4007.27) | 2266.57 (1502.31 to 3183.76) | 1676.19 (1148.94 to 2356.18) | 1050.11 (724.68 to 1433.68) | 645.90 (434.60 to 895.90) | 376.24 (263.71 to 525.95) | 174.14 (120.68 to 239.12) | 56.49 (38.05 to 78.19) | 12.82 (8.78 to 17.79) |
| Southeast Asia | Female | Number of prevalence | 67070.86 (42255.10 to 113637.26) | 146084.67 (83176.78 to 288945.59) | 192187.15 (102603.42 to 363036.02) | 240095.11 (120325.56 to 467129.30) | 282341.15 (135508.11 to 566718.80) | 313559.47 (148335.11 to 637599.26) | 324860.41 (152009.37 to 678830.08) | 322409.56 (148002.48 to 691121.07) | 316419.98 (141107.63 to 699024.34) | 297085.50 (131082.00 to 671221.46) | 274728.95 (121433.87 to 624313.55) | 243463.34 (105611.46 to 558360.39) | 205789.25 (88061.22 to 479515.19) | 157166.27 (66230.25 to 366861.05) | 102305.24 (44466.28 to 238259.53) | 64491.32 (28234.40 to 151345.66) | 37760.56 (16151.78 to 92303.60) | 18022.21 (7461.58 to 42889.09) | 6327.69 (2557.98 to 15637.46) | 1625.47 (603.32 to 4318.95) |
| Southeast Asia | Male | Number of DALYs | 2129.67 (1407.93 to 3193.74) | 10353.21 (5061.56 to 21527.34) | 11095.34 (5262.80 to 22363.80) | 11763.54 (5737.11 to 23395.13) | 12503.40 (6092.84 to 24392.67) | 13175.22 (6562.39 to 25817.69) | 13240.98 (6540.10 to 26089.65) | 12570.55 (6224.64 to 24895.38) | 11823.33 (5985.82 to 23035.77) | 10646.44 (5286.98 to 20303.73) | 9404.16 (4758.42 to 17944.35) | 7917.97 (4052.64 to 15276.58) | 6351.51 (3182.91 to 12059.92) | 4522.33 (2361.96 to 8494.03) | 2706.15 (1395.82 to 5088.85) | 1491.18 (782.49 to 2776.43) | 736.75 (391.59 to 1372.00) | 297.28 (156.17 to 562.60) | 89.64 (46.89 to 167.73) | 21.37 (11.33 to 39.21) |
| Southeast Asia | Male | Number of prevalence | 62666.07 (36094.63 to 112406.05) | 193594.98 (109495.62 to 348429.80) | 248482.53 (137893.94 to 435235.35) | 303786.72 (162994.67 to 534798.04) | 351153.16 (184754.99 to 630226.47) | 385670.57 (201708.01 to 706096.27) | 398263.58 (207984.13 to 743445.90) | 391298.08 (202803.55 to 737092.05) | 379259.42 (196648.81 to 733873.82) | 350841.00 (180113.90 to 695379.69) | 319133.15 (161867.45 to 640359.88) | 275688.88 (138687.91 to 562403.25) | 227569.60 (113337.37 to 479612.69) | 166782.83 (82420.52 to 356263.09) | 103238.17 (50060.25 to 220479.12) | 58505.90 (28417.26 to 122453.86) | 30137.37 (14756.85 to 67267.56) | 12749.87 (6239.53 to 27577.84) | 4129.15 (2028.36 to 8920.59) | 1094.72 (549.51 to 2430.96) |
| Southern Sub-Saharan Africa | Both | Number of DALYs | 213.86 (135.69 to 318.85) | 592.02 (306.55 to 1067.30) | 564.42 (311.50 to 1022.00) | 501.29 (265.83 to 919.85) | 436.81 (236.44 to 801.03) | 364.14 (202.73 to 654.91) | 317.86 (167.74 to 575.79) | 291.08 (160.87 to 519.86) | 246.08 (131.04 to 438.29) | 190.52 (105.70 to 356.16) | 138.38 (78.73 to 239.01) | 100.21 (55.26 to 177.65) | 82.17 (46.47 to 136.83) | 66.18 (37.15 to 115.30) | 40.11 (23.34 to 67.58) | 21.13 (12.42 to 35.20) | 8.98 (5.29 to 14.99) | 3.01 (1.68 to 5.00) | 0.68 (0.39 to 1.15) | 0.12 (0.07 to 0.20) |
| Southern Sub-Saharan Africa | Both | Number of prevalence | 4349.95 (2257.51 to 11827.17) | 10825.63 (5205.05 to 28275.20) | 12390.04 (5222.33 to 35895.97) | 13090.46 (4871.05 to 40885.93) | 12984.52 (4439.49 to 42342.98) | 11779.05 (3825.00 to 39371.41) | 10862.41 (3416.71 to 36835.67) | 10407.65 (3206.12 to 35616.02) | 9077.83 (2749.12 to 31241.39) | 7218.73 (2155.39 to 24949.67) | 5443.47 (1600.12 to 18949.98) | 4138.85 (1191.81 to 14565.49) | 3569.74 (1000.04 to 12684.76) | 2962.50 (822.03 to 10556.58) | 1832.93 (506.29 to 6538.13) | 989.04 (273.87 to 3525.40) | 428.28 (118.91 to 1526.14) | 144.68 (40.12 to 515.58) | 34.04 (9.30 to 121.75) | 6.49 (1.73 to 23.43) |
| Southern Sub-Saharan Africa | Female | Number of DALYs | 125.31 (75.61 to 195.00) | 200.73 (114.18 to 318.97) | 186.95 (108.23 to 288.32) | 165.32 (95.45 to 258.27) | 146.37 (87.55 to 230.56) | 123.50 (75.30 to 183.78) | 108.24 (63.71 to 170.91) | 96.44 (59.83 to 140.94) | 78.76 (46.03 to 124.96) | 58.86 (35.13 to 91.32) | 44.40 (26.43 to 68.43) | 36.06 (21.91 to 54.33) | 31.80 (17.13 to 50.10) | 25.57 (15.24 to 38.45) | 16.01 (9.70 to 24.51) | 8.29 (5.18 to 12.86) | 3.48 (2.08 to 5.21) | 1.17 (0.67 to 1.71) | 0.28 (0.17 to 0.44) | 0.06 (0.04 to 0.09) |
| Southern Sub-Saharan Africa | Female | Number of prevalence | 2336.00 (1290.35 to 6018.13) | 4690.23 (2198.73 to 13596.64) | 5495.15 (2151.84 to 17506.72) | 5974.86 (1998.55 to 20308.58) | 6150.04 (1855.63 to 21657.62) | 5753.86 (1626.99 to 20666.19) | 5375.95 (1451.34 to 19541.87) | 5127.34 (1339.54 to 18792.15) | 4364.75 (1112.26 to 16101.12) | 3391.30 (846.16 to 12575.04) | 2637.20 (645.49 to 9822.13) | 2185.12 (524.45 to 8174.04) | 2002.48 (470.46 to 7527.56) | 1673.30 (390.57 to 6302.54) | 1049.95 (244.78 to 3954.62) | 561.22 (130.71 to 2113.90) | 241.97 (56.48 to 911.32) | 81.78 (19.05 to 308.03) | 20.19 (4.71 to 76.06) | 4.31 (1.00 to 16.25) |
| Southern Sub-Saharan Africa | Male | Number of DALYs | 88.55 (48.79 to 142.35) | 391.29 (168.52 to 834.22) | 377.46 (172.41 to 795.56) | 335.98 (149.84 to 699.92) | 290.44 (125.06 to 611.94) | 240.64 (108.89 to 489.79) | 209.62 (89.59 to 433.60) | 194.64 (85.07 to 405.98) | 167.32 (75.21 to 345.65) | 131.66 (61.29 to 280.13) | 93.98 (43.66 to 185.54) | 64.15 (28.09 to 130.48) | 50.38 (24.30 to 103.33) | 40.61 (18.03 to 80.12) | 24.10 (10.86 to 46.91) | 12.84 (6.06 to 25.53) | 5.50 (2.68 to 11.15) | 1.84 (0.82 to 3.73) | 0.40 (0.19 to 0.78) | 0.06 (0.03 to 0.12) |
| Southern Sub-Saharan Africa | Male | Number of prevalence | 2013.94 (957.04 to 5811.61) | 6135.40 (2734.10 to 15443.25) | 6894.90 (2775.63 to 18707.08) | 7115.60 (2666.82 to 20687.43) | 6834.47 (2428.91 to 20725.33) | 6025.19 (2070.17 to 18722.29) | 5486.46 (1840.67 to 17310.71) | 5280.31 (1741.75 to 16838.79) | 4713.08 (1534.21 to 15152.65) | 3827.44 (1233.73 to 12384.17) | 2806.27 (894.65 to 9134.59) | 1953.73 (615.97 to 6395.86) | 1567.26 (488.56 to 5160.67) | 1289.20 (400.10 to 4256.77) | 782.98 (243.46 to 2585.13) | 427.82 (133.04 to 1412.41) | 186.31 (57.82 to 615.21) | 62.90 (19.54 to 207.69) | 13.85 (4.30 to 45.72) | 2.18 (0.68 to 7.19) |
| Tropical Latin America | Both | Number of DALYs | 85.05 (50.28 to 130.58) | 132.25 (82.19 to 193.31) | 126.61 (81.36 to 198.59) | 123.09 (74.76 to 183.56) | 128.63 (81.76 to 197.23) | 123.96 (75.70 to 180.93) | 123.29 (73.49 to 186.77) | 127.04 (79.39 to 192.04) | 117.61 (73.50 to 180.42) | 100.34 (63.70 to 150.46) | 88.48 (53.82 to 129.56) | 75.74 (46.99 to 112.27) | 60.81 (38.95 to 87.55) | 46.80 (29.73 to 68.13) | 36.62 (22.82 to 53.80) | 23.98 (15.08 to 34.97) | 15.62 (9.73 to 23.38) | 7.48 (4.85 to 11.32) | 2.98 (1.87 to 4.23) | 1.27 (0.79 to 1.83) |
| Tropical Latin America | Both | Number of prevalence | 861.81 (791.49 to 1045.56) | 1365.49 (1237.35 to 1790.56) | 1362.72 (1211.52 to 1961.67) | 1366.50 (1188.28 to 2158.90) | 1468.54 (1253.82 to 2475.07) | 1450.25 (1224.79 to 2549.01) | 1480.03 (1232.55 to 2676.79) | 1524.08 (1258.09 to 2815.64) | 1424.57 (1167.61 to 2678.12) | 1246.74 (1016.78 to 2379.58) | 1111.25 (902.30 to 2144.86) | 973.64 (786.81 to 1903.59) | 798.95 (641.36 to 1587.14) | 623.52 (499.00 to 1245.74) | 489.72 (392.41 to 979.00) | 329.66 (263.99 to 656.60) | 216.21 (173.17 to 429.15) | 105.13 (84.51 to 208.24) | 42.55 (34.16 to 83.96) | 18.70 (14.99 to 37.24) |
| Tropical Latin America | Female | Number of DALYs | 48.84 (26.77 to 77.89) | 76.30 (44.04 to 121.46) | 72.30 (42.79 to 119.43) | 69.93 (41.25 to 108.41) | 74.65 (45.09 to 115.06) | 73.30 (40.63 to 112.75) | 74.28 (41.57 to 116.64) | 76.59 (45.50 to 121.25) | 70.32 (40.41 to 111.40) | 61.22 (37.31 to 98.46) | 54.47 (30.24 to 84.64) | 47.78 (26.15 to 73.91) | 38.48 (23.77 to 59.34) | 30.39 (18.12 to 47.00) | 23.77 (13.53 to 36.29) | 16.07 (9.31 to 23.75) | 10.94 (6.46 to 16.93) | 5.37 (3.26 to 8.42) | 2.22 (1.34 to 3.33) | 0.85 (0.52 to 1.28) |
| Tropical Latin America | Female | Number of prevalence | 491.65 (447.41 to 587.94) | 777.96 (700.80 to 995.99) | 775.01 (685.49 to 1076.64) | 778.79 (674.31 to 1177.68) | 844.85 (717.57 to 1362.18) | 848.95 (713.66 to 1427.18) | 878.96 (732.81 to 1520.61) | 905.46 (748.42 to 1600.39) | 845.60 (695.63 to 1520.13) | 747.73 (611.93 to 1364.55) | 672.69 (547.03 to 1242.84) | 598.15 (484.17 to 1120.10) | 497.02 (400.90 to 946.28) | 396.32 (318.09 to 759.48) | 313.25 (251.96 to 600.95) | 217.42 (174.83 to 416.27) | 147.98 (118.74 to 283.34) | 74.52 (59.73 to 142.69) | 31.42 (25.26 to 60.14) | 12.35 (9.92 to 23.66) |
| Tropical Latin America | Male | Number of DALYs | 36.21 (19.35 to 59.57) | 55.94 (30.83 to 90.14) | 54.31 (30.24 to 88.57) | 53.15 (27.37 to 84.22) | 53.97 (27.83 to 89.92) | 50.66 (26.77 to 82.38) | 49.01 (26.80 to 78.29) | 50.46 (27.73 to 81.14) | 47.29 (25.35 to 77.73) | 39.12 (22.28 to 62.68) | 34.02 (19.02 to 53.89) | 27.96 (15.92 to 43.56) | 22.33 (12.42 to 34.62) | 16.41 (9.18 to 26.32) | 12.86 (7.02 to 20.41) | 7.91 (4.50 to 12.53) | 4.69 (2.41 to 7.60) | 2.11 (1.24 to 3.25) | 0.76 (0.43 to 1.21) | 0.41 (0.23 to 0.63) |
| Tropical Latin America | Male | Number of prevalence | 370.16 (328.26 to 460.21) | 587.53 (514.58 to 796.13) | 587.71 (502.88 to 878.17) | 587.71 (490.12 to 967.19) | 623.69 (509.36 to 1103.80) | 601.31 (484.12 to 1114.45) | 601.07 (478.41 to 1149.79) | 618.61 (488.82 to 1210.49) | 578.97 (453.49 to 1155.59) | 499.01 (387.99 to 1012.35) | 438.56 (338.98 to 901.21) | 375.49 (287.98 to 783.49) | 301.93 (230.33 to 640.87) | 227.20 (172.65 to 486.26) | 176.47 (134.18 to 377.53) | 112.24 (85.42 to 240.12) | 68.23 (51.76 to 145.81) | 30.62 (23.28 to 65.54) | 11.13 (8.46 to 23.82) | 6.35 (4.82 to 13.58) |
| Western Sub-Saharan Africa | Both | Number of DALYs | 7345.33 (4939.74 to 10469.37) | 20627.39 (11885.65 to 36376.03) | 20636.40 (11654.21 to 36576.19) | 19694.34 (11291.61 to 34333.97) | 18035.51 (10613.34 to 30376.28) | 16226.45 (9547.67 to 26961.03) | 14532.29 (8818.91 to 23727.52) | 13098.74 (7987.87 to 20940.73) | 11422.77 (7053.27 to 18054.18) | 9411.16 (5897.74 to 14924.47) | 7269.65 (4491.89 to 11390.96) | 5653.74 (3525.61 to 8910.61) | 4284.33 (2722.91 to 6639.67) | 3007.14 (1901.57 to 4692.00) | 1958.53 (1233.45 to 3077.72) | 1198.87 (754.47 to 1860.44) | 621.42 (390.92 to 966.77) | 258.48 (163.45 to 395.14) | 77.35 (49.71 to 118.23) | 17.11 (11.36 to 25.43) |
| Western Sub-Saharan Africa | Both | Number of prevalence | 239006.15 (164319.88 to 355790.68) | 562334.77 (373369.14 to 822669.53) | 668898.60 (435840.09 to 992062.39) | 738293.66 (475166.55 to 1110661.32) | 736060.84 (467118.13 to 1117731.05) | 695239.93 (442934.38 to 1066142.80) | 636652.01 (402122.25 to 972397.31) | 577888.46 (367090.85 to 879586.94) | 501115.56 (319871.27 to 762351.04) | 416209.16 (267163.61 to 636279.58) | 330062.73 (208921.23 to 511632.74) | 264178.78 (168359.71 to 404966.08) | 204375.98 (129993.81 to 314126.06) | 145928.75 (93992.65 to 225140.20) | 97315.02 (61598.74 to 151288.82) | 61068.68 (38715.05 to 96263.85) | 32474.21 (20419.64 to 51846.77) | 13907.98 (8726.78 to 22255.49) | 4433.55 (2736.64 to 7318.84) | 1176.66 (681.92 to 2092.36) |
| Western Sub-Saharan Africa | Female | Number of DALYs | 4251.71 (2877.91 to 6122.96) | 6630.92 (4478.48 to 9601.88) | 6016.77 (4082.48 to 8581.56) | 5346.13 (3560.47 to 7761.07) | 4554.34 (3060.01 to 6476.58) | 3889.76 (2649.62 to 5563.64) | 3247.64 (2186.56 to 4574.87) | 2706.54 (1853.28 to 3793.57) | 2196.50 (1496.85 to 3116.21) | 1771.10 (1209.50 to 2477.97) | 1430.35 (982.36 to 2004.23) | 1134.58 (780.97 to 1568.30) | 850.35 (578.99 to 1190.82) | 579.18 (405.57 to 809.43) | 379.80 (265.47 to 525.73) | 238.24 (167.10 to 321.02) | 127.55 (88.22 to 176.56) | 54.06 (37.90 to 73.60) | 17.06 (11.96 to 23.34) | 5.24 (3.72 to 7.08) |
| Western Sub-Saharan Africa | Female | Number of prevalence | 123880.43 (86154.51 to 182645.97) | 250405.81 (165545.77 to 382318.46) | 300712.79 (190867.63 to 466619.47) | 338926.71 (207979.96 to 529746.08) | 344331.19 (210054.77 to 547532.95) | 329485.36 (199732.85 to 530165.63) | 298916.79 (179410.93 to 480220.47) | 264660.55 (159765.61 to 426929.97) | 220804.85 (132431.10 to 358103.42) | 182162.89 (109200.62 to 297907.40) | 148691.59 (86793.13 to 249641.20) | 122563.67 (71788.37 to 204079.03) | 95694.06 (55974.79 to 161019.83) | 67675.29 (40015.20 to 115487.32) | 44873.30 (26446.68 to 75681.11) | 28162.63 (16468.18 to 47362.21) | 15194.96 (8875.32 to 25270.15) | 6624.37 (3874.86 to 11111.82) | 2219.48 (1288.36 to 3862.78) | 729.79 (386.72 to 1437.78) |
| Western Sub-Saharan Africa | Male | Number of DALYs | 3093.62 (2071.76 to 4477.97) | 13996.47 (6880.11 to 28121.62) | 14619.62 (7087.54 to 28925.42) | 14348.21 (7327.51 to 27469.26) | 13481.17 (7138.66 to 24638.31) | 12336.69 (6593.93 to 22039.00) | 11284.65 (6227.78 to 19965.59) | 10392.20 (5794.25 to 17616.98) | 9226.27 (5277.88 to 15583.66) | 7640.07 (4528.54 to 12798.26) | 5839.30 (3339.93 to 9659.47) | 4519.16 (2583.57 to 7434.03) | 3433.97 (1996.81 to 5590.88) | 2427.96 (1410.66 to 3973.14) | 1578.73 (931.98 to 2604.25) | 960.63 (565.64 to 1586.36) | 493.87 (288.27 to 815.90) | 204.42 (121.31 to 334.82) | 60.29 (35.20 to 98.53) | 11.87 (7.11 to 19.27) |
| Western Sub-Saharan Africa | Male | Number of prevalence | 115125.71 (77853.97 to 173723.90) | 311928.97 (199590.38 to 456454.79) | 368185.82 (234746.26 to 534521.57) | 399366.95 (256732.20 to 585656.99) | 391729.66 (251837.51 to 574127.77) | 365754.58 (236052.06 to 539612.10) | 337735.22 (219390.26 to 496844.02) | 313227.91 (203820.79 to 462043.04) | 280310.71 (182600.94 to 413432.30) | 234046.28 (153110.68 to 344191.86) | 181371.14 (117823.10 to 267020.59) | 141615.11 (92616.81 to 206076.09) | 108681.91 (71486.81 to 157171.46) | 78253.46 (51905.26 to 112613.33) | 52441.72 (34401.74 to 76808.80) | 32906.05 (21615.78 to 50056.34) | 17279.25 (11295.75 to 26833.50) | 7283.62 (4784.84 to 11114.31) | 2214.08 (1419.86 to 3441.23) | 446.87 (290.86 to 694.03) |
| **Country and territory** |  |  |  |  |  |  |  |  |  |  |  |  |  |  |  |  |  |  |  |  |  |  |
| American Samoa | Both | Number of DALYs | 0.40 (0.26 to 0.60) | 1.94 (1.04 to 3.50) | 2.63 (1.38 to 4.71) | 2.92 (1.56 to 5.13) | 2.38 (1.32 to 4.14) | 2.34 (1.27 to 3.94) | 2.58 (1.46 to 4.38) | 2.80 (1.57 to 4.77) | 3.17 (1.81 to 5.31) | 3.29 (1.88 to 5.52) | 3.33 (1.93 to 5.49) | 3.04 (1.76 to 4.93) | 2.27 (1.39 to 3.67) | 1.63 (0.99 to 2.60) | 1.09 (0.65 to 1.71) | 0.68 (0.41 to 1.08) | 0.38 (0.23 to 0.62) | 0.16 (0.10 to 0.25) | 0.04 (0.03 to 0.07) | 0.01 (0.01 to 0.01) |
| American Samoa | Both | Number of prevalence | 26.40 (11.22 to 60.24) | 87.26 (38.16 to 194.35) | 136.76 (57.67 to 310.19) | 163.45 (67.87 to 373.44) | 138.87 (57.45 to 318.22) | 136.77 (56.59 to 313.50) | 149.26 (62.15 to 341.30) | 159.92 (66.93 to 365.05) | 180.74 (75.90 to 412.10) | 187.70 (79.06 to 427.44) | 189.48 (80.05 to 430.91) | 172.78 (73.23 to 392.43) | 130.64 (55.38 to 296.70) | 96.22 (40.83 to 218.59) | 65.57 (27.82 to 149.00) | 42.00 (17.80 to 95.49) | 24.22 (10.25 to 55.11) | 10.59 (4.46 to 24.19) | 3.19 (1.33 to 7.33) | 0.66 (0.27 to 1.52) |
| American Samoa | Female | Number of DALYs | 0.23 (0.14 to 0.35) | 0.51 (0.30 to 0.80) | 0.60 (0.35 to 0.88) | 0.55 (0.29 to 0.86) | 0.42 (0.25 to 0.65) | 0.38 (0.23 to 0.57) | 0.38 (0.22 to 0.57) | 0.39 (0.23 to 0.59) | 0.41 (0.24 to 0.64) | 0.41 (0.25 to 0.63) | 0.40 (0.25 to 0.60) | 0.35 (0.21 to 0.51) | 0.27 (0.16 to 0.41) | 0.19 (0.12 to 0.28) | 0.13 (0.08 to 0.19) | 0.08 (0.05 to 0.12) | 0.05 (0.03 to 0.07) | 0.02 (0.01 to 0.03) | 0.01 (0.00 to 0.01) | 0.00 (0.00 to 0.00) |
| American Samoa | Female | Number of prevalence | 13.17 (5.78 to 29.60) | 39.26 (16.09 to 90.90) | 60.42 (23.48 to 142.73) | 71.75 (26.99 to 171.53) | 63.11 (23.32 to 151.79) | 62.46 (22.86 to 150.72) | 66.18 (24.11 to 160.00) | 69.80 (25.30 to 168.99) | 78.54 (28.41 to 190.35) | 81.09 (29.24 to 196.66) | 81.26 (29.24 to 197.20) | 73.88 (26.54 to 179.40) | 57.01 (20.45 to 138.53) | 42.71 (15.30 to 103.79) | 29.17 (10.45 to 70.92) | 18.81 (6.74 to 45.72) | 11.00 (3.94 to 26.73) | 5.08 (1.82 to 12.34) | 1.66 (0.59 to 4.03) | 0.36 (0.13 to 0.87) |
| American Samoa | Male | Number of DALYs | 0.17 (0.10 to 0.28) | 1.43 (0.66 to 2.81) | 2.04 (0.93 to 3.94) | 2.37 (1.12 to 4.41) | 1.96 (0.98 to 3.69) | 1.96 (0.98 to 3.50) | 2.20 (1.16 to 3.94) | 2.41 (1.28 to 4.24) | 2.76 (1.48 to 4.75) | 2.88 (1.55 to 4.98) | 2.93 (1.62 to 5.00) | 2.69 (1.48 to 4.47) | 2.01 (1.18 to 3.29) | 1.44 (0.83 to 2.35) | 0.96 (0.54 to 1.56) | 0.60 (0.34 to 0.97) | 0.34 (0.19 to 0.55) | 0.14 (0.08 to 0.23) | 0.04 (0.02 to 0.06) | 0.01 (0.00 to 0.01) |
| American Samoa | Male | Number of prevalence | 13.23 (5.42 to 30.64) | 48.00 (22.74 to 103.01) | 76.34 (34.88 to 166.50) | 91.70 (41.28 to 200.65) | 75.77 (33.94 to 165.30) | 74.31 (33.30 to 161.63) | 83.08 (37.42 to 180.08) | 90.11 (40.91 to 194.76) | 102.19 (46.57 to 220.30) | 106.61 (48.93 to 229.38) | 108.22 (49.86 to 232.56) | 98.90 (45.87 to 212.17) | 73.63 (34.34 to 157.63) | 53.51 (25.07 to 114.44) | 36.39 (17.04 to 77.85) | 23.19 (10.86 to 49.61) | 13.23 (6.19 to 28.29) | 5.52 (2.58 to 11.80) | 1.54 (0.72 to 3.29) | 0.30 (0.14 to 0.64) |
| Angola | Both | Number of DALYs | 549.42 (330.49 to 811.77) | 1509.50 (801.26 to 2659.71) | 1301.23 (702.59 to 2366.92) | 1049.44 (564.81 to 1888.92) | 876.80 (452.38 to 1528.80) | 759.03 (417.60 to 1379.44) | 655.38 (338.34 to 1185.25) | 544.24 (293.15 to 965.01) | 463.36 (254.40 to 830.87) | 378.04 (207.16 to 654.71) | 294.45 (158.81 to 506.56) | 232.94 (128.60 to 410.99) | 167.78 (89.54 to 301.10) | 113.23 (64.23 to 197.85) | 64.55 (35.56 to 111.02) | 36.36 (20.21 to 63.05) | 17.33 (9.46 to 29.28) | 5.61 (3.28 to 9.36) | 1.44 (0.84 to 2.38) | 0.27 (0.16 to 0.43) |
| Angola | Both | Number of prevalence | 13209.06 (6057.38 to 34942.72) | 31816.73 (13893.70 to 83508.95) | 33983.43 (12746.24 to 97139.46) | 33257.13 (11125.91 to 100015.04) | 31245.02 (9679.44 to 96753.23) | 29440.78 (8701.60 to 92648.45) | 26971.59 (7746.16 to 85662.54) | 23169.62 (6528.55 to 74026.99) | 20331.86 (5626.10 to 65272.70) | 17061.11 (4669.92 to 54970.85) | 13630.95 (3684.74 to 44061.47) | 11278.62 (3004.21 to 36617.46) | 8361.51 (2200.04 to 27245.97) | 5797.86 (1514.49 to 18936.48) | 3403.78 (884.48 to 11136.79) | 1979.60 (511.98 to 6490.34) | 985.54 (254.23 to 3236.03) | 331.08 (84.81 to 1089.36) | 89.42 (22.53 to 295.45) | 18.13 (4.51 to 60.27) |
| Angola | Female | Number of DALYs | 318.30 (184.36 to 518.43) | 505.33 (284.71 to 774.36) | 430.21 (251.85 to 687.45) | 342.17 (204.86 to 528.96) | 274.52 (161.49 to 409.61) | 240.93 (140.42 to 372.78) | 207.52 (127.63 to 317.91) | 169.98 (100.73 to 264.41) | 141.83 (83.10 to 216.45) | 114.70 (65.39 to 180.96) | 88.12 (51.45 to 135.38) | 73.05 (44.61 to 110.80) | 52.61 (30.11 to 80.08) | 36.05 (21.72 to 55.96) | 21.52 (12.61 to 33.56) | 12.87 (7.82 to 19.14) | 6.38 (3.57 to 9.71) | 2.19 (1.32 to 3.32) | 0.63 (0.38 to 0.99) | 0.14 (0.08 to 0.21) |
| Angola | Female | Number of prevalence | 7021.14 (3490.05 to 17904.85) | 14117.21 (5891.50 to 39503.50) | 15474.38 (5403.18 to 46623.31) | 15454.37 (4654.73 to 48853.93) | 14820.16 (4045.48 to 48142.99) | 14315.75 (3688.10 to 47179.70) | 13297.73 (3304.17 to 44204.67) | 11371.60 (2752.41 to 38035.02) | 9986.55 (2367.74 to 33555.65) | 8301.76 (1935.69 to 27999.13) | 6626.21 (1521.91 to 22426.23) | 5645.83 (1277.50 to 19166.35) | 4217.02 (937.40 to 14363.77) | 2966.39 (654.45 to 10118.89) | 1811.80 (400.02 to 6179.92) | 1096.43 (241.46 to 3740.54) | 561.31 (123.86 to 1915.06) | 196.53 (43.43 to 670.34) | 57.34 (12.66 to 195.57) | 12.84 (2.83 to 43.80) |
| Angola | Male | Number of DALYs | 231.12 (122.25 to 363.81) | 1004.17 (431.81 to 2052.96) | 871.02 (387.26 to 1817.86) | 707.27 (316.03 to 1466.08) | 602.28 (259.70 to 1212.79) | 518.10 (242.97 to 1063.54) | 447.86 (189.39 to 944.99) | 374.27 (169.88 to 750.18) | 321.54 (150.93 to 659.23) | 263.34 (119.84 to 525.59) | 206.33 (93.89 to 403.83) | 159.89 (70.95 to 302.78) | 115.16 (51.07 to 237.68) | 77.18 (36.41 to 153.15) | 43.04 (18.14 to 83.86) | 23.49 (11.53 to 46.91) | 10.96 (4.93 to 21.39) | 3.42 (1.62 to 6.69) | 0.80 (0.37 to 1.60) | 0.13 (0.06 to 0.25) |
| Angola | Male | Number of prevalence | 6187.92 (2563.18 to 17037.86) | 17699.53 (7592.96 to 42832.68) | 18509.05 (7051.63 to 49278.32) | 17802.76 (6196.86 to 50462.59) | 16424.86 (5478.25 to 48210.49) | 15125.03 (4858.68 to 45237.73) | 13673.86 (4301.01 to 41345.96) | 11798.02 (3647.20 to 35935.12) | 10345.31 (3156.17 to 31705.08) | 8759.35 (2644.51 to 26959.06) | 7004.74 (2090.90 to 21653.92) | 5632.80 (1667.30 to 17467.96) | 4144.49 (1214.55 to 12893.89) | 2831.47 (826.46 to 8825.45) | 1591.99 (464.27 to 4961.21) | 883.17 (257.87 to 2752.20) | 424.23 (123.86 to 1322.13) | 134.56 (39.36 to 419.39) | 32.09 (9.36 to 99.97) | 5.29 (1.54 to 16.49) |
| Bangladesh | Both | Number of DALYs | 1404.91 (870.29 to 2062.37) | 2473.22 (1576.87 to 3798.14) | 4243.24 (2306.68 to 7817.34) | 4027.99 (2174.87 to 7100.10) | 3825.91 (2115.54 to 6842.56) | 3678.70 (1976.15 to 6589.59) | 3463.31 (1889.22 to 5918.85) | 3013.60 (1651.61 to 5333.28) | 2625.07 (1401.82 to 4435.08) | 2271.15 (1307.91 to 4153.67) | 1941.68 (1079.88 to 3471.23) | 1657.83 (894.16 to 2953.54) | 1358.31 (739.63 to 2434.87) | 1085.07 (609.35 to 1967.16) | 800.94 (447.02 to 1478.67) | 550.52 (310.19 to 992.31) | 298.90 (166.45 to 547.89) | 127.64 (71.56 to 224.03) | 39.07 (21.48 to 69.32) | 5.75 (3.28 to 9.96) |
| Bangladesh | Both | Number of prevalence | 16103.21 (15168.87 to 17053.11) | 30598.06 (28743.86 to 32586.14) | 47921.56 (38014.13 to 69819.96) | 49078.76 (39595.11 to 69832.47) | 49899.04 (40794.30 to 69757.39) | 49960.11 (41003.06 to 69342.77) | 48207.31 (39528.96 to 66911.56) | 43432.78 (35639.20 to 60144.26) | 38751.17 (31963.56 to 53244.79) | 34429.55 (28503.96 to 47000.65) | 29596.39 (24384.74 to 40548.37) | 25837.75 (21238.88 to 35497.03) | 21695.67 (17785.90 to 29913.50) | 17463.42 (14283.72 to 24163.23) | 13229.28 (10813.23 to 18329.17) | 9177.75 (7489.22 to 12738.49) | 5093.86 (4161.48 to 7062.13) | 2206.93 (1805.66 to 3054.87) | 683.72 (556.28 to 953.90) | 103.00 (84.45 to 141.45) |
| Bangladesh | Female | Number of DALYs | 798.76 (465.87 to 1252.33) | 1416.68 (810.20 to 2248.44) | 1481.07 (853.39 to 2330.94) | 1465.60 (868.85 to 2230.32) | 1431.48 (856.51 to 2177.54) | 1374.95 (795.19 to 2158.91) | 1240.80 (748.77 to 1869.45) | 1067.01 (629.55 to 1641.39) | 948.98 (550.92 to 1481.90) | 838.37 (484.35 to 1315.02) | 684.96 (412.36 to 1035.91) | 569.82 (325.82 to 877.47) | 452.60 (273.99 to 709.10) | 347.14 (210.18 to 533.57) | 254.61 (152.55 to 379.65) | 173.62 (104.68 to 273.53) | 94.57 (58.25 to 142.58) | 41.54 (24.90 to 62.66) | 11.90 (7.25 to 18.36) | 1.91 (1.23 to 2.77) |
| Bangladesh | Female | Number of prevalence | 9006.43 (8374.35 to 9677.20) | 17033.76 (15820.28 to 18331.42) | 19244.94 (17865.11 to 20788.17) | 20807.11 (19301.14 to 22586.85) | 22187.59 (20427.67 to 24151.38) | 22185.11 (20384.29 to 24244.24) | 20954.68 (19203.03 to 22954.20) | 18695.50 (17096.79 to 20478.40) | 17143.66 (15670.23 to 18842.24) | 15519.58 (14188.44 to 17092.41) | 12904.61 (11796.48 to 14244.23) | 10910.46 (9961.02 to 12045.85) | 8899.74 (8096.16 to 9866.28) | 6979.21 (6352.12 to 7722.30) | 5225.11 (4754.04 to 5790.78) | 3597.47 (3278.94 to 3982.03) | 2004.02 (1828.22 to 2220.98) | 883.77 (803.99 to 979.47) | 259.91 (236.85 to 287.84) | 42.70 (38.86 to 47.38) |
| Bangladesh | Male | Number of DALYs | 606.15 (315.06 to 992.34) | 1056.55 (563.15 to 1733.52) | 2762.16 (1234.25 to 5824.68) | 2562.39 (1095.74 to 5508.03) | 2394.44 (1012.21 to 4911.65) | 2303.75 (1056.62 to 4855.78) | 2222.51 (997.05 to 4405.83) | 1946.59 (828.13 to 4013.39) | 1676.09 (715.16 to 3250.50) | 1432.78 (666.42 to 3102.67) | 1256.72 (576.17 to 2656.97) | 1088.01 (474.56 to 2244.43) | 905.70 (407.75 to 1860.95) | 737.93 (347.30 to 1510.97) | 546.33 (247.33 to 1137.61) | 376.89 (166.97 to 777.37) | 204.32 (92.62 to 423.88) | 86.10 (38.13 to 179.46) | 27.17 (12.38 to 54.87) | 3.85 (1.79 to 7.80) |
| Bangladesh | Male | Number of prevalence | 7096.79 (6526.48 to 7712.93) | 13564.30 (12453.20 to 14795.32) | 28676.62 (19029.50 to 50550.98) | 28271.65 (19227.79 to 48723.92) | 27711.45 (19150.93 to 46980.42) | 27775.00 (19488.27 to 46554.81) | 27252.63 (19263.29 to 45317.35) | 24737.28 (17571.26 to 40836.44) | 21607.52 (15417.46 to 35514.41) | 18909.96 (13569.39 to 30958.70) | 16691.78 (12027.67 to 27197.42) | 14927.29 (10786.67 to 24225.02) | 12795.94 (9300.52 to 20693.13) | 10484.21 (7620.22 to 16926.48) | 8004.17 (5820.31 to 12926.35) | 5580.28 (4058.60 to 9009.70) | 3089.84 (2247.98 to 4981.23) | 1323.17 (962.90 to 2139.92) | 423.82 (308.45 to 684.39) | 60.30 (43.90 to 97.26) |
| Benin | Both | Number of DALYs | 219.32 (130.74 to 334.09) | 565.30 (292.21 to 1034.49) | 481.36 (260.98 to 861.75) | 415.10 (224.06 to 744.54) | 363.37 (190.82 to 638.31) | 314.53 (174.41 to 559.61) | 251.92 (141.08 to 447.65) | 203.39 (112.09 to 368.80) | 168.72 (90.63 to 306.88) | 135.93 (74.25 to 242.12) | 103.68 (58.07 to 182.05) | 80.15 (42.18 to 144.26) | 59.74 (33.81 to 104.31) | 43.56 (23.32 to 77.39) | 27.86 (15.72 to 48.10) | 18.75 (10.86 to 32.17) | 9.85 (5.60 to 16.42) | 4.09 (2.36 to 7.11) | 1.24 (0.75 to 2.06) | 0.31 (0.19 to 0.50) |
| Benin | Both | Number of prevalence | 4148.79 (3202.47 to 5772.95) | 9515.64 (6822.43 to 13847.83) | 9788.97 (6754.24 to 14856.46) | 9952.11 (6716.23 to 15578.52) | 9813.12 (6509.55 to 15284.12) | 9145.13 (6015.44 to 14348.01) | 7792.08 (5086.92 to 12301.33) | 6568.10 (4266.25 to 10416.52) | 5640.91 (3659.27 to 8969.22) | 4634.45 (2995.17 to 7377.07) | 3633.28 (2340.59 to 5801.04) | 2865.19 (1841.20 to 4579.93) | 2229.01 (1429.72 to 3564.01) | 1684.19 (1077.68 to 2689.57) | 1109.66 (709.70 to 1770.62) | 770.74 (492.41 to 1229.94) | 420.47 (268.02 to 671.81) | 181.85 (115.72 to 291.75) | 58.12 (36.88 to 93.65) | 15.50 (9.83 to 25.12) |
| Benin | Female | Number of DALYs | 125.84 (74.48 to 202.29) | 188.83 (106.04 to 298.11) | 158.83 (95.06 to 243.66) | 140.32 (78.09 to 216.58) | 124.69 (71.70 to 195.09) | 103.29 (61.52 to 158.06) | 81.74 (49.13 to 125.79) | 66.98 (39.80 to 105.61) | 56.06 (34.81 to 91.88) | 43.65 (25.95 to 68.40) | 32.43 (19.38 to 48.37) | 24.87 (13.98 to 39.25) | 18.96 (11.11 to 28.06) | 14.12 (8.48 to 21.98) | 9.40 (5.96 to 14.28) | 6.71 (3.95 to 10.14) | 3.84 (2.34 to 5.88) | 1.73 (1.06 to 2.58) | 0.58 (0.34 to 0.87) | 0.16 (0.10 to 0.24) |
| Benin | Female | Number of prevalence | 2220.33 (1745.69 to 3012.78) | 4034.96 (3006.10 to 5782.52) | 4275.87 (3003.68 to 6428.13) | 4537.15 (3038.14 to 7066.60) | 4642.23 (3016.51 to 7385.58) | 4315.13 (2761.61 to 6945.11) | 3645.03 (2310.84 to 5907.22) | 3131.18 (1974.49 to 5101.21) | 2735.43 (1713.79 to 4471.68) | 2202.26 (1374.32 to 3611.13) | 1682.68 (1046.91 to 2766.63) | 1329.08 (822.10 to 2190.88) | 1059.80 (653.48 to 1752.25) | 811.25 (499.50 to 1342.91) | 552.49 (340.04 to 914.94) | 399.55 (245.69 to 661.72) | 231.36 (142.31 to 383.01) | 106.69 (65.62 to 176.62) | 36.32 (22.34 to 60.14) | 10.46 (6.43 to 17.32) |
| Benin | Male | Number of DALYs | 93.48 (48.92 to 153.36) | 376.46 (159.17 to 771.45) | 322.53 (145.57 to 671.52) | 274.78 (118.59 to 563.66) | 238.67 (99.93 to 493.82) | 211.24 (93.99 to 440.17) | 170.18 (74.83 to 364.15) | 136.41 (62.70 to 281.01) | 112.66 (50.55 to 236.53) | 92.28 (41.35 to 187.32) | 71.25 (30.95 to 144.03) | 55.28 (25.92 to 115.96) | 40.77 (19.07 to 84.47) | 29.44 (13.12 to 62.83) | 18.46 (8.31 to 37.72) | 12.04 (5.64 to 24.85) | 6.00 (2.80 to 12.32) | 2.37 (1.08 to 5.06) | 0.67 (0.30 to 1.39) | 0.15 (0.07 to 0.31) |
| Benin | Male | Number of prevalence | 1928.47 (1429.77 to 2775.86) | 5480.68 (3682.22 to 8695.61) | 5513.11 (3635.72 to 8824.56) | 5414.95 (3503.53 to 8550.22) | 5170.89 (3320.74 to 8130.93) | 4830.00 (3082.97 to 7635.65) | 4147.05 (2637.43 to 6622.59) | 3436.92 (2179.53 to 5514.94) | 2905.49 (1838.06 to 4688.81) | 2432.20 (1534.68 to 3933.52) | 1950.60 (1230.04 to 3159.67) | 1536.11 (966.75 to 2497.20) | 1169.21 (734.00 to 1908.06) | 872.94 (548.48 to 1427.17) | 557.17 (349.83 to 910.85) | 371.19 (233.25 to 606.55) | 189.10 (118.73 to 309.06) | 75.16 (47.20 to 122.82) | 21.80 (13.70 to 35.65) | 5.04 (3.17 to 8.24) |
| Brazil | Both | Number of DALYs | 85.05 (50.28 to 130.58) | 132.25 (82.19 to 193.31) | 126.61 (81.36 to 198.59) | 123.09 (74.76 to 183.56) | 128.63 (81.76 to 197.23) | 123.96 (75.70 to 180.93) | 123.29 (73.49 to 186.77) | 127.04 (79.39 to 192.04) | 117.61 (73.50 to 180.42) | 100.34 (63.70 to 150.46) | 88.48 (53.82 to 129.56) | 75.74 (46.99 to 112.27) | 60.81 (38.95 to 87.55) | 46.80 (29.73 to 68.13) | 36.62 (22.82 to 53.80) | 23.98 (15.08 to 34.97) | 15.62 (9.73 to 23.38) | 7.48 (4.85 to 11.32) | 2.98 (1.87 to 4.23) | 1.27 (0.79 to 1.83) |
| Brazil | Both | Number of prevalence | 861.81 (791.49 to 1045.56) | 1365.49 (1237.35 to 1790.56) | 1362.72 (1211.52 to 1961.67) | 1366.50 (1188.28 to 2158.90) | 1468.54 (1253.82 to 2475.07) | 1450.25 (1224.79 to 2549.01) | 1480.03 (1232.55 to 2676.79) | 1524.08 (1258.09 to 2815.64) | 1424.57 (1167.61 to 2678.12) | 1246.74 (1016.78 to 2379.58) | 1111.25 (902.30 to 2144.86) | 973.64 (786.81 to 1903.59) | 798.95 (641.36 to 1587.14) | 623.52 (499.00 to 1245.74) | 489.72 (392.41 to 979.00) | 329.66 (263.99 to 656.60) | 216.21 (173.17 to 429.15) | 105.13 (84.51 to 208.24) | 42.55 (34.16 to 83.96) | 18.70 (14.99 to 37.24) |
| Brazil | Female | Number of DALYs | 48.84 (26.77 to 77.89) | 76.30 (44.04 to 121.46) | 72.30 (42.79 to 119.43) | 69.93 (41.25 to 108.41) | 74.65 (45.09 to 115.06) | 73.30 (40.63 to 112.75) | 74.28 (41.57 to 116.64) | 76.59 (45.50 to 121.25) | 70.32 (40.41 to 111.40) | 61.22 (37.31 to 98.46) | 54.47 (30.24 to 84.64) | 47.78 (26.15 to 73.91) | 38.48 (23.77 to 59.34) | 30.39 (18.12 to 47.00) | 23.77 (13.53 to 36.29) | 16.07 (9.31 to 23.75) | 10.94 (6.46 to 16.93) | 5.37 (3.26 to 8.42) | 2.22 (1.34 to 3.33) | 0.85 (0.52 to 1.28) |
| Brazil | Female | Number of prevalence | 491.65 (447.41 to 587.94) | 777.96 (700.80 to 995.99) | 775.01 (685.49 to 1076.64) | 778.79 (674.31 to 1177.68) | 844.85 (717.57 to 1362.18) | 848.95 (713.66 to 1427.18) | 878.96 (732.81 to 1520.61) | 905.46 (748.42 to 1600.39) | 845.60 (695.63 to 1520.13) | 747.73 (611.93 to 1364.55) | 672.69 (547.03 to 1242.84) | 598.15 (484.17 to 1120.10) | 497.02 (400.90 to 946.28) | 396.32 (318.09 to 759.48) | 313.25 (251.96 to 600.95) | 217.42 (174.83 to 416.27) | 147.98 (118.74 to 283.34) | 74.52 (59.73 to 142.69) | 31.42 (25.26 to 60.14) | 12.35 (9.92 to 23.66) |
| Brazil | Male | Number of DALYs | 36.21 (19.35 to 59.57) | 55.94 (30.83 to 90.14) | 54.31 (30.24 to 88.57) | 53.15 (27.37 to 84.22) | 53.97 (27.83 to 89.92) | 50.66 (26.77 to 82.38) | 49.01 (26.80 to 78.29) | 50.46 (27.73 to 81.14) | 47.29 (25.35 to 77.73) | 39.12 (22.28 to 62.68) | 34.02 (19.02 to 53.89) | 27.96 (15.92 to 43.56) | 22.33 (12.42 to 34.62) | 16.41 (9.18 to 26.32) | 12.86 (7.02 to 20.41) | 7.91 (4.50 to 12.53) | 4.69 (2.41 to 7.60) | 2.11 (1.24 to 3.25) | 0.76 (0.43 to 1.21) | 0.41 (0.23 to 0.63) |
| Brazil | Male | Number of prevalence | 370.16 (328.26 to 460.21) | 587.53 (514.58 to 796.13) | 587.71 (502.88 to 878.17) | 587.71 (490.12 to 967.19) | 623.69 (509.36 to 1103.80) | 601.31 (484.12 to 1114.45) | 601.07 (478.41 to 1149.79) | 618.61 (488.82 to 1210.49) | 578.97 (453.49 to 1155.59) | 499.01 (387.99 to 1012.35) | 438.56 (338.98 to 901.21) | 375.49 (287.98 to 783.49) | 301.93 (230.33 to 640.87) | 227.20 (172.65 to 486.26) | 176.47 (134.18 to 377.53) | 112.24 (85.42 to 240.12) | 68.23 (51.76 to 145.81) | 30.62 (23.28 to 65.54) | 11.13 (8.46 to 23.82) | 6.35 (4.82 to 13.58) |
| Brunei Darussalam | Both | Number of DALYs | 3.04 (1.79 to 4.56) | 9.18 (4.68 to 17.32) | 9.43 (5.01 to 17.09) | 10.71 (5.87 to 19.42) | 12.86 (6.72 to 23.66) | 13.90 (7.47 to 25.32) | 14.99 (7.56 to 27.12) | 14.40 (7.59 to 26.49) | 12.63 (6.69 to 24.01) | 10.29 (5.29 to 18.70) | 8.35 (4.28 to 15.11) | 6.55 (3.61 to 11.88) | 5.04 (2.72 to 8.85) | 3.48 (2.02 to 5.92) | 2.09 (1.14 to 3.67) | 1.19 (0.66 to 2.12) | 0.66 (0.35 to 1.16) | 0.20 (0.12 to 0.33) | 0.03 (0.02 to 0.04) | 0.00 (0.00 to 0.00) |
| Brunei Darussalam | Both | Number of prevalence | 63.29 (28.67 to 275.31) | 169.00 (65.32 to 704.85) | 209.44 (67.16 to 982.35) | 281.97 (77.21 to 1433.05) | 376.40 (92.73 to 1996.85) | 443.06 (101.74 to 2410.32) | 495.56 (109.00 to 2732.68) | 491.52 (104.91 to 2734.97) | 445.07 (92.60 to 2498.49) | 380.85 (77.39 to 2157.56) | 321.40 (63.98 to 1835.68) | 260.99 (51.04 to 1498.74) | 211.16 (40.37 to 1221.33) | 150.02 (28.36 to 870.37) | 91.86 (17.33 to 533.50) | 52.70 (9.96 to 305.31) | 30.53 (5.75 to 177.50) | 10.53 (1.91 to 62.54) | 1.99 (0.33 to 12.22) | 0.11 (0.02 to 0.66) |
| Brunei Darussalam | Female | Number of DALYs | 1.72 (1.02 to 2.69) | 2.96 (1.65 to 4.62) | 2.90 (1.70 to 4.59) | 3.13 (1.87 to 4.71) | 3.63 (2.07 to 5.59) | 3.87 (2.24 to 6.13) | 4.05 (2.37 to 6.18) | 3.74 (2.14 to 5.71) | 3.31 (2.00 to 5.14) | 2.86 (1.67 to 4.61) | 2.40 (1.39 to 3.63) | 1.86 (1.06 to 2.93) | 1.50 (0.92 to 2.29) | 1.03 (0.62 to 1.58) | 0.64 (0.39 to 0.99) | 0.35 (0.21 to 0.51) | 0.21 (0.12 to 0.31) | 0.09 (0.05 to 0.14) | 0.02 (0.01 to 0.04) | 0.00 (0.00 to 0.00) |
| Brunei Darussalam | Female | Number of prevalence | 32.86 (15.93 to 133.93) | 70.58 (27.92 to 329.44) | 87.27 (27.65 to 453.25) | 116.97 (30.23 to 652.74) | 155.02 (34.86 to 898.74) | 185.48 (38.36 to 1098.49) | 204.88 (39.94 to 1228.78) | 199.87 (37.27 to 1209.55) | 183.92 (33.17 to 1120.65) | 164.92 (28.82 to 1010.49) | 145.45 (24.75 to 895.48) | 118.16 (19.62 to 731.02) | 98.08 (15.81 to 609.83) | 70.39 (11.20 to 438.61) | 43.83 (6.97 to 273.15) | 24.22 (3.85 to 150.94) | 14.83 (2.35 to 92.39) | 6.71 (1.06 to 41.83) | 1.79 (0.28 to 11.15) | 0.08 (0.01 to 0.53) |
| Brunei Darussalam | Male | Number of DALYs | 1.32 (0.70 to 2.20) | 6.22 (2.57 to 13.12) | 6.53 (2.93 to 13.85) | 7.59 (3.32 to 15.38) | 9.23 (4.03 to 19.27) | 10.03 (4.60 to 20.76) | 10.95 (4.70 to 23.25) | 10.66 (4.86 to 21.98) | 9.32 (4.29 to 19.37) | 7.42 (3.14 to 15.60) | 5.95 (2.59 to 12.17) | 4.68 (2.16 to 9.54) | 3.54 (1.60 to 6.97) | 2.45 (1.19 to 4.86) | 1.45 (0.65 to 2.87) | 0.84 (0.38 to 1.74) | 0.45 (0.21 to 0.93) | 0.11 (0.05 to 0.22) | 0.01 (0.00 to 0.01) | 0.00 (0.00 to 0.00) |
| Brunei Darussalam | Male | Number of prevalence | 30.43 (12.26 to 141.38) | 98.42 (34.92 to 375.41) | 122.17 (37.17 to 529.10) | 165.00 (44.00 to 780.31) | 221.39 (54.06 to 1098.11) | 257.58 (59.75 to 1311.83) | 290.68 (65.09 to 1503.91) | 291.64 (63.77 to 1525.42) | 261.15 (55.86 to 1377.83) | 215.92 (45.33 to 1147.07) | 175.95 (36.53 to 940.20) | 142.83 (29.08 to 767.72) | 113.08 (22.71 to 611.50) | 79.63 (15.85 to 431.75) | 48.02 (9.55 to 260.36) | 28.47 (5.67 to 154.37) | 15.70 (3.12 to 85.11) | 3.82 (0.76 to 20.71) | 0.20 (0.04 to 1.06) | 0.02 (0.00 to 0.13) |
| Burkina Faso | Both | Number of DALYs | 373.66 (232.46 to 578.10) | 920.33 (514.39 to 1681.83) | 790.42 (436.65 to 1406.75) | 680.45 (356.54 to 1221.89) | 547.15 (293.18 to 958.63) | 452.12 (252.26 to 824.22) | 405.52 (223.08 to 726.60) | 347.69 (187.38 to 645.31) | 271.35 (146.49 to 480.50) | 212.75 (112.44 to 394.15) | 168.01 (91.72 to 301.47) | 132.96 (70.83 to 238.45) | 101.96 (55.47 to 185.31) | 76.68 (42.35 to 133.22) | 53.82 (30.56 to 95.07) | 34.14 (19.39 to 59.88) | 16.79 (9.54 to 28.62) | 6.40 (3.66 to 10.94) | 1.83 (1.06 to 3.03) | 0.40 (0.24 to 0.65) |
| Burkina Faso | Both | Number of prevalence | 6326.33 (4520.69 to 11406.58) | 13998.36 (9179.05 to 24999.88) | 14120.81 (8735.71 to 27179.10) | 14151.96 (8254.22 to 28644.30) | 12861.96 (7174.13 to 27350.78) | 11525.25 (6290.52 to 25173.24) | 10746.19 (5771.87 to 23753.42) | 9590.65 (5092.53 to 21394.68) | 7627.46 (4008.61 to 17130.22) | 6107.62 (3186.87 to 13801.08) | 4993.92 (2586.89 to 11365.48) | 4090.50 (2101.07 to 9368.28) | 3234.78 (1647.25 to 7453.80) | 2500.73 (1270.00 to 5779.13) | 1807.57 (917.86 to 4180.38) | 1172.14 (594.17 to 2717.00) | 595.24 (300.49 to 1385.10) | 235.71 (118.76 to 551.18) | 70.17 (35.34 to 164.86) | 16.46 (8.24 to 39.02) |
| Burkina Faso | Female | Number of DALYs | 216.76 (123.63 to 348.29) | 315.19 (189.97 to 505.82) | 271.57 (167.01 to 414.27) | 237.83 (137.86 to 378.26) | 202.01 (111.77 to 319.97) | 169.37 (102.85 to 259.54) | 144.09 (82.13 to 220.21) | 117.95 (69.45 to 185.14) | 90.63 (53.49 to 140.27) | 69.42 (39.84 to 106.63) | 55.08 (30.99 to 86.14) | 44.08 (25.73 to 67.71) | 33.95 (20.20 to 52.14) | 25.39 (14.51 to 38.27) | 18.09 (9.93 to 28.06) | 12.05 (7.49 to 18.20) | 6.38 (3.82 to 9.94) | 2.63 (1.53 to 4.00) | 0.81 (0.49 to 1.25) | 0.21 (0.12 to 0.31) |
| Burkina Faso | Female | Number of prevalence | 3442.15 (2546.07 to 5977.93) | 5908.08 (4008.45 to 11295.03) | 6170.37 (3803.54 to 12875.07) | 6467.58 (3692.17 to 14394.45) | 6323.79 (3431.57 to 14606.27) | 5814.30 (3044.53 to 13713.77) | 5240.10 (2687.46 to 12513.54) | 4559.22 (2301.61 to 10990.71) | 3553.06 (1772.98 to 8624.32) | 2818.66 (1392.31 to 6881.75) | 2337.56 (1142.81 to 5736.48) | 1934.22 (940.15 to 4768.85) | 1527.52 (734.84 to 3785.89) | 1181.47 (565.03 to 2935.61) | 860.12 (411.60 to 2137.74) | 578.40 (276.22 to 1437.30) | 310.19 (148.35 to 770.75) | 130.56 (62.50 to 324.45) | 41.49 (19.82 to 103.04) | 10.76 (5.15 to 26.74) |
| Burkina Faso | Male | Number of DALYs | 156.90 (80.87 to 266.61) | 605.14 (269.63 to 1303.00) | 518.85 (229.38 to 1096.93) | 442.61 (183.48 to 919.40) | 345.14 (151.12 to 688.54) | 282.75 (130.32 to 596.51) | 261.43 (116.90 to 543.96) | 229.74 (99.57 to 477.80) | 180.72 (80.11 to 361.75) | 143.33 (63.41 to 308.32) | 112.93 (49.28 to 235.69) | 88.88 (41.01 to 183.18) | 68.00 (30.16 to 141.49) | 51.29 (23.53 to 102.52) | 35.74 (16.35 to 70.68) | 22.09 (10.04 to 45.78) | 10.41 (4.69 to 20.52) | 3.77 (1.68 to 7.79) | 1.02 (0.45 to 2.03) | 0.20 (0.09 to 0.40) |
| Burkina Faso | Male | Number of prevalence | 2884.18 (1955.94 to 5415.30) | 8090.28 (4743.66 to 14693.85) | 7950.43 (4554.64 to 14594.15) | 7684.38 (4301.47 to 15179.68) | 6538.17 (3577.87 to 13609.15) | 5710.95 (3072.68 to 11964.81) | 5506.09 (2935.85 to 11580.84) | 5031.42 (2660.52 to 10717.14) | 4074.40 (2142.99 to 8761.86) | 3288.96 (1719.05 to 7112.25) | 2656.36 (1379.60 to 5752.87) | 2156.28 (1114.15 to 4671.35) | 1707.26 (879.59 to 3705.29) | 1319.26 (678.08 to 2865.19) | 947.45 (486.52 to 2059.12) | 593.74 (304.97 to 1289.93) | 285.05 (146.42 to 619.26) | 105.16 (54.04 to 228.38) | 28.68 (14.74 to 62.24) | 5.70 (2.93 to 12.37) |
| Cambodia | Both | Number of DALYs | 0.00 (0.00 to 0.00) | 0.00 (0.00 to 0.00) | 0.00 (0.00 to 0.00) | 0.00 (0.00 to 0.00) | 0.00 (0.00 to 0.00) | 0.00 (0.00 to 0.00) | 0.00 (0.00 to 0.00) | 0.00 (0.00 to 0.00) | 0.00 (0.00 to 0.00) | 0.00 (0.00 to 0.00) | 0.00 (0.00 to 0.00) | 0.00 (0.00 to 0.00) | 0.00 (0.00 to 0.00) | 0.00 (0.00 to 0.00) | 0.00 (0.00 to 0.00) | 0.00 (0.00 to 0.00) | 0.00 (0.00 to 0.00) | 0.00 (0.00 to 0.00) | 0.00 (0.00 to 0.00) | 0.00 (0.00 to 0.00) |
| Cambodia | Both | Number of prevalence | 342.45 (5.79 to 2224.00) | 826.89 (13.97 to 5370.10) | 1157.68 (19.56 to 7518.38) | 1443.87 (24.40 to 9377.00) | 1712.02 (28.93 to 11118.42) | 2027.21 (34.26 to 13165.43) | 2090.80 (35.33 to 13578.40) | 1951.50 (32.98 to 12673.69) | 1568.33 (26.50 to 10185.25) | 1322.60 (22.35 to 8589.39) | 1277.48 (21.59 to 8296.39) | 1269.36 (21.45 to 8243.63) | 1001.21 (16.92 to 6502.18) | 730.02 (12.34 to 4741.01) | 511.72 (8.65 to 3323.26) | 294.12 (4.97 to 1910.10) | 156.21 (2.64 to 1014.49) | 60.60 (1.02 to 393.53) | 15.64 (0.26 to 101.55) | 3.04 (0.05 to 19.77) |
| Cambodia | Female | Number of DALYs | 0.00 (0.00 to 0.00) | 0.00 (0.00 to 0.00) | 0.00 (0.00 to 0.00) | 0.00 (0.00 to 0.00) | 0.00 (0.00 to 0.00) | 0.00 (0.00 to 0.00) | 0.00 (0.00 to 0.00) | 0.00 (0.00 to 0.00) | 0.00 (0.00 to 0.00) | 0.00 (0.00 to 0.00) | 0.00 (0.00 to 0.00) | 0.00 (0.00 to 0.00) | 0.00 (0.00 to 0.00) | 0.00 (0.00 to 0.00) | 0.00 (0.00 to 0.00) | 0.00 (0.00 to 0.00) | 0.00 (0.00 to 0.00) | 0.00 (0.00 to 0.00) | 0.00 (0.00 to 0.00) | 0.00 (0.00 to 0.00) |
| Cambodia | Female | Number of prevalence | 167.37 (2.83 to 1086.97) | 403.14 (6.81 to 2618.13) | 563.03 (9.52 to 3656.52) | 704.30 (11.90 to 4573.99) | 843.44 (14.25 to 5477.59) | 1005.64 (16.99 to 6530.97) | 1046.51 (17.69 to 6796.39) | 987.27 (16.68 to 6411.65) | 812.50 (13.73 to 5276.64) | 694.50 (11.74 to 4510.35) | 692.72 (11.71 to 4498.79) | 672.37 (11.36 to 4366.61) | 577.99 (9.77 to 3753.68) | 445.05 (7.52 to 2890.32) | 315.59 (5.33 to 2049.53) | 184.26 (3.11 to 1196.62) | 101.50 (1.72 to 659.20) | 40.82 (0.69 to 265.13) | 11.16 (0.19 to 72.45) | 2.35 (0.04 to 15.26) |
| Cambodia | Male | Number of DALYs | 0.00 (0.00 to 0.00) | 0.00 (0.00 to 0.00) | 0.00 (0.00 to 0.00) | 0.00 (0.00 to 0.00) | 0.00 (0.00 to 0.00) | 0.00 (0.00 to 0.00) | 0.00 (0.00 to 0.00) | 0.00 (0.00 to 0.00) | 0.00 (0.00 to 0.00) | 0.00 (0.00 to 0.00) | 0.00 (0.00 to 0.00) | 0.00 (0.00 to 0.00) | 0.00 (0.00 to 0.00) | 0.00 (0.00 to 0.00) | 0.00 (0.00 to 0.00) | 0.00 (0.00 to 0.00) | 0.00 (0.00 to 0.00) | 0.00 (0.00 to 0.00) | 0.00 (0.00 to 0.00) | 0.00 (0.00 to 0.00) |
| Cambodia | Male | Number of prevalence | 175.08 (2.96 to 1137.04) | 423.75 (7.16 to 2751.97) | 594.65 (10.05 to 3861.86) | 739.57 (12.50 to 4803.00) | 868.58 (14.68 to 5640.84) | 1021.58 (17.26 to 6634.46) | 1044.30 (17.65 to 6782.01) | 964.23 (16.30 to 6262.04) | 755.83 (12.77 to 4908.61) | 628.09 (10.61 to 4079.04) | 584.76 (9.88 to 3797.61) | 596.99 (10.09 to 3877.03) | 423.21 (7.15 to 2748.50) | 284.97 (4.82 to 1850.70) | 196.13 (3.31 to 1273.73) | 109.86 (1.86 to 713.48) | 54.71 (0.92 to 355.29) | 19.77 (0.33 to 128.41) | 4.48 (0.08 to 29.11) | 0.69 (0.01 to 4.51) |
| Cameroon | Both | Number of DALYs | 461.50 (280.03 to 691.95) | 724.78 (436.51 to 1123.35) | 1080.62 (575.85 to 1904.83) | 908.70 (495.92 to 1692.71) | 760.21 (409.91 to 1345.01) | 657.21 (351.98 to 1207.60) | 573.15 (309.03 to 965.98) | 474.87 (257.57 to 836.85) | 374.52 (209.57 to 678.90) | 285.97 (150.51 to 536.57) | 219.33 (120.00 to 380.29) | 169.19 (90.54 to 307.65) | 129.05 (69.00 to 234.64) | 89.53 (48.14 to 155.05) | 57.04 (30.60 to 98.66) | 35.08 (19.99 to 61.39) | 17.63 (9.99 to 30.46) | 6.93 (3.81 to 11.64) | 1.85 (1.07 to 3.03) | 0.35 (0.21 to 0.56) |
| Cameroon | Both | Number of prevalence | 5164.77 (4603.01 to 6342.08) | 8667.72 (7499.67 to 11351.40) | 11845.85 (8623.29 to 18000.60) | 10668.41 (7652.02 to 15856.10) | 9428.26 (6721.68 to 14256.34) | 8498.82 (6008.30 to 13065.21) | 7634.62 (5361.00 to 12122.17) | 6455.16 (4523.48 to 10053.83) | 5145.14 (3582.14 to 8053.95) | 4034.38 (2793.74 to 6350.20) | 3142.65 (2172.30 to 4994.32) | 2478.53 (1712.18 to 3967.36) | 1937.83 (1328.77 to 3124.63) | 1381.46 (944.71 to 2237.55) | 886.07 (606.57 to 1432.61) | 558.51 (384.54 to 904.24) | 290.83 (201.52 to 471.34) | 117.83 (82.05 to 191.28) | 32.36 (22.68 to 51.77) | 6.36 (4.51 to 10.09) |
| Cameroon | Female | Number of DALYs | 264.48 (150.34 to 412.16) | 414.50 (248.83 to 663.81) | 365.94 (207.09 to 560.64) | 318.43 (178.11 to 522.38) | 272.10 (155.46 to 426.97) | 236.81 (137.97 to 370.16) | 207.10 (115.73 to 318.75) | 168.15 (100.21 to 253.79) | 129.38 (76.53 to 204.63) | 96.94 (54.88 to 152.81) | 76.27 (45.82 to 121.75) | 59.53 (34.95 to 91.02) | 45.21 (25.68 to 67.53) | 31.75 (18.02 to 49.96) | 20.55 (12.26 to 32.25) | 13.45 (8.52 to 20.82) | 7.26 (4.42 to 10.87) | 3.08 (1.82 to 4.96) | 0.91 (0.56 to 1.39) | 0.19 (0.12 to 0.30) |
| Cameroon | Female | Number of prevalence | 2910.70 (2608.49 to 3481.62) | 4853.29 (4236.73 to 6186.78) | 4658.19 (3931.54 to 6379.29) | 4325.70 (3515.09 to 6310.26) | 3959.32 (3125.18 to 6029.24) | 3630.72 (2795.31 to 5695.22) | 3266.64 (2477.02 to 5227.20) | 2742.49 (2053.25 to 4450.08) | 2146.27 (1589.21 to 3519.97) | 1663.85 (1225.10 to 2755.88) | 1317.14 (959.22 to 2202.24) | 1053.34 (762.45 to 1775.69) | 821.83 (589.90 to 1399.31) | 591.76 (423.13 to 1011.24) | 385.57 (275.24 to 658.92) | 254.21 (181.60 to 434.52) | 142.76 (101.95 to 244.02) | 61.69 (44.14 to 105.47) | 18.42 (13.16 to 31.48) | 4.05 (2.89 to 6.93) |
| Cameroon | Male | Number of DALYs | 197.02 (111.06 to 311.76) | 310.28 (166.67 to 513.30) | 714.69 (316.09 to 1466.47) | 590.27 (273.30 to 1300.13) | 488.11 (217.35 to 1015.96) | 420.40 (185.64 to 893.03) | 366.05 (163.50 to 740.28) | 306.72 (132.15 to 624.00) | 245.14 (112.39 to 514.86) | 189.03 (83.03 to 412.76) | 143.06 (62.12 to 300.14) | 109.66 (46.33 to 234.24) | 83.83 (34.80 to 181.80) | 57.78 (26.24 to 115.72) | 36.49 (16.27 to 76.22) | 21.63 (10.08 to 46.21) | 10.37 (4.75 to 21.81) | 3.85 (1.76 to 7.83) | 0.94 (0.43 to 1.92) | 0.15 (0.07 to 0.33) |
| Cameroon | Male | Number of prevalence | 2254.07 (1947.22 to 2845.99) | 3814.43 (3190.53 to 5132.18) | 7187.66 (4437.35 to 12818.00) | 6342.72 (3905.56 to 11440.47) | 5468.95 (3363.63 to 9643.74) | 4868.10 (3002.34 to 8563.10) | 4367.99 (2696.64 to 7678.81) | 3712.67 (2290.36 to 6527.09) | 2998.86 (1844.43 to 5244.32) | 2370.52 (1457.13 to 4142.48) | 1825.51 (1120.73 to 3176.21) | 1425.18 (873.54 to 2459.73) | 1116.00 (681.12 to 1908.20) | 789.70 (480.89 to 1350.34) | 500.50 (304.98 to 855.85) | 304.30 (185.73 to 520.13) | 148.07 (90.35 to 253.27) | 56.14 (34.28 to 95.97) | 13.94 (8.52 to 23.81) | 2.31 (1.41 to 3.93) |
| Central African Republic | Both | Number of DALYs | 84.15 (53.91 to 126.83) | 299.21 (159.92 to 547.33) | 333.16 (170.93 to 603.22) | 365.05 (196.75 to 619.49) | 360.95 (202.95 to 600.12) | 319.16 (181.10 to 535.35) | 300.96 (172.36 to 484.89) | 289.51 (168.19 to 482.34) | 266.86 (153.97 to 435.87) | 240.82 (137.11 to 387.46) | 201.23 (119.67 to 321.03) | 155.58 (93.87 to 251.56) | 112.47 (68.38 to 174.60) | 67.93 (42.13 to 103.03) | 35.43 (22.08 to 55.00) | 17.14 (10.65 to 26.13) | 6.56 (4.28 to 9.86) | 1.93 (1.21 to 2.90) | 0.37 (0.24 to 0.57) | 0.06 (0.04 to 0.09) |
| Central African Republic | Both | Number of prevalence | 5815.63 (1509.18 to 18440.30) | 14002.33 (3919.48 to 42405.86) | 18139.44 (4707.09 to 56303.04) | 21432.87 (5387.98 to 67208.59) | 21912.47 (5450.40 to 69001.89) | 19528.91 (4812.68 to 61542.60) | 18392.51 (4529.61 to 57930.25) | 17239.22 (4295.50 to 54109.80) | 15558.56 (3925.54 to 48664.97) | 13638.58 (3497.86 to 42467.01) | 11385.46 (2926.86 to 35405.74) | 9009.96 (2314.45 to 28060.77) | 6738.96 (1731.12 to 21050.02) | 4301.31 (1093.58 to 13496.70) | 2404.95 (602.40 to 7592.65) | 1265.24 (310.62 to 4025.92) | 529.91 (127.13 to 1697.60) | 164.24 (39.00 to 527.75) | 32.68 (7.74 to 105.10) | 5.75 (1.34 to 18.55) |
| Central African Republic | Female | Number of DALYs | 48.88 (28.95 to 76.95) | 77.61 (44.36 to 119.31) | 76.32 (44.71 to 122.86) | 72.38 (39.68 to 108.83) | 65.84 (41.04 to 100.79) | 54.87 (31.85 to 85.13) | 48.77 (30.36 to 75.39) | 41.86 (24.82 to 64.39) | 35.51 (21.98 to 54.54) | 28.73 (18.21 to 42.97) | 23.38 (14.82 to 35.22) | 18.78 (11.74 to 29.01) | 14.32 (9.00 to 22.06) | 9.65 (6.28 to 14.35) | 5.60 (3.52 to 8.44) | 3.20 (1.97 to 4.68) | 1.37 (0.90 to 2.02) | 0.43 (0.27 to 0.62) | 0.08 (0.05 to 0.12) | 0.02 (0.01 to 0.02) |
| Central African Republic | Female | Number of prevalence | 2928.84 (819.27 to 9131.19) | 6378.10 (1548.86 to 20559.15) | 8313.61 (1797.77 to 27430.26) | 9929.01 (1992.47 to 33211.95) | 10462.22 (2013.67 to 35242.32) | 9500.19 (1788.93 to 32116.18) | 8968.40 (1666.14 to 30384.47) | 8130.41 (1497.51 to 27584.45) | 7109.82 (1301.63 to 24148.70) | 5948.83 (1083.44 to 20223.12) | 4958.63 (898.56 to 16867.64) | 4062.44 (732.28 to 13828.78) | 3210.66 (575.88 to 10937.69) | 2176.04 (389.41 to 7415.63) | 1301.83 (232.98 to 4436.16) | 742.43 (132.87 to 2529.91) | 331.94 (59.43 to 1131.09) | 105.78 (18.95 to 360.46) | 21.18 (3.79 to 72.19) | 3.87 (0.69 to 13.18) |
| Central African Republic | Male | Number of DALYs | 35.28 (19.99 to 55.87) | 221.60 (107.26 to 451.46) | 256.84 (117.48 to 499.48) | 292.67 (143.59 to 539.11) | 295.11 (154.30 to 517.67) | 264.29 (140.57 to 464.03) | 252.19 (134.80 to 423.99) | 247.65 (135.14 to 418.98) | 231.35 (125.49 to 393.10) | 212.09 (115.65 to 351.84) | 177.84 (101.29 to 290.31) | 136.80 (80.39 to 227.97) | 98.15 (57.96 to 157.22) | 58.28 (34.66 to 90.94) | 29.83 (17.81 to 48.36) | 13.94 (8.09 to 22.32) | 5.19 (3.16 to 8.24) | 1.50 (0.88 to 2.36) | 0.29 (0.18 to 0.45) | 0.05 (0.03 to 0.07) |
| Central African Republic | Male | Number of prevalence | 2886.79 (706.33 to 9309.11) | 7624.23 (2426.42 to 21694.37) | 9825.83 (2935.42 to 28675.60) | 11503.86 (3398.76 to 33830.39) | 11450.26 (3438.72 to 33615.14) | 10028.72 (3068.33 to 29333.82) | 9424.12 (2926.91 to 27466.11) | 9108.81 (2875.70 to 26462.87) | 8448.74 (2705.48 to 24475.68) | 7689.75 (2492.03 to 22217.14) | 6426.83 (2106.22 to 18521.24) | 4947.51 (1635.84 to 14218.59) | 3528.30 (1183.60 to 10112.08) | 2125.28 (718.65 to 6081.07) | 1103.12 (372.50 to 3156.49) | 522.81 (176.54 to 1496.01) | 197.97 (67.03 to 566.51) | 58.46 (19.77 to 167.29) | 11.50 (3.88 to 32.91) | 1.88 (0.63 to 5.37) |
| Chad | Both | Number of DALYs | 330.09 (204.08 to 496.34) | 824.02 (440.10 to 1471.93) | 682.20 (350.98 to 1228.97) | 555.66 (302.62 to 1007.93) | 438.08 (233.34 to 773.25) | 343.24 (194.03 to 623.61) | 275.67 (146.16 to 491.30) | 224.54 (119.66 to 389.67) | 186.85 (100.52 to 335.40) | 152.63 (83.31 to 292.85) | 120.70 (64.28 to 217.11) | 93.88 (49.76 to 172.04) | 71.05 (38.11 to 127.42) | 54.09 (28.91 to 100.19) | 34.22 (17.95 to 62.21) | 21.94 (11.46 to 39.65) | 10.93 (6.04 to 19.38) | 4.24 (2.36 to 7.72) | 0.98 (0.54 to 1.81) | 0.23 (0.13 to 0.37) |
| Chad | Both | Number of prevalence | 6265.11 (3625.11 to 14977.60) | 13854.73 (7415.83 to 33318.66) | 13641.28 (6439.87 to 36874.45) | 13281.24 (5670.02 to 38921.91) | 11770.98 (4682.33 to 35887.96) | 10037.32 (3788.93 to 31310.40) | 8520.94 (3122.89 to 26958.37) | 7235.17 (2596.43 to 23103.12) | 6164.59 (2178.91 to 19802.89) | 5172.94 (1807.38 to 16681.14) | 4135.50 (1429.27 to 13370.59) | 3270.65 (1118.45 to 10605.84) | 2521.21 (851.83 to 8206.69) | 1967.08 (661.98 to 6412.55) | 1263.18 (424.95 to 4117.08) | 834.51 (280.58 to 2723.25) | 423.78 (141.91 to 1385.81) | 172.00 (57.48 to 564.35) | 42.31 (14.10 to 139.57) | 10.30 (3.42 to 34.13) |
| Chad | Female | Number of DALYs | 189.69 (110.77 to 296.28) | 279.63 (163.05 to 431.26) | 229.68 (129.93 to 363.55) | 186.51 (100.88 to 284.46) | 148.33 (84.69 to 228.59) | 116.75 (71.86 to 176.59) | 95.00 (53.15 to 147.08) | 77.12 (44.02 to 119.05) | 61.33 (35.19 to 95.53) | 47.06 (27.21 to 70.99) | 34.65 (21.11 to 51.71) | 25.81 (14.91 to 39.80) | 18.42 (11.10 to 28.01) | 13.45 (7.95 to 19.70) | 8.55 (5.31 to 13.12) | 5.63 (3.38 to 8.46) | 3.02 (1.80 to 4.59) | 1.28 (0.73 to 1.96) | 0.34 (0.20 to 0.52) | 0.09 (0.05 to 0.13) |
| Chad | Female | Number of prevalence | 3354.71 (2041.90 to 7671.54) | 5876.87 (3127.18 to 15098.08) | 5978.19 (2735.97 to 16927.83) | 6011.26 (2427.76 to 18234.59) | 5516.06 (2034.23 to 17382.34) | 4822.45 (1687.01 to 15523.60) | 4186.98 (1415.44 to 13650.63) | 3567.45 (1173.63 to 11736.04) | 2946.99 (954.27 to 9764.94) | 2381.56 (757.34 to 7935.51) | 1799.63 (565.34 to 6025.81) | 1358.72 (419.63 to 4571.75) | 1011.13 (307.70 to 3420.34) | 769.85 (232.80 to 2609.98) | 492.04 (148.54 to 1667.99) | 336.03 (101.67 to 1139.00) | 179.60 (54.25 to 608.88) | 78.73 (23.76 to 266.90) | 21.63 (6.53 to 73.34) | 5.70 (1.72 to 19.34) |
| Chad | Male | Number of DALYs | 140.40 (77.97 to 219.79) | 544.40 (233.83 to 1114.13) | 452.53 (196.55 to 957.73) | 369.15 (163.04 to 783.93) | 289.75 (120.21 to 618.12) | 226.49 (105.52 to 470.12) | 180.66 (77.69 to 371.37) | 147.42 (61.61 to 310.09) | 125.52 (52.93 to 255.28) | 105.57 (45.58 to 232.05) | 86.04 (37.80 to 175.81) | 68.08 (29.51 to 138.94) | 52.63 (23.76 to 103.28) | 40.64 (18.81 to 84.83) | 25.67 (11.86 to 50.63) | 16.31 (7.31 to 33.45) | 7.90 (3.68 to 15.71) | 2.97 (1.41 to 6.07) | 0.64 (0.29 to 1.37) | 0.14 (0.06 to 0.27) |
| Chad | Male | Number of prevalence | 2910.40 (1573.96 to 7306.07) | 7977.87 (4070.82 to 17462.26) | 7663.09 (3594.81 to 18937.40) | 7269.98 (3122.20 to 19483.92) | 6254.91 (2534.51 to 17661.76) | 5214.88 (2038.21 to 15172.60) | 4333.96 (1657.01 to 12839.40) | 3667.71 (1379.80 to 10999.92) | 3217.60 (1195.98 to 9745.07) | 2791.38 (1026.75 to 8520.89) | 2335.87 (848.75 to 7178.19) | 1911.93 (689.39 to 5914.66) | 1510.08 (539.22 to 4704.67) | 1197.23 (425.04 to 3741.94) | 771.14 (273.95 to 2410.30) | 498.49 (176.96 to 1557.74) | 244.18 (86.71 to 763.20) | 93.27 (33.14 to 291.50) | 20.67 (7.35 to 64.62) | 4.60 (1.63 to 14.38) |
| Comoros | Both | Number of DALYs | 8.29 (5.10 to 12.90) | 30.31 (16.17 to 54.00) | 34.96 (18.92 to 62.16) | 39.76 (20.92 to 71.23) | 43.11 (23.59 to 73.97) | 44.69 (24.10 to 74.33) | 43.61 (23.72 to 75.58) | 40.54 (22.18 to 67.82) | 38.10 (21.54 to 62.08) | 34.88 (20.42 to 57.72) | 29.65 (16.80 to 48.43) | 23.89 (13.89 to 39.32) | 17.87 (10.57 to 29.37) | 13.43 (7.96 to 20.87) | 9.28 (5.62 to 14.84) | 5.95 (3.51 to 9.37) | 3.25 (1.96 to 5.10) | 1.36 (0.80 to 2.15) | 0.39 (0.24 to 0.60) | 0.09 (0.05 to 0.13) |
| Comoros | Both | Number of prevalence | 505.98 (88.20 to 2423.82) | 1310.24 (251.95 to 6052.81) | 1780.54 (297.32 to 8482.42) | 2235.52 (344.98 to 10813.46) | 2512.40 (376.83 to 12217.64) | 2623.60 (390.45 to 12773.02) | 2534.48 (379.30 to 12330.91) | 2351.67 (355.32 to 11429.22) | 2197.81 (335.03 to 10672.26) | 2028.77 (311.82 to 9843.77) | 1732.26 (265.99 to 8405.40) | 1417.19 (216.84 to 6884.02) | 1105.07 (166.52 to 5382.36) | 853.30 (127.38 to 4164.50) | 607.32 (90.00 to 2967.08) | 397.28 (58.62 to 1941.09) | 218.86 (32.64 to 1068.08) | 94.06 (13.97 to 459.33) | 28.04 (4.08 to 137.33) | 6.84 (0.95 to 33.75) |
| Comoros | Female | Number of DALYs | 4.78 (2.81 to 7.72) | 8.27 (4.91 to 13.03) | 8.44 (5.05 to 13.38) | 8.35 (4.98 to 12.70) | 8.02 (4.73 to 12.10) | 7.66 (4.81 to 11.64) | 6.97 (4.21 to 10.77) | 6.13 (3.72 to 9.03) | 5.51 (3.28 to 8.76) | 4.95 (3.02 to 7.56) | 4.11 (2.50 to 6.17) | 3.34 (2.03 to 4.85) | 2.66 (1.65 to 3.97) | 2.07 (1.27 to 3.14) | 1.47 (0.92 to 2.22) | 0.94 (0.59 to 1.38) | 0.51 (0.32 to 0.75) | 0.21 (0.13 to 0.33) | 0.07 (0.04 to 0.10) | 0.02 (0.01 to 0.02) |
| Comoros | Female | Number of prevalence | 254.51 (50.26 to 1193.96) | 591.13 (89.14 to 2898.97) | 808.40 (94.73 to 4088.00) | 1019.06 (98.50 to 5248.28) | 1148.84 (98.74 to 5972.63) | 1196.44 (95.69 to 6250.73) | 1147.89 (88.03 to 6014.22) | 1055.54 (78.63 to 5541.37) | 982.08 (71.54 to 5163.49) | 905.89 (64.83 to 4768.37) | 781.88 (55.00 to 4119.89) | 657.53 (45.56 to 3468.03) | 537.54 (36.63 to 2838.01) | 428.07 (28.95 to 2261.12) | 308.44 (20.86 to 1629.19) | 202.13 (13.66 to 1067.67) | 109.80 (7.43 to 579.99) | 47.58 (3.22 to 251.33) | 14.67 (0.99 to 77.47) | 3.88 (0.26 to 20.50) |
| Comoros | Male | Number of DALYs | 3.51 (1.98 to 5.66) | 22.04 (10.16 to 44.79) | 26.51 (12.56 to 52.66) | 31.42 (14.97 to 59.64) | 35.09 (17.57 to 62.91) | 37.03 (17.95 to 66.09) | 36.64 (18.52 to 66.65) | 34.41 (17.66 to 59.57) | 32.59 (17.65 to 55.48) | 29.93 (16.79 to 51.70) | 25.54 (13.76 to 43.74) | 20.55 (11.23 to 34.79) | 15.21 (8.34 to 25.77) | 11.35 (6.33 to 18.67) | 7.82 (4.41 to 13.09) | 5.01 (2.79 to 8.19) | 2.74 (1.52 to 4.52) | 1.14 (0.64 to 1.90) | 0.32 (0.19 to 0.53) | 0.07 (0.04 to 0.12) |
| Comoros | Male | Number of prevalence | 251.46 (37.64 to 1229.86) | 719.12 (154.02 to 3153.85) | 972.15 (190.56 to 4394.41) | 1216.46 (233.62 to 5565.18) | 1363.55 (264.18 to 6245.00) | 1427.16 (279.91 to 6522.29) | 1386.59 (274.02 to 6316.69) | 1296.13 (260.53 to 5887.84) | 1215.72 (248.84 to 5508.77) | 1122.88 (233.31 to 5075.40) | 950.38 (200.66 to 4285.51) | 759.66 (163.58 to 3416.19) | 567.53 (124.62 to 2545.30) | 425.24 (94.36 to 1904.51) | 298.89 (66.15 to 1338.59) | 195.16 (43.13 to 873.96) | 109.06 (24.16 to 488.39) | 46.48 (10.30 to 208.13) | 13.38 (2.96 to 59.91) | 2.96 (0.66 to 13.25) |
| Congo | Both | Number of DALYs | 62.71 (37.46 to 95.63) | 186.26 (100.87 to 333.76) | 184.49 (100.75 to 337.29) | 165.28 (87.25 to 294.92) | 144.19 (80.54 to 258.26) | 131.58 (70.31 to 240.37) | 122.31 (65.59 to 224.86) | 115.89 (65.02 to 203.39) | 104.70 (56.65 to 187.05) | 89.48 (47.75 to 167.08) | 69.52 (37.62 to 130.10) | 50.38 (27.39 to 90.35) | 34.96 (19.66 to 65.57) | 23.89 (13.07 to 41.88) | 14.08 (8.02 to 23.85) | 8.51 (4.68 to 14.79) | 4.02 (2.24 to 7.04) | 1.43 (0.81 to 2.51) | 0.33 (0.19 to 0.56) | 0.05 (0.03 to 0.08) |
| Congo | Both | Number of prevalence | 1309.49 (699.77 to 3005.19) | 3460.37 (1652.26 to 7913.33) | 4186.94 (1752.10 to 10344.04) | 4488.53 (1688.70 to 11726.96) | 4395.64 (1543.62 to 11868.67) | 4364.82 (1468.89 to 12009.69) | 4303.89 (1407.86 to 11984.93) | 4226.95 (1359.17 to 11864.28) | 3945.73 (1253.03 to 11120.97) | 3385.19 (1064.26 to 9566.64) | 2685.17 (835.65 to 7614.18) | 2024.10 (622.53 to 5767.50) | 1472.02 (446.47 to 4221.83) | 1048.80 (314.87 to 3021.97) | 638.64 (191.47 to 1843.33) | 393.53 (118.01 to 1136.61) | 193.84 (58.03 to 560.83) | 70.44 (21.07 to 204.08) | 16.44 (4.91 to 47.68) | 2.50 (0.75 to 7.29) |
| Congo | Female | Number of DALYs | 36.31 (19.87 to 59.16) | 63.05 (37.74 to 99.82) | 61.12 (34.16 to 96.88) | 54.38 (29.55 to 85.37) | 46.74 (25.97 to 74.18) | 41.79 (24.16 to 65.58) | 39.00 (22.08 to 60.26) | 36.33 (22.01 to 54.78) | 31.05 (18.88 to 46.92) | 24.92 (14.25 to 38.79) | 18.78 (11.06 to 28.47) | 13.78 (8.20 to 21.13) | 10.10 (6.02 to 15.28) | 7.53 (4.63 to 11.15) | 4.65 (2.80 to 7.08) | 2.84 (1.69 to 4.29) | 1.42 (0.87 to 2.09) | 0.51 (0.29 to 0.77) | 0.12 (0.07 to 0.18) | 0.02 (0.01 to 0.03) |
| Congo | Female | Number of prevalence | 698.35 (394.16 to 1533.33) | 1493.79 (729.36 to 3626.16) | 1855.65 (769.09 to 4892.25) | 2041.99 (740.47 to 5685.08) | 2041.75 (676.26 to 5864.06) | 2047.37 (643.60 to 5982.64) | 2046.81 (623.47 to 6041.88) | 2008.25 (595.21 to 5969.77) | 1812.34 (527.16 to 5418.70) | 1486.88 (425.53 to 4464.49) | 1157.89 (326.92 to 3490.27) | 889.17 (247.74 to 2690.43) | 679.77 (186.47 to 2065.53) | 512.12 (139.69 to 1558.86) | 323.23 (87.89 to 983.95) | 202.23 (55.01 to 615.66) | 102.65 (27.97 to 312.46) | 38.20 (10.42 to 116.29) | 9.16 (2.49 to 27.87) | 1.47 (0.40 to 4.46) |
| Congo | Male | Number of DALYs | 26.39 (14.84 to 42.89) | 123.21 (54.90 to 254.48) | 123.37 (52.62 to 258.22) | 110.90 (49.04 to 225.54) | 97.46 (44.95 to 207.60) | 89.79 (40.10 to 183.62) | 83.31 (36.04 to 172.03) | 79.55 (38.05 to 159.67) | 73.66 (32.68 to 151.85) | 64.56 (29.30 to 135.13) | 50.75 (22.43 to 105.59) | 36.59 (16.73 to 74.79) | 24.86 (11.48 to 51.82) | 16.36 (7.21 to 33.07) | 9.43 (4.29 to 18.36) | 5.67 (2.71 to 11.53) | 2.60 (1.22 to 5.16) | 0.91 (0.41 to 1.85) | 0.20 (0.10 to 0.41) | 0.03 (0.01 to 0.06) |
| Congo | Male | Number of prevalence | 611.14 (305.46 to 1463.41) | 1966.57 (878.72 to 4238.49) | 2331.29 (963.48 to 5513.71) | 2446.54 (922.83 to 6144.78) | 2353.89 (843.05 to 6088.08) | 2317.46 (804.63 to 6101.74) | 2257.08 (769.23 to 6006.28) | 2218.69 (747.28 to 5953.19) | 2133.39 (710.22 to 5758.36) | 1898.31 (625.43 to 5149.98) | 1527.27 (499.29 to 4163.20) | 1134.94 (367.09 to 3106.10) | 792.25 (254.02 to 2179.53) | 536.69 (171.01 to 1479.08) | 315.41 (100.63 to 868.71) | 191.29 (61.14 to 527.03) | 91.19 (29.04 to 251.27) | 32.24 (10.27 to 88.82) | 7.28 (2.32 to 20.06) | 1.04 (0.33 to 2.86) |
| Côte d'Ivoire | Both | Number of DALYs | 466.45 (282.76 to 726.54) | 2106.76 (1150.48 to 3690.84) | 2537.87 (1334.85 to 4263.19) | 3071.51 (1757.06 to 5073.53) | 3492.82 (2102.64 to 5471.51) | 3634.93 (2224.02 to 5646.88) | 3741.18 (2357.08 to 5811.57) | 3669.53 (2297.48 to 5353.69) | 3298.27 (2114.76 to 4916.81) | 2710.83 (1802.67 to 3958.67) | 2094.48 (1365.03 to 3056.20) | 1625.14 (1070.47 to 2350.12) | 1266.79 (841.06 to 1800.06) | 873.52 (579.00 to 1264.95) | 539.35 (362.49 to 761.62) | 307.53 (204.31 to 438.44) | 145.31 (99.47 to 205.52) | 56.96 (38.58 to 79.35) | 15.27 (10.42 to 21.18) | 2.66 (1.82 to 3.60) |
| Côte d'Ivoire | Both | Number of prevalence | 48334.60 (15923.20 to 119414.60) | 116302.54 (44886.30 to 279085.17) | 144008.41 (54072.10 to 346014.29) | 166686.37 (62765.21 to 398032.62) | 180166.44 (67784.70 to 427474.63) | 182282.09 (68329.39 to 430541.34) | 182883.40 (68749.30 to 430202.67) | 171792.12 (65542.54 to 401314.54) | 146943.15 (57130.00 to 340530.20) | 116899.72 (46011.68 to 269535.54) | 91062.89 (35840.84 to 209708.51) | 71586.71 (28151.20 to 164838.43) | 55883.99 (22044.33 to 128444.79) | 38905.41 (15382.94 to 89297.76) | 24759.45 (9748.44 to 56930.62) | 14923.10 (5805.63 to 34460.81) | 7529.14 (2871.12 to 17505.33) | 3200.45 (1194.17 to 7497.29) | 958.44 (347.43 to 2268.12) | 203.05 (70.23 to 488.14) |
| Côte d'Ivoire | Female | Number of DALYs | 267.28 (153.70 to 405.86) | 432.28 (259.63 to 669.75) | 398.04 (242.85 to 610.79) | 377.31 (230.91 to 578.58) | 365.84 (233.34 to 537.20) | 352.75 (220.76 to 531.02) | 337.83 (209.91 to 496.61) | 294.62 (188.87 to 433.95) | 228.51 (140.62 to 342.55) | 172.20 (106.21 to 260.81) | 134.21 (83.23 to 196.68) | 106.22 (68.36 to 153.59) | 82.25 (53.38 to 123.46) | 55.56 (36.56 to 81.17) | 35.89 (22.51 to 50.17) | 22.61 (14.73 to 32.29) | 12.15 (7.89 to 17.50) | 5.48 (3.65 to 7.81) | 1.82 (1.18 to 2.63) | 0.43 (0.29 to 0.61) |
| Côte d'Ivoire | Female | Number of prevalence | 23960.73 (8140.55 to 58581.47) | 51602.30 (16425.09 to 128486.67) | 62549.87 (19024.60 to 157684.68) | 71379.85 (21106.20 to 181190.32) | 77753.99 (22683.90 to 198040.54) | 80078.91 (23178.77 to 204312.27) | 79882.89 (23041.75 to 203989.47) | 71509.11 (20578.52 to 182705.59) | 56671.28 (16288.31 to 144857.28) | 43245.56 (12401.24 to 110571.56) | 34252.34 (9814.55 to 87603.56) | 27757.77 (7946.01 to 71004.78) | 21775.09 (6230.00 to 55711.36) | 15079.92 (4311.02 to 38587.26) | 9815.17 (2805.39 to 25114.10) | 6273.18 (1794.22 to 16051.58) | 3434.32 (981.66 to 8788.04) | 1589.35 (454.37 to 4066.81) | 530.03 (151.52 to 1356.31) | 130.12 (37.21 to 332.95) |
| Côte d'Ivoire | Male | Number of DALYs | 199.16 (114.87 to 316.04) | 1674.48 (781.34 to 3175.88) | 2139.82 (1043.48 to 3756.88) | 2694.20 (1466.49 to 4636.33) | 3126.97 (1844.86 to 5034.81) | 3282.18 (1963.22 to 5148.00) | 3403.35 (2085.41 to 5336.21) | 3374.91 (2101.76 to 4978.96) | 3069.76 (1943.30 to 4605.88) | 2538.64 (1664.89 to 3714.47) | 1960.26 (1257.43 to 2877.62) | 1518.92 (976.63 to 2213.31) | 1184.54 (770.84 to 1695.09) | 817.96 (532.57 to 1188.81) | 503.46 (334.57 to 720.50) | 284.93 (188.82 to 410.29) | 133.16 (89.90 to 189.52) | 51.48 (34.72 to 72.48) | 13.46 (8.93 to 18.68) | 2.23 (1.51 to 3.06) |
| Côte d'Ivoire | Male | Number of prevalence | 24373.87 (7701.45 to 60833.14) | 64700.24 (27477.67 to 149983.45) | 81458.54 (34767.65 to 188338.39) | 95306.52 (41574.47 to 216315.78) | 102412.45 (45178.85 to 228581.15) | 102203.17 (45815.12 to 225304.22) | 103000.50 (46810.13 to 225276.69) | 100283.01 (45794.02 to 217848.88) | 90271.87 (41395.71 to 195274.21) | 73654.17 (34028.09 to 158658.29) | 56810.55 (26395.26 to 121968.53) | 43828.94 (20434.70 to 93760.55) | 34108.90 (15933.90 to 72727.29) | 23825.49 (11162.63 to 50711.18) | 14944.29 (7001.46 to 31817.65) | 8649.92 (4052.94 to 18409.70) | 4094.82 (1918.98 to 8717.33) | 1611.09 (753.75 to 3430.39) | 428.41 (200.71 to 911.82) | 72.93 (34.34 to 155.20) |
| Democratic Republic of the Congo | Both | Number of DALYs | 1341.88 (822.01 to 2060.32) | 4117.36 (2161.60 to 7883.42) | 4027.60 (2163.87 to 7284.26) | 3855.25 (2084.99 to 6951.61) | 3528.78 (1854.87 to 6161.75) | 3213.93 (1732.44 to 5625.32) | 2832.06 (1522.44 to 4940.38) | 2435.27 (1300.24 to 4258.31) | 2029.87 (1104.49 to 3635.57) | 1694.66 (921.29 to 2959.03) | 1410.34 (770.14 to 2460.66) | 1123.19 (632.95 to 2006.71) | 825.98 (450.92 to 1445.70) | 557.48 (307.27 to 933.92) | 294.63 (168.61 to 502.23) | 162.66 (94.73 to 270.35) | 79.35 (46.21 to 129.54) | 30.78 (18.23 to 50.52) | 8.09 (4.92 to 12.71) | 1.54 (0.93 to 2.37) |
| Democratic Republic of the Congo | Both | Number of prevalence | 53736.76 (28215.69 to 102608.22) | 132336.50 (70491.24 to 252777.47) | 161179.76 (81910.25 to 315494.17) | 179775.61 (87940.90 to 357688.54) | 180869.76 (86729.93 to 361427.08) | 171239.11 (81093.66 to 342765.71) | 156494.28 (73519.60 to 313586.38) | 138129.46 (64572.90 to 277437.45) | 116706.00 (54319.26 to 234649.99) | 98634.66 (45789.32 to 198429.59) | 83877.26 (38852.20 to 168851.25) | 69160.62 (31903.04 to 139463.69) | 53001.52 (24374.06 to 107103.27) | 36956.31 (16945.46 to 74820.80) | 20926.37 (9547.84 to 42518.38) | 12117.01 (5509.14 to 24687.72) | 6282.44 (2852.62 to 12837.67) | 2591.58 (1175.10 to 5310.09) | 727.03 (329.17 to 1494.03) | 153.47 (69.12 to 316.57) |
| Democratic Republic of the Congo | Female | Number of DALYs | 781.66 (445.71 to 1212.41) | 1242.66 (694.97 to 1980.35) | 1147.41 (688.64 to 1782.16) | 1016.94 (619.33 to 1549.94) | 858.74 (505.93 to 1349.67) | 740.52 (424.83 to 1130.22) | 622.19 (352.53 to 938.54) | 523.88 (316.95 to 788.22) | 427.53 (253.98 to 651.02) | 349.67 (207.43 to 525.65) | 286.88 (169.21 to 436.48) | 232.82 (144.78 to 346.82) | 179.25 (107.00 to 261.86) | 128.01 (76.79 to 196.20) | 77.55 (46.44 to 119.93) | 46.66 (28.10 to 69.90) | 25.20 (15.47 to 38.24) | 10.73 (6.45 to 16.20) | 3.10 (1.91 to 4.53) | 0.67 (0.40 to 0.97) |
| Democratic Republic of the Congo | Female | Number of prevalence | 27521.00 (14893.75 to 51606.81) | 59232.71 (29685.03 to 115510.46) | 72858.37 (34265.36 to 146530.52) | 81556.29 (36763.47 to 167320.19) | 82112.36 (36087.51 to 170184.30) | 77660.84 (33659.96 to 161865.80) | 70864.60 (30479.39 to 148168.67) | 62455.83 (26700.21 to 130896.20) | 52752.71 (22457.45 to 110753.24) | 44379.78 (18839.97 to 93299.80) | 38077.06 (16099.65 to 80147.59) | 32343.46 (13638.26 to 68158.78) | 25723.36 (10814.12 to 54275.01) | 18683.00 (7842.34 to 39442.69) | 11463.98 (4812.16 to 24202.32) | 7024.21 (2946.93 to 14829.88) | 3862.41 (1620.97 to 8154.42) | 1674.23 (702.43 to 3534.44) | 494.79 (207.67 to 1044.63) | 111.70 (46.87 to 235.80) |
| Democratic Republic of the Congo | Male | Number of DALYs | 560.22 (303.05 to 907.54) | 2874.71 (1238.03 to 6247.05) | 2880.19 (1289.76 to 6089.74) | 2838.31 (1305.93 to 5607.65) | 2670.04 (1198.79 to 5157.23) | 2473.41 (1129.63 to 4733.34) | 2209.87 (1013.28 to 4216.67) | 1911.39 (919.61 to 3628.25) | 1602.34 (774.39 to 3086.94) | 1344.98 (617.91 to 2554.39) | 1123.46 (545.93 to 2105.28) | 890.37 (435.30 to 1673.93) | 646.73 (314.50 to 1229.43) | 429.47 (212.17 to 786.64) | 217.07 (108.46 to 393.68) | 116.00 (57.74 to 209.72) | 54.15 (26.63 to 99.01) | 20.05 (10.28 to 36.13) | 4.99 (2.47 to 9.04) | 0.87 (0.44 to 1.58) |
| Democratic Republic of the Congo | Male | Number of prevalence | 26215.76 (13210.72 to 51168.14) | 73103.79 (39700.55 to 135693.96) | 88321.39 (45893.80 to 170647.25) | 98219.32 (49319.96 to 192931.11) | 98757.40 (49073.08 to 194744.83) | 93578.27 (46203.40 to 184763.12) | 85629.68 (42061.89 to 169119.47) | 75673.63 (37148.94 to 149443.61) | 63953.28 (31341.87 to 126292.45) | 54254.88 (26562.73 to 107116.16) | 45800.20 (22404.46 to 90319.77) | 36817.16 (17972.08 to 72569.92) | 27278.16 (13333.08 to 53729.33) | 18273.32 (8920.47 to 35964.37) | 9462.39 (4623.37 to 18644.84) | 5092.80 (2485.47 to 10020.19) | 2420.03 (1182.61 to 4759.33) | 917.35 (447.79 to 1805.65) | 232.25 (113.40 to 457.16) | 41.77 (20.39 to 82.32) |
| Dominican Republic | Both | Number of DALYs | 101.44 (60.86 to 149.23) | 288.36 (157.96 to 529.04) | 287.53 (154.94 to 526.49) | 311.99 (168.50 to 574.06) | 332.98 (182.80 to 592.67) | 334.80 (182.12 to 624.37) | 320.57 (175.28 to 572.82) | 291.45 (156.44 to 517.70) | 262.83 (142.84 to 467.29) | 232.04 (125.18 to 408.48) | 203.92 (114.98 to 360.01) | 172.46 (91.19 to 317.50) | 139.12 (78.62 to 242.94) | 107.46 (59.87 to 187.31) | 78.61 (43.64 to 139.57) | 54.69 (30.37 to 96.48) | 34.27 (19.48 to 57.60) | 19.01 (10.75 to 31.94) | 8.07 (4.65 to 13.74) | 1.86 (1.08 to 3.15) |
| Dominican Republic | Both | Number of prevalence | 2721.99 (1144.91 to 8313.76) | 6655.16 (2567.07 to 20090.11) | 8254.74 (2739.72 to 27116.71) | 10691.67 (3168.11 to 36785.90) | 12880.67 (3561.64 to 45403.75) | 13983.29 (3713.55 to 49929.59) | 13901.20 (3600.10 to 50006.83) | 13027.95 (3312.37 to 47089.94) | 12109.01 (3034.79 to 43944.07) | 11037.24 (2736.83 to 40211.61) | 10020.62 (2468.98 to 36632.39) | 8774.64 (2139.67 to 32152.38) | 7294.65 (1757.53 to 26812.59) | 5795.77 (1388.91 to 21357.94) | 4326.71 (1035.82 to 15943.18) | 3063.33 (731.37 to 11303.34) | 1977.67 (470.56 to 7304.22) | 1139.93 (269.99 to 4215.84) | 493.01 (116.42 to 1824.16) | 119.09 (27.97 to 441.60) |
| Dominican Republic | Female | Number of DALYs | 57.73 (33.43 to 88.82) | 92.56 (54.94 to 140.57) | 89.47 (52.70 to 139.13) | 93.92 (55.35 to 146.59) | 96.80 (58.11 to 151.20) | 92.83 (54.10 to 143.95) | 86.70 (52.41 to 129.77) | 77.06 (45.83 to 118.63) | 69.03 (40.42 to 104.17) | 61.29 (35.65 to 92.40) | 54.53 (32.05 to 81.98) | 46.42 (27.88 to 71.31) | 37.55 (22.89 to 56.89) | 29.28 (17.74 to 44.37) | 22.04 (12.87 to 33.75) | 15.60 (9.69 to 23.45) | 10.37 (6.11 to 15.62) | 5.97 (3.52 to 9.33) | 2.59 (1.60 to 3.86) | 0.62 (0.38 to 0.93) |
| Dominican Republic | Female | Number of prevalence | 1415.32 (648.22 to 4147.26) | 2896.16 (1095.48 to 9306.50) | 3669.20 (1147.11 to 12646.78) | 4838.54 (1290.97 to 17450.23) | 5896.62 (1418.50 to 21787.02) | 6405.33 (1454.53 to 23977.05) | 6290.03 (1381.67 to 23732.92) | 5842.85 (1249.29 to 22166.12) | 5452.72 (1141.02 to 20772.73) | 5031.40 (1034.06 to 19233.31) | 4633.57 (938.11 to 17766.41) | 4097.11 (817.66 to 15753.60) | 3434.97 (674.93 to 13246.50) | 2766.03 (539.50 to 10682.85) | 2099.71 (409.39 to 8108.27) | 1512.30 (294.85 to 5840.60) | 1015.43 (198.28 to 3921.41) | 597.89 (116.45 to 2309.18) | 262.41 (51.12 to 1013.45) | 65.31 (12.72 to 252.23) |
| Dominican Republic | Male | Number of DALYs | 43.71 (22.80 to 70.77) | 195.80 (85.73 to 416.28) | 198.06 (84.91 to 415.45) | 218.06 (93.95 to 465.85) | 236.17 (109.07 to 471.73) | 241.97 (109.55 to 496.91) | 233.87 (107.30 to 475.90) | 214.40 (97.59 to 422.48) | 193.80 (91.27 to 385.56) | 170.75 (75.12 to 338.18) | 149.40 (68.25 to 295.73) | 126.03 (56.80 to 256.35) | 101.58 (49.79 to 193.70) | 78.18 (37.93 to 152.31) | 56.57 (26.32 to 113.34) | 39.09 (17.61 to 78.15) | 23.90 (11.36 to 45.20) | 13.04 (6.15 to 24.80) | 5.48 (2.57 to 11.01) | 1.24 (0.59 to 2.36) |
| Dominican Republic | Male | Number of prevalence | 1306.67 (493.45 to 4166.50) | 3759.00 (1415.42 to 10674.24) | 4585.54 (1528.13 to 14099.97) | 5853.13 (1759.96 to 18982.66) | 6984.05 (1998.11 to 23347.83) | 7577.97 (2108.14 to 25772.79) | 7611.17 (2081.74 to 26148.42) | 7185.11 (1941.08 to 24849.91) | 6656.28 (1778.59 to 23139.42) | 6005.84 (1592.31 to 20961.79) | 5387.05 (1416.70 to 18859.88) | 4677.53 (1215.88 to 16420.17) | 3859.68 (997.68 to 13583.81) | 3029.74 (780.81 to 10688.59) | 2227.00 (573.61 to 7845.76) | 1551.03 (400.06 to 5470.57) | 962.24 (248.43 to 3388.06) | 542.04 (139.86 to 1909.75) | 230.60 (59.50 to 812.08) | 53.78 (13.90 to 189.69) |
| Egypt | Both | Number of DALYs | 0.00 (0.00 to 0.00) | 0.00 (0.00 to 0.00) | 0.00 (0.00 to 0.00) | 0.00 (0.00 to 0.00) | 0.00 (0.00 to 0.00) | 0.00 (0.00 to 0.00) | 0.00 (0.00 to 0.00) | 0.00 (0.00 to 0.00) | 0.00 (0.00 to 0.00) | 0.00 (0.00 to 0.00) | 0.00 (0.00 to 0.00) | 0.00 (0.00 to 0.00) | 0.00 (0.00 to 0.00) | 0.00 (0.00 to 0.00) | 0.00 (0.00 to 0.00) | 0.00 (0.00 to 0.00) | 0.00 (0.00 to 0.00) | 0.00 (0.00 to 0.00) | 0.00 (0.00 to 0.00) | 0.00 (0.00 to 0.00) |
| Egypt | Both | Number of prevalence | 12494.10 (1346.01 to 54247.50) | 29752.19 (3205.25 to 129179.56) | 37656.79 (4056.82 to 163500.17) | 44783.92 (4824.64 to 194445.10) | 50720.90 (5464.24 to 220222.58) | 52922.60 (5701.43 to 229782.05) | 54527.72 (5874.35 to 236751.21) | 55359.31 (5963.94 to 240361.85) | 48535.83 (5228.84 to 210735.35) | 40264.90 (4337.80 to 174824.21) | 35732.88 (3849.56 to 155146.84) | 32315.16 (3481.36 to 140307.60) | 26949.34 (2903.29 to 117010.00) | 19420.50 (2092.20 to 84320.91) | 11137.90 (1199.90 to 48359.12) | 5814.94 (626.45 to 25247.60) | 2656.34 (286.17 to 11533.42) | 944.00 (101.70 to 4098.71) | 275.31 (29.66 to 1195.36) | 118.01 (12.71 to 512.38) |
| Egypt | Female | Number of DALYs | 0.00 (0.00 to 0.00) | 0.00 (0.00 to 0.00) | 0.00 (0.00 to 0.00) | 0.00 (0.00 to 0.00) | 0.00 (0.00 to 0.00) | 0.00 (0.00 to 0.00) | 0.00 (0.00 to 0.00) | 0.00 (0.00 to 0.00) | 0.00 (0.00 to 0.00) | 0.00 (0.00 to 0.00) | 0.00 (0.00 to 0.00) | 0.00 (0.00 to 0.00) | 0.00 (0.00 to 0.00) | 0.00 (0.00 to 0.00) | 0.00 (0.00 to 0.00) | 0.00 (0.00 to 0.00) | 0.00 (0.00 to 0.00) | 0.00 (0.00 to 0.00) | 0.00 (0.00 to 0.00) | 0.00 (0.00 to 0.00) |
| Egypt | Female | Number of prevalence | 6082.43 (655.27 to 26409.00) | 14369.94 (1548.10 to 62392.15) | 18020.48 (1941.37 to 78242.25) | 21443.91 (2310.18 to 93106.26) | 24782.66 (2669.87 to 107602.62) | 26368.87 (2840.76 to 114489.69) | 26730.28 (2879.69 to 116058.89) | 26727.80 (2879.43 to 116048.11) | 23253.15 (2505.10 to 100961.69) | 19180.55 (2066.35 to 83279.09) | 17049.00 (1836.71 to 74024.20) | 15441.10 (1663.49 to 67042.96) | 12637.84 (1361.49 to 54871.64) | 9028.77 (972.68 to 39201.57) | 5262.09 (566.89 to 22847.23) | 2726.75 (293.76 to 11839.13) | 1128.90 (121.62 to 4901.53) | 236.80 (25.51 to 1028.13) | 13.11 (1.41 to 56.93) | 1.31 (0.14 to 5.69) |
| Egypt | Male | Number of DALYs | 0.00 (0.00 to 0.00) | 0.00 (0.00 to 0.00) | 0.00 (0.00 to 0.00) | 0.00 (0.00 to 0.00) | 0.00 (0.00 to 0.00) | 0.00 (0.00 to 0.00) | 0.00 (0.00 to 0.00) | 0.00 (0.00 to 0.00) | 0.00 (0.00 to 0.00) | 0.00 (0.00 to 0.00) | 0.00 (0.00 to 0.00) | 0.00 (0.00 to 0.00) | 0.00 (0.00 to 0.00) | 0.00 (0.00 to 0.00) | 0.00 (0.00 to 0.00) | 0.00 (0.00 to 0.00) | 0.00 (0.00 to 0.00) | 0.00 (0.00 to 0.00) | 0.00 (0.00 to 0.00) | 0.00 (0.00 to 0.00) |
| Egypt | Male | Number of prevalence | 6411.67 (690.74 to 27838.51) | 15382.24 (1657.15 to 66787.41) | 19636.31 (2115.45 to 85257.92) | 23340.01 (2514.45 to 101338.84) | 25938.24 (2794.37 to 112619.96) | 26553.74 (2860.67 to 115292.37) | 27797.44 (2994.66 to 120692.32) | 28631.51 (3084.52 to 124313.73) | 25282.69 (2723.74 to 109773.66) | 21084.35 (2271.45 to 91545.12) | 18683.89 (2012.84 to 81122.65) | 16874.06 (1817.87 to 73264.65) | 14311.49 (1541.80 to 62138.36) | 10391.73 (1119.52 to 45119.33) | 5875.81 (633.01 to 25511.89) | 3088.19 (332.70 to 13408.47) | 1527.43 (164.55 to 6631.89) | 707.21 (76.19 to 3070.58) | 262.20 (28.25 to 1138.43) | 116.70 (12.57 to 506.69) |
| Equatorial Guinea | Both | Number of DALYs | 18.30 (11.55 to 27.62) | 60.31 (31.92 to 112.10) | 64.04 (31.57 to 121.32) | 63.75 (33.07 to 116.25) | 59.29 (29.98 to 111.82) | 51.11 (26.71 to 94.15) | 42.97 (22.72 to 76.94) | 33.39 (18.12 to 60.29) | 24.02 (13.23 to 42.80) | 17.31 (9.59 to 31.37) | 13.16 (7.12 to 23.06) | 9.28 (5.08 to 16.51) | 6.61 (3.61 to 11.69) | 5.01 (2.87 to 8.86) | 2.82 (1.58 to 4.97) | 1.91 (1.08 to 3.24) | 1.01 (0.58 to 1.65) | 0.37 (0.21 to 0.62) | 0.09 (0.05 to 0.16) | 0.02 (0.01 to 0.03) |
| Equatorial Guinea | Both | Number of prevalence | 497.19 (191.34 to 1882.63) | 1357.71 (502.03 to 4893.72) | 1762.15 (547.97 to 6916.39) | 2072.56 (572.97 to 8600.19) | 2152.97 (549.53 to 9216.86) | 2028.12 (489.91 to 8847.87) | 1806.36 (420.22 to 7983.47) | 1488.78 (337.53 to 6649.16) | 1133.88 (249.32 to 5108.57) | 851.40 (183.12 to 3861.29) | 666.17 (140.22 to 3035.96) | 493.34 (102.44 to 2260.06) | 363.46 (74.34 to 1673.60) | 283.71 (57.65 to 1308.15) | 166.06 (33.55 to 767.54) | 114.86 (23.21 to 530.62) | 61.29 (12.39 to 283.16) | 23.56 (4.72 to 109.13) | 6.23 (1.23 to 29.00) | 1.36 (0.26 to 6.39) |
| Equatorial Guinea | Female | Number of DALYs | 10.14 (6.01 to 15.75) | 17.02 (9.40 to 26.64) | 16.45 (9.81 to 26.86) | 15.13 (9.06 to 23.07) | 13.56 (7.92 to 20.40) | 11.82 (7.08 to 18.14) | 10.60 (6.35 to 16.45) | 8.93 (5.19 to 13.70) | 6.79 (4.02 to 10.52) | 5.14 (3.08 to 7.87) | 4.02 (2.32 to 6.22) | 2.94 (1.75 to 4.48) | 2.14 (1.29 to 3.29) | 1.63 (1.03 to 2.48) | 0.96 (0.58 to 1.51) | 0.65 (0.39 to 0.98) | 0.34 (0.20 to 0.51) | 0.13 (0.08 to 0.20) | 0.04 (0.02 to 0.06) | 0.01 (0.01 to 0.01) |
| Equatorial Guinea | Female | Number of prevalence | 246.98 (103.60 to 893.55) | 531.31 (179.96 to 2125.76) | 664.03 (180.51 to 2866.75) | 768.38 (172.49 to 3482.76) | 812.47 (161.22 to 3779.93) | 801.91 (147.12 to 3785.21) | 760.96 (133.58 to 3622.62) | 671.37 (114.07 to 3215.08) | 543.46 (89.85 to 2614.55) | 425.96 (68.74 to 2056.79) | 340.76 (54.04 to 1650.62) | 260.02 (40.40 to 1263.55) | 196.16 (29.82 to 956.40) | 152.85 (23.06 to 746.31) | 92.60 (13.96 to 452.13) | 63.63 (9.58 to 310.69) | 33.95 (5.12 to 165.77) | 13.55 (2.04 to 66.14) | 3.83 (0.58 to 18.70) | 0.94 (0.14 to 4.60) |
| Equatorial Guinea | Male | Number of DALYs | 8.16 (4.40 to 13.09) | 43.29 (19.55 to 90.82) | 47.59 (19.99 to 102.14) | 48.62 (22.50 to 100.05) | 45.73 (19.98 to 96.72) | 39.29 (17.65 to 80.70) | 32.37 (14.70 to 63.97) | 24.45 (11.51 to 49.36) | 17.23 (8.19 to 34.60) | 12.17 (5.37 to 24.81) | 9.14 (4.24 to 18.25) | 6.34 (2.90 to 12.38) | 4.46 (2.01 to 8.64) | 3.38 (1.47 to 6.61) | 1.86 (0.89 to 3.69) | 1.27 (0.61 to 2.49) | 0.67 (0.33 to 1.28) | 0.24 (0.11 to 0.47) | 0.06 (0.03 to 0.11) | 0.01 (0.00 to 0.02) |
| Equatorial Guinea | Male | Number of prevalence | 250.21 (87.19 to 989.07) | 826.40 (294.99 to 2767.96) | 1098.12 (347.62 to 4049.64) | 1304.18 (373.81 to 5117.43) | 1340.50 (359.60 to 5436.93) | 1226.21 (313.72 to 5062.65) | 1045.40 (261.53 to 4360.86) | 817.41 (200.86 to 3434.08) | 590.42 (143.26 to 2494.02) | 425.43 (102.15 to 1804.49) | 325.40 (77.24 to 1385.34) | 233.32 (54.98 to 996.51) | 167.30 (39.18 to 717.19) | 130.86 (30.52 to 561.84) | 73.46 (17.10 to 315.42) | 51.23 (11.93 to 219.93) | 27.34 (6.35 to 117.39) | 10.01 (2.33 to 42.98) | 2.40 (0.56 to 10.30) | 0.42 (0.10 to 1.79) |
| Eritrea | Both | Number of DALYs | 85.36 (52.51 to 128.36) | 226.99 (121.86 to 417.82) | 206.24 (109.16 to 369.80) | 190.16 (100.54 to 343.38) | 172.45 (90.56 to 310.25) | 155.10 (87.44 to 269.32) | 135.73 (76.09 to 245.37) | 114.97 (61.24 to 212.78) | 95.46 (51.12 to 171.85) | 73.93 (39.46 to 132.29) | 54.23 (30.65 to 93.74) | 40.05 (22.83 to 70.90) | 27.96 (15.33 to 47.90) | 18.68 (9.98 to 32.11) | 10.32 (5.80 to 17.10) | 6.52 (3.79 to 10.74) | 2.93 (1.66 to 4.82) | 0.87 (0.51 to 1.45) | 0.16 (0.10 to 0.26) | 0.02 (0.01 to 0.03) |
| Eritrea | Both | Number of prevalence | 1127.31 (835.28 to 2185.89) | 2713.93 (1657.21 to 5140.46) | 2726.71 (1536.14 to 5808.51) | 2776.15 (1440.33 to 6579.19) | 2762.35 (1325.16 to 7012.79) | 2620.71 (1202.96 to 6934.80) | 2379.09 (1059.43 to 6466.68) | 2083.55 (904.24 to 5786.12) | 1772.27 (755.06 to 5003.74) | 1433.30 (604.86 to 4111.25) | 1103.32 (462.24 to 3218.68) | 841.88 (349.07 to 2499.04) | 614.94 (252.24 to 1860.44) | 421.86 (171.49 to 1289.96) | 240.12 (97.52 to 739.46) | 154.86 (62.87 to 478.21) | 71.76 (29.27 to 222.57) | 22.19 (9.08 to 69.38) | 4.26 (1.75 to 13.41) | 0.62 (0.25 to 1.97) |
| Eritrea | Female | Number of DALYs | 48.57 (27.08 to 77.16) | 76.86 (43.97 to 121.37) | 68.73 (40.23 to 107.82) | 62.63 (36.38 to 97.75) | 56.07 (33.10 to 89.77) | 50.03 (28.03 to 79.57) | 43.73 (24.97 to 67.74) | 37.11 (22.98 to 59.87) | 31.06 (17.47 to 48.20) | 24.73 (14.82 to 37.50) | 19.75 (11.71 to 30.43) | 15.62 (9.22 to 23.64) | 11.74 (6.58 to 17.91) | 8.24 (4.80 to 12.80) | 4.94 (2.83 to 7.64) | 3.22 (1.91 to 5.08) | 1.52 (0.91 to 2.42) | 0.49 (0.28 to 0.75) | 0.10 (0.06 to 0.15) | 0.01 (0.01 to 0.02) |
| Eritrea | Female | Number of prevalence | 617.78 (464.76 to 1137.32) | 1062.93 (735.20 to 2248.09) | 1085.42 (672.95 to 2636.30) | 1121.07 (620.89 to 3035.66) | 1127.03 (574.54 to 3262.20) | 1077.31 (520.20 to 3244.19) | 983.11 (455.78 to 3032.34) | 871.09 (391.61 to 2734.64) | 745.46 (327.57 to 2374.39) | 622.60 (268.34 to 2005.62) | 508.78 (214.57 to 1655.34) | 412.74 (170.72 to 1356.70) | 321.91 (130.70 to 1069.65) | 232.02 (93.39 to 774.54) | 140.03 (56.35 to 467.55) | 92.42 (37.31 to 308.61) | 44.49 (17.95 to 148.54) | 14.63 (5.90 to 48.84) | 2.98 (1.20 to 9.94) | 0.47 (0.19 to 1.56) |
| Eritrea | Male | Number of DALYs | 36.79 (20.00 to 58.19) | 150.13 (69.88 to 322.47) | 137.51 (58.04 to 289.10) | 127.53 (56.57 to 273.19) | 116.37 (48.14 to 246.17) | 105.07 (48.81 to 210.06) | 92.00 (40.34 to 194.98) | 77.86 (34.38 to 168.87) | 64.39 (27.62 to 135.18) | 49.20 (21.27 to 107.15) | 34.48 (15.61 to 70.71) | 24.43 (10.92 to 52.23) | 16.22 (6.91 to 34.60) | 10.45 (4.48 to 21.54) | 5.38 (2.37 to 11.26) | 3.30 (1.50 to 6.76) | 1.41 (0.66 to 2.93) | 0.38 (0.18 to 0.81) | 0.06 (0.03 to 0.13) | 0.01 (0.00 to 0.01) |
| Eritrea | Male | Number of prevalence | 509.53 (356.77 to 1050.20) | 1651.00 (878.86 to 3535.74) | 1641.29 (813.42 to 3533.48) | 1655.07 (769.77 to 3664.12) | 1635.32 (722.66 to 3827.30) | 1543.40 (664.01 to 3769.70) | 1395.98 (586.00 to 3498.25) | 1212.46 (499.20 to 3098.51) | 1026.81 (416.92 to 2662.31) | 810.70 (325.22 to 2131.28) | 594.54 (235.12 to 1580.19) | 429.14 (167.89 to 1153.15) | 293.03 (112.42 to 797.52) | 189.84 (72.61 to 519.68) | 100.09 (38.20 to 273.84) | 62.44 (23.83 to 170.75) | 27.27 (10.39 to 74.56) | 7.57 (2.88 to 20.69) | 1.28 (0.49 to 3.50) | 0.15 (0.06 to 0.41) |
| Ethiopia | Both | Number of DALYs | 1508.12 (1007.88 to 2191.43) | 3987.28 (2263.15 to 7095.42) | 3854.82 (2222.55 to 7072.28) | 3632.69 (2068.94 to 6598.88) | 3132.22 (1743.07 to 5543.44) | 2593.49 (1489.77 to 4648.23) | 2085.63 (1172.79 to 3710.67) | 1732.79 (994.94 to 3111.88) | 1419.57 (798.32 to 2509.88) | 1068.31 (605.93 to 1875.70) | 780.87 (436.90 to 1387.83) | 587.79 (333.51 to 1051.21) | 443.37 (251.94 to 808.86) | 348.88 (199.98 to 630.80) | 235.21 (132.28 to 427.08) | 163.55 (92.68 to 283.37) | 87.72 (49.60 to 153.37) | 36.11 (20.91 to 63.54) | 10.06 (5.93 to 17.50) | 1.86 (1.12 to 3.20) |
| Ethiopia | Both | Number of prevalence | 24484.11 (16861.56 to 43988.75) | 58623.67 (37358.99 to 105184.36) | 66171.55 (38478.82 to 130395.35) | 71830.68 (38952.29 to 148986.71) | 68952.85 (35750.87 to 147541.92) | 60848.80 (30629.78 to 131809.63) | 51360.16 (25344.21 to 112025.85) | 44286.71 (21634.46 to 97843.14) | 37298.62 (18073.74 to 82762.12) | 28460.27 (13758.35 to 63128.68) | 21072.76 (10131.55 to 46961.18) | 16210.73 (7745.00 to 36962.08) | 12688.92 (5985.19 to 29005.74) | 10162.85 (4760.04 to 23303.15) | 6887.10 (3232.84 to 15814.29) | 4887.21 (2289.17 to 11265.61) | 2680.98 (1250.78 to 6244.66) | 1124.58 (523.59 to 2648.51) | 323.62 (150.29 to 763.98) | 61.88 (28.66 to 149.96) |
| Ethiopia | Female | Number of DALYs | 865.25 (579.74 to 1293.71) | 1337.51 (870.65 to 1938.98) | 1277.35 (816.41 to 1879.08) | 1190.66 (768.31 to 1714.17) | 1034.51 (698.31 to 1473.81) | 869.85 (563.58 to 1261.79) | 702.79 (462.88 to 1002.05) | 594.26 (390.63 to 861.62) | 470.17 (308.49 to 671.54) | 336.68 (226.08 to 480.36) | 241.61 (160.35 to 348.74) | 183.52 (122.79 to 258.34) | 140.38 (92.98 to 197.81) | 112.35 (73.41 to 156.00) | 70.20 (46.73 to 98.51) | 49.11 (33.20 to 69.80) | 27.27 (18.30 to 38.67) | 11.52 (7.51 to 16.24) | 3.32 (2.28 to 4.65) | 0.70 (0.48 to 0.99) |
| Ethiopia | Female | Number of prevalence | 13204.84 (9397.76 to 22820.07) | 24086.83 (15455.03 to 46212.26) | 27729.68 (15971.83 to 58624.18) | 30905.88 (16263.77 to 70303.66) | 30530.68 (15023.31 to 71645.98) | 27596.57 (13045.54 to 65153.52) | 23687.61 (10941.09 to 56403.67) | 20658.76 (9358.88 to 49632.62) | 17053.40 (7623.63 to 41131.51) | 12489.94 (5532.72 to 30163.46) | 9180.29 (4025.13 to 22250.62) | 7134.57 (3087.51 to 17366.80) | 5669.42 (2413.29 to 14105.29) | 4570.33 (1931.44 to 11378.63) | 2915.00 (1231.87 to 7225.43) | 2082.11 (880.32 to 5154.75) | 1159.84 (491.58 to 2864.34) | 496.91 (210.97 to 1223.84) | 146.77 (62.48 to 363.26) | 30.89 (13.31 to 78.93) |
| Ethiopia | Male | Number of DALYs | 642.87 (413.98 to 986.67) | 2649.77 (1224.79 to 5527.99) | 2577.47 (1227.81 to 5364.35) | 2442.02 (1181.17 to 4968.84) | 2097.71 (964.50 to 4188.24) | 1723.64 (810.48 to 3521.92) | 1382.84 (640.82 to 2815.71) | 1138.53 (537.92 to 2363.34) | 949.41 (438.38 to 1928.74) | 731.63 (350.09 to 1440.50) | 539.26 (247.39 to 1056.09) | 404.26 (192.45 to 836.36) | 303.00 (146.07 to 619.56) | 236.53 (113.32 to 483.54) | 165.01 (78.87 to 337.16) | 114.44 (54.76 to 224.45) | 60.46 (29.11 to 122.98) | 24.59 (12.03 to 49.63) | 6.74 (3.29 to 13.40) | 1.17 (0.56 to 2.40) |
| Ethiopia | Male | Number of prevalence | 11279.27 (7230.88 to 21219.38) | 34536.85 (20376.41 to 62198.16) | 38441.87 (21687.76 to 73316.47) | 40924.80 (21962.42 to 81780.71) | 38422.17 (19795.13 to 79508.13) | 33252.23 (16712.08 to 69649.67) | 27672.55 (13654.31 to 58194.39) | 23627.96 (11456.61 to 49982.14) | 20245.22 (9706.89 to 43109.23) | 15970.33 (7614.63 to 34272.86) | 11892.47 (5680.49 to 25758.00) | 9076.15 (4335.11 to 19784.32) | 7019.50 (3329.71 to 15292.12) | 5592.53 (2647.13 to 12235.13) | 3972.10 (1877.08 to 8736.92) | 2805.10 (1321.19 to 6216.33) | 1521.13 (715.66 to 3418.40) | 627.67 (295.63 to 1416.43) | 176.85 (82.99 to 401.77) | 30.99 (14.53 to 71.03) |
| Fiji | Both | Number of DALYs | 10.44 (6.52 to 16.19) | 60.18 (31.97 to 106.60) | 84.60 (48.41 to 142.33) | 112.45 (67.89 to 177.80) | 141.41 (88.88 to 208.47) | 163.44 (107.06 to 245.79) | 175.98 (113.79 to 258.19) | 190.30 (123.35 to 276.89) | 181.56 (119.08 to 259.80) | 159.52 (107.85 to 230.79) | 151.58 (100.93 to 214.96) | 145.65 (98.29 to 204.51) | 117.02 (78.92 to 162.84) | 76.33 (51.73 to 105.11) | 46.90 (31.53 to 64.58) | 26.16 (17.86 to 36.14) | 11.75 (8.23 to 15.99) | 3.39 (2.34 to 4.58) | 0.41 (0.29 to 0.56) | 0.04 (0.03 to 0.05) |
| Fiji | Both | Number of prevalence | 1262.01 (239.01 to 3707.79) | 3412.57 (854.26 to 9645.61) | 4703.58 (1183.88 to 13282.44) | 5745.34 (1516.08 to 16059.78) | 6689.54 (1851.51 to 18491.17) | 7426.80 (2092.88 to 20392.70) | 7744.23 (2223.93 to 21156.40) | 8226.81 (2382.63 to 22387.75) | 7789.29 (2271.76 to 21144.38) | 6823.90 (2014.40 to 18499.23) | 6594.49 (1941.81 to 17874.24) | 6464.97 (1905.89 to 17524.40) | 5332.71 (1563.46 to 14472.05) | 3676.31 (1056.18 to 10016.13) | 2382.93 (670.07 to 6519.77) | 1425.42 (390.62 to 3924.20) | 696.67 (182.87 to 1933.58) | 236.05 (57.60 to 665.00) | 46.66 (9.45 to 136.05) | 6.31 (1.13 to 18.72) |
| Fiji | Female | Number of DALYs | 5.92 (3.51 to 9.20) | 10.97 (6.71 to 17.25) | 11.79 (7.21 to 17.62) | 11.60 (7.30 to 17.44) | 12.13 (7.71 to 17.94) | 12.85 (8.09 to 19.20) | 12.78 (8.04 to 18.99) | 13.26 (8.62 to 19.01) | 12.11 (7.46 to 17.80) | 10.49 (6.85 to 15.82) | 10.04 (6.34 to 14.78) | 9.91 (6.24 to 14.51) | 8.27 (5.20 to 12.17) | 5.88 (3.70 to 8.38) | 3.96 (2.48 to 5.74) | 2.47 (1.60 to 3.54) | 1.30 (0.85 to 1.84) | 0.49 (0.33 to 0.71) | 0.12 (0.08 to 0.18) | 0.02 (0.01 to 0.03) |
| Fiji | Female | Number of prevalence | 619.46 (122.82 to 1803.44) | 1516.97 (270.22 to 4489.31) | 2068.77 (341.52 to 6188.15) | 2461.58 (389.11 to 7405.85) | 2808.82 (435.62 to 8473.32) | 3108.60 (477.87 to 9389.05) | 3186.92 (488.31 to 9631.26) | 3349.47 (511.77 to 10125.27) | 3150.62 (480.63 to 9526.45) | 2742.35 (418.00 to 8292.99) | 2698.43 (411.32 to 8160.71) | 2680.54 (408.37 to 8107.31) | 2265.93 (345.24 to 6853.70) | 1643.24 (250.69 to 4970.31) | 1115.07 (170.15 to 3372.62) | 711.02 (108.45 to 2150.56) | 376.66 (57.43 to 1139.25) | 145.78 (22.23 to 440.94) | 37.31 (5.69 to 112.83) | 5.66 (0.86 to 17.12) |
| Fiji | Male | Number of DALYs | 4.52 (2.47 to 7.37) | 49.21 (24.10 to 94.16) | 72.81 (39.38 to 127.43) | 100.85 (59.36 to 162.18) | 129.27 (80.38 to 193.79) | 150.59 (96.52 to 228.58) | 163.20 (104.09 to 240.52) | 177.04 (112.58 to 260.17) | 169.46 (109.74 to 244.21) | 149.03 (99.68 to 216.51) | 141.54 (93.36 to 201.74) | 135.73 (91.01 to 191.79) | 108.75 (72.99 to 151.96) | 70.45 (47.11 to 97.22) | 42.95 (28.78 to 59.64) | 23.69 (15.99 to 32.83) | 10.45 (7.28 to 14.34) | 2.90 (1.99 to 3.95) | 0.29 (0.21 to 0.40) | 0.02 (0.01 to 0.03) |
| Fiji | Male | Number of prevalence | 642.55 (116.35 to 1904.34) | 1895.60 (540.83 to 5096.13) | 2634.81 (805.80 to 7020.82) | 3283.76 (1076.77 to 8589.93) | 3880.72 (1365.73 to 9978.35) | 4318.20 (1581.27 to 10983.04) | 4557.31 (1700.80 to 11520.14) | 4877.34 (1844.18 to 12262.80) | 4638.66 (1763.43 to 11617.93) | 4081.55 (1571.42 to 10206.24) | 3896.05 (1517.12 to 9713.53) | 3784.43 (1475.23 to 9417.09) | 3066.78 (1195.81 to 7618.36) | 2033.06 (793.50 to 5045.82) | 1267.86 (494.81 to 3147.16) | 714.40 (279.55 to 1773.65) | 320.00 (125.46 to 794.33) | 90.27 (35.30 to 224.06) | 9.35 (3.66 to 23.22) | 0.64 (0.25 to 1.60) |
| Gabon | Both | Number of DALYs | 21.15 (12.31 to 33.36) | 63.77 (35.17 to 114.50) | 62.96 (32.29 to 116.49) | 60.17 (32.43 to 111.50) | 56.57 (31.19 to 100.47) | 52.07 (28.12 to 90.41) | 46.78 (25.50 to 85.03) | 41.04 (21.14 to 72.51) | 35.96 (19.04 to 63.00) | 31.08 (16.48 to 54.09) | 27.12 (14.67 to 48.56) | 23.00 (12.37 to 41.84) | 17.99 (9.81 to 31.78) | 13.47 (7.28 to 23.51) | 6.84 (3.77 to 11.99) | 4.09 (2.28 to 6.88) | 2.11 (1.22 to 3.66) | 0.84 (0.50 to 1.37) | 0.21 (0.13 to 0.34) | 0.04 (0.02 to 0.06) |
| Gabon | Both | Number of prevalence | 598.90 (232.92 to 2082.29) | 1556.11 (570.19 to 5352.51) | 1924.20 (593.38 to 7145.69) | 2211.99 (592.45 to 8629.75) | 2349.59 (575.18 to 9411.85) | 2328.41 (539.59 to 9452.92) | 2167.19 (486.10 to 8862.10) | 1961.32 (430.57 to 8054.49) | 1756.37 (380.08 to 7238.63) | 1562.87 (333.03 to 6459.78) | 1390.68 (292.92 to 5762.24) | 1199.06 (250.70 to 4976.27) | 953.45 (197.34 to 3964.45) | 735.02 (151.26 to 3060.10) | 389.00 (78.98 to 1625.42) | 250.84 (49.79 to 1055.00) | 140.62 (27.31 to 595.31) | 59.35 (11.43 to 252.31) | 15.77 (2.99 to 67.33) | 3.33 (0.62 to 14.27) |
| Gabon | Female | Number of DALYs | 12.31 (7.07 to 19.82) | 21.34 (12.07 to 32.65) | 21.03 (12.30 to 32.94) | 19.62 (11.80 to 30.94) | 18.11 (10.51 to 28.15) | 15.95 (9.63 to 23.93) | 13.66 (7.95 to 21.21) | 11.53 (7.04 to 17.46) | 9.86 (6.09 to 14.90) | 8.33 (4.93 to 12.37) | 7.16 (4.34 to 11.09) | 5.77 (3.34 to 8.70) | 4.44 (2.71 to 6.67) | 3.31 (2.04 to 5.01) | 1.85 (1.14 to 2.77) | 1.35 (0.82 to 2.09) | 0.81 (0.49 to 1.24) | 0.36 (0.22 to 0.55) | 0.10 (0.06 to 0.15) | 0.02 (0.01 to 0.03) |
| Gabon | Female | Number of prevalence | 316.20 (132.58 to 1057.84) | 707.53 (243.76 to 2591.90) | 911.45 (256.24 to 3574.87) | 1080.24 (255.96 to 4425.51) | 1166.97 (249.08 to 4895.04) | 1157.11 (230.54 to 4916.08) | 1061.13 (203.30 to 4542.25) | 941.78 (174.57 to 4053.32) | 834.12 (151.05 to 3604.03) | 739.46 (131.10 to 3205.56) | 652.01 (113.71 to 2834.25) | 551.15 (94.21 to 2402.59) | 431.19 (72.37 to 1885.14) | 333.21 (55.43 to 1458.59) | 188.75 (31.47 to 826.37) | 137.92 (22.98 to 603.79) | 86.58 (14.43 to 379.02) | 39.12 (6.53 to 171.28) | 11.10 (1.85 to 48.60) | 2.51 (0.42 to 11.00) |
| Gabon | Male | Number of DALYs | 8.84 (4.72 to 14.18) | 42.42 (19.58 to 86.47) | 41.93 (18.27 to 86.61) | 40.55 (18.39 to 84.68) | 38.46 (17.05 to 77.23) | 36.12 (16.57 to 72.18) | 33.12 (14.58 to 66.11) | 29.51 (12.75 to 58.71) | 26.09 (11.39 to 50.79) | 22.76 (10.04 to 44.42) | 19.96 (9.02 to 39.33) | 17.23 (7.82 to 34.17) | 13.55 (6.31 to 26.79) | 10.16 (4.75 to 19.79) | 4.98 (2.28 to 9.63) | 2.74 (1.30 to 5.25) | 1.30 (0.61 to 2.50) | 0.47 (0.23 to 0.90) | 0.11 (0.05 to 0.21) | 0.02 (0.01 to 0.04) |
| Gabon | Male | Number of prevalence | 282.69 (98.07 to 1024.45) | 848.59 (316.01 to 2719.28) | 1012.75 (323.62 to 3540.46) | 1131.76 (323.15 to 4165.41) | 1182.62 (319.85 to 4473.74) | 1171.29 (307.03 to 4492.62) | 1106.06 (284.29 to 4278.90) | 1019.54 (258.01 to 3965.14) | 922.25 (231.13 to 3602.22) | 823.42 (204.69 to 3225.36) | 738.67 (182.21 to 2902.48) | 647.91 (159.11 to 2551.98) | 522.26 (127.34 to 2062.23) | 401.81 (97.18 to 1588.28) | 200.25 (48.62 to 791.62) | 112.92 (27.40 to 446.42) | 54.04 (13.12 to 213.68) | 20.23 (4.91 to 79.98) | 4.67 (1.13 to 18.45) | 0.81 (0.20 to 3.22) |
| Ghana | Both | Number of DALYs | 443.48 (263.03 to 667.78) | 1214.41 (614.90 to 2289.74) | 1117.14 (597.79 to 2029.15) | 1004.69 (529.54 to 1772.95) | 941.39 (516.98 to 1769.67) | 867.14 (473.74 to 1516.24) | 759.44 (396.16 to 1407.29) | 655.51 (355.79 to 1149.77) | 542.93 (301.69 to 926.68) | 431.21 (234.26 to 763.95) | 332.92 (182.23 to 608.29) | 251.36 (145.04 to 434.29) | 199.32 (110.97 to 354.47) | 150.05 (80.99 to 262.91) | 93.36 (53.43 to 164.58) | 54.61 (31.46 to 92.29) | 27.46 (15.60 to 47.48) | 10.86 (6.61 to 17.90) | 2.73 (1.61 to 4.51) | 0.56 (0.33 to 0.87) |
| Ghana | Both | Number of prevalence | 8484.53 (5034.39 to 18549.08) | 20628.93 (11025.33 to 45272.20) | 22526.41 (10853.92 to 53239.53) | 24113.55 (10450.90 to 61049.08) | 25664.22 (10392.66 to 67744.32) | 25704.29 (10060.10 to 69337.77) | 23928.34 (9164.12 to 65379.72) | 21384.17 (8052.15 to 58906.82) | 18236.47 (6782.14 to 50547.17) | 14964.40 (5504.04 to 41712.61) | 11917.44 (4338.42 to 33430.97) | 9386.70 (3378.65 to 26488.33) | 7708.77 (2741.12 to 21862.13) | 5939.34 (2099.76 to 16888.22) | 3795.64 (1340.93 to 10804.65) | 2291.08 (807.36 to 6537.55) | 1194.21 (420.11 to 3416.42) | 484.34 (170.01 to 1388.72) | 127.44 (43.91 to 366.88) | 28.06 (9.54 to 81.29) |
| Ghana | Female | Number of DALYs | 256.13 (144.91 to 408.39) | 402.83 (236.47 to 637.98) | 364.70 (212.86 to 567.08) | 326.78 (184.72 to 505.23) | 309.17 (185.65 to 494.84) | 297.37 (170.12 to 461.30) | 256.85 (149.88 to 397.82) | 220.81 (132.40 to 342.18) | 178.85 (106.65 to 266.52) | 141.62 (81.57 to 215.97) | 113.31 (68.34 to 174.33) | 87.95 (52.86 to 132.94) | 70.32 (40.47 to 106.73) | 53.11 (31.11 to 81.08) | 34.20 (20.52 to 52.86) | 21.10 (12.97 to 31.48) | 11.41 (6.91 to 16.82) | 4.78 (3.04 to 7.23) | 1.31 (0.78 to 1.97) | 0.30 (0.18 to 0.46) |
| Ghana | Female | Number of prevalence | 4520.01 (2830.51 to 9412.05) | 8685.35 (4754.23 to 20170.89) | 9686.25 (4553.01 to 24663.02) | 10671.37 (4411.57 to 28943.53) | 11935.78 (4562.72 to 33557.81) | 12418.55 (4496.30 to 35617.04) | 11627.99 (4086.39 to 33748.46) | 10385.37 (3559.93 to 30400.76) | 8789.85 (2960.93 to 25898.20) | 7210.41 (2388.69 to 21355.65) | 5927.35 (1937.33 to 17639.97) | 4786.80 (1541.68 to 14311.39) | 3942.55 (1249.73 to 11842.09) | 3065.64 (966.35 to 9228.27) | 2001.76 (630.15 to 6025.57) | 1263.37 (397.45 to 3802.88) | 691.62 (217.70 to 2081.93) | 291.66 (91.93 to 877.92) | 82.03 (25.83 to 246.92) | 19.79 (6.23 to 59.58) |
| Ghana | Male | Number of DALYs | 187.35 (101.87 to 308.22) | 811.58 (336.17 to 1739.13) | 752.44 (313.18 to 1628.92) | 677.90 (307.19 to 1406.72) | 632.22 (281.91 to 1406.66) | 569.77 (258.30 to 1141.11) | 502.59 (217.12 to 1134.75) | 434.70 (179.22 to 888.65) | 364.07 (161.37 to 737.75) | 289.60 (130.95 to 593.74) | 219.61 (96.82 to 453.78) | 163.42 (76.56 to 326.81) | 129.00 (58.19 to 259.41) | 96.94 (42.56 to 191.35) | 59.17 (26.67 to 121.17) | 33.51 (15.06 to 66.05) | 16.04 (7.40 to 34.56) | 6.08 (2.84 to 12.29) | 1.42 (0.63 to 2.82) | 0.25 (0.12 to 0.51) |
| Ghana | Male | Number of prevalence | 3964.52 (2186.87 to 9139.14) | 11943.58 (5999.97 to 24539.95) | 12840.17 (5943.43 to 29460.45) | 13442.18 (5738.90 to 32907.89) | 13728.44 (5604.72 to 34575.31) | 13285.74 (5295.90 to 33984.54) | 12300.34 (4844.59 to 31778.63) | 10998.80 (4283.92 to 28620.98) | 9446.63 (3635.78 to 24719.07) | 7753.99 (2957.65 to 20376.62) | 5990.09 (2269.58 to 15830.68) | 4599.90 (1726.67 to 12229.60) | 3766.21 (1401.47 to 10076.21) | 2873.71 (1064.52 to 7709.97) | 1793.88 (663.66 to 4813.46) | 1027.71 (380.82 to 2757.21) | 502.59 (186.03 to 1348.24) | 192.68 (71.28 to 516.92) | 45.41 (16.79 to 121.83) | 8.26 (3.06 to 22.17) |
| Guinea | Both | Number of DALYs | 211.50 (133.68 to 310.30) | 569.39 (290.89 to 1054.14) | 491.74 (260.36 to 899.39) | 403.13 (227.03 to 721.42) | 333.08 (179.33 to 592.78) | 289.15 (148.30 to 540.45) | 250.99 (137.97 to 447.03) | 200.10 (113.94 to 345.35) | 156.86 (84.71 to 273.17) | 124.90 (69.58 to 226.20) | 101.54 (55.13 to 179.42) | 81.43 (43.35 to 148.47) | 67.24 (36.29 to 118.86) | 50.49 (27.66 to 97.40) | 35.14 (18.97 to 64.27) | 21.21 (11.11 to 37.17) | 11.62 (6.41 to 21.71) | 5.25 (3.00 to 8.91) | 1.71 (1.00 to 2.97) | 0.40 (0.24 to 0.68) |
| Guinea | Both | Number of prevalence | 3839.64 (2326.18 to 8494.31) | 9185.51 (5044.20 to 19954.30) | 9469.72 (4682.79 to 22664.25) | 9222.30 (4159.50 to 23793.39) | 8637.33 (3632.19 to 23332.22) | 8112.50 (3229.30 to 22502.22) | 7501.67 (2886.11 to 21104.17) | 6223.00 (2334.46 to 17685.44) | 5008.09 (1851.32 to 14328.48) | 4072.24 (1486.72 to 11705.29) | 3358.79 (1217.58 to 9685.17) | 2755.37 (988.85 to 7974.66) | 2315.26 (822.25 to 6729.04) | 1785.48 (631.86 to 5194.50) | 1267.85 (448.09 to 3690.88) | 775.49 (274.10 to 2259.56) | 442.40 (155.77 to 1291.41) | 208.03 (72.96 to 609.43) | 70.40 (24.52 to 207.27) | 17.37 (6.01 to 51.45) |
| Guinea | Female | Number of DALYs | 122.18 (71.99 to 181.97) | 192.13 (108.19 to 300.07) | 165.54 (93.57 to 263.85) | 137.89 (77.32 to 212.72) | 119.80 (68.41 to 179.16) | 105.70 (58.23 to 168.04) | 93.33 (54.26 to 149.06) | 73.74 (43.49 to 112.23) | 56.92 (33.96 to 89.39) | 42.72 (25.91 to 66.39) | 32.74 (19.24 to 49.16) | 24.53 (14.54 to 37.01) | 19.24 (12.06 to 30.52) | 13.87 (8.38 to 22.16) | 10.13 (6.07 to 15.58) | 6.21 (3.94 to 9.48) | 3.62 (2.13 to 5.58) | 1.81 (1.07 to 2.73) | 0.65 (0.40 to 0.97) | 0.17 (0.10 to 0.26) |
| Guinea | Female | Number of prevalence | 2057.99 (1306.85 to 4355.46) | 3855.22 (2126.69 to 9140.74) | 4104.03 (1967.51 to 10696.18) | 4206.60 (1780.57 to 11712.88) | 4190.20 (1627.47 to 12128.23) | 4087.30 (1507.17 to 12083.41) | 3827.22 (1368.25 to 11461.30) | 3198.51 (1112.62 to 9663.60) | 2533.19 (864.05 to 7707.25) | 1995.96 (671.55 to 6109.37) | 1569.74 (518.72 to 4829.06) | 1239.60 (404.36 to 3831.91) | 1005.66 (321.88 to 3124.75) | 750.82 (238.33 to 2338.38) | 545.14 (172.91 to 1697.47) | 337.27 (107.31 to 1050.53) | 203.30 (64.58 to 633.24) | 103.46 (32.99 to 322.22) | 37.84 (12.05 to 117.84) | 10.17 (3.23 to 31.69) |
| Guinea | Male | Number of DALYs | 89.32 (48.83 to 147.83) | 377.26 (170.29 to 818.32) | 326.21 (140.98 to 697.90) | 265.24 (111.67 to 542.91) | 213.28 (93.03 to 449.28) | 183.45 (78.94 to 393.78) | 157.66 (69.76 to 328.41) | 126.37 (59.04 to 264.60) | 99.93 (43.66 to 201.65) | 82.18 (38.40 to 172.38) | 68.80 (32.39 to 138.95) | 56.90 (25.16 to 118.38) | 47.99 (22.59 to 99.98) | 36.62 (16.23 to 79.23) | 25.01 (11.47 to 49.78) | 14.99 (6.72 to 29.74) | 8.00 (3.76 to 17.10) | 3.44 (1.55 to 6.91) | 1.06 (0.51 to 2.13) | 0.23 (0.11 to 0.47) |
| Guinea | Male | Number of prevalence | 1781.64 (1018.02 to 4131.34) | 5330.29 (2714.21 to 11370.35) | 5365.69 (2564.95 to 12153.66) | 5015.70 (2234.74 to 12229.29) | 4447.13 (1868.35 to 11335.32) | 4025.20 (1641.30 to 10525.53) | 3674.46 (1466.82 to 9744.03) | 3024.50 (1194.67 to 8101.19) | 2474.89 (967.46 to 6679.68) | 2076.28 (803.94 to 5643.15) | 1789.05 (686.37 to 4894.34) | 1515.77 (577.69 to 4171.44) | 1309.61 (494.69 to 3624.97) | 1034.66 (389.63 to 2870.12) | 722.70 (271.92 to 2004.55) | 438.22 (164.81 to 1215.52) | 239.10 (90.07 to 662.95) | 104.56 (39.34 to 289.95) | 32.56 (12.25 to 90.33) | 7.20 (2.71 to 19.97) |
| Guinea-Bissau | Both | Number of DALYs | 31.60 (19.46 to 48.32) | 88.06 (48.27 to 164.76) | 79.75 (42.22 to 146.89) | 70.42 (37.21 to 131.10) | 61.83 (33.81 to 109.59) | 56.98 (29.95 to 102.66) | 49.93 (26.74 to 86.16) | 40.58 (21.97 to 72.17) | 31.26 (16.90 to 54.91) | 22.94 (12.68 to 41.24) | 18.03 (10.01 to 31.64) | 13.98 (7.52 to 24.80) | 10.50 (5.76 to 18.00) | 7.49 (4.16 to 13.21) | 4.49 (2.46 to 7.83) | 2.50 (1.41 to 4.22) | 1.05 (0.58 to 1.77) | 0.33 (0.19 to 0.55) | 0.07 (0.04 to 0.12) | 0.01 (0.01 to 0.02) |
| Guinea-Bissau | Both | Number of prevalence | 822.54 (327.71 to 3011.34) | 1956.05 (735.04 to 7015.58) | 2195.66 (690.61 to 8558.68) | 2311.00 (624.57 to 9520.07) | 2321.09 (573.37 to 9858.97) | 2299.36 (532.37 to 9938.84) | 2146.77 (477.64 to 9378.41) | 1829.29 (396.15 to 8048.38) | 1442.62 (306.77 to 6376.44) | 1098.34 (229.88 to 4871.89) | 869.84 (179.36 to 3869.62) | 690.00 (140.52 to 3077.70) | 542.77 (108.87 to 2429.47) | 392.20 (77.86 to 1758.85) | 242.30 (47.69 to 1089.72) | 140.43 (27.35 to 633.95) | 62.18 (11.99 to 281.81) | 20.23 (3.87 to 91.93) | 4.60 (0.87 to 20.99) | 0.84 (0.16 to 3.86) |
| Guinea-Bissau | Female | Number of DALYs | 18.28 (10.78 to 27.67) | 28.75 (16.89 to 43.60) | 24.91 (14.52 to 39.99) | 21.65 (12.60 to 33.82) | 18.90 (11.28 to 29.81) | 17.56 (10.61 to 27.06) | 15.74 (9.46 to 24.67) | 12.83 (7.30 to 19.80) | 9.66 (5.98 to 15.08) | 6.87 (4.29 to 10.16) | 5.32 (3.02 to 8.25) | 4.06 (2.26 to 6.45) | 2.97 (1.80 to 4.54) | 2.12 (1.29 to 3.16) | 1.37 (0.80 to 2.07) | 0.84 (0.51 to 1.24) | 0.38 (0.22 to 0.59) | 0.13 (0.08 to 0.20) | 0.03 (0.02 to 0.04) | 0.01 (0.00 to 0.01) |
| Guinea-Bissau | Female | Number of prevalence | 430.31 (186.20 to 1511.42) | 852.94 (299.10 to 3311.53) | 979.86 (275.04 to 4109.29) | 1058.85 (246.73 to 4668.54) | 1092.54 (222.64 to 4950.38) | 1116.17 (210.84 to 5132.11) | 1071.72 (191.08 to 4971.03) | 926.85 (158.90 to 4325.62) | 727.08 (121.13 to 3408.88) | 547.37 (88.76 to 2576.57) | 429.66 (68.07 to 2028.96) | 337.10 (52.27 to 1597.07) | 264.12 (39.96 to 1255.49) | 192.21 (28.77 to 915.03) | 124.33 (18.60 to 591.94) | 76.50 (11.45 to 364.18) | 35.88 (5.36 to 170.82) | 12.16 (1.82 to 57.87) | 2.87 (0.43 to 13.69) | 0.57 (0.08 to 2.69) |
| Guinea-Bissau | Male | Number of DALYs | 13.32 (7.17 to 21.96) | 59.31 (26.52 to 126.33) | 54.84 (23.96 to 113.87) | 48.76 (21.67 to 104.63) | 42.93 (19.16 to 87.03) | 39.43 (17.36 to 81.87) | 34.19 (14.94 to 68.03) | 27.75 (13.00 to 55.22) | 21.60 (9.49 to 41.86) | 16.07 (7.61 to 32.19) | 12.71 (5.67 to 24.87) | 9.92 (4.38 to 19.65) | 7.52 (3.62 to 14.77) | 5.36 (2.41 to 10.72) | 3.12 (1.43 to 6.23) | 1.66 (0.77 to 3.31) | 0.67 (0.31 to 1.30) | 0.20 (0.09 to 0.40) | 0.04 (0.02 to 0.08) | 0.01 (0.00 to 0.01) |
| Guinea-Bissau | Male | Number of prevalence | 392.23 (139.13 to 1499.92) | 1103.11 (395.91 to 3738.61) | 1215.79 (389.52 to 4489.44) | 1252.15 (361.11 to 4894.42) | 1228.55 (330.49 to 4953.25) | 1183.19 (304.09 to 4852.22) | 1075.05 (268.87 to 4451.10) | 902.44 (220.17 to 3760.52) | 715.53 (172.40 to 2995.60) | 550.97 (131.12 to 2316.18) | 440.18 (103.55 to 1856.78) | 352.90 (82.21 to 1492.84) | 278.65 (64.15 to 1183.10) | 199.99 (45.91 to 850.26) | 117.97 (27.09 to 501.56) | 63.93 (14.69 to 271.84) | 26.30 (6.03 to 111.82) | 8.07 (1.86 to 34.32) | 1.73 (0.40 to 7.36) | 0.28 (0.06 to 1.18) |
| Guyana | Both | Number of DALYs | 9.60 (6.18 to 14.09) | 84.87 (48.82 to 142.27) | 137.71 (87.23 to 205.87) | 209.20 (138.07 to 299.96) | 285.67 (188.54 to 401.64) | 290.87 (196.77 to 401.51) | 225.90 (152.21 to 316.42) | 197.62 (133.25 to 273.52) | 202.62 (137.85 to 273.03) | 189.49 (130.37 to 257.52) | 174.17 (119.68 to 238.22) | 150.82 (104.46 to 202.73) | 122.53 (84.31 to 166.59) | 88.61 (61.61 to 119.86) | 54.50 (38.55 to 73.88) | 31.22 (21.88 to 41.76) | 16.76 (11.82 to 22.15) | 7.75 (5.45 to 10.18) | 2.79 (1.99 to 3.69) | 0.84 (0.59 to 1.14) |
| Guyana | Both | Number of prevalence | 1721.70 (478.25 to 3780.14) | 4533.88 (1581.57 to 9392.45) | 6420.38 (2352.33 to 13122.42) | 8904.43 (3405.58 to 17985.72) | 12039.63 (4582.02 to 24249.57) | 12797.07 (4784.81 to 25865.14) | 10400.03 (3861.24 to 21105.16) | 9477.06 (3489.94 to 19300.64) | 10027.81 (3677.59 to 20461.83) | 9582.43 (3501.41 to 19574.91) | 9027.36 (3289.14 to 18466.15) | 8237.92 (2968.25 to 16916.10) | 6973.09 (2490.83 to 14359.57) | 5272.12 (1869.25 to 10886.46) | 3307.68 (1170.69 to 6832.39) | 1984.93 (695.08 to 4111.93) | 1128.94 (389.75 to 2347.73) | 543.19 (186.41 to 1131.57) | 194.34 (67.16 to 404.03) | 51.03 (18.54 to 104.59) |
| Guyana | Female | Number of DALYs | 5.54 (3.43 to 8.17) | 10.12 (6.28 to 15.01) | 11.64 (7.03 to 17.42) | 14.52 (9.21 to 21.78) | 19.47 (12.49 to 28.12) | 21.48 (13.94 to 31.57) | 17.98 (11.72 to 26.51) | 17.00 (11.17 to 24.54) | 17.93 (11.58 to 25.84) | 17.19 (11.05 to 23.85) | 16.15 (10.52 to 23.11) | 15.31 (10.12 to 21.90) | 13.45 (8.88 to 19.89) | 10.46 (6.90 to 14.59) | 6.60 (4.32 to 9.26) | 4.10 (2.76 to 5.78) | 2.45 (1.67 to 3.51) | 1.19 (0.82 to 1.66) | 0.40 (0.28 to 0.57) | 0.08 (0.06 to 0.12) |
| Guyana | Female | Number of prevalence | 849.21 (239.52 to 1853.77) | 1980.81 (539.60 to 4358.56) | 2714.20 (724.10 to 5998.89) | 3689.88 (974.91 to 8169.98) | 4986.97 (1315.57 to 11042.68) | 5472.95 (1445.45 to 12113.62) | 4558.84 (1205.77 to 10085.44) | 4224.91 (1119.35 to 9340.83) | 4454.90 (1182.35 to 9845.49) | 4217.41 (1121.13 to 9315.91) | 3939.45 (1049.18 to 8697.64) | 3714.68 (991.51 to 8196.50) | 3196.66 (855.54 to 7048.64) | 2492.01 (667.43 to 5493.09) | 1577.50 (422.65 to 3477.18) | 992.96 (265.99 to 2188.74) | 597.87 (160.07 to 1317.81) | 295.43 (79.11 to 651.15) | 102.57 (27.47 to 226.08) | 21.23 (5.69 to 46.80) |
| Guyana | Male | Number of DALYs | 4.07 (2.40 to 6.51) | 74.75 (40.98 to 129.30) | 126.07 (78.39 to 192.90) | 194.69 (128.06 to 278.35) | 266.20 (175.10 to 376.20) | 269.38 (180.87 to 372.44) | 207.92 (137.44 to 294.89) | 180.62 (121.37 to 251.43) | 184.70 (124.68 to 252.95) | 172.31 (117.81 to 234.25) | 158.02 (107.68 to 217.81) | 135.51 (93.19 to 182.97) | 109.08 (74.54 to 147.51) | 78.15 (54.17 to 106.22) | 47.89 (33.44 to 64.85) | 27.12 (18.99 to 36.38) | 14.31 (9.98 to 19.19) | 6.57 (4.59 to 8.61) | 2.39 (1.68 to 3.21) | 0.76 (0.54 to 1.04) |
| Guyana | Male | Number of prevalence | 872.49 (237.61 to 1926.37) | 2553.07 (1055.33 to 5098.38) | 3706.19 (1662.01 to 7176.58) | 5214.55 (2439.82 to 9858.73) | 7052.66 (3322.82 to 13231.85) | 7324.12 (3415.18 to 13754.20) | 5841.19 (2681.44 to 11021.87) | 5252.15 (2372.34 to 9959.97) | 5572.91 (2473.96 to 10616.34) | 5365.02 (2359.93 to 10259.00) | 5087.90 (2228.83 to 9768.52) | 4523.24 (1965.89 to 8719.60) | 3776.43 (1626.09 to 7310.93) | 2780.11 (1194.35 to 5393.37) | 1730.18 (743.59 to 3355.21) | 991.97 (426.17 to 1923.18) | 531.07 (227.89 to 1029.92) | 247.76 (106.42 to 480.42) | 91.77 (39.40 to 177.95) | 29.80 (12.80 to 57.79) |
| Haiti | Both | Number of DALYs | 150.20 (91.11 to 224.30) | 411.87 (213.63 to 740.89) | 390.88 (212.56 to 712.15) | 383.69 (209.02 to 699.82) | 368.06 (197.89 to 686.78) | 351.89 (183.33 to 630.36) | 346.93 (188.09 to 641.10) | 309.28 (160.47 to 563.27) | 252.58 (131.47 to 439.52) | 197.61 (107.15 to 358.78) | 157.67 (85.34 to 281.08) | 127.81 (71.62 to 222.38) | 99.31 (56.82 to 169.96) | 73.46 (40.47 to 128.45) | 45.73 (25.07 to 81.21) | 26.14 (14.10 to 46.41) | 13.36 (7.35 to 23.59) | 5.59 (3.16 to 9.75) | 1.54 (0.81 to 2.69) | 0.29 (0.16 to 0.51) |
| Haiti | Both | Number of prevalence | 3514.65 (2235.02 to 6522.64) | 8474.37 (5125.71 to 15752.81) | 9990.29 (5723.33 to 19741.29) | 11659.09 (6339.16 to 23915.37) | 12443.87 (6565.07 to 26068.43) | 13045.58 (6739.84 to 27660.54) | 13507.43 (6892.91 to 28840.86) | 12631.49 (6398.75 to 27101.76) | 10738.98 (5416.69 to 23124.82) | 8643.61 (4342.96 to 18659.04) | 7034.92 (3521.96 to 15215.57) | 5883.36 (2936.27 to 12756.96) | 4741.70 (2356.45 to 10307.76) | 3593.33 (1782.05 to 7820.93) | 2253.11 (1117.09 to 4901.10) | 1301.89 (645.84 to 2833.65) | 676.64 (335.89 to 1471.75) | 286.15 (141.96 to 622.04) | 78.61 (39.00 to 170.71) | 15.50 (7.69 to 33.69) |
| Haiti | Female | Number of DALYs | 87.20 (51.71 to 136.61) | 135.43 (77.36 to 214.26) | 125.91 (72.46 to 198.02) | 121.03 (70.53 to 189.16) | 113.23 (67.81 to 172.55) | 108.32 (64.18 to 168.79) | 107.06 (62.12 to 163.93) | 98.42 (57.50 to 155.63) | 79.16 (47.16 to 122.61) | 60.36 (35.06 to 92.35) | 46.89 (28.75 to 70.31) | 38.37 (22.50 to 59.46) | 29.71 (18.63 to 44.90) | 22.10 (13.86 to 32.85) | 13.70 (8.14 to 21.14) | 7.69 (4.59 to 11.53) | 3.92 (2.28 to 5.98) | 1.57 (0.97 to 2.40) | 0.41 (0.25 to 0.60) | 0.08 (0.05 to 0.12) |
| Haiti | Female | Number of prevalence | 1859.32 (1223.75 to 3352.60) | 3687.40 (2227.00 to 7136.58) | 4445.44 (2466.96 to 9111.79) | 5282.07 (2747.19 to 11246.51) | 5744.07 (2875.54 to 12498.64) | 6141.60 (3005.44 to 13521.27) | 6487.44 (3133.90 to 14379.96) | 6196.11 (2965.75 to 13801.24) | 5273.75 (2503.87 to 11793.15) | 4193.60 (1976.85 to 9404.88) | 3387.39 (1589.32 to 7619.56) | 2847.93 (1328.20 to 6421.44) | 2317.95 (1075.22 to 5241.30) | 1766.25 (815.73 to 3998.83) | 1094.09 (505.36 to 2476.83) | 624.19 (288.38 to 1413.20) | 324.36 (149.80 to 734.20) | 132.33 (61.14 to 299.64) | 34.62 (15.99 to 78.38) | 7.21 (3.33 to 16.34) |
| Haiti | Male | Number of DALYs | 63.00 (33.13 to 102.70) | 276.44 (124.88 to 578.93) | 264.97 (116.22 to 558.85) | 262.67 (115.96 to 547.71) | 254.83 (111.26 to 536.58) | 243.57 (100.29 to 506.76) | 239.87 (107.15 to 494.06) | 210.85 (91.87 to 432.10) | 173.42 (75.76 to 347.09) | 137.24 (62.34 to 280.16) | 110.79 (49.82 to 222.26) | 89.45 (42.73 to 178.39) | 69.60 (31.35 to 135.17) | 51.35 (23.25 to 99.58) | 32.03 (14.83 to 63.49) | 18.45 (8.40 to 37.16) | 9.45 (4.34 to 19.15) | 4.03 (1.96 to 8.18) | 1.13 (0.50 to 2.22) | 0.21 (0.10 to 0.41) |
| Haiti | Male | Number of prevalence | 1655.32 (994.48 to 3170.38) | 4786.97 (2797.62 to 8685.10) | 5544.85 (3119.62 to 10722.13) | 6377.02 (3454.06 to 12608.59) | 6699.81 (3567.78 to 13591.12) | 6903.98 (3638.51 to 14205.50) | 7019.99 (3679.89 to 14554.01) | 6435.38 (3354.04 to 13421.02) | 5465.22 (2839.08 to 11443.89) | 4450.01 (2306.24 to 9350.42) | 3647.54 (1886.13 to 7677.67) | 3035.44 (1566.46 to 6406.56) | 2423.75 (1246.90 to 5125.81) | 1827.08 (937.47 to 3868.53) | 1159.02 (594.48 to 2454.29) | 677.69 (347.85 to 1436.12) | 352.28 (180.92 to 745.82) | 153.82 (78.97 to 325.71) | 44.00 (22.57 to 93.21) | 8.28 (4.25 to 17.54) |
| India | Both | Number of DALYs | 11272.27 (7613.97 to 15897.69) | 41434.53 (22957.42 to 75468.31) | 50903.02 (28294.96 to 90176.59) | 60175.19 (33695.56 to 106332.74) | 66332.56 (37753.84 to 114685.12) | 65960.12 (37576.04 to 111319.18) | 63814.65 (36052.05 to 107872.23) | 60467.64 (34657.35 to 101569.67) | 53802.89 (30964.82 to 88832.73) | 47632.87 (27795.85 to 78614.64) | 40661.16 (23816.06 to 67119.19) | 33559.31 (19688.76 to 54735.99) | 29044.74 (17489.22 to 46724.37) | 23466.84 (14219.02 to 37484.57) | 16781.59 (10121.71 to 26826.58) | 10102.30 (6255.74 to 16223.01) | 5301.42 (3306.02 to 8257.44) | 2056.91 (1276.79 to 3196.49) | 569.41 (359.78 to 891.55) | 100.05 (64.27 to 153.34) |
| India | Both | Number of prevalence | 502901.44 (443876.82 to 566343.16) | 1392908.06 (1195117.03 to 1688250.04) | 2060996.85 (1771114.53 to 2459554.24) | 2748770.29 (2361668.17 to 3259089.35) | 3203388.26 (2761319.85 to 3764254.93) | 3252150.67 (2812097.91 to 3794892.09) | 3172703.44 (2756255.73 to 3681632.84) | 3049787.34 (2656138.66 to 3521998.89) | 2737228.70 (2385750.18 to 3155364.49) | 2433656.02 (2123119.83 to 2800182.11) | 2107358.85 (1842541.68 to 2417565.99) | 1779343.13 (1559395.72 to 2037791.91) | 1575132.74 (1382612.94 to 1800457.61) | 1301609.84 (1141063.96 to 1487013.42) | 956152.24 (836106.75 to 1091887.61) | 595228.76 (520510.85 to 679016.54) | 330681.85 (289926.91 to 377363.02) | 134320.58 (118252.81 to 153316.14) | 38705.94 (33966.82 to 44089.03) | 7990.63 (6972.17 to 9234.98) |
| India | Female | Number of DALYs | 6320.25 (4218.40 to 8915.43) | 11636.34 (7960.15 to 16495.91) | 12904.86 (8611.88 to 18446.25) | 13582.63 (9194.86 to 19079.61) | 13741.51 (9285.16 to 19741.34) | 13020.41 (8861.61 to 18354.96) | 12186.55 (8225.29 to 17271.41) | 11258.20 (7728.30 to 15854.25) | 9759.30 (6692.54 to 13657.30) | 8405.15 (5700.72 to 11727.30) | 7261.33 (5051.74 to 10071.60) | 6084.82 (4200.42 to 8648.37) | 5246.65 (3576.91 to 7486.91) | 4177.77 (2870.37 to 5829.36) | 2989.87 (2067.69 to 4104.25) | 1845.11 (1264.86 to 2533.49) | 1105.28 (773.65 to 1524.62) | 474.70 (336.54 to 638.26) | 143.79 (102.98 to 193.15) | 34.53 (24.82 to 46.40) |
| India | Female | Number of prevalence | 248300.26 (219826.57 to 278356.84) | 601017.46 (527293.35 to 675085.13) | 892383.88 (777325.21 to 1007871.06) | 1191006.87 (1031920.15 to 1351751.10) | 1399276.10 (1211085.53 to 1593724.59) | 1425129.09 (1236754.10 to 1628122.63) | 1383607.25 (1205040.26 to 1576462.95) | 1335620.49 (1165464.86 to 1520019.24) | 1196794.43 (1043815.47 to 1362310.17) | 1057476.31 (924044.67 to 1203909.87) | 928941.93 (814325.79 to 1056476.53) | 800506.42 (702825.16 to 910929.58) | 721658.04 (633699.68 to 821806.99) | 599725.40 (526159.21 to 683261.84) | 442967.98 (388743.82 to 504136.42) | 279362.77 (245505.52 to 316745.36) | 166830.32 (147106.10 to 188839.72) | 70966.28 (62636.82 to 80483.66) | 21225.52 (18733.12 to 24138.78) | 5208.23 (4594.26 to 5920.65) |
| India | Male | Number of DALYs | 4952.02 (3257.21 to 7063.00) | 29798.19 (14276.76 to 59867.77) | 37998.17 (18246.24 to 73242.60) | 46592.55 (23152.12 to 89375.22) | 52591.05 (26727.19 to 98939.85) | 52939.71 (27592.46 to 96555.14) | 51628.10 (26775.01 to 92323.01) | 49209.45 (25735.59 to 87835.94) | 44043.59 (23259.24 to 77819.28) | 39227.72 (21460.42 to 69240.93) | 33399.83 (18222.41 to 58615.07) | 27474.49 (15038.52 to 47473.61) | 23798.09 (13156.87 to 40307.44) | 19289.08 (10885.08 to 32581.09) | 13791.72 (7767.29 to 23241.84) | 8257.19 (4706.84 to 14023.82) | 4196.14 (2380.26 to 6996.73) | 1582.22 (902.76 to 2668.51) | 425.62 (242.22 to 724.99) | 65.52 (36.57 to 113.34) |
| India | Male | Number of prevalence | 254601.17 (223702.08 to 288705.11) | 791890.60 (645907.71 to 1043994.01) | 1168612.97 (970406.69 to 1483875.42) | 1557763.43 (1302549.67 to 1925622.60) | 1804112.16 (1513412.92 to 2201565.18) | 1827021.58 (1540576.30 to 2213444.90) | 1789096.20 (1516129.62 to 2162437.27) | 1714166.85 (1458993.43 to 2066077.97) | 1540434.27 (1312672.49 to 1855021.01) | 1376179.70 (1175556.44 to 1655913.44) | 1178416.92 (1007931.90 to 1415022.20) | 978836.71 (838710.11 to 1173128.05) | 853474.71 (733357.48 to 1016733.22) | 701884.44 (604304.65 to 832447.21) | 513184.26 (441968.17 to 608116.80) | 315865.99 (271944.37 to 374002.93) | 163851.54 (140899.15 to 194074.30) | 63354.30 (54501.24 to 75196.99) | 17480.42 (15013.63 to 20799.37) | 2782.39 (2363.34 to 3416.42) |
| Indonesia | Both | Number of DALYs | 2169.09 (1462.04 to 3120.63) | 6804.63 (3837.15 to 12716.21) | 7416.13 (4154.14 to 13387.04) | 8085.94 (4553.09 to 14457.59) | 8813.43 (4959.81 to 15488.11) | 9226.90 (5160.53 to 16137.81) | 9294.28 (5213.52 to 16436.95) | 9074.65 (5069.38 to 15895.18) | 8795.12 (4967.03 to 15262.50) | 8027.95 (4592.26 to 14006.13) | 7067.34 (4034.47 to 12333.62) | 5924.65 (3413.79 to 10249.18) | 4687.17 (2662.33 to 8050.40) | 3378.45 (1956.23 to 5692.99) | 2020.87 (1175.78 to 3476.22) | 1101.28 (653.11 to 1843.36) | 529.91 (321.83 to 873.89) | 192.12 (117.09 to 316.49) | 52.08 (31.82 to 82.21) | 9.47 (6.09 to 14.90) |
| Indonesia | Both | Number of prevalence | 63270.50 (31979.99 to 141110.11) | 165814.06 (79944.43 to 406387.46) | 223171.87 (101409.13 to 509504.48) | 285380.12 (123146.13 to 666433.03) | 343773.75 (143808.97 to 818659.69) | 381195.87 (156418.72 to 920188.42) | 398721.67 (162439.98 to 974532.61) | 399370.29 (160578.72 to 996115.06) | 396536.34 (158467.64 to 1012283.13) | 370085.16 (146452.12 to 969666.73) | 332896.48 (130555.78 to 891795.02) | 285382.49 (110353.16 to 790779.67) | 231837.45 (87611.78 to 684910.43) | 171741.14 (64287.28 to 517809.36) | 106362.04 (38863.09 to 334494.79) | 60601.26 (21764.91 to 194241.21) | 30369.40 (10622.51 to 102113.75) | 11563.59 (3929.07 to 40950.53) | 3307.64 (1100.64 to 12000.15) | 653.63 (210.35 to 2396.34) |
| Indonesia | Female | Number of DALYs | 1237.64 (817.57 to 1785.35) | 2144.83 (1422.78 to 3110.17) | 2189.70 (1461.81 to 3172.16) | 2229.23 (1469.28 to 3239.40) | 2307.92 (1503.70 to 3288.04) | 2319.97 (1602.68 to 3357.42) | 2306.09 (1527.35 to 3309.04) | 2203.25 (1471.84 to 3118.86) | 2123.24 (1433.01 to 3023.51) | 1926.08 (1301.29 to 2766.86) | 1684.54 (1117.89 to 2418.67) | 1398.22 (950.56 to 1990.26) | 1094.46 (715.71 to 1573.40) | 796.09 (523.47 to 1116.20) | 489.73 (343.06 to 675.56) | 287.44 (189.19 to 403.50) | 149.72 (103.14 to 212.01) | 59.18 (40.68 to 81.91) | 17.93 (12.17 to 24.56) | 3.79 (2.68 to 5.20) |
| Indonesia | Female | Number of prevalence | 32631.84 (17294.99 to 70786.95) | 72083.14 (33113.40 to 188729.62) | 97524.66 (40804.19 to 236831.57) | 125318.61 (49283.03 to 312347.74) | 151767.16 (56595.77 to 385242.83) | 168974.00 (60864.99 to 434210.19) | 177900.64 (62882.95 to 461600.80) | 179329.41 (62648.37 to 471699.64) | 179194.51 (62066.53 to 479745.98) | 167882.17 (57226.16 to 463161.29) | 151239.05 (50522.45 to 429694.45) | 130247.67 (42226.63 to 384838.78) | 105902.35 (33107.84 to 336060.55) | 79351.97 (24366.11 to 257269.46) | 50348.54 (15077.56 to 170191.97) | 30255.18 (8794.68 to 106475.42) | 15910.18 (4389.03 to 58364.35) | 6391.56 (1727.53 to 24598.33) | 1959.57 (517.95 to 7839.17) | 426.73 (110.76 to 1743.03) |
| Indonesia | Male | Number of DALYs | 931.45 (612.90 to 1369.85) | 4659.80 (2271.14 to 9584.30) | 5226.42 (2508.36 to 10530.63) | 5856.72 (2858.17 to 11780.85) | 6505.51 (3156.63 to 12536.37) | 6906.94 (3435.70 to 13449.47) | 6988.19 (3537.04 to 13535.17) | 6871.39 (3460.22 to 13058.89) | 6671.88 (3335.92 to 12556.03) | 6101.87 (3058.20 to 11445.26) | 5382.80 (2701.20 to 10241.90) | 4526.42 (2325.58 to 8567.35) | 3592.71 (1824.61 to 6648.58) | 2582.36 (1341.20 to 4693.39) | 1531.14 (795.80 to 2845.76) | 813.85 (430.10 to 1501.31) | 380.19 (202.81 to 695.22) | 132.94 (71.45 to 243.22) | 34.15 (17.73 to 61.55) | 5.68 (3.05 to 10.35) |
| Indonesia | Male | Number of prevalence | 30638.66 (14560.56 to 70323.16) | 93730.92 (44815.59 to 217229.39) | 125647.21 (57697.61 to 276999.24) | 160061.51 (70508.74 to 356443.62) | 192006.59 (83336.15 to 438867.88) | 212221.87 (91312.11 to 493195.50) | 220821.04 (94988.87 to 520554.36) | 220040.89 (93408.24 to 529088.72) | 217341.83 (91463.45 to 533236.38) | 202202.99 (84168.02 to 504789.79) | 181657.43 (75261.15 to 462100.57) | 155134.82 (63999.21 to 405940.89) | 125935.10 (51115.87 to 349510.54) | 92389.16 (37363.32 to 260539.90) | 56013.51 (22253.66 to 164607.38) | 30346.08 (12076.35 to 88387.20) | 14459.22 (5689.97 to 43886.92) | 5172.03 (2000.93 to 16179.03) | 1348.08 (522.63 to 4228.10) | 226.90 (89.66 to 669.16) |
| Kenya | Both | Number of DALYs | 591.44 (393.09 to 845.37) | 1830.62 (1052.13 to 3346.94) | 1936.60 (1103.22 to 3526.50) | 1870.57 (1057.10 to 3375.93) | 1598.73 (908.86 to 2884.69) | 1375.70 (785.11 to 2422.21) | 1230.85 (696.63 to 2173.01) | 1074.49 (606.54 to 1931.74) | 880.40 (493.36 to 1590.63) | 706.99 (399.44 to 1273.78) | 532.85 (301.78 to 940.70) | 401.71 (229.58 to 704.26) | 322.69 (184.41 to 564.18) | 242.64 (141.11 to 420.28) | 156.65 (92.99 to 268.35) | 85.80 (51.54 to 145.70) | 37.48 (22.82 to 62.41) | 13.89 (8.66 to 22.24) | 3.65 (2.38 to 5.65) | 0.80 (0.54 to 1.17) |
| Kenya | Both | Number of prevalence | 14595.39 (7618.19 to 33432.89) | 38054.09 (19472.69 to 89121.94) | 48048.67 (22120.00 to 122481.88) | 54533.01 (22775.03 to 148741.62) | 53193.37 (20552.50 to 153287.01) | 49755.79 (18381.95 to 146780.11) | 45681.86 (16468.80 to 135558.42) | 40664.80 (14410.70 to 123101.47) | 33664.11 (11826.47 to 102166.90) | 27297.83 (9444.33 to 83132.97) | 21017.02 (7215.86 to 64445.75) | 16264.14 (5546.05 to 50115.04) | 13475.58 (4580.77 to 40555.08) | 10438.80 (3536.30 to 30944.32) | 6969.17 (2356.65 to 20544.27) | 3979.67 (1347.76 to 11350.84) | 1825.86 (610.65 to 5344.89) | 701.05 (230.65 to 2055.32) | 197.85 (63.25 to 585.04) | 48.71 (14.83 to 153.73) |
| Kenya | Female | Number of DALYs | 341.47 (227.65 to 496.51) | 601.64 (404.06 to 860.18) | 610.73 (405.64 to 870.91) | 570.44 (388.14 to 816.10) | 497.40 (336.33 to 709.63) | 422.88 (289.03 to 594.23) | 361.95 (250.34 to 515.87) | 305.48 (207.30 to 433.24) | 238.86 (164.11 to 340.12) | 185.31 (127.87 to 259.99) | 143.12 (96.83 to 200.84) | 111.03 (75.96 to 154.28) | 91.95 (63.76 to 130.50) | 72.40 (50.13 to 100.90) | 46.97 (32.93 to 63.96) | 27.36 (19.12 to 37.29) | 14.27 (10.01 to 19.62) | 6.17 (4.33 to 8.41) | 1.88 (1.32 to 2.58) | 0.49 (0.35 to 0.67) |
| Kenya | Female | Number of prevalence | 7668.45 (4206.99 to 16939.93) | 16488.27 (7950.41 to 40486.90) | 20814.93 (8806.26 to 57520.86) | 23954.09 (8971.15 to 70248.43) | 24665.16 (8416.40 to 75715.19) | 23500.72 (7616.76 to 73555.95) | 21101.75 (6578.47 to 66171.32) | 18503.35 (5585.95 to 58970.29) | 14836.87 (4414.04 to 47386.14) | 11783.02 (3484.87 to 37770.33) | 9203.29 (2717.13 to 29928.40) | 7271.54 (2131.19 to 24065.07) | 6219.42 (1814.45 to 19852.03) | 5017.21 (1456.02 to 15803.48) | 3368.31 (963.34 to 10504.59) | 2009.92 (574.01 to 6201.46) | 1040.28 (300.23 to 3213.61) | 439.85 (127.51 to 1368.78) | 136.54 (38.91 to 426.58) | 37.65 (10.35 to 122.50) |
| Kenya | Male | Number of DALYs | 249.97 (164.76 to 354.28) | 1228.98 (587.63 to 2518.02) | 1325.88 (641.39 to 2730.64) | 1300.13 (635.35 to 2636.00) | 1101.32 (539.82 to 2233.44) | 952.82 (458.88 to 1880.73) | 868.90 (418.65 to 1740.94) | 769.01 (379.69 to 1553.16) | 641.55 (314.36 to 1303.39) | 521.67 (254.60 to 1038.98) | 389.73 (191.82 to 755.58) | 290.67 (140.99 to 568.11) | 230.74 (113.96 to 449.52) | 170.24 (84.79 to 332.40) | 109.68 (55.07 to 209.30) | 58.44 (29.94 to 109.97) | 23.22 (11.73 to 45.11) | 7.72 (3.95 to 14.89) | 1.78 (0.91 to 3.40) | 0.31 (0.16 to 0.59) |
| Kenya | Male | Number of prevalence | 6926.94 (3293.79 to 16492.96) | 21565.82 (10816.64 to 48614.57) | 27233.74 (12642.61 to 65774.89) | 30578.92 (13184.83 to 78573.15) | 28528.21 (11699.89 to 77348.95) | 26255.06 (10530.09 to 73224.16) | 24580.12 (9640.66 to 70491.35) | 22161.45 (8565.70 to 64594.94) | 18827.24 (7221.95 to 54829.75) | 15514.81 (5911.79 to 45098.82) | 11813.73 (4481.06 to 34423.38) | 8992.60 (3398.22 to 26438.16) | 7256.16 (2741.70 to 21174.98) | 5421.59 (2042.97 to 15444.96) | 3600.86 (1378.26 to 9936.22) | 1969.75 (765.35 to 5224.92) | 785.58 (298.28 to 2092.55) | 261.20 (98.05 to 711.43) | 61.31 (22.35 to 167.90) | 11.06 (3.88 to 30.28) |
| Kiribati | Both | Number of DALYs | 1.40 (0.85 to 2.08) | 4.26 (2.23 to 7.94) | 4.24 (2.24 to 7.76) | 3.83 (1.97 to 7.08) | 3.67 (1.91 to 6.40) | 3.74 (1.98 to 6.88) | 3.46 (1.81 to 6.07) | 3.07 (1.66 to 5.47) | 2.63 (1.42 to 4.71) | 2.02 (1.14 to 3.52) | 1.73 (0.92 to 3.07) | 1.71 (0.93 to 2.95) | 1.37 (0.74 to 2.47) | 0.76 (0.42 to 1.32) | 0.40 (0.23 to 0.68) | 0.25 (0.15 to 0.42) | 0.12 (0.07 to 0.19) | 0.04 (0.02 to 0.06) | 0.01 (0.01 to 0.01) | 0.00 (0.00 to 0.00) |
| Kiribati | Both | Number of prevalence | 40.64 (14.08 to 123.84) | 104.83 (34.66 to 308.65) | 128.55 (35.71 to 404.68) | 138.89 (32.67 to 456.81) | 150.96 (31.86 to 509.92) | 164.68 (32.88 to 564.22) | 160.35 (30.95 to 553.84) | 148.00 (27.80 to 514.33) | 131.80 (24.29 to 460.26) | 104.83 (18.97 to 367.44) | 91.04 (16.27 to 319.82) | 91.85 (16.23 to 323.26) | 77.06 (13.34 to 272.45) | 46.74 (7.83 to 166.77) | 25.96 (4.27 to 93.24) | 16.61 (2.74 to 59.60) | 7.85 (1.29 to 28.19) | 2.67 (0.43 to 9.62) | 0.64 (0.10 to 2.32) | 0.11 (0.02 to 0.41) |
| Kiribati | Female | Number of DALYs | 0.80 (0.46 to 1.22) | 1.33 (0.76 to 2.04) | 1.30 (0.79 to 1.98) | 1.15 (0.70 to 1.79) | 1.07 (0.61 to 1.65) | 1.08 (0.64 to 1.62) | 0.98 (0.58 to 1.53) | 0.86 (0.52 to 1.31) | 0.75 (0.43 to 1.15) | 0.57 (0.35 to 0.86) | 0.48 (0.28 to 0.73) | 0.46 (0.28 to 0.72) | 0.38 (0.23 to 0.56) | 0.25 (0.15 to 0.38) | 0.15 (0.09 to 0.23) | 0.09 (0.06 to 0.14) | 0.04 (0.03 to 0.07) | 0.02 (0.01 to 0.02) | 0.00 (0.00 to 0.01) | 0.00 (0.00 to 0.00) |
| Kiribati | Female | Number of prevalence | 20.74 (7.95 to 60.84) | 45.29 (13.70 to 144.45) | 57.26 (13.57 to 194.45) | 63.82 (12.41 to 225.65) | 70.76 (12.00 to 255.77) | 78.07 (12.19 to 285.53) | 76.47 (11.32 to 281.62) | 71.37 (10.13 to 264.10) | 64.42 (8.86 to 239.30) | 51.71 (6.95 to 192.63) | 44.48 (5.82 to 166.15) | 44.28 (5.67 to 165.83) | 38.31 (4.77 to 143.83) | 26.22 (3.23 to 98.58) | 15.96 (1.97 to 60.01) | 10.09 (1.24 to 37.93) | 4.83 (0.60 to 18.17) | 1.71 (0.21 to 6.42) | 0.43 (0.05 to 1.62) | 0.08 (0.01 to 0.31) |
| Kiribati | Male | Number of DALYs | 0.60 (0.33 to 0.97) | 2.93 (1.27 to 6.36) | 2.94 (1.28 to 6.11) | 2.68 (1.14 to 5.60) | 2.60 (1.14 to 5.04) | 2.65 (1.13 to 5.23) | 2.47 (1.08 to 4.89) | 2.21 (1.00 to 4.46) | 1.88 (0.88 to 3.70) | 1.45 (0.68 to 2.91) | 1.25 (0.56 to 2.46) | 1.25 (0.57 to 2.42) | 0.99 (0.45 to 1.99) | 0.51 (0.24 to 1.01) | 0.24 (0.11 to 0.47) | 0.16 (0.08 to 0.30) | 0.07 (0.03 to 0.14) | 0.02 (0.01 to 0.04) | 0.00 (0.00 to 0.01) | 0.00 (0.00 to 0.00) |
| Kiribati | Male | Number of prevalence | 19.90 (6.18 to 63.00) | 59.54 (19.35 to 162.36) | 71.29 (20.03 to 211.75) | 75.07 (18.87 to 235.51) | 80.19 (18.90 to 257.77) | 86.61 (19.58 to 281.84) | 83.88 (18.53 to 274.87) | 76.63 (16.64 to 252.32) | 67.38 (14.41 to 222.75) | 53.12 (11.23 to 176.07) | 46.56 (9.80 to 154.74) | 47.56 (9.93 to 158.45) | 38.75 (8.05 to 129.38) | 20.52 (4.23 to 68.58) | 10.00 (2.07 to 33.42) | 6.52 (1.35 to 21.79) | 3.01 (0.62 to 10.08) | 0.96 (0.20 to 3.22) | 0.21 (0.04 to 0.71) | 0.03 (0.01 to 0.11) |
| Lao People's Democratic Republic | Both | Number of DALYs | 79.56 (49.73 to 119.74) | 207.00 (110.12 to 374.73) | 193.80 (103.01 to 350.20) | 192.20 (98.24 to 351.69) | 190.65 (106.82 to 341.21) | 190.48 (103.88 to 346.35) | 177.19 (95.31 to 312.30) | 150.12 (83.78 to 269.13) | 123.10 (65.17 to 227.80) | 101.27 (54.91 to 187.82) | 85.11 (46.13 to 148.51) | 69.09 (37.92 to 131.52) | 53.46 (28.75 to 95.82) | 36.17 (20.55 to 64.28) | 23.27 (12.88 to 41.53) | 13.84 (7.70 to 24.75) | 7.62 (4.25 to 13.36) | 3.09 (1.78 to 5.30) | 0.90 (0.48 to 1.52) | 0.21 (0.13 to 0.33) |
| Lao People's Democratic Republic | Both | Number of prevalence | 1129.42 (768.78 to 2875.66) | 2688.25 (1559.68 to 6368.23) | 2882.21 (1494.39 to 7996.54) | 3248.81 (1515.80 to 10167.71) | 3539.18 (1531.80 to 11911.20) | 3735.31 (1535.27 to 13096.73) | 3656.72 (1450.95 to 13144.49) | 3205.53 (1240.14 to 11742.88) | 2696.58 (1022.21 to 10026.02) | 2305.08 (855.38 to 8684.41) | 1991.36 (727.90 to 7596.91) | 1673.11 (600.26 to 6448.50) | 1337.57 (470.81 to 5209.24) | 932.96 (324.60 to 3653.62) | 614.38 (213.91 to 2416.13) | 375.19 (130.55 to 1478.56) | 212.97 (74.08 to 844.53) | 88.79 (30.87 to 354.43) | 26.69 (9.25 to 107.15) | 6.67 (2.32 to 27.18) |
| Lao People's Democratic Republic | Female | Number of DALYs | 45.71 (26.79 to 71.41) | 70.07 (39.85 to 107.31) | 65.87 (38.06 to 103.59) | 63.72 (36.30 to 104.43) | 63.55 (38.43 to 97.20) | 63.07 (37.02 to 96.62) | 57.33 (35.44 to 89.40) | 49.04 (28.77 to 74.59) | 40.20 (23.60 to 64.86) | 33.52 (19.77 to 52.11) | 28.80 (16.88 to 44.04) | 23.41 (13.89 to 36.50) | 17.81 (10.50 to 27.42) | 12.14 (7.29 to 18.68) | 8.17 (4.84 to 12.80) | 4.95 (2.95 to 7.65) | 2.97 (1.79 to 4.57) | 1.31 (0.77 to 2.03) | 0.41 (0.23 to 0.62) | 0.11 (0.07 to 0.17) |
| Lao People's Democratic Republic | Female | Number of prevalence | 618.95 (434.10 to 1470.68) | 1090.45 (683.85 to 3040.55) | 1196.31 (653.76 to 3859.14) | 1381.78 (655.73 to 4963.93) | 1534.97 (659.91 to 5875.09) | 1621.95 (652.45 to 6438.82) | 1581.82 (607.82 to 6421.66) | 1394.16 (520.09 to 5751.10) | 1176.59 (426.13 to 4914.51) | 1021.97 (361.99 to 4311.67) | 902.51 (313.22 to 3841.39) | 757.00 (256.95 to 3251.28) | 601.81 (199.40 to 2609.20) | 422.81 (138.40 to 1841.01) | 291.26 (95.43 to 1268.12) | 181.37 (59.60 to 789.68) | 109.48 (35.87 to 476.71) | 48.63 (15.94 to 211.74) | 15.38 (5.04 to 66.99) | 4.36 (1.43 to 19.00) |
| Lao People's Democratic Republic | Male | Number of DALYs | 33.85 (18.70 to 55.39) | 136.93 (59.83 to 293.36) | 127.93 (57.38 to 271.31) | 128.48 (54.07 to 266.11) | 127.10 (55.12 to 274.81) | 127.40 (57.09 to 260.58) | 119.86 (52.13 to 237.74) | 101.08 (45.34 to 208.13) | 82.90 (36.95 to 173.67) | 67.75 (30.22 to 145.48) | 56.31 (23.62 to 114.05) | 45.69 (20.35 to 98.96) | 35.65 (15.63 to 72.88) | 24.04 (10.94 to 48.11) | 15.10 (6.72 to 32.63) | 8.89 (3.97 to 18.83) | 4.65 (2.20 to 10.08) | 1.78 (0.81 to 3.85) | 0.49 (0.22 to 0.99) | 0.10 (0.04 to 0.20) |
| Lao People's Democratic Republic | Male | Number of prevalence | 510.47 (322.76 to 1404.98) | 1597.80 (826.23 to 3506.63) | 1685.89 (801.22 to 4167.15) | 1867.03 (823.72 to 5238.93) | 2004.21 (835.42 to 6077.86) | 2113.35 (846.39 to 6706.60) | 2074.90 (812.15 to 6771.89) | 1811.38 (693.53 to 6030.19) | 1519.98 (573.01 to 5142.12) | 1283.11 (476.35 to 4396.52) | 1088.86 (398.67 to 3775.03) | 916.10 (331.57 to 3212.07) | 735.75 (262.50 to 2610.71) | 510.16 (180.34 to 1820.69) | 323.12 (114.20 to 1152.61) | 193.83 (68.52 to 691.64) | 103.49 (36.61 to 369.28) | 40.16 (14.20 to 143.26) | 11.30 (4.01 to 40.34) | 2.30 (0.82 to 8.22) |
| Liberia | Both | Number of DALYs | 88.59 (54.88 to 131.70) | 568.85 (322.25 to 970.06) | 856.82 (513.11 to 1389.91) | 1151.47 (731.30 to 1724.64) | 1269.95 (818.34 to 1881.16) | 1250.90 (823.02 to 1809.14) | 1175.87 (785.43 to 1675.70) | 1242.48 (829.05 to 1764.03) | 1196.24 (798.13 to 1652.29) | 987.64 (671.33 to 1378.99) | 575.69 (382.44 to 802.55) | 462.66 (315.38 to 634.79) | 327.08 (221.76 to 446.87) | 241.74 (162.77 to 326.60) | 138.72 (95.31 to 189.16) | 84.86 (58.61 to 114.81) | 49.84 (34.35 to 67.41) | 23.96 (16.59 to 32.27) | 6.82 (4.70 to 9.01) | 1.70 (1.18 to 2.27) |
| Liberia | Both | Number of prevalence | 12675.21 (9786.69 to 16438.93) | 32720.15 (24991.56 to 42377.50) | 46040.07 (35325.64 to 59457.54) | 55465.58 (42924.87 to 71079.49) | 57247.88 (44601.96 to 73051.37) | 55282.09 (43228.48 to 70369.39) | 51284.62 (40234.71 to 64960.25) | 53746.67 (42316.53 to 67870.16) | 49781.21 (39397.11 to 62636.21) | 41678.83 (33028.36 to 52413.99) | 24283.84 (19278.29 to 30500.75) | 20415.43 (16208.55 to 25710.03) | 14453.81 (11510.70 to 18189.46) | 11070.55 (8803.77 to 13940.37) | 6618.31 (5255.56 to 8344.38) | 4238.31 (3361.08 to 5348.11) | 2508.73 (1990.10 to 3165.40) | 1150.70 (915.71 to 1448.11) | 356.77 (283.01 to 450.32) | 90.99 (72.19 to 114.86) |
| Liberia | Female | Number of DALYs | 50.63 (30.19 to 78.16) | 90.17 (53.74 to 142.57) | 99.21 (60.30 to 148.21) | 100.73 (62.54 to 150.21) | 97.69 (60.48 to 140.00) | 91.29 (57.05 to 134.00) | 82.17 (51.52 to 115.28) | 83.41 (52.82 to 121.79) | 70.95 (45.31 to 101.90) | 59.60 (38.32 to 87.35) | 33.50 (22.06 to 49.30) | 30.08 (19.89 to 42.34) | 20.54 (13.49 to 29.56) | 16.14 (10.54 to 22.97) | 9.91 (6.55 to 14.11) | 6.55 (4.46 to 9.34) | 3.76 (2.53 to 5.39) | 1.53 (1.01 to 2.19) | 0.52 (0.36 to 0.73) | 0.13 (0.09 to 0.18) |
| Liberia | Female | Number of prevalence | 6270.20 (4851.50 to 8112.99) | 14556.73 (11201.95 to 18922.78) | 20040.53 (15374.57 to 26125.03) | 23813.45 (18240.86 to 31092.22) | 24557.27 (18802.72 to 32078.19) | 23804.64 (18223.06 to 31105.55) | 21855.97 (16732.93 to 28561.17) | 22485.12 (17215.48 to 29382.47) | 19203.41 (14705.23 to 25090.53) | 16049.51 (12291.44 to 20973.02) | 9158.46 (7013.81 to 11965.70) | 8189.56 (6271.95 to 10699.35) | 5617.16 (4303.07 to 7338.72) | 4431.33 (3394.36 to 5788.00) | 2757.41 (2112.25 to 3601.82) | 1842.54 (1411.31 to 2406.79) | 1080.94 (828.09 to 1412.13) | 445.18 (341.01 to 581.53) | 155.30 (118.97 to 202.87) | 39.49 (30.26 to 51.59) |
| Liberia | Male | Number of DALYs | 37.96 (20.78 to 60.57) | 478.67 (245.04 to 865.88) | 757.61 (431.44 to 1263.74) | 1050.75 (655.58 to 1591.60) | 1172.26 (741.70 to 1734.26) | 1159.61 (762.68 to 1681.35) | 1093.71 (721.78 to 1567.61) | 1159.07 (769.29 to 1643.87) | 1125.28 (749.30 to 1562.86) | 928.04 (622.55 to 1295.74) | 542.19 (358.84 to 763.94) | 432.58 (293.34 to 596.31) | 306.54 (204.72 to 420.91) | 225.61 (151.52 to 306.90) | 128.81 (88.51 to 176.72) | 78.32 (53.80 to 105.59) | 46.08 (31.57 to 62.43) | 22.43 (15.53 to 30.16) | 6.30 (4.30 to 8.42) | 1.57 (1.08 to 2.10) |
| Liberia | Male | Number of prevalence | 6405.01 (4935.19 to 8326.69) | 18163.42 (13946.47 to 23247.23) | 25999.54 (20248.68 to 33063.54) | 31652.12 (25192.82 to 39762.61) | 32690.61 (26281.82 to 40682.41) | 31477.45 (25473.47 to 39022.90) | 29428.65 (23986.64 to 36336.90) | 31261.55 (25610.63 to 38503.11) | 30577.80 (25186.74 to 37589.58) | 25629.32 (21120.45 to 31495.75) | 15125.38 (12461.15 to 18549.37) | 12225.87 (10072.81 to 15014.50) | 8836.66 (7283.20 to 10854.51) | 6639.22 (5462.24 to 8154.00) | 3860.90 (3176.01 to 4743.70) | 2395.77 (1968.85 to 2942.09) | 1427.79 (1173.70 to 1753.87) | 705.52 (579.20 to 866.76) | 201.47 (165.50 to 247.53) | 51.50 (42.29 to 63.29) |
| Madagascar | Both | Number of DALYs | 400.97 (246.94 to 603.93) | 1132.41 (537.84 to 2165.71) | 1055.41 (571.24 to 1856.07) | 987.67 (528.94 to 1783.27) | 857.98 (459.69 to 1523.65) | 729.84 (394.47 to 1306.73) | 614.14 (339.74 to 1124.82) | 517.31 (276.66 to 915.86) | 443.23 (234.18 to 794.37) | 363.06 (194.54 to 660.04) | 286.74 (156.63 to 498.43) | 223.93 (120.02 to 395.42) | 168.98 (89.70 to 300.55) | 108.39 (58.35 to 187.94) | 61.59 (34.36 to 110.24) | 31.55 (17.38 to 55.55) | 14.86 (8.47 to 25.76) | 5.68 (3.23 to 9.80) | 1.55 (0.88 to 2.62) | 0.32 (0.18 to 0.53) |
| Madagascar | Both | Number of prevalence | 9070.14 (5058.99 to 18862.88) | 22691.16 (11461.46 to 46704.11) | 26316.73 (12210.74 to 57787.06) | 28666.63 (12379.23 to 66466.62) | 28506.55 (11667.36 to 67771.76) | 26264.34 (10397.45 to 63145.98) | 23205.35 (8974.21 to 56109.37) | 20397.80 (7772.32 to 49520.01) | 17774.75 (6695.39 to 43308.12) | 15070.03 (5628.98 to 36834.20) | 12289.52 (4545.82 to 30137.49) | 9825.38 (3614.02 to 24160.86) | 7588.58 (2768.33 to 18715.89) | 5031.65 (1827.10 to 12444.77) | 2936.08 (1062.56 to 7272.29) | 1549.35 (558.78 to 3846.43) | 750.98 (270.62 to 1869.48) | 296.60 (107.01 to 739.14) | 82.85 (29.88 to 206.82) | 18.15 (6.53 to 45.52) |
| Madagascar | Female | Number of DALYs | 231.32 (131.07 to 363.71) | 378.47 (217.66 to 610.08) | 347.08 (204.95 to 547.23) | 318.61 (191.82 to 501.52) | 271.42 (154.11 to 423.92) | 228.36 (129.70 to 361.99) | 190.93 (110.67 to 296.89) | 156.95 (92.69 to 245.62) | 133.34 (77.69 to 196.00) | 109.08 (62.78 to 160.85) | 84.98 (50.82 to 129.95) | 65.39 (37.24 to 101.45) | 48.02 (28.38 to 75.10) | 31.88 (19.54 to 49.42) | 18.30 (10.84 to 28.35) | 9.96 (6.18 to 15.33) | 4.91 (2.80 to 7.68) | 1.95 (1.17 to 2.93) | 0.55 (0.35 to 0.85) | 0.12 (0.07 to 0.18) |
| Madagascar | Female | Number of prevalence | 4804.82 (2819.31 to 9609.50) | 9909.39 (5126.04 to 21606.48) | 11839.29 (5444.48 to 27647.48) | 13193.19 (5501.67 to 32289.28) | 13345.23 (5214.73 to 33511.81) | 12404.70 (4669.66 to 31604.67) | 10992.83 (4032.86 to 28241.90) | 9665.65 (3496.78 to 24983.32) | 8402.90 (2997.76 to 21818.76) | 7148.57 (2525.22 to 18632.22) | 5845.33 (2041.63 to 15286.39) | 4608.56 (1592.39 to 12087.90) | 3510.72 (1201.28 to 9238.94) | 2371.76 (807.31 to 6251.62) | 1418.50 (482.88 to 3738.20) | 777.54 (264.68 to 2049.25) | 392.11 (133.53 to 1033.48) | 157.86 (53.81 to 416.01) | 45.29 (15.44 to 119.37) | 10.60 (3.61 to 27.95) |
| Madagascar | Male | Number of DALYs | 169.65 (95.61 to 274.55) | 753.93 (301.79 to 1670.55) | 708.34 (318.50 to 1489.70) | 669.06 (292.10 to 1406.51) | 586.56 (255.44 to 1196.66) | 501.48 (225.13 to 1007.17) | 423.21 (181.52 to 892.31) | 360.36 (155.69 to 734.04) | 309.89 (138.14 to 648.26) | 253.97 (121.26 to 516.27) | 201.76 (88.81 to 403.00) | 158.54 (72.05 to 316.22) | 120.96 (55.97 to 239.77) | 76.51 (33.97 to 152.93) | 43.29 (19.57 to 87.07) | 21.58 (9.65 to 42.84) | 9.96 (4.77 to 19.71) | 3.73 (1.70 to 7.45) | 1.00 (0.46 to 2.03) | 0.20 (0.09 to 0.39) |
| Madagascar | Male | Number of prevalence | 4265.33 (2194.06 to 9253.38) | 12781.76 (6045.56 to 25859.04) | 14477.43 (6489.62 to 30819.65) | 15473.44 (6555.13 to 34557.12) | 15161.32 (6176.26 to 34851.50) | 13859.64 (5540.35 to 32276.10) | 12212.53 (4828.67 to 28703.84) | 10732.15 (4203.85 to 25357.16) | 9371.85 (3644.12 to 22243.85) | 7921.47 (3055.07 to 18870.33) | 6444.18 (2474.65 to 15385.63) | 5216.82 (1990.11 to 12490.36) | 4077.85 (1547.64 to 9789.13) | 2659.89 (1008.28 to 6404.85) | 1517.58 (576.01 to 3648.24) | 771.82 (292.64 to 1857.18) | 358.87 (136.03 to 863.59) | 138.74 (52.70 to 333.84) | 37.55 (14.25 to 90.24) | 7.55 (2.86 to 18.16) |
| Malawi | Both | Number of DALYs | 260.52 (163.48 to 382.10) | 717.24 (386.84 to 1309.63) | 727.98 (396.93 to 1280.52) | 668.51 (353.44 to 1207.90) | 518.42 (278.35 to 952.30) | 390.71 (223.00 to 717.00) | 341.88 (184.37 to 603.16) | 281.17 (148.78 to 494.69) | 230.42 (122.20 to 405.66) | 173.24 (90.27 to 321.30) | 127.34 (68.35 to 232.13) | 91.40 (50.37 to 164.54) | 73.73 (40.32 to 132.29) | 57.90 (32.00 to 100.00) | 39.02 (22.77 to 64.46) | 20.95 (12.14 to 36.11) | 9.29 (5.48 to 15.05) | 3.43 (1.91 to 5.52) | 0.96 (0.58 to 1.50) | 0.22 (0.13 to 0.34) |
| Malawi | Both | Number of prevalence | 3464.27 (2897.75 to 4514.75) | 8781.82 (6483.48 to 13184.28) | 9917.46 (7138.91 to 15055.37) | 10158.75 (7186.09 to 15671.06) | 8715.35 (6060.43 to 13780.43) | 6977.39 (4797.47 to 11170.79) | 6304.81 (4289.24 to 10203.29) | 5394.57 (3643.88 to 8812.68) | 4553.59 (3057.17 to 7490.83) | 3494.63 (2324.95 to 5768.09) | 2629.90 (1747.86 to 4377.21) | 1955.55 (1294.52 to 3274.99) | 1635.40 (1077.53 to 2754.18) | 1320.10 (867.95 to 2227.49) | 915.39 (601.03 to 1545.15) | 508.38 (335.29 to 858.47) | 236.08 (156.06 to 398.29) | 90.29 (59.91 to 152.19) | 26.52 (17.50 to 44.88) | 6.40 (4.23 to 10.93) |
| Malawi | Female | Number of DALYs | 150.69 (91.03 to 230.22) | 253.36 (142.84 to 403.54) | 255.88 (150.83 to 379.15) | 235.39 (134.39 to 360.27) | 187.59 (111.58 to 293.56) | 141.71 (83.53 to 224.67) | 124.54 (72.05 to 193.21) | 100.35 (60.44 to 153.86) | 78.19 (45.27 to 121.40) | 55.74 (32.13 to 86.95) | 43.25 (23.99 to 66.47) | 32.58 (18.42 to 50.22) | 27.32 (16.62 to 44.01) | 22.46 (13.25 to 35.60) | 15.96 (9.35 to 24.33) | 9.27 (5.84 to 14.01) | 4.35 (2.62 to 6.75) | 1.82 (1.04 to 2.77) | 0.57 (0.34 to 0.84) | 0.15 (0.09 to 0.22) |
| Malawi | Female | Number of prevalence | 1940.56 (1641.34 to 2492.04) | 3623.15 (2929.63 to 4951.14) | 4184.94 (3207.47 to 6105.34) | 4388.41 (3200.02 to 6777.99) | 3910.36 (2742.64 to 6263.85) | 3199.56 (2187.37 to 5235.30) | 2914.01 (1962.30 to 4831.80) | 2507.47 (1667.01 to 4201.97) | 2028.97 (1341.05 to 3428.44) | 1492.30 (977.16 to 2538.37) | 1168.77 (761.36 to 1998.76) | 907.57 (585.59 to 1559.76) | 786.14 (503.25 to 1359.34) | 655.04 (417.81 to 1135.49) | 476.07 (303.16 to 825.70) | 279.79 (178.12 to 485.09) | 138.38 (88.10 to 240.02) | 57.44 (36.63 to 99.58) | 18.46 (11.75 to 32.03) | 4.90 (3.12 to 8.49) |
| Malawi | Male | Number of DALYs | 109.83 (60.76 to 178.06) | 463.88 (206.25 to 997.96) | 472.09 (197.85 to 999.00) | 433.12 (186.93 to 932.58) | 330.84 (148.05 to 702.53) | 249.00 (109.60 to 544.88) | 217.35 (96.90 to 460.57) | 180.82 (78.73 to 380.48) | 152.23 (65.81 to 310.51) | 117.49 (53.56 to 249.31) | 84.09 (36.11 to 176.05) | 58.82 (26.52 to 124.78) | 46.41 (19.86 to 100.79) | 35.44 (15.17 to 74.67) | 23.06 (10.60 to 45.68) | 11.68 (5.16 to 24.48) | 4.94 (2.19 to 10.05) | 1.61 (0.72 to 3.23) | 0.39 (0.18 to 0.82) | 0.07 (0.03 to 0.16) |
| Malawi | Male | Number of prevalence | 1523.71 (1234.90 to 2033.56) | 5158.66 (3258.32 to 8974.67) | 5732.52 (3640.43 to 9840.92) | 5770.34 (3657.30 to 9869.96) | 4804.99 (3035.54 to 8274.43) | 3777.83 (2400.03 to 6473.29) | 3390.79 (2146.43 to 5788.35) | 2887.10 (1823.38 to 4902.62) | 2524.62 (1590.50 to 4255.75) | 2002.33 (1260.53 to 3356.34) | 1461.13 (920.94 to 2452.63) | 1047.98 (658.78 to 1761.63) | 849.26 (532.66 to 1455.50) | 665.06 (415.60 to 1116.35) | 439.32 (275.19 to 737.95) | 228.59 (143.03 to 383.96) | 97.69 (61.16 to 163.97) | 32.85 (20.57 to 55.15) | 8.06 (5.04 to 13.53) | 1.50 (0.94 to 2.52) |
| Malaysia | Both | Number of DALYs | 242.89 (144.81 to 380.93) | 736.33 (379.12 to 1336.93) | 771.93 (402.95 to 1353.77) | 817.83 (428.42 to 1491.31) | 912.80 (473.02 to 1767.81) | 984.86 (496.49 to 1831.79) | 974.90 (521.86 to 1766.06) | 883.40 (468.96 to 1604.90) | 729.47 (383.46 to 1315.15) | 596.71 (296.74 to 1117.39) | 528.37 (284.98 to 924.24) | 470.54 (251.55 to 845.00) | 398.17 (214.56 to 710.16) | 305.58 (164.15 to 548.33) | 207.00 (114.84 to 367.27) | 132.10 (71.91 to 234.81) | 64.75 (36.39 to 111.08) | 31.61 (16.67 to 55.87) | 11.58 (6.23 to 20.16) | 2.90 (1.53 to 5.35) |
| Malaysia | Both | Number of prevalence | 5471.06 (2660.31 to 16497.75) | 14390.71 (6496.29 to 43143.78) | 18454.06 (7153.79 to 60555.06) | 23271.99 (7976.86 to 81092.54) | 29274.97 (9350.73 to 105479.82) | 33575.39 (10281.86 to 123096.13) | 34720.80 (10328.54 to 128594.77) | 32793.22 (9578.58 to 122380.74) | 27836.92 (7979.38 to 104553.68) | 23786.68 (6694.74 to 89902.05) | 21748.38 (6051.61 to 82599.27) | 20021.67 (5493.20 to 76387.41) | 17656.74 (4761.98 to 67707.77) | 14102.41 (3765.34 to 54239.41) | 9682.21 (2591.15 to 37217.91) | 6343.40 (1692.51 to 24400.57) | 3186.51 (848.41 to 12270.72) | 1575.67 (422.22 to 6053.31) | 566.97 (154.13 to 2166.83) | 133.43 (37.28 to 503.24) |
| Malaysia | Female | Number of DALYs | 138.12 (81.13 to 215.53) | 239.70 (136.16 to 378.05) | 243.66 (141.15 to 377.64) | 250.04 (140.80 to 388.43) | 266.41 (149.96 to 403.95) | 274.09 (162.95 to 427.41) | 260.88 (154.96 to 399.58) | 231.41 (140.27 to 347.23) | 195.84 (113.97 to 303.91) | 160.38 (95.40 to 238.44) | 146.95 (85.96 to 224.09) | 133.19 (78.13 to 200.85) | 113.73 (66.11 to 172.09) | 88.61 (52.51 to 131.25) | 60.28 (37.73 to 89.93) | 39.07 (23.69 to 59.33) | 19.84 (11.91 to 29.36) | 9.01 (5.21 to 13.34) | 2.82 (1.83 to 4.24) | 0.44 (0.27 to 0.65) |
| Malaysia | Female | Number of prevalence | 2851.20 (1489.42 to 8189.81) | 6092.58 (2675.01 to 19519.28) | 7977.75 (2882.47 to 27889.65) | 10222.79 (3157.69 to 37837.69) | 12835.50 (3554.69 to 49037.55) | 14461.45 (3741.56 to 56196.58) | 14692.10 (3624.34 to 57680.45) | 13850.46 (3313.14 to 54779.72) | 11945.16 (2785.31 to 47504.37) | 10476.21 (2395.33 to 41853.64) | 9779.70 (2190.96 to 39224.57) | 9135.07 (2009.73 to 36778.64) | 8191.14 (1769.33 to 33111.47) | 6625.69 (1418.03 to 26834.52) | 4551.73 (977.12 to 18433.80) | 3021.04 (649.03 to 12234.79) | 1554.77 (333.69 to 6296.87) | 727.92 (155.95 to 2948.38) | 232.45 (49.78 to 941.47) | 37.68 (8.09 to 152.59) |
| Malaysia | Male | Number of DALYs | 104.78 (55.02 to 171.57) | 496.62 (213.94 to 1039.11) | 528.27 (237.83 to 1068.84) | 567.79 (248.57 to 1175.50) | 646.40 (272.17 to 1415.82) | 710.78 (303.25 to 1440.51) | 714.02 (318.01 to 1395.97) | 651.99 (291.91 to 1323.04) | 533.63 (234.39 to 1099.94) | 436.33 (194.98 to 911.35) | 381.42 (178.22 to 780.33) | 337.35 (148.36 to 683.46) | 284.44 (126.24 to 568.27) | 216.97 (96.18 to 438.88) | 146.71 (67.86 to 300.81) | 93.03 (42.67 to 187.85) | 44.91 (21.23 to 87.63) | 22.60 (10.11 to 45.09) | 8.76 (4.06 to 17.34) | 2.46 (1.18 to 4.94) |
| Malaysia | Male | Number of prevalence | 2619.86 (1150.20 to 8307.94) | 8298.14 (3675.64 to 22766.54) | 10476.31 (4100.63 to 32219.04) | 13049.20 (4623.04 to 43317.28) | 16439.47 (5478.18 to 56495.61) | 19113.95 (6126.34 to 66941.90) | 20028.70 (6251.26 to 70945.89) | 18942.76 (5841.84 to 67620.56) | 15891.76 (4835.28 to 57059.16) | 13310.48 (4001.62 to 48055.19) | 11968.69 (3568.07 to 43374.69) | 10886.60 (3212.75 to 39608.77) | 9465.59 (2766.42 to 34596.30) | 7476.72 (2178.65 to 27404.89) | 5130.48 (1495.91 to 18784.10) | 3322.35 (965.66 to 12165.79) | 1631.74 (475.14 to 5973.85) | 847.75 (246.77 to 3104.92) | 334.52 (97.43 to 1225.36) | 95.75 (27.88 to 350.65) |
| Maldives | Both | Number of DALYs | 3.13 (1.92 to 4.99) | 9.47 (5.08 to 17.26) | 9.08 (4.73 to 16.26) | 8.34 (4.37 to 15.50) | 10.41 (5.58 to 19.82) | 16.99 (8.58 to 32.03) | 22.46 (11.30 to 42.64) | 20.25 (9.99 to 38.84) | 14.07 (7.35 to 26.06) | 9.58 (4.91 to 18.74) | 6.94 (3.69 to 13.52) | 5.23 (2.74 to 9.33) | 3.71 (2.07 to 6.66) | 2.29 (1.22 to 4.13) | 1.28 (0.69 to 2.41) | 0.92 (0.50 to 1.59) | 0.69 (0.39 to 1.24) | 0.40 (0.22 to 0.69) | 0.13 (0.07 to 0.24) | 0.03 (0.02 to 0.05) |
| Maldives | Both | Number of prevalence | 38.82 (28.92 to 67.12) | 107.01 (64.08 to 189.84) | 113.18 (62.13 to 226.49) | 113.07 (56.40 to 248.83) | 149.48 (68.39 to 348.05) | 247.16 (105.35 to 590.14) | 336.86 (137.89 to 819.87) | 314.78 (126.24 to 778.45) | 224.21 (88.94 to 564.57) | 156.48 (61.69 to 399.19) | 118.66 (46.58 to 306.97) | 92.71 (36.15 to 243.48) | 68.64 (26.35 to 182.88) | 43.13 (16.47 to 115.44) | 24.95 (9.52 to 66.91) | 18.21 (6.97 to 48.87) | 14.21 (5.44 to 38.15) | 8.09 (3.09 to 21.68) | 2.72 (1.04 to 7.28) | 0.63 (0.24 to 1.68) |
| Maldives | Female | Number of DALYs | 1.79 (1.04 to 2.85) | 3.21 (1.84 to 5.37) | 3.06 (1.71 to 4.92) | 2.64 (1.56 to 4.11) | 2.70 (1.58 to 4.16) | 3.37 (2.04 to 5.29) | 3.94 (2.33 to 6.06) | 3.66 (2.13 to 5.63) | 2.82 (1.63 to 4.44) | 2.14 (1.29 to 3.42) | 1.75 (0.96 to 2.70) | 1.47 (0.82 to 2.23) | 1.14 (0.67 to 1.83) | 0.71 (0.43 to 1.09) | 0.40 (0.25 to 0.62) | 0.30 (0.18 to 0.45) | 0.23 (0.13 to 0.36) | 0.13 (0.07 to 0.19) | 0.04 (0.02 to 0.06) | 0.01 (0.01 to 0.01) |
| Maldives | Female | Number of prevalence | 21.48 (16.26 to 35.83) | 41.58 (28.96 to 77.99) | 44.61 (27.85 to 94.78) | 43.39 (24.26 to 102.34) | 49.23 (25.23 to 124.31) | 65.42 (31.78 to 171.88) | 79.53 (37.18 to 214.17) | 76.80 (34.90 to 210.31) | 60.37 (26.73 to 167.77) | 47.11 (20.43 to 132.45) | 39.93 (16.98 to 113.44) | 34.27 (14.29 to 98.44) | 27.16 (11.10 to 78.82) | 17.41 (7.04 to 50.81) | 10.13 (4.10 to 29.55) | 7.68 (3.10 to 22.41) | 6.02 (2.44 to 17.56) | 3.33 (1.35 to 9.73) | 1.07 (0.43 to 3.12) | 0.26 (0.10 to 0.74) |
| Maldives | Male | Number of DALYs | 1.34 (0.71 to 2.16) | 6.26 (2.77 to 13.59) | 6.02 (2.66 to 12.36) | 5.69 (2.50 to 11.98) | 7.71 (3.33 to 16.17) | 13.61 (6.15 to 27.90) | 18.52 (8.32 to 37.76) | 16.59 (7.04 to 35.14) | 11.25 (5.17 to 22.59) | 7.44 (3.37 to 16.25) | 5.19 (2.40 to 10.96) | 3.76 (1.61 to 7.63) | 2.57 (1.18 to 5.24) | 1.57 (0.73 to 3.34) | 0.88 (0.38 to 1.89) | 0.62 (0.25 to 1.27) | 0.46 (0.22 to 0.97) | 0.27 (0.13 to 0.54) | 0.09 (0.04 to 0.19) | 0.02 (0.01 to 0.04) |
| Maldives | Male | Number of prevalence | 17.33 (12.06 to 31.42) | 65.43 (33.32 to 132.14) | 68.57 (32.46 to 139.75) | 69.67 (30.56 to 144.51) | 100.25 (41.33 to 221.92) | 181.75 (71.82 to 418.31) | 257.33 (99.18 to 606.60) | 237.98 (89.83 to 571.52) | 163.84 (60.65 to 399.96) | 109.37 (39.87 to 269.28) | 78.73 (28.36 to 195.54) | 58.45 (20.70 to 146.71) | 41.48 (14.49 to 105.32) | 25.72 (8.90 to 65.45) | 14.82 (5.14 to 37.83) | 10.53 (3.65 to 26.81) | 8.19 (2.84 to 20.87) | 4.76 (1.65 to 12.11) | 1.65 (0.57 to 4.21) | 0.37 (0.13 to 0.95) |
| Mali | Both | Number of DALYs | 423.80 (267.09 to 623.52) | 1129.54 (611.24 to 2054.17) | 999.89 (520.74 to 1842.66) | 900.70 (473.33 to 1612.09) | 761.39 (407.67 to 1403.03) | 613.07 (329.96 to 1102.94) | 493.66 (266.23 to 906.69) | 404.08 (217.93 to 707.42) | 346.88 (193.69 to 614.45) | 288.92 (150.29 to 512.18) | 239.72 (129.46 to 411.65) | 192.78 (104.84 to 348.07) | 151.66 (81.52 to 266.27) | 111.12 (59.76 to 194.83) | 74.50 (40.11 to 133.32) | 42.96 (22.99 to 73.92) | 22.41 (12.41 to 39.75) | 8.30 (4.65 to 14.20) | 1.58 (0.87 to 2.80) | 0.24 (0.13 to 0.42) |
| Mali | Both | Number of prevalence | 13025.49 (5501.57 to 37176.18) | 29158.06 (11878.94 to 81883.65) | 31975.33 (11545.81 to 95861.87) | 34124.42 (11055.09 to 106311.14) | 32762.42 (10027.11 to 104277.54) | 28599.28 (8449.45 to 92201.26) | 24102.03 (6974.83 to 78310.68) | 20555.33 (5864.69 to 67079.99) | 17757.90 (5018.28 to 58027.66) | 14879.29 (4187.93 to 48626.57) | 12498.17 (3498.53 to 40902.46) | 10235.08 (2849.31 to 33567.99) | 8296.29 (2291.56 to 27265.48) | 6235.92 (1716.61 to 20516.78) | 4259.56 (1171.99 to 14013.84) | 2524.48 (694.42 to 8306.17) | 1331.72 (366.01 to 4383.36) | 497.12 (136.89 to 1635.13) | 95.04 (26.23 to 312.09) | 15.29 (4.21 to 50.30) |
| Mali | Female | Number of DALYs | 243.23 (142.89 to 371.88) | 360.17 (209.90 to 554.81) | 302.22 (174.14 to 465.23) | 266.10 (155.92 to 412.32) | 221.71 (126.83 to 343.61) | 179.72 (108.88 to 277.35) | 145.76 (83.03 to 228.05) | 116.29 (68.01 to 174.40) | 92.99 (58.20 to 140.23) | 71.32 (39.43 to 110.08) | 56.67 (32.33 to 87.80) | 45.37 (26.68 to 68.40) | 34.58 (20.88 to 51.76) | 25.15 (15.18 to 38.23) | 17.02 (10.66 to 25.61) | 9.97 (5.80 to 14.74) | 5.18 (3.16 to 7.55) | 1.91 (1.19 to 2.74) | 0.34 (0.22 to 0.51) | 0.05 (0.03 to 0.08) |
| Mali | Female | Number of prevalence | 6762.17 (3043.10 to 18633.04) | 12853.38 (4939.15 to 38302.13) | 14346.22 (4721.61 to 45348.93) | 15685.49 (4555.70 to 51472.78) | 15528.29 (4193.85 to 51993.80) | 14057.88 (3644.80 to 47582.93) | 12114.27 (3055.87 to 41273.74) | 10279.08 (2544.04 to 35178.89) | 8517.03 (2079.78 to 29249.13) | 6778.58 (1634.81 to 23344.47) | 5562.19 (1327.89 to 19201.24) | 4541.55 (1072.62 to 15714.50) | 3675.09 (858.28 to 12748.43) | 2762.42 (642.14 to 9592.27) | 1890.27 (439.22 to 6563.81) | 1124.27 (260.91 to 3903.90) | 596.64 (138.49 to 2071.75) | 219.86 (51.05 to 763.50) | 40.53 (9.41 to 140.73) | 6.76 (1.57 to 23.46) |
| Mali | Male | Number of DALYs | 180.57 (103.40 to 294.61) | 769.36 (339.44 to 1609.84) | 697.67 (306.13 to 1424.29) | 634.59 (278.90 to 1326.93) | 539.68 (219.08 to 1100.83) | 433.35 (200.24 to 869.26) | 347.90 (153.09 to 687.37) | 287.79 (129.12 to 554.67) | 253.89 (117.65 to 501.41) | 217.60 (95.39 to 422.39) | 183.06 (87.31 to 345.09) | 147.42 (67.97 to 297.48) | 117.08 (55.15 to 221.77) | 85.96 (38.56 to 162.40) | 57.48 (27.00 to 115.15) | 32.98 (15.68 to 64.38) | 17.23 (8.16 to 32.67) | 6.39 (3.07 to 12.11) | 1.24 (0.61 to 2.41) | 0.19 (0.09 to 0.35) |
| Mali | Male | Number of prevalence | 6263.32 (2431.51 to 18543.14) | 16304.68 (6704.51 to 42992.91) | 17629.11 (6635.65 to 50053.22) | 18438.94 (6310.23 to 54773.90) | 17234.14 (5594.55 to 52453.43) | 14541.40 (4578.32 to 44831.42) | 11987.75 (3695.49 to 37216.70) | 10276.25 (3133.71 to 32067.45) | 9240.87 (2788.26 to 28937.31) | 8100.70 (2429.72 to 25425.17) | 6935.99 (2069.61 to 21821.30) | 5693.54 (1696.03 to 17941.99) | 4621.20 (1370.66 to 14593.66) | 3473.51 (1028.68 to 10985.01) | 2369.29 (701.64 to 7490.62) | 1400.21 (414.75 to 4425.43) | 735.08 (217.66 to 2323.96) | 277.26 (82.19 to 875.95) | 54.52 (16.15 to 172.26) | 8.53 (2.53 to 26.97) |
| Marshall Islands | Both | Number of DALYs | 0.54 (0.33 to 0.79) | 0.93 (0.55 to 1.38) | 0.94 (0.58 to 1.45) | 0.89 (0.55 to 1.39) | 0.79 (0.48 to 1.20) | 0.70 (0.42 to 1.02) | 0.67 (0.43 to 1.05) | 0.65 (0.41 to 0.99) | 0.57 (0.34 to 0.87) | 0.45 (0.28 to 0.66) | 0.37 (0.23 to 0.57) | 0.30 (0.19 to 0.44) | 0.23 (0.14 to 0.34) | 0.16 (0.10 to 0.24) | 0.10 (0.06 to 0.14) | 0.04 (0.03 to 0.06) | 0.02 (0.01 to 0.02) | 0.01 (0.00 to 0.01) | 0.00 (0.00 to 0.00) | 0.00 (0.00 to 0.00) |
| Marshall Islands | Both | Number of prevalence | 5.32 (4.99 to 5.63) | 9.14 (8.55 to 9.70) | 9.32 (8.72 to 9.97) | 8.85 (8.26 to 9.49) | 7.94 (7.40 to 8.57) | 7.09 (6.59 to 7.68) | 6.85 (6.37 to 7.43) | 6.71 (6.23 to 7.33) | 5.96 (5.54 to 6.53) | 4.80 (4.47 to 5.27) | 3.93 (3.65 to 4.32) | 3.20 (2.97 to 3.53) | 2.53 (2.34 to 2.79) | 1.81 (1.68 to 2.00) | 1.07 (0.99 to 1.18) | 0.48 (0.45 to 0.53) | 0.19 (0.18 to 0.21) | 0.06 (0.06 to 0.07) | 0.01 (0.01 to 0.02) | 0.00 (0.00 to 0.00) |
| Marshall Islands | Female | Number of DALYs | 0.31 (0.18 to 0.49) | 0.53 (0.29 to 0.84) | 0.54 (0.30 to 0.82) | 0.52 (0.31 to 0.85) | 0.45 (0.24 to 0.71) | 0.40 (0.22 to 0.61) | 0.37 (0.23 to 0.57) | 0.36 (0.21 to 0.57) | 0.32 (0.19 to 0.48) | 0.26 (0.15 to 0.39) | 0.21 (0.13 to 0.33) | 0.17 (0.10 to 0.27) | 0.14 (0.08 to 0.21) | 0.09 (0.05 to 0.14) | 0.05 (0.03 to 0.08) | 0.02 (0.01 to 0.04) | 0.01 (0.01 to 0.01) | 0.00 (0.00 to 0.00) | 0.00 (0.00 to 0.00) | 0.00 (0.00 to 0.00) |
| Marshall Islands | Female | Number of prevalence | 3.03 (2.80 to 3.29) | 5.24 (4.85 to 5.69) | 5.37 (4.97 to 5.83) | 5.11 (4.71 to 5.59) | 4.56 (4.20 to 5.01) | 4.02 (3.69 to 4.43) | 3.83 (3.52 to 4.22) | 3.78 (3.46 to 4.18) | 3.41 (3.14 to 3.78) | 2.77 (2.54 to 3.07) | 2.29 (2.10 to 2.54) | 1.88 (1.72 to 2.09) | 1.49 (1.37 to 1.66) | 1.05 (0.96 to 1.17) | 0.59 (0.55 to 0.66) | 0.27 (0.25 to 0.30) | 0.11 (0.10 to 0.12) | 0.04 (0.03 to 0.04) | 0.01 (0.01 to 0.01) | 0.00 (0.00 to 0.00) |
| Marshall Islands | Male | Number of DALYs | 0.23 (0.13 to 0.37) | 0.40 (0.22 to 0.66) | 0.40 (0.23 to 0.65) | 0.38 (0.19 to 0.62) | 0.34 (0.18 to 0.56) | 0.31 (0.17 to 0.48) | 0.30 (0.15 to 0.50) | 0.29 (0.16 to 0.46) | 0.25 (0.13 to 0.41) | 0.19 (0.10 to 0.31) | 0.16 (0.08 to 0.26) | 0.12 (0.07 to 0.20) | 0.10 (0.05 to 0.16) | 0.07 (0.04 to 0.11) | 0.04 (0.02 to 0.07) | 0.02 (0.01 to 0.03) | 0.01 (0.00 to 0.01) | 0.00 (0.00 to 0.00) | 0.00 (0.00 to 0.00) | 0.00 (0.00 to 0.00) |
| Marshall Islands | Male | Number of prevalence | 2.29 (2.10 to 2.52) | 3.91 (3.57 to 4.29) | 3.95 (3.60 to 4.37) | 3.74 (3.39 to 4.16) | 3.37 (3.05 to 3.79) | 3.07 (2.77 to 3.48) | 3.02 (2.72 to 3.43) | 2.94 (2.65 to 3.34) | 2.55 (2.30 to 2.90) | 2.03 (1.83 to 2.32) | 1.65 (1.48 to 1.89) | 1.32 (1.19 to 1.52) | 1.04 (0.93 to 1.20) | 0.76 (0.69 to 0.89) | 0.48 (0.43 to 0.55) | 0.21 (0.19 to 0.24) | 0.08 (0.07 to 0.09) | 0.03 (0.02 to 0.03) | 0.01 (0.01 to 0.01) | 0.00 (0.00 to 0.00) |
| Micronesia (Federated States of) | Both | Number of DALYs | 0.96 (0.60 to 1.45) | 3.34 (1.78 to 6.04) | 3.91 (2.06 to 6.92) | 4.37 (2.31 to 7.95) | 4.27 (2.24 to 7.82) | 3.96 (2.10 to 7.14) | 3.58 (1.88 to 6.35) | 3.31 (1.81 to 5.82) | 3.11 (1.68 to 5.31) | 2.98 (1.60 to 5.31) | 2.68 (1.43 to 4.73) | 2.42 (1.29 to 4.19) | 2.27 (1.25 to 3.95) | 1.26 (0.71 to 2.18) | 0.69 (0.39 to 1.20) | 0.28 (0.17 to 0.46) | 0.16 (0.10 to 0.27) | 0.07 (0.04 to 0.12) | 0.02 (0.01 to 0.03) | 0.00 (0.00 to 0.01) |
| Micronesia (Federated States of) | Both | Number of prevalence | 39.10 (9.90 to 249.76) | 107.91 (27.36 to 654.61) | 156.07 (32.92 to 1006.52) | 201.98 (37.45 to 1349.88) | 214.38 (37.13 to 1457.96) | 210.43 (35.08 to 1443.21) | 195.61 (31.91 to 1347.43) | 184.47 (29.71 to 1274.52) | 176.44 (28.18 to 1221.87) | 172.31 (27.15 to 1195.81) | 160.22 (24.90 to 1115.29) | 146.16 (22.56 to 1018.65) | 138.69 (21.46 to 966.63) | 80.42 (12.23 to 562.76) | 46.61 (6.89 to 327.99) | 20.88 (2.99 to 147.99) | 12.65 (1.77 to 90.26) | 5.97 (0.83 to 42.71) | 1.70 (0.24 to 12.13) | 0.34 (0.05 to 2.41) |
| Micronesia (Federated States of) | Female | Number of DALYs | 0.54 (0.32 to 0.85) | 0.98 (0.55 to 1.46) | 1.08 (0.62 to 1.65) | 1.11 (0.64 to 1.77) | 1.00 (0.57 to 1.57) | 0.88 (0.53 to 1.39) | 0.76 (0.43 to 1.16) | 0.68 (0.41 to 1.04) | 0.62 (0.38 to 0.95) | 0.60 (0.37 to 0.91) | 0.55 (0.31 to 0.85) | 0.48 (0.29 to 0.73) | 0.44 (0.26 to 0.68) | 0.26 (0.16 to 0.37) | 0.16 (0.10 to 0.24) | 0.08 (0.05 to 0.11) | 0.05 (0.03 to 0.07) | 0.02 (0.01 to 0.03) | 0.01 (0.00 to 0.01) | 0.00 (0.00 to 0.00) |
| Micronesia (Federated States of) | Female | Number of prevalence | 19.70 (5.58 to 121.46) | 47.57 (10.28 to 315.73) | 69.40 (11.67 to 485.49) | 90.21 (12.29 to 651.52) | 95.86 (11.40 to 704.00) | 93.88 (10.35 to 695.78) | 86.88 (9.07 to 647.34) | 81.96 (8.25 to 612.91) | 78.65 (7.72 to 589.78) | 77.46 (7.41 to 582.15) | 73.85 (6.91 to 556.09) | 67.38 (6.17 to 508.39) | 62.99 (5.66 to 476.18) | 38.38 (3.40 to 290.40) | 23.92 (2.12 to 181.02) | 11.71 (1.04 to 88.62) | 7.63 (0.68 to 57.70) | 3.67 (0.33 to 27.81) | 1.01 (0.09 to 7.67) | 0.20 (0.02 to 1.55) |
| Micronesia (Federated States of) | Male | Number of DALYs | 0.42 (0.23 to 0.65) | 2.36 (1.06 to 4.85) | 2.83 (1.26 to 5.63) | 3.26 (1.46 to 6.75) | 3.27 (1.45 to 6.62) | 3.08 (1.40 to 5.99) | 2.82 (1.31 to 5.35) | 2.63 (1.25 to 5.03) | 2.48 (1.20 to 4.63) | 2.38 (1.11 to 4.50) | 2.13 (1.03 to 3.90) | 1.93 (0.94 to 3.63) | 1.83 (0.91 to 3.36) | 1.00 (0.50 to 1.88) | 0.53 (0.26 to 0.98) | 0.21 (0.11 to 0.38) | 0.11 (0.06 to 0.21) | 0.05 (0.03 to 0.09) | 0.01 (0.01 to 0.03) | 0.00 (0.00 to 0.01) |
| Micronesia (Federated States of) | Male | Number of prevalence | 19.40 (4.31 to 128.29) | 60.35 (16.07 to 338.88) | 86.67 (20.16 to 521.03) | 111.77 (23.24 to 698.36) | 118.52 (23.58 to 753.96) | 116.55 (22.61 to 747.43) | 108.73 (20.82 to 700.09) | 102.51 (19.48 to 661.61) | 97.80 (18.58 to 632.09) | 94.85 (17.99 to 613.66) | 86.38 (16.39 to 559.20) | 78.78 (14.99 to 510.25) | 75.70 (14.50 to 490.45) | 42.03 (8.06 to 272.35) | 22.68 (4.34 to 146.96) | 9.16 (1.75 to 59.37) | 5.03 (0.96 to 32.57) | 2.30 (0.44 to 14.90) | 0.69 (0.13 to 4.45) | 0.13 (0.03 to 0.86) |
| Mozambique | Both | Number of DALYs | 503.75 (311.17 to 754.67) | 1385.59 (767.22 to 2601.74) | 1217.23 (638.72 to 2157.18) | 1033.62 (554.55 to 1837.59) | 873.34 (479.96 to 1603.59) | 724.16 (398.30 to 1270.64) | 562.40 (299.76 to 1020.53) | 470.77 (263.59 to 809.37) | 392.43 (219.70 to 699.10) | 318.62 (171.68 to 593.64) | 237.95 (127.60 to 425.68) | 184.61 (101.96 to 318.78) | 143.69 (79.79 to 257.85) | 105.87 (60.05 to 188.81) | 66.30 (37.44 to 115.64) | 34.40 (19.86 to 58.05) | 15.54 (9.07 to 25.91) | 5.41 (3.11 to 8.58) | 1.40 (0.81 to 2.23) | 0.35 (0.20 to 0.53) |
| Mozambique | Both | Number of prevalence | 11018.13 (6430.55 to 22591.47) | 26889.80 (14977.74 to 54111.76) | 28989.68 (14683.99 to 62207.19) | 29747.75 (13729.20 to 67330.70) | 28655.66 (12295.84 to 67018.13) | 25736.86 (10636.82 to 61143.16) | 20939.71 (8501.27 to 50194.79) | 18246.17 (7297.52 to 44045.52) | 15739.35 (6236.56 to 38187.41) | 13270.29 (5199.55 to 32345.84) | 10272.30 (3997.34 to 25146.02) | 8122.18 (3133.56 to 19944.01) | 6440.70 (2465.58 to 15860.46) | 4966.01 (1890.63 to 12273.35) | 3234.86 (1226.65 to 8022.36) | 1759.04 (657.23 to 4382.19) | 845.82 (310.33 to 2118.07) | 314.06 (113.36 to 790.77) | 86.31 (31.01 to 218.26) | 22.87 (8.18 to 58.14) |
| Mozambique | Female | Number of DALYs | 292.01 (172.90 to 468.20) | 466.09 (271.54 to 717.72) | 405.16 (233.54 to 632.05) | 347.22 (207.38 to 539.76) | 301.06 (178.66 to 470.93) | 240.41 (142.57 to 362.07) | 180.46 (102.96 to 284.20) | 147.80 (87.66 to 227.93) | 123.30 (76.06 to 185.95) | 100.86 (60.15 to 157.99) | 77.11 (45.46 to 119.18) | 58.31 (34.16 to 88.54) | 43.59 (26.25 to 66.07) | 34.12 (19.95 to 51.28) | 23.14 (13.83 to 37.12) | 13.59 (7.98 to 20.49) | 6.91 (4.06 to 10.76) | 2.72 (1.58 to 4.16) | 0.77 (0.45 to 1.13) | 0.22 (0.13 to 0.32) |
| Mozambique | Female | Number of prevalence | 5888.81 (3562.45 to 11541.91) | 11786.49 (6375.84 to 25217.28) | 13051.72 (6252.31 to 29945.02) | 14076.51 (6083.09 to 33891.44) | 14216.41 (5764.82 to 35139.68) | 12757.29 (4986.63 to 32004.16) | 10230.31 (3901.53 to 25892.55) | 8923.45 (3341.85 to 22721.80) | 7675.86 (2840.57 to 19637.82) | 6537.22 (2388.66 to 16788.77) | 5090.11 (1840.09 to 13118.81) | 4006.57 (1438.35 to 10359.64) | 3128.66 (1110.07 to 8117.19) | 2499.70 (882.97 to 6495.12) | 1727.14 (609.32 to 4487.30) | 1019.33 (360.45 to 2648.55) | 534.53 (188.61 to 1388.70) | 214.78 (75.78 to 557.95) | 62.59 (22.07 to 162.65) | 17.85 (6.30 to 46.38) |
| Mozambique | Male | Number of DALYs | 211.74 (119.92 to 325.00) | 919.50 (429.60 to 1959.67) | 812.08 (360.87 to 1691.70) | 686.40 (299.83 to 1411.89) | 572.27 (249.31 to 1232.90) | 483.75 (223.26 to 971.07) | 381.94 (171.38 to 789.46) | 322.97 (152.37 to 637.52) | 269.13 (122.20 to 538.31) | 217.76 (95.21 to 466.98) | 160.84 (70.71 to 327.81) | 126.30 (57.02 to 253.05) | 100.11 (44.32 to 201.40) | 71.75 (32.27 to 146.81) | 43.16 (19.50 to 88.71) | 20.81 (9.75 to 42.62) | 8.63 (4.20 to 17.41) | 2.68 (1.25 to 5.39) | 0.63 (0.28 to 1.22) | 0.13 (0.06 to 0.26) |
| Mozambique | Male | Number of prevalence | 5129.32 (2844.27 to 11056.96) | 15103.31 (8010.36 to 29416.18) | 15937.95 (8122.69 to 33110.78) | 15671.24 (7609.43 to 34353.28) | 14439.24 (6733.04 to 32453.07) | 12979.57 (5900.59 to 29554.27) | 10709.40 (4782.14 to 24608.33) | 9322.72 (4124.93 to 21509.98) | 8063.49 (3542.13 to 18691.80) | 6733.06 (2941.73 to 15660.87) | 5182.19 (2249.70 to 12093.63) | 4115.60 (1776.04 to 9626.72) | 3312.05 (1419.82 to 7772.35) | 2466.31 (1052.93 to 5796.62) | 1507.71 (643.35 to 3545.60) | 739.70 (315.96 to 1738.89) | 311.29 (133.16 to 731.90) | 99.28 (42.43 to 233.54) | 23.72 (10.15 to 55.78) | 5.02 (2.14 to 11.80) |
| Myanmar | Both | Number of DALYs | 517.27 (310.64 to 771.81) | 1587.30 (844.51 to 2905.99) | 1674.48 (879.35 to 3041.49) | 1786.50 (926.91 to 3230.11) | 1796.52 (927.46 to 3312.29) | 1687.57 (926.38 to 3037.53) | 1612.62 (857.73 to 2888.36) | 1564.67 (828.77 to 2840.94) | 1489.32 (812.53 to 2676.24) | 1341.67 (743.44 to 2406.64) | 1209.46 (683.41 to 2101.18) | 1048.84 (570.69 to 1852.90) | 851.25 (465.85 to 1461.50) | 630.76 (350.63 to 1065.44) | 390.24 (219.28 to 678.91) | 226.48 (134.26 to 376.76) | 126.40 (72.63 to 210.18) | 59.86 (35.65 to 100.92) | 19.73 (11.54 to 32.34) | 3.88 (2.38 to 6.12) |
| Myanmar | Both | Number of prevalence | 16339.32 (5703.54 to 56348.38) | 41869.87 (13888.04 to 140419.64) | 55362.38 (15644.29 to 198454.81) | 70280.67 (17608.99 to 262732.11) | 79083.96 (18324.60 to 302344.43) | 80157.72 (17775.48 to 310149.00) | 79492.74 (17152.26 to 309646.56) | 79504.38 (16838.56 to 311119.50) | 77708.84 (16256.19 to 305243.96) | 72621.36 (14946.58 to 286278.49) | 67745.27 (13762.40 to 267925.66) | 60525.83 (12165.93 to 240127.65) | 50675.83 (10090.60 to 201654.22) | 39110.96 (7735.71 to 156018.73) | 24974.38 (4928.65 to 99743.59) | 15196.34 (2977.65 to 60852.19) | 8812.11 (1711.36 to 35366.94) | 4382.82 (838.69 to 17640.80) | 1535.01 (287.29 to 6207.03) | 340.23 (61.57 to 1387.40) |
| Myanmar | Female | Number of DALYs | 294.65 (167.74 to 448.95) | 502.15 (303.18 to 811.45) | 496.99 (288.90 to 800.26) | 517.37 (301.37 to 796.13) | 503.03 (293.58 to 801.14) | 470.74 (285.11 to 751.39) | 443.90 (267.00 to 685.77) | 420.02 (251.66 to 661.10) | 395.31 (224.48 to 623.48) | 358.21 (209.55 to 542.73) | 332.22 (195.73 to 509.19) | 290.77 (165.81 to 440.52) | 237.72 (131.27 to 371.66) | 182.84 (117.33 to 268.43) | 116.48 (72.09 to 178.42) | 71.78 (43.66 to 108.29) | 42.73 (25.38 to 64.54) | 21.50 (12.92 to 31.78) | 7.93 (4.83 to 12.19) | 1.90 (1.21 to 2.86) |
| Myanmar | Female | Number of prevalence | 8420.39 (3190.46 to 27993.52) | 18469.78 (5634.30 to 66535.07) | 24913.60 (6143.65 to 95247.43) | 32272.18 (6748.50 to 128046.48) | 37070.62 (7000.94 to 150040.84) | 38155.95 (6732.91 to 156113.33) | 38017.78 (6445.11 to 156554.87) | 38023.41 (6273.08 to 157281.05) | 37530.47 (6034.17 to 155774.18) | 35709.31 (5656.72 to 148607.10) | 33924.01 (5279.44 to 141521.71) | 30851.24 (4713.59 to 128989.67) | 26236.16 (3942.85 to 109959.92) | 20736.94 (3094.60 to 87011.43) | 13529.64 (2019.60 to 56769.78) | 8595.30 (1282.31 to 36068.15) | 5162.35 (770.10 to 21660.73) | 2684.91 (400.80 to 11265.24) | 1006.22 (149.76 to 4221.78) | 249.29 (37.28 to 1045.98) |
| Myanmar | Male | Number of DALYs | 222.62 (118.20 to 360.72) | 1085.15 (473.84 to 2362.61) | 1177.49 (511.54 to 2365.25) | 1269.13 (544.79 to 2643.44) | 1293.49 (596.85 to 2621.95) | 1216.83 (569.91 to 2335.99) | 1168.72 (509.27 to 2349.17) | 1144.65 (500.32 to 2274.86) | 1094.01 (506.77 to 2163.55) | 983.46 (449.64 to 1930.60) | 877.24 (406.48 to 1649.03) | 758.07 (348.70 to 1508.55) | 613.53 (289.34 to 1162.86) | 447.92 (206.94 to 866.55) | 273.77 (133.98 to 537.74) | 154.70 (75.49 to 298.27) | 83.67 (40.38 to 159.29) | 38.37 (19.19 to 74.76) | 11.81 (5.59 to 22.31) | 1.98 (0.92 to 3.86) |
| Myanmar | Male | Number of prevalence | 7918.93 (2522.10 to 28385.01) | 23400.09 (8090.69 to 73961.57) | 30448.78 (9183.40 to 103237.66) | 38008.49 (10224.33 to 134685.63) | 42013.35 (10628.83 to 152303.59) | 42001.78 (10343.47 to 154035.67) | 41474.96 (10033.38 to 153091.69) | 41480.97 (9963.22 to 153838.45) | 40178.36 (9563.25 to 149469.78) | 36912.05 (8752.08 to 137671.39) | 33821.26 (7969.31 to 126403.96) | 29674.59 (6935.56 to 111137.98) | 24439.67 (5663.66 to 91694.30) | 18374.03 (4250.59 to 69007.30) | 11444.73 (2647.48 to 42973.81) | 6601.03 (1525.19 to 24784.04) | 3649.76 (844.76 to 13706.21) | 1697.91 (392.78 to 6375.56) | 528.79 (122.28 to 1985.25) | 90.95 (21.02 to 341.42) |
| Nepal | Both | Number of DALYs | 311.56 (191.44 to 473.83) | 1014.84 (525.61 to 1897.98) | 1139.32 (601.53 to 2055.08) | 1337.12 (702.32 to 2250.48) | 1408.40 (766.70 to 2497.18) | 1274.44 (699.72 to 2240.21) | 1111.17 (612.11 to 1919.31) | 986.53 (535.29 to 1717.25) | 903.05 (496.32 to 1557.88) | 817.88 (443.40 to 1425.63) | 735.42 (390.85 to 1262.62) | 651.83 (366.05 to 1116.15) | 561.00 (312.59 to 971.73) | 448.92 (257.75 to 746.21) | 320.44 (177.97 to 549.95) | 198.07 (112.19 to 340.19) | 101.27 (54.63 to 171.92) | 39.96 (22.94 to 66.58) | 11.51 (6.62 to 18.77) | 2.65 (1.56 to 4.42) |
| Nepal | Both | Number of prevalence | 13748.88 (11080.05 to 17933.13) | 34930.13 (27184.73 to 47410.91) | 48652.49 (37746.62 to 66160.55) | 66515.05 (51404.79 to 90043.85) | 77355.59 (59872.39 to 104816.30) | 75524.58 (58503.79 to 102448.00) | 68869.87 (53390.73 to 93468.79) | 62966.89 (48799.25 to 85470.81) | 57518.08 (44554.81 to 78110.82) | 51779.66 (40093.29 to 70319.56) | 46629.95 (36097.90 to 63307.36) | 41343.27 (31971.29 to 56129.09) | 36171.51 (27986.81 to 49110.80) | 29548.34 (22869.36 to 40116.12) | 21520.70 (16649.52 to 29220.98) | 13471.18 (10426.18 to 18289.96) | 7003.98 (5419.69 to 9507.72) | 2840.97 (2198.68 to 3857.07) | 859.10 (665.03 to 1166.81) | 216.86 (167.96 to 294.67) |
| Nepal | Female | Number of DALYs | 178.62 (96.64 to 277.30) | 293.87 (177.69 to 446.29) | 313.16 (167.88 to 505.20) | 342.20 (209.47 to 530.77) | 360.76 (218.50 to 553.63) | 330.18 (206.85 to 490.33) | 292.76 (176.71 to 464.49) | 257.38 (156.39 to 403.22) | 220.02 (130.53 to 352.60) | 183.55 (113.96 to 285.41) | 151.75 (96.13 to 227.06) | 127.81 (79.66 to 192.52) | 110.31 (66.23 to 166.46) | 87.59 (53.01 to 132.96) | 62.50 (39.16 to 94.20) | 38.26 (23.82 to 57.35) | 19.84 (12.15 to 30.10) | 8.14 (5.17 to 11.87) | 2.59 (1.63 to 4.06) | 0.70 (0.41 to 1.03) |
| Nepal | Female | Number of prevalence | 6914.12 (5621.06 to 8953.53) | 15501.14 (12359.22 to 20439.73) | 21957.28 (17242.94 to 29359.33) | 31004.59 (24167.00 to 41814.69) | 37941.20 (29420.56 to 51388.76) | 38771.08 (29979.79 to 52630.12) | 36564.02 (28224.84 to 49699.95) | 33758.31 (26037.62 to 45938.87) | 30141.49 (23228.32 to 41048.29) | 26142.28 (20138.77 to 35625.52) | 22602.76 (17397.63 to 30815.88) | 19664.94 (15128.26 to 26824.28) | 17344.98 (13332.72 to 23667.91) | 14292.90 (10984.55 to 19510.79) | 10422.13 (8008.76 to 14227.30) | 6489.29 (4986.50 to 8859.17) | 3382.33 (2599.67 to 4616.88) | 1402.99 (1078.35 to 1915.10) | 450.68 (346.47 to 615.20) | 125.49 (96.43 to 171.32) |
| Nepal | Male | Number of DALYs | 132.94 (78.05 to 213.63) | 720.97 (316.90 to 1523.62) | 826.16 (366.71 to 1676.47) | 994.92 (440.49 to 1875.10) | 1047.64 (492.03 to 2047.39) | 944.26 (444.02 to 1797.31) | 818.41 (400.17 to 1542.49) | 729.15 (342.19 to 1420.55) | 683.03 (336.22 to 1290.50) | 634.33 (310.67 to 1201.07) | 583.68 (278.99 to 1083.46) | 524.02 (260.44 to 963.23) | 450.69 (222.79 to 828.28) | 361.33 (187.70 to 663.37) | 257.95 (135.18 to 455.58) | 159.81 (83.48 to 288.06) | 81.42 (39.49 to 147.50) | 31.83 (16.65 to 57.31) | 8.92 (4.76 to 15.87) | 1.96 (1.02 to 3.45) |
| Nepal | Male | Number of prevalence | 6834.76 (5451.43 to 8979.60) | 19428.98 (14497.54 to 27305.82) | 26695.21 (20022.01 to 36923.64) | 35510.46 (26765.21 to 49176.52) | 39414.39 (29747.59 to 54433.46) | 36753.50 (27769.12 to 50622.14) | 32305.85 (24412.90 to 44313.58) | 29208.58 (22073.60 to 40016.85) | 27376.59 (20711.56 to 37528.68) | 25637.38 (19393.51 to 35113.90) | 24027.19 (18183.57 to 32884.89) | 21678.32 (16422.61 to 29620.95) | 18826.53 (14264.77 to 25690.17) | 15255.44 (11575.24 to 20812.75) | 11098.57 (8419.13 to 15146.48) | 6981.88 (5296.75 to 9528.10) | 3621.65 (2748.09 to 4940.81) | 1437.98 (1091.07 to 1961.35) | 408.43 (309.66 to 557.01) | 91.37 (69.33 to 124.63) |
| Niger | Both | Number of DALYs | 473.29 (284.66 to 711.35) | 1255.60 (653.10 to 2210.57) | 1088.50 (585.93 to 2094.58) | 945.28 (515.17 to 1702.66) | 766.16 (408.90 to 1373.94) | 616.16 (322.97 to 1131.44) | 475.79 (249.98 to 871.02) | 395.99 (211.64 to 720.38) | 320.65 (181.23 to 576.73) | 241.29 (134.53 to 434.99) | 199.43 (105.05 to 349.59) | 188.10 (100.66 to 347.69) | 149.95 (82.35 to 267.14) | 95.27 (51.53 to 166.96) | 66.25 (36.26 to 115.58) | 42.54 (23.73 to 74.72) | 16.33 (9.28 to 28.48) | 5.59 (3.07 to 9.40) | 2.11 (1.18 to 3.58) | 0.40 (0.23 to 0.69) |
| Niger | Both | Number of prevalence | 14229.64 (8004.98 to 31153.96) | 31836.06 (17768.27 to 68733.93) | 34016.88 (18002.66 to 78280.42) | 35532.21 (17792.83 to 85096.93) | 32238.91 (15752.04 to 78449.64) | 27820.87 (13394.42 to 68220.79) | 22361.74 (10677.70 to 55082.10) | 19191.55 (9102.42 to 47434.86) | 16218.99 (7632.47 to 40234.18) | 12818.68 (6012.22 to 31908.45) | 10746.39 (5030.65 to 26776.04) | 10106.69 (4727.34 to 25188.19) | 8133.36 (3796.46 to 20278.23) | 5355.91 (2493.87 to 13372.37) | 3857.38 (1794.88 to 9640.57) | 2518.71 (1172.95 to 6293.36) | 983.49 (457.99 to 2458.49) | 340.68 (158.72 to 851.13) | 128.34 (59.83 to 320.27) | 25.92 (12.06 to 64.85) |
| Niger | Female | Number of DALYs | 271.25 (156.40 to 426.23) | 402.70 (222.14 to 634.53) | 338.54 (197.58 to 559.91) | 288.27 (162.41 to 442.34) | 227.27 (133.17 to 350.49) | 173.47 (97.13 to 280.33) | 126.46 (75.24 to 190.16) | 103.59 (63.15 to 158.92) | 88.81 (53.49 to 138.32) | 70.20 (40.57 to 106.60) | 55.64 (33.56 to 85.26) | 47.75 (28.49 to 71.10) | 36.22 (20.97 to 55.15) | 23.27 (14.18 to 35.70) | 17.11 (9.85 to 26.24) | 10.97 (6.64 to 16.56) | 4.22 (2.58 to 6.33) | 1.42 (0.89 to 2.15) | 0.50 (0.30 to 0.75) | 0.11 (0.07 to 0.16) |
| Niger | Female | Number of prevalence | 7374.35 (4292.23 to 15662.49) | 13985.64 (7435.79 to 31689.10) | 15382.95 (7567.90 to 36633.88) | 16701.29 (7747.42 to 41080.94) | 15346.42 (6910.96 to 38403.66) | 13104.42 (5793.14 to 33097.13) | 10355.52 (4524.02 to 26304.52) | 8946.15 (3881.53 to 22811.70) | 7915.70 (3412.13 to 20241.24) | 6543.44 (2808.47 to 16776.07) | 5358.03 (2290.01 to 13764.20) | 4781.68 (2034.00 to 12308.94) | 3705.55 (1570.27 to 9559.74) | 2497.98 (1056.01 to 6450.70) | 1862.36 (787.27 to 4808.57) | 1206.25 (510.38 to 3114.42) | 476.26 (201.27 to 1229.58) | 162.33 (68.63 to 419.16) | 58.57 (24.75 to 151.24) | 12.91 (5.45 to 33.32) |
| Niger | Male | Number of DALYs | 202.04 (108.37 to 332.57) | 852.90 (372.04 to 1767.38) | 749.97 (326.96 to 1645.94) | 657.01 (312.98 to 1335.55) | 538.89 (241.16 to 1060.69) | 442.68 (187.26 to 892.06) | 349.33 (159.49 to 713.51) | 292.40 (129.10 to 588.36) | 231.85 (106.64 to 459.02) | 171.09 (78.08 to 331.70) | 143.79 (60.33 to 282.12) | 140.35 (65.42 to 284.97) | 113.73 (53.03 to 222.78) | 72.00 (32.75 to 137.78) | 49.14 (22.72 to 95.67) | 31.58 (14.78 to 61.05) | 12.11 (5.98 to 23.23) | 4.17 (1.93 to 7.76) | 1.61 (0.76 to 2.99) | 0.29 (0.14 to 0.56) |
| Niger | Male | Number of prevalence | 6855.29 (3707.17 to 15491.47) | 17850.43 (9698.59 to 37674.91) | 18633.94 (9673.75 to 40282.48) | 18830.92 (9535.10 to 42618.11) | 16892.48 (8425.91 to 39167.71) | 14716.45 (7294.30 to 34561.59) | 12006.22 (5928.08 to 28412.00) | 10245.41 (5046.94 to 24365.23) | 8303.28 (4085.29 to 19821.83) | 6275.24 (3085.26 to 15022.99) | 5388.35 (2644.27 to 12936.17) | 5325.00 (2609.64 to 12816.78) | 4427.81 (2165.53 to 10683.60) | 2857.93 (1397.73 to 6901.51) | 1995.02 (975.64 to 4817.74) | 1312.46 (641.81 to 3169.58) | 507.22 (248.10 to 1225.28) | 178.35 (87.24 to 430.74) | 69.77 (34.09 to 168.50) | 13.02 (6.36 to 31.42) |
| Nigeria | Both | Number of DALYs | 3471.14 (2381.39 to 4911.73) | 9615.12 (5490.46 to 17185.08) | 9374.63 (5331.45 to 16866.43) | 8510.52 (4793.92 to 15200.29) | 7250.23 (4111.68 to 12884.54) | 6171.95 (3532.71 to 10800.10) | 5225.40 (2961.21 to 9268.83) | 4521.75 (2575.98 to 7887.42) | 3915.00 (2223.25 to 6775.20) | 3311.51 (1882.29 to 5767.12) | 2684.13 (1552.65 to 4628.23) | 2028.26 (1172.57 to 3454.86) | 1482.00 (867.14 to 2492.33) | 1018.94 (591.26 to 1716.61) | 700.92 (405.38 to 1197.93) | 444.77 (258.93 to 758.63) | 245.80 (141.05 to 420.23) | 107.70 (62.78 to 182.24) | 35.96 (21.17 to 60.48) | 8.67 (5.47 to 13.55) |
| Nigeria | Both | Number of prevalence | 102698.37 (52902.20 to 190866.63) | 243639.99 (123510.93 to 437524.22) | 291640.44 (137993.41 to 551732.52) | 318100.89 (139684.84 to 626854.65) | 305405.51 (128961.44 to 608975.25) | 280293.82 (115432.11 to 567994.66) | 246294.93 (101024.54 to 508594.76) | 218324.57 (89325.12 to 459017.09) | 190883.12 (77878.61 to 400130.56) | 165367.95 (67052.51 to 343006.79) | 139541.56 (55991.01 to 288880.38) | 110124.86 (43743.95 to 231268.80) | 84158.97 (33283.52 to 182809.81) | 59058.94 (23396.11 to 127521.53) | 40635.59 (16058.76 to 87495.04) | 25637.58 (10227.36 to 55373.30) | 14136.49 (5531.00 to 30786.74) | 6310.96 (2530.09 to 13744.40) | 2199.32 (869.53 to 5000.42) | 676.56 (251.06 to 1648.38) |
| Nigeria | Female | Number of DALYs | 2023.74 (1335.33 to 2941.00) | 3203.19 (2161.01 to 4612.75) | 3001.31 (2047.84 to 4275.75) | 2666.99 (1805.86 to 3852.00) | 2195.80 (1467.14 to 3104.75) | 1826.21 (1229.78 to 2556.67) | 1472.42 (1005.91 to 2123.07) | 1219.53 (833.43 to 1713.31) | 1008.81 (689.86 to 1401.51) | 852.43 (580.80 to 1204.92) | 723.44 (502.02 to 1015.61) | 572.45 (398.33 to 786.81) | 419.69 (286.84 to 582.78) | 270.32 (186.41 to 369.97) | 172.80 (119.52 to 237.46) | 105.20 (72.44 to 143.96) | 56.69 (38.40 to 78.98) | 24.36 (16.82 to 33.84) | 8.02 (5.52 to 11.04) | 3.08 (2.22 to 4.17) |
| Nigeria | Female | Number of prevalence | 53878.06 (29048.51 to 98132.05) | 109565.44 (52806.52 to 210734.38) | 135572.33 (59285.99 to 273510.65) | 153582.36 (62761.72 to 318589.31) | 151248.76 (59148.13 to 318464.97) | 140805.09 (53184.52 to 299277.33) | 122328.58 (45453.66 to 266505.03) | 106754.36 (39107.11 to 233477.19) | 91285.43 (33121.56 to 199148.71) | 79943.99 (28901.93 to 175976.43) | 70651.18 (25528.90 to 157224.52) | 58229.03 (21035.03 to 132386.16) | 45695.38 (16517.58 to 105434.35) | 31299.21 (11127.62 to 72380.58) | 20486.87 (7082.77 to 47807.40) | 12275.89 (4345.72 to 27453.16) | 6558.69 (2290.08 to 15005.07) | 2932.21 (1021.73 to 6877.78) | 1047.56 (358.29 to 2607.64) | 440.76 (143.85 to 1161.80) |
| Nigeria | Male | Number of DALYs | 1447.40 (982.38 to 2056.40) | 6411.93 (3155.62 to 12979.23) | 6373.32 (3050.29 to 12817.03) | 5843.53 (2825.66 to 11757.08) | 5054.43 (2423.94 to 10085.17) | 4345.74 (2114.48 to 8519.18) | 3752.97 (1871.35 to 7411.61) | 3302.21 (1637.47 to 6437.54) | 2906.19 (1448.38 to 5587.26) | 2459.08 (1220.10 to 4760.89) | 1960.69 (991.47 to 3656.21) | 1455.81 (727.52 to 2762.33) | 1062.31 (546.63 to 1977.73) | 748.62 (379.80 to 1384.94) | 528.12 (270.64 to 980.17) | 339.58 (175.27 to 625.25) | 189.12 (97.29 to 346.18) | 83.34 (43.48 to 151.99) | 27.93 (14.69 to 51.05) | 5.59 (3.00 to 9.82) |
| Nigeria | Male | Number of prevalence | 48820.31 (23188.06 to 93145.26) | 134074.55 (69913.76 to 231007.16) | 156068.11 (77482.31 to 288727.17) | 164518.53 (77127.23 to 310832.91) | 154156.75 (70406.38 to 291397.58) | 139488.73 (62578.52 to 268708.82) | 123966.35 (55550.88 to 243072.69) | 111570.21 (49920.69 to 225315.28) | 99597.69 (44495.13 to 200723.99) | 85423.96 (38170.70 to 168349.60) | 68890.38 (30675.97 to 133015.10) | 51895.84 (23227.07 to 99006.63) | 38463.59 (17239.18 to 74003.04) | 27759.73 (12385.57 to 53312.75) | 20148.72 (8900.23 to 38834.65) | 13361.69 (5801.97 to 27385.66) | 7577.80 (3234.12 to 15813.02) | 3378.75 (1493.13 to 6934.14) | 1151.76 (513.60 to 2392.68) | 235.80 (104.07 to 482.62) |
| Niue | Both | Number of DALYs | 0.01 (0.01 to 0.02) | 0.04 (0.02 to 0.07) | 0.05 (0.02 to 0.08) | 0.05 (0.02 to 0.09) | 0.04 (0.02 to 0.08) | 0.04 (0.02 to 0.07) | 0.04 (0.02 to 0.07) | 0.04 (0.02 to 0.07) | 0.04 (0.02 to 0.07) | 0.04 (0.02 to 0.08) | 0.04 (0.02 to 0.08) | 0.04 (0.02 to 0.08) | 0.04 (0.02 to 0.07) | 0.03 (0.01 to 0.04) | 0.02 (0.01 to 0.03) | 0.01 (0.01 to 0.02) | 0.01 (0.00 to 0.01) | 0.00 (0.00 to 0.01) | 0.00 (0.00 to 0.00) | 0.00 (0.00 to 0.00) |
| Niue | Both | Number of prevalence | 0.34 (0.14 to 0.99) | 0.96 (0.39 to 2.64) | 1.35 (0.48 to 3.98) | 1.73 (0.55 to 5.34) | 1.69 (0.50 to 5.39) | 1.73 (0.49 to 5.60) | 1.74 (0.49 to 5.68) | 1.83 (0.50 to 6.01) | 2.01 (0.55 to 6.62) | 2.12 (0.57 to 7.02) | 2.26 (0.61 to 7.49) | 2.31 (0.61 to 7.66) | 2.11 (0.56 to 7.00) | 1.46 (0.38 to 4.90) | 1.02 (0.26 to 3.43) | 0.71 (0.18 to 2.40) | 0.50 (0.13 to 1.71) | 0.26 (0.06 to 0.87) | 0.08 (0.02 to 0.28) | 0.02 (0.00 to 0.06) |
| Niue | Female | Number of DALYs | 0.01 (0.00 to 0.01) | 0.01 (0.01 to 0.02) | 0.01 (0.01 to 0.02) | 0.01 (0.01 to 0.02) | 0.01 (0.01 to 0.02) | 0.01 (0.01 to 0.02) | 0.01 (0.01 to 0.02) | 0.01 (0.01 to 0.02) | 0.01 (0.01 to 0.02) | 0.01 (0.01 to 0.02) | 0.01 (0.01 to 0.02) | 0.01 (0.01 to 0.02) | 0.01 (0.01 to 0.01) | 0.01 (0.00 to 0.01) | 0.01 (0.00 to 0.01) | 0.00 (0.00 to 0.01) | 0.00 (0.00 to 0.00) | 0.00 (0.00 to 0.00) | 0.00 (0.00 to 0.00) | 0.00 (0.00 to 0.00) |
| Niue | Female | Number of prevalence | 0.18 (0.08 to 0.49) | 0.42 (0.16 to 1.28) | 0.56 (0.18 to 1.80) | 0.70 (0.20 to 2.36) | 0.75 (0.20 to 2.58) | 0.81 (0.20 to 2.80) | 0.82 (0.20 to 2.84) | 0.85 (0.20 to 2.98) | 0.93 (0.22 to 3.27) | 0.97 (0.22 to 3.43) | 1.02 (0.23 to 3.60) | 1.02 (0.23 to 3.60) | 0.93 (0.21 to 3.30) | 0.72 (0.16 to 2.56) | 0.56 (0.12 to 2.00) | 0.42 (0.09 to 1.51) | 0.30 (0.07 to 1.07) | 0.15 (0.03 to 0.55) | 0.05 (0.01 to 0.19) | 0.01 (0.00 to 0.04) |
| Niue | Male | Number of DALYs | 0.01 (0.00 to 0.01) | 0.03 (0.01 to 0.06) | 0.03 (0.01 to 0.07) | 0.04 (0.02 to 0.08) | 0.03 (0.01 to 0.06) | 0.03 (0.01 to 0.06) | 0.03 (0.01 to 0.06) | 0.03 (0.01 to 0.05) | 0.03 (0.01 to 0.06) | 0.03 (0.01 to 0.06) | 0.03 (0.02 to 0.07) | 0.03 (0.01 to 0.07) | 0.03 (0.01 to 0.06) | 0.02 (0.01 to 0.04) | 0.01 (0.01 to 0.02) | 0.01 (0.00 to 0.01) | 0.00 (0.00 to 0.01) | 0.00 (0.00 to 0.00) | 0.00 (0.00 to 0.00) | 0.00 (0.00 to 0.00) |
| Niue | Male | Number of prevalence | 0.17 (0.06 to 0.50) | 0.54 (0.22 to 1.37) | 0.79 (0.29 to 2.18) | 1.02 (0.35 to 2.97) | 0.94 (0.30 to 2.81) | 0.92 (0.29 to 2.81) | 0.93 (0.28 to 2.84) | 0.98 (0.30 to 3.03) | 1.08 (0.32 to 3.36) | 1.15 (0.34 to 3.58) | 1.25 (0.37 to 3.90) | 1.29 (0.38 to 4.06) | 1.18 (0.34 to 3.71) | 0.74 (0.22 to 2.34) | 0.45 (0.13 to 1.43) | 0.28 (0.08 to 0.89) | 0.20 (0.06 to 0.64) | 0.10 (0.03 to 0.32) | 0.03 (0.01 to 0.09) | 0.00 (0.00 to 0.02) |
| Palau | Both | Number of DALYs | 0.09 (0.06 to 0.14) | 0.17 (0.10 to 0.28) | 0.19 (0.12 to 0.30) | 0.18 (0.11 to 0.28) | 0.18 (0.10 to 0.27) | 0.17 (0.11 to 0.25) | 0.18 (0.11 to 0.27) | 0.20 (0.12 to 0.29) | 0.23 (0.14 to 0.35) | 0.24 (0.15 to 0.36) | 0.24 (0.15 to 0.34) | 0.21 (0.13 to 0.32) | 0.16 (0.10 to 0.24) | 0.11 (0.07 to 0.16) | 0.07 (0.05 to 0.11) | 0.04 (0.02 to 0.05) | 0.01 (0.01 to 0.02) | 0.01 (0.00 to 0.01) | 0.00 (0.00 to 0.00) | 0.00 (0.00 to 0.00) |
| Palau | Both | Number of prevalence | 0.93 (0.88 to 0.99) | 1.77 (1.66 to 1.90) | 2.05 (1.91 to 2.22) | 1.99 (1.84 to 2.20) | 1.91 (1.75 to 2.14) | 1.89 (1.72 to 2.16) | 2.03 (1.84 to 2.33) | 2.32 (2.10 to 2.68) | 2.73 (2.46 to 3.15) | 2.96 (2.67 to 3.43) | 2.89 (2.60 to 3.36) | 2.64 (2.38 to 3.08) | 2.07 (1.85 to 2.42) | 1.39 (1.24 to 1.63) | 0.92 (0.82 to 1.08) | 0.48 (0.43 to 0.56) | 0.19 (0.17 to 0.22) | 0.09 (0.08 to 0.11) | 0.03 (0.02 to 0.03) | 0.00 (0.00 to 0.00) |
| Palau | Female | Number of DALYs | 0.05 (0.03 to 0.08) | 0.10 (0.06 to 0.16) | 0.11 (0.07 to 0.17) | 0.10 (0.06 to 0.16) | 0.09 (0.05 to 0.14) | 0.08 (0.04 to 0.11) | 0.08 (0.05 to 0.13) | 0.09 (0.05 to 0.15) | 0.11 (0.06 to 0.18) | 0.13 (0.07 to 0.19) | 0.13 (0.07 to 0.19) | 0.12 (0.07 to 0.19) | 0.09 (0.06 to 0.15) | 0.06 (0.04 to 0.09) | 0.04 (0.02 to 0.06) | 0.02 (0.01 to 0.03) | 0.01 (0.00 to 0.01) | 0.00 (0.00 to 0.01) | 0.00 (0.00 to 0.00) | 0.00 (0.00 to 0.00) |
| Palau | Female | Number of prevalence | 0.52 (0.49 to 0.57) | 1.00 (0.92 to 1.09) | 1.17 (1.08 to 1.29) | 1.11 (1.02 to 1.23) | 0.96 (0.87 to 1.07) | 0.84 (0.76 to 0.94) | 0.90 (0.82 to 1.01) | 1.07 (0.96 to 1.21) | 1.32 (1.19 to 1.50) | 1.51 (1.35 to 1.72) | 1.54 (1.38 to 1.76) | 1.48 (1.33 to 1.70) | 1.18 (1.06 to 1.36) | 0.81 (0.72 to 0.93) | 0.52 (0.46 to 0.60) | 0.27 (0.24 to 0.31) | 0.11 (0.09 to 0.12) | 0.05 (0.04 to 0.05) | 0.01 (0.01 to 0.01) | 0.00 (0.00 to 0.00) |
| Palau | Male | Number of DALYs | 0.04 (0.02 to 0.06) | 0.08 (0.04 to 0.12) | 0.08 (0.04 to 0.14) | 0.08 (0.04 to 0.14) | 0.09 (0.04 to 0.15) | 0.09 (0.05 to 0.15) | 0.10 (0.05 to 0.16) | 0.10 (0.06 to 0.16) | 0.12 (0.06 to 0.19) | 0.12 (0.06 to 0.20) | 0.11 (0.06 to 0.17) | 0.09 (0.05 to 0.14) | 0.07 (0.04 to 0.11) | 0.04 (0.02 to 0.07) | 0.03 (0.02 to 0.05) | 0.02 (0.01 to 0.02) | 0.01 (0.00 to 0.01) | 0.00 (0.00 to 0.01) | 0.00 (0.00 to 0.00) | 0.00 (0.00 to 0.00) |
| Palau | Male | Number of prevalence | 0.41 (0.37 to 0.45) | 0.77 (0.70 to 0.85) | 0.88 (0.79 to 0.98) | 0.88 (0.79 to 1.00) | 0.96 (0.86 to 1.11) | 1.05 (0.93 to 1.23) | 1.13 (1.00 to 1.33) | 1.25 (1.10 to 1.48) | 1.41 (1.24 to 1.66) | 1.45 (1.27 to 1.72) | 1.35 (1.18 to 1.61) | 1.16 (1.01 to 1.39) | 0.89 (0.77 to 1.07) | 0.58 (0.51 to 0.70) | 0.40 (0.35 to 0.48) | 0.20 (0.18 to 0.25) | 0.08 (0.07 to 0.10) | 0.04 (0.04 to 0.05) | 0.01 (0.01 to 0.02) | 0.00 (0.00 to 0.00) |
| Papua New Guinea | Both | Number of DALYs | 160.40 (99.71 to 232.22) | 769.52 (420.80 to 1344.32) | 989.41 (558.90 to 1666.22) | 1293.61 (761.59 to 2101.24) | 1650.46 (1041.79 to 2522.14) | 1812.88 (1154.93 to 2756.64) | 1738.71 (1115.23 to 2543.59) | 1645.93 (1076.62 to 2433.04) | 1506.70 (996.42 to 2159.71) | 1332.99 (872.66 to 1912.39) | 1136.96 (748.26 to 1627.16) | 928.17 (613.64 to 1329.53) | 683.76 (456.40 to 979.87) | 466.54 (311.44 to 662.97) | 284.71 (192.84 to 400.68) | 160.64 (109.00 to 225.63) | 70.77 (48.14 to 98.25) | 25.77 (17.69 to 35.68) | 7.58 (5.29 to 10.38) | 1.70 (1.19 to 2.31) |
| Papua New Guinea | Both | Number of prevalence | 18483.44 (4480.69 to 50093.56) | 42949.13 (13028.07 to 109565.46) | 54638.90 (16633.70 to 139541.23) | 66201.16 (20751.34 to 167880.18) | 79797.60 (25751.05 to 201044.27) | 84808.22 (27848.44 to 212831.48) | 80803.63 (26761.18 to 202645.06) | 74855.82 (24972.44 to 187167.11) | 67772.35 (22719.31 to 169069.96) | 58848.29 (19818.87 to 146378.62) | 49024.42 (16717.94 to 121379.82) | 39790.33 (13623.09 to 98257.39) | 29098.81 (10023.90 to 71680.18) | 19979.33 (6909.38 to 49156.57) | 12513.61 (4328.25 to 30805.62) | 7232.24 (2494.34 to 17818.26) | 3262.68 (1121.14 to 8047.15) | 1235.95 (419.53 to 3059.76) | 389.88 (129.06 to 972.47) | 95.55 (30.62 to 240.67) |
| Papua New Guinea | Female | Number of DALYs | 90.45 (55.07 to 133.48) | 142.61 (84.55 to 219.74) | 138.13 (77.06 to 218.84) | 136.96 (84.41 to 204.17) | 146.38 (88.07 to 220.41) | 150.62 (93.42 to 220.50) | 143.03 (90.90 to 209.39) | 126.30 (78.25 to 189.80) | 111.15 (67.79 to 167.36) | 93.94 (59.12 to 143.08) | 74.37 (48.08 to 110.75) | 57.26 (35.85 to 85.33) | 40.28 (25.51 to 58.85) | 26.88 (16.84 to 39.03) | 16.67 (10.70 to 24.29) | 9.64 (6.26 to 13.82) | 4.29 (2.74 to 6.12) | 1.66 (1.08 to 2.39) | 0.55 (0.37 to 0.77) | 0.15 (0.10 to 0.21) |
| Papua New Guinea | Female | Number of prevalence | 8959.26 (2258.13 to 24068.33) | 18670.58 (4323.79 to 51005.40) | 23161.37 (5060.64 to 63971.71) | 27454.35 (5787.91 to 76297.77) | 33008.60 (6850.08 to 91993.30) | 35680.07 (7341.79 to 99571.33) | 34782.25 (7126.13 to 97133.05) | 31859.29 (6507.73 to 89012.16) | 28484.52 (5807.19 to 79603.45) | 24197.55 (4923.92 to 67633.31) | 19296.83 (3923.16 to 53947.00) | 15289.82 (3104.35 to 42748.23) | 10910.05 (2215.06 to 30507.82) | 7382.80 (1498.70 to 20645.08) | 4655.68 (945.30 to 13019.11) | 2721.13 (552.18 to 7609.57) | 1245.40 (252.86 to 3482.53) | 494.31 (100.32 to 1382.23) | 169.87 (34.49 to 475.03) | 46.20 (9.38 to 129.18) |
| Papua New Guinea | Male | Number of DALYs | 69.95 (37.77 to 110.58) | 626.92 (322.08 to 1170.55) | 851.28 (448.27 to 1528.39) | 1156.65 (651.23 to 1947.98) | 1504.08 (917.52 to 2351.05) | 1662.26 (1042.59 to 2553.15) | 1595.68 (1007.29 to 2375.17) | 1519.63 (989.37 to 2259.67) | 1395.55 (915.19 to 2025.66) | 1239.05 (800.90 to 1794.74) | 1062.59 (693.37 to 1536.08) | 870.91 (574.74 to 1254.62) | 643.49 (429.33 to 927.31) | 439.66 (293.70 to 626.17) | 268.04 (178.60 to 377.77) | 151.01 (103.30 to 213.69) | 66.48 (44.56 to 93.36) | 24.10 (16.43 to 33.88) | 7.03 (4.80 to 9.70) | 1.55 (1.08 to 2.12) |
| Papua New Guinea | Male | Number of prevalence | 9524.17 (2221.84 to 26025.22) | 24278.55 (8164.68 to 59593.35) | 31477.53 (10934.56 to 76819.74) | 38746.81 (14195.92 to 92959.02) | 46789.00 (18384.32 to 110348.78) | 49128.15 (19967.59 to 114378.02) | 46021.37 (19110.48 to 106317.87) | 42996.53 (18002.74 to 98778.18) | 39287.82 (16606.23 to 89885.35) | 34650.74 (14747.68 to 79076.92) | 29727.59 (12796.49 to 67693.27) | 24500.51 (10615.10 to 55709.61) | 18188.77 (7912.16 to 41313.78) | 12596.53 (5483.17 to 28607.07) | 7857.93 (3418.83 to 17846.79) | 4511.11 (1963.27 to 10244.17) | 2017.28 (877.63 to 4580.67) | 741.63 (323.86 to 1683.89) | 220.01 (95.96 to 499.64) | 49.36 (21.50 to 112.09) |
| Philippines | Both | Number of DALYs | 1088.40 (729.31 to 1596.41) | 3243.67 (1855.72 to 5841.74) | 3369.65 (1905.73 to 6059.82) | 3304.97 (1872.10 to 5887.54) | 3266.36 (1854.56 to 5817.03) | 3157.75 (1800.11 to 5676.84) | 2884.42 (1646.44 to 5168.88) | 2529.12 (1431.69 to 4506.06) | 2330.08 (1329.83 to 4138.69) | 1988.72 (1148.36 to 3510.99) | 1777.20 (1026.12 to 3180.52) | 1439.91 (840.24 to 2537.25) | 1157.32 (680.77 to 2013.19) | 819.01 (484.45 to 1397.48) | 518.00 (310.88 to 884.23) | 283.87 (174.76 to 472.67) | 165.66 (103.21 to 270.09) | 76.03 (47.44 to 124.72) | 28.10 (17.23 to 47.40) | 10.04 (5.79 to 17.51) |
| Philippines | Both | Number of prevalence | 23036.81 (16030.43 to 41976.89) | 59933.36 (39670.35 to 106000.27) | 74328.94 (47235.98 to 141586.27) | 84544.58 (51050.25 to 171992.55) | 90837.80 (52905.03 to 194671.14) | 94297.71 (54247.56 to 206344.48) | 89609.02 (51245.71 to 198412.63) | 81069.40 (45775.04 to 184486.84) | 76808.64 (43026.01 to 177342.57) | 67530.91 (38145.94 to 155893.13) | 61698.62 (34720.64 to 146292.43) | 51568.74 (29093.51 to 123558.18) | 42877.95 (23798.16 to 104839.71) | 31490.13 (17415.90 to 77898.16) | 20929.68 (11567.11 to 53199.17) | 12186.00 (6826.78 to 30151.65) | 7420.34 (4137.78 to 18220.83) | 3461.89 (1918.20 to 8548.61) | 1264.02 (723.18 to 3021.19) | 398.81 (230.86 to 886.66) |
| Philippines | Female | Number of DALYs | 615.80 (414.24 to 916.96) | 1055.14 (703.82 to 1508.89) | 1061.88 (706.17 to 1534.62) | 1011.11 (683.93 to 1412.36) | 981.02 (668.68 to 1376.22) | 930.85 (630.69 to 1285.47) | 822.30 (547.06 to 1160.02) | 709.94 (483.63 to 998.52) | 646.86 (446.66 to 913.07) | 549.54 (370.36 to 773.93) | 497.25 (340.32 to 709.18) | 409.25 (277.30 to 580.89) | 339.38 (233.12 to 473.95) | 254.27 (174.94 to 356.84) | 172.58 (119.81 to 236.72) | 104.84 (72.00 to 142.89) | 67.29 (47.04 to 92.32) | 30.28 (20.98 to 41.52) | 8.64 (6.09 to 11.90) | 1.26 (0.90 to 1.69) |
| Philippines | Female | Number of prevalence | 11994.79 (8636.53 to 21007.15) | 25151.96 (16998.64 to 47903.71) | 31803.78 (20061.03 to 65102.71) | 36783.91 (21827.85 to 80626.72) | 39950.11 (22429.04 to 92621.71) | 41767.73 (22675.33 to 98940.48) | 39248.65 (20897.13 to 94224.97) | 35426.95 (18590.73 to 87564.61) | 33540.05 (17380.45 to 84096.98) | 29653.18 (15442.32 to 74499.24) | 27339.21 (14169.51 to 70609.35) | 23216.51 (12125.22 to 61045.94) | 19827.99 (10295.58 to 53160.72) | 15187.06 (7804.65 to 41267.76) | 10670.27 (5487.94 to 29128.54) | 6731.65 (3502.52 to 17837.59) | 4363.81 (2244.03 to 11680.43) | 2004.88 (1018.64 to 5411.41) | 626.07 (328.33 to 1622.34) | 102.55 (54.77 to 235.55) |
| Philippines | Male | Number of DALYs | 472.60 (315.62 to 676.55) | 2188.53 (1073.16 to 4529.89) | 2307.77 (1089.41 to 4743.19) | 2293.86 (1108.20 to 4640.18) | 2285.33 (1091.04 to 4594.09) | 2226.90 (1089.73 to 4520.52) | 2062.12 (1019.64 to 4044.93) | 1819.17 (905.61 to 3610.07) | 1683.23 (837.27 to 3372.04) | 1439.18 (722.53 to 2864.26) | 1279.95 (649.13 to 2541.09) | 1030.66 (521.29 to 2036.04) | 817.94 (418.57 to 1589.64) | 564.74 (286.59 to 1087.64) | 345.42 (179.07 to 668.63) | 179.02 (92.61 to 338.26) | 98.37 (51.25 to 187.30) | 45.75 (24.17 to 86.54) | 19.46 (10.46 to 36.42) | 8.78 (4.69 to 15.98) |
| Philippines | Male | Number of prevalence | 11042.02 (7412.49 to 21021.06) | 34781.40 (21502.64 to 61864.28) | 42525.16 (25635.92 to 77787.66) | 47760.68 (28472.48 to 92744.71) | 50887.68 (29340.55 to 103237.31) | 52529.97 (29701.17 to 108786.87) | 50360.37 (28360.83 to 105396.63) | 45642.45 (25614.33 to 97711.66) | 43268.59 (24175.79 to 93867.94) | 37877.73 (21357.26 to 82334.51) | 34359.41 (19244.14 to 76415.22) | 28352.22 (15874.76 to 63298.88) | 23049.96 (12925.51 to 52189.15) | 16303.06 (9155.37 to 36966.43) | 10259.42 (5803.04 to 23488.82) | 5454.35 (3155.62 to 12122.56) | 3056.53 (1782.24 to 6732.98) | 1457.01 (855.95 to 3235.09) | 637.95 (368.91 to 1393.21) | 296.26 (172.81 to 659.59) |
| Samoa | Both | Number of DALYs | 3.12 (1.99 to 4.54) | 13.61 (6.92 to 23.62) | 16.39 (8.65 to 29.04) | 19.97 (11.35 to 34.00) | 21.76 (12.57 to 35.43) | 23.04 (13.98 to 36.03) | 23.14 (13.97 to 35.66) | 20.84 (13.08 to 31.70) | 19.98 (12.87 to 30.65) | 20.18 (12.72 to 29.69) | 19.11 (12.46 to 28.00) | 16.93 (11.05 to 24.37) | 13.61 (9.08 to 19.74) | 9.52 (6.28 to 13.67) | 6.31 (4.21 to 9.17) | 4.04 (2.72 to 5.91) | 2.22 (1.51 to 3.15) | 0.99 (0.67 to 1.42) | 0.31 (0.21 to 0.43) | 0.06 (0.04 to 0.08) |
| Samoa | Both | Number of prevalence | 296.29 (51.70 to 1165.58) | 726.27 (163.74 to 2741.33) | 930.86 (201.57 to 3542.97) | 1111.95 (244.23 to 4219.51) | 1153.95 (260.29 to 4346.56) | 1178.84 (271.91 to 4414.44) | 1157.95 (271.40 to 4320.20) | 1022.60 (243.29 to 3797.93) | 946.09 (231.29 to 3493.15) | 934.89 (232.88 to 3434.26) | 882.49 (221.57 to 3234.56) | 792.09 (197.94 to 2902.01) | 647.74 (161.76 to 2372.02) | 464.55 (115.37 to 1703.71) | 321.75 (78.61 to 1184.96) | 216.42 (51.68 to 800.46) | 125.15 (29.19 to 465.48) | 58.06 (13.36 to 216.46) | 18.99 (4.30 to 71.06) | 3.98 (0.87 to 14.97) |
| Samoa | Female | Number of DALYs | 1.77 (1.08 to 2.74) | 2.93 (1.72 to 4.68) | 2.83 (1.64 to 4.27) | 2.69 (1.63 to 4.42) | 2.47 (1.50 to 3.77) | 2.31 (1.44 to 3.37) | 2.23 (1.41 to 3.41) | 1.87 (1.17 to 2.84) | 1.64 (0.98 to 2.46) | 1.52 (0.94 to 2.29) | 1.41 (0.88 to 2.13) | 1.26 (0.81 to 1.86) | 1.04 (0.66 to 1.52) | 0.74 (0.45 to 1.06) | 0.53 (0.34 to 0.79) | 0.37 (0.24 to 0.53) | 0.22 (0.14 to 0.32) | 0.10 (0.07 to 0.14) | 0.03 (0.02 to 0.05) | 0.01 (0.00 to 0.01) |
| Samoa | Female | Number of prevalence | 145.35 (27.67 to 563.89) | 321.93 (52.02 to 1281.15) | 411.24 (58.28 to 1664.83) | 483.36 (62.89 to 1976.28) | 493.21 (61.31 to 2026.29) | 503.17 (61.06 to 2072.43) | 498.07 (59.69 to 2054.08) | 434.72 (51.63 to 1794.37) | 387.23 (45.70 to 1599.48) | 371.28 (43.50 to 1534.39) | 350.35 (40.93 to 1448.43) | 321.85 (37.45 to 1331.11) | 269.10 (31.21 to 1113.39) | 197.93 (22.89 to 819.05) | 143.16 (16.57 to 592.43) | 100.60 (11.64 to 416.31) | 61.24 (7.09 to 253.41) | 29.04 (3.36 to 120.16) | 9.82 (1.14 to 40.62) | 2.16 (0.25 to 8.94) |
| Samoa | Male | Number of DALYs | 1.35 (0.75 to 2.15) | 10.68 (4.87 to 19.72) | 13.56 (6.60 to 25.55) | 17.28 (9.25 to 30.10) | 19.29 (10.76 to 32.51) | 20.72 (12.23 to 33.08) | 20.91 (12.57 to 33.12) | 18.96 (11.59 to 29.85) | 18.33 (11.62 to 28.64) | 18.66 (11.71 to 27.54) | 17.69 (11.40 to 25.99) | 15.68 (10.10 to 22.86) | 12.57 (8.23 to 18.39) | 8.78 (5.75 to 12.78) | 5.78 (3.74 to 8.46) | 3.68 (2.43 to 5.41) | 2.00 (1.32 to 2.89) | 0.89 (0.59 to 1.29) | 0.27 (0.18 to 0.39) | 0.05 (0.04 to 0.08) |
| Samoa | Male | Number of prevalence | 150.94 (23.83 to 601.69) | 404.34 (102.14 to 1460.18) | 519.63 (131.82 to 1878.14) | 628.59 (169.12 to 2243.22) | 660.74 (189.11 to 2320.27) | 675.67 (204.19 to 2342.01) | 659.88 (205.24 to 2266.11) | 587.88 (187.14 to 2003.56) | 558.87 (181.48 to 1893.67) | 563.61 (185.91 to 1899.87) | 532.14 (178.20 to 1786.13) | 470.25 (159.10 to 1570.90) | 378.64 (129.40 to 1258.63) | 266.63 (91.95 to 884.66) | 178.59 (61.69 to 592.53) | 115.82 (39.93 to 384.15) | 63.92 (22.05 to 212.08) | 29.03 (10.01 to 96.30) | 9.18 (3.17 to 30.44) | 1.82 (0.63 to 6.03) |
| Sao Tome and Principe | Both | Number of DALYs | 2.55 (1.59 to 3.78) | 9.15 (4.97 to 16.55) | 10.63 (5.67 to 18.57) | 11.02 (5.92 to 19.85) | 10.56 (5.57 to 18.55) | 9.96 (5.46 to 17.72) | 9.80 (5.20 to 17.17) | 9.37 (5.18 to 16.31) | 8.44 (4.58 to 14.62) | 7.19 (3.88 to 12.04) | 5.61 (3.06 to 9.48) | 4.48 (2.51 to 7.32) | 3.49 (1.88 to 5.91) | 2.44 (1.42 to 4.04) | 1.42 (0.81 to 2.35) | 0.85 (0.49 to 1.39) | 0.55 (0.32 to 0.88) | 0.25 (0.15 to 0.40) | 0.07 (0.04 to 0.11) | 0.01 (0.01 to 0.02) |
| Sao Tome and Principe | Both | Number of prevalence | 131.44 (26.55 to 697.13) | 354.13 (73.63 to 1785.55) | 494.75 (86.75 to 2605.65) | 586.80 (92.79 to 3166.46) | 591.03 (89.48 to 3221.94) | 572.10 (85.61 to 3131.06) | 570.83 (85.42 to 3128.82) | 544.87 (81.53 to 2987.14) | 489.00 (73.28 to 2679.71) | 417.94 (62.77 to 2289.90) | 332.31 (49.92 to 1822.66) | 269.93 (40.51 to 1483.01) | 214.20 (32.08 to 1177.60) | 152.18 (22.83 to 837.54) | 92.65 (13.69 to 511.87) | 60.12 (8.63 to 334.44) | 40.02 (5.64 to 223.42) | 18.44 (2.58 to 103.01) | 5.03 (0.70 to 28.12) | 0.99 (0.13 to 5.59) |
| Sao Tome and Principe | Female | Number of DALYs | 1.45 (0.87 to 2.27) | 2.64 (1.55 to 4.19) | 2.80 (1.59 to 4.33) | 2.63 (1.50 to 4.09) | 2.27 (1.43 to 3.40) | 1.96 (1.20 to 2.85) | 1.82 (1.07 to 2.78) | 1.64 (0.98 to 2.41) | 1.41 (0.86 to 2.19) | 1.15 (0.69 to 1.78) | 0.89 (0.54 to 1.36) | 0.72 (0.43 to 1.11) | 0.55 (0.33 to 0.80) | 0.39 (0.24 to 0.59) | 0.25 (0.15 to 0.36) | 0.17 (0.10 to 0.26) | 0.12 (0.07 to 0.18) | 0.05 (0.03 to 0.08) | 0.01 (0.01 to 0.02) | 0.00 (0.00 to 0.00) |
| Sao Tome and Principe | Female | Number of prevalence | 66.57 (14.96 to 344.13) | 160.86 (27.79 to 878.59) | 228.03 (30.82 to 1294.33) | 272.21 (30.12 to 1581.00) | 272.85 (26.76 to 1604.14) | 262.19 (23.90 to 1551.39) | 259.64 (22.57 to 1541.99) | 244.37 (20.56 to 1455.06) | 216.11 (17.75 to 1289.39) | 183.58 (14.75 to 1096.97) | 147.83 (11.68 to 884.53) | 122.61 (9.50 to 734.58) | 98.23 (7.43 to 589.33) | 70.82 (5.32 to 425.16) | 45.27 (3.41 to 271.76) | 31.82 (2.39 to 191.00) | 22.03 (1.66 to 132.23) | 10.22 (0.77 to 61.36) | 2.79 (0.21 to 16.77) | 0.60 (0.05 to 3.60) |
| Sao Tome and Principe | Male | Number of DALYs | 1.09 (0.60 to 1.78) | 6.51 (2.94 to 13.51) | 7.84 (3.54 to 15.34) | 8.40 (3.82 to 16.63) | 8.29 (3.82 to 15.89) | 8.00 (3.85 to 15.14) | 7.98 (3.89 to 14.67) | 7.73 (3.88 to 14.30) | 7.04 (3.41 to 13.01) | 6.05 (2.97 to 10.81) | 4.72 (2.36 to 8.50) | 3.76 (1.97 to 6.48) | 2.94 (1.51 to 5.21) | 2.05 (1.09 to 3.53) | 1.17 (0.63 to 2.04) | 0.68 (0.36 to 1.19) | 0.43 (0.22 to 0.75) | 0.19 (0.11 to 0.33) | 0.05 (0.03 to 0.09) | 0.01 (0.00 to 0.02) |
| Sao Tome and Principe | Male | Number of prevalence | 64.86 (11.31 to 353.00) | 193.27 (42.02 to 912.72) | 266.72 (51.29 to 1318.52) | 314.59 (57.34 to 1591.85) | 318.18 (57.48 to 1622.93) | 309.91 (56.28 to 1583.80) | 311.20 (56.73 to 1590.63) | 300.51 (55.39 to 1535.42) | 272.88 (50.70 to 1393.08) | 234.35 (44.06 to 1195.11) | 184.48 (34.90 to 939.83) | 147.32 (28.04 to 749.62) | 115.97 (22.35 to 589.13) | 81.36 (15.76 to 413.01) | 47.38 (9.17 to 240.48) | 28.30 (5.47 to 143.66) | 18.00 (3.48 to 91.32) | 8.22 (1.59 to 41.71) | 2.24 (0.43 to 11.37) | 0.39 (0.08 to 2.00) |
| Senegal | Both | Number of DALYs | 214.88 (135.07 to 323.34) | 574.16 (308.55 to 1021.21) | 548.60 (292.21 to 991.08) | 497.96 (252.57 to 931.45) | 428.92 (228.24 to 778.09) | 357.99 (194.77 to 665.51) | 299.74 (162.35 to 547.97) | 247.94 (133.72 to 456.07) | 201.07 (108.35 to 358.38) | 163.52 (89.34 to 306.72) | 132.88 (72.09 to 240.48) | 110.81 (61.45 to 201.53) | 88.20 (49.57 to 155.75) | 63.39 (34.52 to 109.46) | 42.65 (23.59 to 76.43) | 27.54 (15.37 to 48.48) | 14.79 (8.57 to 25.28) | 6.33 (3.57 to 11.31) | 1.92 (1.10 to 3.28) | 0.43 (0.26 to 0.72) |
| Senegal | Both | Number of prevalence | 3425.24 (2184.93 to 8732.60) | 8306.87 (4575.70 to 20361.63) | 9195.95 (4557.57 to 25682.05) | 9565.28 (4271.50 to 29361.52) | 9164.81 (3803.11 to 29835.41) | 8311.70 (3280.57 to 28119.36) | 7366.42 (2799.08 to 25516.11) | 6304.60 (2334.84 to 22163.29) | 5305.64 (1922.52 to 18856.90) | 4441.66 (1577.43 to 15943.84) | 3716.25 (1299.50 to 13455.07) | 3199.27 (1102.41 to 11663.83) | 2616.14 (888.08 to 9607.26) | 1932.89 (650.77 to 7129.87) | 1324.29 (445.84 to 4889.85) | 877.90 (294.98 to 3249.97) | 484.50 (162.10 to 1797.70) | 212.90 (71.07 to 791.51) | 66.80 (22.26 to 249.64) | 15.81 (5.23 to 59.62) |
| Senegal | Female | Number of DALYs | 123.36 (73.56 to 200.08) | 194.50 (106.81 to 312.84) | 177.45 (104.36 to 279.80) | 158.00 (88.63 to 243.88) | 138.20 (79.32 to 212.09) | 119.64 (67.53 to 189.82) | 103.74 (59.84 to 159.10) | 84.65 (48.03 to 127.73) | 68.37 (40.19 to 108.89) | 55.25 (32.08 to 84.31) | 45.21 (27.36 to 69.64) | 37.15 (22.25 to 56.73) | 28.82 (15.37 to 45.36) | 20.73 (12.12 to 32.13) | 14.32 (8.68 to 22.53) | 9.55 (5.78 to 13.98) | 5.39 (3.19 to 8.18) | 2.36 (1.49 to 3.48) | 0.77 (0.45 to 1.18) | 0.20 (0.12 to 0.30) |
| Senegal | Female | Number of prevalence | 1848.77 (1235.94 to 4457.00) | 3371.61 (1989.13 to 9371.29) | 3761.41 (1898.57 to 11842.15) | 3971.37 (1736.54 to 13670.93) | 3963.77 (1559.39 to 14380.62) | 3783.53 (1390.23 to 14136.67) | 3459.38 (1214.92 to 13160.50) | 2953.58 (1005.92 to 11383.02) | 2476.87 (821.55 to 9637.84) | 2094.66 (678.74 to 8216.83) | 1759.83 (559.81 to 6952.64) | 1492.90 (465.00 to 5940.26) | 1204.29 (367.77 to 4826.49) | 895.33 (270.51 to 3600.71) | 619.86 (187.40 to 2492.30) | 424.80 (128.24 to 1708.31) | 241.27 (72.85 to 970.04) | 108.35 (32.80 to 435.77) | 36.22 (10.95 to 145.63) | 9.43 (2.85 to 37.91) |
| Senegal | Male | Number of DALYs | 91.52 (49.54 to 149.80) | 379.66 (166.95 to 788.55) | 371.15 (164.04 to 765.67) | 339.96 (149.32 to 726.12) | 290.72 (125.87 to 617.31) | 238.35 (109.94 to 495.37) | 196.01 (87.50 to 416.21) | 163.29 (73.51 to 351.31) | 132.70 (60.11 to 271.54) | 108.27 (48.23 to 236.67) | 87.67 (38.41 to 188.66) | 73.66 (32.70 to 151.83) | 59.38 (27.24 to 117.59) | 42.66 (19.25 to 84.57) | 28.34 (12.35 to 58.29) | 17.99 (8.21 to 37.38) | 9.40 (4.25 to 18.62) | 3.97 (1.70 to 8.44) | 1.15 (0.51 to 2.30) | 0.24 (0.10 to 0.48) |
| Senegal | Male | Number of prevalence | 1576.46 (926.77 to 4275.60) | 4935.26 (2446.64 to 11060.66) | 5434.54 (2483.33 to 13789.96) | 5593.91 (2351.80 to 15831.82) | 5201.04 (2093.30 to 15655.02) | 4528.16 (1763.20 to 14136.74) | 3907.04 (1490.87 to 12471.43) | 3351.02 (1261.28 to 10864.61) | 2828.77 (1048.30 to 9286.89) | 2347.00 (860.30 to 7780.29) | 1956.42 (708.29 to 6545.78) | 1706.37 (610.00 to 5757.58) | 1411.85 (496.80 to 4809.77) | 1037.56 (363.06 to 3548.50) | 704.43 (246.58 to 2409.80) | 453.09 (158.28 to 1549.64) | 243.24 (85.22 to 831.91) | 104.55 (36.60 to 357.54) | 30.58 (10.68 to 104.58) | 6.38 (2.23 to 21.83) |
| Sierra Leone | Both | Number of DALYs | 133.38 (81.06 to 195.04) | 461.69 (253.77 to 828.58) | 495.97 (271.45 to 871.34) | 567.51 (303.57 to 977.46) | 610.18 (319.91 to 1041.95) | 590.94 (332.26 to 983.39) | 544.05 (298.50 to 867.33) | 460.78 (263.99 to 767.84) | 403.59 (233.29 to 666.30) | 333.81 (198.27 to 546.41) | 273.42 (165.06 to 446.46) | 218.48 (129.60 to 358.96) | 176.26 (103.02 to 286.64) | 128.80 (78.15 to 204.35) | 88.76 (54.19 to 138.59) | 59.57 (36.75 to 94.28) | 31.06 (19.09 to 48.81) | 11.28 (6.90 to 17.15) | 3.20 (1.96 to 5.02) | 0.72 (0.44 to 1.09) |
| Sierra Leone | Both | Number of prevalence | 9052.47 (2070.74 to 29182.53) | 21304.76 (5324.19 to 67853.00) | 26761.50 (6118.28 to 86485.33) | 33158.79 (7234.17 to 107924.50) | 36543.36 (7902.35 to 119130.03) | 35308.65 (7637.72 to 115002.68) | 32115.18 (6985.81 to 104441.18) | 26848.13 (5894.09 to 87085.92) | 23318.71 (5164.44 to 75451.43) | 18625.42 (4192.69 to 60028.16) | 15022.94 (3418.56 to 48303.00) | 12056.91 (2769.02 to 38705.34) | 9650.76 (2245.28 to 30909.32) | 7221.72 (1682.54 to 23142.57) | 5169.01 (1195.88 to 16606.78) | 3607.26 (828.24 to 11613.19) | 1968.30 (446.27 to 6360.95) | 734.51 (166.14 to 2374.81) | 212.12 (47.92 to 686.01) | 51.71 (11.47 to 168.10) |
| Sierra Leone | Female | Number of DALYs | 77.34 (45.08 to 123.30) | 123.33 (74.23 to 189.42) | 115.97 (71.32 to 179.58) | 116.62 (69.45 to 176.45) | 110.50 (65.37 to 164.31) | 97.84 (62.64 to 153.86) | 83.56 (52.08 to 123.53) | 65.20 (40.13 to 97.78) | 53.78 (33.95 to 81.61) | 40.66 (24.56 to 61.13) | 30.98 (18.58 to 45.76) | 24.01 (15.24 to 37.27) | 18.61 (11.27 to 27.96) | 13.81 (8.90 to 20.74) | 10.22 (6.37 to 14.96) | 7.23 (4.51 to 10.71) | 4.13 (2.49 to 6.01) | 1.51 (0.96 to 2.22) | 0.43 (0.27 to 0.63) | 0.11 (0.07 to 0.16) |
| Sierra Leone | Female | Number of prevalence | 4595.46 (1128.13 to 14558.51) | 9800.68 (2022.53 to 32159.83) | 12411.02 (2228.64 to 41728.53) | 15578.36 (2543.42 to 53139.58) | 17281.83 (2684.53 to 59382.45) | 16635.90 (2509.86 to 57385.09) | 15019.51 (2232.10 to 51924.85) | 12254.29 (1798.42 to 42428.18) | 10474.40 (1522.42 to 36307.51) | 7986.68 (1153.45 to 27709.49) | 6321.57 (909.33 to 21950.69) | 5071.05 (724.36 to 17620.53) | 4028.90 (572.15 to 14010.86) | 3077.96 (435.75 to 10707.04) | 2297.39 (325.16 to 7991.77) | 1648.94 (233.15 to 5736.01) | 948.87 (134.08 to 3300.88) | 357.04 (50.56 to 1241.96) | 103.42 (14.63 to 359.76) | 27.13 (3.83 to 94.36) |
| Sierra Leone | Male | Number of DALYs | 56.04 (30.19 to 89.64) | 338.36 (155.92 to 685.97) | 380.00 (179.81 to 732.02) | 450.89 (222.04 to 846.56) | 499.67 (244.88 to 904.32) | 493.10 (252.70 to 851.69) | 460.49 (236.57 to 774.70) | 395.58 (216.07 to 688.67) | 349.81 (192.89 to 605.98) | 293.15 (160.03 to 493.67) | 242.44 (141.81 to 404.93) | 194.48 (111.39 to 324.66) | 157.65 (90.35 to 261.27) | 114.99 (67.51 to 188.87) | 78.54 (45.99 to 124.58) | 52.34 (30.90 to 84.77) | 26.93 (15.86 to 43.81) | 9.77 (5.69 to 15.44) | 2.77 (1.63 to 4.44) | 0.61 (0.36 to 0.96) |
| Sierra Leone | Male | Number of prevalence | 4457.01 (942.32 to 14624.02) | 11504.08 (3222.63 to 36092.55) | 14350.48 (3803.58 to 45393.19) | 17580.44 (4539.72 to 55384.41) | 19261.53 (5000.60 to 60149.36) | 18672.75 (4933.10 to 57705.77) | 17095.68 (4582.10 to 52592.41) | 14593.84 (3968.78 to 44720.19) | 12844.31 (3536.25 to 39195.02) | 10638.74 (2960.78 to 32356.30) | 8701.37 (2447.72 to 26382.64) | 6985.86 (1991.31 to 21108.38) | 5621.86 (1626.80 to 16917.54) | 4143.77 (1208.16 to 12449.66) | 2871.62 (840.43 to 8625.45) | 1958.32 (572.61 to 5884.89) | 1019.43 (297.17 to 3064.31) | 377.47 (110.70 to 1134.47) | 108.70 (31.75 to 326.74) | 24.58 (7.17 to 73.87) |
| South Sudan | Both | Number of DALYs | 139.22 (84.88 to 204.63) | 396.80 (212.44 to 722.47) | 409.17 (222.37 to 745.18) | 374.67 (198.40 to 695.82) | 263.78 (147.34 to 474.30) | 165.17 (91.43 to 293.08) | 163.99 (90.58 to 285.21) | 164.80 (91.53 to 294.66) | 153.06 (79.12 to 269.43) | 131.97 (70.00 to 233.63) | 106.03 (55.16 to 194.61) | 79.49 (41.16 to 142.43) | 55.91 (30.35 to 103.73) | 36.32 (20.11 to 66.29) | 21.90 (11.47 to 40.58) | 13.81 (7.48 to 25.04) | 6.96 (3.80 to 12.31) | 2.98 (1.63 to 5.30) | 0.90 (0.50 to 1.58) | 0.25 (0.14 to 0.43) |
| South Sudan | Both | Number of prevalence | 3373.12 (1772.04 to 7394.56) | 8207.06 (4216.05 to 17170.70) | 10403.20 (4762.43 to 23525.58) | 11339.29 (4635.05 to 27016.30) | 9341.47 (3557.77 to 23011.55) | 6344.07 (2328.62 to 15905.00) | 6804.90 (2438.46 to 17278.72) | 7052.30 (2493.93 to 17993.63) | 6624.22 (2321.72 to 16950.65) | 5744.54 (2003.32 to 14715.48) | 4707.85 (1634.16 to 12074.09) | 3580.56 (1236.62 to 9193.14) | 2574.26 (883.37 to 6626.54) | 1724.51 (589.31 to 4445.44) | 1078.64 (367.39 to 2787.81) | 685.40 (234.16 to 1767.77) | 351.74 (120.08 to 907.58) | 153.32 (52.36 to 395.43) | 47.94 (16.33 to 123.83) | 14.26 (4.81 to 37.08) |
| South Sudan | Female | Number of DALYs | 79.38 (46.12 to 121.33) | 126.06 (72.26 to 199.56) | 124.05 (68.80 to 195.48) | 111.99 (65.41 to 171.22) | 84.61 (50.75 to 136.52) | 54.20 (32.24 to 83.40) | 56.75 (34.47 to 87.06) | 54.34 (31.23 to 83.63) | 48.69 (27.86 to 76.76) | 38.06 (22.30 to 59.42) | 28.71 (17.13 to 43.49) | 20.34 (12.19 to 31.38) | 13.61 (7.92 to 20.70) | 8.80 (5.13 to 13.22) | 5.69 (3.40 to 8.41) | 3.35 (1.93 to 5.00) | 1.71 (1.03 to 2.61) | 0.73 (0.45 to 1.10) | 0.23 (0.14 to 0.35) | 0.08 (0.05 to 0.12) |
| South Sudan | Female | Number of prevalence | 1745.44 (974.70 to 3668.56) | 3444.14 (1664.32 to 7877.30) | 4419.82 (1853.04 to 10814.64) | 4925.94 (1836.48 to 12595.31) | 4467.71 (1550.82 to 11702.10) | 3152.52 (1048.66 to 8369.51) | 3572.14 (1157.14 to 9562.68) | 3610.78 (1146.90 to 9721.82) | 3278.17 (1025.93 to 8864.20) | 2700.86 (835.55 to 7328.00) | 2120.95 (648.44 to 5772.46) | 1542.63 (466.04 to 4210.56) | 1084.72 (324.18 to 2970.25) | 725.20 (215.81 to 1988.42) | 479.41 (142.56 to 1314.63) | 292.19 (86.93 to 801.13) | 151.07 (44.97 to 414.24) | 65.32 (19.41 to 179.08) | 21.08 (6.28 to 57.80) | 7.15 (2.13 to 19.59) |
| South Sudan | Male | Number of DALYs | 59.84 (30.98 to 97.42) | 270.74 (117.92 to 573.10) | 285.13 (132.90 to 581.26) | 262.68 (109.26 to 562.24) | 179.17 (76.94 to 375.39) | 110.96 (50.18 to 225.97) | 107.24 (48.25 to 215.20) | 110.46 (47.88 to 228.50) | 104.37 (44.99 to 202.56) | 93.91 (42.70 to 184.41) | 77.32 (34.87 to 154.26) | 59.15 (26.65 to 120.61) | 42.30 (19.37 to 86.55) | 27.51 (12.79 to 55.18) | 16.21 (7.02 to 33.32) | 10.47 (4.90 to 21.14) | 5.26 (2.48 to 10.02) | 2.25 (1.05 to 4.46) | 0.67 (0.31 to 1.25) | 0.17 (0.08 to 0.34) |
| South Sudan | Male | Number of prevalence | 1627.68 (790.84 to 3719.78) | 4762.92 (2433.00 to 9587.73) | 5983.38 (2827.06 to 12732.74) | 6413.35 (2828.40 to 14421.00) | 4873.76 (2060.82 to 11309.45) | 3191.54 (1304.42 to 7535.49) | 3232.76 (1297.92 to 7716.04) | 3441.52 (1358.10 to 8271.82) | 3346.05 (1303.69 to 8086.45) | 3043.68 (1175.95 to 7387.48) | 2586.89 (990.11 to 6301.63) | 2037.93 (774.83 to 4982.57) | 1489.53 (559.88 to 3656.29) | 999.30 (374.25 to 2457.02) | 599.23 (224.75 to 1473.18) | 393.21 (147.57 to 966.64) | 200.67 (75.23 to 493.33) | 88.00 (33.04 to 216.36) | 26.86 (10.08 to 66.03) | 7.11 (2.67 to 17.49) |
| Sri Lanka | Both | Number of DALYs | 0.00 (0.00 to 0.00) | 0.00 (0.00 to 0.00) | 0.00 (0.00 to 0.00) | 0.00 (0.00 to 0.00) | 0.00 (0.00 to 0.00) | 0.00 (0.00 to 0.00) | 0.00 (0.00 to 0.00) | 0.00 (0.00 to 0.00) | 0.00 (0.00 to 0.00) | 0.00 (0.00 to 0.00) | 0.00 (0.00 to 0.00) | 0.00 (0.00 to 0.00) | 0.00 (0.00 to 0.00) | 0.00 (0.00 to 0.00) | 0.00 (0.00 to 0.00) | 0.00 (0.00 to 0.00) | 0.00 (0.00 to 0.00) | 0.00 (0.00 to 0.00) | 0.00 (0.00 to 0.00) | 0.00 (0.00 to 0.00) |
| Sri Lanka | Both | Number of prevalence | 350.98 (256.42 to 477.24) | 929.51 (679.09 to 1263.88) | 1436.75 (1049.68 to 1953.59) | 1918.41 (1401.57 to 2608.52) | 2109.29 (1541.03 to 2868.07) | 2356.94 (1721.96 to 3204.81) | 2415.26 (1764.57 to 3284.11) | 2673.50 (1953.24 to 3635.25) | 2826.42 (2064.96 to 3843.17) | 2585.03 (1888.60 to 3514.94) | 2566.47 (1875.04 to 3489.72) | 2495.54 (1823.22 to 3393.26) | 2350.02 (1716.91 to 3195.40) | 1981.22 (1447.46 to 2693.92) | 1481.80 (1082.59 to 2014.85) | 922.84 (674.22 to 1254.82) | 501.45 (366.36 to 681.84) | 237.18 (173.28 to 322.49) | 79.68 (58.21 to 108.34) | 18.98 (13.87 to 25.81) |
| Sri Lanka | Female | Number of DALYs | 0.00 (0.00 to 0.00) | 0.00 (0.00 to 0.00) | 0.00 (0.00 to 0.00) | 0.00 (0.00 to 0.00) | 0.00 (0.00 to 0.00) | 0.00 (0.00 to 0.00) | 0.00 (0.00 to 0.00) | 0.00 (0.00 to 0.00) | 0.00 (0.00 to 0.00) | 0.00 (0.00 to 0.00) | 0.00 (0.00 to 0.00) | 0.00 (0.00 to 0.00) | 0.00 (0.00 to 0.00) | 0.00 (0.00 to 0.00) | 0.00 (0.00 to 0.00) | 0.00 (0.00 to 0.00) | 0.00 (0.00 to 0.00) | 0.00 (0.00 to 0.00) | 0.00 (0.00 to 0.00) | 0.00 (0.00 to 0.00) |
| Sri Lanka | Female | Number of prevalence | 172.41 (125.96 to 234.43) | 458.89 (335.26 to 623.97) | 711.40 (519.74 to 967.31) | 955.27 (697.91 to 1298.90) | 1061.43 (775.47 to 1443.26) | 1210.48 (884.36 to 1645.93) | 1260.13 (920.64 to 1713.44) | 1392.42 (1017.29 to 1893.31) | 1455.02 (1063.03 to 1978.44) | 1335.46 (975.67 to 1815.86) | 1338.11 (977.61 to 1819.48) | 1323.22 (966.73 to 1799.22) | 1264.69 (923.97 to 1719.64) | 1089.47 (795.96 to 1481.38) | 838.31 (612.46 to 1139.87) | 539.36 (394.05 to 733.39) | 305.07 (222.88 to 414.82) | 153.28 (111.99 to 208.42) | 54.63 (39.91 to 74.28) | 14.02 (10.24 to 19.07) |
| Sri Lanka | Male | Number of DALYs | 0.00 (0.00 to 0.00) | 0.00 (0.00 to 0.00) | 0.00 (0.00 to 0.00) | 0.00 (0.00 to 0.00) | 0.00 (0.00 to 0.00) | 0.00 (0.00 to 0.00) | 0.00 (0.00 to 0.00) | 0.00 (0.00 to 0.00) | 0.00 (0.00 to 0.00) | 0.00 (0.00 to 0.00) | 0.00 (0.00 to 0.00) | 0.00 (0.00 to 0.00) | 0.00 (0.00 to 0.00) | 0.00 (0.00 to 0.00) | 0.00 (0.00 to 0.00) | 0.00 (0.00 to 0.00) | 0.00 (0.00 to 0.00) | 0.00 (0.00 to 0.00) | 0.00 (0.00 to 0.00) | 0.00 (0.00 to 0.00) |
| Sri Lanka | Male | Number of prevalence | 178.57 (130.46 to 242.80) | 470.61 (343.83 to 639.91) | 725.35 (529.93 to 986.28) | 963.14 (703.66 to 1309.62) | 1047.86 (765.56 to 1424.82) | 1146.47 (837.60 to 1558.89) | 1155.13 (843.92 to 1570.66) | 1281.09 (935.95 to 1741.93) | 1371.40 (1001.93 to 1864.74) | 1249.57 (912.93 to 1699.08) | 1228.36 (897.43 to 1670.24) | 1172.32 (856.49 to 1594.04) | 1085.34 (792.94 to 1475.76) | 891.75 (651.50 to 1212.54) | 643.49 (470.13 to 874.97) | 383.48 (280.17 to 521.43) | 196.38 (143.47 to 267.02) | 83.89 (61.29 to 114.07) | 25.05 (18.30 to 34.06) | 4.96 (3.63 to 6.75) |
| Sudan | Both | Number of DALYs | 546.25 (331.50 to 842.99) | 1599.78 (845.85 to 3001.66) | 1568.75 (799.68 to 2891.32) | 1449.69 (772.23 to 2665.72) | 1318.22 (701.21 to 2521.24) | 1166.01 (632.13 to 2074.49) | 997.96 (552.77 to 1775.46) | 823.44 (460.83 to 1480.08) | 687.16 (384.11 to 1169.52) | 555.53 (296.87 to 1001.26) | 447.37 (233.89 to 824.91) | 338.14 (179.05 to 610.95) | 246.23 (129.96 to 458.84) | 180.67 (92.70 to 334.83) | 128.60 (66.83 to 236.66) | 81.20 (43.79 to 143.57) | 45.31 (24.57 to 81.95) | 19.36 (10.79 to 33.92) | 6.26 (3.38 to 11.60) | 1.91 (1.06 to 3.40) |
| Sudan | Both | Number of prevalence | 11780.07 (5559.85 to 35518.98) | 30067.37 (12477.05 to 89556.89) | 36047.15 (12610.71 to 118390.53) | 39635.16 (12266.67 to 139181.85) | 40523.73 (11545.69 to 147854.48) | 39039.64 (10491.95 to 145625.09) | 35282.00 (9072.76 to 133480.38) | 31117.47 (7718.88 to 118989.63) | 26451.31 (6398.65 to 101764.52) | 21741.99 (5211.44 to 83830.81) | 17852.70 (4232.83 to 68990.76) | 13629.43 (3193.33 to 52792.20) | 10174.44 (2341.99 to 39550.37) | 7628.01 (1741.75 to 29722.69) | 5436.29 (1253.68 to 21115.71) | 3545.72 (810.29 to 13806.19) | 2022.90 (463.38 to 7877.34) | 886.18 (202.68 to 3452.00) | 294.52 (66.94 to 1149.32) | 94.07 (20.96 to 368.99) |
| Sudan | Female | Number of DALYs | 309.93 (177.05 to 477.53) | 518.38 (302.44 to 800.75) | 495.61 (277.02 to 758.04) | 444.50 (255.80 to 732.09) | 398.83 (247.38 to 636.67) | 356.73 (199.25 to 562.93) | 317.82 (191.53 to 504.86) | 273.80 (162.47 to 436.36) | 222.13 (129.45 to 338.97) | 162.06 (96.57 to 253.92) | 123.42 (74.86 to 189.40) | 88.18 (52.42 to 140.06) | 60.63 (36.35 to 95.26) | 45.02 (27.43 to 65.85) | 29.34 (17.81 to 44.28) | 19.83 (11.89 to 29.77) | 11.26 (6.70 to 17.00) | 4.81 (2.84 to 7.23) | 1.63 (0.99 to 2.49) | 0.57 (0.33 to 0.84) |
| Sudan | Female | Number of prevalence | 6148.98 (3134.07 to 17617.54) | 12649.97 (5305.96 to 40725.91) | 15396.70 (5282.45 to 54355.95) | 17221.32 (5004.87 to 64653.29) | 17872.23 (4587.84 to 69441.47) | 17783.08 (4247.25 to 70421.59) | 16808.57 (3814.96 to 67304.28) | 15446.17 (3391.43 to 62339.73) | 12973.18 (2767.17 to 52687.50) | 10047.65 (2091.83 to 41002.75) | 7837.39 (1596.52 to 32121.99) | 5740.82 (1145.41 to 23627.34) | 4166.69 (811.58 to 17224.73) | 3143.08 (605.35 to 13019.18) | 2088.97 (402.56 to 8651.89) | 1442.12 (277.67 to 5973.56) | 821.31 (158.09 to 3402.29) | 364.07 (70.18 to 1508.02) | 125.58 (24.18 to 520.22) | 44.65 (8.60 to 184.95) |
| Sudan | Male | Number of DALYs | 236.32 (127.59 to 386.09) | 1081.41 (475.89 to 2322.90) | 1073.14 (445.61 to 2315.58) | 1005.19 (450.04 to 2112.36) | 919.39 (402.31 to 1991.06) | 809.28 (347.44 to 1688.43) | 680.14 (295.91 to 1373.37) | 549.64 (251.29 to 1154.25) | 465.04 (212.33 to 915.74) | 393.46 (168.38 to 782.30) | 323.95 (144.49 to 672.10) | 249.96 (112.70 to 517.36) | 185.60 (85.19 to 387.52) | 135.65 (60.31 to 273.95) | 99.26 (44.32 to 201.62) | 61.38 (28.35 to 119.68) | 34.05 (15.42 to 68.93) | 14.54 (6.61 to 28.01) | 4.63 (2.04 to 9.54) | 1.34 (0.63 to 2.75) |
| Sudan | Male | Number of prevalence | 5631.09 (2433.34 to 17901.44) | 17417.40 (6707.15 to 50192.05) | 20650.45 (7066.81 to 64034.57) | 22413.84 (6959.67 to 74528.56) | 22651.49 (6426.56 to 78413.01) | 21256.55 (5750.28 to 75203.50) | 18473.43 (4816.70 to 66176.11) | 15671.29 (4022.42 to 56649.90) | 13478.13 (3405.32 to 49077.02) | 11694.33 (2920.02 to 42828.06) | 10015.31 (2464.81 to 36868.77) | 7888.61 (1919.31 to 29164.86) | 6007.74 (1441.47 to 22325.64) | 4484.93 (1075.09 to 16703.51) | 3347.32 (799.75 to 12463.82) | 2103.59 (502.71 to 7832.63) | 1201.60 (287.82 to 4475.05) | 522.11 (124.52 to 1943.99) | 168.95 (40.32 to 629.10) | 49.42 (11.83 to 184.05) |
| Thailand | Both | Number of DALYs | 0.00 (0.00 to 0.00) | 0.00 (0.00 to 0.00) | 0.00 (0.00 to 0.00) | 0.00 (0.00 to 0.00) | 0.00 (0.00 to 0.00) | 0.00 (0.00 to 0.00) | 0.00 (0.00 to 0.00) | 0.00 (0.00 to 0.00) | 0.00 (0.00 to 0.00) | 0.00 (0.00 to 0.00) | 0.00 (0.00 to 0.00) | 0.00 (0.00 to 0.00) | 0.00 (0.00 to 0.00) | 0.00 (0.00 to 0.00) | 0.00 (0.00 to 0.00) | 0.00 (0.00 to 0.00) | 0.00 (0.00 to 0.00) | 0.00 (0.00 to 0.00) | 0.00 (0.00 to 0.00) | 0.00 (0.00 to 0.00) |
| Thailand | Both | Number of prevalence | 2076.66 (111.05 to 10367.19) | 5748.64 (307.40 to 28698.62) | 9440.98 (504.85 to 47131.64) | 13525.85 (723.29 to 67524.31) | 17885.42 (956.41 to 89288.35) | 20701.08 (1106.98 to 103344.84) | 21358.44 (1142.13 to 106626.53) | 26483.45 (1416.18 to 132211.80) | 30558.15 (1634.08 to 152553.71) | 33350.01 (1783.37 to 166491.34) | 35073.79 (1875.55 to 175096.87) | 34001.18 (1818.19 to 169742.16) | 31300.27 (1673.76 to 156258.57) | 23666.94 (1265.57 to 118151.10) | 17070.73 (912.85 to 85221.23) | 11272.11 (602.77 to 56273.13) | 7709.90 (412.28 to 38489.67) | 4405.52 (235.58 to 21993.42) | 2005.43 (107.24 to 10011.59) | 744.53 (39.81 to 3716.89) |
| Thailand | Female | Number of DALYs | 0.00 (0.00 to 0.00) | 0.00 (0.00 to 0.00) | 0.00 (0.00 to 0.00) | 0.00 (0.00 to 0.00) | 0.00 (0.00 to 0.00) | 0.00 (0.00 to 0.00) | 0.00 (0.00 to 0.00) | 0.00 (0.00 to 0.00) | 0.00 (0.00 to 0.00) | 0.00 (0.00 to 0.00) | 0.00 (0.00 to 0.00) | 0.00 (0.00 to 0.00) | 0.00 (0.00 to 0.00) | 0.00 (0.00 to 0.00) | 0.00 (0.00 to 0.00) | 0.00 (0.00 to 0.00) | 0.00 (0.00 to 0.00) | 0.00 (0.00 to 0.00) | 0.00 (0.00 to 0.00) | 0.00 (0.00 to 0.00) |
| Thailand | Female | Number of prevalence | 1008.73 (53.94 to 5035.84) | 2792.02 (149.30 to 13938.46) | 4607.04 (246.36 to 22999.47) | 6696.51 (358.09 to 33430.58) | 8977.02 (480.04 to 44815.48) | 10749.55 (574.83 to 53664.38) | 11051.88 (590.99 to 55173.65) | 13640.75 (729.43 to 68097.92) | 15535.76 (830.76 to 77558.30) | 17186.46 (919.03 to 85799.00) | 18294.52 (978.29 to 91330.70) | 17881.43 (956.20 to 89268.44) | 16612.64 (888.35 to 82934.32) | 12759.70 (682.32 to 63699.54) | 9287.52 (496.64 to 46365.56) | 6319.70 (337.94 to 31549.50) | 4489.03 (240.05 to 22410.34) | 2683.87 (143.52 to 13398.51) | 1231.51 (65.85 to 6147.99) | 443.59 (23.72 to 2214.49) |
| Thailand | Male | Number of DALYs | 0.00 (0.00 to 0.00) | 0.00 (0.00 to 0.00) | 0.00 (0.00 to 0.00) | 0.00 (0.00 to 0.00) | 0.00 (0.00 to 0.00) | 0.00 (0.00 to 0.00) | 0.00 (0.00 to 0.00) | 0.00 (0.00 to 0.00) | 0.00 (0.00 to 0.00) | 0.00 (0.00 to 0.00) | 0.00 (0.00 to 0.00) | 0.00 (0.00 to 0.00) | 0.00 (0.00 to 0.00) | 0.00 (0.00 to 0.00) | 0.00 (0.00 to 0.00) | 0.00 (0.00 to 0.00) | 0.00 (0.00 to 0.00) | 0.00 (0.00 to 0.00) | 0.00 (0.00 to 0.00) | 0.00 (0.00 to 0.00) |
| Thailand | Male | Number of prevalence | 1067.93 (57.11 to 5331.35) | 2956.62 (158.10 to 14760.16) | 4833.93 (258.49 to 24132.17) | 6829.34 (365.19 to 34093.73) | 8908.40 (476.37 to 44472.88) | 9951.53 (532.15 to 49680.46) | 10306.57 (551.14 to 51452.88) | 12842.70 (686.75 to 64113.88) | 15022.39 (803.31 to 74995.41) | 16163.55 (864.33 to 80692.34) | 16779.27 (897.26 to 83766.17) | 16119.75 (861.99 to 80473.72) | 14687.64 (785.41 to 73324.25) | 10907.23 (583.26 to 54451.55) | 7783.21 (416.20 to 38855.68) | 4952.41 (264.83 to 24723.63) | 3220.86 (172.23 to 16079.34) | 1721.65 (92.06 to 8594.91) | 773.92 (41.38 to 3863.60) | 300.95 (16.09 to 1502.40) |
| Timor-Leste | Both | Number of DALYs | 20.74 (13.04 to 30.48) | 120.69 (64.09 to 205.44) | 180.44 (99.60 to 302.01) | 271.76 (163.82 to 421.70) | 309.04 (198.23 to 458.16) | 280.86 (185.61 to 409.69) | 239.29 (160.36 to 347.46) | 201.14 (134.33 to 286.29) | 195.97 (129.24 to 281.30) | 200.77 (133.35 to 284.73) | 166.31 (111.14 to 233.88) | 128.96 (87.24 to 178.21) | 121.07 (82.10 to 168.84) | 101.35 (68.40 to 141.73) | 71.88 (49.51 to 99.54) | 49.57 (33.60 to 67.69) | 20.11 (13.79 to 27.45) | 7.73 (5.38 to 10.56) | 2.28 (1.59 to 3.09) | 0.42 (0.29 to 0.55) |
| Timor-Leste | Both | Number of prevalence | 2667.31 (232.09 to 13562.92) | 6774.87 (970.40 to 32185.82) | 9728.64 (1452.27 to 45988.07) | 13326.25 (2216.39 to 62094.43) | 14253.69 (2554.10 to 65758.77) | 12732.89 (2374.86 to 58504.25) | 10680.87 (2052.20 to 48890.88) | 8848.69 (1736.38 to 40341.63) | 8485.48 (1719.03 to 38522.32) | 8493.60 (1776.28 to 38404.59) | 6945.39 (1480.63 to 31339.49) | 5455.77 (1171.57 to 24668.13) | 5396.17 (1113.53 to 24684.85) | 4769.15 (949.40 to 22009.11) | 3504.04 (689.89 to 16205.92) | 2417.04 (484.97 to 11135.88) | 953.38 (199.35 to 4353.60) | 393.60 (78.46 to 1816.50) | 117.78 (23.58 to 542.99) | 22.49 (4.43 to 104.06) |
| Timor-Leste | Female | Number of DALYs | 11.72 (7.09 to 17.69) | 20.44 (12.65 to 31.45) | 22.52 (14.32 to 36.10) | 25.26 (15.28 to 38.73) | 25.64 (15.63 to 39.02) | 22.57 (13.85 to 34.98) | 18.70 (11.39 to 28.39) | 15.07 (9.71 to 22.28) | 13.65 (8.77 to 20.04) | 13.07 (8.15 to 19.75) | 10.08 (6.51 to 14.75) | 7.83 (5.17 to 11.32) | 8.40 (5.32 to 12.59) | 7.73 (4.98 to 11.48) | 5.67 (3.64 to 8.02) | 3.66 (2.42 to 5.31) | 1.32 (0.85 to 1.96) | 0.59 (0.39 to 0.82) | 0.17 (0.11 to 0.24) | 0.03 (0.02 to 0.05) |
| Timor-Leste | Female | Number of prevalence | 1310.72 (128.75 to 6592.42) | 2977.64 (232.10 to 15260.73) | 4189.91 (270.39 to 21733.00) | 5640.83 (324.12 to 29446.69) | 6097.35 (330.18 to 31920.60) | 5585.30 (295.49 to 29275.84) | 4680.67 (244.77 to 24547.92) | 3810.65 (198.07 to 19991.82) | 3539.61 (183.80 to 18573.57) | 3384.54 (175.07 to 17786.03) | 2667.87 (138.02 to 14049.52) | 2089.72 (108.27 to 11031.58) | 2238.45 (116.12 to 11848.72) | 2105.50 (109.37 to 11159.88) | 1577.49 (81.97 to 8360.94) | 1054.25 (54.79 to 5587.68) | 384.06 (19.95 to 2035.63) | 174.02 (9.05 to 922.33) | 51.58 (2.68 to 273.40) | 10.15 (0.53 to 53.81) |
| Timor-Leste | Male | Number of DALYs | 9.02 (4.91 to 14.38) | 100.25 (49.22 to 181.13) | 157.92 (82.89 to 270.98) | 246.50 (144.76 to 388.55) | 283.40 (177.82 to 422.60) | 258.28 (169.09 to 380.46) | 220.58 (145.89 to 321.50) | 186.07 (123.75 to 265.42) | 182.32 (119.12 to 264.57) | 187.70 (124.24 to 266.37) | 156.23 (104.03 to 221.14) | 121.13 (81.72 to 168.57) | 112.67 (76.03 to 158.08) | 93.61 (63.11 to 131.47) | 66.21 (44.76 to 91.47) | 45.91 (30.77 to 63.10) | 18.79 (12.87 to 25.80) | 7.14 (4.94 to 9.83) | 2.11 (1.47 to 2.86) | 0.38 (0.27 to 0.51) |
| Timor-Leste | Male | Number of prevalence | 1356.59 (103.61 to 6970.51) | 3797.24 (713.36 to 16925.09) | 5538.73 (1156.03 to 24255.08) | 7685.42 (1853.91 to 32647.74) | 8156.34 (2153.40 to 33838.17) | 7147.59 (2025.10 to 29228.41) | 6000.20 (1762.93 to 24342.96) | 5038.04 (1503.75 to 20349.82) | 4945.87 (1505.17 to 19948.75) | 5109.06 (1564.01 to 20618.56) | 4277.51 (1311.71 to 17289.97) | 3366.05 (1035.93 to 13636.54) | 3157.72 (976.11 to 12836.13) | 2663.65 (820.80 to 10849.23) | 1926.55 (595.54 to 7844.98) | 1362.79 (420.05 to 5548.19) | 569.32 (175.63 to 2317.97) | 219.58 (67.85 to 894.17) | 66.20 (20.42 to 269.59) | 12.34 (3.81 to 50.25) |
| Togo | Both | Number of DALYs | 0.00 (0.00 to 0.00) | 0.00 (0.00 to 0.00) | 0.00 (0.00 to 0.00) | 0.00 (0.00 to 0.00) | 0.00 (0.00 to 0.00) | 0.00 (0.00 to 0.00) | 0.00 (0.00 to 0.00) | 0.00 (0.00 to 0.00) | 0.00 (0.00 to 0.00) | 0.00 (0.00 to 0.00) | 0.00 (0.00 to 0.00) | 0.00 (0.00 to 0.00) | 0.00 (0.00 to 0.00) | 0.00 (0.00 to 0.00) | 0.00 (0.00 to 0.00) | 0.00 (0.00 to 0.00) | 0.00 (0.00 to 0.00) | 0.00 (0.00 to 0.00) | 0.00 (0.00 to 0.00) | 0.00 (0.00 to 0.00) |
| Togo | Both | Number of prevalence | 379.18 (45.23 to 1609.86) | 898.71 (107.20 to 3815.57) | 1168.77 (139.41 to 4962.11) | 1364.14 (162.71 to 5791.56) | 1434.94 (171.16 to 6092.18) | 1438.55 (171.59 to 6107.51) | 1394.80 (166.37 to 5921.77) | 1288.34 (153.67 to 5469.76) | 1146.72 (136.78 to 4868.52) | 990.44 (118.14 to 4205.00) | 803.12 (95.80 to 3409.73) | 643.87 (76.80 to 2733.61) | 476.44 (56.83 to 2022.76) | 343.05 (40.92 to 1456.43) | 225.38 (26.88 to 956.87) | 137.59 (16.41 to 584.15) | 62.33 (7.43 to 264.63) | 22.06 (2.63 to 93.65) | 6.24 (0.74 to 26.48) | 1.43 (0.17 to 6.07) |
| Togo | Female | Number of DALYs | 0.00 (0.00 to 0.00) | 0.00 (0.00 to 0.00) | 0.00 (0.00 to 0.00) | 0.00 (0.00 to 0.00) | 0.00 (0.00 to 0.00) | 0.00 (0.00 to 0.00) | 0.00 (0.00 to 0.00) | 0.00 (0.00 to 0.00) | 0.00 (0.00 to 0.00) | 0.00 (0.00 to 0.00) | 0.00 (0.00 to 0.00) | 0.00 (0.00 to 0.00) | 0.00 (0.00 to 0.00) | 0.00 (0.00 to 0.00) | 0.00 (0.00 to 0.00) | 0.00 (0.00 to 0.00) | 0.00 (0.00 to 0.00) | 0.00 (0.00 to 0.00) | 0.00 (0.00 to 0.00) | 0.00 (0.00 to 0.00) |
| Togo | Female | Number of prevalence | 186.46 (22.24 to 791.65) | 439.54 (52.43 to 1866.11) | 564.16 (67.29 to 2395.19) | 659.87 (78.71 to 2801.54) | 714.07 (85.17 to 3031.63) | 744.33 (88.78 to 3160.13) | 771.86 (92.07 to 3277.02) | 720.34 (85.92 to 3058.27) | 610.16 (72.78 to 2590.49) | 514.68 (61.39 to 2185.12) | 414.68 (49.46 to 1760.54) | 337.23 (40.22 to 1431.74) | 260.65 (31.09 to 1106.63) | 196.55 (23.44 to 834.47) | 136.73 (16.31 to 580.49) | 89.28 (10.65 to 379.03) | 41.04 (4.90 to 174.23) | 14.80 (1.76 to 62.82) | 4.42 (0.53 to 18.78) | 1.08 (0.13 to 4.59) |
| Togo | Male | Number of DALYs | 0.00 (0.00 to 0.00) | 0.00 (0.00 to 0.00) | 0.00 (0.00 to 0.00) | 0.00 (0.00 to 0.00) | 0.00 (0.00 to 0.00) | 0.00 (0.00 to 0.00) | 0.00 (0.00 to 0.00) | 0.00 (0.00 to 0.00) | 0.00 (0.00 to 0.00) | 0.00 (0.00 to 0.00) | 0.00 (0.00 to 0.00) | 0.00 (0.00 to 0.00) | 0.00 (0.00 to 0.00) | 0.00 (0.00 to 0.00) | 0.00 (0.00 to 0.00) | 0.00 (0.00 to 0.00) | 0.00 (0.00 to 0.00) | 0.00 (0.00 to 0.00) | 0.00 (0.00 to 0.00) | 0.00 (0.00 to 0.00) |
| Togo | Male | Number of prevalence | 192.72 (22.99 to 818.21) | 459.17 (54.77 to 1949.46) | 604.61 (72.12 to 2566.91) | 704.27 (84.00 to 2990.03) | 720.88 (85.98 to 3060.55) | 694.22 (82.81 to 2947.38) | 622.94 (74.30 to 2644.75) | 568.00 (67.75 to 2411.48) | 536.57 (64.00 to 2278.04) | 475.76 (56.75 to 2019.88) | 388.45 (46.33 to 1649.19) | 306.64 (36.58 to 1301.87) | 215.78 (25.74 to 916.13) | 146.50 (17.47 to 621.96) | 88.65 (10.57 to 376.37) | 48.31 (5.76 to 205.12) | 21.29 (2.54 to 90.40) | 7.26 (0.87 to 30.83) | 1.81 (0.22 to 7.70) | 0.35 (0.04 to 1.48) |
| Tonga | Both | Number of DALYs | 1.37 (0.84 to 2.08) | 3.54 (1.91 to 6.65) | 3.22 (1.71 to 5.72) | 2.94 (1.59 to 5.44) | 2.45 (1.32 to 4.42) | 1.98 (1.08 to 3.56) | 1.70 (0.90 to 3.02) | 1.58 (0.87 to 2.85) | 1.46 (0.80 to 2.75) | 1.33 (0.72 to 2.37) | 1.27 (0.72 to 2.36) | 1.03 (0.57 to 1.88) | 0.79 (0.43 to 1.43) | 0.60 (0.32 to 1.06) | 0.43 (0.24 to 0.77) | 0.30 (0.17 to 0.51) | 0.20 (0.11 to 0.36) | 0.10 (0.06 to 0.16) | 0.03 (0.02 to 0.05) | 0.01 (0.01 to 0.01) |
| Tonga | Both | Number of prevalence | 18.28 (12.73 to 50.19) | 43.13 (24.71 to 111.91) | 43.58 (22.54 to 132.79) | 44.49 (20.87 to 155.74) | 40.86 (17.73 to 156.14) | 35.64 (14.73 to 143.89) | 31.68 (12.64 to 132.47) | 30.84 (11.97 to 131.91) | 28.95 (10.94 to 125.62) | 27.32 (10.12 to 119.82) | 26.38 (9.58 to 116.78) | 22.03 (7.84 to 98.60) | 17.54 (6.12 to 79.72) | 13.73 (4.75 to 63.01) | 10.07 (3.49 to 46.36) | 7.24 (2.51 to 33.43) | 4.86 (1.69 to 22.59) | 2.58 (0.90 to 12.09) | 0.92 (0.33 to 4.38) | 0.25 (0.09 to 1.19) |
| Tonga | Female | Number of DALYs | 0.78 (0.44 to 1.18) | 1.18 (0.65 to 1.82) | 1.07 (0.60 to 1.67) | 0.97 (0.57 to 1.58) | 0.83 (0.50 to 1.29) | 0.70 (0.41 to 1.13) | 0.63 (0.35 to 0.99) | 0.59 (0.34 to 0.94) | 0.53 (0.31 to 0.87) | 0.46 (0.27 to 0.72) | 0.42 (0.24 to 0.64) | 0.34 (0.21 to 0.53) | 0.27 (0.15 to 0.42) | 0.21 (0.12 to 0.33) | 0.16 (0.09 to 0.25) | 0.11 (0.07 to 0.17) | 0.08 (0.05 to 0.12) | 0.04 (0.03 to 0.07) | 0.02 (0.01 to 0.03) | 0.01 (0.00 to 0.01) |
| Tonga | Female | Number of prevalence | 9.86 (7.08 to 25.20) | 16.70 (10.79 to 50.97) | 17.27 (9.80 to 62.19) | 18.22 (9.09 to 74.21) | 17.46 (7.89 to 76.67) | 16.07 (6.79 to 73.73) | 14.95 (6.04 to 70.47) | 14.70 (5.75 to 70.62) | 13.52 (5.15 to 65.91) | 12.35 (4.60 to 60.97) | 11.59 (4.22 to 57.82) | 9.62 (3.43 to 48.51) | 7.84 (2.73 to 39.99) | 6.38 (2.19 to 32.72) | 4.82 (1.66 to 24.72) | 3.58 (1.23 to 18.35) | 2.53 (0.87 to 12.95) | 1.44 (0.50 to 7.38) | 0.57 (0.20 to 2.94) | 0.18 (0.06 to 0.91) |
| Tonga | Male | Number of DALYs | 0.59 (0.31 to 1.00) | 2.36 (1.01 to 5.12) | 2.15 (0.98 to 4.48) | 1.97 (0.87 to 3.97) | 1.62 (0.72 to 3.42) | 1.28 (0.57 to 2.67) | 1.06 (0.46 to 2.25) | 0.99 (0.45 to 2.05) | 0.93 (0.42 to 1.97) | 0.87 (0.37 to 1.81) | 0.85 (0.38 to 1.81) | 0.69 (0.32 to 1.42) | 0.52 (0.24 to 1.08) | 0.39 (0.18 to 0.82) | 0.27 (0.13 to 0.58) | 0.18 (0.08 to 0.39) | 0.12 (0.05 to 0.25) | 0.06 (0.03 to 0.11) | 0.02 (0.01 to 0.04) | 0.00 (0.00 to 0.01) |
| Tonga | Male | Number of prevalence | 8.42 (5.55 to 25.02) | 26.43 (13.14 to 65.66) | 26.30 (11.97 to 74.35) | 26.26 (10.93 to 83.18) | 23.40 (9.17 to 79.73) | 19.57 (7.36 to 70.16) | 16.73 (6.11 to 61.99) | 16.14 (5.80 to 61.30) | 15.43 (5.46 to 59.71) | 14.97 (5.23 to 58.85) | 14.80 (5.10 to 58.96) | 12.41 (4.23 to 50.09) | 9.70 (3.26 to 39.73) | 7.35 (2.45 to 30.29) | 5.25 (1.75 to 21.64) | 3.66 (1.22 to 15.08) | 2.34 (0.78 to 9.64) | 1.14 (0.38 to 4.71) | 0.35 (0.12 to 1.44) | 0.07 (0.02 to 0.28) |
| Uganda | Both | Number of DALYs | 694.54 (424.25 to 1032.12) | 1049.35 (639.91 to 1620.00) | 1585.60 (855.21 to 2828.49) | 1358.25 (716.41 to 2549.36) | 1081.54 (597.97 to 1901.70) | 854.55 (477.95 to 1559.80) | 679.46 (370.10 to 1276.63) | 520.02 (282.64 to 928.03) | 415.81 (230.53 to 723.64) | 330.44 (185.23 to 573.08) | 255.33 (132.29 to 462.31) | 193.90 (106.97 to 341.31) | 144.30 (80.94 to 251.81) | 99.55 (57.26 to 172.74) | 63.68 (35.22 to 108.71) | 39.82 (22.35 to 68.40) | 21.30 (11.98 to 34.86) | 8.43 (5.02 to 13.43) | 2.28 (1.34 to 3.50) | 0.53 (0.33 to 0.84) |
| Uganda | Both | Number of prevalence | 7860.86 (6733.88 to 11199.94) | 12890.96 (10532.49 to 20447.97) | 17804.77 (12310.10 to 31399.02) | 16417.75 (10923.27 to 30922.85) | 13992.52 (9075.80 to 27221.33) | 11531.53 (7327.72 to 22876.95) | 9421.65 (5894.65 to 19322.66) | 7469.26 (4625.47 to 15607.73) | 6039.19 (3695.39 to 12777.32) | 4898.33 (2964.27 to 10479.24) | 3885.72 (2323.00 to 8399.36) | 3041.44 (1807.13 to 6635.11) | 2285.84 (1348.12 to 5043.69) | 1634.12 (962.11 to 3633.34) | 1071.49 (635.65 to 2387.01) | 686.89 (409.87 to 1534.55) | 380.10 (228.87 to 851.56) | 154.20 (93.53 to 347.49) | 44.19 (27.09 to 99.94) | 10.90 (6.74 to 24.57) |
| Uganda | Female | Number of DALYs | 398.48 (226.43 to 625.27) | 604.83 (335.94 to 969.00) | 540.70 (316.08 to 861.89) | 469.45 (277.54 to 752.76) | 389.96 (229.55 to 596.15) | 317.90 (183.88 to 497.08) | 254.76 (157.12 to 410.20) | 195.68 (120.09 to 305.16) | 155.34 (91.68 to 236.86) | 121.39 (70.67 to 197.41) | 93.15 (54.65 to 144.88) | 73.35 (42.88 to 106.86) | 56.69 (33.89 to 86.90) | 39.77 (22.34 to 61.71) | 27.41 (15.92 to 41.83) | 17.73 (10.26 to 26.95) | 10.15 (6.09 to 15.43) | 4.47 (2.72 to 6.79) | 1.41 (0.85 to 2.13) | 0.38 (0.23 to 0.55) |
| Uganda | Female | Number of prevalence | 4419.41 (3806.85 to 6029.19) | 7157.81 (5917.90 to 10760.47) | 6958.51 (5444.90 to 11680.43) | 6644.87 (4899.52 to 12326.21) | 5962.26 (4189.74 to 11801.89) | 5096.97 (3456.17 to 10511.46) | 4214.77 (2794.04 to 8902.81) | 3351.86 (2187.86 to 7220.86) | 2708.26 (1742.54 to 5919.76) | 2180.52 (1385.53 to 4828.68) | 1722.49 (1085.44 to 3852.31) | 1384.56 (863.62 to 3135.77) | 1072.07 (659.56 to 2455.47) | 787.95 (482.51 to 1815.18) | 546.37 (333.90 to 1259.73) | 365.81 (223.98 to 842.82) | 212.46 (129.72 to 489.54) | 93.51 (57.13 to 215.56) | 30.63 (18.72 to 70.64) | 8.44 (5.16 to 19.45) |
| Uganda | Male | Number of DALYs | 296.06 (160.17 to 485.94) | 444.52 (252.89 to 741.49) | 1044.91 (458.40 to 2147.78) | 888.81 (373.47 to 1980.20) | 691.58 (322.82 to 1393.92) | 536.65 (247.71 to 1110.57) | 424.70 (192.42 to 948.23) | 324.33 (133.51 to 681.80) | 260.47 (113.00 to 535.80) | 209.06 (90.51 to 432.35) | 162.18 (68.77 to 344.50) | 120.55 (53.48 to 262.32) | 87.61 (40.62 to 179.52) | 59.78 (27.54 to 124.20) | 36.27 (15.89 to 73.94) | 22.09 (9.92 to 47.97) | 11.16 (4.99 to 22.73) | 3.96 (1.80 to 8.30) | 0.87 (0.40 to 1.79) | 0.15 (0.07 to 0.32) |
| Uganda | Male | Number of prevalence | 3441.45 (2848.83 to 5121.70) | 5733.15 (4497.18 to 9575.13) | 10846.26 (6481.15 to 20279.67) | 9772.89 (5703.68 to 18926.46) | 8030.26 (4589.63 to 16082.42) | 6434.56 (3622.49 to 13288.29) | 5206.88 (2913.06 to 10984.53) | 4117.40 (2285.59 to 8756.72) | 3330.93 (1835.15 to 7128.48) | 2717.81 (1490.89 to 5912.86) | 2163.23 (1179.15 to 4643.83) | 1656.88 (900.30 to 3673.79) | 1213.76 (653.27 to 2643.29) | 846.17 (454.64 to 1898.08) | 525.12 (282.45 to 1176.39) | 321.07 (172.41 to 702.80) | 167.63 (90.22 to 375.06) | 60.69 (32.60 to 136.02) | 13.56 (7.28 to 30.38) | 2.46 (1.32 to 5.51) |
| United Republic of Tanzania | Both | Number of DALYs | 839.53 (515.49 to 1292.74) | 2218.22 (1163.37 to 4061.41) | 2019.95 (1091.99 to 3676.04) | 1805.98 (952.42 to 3252.51) | 1513.21 (811.94 to 2723.68) | 1250.19 (705.04 to 2269.55) | 1051.19 (574.07 to 1818.35) | 901.68 (495.42 to 1600.36) | 755.09 (416.91 to 1309.99) | 618.99 (335.77 to 1116.08) | 490.15 (262.26 to 888.45) | 373.94 (205.01 to 686.07) | 279.36 (149.89 to 512.27) | 206.50 (112.59 to 371.74) | 133.70 (73.27 to 235.01) | 84.65 (46.65 to 148.46) | 47.28 (27.01 to 82.35) | 19.89 (11.31 to 34.24) | 5.51 (3.23 to 9.15) | 1.28 (0.78 to 2.05) |
| United Republic of Tanzania | Both | Number of prevalence | 13378.52 (10679.83 to 18721.32) | 32069.50 (22655.27 to 49634.27) | 34175.37 (23194.38 to 53958.82) | 34989.80 (22954.52 to 56839.96) | 33096.18 (21078.41 to 54998.63) | 29564.29 (18599.29 to 49669.52) | 26032.50 (16212.39 to 44192.26) | 23142.35 (14307.58 to 39574.51) | 20099.35 (12348.24 to 34565.53) | 16801.41 (10253.99 to 29023.84) | 13665.94 (8291.22 to 23687.24) | 10638.24 (6424.04 to 18494.24) | 8191.90 (4917.78 to 14306.24) | 6200.31 (3721.76 to 10852.44) | 4137.40 (2479.22 to 7250.35) | 2722.38 (1633.34 to 4778.20) | 1578.52 (948.41 to 2776.73) | 685.75 (412.40 to 1209.86) | 198.76 (119.49 to 352.58) | 48.88 (29.32 to 86.88) |
| United Republic of Tanzania | Female | Number of DALYs | 488.64 (281.71 to 760.45) | 772.47 (459.14 to 1210.83) | 694.87 (417.57 to 1085.22) | 628.13 (339.52 to 985.18) | 541.98 (331.48 to 849.02) | 455.27 (262.55 to 694.85) | 371.17 (214.24 to 593.29) | 309.78 (183.92 to 477.04) | 259.48 (149.05 to 404.50) | 213.10 (130.02 to 328.16) | 162.28 (93.75 to 252.53) | 122.34 (72.45 to 182.90) | 89.57 (51.74 to 137.54) | 65.53 (38.82 to 106.70) | 45.06 (26.67 to 72.61) | 30.11 (17.28 to 45.25) | 18.32 (11.07 to 27.76) | 8.11 (4.90 to 12.16) | 2.50 (1.47 to 3.93) | 0.67 (0.41 to 0.98) |
| United Republic of Tanzania | Female | Number of prevalence | 7333.09 (5903.51 to 10006.29) | 13440.58 (10172.86 to 19698.64) | 14737.22 (10504.51 to 22930.84) | 15717.87 (10602.50 to 25594.91) | 15639.53 (10124.49 to 26167.76) | 14370.41 (9079.58 to 24441.61) | 12429.42 (7743.53 to 21329.96) | 10887.40 (6716.19 to 18821.69) | 9565.11 (5848.07 to 16625.76) | 7968.12 (4833.24 to 13908.28) | 6351.90 (3832.35 to 11129.66) | 4884.29 (2928.32 to 8599.15) | 3718.83 (2217.99 to 6574.54) | 2786.78 (1654.15 to 4932.31) | 1944.09 (1154.49 to 3443.02) | 1340.96 (797.10 to 2374.76) | 823.10 (488.95 to 1457.78) | 376.98 (223.74 to 667.52) | 117.88 (69.99 to 208.75) | 32.29 (19.16 to 57.19) |
| United Republic of Tanzania | Male | Number of DALYs | 350.89 (195.74 to 572.04) | 1445.74 (614.60 to 3172.56) | 1325.09 (575.44 to 2753.91) | 1177.85 (500.72 to 2592.35) | 971.23 (411.02 to 2023.58) | 794.93 (348.94 to 1669.19) | 680.02 (295.59 to 1401.15) | 591.90 (242.08 to 1223.09) | 495.61 (215.87 to 987.09) | 405.89 (176.79 to 818.49) | 327.88 (150.40 to 666.96) | 251.60 (113.09 to 528.15) | 189.78 (84.57 to 393.73) | 140.98 (62.23 to 284.45) | 88.64 (39.75 to 181.02) | 54.54 (24.66 to 112.81) | 28.95 (13.09 to 59.23) | 11.79 (5.18 to 24.30) | 3.01 (1.31 to 6.16) | 0.61 (0.29 to 1.26) |
| United Republic of Tanzania | Male | Number of prevalence | 6045.42 (4583.61 to 8645.75) | 18628.92 (11962.06 to 32179.05) | 19438.15 (12362.52 to 32961.10) | 19271.93 (11840.63 to 32570.79) | 17456.65 (10424.08 to 30050.56) | 15193.87 (9027.92 to 26155.91) | 13603.08 (8063.96 to 23351.03) | 12254.95 (7238.95 to 21080.22) | 10534.24 (6210.30 to 18140.51) | 8833.29 (5190.33 to 15211.96) | 7314.05 (4281.21 to 12609.37) | 5753.95 (3357.72 to 9923.54) | 4473.07 (2597.71 to 7750.54) | 3413.53 (1981.24 to 5925.09) | 2193.31 (1273.36 to 3806.44) | 1381.43 (801.81 to 2397.59) | 755.42 (438.77 to 1310.12) | 308.77 (178.87 to 535.68) | 80.88 (46.92 to 140.32) | 16.59 (9.63 to 28.79) |
| Vanuatu | Both | Number of DALYs | 4.19 (2.57 to 6.38) | 13.66 (7.21 to 25.54) | 13.47 (7.33 to 23.60) | 13.69 (7.31 to 23.95) | 13.93 (7.49 to 24.22) | 13.44 (7.31 to 24.04) | 11.95 (6.38 to 20.86) | 10.94 (5.88 to 18.70) | 10.37 (5.72 to 18.33) | 9.15 (4.85 to 15.73) | 7.46 (3.89 to 12.49) | 6.06 (3.28 to 10.23) | 4.62 (2.52 to 7.86) | 3.65 (1.96 to 6.10) | 2.04 (1.19 to 3.44) | 1.27 (0.73 to 2.19) | 0.58 (0.33 to 0.98) | 0.19 (0.11 to 0.31) | 0.04 (0.03 to 0.07) | 0.01 (0.00 to 0.01) |
| Vanuatu | Both | Number of prevalence | 198.25 (40.43 to 1415.01) | 489.29 (92.65 to 3343.65) | 584.20 (89.37 to 4194.01) | 685.50 (90.73 to 5064.36) | 756.77 (92.39 to 5675.97) | 762.42 (89.97 to 5754.89) | 694.46 (80.68 to 5276.56) | 641.46 (74.04 to 4900.50) | 603.58 (70.04 to 4632.33) | 537.73 (62.46 to 4158.31) | 446.09 (51.61 to 3479.45) | 366.01 (42.28 to 2882.75) | 284.64 (33.06 to 2267.15) | 226.86 (26.34 to 1816.01) | 129.66 (15.07 to 1037.80) | 84.89 (9.62 to 682.97) | 39.12 (4.47 to 314.36) | 13.00 (1.46 to 104.69) | 3.23 (0.36 to 26.15) | 0.54 (0.06 to 4.42) |
| Vanuatu | Female | Number of DALYs | 2.40 (1.37 to 3.89) | 3.90 (2.29 to 6.07) | 3.47 (1.94 to 5.63) | 3.23 (1.98 to 5.17) | 3.19 (1.98 to 4.89) | 2.94 (1.77 to 4.51) | 2.50 (1.57 to 3.73) | 2.19 (1.30 to 3.28) | 1.93 (1.15 to 3.06) | 1.63 (0.98 to 2.44) | 1.34 (0.79 to 2.02) | 1.09 (0.65 to 1.62) | 0.82 (0.51 to 1.27) | 0.64 (0.40 to 0.95) | 0.36 (0.22 to 0.55) | 0.25 (0.15 to 0.36) | 0.11 (0.06 to 0.16) | 0.04 (0.02 to 0.06) | 0.01 (0.01 to 0.01) | 0.00 (0.00 to 0.00) |
| Vanuatu | Female | Number of prevalence | 99.20 (22.40 to 686.73) | 215.04 (36.52 to 1586.77) | 257.26 (32.86 to 1985.27) | 306.71 (31.07 to 2432.02) | 352.24 (30.77 to 2833.47) | 359.00 (28.66 to 2909.35) | 328.64 (24.86 to 2683.29) | 300.89 (21.83 to 2474.36) | 273.24 (19.29 to 2264.66) | 241.67 (16.69 to 2021.05) | 203.41 (13.78 to 1715.46) | 168.29 (11.19 to 1433.68) | 131.29 (8.55 to 1131.78) | 104.16 (6.73 to 903.21) | 59.40 (3.83 to 515.06) | 41.76 (2.70 to 362.15) | 18.90 (1.22 to 163.87) | 6.50 (0.42 to 56.33) | 1.71 (0.11 to 14.85) | 0.31 (0.02 to 2.73) |
| Vanuatu | Male | Number of DALYs | 1.79 (1.01 to 2.74) | 9.76 (4.10 to 20.71) | 10.00 (4.70 to 19.51) | 10.46 (4.72 to 20.03) | 10.73 (4.95 to 20.57) | 10.50 (5.19 to 20.41) | 9.46 (4.39 to 17.74) | 8.76 (4.39 to 16.17) | 8.44 (4.20 to 15.94) | 7.52 (3.71 to 13.73) | 6.13 (2.98 to 10.84) | 4.97 (2.41 to 9.23) | 3.80 (1.91 to 6.75) | 3.01 (1.57 to 5.28) | 1.68 (0.88 to 2.99) | 1.03 (0.53 to 1.85) | 0.47 (0.24 to 0.84) | 0.15 (0.08 to 0.27) | 0.03 (0.02 to 0.06) | 0.00 (0.00 to 0.01) |
| Vanuatu | Male | Number of prevalence | 99.05 (17.13 to 728.28) | 274.25 (53.62 to 1756.87) | 326.95 (54.38 to 2208.74) | 378.79 (57.34 to 2632.34) | 404.52 (59.11 to 2842.50) | 403.43 (58.83 to 2845.54) | 365.82 (53.64 to 2593.27) | 340.57 (49.93 to 2426.14) | 330.33 (48.71 to 2367.67) | 296.06 (43.99 to 2137.26) | 242.69 (36.25 to 1763.99) | 197.72 (29.71 to 1449.06) | 153.35 (23.36 to 1135.36) | 122.70 (18.78 to 912.80) | 70.27 (10.74 to 522.74) | 43.12 (6.60 to 320.82) | 20.23 (3.09 to 150.50) | 6.50 (0.99 to 48.35) | 1.52 (0.23 to 11.30) | 0.23 (0.03 to 1.69) |
| Viet Nam | Both | Number of DALYs | 803.34 (494.20 to 1231.99) | 2461.86 (1291.76 to 4594.75) | 2281.52 (1195.19 to 4181.39) | 2011.44 (1044.34 to 3486.17) | 1950.78 (1056.41 to 3566.26) | 2459.51 (1286.88 to 4686.90) | 2763.48 (1464.90 to 5063.20) | 2536.64 (1348.79 to 4702.75) | 2240.91 (1237.37 to 4026.74) | 2023.07 (1072.76 to 3595.91) | 1826.77 (970.99 to 3248.72) | 1614.16 (894.73 to 2902.58) | 1333.91 (747.03 to 2473.28) | 916.27 (520.84 to 1623.40) | 518.47 (287.39 to 906.78) | 326.05 (190.37 to 548.70) | 196.30 (118.03 to 327.93) | 99.93 (58.09 to 159.30) | 31.12 (18.98 to 49.70) | 7.21 (4.39 to 11.23) |
| Viet Nam | Both | Number of prevalence | 14832.66 (8134.20 to 45151.94) | 40122.70 (19535.89 to 117618.71) | 43978.35 (18682.75 to 145385.46) | 46069.60 (17495.45 to 166275.60) | 49991.17 (17383.61 to 189723.74) | 67227.46 (22232.68 to 262340.88) | 79032.20 (25346.73 to 313429.13) | 76497.42 (23899.34 to 307020.10) | 69459.18 (21310.04 to 281481.05) | 64785.88 (19598.77 to 264545.96) | 60971.88 (18157.36 to 250866.08) | 55941.71 (16357.28 to 232244.38) | 48252.55 (13856.00 to 202067.63) | 34929.20 (9848.18 to 147161.65) | 20100.79 (5629.09 to 84841.22) | 13199.18 (3657.40 to 55968.00) | 8466.74 (2339.29 to 36183.13) | 4551.42 (1242.09 to 19586.97) | 1520.67 (404.59 to 6607.11) | 393.95 (100.58 to 1736.88) |
| Viet Nam | Female | Number of DALYs | 452.30 (264.81 to 678.33) | 796.63 (466.25 to 1245.90) | 733.49 (431.61 to 1126.82) | 632.47 (373.97 to 969.64) | 613.77 (354.98 to 955.80) | 763.42 (436.08 to 1249.68) | 832.99 (500.89 to 1323.60) | 774.57 (460.55 to 1220.05) | 693.28 (420.58 to 1079.08) | 615.20 (357.94 to 950.23) | 574.87 (320.54 to 879.24) | 530.30 (314.93 to 840.88) | 450.77 (264.62 to 719.48) | 331.47 (194.10 to 502.32) | 195.33 (118.76 to 306.26) | 132.96 (77.34 to 200.66) | 91.62 (52.34 to 140.49) | 51.91 (30.22 to 78.46) | 18.48 (10.91 to 27.85) | 5.26 (3.16 to 7.89) |
| Viet Nam | Female | Number of prevalence | 7779.42 (4535.20 to 22286.15) | 16319.74 (8127.95 to 53571.28) | 18387.00 (7716.47 to 67431.48) | 19740.66 (7029.67 to 78302.39) | 21760.53 (6902.32 to 90276.17) | 29524.66 (8702.91 to 125550.25) | 34847.59 (9836.39 to 150242.46) | 34027.59 (9247.62 to 148285.06) | 31188.59 (8253.92 to 137008.03) | 29280.22 (7562.62 to 129448.02) | 28128.11 (7117.53 to 125070.75) | 26915.25 (6645.47 to 120354.78) | 24021.83 (5801.07 to 108028.32) | 18205.45 (4341.87 to 82080.65) | 10742.08 (2562.12 to 48444.23) | 7511.58 (1797.32 to 33868.45) | 5321.62 (1268.81 to 23991.04) | 3083.85 (735.43 to 13904.22) | 1129.24 (269.52 to 5091.48) | 332.22 (79.21 to 1497.90) |
| Viet Nam | Male | Number of DALYs | 351.04 (183.44 to 576.41) | 1665.23 (737.17 to 3738.85) | 1548.03 (672.25 to 3302.86) | 1378.96 (568.33 to 2875.91) | 1337.02 (593.84 to 2836.56) | 1696.09 (723.69 to 3502.43) | 1930.49 (858.45 to 4027.80) | 1762.07 (757.12 to 3696.12) | 1547.63 (686.70 to 3296.68) | 1407.87 (636.47 to 2902.34) | 1251.90 (545.15 to 2558.09) | 1083.86 (485.40 to 2215.66) | 883.14 (398.20 to 1904.54) | 584.80 (270.81 to 1168.42) | 323.15 (145.47 to 670.29) | 193.09 (89.87 to 393.94) | 104.68 (49.04 to 206.62) | 48.02 (22.52 to 101.89) | 12.64 (5.81 to 26.00) | 1.94 (0.82 to 4.02) |
| Viet Nam | Male | Number of prevalence | 7053.23 (3537.57 to 22865.78) | 23802.96 (10629.32 to 64429.46) | 25591.36 (10378.43 to 78360.30) | 26328.94 (9763.35 to 88377.21) | 28230.65 (9750.96 to 99935.17) | 37702.80 (12471.41 to 137482.68) | 44184.61 (14289.17 to 163976.26) | 42469.83 (13463.51 to 159548.71) | 38270.59 (11911.53 to 145226.97) | 35505.66 (10896.47 to 135795.86) | 32843.77 (9959.60 to 126484.93) | 29026.46 (8669.88 to 112558.09) | 24230.72 (7134.74 to 94622.23) | 16723.75 (4866.59 to 65530.95) | 9358.72 (2718.48 to 36666.16) | 5687.59 (1651.91 to 22283.58) | 3145.12 (915.43 to 12321.72) | 1467.58 (425.98 to 5750.53) | 391.44 (114.21 to 1533.51) | 61.73 (17.93 to 241.82) |
| Yemen | Both | Number of DALYs | 438.45 (278.00 to 667.78) | 1248.03 (652.17 to 2318.49) | 1186.19 (642.74 to 2134.23) | 996.45 (528.80 to 1781.66) | 774.41 (415.68 to 1392.61) | 681.92 (362.45 to 1286.17) | 654.76 (348.53 to 1197.03) | 587.65 (310.45 to 1064.39) | 466.33 (244.04 to 834.34) | 329.00 (165.93 to 602.14) | 235.28 (128.33 to 417.69) | 179.63 (97.48 to 328.72) | 141.55 (80.04 to 253.87) | 107.36 (57.64 to 195.22) | 70.54 (39.42 to 126.16) | 43.80 (24.25 to 77.40) | 21.91 (12.16 to 38.61) | 8.62 (4.88 to 15.26) | 2.46 (1.45 to 4.21) | 0.56 (0.34 to 0.91) |
| Yemen | Both | Number of prevalence | 5714.53 (4385.45 to 11550.59) | 14619.80 (9332.24 to 29570.80) | 15377.28 (9151.25 to 35775.76) | 14189.16 (7853.60 to 36625.11) | 12062.71 (6305.29 to 33568.27) | 11248.62 (5635.27 to 32860.61) | 11183.83 (5464.03 to 33628.47) | 10323.66 (4930.45 to 31654.24) | 8412.22 (3944.87 to 26192.03) | 6077.45 (2802.78 to 19189.81) | 4435.39 (2017.30 to 14214.30) | 3512.02 (1578.20 to 11419.50) | 2866.97 (1268.20 to 9449.00) | 2220.16 (976.82 to 7369.41) | 1482.12 (649.06 to 4897.60) | 929.50 (407.66 to 3079.14) | 476.39 (209.37 to 1579.22) | 192.16 (84.62 to 639.83) | 56.85 (25.19 to 190.91) | 13.68 (6.07 to 46.90) |
| Yemen | Female | Number of DALYs | 251.87 (149.32 to 399.65) | 424.74 (251.62 to 678.84) | 399.47 (236.85 to 655.26) | 335.29 (200.63 to 523.68) | 260.98 (150.84 to 409.05) | 233.11 (141.64 to 367.64) | 223.91 (129.19 to 348.10) | 197.89 (116.55 to 311.94) | 153.32 (92.51 to 236.66) | 107.77 (57.31 to 161.79) | 78.36 (47.32 to 121.46) | 61.94 (38.70 to 97.22) | 49.00 (29.07 to 74.52) | 37.88 (22.15 to 59.05) | 23.67 (14.52 to 36.12) | 15.08 (9.33 to 22.69) | 7.69 (4.75 to 11.27) | 3.20 (1.91 to 4.76) | 0.99 (0.61 to 1.51) | 0.28 (0.18 to 0.42) |
| Yemen | Female | Number of prevalence | 3155.64 (2458.75 to 6036.29) | 5751.29 (4146.97 to 12736.65) | 6145.24 (3983.00 to 15708.69) | 5825.47 (3407.32 to 16751.96) | 5118.54 (2770.69 to 15843.15) | 4866.80 (2494.70 to 15747.40) | 4885.38 (2413.02 to 16238.99) | 4485.52 (2161.05 to 15204.55) | 3602.93 (1691.61 to 12403.83) | 2603.09 (1201.86 to 9078.32) | 1942.38 (876.39 to 6849.33) | 1571.14 (698.87 to 5601.91) | 1285.48 (559.72 to 4637.27) | 1007.29 (433.44 to 3653.96) | 641.77 (276.86 to 2328.07) | 411.88 (177.98 to 1494.54) | 213.34 (92.06 to 773.61) | 90.04 (38.88 to 326.67) | 28.86 (12.46 to 104.72) | 8.31 (3.58 to 30.16) |
| Yemen | Male | Number of DALYs | 186.58 (104.17 to 304.77) | 823.29 (353.82 to 1642.96) | 786.72 (332.17 to 1640.46) | 661.16 (285.53 to 1397.31) | 513.42 (222.07 to 1069.70) | 448.80 (194.19 to 988.17) | 430.85 (184.58 to 908.29) | 389.77 (169.47 to 834.30) | 313.00 (129.09 to 628.22) | 221.23 (96.16 to 483.18) | 156.92 (73.29 to 328.11) | 117.69 (52.69 to 257.31) | 92.55 (43.22 to 192.15) | 69.48 (30.18 to 152.98) | 46.88 (20.27 to 96.31) | 28.72 (13.16 to 57.81) | 14.22 (6.50 to 29.58) | 5.42 (2.49 to 11.14) | 1.47 (0.72 to 3.09) | 0.28 (0.13 to 0.58) |
| Yemen | Male | Number of prevalence | 2558.90 (1842.64 to 5516.94) | 8868.51 (4924.84 to 17824.73) | 9232.03 (4911.92 to 20066.18) | 8363.69 (4196.54 to 20082.54) | 6944.16 (3318.13 to 18037.64) | 6381.82 (2946.69 to 17405.75) | 6298.45 (2845.85 to 17640.09) | 5838.14 (2594.86 to 16498.88) | 4809.29 (2108.50 to 13808.17) | 3474.35 (1510.29 to 10099.95) | 2493.01 (1071.22 to 7339.06) | 1940.88 (823.60 to 5780.62) | 1581.49 (661.55 to 4770.76) | 1212.87 (502.90 to 3678.28) | 840.35 (348.74 to 2548.58) | 517.62 (215.01 to 1569.52) | 263.06 (109.44 to 798.42) | 102.12 (42.40 to 309.81) | 27.98 (11.65 to 84.87) | 5.36 (2.23 to 16.27) |
| Zambia | Both | Number of DALYs | 289.30 (177.53 to 454.09) | 884.97 (461.20 to 1559.20) | 868.26 (474.44 to 1575.26) | 826.98 (430.46 to 1483.49) | 767.88 (412.04 to 1356.47) | 694.85 (382.31 to 1241.15) | 604.80 (328.56 to 1088.75) | 514.02 (278.81 to 924.65) | 439.77 (240.62 to 796.62) | 353.86 (187.44 to 619.81) | 263.94 (146.45 to 460.32) | 189.73 (102.32 to 324.78) | 138.22 (75.96 to 246.76) | 95.04 (53.96 to 155.60) | 63.09 (34.27 to 104.64) | 38.97 (21.63 to 66.16) | 18.50 (10.71 to 31.05) | 6.62 (3.65 to 11.00) | 1.49 (0.86 to 2.40) | 0.26 (0.15 to 0.41) |
| Zambia | Both | Number of prevalence | 11319.19 (4519.75 to 30588.94) | 28052.38 (11668.92 to 75233.30) | 34193.49 (12970.66 to 95765.45) | 38222.09 (13567.43 to 110062.22) | 39451.62 (13422.24 to 115261.28) | 37931.41 (12690.79 to 111510.66) | 33772.32 (11193.20 to 99628.51) | 29274.33 (9657.39 to 86483.50) | 24967.44 (8227.65 to 73797.63) | 20159.50 (6649.07 to 59562.07) | 15308.73 (5037.58 to 45272.46) | 11336.70 (3701.78 to 33624.60) | 8647.29 (2802.23 to 25730.04) | 6138.97 (1982.29 to 18295.80) | 4205.53 (1355.85 to 12542.24) | 2702.25 (866.30 to 8069.67) | 1327.37 (423.37 to 3968.08) | 493.50 (156.85 to 1476.76) | 116.01 (36.54 to 347.87) | 21.94 (6.76 to 66.06) |
| Zambia | Female | Number of DALYs | 168.23 (92.55 to 269.23) | 275.00 (161.01 to 425.47) | 256.09 (151.28 to 404.10) | 230.61 (135.44 to 356.07) | 204.25 (120.25 to 319.19) | 175.98 (106.69 to 269.39) | 147.69 (85.28 to 225.01) | 116.34 (71.84 to 177.80) | 92.64 (57.65 to 139.01) | 69.99 (40.90 to 105.63) | 50.57 (30.36 to 75.95) | 38.35 (23.44 to 58.22) | 29.73 (17.87 to 45.97) | 21.59 (13.68 to 31.75) | 14.61 (8.82 to 22.03) | 9.49 (5.60 to 14.21) | 4.80 (2.97 to 7.09) | 1.78 (1.08 to 2.68) | 0.44 (0.27 to 0.66) | 0.09 (0.05 to 0.13) |
| Zambia | Female | Number of prevalence | 5866.74 (2503.92 to 15415.14) | 12733.22 (4723.55 to 35475.94) | 15788.54 (5197.71 to 45905.65) | 18035.29 (5472.35 to 53838.51) | 18974.16 (5481.07 to 57456.45) | 18240.66 (5128.15 to 55654.43) | 16021.97 (4430.24 to 49099.99) | 13664.01 (3735.29 to 42002.11) | 11257.07 (3048.03 to 34684.99) | 8723.39 (2345.30 to 26926.65) | 6620.72 (1769.82 to 20470.79) | 5128.58 (1361.22 to 15882.15) | 4126.61 (1089.25 to 12800.79) | 3044.67 (801.10 to 9451.27) | 2126.51 (559.37 to 6601.08) | 1415.37 (372.25 to 4393.86) | 716.75 (188.49 to 2224.84) | 272.84 (71.72 to 847.04) | 67.58 (17.77 to 209.80) | 14.06 (3.70 to 43.64) |
| Zambia | Male | Number of DALYs | 121.07 (69.48 to 196.88) | 609.97 (260.22 to 1239.20) | 612.17 (271.03 to 1258.49) | 596.37 (273.40 to 1207.18) | 563.63 (258.52 to 1141.22) | 518.87 (242.57 to 1048.08) | 457.11 (213.91 to 929.50) | 397.68 (186.15 to 783.07) | 347.13 (165.38 to 672.51) | 283.87 (134.82 to 531.68) | 213.37 (100.45 to 394.46) | 151.37 (75.24 to 274.97) | 108.49 (51.26 to 211.29) | 73.45 (35.57 to 134.33) | 48.48 (23.37 to 88.96) | 29.47 (14.63 to 55.07) | 13.70 (7.06 to 25.42) | 4.84 (2.40 to 8.79) | 1.05 (0.53 to 1.92) | 0.17 (0.09 to 0.31) |
| Zambia | Male | Number of prevalence | 5452.44 (2036.46 to 15173.80) | 15319.15 (6380.99 to 39496.21) | 18404.95 (7288.44 to 49566.80) | 20186.80 (7653.53 to 55859.27) | 20477.46 (7589.66 to 57425.63) | 19690.74 (7198.08 to 55494.08) | 17750.35 (6439.78 to 50209.94) | 15610.31 (5652.17 to 44206.21) | 13710.37 (4954.04 to 38890.24) | 11436.11 (4125.29 to 32461.52) | 8688.01 (3129.03 to 24669.31) | 6208.12 (2237.43 to 17638.93) | 4520.68 (1626.37 to 12857.63) | 3094.30 (1115.33 to 8799.74) | 2079.02 (747.99 to 5913.29) | 1286.88 (462.44 to 3660.06) | 610.62 (219.68 to 1736.52) | 220.66 (79.33 to 627.49) | 48.43 (17.42 to 137.72) | 7.88 (2.84 to 22.42) |
| Zimbabwe | Both | Number of DALYs | 213.86 (135.69 to 318.85) | 592.02 (306.55 to 1067.30) | 564.42 (311.50 to 1022.00) | 501.29 (265.83 to 919.85) | 436.81 (236.44 to 801.03) | 364.14 (202.73 to 654.91) | 317.86 (167.74 to 575.79) | 291.08 (160.87 to 519.86) | 246.08 (131.04 to 438.29) | 190.52 (105.70 to 356.16) | 138.38 (78.73 to 239.01) | 100.21 (55.26 to 177.65) | 82.17 (46.47 to 136.83) | 66.18 (37.15 to 115.30) | 40.11 (23.34 to 67.58) | 21.13 (12.42 to 35.20) | 8.98 (5.29 to 14.99) | 3.01 (1.68 to 5.00) | 0.68 (0.39 to 1.15) | 0.12 (0.07 to 0.20) |
| Zimbabwe | Both | Number of prevalence | 4349.95 (2257.51 to 11827.17) | 10825.63 (5205.05 to 28275.20) | 12390.04 (5222.33 to 35895.97) | 13090.46 (4871.05 to 40885.93) | 12984.52 (4439.49 to 42342.98) | 11779.05 (3825.00 to 39371.41) | 10862.41 (3416.71 to 36835.67) | 10407.65 (3206.12 to 35616.02) | 9077.83 (2749.12 to 31241.39) | 7218.73 (2155.39 to 24949.67) | 5443.47 (1600.12 to 18949.98) | 4138.85 (1191.81 to 14565.49) | 3569.74 (1000.04 to 12684.76) | 2962.50 (822.03 to 10556.58) | 1832.93 (506.29 to 6538.13) | 989.04 (273.87 to 3525.40) | 428.28 (118.91 to 1526.14) | 144.68 (40.12 to 515.58) | 34.04 (9.30 to 121.75) | 6.49 (1.73 to 23.43) |
| Zimbabwe | Female | Number of DALYs | 125.31 (75.61 to 195.00) | 200.73 (114.18 to 318.97) | 186.95 (108.23 to 288.32) | 165.32 (95.45 to 258.27) | 146.37 (87.55 to 230.56) | 123.50 (75.30 to 183.78) | 108.24 (63.71 to 170.91) | 96.44 (59.83 to 140.94) | 78.76 (46.03 to 124.96) | 58.86 (35.13 to 91.32) | 44.40 (26.43 to 68.43) | 36.06 (21.91 to 54.33) | 31.80 (17.13 to 50.10) | 25.57 (15.24 to 38.45) | 16.01 (9.70 to 24.51) | 8.29 (5.18 to 12.86) | 3.48 (2.08 to 5.21) | 1.17 (0.67 to 1.71) | 0.28 (0.17 to 0.44) | 0.06 (0.04 to 0.09) |
| Zimbabwe | Female | Number of prevalence | 2336.00 (1290.35 to 6018.13) | 4690.23 (2198.73 to 13596.64) | 5495.15 (2151.84 to 17506.72) | 5974.86 (1998.55 to 20308.58) | 6150.04 (1855.63 to 21657.62) | 5753.86 (1626.99 to 20666.19) | 5375.95 (1451.34 to 19541.87) | 5127.34 (1339.54 to 18792.15) | 4364.75 (1112.26 to 16101.12) | 3391.30 (846.16 to 12575.04) | 2637.20 (645.49 to 9822.13) | 2185.12 (524.45 to 8174.04) | 2002.48 (470.46 to 7527.56) | 1673.30 (390.57 to 6302.54) | 1049.95 (244.78 to 3954.62) | 561.22 (130.71 to 2113.90) | 241.97 (56.48 to 911.32) | 81.78 (19.05 to 308.03) | 20.19 (4.71 to 76.06) | 4.31 (1.00 to 16.25) |
| Zimbabwe | Male | Number of DALYs | 88.55 (48.79 to 142.35) | 391.29 (168.52 to 834.22) | 377.46 (172.41 to 795.56) | 335.98 (149.84 to 699.92) | 290.44 (125.06 to 611.94) | 240.64 (108.89 to 489.79) | 209.62 (89.59 to 433.60) | 194.64 (85.07 to 405.98) | 167.32 (75.21 to 345.65) | 131.66 (61.29 to 280.13) | 93.98 (43.66 to 185.54) | 64.15 (28.09 to 130.48) | 50.38 (24.30 to 103.33) | 40.61 (18.03 to 80.12) | 24.10 (10.86 to 46.91) | 12.84 (6.06 to 25.53) | 5.50 (2.68 to 11.15) | 1.84 (0.82 to 3.73) | 0.40 (0.19 to 0.78) | 0.06 (0.03 to 0.12) |
| Zimbabwe | Male | Number of prevalence | 2013.94 (957.04 to 5811.61) | 6135.40 (2734.10 to 15443.25) | 6894.90 (2775.63 to 18707.08) | 7115.60 (2666.82 to 20687.43) | 6834.47 (2428.91 to 20725.33) | 6025.19 (2070.17 to 18722.29) | 5486.46 (1840.67 to 17310.71) | 5280.31 (1741.75 to 16838.79) | 4713.08 (1534.21 to 15152.65) | 3827.44 (1233.73 to 12384.17) | 2806.27 (894.65 to 9134.59) | 1953.73 (615.97 to 6395.86) | 1567.26 (488.56 to 5160.67) | 1289.20 (400.10 to 4256.77) | 782.98 (243.46 to 2585.13) | 427.82 (133.04 to 1412.41) | 186.31 (57.82 to 615.21) | 62.90 (19.54 to 207.69) | 13.85 (4.30 to 45.72) | 2.18 (0.68 to 7.19) |

**Abbreviations:** GBD, Global Burden of Disease, DALYs, disability-adjusted life years; SDI, socio-demographic index; UI, uncertainty interval.
