# Supplementary material for: Global, regional, and national disability-adjusted life years and prevalence of lymphatic filariasis from 1990 to 2021: A trend and health inequality analysis based on the global burden of disease study 2021
Source: PLoS Negl Trop Dis. 2025 Apr 29;19(4):e0013017. doi: 10.1371/journal.pntd.0013017 (PMC12040265; doi:10.1371/journal.pntd.0013017)
Supplement: S11 Table — Abbreviations: GBD, Global Burden of Disease, SDI, sociodemographic index; DALYs, disability adjusted life years. (DOCX) [file pntd.0013017.s011.docx]

**S11 Table Changes in case numbers of lymphatic filariasis according to population-level determinants, including aging, population growth, and epidemiological change, by sex, SDI levels, GBD regions, among 67 countries and territories, from 1990 to 2021.**

| **Location** | **Sex** | **Overall difference** | **Aging** | **Population** | **Epidemiological change** | **Percent change of aging** | **Percent change of population** | **Percent change of epidemiological change** |
| --- | --- | --- | --- | --- | --- | --- | --- | --- |
| Global | Male | -2505669.45 | 143833.29 | 1006243.14 | -3655745.88 | -5.74 | -40.16 | 145.90 |
| Global | Female | -262375.58 | 25075.53 | 199555.76 | -487006.87 | -9.56 | -76.06 | 185.61 |
| Global | Both | -2768045.03 | 155850.15 | 1213128.69 | -4137023.87 | -5.63 | -43.83 | 149.46 |
| **SDI region** | | | | | | | | |
| Low SDI | Male | -684741.91 | 42002.31 | 670816.15 | -1397560.38 | -6.13 | -97.97 | 204.10 |
| Low SDI | Female | -58919.01 | 6602.30 | 121785.53 | -187306.84 | -11.21 | -206.70 | 317.91 |
| Low SDI | Both | -743660.92 | 49887.04 | 798476.21 | -1592024.16 | -6.71 | -107.37 | 214.08 |
| Low-middle SDI | Male | -1097183.75 | 137178.56 | 587297.66 | -1821659.97 | -12.50 | -53.53 | 166.03 |
| Low-middle SDI | Female | -160221.25 | 35582.58 | 132757.37 | -328561.20 | -22.21 | -82.86 | 205.07 |
| Low-middle SDI | Both | -1257404.99 | 177302.48 | 735008.37 | -2169715.85 | -14.10 | -58.45 | 172.56 |
| Middle SDI | Male | -687989.12 | 83867.65 | 238818.68 | -1010675.45 | -12.19 | -34.71 | 146.90 |
| Middle SDI | Female | -44204.60 | 10838.96 | 43518.50 | -98562.06 | -24.52 | -98.45 | 222.97 |
| Middle SDI | Both | -732193.71 | 94856.30 | 289511.23 | -1116561.24 | -12.96 | -39.54 | 152.50 |
| High-middle SDI | Male | -32900.61 | 619.26 | 8016.75 | -41536.61 | -1.88 | -24.37 | 126.25 |
| High-middle SDI | Female | 1531.74 | -707.28 | 1338.20 | 900.82 | -46.17 | 87.36 | 58.81 |
| High-middle SDI | Both | -31368.87 | -618.21 | 9143.00 | -39893.65 | 1.97 | -29.15 | 127.18 |
| High SDI | Male | 0.00 | 0.00 | 0.00 | 0.00 | - | - | - |
| High SDI | Female | 0.00 | 0.00 | 0.00 | 0.00 | - | - | - |
| High SDI | Both | 0.00 | 0.00 | 0.00 | 0.00 | - | - | - |
| **GBD region** | | | | | | | | |
| Caribbean | Both | -26847.47 | 1351.66 | 7810.49 | -36009.61 | -5.03 | -29.09 | 134.13 |
| Caribbean | Male | -23927.24 | 1134.01 | 6545.81 | -31607.06 | -4.74 | -27.36 | 132.10 |
| Caribbean | Female | -2920.23 | 325.82 | 1305.57 | -4551.62 | -11.16 | -44.71 | 155.87 |
| Central Sub-Saharan Africa | Both | -77943.71 | 5495.73 | 110349.58 | -193789.01 | -7.05 | -141.58 | 248.63 |
| Central Sub-Saharan Africa | Male | -78621.52 | 5491.07 | 98221.01 | -182333.61 | -6.98 | -124.93 | 231.91 |
| Central Sub-Saharan Africa | Female | 677.81 | 399.86 | 13207.40 | -12929.45 | 58.99 | 1948.54 | -1907.53 |
| Eastern Sub-Saharan Africa | Both | -278732.39 | 20492.01 | 265947.84 | -565172.25 | -7.35 | -95.41 | 202.77 |
| Eastern Sub-Saharan Africa | Male | -247928.23 | 17207.05 | 215717.01 | -480852.29 | -6.94 | -87.01 | 193.95 |
| Eastern Sub-Saharan Africa | Female | -30804.17 | 3767.70 | 50257.88 | -84829.75 | -12.23 | -163.15 | 275.38 |
| High-income Asia Pacific | Both | 45.44 | -25.56 | 7.96 | 63.04 | -56.25 | 17.52 | 138.74 |
| High-income Asia Pacific | Male | 28.80 | -15.68 | 5.33 | 39.15 | -54.44 | 18.52 | 135.94 |
| High-income Asia Pacific | Female | 16.64 | -8.45 | 2.36 | 22.73 | -50.77 | 14.16 | 136.62 |
| North Africa and Middle East | Both | -25390.21 | 3828.22 | 24794.16 | -54012.60 | -15.08 | -97.65 | 212.73 |
| North Africa and Middle East | Male | -23086.07 | 3808.36 | 19909.41 | -46803.84 | -16.50 | -86.24 | 202.74 |
| North Africa and Middle East | Female | -2304.14 | 82.36 | 5276.72 | -7663.21 | -3.57 | -229.01 | 332.58 |
| Oceania | Both | -13129.67 | 2013.69 | 24197.76 | -39341.12 | -15.34 | -184.30 | 299.64 |
| Oceania | Male | -7550.95 | 1331.39 | 19257.83 | -28140.17 | -17.63 | -255.04 | 372.67 |
| Oceania | Female | -5578.72 | 653.87 | 4872.50 | -11105.08 | -11.72 | -87.34 | 199.06 |
| South Asia | Both | -1270338.77 | 234225.33 | 874128.29 | -2378692.40 | -18.44 | -68.81 | 187.25 |
| South Asia | Male | -1107309.93 | 176545.45 | 688412.97 | -1972268.35 | -15.94 | -62.17 | 178.11 |
| South Asia | Female | -163028.84 | 47666.10 | 159021.99 | -369716.93 | -29.24 | -97.54 | 226.78 |
| Southeast Asia | Both | -744931.33 | 94449.35 | 281581.48 | -1120962.16 | -12.68 | -37.80 | 150.48 |
| Southeast Asia | Male | -708538.15 | 93313.94 | 254658.99 | -1056511.07 | -13.17 | -35.94 | 149.11 |
| Southeast Asia | Female | -36393.18 | 7077.12 | 32747.34 | -76217.64 | -19.45 | -89.98 | 209.43 |
| Southern Sub-Saharan Africa | Both | 887.13 | -11.97 | 1616.08 | -716.98 | -1.35 | 182.17 | -80.82 |
| Southern Sub-Saharan Africa | Male | 417.35 | 51.51 | 1114.86 | -749.02 | 12.34 | 267.13 | -179.47 |
| Southern Sub-Saharan Africa | Female | 469.78 | -50.95 | 510.90 | 9.83 | -10.85 | 108.75 | 2.09 |
| Tropical Latin America | Both | -5227.51 | 225.63 | 1954.83 | -7407.98 | -4.32 | -37.40 | 141.71 |
| Tropical Latin America | Male | -3638.45 | 261.22 | 1145.71 | -5045.38 | -7.18 | -31.49 | 138.67 |
| Tropical Latin America | Female | -1589.06 | -37.14 | 793.34 | -2345.26 | 2.34 | -49.93 | 147.59 |
| Western Sub-Saharan Africa | Both | -326436.54 | 10232.73 | 441200.06 | -777869.33 | -3.13 | -135.16 | 238.29 |
| Western Sub-Saharan Africa | Male | -305515.06 | 2720.91 | 364218.46 | -672454.44 | -0.89 | -119.21 | 220.11 |
| Western Sub-Saharan Africa | Female | -20921.48 | 2340.14 | 67019.16 | -90280.78 | -11.19 | -320.34 | 431.52 |
| **Abbreviations:** GBD, Global Burden of Disease, SDI, sociodemographic index; DALYs, disability adjusted life years. | | | | | | | | |
