# Supplementary material for: Global, regional, and national disability-adjusted life years and prevalence of lymphatic filariasis from 1990 to 2021: A trend and health inequality analysis based on the global burden of disease study 2021
Source: PLoS Negl Trop Dis. 2025 Apr 29;19(4):e0013017. doi: 10.1371/journal.pntd.0013017 (PMC12040265; doi:10.1371/journal.pntd.0013017)
Supplement: S12 Table — Abbreviations: ASR, age-standardized rate; CrI, credible interval. 1Niue and Palau are excluded from Institute for Health Metrics and Evaluation population forecast data, thus inapplicable to BAPC. (DOCX) [file pntd.0013017.s012.docx]

**S12 Table The predicted age-standardized rates (per 100,000) and number of cases of lymphatic filariasis, by sex, at the global level, among 65 countries and territories^1^, from 2022 to 2030.**

| **Location** | **Year** | **Sex** | **Number of cases (95% CrI)** | **ASR (95% CrI)** |
| --- | --- | --- | --- | --- |
| Global | 2022 | Male | 29187961.40 (26648476.40 to 31727446.39) | 723.11 (660.20 to 786.03) |
| Global | 2023 | Male | 28089220.64 (23116953.32 to 33061487.96) | 689.33 (567.30 to 811.35) |
| Global | 2024 | Male | 27035290.30 (19250791.63 to 34819788.96) | 657.39 (468.10 to 846.67) |
| Global | 2025 | Male | 26023926.41 (15202273.89 to 36845578.93) | 627.18 (366.38 to 887.99) |
| Global | 2026 | Male | 25042470.90 (11054541.74 to 39030400.06) | 598.35 (264.13 to 932.58) |
| Global | 2027 | Male | 24085477.01 (6870720.07 to 41300233.95) | 570.72 (162.81 to 978.63) |
| Global | 2028 | Male | 23160965.29 (2704425.59 to 43617504.98) | 544.42 (63.57 to 1025.26) |
| Global | 2029 | Male | 22275197.65 (0.00 to 45958126.44) | 519.54 (0.00 to 1071.91) |
| Global | 2030 | Male | 21426581.76 (0.00 to 48293928.36) | 496.01 (0.00 to 1117.98) |
| Global | 2022 | Female | 24238508.69 (22558982.96 to 25918034.41) | 604.61 (562.72 to 646.51) |
| Global | 2023 | Female | 23421733.60 (19952592.01 to 26890875.20) | 578.61 (492.91 to 664.31) |
| Global | 2024 | Female | 22640008.41 (17101904.83 to 28178111.98) | 554.06 (418.53 to 689.59) |
| Global | 2025 | Female | 21892836.91 (14102843.67 to 29682830.16) | 530.90 (341.99 to 719.80) |
| Global | 2026 | Female | 21167834.03 (11007903.86 to 31327764.21) | 508.78 (264.58 to 752.99) |
| Global | 2027 | Female | 20454565.39 (7856416.46 to 33052714.32) | 487.43 (187.22 to 787.65) |
| Global | 2028 | Female | 19762591.82 (4690433.99 to 34834749.64) | 467.03 (110.85 to 823.22) |
| Global | 2029 | Female | 19100565.96 (1538313.51 to 36662818.42) | 447.76 (36.06 to 859.45) |
| Global | 2030 | Female | 18468709.87 (0.00 to 38520035.26) | 429.57 (0.00 to 895.96) |
| Global | 2022 | Both | 53269174.62 (42151164.81 to 64387184.42) | 662.11 (523.92 to 800.30) |
| Global | 2023 | Both | 51128742.92 (27488805.43 to 74768680.42) | 629.45 (338.42 to 920.48) |
| Global | 2024 | Both | 49205073.44 (11253149.25 to 87156997.63) | 600.15 (137.25 to 1063.05) |
| Global | 2025 | Both | 47485980.22 (0.00 to 101005986.60) | 573.98 (0.00 to 1220.90) |
| Global | 2026 | Both | 45761797.12 (0.00 to 115506613.64) | 548.33 (0.00 to 1384.03) |
| Global | 2027 | Both | 43921867.05 (0.00 to 129984342.13) | 521.85 (0.00 to 1544.39) |
| Global | 2028 | Both | 42170329.02 (0.00 to 144632906.07) | 496.95 (0.00 to 1704.41) |
| Global | 2029 | Both | 40592054.24 (0.00 to 159622830.15) | 474.58 (0.00 to 1866.21) |
| Global | 2030 | Both | 39177366.03 (0.00 to 174937405.06) | 454.54 (0.00 to 2029.66) |
| American Samoa | 2022 | Male | 1285.64 (969.41 to 1601.86) | 4263.97 (3241.41 to 5286.54) |
| American Samoa | 2023 | Male | 1223.90 (605.36 to 1842.44) | 4001.24 (1991.54 to 6010.95) |
| American Samoa | 2024 | Male | 1165.28 (205.82 to 2124.75) | 3755.02 (670.75 to 6839.29) |
| American Samoa | 2025 | Male | 1109.64 (0.00 to 2429.66) | 3524.36 (0.00 to 7711.81) |
| American Samoa | 2026 | Male | 1056.84 (0.00 to 2745.82) | 3308.40 (0.00 to 8591.98) |
| American Samoa | 2027 | Male | 1006.68 (0.00 to 3065.29) | 3106.14 (0.00 to 9455.20) |
| American Samoa | 2028 | Male | 958.89 (0.00 to 3382.00) | 2916.63 (0.00 to 10284.60) |
| American Samoa | 2029 | Male | 913.33 (0.00 to 3691.49) | 2739.01 (0.00 to 11068.66) |
| American Samoa | 2030 | Male | 869.85 (0.00 to 3990.41) | 2572.59 (0.00 to 11800.05) |
| American Samoa | 2022 | Female | 1045.90 (796.54 to 1295.26) | 3531.84 (2717.45 to 4346.22) |
| American Samoa | 2023 | Female | 1001.71 (524.09 to 1479.33) | 3334.09 (1757.83 to 4910.34) |
| American Samoa | 2024 | Female | 959.45 (221.04 to 1697.86) | 3147.63 (733.36 to 5561.90) |
| American Samoa | 2025 | Female | 919.05 (0.00 to 1936.22) | 2971.89 (0.00 to 6255.48) |
| American Samoa | 2026 | Female | 880.42 (0.00 to 2185.84) | 2806.30 (0.00 to 6963.15) |
| American Samoa | 2027 | Female | 843.45 (0.00 to 2440.69) | 2650.24 (0.00 to 7665.86) |
| American Samoa | 2028 | Female | 807.97 (0.00 to 2696.13) | 2503.12 (0.00 to 8350.21) |
| American Samoa | 2029 | Female | 773.88 (0.00 to 2948.63) | 2364.42 (0.00 to 9006.77) |
| American Samoa | 2030 | Female | 741.12 (0.00 to 3195.44) | 2233.68 (0.00 to 9629.05) |
| American Samoa | 2022 | Both | 2334.23 (1808.45 to 2860.00) | 3905.69 (3040.34 to 4771.05) |
| American Samoa | 2023 | Both | 2229.83 (1169.49 to 3290.17) | 3677.62 (1935.50 to 5419.75) |
| American Samoa | 2024 | Both | 2130.39 (465.20 to 3795.59) | 3463.25 (760.24 to 6166.26) |
| American Samoa | 2025 | Both | 2035.66 (0.00 to 4344.62) | 3261.79 (0.00 to 6958.77) |
| American Samoa | 2026 | Both | 1945.45 (0.00 to 4917.69) | 3072.57 (0.00 to 7764.80) |
| American Samoa | 2027 | Both | 1859.43 (0.00 to 5500.82) | 2894.75 (0.00 to 8562.14) |
| American Samoa | 2028 | Both | 1777.16 (0.00 to 6083.18) | 2727.59 (0.00 to 9335.26) |
| American Samoa | 2029 | Both | 1698.41 (0.00 to 6656.64) | 2570.41 (0.00 to 10073.31) |
| American Samoa | 2030 | Both | 1622.95 (0.00 to 7214.93) | 2422.62 (0.00 to 10769.06) |
| Angola | 2022 | Male | 189311.25 (174796.33 to 203826.16) | 1181.17 (1090.76 to 1271.57) |
| Angola | 2023 | Male | 186580.24 (155711.67 to 217448.81) | 1129.40 (942.61 to 1316.18) |
| Angola | 2024 | Male | 183867.38 (133512.62 to 234222.14) | 1080.37 (784.54 to 1376.20) |
| Angola | 2025 | Male | 181190.06 (108934.03 to 253446.10) | 1033.93 (621.64 to 1446.22) |
| Angola | 2026 | Male | 178538.54 (82439.39 to 274637.69) | 989.89 (457.10 to 1522.68) |
| Angola | 2027 | Male | 175892.13 (54368.05 to 297416.21) | 948.09 (293.07 to 1603.12) |
| Angola | 2028 | Male | 173227.46 (25001.65 to 321453.28) | 908.40 (131.12 to 1685.69) |
| Angola | 2029 | Male | 170562.95 (0.00 to 346537.96) | 870.70 (0.00 to 1769.02) |
| Angola | 2030 | Male | 167923.48 (0.00 to 372520.60) | 834.87 (0.00 to 1852.07) |
| Angola | 2022 | Female | 168093.80 (156554.43 to 179633.16) | 998.56 (930.18 to 1066.95) |
| Angola | 2023 | Female | 164960.19 (140238.75 to 189681.63) | 952.40 (809.74 to 1095.05) |
| Angola | 2024 | Female | 161883.33 (121581.94 to 202184.71) | 908.79 (682.59 to 1135.00) |
| Angola | 2025 | Female | 158880.32 (101185.18 to 216575.46) | 867.61 (552.58 to 1182.64) |
| Angola | 2026 | Female | 155945.75 (79432.67 to 232458.83) | 828.69 (422.12 to 1235.25) |
| Angola | 2027 | Female | 153059.16 (56599.30 to 249519.01) | 791.88 (292.84 to 1290.92) |
| Angola | 2028 | Female | 150201.82 (32910.37 to 267493.28) | 757.06 (165.89 to 1348.24) |
| Angola | 2029 | Female | 147389.33 (8561.42 to 286217.23) | 724.12 (42.07 to 1406.18) |
| Angola | 2030 | Female | 144645.02 (0.00 to 305584.11) | 692.97 (0.00 to 1463.99) |
| Angola | 2022 | Both | 356995.94 (331253.79 to 382738.09) | 1086.38 (1008.12 to 1164.64) |
| Angola | 2023 | Both | 351226.17 (296037.30 to 406415.03) | 1037.88 (874.83 to 1200.92) |
| Angola | 2024 | Both | 345528.21 (255379.25 to 435677.18) | 991.99 (733.20 to 1250.78) |
| Angola | 2025 | Both | 339935.58 (210596.71 to 469274.46) | 948.56 (587.67 to 1309.46) |
| Angola | 2026 | Both | 334434.40 (162532.28 to 506336.53) | 907.44 (441.02 to 1373.87) |
| Angola | 2027 | Both | 328986.22 (111797.83 to 546174.60) | 868.48 (295.14 to 1441.82) |
| Angola | 2028 | Both | 323546.01 (58895.45 to 588196.56) | 831.53 (151.37 to 1511.70) |
| Angola | 2029 | Both | 318146.41 (4266.22 to 632026.61) | 796.50 (10.69 to 1582.31) |
| Angola | 2030 | Both | 312835.16 (0.00 to 677408.33) | 763.26 (0.00 to 1652.74) |
| Bangladesh | 2022 | Male | 294307.41 (266379.44 to 322235.38) | 364.58 (330.01 to 399.15) |
| Bangladesh | 2023 | Male | 292386.72 (235228.75 to 349544.69) | 359.89 (289.55 to 430.24) |
| Bangladesh | 2024 | Male | 290717.68 (197972.18 to 383463.18) | 355.71 (242.24 to 469.18) |
| Bangladesh | 2025 | Male | 289327.93 (155860.54 to 422795.33) | 352.03 (189.64 to 514.42) |
| Bangladesh | 2026 | Male | 288155.21 (109487.62 to 466822.79) | 348.76 (132.52 to 565.00) |
| Bangladesh | 2027 | Male | 287202.80 (59226.89 to 515178.71) | 345.93 (71.34 to 620.52) |
| Bangladesh | 2028 | Male | 286473.02 (5357.91 to 567588.13) | 343.54 (6.43 to 680.65) |
| Bangladesh | 2029 | Male | 286019.38 (0.00 to 623978.61) | 341.64 (0.00 to 745.32) |
| Bangladesh | 2030 | Male | 285872.71 (0.00 to 684342.58) | 340.25 (0.00 to 814.52) |
| Bangladesh | 2022 | Female | 227421.60 (209533.63 to 245309.58) | 274.99 (253.39 to 296.59) |
| Bangladesh | 2023 | Female | 225454.38 (187385.63 to 263523.14) | 270.73 (225.03 to 316.43) |
| Bangladesh | 2024 | Female | 223738.08 (161288.32 to 286187.84) | 266.93 (192.43 to 341.42) |
| Bangladesh | 2025 | Female | 222284.74 (131996.66 to 312572.82) | 263.57 (156.52 to 370.62) |
| Bangladesh | 2026 | Female | 221090.27 (99923.91 to 342256.64) | 260.63 (117.80 to 403.47) |
| Bangladesh | 2027 | Female | 220141.84 (65315.61 to 374968.07) | 258.12 (76.59 to 439.66) |
| Bangladesh | 2028 | Female | 219406.38 (28326.83 to 410485.92) | 255.99 (33.05 to 478.93) |
| Bangladesh | 2029 | Female | 218874.31 (0.00 to 448684.56) | 254.22 (0.00 to 521.15) |
| Bangladesh | 2030 | Female | 218559.33 (0.00 to 489533.99) | 252.81 (0.00 to 566.25) |
| Bangladesh | 2022 | Both | 522384.53 (477285.62 to 567483.44) | 319.65 (292.06 to 347.23) |
| Bangladesh | 2023 | Both | 518523.97 (424116.80 to 612931.13) | 315.18 (257.80 to 372.56) |
| Bangladesh | 2024 | Both | 515114.05 (360911.87 to 669316.23) | 311.15 (218.01 to 404.30) |
| Bangladesh | 2025 | Both | 512198.38 (289646.17 to 734750.59) | 307.58 (173.94 to 441.22) |
| Bangladesh | 2026 | Both | 509759.21 (211367.01 to 808151.40) | 304.42 (126.23 to 482.62) |
| Bangladesh | 2027 | Both | 507766.80 (126713.90 to 888819.69) | 301.69 (75.29 to 528.09) |
| Bangladesh | 2028 | Both | 506170.85 (36126.42 to 976215.27) | 299.34 (21.37 to 577.31) |
| Bangladesh | 2029 | Both | 504991.15 (0.00 to 1070070.07) | 297.38 (0.00 to 630.14) |
| Bangladesh | 2030 | Both | 504275.66 (0.00 to 1170315.83) | 295.81 (0.00 to 686.52) |
| Benin | 2022 | Male | 54876.52 (47194.52 to 62558.52) | 831.54 (715.34 to 947.74) |
| Benin | 2023 | Male | 54413.27 (37656.99 to 71169.55) | 801.08 (554.48 to 1047.68) |
| Benin | 2024 | Male | 53941.91 (26283.15 to 81600.68) | 771.90 (376.16 to 1167.64) |
| Benin | 2025 | Male | 53458.26 (13429.96 to 93486.56) | 743.94 (186.93 to 1300.96) |
| Benin | 2026 | Male | 52960.92 (0.00 to 106577.39) | 717.14 (0.00 to 1443.13) |
| Benin | 2027 | Male | 52449.85 (0.00 to 120684.79) | 691.44 (0.00 to 1590.95) |
| Benin | 2028 | Male | 51926.65 (0.00 to 135659.58) | 666.78 (0.00 to 1741.98) |
| Benin | 2029 | Male | 51390.48 (0.00 to 151370.80) | 643.12 (0.00 to 1894.29) |
| Benin | 2030 | Male | 50837.44 (0.00 to 167692.47) | 620.38 (0.00 to 2046.38) |
| Benin | 2022 | Female | 46588.77 (39879.47 to 53298.07) | 685.85 (587.27 to 784.42) |
| Benin | 2023 | Female | 45954.46 (31343.72 to 60565.20) | 658.19 (449.01 to 867.37) |
| Benin | 2024 | Female | 45328.67 (21305.81 to 69351.53) | 631.88 (297.05 to 966.71) |
| Benin | 2025 | Female | 44708.22 (10089.10 to 79327.34) | 606.86 (136.98 to 1076.73) |
| Benin | 2026 | Female | 44092.52 (0.00 to 90269.15) | 583.05 (0.00 to 1193.64) |
| Benin | 2027 | Female | 43481.06 (0.00 to 102009.85) | 560.40 (0.00 to 1314.72) |
| Benin | 2028 | Female | 42874.85 (0.00 to 114420.25) | 538.83 (0.00 to 1437.96) |
| Benin | 2029 | Female | 42272.99 (0.00 to 127390.34) | 518.28 (0.00 to 1561.83) |
| Benin | 2030 | Female | 41672.59 (0.00 to 140818.99) | 498.70 (0.00 to 1685.17) |
| Benin | 2022 | Both | 101641.37 (87244.72 to 116038.02) | 758.96 (651.56 to 866.35) |
| Benin | 2023 | Both | 100590.82 (69111.76 to 132069.89) | 730.27 (501.78 to 958.76) |
| Benin | 2024 | Both | 99539.24 (47603.00 to 151475.48) | 702.87 (336.16 to 1069.58) |
| Benin | 2025 | Both | 98477.89 (23394.27 to 173561.51) | 676.69 (160.77 to 1192.60) |
| Benin | 2026 | Both | 97404.12 (0.00 to 197852.95) | 651.65 (0.00 to 1323.65) |
| Benin | 2027 | Both | 96318.69 (0.00 to 223995.50) | 627.70 (0.00 to 1459.76) |
| Benin | 2028 | Both | 95225.06 (0.00 to 251711.99) | 604.81 (0.00 to 1598.70) |
| Benin | 2029 | Both | 94121.56 (0.00 to 280761.09) | 582.90 (0.00 to 1738.75) |
| Benin | 2030 | Both | 92999.91 (0.00 to 310910.15) | 561.90 (0.00 to 1878.51) |
| Brazil | 2022 | Male | 7383.99 (6337.96 to 8430.02) | 6.88 (5.92 to 7.84) |
| Brazil | 2023 | Male | 7435.91 (5323.88 to 9547.94) | 6.88 (4.93 to 8.83) |
| Brazil | 2024 | Male | 7496.73 (4054.23 to 10939.24) | 6.89 (3.73 to 10.06) |
| Brazil | 2025 | Male | 7567.44 (2565.35 to 12569.52) | 6.92 (2.35 to 11.49) |
| Brazil | 2026 | Male | 7648.36 (871.24 to 14425.49) | 6.96 (0.79 to 13.12) |
| Brazil | 2027 | Male | 7737.98 (0.00 to 16499.52) | 7.01 (0.00 to 14.94) |
| Brazil | 2028 | Male | 7834.67 (0.00 to 18786.33) | 7.06 (0.00 to 16.93) |
| Brazil | 2029 | Male | 7938.64 (0.00 to 21287.05) | 7.12 (0.00 to 19.10) |
| Brazil | 2030 | Male | 8050.63 (0.00 to 24006.90) | 7.20 (0.00 to 21.46) |
| Brazil | 2022 | Female | 10765.23 (9205.17 to 12325.30) | 9.56 (8.19 to 10.93) |
| Brazil | 2023 | Female | 10840.09 (7653.61 to 14026.58) | 9.56 (6.76 to 12.37) |
| Brazil | 2024 | Female | 10919.53 (5708.64 to 16130.43) | 9.57 (5.01 to 14.13) |
| Brazil | 2025 | Female | 11003.24 (3428.91 to 18577.56) | 9.58 (2.99 to 16.18) |
| Brazil | 2026 | Female | 11091.20 (843.37 to 21339.02) | 9.60 (0.73 to 18.48) |
| Brazil | 2027 | Female | 11182.97 (0.00 to 24397.06) | 9.63 (0.00 to 21.01) |
| Brazil | 2028 | Female | 11277.88 (0.00 to 27738.93) | 9.66 (0.00 to 23.77) |
| Brazil | 2029 | Female | 11375.85 (0.00 to 31356.85) | 9.70 (0.00 to 26.74) |
| Brazil | 2030 | Female | 11476.47 (0.00 to 35244.74) | 9.74 (0.00 to 29.93) |
| Brazil | 2022 | Both | 18248.33 (15790.91 to 20705.75) | 8.30 (7.18 to 9.41) |
| Brazil | 2023 | Both | 18393.09 (13358.21 to 23427.96) | 8.31 (6.04 to 10.58) |
| Brazil | 2024 | Both | 18551.49 (10301.16 to 26801.82) | 8.32 (4.62 to 12.02) |
| Brazil | 2025 | Both | 18723.87 (6707.62 to 30740.11) | 8.35 (2.99 to 13.71) |
| Brazil | 2026 | Both | 18910.79 (2617.33 to 35204.25) | 8.39 (1.16 to 15.62) |
| Brazil | 2027 | Both | 19110.87 (0.00 to 40172.23) | 8.44 (0.00 to 17.73) |
| Brazil | 2028 | Both | 19321.55 (0.00 to 45627.54) | 8.49 (0.00 to 20.04) |
| Brazil | 2029 | Both | 19542.75 (0.00 to 51564.57) | 8.55 (0.00 to 22.55) |
| Brazil | 2030 | Both | 19774.58 (0.00 to 57983.01) | 8.61 (0.00 to 25.25) |
| Brunei Darussalam | 2022 | Male | 2338.34 (2016.80 to 2659.87) | 976.39 (848.10 to 1104.69) |
| Brunei Darussalam | 2023 | Male | 2259.45 (1652.71 to 2866.19) | 933.54 (685.82 to 1181.25) |
| Brunei Darussalam | 2024 | Male | 2182.40 (1243.63 to 3121.18) | 892.62 (510.49 to 1274.76) |
| Brunei Darussalam | 2025 | Male | 2107.17 (808.37 to 3405.96) | 853.58 (328.72 to 1378.44) |
| Brunei Darussalam | 2026 | Male | 2033.72 (357.92 to 3709.52) | 816.34 (144.60 to 1488.07) |
| Brunei Darussalam | 2027 | Male | 1962.01 (0.00 to 4024.02) | 780.78 (0.00 to 1600.64) |
| Brunei Darussalam | 2028 | Male | 1892.08 (0.00 to 4343.62) | 746.83 (0.00 to 1713.89) |
| Brunei Darussalam | 2029 | Male | 1823.84 (0.00 to 4663.41) | 714.39 (0.00 to 1826.16) |
| Brunei Darussalam | 2030 | Male | 1757.25 (0.00 to 4979.50) | 683.41 (0.00 to 1936.17) |
| Brunei Darussalam | 2022 | Female | 1803.46 (1526.73 to 2080.19) | 819.87 (699.89 to 939.85) |
| Brunei Darussalam | 2023 | Female | 1740.50 (1221.78 to 2259.22) | 782.20 (552.00 to 1012.41) |
| Brunei Darussalam | 2024 | Female | 1679.15 (879.15 to 2479.15) | 746.35 (392.56 to 1100.14) |
| Brunei Darussalam | 2025 | Female | 1619.38 (514.99 to 2723.77) | 712.23 (227.74 to 1196.72) |
| Brunei Darussalam | 2026 | Female | 1561.19 (138.70 to 2983.67) | 679.78 (61.31 to 1298.24) |
| Brunei Darussalam | 2027 | Female | 1504.52 (0.00 to 3252.22) | 648.89 (0.00 to 1401.94) |
| Brunei Darussalam | 2028 | Female | 1449.41 (0.00 to 3524.51) | 619.47 (0.00 to 1505.78) |
| Brunei Darussalam | 2029 | Female | 1395.78 (0.00 to 3796.45) | 591.45 (0.00 to 1608.24) |
| Brunei Darussalam | 2030 | Female | 1343.59 (0.00 to 4064.84) | 564.77 (0.00 to 1708.23) |
| Brunei Darussalam | 2022 | Both | 4139.35 (3609.33 to 4669.36) | 900.92 (788.88 to 1012.97) |
| Brunei Darussalam | 2023 | Both | 3996.40 (2952.81 to 5039.99) | 860.28 (637.23 to 1083.34) |
| Brunei Darussalam | 2024 | Both | 3856.94 (2219.41 to 5494.48) | 821.55 (473.71 to 1169.38) |
| Brunei Darussalam | 2025 | Both | 3720.93 (1440.55 to 6001.30) | 784.63 (304.43 to 1264.83) |
| Brunei Darussalam | 2026 | Both | 3588.30 (635.47 to 6541.13) | 749.45 (133.21 to 1365.70) |
| Brunei Darussalam | 2027 | Both | 3458.95 (0.00 to 7099.95) | 715.92 (0.00 to 1469.14) |
| Brunei Darussalam | 2028 | Both | 3332.95 (0.00 to 7667.26) | 683.93 (0.00 to 1573.03) |
| Brunei Darussalam | 2029 | Both | 3210.10 (0.00 to 8234.24) | 653.40 (0.00 to 1675.79) |
| Brunei Darussalam | 2030 | Both | 3090.37 (0.00 to 8793.92) | 624.28 (0.00 to 1776.24) |
| Burkina Faso | 2022 | Male | 83089.58 (51749.54 to 114429.63) | 694.22 (432.42 to 956.03) |
| Burkina Faso | 2023 | Male | 82900.94 (13509.95 to 152291.92) | 671.70 (109.48 to 1233.92) |
| Burkina Faso | 2024 | Male | 82790.83 (0.00 to 198476.99) | 650.61 (0.00 to 1559.71) |
| Burkina Faso | 2025 | Male | 82746.57 (0.00 to 251801.68) | 630.81 (0.00 to 1919.56) |
| Burkina Faso | 2026 | Male | 82756.44 (0.00 to 311519.74) | 612.15 (0.00 to 2304.29) |
| Burkina Faso | 2027 | Male | 82819.89 (0.00 to 377161.46) | 594.55 (0.00 to 2707.58) |
| Burkina Faso | 2028 | Male | 82943.93 (0.00 to 448457.26) | 578.02 (0.00 to 3125.20) |
| Burkina Faso | 2029 | Male | 83128.38 (0.00 to 525228.49) | 562.50 (0.00 to 3554.05) |
| Burkina Faso | 2030 | Male | 83361.81 (0.00 to 607296.58) | 547.91 (0.00 to 3991.56) |
| Burkina Faso | 2022 | Female | 71157.19 (46314.52 to 95999.87) | 565.04 (367.82 to 762.27) |
| Burkina Faso | 2023 | Female | 70636.31 (15720.99 to 125551.64) | 544.73 (121.25 to 968.20) |
| Burkina Faso | 2024 | Female | 70221.45 (0.00 to 161450.57) | 525.96 (0.00 to 1209.25) |
| Burkina Faso | 2025 | Female | 69898.23 (0.00 to 202736.99) | 508.57 (0.00 to 1475.09) |
| Burkina Faso | 2026 | Female | 69648.33 (0.00 to 248793.26) | 492.36 (0.00 to 1758.79) |
| Burkina Faso | 2027 | Female | 69476.50 (0.00 to 299276.36) | 477.29 (0.00 to 2055.97) |
| Burkina Faso | 2028 | Female | 69395.00 (0.00 to 354039.09) | 463.37 (0.00 to 2364.02) |
| Burkina Faso | 2029 | Female | 69406.82 (0.00 to 413022.71) | 450.57 (0.00 to 2681.21) |
| Burkina Faso | 2030 | Female | 69498.98 (0.00 to 476150.00) | 438.76 (0.00 to 3006.01) |
| Burkina Faso | 2022 | Both | 153836.66 (97696.11 to 209977.22) | 626.32 (397.78 to 854.87) |
| Burkina Faso | 2023 | Both | 153173.73 (28798.33 to 277549.13) | 605.21 (113.80 to 1096.62) |
| Burkina Faso | 2024 | Both | 152697.20 (0.00 to 359843.71) | 585.58 (0.00 to 1379.96) |
| Burkina Faso | 2025 | Both | 152379.73 (0.00 to 454736.11) | 567.28 (0.00 to 1692.88) |
| Burkina Faso | 2026 | Both | 152189.21 (0.00 to 560867.37) | 550.12 (0.00 to 2027.37) |
| Burkina Faso | 2027 | Both | 152129.79 (0.00 to 677433.45) | 534.05 (0.00 to 2378.11) |
| Burkina Faso | 2028 | Both | 152221.90 (0.00 to 804033.49) | 519.07 (0.00 to 2741.72) |
| Burkina Faso | 2029 | Both | 152469.57 (0.00 to 940450.73) | 505.16 (0.00 to 3115.87) |
| Burkina Faso | 2030 | Both | 152848.25 (0.00 to 1086443.96) | 492.19 (0.00 to 3498.52) |
| Cambodia | 2022 | Male | 9598.24 (0.00 to 19407.48) | 113.33 (0.00 to 229.13) |
| Cambodia | 2023 | Male | 9227.52 (0.00 to 30230.99) | 107.64 (0.00 to 352.62) |
| Cambodia | 2024 | Male | 8876.19 (0.00 to 42612.16) | 102.35 (0.00 to 491.36) |
| Cambodia | 2025 | Male | 8543.11 (0.00 to 56007.36) | 97.42 (0.00 to 638.65) |
| Cambodia | 2026 | Male | 8225.02 (0.00 to 70040.46) | 92.78 (0.00 to 790.09) |
| Cambodia | 2027 | Male | 7917.44 (0.00 to 84411.51) | 88.41 (0.00 to 942.54) |
| Cambodia | 2028 | Male | 7614.06 (0.00 to 98829.55) | 84.26 (0.00 to 1093.70) |
| Cambodia | 2029 | Male | 7319.24 (0.00 to 113154.52) | 80.32 (0.00 to 1241.80) |
| Cambodia | 2030 | Male | 7035.86 (0.00 to 127296.17) | 76.57 (0.00 to 1385.42) |
| Cambodia | 2022 | Female | 9997.51 (0.00 to 20215.93) | 113.87 (0.00 to 230.23) |
| Cambodia | 2023 | Female | 9609.13 (0.00 to 31493.65) | 108.19 (0.00 to 354.57) |
| Cambodia | 2024 | Female | 9238.78 (0.00 to 44377.57) | 102.89 (0.00 to 494.21) |
| Cambodia | 2025 | Female | 8885.21 (0.00 to 58288.05) | 97.91 (0.00 to 642.33) |
| Cambodia | 2026 | Female | 8545.74 (0.00 to 72823.46) | 93.22 (0.00 to 794.36) |
| Cambodia | 2027 | Female | 8216.24 (0.00 to 87663.33) | 88.76 (0.00 to 947.08) |
| Cambodia | 2028 | Female | 7890.77 (0.00 to 102501.70) | 84.53 (0.00 to 1098.04) |
| Cambodia | 2029 | Female | 7573.32 (0.00 to 117177.59) | 80.49 (0.00 to 1245.38) |
| Cambodia | 2030 | Female | 7266.84 (0.00 to 131584.23) | 76.63 (0.00 to 1387.57) |
| Cambodia | 2022 | Both | 19640.37 (0.00 to 39877.35) | 113.86 (0.00 to 231.17) |
| Cambodia | 2023 | Both | 18881.29 (0.00 to 62232.89) | 108.17 (0.00 to 356.53) |
| Cambodia | 2024 | Both | 18159.84 (0.00 to 87791.00) | 102.88 (0.00 to 497.35) |
| Cambodia | 2025 | Both | 17473.55 (0.00 to 115418.45) | 97.92 (0.00 to 646.82) |
| Cambodia | 2026 | Both | 16816.40 (0.00 to 144330.29) | 93.26 (0.00 to 800.39) |
| Cambodia | 2027 | Both | 16179.68 (0.00 to 173899.78) | 88.84 (0.00 to 954.87) |
| Cambodia | 2028 | Both | 15551.03 (0.00 to 203523.15) | 84.65 (0.00 to 1107.84) |
| Cambodia | 2029 | Both | 14938.80 (0.00 to 232895.25) | 80.66 (0.00 to 1257.45) |
| Cambodia | 2030 | Both | 14348.88 (0.00 to 261816.80) | 76.85 (0.00 to 1402.24) |
| Cameroon | 2022 | Male | 52789.81 (41268.72 to 64310.90) | 340.83 (266.51 to 415.16) |
| Cameroon | 2023 | Male | 53246.90 (27660.16 to 78833.64) | 336.68 (174.92 to 498.43) |
| Cameroon | 2024 | Male | 53820.73 (10746.15 to 96895.31) | 333.48 (66.60 to 600.37) |
| Cameroon | 2025 | Male | 54479.00 (0.00 to 118156.82) | 330.98 (0.00 to 717.83) |
| Cameroon | 2026 | Male | 55161.96 (0.00 to 142339.70) | 328.76 (0.00 to 848.31) |
| Cameroon | 2027 | Male | 55921.85 (0.00 to 169499.38) | 327.15 (0.00 to 991.58) |
| Cameroon | 2028 | Male | 56844.72 (0.00 to 199954.53) | 326.64 (0.00 to 1148.96) |
| Cameroon | 2029 | Male | 57945.63 (0.00 to 233960.00) | 327.24 (0.00 to 1321.26) |
| Cameroon | 2030 | Male | 59180.72 (0.00 to 271582.77) | 328.63 (0.00 to 1508.11) |
| Cameroon | 2022 | Female | 40135.87 (31192.47 to 49079.27) | 257.10 (199.86 to 314.33) |
| Cameroon | 2023 | Female | 40361.64 (20471.39 to 60251.88) | 253.17 (128.43 to 377.91) |
| Cameroon | 2024 | Female | 40679.33 (7246.03 to 74112.63) | 250.02 (44.55 to 455.48) |
| Cameroon | 2025 | Female | 41094.88 (0.00 to 90455.28) | 247.60 (0.00 to 545.00) |
| Cameroon | 2026 | Female | 41611.63 (0.00 to 109219.70) | 245.91 (0.00 to 645.43) |
| Cameroon | 2027 | Female | 42243.37 (0.00 to 130470.04) | 244.99 (0.00 to 756.65) |
| Cameroon | 2028 | Female | 43006.54 (0.00 to 154364.33) | 244.92 (0.00 to 879.11) |
| Cameroon | 2029 | Female | 43909.08 (0.00 to 181106.40) | 245.70 (0.00 to 1013.42) |
| Cameroon | 2030 | Female | 44954.35 (0.00 to 210935.95) | 247.28 (0.00 to 1160.28) |
| Cameroon | 2022 | Both | 92584.20 (72223.93 to 112944.47) | 297.70 (232.26 to 363.14) |
| Cameroon | 2023 | Both | 93315.06 (47895.87 to 138734.25) | 293.83 (150.83 to 436.84) |
| Cameroon | 2024 | Both | 94261.58 (17725.55 to 170797.61) | 290.85 (54.70 to 526.99) |
| Cameroon | 2025 | Both | 95399.09 (0.00 to 208615.71) | 288.59 (0.00 to 631.07) |
| Cameroon | 2026 | Both | 96688.76 (0.00 to 251913.65) | 286.90 (0.00 to 747.50) |
| Cameroon | 2027 | Both | 98205.60 (0.00 to 300880.99) | 286.01 (0.00 to 876.26) |
| Cameroon | 2028 | Both | 100047.13 (0.00 to 356042.36) | 286.16 (0.00 to 1018.36) |
| Cameroon | 2029 | Both | 102227.86 (0.00 to 417885.12) | 287.33 (0.00 to 1174.55) |
| Cameroon | 2030 | Both | 104707.78 (0.00 to 486769.29) | 289.35 (0.00 to 1345.12) |
| Central African Republic | 2022 | Male | 109563.46 (98532.08 to 120594.83) | 4617.51 (4153.40 to 5081.62) |
| Central African Republic | 2023 | Male | 104016.20 (81173.58 to 126858.82) | 4360.10 (3402.96 to 5317.24) |
| Central African Republic | 2024 | Male | 98737.31 (62724.54 to 134750.09) | 4117.65 (2616.03 to 5619.28) |
| Central African Republic | 2025 | Male | 93745.01 (43875.32 to 143614.69) | 3889.58 (1820.58 to 5958.57) |
| Central African Republic | 2026 | Male | 89024.79 (25040.57 to 153009.01) | 3675.07 (1033.82 to 6316.31) |
| Central African Republic | 2027 | Male | 84522.40 (6495.50 to 162549.31) | 3473.05 (266.99 to 6679.11) |
| Central African Republic | 2028 | Male | 80232.45 (0.00 to 172005.70) | 3282.78 (0.00 to 7037.69) |
| Central African Republic | 2029 | Male | 76149.59 (0.00 to 181211.68) | 3103.67 (0.00 to 7385.70) |
| Central African Republic | 2030 | Male | 72277.66 (0.00 to 190072.12) | 2935.27 (0.00 to 7718.98) |
| Central African Republic | 2022 | Female | 91646.67 (82438.86 to 100854.49) | 3778.49 (3399.65 to 4157.33) |
| Central African Republic | 2023 | Female | 87676.59 (68160.48 to 107192.69) | 3587.26 (2789.12 to 4385.40) |
| Central African Republic | 2024 | Female | 83870.82 (52717.74 to 115023.89) | 3406.40 (2141.33 to 4671.47) |
| Central African Republic | 2025 | Female | 80242.93 (36670.81 to 123815.06) | 3235.37 (1478.70 to 4992.04) |
| Central African Republic | 2026 | Female | 76781.46 (20375.02 to 133187.89) | 3073.48 (815.69 to 5331.26) |
| Central African Republic | 2027 | Female | 73450.13 (4079.98 to 142820.28) | 2920.21 (162.29 to 5678.13) |
| Central African Republic | 2028 | Female | 70250.46 (0.00 to 152514.01) | 2775.16 (0.00 to 6024.82) |
| Central African Republic | 2029 | Female | 67179.66 (0.00 to 162113.96) | 2637.90 (0.00 to 6365.57) |
| Central African Republic | 2030 | Female | 64239.55 (0.00 to 171514.55) | 2508.01 (0.00 to 6696.15) |
| Central African Republic | 2022 | Both | 200256.87 (180514.85 to 219998.90) | 4173.53 (3762.49 to 4584.56) |
| Central African Republic | 2023 | Both | 190733.51 (149054.52 to 232412.50) | 3949.14 (3086.36 to 4811.92) |
| Central African Republic | 2024 | Both | 181644.20 (115398.88 to 247889.52) | 3737.49 (2374.54 to 5100.44) |
| Central African Republic | 2025 | Both | 173021.29 (80769.49 to 265273.09) | 3538.02 (1651.69 to 5424.36) |
| Central African Republic | 2026 | Both | 164840.68 (45928.26 to 283753.09) | 3350.02 (933.44 to 5766.59) |
| Central African Republic | 2027 | Both | 157009.84 (11393.80 to 302625.88) | 3172.62 (230.27 to 6114.97) |
| Central African Republic | 2028 | Both | 149524.01 (0.00 to 321465.21) | 3005.24 (0.00 to 6461.00) |
| Central African Republic | 2029 | Both | 142375.32 (0.00 to 339952.65) | 2847.37 (0.00 to 6798.70) |
| Central African Republic | 2030 | Both | 135570.52 (0.00 to 357892.87) | 2698.59 (0.00 to 7123.99) |
| Chad | 2022 | Male | 75772.11 (68389.49 to 83154.73) | 829.35 (748.76 to 909.94) |
| Chad | 2023 | Male | 75901.41 (59898.20 to 91904.63) | 799.50 (631.03 to 967.97) |
| Chad | 2024 | Male | 76061.72 (49493.31 to 102630.13) | 771.04 (501.77 to 1040.31) |
| Chad | 2025 | Male | 76256.13 (37454.77 to 115057.50) | 743.91 (365.42 to 1122.39) |
| Chad | 2026 | Male | 76479.79 (23942.67 to 129016.90) | 717.98 (224.80 to 1211.16) |
| Chad | 2027 | Male | 76726.16 (9062.51 to 144389.81) | 693.19 (81.90 to 1304.48) |
| Chad | 2028 | Male | 76995.67 (0.00 to 161099.32) | 669.51 (0.00 to 1400.80) |
| Chad | 2029 | Male | 77286.14 (0.00 to 179085.23) | 646.88 (0.00 to 1498.93) |
| Chad | 2030 | Male | 77594.94 (0.00 to 198299.85) | 625.26 (0.00 to 1597.88) |
| Chad | 2022 | Female | 63327.53 (57478.87 to 69176.20) | 684.34 (621.36 to 747.32) |
| Chad | 2023 | Female | 63086.19 (50327.90 to 75844.48) | 656.55 (523.87 to 789.23) |
| Chad | 2024 | Female | 62887.38 (41741.53 to 84033.23) | 630.31 (418.43 to 842.20) |
| Chad | 2025 | Female | 62735.98 (31953.06 to 93518.90) | 605.55 (308.46 to 902.65) |
| Chad | 2026 | Female | 62630.52 (21098.92 to 104162.11) | 582.18 (196.15 to 968.20) |
| Chad | 2027 | Female | 62565.73 (9266.19 to 115865.26) | 560.09 (82.97 to 1037.20) |
| Chad | 2028 | Female | 62542.13 (0.00 to 128567.79) | 539.23 (0.00 to 1108.49) |
| Chad | 2029 | Female | 62558.62 (0.00 to 142226.65) | 519.55 (0.00 to 1181.19) |
| Chad | 2030 | Female | 62615.69 (0.00 to 156817.01) | 500.99 (0.00 to 1254.68) |
| Chad | 2022 | Both | 139052.57 (125920.83 to 152184.31) | 756.13 (684.83 to 827.42) |
| Chad | 2023 | Both | 138992.62 (110280.10 to 167705.14) | 727.62 (577.36 to 877.88) |
| Chad | 2024 | Both | 139005.63 (91274.62 to 186736.64) | 700.57 (460.04 to 941.09) |
| Chad | 2025 | Both | 139097.78 (69407.85 to 208787.71) | 674.88 (336.77 to 1012.98) |
| Chad | 2026 | Both | 139261.00 (44974.19 to 233547.81) | 650.45 (210.07 to 1090.82) |
| Chad | 2027 | Both | 139485.23 (18167.24 to 260803.23) | 627.20 (81.70 to 1172.70) |
| Chad | 2028 | Both | 139773.54 (0.00 to 290420.99) | 605.11 (0.00 to 1257.30) |
| Chad | 2029 | Both | 140123.08 (0.00 to 322300.05) | 584.13 (0.00 to 1343.57) |
| Chad | 2030 | Both | 140529.62 (0.00 to 356364.21) | 564.18 (0.00 to 1430.69) |
| Comoros | 2022 | Male | 15083.01 (13649.34 to 16516.69) | 3915.17 (3548.31 to 4282.04) |
| Comoros | 2023 | Male | 14406.15 (11508.09 to 17304.20) | 3690.91 (2950.87 to 4430.95) |
| Comoros | 2024 | Male | 13755.26 (9202.85 to 18307.66) | 3480.12 (2329.81 to 4630.42) |
| Comoros | 2025 | Male | 13131.88 (6822.37 to 19441.38) | 3282.09 (1706.13 to 4858.05) |
| Comoros | 2026 | Male | 12535.97 (4420.85 to 20651.09) | 3096.07 (1092.57 to 5099.56) |
| Comoros | 2027 | Male | 11965.49 (2034.93 to 21896.04) | 2921.21 (497.37 to 5345.05) |
| Comoros | 2028 | Male | 11419.70 (0.00 to 23147.45) | 2756.82 (0.00 to 5587.55) |
| Comoros | 2029 | Male | 10897.70 (0.00 to 24383.34) | 2602.28 (0.00 to 5822.17) |
| Comoros | 2030 | Male | 10399.71 (0.00 to 25589.81) | 2457.05 (0.00 to 6045.58) |
| Comoros | 2022 | Female | 12606.77 (11426.61 to 13786.92) | 3255.15 (2955.77 to 3554.53) |
| Comoros | 2023 | Female | 12099.74 (9721.13 to 14478.35) | 3085.42 (2481.38 to 3689.47) |
| Comoros | 2024 | Female | 11609.00 (7863.73 to 15354.27) | 2924.90 (1982.78 to 3867.03) |
| Comoros | 2025 | Female | 11135.93 (5926.53 to 16345.33) | 2773.14 (1476.89 to 4069.40) |
| Comoros | 2026 | Female | 10680.62 (3953.73 to 17407.52) | 2629.62 (974.18 to 4285.06) |
| Comoros | 2027 | Female | 10241.87 (1975.87 to 18507.87) | 2493.81 (481.69 to 4505.94) |
| Comoros | 2028 | Female | 9819.41 (16.19 to 19622.64) | 2365.32 (4.36 to 4726.28) |
| Comoros | 2029 | Female | 9412.82 (0.00 to 20733.40) | 2243.77 (0.00 to 4941.91) |
| Comoros | 2030 | Female | 9022.57 (0.00 to 21827.99) | 2128.81 (0.00 to 5149.84) |
| Comoros | 2022 | Both | 27622.73 (25173.66 to 30071.79) | 3575.61 (3261.41 to 3889.81) |
| Comoros | 2023 | Both | 26438.38 (21378.59 to 31498.17) | 3378.82 (2733.47 to 4024.18) |
| Comoros | 2024 | Both | 25296.64 (17282.01 to 33311.26) | 3193.40 (2182.41 to 4204.38) |
| Comoros | 2025 | Both | 24200.32 (13035.12 to 35365.51) | 3018.74 (1626.51 to 4410.96) |
| Comoros | 2026 | Both | 23149.56 (8733.37 to 37565.74) | 2854.21 (1077.16 to 4631.27) |
| Comoros | 2027 | Both | 22141.08 (4442.33 to 39839.84) | 2699.15 (541.84 to 4856.45) |
| Comoros | 2028 | Both | 21173.84 (211.48 to 42136.21) | 2552.99 (25.73 to 5080.24) |
| Comoros | 2029 | Both | 20246.39 (0.00 to 44415.03) | 2415.22 (0.00 to 5298.13) |
| Comoros | 2030 | Both | 19359.40 (0.00 to 46650.62) | 2285.40 (0.00 to 5507.00) |
| Congo | 2022 | Male | 25536.55 (22471.22 to 28601.89) | 966.60 (851.18 to 1082.02) |
| Congo | 2023 | Male | 24734.71 (18245.59 to 31223.83) | 923.95 (681.82 to 1166.07) |
| Congo | 2024 | Male | 23952.50 (13509.65 to 34395.35) | 883.53 (498.49 to 1268.58) |
| Congo | 2025 | Male | 23191.49 (8440.04 to 37942.94) | 845.24 (307.72 to 1382.76) |
| Congo | 2026 | Male | 22451.69 (3154.11 to 41749.26) | 808.92 (113.72 to 1504.11) |
| Congo | 2027 | Male | 21732.79 (0.00 to 45728.22) | 774.45 (0.00 to 1629.46) |
| Congo | 2028 | Male | 21034.59 (0.00 to 49813.62) | 741.71 (0.00 to 1756.45) |
| Congo | 2029 | Male | 20356.84 (0.00 to 53953.16) | 710.60 (0.00 to 1883.31) |
| Congo | 2030 | Male | 19701.25 (0.00 to 58110.76) | 681.03 (0.00 to 2008.74) |
| Congo | 2022 | Female | 21595.08 (18834.05 to 24356.11) | 810.57 (707.50 to 913.64) |
| Congo | 2023 | Female | 20844.39 (15010.06 to 26678.71) | 772.73 (556.70 to 988.76) |
| Congo | 2024 | Female | 20118.68 (10755.75 to 29481.61) | 737.07 (394.20 to 1079.93) |
| Congo | 2025 | Female | 19419.52 (6230.96 to 32608.08) | 703.45 (225.81 to 1181.09) |
| Congo | 2026 | Female | 18746.84 (1540.03 to 35953.65) | 671.76 (55.26 to 1288.26) |
| Congo | 2027 | Female | 18099.73 (0.00 to 39442.32) | 641.87 (0.00 to 1398.69) |
| Congo | 2028 | Female | 17477.69 (0.00 to 43017.58) | 613.67 (0.00 to 1510.37) |
| Congo | 2029 | Female | 16879.99 (0.00 to 46635.67) | 587.05 (0.00 to 1621.84) |
| Congo | 2030 | Female | 16307.67 (0.00 to 50268.03) | 561.92 (0.00 to 1732.06) |
| Congo | 2022 | Both | 47139.74 (41403.79 to 52875.69) | 888.41 (780.61 to 996.21) |
| Congo | 2023 | Both | 45615.82 (33385.87 to 57845.77) | 848.74 (621.32 to 1076.15) |
| Congo | 2024 | Both | 44133.95 (24421.50 to 63846.41) | 811.20 (448.96 to 1173.45) |
| Congo | 2025 | Both | 42697.22 (14840.76 to 70553.67) | 775.69 (269.67 to 1281.72) |
| Congo | 2026 | Both | 41305.44 (4864.27 to 77746.61) | 742.07 (87.43 to 1396.72) |
| Congo | 2027 | Both | 39957.84 (0.00 to 85263.61) | 710.23 (0.00 to 1515.48) |
| Congo | 2028 | Both | 38653.98 (0.00 to 92981.87) | 680.04 (0.00 to 1635.82) |
| Congo | 2029 | Both | 37393.03 (0.00 to 100804.87) | 651.43 (0.00 to 1756.12) |
| Congo | 2030 | Both | 36177.61 (0.00 to 108666.45) | 624.29 (0.00 to 1875.16) |
| Cote d'Ivoire | 2022 | Male | 1193204.39 (1126092.17 to 1260316.61) | 8183.05 (7723.03 to 8643.07) |
| Cote d'Ivoire | 2023 | Male | 1150370.88 (1012530.68 to 1288211.09) | 7707.08 (6783.71 to 8630.46) |
| Cote d'Ivoire | 2024 | Male | 1108729.03 (889624.88 to 1327833.19) | 7259.63 (5825.07 to 8694.20) |
| Cote d'Ivoire | 2025 | Male | 1068331.74 (761441.18 to 1375222.31) | 6839.04 (4874.49 to 8803.59) |
| Cote d'Ivoire | 2026 | Male | 1029022.49 (630447.93 to 1427597.05) | 6443.02 (3947.45 to 8938.58) |
| Cote d'Ivoire | 2027 | Male | 990747.07 (498491.05 to 1483003.10) | 6070.26 (3054.25 to 9086.26) |
| Cote d'Ivoire | 2028 | Male | 953572.20 (367067.61 to 1540076.79) | 5719.66 (2201.74 to 9237.58) |
| Cote d'Ivoire | 2029 | Male | 917558.43 (237323.30 to 1597793.55) | 5390.30 (1394.20 to 9386.40) |
| Cote d'Ivoire | 2030 | Male | 882827.61 (110140.40 to 1655514.82) | 5080.95 (633.91 to 9528.00) |
| Cote d'Ivoire | 2022 | Female | 861788.08 (813445.77 to 910130.39) | 6222.59 (5873.78 to 6571.40) |
| Cote d'Ivoire | 2023 | Female | 838434.49 (734732.92 to 942136.06) | 5900.67 (5170.96 to 6630.38) |
| Cote d'Ivoire | 2024 | Female | 815529.62 (647449.86 to 983609.39) | 5597.04 (4443.56 to 6750.52) |
| Cote d'Ivoire | 2025 | Female | 793078.71 (554297.39 to 1031860.03) | 5310.31 (3711.52 to 6909.11) |
| Cote d'Ivoire | 2026 | Female | 770919.57 (457027.55 to 1084811.59) | 5038.58 (2987.08 to 7090.09) |
| Cote d'Ivoire | 2027 | Female | 749088.59 (357082.20 to 1141094.98) | 4781.53 (2279.33 to 7283.74) |
| Cote d'Ivoire | 2028 | Female | 727692.33 (255628.32 to 1199756.34) | 4538.79 (1594.43 to 7483.14) |
| Cote d'Ivoire | 2029 | Female | 706759.33 (153553.34 to 1259965.33) | 4309.64 (936.35 to 7682.94) |
| Cote d'Ivoire | 2030 | Female | 686356.72 (51575.47 to 1321137.96) | 4093.08 (307.58 to 7878.58) |
| Cote d'Ivoire | 2022 | Both | 2059852.09 (1947173.00 to 2172531.18) | 7245.15 (6848.95 to 7641.36) |
| Cote d'Ivoire | 2023 | Both | 1993138.49 (1754907.71 to 2231369.27) | 6840.98 (6023.36 to 7658.59) |
| Cote d'Ivoire | 2024 | Both | 1928046.65 (1545048.81 to 2311044.48) | 6460.58 (5177.25 to 7743.91) |
| Cote d'Ivoire | 2025 | Both | 1864623.47 (1324201.49 to 2405045.45) | 6102.36 (4333.74 to 7870.98) |
| Cote d'Ivoire | 2026 | Both | 1802550.74 (1096568.48 to 2508533.01) | 5764.20 (3506.63 to 8021.78) |
| Cote d'Ivoire | 2027 | Both | 1741845.90 (865465.76 to 2618226.03) | 5445.37 (2705.64 to 8185.11) |
| Cote d'Ivoire | 2028 | Both | 1682678.23 (633556.84 to 2731799.62) | 5145.08 (1937.22 to 8352.95) |
| Cote d'Ivoire | 2029 | Both | 1625137.14 (402878.73 to 2847395.56) | 4862.49 (1205.44 to 8519.55) |
| Cote d'Ivoire | 2030 | Both | 1569409.53 (175036.69 to 2963782.38) | 4596.45 (512.65 to 8680.25) |
| Democratic Republic of the Congo | 2022 | Male | 1049625.41 (964361.86 to 1134888.96) | 2254.31 (2071.23 to 2437.38) |
| Democratic Republic of the Congo | 2023 | Male | 1022895.18 (839686.64 to 1206103.72) | 2137.35 (1754.55 to 2520.14) |
| Democratic Republic of the Congo | 2024 | Male | 996713.86 (699235.41 to 1294192.30) | 2027.07 (1422.08 to 2632.05) |
| Democratic Republic of the Congo | 2025 | Male | 971151.80 (547714.37 to 1394589.22) | 1923.15 (1084.63 to 2761.66) |
| Democratic Republic of the Congo | 2026 | Male | 946262.32 (388296.32 to 1504228.33) | 1825.33 (749.03 to 2901.64) |
| Democratic Republic of the Congo | 2027 | Male | 921856.13 (223219.35 to 1620492.92) | 1733.12 (419.67 to 3046.58) |
| Democratic Republic of the Congo | 2028 | Male | 897884.65 (54347.26 to 1741422.04) | 1646.14 (99.64 to 3192.63) |
| Democratic Republic of the Congo | 2029 | Male | 874313.30 (0.00 to 1865408.33) | 1564.03 (0.00 to 3336.95) |
| Democratic Republic of the Congo | 2030 | Male | 851220.48 (0.00 to 1991342.75) | 1486.55 (0.00 to 3477.62) |
| Democratic Republic of the Congo | 2022 | Female | 902602.31 (838847.91 to 966356.70) | 1936.37 (1799.65 to 2073.09) |
| Democratic Republic of the Congo | 2023 | Female | 881242.37 (743023.72 to 1019461.01) | 1839.17 (1550.73 to 2127.61) |
| Democratic Republic of the Congo | 2024 | Female | 860334.80 (635007.31 to 1085662.28) | 1747.45 (1289.79 to 2205.10) |
| Democratic Republic of the Congo | 2025 | Female | 839903.68 (518219.06 to 1161588.31) | 1660.86 (1024.76 to 2296.96) |
| Democratic Republic of the Congo | 2026 | Female | 819895.15 (394969.21 to 1244821.09) | 1579.04 (760.68 to 2397.40) |
| Democratic Republic of the Congo | 2027 | Female | 800204.08 (266966.69 to 1333441.47) | 1501.71 (501.01 to 2502.40) |
| Democratic Republic of the Congo | 2028 | Female | 780835.03 (135629.21 to 1426040.85) | 1428.65 (248.16 to 2609.14) |
| Democratic Republic of the Congo | 2029 | Female | 761782.93 (2108.06 to 1521457.81) | 1359.64 (3.77 to 2715.52) |
| Democratic Republic of the Congo | 2030 | Female | 743090.47 (0.00 to 1618824.33) | 1294.44 (0.00 to 2819.94) |
| Democratic Republic of the Congo | 2022 | Both | 1951026.48 (1803091.93 to 2098961.02) | 2093.96 (1935.21 to 2252.70) |
| Democratic Republic of the Congo | 2023 | Both | 1903147.02 (1583008.91 to 2223285.12) | 1987.14 (1652.88 to 2321.39) |
| Democratic Republic of the Congo | 2024 | Both | 1856205.26 (1334941.31 to 2377469.21) | 1886.31 (1356.60 to 2416.02) |
| Democratic Republic of the Congo | 2025 | Both | 1810325.78 (1066978.06 to 2553673.50) | 1791.19 (1055.70 to 2526.67) |
| Democratic Republic of the Congo | 2026 | Both | 1765581.09 (784629.41 to 2746532.77) | 1701.53 (756.17 to 2646.89) |
| Democratic Republic of the Congo | 2027 | Both | 1721646.80 (491804.48 to 2951489.12) | 1616.92 (461.89 to 2771.95) |
| Democratic Republic of the Congo | 2028 | Both | 1678429.60 (191762.25 to 3165096.94) | 1537.02 (175.61 to 2898.43) |
| Democratic Republic of the Congo | 2029 | Both | 1635875.62 (0.00 to 3384546.76) | 1461.52 (0.00 to 3023.81) |
| Democratic Republic of the Congo | 2030 | Both | 1594131.91 (0.00 to 3607893.42) | 1390.22 (0.00 to 3146.39) |
| Dominican Republic | 2022 | Male | 77363.01 (54433.25 to 100292.77) | 1408.14 (990.90 to 1825.38) |
| Dominican Republic | 2023 | Male | 74623.58 (26204.66 to 123042.50) | 1348.08 (473.44 to 2222.71) |
| Dominican Republic | 2024 | Male | 71959.44 (0.00 to 149602.49) | 1290.76 (0.00 to 2683.43) |
| Dominican Republic | 2025 | Male | 69368.70 (0.00 to 178605.31) | 1236.03 (0.00 to 3182.41) |
| Dominican Republic | 2026 | Male | 66850.01 (0.00 to 209131.87) | 1183.76 (0.00 to 3703.22) |
| Dominican Republic | 2027 | Male | 64400.05 (0.00 to 240499.93) | 1133.80 (0.00 to 4234.12) |
| Dominican Republic | 2028 | Male | 62016.45 (0.00 to 272180.80) | 1085.96 (0.00 to 4766.12) |
| Dominican Republic | 2029 | Male | 59696.34 (0.00 to 303748.09) | 1040.14 (0.00 to 5292.44) |
| Dominican Republic | 2030 | Male | 57439.06 (0.00 to 334862.88) | 996.23 (0.00 to 5807.89) |
| Dominican Republic | 2022 | Female | 65096.46 (47243.68 to 82949.23) | 1186.98 (861.58 to 1512.39) |
| Dominican Republic | 2023 | Female | 62766.00 (25035.69 to 100496.31) | 1134.13 (452.43 to 1815.82) |
| Dominican Republic | 2024 | Female | 60493.00 (0.23 to 120985.77) | 1083.66 (0.04 to 2167.29) |
| Dominican Republic | 2025 | Female | 58276.60 (0.00 to 143342.90) | 1035.46 (0.00 to 2546.89) |
| Dominican Republic | 2026 | Female | 56115.54 (0.00 to 166837.59) | 989.40 (0.00 to 2941.57) |
| Dominican Republic | 2027 | Female | 54007.54 (0.00 to 190925.22) | 945.35 (0.00 to 3341.94) |
| Dominican Republic | 2028 | Female | 51952.41 (0.00 to 215186.41) | 903.17 (0.00 to 3740.91) |
| Dominican Republic | 2029 | Female | 49949.01 (0.00 to 239282.94) | 862.78 (0.00 to 4133.18) |
| Dominican Republic | 2030 | Female | 47997.19 (0.00 to 262943.62) | 824.10 (0.00 to 4514.66) |
| Dominican Republic | 2022 | Both | 143020.17 (102084.62 to 183955.72) | 1302.77 (929.95 to 1675.59) |
| Dominican Republic | 2023 | Both | 137973.52 (51499.52 to 224447.51) | 1246.39 (465.25 to 2027.53) |
| Dominican Republic | 2024 | Both | 133053.62 (0.00 to 271741.00) | 1192.53 (0.00 to 2435.54) |
| Dominican Republic | 2025 | Both | 128258.18 (0.00 to 323381.72) | 1141.05 (0.00 to 2876.97) |
| Dominican Republic | 2026 | Both | 123585.40 (0.00 to 377712.23) | 1091.84 (0.00 to 3336.98) |
| Dominican Republic | 2027 | Both | 119030.23 (0.00 to 433497.87) | 1044.77 (0.00 to 3804.94) |
| Dominican Republic | 2028 | Both | 114589.49 (0.00 to 489778.61) | 999.65 (0.00 to 4272.70) |
| Dominican Republic | 2029 | Both | 110259.22 (0.00 to 545778.91) | 956.40 (0.00 to 4734.13) |
| Dominican Republic | 2030 | Both | 106039.13 (0.00 to 600879.45) | 914.93 (0.00 to 5184.53) |
| Egypt | 2022 | Male | 297049.41 (181681.32 to 412417.50) | 550.16 (336.50 to 763.82) |
| Egypt | 2023 | Male | 285804.31 (38708.18 to 532900.45) | 520.96 (70.56 to 971.37) |
| Egypt | 2024 | Male | 274994.46 (0.00 to 672258.97) | 493.50 (0.00 to 1206.43) |
| Egypt | 2025 | Male | 264554.37 (0.00 to 823597.77) | 467.63 (0.00 to 1455.82) |
| Egypt | 2026 | Male | 254443.69 (0.00 to 982131.93) | 443.21 (0.00 to 1710.77) |
| Egypt | 2027 | Male | 244679.77 (0.00 to 1144513.48) | 420.17 (0.00 to 1965.37) |
| Egypt | 2028 | Male | 235278.36 (0.00 to 1308289.97) | 398.44 (0.00 to 2215.55) |
| Egypt | 2029 | Male | 226249.55 (0.00 to 1471641.57) | 377.95 (0.00 to 2458.38) |
| Egypt | 2030 | Male | 217569.81 (0.00 to 1633005.73) | 358.61 (0.00 to 2691.62) |
| Egypt | 2022 | Female | 282467.35 (172746.44 to 392188.25) | 550.37 (336.59 to 764.14) |
| Egypt | 2023 | Female | 272483.03 (36449.86 to 508516.21) | 521.25 (69.73 to 972.78) |
| Egypt | 2024 | Female | 262781.54 (0.00 to 643327.22) | 493.74 (0.00 to 1208.75) |
| Egypt | 2025 | Female | 253304.70 (0.00 to 790008.00) | 467.70 (0.00 to 1458.67) |
| Egypt | 2026 | Female | 244025.52 (0.00 to 943884.93) | 443.00 (0.00 to 1713.52) |
| Egypt | 2027 | Female | 234972.91 (0.00 to 1101625.04) | 419.60 (0.00 to 1967.19) |
| Egypt | 2028 | Female | 226177.68 (0.00 to 1260758.25) | 397.45 (0.00 to 2215.45) |
| Egypt | 2029 | Female | 217655.59 (0.00 to 1419373.24) | 376.50 (0.00 to 2455.22) |
| Egypt | 2030 | Female | 209384.92 (0.00 to 1575755.57) | 356.65 (0.00 to 2684.02) |
| Egypt | 2022 | Both | 579163.48 (354274.41 to 804052.55) | 549.93 (336.39 to 763.46) |
| Egypt | 2023 | Both | 557913.93 (75060.51 to 1040767.36) | 520.76 (70.06 to 971.45) |
| Egypt | 2024 | Both | 537398.35 (0.00 to 1314824.47) | 493.27 (0.00 to 1206.87) |
| Egypt | 2025 | Both | 517485.33 (0.00 to 1612727.88) | 467.33 (0.00 to 1456.42) |
| Egypt | 2026 | Both | 498090.39 (0.00 to 1924971.26) | 442.77 (0.00 to 1711.19) |
| Egypt | 2027 | Both | 479265.85 (0.00 to 2244880.90) | 419.55 (0.00 to 1965.16) |
| Egypt | 2028 | Both | 461067.36 (0.00 to 2567576.10) | 397.62 (0.00 to 2214.23) |
| Egypt | 2029 | Both | 443524.99 (0.00 to 2889369.47) | 376.91 (0.00 to 2455.43) |
| Egypt | 2030 | Both | 426589.00 (0.00 to 3206981.26) | 357.34 (0.00 to 2686.39) |
| Equatorial Guinea | 2022 | Male | 11122.79 (9017.17 to 13228.42) | 1343.09 (1090.07 to 1596.12) |
| Equatorial Guinea | 2023 | Male | 10821.01 (6330.74 to 15311.28) | 1276.20 (747.17 to 1805.22) |
| Equatorial Guinea | 2024 | Male | 10523.61 (3260.54 to 17786.68) | 1213.23 (376.22 to 2050.25) |
| Equatorial Guinea | 2025 | Male | 10234.12 (0.00 to 20543.05) | 1153.97 (0.00 to 2316.16) |
| Equatorial Guinea | 2026 | Male | 9953.98 (0.00 to 23506.11) | 1098.20 (0.00 to 2593.22) |
| Equatorial Guinea | 2027 | Male | 9679.62 (0.00 to 26611.01) | 1045.69 (0.00 to 2874.66) |
| Equatorial Guinea | 2028 | Male | 9412.01 (0.00 to 29814.92) | 996.20 (0.00 to 3155.63) |
| Equatorial Guinea | 2029 | Male | 9151.44 (0.00 to 33083.20) | 949.53 (0.00 to 3432.56) |
| Equatorial Guinea | 2030 | Male | 8899.68 (0.00 to 36394.64) | 905.51 (0.00 to 3702.94) |
| Equatorial Guinea | 2022 | Female | 8273.31 (6805.90 to 9740.71) | 1141.53 (940.56 to 1342.49) |
| Equatorial Guinea | 2023 | Female | 8070.51 (4957.94 to 11183.09) | 1084.74 (667.06 to 1502.42) |
| Equatorial Guinea | 2024 | Female | 7868.11 (2833.45 to 12902.78) | 1031.25 (371.77 to 1690.74) |
| Equatorial Guinea | 2025 | Female | 7669.47 (515.43 to 14823.51) | 980.88 (66.18 to 1895.58) |
| Equatorial Guinea | 2026 | Female | 7475.77 (0.00 to 16894.18) | 933.43 (0.00 to 2109.22) |
| Equatorial Guinea | 2027 | Female | 7284.18 (0.00 to 19068.87) | 888.69 (0.00 to 2326.32) |
| Equatorial Guinea | 2028 | Female | 7095.94 (0.00 to 21318.36) | 846.52 (0.00 to 2543.08) |
| Equatorial Guinea | 2029 | Female | 6911.73 (0.00 to 23619.26) | 806.75 (0.00 to 2756.78) |
| Equatorial Guinea | 2030 | Female | 6733.27 (0.00 to 25957.86) | 769.25 (0.00 to 2965.50) |
| Equatorial Guinea | 2022 | Both | 19300.59 (15818.73 to 22782.44) | 1242.87 (1019.34 to 1466.40) |
| Equatorial Guinea | 2023 | Both | 18808.32 (11317.64 to 26298.99) | 1181.49 (711.25 to 1651.73) |
| Equatorial Guinea | 2024 | Both | 18320.10 (6158.41 to 30481.78) | 1123.68 (377.91 to 1869.45) |
| Equatorial Guinea | 2025 | Both | 17842.74 (536.74 to 35148.73) | 1069.22 (32.28 to 2106.16) |
| Equatorial Guinea | 2026 | Both | 17378.98 (0.00 to 40176.28) | 1017.93 (0.00 to 2353.14) |
| Equatorial Guinea | 2027 | Both | 16922.59 (0.00 to 45454.76) | 969.60 (0.00 to 2604.31) |
| Equatorial Guinea | 2028 | Both | 16475.87 (0.00 to 50912.76) | 924.03 (0.00 to 2855.34) |
| Equatorial Guinea | 2029 | Both | 16039.66 (0.00 to 56492.20) | 881.05 (0.00 to 3103.03) |
| Equatorial Guinea | 2030 | Both | 15617.39 (0.00 to 62157.84) | 840.48 (0.00 to 3345.12) |
| Eritrea | 2022 | Male | 14892.94 (12986.77 to 16799.11) | 464.23 (405.29 to 523.18) |
| Eritrea | 2023 | Male | 14759.13 (10717.81 to 18800.46) | 452.14 (328.55 to 575.73) |
| Eritrea | 2024 | Male | 14621.04 (8012.04 to 21230.05) | 440.42 (241.47 to 639.37) |
| Eritrea | 2025 | Male | 14484.00 (4963.80 to 24004.21) | 429.09 (147.14 to 711.05) |
| Eritrea | 2026 | Male | 14345.34 (1629.50 to 27061.18) | 418.15 (47.56 to 788.74) |
| Eritrea | 2027 | Male | 14199.41 (0.00 to 30346.54) | 407.53 (0.00 to 870.91) |
| Eritrea | 2028 | Male | 14042.19 (0.00 to 33811.27) | 397.19 (0.00 to 956.34) |
| Eritrea | 2029 | Male | 13879.38 (0.00 to 37433.03) | 387.12 (0.00 to 1044.05) |
| Eritrea | 2030 | Male | 13716.71 (0.00 to 41199.57) | 377.33 (0.00 to 1133.31) |
| Eritrea | 2022 | Female | 11317.43 (9914.20 to 12720.66) | 349.91 (307.01 to 392.81) |
| Eritrea | 2023 | Female | 11170.11 (8210.15 to 14130.08) | 339.29 (249.60 to 428.98) |
| Eritrea | 2024 | Female | 11026.02 (6204.02 to 15848.02) | 329.21 (185.37 to 473.05) |
| Eritrea | 2025 | Female | 10888.21 (3964.61 to 17811.80) | 319.63 (116.47 to 522.78) |
| Eritrea | 2026 | Female | 10755.16 (1532.41 to 19977.91) | 310.53 (44.31 to 576.74) |
| Eritrea | 2027 | Female | 10623.05 (0.00 to 22309.03) | 301.88 (0.00 to 633.91) |
| Eritrea | 2028 | Female | 10488.71 (0.00 to 24772.58) | 293.63 (0.00 to 693.47) |
| Eritrea | 2029 | Female | 10356.47 (0.00 to 27356.80) | 285.77 (0.00 to 754.84) |
| Eritrea | 2030 | Female | 10229.99 (0.00 to 30056.05) | 278.28 (0.00 to 817.58) |
| Eritrea | 2022 | Both | 26185.92 (23058.01 to 29313.83) | 406.46 (358.16 to 454.76) |
| Eritrea | 2023 | Both | 25933.33 (19179.72 to 32686.94) | 395.54 (292.64 to 498.43) |
| Eritrea | 2024 | Both | 25678.35 (14573.65 to 36783.04) | 385.04 (218.59 to 551.48) |
| Eritrea | 2025 | Both | 25428.83 (9391.30 to 41466.35) | 374.94 (138.52 to 611.37) |
| Eritrea | 2026 | Both | 25180.29 (3725.94 to 46634.64) | 365.24 (54.08 to 676.40) |
| Eritrea | 2027 | Both | 24924.19 (0.00 to 52199.61) | 355.89 (0.00 to 745.33) |
| Eritrea | 2028 | Both | 24652.89 (0.00 to 58080.33) | 346.86 (0.00 to 817.16) |
| Eritrea | 2029 | Both | 24376.35 (0.00 to 64243.10) | 338.12 (0.00 to 891.10) |
| Eritrea | 2030 | Both | 24103.82 (0.00 to 70669.43) | 329.68 (0.00 to 966.56) |
| Ethiopia | 2022 | Male | 392505.60 (362416.92 to 422594.28) | 653.21 (603.18 to 703.24) |
| Ethiopia | 2023 | Male | 391267.64 (328784.94 to 453750.34) | 633.33 (532.21 to 734.44) |
| Ethiopia | 2024 | Male | 389960.18 (287978.32 to 491942.05) | 614.29 (453.66 to 774.93) |
| Ethiopia | 2025 | Male | 388626.23 (241396.66 to 535855.79) | 596.08 (370.27 to 821.90) |
| Ethiopia | 2026 | Male | 387230.90 (189827.33 to 584634.48) | 578.63 (283.66 to 873.59) |
| Ethiopia | 2027 | Male | 385719.68 (133830.91 to 637608.45) | 561.87 (194.95 to 928.78) |
| Ethiopia | 2028 | Male | 384069.81 (73890.38 to 694249.23) | 545.73 (105.00 to 986.47) |
| Ethiopia | 2029 | Male | 382274.38 (10425.16 to 754123.59) | 530.19 (14.46 to 1045.92) |
| Ethiopia | 2030 | Male | 380354.42 (0.00 to 816904.90) | 515.22 (0.00 to 1106.57) |
| Ethiopia | 2022 | Female | 308181.86 (285212.79 to 331150.93) | 521.71 (482.87 to 560.55) |
| Ethiopia | 2023 | Female | 305790.89 (258025.82 to 353555.96) | 503.45 (424.83 to 582.07) |
| Ethiopia | 2024 | Female | 303423.95 (225668.76 to 381179.13) | 486.14 (361.57 to 610.70) |
| Ethiopia | 2025 | Female | 301121.01 (189243.03 to 412998.99) | 469.73 (295.21 to 644.24) |
| Ethiopia | 2026 | Female | 298862.70 (149373.88 to 448351.52) | 454.16 (227.00 to 681.33) |
| Ethiopia | 2027 | Female | 296605.10 (106494.92 to 486715.27) | 439.37 (157.76 to 720.99) |
| Ethiopia | 2028 | Female | 294333.36 (60971.62 to 527695.10) | 425.29 (88.10 to 762.49) |
| Ethiopia | 2029 | Female | 292046.74 (13110.67 to 570982.81) | 411.89 (18.49 to 805.28) |
| Ethiopia | 2030 | Female | 289767.79 (0.00 to 616366.27) | 399.13 (0.00 to 848.99) |
| Ethiopia | 2022 | Both | 701786.79 (649258.30 to 754315.29) | 588.95 (544.89 to 633.01) |
| Ethiopia | 2023 | Both | 698271.04 (588489.46 to 808052.62) | 569.93 (480.34 to 659.53) |
| Ethiopia | 2024 | Both | 694707.78 (515364.99 to 874050.58) | 551.81 (409.36 to 694.26) |
| Ethiopia | 2025 | Both | 691178.23 (432356.12 to 950000.35) | 534.54 (334.38 to 734.71) |
| Ethiopia | 2026 | Both | 687625.87 (340868.60 to 1034383.15) | 518.07 (256.82 to 779.32) |
| Ethiopia | 2027 | Both | 683956.69 (241897.91 to 1126015.47) | 502.33 (177.66 to 827.00) |
| Ethiopia | 2028 | Both | 680130.31 (136290.08 to 1223970.54) | 487.26 (97.64 to 876.87) |
| Ethiopia | 2029 | Both | 676139.08 (24771.19 to 1327506.98) | 472.81 (17.32 to 928.29) |
| Ethiopia | 2030 | Both | 672026.20 (0.00 to 1436084.85) | 458.96 (0.00 to 980.78) |
| Fiji | 2022 | Male | 48955.00 (43979.27 to 53930.72) | 10252.63 (9214.53 to 11290.73) |
| Fiji | 2023 | Male | 46506.78 (36162.64 to 56850.92) | 9673.92 (7524.02 to 11823.81) |
| Fiji | 2024 | Male | 44169.94 (27836.49 to 60503.39) | 9127.41 (5753.29 to 12501.53) |
| Fiji | 2025 | Male | 41946.68 (19311.70 to 64581.67) | 8611.45 (3965.33 to 13257.57) |
| Fiji | 2026 | Male | 39830.82 (10783.45 to 68878.19) | 8124.51 (2200.10 to 14048.93) |
| Fiji | 2027 | Male | 37810.32 (2386.45 to 73234.18) | 7664.89 (484.20 to 14845.59) |
| Fiji | 2028 | Male | 35884.94 (0.00 to 77548.30) | 7231.16 (0.00 to 15626.39) |
| Fiji | 2029 | Male | 34051.00 (0.00 to 81741.49) | 6821.88 (0.00 to 16376.06) |
| Fiji | 2030 | Male | 32309.26 (0.00 to 85766.78) | 6435.73 (0.00 to 17083.77) |
| Fiji | 2022 | Female | 35513.58 (32199.49 to 38827.68) | 7635.60 (6927.50 to 8343.71) |
| Fiji | 2023 | Female | 33925.49 (27015.45 to 40835.54) | 7239.34 (5766.83 to 8711.86) |
| Fiji | 2024 | Female | 32403.12 (21438.99 to 43367.24) | 6863.89 (4542.58 to 9185.20) |
| Fiji | 2025 | Female | 30948.95 (15673.27 to 46224.64) | 6508.23 (3296.74 to 9719.72) |
| Fiji | 2026 | Female | 29559.27 (9846.12 to 49272.43) | 6171.45 (2056.30 to 10286.61) |
| Fiji | 2027 | Female | 28225.71 (4046.57 to 52404.85) | 5852.45 (839.50 to 10865.41) |
| Fiji | 2028 | Female | 26948.26 (0.00 to 55552.95) | 5550.19 (0.00 to 11441.16) |
| Fiji | 2029 | Female | 25724.78 (0.00 to 58661.70) | 5263.73 (0.00 to 12002.89) |
| Fiji | 2030 | Female | 24556.99 (0.00 to 61698.47) | 4992.31 (0.00 to 12542.72) |
| Fiji | 2022 | Both | 84224.48 (76233.31 to 92215.66) | 8935.41 (8089.77 to 9781.05) |
| Fiji | 2023 | Both | 80212.12 (63409.77 to 97014.48) | 8448.98 (6680.10 to 10217.85) |
| Fiji | 2024 | Both | 76375.43 (49705.86 to 103045.00) | 7989.00 (5199.89 to 10778.11) |
| Fiji | 2025 | Both | 72718.61 (35616.13 to 109821.09) | 7554.09 (3700.23 to 11407.94) |
| Fiji | 2026 | Both | 69231.63 (21456.38 to 117006.88) | 7143.00 (2214.06 to 12071.95) |
| Fiji | 2027 | Both | 65894.56 (7446.49 to 124342.64) | 6754.39 (763.51 to 12745.28) |
| Fiji | 2028 | Both | 62708.05 (0.00 to 131662.26) | 6387.10 (0.00 to 13410.22) |
| Fiji | 2029 | Both | 59666.11 (0.00 to 138833.46) | 6039.93 (0.00 to 14053.79) |
| Fiji | 2030 | Both | 56770.88 (0.00 to 145776.70) | 5711.78 (0.00 to 14666.63) |
| Gabon | 2022 | Male | 12844.41 (11251.55 to 14437.27) | 1439.33 (1262.58 to 1616.08) |
| Gabon | 2023 | Male | 12386.32 (9078.36 to 15694.29) | 1368.56 (1003.86 to 1733.26) |
| Gabon | 2024 | Male | 11941.38 (6665.69 to 17217.06) | 1301.60 (727.03 to 1876.17) |
| Gabon | 2025 | Male | 11510.50 (4107.85 to 18913.16) | 1238.24 (442.22 to 2034.25) |
| Gabon | 2026 | Male | 11093.46 (1465.60 to 20721.32) | 1178.27 (155.90 to 2200.64) |
| Gabon | 2027 | Male | 10689.66 (0.00 to 22596.58) | 1121.49 (0.00 to 2370.50) |
| Gabon | 2028 | Male | 10299.61 (0.00 to 24506.77) | 1067.69 (0.00 to 2540.29) |
| Gabon | 2029 | Male | 9921.76 (0.00 to 26422.58) | 1016.69 (0.00 to 2707.41) |
| Gabon | 2030 | Male | 9556.35 (0.00 to 28323.98) | 968.34 (0.00 to 2869.95) |
| Gabon | 2022 | Female | 11739.88 (10291.79 to 13187.96) | 1230.73 (1080.57 to 1380.90) |
| Gabon | 2023 | Female | 11311.89 (8313.93 to 14309.86) | 1168.72 (859.73 to 1477.72) |
| Gabon | 2024 | Female | 10897.17 (6122.92 to 15671.42) | 1110.12 (624.20 to 1596.04) |
| Gabon | 2025 | Female | 10496.54 (3804.51 to 17188.57) | 1054.76 (382.60 to 1726.91) |
| Gabon | 2026 | Female | 10109.84 (1413.49 to 18806.20) | 1002.45 (140.38 to 1864.53) |
| Gabon | 2027 | Female | 9736.42 (0.00 to 20483.88) | 953.01 (0.00 to 2004.82) |
| Gabon | 2028 | Female | 9376.46 (0.00 to 22192.52) | 906.25 (0.00 to 2144.81) |
| Gabon | 2029 | Female | 9028.62 (0.00 to 23906.29) | 862.02 (0.00 to 2282.37) |
| Gabon | 2030 | Female | 8693.19 (0.00 to 25608.05) | 820.17 (0.00 to 2415.94) |
| Gabon | 2022 | Both | 24569.08 (21643.61 to 27494.54) | 1330.73 (1173.16 to 1488.31) |
| Gabon | 2023 | Both | 23698.94 (17533.30 to 29864.58) | 1265.33 (936.53 to 1594.13) |
| Gabon | 2024 | Both | 22854.16 (12974.01 to 32734.30) | 1203.45 (683.42 to 1723.48) |
| Gabon | 2025 | Both | 22036.34 (8138.23 to 35934.45) | 1144.89 (422.98 to 1866.81) |
| Gabon | 2026 | Both | 21245.05 (3139.57 to 39350.53) | 1089.48 (161.12 to 2017.85) |
| Gabon | 2027 | Both | 20479.23 (0.00 to 42898.26) | 1037.02 (0.00 to 2172.18) |
| Gabon | 2028 | Both | 19739.72 (0.00 to 46517.02) | 987.33 (0.00 to 2326.59) |
| Gabon | 2029 | Both | 19023.67 (0.00 to 50151.92) | 940.24 (0.00 to 2478.69) |
| Gabon | 2030 | Both | 18331.53 (0.00 to 53765.47) | 895.62 (0.00 to 2626.75) |
| Ghana | 2022 | Male | 137463.85 (127408.69 to 147519.01) | 844.78 (783.15 to 906.41) |
| Ghana | 2023 | Male | 134961.20 (113909.15 to 156013.26) | 813.83 (686.96 to 940.70) |
| Ghana | 2024 | Male | 132486.42 (98401.11 to 166571.73) | 784.37 (582.62 to 986.13) |
| Ghana | 2025 | Male | 130071.51 (81428.78 to 178714.24) | 756.34 (473.52 to 1039.16) |
| Ghana | 2026 | Male | 127708.40 (63313.92 to 192102.88) | 729.67 (361.77 to 1097.57) |
| Ghana | 2027 | Male | 125372.71 (44284.27 to 206461.14) | 704.23 (248.77 to 1159.70) |
| Ghana | 2028 | Male | 123071.20 (24536.83 to 221605.56) | 679.94 (135.57 to 1224.31) |
| Ghana | 2029 | Male | 120802.86 (4227.96 to 237377.77) | 656.73 (23.00 to 1290.46) |
| Ghana | 2030 | Male | 118580.82 (0.00 to 253675.07) | 634.56 (0.00 to 1357.47) |
| Ghana | 2022 | Female | 119460.38 (111897.14 to 127023.62) | 697.14 (653.18 to 741.10) |
| Ghana | 2023 | Female | 116727.41 (100599.24 to 132855.58) | 668.80 (576.47 to 761.13) |
| Ghana | 2024 | Female | 114075.62 (87893.80 to 140257.44) | 642.04 (494.73 to 789.36) |
| Ghana | 2025 | Female | 111527.35 (74191.26 to 148863.44) | 616.79 (410.34 to 823.25) |
| Ghana | 2026 | Female | 109065.87 (59734.43 to 158397.31) | 592.92 (324.76 to 861.08) |
| Ghana | 2027 | Female | 106674.61 (44699.30 to 168649.92) | 570.33 (239.00 to 901.66) |
| Ghana | 2028 | Female | 104365.05 (29234.71 to 179495.39) | 548.98 (153.79 to 944.16) |
| Ghana | 2029 | Female | 102137.71 (13449.65 to 190825.77) | 528.80 (69.64 to 987.95) |
| Ghana | 2030 | Female | 100000.43 (0.00 to 202572.17) | 509.73 (0.00 to 1032.56) |
| Ghana | 2022 | Both | 256515.37 (239261.92 to 273768.82) | 767.83 (716.27 to 819.39) |
| Ghana | 2023 | Both | 251333.90 (214639.23 to 288028.56) | 738.42 (630.65 to 846.19) |
| Ghana | 2024 | Both | 246255.83 (186628.80 to 305882.87) | 710.52 (538.50 to 882.54) |
| Ghana | 2025 | Both | 241335.64 (156165.35 to 326505.93) | 684.07 (442.67 to 925.47) |
| Ghana | 2026 | Both | 236553.28 (123810.75 to 349295.81) | 658.98 (344.92 to 973.04) |
| Ghana | 2027 | Both | 231868.87 (89965.22 to 373772.52) | 635.14 (246.44 to 1023.84) |
| Ghana | 2028 | Both | 227296.62 (54969.66 to 399623.58) | 612.48 (148.13 to 1076.82) |
| Ghana | 2029 | Both | 222834.48 (19087.59 to 426581.37) | 590.92 (50.62 to 1131.22) |
| Ghana | 2030 | Both | 218503.66 (0.00 to 454474.89) | 570.42 (0.00 to 1186.44) |
| Guinea | 2022 | Male | 51420.07 (45791.32 to 57048.82) | 787.73 (701.77 to 873.69) |
| Guinea | 2023 | Male | 50934.86 (38706.74 to 63162.97) | 760.61 (578.13 to 943.10) |
| Guinea | 2024 | Male | 50449.07 (30301.39 to 70596.76) | 734.80 (441.41 to 1028.18) |
| Guinea | 2025 | Male | 49976.13 (20843.41 to 79108.84) | 710.21 (296.25 to 1124.16) |
| Guinea | 2026 | Male | 49522.71 (10502.17 to 88543.24) | 686.76 (145.67 to 1227.85) |
| Guinea | 2027 | Male | 49077.98 (0.00 to 98761.88) | 664.42 (0.00 to 1337.01) |
| Guinea | 2028 | Male | 48644.68 (0.00 to 109675.66) | 643.12 (0.00 to 1449.98) |
| Guinea | 2029 | Male | 48216.13 (0.00 to 121193.69) | 622.81 (0.00 to 1565.45) |
| Guinea | 2030 | Male | 47791.92 (0.00 to 133249.11) | 603.42 (0.00 to 1682.38) |
| Guinea | 2022 | Female | 44750.64 (39920.72 to 49580.56) | 647.47 (577.85 to 717.09) |
| Guinea | 2023 | Female | 44086.15 (33620.98 to 54551.32) | 622.65 (474.96 to 770.34) |
| Guinea | 2024 | Female | 43445.25 (26274.34 to 60616.16) | 599.28 (362.50 to 836.07) |
| Guinea | 2025 | Female | 42840.53 (18115.41 to 67565.65) | 577.31 (244.17 to 910.46) |
| Guinea | 2026 | Female | 42276.88 (9287.96 to 75265.80) | 556.65 (122.32 to 990.97) |
| Guinea | 2027 | Female | 41744.52 (0.00 to 83602.74) | 537.22 (0.00 to 1075.87) |
| Guinea | 2028 | Female | 41246.51 (0.00 to 92510.15) | 518.97 (0.00 to 1163.96) |
| Guinea | 2029 | Female | 40779.00 (0.00 to 101927.03) | 501.86 (0.00 to 1254.37) |
| Guinea | 2030 | Female | 40343.30 (0.00 to 111817.14) | 485.82 (0.00 to 1346.51) |
| Guinea | 2022 | Both | 95893.42 (85576.17 to 106210.66) | 713.53 (636.89 to 790.17) |
| Guinea | 2023 | Both | 94688.74 (72177.07 to 117200.41) | 687.30 (523.96 to 850.64) |
| Guinea | 2024 | Both | 93507.07 (56440.78 to 130573.36) | 662.45 (399.89 to 925.02) |
| Guinea | 2025 | Both | 92373.03 (38865.51 to 145880.55) | 638.93 (268.85 to 1009.01) |
| Guinea | 2026 | Both | 91299.70 (19767.42 to 162831.98) | 616.64 (133.53 to 1099.75) |
| Guinea | 2027 | Both | 90268.39 (0.00 to 181176.86) | 595.55 (0.00 to 1195.31) |
| Guinea | 2028 | Both | 89284.52 (0.00 to 200757.97) | 575.60 (0.00 to 1294.24) |
| Guinea | 2029 | Both | 88335.60 (0.00 to 221417.56) | 556.71 (0.00 to 1395.43) |
| Guinea | 2030 | Both | 87420.91 (0.00 to 243045.76) | 538.83 (0.00 to 1498.02) |
| Guinea-Bissau | 2022 | Male | 13801.24 (9470.55 to 18131.93) | 1339.94 (920.07 to 1759.80) |
| Guinea-Bissau | 2023 | Male | 13574.06 (4121.44 to 23026.68) | 1286.49 (390.87 to 2182.11) |
| Guinea-Bissau | 2024 | Male | 13347.06 (0.00 to 28863.08) | 1235.70 (0.00 to 2672.05) |
| Guinea-Bissau | 2025 | Male | 13122.45 (0.00 to 35426.80) | 1187.47 (0.00 to 3205.72) |
| Guinea-Bissau | 2026 | Male | 12900.00 (0.00 to 42566.84) | 1141.71 (0.00 to 3767.28) |
| Guinea-Bissau | 2027 | Male | 12678.69 (0.00 to 50166.25) | 1098.25 (0.00 to 4345.44) |
| Guinea-Bissau | 2028 | Male | 12458.49 (0.00 to 58133.28) | 1056.95 (0.00 to 4931.86) |
| Guinea-Bissau | 2029 | Male | 12239.87 (0.00 to 66394.54) | 1017.69 (0.00 to 5520.38) |
| Guinea-Bissau | 2030 | Male | 12025.91 (0.00 to 74905.41) | 980.41 (0.00 to 6106.63) |
| Guinea-Bissau | 2022 | Female | 11963.69 (8533.87 to 15393.50) | 1108.93 (791.63 to 1426.22) |
| Guinea-Bissau | 2023 | Female | 11724.38 (4267.89 to 19180.87) | 1061.84 (386.80 to 1736.87) |
| Guinea-Bissau | 2024 | Female | 11488.51 (0.00 to 23684.63) | 1017.27 (0.00 to 2097.03) |
| Guinea-Bissau | 2025 | Female | 11257.54 (0.00 to 28730.46) | 975.11 (0.00 to 2488.48) |
| Guinea-Bissau | 2026 | Female | 11030.78 (0.00 to 34195.48) | 935.21 (0.00 to 2899.07) |
| Guinea-Bissau | 2027 | Female | 10807.48 (0.00 to 39986.53) | 897.43 (0.00 to 3320.35) |
| Guinea-Bissau | 2028 | Female | 10587.85 (0.00 to 46032.29) | 861.67 (0.00 to 3746.20) |
| Guinea-Bissau | 2029 | Female | 10372.28 (0.00 to 52276.53) | 827.81 (0.00 to 4172.14) |
| Guinea-Bissau | 2030 | Female | 10163.06 (0.00 to 58684.77) | 795.77 (0.00 to 4595.01) |
| Guinea-Bissau | 2022 | Both | 25597.34 (17913.26 to 33281.42) | 1213.81 (849.74 to 1577.88) |
| Guinea-Bissau | 2023 | Both | 25096.94 (8325.05 to 41868.83) | 1162.28 (385.68 to 1938.88) |
| Guinea-Bissau | 2024 | Both | 24603.04 (0.00 to 52076.84) | 1113.52 (0.00 to 2356.90) |
| Guinea-Bissau | 2025 | Both | 24119.74 (0.00 to 63518.90) | 1067.45 (0.00 to 2811.06) |
| Guinea-Bissau | 2026 | Both | 23646.50 (0.00 to 75923.55) | 1023.93 (0.00 to 3287.57) |
| Guinea-Bissau | 2027 | Both | 23181.48 (0.00 to 89083.59) | 982.80 (0.00 to 3776.77) |
| Guinea-Bissau | 2028 | Both | 22724.64 (0.00 to 102839.42) | 943.92 (0.00 to 4271.64) |
| Guinea-Bissau | 2029 | Both | 22276.56 (0.00 to 117065.07) | 907.14 (0.00 to 4767.08) |
| Guinea-Bissau | 2030 | Both | 21842.46 (0.00 to 131686.18) | 872.39 (0.00 to 5259.54) |
| Guyana | 2022 | Male | 66540.10 (64004.20 to 69076.00) | 17582.03 (16925.41 to 18238.64) |
| Guyana | 2023 | Male | 63706.17 (58830.35 to 68581.99) | 16734.74 (15460.54 to 18008.94) |
| Guyana | 2024 | Male | 60977.06 (53430.04 to 68524.08) | 15928.13 (13960.79 to 17895.46) |
| Guyana | 2025 | Male | 58355.43 (47958.52 to 68752.34) | 15160.37 (12462.12 to 17858.61) |
| Guyana | 2026 | Male | 55836.33 (42500.86 to 69171.79) | 14429.61 (10985.45 to 17873.78) |
| Guyana | 2027 | Male | 53405.97 (37107.75 to 69704.19) | 13733.74 (9544.15 to 17923.33) |
| Guyana | 2028 | Male | 51066.25 (31827.91 to 70304.60) | 13071.20 (8148.15 to 17994.24) |
| Guyana | 2029 | Male | 48810.25 (26692.48 to 70928.02) | 12440.71 (6804.43 to 18076.98) |
| Guyana | 2030 | Male | 46641.18 (21730.41 to 71551.95) | 11840.80 (5517.61 to 18163.99) |
| Guyana | 2022 | Female | 51073.58 (48927.10 to 53220.07) | 13102.17 (12563.37 to 13640.96) |
| Guyana | 2023 | Female | 48542.98 (44352.66 to 52733.31) | 12370.40 (11308.25 to 13432.55) |
| Guyana | 2024 | Female | 46130.70 (39641.77 to 52619.64) | 11680.35 (10040.81 to 13319.90) |
| Guyana | 2025 | Female | 43835.08 (34925.17 to 52744.99) | 11029.64 (8790.14 to 13269.15) |
| Guyana | 2026 | Female | 41650.93 (30276.30 to 53025.56) | 10416.31 (7573.43 to 13259.18) |
| Guyana | 2027 | Female | 39565.56 (25738.02 to 53393.10) | 9837.95 (6401.11 to 13274.80) |
| Guyana | 2028 | Female | 37576.73 (21347.43 to 53806.04) | 9292.33 (5280.09 to 13304.56) |
| Guyana | 2029 | Female | 35676.38 (17127.67 to 54225.10) | 8777.54 (4214.87 to 13340.21) |
| Guyana | 2030 | Female | 33864.66 (13098.93 to 54630.38) | 8291.84 (3208.07 to 13375.60) |
| Guyana | 2022 | Both | 117333.42 (113015.45 to 121651.39) | 15272.51 (14717.30 to 15827.72) |
| Guyana | 2023 | Both | 111933.97 (103305.71 to 120562.23) | 14478.69 (13365.85 to 15591.53) |
| Guyana | 2024 | Both | 106761.40 (93265.23 to 120257.57) | 13726.62 (11993.33 to 15459.91) |
| Guyana | 2025 | Both | 101815.77 (83159.06 to 120472.47) | 13014.08 (10630.72 to 15397.44) |
| Guyana | 2026 | Both | 97087.65 (73140.74 to 121034.56) | 12339.26 (9296.74 to 15381.78) |
| Guyana | 2027 | Both | 92551.24 (63301.50 to 121800.98) | 11699.95 (8003.09 to 15396.82) |
| Guyana | 2028 | Both | 88205.53 (53724.90 to 122686.16) | 11094.17 (6757.94 to 15430.40) |
| Guyana | 2029 | Both | 84034.84 (44463.45 to 123606.24) | 10520.21 (5566.83 to 15473.60) |
| Guyana | 2030 | Both | 80041.87 (35565.14 to 124518.60) | 9976.40 (4433.26 to 15519.55) |
| Haiti | 2022 | Male | 69377.06 (59288.01 to 79466.10) | 1121.42 (958.56 to 1284.29) |
| Haiti | 2023 | Male | 67325.01 (45996.31 to 88653.71) | 1072.71 (732.97 to 1412.45) |
| Haiti | 2024 | Male | 65321.39 (30962.99 to 99679.78) | 1026.55 (486.65 to 1566.45) |
| Haiti | 2025 | Male | 63381.07 (14764.29 to 111997.86) | 982.81 (228.98 to 1736.63) |
| Haiti | 2026 | Male | 61491.59 (0.00 to 125212.09) | 941.31 (0.00 to 1916.71) |
| Haiti | 2027 | Male | 59644.70 (0.00 to 139026.58) | 901.92 (0.00 to 2102.28) |
| Haiti | 2028 | Male | 57855.68 (0.00 to 153260.84) | 864.55 (0.00 to 2290.20) |
| Haiti | 2029 | Male | 56116.58 (0.00 to 167730.23) | 829.08 (0.00 to 2478.07) |
| Haiti | 2030 | Male | 54417.48 (0.00 to 182266.80) | 795.37 (0.00 to 2664.02) |
| Haiti | 2022 | Female | 62529.40 (53833.15 to 71225.66) | 951.61 (819.47 to 1083.74) |
| Haiti | 2023 | Female | 60531.93 (41860.82 to 79203.04) | 909.15 (628.82 to 1189.49) |
| Haiti | 2024 | Female | 58605.10 (28430.90 to 88779.30) | 869.16 (421.71 to 1316.61) |
| Haiti | 2025 | Female | 56754.47 (14030.08 to 99478.86) | 831.43 (205.57 to 1457.30) |
| Haiti | 2026 | Female | 54966.22 (0.00 to 110952.23) | 795.76 (0.00 to 1606.25) |
| Haiti | 2027 | Female | 53236.07 (0.00 to 122950.40) | 762.04 (0.00 to 1759.93) |
| Haiti | 2028 | Female | 51572.94 (0.00 to 135313.14) | 730.20 (0.00 to 1915.82) |
| Haiti | 2029 | Female | 49970.87 (0.00 to 147890.58) | 700.14 (0.00 to 2072.08) |
| Haiti | 2030 | Female | 48424.24 (0.00 to 160558.54) | 671.73 (0.00 to 2227.24) |
| Haiti | 2022 | Both | 131970.71 (113355.57 to 150585.84) | 1034.46 (888.65 to 1180.27) |
| Haiti | 2023 | Both | 127971.48 (88163.90 to 167779.05) | 989.40 (681.68 to 1297.12) |
| Haiti | 2024 | Both | 124085.97 (59763.54 to 188408.39) | 946.79 (456.03 to 1437.55) |
| Haiti | 2025 | Both | 120332.95 (29205.63 to 211460.28) | 906.46 (220.02 to 1592.89) |
| Haiti | 2026 | Both | 116688.46 (0.00 to 236194.73) | 868.22 (0.00 to 1757.39) |
| Haiti | 2027 | Both | 113140.77 (0.00 to 262068.48) | 831.97 (0.00 to 1927.09) |
| Haiti | 2028 | Both | 109713.96 (0.00 to 288739.53) | 797.64 (0.00 to 2099.18) |
| Haiti | 2029 | Both | 106393.12 (0.00 to 315868.57) | 765.10 (0.00 to 2271.48) |
| Haiti | 2030 | Both | 103161.29 (0.00 to 343155.15) | 734.21 (0.00 to 2442.28) |
| India | 2022 | Male | 17848641.39 (16295793.72 to 19401489.05) | 2402.03 (2193.06 to 2611.01) |
| India | 2023 | Male | 17065603.30 (13843926.02 to 20287280.58) | 2277.33 (1847.41 to 2707.25) |
| India | 2024 | Male | 16316432.92 (11206916.29 to 21425949.56) | 2159.89 (1483.51 to 2836.26) |
| India | 2025 | Male | 15598888.96 (8478028.76 to 22719749.17) | 2049.32 (1113.81 to 2984.83) |
| India | 2026 | Male | 14909078.23 (5716539.59 to 24101616.88) | 1944.86 (745.71 to 3144.01) |
| India | 2027 | Male | 14246269.36 (2966033.87 to 25526504.85) | 1846.01 (384.34 to 3307.69) |
| India | 2028 | Male | 13611062.10 (258987.41 to 26963136.80) | 1752.59 (33.35 to 3471.83) |
| India | 2029 | Male | 13004585.79 (0.00 to 28390582.26) | 1664.53 (0.00 to 3633.86) |
| India | 2030 | Male | 12425296.63 (0.00 to 29789182.93) | 1581.56 (0.00 to 3791.74) |
| India | 2022 | Female | 13992881.10 (12898784.25 to 15086977.95) | 1986.48 (1831.16 to 2141.79) |
| India | 2023 | Female | 13421819.32 (11093081.64 to 15750557.00) | 1889.67 (1561.81 to 2217.53) |
| India | 2024 | Female | 12874084.12 (9144637.97 to 16603530.27) | 1798.22 (1277.30 to 2319.14) |
| India | 2025 | Female | 12347892.34 (7117267.98 to 17578516.69) | 1711.84 (986.70 to 2436.99) |
| India | 2026 | Female | 11841082.72 (5053820.10 to 18628345.34) | 1630.06 (695.71 to 2564.40) |
| India | 2027 | Female | 11353484.02 (2986052.85 to 19720915.18) | 1552.52 (408.32 to 2696.71) |
| India | 2028 | Female | 10884849.51 (937629.00 to 20832070.02) | 1479.00 (127.40 to 2830.60) |
| India | 2029 | Female | 10435920.23 (0.00 to 21945664.90) | 1409.46 (0.00 to 2963.94) |
| India | 2030 | Female | 10005670.63 (0.00 to 23046749.51) | 1343.72 (0.00 to 3095.08) |
| India | 2022 | Both | 31770008.92 (29239610.88 to 34300406.97) | 2194.86 (2020.05 to 2369.68) |
| India | 2023 | Both | 30410055.94 (25093261.23 to 35726850.64) | 2083.39 (1719.14 to 2447.64) |
| India | 2024 | Both | 29107998.40 (20637369.00 to 37578627.80) | 1978.30 (1402.60 to 2554.00) |
| India | 2025 | Both | 27859528.91 (16021776.01 to 39697281.81) | 1879.23 (1080.73 to 2677.74) |
| India | 2026 | Both | 26658651.28 (11344285.86 to 41973016.70) | 1785.57 (759.83 to 2811.30) |
| India | 2027 | Both | 25504641.33 (6677290.85 to 44331991.81) | 1696.88 (444.26 to 2949.51) |
| India | 2028 | Both | 24398228.58 (2074564.63 to 46721892.52) | 1613.01 (137.15 to 3088.87) |
| India | 2029 | Both | 23340840.59 (0.00 to 49106699.33) | 1533.87 (0.00 to 3227.09) |
| India | 2030 | Both | 22329558.62 (0.00 to 51452523.18) | 1459.20 (0.00 to 3362.34) |
| Indonesia | 2022 | Male | 2060178.47 (1956521.48 to 2163835.46) | 1517.90 (1441.55 to 1594.24) |
| Indonesia | 2023 | Male | 1975373.54 (1770227.33 to 2180519.76) | 1444.09 (1294.13 to 1594.05) |
| Indonesia | 2024 | Male | 1893952.76 (1572717.08 to 2215188.44) | 1374.13 (1141.07 to 1607.19) |
| Indonesia | 2025 | Male | 1815753.69 (1370176.38 to 2261331.00) | 1307.86 (986.92 to 1628.80) |
| Indonesia | 2026 | Male | 1740662.93 (1166303.24 to 2315022.63) | 1245.11 (834.27 to 1655.95) |
| Indonesia | 2027 | Male | 1668540.87 (963606.85 to 2373474.89) | 1185.60 (684.70 to 1686.49) |
| Indonesia | 2028 | Male | 1599267.11 (763953.80 to 2434580.42) | 1129.14 (539.38 to 1718.90) |
| Indonesia | 2029 | Male | 1532752.06 (568751.53 to 2496752.59) | 1075.58 (399.11 to 1752.05) |
| Indonesia | 2030 | Male | 1468838.92 (379039.80 to 2558638.05) | 1024.80 (264.45 to 1785.14) |
| Indonesia | 2022 | Female | 1702852.42 (1611948.84 to 1793756.00) | 1271.62 (1203.77 to 1339.48) |
| Indonesia | 2023 | Female | 1634815.02 (1464128.55 to 1805501.50) | 1210.38 (1084.02 to 1336.74) |
| Indonesia | 2024 | Female | 1569457.26 (1306559.55 to 1832354.96) | 1152.30 (959.29 to 1345.32) |
| Indonesia | 2025 | Female | 1506664.25 (1144664.79 to 1868663.70) | 1097.28 (833.65 to 1360.92) |
| Indonesia | 2026 | Female | 1446388.25 (981490.15 to 1911286.35) | 1045.20 (709.25 to 1381.14) |
| Indonesia | 2027 | Female | 1388502.68 (819007.79 to 1957997.57) | 995.82 (587.39 to 1404.25) |
| Indonesia | 2028 | Female | 1332888.71 (658666.39 to 2007111.03) | 948.97 (468.95 to 1428.99) |
| Indonesia | 2029 | Female | 1279482.10 (501569.23 to 2057394.98) | 904.53 (354.59 to 1454.48) |
| Indonesia | 2030 | Female | 1228167.06 (348526.33 to 2107807.78) | 862.42 (244.74 to 1480.09) |
| Indonesia | 2022 | Both | 3760029.41 (3566758.42 to 3953300.40) | 1394.48 (1322.81 to 1466.14) |
| Indonesia | 2023 | Both | 3607097.66 (3234134.26 to 3980061.06) | 1326.84 (1189.66 to 1464.03) |
| Indonesia | 2024 | Both | 3460259.65 (2880720.40 to 4039798.91) | 1262.73 (1051.24 to 1474.21) |
| Indonesia | 2025 | Both | 3319238.60 (2518103.70 to 4120373.51) | 1202.00 (911.89 to 1492.12) |
| Indonesia | 2026 | Both | 3183887.48 (2152987.66 to 4214787.31) | 1144.52 (773.94 to 1515.10) |
| Indonesia | 2027 | Both | 3053934.32 (1789804.21 to 4318064.43) | 1090.04 (638.84 to 1541.24) |
| Indonesia | 2028 | Both | 2929129.47 (1431823.56 to 4426435.38) | 1038.36 (507.57 to 1569.15) |
| Indonesia | 2029 | Both | 2809313.74 (1081522.81 to 4537104.68) | 989.34 (380.88 to 1597.81) |
| Indonesia | 2030 | Both | 2694214.74 (740725.40 to 4647704.09) | 942.89 (259.23 to 1626.55) |
| Kenya | 2022 | Male | 295197.74 (252140.51 to 338254.96) | 1106.24 (944.94 to 1267.55) |
| Kenya | 2023 | Male | 287130.54 (194687.45 to 379573.62) | 1055.86 (715.94 to 1395.78) |
| Kenya | 2024 | Male | 279378.64 (129445.56 to 429311.72) | 1008.43 (467.25 to 1549.61) |
| Kenya | 2025 | Male | 271896.72 (58694.26 to 485099.19) | 963.71 (208.04 to 1719.38) |
| Kenya | 2026 | Male | 264651.81 (0.00 to 545322.91) | 921.48 (0.00 to 1898.72) |
| Kenya | 2027 | Male | 257655.02 (0.00 to 608874.67) | 881.62 (0.00 to 2083.39) |
| Kenya | 2028 | Male | 250912.88 (0.00 to 674923.84) | 844.07 (0.00 to 2270.44) |
| Kenya | 2029 | Male | 244404.57 (0.00 to 742764.17) | 808.65 (0.00 to 2457.56) |
| Kenya | 2030 | Male | 238082.13 (0.00 to 811715.50) | 775.18 (0.00 to 2642.88) |
| Kenya | 2022 | Female | 239576.25 (204026.46 to 275126.04) | 892.29 (759.93 to 1024.64) |
| Kenya | 2023 | Female | 233528.23 (157450.85 to 309605.60) | 853.00 (575.13 to 1130.86) |
| Kenya | 2024 | Female | 227781.35 (104284.49 to 351278.21) | 816.19 (373.69 to 1258.70) |
| Kenya | 2025 | Female | 222296.58 (46332.51 to 398260.66) | 781.67 (162.93 to 1400.41) |
| Kenya | 2026 | Female | 217033.99 (0.00 to 449284.96) | 749.20 (0.00 to 1550.92) |
| Kenya | 2027 | Female | 211995.95 (0.00 to 503499.89) | 718.67 (0.00 to 1706.88) |
| Kenya | 2028 | Female | 207190.55 (0.00 to 560298.56) | 690.04 (0.00 to 1866.05) |
| Kenya | 2029 | Female | 202606.72 (0.00 to 619189.03) | 663.18 (0.00 to 2026.75) |
| Kenya | 2030 | Female | 198204.69 (0.00 to 679687.08) | 637.93 (0.00 to 2187.61) |
| Kenya | 2022 | Both | 533388.06 (454754.85 to 612021.27) | 996.35 (849.49 to 1143.21) |
| Kenya | 2023 | Both | 519522.64 (350693.27 to 688352.01) | 952.01 (642.64 to 1261.37) |
| Kenya | 2024 | Both | 506275.76 (232129.33 to 780422.19) | 910.37 (417.41 to 1403.33) |
| Kenya | 2025 | Both | 493554.63 (103125.52 to 883983.73) | 871.20 (182.04 to 1560.37) |
| Kenya | 2026 | Both | 481273.98 (0.00 to 996137.27) | 834.25 (0.00 to 1726.73) |
| Kenya | 2027 | Both | 469452.02 (0.00 to 1114928.67) | 799.43 (0.00 to 1898.61) |
| Kenya | 2028 | Both | 458112.25 (0.00 to 1238940.67) | 766.68 (0.00 to 2073.45) |
| Kenya | 2029 | Both | 447226.31 (0.00 to 1366989.13) | 735.88 (0.00 to 2249.28) |
| Kenya | 2030 | Both | 436699.62 (0.00 to 1497892.10) | 706.83 (0.00 to 2424.43) |
| Kiribati | 2022 | Male | 985.20 (770.32 to 1200.08) | 1518.58 (1201.23 to 1835.93) |
| Kiribati | 2023 | Male | 960.53 (554.07 to 1366.99) | 1449.48 (843.01 to 2055.96) |
| Kiribati | 2024 | Male | 936.25 (303.69 to 1568.82) | 1383.86 (453.09 to 2314.64) |
| Kiribati | 2025 | Male | 912.40 (30.76 to 1794.05) | 1321.61 (47.43 to 2595.78) |
| Kiribati | 2026 | Male | 889.02 (0.00 to 2036.05) | 1262.55 (0.00 to 2889.41) |
| Kiribati | 2027 | Male | 866.11 (0.00 to 2290.15) | 1206.50 (0.00 to 3188.56) |
| Kiribati | 2028 | Male | 843.73 (0.00 to 2552.89) | 1153.27 (0.00 to 3488.19) |
| Kiribati | 2029 | Male | 821.91 (0.00 to 2821.60) | 1102.74 (0.00 to 3784.62) |
| Kiribati | 2030 | Male | 800.68 (0.00 to 3094.13) | 1054.80 (0.00 to 4075.26) |
| Kiribati | 2022 | Female | 848.93 (662.53 to 1035.32) | 1269.16 (1003.89 to 1534.43) |
| Kiribati | 2023 | Female | 823.93 (476.96 to 1170.90) | 1206.98 (705.42 to 1708.54) |
| Kiribati | 2024 | Female | 799.61 (264.12 to 1335.10) | 1148.24 (383.40 to 1913.07) |
| Kiribati | 2025 | Female | 775.95 (34.19 to 1517.71) | 1092.75 (50.99 to 2134.52) |
| Kiribati | 2026 | Female | 752.97 (0.00 to 1712.95) | 1040.36 (0.00 to 2364.66) |
| Kiribati | 2027 | Female | 730.66 (0.00 to 1916.85) | 990.85 (0.00 to 2597.85) |
| Kiribati | 2028 | Female | 709.04 (0.00 to 2126.49) | 944.06 (0.00 to 2830.06) |
| Kiribati | 2029 | Female | 688.11 (0.00 to 2339.62) | 899.81 (0.00 to 3058.36) |
| Kiribati | 2030 | Female | 667.88 (0.00 to 2554.45) | 857.99 (0.00 to 3280.69) |
| Kiribati | 2022 | Both | 1822.87 (1467.46 to 2178.27) | 1383.42 (1121.28 to 1645.57) |
| Kiribati | 2023 | Both | 1773.87 (1070.97 to 2476.78) | 1318.57 (799.70 to 1837.44) |
| Kiribati | 2024 | Both | 1726.02 (615.85 to 2836.18) | 1257.18 (450.74 to 2063.61) |
| Kiribati | 2025 | Both | 1679.28 (121.65 to 3236.91) | 1199.09 (88.34 to 2309.83) |
| Kiribati | 2026 | Both | 1633.70 (0.00 to 3667.19) | 1144.12 (0.00 to 2567.16) |
| Kiribati | 2027 | Both | 1589.29 (0.00 to 4118.67) | 1092.09 (0.00 to 2829.33) |
| Kiribati | 2028 | Both | 1546.11 (0.00 to 4585.11) | 1042.80 (0.00 to 3091.85) |
| Kiribati | 2029 | Both | 1504.19 (0.00 to 5061.62) | 996.11 (0.00 to 3351.39) |
| Kiribati | 2030 | Both | 1463.52 (0.00 to 5544.32) | 951.88 (0.00 to 3605.60) |
| Lao People's Democratic Republic | 2022 | Male | 20659.71 (16985.41 to 24334.01) | 549.63 (452.17 to 647.09) |
| Lao People's Democratic Republic | 2023 | Male | 20298.82 (12422.07 to 28175.57) | 533.28 (326.47 to 740.08) |
| Lao People's Democratic Republic | 2024 | Male | 19940.65 (7088.26 to 32793.03) | 517.59 (184.07 to 851.12) |
| Lao People's Democratic Republic | 2025 | Male | 19586.16 (1173.17 to 37999.14) | 502.54 (30.15 to 974.92) |
| Lao People's Democratic Republic | 2026 | Male | 19236.19 (0.00 to 43669.26) | 488.08 (0.00 to 1107.98) |
| Lao People's Democratic Republic | 2027 | Male | 18888.66 (0.00 to 49704.63) | 474.18 (0.00 to 1247.77) |
| Lao People's Democratic Republic | 2028 | Male | 18543.92 (0.00 to 56030.33) | 460.82 (0.00 to 1392.35) |
| Lao People's Democratic Republic | 2029 | Male | 18201.55 (0.00 to 62582.60) | 447.95 (0.00 to 1540.19) |
| Lao People's Democratic Republic | 2030 | Male | 17863.01 (0.00 to 69313.75) | 435.54 (0.00 to 1690.02) |
| Lao People's Democratic Republic | 2022 | Female | 16091.68 (12963.25 to 19220.10) | 430.42 (347.01 to 513.83) |
| Lao People's Democratic Republic | 2023 | Female | 15748.03 (9019.33 to 22476.73) | 415.70 (238.20 to 593.20) |
| Lao People's Democratic Republic | 2024 | Female | 15414.29 (4450.81 to 26377.78) | 401.77 (116.08 to 687.47) |
| Lao People's Democratic Republic | 2025 | Female | 15091.21 (0.00 to 30763.82) | 388.59 (0.00 to 792.10) |
| Lao People's Democratic Republic | 2026 | Female | 14779.37 (0.00 to 35530.07) | 376.10 (0.00 to 904.13) |
| Lao People's Democratic Republic | 2027 | Female | 14476.84 (0.00 to 40595.45) | 364.29 (0.00 to 1021.50) |
| Lao People's Democratic Republic | 2028 | Female | 14183.56 (0.00 to 45900.49) | 353.09 (0.00 to 1142.64) |
| Lao People's Democratic Republic | 2029 | Female | 13899.01 (0.00 to 51396.87) | 342.47 (0.00 to 1266.39) |
| Lao People's Democratic Republic | 2030 | Female | 13624.27 (0.00 to 57051.49) | 332.39 (0.00 to 1391.87) |
| Lao People's Democratic Republic | 2022 | Both | 36934.89 (30057.86 to 43811.91) | 492.63 (401.05 to 584.22) |
| Lao People's Democratic Republic | 2023 | Both | 36257.89 (21454.64 to 51061.13) | 477.41 (282.56 to 672.26) |
| Lao People's Democratic Republic | 2024 | Both | 35591.79 (11418.29 to 59765.29) | 462.88 (148.54 to 777.23) |
| Lao People's Democratic Republic | 2025 | Both | 34937.87 (300.66 to 69575.08) | 449.01 (3.89 to 894.14) |
| Lao People's Democratic Republic | 2026 | Both | 34297.35 (0.00 to 80258.03) | 435.75 (0.00 to 1019.67) |
| Lao People's Democratic Republic | 2027 | Both | 33666.62 (0.00 to 91632.41) | 423.09 (0.00 to 1151.52) |
| Lao People's Democratic Republic | 2028 | Both | 33046.47 (0.00 to 103562.64) | 410.97 (0.00 to 1287.91) |
| Lao People's Democratic Republic | 2029 | Both | 32436.03 (0.00 to 115934.66) | 399.37 (0.00 to 1427.45) |
| Lao People's Democratic Republic | 2030 | Both | 31837.41 (0.00 to 128663.58) | 388.25 (0.00 to 1569.02) |
| Liberia | 2022 | Male | 338840.14 (263994.91 to 413685.38) | 12666.39 (9868.88 to 15463.90) |
| Liberia | 2023 | Male | 327516.56 (166972.16 to 488060.95) | 11972.04 (6103.64 to 17840.44) |
| Liberia | 2024 | Male | 316365.78 (57453.82 to 575277.74) | 11315.29 (2055.00 to 20575.58) |
| Liberia | 2025 | Male | 305500.65 (0.00 to 671089.03) | 10694.29 (0.00 to 23491.95) |
| Liberia | 2026 | Male | 294896.71 (0.00 to 772408.22) | 10107.15 (0.00 to 26473.10) |
| Liberia | 2027 | Male | 284409.57 (0.00 to 876526.68) | 9551.64 (0.00 to 29437.34) |
| Liberia | 2028 | Male | 274078.07 (0.00 to 981612.21) | 9026.14 (0.00 to 32327.14) |
| Liberia | 2029 | Male | 263976.45 (0.00 to 1086392.42) | 8529.16 (0.00 to 35101.66) |
| Liberia | 2030 | Male | 254144.02 (0.00 to 1189851.13) | 8059.29 (0.00 to 37731.93) |
| Liberia | 2022 | Female | 246486.57 (196525.59 to 296447.54) | 9337.00 (7444.82 to 11229.18) |
| Liberia | 2023 | Female | 238049.17 (130356.72 to 345741.63) | 8822.67 (4831.49 to 12813.84) |
| Liberia | 2024 | Female | 229794.29 (55941.63 to 403646.96) | 8337.61 (2029.82 to 14645.40) |
| Liberia | 2025 | Female | 221795.24 (0.00 to 467356.44) | 7880.07 (0.00 to 16604.45) |
| Liberia | 2026 | Female | 214026.13 (0.00 to 534811.49) | 7448.31 (0.00 to 18611.89) |
| Liberia | 2027 | Female | 206380.63 (0.00 to 604216.12) | 7040.63 (0.00 to 20612.67) |
| Liberia | 2028 | Female | 198882.44 (0.00 to 674369.49) | 6655.71 (0.00 to 22568.13) |
| Liberia | 2029 | Female | 191585.72 (0.00 to 744467.12) | 6292.50 (0.00 to 24451.49) |
| Liberia | 2030 | Female | 184515.64 (0.00 to 813866.19) | 5949.74 (0.00 to 26243.23) |
| Liberia | 2022 | Both | 587894.78 (463161.39 to 712628.17) | 11061.04 (8714.40 to 13407.69) |
| Liberia | 2023 | Both | 568139.48 (299494.09 to 836784.86) | 10455.59 (5511.73 to 15399.46) |
| Liberia | 2024 | Both | 548730.10 (114996.82 to 982463.37) | 9883.41 (2071.30 to 17695.52) |
| Liberia | 2025 | Both | 529855.58 (0.00 to 1142627.05) | 9342.75 (0.00 to 20147.49) |
| Liberia | 2026 | Both | 511460.13 (0.00 to 1312106.32) | 8831.69 (0.00 to 22656.91) |
| Liberia | 2027 | Both | 493291.01 (0.00 to 1486370.69) | 8348.30 (0.00 to 25154.85) |
| Liberia | 2028 | Both | 475415.11 (0.00 to 1662377.18) | 7891.18 (0.00 to 27592.96) |
| Liberia | 2029 | Both | 457962.86 (0.00 to 1838036.83) | 7459.10 (0.00 to 29937.13) |
| Liberia | 2030 | Both | 440999.57 (0.00 to 2011676.02) | 7050.72 (0.00 to 32162.77) |
| Madagascar | 2022 | Male | 164508.92 (140496.99 to 188520.84) | 1077.95 (920.70 to 1235.20) |
| Madagascar | 2023 | Male | 162211.93 (110315.07 to 214108.79) | 1030.78 (701.04 to 1360.52) |
| Madagascar | 2024 | Male | 159976.57 (74842.71 to 245110.44) | 986.12 (461.37 to 1510.88) |
| Madagascar | 2025 | Male | 157787.85 (35226.35 to 280349.34) | 943.87 (210.73 to 1677.00) |
| Madagascar | 2026 | Male | 155649.35 (0.00 to 319064.99) | 903.92 (0.00 to 1852.94) |
| Madagascar | 2027 | Male | 153559.24 (0.00 to 360697.63) | 866.09 (0.00 to 2034.36) |
| Madagascar | 2028 | Male | 151512.33 (0.00 to 404802.37) | 830.21 (0.00 to 2218.12) |
| Madagascar | 2029 | Male | 149503.79 (0.00 to 451015.87) | 796.17 (0.00 to 2401.84) |
| Madagascar | 2030 | Male | 147521.51 (0.00 to 499005.87) | 763.85 (0.00 to 2583.78) |
| Madagascar | 2022 | Female | 137926.61 (118606.39 to 157246.83) | 909.43 (782.13 to 1036.73) |
| Madagascar | 2023 | Female | 135588.79 (93439.81 to 177737.78) | 867.55 (597.91 to 1137.20) |
| Madagascar | 2024 | Female | 133345.95 (64141.91 to 202549.99) | 828.16 (398.38 to 1257.93) |
| Madagascar | 2025 | Female | 131175.88 (31631.84 to 230719.92) | 791.06 (190.77 to 1391.34) |
| Madagascar | 2026 | Female | 129060.22 (0.00 to 261580.18) | 756.03 (0.00 to 1532.32) |
| Madagascar | 2027 | Female | 127011.12 (0.00 to 294689.20) | 722.98 (0.00 to 1677.45) |
| Madagascar | 2028 | Female | 125035.01 (0.00 to 329713.27) | 691.84 (0.00 to 1824.35) |
| Madagascar | 2029 | Female | 123131.33 (0.00 to 366383.60) | 662.48 (0.00 to 1971.25) |
| Madagascar | 2030 | Female | 121282.50 (0.00 to 404431.02) | 634.77 (0.00 to 2116.73) |
| Madagascar | 2022 | Both | 302121.33 (259029.60 to 345213.05) | 992.92 (851.35 to 1134.50) |
| Madagascar | 2023 | Both | 297576.10 (203772.12 to 391380.08) | 948.73 (649.68 to 1247.78) |
| Madagascar | 2024 | Both | 293179.68 (139071.87 to 447287.50) | 907.00 (430.25 to 1383.74) |
| Madagascar | 2025 | Both | 288895.24 (66978.16 to 510812.32) | 867.57 (201.15 to 1533.99) |
| Madagascar | 2026 | Both | 284715.67 (0.00 to 580544.79) | 830.32 (0.00 to 1693.04) |
| Madagascar | 2027 | Both | 280649.66 (0.00 to 655485.53) | 795.09 (0.00 to 1857.01) |
| Madagascar | 2028 | Both | 276694.76 (0.00 to 734850.02) | 761.77 (0.00 to 2023.12) |
| Madagascar | 2029 | Both | 272843.29 (0.00 to 817998.61) | 730.23 (0.00 to 2189.25) |
| Madagascar | 2030 | Both | 269064.39 (0.00 to 904321.58) | 700.34 (0.00 to 2353.82) |
| Malawi | 2022 | Male | 46251.98 (40900.25 to 51603.71) | 483.44 (427.67 to 539.20) |
| Malawi | 2023 | Male | 46345.31 (35056.40 to 57634.22) | 471.74 (356.91 to 586.57) |
| Malawi | 2024 | Male | 46455.62 (27870.48 to 65040.77) | 460.79 (276.49 to 645.09) |
| Malawi | 2025 | Male | 46594.10 (19555.10 to 73633.11) | 450.55 (189.13 to 711.98) |
| Malawi | 2026 | Male | 46754.14 (10217.59 to 83290.70) | 440.96 (96.39 to 785.52) |
| Malawi | 2027 | Male | 46921.73 (0.00 to 93915.76) | 431.96 (0.00 to 864.56) |
| Malawi | 2028 | Male | 47102.97 (0.00 to 105464.93) | 423.56 (0.00 to 948.35) |
| Malawi | 2029 | Male | 47292.85 (0.00 to 117888.22) | 415.73 (0.00 to 1036.30) |
| Malawi | 2030 | Male | 47503.10 (0.00 to 131187.34) | 408.44 (0.00 to 1127.95) |
| Malawi | 2022 | Female | 37741.76 (33576.01 to 41907.52) | 371.75 (330.89 to 412.61) |
| Malawi | 2023 | Female | 37551.38 (28550.18 to 46552.58) | 360.26 (273.98 to 446.54) |
| Malawi | 2024 | Female | 37415.18 (22540.91 to 52289.45) | 349.84 (210.81 to 488.88) |
| Malawi | 2025 | Female | 37340.95 (15705.08 to 58976.81) | 340.42 (143.21 to 537.64) |
| Malawi | 2026 | Female | 37320.96 (8118.10 to 66523.81) | 331.89 (72.22 to 591.57) |
| Malawi | 2027 | Female | 37349.40 (0.00 to 74876.23) | 324.23 (0.00 to 649.98) |
| Malawi | 2028 | Female | 37435.74 (0.00 to 84031.85) | 317.45 (0.00 to 712.56) |
| Malawi | 2029 | Female | 37579.12 (0.00 to 93989.09) | 311.52 (0.00 to 779.12) |
| Malawi | 2030 | Female | 37788.06 (0.00 to 104785.07) | 306.38 (0.00 to 849.57) |
| Malawi | 2022 | Both | 83685.78 (74508.43 to 92863.12) | 424.37 (377.92 to 470.82) |
| Malawi | 2023 | Both | 83620.85 (63807.03 to 103434.67) | 412.99 (315.17 to 510.81) |
| Malawi | 2024 | Both | 83626.54 (50812.99 to 116440.08) | 402.50 (244.59 to 560.42) |
| Malawi | 2025 | Both | 83721.27 (35876.97 to 131565.57) | 392.86 (168.37 to 617.36) |
| Malawi | 2026 | Both | 83891.68 (19175.98 to 148607.39) | 383.98 (87.78 to 680.18) |
| Malawi | 2027 | Both | 84118.77 (818.92 to 167418.61) | 375.83 (3.67 to 748.00) |
| Malawi | 2028 | Both | 84417.93 (0.00 to 187956.17) | 368.42 (0.00 to 820.28) |
| Malawi | 2029 | Both | 84782.15 (0.00 to 210167.96) | 361.71 (0.00 to 896.65) |
| Malawi | 2030 | Both | 85230.83 (0.00 to 234090.92) | 355.66 (0.00 to 976.83) |
| Malaysia | 2022 | Male | 180302.87 (160323.26 to 200282.48) | 1068.70 (950.38 to 1187.02) |
| Malaysia | 2023 | Male | 175000.70 (132158.66 to 217842.74) | 1024.32 (773.60 to 1275.04) |
| Malaysia | 2024 | Male | 169850.80 (100519.80 to 239181.80) | 981.96 (581.16 to 1382.75) |
| Malaysia | 2025 | Male | 164845.09 (66516.43 to 263173.76) | 941.49 (379.92 to 1503.06) |
| Malaysia | 2026 | Male | 159976.39 (30904.66 to 289048.13) | 902.86 (174.43 to 1631.28) |
| Malaysia | 2027 | Male | 155234.28 (0.00 to 316226.39) | 866.00 (0.00 to 1764.11) |
| Malaysia | 2028 | Male | 150610.18 (0.00 to 344253.40) | 830.79 (0.00 to 1898.94) |
| Malaysia | 2029 | Male | 146092.76 (0.00 to 372749.34) | 797.10 (0.00 to 2033.77) |
| Malaysia | 2030 | Male | 141668.72 (0.00 to 401383.58) | 764.86 (0.00 to 2167.03) |
| Malaysia | 2022 | Female | 142746.91 (127566.44 to 157927.39) | 898.59 (803.14 to 994.04) |
| Malaysia | 2023 | Female | 138394.62 (105758.41 to 171030.83) | 859.07 (656.53 to 1061.60) |
| Malaysia | 2024 | Female | 134181.69 (81364.36 to 186999.02) | 821.52 (498.18 to 1144.86) |
| Malaysia | 2025 | Female | 130102.24 (55226.69 to 204977.79) | 785.83 (333.60 to 1238.07) |
| Malaysia | 2026 | Female | 126149.24 (27918.73 to 224379.74) | 751.94 (166.43 to 1337.46) |
| Malaysia | 2027 | Female | 122312.01 (0.00 to 244763.09) | 719.76 (0.00 to 1440.33) |
| Malaysia | 2028 | Female | 118584.54 (0.00 to 265787.68) | 689.17 (0.00 to 1544.64) |
| Malaysia | 2029 | Female | 114959.31 (0.00 to 287175.24) | 660.06 (0.00 to 1648.86) |
| Malaysia | 2030 | Female | 111427.39 (0.00 to 308688.83) | 632.36 (0.00 to 1751.82) |
| Malaysia | 2022 | Both | 323394.45 (288344.16 to 358444.75) | 987.25 (880.31 to 1094.20) |
| Malaysia | 2023 | Both | 313793.78 (238418.87 to 389168.69) | 945.32 (718.27 to 1172.37) |
| Malaysia | 2024 | Both | 304479.15 (182417.16 to 426541.14) | 905.36 (542.43 to 1268.30) |
| Malaysia | 2025 | Both | 295434.32 (122282.33 to 468586.32) | 867.27 (358.98 to 1375.56) |
| Malaysia | 2026 | Both | 286646.46 (59344.46 to 513948.45) | 830.97 (172.04 to 1489.90) |
| Malaysia | 2027 | Both | 278098.41 (0.00 to 561613.46) | 796.41 (0.00 to 1608.33) |
| Malaysia | 2028 | Both | 269774.87 (0.00 to 610784.64) | 763.47 (0.00 to 1728.52) |
| Malaysia | 2029 | Both | 261655.84 (0.00 to 660800.39) | 732.02 (0.00 to 1848.67) |
| Malaysia | 2030 | Both | 253716.96 (0.00 to 711085.06) | 701.98 (0.00 to 1967.41) |
| Maldives | 2022 | Male | 1257.58 (358.68 to 2156.48) | 434.31 (124.80 to 743.81) |
| Maldives | 2023 | Male | 1248.71 (0.00 to 3224.48) | 425.77 (0.00 to 1099.03) |
| Maldives | 2024 | Male | 1240.70 (0.00 to 4514.52) | 417.90 (0.00 to 1520.36) |
| Maldives | 2025 | Male | 1233.39 (0.00 to 5988.97) | 410.61 (0.00 to 1993.63) |
| Maldives | 2026 | Male | 1226.75 (0.00 to 7623.96) | 403.85 (0.00 to 2509.74) |
| Maldives | 2027 | Male | 1220.73 (0.00 to 9403.03) | 397.57 (0.00 to 3062.33) |
| Maldives | 2028 | Male | 1215.36 (0.00 to 11315.00) | 391.71 (0.00 to 3646.75) |
| Maldives | 2029 | Male | 1210.54 (0.00 to 13350.23) | 386.23 (0.00 to 4259.42) |
| Maldives | 2030 | Male | 1206.25 (0.00 to 15502.06) | 381.08 (0.00 to 4897.36) |
| Maldives | 2022 | Female | 677.16 (195.60 to 1158.71) | 325.02 (95.18 to 554.86) |
| Maldives | 2023 | Female | 671.67 (0.00 to 1719.25) | 316.59 (0.00 to 809.78) |
| Maldives | 2024 | Female | 666.65 (0.00 to 2395.72) | 308.82 (0.00 to 1109.45) |
| Maldives | 2025 | Female | 662.02 (0.00 to 3167.56) | 301.64 (0.00 to 1443.01) |
| Maldives | 2026 | Female | 657.76 (0.00 to 4021.92) | 294.99 (0.00 to 1803.56) |
| Maldives | 2027 | Female | 653.89 (0.00 to 4950.21) | 288.82 (0.00 to 2186.37) |
| Maldives | 2028 | Female | 650.45 (0.00 to 5946.82) | 283.09 (0.00 to 2588.07) |
| Maldives | 2029 | Female | 647.40 (0.00 to 7006.97) | 277.76 (0.00 to 3006.16) |
| Maldives | 2030 | Female | 644.75 (0.00 to 8127.97) | 272.78 (0.00 to 3438.68) |
| Maldives | 2022 | Both | 1965.13 (545.54 to 3384.72) | 394.68 (110.10 to 679.26) |
| Maldives | 2023 | Both | 1954.38 (0.00 to 5088.42) | 386.67 (0.00 to 1006.49) |
| Maldives | 2024 | Both | 1944.64 (0.00 to 7149.26) | 379.25 (0.00 to 1394.13) |
| Maldives | 2025 | Both | 1935.59 (0.00 to 9508.00) | 372.33 (0.00 to 1828.88) |
| Maldives | 2026 | Both | 1927.04 (0.00 to 12125.63) | 365.84 (0.00 to 2301.95) |
| Maldives | 2027 | Both | 1919.02 (0.00 to 14975.13) | 359.74 (0.00 to 2807.20) |
| Maldives | 2028 | Both | 1911.70 (0.00 to 18038.20) | 353.99 (0.00 to 3340.13) |
| Maldives | 2029 | Both | 1904.91 (0.00 to 21298.57) | 348.56 (0.00 to 3897.21) |
| Maldives | 2030 | Both | 1898.64 (0.00 to 24744.03) | 343.40 (0.00 to 4475.29) |
| Mali | 2022 | Male | 186940.91 (141489.06 to 232392.77) | 1574.44 (1191.70 to 1957.17) |
| Mali | 2023 | Male | 184571.80 (85127.06 to 284016.54) | 1503.55 (693.49 to 2313.61) |
| Mali | 2024 | Male | 182314.62 (18389.51 to 346239.72) | 1436.53 (144.91 to 2728.14) |
| Mali | 2025 | Male | 180153.69 (0.00 to 416957.42) | 1373.09 (0.00 to 3177.94) |
| Mali | 2026 | Male | 178071.80 (0.00 to 494748.41) | 1312.98 (0.00 to 3647.93) |
| Mali | 2027 | Male | 176046.63 (0.00 to 578540.61) | 1256.04 (0.00 to 4127.70) |
| Mali | 2028 | Male | 174080.88 (0.00 to 667555.86) | 1202.14 (0.00 to 4609.92) |
| Mali | 2029 | Male | 172168.86 (0.00 to 761150.72) | 1151.12 (0.00 to 5089.05) |
| Mali | 2030 | Male | 170300.45 (0.00 to 858765.29) | 1102.73 (0.00 to 5560.70) |
| Mali | 2022 | Female | 161246.82 (125242.97 to 197250.68) | 1348.22 (1047.26 to 1649.19) |
| Mali | 2023 | Female | 159070.07 (79964.97 to 238175.18) | 1287.50 (647.26 to 1927.74) |
| Mali | 2024 | Female | 157008.16 (26515.59 to 287500.72) | 1230.19 (207.77 to 2252.61) |
| Mali | 2025 | Female | 155043.92 (0.00 to 343559.99) | 1176.00 (0.00 to 2605.88) |
| Mali | 2026 | Female | 153153.18 (0.00 to 405198.50) | 1124.65 (0.00 to 2975.49) |
| Mali | 2027 | Female | 151319.79 (0.00 to 471563.98) | 1076.02 (0.00 to 3353.23) |
| Mali | 2028 | Female | 149544.48 (0.00 to 542031.57) | 1030.00 (0.00 to 3733.30) |
| Mali | 2029 | Female | 147821.53 (0.00 to 616085.80) | 986.45 (0.00 to 4111.27) |
| Mali | 2030 | Female | 146138.51 (0.00 to 693264.01) | 945.12 (0.00 to 4483.53) |
| Mali | 2022 | Both | 348466.42 (266814.38 to 430118.46) | 1462.09 (1119.53 to 1804.65) |
| Mali | 2023 | Both | 344019.88 (164717.01 to 523322.76) | 1396.71 (668.76 to 2124.66) |
| Mali | 2024 | Both | 339798.79 (43920.33 to 635677.26) | 1334.94 (172.55 to 2497.32) |
| Mali | 2025 | Both | 335763.02 (0.00 to 763402.88) | 1276.46 (0.00 to 2902.19) |
| Mali | 2026 | Both | 331867.65 (0.00 to 903904.63) | 1220.99 (0.00 to 3325.59) |
| Mali | 2027 | Both | 328078.79 (0.00 to 1055256.20) | 1168.41 (0.00 to 3758.17) |
| Mali | 2028 | Both | 324408.53 (0.00 to 1216075.83) | 1118.66 (0.00 to 4193.41) |
| Mali | 2029 | Both | 320845.38 (0.00 to 1385206.74) | 1071.56 (0.00 to 4626.31) |
| Mali | 2030 | Both | 317356.88 (0.00 to 1561580.19) | 1026.85 (0.00 to 5052.69) |
| Marshall Islands | 2022 | Male | 37.58 (17.93 to 57.22) | 121.75 (71.40 to 172.10) |
| Marshall Islands | 2023 | Male | 38.33 (11.57 to 65.09) | 122.64 (46.33 to 198.95) |
| Marshall Islands | 2024 | Male | 39.12 (2.94 to 75.30) | 123.64 (16.05 to 231.24) |
| Marshall Islands | 2025 | Male | 39.96 (0.00 to 87.56) | 124.79 (0.00 to 268.29) |
| Marshall Islands | 2026 | Male | 40.86 (0.00 to 101.72) | 126.08 (0.00 to 309.84) |
| Marshall Islands | 2027 | Male | 41.82 (0.00 to 117.75) | 127.54 (0.00 to 355.84) |
| Marshall Islands | 2028 | Male | 42.85 (0.00 to 135.67) | 129.18 (0.00 to 406.33) |
| Marshall Islands | 2029 | Male | 43.94 (0.00 to 155.56) | 131.00 (0.00 to 461.44) |
| Marshall Islands | 2030 | Male | 45.12 (0.00 to 177.51) | 133.01 (0.00 to 521.39) |
| Marshall Islands | 2022 | Female | 51.16 (29.90 to 72.42) | 172.77 (118.79 to 226.75) |
| Marshall Islands | 2023 | Female | 52.07 (24.87 to 79.27) | 173.45 (96.04 to 250.85) |
| Marshall Islands | 2024 | Female | 53.03 (17.95 to 88.11) | 174.29 (68.98 to 279.60) |
| Marshall Islands | 2025 | Female | 54.05 (9.41 to 98.70) | 175.32 (38.26 to 312.39) |
| Marshall Islands | 2026 | Female | 55.14 (0.00 to 110.90) | 176.54 (4.18 to 348.91) |
| Marshall Islands | 2027 | Female | 56.29 (0.00 to 124.65) | 177.95 (0.00 to 389.05) |
| Marshall Islands | 2028 | Female | 57.50 (0.00 to 139.94) | 179.56 (0.00 to 432.79) |
| Marshall Islands | 2029 | Female | 58.79 (0.00 to 156.80) | 181.37 (0.00 to 480.16) |
| Marshall Islands | 2030 | Female | 60.15 (0.00 to 175.26) | 183.39 (0.00 to 531.26) |
| Marshall Islands | 2022 | Both | 89.04 (56.73 to 121.35) | 147.23 (103.42 to 191.04) |
| Marshall Islands | 2023 | Both | 90.11 (44.09 to 136.13) | 147.06 (78.37 to 215.76) |
| Marshall Islands | 2024 | Both | 91.30 (27.37 to 155.22) | 147.10 (48.62 to 245.58) |
| Marshall Islands | 2025 | Both | 92.61 (7.29 to 177.93) | 147.34 (14.96 to 279.72) |
| Marshall Islands | 2026 | Both | 94.06 (0.00 to 203.95) | 147.79 (0.00 to 317.87) |
| Marshall Islands | 2027 | Both | 95.63 (0.00 to 233.16) | 148.45 (0.00 to 359.86) |
| Marshall Islands | 2028 | Both | 97.35 (0.00 to 265.58) | 149.32 (0.00 to 405.67) |
| Marshall Islands | 2029 | Both | 99.21 (0.00 to 301.29) | 150.41 (0.00 to 455.34) |
| Marshall Islands | 2030 | Both | 101.22 (0.00 to 340.41) | 151.70 (0.00 to 508.98) |
| Micronesia (Federated States of) | 2022 | Male | 1306.22 (1049.08 to 1563.36) | 2309.32 (1872.30 to 2746.34) |
| Micronesia (Federated States of) | 2023 | Male | 1251.63 (766.31 to 1736.96) | 2183.56 (1345.57 to 3021.56) |
| Micronesia (Federated States of) | 2024 | Male | 1199.37 (452.52 to 1946.22) | 2064.75 (784.35 to 3345.16) |
| Micronesia (Federated States of) | 2025 | Male | 1149.33 (122.65 to 2176.01) | 1952.55 (212.02 to 3693.08) |
| Micronesia (Federated States of) | 2026 | Male | 1101.41 (0.00 to 2417.57) | 1846.63 (0.00 to 4050.61) |
| Micronesia (Federated States of) | 2027 | Male | 1055.46 (0.00 to 2664.75) | 1746.59 (0.00 to 4407.58) |
| Micronesia (Federated States of) | 2028 | Male | 1011.34 (0.00 to 2912.94) | 1652.06 (0.00 to 4756.75) |
| Micronesia (Federated States of) | 2029 | Male | 968.94 (0.00 to 3158.62) | 1562.74 (0.00 to 5092.97) |
| Micronesia (Federated States of) | 2030 | Male | 928.20 (0.00 to 3399.10) | 1478.36 (0.00 to 5412.69) |
| Micronesia (Federated States of) | 2022 | Female | 1081.56 (862.47 to 1300.65) | 1980.11 (1596.75 to 2363.46) |
| Micronesia (Federated States of) | 2023 | Female | 1036.98 (629.47 to 1444.49) | 1872.04 (1145.25 to 2598.83) |
| Micronesia (Federated States of) | 2024 | Female | 994.23 (370.20 to 1618.27) | 1769.86 (664.46 to 2875.26) |
| Micronesia (Federated States of) | 2025 | Female | 953.24 (97.29 to 1809.20) | 1673.29 (174.55 to 3172.03) |
| Micronesia (Federated States of) | 2026 | Female | 913.94 (0.00 to 2009.96) | 1582.06 (0.00 to 3476.52) |
| Micronesia (Federated States of) | 2027 | Female | 876.19 (0.00 to 2215.44) | 1495.85 (0.00 to 3780.09) |
| Micronesia (Federated States of) | 2028 | Female | 839.89 (0.00 to 2421.77) | 1414.36 (0.00 to 4076.49) |
| Micronesia (Federated States of) | 2029 | Female | 804.95 (0.00 to 2626.00) | 1337.31 (0.00 to 4361.32) |
| Micronesia (Federated States of) | 2030 | Female | 771.30 (0.00 to 2825.83) | 1264.49 (0.00 to 4631.53) |
| Micronesia (Federated States of) | 2022 | Both | 2373.97 (1948.96 to 2798.97) | 2135.16 (1762.69 to 2507.64) |
| Micronesia (Federated States of) | 2023 | Both | 2273.58 (1439.14 to 3108.02) | 2017.13 (1281.47 to 2752.79) |
| Micronesia (Federated States of) | 2024 | Both | 2177.50 (876.28 to 3478.71) | 1905.68 (769.71 to 3041.64) |
| Micronesia (Federated States of) | 2025 | Both | 2085.53 (285.45 to 3885.60) | 1800.49 (248.36 to 3352.61) |
| Micronesia (Federated States of) | 2026 | Both | 1997.49 (0.00 to 4313.32) | 1701.24 (0.00 to 3672.20) |
| Micronesia (Federated States of) | 2027 | Both | 1913.08 (0.00 to 4750.86) | 1607.57 (0.00 to 3991.08) |
| Micronesia (Federated States of) | 2028 | Both | 1832.03 (0.00 to 5189.89) | 1519.10 (0.00 to 4302.53) |
| Micronesia (Federated States of) | 2029 | Both | 1754.13 (0.00 to 5623.99) | 1435.53 (0.00 to 4601.79) |
| Micronesia (Federated States of) | 2030 | Both | 1679.26 (0.00 to 6048.27) | 1356.62 (0.00 to 4885.60) |
| Mozambique | 2022 | Male | 168983.87 (149076.86 to 188890.88) | 1043.94 (921.06 to 1166.82) |
| Mozambique | 2023 | Male | 166152.71 (123798.87 to 208506.55) | 1004.03 (748.14 to 1259.93) |
| Mozambique | 2024 | Male | 163424.54 (94402.58 to 232446.49) | 966.40 (558.27 to 1374.53) |
| Mozambique | 2025 | Male | 160809.84 (61866.25 to 259753.42) | 930.84 (358.13 to 1503.56) |
| Mozambique | 2026 | Male | 158281.38 (26796.75 to 289766.00) | 897.16 (151.90 to 1642.41) |
| Mozambique | 2027 | Male | 155813.72 (0.00 to 321986.39) | 865.32 (0.00 to 1788.16) |
| Mozambique | 2028 | Male | 153424.30 (0.00 to 356097.86) | 835.36 (0.00 to 1938.87) |
| Mozambique | 2029 | Male | 151113.12 (0.00 to 391831.21) | 807.20 (0.00 to 2093.02) |
| Mozambique | 2030 | Male | 148887.85 (0.00 to 428994.60) | 780.63 (0.00 to 2249.26) |
| Mozambique | 2022 | Female | 153715.18 (137076.43 to 170353.93) | 875.90 (781.19 to 970.60) |
| Mozambique | 2023 | Female | 150397.88 (114481.08 to 186314.68) | 838.74 (638.48 to 1039.00) |
| Mozambique | 2024 | Female | 147271.66 (88658.60 to 205884.72) | 804.12 (484.11 to 1124.12) |
| Mozambique | 2025 | Female | 144326.40 (60408.45 to 228244.36) | 771.73 (323.03 to 1220.43) |
| Mozambique | 2026 | Female | 141521.89 (30237.34 to 252806.44) | 741.29 (158.40 to 1324.19) |
| Mozambique | 2027 | Female | 138853.06 (0.00 to 279187.21) | 712.86 (0.00 to 1433.31) |
| Mozambique | 2028 | Female | 136342.57 (0.00 to 307162.35) | 686.45 (0.00 to 1546.48) |
| Mozambique | 2029 | Female | 133984.00 (0.00 to 336531.49) | 661.95 (0.00 to 1662.63) |
| Mozambique | 2030 | Female | 131769.22 (0.00 to 367139.58) | 639.10 (0.00 to 1780.69) |
| Mozambique | 2022 | Both | 321762.60 (285794.89 to 357730.32) | 953.75 (847.19 to 1060.31) |
| Mozambique | 2023 | Both | 315527.16 (238060.71 to 392993.60) | 915.10 (690.46 to 1139.75) |
| Mozambique | 2024 | Both | 309588.62 (183080.71 to 436096.53) | 878.88 (519.75 to 1238.01) |
| Mozambique | 2025 | Both | 303949.08 (122606.95 to 485291.21) | 844.83 (340.80 to 1348.87) |
| Mozambique | 2026 | Both | 298543.73 (57747.70 to 539339.77) | 812.72 (157.21 to 1468.23) |
| Mozambique | 2027 | Both | 293339.66 (0.00 to 597360.38) | 782.56 (0.00 to 1593.60) |
| Mozambique | 2028 | Both | 288376.77 (0.00 to 658815.03) | 754.36 (0.00 to 1723.38) |
| Mozambique | 2029 | Both | 283647.19 (0.00 to 723233.76) | 728.02 (0.00 to 1856.27) |
| Mozambique | 2030 | Both | 279149.73 (0.00 to 790269.42) | 703.32 (0.00 to 1991.08) |
| Myanmar | 2022 | Male | 446311.02 (359492.40 to 533129.64) | 1676.52 (1350.44 to 2002.61) |
| Myanmar | 2023 | Male | 428865.75 (244178.49 to 613553.00) | 1596.53 (909.01 to 2284.04) |
| Myanmar | 2024 | Male | 412224.01 (116045.30 to 708402.72) | 1520.94 (428.17 to 2613.71) |
| Myanmar | 2025 | Male | 396284.64 (0.00 to 812488.95) | 1449.39 (0.00 to 2971.63) |
| Myanmar | 2026 | Male | 380967.34 (0.00 to 922268.79) | 1381.51 (0.00 to 3344.43) |
| Myanmar | 2027 | Male | 366264.33 (0.00 to 1035288.22) | 1317.11 (0.00 to 3722.97) |
| Myanmar | 2028 | Male | 352153.93 (0.00 to 1149689.89) | 1256.09 (0.00 to 4100.81) |
| Myanmar | 2029 | Male | 338602.86 (0.00 to 1264007.44) | 1198.28 (0.00 to 4473.20) |
| Myanmar | 2030 | Male | 325559.96 (0.00 to 1376993.00) | 1143.42 (0.00 to 4836.22) |
| Myanmar | 2022 | Female | 412953.56 (340686.45 to 485220.67) | 1443.43 (1190.86 to 1695.99) |
| Myanmar | 2023 | Female | 396771.85 (242203.33 to 551340.37) | 1374.44 (839.02 to 1909.86) |
| Myanmar | 2024 | Female | 381278.07 (133065.06 to 629491.08) | 1309.09 (456.88 to 2161.30) |
| Myanmar | 2025 | Female | 366400.24 (17458.86 to 715341.62) | 1247.13 (59.43 to 2434.83) |
| Myanmar | 2026 | Female | 352102.32 (0.00 to 805956.84) | 1188.36 (0.00 to 2720.14) |
| Myanmar | 2027 | Female | 338375.64 (0.00 to 899261.52) | 1132.63 (0.00 to 3010.05) |
| Myanmar | 2028 | Female | 325181.32 (0.00 to 993626.67) | 1079.75 (0.00 to 3299.30) |
| Myanmar | 2029 | Female | 312484.04 (0.00 to 1087770.05) | 1029.57 (0.00 to 3583.99) |
| Myanmar | 2030 | Female | 300250.48 (0.00 to 1180654.71) | 981.92 (0.00 to 3861.12) |
| Myanmar | 2022 | Both | 858513.85 (699862.87 to 1017164.84) | 1554.42 (1267.19 to 1841.65) |
| Myanmar | 2023 | Both | 824968.75 (486328.55 to 1163608.96) | 1480.29 (872.66 to 2087.92) |
| Myanmar | 2024 | Both | 792914.66 (249335.45 to 1336493.87) | 1410.16 (443.44 to 2376.89) |
| Myanmar | 2025 | Both | 762171.65 (0.00 to 1526316.49) | 1343.72 (0.00 to 2690.92) |
| Myanmar | 2026 | Both | 732611.60 (0.00 to 1726579.50) | 1280.67 (0.00 to 3018.21) |
| Myanmar | 2027 | Both | 704223.46 (0.00 to 1932756.26) | 1220.84 (0.00 to 3350.63) |
| Myanmar | 2028 | Both | 676956.20 (0.00 to 2141378.13) | 1164.12 (0.00 to 3682.39) |
| Myanmar | 2029 | Both | 650746.10 (0.00 to 2349711.37) | 1110.33 (0.00 to 4009.19) |
| Myanmar | 2030 | Both | 625502.42 (0.00 to 2555450.95) | 1059.27 (0.00 to 4327.57) |
| Nepal | 2022 | Male | 388853.83 (374441.66 to 403266.01) | 2576.21 (2481.08 to 2671.35) |
| Nepal | 2023 | Male | 371689.43 (342484.56 to 400894.30) | 2439.81 (2248.26 to 2631.35) |
| Nepal | 2024 | Male | 355216.46 (309263.79 to 401169.13) | 2311.23 (2012.34 to 2610.13) |
| Nepal | 2025 | Male | 339412.55 (275643.12 to 403181.98) | 2190.06 (1778.65 to 2601.46) |
| Nepal | 2026 | Male | 324250.54 (242152.02 to 406349.06) | 2075.83 (1550.29 to 2601.38) |
| Nepal | 2027 | Male | 309686.30 (209140.22 to 410232.38) | 1968.05 (1329.12 to 2606.98) |
| Nepal | 2028 | Male | 295724.33 (176899.04 to 414549.62) | 1866.37 (1116.47 to 2616.27) |
| Nepal | 2029 | Male | 282323.25 (145616.04 to 419030.47) | 1770.43 (913.17 to 2627.69) |
| Nepal | 2030 | Male | 269449.82 (115436.69 to 423462.95) | 1679.89 (719.72 to 2640.07) |
| Nepal | 2022 | Female | 362719.78 (352001.18 to 373438.37) | 2208.94 (2144.06 to 2273.82) |
| Nepal | 2023 | Female | 347451.36 (325389.66 to 369513.07) | 2097.33 (1964.34 to 2230.32) |
| Nepal | 2024 | Female | 332753.67 (297824.40 to 367682.93) | 1991.76 (1782.80 to 2200.73) |
| Nepal | 2025 | Female | 318615.55 (269946.19 to 367284.90) | 1891.97 (1603.04 to 2180.89) |
| Nepal | 2026 | Female | 305010.45 (242149.99 to 367870.92) | 1797.58 (1427.16 to 2167.99) |
| Nepal | 2027 | Female | 291904.17 (214701.76 to 369106.58) | 1708.22 (1256.48 to 2159.97) |
| Nepal | 2028 | Female | 279295.38 (187825.82 to 370764.94) | 1623.62 (1091.92 to 2155.33) |
| Nepal | 2029 | Female | 267157.56 (161673.57 to 372641.56) | 1543.54 (934.12 to 2152.97) |
| Nepal | 2030 | Female | 255471.54 (136363.98 to 374579.09) | 1467.79 (783.49 to 2152.09) |
| Nepal | 2022 | Both | 749422.08 (724972.93 to 773871.23) | 2378.02 (2300.63 to 2455.41) |
| Nepal | 2023 | Both | 716916.90 (666452.35 to 767381.45) | 2254.40 (2095.80 to 2413.01) |
| Nepal | 2024 | Both | 685691.51 (605788.44 to 765594.58) | 2137.73 (1888.68 to 2386.79) |
| Nepal | 2025 | Both | 655703.37 (544436.76 to 766969.98) | 2027.63 (1683.60 to 2371.67) |
| Nepal | 2026 | Both | 626902.75 (483315.82 to 770489.68) | 1923.72 (1483.13 to 2364.30) |
| Nepal | 2027 | Both | 599212.53 (423038.26 to 775386.79) | 1825.54 (1288.83 to 2362.24) |
| Nepal | 2028 | Both | 572640.83 (364119.23 to 781162.43) | 1732.81 (1101.84 to 2363.79) |
| Nepal | 2029 | Both | 547112.16 (306888.55 to 787335.76) | 1645.22 (922.86 to 2367.58) |
| Nepal | 2030 | Both | 522566.60 (251603.78 to 793529.42) | 1562.47 (752.30 to 2372.64) |
| Niger | 2022 | Male | 201695.25 (186133.59 to 217256.90) | 1550.55 (1431.11 to 1669.99) |
| Niger | 2023 | Male | 200843.48 (167352.85 to 234334.10) | 1481.31 (1234.38 to 1728.23) |
| Niger | 2024 | Male | 200087.10 (144831.15 to 255343.06) | 1415.62 (1024.73 to 1806.51) |
| Niger | 2025 | Male | 199424.28 (119211.70 to 279636.85) | 1353.40 (809.07 to 1897.73) |
| Niger | 2026 | Male | 198848.75 (90880.93 to 306816.56) | 1294.47 (591.64 to 1997.30) |
| Niger | 2027 | Male | 198330.68 (60091.83 to 336569.52) | 1238.53 (375.28 to 2101.79) |
| Niger | 2028 | Male | 197857.49 (27047.63 to 368667.34) | 1185.42 (162.06 to 2208.77) |
| Niger | 2029 | Male | 197420.23 (0.00 to 402930.30) | 1134.97 (0.00 to 2316.45) |
| Niger | 2030 | Male | 197018.21 (0.00 to 439225.18) | 1087.12 (0.00 to 2423.58) |
| Niger | 2022 | Female | 173245.06 (161737.86 to 184752.27) | 1323.39 (1235.71 to 1411.07) |
| Niger | 2023 | Female | 172069.78 (146965.00 to 197174.55) | 1262.70 (1078.57 to 1446.83) |
| Niger | 2024 | Female | 171015.45 (129490.57 to 212540.34) | 1205.39 (912.76 to 1498.01) |
| Niger | 2025 | Female | 170070.93 (109781.64 to 230360.22) | 1151.27 (743.19 to 1559.35) |
| Niger | 2026 | Female | 169214.55 (88124.00 to 250305.10) | 1100.08 (572.93 to 1627.23) |
| Niger | 2027 | Female | 168432.84 (64718.99 to 272146.69) | 1051.62 (404.10 to 1699.14) |
| Niger | 2028 | Female | 167723.69 (39721.56 to 295725.82) | 1005.79 (238.21 to 1773.36) |
| Niger | 2029 | Female | 167083.57 (13247.53 to 320919.61) | 962.45 (76.32 to 1848.58) |
| Niger | 2030 | Female | 166505.67 (0.00 to 347625.02) | 921.49 (0.00 to 1923.84) |
| Niger | 2022 | Both | 374790.98 (348375.23 to 401206.72) | 1436.04 (1334.93 to 1537.15) |
| Niger | 2023 | Both | 372861.70 (315100.81 to 430622.60) | 1371.54 (1159.11 to 1583.96) |
| Niger | 2024 | Both | 371146.01 (275436.02 to 466856.01) | 1310.46 (972.55 to 1648.37) |
| Niger | 2025 | Both | 369631.29 (230452.07 to 508810.51) | 1252.67 (781.01 to 1724.32) |
| Niger | 2026 | Both | 368293.10 (180812.41 to 555773.79) | 1197.96 (588.15 to 1807.77) |
| Niger | 2027 | Both | 367085.73 (126963.53 to 607207.93) | 1146.07 (396.40 to 1895.75) |
| Niger | 2028 | Both | 365992.99 (69258.32 to 662727.65) | 1096.88 (207.57 to 1986.18) |
| Niger | 2029 | Both | 365001.33 (7970.96 to 722031.70) | 1050.23 (22.94 to 2077.52) |
| Niger | 2030 | Both | 364102.96 (0.00 to 784884.87) | 1006.03 (0.00 to 2168.66) |
| Nigeria | 2022 | Male | 1793845.30 (1371708.39 to 2215982.20) | 1534.90 (1173.71 to 1896.09) |
| Nigeria | 2023 | Male | 1769261.31 (859136.01 to 2679386.62) | 1466.09 (711.92 to 2220.26) |
| Nigeria | 2024 | Male | 1745452.62 (252898.89 to 3238006.34) | 1400.88 (202.98 to 2598.79) |
| Nigeria | 2025 | Male | 1722258.73 (0.00 to 3871275.74) | 1339.03 (0.00 to 3009.86) |
| Nigeria | 2026 | Male | 1699519.51 (0.00 to 4565489.87) | 1280.32 (0.00 to 3439.36) |
| Nigeria | 2027 | Male | 1677180.20 (0.00 to 5310671.00) | 1224.62 (0.00 to 3877.67) |
| Nigeria | 2028 | Male | 1655307.41 (0.00 to 6099394.53) | 1171.87 (0.00 to 4318.05) |
| Nigeria | 2029 | Male | 1633849.41 (0.00 to 6925381.90) | 1121.91 (0.00 to 4755.43) |
| Nigeria | 2030 | Male | 1612667.86 (0.00 to 7782905.14) | 1074.53 (0.00 to 5185.82) |
| Nigeria | 2022 | Female | 1623184.50 (1203995.87 to 2042373.13) | 1316.93 (976.84 to 1657.02) |
| Nigeria | 2023 | Female | 1602253.21 (717235.70 to 2487270.72) | 1262.00 (564.93 to 1959.08) |
| Nigeria | 2024 | Female | 1581890.85 (138788.76 to 3024992.93) | 1209.67 (106.13 to 2313.21) |
| Nigeria | 2025 | Female | 1561956.38 (0.00 to 3634783.13) | 1159.76 (0.00 to 2698.85) |
| Nigeria | 2026 | Female | 1542312.88 (0.00 to 4303371.77) | 1112.12 (0.00 to 3103.05) |
| Nigeria | 2027 | Female | 1522895.52 (0.00 to 5021094.58) | 1066.67 (0.00 to 3516.90) |
| Nigeria | 2028 | Female | 1503742.72 (0.00 to 5780659.89) | 1023.38 (0.00 to 3934.04) |
| Nigeria | 2029 | Female | 1484807.13 (0.00 to 6575888.77) | 982.12 (0.00 to 4349.61) |
| Nigeria | 2030 | Female | 1465971.91 (0.00 to 7401137.73) | 942.78 (0.00 to 4759.74) |
| Nigeria | 2022 | Both | 3403701.38 (2561973.72 to 4245429.04) | 1417.46 (1066.93 to 1768.00) |
| Nigeria | 2023 | Both | 3357045.68 (1562617.73 to 5151473.62) | 1355.62 (631.01 to 2080.23) |
| Nigeria | 2024 | Both | 3311882.42 (378832.66 to 6244932.17) | 1296.91 (148.35 to 2445.48) |
| Nigeria | 2025 | Both | 3267886.90 (0.00 to 7484326.83) | 1241.13 (0.00 to 2842.53) |
| Nigeria | 2026 | Both | 3224750.50 (0.00 to 8842663.88) | 1188.08 (0.00 to 3257.87) |
| Nigeria | 2027 | Both | 3182363.82 (0.00 to 10300392.91) | 1137.67 (0.00 to 3682.32) |
| Nigeria | 2028 | Both | 3140848.94 (0.00 to 11842977.51) | 1089.84 (0.00 to 4109.39) |
| Nigeria | 2029 | Both | 3100103.17 (0.00 to 13458123.52) | 1044.46 (0.00 to 4534.19) |
| Nigeria | 2030 | Both | 3059850.01 (0.00 to 15134557.05) | 1001.34 (0.00 to 4952.81) |
| Papua New Guinea | 2022 | Male | 497433.53 (465638.51 to 529228.54) | 9319.62 (8724.49 to 9914.75) |
| Papua New Guinea | 2023 | Male | 478364.34 (412359.81 to 544368.87) | 8773.67 (7563.33 to 9984.00) |
| Papua New Guinea | 2024 | Male | 459806.57 (354754.21 to 564858.92) | 8259.15 (6372.32 to 10145.97) |
| Papua New Guinea | 2025 | Male | 441725.56 (294742.35 to 588708.77) | 7774.46 (5187.63 to 10361.29) |
| Papua New Guinea | 2026 | Male | 424166.93 (233641.49 to 614692.36) | 7318.57 (4031.32 to 10605.82) |
| Papua New Guinea | 2027 | Male | 407158.64 (172386.52 to 641930.76) | 6889.67 (2917.07 to 10862.27) |
| Papua New Guinea | 2028 | Male | 390709.53 (111675.94 to 669743.11) | 6485.87 (1853.89 to 11117.85) |
| Papua New Guinea | 2029 | Male | 374812.35 (52039.35 to 697585.35) | 6105.59 (847.74 to 11363.43) |
| Papua New Guinea | 2030 | Male | 359444.88 (0.00 to 725001.20) | 5747.59 (0.00 to 11592.87) |
| Papua New Guinea | 2022 | Female | 352097.02 (331817.32 to 372376.73) | 6979.59 (6578.25 to 7380.93) |
| Papua New Guinea | 2023 | Female | 341643.49 (298320.20 to 384966.79) | 6615.15 (5776.59 to 7453.72) |
| Papua New Guinea | 2024 | Female | 331360.11 (261375.15 to 401345.07) | 6270.29 (4946.15 to 7594.44) |
| Papua New Guinea | 2025 | Female | 321213.72 (222129.29 to 420298.16) | 5943.87 (4110.48 to 7777.25) |
| Papua New Guinea | 2026 | Female | 311233.05 (181418.69 to 441047.41) | 5635.16 (3284.84 to 7985.49) |
| Papua New Guinea | 2027 | Female | 301460.34 (139870.19 to 463050.50) | 5343.29 (2479.22 to 8207.36) |
| Papua New Guinea | 2028 | Female | 291909.37 (97956.45 to 485862.30) | 5067.07 (1700.41 to 8433.72) |
| Papua New Guinea | 2029 | Female | 282580.04 (56047.99 to 509112.08) | 4805.47 (953.18 to 8657.76) |
| Papua New Guinea | 2030 | Female | 273456.65 (14445.81 to 532467.48) | 4557.69 (240.80 to 8874.58) |
| Papua New Guinea | 2022 | Both | 851468.41 (800016.09 to 902920.72) | 8201.27 (7706.00 to 8696.55) |
| Papua New Guinea | 2023 | Both | 821958.37 (713305.53 to 930611.20) | 7742.03 (6718.77 to 8765.29) |
| Papua New Guinea | 2024 | Both | 793096.55 (618807.81 to 967385.28) | 7308.40 (5702.41 to 8914.40) |
| Papua New Guinea | 2025 | Both | 764818.15 (519558.51 to 1010077.79) | 6899.03 (4686.73 to 9111.34) |
| Papua New Guinea | 2026 | Both | 737214.09 (417720.56 to 1056707.61) | 6513.17 (3690.54 to 9335.81) |
| Papua New Guinea | 2027 | Both | 710358.91 (314860.60 to 1105857.22) | 6149.47 (2725.73 to 9573.21) |
| Papua New Guinea | 2028 | Both | 684271.59 (212151.76 to 1156391.41) | 5806.33 (1800.22 to 9812.44) |
| Papua New Guinea | 2029 | Both | 658941.27 (110500.01 to 1207382.54) | 5482.39 (919.38 to 10045.41) |
| Papua New Guinea | 2030 | Both | 634332.25 (10627.72 to 1258036.77) | 5176.65 (86.75 to 10266.54) |
| Philippines | 2022 | Male | 528645.81 (470882.36 to 586409.25) | 929.25 (827.75 to 1030.76) |
| Philippines | 2023 | Male | 513861.08 (389939.30 to 637782.86) | 889.67 (675.13 to 1104.21) |
| Philippines | 2024 | Male | 499510.59 (298712.79 to 700308.39) | 852.14 (509.60 to 1194.68) |
| Philippines | 2025 | Male | 485552.18 (200358.51 to 770745.86) | 816.53 (336.94 to 1296.11) |
| Philippines | 2026 | Male | 471964.73 (97032.55 to 846896.92) | 782.71 (160.92 to 1404.49) |
| Philippines | 2027 | Male | 458763.03 (0.00 to 927201.65) | 750.60 (0.00 to 1517.03) |
| Philippines | 2028 | Male | 445941.61 (0.00 to 1010434.87) | 720.09 (0.00 to 1631.62) |
| Philippines | 2029 | Male | 433488.32 (0.00 to 1095611.79) | 691.09 (0.00 to 1746.67) |
| Philippines | 2030 | Male | 421365.72 (0.00 to 1181859.11) | 663.48 (0.00 to 1860.95) |
| Philippines | 2022 | Female | 419094.95 (377800.19 to 460389.70) | 756.53 (682.03 to 831.04) |
| Philippines | 2023 | Female | 407781.86 (318694.44 to 496869.28) | 724.65 (566.35 to 882.95) |
| Philippines | 2024 | Female | 396865.69 (252146.63 to 541584.75) | 694.53 (441.28 to 947.79) |
| Philippines | 2025 | Female | 386304.49 (180345.39 to 592263.59) | 666.07 (310.96 to 1021.18) |
| Philippines | 2026 | Female | 376091.46 (104791.65 to 647391.27) | 639.16 (178.10 to 1100.23) |
| Philippines | 2027 | Female | 366251.50 (26573.95 to 705929.04) | 613.77 (44.54 to 1183.00) |
| Philippines | 2028 | Female | 356778.21 (0.00 to 767058.95) | 589.79 (0.00 to 1268.02) |
| Philippines | 2029 | Female | 347646.02 (0.00 to 830104.78) | 567.11 (0.00 to 1354.14) |
| Philippines | 2030 | Female | 338818.73 (0.00 to 894481.43) | 545.64 (0.00 to 1440.49) |
| Philippines | 2022 | Both | 945682.32 (847595.95 to 1043768.69) | 842.21 (754.87 to 929.55) |
| Philippines | 2023 | Both | 919598.86 (708221.20 to 1130976.52) | 806.44 (621.08 to 991.80) |
| Philippines | 2024 | Both | 894351.52 (551260.08 to 1237442.96) | 772.59 (476.21 to 1068.97) |
| Philippines | 2025 | Both | 869847.48 (381993.00 to 1357701.97) | 740.53 (325.20 to 1155.85) |
| Philippines | 2026 | Both | 846051.66 (204050.82 to 1488052.51) | 710.13 (171.27 to 1248.99) |
| Philippines | 2027 | Both | 823010.90 (20101.11 to 1625920.69) | 681.35 (16.64 to 1346.05) |
| Philippines | 2028 | Both | 800716.55 (0.00 to 1769282.03) | 654.07 (0.00 to 1445.25) |
| Philippines | 2029 | Both | 779130.73 (0.00 to 1916485.50) | 628.20 (0.00 to 1545.22) |
| Philippines | 2030 | Both | 758172.08 (0.00 to 2066060.62) | 603.62 (0.00 to 1644.90) |
| Samoa | 2022 | Male | 8135.76 (6952.21 to 9319.31) | 7221.65 (6182.86 to 8260.44) |
| Samoa | 2023 | Male | 7821.69 (5383.85 to 10259.54) | 6801.59 (4687.05 to 8916.13) |
| Samoa | 2024 | Male | 7518.45 (3648.30 to 11388.59) | 6405.92 (3111.63 to 9700.20) |
| Samoa | 2025 | Male | 7227.89 (1816.25 to 12639.53) | 6033.27 (1518.21 to 10548.34) |
| Samoa | 2026 | Male | 6949.17 (0.00 to 13967.11) | 5682.13 (0.00 to 11418.93) |
| Samoa | 2027 | Male | 6679.22 (0.00 to 15333.59) | 5351.18 (0.00 to 12283.59) |
| Samoa | 2028 | Male | 6418.61 (0.00 to 16715.36) | 5039.39 (0.00 to 13122.65) |
| Samoa | 2029 | Male | 6166.47 (0.00 to 18091.27) | 4745.77 (0.00 to 13922.42) |
| Samoa | 2030 | Male | 5923.84 (0.00 to 19449.52) | 4469.30 (0.00 to 14673.26) |
| Samoa | 2022 | Female | 5937.19 (5113.95 to 6760.43) | 5609.32 (4844.74 to 6373.90) |
| Samoa | 2023 | Female | 5742.23 (4067.71 to 7416.75) | 5320.54 (3775.11 to 6865.97) |
| Samoa | 2024 | Female | 5552.86 (2894.19 to 8211.52) | 5046.53 (2633.94 to 7459.12) |
| Samoa | 2025 | Female | 5370.70 (1641.26 to 9100.14) | 4786.62 (1465.23 to 8108.01) |
| Samoa | 2026 | Female | 5195.51 (337.56 to 10053.45) | 4540.17 (296.78 to 8783.56) |
| Samoa | 2027 | Female | 5024.97 (0.00 to 11046.27) | 4306.40 (0.00 to 9465.28) |
| Samoa | 2028 | Female | 4859.42 (0.00 to 12062.74) | 4084.60 (0.00 to 10138.28) |
| Samoa | 2029 | Female | 4698.12 (0.00 to 13088.03) | 3874.15 (0.00 to 10791.71) |
| Samoa | 2030 | Female | 4542.07 (0.00 to 14114.39) | 3674.52 (0.00 to 11417.76) |
| Samoa | 2022 | Both | 14049.90 (12169.86 to 15929.93) | 6430.07 (5576.25 to 7283.89) |
| Samoa | 2023 | Both | 13544.26 (9612.21 to 17476.31) | 6075.74 (4314.85 to 7836.62) |
| Samoa | 2024 | Both | 13054.82 (6770.84 to 19338.81) | 5740.90 (2979.25 to 8502.54) |
| Samoa | 2025 | Both | 12585.09 (3756.83 to 21413.34) | 5424.54 (1620.48 to 9228.60) |
| Samoa | 2026 | Both | 12133.86 (639.70 to 23628.02) | 5125.55 (271.08 to 9980.02) |
| Samoa | 2027 | Both | 11695.80 (0.00 to 25921.52) | 4842.91 (0.00 to 10732.73) |
| Samoa | 2028 | Both | 11271.86 (0.00 to 28255.47) | 4575.77 (0.00 to 11469.68) |
| Samoa | 2029 | Both | 10860.52 (0.00 to 30594.70) | 4323.37 (0.00 to 12178.76) |
| Samoa | 2030 | Both | 10463.76 (0.00 to 32920.11) | 4084.93 (0.00 to 12851.29) |
| Sao Tome and Principe | 2022 | Male | 3314.03 (2859.79 to 3768.27) | 3086.55 (2676.74 to 3496.35) |
| Sao Tome and Principe | 2023 | Male | 3146.41 (2278.32 to 4014.50) | 2891.77 (2100.36 to 3683.18) |
| Sao Tome and Principe | 2024 | Male | 2987.17 (1653.57 to 4320.77) | 2709.78 (1503.93 to 3915.64) |
| Sao Tome and Principe | 2025 | Male | 2836.38 (1012.98 to 4659.78) | 2539.76 (909.72 to 4169.80) |
| Sao Tome and Principe | 2026 | Male | 2693.60 (371.93 to 5015.28) | 2380.96 (330.73 to 4431.18) |
| Sao Tome and Principe | 2027 | Male | 2557.66 (0.00 to 5374.76) | 2232.57 (0.00 to 4690.08) |
| Sao Tome and Principe | 2028 | Male | 2427.79 (0.00 to 5729.01) | 2093.86 (0.00 to 4939.78) |
| Sao Tome and Principe | 2029 | Male | 2303.78 (0.00 to 6071.83) | 1964.16 (0.00 to 5175.72) |
| Sao Tome and Principe | 2030 | Male | 2186.01 (0.00 to 6400.42) | 1842.89 (0.00 to 5394.94) |
| Sao Tome and Principe | 2022 | Female | 2815.97 (2431.14 to 3200.79) | 2623.28 (2278.13 to 2968.43) |
| Sao Tome and Principe | 2023 | Female | 2688.96 (1962.57 to 3415.35) | 2471.70 (1810.57 to 3132.84) |
| Sao Tome and Principe | 2024 | Female | 2567.49 (1452.24 to 3682.74) | 2329.10 (1321.42 to 3336.78) |
| Sao Tome and Principe | 2025 | Female | 2451.75 (923.04 to 3980.47) | 2194.98 (829.13 to 3560.83) |
| Sao Tome and Principe | 2026 | Female | 2341.49 (387.72 to 4295.26) | 2068.89 (344.62 to 3793.16) |
| Sao Tome and Principe | 2027 | Female | 2235.80 (0.00 to 4616.80) | 1950.31 (0.00 to 4025.70) |
| Sao Tome and Principe | 2028 | Female | 2134.12 (0.00 to 4937.31) | 1838.76 (0.00 to 4252.73) |
| Sao Tome and Principe | 2029 | Female | 2036.33 (0.00 to 5251.49) | 1733.79 (0.00 to 4470.23) |
| Sao Tome and Principe | 2030 | Female | 1942.90 (0.00 to 5556.82) | 1635.06 (0.00 to 4675.50) |
| Sao Tome and Principe | 2022 | Both | 6117.20 (5365.05 to 6869.36) | 2848.98 (2506.03 to 3191.94) |
| Sao Tome and Principe | 2023 | Both | 5824.53 (4336.54 to 7312.51) | 2676.77 (1996.40 to 3357.13) |
| Sao Tome and Principe | 2024 | Both | 5545.61 (3228.64 to 7862.59) | 2515.34 (1466.51 to 3564.17) |
| Sao Tome and Principe | 2025 | Both | 5280.74 (2087.00 to 8474.48) | 2364.05 (935.72 to 3792.37) |
| Sao Tome and Principe | 2026 | Both | 5029.22 (938.31 to 9120.12) | 2222.29 (415.66 to 4028.92) |
| Sao Tome and Principe | 2027 | Both | 4788.95 (0.00 to 9777.36) | 2089.43 (0.00 to 4265.07) |
| Sao Tome and Principe | 2028 | Both | 4558.64 (0.00 to 10429.63) | 1964.83 (0.00 to 4494.66) |
| Sao Tome and Principe | 2029 | Both | 4337.94 (0.00 to 11065.64) | 1847.97 (0.00 to 4713.46) |
| Sao Tome and Principe | 2030 | Both | 4127.73 (0.00 to 11680.13) | 1738.38 (0.00 to 4918.61) |
| Senegal | 2022 | Male | 54162.45 (48185.90 to 60139.00) | 647.58 (576.33 to 718.83) |
| Senegal | 2023 | Male | 53729.98 (40735.71 to 66724.24) | 626.74 (475.26 to 778.23) |
| Senegal | 2024 | Male | 53311.32 (31867.75 to 74754.89) | 606.96 (362.87 to 851.04) |
| Senegal | 2025 | Male | 52903.71 (21846.04 to 83961.37) | 588.15 (242.91 to 933.39) |
| Senegal | 2026 | Male | 52502.28 (10843.50 to 94161.06) | 570.24 (117.80 to 1022.68) |
| Senegal | 2027 | Male | 52106.22 (0.00 to 105224.29) | 553.19 (0.00 to 1117.09) |
| Senegal | 2028 | Male | 51720.44 (0.00 to 117061.99) | 536.96 (0.00 to 1215.33) |
| Senegal | 2029 | Male | 51343.86 (0.00 to 129594.75) | 521.52 (0.00 to 1316.33) |
| Senegal | 2030 | Male | 50975.37 (0.00 to 142756.13) | 506.78 (0.00 to 1419.24) |
| Senegal | 2022 | Female | 43453.13 (38649.09 to 48257.16) | 520.72 (463.36 to 578.08) |
| Senegal | 2023 | Female | 42908.26 (32469.61 to 53346.90) | 501.66 (379.71 to 623.61) |
| Senegal | 2024 | Female | 42398.72 (25222.42 to 59575.02) | 483.82 (287.87 to 679.77) |
| Senegal | 2025 | Female | 41924.45 (17121.15 to 66727.75) | 467.17 (190.82 to 743.52) |
| Senegal | 2026 | Female | 41482.35 (8300.01 to 74664.68) | 451.60 (90.38 to 812.81) |
| Senegal | 2027 | Female | 41072.17 (0.00 to 83292.40) | 437.07 (0.00 to 886.33) |
| Senegal | 2028 | Female | 40699.31 (0.00 to 92556.68) | 423.54 (0.00 to 963.18) |
| Senegal | 2029 | Female | 40364.72 (0.00 to 102416.85) | 410.97 (0.00 to 1042.74) |
| Senegal | 2030 | Female | 40069.58 (0.00 to 112848.45) | 399.31 (0.00 to 1124.57) |
| Senegal | 2022 | Both | 97329.03 (86677.52 to 107980.54) | 582.50 (518.86 to 646.15) |
| Senegal | 2023 | Both | 96339.42 (73057.56 to 119621.28) | 562.53 (426.63 to 698.42) |
| Senegal | 2024 | Both | 95398.28 (56966.19 to 133830.38) | 543.68 (324.68 to 762.68) |
| Senegal | 2025 | Both | 94501.50 (38874.47 to 150128.53) | 525.91 (216.36 to 835.46) |
| Senegal | 2026 | Both | 93641.91 (19090.48 to 168193.35) | 509.12 (103.81 to 914.44) |
| Senegal | 2027 | Both | 92819.88 (0.00 to 187803.00) | 493.29 (0.00 to 998.06) |
| Senegal | 2028 | Both | 92045.88 (0.00 to 208814.11) | 478.38 (0.00 to 1085.23) |
| Senegal | 2029 | Both | 91318.33 (0.00 to 231102.42) | 464.33 (0.00 to 1175.08) |
| Senegal | 2030 | Both | 90635.62 (0.00 to 254570.81) | 451.07 (0.00 to 1266.94) |
| Sierra Leone | 2022 | Male | 193744.42 (173197.49 to 214291.34) | 4439.01 (3968.66 to 4909.36) |
| Sierra Leone | 2023 | Male | 186728.35 (143202.98 to 230253.72) | 4184.42 (3209.24 to 5159.61) |
| Sierra Leone | 2024 | Male | 179897.90 (110088.81 to 249706.98) | 3945.00 (2414.26 to 5475.75) |
| Sierra Leone | 2025 | Male | 173257.14 (75084.33 to 271429.96) | 3719.99 (1612.20 to 5827.78) |
| Sierra Leone | 2026 | Male | 166809.61 (39020.27 to 294598.96) | 3508.53 (820.77 to 6196.29) |
| Sierra Leone | 2027 | Male | 160518.54 (2499.61 to 318537.47) | 3309.70 (51.58 to 6567.82) |
| Sierra Leone | 2028 | Male | 154406.60 (0.00 to 342809.04) | 3122.75 (0.00 to 6933.00) |
| Sierra Leone | 2029 | Male | 148457.14 (0.00 to 367002.83) | 2946.98 (0.00 to 7285.25) |
| Sierra Leone | 2030 | Male | 142698.34 (0.00 to 390890.09) | 2781.85 (0.00 to 7620.23) |
| Sierra Leone | 2022 | Female | 162290.05 (145247.91 to 179332.19) | 3650.89 (3267.92 to 4033.86) |
| Sierra Leone | 2023 | Female | 157271.95 (120578.83 to 193965.07) | 3462.96 (2655.20 to 4270.72) |
| Sierra Leone | 2024 | Female | 152370.31 (92999.41 to 211741.21) | 3285.52 (2005.43 to 4565.61) |
| Sierra Leone | 2025 | Female | 147580.99 (63482.50 to 231679.48) | 3117.89 (1341.24 to 4894.54) |
| Sierra Leone | 2026 | Female | 142898.20 (32704.39 to 253092.01) | 2959.34 (677.34 to 5241.35) |
| Sierra Leone | 2027 | Female | 138302.54 (1171.39 to 275433.69) | 2809.49 (23.84 to 5595.15) |
| Sierra Leone | 2028 | Female | 133816.35 (0.00 to 298345.10) | 2667.92 (0.00 to 5948.13) |
| Sierra Leone | 2029 | Female | 129423.11 (0.00 to 321470.33) | 2534.13 (0.00 to 6294.42) |
| Sierra Leone | 2030 | Female | 125141.80 (0.00 to 344592.94) | 2407.62 (0.00 to 6629.66) |
| Sierra Leone | 2022 | Both | 356809.00 (319509.99 to 394108.01) | 4050.13 (3626.96 to 4473.31) |
| Sierra Leone | 2023 | Both | 344851.02 (264790.50 to 424911.54) | 3829.97 (2940.90 to 4719.05) |
| Sierra Leone | 2024 | Both | 333187.78 (204014.92 to 462360.65) | 3622.48 (2218.14 to 5026.82) |
| Sierra Leone | 2025 | Both | 321818.39 (139362.71 to 504274.07) | 3426.95 (1484.07 to 5369.83) |
| Sierra Leone | 2026 | Both | 310739.49 (72340.75 to 549138.23) | 3242.57 (754.90 to 5730.25) |
| Sierra Leone | 2027 | Both | 299897.27 (4063.25 to 595731.29) | 3068.75 (41.60 to 6095.90) |
| Sierra Leone | 2028 | Both | 289338.15 (0.00 to 643256.19) | 2904.91 (0.00 to 6458.17) |
| Sierra Leone | 2029 | Both | 279029.00 (0.00 to 690942.48) | 2750.46 (0.00 to 6810.79) |
| Sierra Leone | 2030 | Both | 269017.90 (0.00 to 738343.27) | 2604.91 (0.00 to 7149.38) |
| South Sudan | 2022 | Male | 68624.30 (61619.60 to 75629.00) | 1149.61 (1032.58 to 1266.64) |
| South Sudan | 2023 | Male | 67957.87 (52840.23 to 83075.50) | 1102.54 (857.41 to 1347.67) |
| South Sudan | 2024 | Male | 67306.19 (42448.19 to 92164.19) | 1057.64 (667.11 to 1448.18) |
| South Sudan | 2025 | Male | 66685.18 (30771.41 to 102598.95) | 1014.80 (468.33 to 1561.28) |
| South Sudan | 2026 | Male | 66094.52 (18009.37 to 114179.67) | 973.88 (265.40 to 1682.35) |
| South Sudan | 2027 | Male | 65507.80 (4299.87 to 126715.72) | 934.76 (61.39 to 1808.13) |
| South Sudan | 2028 | Male | 64924.23 (0.00 to 140083.83) | 897.37 (0.00 to 1936.18) |
| South Sudan | 2029 | Male | 64346.46 (0.00 to 154190.92) | 861.62 (0.00 to 2064.64) |
| South Sudan | 2030 | Male | 63796.79 (0.00 to 169014.61) | 827.42 (0.00 to 2192.05) |
| South Sudan | 2022 | Female | 56105.64 (50691.79 to 61519.50) | 970.94 (877.60 to 1064.29) |
| South Sudan | 2023 | Female | 55528.89 (43798.16 to 67259.62) | 928.85 (732.78 to 1124.92) |
| South Sudan | 2024 | Female | 54963.75 (35659.75 to 74267.74) | 888.86 (576.77 to 1200.95) |
| South Sudan | 2025 | Female | 54425.18 (26533.04 to 82317.31) | 850.87 (414.87 to 1286.87) |
| South Sudan | 2026 | Female | 53914.85 (16574.58 to 91255.12) | 814.76 (250.52 to 1379.01) |
| South Sudan | 2027 | Female | 53410.17 (5890.07 to 100930.28) | 780.42 (86.10 to 1474.74) |
| South Sudan | 2028 | Female | 52910.50 (0.00 to 111247.07) | 747.74 (0.00 to 1572.14) |
| South Sudan | 2029 | Female | 52418.24 (0.00 to 122134.27) | 716.65 (0.00 to 1669.78) |
| South Sudan | 2030 | Female | 51953.58 (0.00 to 133581.40) | 687.07 (0.00 to 1766.56) |
| South Sudan | 2022 | Both | 124584.69 (112310.88 to 136858.50) | 1060.49 (956.18 to 1164.80) |
| South Sudan | 2023 | Both | 123373.27 (96679.18 to 150067.36) | 1016.09 (796.31 to 1235.86) |
| South Sudan | 2024 | Both | 122188.73 (78190.58 to 166186.87) | 973.82 (623.20 to 1324.43) |
| South Sudan | 2025 | Both | 121060.54 (57419.14 to 184701.95) | 933.56 (442.82 to 1424.30) |
| South Sudan | 2026 | Both | 119988.60 (34718.00 to 205259.19) | 895.17 (259.03 to 1531.31) |
| South Sudan | 2027 | Both | 118924.97 (10330.50 to 227519.45) | 858.55 (74.59 to 1642.51) |
| South Sudan | 2028 | Both | 117868.93 (0.00 to 251265.66) | 823.62 (0.00 to 1755.74) |
| South Sudan | 2029 | Both | 116825.64 (0.00 to 276334.50) | 790.30 (0.00 to 1869.34) |
| South Sudan | 2030 | Both | 115836.45 (0.00 to 302690.89) | 758.49 (0.00 to 1982.00) |
| Sri Lanka | 2022 | Male | 13451.23 (12195.00 to 14707.47) | 126.70 (115.06 to 138.33) |
| Sri Lanka | 2023 | Male | 12733.76 (10415.72 to 15051.80) | 119.63 (97.96 to 141.31) |
| Sri Lanka | 2024 | Male | 12048.18 (8536.81 to 15559.54) | 112.95 (80.09 to 145.81) |
| Sri Lanka | 2025 | Male | 11395.94 (6638.06 to 16153.82) | 106.64 (62.16 to 151.12) |
| Sri Lanka | 2026 | Male | 10775.49 (4761.98 to 16789.00) | 100.68 (44.52 to 156.83) |
| Sri Lanka | 2027 | Male | 10183.09 (2935.51 to 17430.67) | 95.03 (27.42 to 162.65) |
| Sri Lanka | 2028 | Male | 9618.73 (1179.25 to 18058.21) | 89.69 (11.02 to 168.37) |
| Sri Lanka | 2029 | Male | 9082.22 (0.00 to 18657.22) | 84.65 (0.00 to 173.87) |
| Sri Lanka | 2030 | Male | 8573.53 (0.00 to 19218.61) | 79.89 (0.00 to 179.07) |
| Sri Lanka | 2022 | Female | 14457.99 (13244.52 to 15671.45) | 126.90 (116.45 to 137.35) |
| Sri Lanka | 2023 | Female | 13694.59 (11452.30 to 15936.88) | 119.78 (100.27 to 139.29) |
| Sri Lanka | 2024 | Female | 12961.61 (9562.26 to 16360.95) | 113.03 (83.45 to 142.61) |
| Sri Lanka | 2025 | Female | 12260.87 (7653.19 to 16868.55) | 106.62 (66.60 to 146.65) |
| Sri Lanka | 2026 | Female | 11590.99 (5767.51 to 17414.47) | 100.55 (50.07 to 151.04) |
| Sri Lanka | 2027 | Female | 10948.45 (3932.44 to 17964.46) | 94.79 (34.07 to 155.50) |
| Sri Lanka | 2028 | Female | 10333.60 (2169.42 to 18497.78) | 89.31 (18.77 to 159.86) |
| Sri Lanka | 2029 | Female | 9746.60 (493.39 to 18999.81) | 84.13 (4.28 to 163.97) |
| Sri Lanka | 2030 | Female | 9187.80 (0.00 to 19461.23) | 79.22 (0.00 to 167.78) |
| Sri Lanka | 2022 | Both | 27948.61 (25535.27 to 30361.95) | 126.98 (116.12 to 137.84) |
| Sri Lanka | 2023 | Both | 26473.05 (22001.24 to 30944.86) | 119.91 (99.71 to 140.12) |
| Sri Lanka | 2024 | Both | 25059.05 (18271.30 to 31846.79) | 113.21 (82.58 to 143.85) |
| Sri Lanka | 2025 | Both | 23709.82 (14501.69 to 32917.94) | 106.87 (65.39 to 148.35) |
| Sri Lanka | 2026 | Both | 22422.62 (10776.29 to 34068.94) | 100.87 (48.49 to 153.24) |
| Sri Lanka | 2027 | Both | 21190.40 (7148.64 to 35232.17) | 95.17 (32.12 to 158.22) |
| Sri Lanka | 2028 | Both | 20013.44 (3660.25 to 36366.63) | 89.77 (16.43 to 163.11) |
| Sri Lanka | 2029 | Both | 18891.58 (339.87 to 37443.30) | 84.66 (1.53 to 167.78) |
| Sri Lanka | 2030 | Both | 17825.15 (0.00 to 38443.71) | 79.83 (0.00 to 172.15) |
| Sudan | 2022 | Male | 228996.07 (222106.10 to 235886.03) | 995.99 (966.30 to 1025.68) |
| Sudan | 2023 | Male | 223926.29 (210064.03 to 237788.56) | 952.96 (894.10 to 1011.82) |
| Sudan | 2024 | Male | 218884.87 (196797.79 to 240971.95) | 912.10 (820.14 to 1004.06) |
| Sudan | 2025 | Male | 213896.87 (182707.03 to 245086.71) | 873.32 (746.03 to 1000.61) |
| Sudan | 2026 | Male | 208973.45 (168032.92 to 249913.98) | 836.48 (672.64 to 1000.32) |
| Sudan | 2027 | Male | 204101.26 (152929.90 to 255272.62) | 801.44 (600.54 to 1002.34) |
| Sudan | 2028 | Male | 199287.35 (137540.20 to 261034.49) | 768.08 (530.12 to 1006.04) |
| Sudan | 2029 | Male | 194539.04 (121979.44 to 267098.64) | 736.33 (461.71 to 1010.95) |
| Sudan | 2030 | Male | 189888.65 (106357.27 to 273420.02) | 706.12 (395.52 to 1016.73) |
| Sudan | 2022 | Female | 187724.06 (182351.16 to 193096.95) | 834.65 (811.07 to 858.24) |
| Sudan | 2023 | Female | 183099.86 (172308.36 to 193891.37) | 796.15 (749.37 to 842.93) |
| Sudan | 2024 | Female | 178553.11 (161397.04 to 195709.17) | 759.82 (686.90 to 832.74) |
| Sudan | 2025 | Female | 174105.87 (149931.02 to 198280.72) | 725.58 (624.89 to 826.27) |
| Sudan | 2026 | Female | 169766.41 (138095.65 to 201437.17) | 693.28 (563.99 to 822.58) |
| Sudan | 2027 | Female | 165519.43 (126002.82 to 205036.03) | 662.77 (504.57 to 820.97) |
| Sudan | 2028 | Female | 161374.02 (113761.12 to 208986.91) | 633.93 (446.92 to 820.95) |
| Sudan | 2029 | Female | 157336.58 (101454.17 to 213219.00) | 606.71 (391.24 to 822.18) |
| Sudan | 2030 | Female | 153434.08 (89160.53 to 217707.62) | 581.02 (337.65 to 824.40) |
| Sudan | 2022 | Both | 417241.06 (405631.50 to 428850.61) | 917.36 (891.98 to 942.73) |
| Sudan | 2023 | Both | 407558.26 (383742.62 to 431373.91) | 876.54 (825.39 to 927.69) |
| Sudan | 2024 | Both | 397980.86 (359817.02 to 436144.69) | 837.90 (757.60 to 918.21) |
| Sudan | 2025 | Both | 388554.40 (334532.23 to 442576.57) | 801.35 (689.96 to 912.73) |
| Sudan | 2026 | Both | 379298.39 (308305.00 to 450291.78) | 766.73 (623.24 to 910.21) |
| Sudan | 2027 | Both | 370188.66 (281400.48 to 458976.85) | 733.91 (557.90 to 909.92) |
| Sudan | 2028 | Both | 361239.40 (254063.62 to 468415.18) | 702.77 (494.28 to 911.26) |
| Sudan | 2029 | Both | 352462.55 (226489.36 to 478435.75) | 673.24 (432.63 to 913.86) |
| Sudan | 2030 | Both | 343916.82 (198863.96 to 488969.69) | 645.26 (373.12 to 917.40) |
| Thailand | 2022 | Male | 144294.21 (112686.07 to 175902.35) | 414.25 (323.53 to 504.96) |
| Thailand | 2023 | Male | 136549.30 (69976.89 to 203121.71) | 391.55 (200.67 to 582.43) |
| Thailand | 2024 | Male | 129197.64 (23945.74 to 234449.54) | 370.15 (68.61 to 671.68) |
| Thailand | 2025 | Male | 122225.77 (0.00 to 267883.73) | 349.97 (0.00 to 767.04) |
| Thailand | 2026 | Male | 115619.36 (0.00 to 302103.51) | 330.97 (0.00 to 864.79) |
| Thailand | 2027 | Male | 109355.12 (0.00 to 336169.57) | 313.06 (0.00 to 962.37) |
| Thailand | 2028 | Male | 103410.39 (0.00 to 369394.80) | 296.15 (0.00 to 1057.88) |
| Thailand | 2029 | Male | 97765.60 (0.00 to 401273.20) | 280.19 (0.00 to 1150.01) |
| Thailand | 2030 | Male | 92414.96 (0.00 to 431484.92) | 265.13 (0.00 to 1237.90) |
| Thailand | 2022 | Female | 151244.08 (118298.55 to 184189.60) | 412.29 (322.51 to 502.08) |
| Thailand | 2023 | Female | 143171.57 (73592.92 to 212750.22) | 389.32 (200.13 to 578.51) |
| Thailand | 2024 | Female | 135473.87 (25383.65 to 245564.09) | 367.59 (68.88 to 666.30) |
| Thailand | 2025 | Female | 128140.92 (0.00 to 280519.15) | 347.05 (0.00 to 759.74) |
| Thailand | 2026 | Female | 121160.67 (0.00 to 316200.39) | 327.65 (0.00 to 855.07) |
| Thailand | 2027 | Female | 114511.52 (0.00 to 351587.93) | 309.30 (0.00 to 949.64) |
| Thailand | 2028 | Female | 108177.50 (0.00 to 385942.61) | 291.94 (0.00 to 1041.54) |
| Thailand | 2029 | Female | 102143.97 (0.00 to 418718.46) | 275.52 (0.00 to 1129.45) |
| Thailand | 2030 | Female | 96406.14 (0.00 to 449553.26) | 260.01 (0.00 to 1212.43) |
| Thailand | 2022 | Both | 295681.13 (231127.46 to 360234.81) | 413.44 (323.19 to 503.70) |
| Thailand | 2023 | Both | 279884.65 (143673.94 to 416095.37) | 390.63 (200.53 to 580.73) |
| Thailand | 2024 | Both | 264852.08 (49372.73 to 480331.42) | 369.09 (68.81 to 669.36) |
| Thailand | 2025 | Both | 250561.70 (0.00 to 548831.55) | 348.74 (0.00 to 763.89) |
| Thailand | 2026 | Both | 236990.51 (0.00 to 618866.89) | 329.55 (0.00 to 860.58) |
| Thailand | 2027 | Both | 224092.45 (0.00 to 688476.69) | 311.44 (0.00 to 956.82) |
| Thailand | 2028 | Both | 211826.25 (0.00 to 756225.74) | 294.31 (0.00 to 1050.70) |
| Thailand | 2029 | Both | 200157.24 (0.00 to 821056.67) | 278.13 (0.00 to 1140.90) |
| Thailand | 2030 | Both | 189076.61 (0.00 to 882290.66) | 262.85 (0.00 to 1226.52) |
| Timor-Leste | 2022 | Male | 80338.78 (53407.93 to 107269.63) | 11222.84 (7461.56 to 14984.11) |
| Timor-Leste | 2023 | Male | 77453.99 (19529.57 to 135378.40) | 10617.04 (2677.38 to 18556.70) |
| Timor-Leste | 2024 | Male | 74676.01 (0.00 to 168053.28) | 10044.45 (0.00 to 22604.15) |
| Timor-Leste | 2025 | Male | 72005.45 (0.00 to 203752.46) | 9501.49 (0.00 to 26886.06) |
| Timor-Leste | 2026 | Male | 69415.32 (0.00 to 241340.00) | 8985.77 (0.00 to 31241.21) |
| Timor-Leste | 2027 | Male | 66896.47 (0.00 to 279981.11) | 8497.52 (0.00 to 35564.49) |
| Timor-Leste | 2028 | Male | 64451.98 (0.00 to 319065.43) | 8036.67 (0.00 to 39784.97) |
| Timor-Leste | 2029 | Male | 62065.94 (0.00 to 358017.49) | 7601.17 (0.00 to 43846.11) |
| Timor-Leste | 2030 | Male | 59739.15 (0.00 to 396419.49) | 7188.22 (0.00 to 47699.82) |
| Timor-Leste | 2022 | Female | 57762.89 (40342.70 to 75183.08) | 8255.23 (5766.52 to 10743.94) |
| Timor-Leste | 2023 | Female | 55849.14 (18272.00 to 93426.27) | 7826.60 (2561.01 to 13092.19) |
| Timor-Leste | 2024 | Female | 54007.62 (0.00 to 114767.11) | 7421.80 (0.00 to 15771.22) |
| Timor-Leste | 2025 | Female | 52240.67 (0.00 to 138239.17) | 7038.33 (0.00 to 18624.65) |
| Timor-Leste | 2026 | Female | 50528.19 (0.00 to 163125.73) | 6674.27 (0.00 to 21547.16) |
| Timor-Leste | 2027 | Female | 48862.00 (0.00 to 188896.68) | 6329.68 (0.00 to 24469.96) |
| Timor-Leste | 2028 | Female | 47241.76 (0.00 to 215156.13) | 6004.23 (0.00 to 27345.37) |
| Timor-Leste | 2029 | Female | 45656.32 (0.00 to 241533.84) | 5696.49 (0.00 to 30135.87) |
| Timor-Leste | 2030 | Female | 44108.23 (0.00 to 267770.74) | 5404.62 (0.00 to 32810.12) |
| Timor-Leste | 2022 | Both | 137822.90 (93748.74 to 181897.06) | 9736.26 (6623.14 to 12849.38) |
| Timor-Leste | 2023 | Both | 133033.29 (38050.85 to 228015.72) | 9218.54 (2636.92 to 15800.15) |
| Timor-Leste | 2024 | Both | 128425.89 (0.00 to 281773.93) | 8729.66 (0.00 to 19153.28) |
| Timor-Leste | 2025 | Both | 123997.02 (0.00 to 340668.83) | 8266.11 (0.00 to 22710.21) |
| Timor-Leste | 2026 | Both | 119695.41 (0.00 to 402838.96) | 7825.47 (0.00 to 26336.82) |
| Timor-Leste | 2027 | Both | 115510.70 (0.00 to 466939.61) | 7408.34 (0.00 to 29947.38) |
| Timor-Leste | 2028 | Both | 111451.48 (0.00 to 531995.20) | 7014.90 (0.00 to 33484.44) |
| Timor-Leste | 2029 | Both | 107488.61 (0.00 to 597064.34) | 6643.25 (0.00 to 36901.07) |
| Timor-Leste | 2030 | Both | 103619.33 (0.00 to 661442.87) | 6290.67 (0.00 to 40155.77) |
| Togo | 2022 | Male | 7542.88 (5325.14 to 9760.62) | 184.28 (130.26 to 238.30) |
| Togo | 2023 | Male | 7257.45 (2617.00 to 11897.90) | 173.98 (62.81 to 285.15) |
| Togo | 2024 | Male | 6978.50 (0.00 to 14382.04) | 164.28 (0.00 to 338.52) |
| Togo | 2025 | Male | 6707.73 (0.00 to 17081.76) | 155.13 (0.00 to 395.02) |
| Togo | 2026 | Male | 6445.10 (0.00 to 19908.09) | 146.51 (0.00 to 452.52) |
| Togo | 2027 | Male | 6188.85 (0.00 to 22790.12) | 138.37 (0.00 to 509.53) |
| Togo | 2028 | Male | 5939.54 (0.00 to 25678.11) | 130.70 (0.00 to 565.03) |
| Togo | 2029 | Male | 5696.98 (0.00 to 28530.70) | 123.46 (0.00 to 618.27) |
| Togo | 2030 | Male | 5462.32 (0.00 to 31321.98) | 116.62 (0.00 to 668.73) |
| Togo | 2022 | Female | 7904.10 (5707.74 to 10100.47) | 185.93 (134.43 to 237.43) |
| Togo | 2023 | Female | 7609.41 (2987.42 to 12231.39) | 175.70 (69.05 to 282.35) |
| Togo | 2024 | Female | 7319.27 (0.00 to 14707.43) | 166.00 (0.00 to 333.52) |
| Togo | 2025 | Female | 7035.64 (0.00 to 17396.67) | 156.80 (0.00 to 387.68) |
| Togo | 2026 | Female | 6758.66 (0.00 to 20208.05) | 148.08 (0.00 to 442.72) |
| Togo | 2027 | Female | 6486.64 (0.00 to 23067.86) | 139.80 (0.00 to 497.14) |
| Togo | 2028 | Female | 6220.27 (0.00 to 25923.33) | 131.95 (0.00 to 549.88) |
| Togo | 2029 | Female | 5959.49 (0.00 to 28730.15) | 124.50 (0.00 to 600.17) |
| Togo | 2030 | Female | 5705.69 (0.00 to 31459.67) | 117.43 (0.00 to 647.46) |
| Togo | 2022 | Both | 15558.29 (11118.10 to 19998.48) | 186.45 (133.32 to 239.58) |
| Togo | 2023 | Both | 14977.03 (5634.70 to 24319.35) | 176.15 (66.31 to 286.00) |
| Togo | 2024 | Both | 14406.34 (0.00 to 29340.36) | 166.41 (0.00 to 338.89) |
| Togo | 2025 | Both | 13849.88 (0.00 to 34796.07) | 157.19 (0.00 to 394.90) |
| Togo | 2026 | Both | 13307.80 (0.00 to 40504.19) | 148.47 (0.00 to 451.87) |
| Togo | 2027 | Both | 12776.73 (0.00 to 46318.21) | 140.21 (0.00 to 508.28) |
| Togo | 2028 | Both | 12257.98 (0.00 to 52133.98) | 132.39 (0.00 to 563.07) |
| Togo | 2029 | Both | 11751.31 (0.00 to 57864.21) | 124.99 (0.00 to 615.48) |
| Togo | 2030 | Both | 11259.25 (0.00 to 63452.88) | 117.99 (0.00 to 664.94) |
| Tonga | 2022 | Male | 269.86 (175.08 to 364.64) | 486.61 (325.87 to 647.35) |
| Tonga | 2023 | Male | 265.54 (92.55 to 438.53) | 471.16 (169.49 to 772.83) |
| Tonga | 2024 | Male | 261.43 (0.00 to 528.90) | 456.38 (0.00 to 919.98) |
| Tonga | 2025 | Male | 257.56 (0.00 to 631.29) | 442.24 (0.00 to 1081.66) |
| Tonga | 2026 | Male | 253.92 (0.00 to 743.44) | 428.73 (0.00 to 1253.56) |
| Tonga | 2027 | Male | 250.51 (0.00 to 863.88) | 415.83 (0.00 to 1432.70) |
| Tonga | 2028 | Male | 247.31 (0.00 to 991.58) | 403.51 (0.00 to 1616.84) |
| Tonga | 2029 | Male | 244.30 (0.00 to 1125.76) | 391.73 (0.00 to 1804.28) |
| Tonga | 2030 | Male | 241.48 (0.00 to 1265.82) | 380.48 (0.00 to 1993.75) |
| Tonga | 2022 | Female | 211.74 (134.45 to 289.03) | 380.57 (251.46 to 509.68) |
| Tonga | 2023 | Female | 209.19 (71.25 to 347.12) | 369.92 (131.21 to 608.64) |
| Tonga | 2024 | Female | 206.82 (0.00 to 418.80) | 359.78 (0.00 to 725.24) |
| Tonga | 2025 | Female | 204.66 (0.00 to 500.62) | 350.12 (0.00 to 854.16) |
| Tonga | 2026 | Female | 202.69 (0.00 to 590.91) | 340.95 (0.00 to 992.28) |
| Tonga | 2027 | Female | 200.92 (0.00 to 688.66) | 332.24 (0.00 to 1137.44) |
| Tonga | 2028 | Female | 199.33 (0.00 to 793.20) | 323.96 (0.00 to 1288.09) |
| Tonga | 2029 | Female | 197.92 (0.00 to 904.05) | 316.10 (0.00 to 1443.04) |
| Tonga | 2030 | Female | 196.67 (0.00 to 1020.89) | 308.64 (0.00 to 1601.41) |
| Tonga | 2022 | Both | 478.91 (332.94 to 624.87) | 431.07 (305.49 to 556.66) |
| Tonga | 2023 | Both | 471.63 (192.41 to 750.85) | 417.71 (173.30 to 662.12) |
| Tonga | 2024 | Both | 464.80 (25.07 to 904.53) | 404.97 (23.62 to 786.33) |
| Tonga | 2025 | Both | 458.42 (0.00 to 1079.02) | 392.84 (0.00 to 923.44) |
| Tonga | 2026 | Both | 452.50 (0.00 to 1270.72) | 381.29 (0.00 to 1069.85) |
| Tonga | 2027 | Both | 447.00 (0.00 to 1477.28) | 370.29 (0.00 to 1223.06) |
| Tonga | 2028 | Both | 441.90 (0.00 to 1697.01) | 359.80 (0.00 to 1381.17) |
| Tonga | 2029 | Both | 437.16 (0.00 to 1928.64) | 349.79 (0.00 to 1542.75) |
| Tonga | 2030 | Both | 432.76 (0.00 to 2171.23) | 340.25 (0.00 to 1706.74) |
| Uganda | 2022 | Male | 80053.76 (66129.20 to 93978.33) | 353.20 (291.81 to 414.59) |
| Uganda | 2023 | Male | 81498.21 (51352.56 to 111643.86) | 348.25 (219.45 to 477.04) |
| Uganda | 2024 | Male | 83039.56 (32419.98 to 133659.14) | 343.83 (134.25 to 553.41) |
| Uganda | 2025 | Male | 84679.38 (9675.29 to 159683.47) | 339.93 (38.85 to 641.01) |
| Uganda | 2026 | Male | 86412.17 (0.00 to 189588.66) | 336.51 (0.00 to 738.31) |
| Uganda | 2027 | Male | 88237.27 (0.00 to 223357.49) | 333.57 (0.00 to 844.38) |
| Uganda | 2028 | Male | 90164.02 (0.00 to 261060.38) | 331.13 (0.00 to 958.74) |
| Uganda | 2029 | Male | 92196.98 (0.00 to 302808.33) | 329.17 (0.00 to 1081.10) |
| Uganda | 2030 | Male | 94338.41 (0.00 to 348743.75) | 327.67 (0.00 to 1211.32) |
| Uganda | 2022 | Female | 62529.86 (51575.00 to 73484.71) | 267.46 (220.65 to 314.27) |
| Uganda | 2023 | Female | 63499.26 (39309.68 to 87688.84) | 263.11 (162.90 to 363.32) |
| Uganda | 2024 | Female | 64605.70 (23769.64 to 105441.76) | 259.44 (95.47 to 423.42) |
| Uganda | 2025 | Female | 65853.00 (5171.44 to 126534.57) | 256.44 (20.15 to 492.72) |
| Uganda | 2026 | Female | 67237.35 (0.00 to 150921.36) | 254.04 (0.00 to 570.20) |
| Uganda | 2027 | Female | 68763.74 (0.00 to 178669.79) | 252.24 (0.00 to 655.38) |
| Uganda | 2028 | Female | 70444.75 (0.00 to 209936.48) | 251.05 (0.00 to 748.16) |
| Uganda | 2029 | Female | 72287.45 (0.00 to 244925.03) | 250.46 (0.00 to 848.62) |
| Uganda | 2030 | Female | 74294.43 (0.00 to 283870.82) | 250.45 (0.00 to 956.92) |
| Uganda | 2022 | Both | 141850.00 (117167.08 to 166532.92) | 308.07 (254.49 to 361.66) |
| Uganda | 2023 | Both | 144379.71 (90151.97 to 198607.45) | 303.72 (189.66 to 417.79) |
| Uganda | 2024 | Both | 147163.00 (55646.79 to 238679.22) | 300.01 (113.45 to 486.57) |
| Uganda | 2025 | Both | 150209.08 (14170.34 to 286247.82) | 296.91 (28.01 to 565.80) |
| Uganda | 2026 | Both | 153515.78 (0.00 to 341192.85) | 294.39 (0.00 to 654.30) |
| Uganda | 2027 | Both | 157091.37 (0.00 to 403619.76) | 292.46 (0.00 to 751.43) |
| Uganda | 2028 | Both | 160958.71 (0.00 to 473812.81) | 291.12 (0.00 to 856.96) |
| Uganda | 2029 | Both | 165133.03 (0.00 to 552158.22) | 290.37 (0.00 to 970.90) |
| Uganda | 2030 | Both | 169625.82 (0.00 to 639126.86) | 290.18 (0.00 to 1093.36) |
| United Republic of Tanzania | 2022 | Male | 196667.07 (175571.03 to 217763.11) | 641.26 (572.53 to 709.99) |
| United Republic of Tanzania | 2023 | Male | 196291.12 (151288.94 to 241293.30) | 620.85 (478.54 to 763.16) |
| United Republic of Tanzania | 2024 | Male | 195997.25 (121823.70 to 270170.80) | 601.60 (373.94 to 829.25) |
| United Republic of Tanzania | 2025 | Male | 195782.80 (88037.19 to 303528.41) | 583.45 (262.37 to 904.53) |
| United Republic of Tanzania | 2026 | Male | 195630.63 (50429.37 to 340831.90) | 566.31 (145.99 to 986.63) |
| United Republic of Tanzania | 2027 | Male | 195525.32 (9342.14 to 381708.51) | 550.11 (26.29 to 1073.92) |
| United Republic of Tanzania | 2028 | Male | 195485.31 (0.00 to 425938.73) | 534.82 (0.00 to 1165.31) |
| United Republic of Tanzania | 2029 | Male | 195513.01 (0.00 to 473346.60) | 520.41 (0.00 to 1259.94) |
| United Republic of Tanzania | 2030 | Male | 195602.21 (0.00 to 523782.50) | 506.82 (0.00 to 1357.15) |
| United Republic of Tanzania | 2022 | Female | 167158.19 (152122.74 to 182193.65) | 519.62 (472.95 to 566.30) |
| United Republic of Tanzania | 2023 | Female | 165849.86 (133140.65 to 198559.06) | 500.65 (401.94 to 599.36) |
| United Republic of Tanzania | 2024 | Female | 164715.97 (110677.61 to 218754.33) | 483.03 (324.58 to 641.48) |
| United Republic of Tanzania | 2025 | Female | 163753.93 (85339.02 to 242168.84) | 466.69 (243.23 to 690.16) |
| United Republic of Tanzania | 2026 | Female | 162936.06 (57475.31 to 268396.82) | 451.50 (159.27 to 743.73) |
| United Republic of Tanzania | 2027 | Female | 162262.13 (27324.38 to 297199.89) | 437.39 (73.66 to 801.12) |
| United Republic of Tanzania | 2028 | Female | 161757.13 (0.00 to 328469.84) | 424.36 (0.00 to 861.72) |
| United Republic of Tanzania | 2029 | Female | 161429.09 (0.00 to 362140.22) | 412.37 (0.00 to 925.07) |
| United Republic of Tanzania | 2030 | Female | 161273.10 (0.00 to 398165.15) | 401.33 (0.00 to 990.84) |
| United Republic of Tanzania | 2022 | Both | 363219.65 (327768.49 to 398670.81) | 578.02 (521.64 to 634.41) |
| United Republic of Tanzania | 2023 | Both | 361620.05 (284825.80 to 438414.29) | 558.55 (439.95 to 677.15) |
| United Republic of Tanzania | 2024 | Both | 360275.30 (233290.64 to 487259.96) | 540.31 (349.88 to 730.74) |
| United Republic of Tanzania | 2025 | Both | 359178.21 (174589.28 to 543767.13) | 523.25 (254.34 to 792.15) |
| United Republic of Tanzania | 2026 | Both | 358290.15 (109561.77 to 607018.53) | 507.26 (155.12 to 859.40) |
| United Republic of Tanzania | 2027 | Both | 357595.92 (38777.08 to 676414.76) | 492.28 (53.38 to 931.17) |
| United Republic of Tanzania | 2028 | Both | 357138.58 (0.00 to 751638.69) | 478.29 (0.00 to 1006.62) |
| United Republic of Tanzania | 2029 | Both | 356926.73 (0.00 to 832454.75) | 465.26 (0.00 to 1085.11) |
| United Republic of Tanzania | 2030 | Both | 356946.50 (0.00 to 918670.19) | 453.10 (0.00 to 1166.14) |
| Vanuatu | 2022 | Male | 4486.24 (2033.12 to 6939.37) | 2776.21 (1260.33 to 4292.10) |
| Vanuatu | 2023 | Male | 4366.31 (0.00 to 9672.93) | 2649.02 (0.00 to 5867.56) |
| Vanuatu | 2024 | Male | 4249.75 (0.00 to 12874.52) | 2528.35 (0.00 to 7659.02) |
| Vanuatu | 2025 | Male | 4136.59 (0.00 to 16412.09) | 2413.75 (0.00 to 9576.26) |
| Vanuatu | 2026 | Male | 4026.38 (0.00 to 20193.43) | 2304.71 (0.00 to 11558.47) |
| Vanuatu | 2027 | Male | 3918.79 (0.00 to 24149.49) | 2200.98 (0.00 to 13563.27) |
| Vanuatu | 2028 | Male | 3814.03 (0.00 to 28229.20) | 2102.36 (0.00 to 15560.27) |
| Vanuatu | 2029 | Male | 3712.07 (0.00 to 32391.10) | 2008.62 (0.00 to 17526.86) |
| Vanuatu | 2030 | Male | 3613.12 (0.00 to 36604.15) | 1919.43 (0.00 to 19445.44) |
| Vanuatu | 2022 | Female | 3728.06 (1738.31 to 5717.82) | 2355.46 (1100.57 to 3610.35) |
| Vanuatu | 2023 | Female | 3637.47 (0.00 to 7947.27) | 2249.97 (0.00 to 4914.81) |
| Vanuatu | 2024 | Female | 3548.77 (0.00 to 10567.64) | 2149.73 (0.00 to 6400.95) |
| Vanuatu | 2025 | Female | 3462.07 (0.00 to 13472.86) | 2054.38 (0.00 to 7994.37) |
| Vanuatu | 2026 | Female | 3377.14 (0.00 to 16588.85) | 1963.57 (0.00 to 9644.99) |
| Vanuatu | 2027 | Female | 3293.67 (0.00 to 19859.20) | 1877.05 (0.00 to 11317.49) |
| Vanuatu | 2028 | Female | 3211.81 (0.00 to 23241.33) | 1794.64 (0.00 to 12986.21) |
| Vanuatu | 2029 | Female | 3131.53 (0.00 to 26700.17) | 1716.11 (0.00 to 14631.81) |
| Vanuatu | 2030 | Female | 3053.11 (0.00 to 30209.51) | 1641.23 (0.00 to 16239.32) |
| Vanuatu | 2022 | Both | 8198.96 (3636.33 to 12761.60) | 2563.23 (1137.90 to 3988.56) |
| Vanuatu | 2023 | Both | 7995.73 (0.00 to 17907.81) | 2448.96 (0.00 to 5484.39) |
| Vanuatu | 2024 | Both | 7797.62 (0.00 to 23950.12) | 2340.48 (0.00 to 7188.42) |
| Vanuatu | 2025 | Both | 7604.65 (0.00 to 30646.05) | 2237.34 (0.00 to 9016.08) |
| Vanuatu | 2026 | Both | 7415.90 (0.00 to 37824.39) | 2139.04 (0.00 to 10909.95) |
| Vanuatu | 2027 | Both | 7230.84 (0.00 to 45356.62) | 2045.40 (0.00 to 12829.97) |
| Vanuatu | 2028 | Both | 7050.02 (0.00 to 53147.49) | 1956.26 (0.00 to 14747.40) |
| Vanuatu | 2029 | Both | 6873.42 (0.00 to 61118.98) | 1871.41 (0.00 to 16640.66) |
| Vanuatu | 2030 | Both | 6701.47 (0.00 to 69211.61) | 1790.57 (0.00 to 18492.63) |
| Viet Nam | 2022 | Male | 402368.50 (314398.92 to 490338.07) | 811.65 (634.21 to 989.08) |
| Viet Nam | 2023 | Male | 390616.02 (201588.88 to 579643.16) | 782.64 (403.91 to 1161.37) |
| Viet Nam | 2024 | Male | 379213.04 (73059.84 to 685366.24) | 755.06 (145.48 to 1364.64) |
| Viet Nam | 2025 | Male | 368092.46 (0.00 to 802481.58) | 728.75 (0.00 to 1588.74) |
| Viet Nam | 2026 | Male | 357226.09 (0.00 to 927534.55) | 703.59 (0.00 to 1826.87) |
| Viet Nam | 2027 | Male | 346657.18 (0.00 to 1058128.81) | 679.56 (0.00 to 2074.28) |
| Viet Nam | 2028 | Male | 336358.55 (0.00 to 1192267.78) | 656.61 (0.00 to 2327.44) |
| Viet Nam | 2029 | Male | 326371.39 (0.00 to 1328582.21) | 634.68 (0.00 to 2583.63) |
| Viet Nam | 2030 | Male | 316666.28 (0.00 to 1465766.39) | 613.65 (0.00 to 2840.42) |
| Viet Nam | 2022 | Female | 338095.03 (259542.37 to 416647.68) | 667.69 (512.57 to 822.80) |
| Viet Nam | 2023 | Female | 327168.02 (158551.81 to 495784.22) | 641.38 (310.83 to 971.93) |
| Viet Nam | 2024 | Female | 316654.40 (44213.94 to 589094.87) | 616.51 (86.09 to 1146.93) |
| Viet Nam | 2025 | Female | 306487.91 (0.00 to 692053.60) | 592.92 (0.00 to 1338.81) |
| Viet Nam | 2026 | Female | 296637.01 (0.00 to 801569.50) | 570.49 (0.00 to 1541.57) |
| Viet Nam | 2027 | Female | 287138.64 (0.00 to 915548.25) | 549.19 (0.00 to 1751.12) |
| Viet Nam | 2028 | Female | 277971.28 (0.00 to 1032293.67) | 529.00 (0.00 to 1964.52) |
| Viet Nam | 2029 | Female | 269164.48 (0.00 to 1150671.42) | 509.84 (0.00 to 2179.54) |
| Viet Nam | 2030 | Female | 260678.51 (0.00 to 1269572.30) | 491.59 (0.00 to 2394.17) |
| Viet Nam | 2022 | Both | 741184.03 (574000.64 to 908367.42) | 739.62 (572.80 to 906.44) |
| Viet Nam | 2023 | Both | 718660.08 (359410.61 to 1077909.54) | 712.11 (356.14 to 1068.08) |
| Viet Nam | 2024 | Both | 696894.97 (115480.98 to 1278308.97) | 686.02 (113.68 to 1258.35) |
| Viet Nam | 2025 | Both | 675750.48 (0.00 to 1499985.60) | 661.19 (0.00 to 1467.67) |
| Viet Nam | 2026 | Both | 655162.07 (0.00 to 1736362.04) | 637.51 (0.00 to 1689.59) |
| Viet Nam | 2027 | Both | 635213.84 (0.00 to 1982935.29) | 614.95 (0.00 to 1919.67) |
| Viet Nam | 2028 | Both | 615862.94 (0.00 to 2236013.58) | 593.47 (0.00 to 2154.71) |
| Viet Nam | 2029 | Both | 597181.72 (0.00 to 2493090.43) | 573.02 (0.00 to 2392.21) |
| Viet Nam | 2030 | Both | 579096.78 (0.00 to 2751724.23) | 553.46 (0.00 to 2629.92) |
| Yemen | 2022 | Male | 79044.79 (67742.47 to 90347.12) | 451.49 (387.01 to 515.97) |
| Yemen | 2023 | Male | 78905.98 (54784.52 to 103027.44) | 440.47 (305.85 to 575.09) |
| Yemen | 2024 | Male | 78698.99 (38984.69 to 118413.30) | 429.68 (212.87 to 646.49) |
| Yemen | 2025 | Male | 78428.63 (20881.34 to 135975.91) | 419.11 (111.60 to 726.61) |
| Yemen | 2026 | Male | 78110.66 (824.00 to 155397.32) | 408.79 (4.32 to 813.27) |
| Yemen | 2027 | Male | 77740.30 (0.00 to 176421.42) | 398.74 (0.00 to 904.88) |
| Yemen | 2028 | Male | 77308.73 (0.00 to 198802.72) | 388.90 (0.00 to 1000.06) |
| Yemen | 2029 | Male | 76821.60 (0.00 to 222352.64) | 379.24 (0.00 to 1097.67) |
| Yemen | 2030 | Male | 76281.85 (0.00 to 246897.96) | 369.73 (0.00 to 1196.68) |
| Yemen | 2022 | Female | 58466.98 (49758.30 to 67175.65) | 342.02 (291.15 to 392.89) |
| Yemen | 2023 | Female | 58092.07 (39717.13 to 76467.00) | 332.15 (227.12 to 437.17) |
| Yemen | 2024 | Female | 57690.75 (27630.90 to 87750.61) | 322.65 (154.56 to 490.75) |
| Yemen | 2025 | Female | 57268.34 (13919.08 to 100617.59) | 313.53 (76.22 to 550.84) |
| Yemen | 2026 | Female | 56831.42 (0.00 to 114814.56) | 304.76 (0.00 to 615.68) |
| Yemen | 2027 | Female | 56376.39 (0.00 to 130138.68) | 296.32 (0.00 to 684.02) |
| Yemen | 2028 | Female | 55900.72 (0.00 to 146423.10) | 288.21 (0.00 to 754.90) |
| Yemen | 2029 | Female | 55412.70 (0.00 to 163553.67) | 280.39 (0.00 to 827.58) |
| Yemen | 2030 | Female | 54917.81 (0.00 to 181434.69) | 272.86 (0.00 to 901.45) |
| Yemen | 2022 | Both | 137265.80 (118629.97 to 155901.63) | 396.70 (342.88 to 450.51) |
| Yemen | 2023 | Both | 136802.81 (96409.19 to 177196.42) | 386.40 (272.33 to 500.48) |
| Yemen | 2024 | Both | 136246.52 (69484.59 to 203008.45) | 376.42 (191.98 to 560.85) |
| Yemen | 2025 | Both | 135593.42 (38728.12 to 232458.72) | 366.68 (104.74 to 628.62) |
| Yemen | 2026 | Both | 134865.62 (4735.03 to 264996.21) | 357.21 (12.55 to 701.87) |
| Yemen | 2027 | Both | 134071.43 (0.00 to 300203.82) | 348.04 (0.00 to 779.30) |
| Yemen | 2028 | Both | 133199.74 (0.00 to 337689.59) | 339.15 (0.00 to 859.81) |
| Yemen | 2029 | Both | 132261.83 (0.00 to 377159.63) | 330.49 (0.00 to 942.44) |
| Yemen | 2030 | Both | 131254.38 (0.00 to 418319.72) | 322.03 (0.00 to 1026.33) |
| Zambia | 2022 | Male | 216385.19 (202975.37 to 229795.01) | 2182.02 (2047.11 to 2316.93) |
| Zambia | 2023 | Male | 210815.63 (182625.95 to 239005.31) | 2067.79 (1791.43 to 2344.14) |
| Zambia | 2024 | Male | 205351.20 (159899.48 to 250802.91) | 1960.23 (1526.44 to 2394.01) |
| Zambia | 2025 | Male | 200001.05 (135543.24 to 264458.86) | 1859.07 (1259.97 to 2458.17) |
| Zambia | 2026 | Male | 194773.23 (110048.49 to 279497.97) | 1763.98 (996.70 to 2531.26) |
| Zambia | 2027 | Male | 189650.70 (83759.67 to 295541.73) | 1674.41 (739.54 to 2609.28) |
| Zambia | 2028 | Male | 184631.84 (56958.72 to 312304.96) | 1590.02 (490.54 to 2689.50) |
| Zambia | 2029 | Male | 179713.93 (29872.74 to 329555.12) | 1510.52 (251.10 to 2769.94) |
| Zambia | 2030 | Male | 174906.44 (2690.02 to 347122.86) | 1435.70 (22.10 to 2849.31) |
| Zambia | 2022 | Female | 189892.40 (179216.36 to 200568.44) | 1868.36 (1763.65 to 1973.06) |
| Zambia | 2023 | Female | 185305.60 (162457.99 to 208153.20) | 1772.33 (1553.95 to 1990.70) |
| Zambia | 2024 | Female | 180802.05 (143719.45 to 217884.65) | 1681.90 (1337.02 to 2026.77) |
| Zambia | 2025 | Female | 176381.85 (123579.04 to 229184.66) | 1596.76 (1118.80 to 2074.72) |
| Zambia | 2026 | Female | 172040.32 (102426.54 to 241654.10) | 1516.54 (902.94 to 2130.15) |
| Zambia | 2027 | Female | 167774.83 (80551.86 to 254997.80) | 1440.90 (691.83 to 2189.96) |
| Zambia | 2028 | Female | 163590.70 (58188.79 to 268992.61) | 1369.60 (487.19 to 2252.02) |
| Zambia | 2029 | Female | 159487.45 (35522.92 to 283451.99) | 1302.43 (290.11 to 2314.74) |
| Zambia | 2030 | Female | 155468.32 (12707.87 to 298228.77) | 1239.15 (101.30 to 2377.00) |
| Zambia | 2022 | Both | 406308.96 (382858.26 to 429759.67) | 2023.42 (1906.80 to 2140.04) |
| Zambia | 2023 | Both | 396117.59 (345865.18 to 446370.00) | 1918.18 (1674.91 to 2161.45) |
| Zambia | 2024 | Both | 386115.87 (304574.04 to 467657.70) | 1819.09 (1434.97 to 2203.21) |
| Zambia | 2025 | Both | 376313.40 (260273.81 to 492352.99) | 1725.87 (1193.71 to 2258.02) |
| Zambia | 2026 | Both | 366715.30 (213838.20 to 519592.40) | 1638.15 (955.25 to 2321.05) |
| Zambia | 2027 | Both | 357297.19 (165895.47 to 548698.92) | 1555.48 (722.24 to 2388.73) |
| Zambia | 2028 | Both | 348063.94 (116960.23 to 579167.65) | 1477.58 (496.53 to 2458.64) |
| Zambia | 2029 | Both | 339011.95 (67443.93 to 610579.97) | 1404.19 (279.36 to 2529.02) |
| Zambia | 2030 | Both | 330154.86 (17688.29 to 642621.42) | 1335.09 (71.54 to 2598.65) |
| Zimbabwe | 2022 | Male | 74967.64 (67757.80 to 82177.48) | 937.99 (848.03 to 1027.95) |
| Zimbabwe | 2023 | Male | 73672.52 (58197.62 to 89147.43) | 900.28 (711.29 to 1089.27) |
| Zimbabwe | 2024 | Male | 72379.41 (47120.47 to 97638.35) | 864.24 (562.70 to 1165.78) |
| Zimbabwe | 2025 | Male | 71088.89 (34899.66 to 107278.11) | 829.81 (407.42 to 1252.19) |
| Zimbabwe | 2026 | Male | 69799.57 (21788.68 to 117810.46) | 796.88 (248.79 to 1344.97) |
| Zimbabwe | 2027 | Male | 68510.15 (7979.48 to 129040.82) | 765.38 (89.17 to 1441.59) |
| Zimbabwe | 2028 | Male | 67225.00 (0.00 to 140822.13) | 735.26 (0.00 to 1540.20) |
| Zimbabwe | 2029 | Male | 65944.53 (0.00 to 153027.45) | 706.46 (0.00 to 1639.36) |
| Zimbabwe | 2030 | Male | 64670.86 (0.00 to 165553.03) | 678.91 (0.00 to 1737.94) |
| Zimbabwe | 2022 | Female | 67096.64 (60550.85 to 73642.44) | 782.41 (706.31 to 858.51) |
| Zimbabwe | 2023 | Female | 65670.52 (51595.02 to 79746.03) | 748.67 (588.30 to 909.03) |
| Zimbabwe | 2024 | Female | 64269.55 (41341.64 to 87197.46) | 716.62 (461.03 to 972.22) |
| Zimbabwe | 2025 | Female | 62893.94 (30134.85 to 95653.02) | 686.20 (328.82 to 1043.57) |
| Zimbabwe | 2026 | Female | 61542.67 (18207.53 to 104877.80) | 657.30 (194.49 to 1120.11) |
| Zimbabwe | 2027 | Female | 60214.06 (5732.42 to 114695.70) | 629.84 (59.98 to 1199.69) |
| Zimbabwe | 2028 | Female | 58911.12 (0.00 to 124975.96) | 603.74 (0.00 to 1280.78) |
| Zimbabwe | 2029 | Female | 57633.45 (0.00 to 135608.54) | 578.95 (0.00 to 1362.23) |
| Zimbabwe | 2030 | Female | 56382.63 (0.00 to 146507.36) | 555.39 (0.00 to 1443.15) |
| Zimbabwe | 2022 | Both | 141388.54 (127748.48 to 155028.60) | 853.38 (771.18 to 935.59) |
| Zimbabwe | 2023 | Both | 138671.82 (109277.48 to 168066.16) | 817.88 (644.57 to 991.20) |
| Zimbabwe | 2024 | Both | 135983.65 (88004.65 to 183962.64) | 784.07 (507.46 to 1060.68) |
| Zimbabwe | 2025 | Both | 133323.44 (64640.57 to 202006.31) | 751.86 (364.55 to 1139.17) |
| Zimbabwe | 2026 | Both | 130687.55 (39669.85 to 221705.24) | 721.15 (218.92 to 1223.38) |
| Zimbabwe | 2027 | Both | 128073.88 (13457.58 to 242690.18) | 691.86 (72.71 to 1311.02) |
| Zimbabwe | 2028 | Both | 125491.18 (0.00 to 264686.30) | 663.95 (0.00 to 1400.40) |
| Zimbabwe | 2029 | Both | 122939.57 (0.00 to 287457.77) | 637.35 (0.00 to 1490.24) |
| Zimbabwe | 2030 | Both | 120421.15 (0.00 to 310811.50) | 611.97 (0.00 to 1579.51) |
| **Abbreviations:** ASR, age-standardized rate; CrI, credible interval.  ^1^Niue and Palau are excluded from IHME's population forecast data, thus inapplicable to BAPC. | | | | |
