# Supplementary figures and images for: Global, regional, and national disability-adjusted life years and prevalence of lymphatic filariasis from 1990 to 2021: A trend and health inequality analysis based on the global burden of disease study 2021
Source: PLoS Negl Trop Dis. 2025 Apr 29;19(4):e0013017. doi: 10.1371/journal.pntd.0013017 (PMC12040265; doi:10.1371/journal.pntd.0013017)

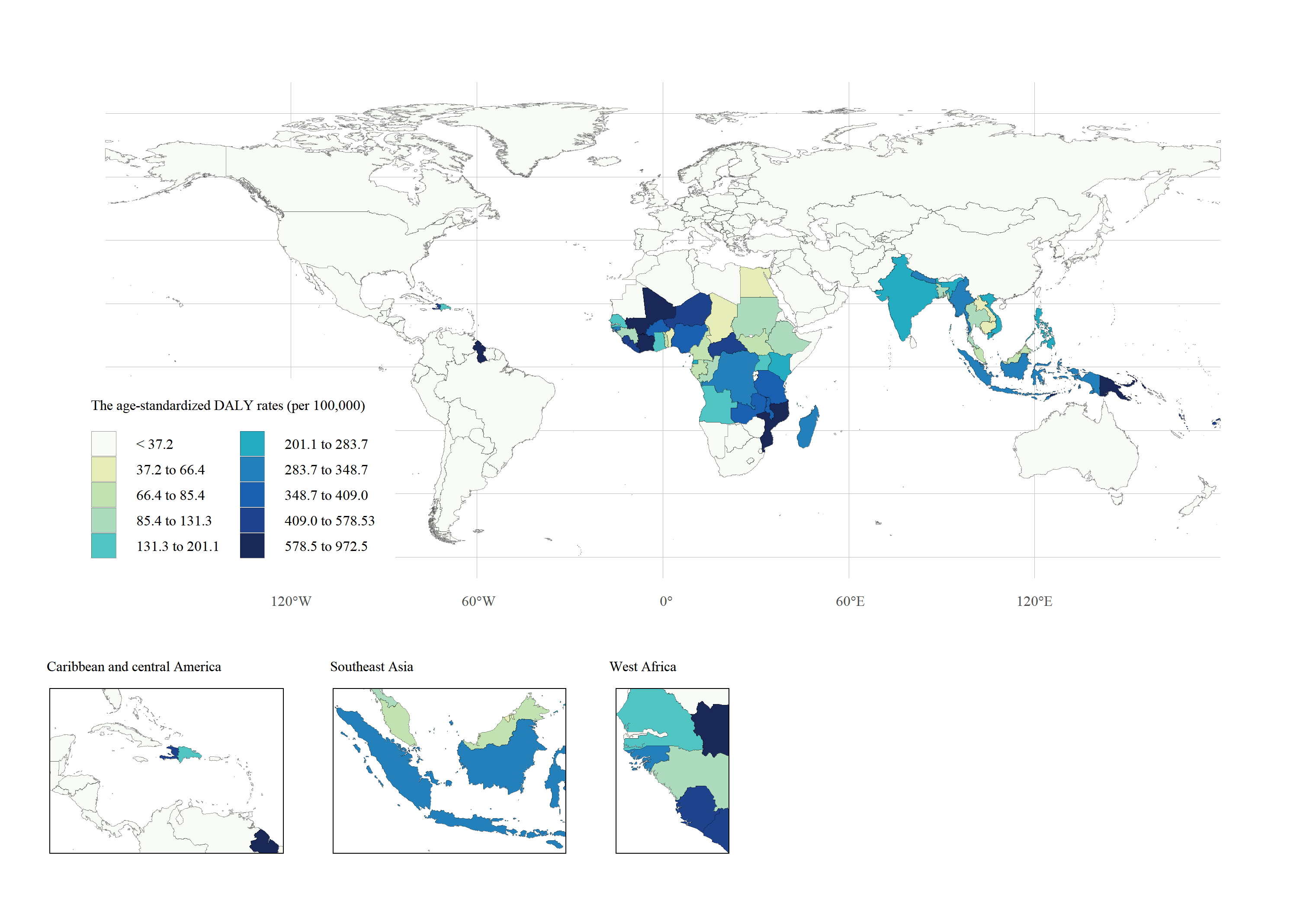

Supplement: S1 Fig — The shapefiles used for spatial visualization are sourced from Natural Earth. The terms of free use for these shapefiles can be found at: https://www.naturalearthdata.com/about/terms-of-use/, the shapefiles available at: https://www.naturalearthdata.com/downloads/10m-cultural-vectors/10m-admin-0-countries/. (TIF) [file pntd.0013017.s013.tif]

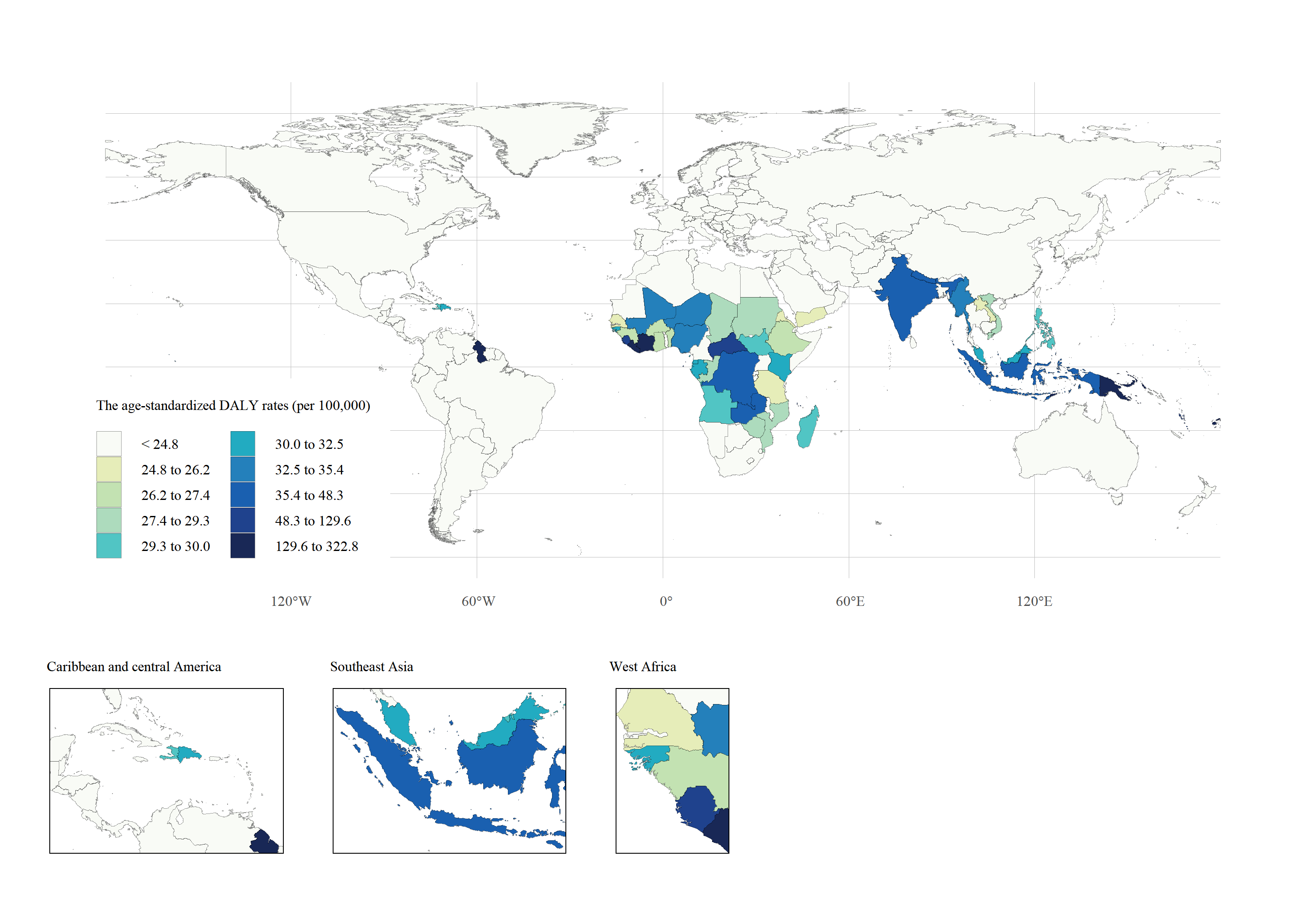

Supplement: S2 Fig — The shapefiles used for spatial visualization are sourced from Natural Earth. The terms of free use for these shapefiles can be found at: https://www.naturalearthdata.com/about/terms-of-use/, the shapefiles available at: https://www.naturalearthdata.com/downloads/10m-cultural-vectors/10m-admin-0-countries/. (TIF) [file pntd.0013017.s014.tif]

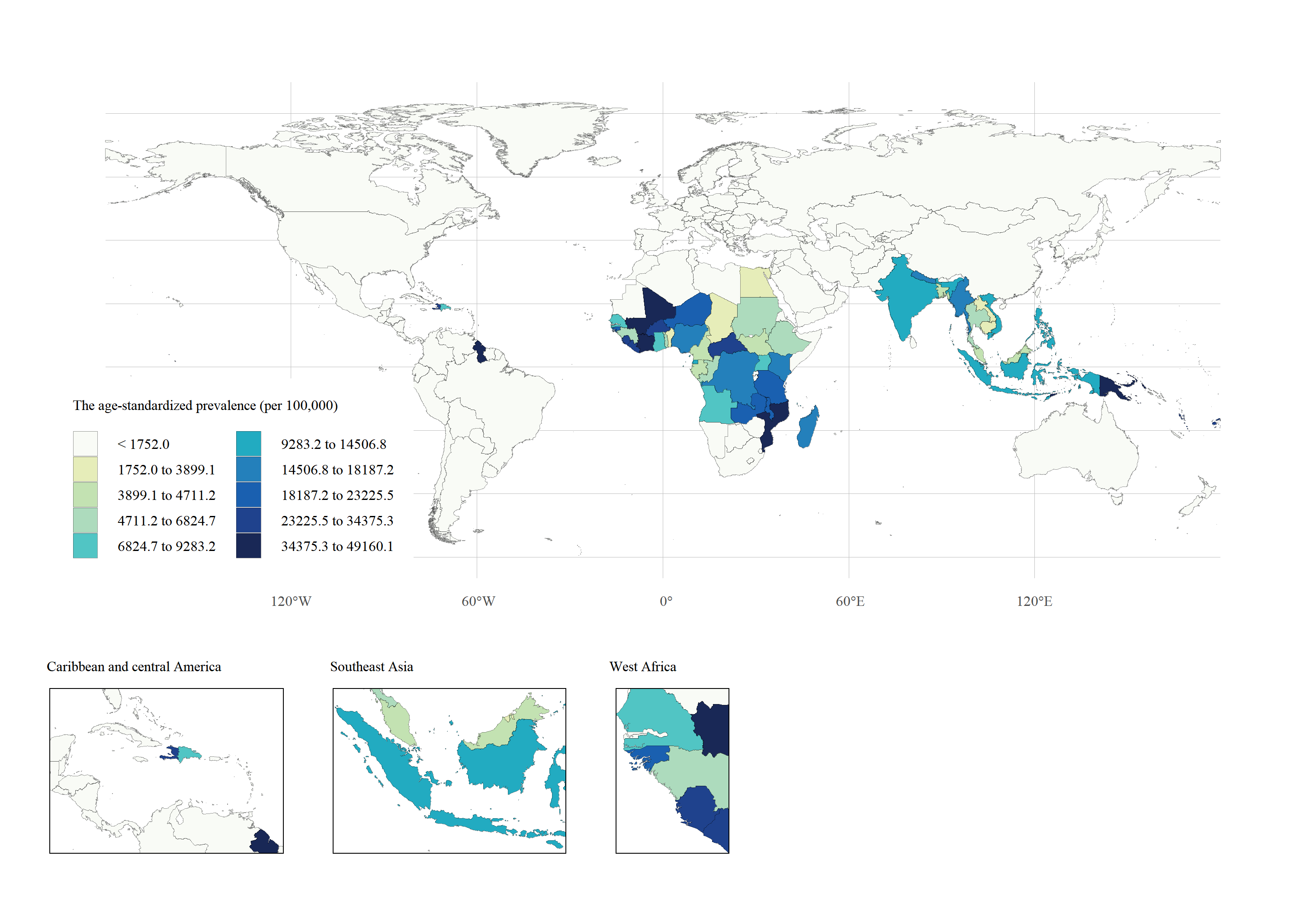

Supplement: S3 Fig — The shapefiles used for spatial visualization are sourced from Natural Earth. The terms of free use for these shapefiles can be found at: https://www.naturalearthdata.com/about/terms-of-use/, the shapefiles available at: https://www.naturalearthdata.com/downloads/10m-cultural-vectors/10m-admin-0-countries/. (TIF) [file pntd.0013017.s015.tif]

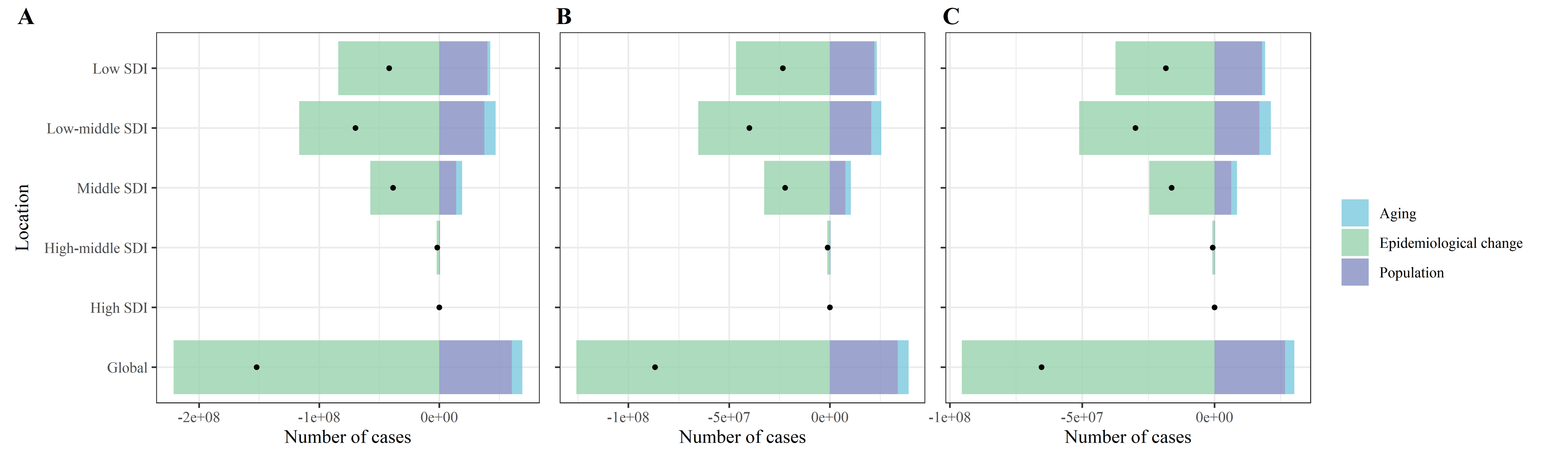

Supplement: S4 Fig — (A) Both sex (B) Male (C) Female. Abbreviations: SDI, socio-demographic index; DALYs, disability adjusted life years. (TIF) [file pntd.0013017.s016.tif]

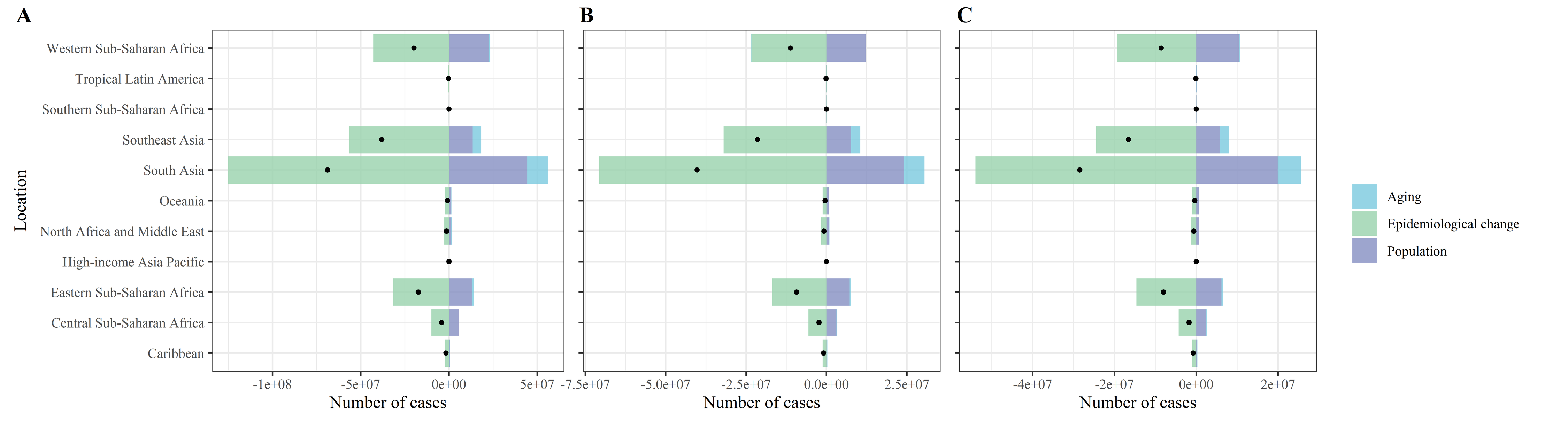

Supplement: S5 Fig — (A) Both sex (B) Male (C) Female. Abbreviations: SDI, socio-demographic index; DALYs, disability adjusted life years. (TIF) [file pntd.0013017.s017.tif]
